# Integrative analysis to explore the biological association between environmental skin diseases and ambient particulate matter

**DOI:** 10.1038/s41598-022-13001-x

**Published:** 2022-06-13

**Authors:** Hyun Soo Kim, Hye-Won Na, Yujin Jang, Su Ji Kim, Nam Gook Kee, Dong Yeop Shin, Hyunjung Choi, Hyoung-June Kim, Young Rok Seo

**Affiliations:** 1 Institute of Environmental Medicine, Department of Life Science, Dongguk University Biomedi Campus, 32, Dongguk-ro, Ilsandong-gu, Goyang-si, Gyeonggi-do 10326 Republic of Korea; 2 National Institute of Environmental Research, Hwangyeong-ro 42, Seo-gu, Incheon, 22689 Republic of Korea; 3 Bioscience Research Institute, Amorepacific Corporation R&I Center, 1920, Yonggu-daero, Giheung-gu, Gyeonggi-do 17074 Republic of Korea

**Keywords:** Biomarkers, Health care

## Abstract

Although numerous experimental studies have suggested a significant association between ambient particulate matter (PM) and respiratory damage, the etiological relationship between ambient PM and environmental skin diseases is not clearly understood. Here, we aimed to explore the association between PM and skin diseases through biological big data analysis. Differential gene expression profiles associated with PM and environmental skin diseases were retrieved from public genome databases. The co-expression among them was analyzed using a text-mining-based network analysis software. Activation/inhibition patterns from RNA-sequencing data performed with PM_2.5_-treated normal human epidermal keratinocytes (NHEK) were overlapped to select key regulators of the analyzed pathways. We explored the adverse effects of PM on the skin and attempted to elucidate their relationships using public genome data. We found that changes in upstream regulators and inflammatory signaling networks mediated by MMP-1, MMP-9, PLAU, S100A9, IL-6, and S100A8 were predicted as the key pathways underlying PM-induced skin diseases. Our integrative approach using a literature-based co-expression analysis and experimental validation not only improves the reliability of prediction but also provides assistance to clarify underlying mechanisms of ambient PM-induced dermal toxicity that can be applied to screen the relationship between other chemicals and adverse effects.

## Introduction

Ambient air pollution is a serious public health hazard in modern industrialized societies. The World Health Organization (WHO) reported that seven million people die annually from exposure to polluted air^1^. Airborne pollutants comprise various contaminants, including inorganic compounds, volatile organic compounds (VOC), persistent free radicals, and biological allergens^2^. Such contaminants directly affect human health by inducing various adverse outcomes in the skin, respiratory, cardiovascular, and nervous systems through exposure from inhalation or physical contact^3^.

Particulate matter (PM) is a complex mixture of diverse harmful substances of ≤ 10 μm in size and is considered a representative airborne pollutant. PM is composed of various chemical species of diverse sizes and shapes, making it difficult to predict toxicity^4^. Harmful inorganic metals, carbon compounds, polycyclic aromatic hydrocarbons (PAH), and VOC comprise the largest proportion of the components. Based on their aerodynamic diameter, PM ≤ 10 μm and PM ≤ 2.5 μm are called the coarse fraction (PM_10_) and fine particles (PM_2.5_), respectively^5^. In addition to the physiological toxicity induced by the harmful components, the physical damage caused by PM penetration has been studied by various researchers^6,7^.

Most studies have focused on the respiratory tract or pulmonary damage derived from inhaling PM. Numerous studies have established an association between ambient PM concentration and respiratory disorders, including aggravation of asthma, decreased lung function, increased coughing, or difficulty breathing^8–10^. As with the respiratory system, the skin also directly contacts the external environment. Skin is the primary interface that protects the body from environmental stressors, such as ultraviolet light, ozone, and PM^11^, so physical or physiological adverse effects in the epidermis, dermis, and deeper subcutaneous layer of the skin are the earliest response against changes in the surrounding environment. Several epidemiological, in vitro, and in vivo studies have reported that skin irritation due to exposure to PM may exacerbate skin diseases or symptoms^7,12,13^. A correlation between ambient PM exposure and outbreaks of environmental skin diseases, including atopic dermatitis, allergic contact dermatitis, and eczema, has also been suggested^14–16^. However, the detailed mechanism underlying air pollutant toxicity via skin absorption is not fully understood.

With the rapid development of high-throughput techniques, gene expression profiles associated with such diseases or chemicals are actively accumulated. The data that have been curated depend on the objectives of researchers and are provided through various public databases. Genome-wide clustering of large-scale gene expression profiles provides clues to interpreting dynamic co-regulation of genes and uncovering the mechanisms linking the chemical, genotype, and phenotype. In this respect, gene co-expression network analysis is actively suggested as an efficient method for biological big data analysis^17^. Various algorithms and tools for interpreting differential gene expression profiles have been developed. However, a lack of approaches can be expected to comprehensively interpret accumulated literature-based data and experimental results.

In this study, we focused on interpreting the etiological relationships between ambient PM and environmental skin diseases by using large-scale gene expression profiles available in public databases. Knowing that the skin toxicity of PM relates to the chemical and physical characteristics of the PM, we explored the biological associations between ambient PM and major environmental skin diseases (atopic dermatitis, allergic contact dermatitis, and eczema) via a text-mining-based pathway analysis of publicly available gene expression data. The detailed relationships predicted by our analysis were validated using experimental data and RNA samples from RNA-sequencing (RNA-seq) of PM_2.5_-treated normal human keratinocytes. This study not only provides a comprehensive overview of PM-induced skin damage but also offers new perspectives on the biological association between harmful substances and potential adverse effects, using a public gene expression dataset.

## Results

### Data grouping and identification of gene lists predicted to be involved with PM-induced skin diseases using data collected from public databases

**Figure 1:**
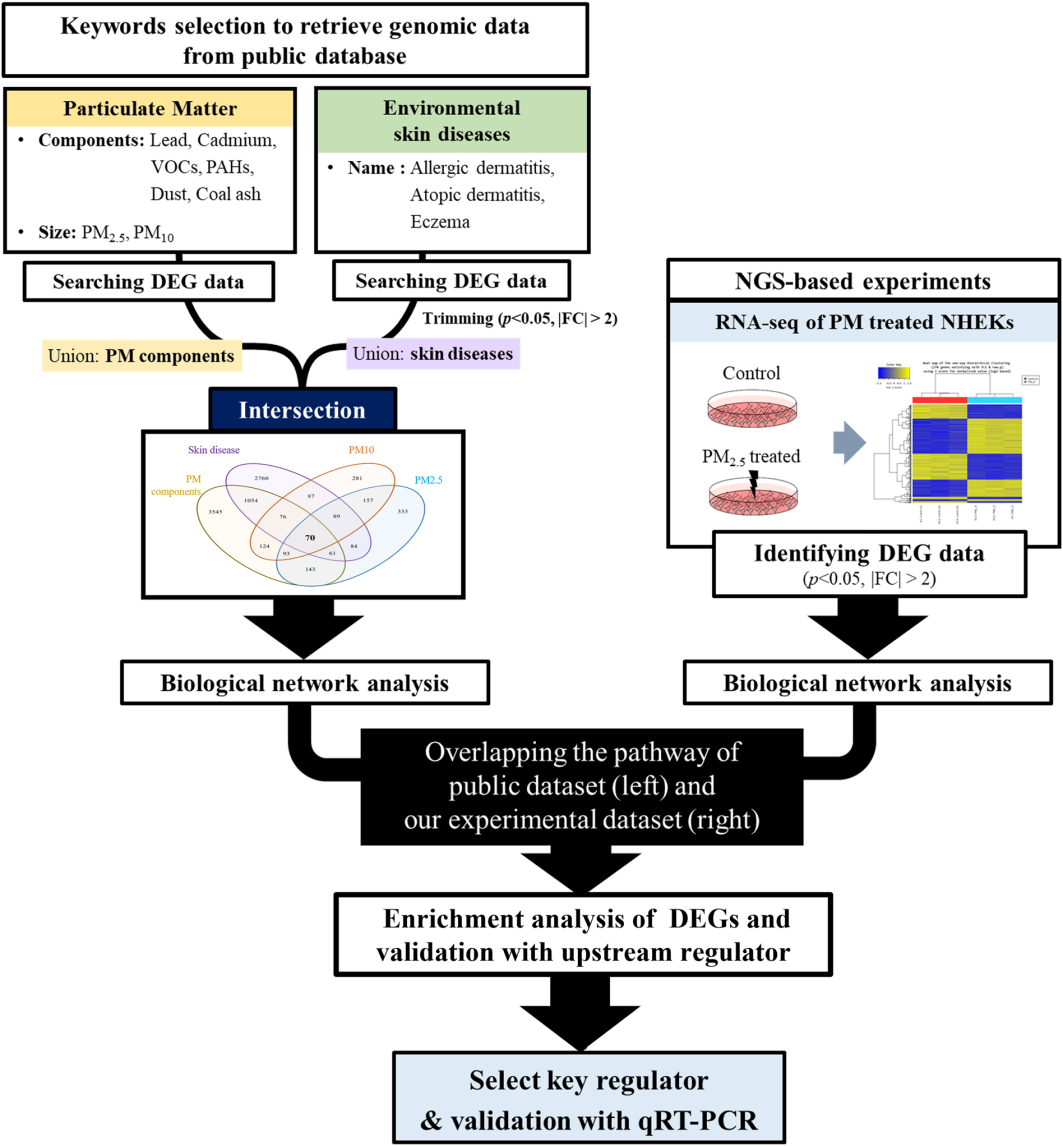
Principle of data crawling from public genome databases and next-generation sequencing (NGS)-based analysis. Keywords for crawling genome data were selected based on characteristics of PM and names of environmental skin diseases. Criteria for differential gene expression were *p* < 0.05 and |fold-change| > 2. Groups of retrieved genes were compared, and intersections were identified. DEG data and RNA samples from NGS experiments were used for further analysis to select key regulators of the PM-induced pathway and for validation.

Before analysis, retrieved genomic data were categorized by referring to the search keywords. Chemical–gene association data associated with “Lead,” “Cadmium,” “VOC,” “PAH,” or “Coal Ash” were grouped into “PM components.” Gene expression data of “PM_10_” and “PM_2.5_” shared a topic of “PM size,” but they were independently grouped to distinguish the effect of PM size. Microarray datasets related to “Allergic dermatitis,” “Atopic dermatitis,” and “Eczema” were grouped as “Skin diseases.” The group names, keywords, and the number of retrieved genes are summarized in Table 1. All genes are listed in the Supplementary Data. To identify the common genes associated with PM and environmental skin diseases, the intersection of each set was established (Fig. 1 and Supplementary Fig. S1).

**Table 1:** Keywords used for retrieving the public genomic data relevant to PM and skin diseases.

Topic	Keyword used for searching the DEG data	Number of searched genes	Reference (or GEO accession number)
PM components	Lead	596	^60^
	Cadmium	1854	^61–65^
	VOCs	507	^66^
	PAHs	2761	^67–80^
	Coal Ash	134	^81–84^
PM size	PM_2.5_	1030	^85–91^
	PM_10_	977	^92–95^
Skin diseases	Allergic dermatitis	178	GSE6281^96^
	Atopic dermatitis	4087	GSE5667, GSE32924, GSE26952^46^
	Eczema	153	^97–99^

### Pathway analysis of publicly available data to explore altered biological functions and cellular processes related to the common properties of PM and environmental skin diseases

**Figure 2:**
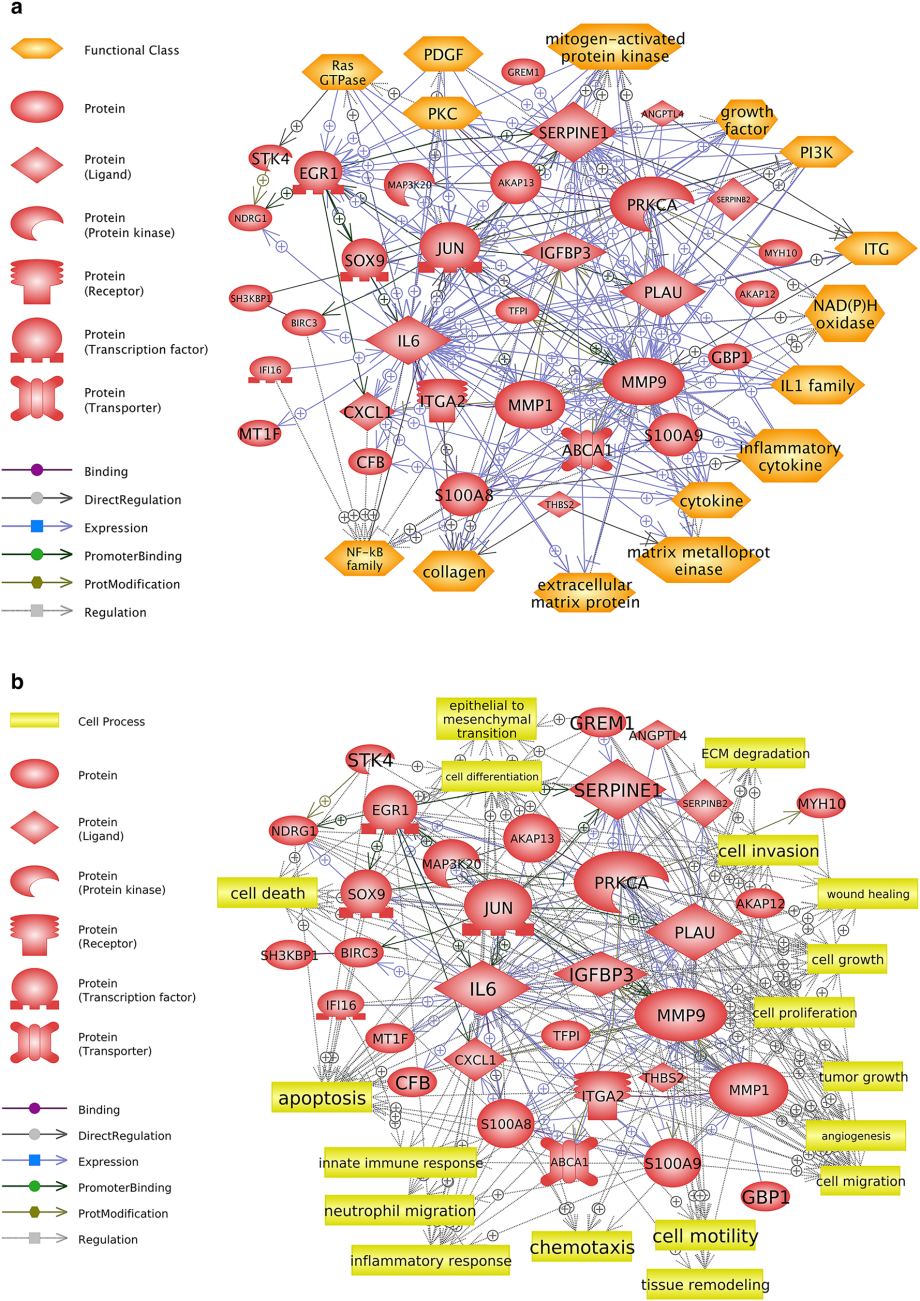
Potential biological signaling networks involved with PM-induced skin diseases predicted from the public data-based analysis. Direct biological interaction among the identified genes related to PM-induced skin diseases. (**a**) Biological functions of the genes predicted to be altered by signaling networks were analyzed using Pathway Studio. The predicted functions are presented as a “functional class” in the pathway. (**b**) Cellular processes predicted to be affected by signaling networks among the identified genes were analyzed using Pathway Studio. The descriptions of the schematic symbols are located to the left of each figure.

To explore the biological association between environmental skin diseases and PM, the biological networks among intersection genes were analyzed. Among 70 identified genes, 32 genes established significant signaling networks. To investigate the cellular functions or processes altered by PM-induced signaling networks in environmental skin diseases, we expanded the analysis of the genes to include their functional classes and the cell processes involved (Fig. 2). “Functional class” categorizes the genes by their biological functions listed in the database of Pathway Studio. Based on the text-mining algorithm of the software, this analysis enables the prediction of the cellular functions that may be altered in PM-induced skin diseases. The predicted genes in the pathway belong to classes related to inflammatory functions, including “inflammatory cytokine,” “IL-1 family,” and “NF-κB family” (Fig. 2a). In terms of the “cell processes,” the networks among the genes were closely related to alteration of immune processes, such as “innate immune response,” “neutrophil migration,” “inflammatory response,” and “chemotaxis” (see Fig. 2b). In addition, associations with carcinogenesis, such as “angiogenesis,” “tumor growth,” and “epithelial-to-mesenchymal transition,” were also predicted. IL-6, MMP-9, PRKCA, SERPINE1, JUN, PLAU, MMP-1, and EGR-1 showed high betweenness and degree centrality with surrounded entities (Table 2) and were predicted as the key hubs of the analyzed pathway.

**Table 2:** Profiles of the 32 genes identified through literature-based analysis and used to construct the significant signaling networks.

Gene name	Description	Centrality with functional classes		Centrality with cell processes	
		Betweenness	Degree	Betweenness	Degree
IL6	Interleukin 6	0.32786356	60	0.2606371	53
MMP9	Matrix metallopeptidase 9	0.14008759	43	0.08874194	42
PRKCA	Protein kinase C alpha	0.10249921	23	0.06604883	26
SERPINE1	Serpin family E member 1	0.0940557	31	0.03619284	29
JUN	Jun proto-oncogene, AP-1 transcription factor subunit	0.07616875	34	0.05170048	31
PLAU	Plasminogen activator, urokinase	0.0535497	33	0.03590776	31
MMP1	Matrix metallopeptidase 1	0.04540664	18	0.06081282	24
EGR1	Early growth response 1	0.02858809	23	0.01102559	27
BIRC3	Baculoviral IAP repeat containing 3	0.01057176	4	0.04105898	6
NDRG1	N-myc downstream regulated 1	0.01028651	3	0.00756754	11
AKAP13	A-kinase anchoring protein 13	0.00621003	4	0.00486438	3
S100A8	S100 calcium binding protein A8	0.00602783	19	0.01403312	23
IGFBP3	Insulin like growth factor binding protein 3	0.00470338	12	0.00350003	14
S100A9	S100 calcium binding protein A9	0.00295347	13	0.01168803	18
ABCA1	ATP binding cassette subfamily A member 1	0.00271365	7	4.68E−04	6
SOX9	SRY-box 9	0.00195307	9	0.00333093	13
CXCL1	C-X-C motif chemokine ligand 1	0.00167127	8	0.01085271	17
ANGPTL4	Angiopoietin like 4	7.66E−04	3	3.80E−04	6
SH3KBP1	SH3 domain containing kinase binding protein 1	3.22E−04	2	0	1
STK4	Serine/threonine kinase 4	3.22E−04	2	7.43E−04	5
MAP3K20	Mitogen-activated protein kinase kinase kinase 20	2.42E−04	2	2.83E−04	2
GBP1	Guanylate binding protein 1	2.25E−04	2	0	1
THBS2	Thrombospondin 2	1.93E−04	6	4.16E−04	7
AKAP12	A-kinase anchoring protein 12	0	2	0	3
CFB	Complement factor B	0	2	0	1
GREM1	Gremlin 1, DAN family BMP antagonist	0	1	0.00179531	7
IFI16	Interferon gamma inducible protein 16	0	2	0.00127899	5
ITGA2	Integrin subunit alpha 2	0	3	2.46E−04	4
MT1F	Metallothionein 1F	0	1	0	2
MYH10	Myosin heavy chain 10	0	1	0	2
SERPINB2	Serpin family B member 2	0	4	1.47E−04	7
TFPI	Tissue factor pathway inhibitor	0	2	3.20E−04	6

### Identification of the differentially expressed genes (DEG) in PM_2.5_-exposed skin cells through RNA-seq and pathway analyses to seek the altered signaling networks

**Figure 3:**
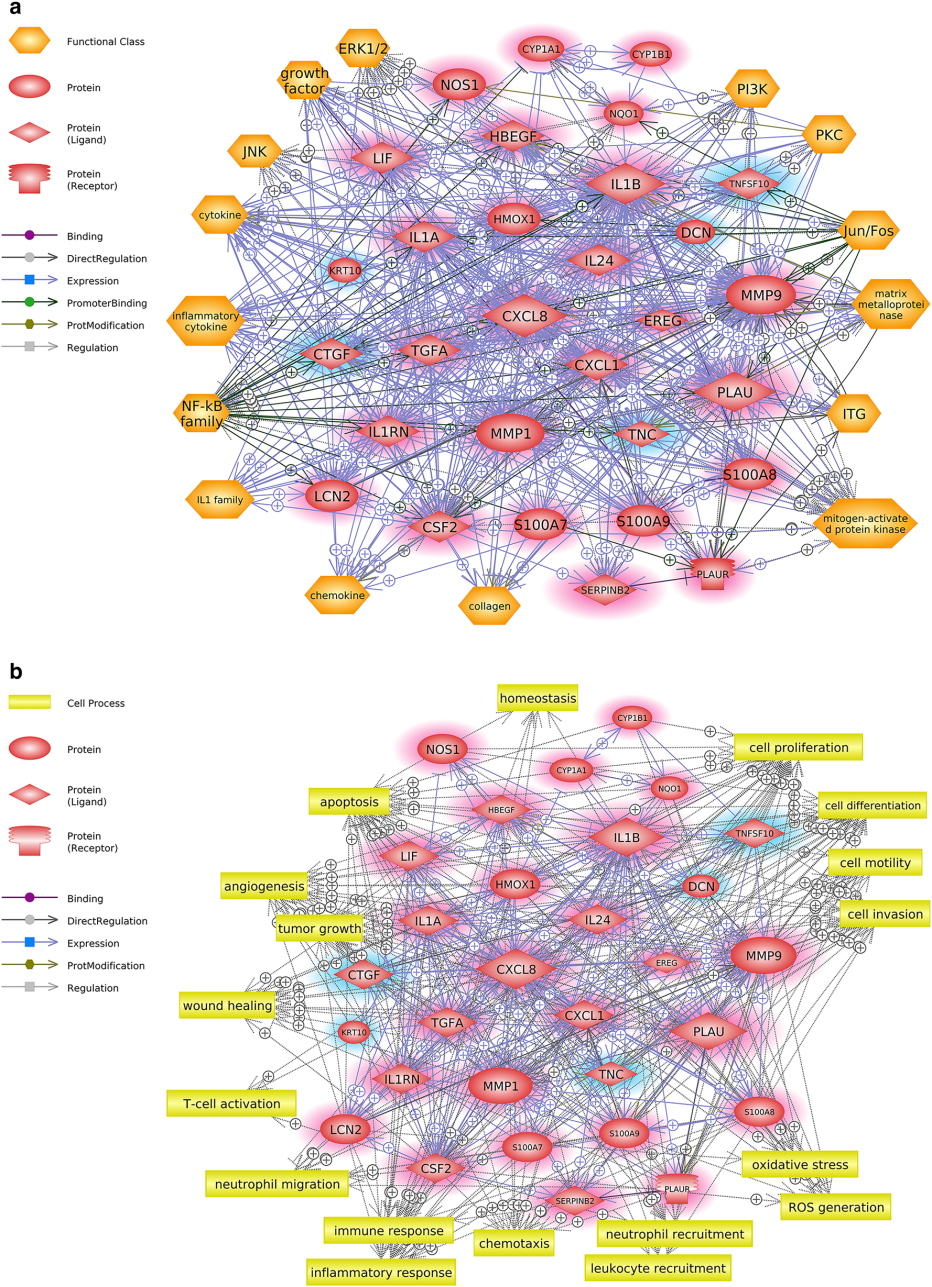
Potential biological signaling networks related to PM-induced skin diseases predicted from our NGS-based experimental data. Direct biological interaction among the identified DEG from in vitro RNA-seq analysis. (**a**) Biological functions predicted to be altered according to the signaling networks were analyzed using Pathway Studio. The predicted functions are presented as a “functional class” among the genes in the pathway. (**b**) Cellular processes predicted to be affected according to the signaling networks were analyzed using Pathway Studio. The descriptions of the schematic symbols are located to the left of each figure. Upregulated and down regulated genes are highlighted in pink and blue respectively.

To verify our public data-based prediction, we obtained transcriptomic profile data from skin cell-based experiments by performing RNA-seq using normal human epidermal keratinocytes (NHEK) that had been exposed to PM_2.5_. In total, 122 genes were downregulated, and 148 genes were upregulated based on a |fold-change| > 2 and *p*-value < 0.05. With these 271 DEG, pathway analysis was conducted to determine the biological alterations in human skin cells under PM exposure. Finally, 30 genes were used to construct the significant signaling networks, and the analysis of cellular functions or processes altered by PM exposure was expanded among the genes in terms of their functional classes and involved cell processes. “NF-κB family,” “cytokine,” “Jun/Fos,” and “inflammatory cytokine” were predicted as the major functional classes that may be altered by differential gene expression upon PM exposure (Fig. 3a). Cell processes involved with basic cell function (cell proliferation, cell differentiation, and cell motility), immune response (inflammatory response, T-cell activation, neutrophil migration/recruitment, and leukocyte recruitment), oxidative stress, and ROS generation were predicted (Fig. 3b). IL-1B, MMP-9, CXCL-8, CSF-2, IL-1A, HMOX-1, MMP-1, and S100A9 were predicted as key hub regulators of the pathway based on their betweenness and degree centrality (Table 3).

**Table 3:** Profiles of the 30 genes were identified through NGS experiment-based analysis and used to construct the significant signaling networks.

Gene	Description	Centrality with functional classes		Centrality with cell processes	
		Betweenness	Degree	Betweenness	Degree
IL1B	Interleukin 1 beta	0.13962021	101	0.12458578	92
MMP9	Matrix metallopeptidase 9	0.05568858	96	0.05479244	82
CXCL8	C-X-C motif chemokine ligand 8	0.06465637	74	0.05802507	76
CSF2	Colony stimulating factor 2	0.0474571	61	0.03368819	60
IL1A	Interleukin 1 alpha	0.03261944	57	0.0646008	57
MMP1	Matrix metallopeptidase 1	0.01289877	53	0.03324277	48
PLAU	Plasminogen activator, urokinase	0.01147841	51	0.02266642	38
HMOX1	Heme oxygenase 1	0.02780391	46	0.03061453	50
CTGF	Connective tissue growth factor	0.00517551	45	0.01824768	31
HBEGF	Heparin binding EGF like growth factor	0.01092291	35	0.01220442	33
S100A8	S100 calcium binding protein A8	0.00613667	34	0.00206479	34
CXCL1	C-X-C motif chemokine ligand 1	0.01436634	33	0.0052915	39
TNFSF10	TNF superfamily member 10	0.01905219	33	0.03689294	32
S100A9	S100 calcium binding protein A9	0.00771954	31	0.00239278	42
IL1RN	Interleukin 1 receptor antagonist	0.00817305	28	0.00851368	31
LCN2	Lipocalin 2	0.00851023	28	0.00293573	29
TGFA	Transforming growth factor alpha	0.0128731	28	0.02490869	26
TNC	Tenascin C	0.00345552	28	0.00364948	24
LIF	LIF, interleukin 6 family cytokine	0.00398179	26	0.00563199	28
PLAUR	Plasminogen activator, urokinase receptor	0.00785025	26	0.00437903	26
CYP1A1	Cytochrome P450 family 1 subfamily A member 1	0.00158859	18	0.0077291	24
DCN	Decorin	7.98E−04	14	4.88E−04	14
NQO1	NAD(P)H quinone dehydrogenase 1	0.00275464	14	0.0074225	17
SERPINB2	Serpin family B member 2	2.35E−04	13	2.09E−04	20
IL24	Interleukin 24	0.0029033	11	9.61E−04	17
NOS1	Nitric oxide synthase 1	0.00371168	10	9.83E−04	13
S100A7	S100 calcium binding protein A7	0.00322041	9	0.00362133	14
CYP1B1	Cytochrome P450 family 1 subfamily B member 1	8.45E−04	8	4.23E−04	15
EREG	Epiregulin	1.00E−04	7	6.22E−05	10
KRT10	Keratin 10	1.50E−04	3	2.11E−04	5

### Integrative analysis of literature and experiment-based results in terms of differentially expressed signals and PM component association to identify biological alterations in the skin caused by PM

**Figure 4:**
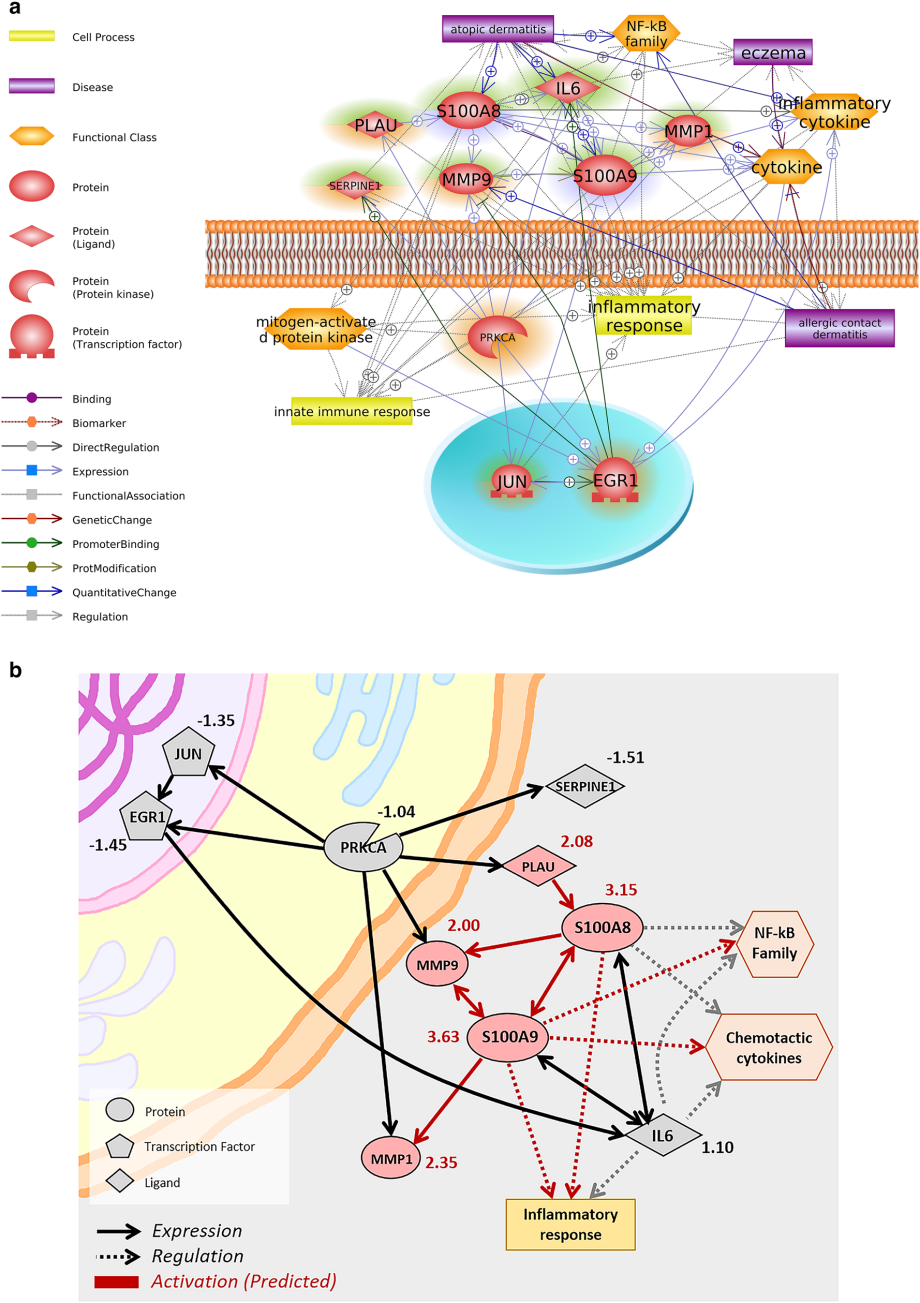

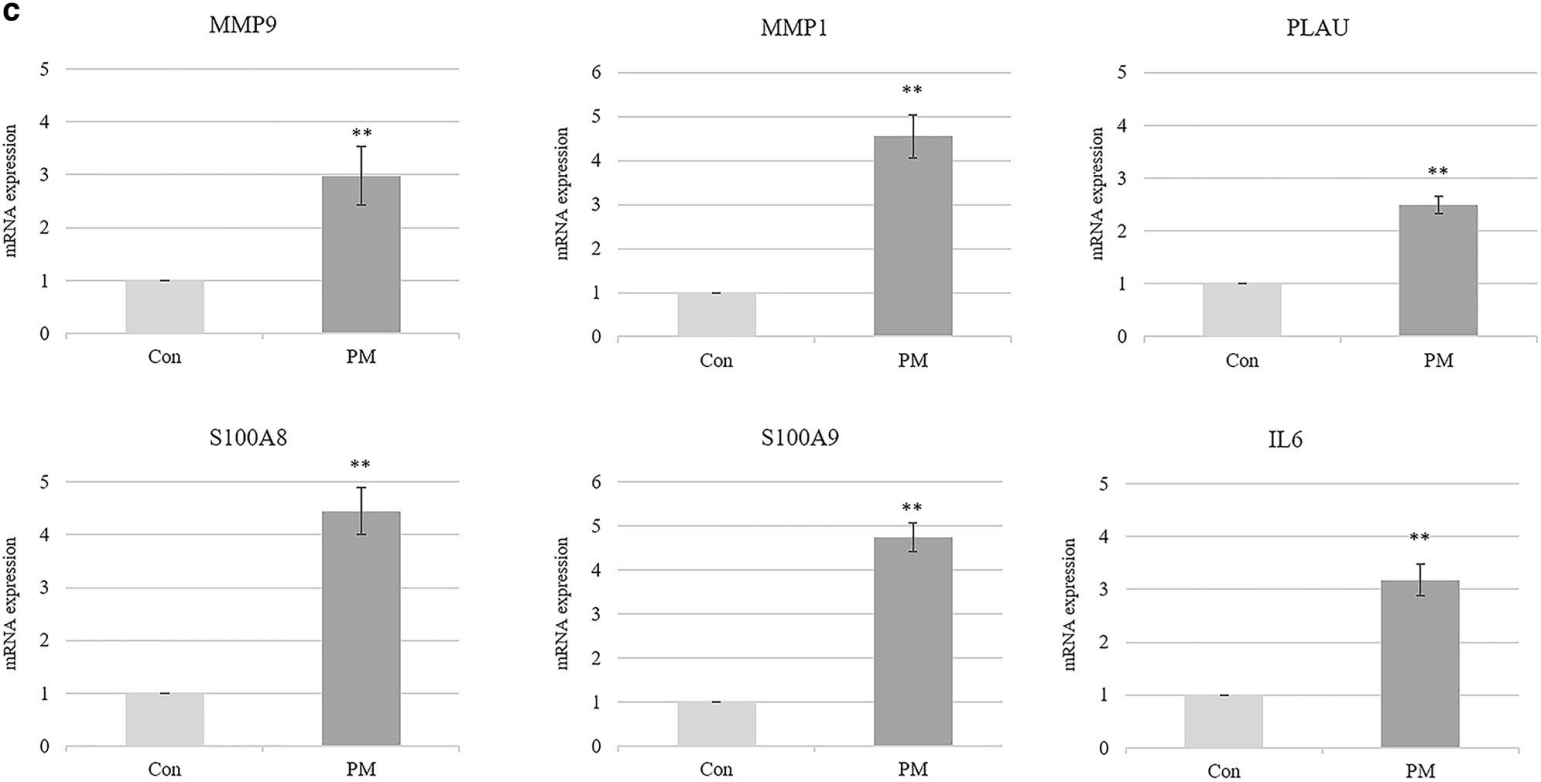
Integrative identification of potential mechanisms and key regulators involved in PM-induced skin diseases. (**a**) Simplified pathway relevant to PM-induced skin diseases (refer to public data-based network in Fig. 2). Genes highlighted in orange, violet, and green, respectively, indicate atopic dermatitis-associated, allergic contact dermatitis-associated, and eczema-associated. Descriptions of the schematic symbols are located to the left of the figure. (**b**) Overlap with fold-change values from our RNA-seq experiments to identify activation/inhibition patterns. Criteria for differential expression is *p* < 0.05 and |fold-change| > 2. Upregulated genes are highlighted in pink. Schematic legends are located to the left of the figure. (**c**) Validation of mRNA expression profiles of key regulators using qRT-PCR. The upregulation of *MMP9*, *MMP1*, *S100A8*, *S1009*, and *PLAU* was confirmed. *IL6* did not show significant fold-changes in RNA-seq, but changes in RNA expression levels were confirmed by qRT-PCR.

We simplified the literature-based network from Fig. 2 to select the final potential key networks that elucidate the etiological relationships between PM and skin diseases (Fig. 4a). All entities were selected based on the betweenness and degree centrality with surrounding nodes in the pathway (Table 2 and Supplementary Table S1). The top 10 genes with high degree values were selected as key genes for simplification. The relation between the selected key genes and centrality is presented in Supplementary Table S1. “Inflammatory cytokine” and “NF-κB family” were predicted as the major functional classes, and “inflammatory response” was predicted as the major cell process closely related to PM-induced skin diseases. Each key regulator was highlighted based on gene–disease associations referred to in our retrieved dataset. S100A8, S100A9, and IL-6 were predicted as important hubs in the pathway, with relationships between more than two environmental skin diseases.

For integrative prediction about the potential mechanism of PM-induced environmental skin diseases, we overlapped activation/inhibition patterns in fold-change values from our next-generation sequencing (NGS)-based experimental data with the result of our literature-based analysis. Activation of inflammatory responses by altered NF-κB family and inflammatory cytokine functions were predicted based on the up-regulation of PLAU, MMP-9, MMP-1, S100A8, and S100A9 (Fig. 4b), which we verified by quantitative real-time PCR (qRT-PCR). All five genes showed significantly upregulated expression under PM_2.5_ exposure compared to the control group (Fig. 4c). Interestingly, significant changes in mRNA expression levels of IL-6 were confirmed by qRT-PCR, and expression alteration of IL-6 at the protein level was validated by enzyme-linked immunosorbent assays (ELISA) (Supplementary Fig. S2), although fold-change values of IL-6 were not detected in the RNA-seq experiment.

### Integrative prediction of biological alterations and upstream regulators in skin exposed to PM

**Figure 5:**
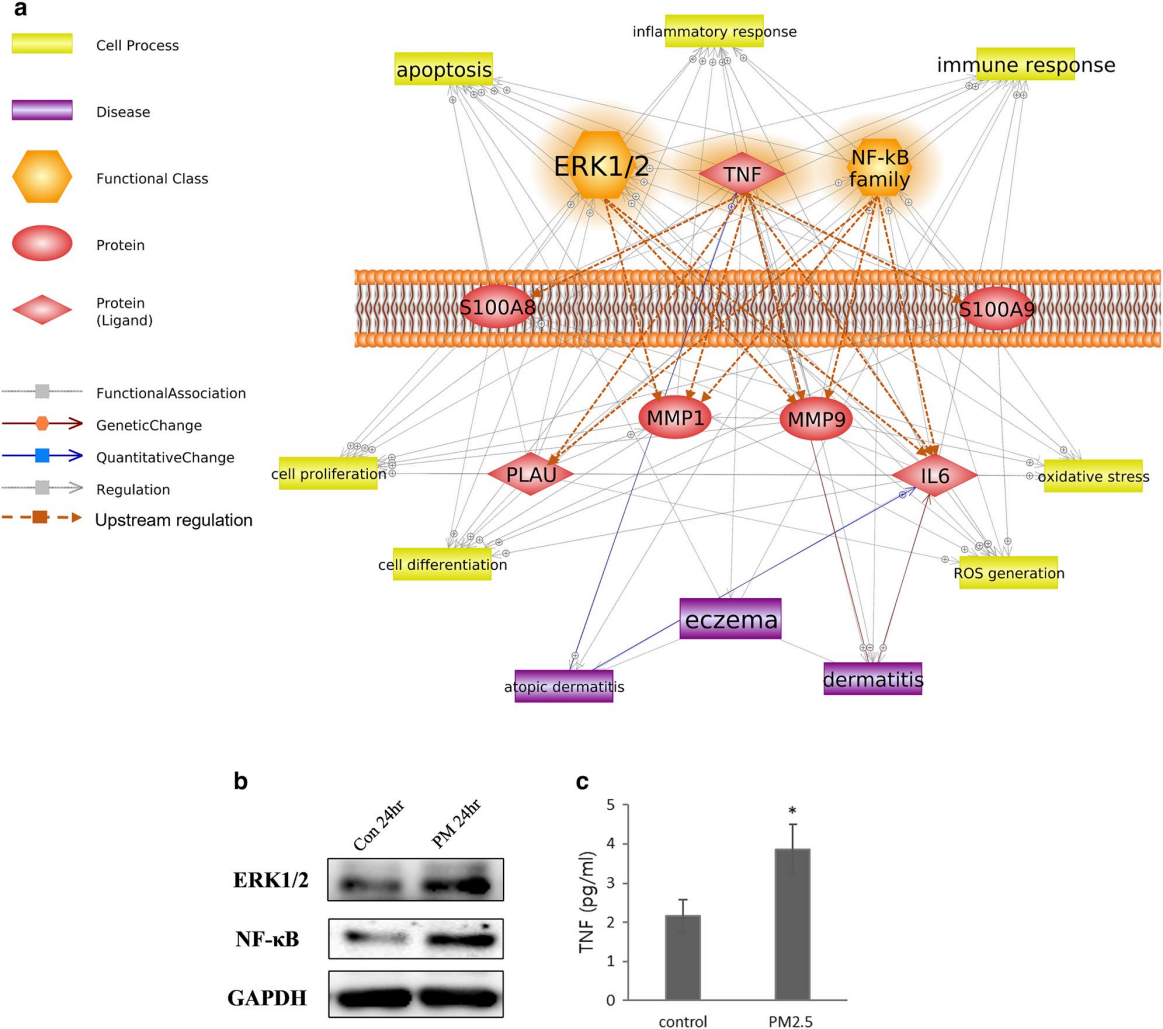
Integrative identification through simplified signaling network of upstream regulators and hub genes altered in skin under PM exposure. (**a**) PM-induced biological pathway consisting of hub genes, upstream regulators, cell processes, and disease. Genes highlighted in orange indicate upstream regulators. (**b**) Validation at the protein level was performed through Western blot. The expressions of NF-κB and ERK1/2 changed, as predicted. **c** ELISA-based validation of upstream regulator proteins. TNF expression changed significantly, as predicted. * indicates 0.001 < *p* < 0.05.

Based on the association obtained from the integrative analysis, we explored significant upstream regulators for the key genes using upstream analysis algorithm in Ingenuity Pathway Analysis (IPA) software (Table 4). Among the predicted regulators, TNF, NF-κB, and ERK1/2 formed significant networks with the six key genes. Final key networks were summarized in Fig. 5a, which showed biological alteration in skin regulated by PM-induced functional activation of TNF, NF-κB, and ERK1/2. Protein level change of upstream regulators were verified using Western blot or ELISA assay, it was found that the expression patterns were significantly changed, as predicted in the upstream analysis (Fig. 5b,c, and Table 4).

**Table 4:** Upstream regulators identified from intersection between public data and NGS experimental data under PM exposure. Bold texts indicate the hub genes consonant with Fig. 5.

Upstream regulator	Activation z-score	p-value of overlap	Target molecules in dataset
TNF	2.403	1.96E−14	CXCL1, GBP1, **IL6**, **MMP1**, **MMP9**, **PLAU**, **S100A8**, **S100A9**, SERPINB2
CD36	2.236	7.12E−12	CXCL1, **IL6**, **MMP1**, **PLAU**, SERPINB2
NFkB (complex)	2.376	5.32E−10	CXCL1, **IL6**, **MMP1**, **MMP9**, **PLAU**, SERPINB2
ERK	2.219	7.98E−10	CXCL1, **IL6**, **MMP1**, **MMP9**, SERPINB2
IL1B	2.188	6.52E−08	CXCL1, **IL6**, **MMP1**, **MMP9**, SERPINB2
TP53	− 1.913	0.00024	EGR1, **IL6**, **MMP9**, SERPINE1

## Discussion

In recent years, biological evidence supporting the role of PM in skin damage has been suggested via various approaches. Numerous epidemiological studies have reported a phenomenological association between the increase in airborne PM level and diagnosis frequency of skin diseases. Although characteristic variables of PM depend on weather or location, studies have commonly reported associations between ambient PM and progression of inflammatory skin symptoms or diseases, such as eczema, allergic contact dermatitis, and atopic dermatitis^14,16,18,19^. However, the underlying mechanism for these associations was not fully understood.

The size and component characteristics of PM are closely related to the source of the PM^8,20^. PM released directly from natural sources (e.g., crustal movement, dust storms, forest fires, and weathering of geographical features) and micro-sized biological particles (e.g., bacteria endotoxins, pollen, and spores) are classified as primary particles (mostly PM_10_)^21^. Anthropogenic sources (e.g., solid fuel combustion, attrition of brakes and tires on urban roads, and erosion during manufactural processes) are major causes of micro-sized solid chemical particles and gas or liquid particles in urban PM^22,23^. Most of the PM that originates from anthropogenic sources is derived from chemical reactions between oxides of sulfur and nitrogen, VOC, PAH, and other chemical derivatives of primary particles. Being mostly smaller than primary particles, this PM belongs to PM_2.5_. All PM types consist of organic carbon, elemental carbon, PAH, VOC, and metals^24,25^. The individual toxicity of each component has been widely studied; inhaled or penetrated ambient PM-sized heavy metals can accumulate in the body and stimulate chronic illnesses, including bronchial damage, lung malfunction, or skin carcinogenesis^26,27^. Organic components, such as PAH and their oxygenated derivatives, cause severe oxidative stress and mitochondrial damage^28,29^. However, in terms of heterogeneous particles, the contribution of each component to the PM-induced adverse effects is not fully understood. Here, we analyzed the contribution of chemical components in the biological pathway of PM-induced skin diseases (Supplement Fig. S3). Identified genes in the pathway were sorted and marked based on knowledge of chemical–gene associations from our retrieved dataset in Table 1. PAHs and cadmium are the largest contributors to the pathway. MMP1, PLAU, SERPINB2, ITGA2, and CFB are commonly associated with the largest number of PM components and are closely related closely to ‘psoriasis,’ ‘melanoma,’ ‘dermatitis,’ ‘wounds,’ and ‘wounds and injuries.’ This suggestion has limitations that arise from inadequate keywords of PM components and the required validation for specifying components “in PM.” However, our approach provides important clues for clarifying the comprehensive toxic effects and main causes of skin disorders from PM exposure by considering comparative data on the differential contribution of the components to each mode of action.

The rapid evolution of NGS has accelerated research in genomics field by providing a massive amount of data. Researchers can quickly and easily access data through various public databases and utilize genome-wide gene expression profiling for their studies. In interpreting the dynamic pattern of gene expression profiles, co-expression network analysis for exploring gene functions and gene–disease associations have emerged as respective data-analysis methods^17^. In the present study, we aimed to integrate previous knowledge from literature-based data with our experimental data to identify the relationship between PM exposure and skin diseases. To interpret/expand the biological meaning from the list of genes associated with the chosen keywords (Table 1), we applied text-mining software-based pathway analysis among the identified genes to obtain their co-expression network, related functional processes, and disease-related phenotypes. During the construction of the pathway, each gene was referred to as an “entity” and linked to another entity by “relation,” which represents the biological relationship between two entities. A relation between two entities can be established by screening the sentences in massive volumes of scientific literature based on co-occurrence frequency in the same scientific publications^30^. The importance of each entity was determined by centrality concepts. Betweenness centrality quantifies the shortest path between adjacent entities, and degree centrality defines the number of relations between adjacent entities^31^. Higher values of these two parameters in the pathway explain the crucial point of interaction among multiple biological networks. We considered both values to cover connectivity with surrounding entities and the role of the hub genes in the predicted pathway, which shows the etiological relationship between PM and skin diseases.

From the experimental perspective, several in vitro studies have elucidated that PM-induced cytotoxicity may derive from activation of the IL-1β, IL-6, and NF-κB signaling pathways in keratinocytes^32–34^. However, cell line-based studies have critical weak points in that they do not account for the systemic response in the skin. Thus, recent publications have attempted to demonstrate PM-induced skin damage with consideration of the comprehensive profile of macrophenomena aspects and molecular mechanisms using both in vitro and in vivo models or artificial three-dimensional skin tissue models. Such studies have suggested a significant alteration of several specific inflammatory markers, such as IL-1α, IL-8, or oxidative stress-induced NF-κB nucleus translocation^7,35,36^. With this consideration, we attempted to identify the comprehensive biological responses associated with PM-induced skin toxicity, as well as marker-specific knowledge, by interpreting the interactions among the entities and surrounding molecular networks, as shown in Figs. 2 and 3. From the public database (Fig. 2a) and experimental data (Fig. 3a), the NF-κB family and cytokines were commonly predicted as possibly altered cellular functions in the skin under PM exposure (Fig. 4a). In addition, inflammatory response-related cell processes mediated by MMP-9, S100A8, S100A9, and IL-6 were commonly predicted (Figs. 2b and 3b). These results reflect the previously mentioned data from various in vivo and/or in vitro approaches to demonstrate dermal toxicity of PM and provide improved information at the pathway level. All genes and their target proteins play individual and collective roles by interacting with each other. Our results suggest that PM exposure causes an inflammatory response mediated by alteration of the NF-κB family and cytokine functions through differential expression of MMP-9, S100A8, S100A9, and IL-6.

The inflammatory response is a complex and rapid biological process induced by extrinsic irritants^37^. The primary purpose of the inflammatory response is to defend the system against injurious stimuli^38^. Exposure to a toxicant or pathogen can cause a response that leads to tissue-level pathological conditions because of uncontrolled, excessive activation of the immune system. Inflammatory and immune responses induced by airborne toxicants are a major inducer of drastic adverse effects in skin cells during penetration of extrinsic irritants through skin. IL-6, S100A8, and S100A9 are three key regulators that display a positive biological relationship with atopic dermatitis (Fig. 4a) and serve as major inflammatory markers under PM exposure. IL-6 is a well-known cellular stress and pro-inflammatory marker that is overexpressed after exposure to various extrinsic harmful substances^39^. It is also actively studied as a marker for skin diseases, such as atopic dermatitis and allergic dermatitis^40^. S100A8 and S100A9 belong to the S100 protein family and are released to the acellular compartment, where they bind cell surface receptors and could act as major regulators of the inflammatory response^41^. One of those receptors is Toll-like receptor 4, TLR-4^42^. Upon binding to TLR-4, signaling cascades are initiated that regulate inflammation and NF-κB-dependent tumor development^42–44^.

The activation pattern of the summarized pathway involved in PM-induced skin diseases was predicted using fold-change values from RNA-seq data analysis of human epidermal keratinocytes. Owing to the essential roles of keratinocytes in immune responses to exposure and penetration of extrinsic factors^45^, the epidermal keratinocyte model is widely used to determine the detrimental effects of PM^33,46^. This experimental procedure allowed validation of the relevance between PM exposure and the changes in the levels of key regulators in skin cells, as predicted from the integrative pathway analysis. The up-regulation of matrix metalloproteins (MMP) plays an important role in the skin and modulation of inflammation. MMP-1 intermediates cleave fibrillar collagens and contribute to collagen degradation and extracellular matrix remodeling in keratinocytes^47,48^. MMP-9 also cleaves extracellular matrix components and causes skin inflammation via activation of cytokines, including IL-1β and IL-13^49^. The direct association between upregulated PLAU and the skin or PM has not been fully understood, but it is closely related to MMP-1 and MMP-9. Upregulated urokinase-type plasminogen activator—the enzyme encoded by the *PLAU* gene—causes plasmin-dependent activation of MMP via catalysis-mediated conversion of plasminogen to plasmin^50^. This knowledge revealed predictable roles for additional key genes in PM-induced skin diseases. Based on the experimental validation using our NHEK sample (Fig. 4c), not only are the key regulators differentially expressed in experimental data, but another gene indicated changes in expression levels not included in the DEG data. In this regard, the integrative approach for screening/exploring the potential association between environmental factors and diseases was informative in terms of important aspects that researchers might have missed if only the public or experimental data had been considered.

Here, we explored the adverse effects of PM on the skin and attempted to elucidate their relationships using public genome data. Through the literature-based biological pathway analysis of gene expression data from a public database, we identified the chemical–gene–disease associations of PM-induced environmental skin diseases. Through upstream analysis and validation, changes in biological functions and cellular processes of cytokines elicited by the inflammatory response were predicted as the major contributors of adverse outcomes, and expression level changes of key regulators in the pathway were validated (Fig. 5b,c). Further mechanism studies will be required to demonstrate the exact molecular interaction. However, our results provide evidence to assist in clarifying the underlying mechanism of ambient PM-induced dermal toxicity and exemplify the unconventional approach to screening the biological relationships between chemicals and diseases.

## Materials and methods

### Collection of global gene expression profiles from public databases

We proceeded with keyword selection to retrieve chemical–gene–disease associations from public databases. The genomic data were collected in three categories: “PM size,” “PM components,” and “Skin disease.” First, cadmium, lead, PAH, and VOC were selected as the major chemical components of PM in view of their frequent or common mention in numerous papers to eliminate possible variation in chemical composition because of geographical or time-based factors^51–54^. “Coal Ash” was also added to expand the informal definition of PM provided in the Comparative Toxicogenomics Database (CTD). DEG were collected from the CTD (http://ctdbase.org/, last access date: September 2019), a literature-based public resource that provides curated information about chemical–gene/protein interactions and chemical–gene–disease relationships^55^. Second, PM size was integrated into the analysis by directly searching the keywords “PM_10_” and “PM_2.5_.” Gene expression data concerning PM size were obtained from research publications in the PubMed database, and the collection period was from 2018 to 2019. Finally, “atopic dermatitis,” “allergic dermatitis,” and “eczema” were selected as keywords for collecting gene expression data involving environmental skin diseases. The Gene Expression Omnibus (GEO) (https://www.ncbi.nlm.nih.gov/geo/, last access date: October 2019), a public functional genomics data repository, was used for collecting the disease-specific gene set analyzed from human-based research publications. All data in the dataset were trimmed based on *p*-value < 0.05, and |fold-change| > 2. The workflow scheme of the data crawling is provided in Fig. 1.

### Chemical preparation and cell treatment

Ambient PM_2.5_ was collected on a polytetrafluoroethylene (PTFE) filter (Zefluor™, Pall Life Sciences, Mexico City, Mexico) with a high-volume sampler machine (TE6070, Tisch Environmental, Inc., Cleves, OH, USA), equipped with a PM_2.5_ selective-inlet head at a flow rate of 1.13 m^3^/min. The sample collection of PM_2.5_ was carried out on the rooftop of the Amorepacific Corporation R&D building, located in Yongin, Korea (37°15′N, 127°06′E). PM_2.5_ was extracted with ethanol (EtOH) in a sonicator for 30 min. The obtained extract was dried using an evaporator and resuspended with 20% EtOH. NHEK from neonatal foreskin (Lonza, Walkersville, MD, USA) were cultured in keratinocyte growth medium (KBM-GOLD) with SingleQuots™ supplement (Lonza) containing hydrocortisone, transferrin, epinephrine, gentamicin/amphotericin B, bovine pituitary extract, human epidermal growth factor, and insulin. NHEK were starved for 24 h in KBM-GOLD medium with gentamicin/amphotericin B, followed by stimulation with PM_2.5_ (100 μg/mL, 95% cell viability) for 24 h. All experiments were performed with cells passaged less than three times.

### RNA-seq and identification of DEG

Transcriptome analysis using RNA-seq was performed by Macrogen, Inc. (Seoul, Korea). RNA was extracted using an RNeasy mini kit (Qiagen, Hilden, Germany) following the manufacturer’s instructions. The quality of RNA samples was checked using the Agilent 2100 Bioanalyzer (Agilent Technologies, Inc., Santa Clara, CA, USA). Libraries were generated with the TruSeq Stranded mRNA Prep Kit (Part #15031047 Rev. E). Purified mRNA was fragmented, and pair-end RNA-seq was conducted using a HiSeq 2500 (Illumina, San Diego, CA, USA) sequencing system. TruSeq Stranded mRNA LT Sample Prep kits (Illumina) were used to establish libraries according to the sample preparation guide. To determine the RNA expression profiles, the RNA-seq reads were mapped to a human reference genome (hg19) using HISAT2^56^. Human reference genome sequence and annotation data were downloaded from the University of California, Santa Cruz (UCSC) Genome Browser (http://genome.uscs.edu). Mapped reads were assembled using StringTie^57^. Transcript counts were calculated at the isoform and gene levels, and the relative transcript abundances were measured in Fragments Per Kilobase of exon per Million fragments mapped (FPKM). *P*-value < 0.05 and |fold-change| ≥ 2 were considered as criteria for DEG. All RNA-seq datasets used are available at the GEO repository: GSE143709. RNA samples and identified DEG were used in further analyses, as illustrated in Fig. 1.

### Biological network analysis using retrieved gene set and overlapping with experimental data

Pathway Studio web 12.1.0.9 (Elsevier), a literature-based software, was used to analyze biological networks among the identified gene set. Pathway Studio contains a curated literature database based on its own text-mining module, which extracts relevant sentences concerning the relationship between two entities^58^. It provides molecular interactions among the entered genes, as well as an investigation of protein–protein or protein–cell process interaction maps. The relations with less than five references were excluded for analysis. The description of each relation type between entities is as follows: *Binding*: physical contact between two molecules; *DirectRegulation*: influences target activity by direct physical interaction; *Expression*: regulator changes protein abundance by affecting levels of transcript or protein stability; *ProtModification*: regulator that changes the modification of the target molecule; *PromoterBinding*: regulator that binds to the promoter of a gene^30^. The importance of the entities in the pathway was analyzed according to their betweenness and degree centrality, which were calculated using NetworkAnalyzer in Cytoscape 3.7.2 software. IPA (Qiagen) was utilized to perform upstream analysis. IPA is a software application that provides comprehensive biological knowledge and predictions relevant to entered gene expression data based on their curated database via a data mining interface^59^. The upstream analysis function enables the prediction of the upstream transcriptional regulators linked to observed gene expression changes in the signaling networks.

### qRT-PCR

cDNA was synthesized using the ImProm-II™ Reverse Transcription System (Promega, Madison, WI, USA) from 500 μg of extracted RNA following the manufacturer’s instructions. qRT-PCR was conducted in the Rotor-Gene Q Real-Time PCR system (Qiagen) using Takara SYBR Premix Ex Taq (Takara Bio, Inc., Japan). Thermal cycling conditions for PCR included an initial denaturation step at 95 °C for 5 min, followed by 40 cycles of denaturation at 95 °C for 30 s, annealing at 55 °C for 30 s, and extension at 72 °C for 30 s. Melting curve analysis of the PCR products was performed at the end of the PCR step, and the data were analyzed using the Rotor-Gene Q Real-Time PCR system (Qiagen). Glyceraldehyde-3-phosphate dehydrogenase-encoding gene (GAPDH) was used to normalize the relative level of gene expression using the 2^−ΔΔ*C*T^ method. The sequences of the primers used for qRT-PCR are shown in Supplementary Table S2.

### Western blot and ELISA assay

Total proteins of NHEK cells were extracted with RIPA buffer containing 1 mM DTT and 1 U of EDTA-free protease inhibitor cocktail (Roche, Manheim, Germany). An equal amount of protein was separated on 7.5% SDS-PAGE and then transferred onto polyvinylidene fluoride membranes. The membranes were blocked using 5% skim milk in TBS at 25 °C for 2 h and incubated with specific primary antibodies: anti-GAPDH (2188, Cell Signaling, MA, USA), anti-NF-κB (3035, Cell Signaling), and anti-ERK1/2 (sc-514302, Santa Cruz Biotechnology, CA, USA) at 4 °C overnight. Membranes were incubated with secondary antibodies (HRP-linked IgG) at 25 °C for 1 h. The secondary antibodies were anti-mouse (A90-116P, Bethyl Laboratories, Montgomery, TX) and anti-rabbit (A120-101P, Bethyl Laboratories). Membranes were washed with TBST, and proteins were detected using enhanced chemiluminescence (ECL) Prime Western Blotting Detection Reagent (GE Healthcare, Piscataway, NJ, USA). The GAPDH band was used as a loading control.

The supernatants of NHEK cells were collected, and TNF (TNF-alpha Human ELISA Kit, High Sensitivity, BMS223HS) and IL-6 (IL-6 Human ELISA Kit, High Sensitivity, BMS213HS) were measured using commercial ELISA kits (Invitrogen, CA, USA) in accordance with the manufacturer’s protocols.

### Statistical analysis

All data were obtained from at least three independent experiments conducted in triplicate. All graph data indicate mean ± standard error. Differences between experimental groups were evaluated using the Student’s *t*-test, and comparisons among more than two groups were analyzed using ANOVA. *P*-values < 0.05 or < 0.01 were considered statistically significant.

## Supplementary Information

Supplementary Information 1.

Supplementary Information 2.

Supplementary Information 3.

Supplementary Information 4.

Supplementary Information 5.

Supplementary Information 6.

Supplementary Information 7.

Supplementary Figures.

Supplementary Tables.

Supplementary Information 8.

Supplementary Information 9.
