# Supplementary figures and images for: Integrative analysis to explore the biological association between environmental skin diseases and ambient particulate matter
Source: Sci Rep. 2022 Jun 13;12:9750. doi: 10.1038/s41598-022-13001-x (PMC9192598; doi:10.1038/s41598-022-13001-x)

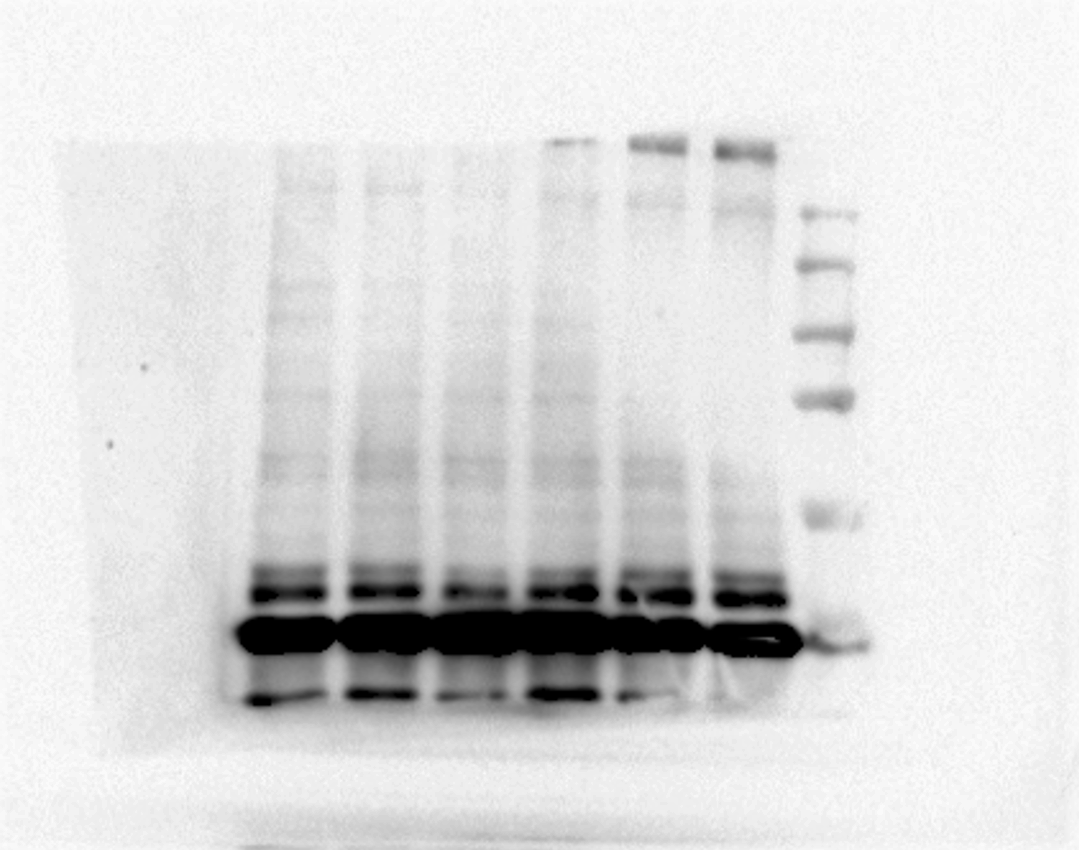

Supplement: Supplementary file 1 — Supplementary Information 1. [file 41598_2022_13001_MOESM1_ESM.tif]

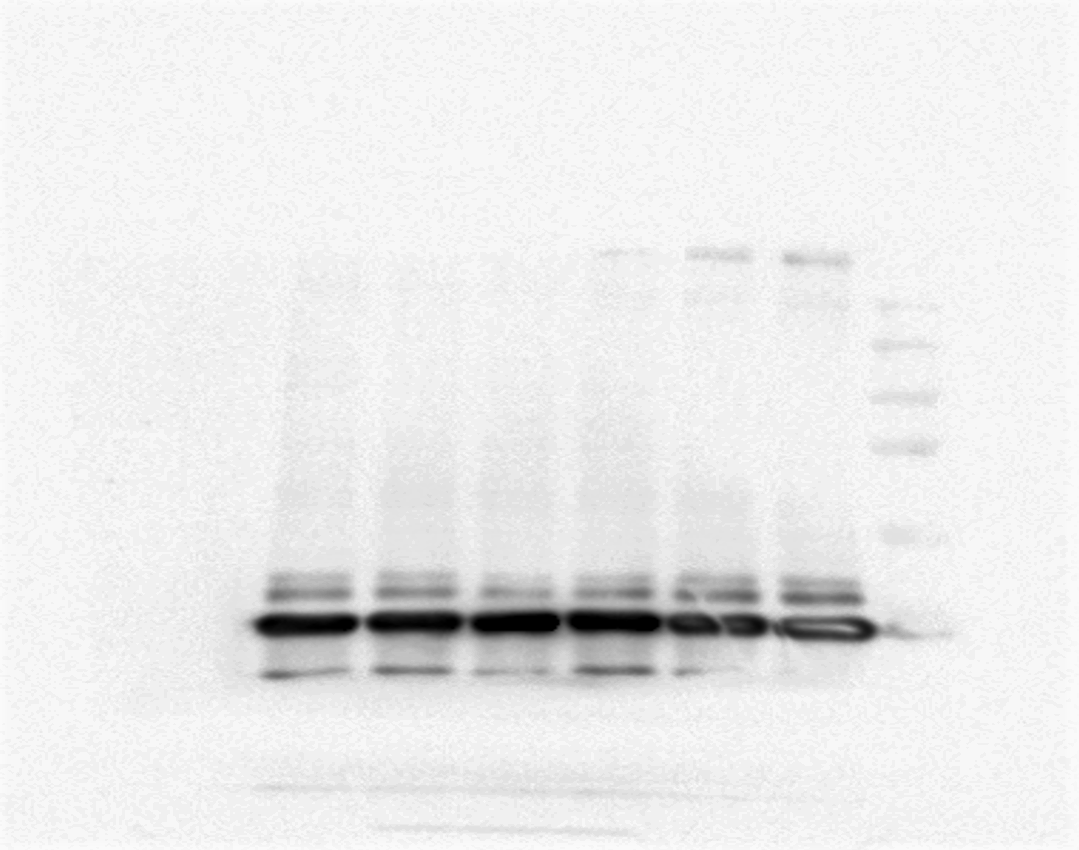

Supplement: Supplementary file 2 — Supplementary Information 2. [file 41598_2022_13001_MOESM2_ESM.tif]

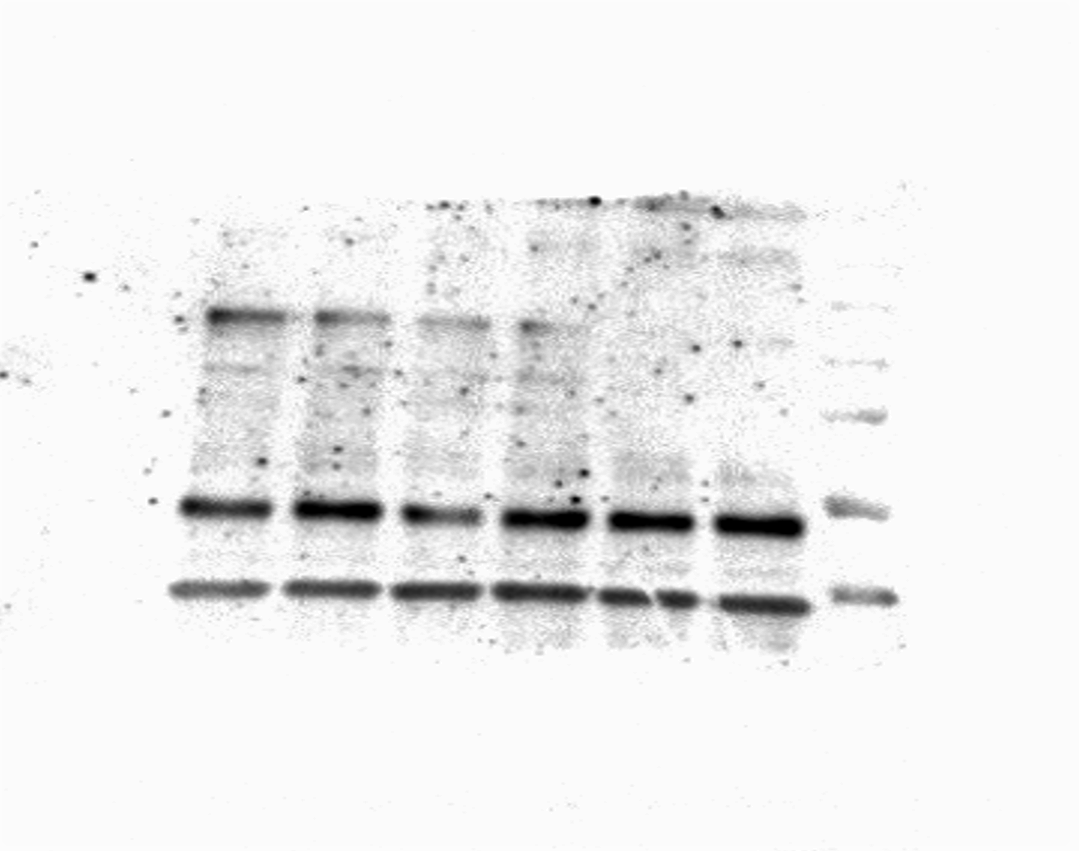

Supplement: Supplementary file 3 — Supplementary Information 3. [file 41598_2022_13001_MOESM3_ESM.tif]

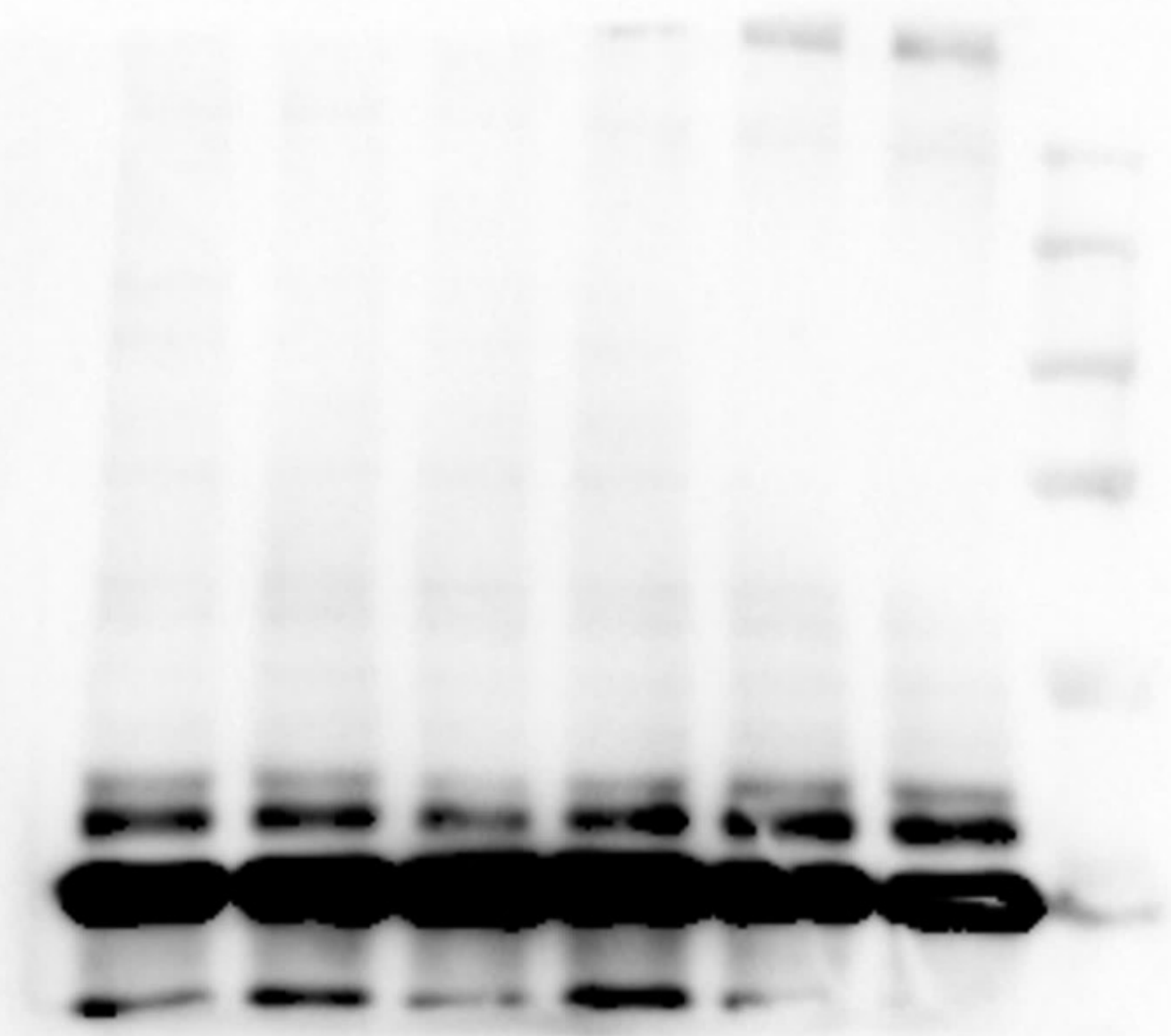

Supplement: Supplementary file 4 — Supplementary Information 4. [file 41598_2022_13001_MOESM4_ESM.pdf]

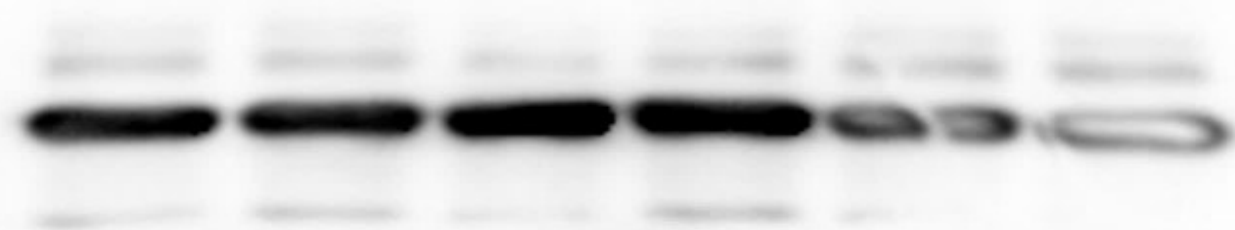

Supplement: Supplementary file 5 — Supplementary Information 5. [file 41598_2022_13001_MOESM5_ESM.pdf]

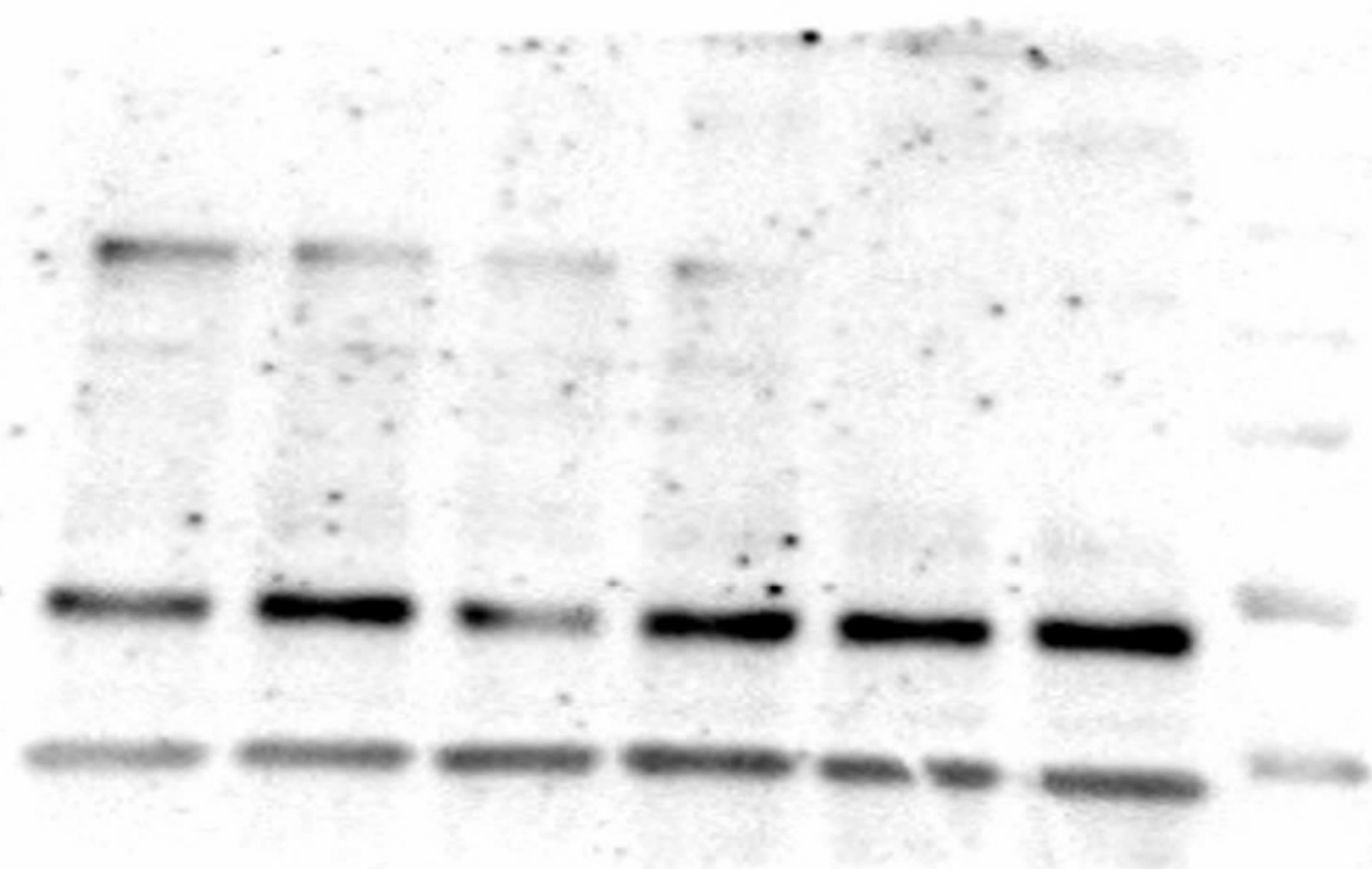

Supplement: Supplementary file 6 — Supplementary Information 6. [file 41598_2022_13001_MOESM6_ESM.pdf]
