# Supplementary material for: Integrative analysis to explore the biological association between environmental skin diseases and ambient particulate matter
Source: Sci Rep. 2022 Jun 13;12:9750. doi: 10.1038/s41598-022-13001-x (PMC9192598; doi:10.1038/s41598-022-13001-x)
Supplement: Supplementary file 7 — Supplementary Information 7. [file 41598_2022_13001_MOESM7_ESM.pdf]

Keywords	Gene symbol	Data type	Platform	Series #	reference title	Journal
allergic contact dermatitis	SELE	Expression profiling by array, count, 3 development stage, 2 disease state, 11 individual sets	GPL570	GSE6281	Gene Expression Time Course in the Human Skin during Elicitation of Allergic Contact Dermatitis	J Invest Dermatol.
allergic contact dermatitis	RGS1	Expression profiling by array, count, 3 development stage, 2 disease state, 11 individual sets	GPL570	GSE6281	Gene Expression Time Course in the Human Skin during Elicitation of Allergic Contact Dermatitis	J Invest Dermatol.
allergic contact dermatitis	LTB	Expression profiling by array, count, 3 development stage, 2 disease state, 11 individual sets	GPL570	GSE6281	Gene Expression Time Course in the Human Skin during Elicitation of Allergic Contact Dermatitis	J Invest Dermatol.
allergic contact dermatitis	CCR7	Expression profiling by array, count, 3 development stage, 2 disease state, 11 individual sets	GPL570	GSE6281	Gene Expression Time Course in the Human Skin during Elicitation of Allergic Contact Dermatitis	J Invest Dermatol.
allergic contact dermatitis	CCL19	Expression profiling by array, count, 3 development stage, 2 disease state, 11 individual sets	GPL570	GSE6281	Gene Expression Time Course in the Human Skin during Elicitation of Allergic Contact Dermatitis	J Invest Dermatol.
allergic contact dermatitis	TRBV19	Expression profiling by array, count, 3 development stage, 2 disease state, 11 individual sets	GPL570	GSE6281	Gene Expression Time Course in the Human Skin during Elicitation of Allergic Contact Dermatitis	J Invest Dermatol.
allergic contact dermatitis	TRBV19	Expression profiling by array, count, 3 development stage, 2 disease state, 11 individual sets	GPL570	GSE6281	Gene Expression Time Course in the Human Skin during Elicitation of Allergic Contact Dermatitis	J Invest Dermatol.
allergic contact dermatitis	CD3D	Expression profiling by array, count, 3 development stage, 2 disease state, 11 individual sets	GPL570	GSE6281	Gene Expression Time Course in the Human Skin during Elicitation of Allergic Contact Dermatitis	J Invest Dermatol.
allergic contact dermatitis	GBP1	Expression profiling by array, count, 3 development stage, 2 disease state, 11 individual sets	GPL570	GSE6281	Gene Expression Time Course in the Human Skin during Elicitation of Allergic Contact Dermatitis	J Invest Dermatol.
allergic contact dermatitis	IL2RG	Expression profiling by array, count, 3 development stage, 2 disease state, 11 individual sets	GPL570	GSE6281	Gene Expression Time Course in the Human Skin during Elicitation of Allergic Contact Dermatitis	J Invest Dermatol.
allergic contact dermatitis	GBP1	Expression profiling by array, count, 3 development stage, 2 disease state, 11 individual sets	GPL570	GSE6281	Gene Expression Time Course in the Human Skin during Elicitation of Allergic Contact Dermatitis	J Invest Dermatol.
allergic contact dermatitis	IL32	Expression profiling by array, count, 3 development stage, 2 disease state, 11 individual sets	GPL570	GSE6281	Gene Expression Time Course in the Human Skin during Elicitation of Allergic Contact Dermatitis	J Invest Dermatol.
allergic contact dermatitis	IFI30	Expression profiling by array, count, 3 development stage, 2 disease state, 11 individual sets	GPL570	GSE6281	Gene Expression Time Course in the Human Skin during Elicitation of Allergic Contact Dermatitis	J Invest Dermatol.
allergic contact dermatitis	PSMB10	Expression profiling by array, count, 3 development stage, 2 disease state, 11 individual sets	GPL570	GSE6281	Gene Expression Time Course in the Human Skin during Elicitation of Allergic Contact Dermatitis	J Invest Dermatol.
allergic contact dermatitis	LILRB2	Expression profiling by array, count, 3 development stage, 2 disease state, 11 individual sets	GPL570	GSE6281	Gene Expression Time Course in the Human Skin during Elicitation of Allergic Contact Dermatitis	J Invest Dermatol.
allergic contact dermatitis	PSMB8	Expression profiling by array, count, 3 development stage, 2 disease state, 11 individual sets	GPL570	GSE6281	Gene Expression Time Course in the Human Skin during Elicitation of Allergic Contact Dermatitis	J Invest Dermatol.
allergic contact dermatitis	PSME1	Expression profiling by array, count, 3 development stage, 2 disease state, 11 individual sets	GPL570	GSE6281	Gene Expression Time Course in the Human Skin during Elicitation of Allergic Contact Dermatitis	J Invest Dermatol.
allergic contact dermatitis	LILRA2	Expression profiling by array, count, 3 development stage, 2 disease state, 11 individual sets	GPL570	GSE6281	Gene Expression Time Course in the Human Skin during Elicitation of Allergic Contact Dermatitis	J Invest Dermatol.
allergic contact dermatitis	SELL	Expression profiling by array, count, 3 development stage, 2 disease state, 11 individual sets	GPL570	GSE6281	Gene Expression Time Course in the Human Skin during Elicitation of Allergic Contact Dermatitis	J Invest Dermatol.
allergic contact dermatitis	CORO1A	Expression profiling by array, count, 3 development stage, 2 disease state, 11 individual sets	GPL570	GSE6281	Gene Expression Time Course in the Human Skin during Elicitation of Allergic Contact Dermatitis	J Invest Dermatol.
allergic contact dermatitis	ARPC1B	Expression profiling by array, count, 3 development stage, 2 disease state, 11 individual sets	GPL570	GSE6281	Gene Expression Time Course in the Human Skin during Elicitation of Allergic Contact Dermatitis	J Invest Dermatol.
allergic contact dermatitis	RAB31	Expression profiling by array, count, 3 development stage, 2 disease state, 11 individual sets	GPL570	GSE6281	Gene Expression Time Course in the Human Skin during Elicitation of Allergic Contact Dermatitis	J Invest Dermatol.
allergic contact dermatitis	CXCR4	Expression profiling by array, count, 3 development stage, 2 disease state, 11 individual sets	GPL570	GSE6281	Gene Expression Time Course in the Human Skin during Elicitation of Allergic Contact Dermatitis	J Invest Dermatol.
allergic contact dermatitis	JAK3	Expression profiling by array, count, 3 development stage, 2 disease state, 11 individual sets	GPL570	GSE6281	Gene Expression Time Course in the Human Skin during Elicitation of Allergic Contact Dermatitis	J Invest Dermatol.
allergic contact dermatitis	CD3Z	Expression profiling by array, count, 3 development stage, 2 disease state, 11 individual sets	GPL570	GSE6281	Gene Expression Time Course in the Human Skin during Elicitation of Allergic Contact Dermatitis	J Invest Dermatol.
allergic contact dermatitis	CXCR4	Expression profiling by array, count, 3 development stage, 2 disease state, 11 individual sets	GPL570	GSE6281	Gene Expression Time Course in the Human Skin during Elicitation of Allergic Contact Dermatitis	J Invest Dermatol.
allergic contact dermatitis	STAT1	Expression profiling by array, count, 3 development stage, 2 disease state, 11 individual sets	GPL570	GSE6281	Gene Expression Time Course in the Human Skin during Elicitation of Allergic Contact Dermatitis	J Invest Dermatol.
allergic contact dermatitis	ECOP	Expression profiling by array, count, 3 development stage, 2 disease state, 11 individual sets	GPL570	GSE6281	Gene Expression Time Course in the Human Skin during Elicitation of Allergic Contact Dermatitis	J Invest Dermatol.
allergic contact dermatitis	IFNAR2	Expression profiling by array, count, 3 development stage, 2 disease state, 11 individual sets	GPL570	GSE6281	Gene Expression Time Course in the Human Skin during Elicitation of Allergic Contact Dermatitis	J Invest Dermatol.
allergic contact dermatitis	P2RY10	Expression profiling by array, count, 3 development stage, 2 disease state, 11 individual sets	GPL570	GSE6281	Gene Expression Time Course in the Human Skin during Elicitation of Allergic Contact Dermatitis	J Invest Dermatol.
allergic contact dermatitis	PGRMC23	Expression profiling by array, count, 3 development stage, 2 disease state, 11 individual sets	GPL570	GSE6281	Gene Expression Time Course in the Human Skin during Elicitation of Allergic Contact Dermatitis	J Invest Dermatol.
allergic contact dermatitis	TSC1	Expression profiling by array, count, 3 development stage, 2 disease state, 11 individual sets	GPL570	GSE6281	Gene Expression Time Course in the Human Skin during Elicitation of Allergic Contact Dermatitis	J Invest Dermatol.
allergic contact dermatitis	COTL1	Expression profiling by array, count, 3 development stage, 2 disease state, 11 individual sets	GPL570	GSE6281	Gene Expression Time Course in the Human Skin during Elicitation of Allergic Contact Dermatitis	J Invest Dermatol.
allergic contact dermatitis	TMSB10	Expression profiling by array, count, 3 development stage, 2 disease state, 11 individual sets	GPL570	GSE6281	Gene Expression Time Course in the Human Skin during Elicitation of Allergic Contact Dermatitis	J Invest Dermatol.
allergic contact dermatitis	TPM3	Expression profiling by array, count, 3 development stage, 2 disease state, 11 individual sets	GPL570	GSE6281	Gene Expression Time Course in the Human Skin during Elicitation of Allergic Contact Dermatitis	J Invest Dermatol.
allergic contact dermatitis	IRF1	Expression profiling by array, count, 3 development stage, 2 disease state, 11 individual sets	GPL570	GSE6281	Gene Expression Time Course in the Human Skin during Elicitation of Allergic Contact Dermatitis	J Invest Dermatol.
allergic contact dermatitis	WARS	Expression profiling by array, count, 3 development stage, 2 disease state, 11 individual sets	GPL570	GSE6281	Gene Expression Time Course in the Human Skin during Elicitation of Allergic Contact Dermatitis	J Invest Dermatol.
allergic contact dermatitis	ODC1	Expression profiling by array, count, 3 development stage, 2 disease state, 11 individual sets	GPL570	GSE6281	Gene Expression Time Course in the Human Skin during Elicitation of Allergic Contact Dermatitis	J Invest Dermatol.
allergic contact dermatitis	LAP3	Expression profiling by array, count, 3 development stage, 2 disease state, 11 individual sets	GPL570	GSE6281	Gene Expression Time Course in the Human Skin during Elicitation of Allergic Contact Dermatitis	J Invest Dermatol.
allergic contact dermatitis	PIM2	Expression profiling by array, count, 3 development stage, 2 disease state, 11 individual sets	GPL570	GSE6281	Gene Expression Time Course in the Human Skin during Elicitation of Allergic Contact Dermatitis	J Invest Dermatol.
allergic contact dermatitis	ADSL	Expression profiling by array, count, 3 development stage, 2 disease state, 11 individual sets	GPL570	GSE6281	Gene Expression Time Course in the Human Skin during Elicitation of Allergic Contact Dermatitis	J Invest Dermatol.
allergic contact dermatitis	PRPS1	Expression profiling by array, count, 3 development stage, 2 disease state, 11 individual sets	GPL570	GSE6281	Gene Expression Time Course in the Human Skin during Elicitation of Allergic Contact Dermatitis	J Invest Dermatol.
allergic contact dermatitis	ALOX5	Expression profiling by array, count, 3 development stage, 2 disease state, 11 individual sets	GPL570	GSE6281	Gene Expression Time Course in the Human Skin during Elicitation of Allergic Contact Dermatitis	J Invest Dermatol.
allergic contact dermatitis	IFT122	Expression profiling by array, count, 3 development stage, 2 disease state, 11 individual sets	GPL570	GSE6281	Gene Expression Time Course in the Human Skin during Elicitation of Allergic Contact Dermatitis	J Invest Dermatol.
allergic contact dermatitis	CAND2	Expression profiling by array, count, 3 development stage, 2 disease state, 11 individual sets	GPL570	GSE6281	Gene Expression Time Course in the Human Skin during Elicitation of Allergic Contact Dermatitis	J Invest Dermatol.
allergic contact dermatitis	GATAD1	Expression profiling by array, count, 3 development stage, 2 disease state, 11 individual sets	GPL570	GSE6281	Gene Expression Time Course in the Human Skin during Elicitation of Allergic Contact Dermatitis	J Invest Dermatol.
allergic contact dermatitis	USP31	Expression profiling by array, count, 3 development stage, 2 disease state, 11 individual sets	GPL570	GSE6281	Gene Expression Time Course in the Human Skin during Elicitation of Allergic Contact Dermatitis	J Invest Dermatol.
allergic contact dermatitis	ARHGAP9	Expression profiling by array, count, 3 development stage, 2 disease state, 11 individual sets	GPL570	GSE6281	Gene Expression Time Course in the Human Skin during Elicitation of Allergic Contact Dermatitis	J Invest Dermatol.
allergic contact dermatitis	S100A7	Expression profiling by array, count, 3 development stage, 2 disease state, 11 individual sets	GPL570	GSE6281	Gene Expression Time Course in the Human Skin during Elicitation of Allergic Contact Dermatitis	J Invest Dermatol.
allergic contact dermatitis	FKBP1A	Expression profiling by array, count, 3 development stage, 2 disease state, 11 individual sets	GPL570	GSE6281	Gene Expression Time Course in the Human Skin during Elicitation of Allergic Contact Dermatitis	J Invest Dermatol.
allergic contact dermatitis	CLIC1	Expression profiling by array, count, 3 development stage, 2 disease state, 11 individual sets	GPL570	GSE6281	Gene Expression Time Course in the Human Skin during Elicitation of Allergic Contact Dermatitis	J Invest Dermatol.
allergic contact dermatitis	HMGN4	Expression profiling by array, count, 3 development stage, 2 disease state, 11 individual sets	GPL570	GSE6281	Gene Expression Time Course in the Human Skin during Elicitation of Allergic Contact Dermatitis	J Invest Dermatol.
allergic contact dermatitis	HIST2H2BE	Expression profiling by array, count, 3 development stage, 2 disease state, 11 individual sets	GPL570	GSE6281	Gene Expression Time Course in the Human Skin during Elicitation of Allergic Contact Dermatitis	J Invest Dermatol.
allergic contact dermatitis	RICS	Expression profiling by array, count, 3 development stage, 2 disease state, 11 individual sets	GPL570	GSE6281	Gene Expression Time Course in the Human Skin during Elicitation of Allergic Contact Dermatitis	J Invest Dermatol.
allergic contact dermatitis	NRXN1	Expression profiling by array, count, 3 development stage, 2 disease state, 11 individual sets	GPL570	GSE6281	Gene Expression Time Course in the Human Skin during Elicitation of Allergic Contact Dermatitis	J Invest Dermatol.
allergic contact dermatitis	PRG1	Expression profiling by array, count, 3 development stage, 2 disease state, 11 individual sets	GPL570	GSE6281	Gene Expression Time Course in the Human Skin during Elicitation of Allergic Contact Dermatitis	J Invest Dermatol.
allergic contact dermatitis	CXORF9	Expression profiling by array, count, 3 development stage, 2 disease state, 11 individual sets	GPL570	GSE6281	Gene Expression Time Course in the Human Skin during Elicitation of Allergic Contact Dermatitis	J Invest Dermatol.
allergic contact dermatitis	PRG1	Expression profiling by array, count, 3 development stage, 2 disease state, 11 individual sets	GPL570	GSE6281	Gene Expression Time Course in the Human Skin during Elicitation of Allergic Contact Dermatitis	J Invest Dermatol.
allergic contact dermatitis	KIAA0746	Expression profiling by array, count, 3 development stage, 2 disease state, 11 individual sets	GPL570	GSE6281	Gene Expression Time Course in the Human Skin during Elicitation of Allergic Contact Dermatitis	J Invest Dermatol.
allergic contact dermatitis	FLJ20647	Expression profiling by array, count, 3 development stage, 2 disease state, 11 individual sets	GPL570	GSE6281	Gene Expression Time Course in the Human Skin during Elicitation of Allergic Contact Dermatitis	J Invest Dermatol.
allergic contact dermatitis	C6ORF129	Expression profiling by array, count, 3 development stage, 2 disease state, 11 individual sets	GPL570	GSE6281	Gene Expression Time Course in the Human Skin during Elicitation of Allergic Contact Dermatitis	J Invest Dermatol.

allergic contact dermatitis	FRMD4A	Expression profiling by array, count, 3 development stage, 2 disease state, 11 individual sets	GPL570	GSE6281	Gene Expression Time Course in the Human Skin during Elicitation of Allergic Contact Dermatitis	J Invest Dermatol.
allergic contact dermatitis	GRTP1	Expression profiling by array, count, 3 development stage, 2 disease state, 11 individual sets	GPL570	GSE6281	Gene Expression Time Course in the Human Skin during Elicitation of Allergic Contact Dermatitis	J Invest Dermatol.
allergic contact dermatitis	TANC	Expression profiling by array, count, 3 development stage, 2 disease state, 11 individual sets	GPL570	GSE6281	Gene Expression Time Course in the Human Skin during Elicitation of Allergic Contact Dermatitis	J Invest Dermatol.
allergic contact dermatitis	KIAA1704	Expression profiling by array, count, 3 development stage, 2 disease state, 11 individual sets	GPL570	GSE6281	Gene Expression Time Course in the Human Skin during Elicitation of Allergic Contact Dermatitis	J Invest Dermatol.
Allergic Contact	GZMB	Expression profiling by array	GPL570	GSE6281	Gene Expression Time Course in the Human Skin during Elicitation of Allergic Contact Dermatitis	J invest Dermatol.
Allergic Contact	SELE	Expression profiling by array	GPL570	GSE6281	Gene Expression Time Course in the Human Skin during Elicitation of Allergic Contact Dermatitis	J invest Dermatol.
Allergic Contact	RGS1	Expression profiling by array	GPL570	GSE6281	Gene Expression Time Course in the Human Skin during Elicitation of Allergic Contact Dermatitis	J invest Dermatol.
Allergic Contact	LTB	Expression profiling by array	GPL570	GSE6281	Gene Expression Time Course in the Human Skin during Elicitation of Allergic Contact Dermatitis	J invest Dermatol.
Allergic Contact	CCR7	Expression profiling by array	GPL570	GSE6281	Gene Expression Time Course in the Human Skin during Elicitation of Allergic Contact Dermatitis	J invest Dermatol.
Allergic Contact	CCL19	Expression profiling by array	GPL570	GSE6281	Gene Expression Time Course in the Human Skin during Elicitation of Allergic Contact Dermatitis	J invest Dermatol.
Allergic Contact	TRBV19	Expression profiling by array	GPL570	GSE6281	Gene Expression Time Course in the Human Skin during Elicitation of Allergic Contact Dermatitis	J invest Dermatol.
Allergic Contact	TRBV19	Expression profiling by array	GPL570	GSE6281	Gene Expression Time Course in the Human Skin during Elicitation of Allergic Contact Dermatitis	J invest Dermatol.
Allergic Contact	CD3D	Expression profiling by array	GPL570	GSE6281	Gene Expression Time Course in the Human Skin during Elicitation of Allergic Contact Dermatitis	J invest Dermatol.
Allergic Contact	GBP1	Expression profiling by array	GPL570	GSE6281	Gene Expression Time Course in the Human Skin during Elicitation of Allergic Contact Dermatitis	J invest Dermatol.
Allergic Contact	IL2RG	Expression profiling by array	GPL570	GSE6281	Gene Expression Time Course in the Human Skin during Elicitation of Allergic Contact Dermatitis	J invest Dermatol.
Allergic Contact	GBP1	Expression profiling by array	GPL570	GSE6281	Gene Expression Time Course in the Human Skin during Elicitation of Allergic Contact Dermatitis	J invest Dermatol.
Allergic Contact	IL32	Expression profiling by array	GPL570	GSE6281	Gene Expression Time Course in the Human Skin during Elicitation of Allergic Contact Dermatitis	J invest Dermatol.
Allergic Contact	IFI30	Expression profiling by array	GPL570	GSE6281	Gene Expression Time Course in the Human Skin during Elicitation of Allergic Contact Dermatitis	J invest Dermatol.
Allergic Contact	PSMB10	Expression profiling by array	GPL570	GSE6281	Gene Expression Time Course in the Human Skin during Elicitation of Allergic Contact Dermatitis	J invest Dermatol.
Allergic Contact	LILRB2	Expression profiling by array	GPL570	GSE6281	Gene Expression Time Course in the Human Skin during Elicitation of Allergic Contact Dermatitis	J invest Dermatol.
Allergic Contact	PSMB8	Expression profiling by array	GPL570	GSE6281	Gene Expression Time Course in the Human Skin during Elicitation of Allergic Contact Dermatitis	J invest Dermatol.
Allergic Contact	PSME1	Expression profiling by array	GPL570	GSE6281	Gene Expression Time Course in the Human Skin during Elicitation of Allergic Contact Dermatitis	J invest Dermatol.
Allergic Contact	LILRA2	Expression profiling by array	GPL570	GSE6281	Gene Expression Time Course in the Human Skin during Elicitation of Allergic Contact Dermatitis	J invest Dermatol.
Allergic Contact	SELL	Expression profiling by array	GPL570	GSE6281	Gene Expression Time Course in the Human Skin during Elicitation of Allergic Contact Dermatitis	J invest Dermatol.
Allergic Contact	PARP9	Expression profiling by array	GPL570	GSE6281	Gene Expression Time Course in the Human Skin during Elicitation of Allergic Contact Dermatitis	J invest Dermatol.
Allergic Contact	CORO1A	Expression profiling by array	GPL570	GSE6281	Gene Expression Time Course in the Human Skin during Elicitation of Allergic Contact Dermatitis	J invest Dermatol.
Allergic Contact	ARPC1B	Expression profiling by array	GPL570	GSE6281	Gene Expression Time Course in the Human Skin during Elicitation of Allergic Contact Dermatitis	J invest Dermatol.
Allergic Contact	RAB31	Expression profiling by array	GPL570	GSE6281	Gene Expression Time Course in the Human Skin during Elicitation of Allergic Contact Dermatitis	J invest Dermatol.
Allergic Contact	CXCR4	Expression profiling by array	GPL570	GSE6281	Gene Expression Time Course in the Human Skin during Elicitation of Allergic Contact Dermatitis	J invest Dermatol.
Allergic Contact	JAK3	Expression profiling by array	GPL570	GSE6281	Gene Expression Time Course in the Human Skin during Elicitation of Allergic Contact Dermatitis	J invest Dermatol.
Allergic Contact	IL27RA	Expression profiling by array	GPL570	GSE6281	Gene Expression Time Course in the Human Skin during Elicitation of Allergic Contact Dermatitis	J invest Dermatol.
Allergic Contact	CD3Z	Expression profiling by array	GPL570	GSE6281	Gene Expression Time Course in the Human Skin during Elicitation of Allergic Contact Dermatitis	J invest Dermatol.
Allergic Contact	CXCR4	Expression profiling by array	GPL570	GSE6281	Gene Expression Time Course in the Human Skin during Elicitation of Allergic Contact Dermatitis	J invest Dermatol.
Allergic Contact	STAT1	Expression profiling by array	GPL570	GSE6281	Gene Expression Time Course in the Human Skin during Elicitation of Allergic Contact Dermatitis	J invest Dermatol.
Allergic Contact	ECOP	Expression profiling by array	GPL570	GSE6281	Gene Expression Time Course in the Human Skin during Elicitation of Allergic Contact Dermatitis	J invest Dermatol.
Allergic Contact	IFNAR2	Expression profiling by array	GPL570	GSE6281	Gene Expression Time Course in the Human Skin during Elicitation of Allergic Contact Dermatitis	J invest Dermatol.
Allergic Contact	P2RY10	Expression profiling by array	GPL570	GSE6281	Gene Expression Time Course in the Human Skin during Elicitation of Allergic Contact Dermatitis	J invest Dermatol.
Allergic Contact	PGRMC23	Expression profiling by array	GPL570	GSE6281	Gene Expression Time Course in the Human Skin during Elicitation of Allergic Contact Dermatitis	J invest Dermatol.
Allergic Contact	TSC1	Expression profiling by array	GPL570	GSE6281	Gene Expression Time Course in the Human Skin during Elicitation of Allergic Contact Dermatitis	J invest Dermatol.
Allergic Contact	COTL1	Expression profiling by array	GPL570	GSE6281	Gene Expression Time Course in the Human Skin during Elicitation of Allergic Contact Dermatitis	J invest Dermatol.
Allergic Contact	TMSB10	Expression profiling by array	GPL570	GSE6281	Gene Expression Time Course in the Human Skin during Elicitation of Allergic Contact Dermatitis	J invest Dermatol.
Allergic Contact	TPM3	Expression profiling by array	GPL570	GSE6281	Gene Expression Time Course in the Human Skin during Elicitation of Allergic Contact Dermatitis	J invest Dermatol.
Allergic Contact	FGD4	Expression profiling by array	GPL570	GSE6281	Gene Expression Time Course in the Human Skin during Elicitation of Allergic Contact Dermatitis	J invest Dermatol.
Allergic Contact	IRF1	Expression profiling by array	GPL570	GSE6281	Gene Expression Time Course in the Human Skin during Elicitation of Allergic Contact Dermatitis	J invest Dermatol.
Allergic Contact	WARS	Expression profiling by array	GPL570	GSE6281	Gene Expression Time Course in the Human Skin during Elicitation of Allergic Contact Dermatitis	J invest Dermatol.
Allergic Contact	ODC1	Expression profiling by array	GPL570	GSE6281	Gene Expression Time Course in the Human Skin during Elicitation of Allergic Contact Dermatitis	J invest Dermatol.
Allergic Contact	LAP3	Expression profiling by array	GPL570	GSE6281	Gene Expression Time Course in the Human Skin during Elicitation of Allergic Contact Dermatitis	J invest Dermatol.
Allergic Contact	UBE2L6	Expression profiling by array	GPL570	GSE6281	Gene Expression Time Course in the Human Skin during Elicitation of Allergic Contact Dermatitis	J invest Dermatol.
Allergic Contact	PIM2	Expression profiling by array	GPL570	GSE6281	Gene Expression Time Course in the Human Skin during Elicitation of Allergic Contact Dermatitis	J invest Dermatol.
Allergic Contact	ADSL	Expression profiling by array	GPL570	GSE6281	Gene Expression Time Course in the Human Skin during Elicitation of Allergic Contact Dermatitis	J invest Dermatol.
Allergic Contact	PRPS1	Expression profiling by array	GPL570	GSE6281	Gene Expression Time Course in the Human Skin during Elicitation of Allergic Contact Dermatitis	J invest Dermatol.
Allergic Contact	ALOX5	Expression profiling by array	GPL570	GSE6281	Gene Expression Time Course in the Human Skin during Elicitation of Allergic Contact Dermatitis	J invest Dermatol.
Allergic Contact	IFT122	Expression profiling by array	GPL570	GSE6281	Gene Expression Time Course in the Human Skin during Elicitation of Allergic Contact Dermatitis	J invest Dermatol.
Allergic Contact	CAND2	Expression profiling by array	GPL570	GSE6281	Gene Expression Time Course in the Human Skin during Elicitation of Allergic Contact Dermatitis	J invest Dermatol.
Allergic Contact	GATAD1	Expression profiling by array	GPL570	GSE6281	Gene Expression Time Course in the Human Skin during Elicitation of Allergic Contact Dermatitis	J invest Dermatol.
Allergic Contact	USP31	Expression profiling by array	GPL570	GSE6281	Gene Expression Time Course in the Human Skin during Elicitation of Allergic Contact Dermatitis	J invest Dermatol.
Allergic Contact	ARHGAP9	Expression profiling by array	GPL570	GSE6281	Gene Expression Time Course in the Human Skin during Elicitation of Allergic Contact Dermatitis	J invest Dermatol.
Allergic Contact	S100A7	Expression profiling by array	GPL570	GSE6281	Gene Expression Time Course in the Human Skin during Elicitation of Allergic Contact Dermatitis	J invest Dermatol.
Allergic Contact	FKBP1A	Expression profiling by array	GPL570	GSE6281	Gene Expression Time Course in the Human Skin during Elicitation of Allergic Contact Dermatitis	J invest Dermatol.
Allergic Contact	CLIC1	Expression profiling by array	GPL570	GSE6281	Gene Expression Time Course in the Human Skin during Elicitation of Allergic Contact Dermatitis	J invest Dermatol.
Allergic Contact	HMGN4	Expression profiling by array	GPL570	GSE6281	Gene Expression Time Course in the Human Skin during Elicitation of Allergic Contact Dermatitis	J invest Dermatol.
Allergic Contact	SCUBE2	Expression profiling by array	GPL570	GSE6281	Gene Expression Time Course in the Human Skin during Elicitation of Allergic Contact Dermatitis	J invest Dermatol.

Allergic Contact	HIST2H2BE	Expression profiling by array	GPL570	GSE6281	Gene Expression Time Course in the Human Skin during Elicitation of Allergic Contact Dermatitis	J invest Dermatol.
Allergic Contact	RICS	Expression profiling by array	GPL570	GSE6281	Gene Expression Time Course in the Human Skin during Elicitation of Allergic Contact Dermatitis	J invest Dermatol.
Allergic Contact	NRXN1	Expression profiling by array	GPL570	GSE6281	Gene Expression Time Course in the Human Skin during Elicitation of Allergic Contact Dermatitis	J invest Dermatol.
Allergic Contact	PRG1	Expression profiling by array	GPL570	GSE6281	Gene Expression Time Course in the Human Skin during Elicitation of Allergic Contact Dermatitis	J invest Dermatol.
Allergic Contact	CXORF9	Expression profiling by array	GPL570	GSE6281	Gene Expression Time Course in the Human Skin during Elicitation of Allergic Contact Dermatitis	J invest Dermatol.
Allergic Contact	PRG1	Expression profiling by array	GPL570	GSE6281	Gene Expression Time Course in the Human Skin during Elicitation of Allergic Contact Dermatitis	J invest Dermatol.
Allergic Contact	KIAA0746	Expression profiling by array	GPL570	GSE6281	Gene Expression Time Course in the Human Skin during Elicitation of Allergic Contact Dermatitis	J invest Dermatol.
Allergic Contact	FLJ20647	Expression profiling by array	GPL570	GSE6281	Gene Expression Time Course in the Human Skin during Elicitation of Allergic Contact Dermatitis	J invest Dermatol.
Allergic Contact	C6ORF129	Expression profiling by array	GPL570	GSE6281	Gene Expression Time Course in the Human Skin during Elicitation of Allergic Contact Dermatitis	J invest Dermatol.
Allergic Contact	KIAA1189	Expression profiling by array	GPL570	GSE6281	Gene Expression Time Course in the Human Skin during Elicitation of Allergic Contact Dermatitis	J invest Dermatol.
Allergic Contact	FRMD4A	Expression profiling by array	GPL570	GSE6281	Gene Expression Time Course in the Human Skin during Elicitation of Allergic Contact Dermatitis	J invest Dermatol.
Allergic Contact	FRMD4A	Expression profiling by array	GPL570	GSE6281	Gene Expression Time Course in the Human Skin during Elicitation of Allergic Contact Dermatitis	J invest Dermatol.
Allergic Contact	GRTP1	Expression profiling by array	GPL570	GSE6281	Gene Expression Time Course in the Human Skin during Elicitation of Allergic Contact Dermatitis	J invest Dermatol.
Allergic Contact	TANC	Expression profiling by array	GPL570	GSE6281	Gene Expression Time Course in the Human Skin during Elicitation of Allergic Contact Dermatitis	J invest Dermatol.
Allergic Contact	KIAA1704	Expression profiling by array	GPL570	GSE6281	Gene Expression Time Course in the Human Skin during Elicitation of Allergic Contact Dermatitis	J invest Dermatol.
Allergic Contact	KIAA1002	Expression profiling by array	GPL570	GSE6281	Gene Expression Time Course in the Human Skin during Elicitation of Allergic Contact Dermatitis	J invest Dermatol.
Atopic dermatitis	ACTN1	Expression profiling by array, transformed count, 3 disease state sets	GPL2700	GSE26952 Samples	Tight Junction Defects in Atopic Dermatitis	J Allergy Clin Immunol.
Atopic dermatitis	ACTN4	Expression profiling by array, transformed count, 3 disease state sets	GPL2700	GSE26952 Samples	Tight Junction Defects in Atopic Dermatitis	J Allergy Clin Immunol.
Atopic dermatitis	CGN	Expression profiling by array, transformed count, 3 disease state sets	GPL2700	GSE26952 Samples	Tight Junction Defects in Atopic Dermatitis	J Allergy Clin Immunol.
Atopic dermatitis	CIP98	Expression profiling by array, transformed count, 3 disease state sets	GPL2700	GSE26952 Samples	Tight Junction Defects in Atopic Dermatitis	J Allergy Clin Immunol.
Atopic dermatitis	CLDN1	Expression profiling by array, transformed count, 3 disease state sets	GPL2700	GSE26952 Samples	Tight Junction Defects in Atopic Dermatitis	J Allergy Clin Immunol.
Atopic dermatitis	CLDN10	Expression profiling by array, transformed count, 3 disease state sets	GPL2700	GSE26952 Samples	Tight Junction Defects in Atopic Dermatitis	J Allergy Clin Immunol.
Atopic dermatitis	CLDN12	Expression profiling by array, transformed count, 3 disease state sets	GPL2700	GSE26952 Samples	Tight Junction Defects in Atopic Dermatitis	J Allergy Clin Immunol.
Atopic dermatitis	CLDN14	Expression profiling by array, transformed count, 3 disease state sets	GPL2700	GSE26952 Samples	Tight Junction Defects in Atopic Dermatitis	J Allergy Clin Immunol.
Atopic dermatitis	CLDN15	Expression profiling by array, transformed count, 3 disease state sets	GPL2700	GSE26952 Samples	Tight Junction Defects in Atopic Dermatitis	J Allergy Clin Immunol.
Atopic dermatitis	CLDN16	Expression profiling by array, transformed count, 3 disease state sets	GPL2700	GSE26952 Samples	Tight Junction Defects in Atopic Dermatitis	J Allergy Clin Immunol.
Atopic dermatitis	CLDN18	Expression profiling by array, transformed count, 3 disease state sets	GPL2700	GSE26952 Samples	Tight Junction Defects in Atopic Dermatitis	J Allergy Clin Immunol.
Atopic dermatitis	CLDN23	Expression profiling by array, transformed count, 3 disease state sets	GPL2700	GSE26952 Samples	Tight Junction Defects in Atopic Dermatitis	J Allergy Clin Immunol.
Atopic dermatitis	CLDN4	Expression profiling by array, transformed count, 3 disease state sets	GPL2700	GSE26952 Samples	Tight Junction Defects in Atopic Dermatitis	J Allergy Clin Immunol.
Atopic dermatitis	CLDN6	Expression profiling by array, transformed count, 3 disease state sets	GPL2700	GSE26952 Samples	Tight Junction Defects in Atopic Dermatitis	J Allergy Clin Immunol.
Atopic dermatitis	CLDN7	Expression profiling by array, transformed count, 3 disease state sets	GPL2700	GSE26952 Samples	Tight Junction Defects in Atopic Dermatitis	J Allergy Clin Immunol.
Atopic dermatitis	CLDN8	Expression profiling by array, transformed count, 3 disease state sets	GPL2700	GSE26952 Samples	Tight Junction Defects in Atopic Dermatitis	J Allergy Clin Immunol.
Atopic dermatitis	CRB3	Expression profiling by array, transformed count, 3 disease state sets	GPL2700	GSE26952 Samples	Tight Junction Defects in Atopic Dermatitis	J Allergy Clin Immunol.
Atopic dermatitis	CSDA	Expression profiling by array, transformed count, 3 disease state sets	GPL2700	GSE26952 Samples	Tight Junction Defects in Atopic Dermatitis	J Allergy Clin Immunol.
Atopic dermatitis	CTNNA1	Expression profiling by array, transformed count, 3 disease state sets	GPL2700	GSE26952 Samples	Tight Junction Defects in Atopic Dermatitis	J Allergy Clin Immunol.
Atopic dermatitis	CTNNB1	Expression profiling by array, transformed count, 3 disease state sets	GPL2700	GSE26952 Samples	Tight Junction Defects in Atopic Dermatitis	J Allergy Clin Immunol.
Atopic dermatitis	INADL	Expression profiling by array, transformed count, 3 disease state sets	GPL2700	GSE26952 Samples	Tight Junction Defects in Atopic Dermatitis	J Allergy Clin Immunol.
Atopic dermatitis	LLGL2	Expression profiling by array, transformed count, 3 disease state sets	GPL2700	GSE26952 Samples	Tight Junction Defects in Atopic Dermatitis	J Allergy Clin Immunol.
Atopic dermatitis	MAGI1	Expression profiling by array, transformed count, 3 disease state sets	GPL2700	GSE26952 Samples	Tight Junction Defects in Atopic Dermatitis	J Allergy Clin Immunol.
Atopic dermatitis	MLLT4	Expression profiling by array, transformed count, 3 disease state sets	GPL2700	GSE26952 Samples	Tight Junction Defects in Atopic Dermatitis	J Allergy Clin Immunol.
Atopic dermatitis	MPDZ	Expression profiling by array, transformed count, 3 disease state sets	GPL2700	GSE26952 Samples	Tight Junction Defects in Atopic Dermatitis	J Allergy Clin Immunol.
Atopic dermatitis	MPP5	Expression profiling by array, transformed count, 3 disease state sets	GPL2700	GSE26952 Samples	Tight Junction Defects in Atopic Dermatitis	J Allergy Clin Immunol.
Atopic dermatitis	NR3C1	Expression profiling by array, transformed count, 3 disease state sets	GPL2700	GSE26952 Samples	Tight Junction Defects in Atopic Dermatitis	J Allergy Clin Immunol.
Atopic dermatitis	OCLN	Expression profiling by array, transformed count, 3 disease state sets	GPL2700	GSE26952 Samples	Tight Junction Defects in Atopic Dermatitis	J Allergy Clin Immunol.
Atopic dermatitis	PARD3	Expression profiling by array, transformed count, 3 disease state sets	GPL2700	GSE26952 Samples	Tight Junction Defects in Atopic Dermatitis	J Allergy Clin Immunol.
Atopic dermatitis	PPP2CA	Expression profiling by array, transformed count, 3 disease state sets	GPL2700	GSE26952 Samples	Tight Junction Defects in Atopic Dermatitis	J Allergy Clin Immunol.
Atopic dermatitis	PRKCA	Expression profiling by array, transformed count, 3 disease state sets	GPL2700	GSE26952 Samples	Tight Junction Defects in Atopic Dermatitis	J Allergy Clin Immunol.
Atopic dermatitis	PRKCI	Expression profiling by array, transformed count, 3 disease state sets	GPL2700	GSE26952 Samples	Tight Junction Defects in Atopic Dermatitis	J Allergy Clin Immunol.
Atopic dermatitis	RAB13	Expression profiling by array, transformed count, 3 disease state sets	GPL2700	GSE26952 Samples	Tight Junction Defects in Atopic Dermatitis	J Allergy Clin Immunol.
Atopic dermatitis	SEC6L1	Expression profiling by array, transformed count, 3 disease state sets	GPL2700	GSE26952 Samples	Tight Junction Defects in Atopic Dermatitis	J Allergy Clin Immunol.
Atopic dermatitis	SEC8L1	Expression profiling by array, transformed count, 3 disease state sets	GPL2700	GSE26952 Samples	Tight Junction Defects in Atopic Dermatitis	J Allergy Clin Immunol.
Atopic dermatitis	SPTAN1	Expression profiling by array, transformed count, 3 disease state sets	GPL2700	GSE26952 Samples	Tight Junction Defects in Atopic Dermatitis	J Allergy Clin Immunol.
Atopic dermatitis	SRC	Expression profiling by array, transformed count, 3 disease state sets	GPL2700	GSE26952 Samples	Tight Junction Defects in Atopic Dermatitis	J Allergy Clin Immunol.
Atopic dermatitis	TJP1	Expression profiling by array, transformed count, 3 disease state sets	GPL2700	GSE26952 Samples	Tight Junction Defects in Atopic Dermatitis	J Allergy Clin Immunol.
Atopic dermatitis	TJP2	Expression profiling by array, transformed count, 3 disease state sets	GPL2700	GSE26952 Samples	Tight Junction Defects in Atopic Dermatitis	J Allergy Clin Immunol.
Atopic dermatitis	TJP3	Expression profiling by array, transformed count, 3 disease state sets	GPL2700	GSE26952 Samples	Tight Junction Defects in Atopic Dermatitis	J Allergy Clin Immunol.
Atopic dermatitis	TJP4	Expression profiling by array, transformed count, 3 disease state sets	GPL2700	GSE26952 Samples	Tight Junction Defects in Atopic Dermatitis	J Allergy Clin Immunol.
Atopic dermatitis	YES1	Expression profiling by array, transformed count, 3 disease state sets	GPL2700	GSE26952 Samples	Tight Junction Defects in Atopic Dermatitis	J Allergy Clin Immunol.
Atopic dermatitis	ZAK	Expression profiling by array, transformed count, 3 disease state sets	GPL2700	GSE26952 Samples	Tight Junction Defects in Atopic Dermatitis	J Allergy Clin Immunol.
atopic dermatitis	ARPC5	Expression profiling by array, count, 3 development stage, 2 disease state, 11 individual sets	GPL570	GSE32924 Samples	Nonlesional atopic dermatitis skin is characterized by broad terminal differentiation defects and variable immune abnormalities.	J Allergy Clin Immunol.
atopic dermatitis	CCT2	Expression profiling by array, count, 3 development stage, 2 disease state, 11 individual sets	GPL570	GSE32924 Samples	Nonlesional atopic dermatitis skin is characterized by broad terminal differentiation defects and variable immune abnormalities.	J Allergy Clin Immunol.

atopic dermatitis	VAMP3	Expression profiling by array, count, 3 development stage, 2 disease state, 11 individual sets	GPL570	GSE32924 Samples	Nonlesional atopic dermatitis skin is characterized by broad terminal differentiation defects and variable immune abnormalities.	J Allergy Clin Immunol.
atopic dermatitis	DAZAP2	Expression profiling by array, count, 3 development stage, 2 disease state, 11 individual sets	GPL570	GSE32924 Samples	Nonlesional atopic dermatitis skin is characterized by broad terminal differentiation defects and variable immune abnormalities.	J Allergy Clin Immunol.
atopic dermatitis	NAT13	Expression profiling by array, count, 3 development stage, 2 disease state, 11 individual sets	GPL570	GSE32924 Samples	Nonlesional atopic dermatitis skin is characterized by broad terminal differentiation defects and variable immune abnormalities.	J Allergy Clin Immunol.
atopic dermatitis	SCOC	Expression profiling by array, count, 3 development stage, 2 disease state, 11 individual sets	GPL570	GSE32924 Samples	Nonlesional atopic dermatitis skin is characterized by broad terminal differentiation defects and variable immune abnormalities.	J Allergy Clin Immunol.
atopic dermatitis	GDE1	Expression profiling by array, count, 3 development stage, 2 disease state, 11 individual sets	GPL570	GSE32924 Samples	Nonlesional atopic dermatitis skin is characterized by broad terminal differentiation defects and variable immune abnormalities.	J Allergy Clin Immunol.
atopic dermatitis	NA	Expression profiling by array, count, 3 development stage, 2 disease state, 11 individual sets	GPL570	GSE32924 Samples	Nonlesional atopic dermatitis skin is characterized by broad terminal differentiation defects and variable immune abnormalities.	J Allergy Clin Immunol.
atopic dermatitis	ASAH1	Expression profiling by array, count, 3 development stage, 2 disease state, 11 individual sets	GPL570	GSE32924 Samples	Nonlesional atopic dermatitis skin is characterized by broad terminal differentiation defects and variable immune abnormalities.	J Allergy Clin Immunol.
atopic dermatitis	CAT	Expression profiling by array, count, 3 development stage, 2 disease state, 11 individual sets	GPL570	GSE32924 Samples	Nonlesional atopic dermatitis skin is characterized by broad terminal differentiation defects and variable immune abnormalities.	J Allergy Clin Immunol.
atopic dermatitis	MAX	Expression profiling by array, count, 3 development stage, 2 disease state, 11 individual sets	GPL570	GSE32924 Samples	Nonlesional atopic dermatitis skin is characterized by broad terminal differentiation defects and variable immune abnormalities.	J Allergy Clin Immunol.
atopic dermatitis	HLF	Expression profiling by array, count, 3 development stage, 2 disease state, 11 individual sets	GPL570	GSE32924 Samples	Nonlesional atopic dermatitis skin is characterized by broad terminal differentiation defects and variable immune abnormalities.	J Allergy Clin Immunol.
atopic dermatitis	GGTA1	Expression profiling by array, count, 3 development stage, 2 disease state, 11 individual sets	GPL570	GSE32924 Samples	Nonlesional atopic dermatitis skin is characterized by broad terminal differentiation defects and variable immune abnormalities.	J Allergy Clin Immunol.
atopic dermatitis	RCN2	Expression profiling by array, count, 3 development stage, 2 disease state, 11 individual sets	GPL570	GSE32924 Samples	Nonlesional atopic dermatitis skin is characterized by broad terminal differentiation defects and variable immune abnormalities.	J Allergy Clin Immunol.
atopic dermatitis	RMND5A	Expression profiling by array, count, 3 development stage, 2 disease state, 11 individual sets	GPL570	GSE32924 Samples	Nonlesional atopic dermatitis skin is characterized by broad terminal differentiation defects and variable immune abnormalities.	J Allergy Clin Immunol.
atopic dermatitis	AIDA	Expression profiling by array, count, 3 development stage, 2 disease state, 11 individual sets	GPL570	GSE32924 Samples	Nonlesional atopic dermatitis skin is characterized by broad terminal differentiation defects and variable immune abnormalities.	J Allergy Clin Immunol.
atopic dermatitis	TRAM1	Expression profiling by array, count, 3 development stage, 2 disease state, 11 individual sets	GPL570	GSE32924 Samples	Nonlesional atopic dermatitis skin is characterized by broad terminal differentiation defects and variable immune abnormalities.	J Allergy Clin Immunol.
atopic dermatitis	MALAT1	Expression profiling by array, count, 3 development stage, 2 disease state, 11 individual sets	GPL570	GSE32924 Samples	Nonlesional atopic dermatitis skin is characterized by broad terminal differentiation defects and variable immune abnormalities.	J Allergy Clin Immunol.
atopic dermatitis	UBE2B	Expression profiling by array, count, 3 development stage, 2 disease state, 11 individual sets	GPL570	GSE32924 Samples	Nonlesional atopic dermatitis skin is characterized by broad terminal differentiation defects and variable immune abnormalities.	J Allergy Clin Immunol.
atopic dermatitis	GALNT1	Expression profiling by array, count, 3 development stage, 2 disease state, 11 individual sets	GPL570	GSE32924 Samples	Nonlesional atopic dermatitis skin is characterized by broad terminal differentiation defects and variable immune abnormalities.	J Allergy Clin Immunol.
atopic dermatitis	PARD3	Expression profiling by array, count, 3 development stage, 2 disease state, 11 individual sets	GPL570	GSE32924 Samples	Nonlesional atopic dermatitis skin is characterized by broad terminal differentiation defects and variable immune abnormalities.	J Allergy Clin Immunol.
atopic dermatitis	GALNT1	Expression profiling by array, count, 3 development stage, 2 disease state, 11 individual sets	GPL570	GSE32924 Samples	Nonlesional atopic dermatitis skin is characterized by broad terminal differentiation defects and variable immune abnormalities.	J Allergy Clin Immunol.
atopic dermatitis	HADHA	Expression profiling by array, count, 3 development stage, 2 disease state, 11 individual sets	GPL570	GSE32924 Samples	Nonlesional atopic dermatitis skin is characterized by broad terminal differentiation defects and variable immune abnormalities.	J Allergy Clin Immunol.
atopic dermatitis	TCF4	Expression profiling by array, count, 3 development stage, 2 disease state, 11 individual sets	GPL570	GSE32924 Samples	Nonlesional atopic dermatitis skin is characterized by broad terminal differentiation defects and variable immune abnormalities.	J Allergy Clin Immunol.
atopic dermatitis	LAMB4	Expression profiling by array, count, 3 development stage, 2 disease state, 11 individual sets	GPL570	GSE32924 Samples	Nonlesional atopic dermatitis skin is characterized by broad terminal differentiation defects and variable immune abnormalities.	J Allergy Clin Immunol.
atopic dermatitis	RAPGEF4	Expression profiling by array, count, 3 development stage, 2 disease state, 11 individual sets	GPL570	GSE32924 Samples	Nonlesional atopic dermatitis skin is characterized by broad terminal differentiation defects and variable immune abnormalities.	J Allergy Clin Immunol.
atopic dermatitis	ALDH3A2	Expression profiling by array, count, 3 development stage, 2 disease state, 11 individual sets	GPL570	GSE32924 Samples	Nonlesional atopic dermatitis skin is characterized by broad terminal differentiation defects and variable immune abnormalities.	J Allergy Clin Immunol.
atopic dermatitis	NUCKS1	Expression profiling by array, count, 3 development stage, 2 disease state, 11 individual sets	GPL570	GSE32924 Samples	Nonlesional atopic dermatitis skin is characterized by broad terminal differentiation defects and variable immune abnormalities.	J Allergy Clin Immunol.
atopic dermatitis	COLEC12	Expression profiling by array, count, 3 development stage, 2 disease state, 11 individual sets	GPL570	GSE32924 Samples	Nonlesional atopic dermatitis skin is characterized by broad terminal differentiation defects and variable immune abnormalities.	J Allergy Clin Immunol.
atopic dermatitis	OLFML1	Expression profiling by array, count, 3 development stage, 2 disease state, 11 individual sets	GPL570	GSE32924 Samples	Nonlesional atopic dermatitis skin is characterized by broad terminal differentiation defects and variable immune abnormalities.	J Allergy Clin Immunol.
atopic dermatitis	RNASE4	Expression profiling by array, count, 3 development stage, 2 disease state, 11 individual sets	GPL570	GSE32924 Samples	Nonlesional atopic dermatitis skin is characterized by broad terminal differentiation defects and variable immune abnormalities.	J Allergy Clin Immunol.
atopic dermatitis	FBLN5	Expression profiling by array, count, 3 development stage, 2 disease state, 11 individual sets	GPL570	GSE32924 Samples	Nonlesional atopic dermatitis skin is characterized by broad terminal differentiation defects and variable immune abnormalities.	J Allergy Clin Immunol.
atopic dermatitis	CRTAP	Expression profiling by array, count, 3 development stage, 2 disease state, 11 individual sets	GPL570	GSE32924 Samples	Nonlesional atopic dermatitis skin is characterized by broad terminal differentiation defects and variable immune abnormalities.	J Allergy Clin Immunol.
atopic dermatitis	PSMD10	Expression profiling by array, count, 3 development stage, 2 disease state, 11 individual sets	GPL570	GSE32924 Samples	Nonlesional atopic dermatitis skin is characterized by broad terminal differentiation defects and variable immune abnormalities.	J Allergy Clin Immunol.
atopic dermatitis	SMARCC1	Expression profiling by array, count, 3 development stage, 2 disease state, 11 individual sets	GPL570	GSE32924 Samples	Nonlesional atopic dermatitis skin is characterized by broad terminal differentiation defects and variable immune abnormalities.	J Allergy Clin Immunol.
atopic dermatitis	NCK1	Expression profiling by array, count, 3 development stage, 2 disease state, 11 individual sets	GPL570	GSE32924 Samples	Nonlesional atopic dermatitis skin is characterized by broad terminal differentiation defects and variable immune abnormalities.	J Allergy Clin Immunol.
atopic dermatitis	TMEM50B	Expression profiling by array, count, 3 development stage, 2 disease state, 11 individual sets	GPL570	GSE32924 Samples	Nonlesional atopic dermatitis skin is characterized by broad terminal differentiation defects and variable immune abnormalities.	J Allergy Clin Immunol.
atopic dermatitis	WDR26	Expression profiling by array, count, 3 development stage, 2 disease state, 11 individual sets	GPL570	GSE32924 Samples	Nonlesional atopic dermatitis skin is characterized by broad terminal differentiation defects and variable immune abnormalities.	J Allergy Clin Immunol.
atopic dermatitis	IDS	Expression profiling by array, count, 3 development stage, 2 disease state, 11 individual sets	GPL570	GSE32924 Samples	Nonlesional atopic dermatitis skin is characterized by broad terminal differentiation defects and variable immune abnormalities.	J Allergy Clin Immunol.
atopic dermatitis	MACF1	Expression profiling by array, count, 3 development stage, 2 disease state, 11 individual sets	GPL570	GSE32924 Samples	Nonlesional atopic dermatitis skin is characterized by broad terminal differentiation defects and variable immune abnormalities.	J Allergy Clin Immunol.
atopic dermatitis	ARL2BP	Expression profiling by array, count, 3 development stage, 2 disease state, 11 individual sets	GPL570	GSE32924 Samples	Nonlesional atopic dermatitis skin is characterized by broad terminal differentiation defects and variable immune abnormalities.	J Allergy Clin Immunol.
atopic dermatitis	CORO1C	Expression profiling by array, count, 3 development stage, 2 disease state, 11 individual sets	GPL570	GSE32924 Samples	Nonlesional atopic dermatitis skin is characterized by broad terminal differentiation defects and variable immune abnormalities.	J Allergy Clin Immunol.
atopic dermatitis	MAPRE2	Expression profiling by array, count, 3 development stage, 2 disease state, 11 individual sets	GPL570	GSE32924 Samples	Nonlesional atopic dermatitis skin is characterized by broad terminal differentiation defects and variable immune abnormalities.	J Allergy Clin Immunol.

atopic dermatitis	ZFYVE21	Expression profiling by array, count, 3 development stage, 2 disease state, 11 individual sets	GPL570	GSE32924 Samples	Nonlesional atopic dermatitis skin is characterized by broad terminal differentiation defects and variable immune abnormalities.	J Allergy Clin Immunol.
atopic dermatitis	38969	Expression profiling by array, count, 3 development stage, 2 disease state, 11 individual sets	GPL570	GSE32924 Samples	Nonlesional atopic dermatitis skin is characterized by broad terminal differentiation defects and variable immune abnormalities.	J Allergy Clin Immunol.
atopic dermatitis	PTPN11	Expression profiling by array, count, 3 development stage, 2 disease state, 11 individual sets	GPL570	GSE32924 Samples	Nonlesional atopic dermatitis skin is characterized by broad terminal differentiation defects and variable immune abnormalities.	J Allergy Clin Immunol.
atopic dermatitis	PEA15	Expression profiling by array, count, 3 development stage, 2 disease state, 11 individual sets	GPL570	GSE32924 Samples	Nonlesional atopic dermatitis skin is characterized by broad terminal differentiation defects and variable immune abnormalities.	J Allergy Clin Immunol.
atopic dermatitis	C4ORF41	Expression profiling by array, count, 3 development stage, 2 disease state, 11 individual sets	GPL570	GSE32924 Samples	Nonlesional atopic dermatitis skin is characterized by broad terminal differentiation defects and variable immune abnormalities.	J Allergy Clin Immunol.
atopic dermatitis	MRPS10	Expression profiling by array, count, 3 development stage, 2 disease state, 11 individual sets	GPL570	GSE32924 Samples	Nonlesional atopic dermatitis skin is characterized by broad terminal differentiation defects and variable immune abnormalities.	J Allergy Clin Immunol.
atopic dermatitis	PIGY	Expression profiling by array, count, 3 development stage, 2 disease state, 11 individual sets	GPL570	GSE32924 Samples	Nonlesional atopic dermatitis skin is characterized by broad terminal differentiation defects and variable immune abnormalities.	J Allergy Clin Immunol.
atopic dermatitis	C20ORF108	Expression profiling by array, count, 3 development stage, 2 disease state, 11 individual sets	GPL570	GSE32924 Samples	Nonlesional atopic dermatitis skin is characterized by broad terminal differentiation defects and variable immune abnormalities.	J Allergy Clin Immunol.
atopic dermatitis	38961	Expression profiling by array, count, 3 development stage, 2 disease state, 11 individual sets	GPL570	GSE32924 Samples	Nonlesional atopic dermatitis skin is characterized by broad terminal differentiation defects and variable immune abnormalities.	J Allergy Clin Immunol.
atopic dermatitis	SLC11A2	Expression profiling by array, count, 3 development stage, 2 disease state, 11 individual sets	GPL570	GSE32924 Samples	Nonlesional atopic dermatitis skin is characterized by broad terminal differentiation defects and variable immune abnormalities.	J Allergy Clin Immunol.
atopic dermatitis	ERMP1	Expression profiling by array, count, 3 development stage, 2 disease state, 11 individual sets	GPL570	GSE32924 Samples	Nonlesional atopic dermatitis skin is characterized by broad terminal differentiation defects and variable immune abnormalities.	J Allergy Clin Immunol.
atopic dermatitis	SCCPDH	Expression profiling by array, count, 3 development stage, 2 disease state, 11 individual sets	GPL570	GSE32924 Samples	Nonlesional atopic dermatitis skin is characterized by broad terminal differentiation defects and variable immune abnormalities.	J Allergy Clin Immunol.
atopic dermatitis	SETD8	Expression profiling by array, count, 3 development stage, 2 disease state, 11 individual sets	GPL570	GSE32924 Samples	Nonlesional atopic dermatitis skin is characterized by broad terminal differentiation defects and variable immune abnormalities.	J Allergy Clin Immunol.
atopic dermatitis	TPM4	Expression profiling by array, count, 3 development stage, 2 disease state, 11 individual sets	GPL570	GSE32924 Samples	Nonlesional atopic dermatitis skin is characterized by broad terminal differentiation defects and variable immune abnormalities.	J Allergy Clin Immunol.
atopic dermatitis	NA	Expression profiling by array, count, 3 development stage, 2 disease state, 11 individual sets	GPL570	GSE32924 Samples	Nonlesional atopic dermatitis skin is characterized by broad terminal differentiation defects and variable immune abnormalities.	J Allergy Clin Immunol.
atopic dermatitis	CDYL	Expression profiling by array, count, 3 development stage, 2 disease state, 11 individual sets	GPL570	GSE32924 Samples	Nonlesional atopic dermatitis skin is characterized by broad terminal differentiation defects and variable immune abnormalities.	J Allergy Clin Immunol.
atopic dermatitis	C19ORF2	Expression profiling by array, count, 3 development stage, 2 disease state, 11 individual sets	GPL570	GSE32924 Samples	Nonlesional atopic dermatitis skin is characterized by broad terminal differentiation defects and variable immune abnormalities.	J Allergy Clin Immunol.
atopic dermatitis	CLIP1	Expression profiling by array, count, 3 development stage, 2 disease state, 11 individual sets	GPL570	GSE32924 Samples	Nonlesional atopic dermatitis skin is characterized by broad terminal differentiation defects and variable immune abnormalities.	J Allergy Clin Immunol.
atopic dermatitis	RANBP9	Expression profiling by array, count, 3 development stage, 2 disease state, 11 individual sets	GPL570	GSE32924 Samples	Nonlesional atopic dermatitis skin is characterized by broad terminal differentiation defects and variable immune abnormalities.	J Allergy Clin Immunol.
atopic dermatitis	NRBF2	Expression profiling by array, count, 3 development stage, 2 disease state, 11 individual sets	GPL570	GSE32924 Samples	Nonlesional atopic dermatitis skin is characterized by broad terminal differentiation defects and variable immune abnormalities.	J Allergy Clin Immunol.
atopic dermatitis	NRBF2	Expression profiling by array, count, 3 development stage, 2 disease state, 11 individual sets	GPL570	GSE32924 Samples	Nonlesional atopic dermatitis skin is characterized by broad terminal differentiation defects and variable immune abnormalities.	J Allergy Clin Immunol.
atopic dermatitis	LYPLA1	Expression profiling by array, count, 3 development stage, 2 disease state, 11 individual sets	GPL570	GSE32924 Samples	Nonlesional atopic dermatitis skin is characterized by broad terminal differentiation defects and variable immune abnormalities.	J Allergy Clin Immunol.
atopic dermatitis	MYO5B	Expression profiling by array, count, 3 development stage, 2 disease state, 11 individual sets	GPL570	GSE32924 Samples	Nonlesional atopic dermatitis skin is characterized by broad terminal differentiation defects and variable immune abnormalities.	J Allergy Clin Immunol.
atopic dermatitis	NIPAL1	Expression profiling by array, count, 3 development stage, 2 disease state, 11 individual sets	GPL570	GSE32924 Samples	Nonlesional atopic dermatitis skin is characterized by broad terminal differentiation defects and variable immune abnormalities.	J Allergy Clin Immunol.
atopic dermatitis	JAK1	Expression profiling by array, count, 3 development stage, 2 disease state, 11 individual sets	GPL570	GSE32924 Samples	Nonlesional atopic dermatitis skin is characterized by broad terminal differentiation defects and variable immune abnormalities.	J Allergy Clin Immunol.
atopic dermatitis	CITED2	Expression profiling by array, count, 3 development stage, 2 disease state, 11 individual sets	GPL570	GSE32924 Samples	Nonlesional atopic dermatitis skin is characterized by broad terminal differentiation defects and variable immune abnormalities.	J Allergy Clin Immunol.
atopic dermatitis	PSAT1	Expression profiling by array, count, 3 development stage, 2 disease state, 11 individual sets	GPL570	GSE32924 Samples	Nonlesional atopic dermatitis skin is characterized by broad terminal differentiation defects and variable immune abnormalities.	J Allergy Clin Immunol.
atopic dermatitis	CLDN1	Expression profiling by array, count, 3 development stage, 2 disease state, 11 individual sets	GPL570	GSE32924 Samples	Nonlesional atopic dermatitis skin is characterized by broad terminal differentiation defects and variable immune abnormalities.	J Allergy Clin Immunol.
atopic dermatitis	KLF4	Expression profiling by array, count, 3 development stage, 2 disease state, 11 individual sets	GPL570	GSE32924 Samples	Nonlesional atopic dermatitis skin is characterized by broad terminal differentiation defects and variable immune abnormalities.	J Allergy Clin Immunol.
atopic dermatitis	GLRX	Expression profiling by array, count, 3 development stage, 2 disease state, 11 individual sets	GPL570	GSE32924 Samples	Nonlesional atopic dermatitis skin is characterized by broad terminal differentiation defects and variable immune abnormalities.	J Allergy Clin Immunol.
atopic dermatitis	SGEF	Expression profiling by array, count, 3 development stage, 2 disease state, 11 individual sets	GPL570	GSE32924 Samples	Nonlesional atopic dermatitis skin is characterized by broad terminal differentiation defects and variable immune abnormalities.	J Allergy Clin Immunol.
atopic dermatitis	NA	Expression profiling by array, count, 3 development stage, 2 disease state, 11 individual sets	GPL570	GSE32924 Samples	Nonlesional atopic dermatitis skin is characterized by broad terminal differentiation defects and variable immune abnormalities.	J Allergy Clin Immunol.
atopic dermatitis	TSPAN5	Expression profiling by array, count, 3 development stage, 2 disease state, 11 individual sets	GPL570	GSE32924 Samples	Nonlesional atopic dermatitis skin is characterized by broad terminal differentiation defects and variable immune abnormalities.	J Allergy Clin Immunol.
atopic dermatitis	LGALS8	Expression profiling by array, count, 3 development stage, 2 disease state, 11 individual sets	GPL570	GSE32924 Samples	Nonlesional atopic dermatitis skin is characterized by broad terminal differentiation defects and variable immune abnormalities.	J Allergy Clin Immunol.
atopic dermatitis	RAB11FIP1	Expression profiling by array, count, 3 development stage, 2 disease state, 11 individual sets	GPL570	GSE32924 Samples	Nonlesional atopic dermatitis skin is characterized by broad terminal differentiation defects and variable immune abnormalities.	J Allergy Clin Immunol.
atopic dermatitis	RNF39	Expression profiling by array, count, 3 development stage, 2 disease state, 11 individual sets	GPL570	GSE32924 Samples	Nonlesional atopic dermatitis skin is characterized by broad terminal differentiation defects and variable immune abnormalities.	J Allergy Clin Immunol.
atopic dermatitis	KDM5B	Expression profiling by array, count, 3 development stage, 2 disease state, 11 individual sets	GPL570	GSE32924 Samples	Nonlesional atopic dermatitis skin is characterized by broad terminal differentiation defects and variable immune abnormalities.	J Allergy Clin Immunol.
atopic dermatitis	NA	Expression profiling by array, count, 3 development stage, 2 disease state, 11 individual sets	GPL570	GSE32924 Samples	Nonlesional atopic dermatitis skin is characterized by broad terminal differentiation defects and variable immune abnormalities.	J Allergy Clin Immunol.
atopic dermatitis	LOC100049716	Expression profiling by array, count, 3 development stage, 2 disease state, 11 individual sets	GPL570	GSE32924 Samples	Nonlesional atopic dermatitis skin is characterized by broad terminal differentiation defects and variable immune abnormalities.	J Allergy Clin Immunol.
atopic dermatitis	TMEM99	Expression profiling by array, count, 3 development stage, 2 disease state, 11 individual sets	GPL570	GSE32924 Samples	Nonlesional atopic dermatitis skin is characterized by broad terminal differentiation defects and variable immune abnormalities.	J Allergy Clin Immunol.
atopic dermatitis	CYP39A1	Expression profiling by array, count, 3 development stage, 2 disease state, 11 individual sets	GPL570	GSE32924 Samples	Nonlesional atopic dermatitis skin is characterized by broad terminal differentiation defects and variable immune abnormalities.	J Allergy Clin Immunol.

atopic dermatitis	MOSPD2	Expression profiling by array, count, 3 development stage, 2 disease state, 11 individual sets	GPL570	GSE32924 Samples	Nonlesional atopic dermatitis skin is characterized by broad terminal differentiation defects and variable immune abnormalities.	J Allergy Clin Immunol.
atopic dermatitis	FAM171B	Expression profiling by array, count, 3 development stage, 2 disease state, 11 individual sets	GPL570	GSE32924 Samples	Nonlesional atopic dermatitis skin is characterized by broad terminal differentiation defects and variable immune abnormalities.	J Allergy Clin Immunol.
atopic dermatitis	HLF	Expression profiling by array, count, 3 development stage, 2 disease state, 11 individual sets	GPL570	GSE32924 Samples	Nonlesional atopic dermatitis skin is characterized by broad terminal differentiation defects and variable immune abnormalities.	J Allergy Clin Immunol.
atopic dermatitis	SGEF	Expression profiling by array, count, 3 development stage, 2 disease state, 11 individual sets	GPL570	GSE32924 Samples	Nonlesional atopic dermatitis skin is characterized by broad terminal differentiation defects and variable immune abnormalities.	J Allergy Clin Immunol.
atopic dermatitis	NPIPL1	Expression profiling by array, count, 3 development stage, 2 disease state, 11 individual sets	GPL570	GSE32924 Samples	Nonlesional atopic dermatitis skin is characterized by broad terminal differentiation defects and variable immune abnormalities.	J Allergy Clin Immunol.
atopic dermatitis	UST	Expression profiling by array, count, 3 development stage, 2 disease state, 11 individual sets	GPL570	GSE32924 Samples	Nonlesional atopic dermatitis skin is characterized by broad terminal differentiation defects and variable immune abnormalities.	J Allergy Clin Immunol.
atopic dermatitis	ZNF720	Expression profiling by array, count, 3 development stage, 2 disease state, 11 individual sets	GPL570	GSE32924 Samples	Nonlesional atopic dermatitis skin is characterized by broad terminal differentiation defects and variable immune abnormalities.	J Allergy Clin Immunol.
atopic dermatitis	RANBP2	Expression profiling by array, count, 3 development stage, 2 disease state, 11 individual sets	GPL570	GSE32924 Samples	Nonlesional atopic dermatitis skin is characterized by broad terminal differentiation defects and variable immune abnormalities.	J Allergy Clin Immunol.
atopic dermatitis	SVIL	Expression profiling by array, count, 3 development stage, 2 disease state, 11 individual sets	GPL570	GSE32924 Samples	Nonlesional atopic dermatitis skin is characterized by broad terminal differentiation defects and variable immune abnormalities.	J Allergy Clin Immunol.
atopic dermatitis	ATP2B1	Expression profiling by array, count, 3 development stage, 2 disease state, 11 individual sets	GPL570	GSE32924 Samples	Nonlesional atopic dermatitis skin is characterized by broad terminal differentiation defects and variable immune abnormalities.	J Allergy Clin Immunol.
atopic dermatitis	NA	Expression profiling by array, count, 3 development stage, 2 disease state, 11 individual sets	GPL570	GSE32924 Samples	Nonlesional atopic dermatitis skin is characterized by broad terminal differentiation defects and variable immune abnormalities.	J Allergy Clin Immunol.
atopic dermatitis	IFI16	Expression profiling by array, count, 3 development stage, 2 disease state, 11 individual sets	GPL570	GSE32924 Samples	Nonlesional atopic dermatitis skin is characterized by broad terminal differentiation defects and variable immune abnormalities.	J Allergy Clin Immunol.
atopic dermatitis	FGFR2	Expression profiling by array, count, 3 development stage, 2 disease state, 11 individual sets	GPL570	GSE32924 Samples	Nonlesional atopic dermatitis skin is characterized by broad terminal differentiation defects and variable immune abnormalities.	J Allergy Clin Immunol.
atopic dermatitis	CNTN1	Expression profiling by array, count, 3 development stage, 2 disease state, 11 individual sets	GPL570	GSE32924 Samples	Nonlesional atopic dermatitis skin is characterized by broad terminal differentiation defects and variable immune abnormalities.	J Allergy Clin Immunol.
atopic dermatitis	38778	Expression profiling by array, count, 3 development stage, 2 disease state, 11 individual sets	GPL570	GSE32924 Samples	Nonlesional atopic dermatitis skin is characterized by broad terminal differentiation defects and variable immune abnormalities.	J Allergy Clin Immunol.
atopic dermatitis	SPTLC3	Expression profiling by array, count, 3 development stage, 2 disease state, 11 individual sets	GPL570	GSE32924 Samples	Nonlesional atopic dermatitis skin is characterized by broad terminal differentiation defects and variable immune abnormalities.	J Allergy Clin Immunol.
atopic dermatitis	RORA	Expression profiling by array, count, 3 development stage, 2 disease state, 11 individual sets	GPL570	GSE32924 Samples	Nonlesional atopic dermatitis skin is characterized by broad terminal differentiation defects and variable immune abnormalities.	J Allergy Clin Immunol.
atopic dermatitis	SPAG1	Expression profiling by array, count, 3 development stage, 2 disease state, 11 individual sets	GPL570	GSE32924 Samples	Nonlesional atopic dermatitis skin is characterized by broad terminal differentiation defects and variable immune abnormalities.	J Allergy Clin Immunol.
atopic dermatitis	ABHD5	Expression profiling by array, count, 3 development stage, 2 disease state, 11 individual sets	GPL570	GSE32924 Samples	Nonlesional atopic dermatitis skin is characterized by broad terminal differentiation defects and variable immune abnormalities.	J Allergy Clin Immunol.
atopic dermatitis	CHP2	Expression profiling by array, count, 3 development stage, 2 disease state, 11 individual sets	GPL570	GSE32924 Samples	Nonlesional atopic dermatitis skin is characterized by broad terminal differentiation defects and variable immune abnormalities.	J Allergy Clin Immunol.
atopic dermatitis	NA	Expression profiling by array, count, 3 development stage, 2 disease state, 11 individual sets	GPL570	GSE32924 Samples	Nonlesional atopic dermatitis skin is characterized by broad terminal differentiation defects and variable immune abnormalities.	J Allergy Clin Immunol.
atopic dermatitis	CLINT1	Expression profiling by array, count, 3 development stage, 2 disease state, 11 individual sets	GPL570	GSE32924 Samples	Nonlesional atopic dermatitis skin is characterized by broad terminal differentiation defects and variable immune abnormalities.	J Allergy Clin Immunol.
atopic dermatitis	ZRANB1	Expression profiling by array, count, 3 development stage, 2 disease state, 11 individual sets	GPL570	GSE32924 Samples	Nonlesional atopic dermatitis skin is characterized by broad terminal differentiation defects and variable immune abnormalities.	J Allergy Clin Immunol.
atopic dermatitis	TMCC1	Expression profiling by array, count, 3 development stage, 2 disease state, 11 individual sets	GPL570	GSE32924 Samples	Nonlesional atopic dermatitis skin is characterized by broad terminal differentiation defects and variable immune abnormalities.	J Allergy Clin Immunol.
atopic dermatitis	USP38	Expression profiling by array, count, 3 development stage, 2 disease state, 11 individual sets	GPL570	GSE32924 Samples	Nonlesional atopic dermatitis skin is characterized by broad terminal differentiation defects and variable immune abnormalities.	J Allergy Clin Immunol.
atopic dermatitis	TSN	Expression profiling by array, count, 3 development stage, 2 disease state, 11 individual sets	GPL570	GSE32924 Samples	Nonlesional atopic dermatitis skin is characterized by broad terminal differentiation defects and variable immune abnormalities.	J Allergy Clin Immunol.
atopic dermatitis	SIAH2	Expression profiling by array, count, 3 development stage, 2 disease state, 11 individual sets	GPL570	GSE32924 Samples	Nonlesional atopic dermatitis skin is characterized by broad terminal differentiation defects and variable immune abnormalities.	J Allergy Clin Immunol.
atopic dermatitis	USP46	Expression profiling by array, count, 3 development stage, 2 disease state, 11 individual sets	GPL570	GSE32924 Samples	Nonlesional atopic dermatitis skin is characterized by broad terminal differentiation defects and variable immune abnormalities.	J Allergy Clin Immunol.
atopic dermatitis	DIS3	Expression profiling by array, count, 3 development stage, 2 disease state, 11 individual sets	GPL570	GSE32924 Samples	Nonlesional atopic dermatitis skin is characterized by broad terminal differentiation defects and variable immune abnormalities.	J Allergy Clin Immunol.
atopic dermatitis	TPM4	Expression profiling by array, count, 3 development stage, 2 disease state, 11 individual sets	GPL570	GSE32924 Samples	Nonlesional atopic dermatitis skin is characterized by broad terminal differentiation defects and variable immune abnormalities.	J Allergy Clin Immunol.
atopic dermatitis	TPM4	Expression profiling by array, count, 3 development stage, 2 disease state, 11 individual sets	GPL570	GSE32924 Samples	Nonlesional atopic dermatitis skin is characterized by broad terminal differentiation defects and variable immune abnormalities.	J Allergy Clin Immunol.
atopic dermatitis	DENR	Expression profiling by array, count, 3 development stage, 2 disease state, 11 individual sets	GPL570	GSE32924 Samples	Nonlesional atopic dermatitis skin is characterized by broad terminal differentiation defects and variable immune abnormalities.	J Allergy Clin Immunol.
atopic dermatitis	MAP7	Expression profiling by array, count, 3 development stage, 2 disease state, 11 individual sets	GPL570	GSE32924 Samples	Nonlesional atopic dermatitis skin is characterized by broad terminal differentiation defects and variable immune abnormalities.	J Allergy Clin Immunol.
atopic dermatitis	XRN2	Expression profiling by array, count, 3 development stage, 2 disease state, 11 individual sets	GPL570	GSE32924 Samples	Nonlesional atopic dermatitis skin is characterized by broad terminal differentiation defects and variable immune abnormalities.	J Allergy Clin Immunol.
atopic dermatitis	FYTTD1	Expression profiling by array, count, 3 development stage, 2 disease state, 11 individual sets	GPL570	GSE32924 Samples	Nonlesional atopic dermatitis skin is characterized by broad terminal differentiation defects and variable immune abnormalities.	J Allergy Clin Immunol.
atopic dermatitis	TMEM9B	Expression profiling by array, count, 3 development stage, 2 disease state, 11 individual sets	GPL570	GSE32924 Samples	Nonlesional atopic dermatitis skin is characterized by broad terminal differentiation defects and variable immune abnormalities.	J Allergy Clin Immunol.
atopic dermatitis	KLF3	Expression profiling by array, count, 3 development stage, 2 disease state, 11 individual sets	GPL570	GSE32924 Samples	Nonlesional atopic dermatitis skin is characterized by broad terminal differentiation defects and variable immune abnormalities.	J Allergy Clin Immunol.
atopic dermatitis	AGA	Expression profiling by array, count, 3 development stage, 2 disease state, 11 individual sets	GPL570	GSE32924 Samples	Nonlesional atopic dermatitis skin is characterized by broad terminal differentiation defects and variable immune abnormalities.	J Allergy Clin Immunol.
atopic dermatitis	SCAMP1	Expression profiling by array, count, 3 development stage, 2 disease state, 11 individual sets	GPL570	GSE32924 Samples	Nonlesional atopic dermatitis skin is characterized by broad terminal differentiation defects and variable immune abnormalities.	J Allergy Clin Immunol.
atopic dermatitis	HIST2H2BE	Expression profiling by array, count, 3 development stage, 2 disease state, 11 individual sets	GPL570	GSE32924 Samples	Nonlesional atopic dermatitis skin is characterized by broad terminal differentiation defects and variable immune abnormalities.	J Allergy Clin Immunol.
atopic dermatitis	PNRC2	Expression profiling by array, count, 3 development stage, 2 disease state, 11 individual sets	GPL570	GSE32924 Samples	Nonlesional atopic dermatitis skin is characterized by broad terminal differentiation defects and variable immune abnormalities.	J Allergy Clin Immunol.

atopic dermatitis	CAPN7	Expression profiling by array, count, 3 development stage, 2 disease state, 11 individual sets	GPL570	GSE32924 Samples	Nonlesional atopic dermatitis skin is characterized by broad terminal differentiation defects and variable immune abnormalities.	J Allergy Clin Immunol.
atopic dermatitis	AKTIP	Expression profiling by array, count, 3 development stage, 2 disease state, 11 individual sets	GPL570	GSE32924 Samples	Nonlesional atopic dermatitis skin is characterized by broad terminal differentiation defects and variable immune abnormalities.	J Allergy Clin Immunol.
atopic dermatitis	GOLGA7	Expression profiling by array, count, 3 development stage, 2 disease state, 11 individual sets	GPL570	GSE32924 Samples	Nonlesional atopic dermatitis skin is characterized by broad terminal differentiation defects and variable immune abnormalities.	J Allergy Clin Immunol.
atopic dermatitis	ZNF277	Expression profiling by array, count, 3 development stage, 2 disease state, 11 individual sets	GPL570	GSE32924 Samples	Nonlesional atopic dermatitis skin is characterized by broad terminal differentiation defects and variable immune abnormalities.	J Allergy Clin Immunol.
atopic dermatitis	RDX	Expression profiling by array, count, 3 development stage, 2 disease state, 11 individual sets	GPL570	GSE32924 Samples	Nonlesional atopic dermatitis skin is characterized by broad terminal differentiation defects and variable immune abnormalities.	J Allergy Clin Immunol.
atopic dermatitis	APPL1	Expression profiling by array, count, 3 development stage, 2 disease state, 11 individual sets	GPL570	GSE32924 Samples	Nonlesional atopic dermatitis skin is characterized by broad terminal differentiation defects and variable immune abnormalities.	J Allergy Clin Immunol.
atopic dermatitis	RIT1	Expression profiling by array, count, 3 development stage, 2 disease state, 11 individual sets	GPL570	GSE32924 Samples	Nonlesional atopic dermatitis skin is characterized by broad terminal differentiation defects and variable immune abnormalities.	J Allergy Clin Immunol.
atopic dermatitis	FXR1	Expression profiling by array, count, 3 development stage, 2 disease state, 11 individual sets	GPL570	GSE32924 Samples	Nonlesional atopic dermatitis skin is characterized by broad terminal differentiation defects and variable immune abnormalities.	J Allergy Clin Immunol.
atopic dermatitis	SPIRE1	Expression profiling by array, count, 3 development stage, 2 disease state, 11 individual sets	GPL570	GSE32924 Samples	Nonlesional atopic dermatitis skin is characterized by broad terminal differentiation defects and variable immune abnormalities.	J Allergy Clin Immunol.
atopic dermatitis	GTF2H1	Expression profiling by array, count, 3 development stage, 2 disease state, 11 individual sets	GPL570	GSE32924 Samples	Nonlesional atopic dermatitis skin is characterized by broad terminal differentiation defects and variable immune abnormalities.	J Allergy Clin Immunol.
atopic dermatitis	TMEM43	Expression profiling by array, count, 3 development stage, 2 disease state, 11 individual sets	GPL570	GSE32924 Samples	Nonlesional atopic dermatitis skin is characterized by broad terminal differentiation defects and variable immune abnormalities.	J Allergy Clin Immunol.
atopic dermatitis	PICALM	Expression profiling by array, count, 3 development stage, 2 disease state, 11 individual sets	GPL570	GSE32924 Samples	Nonlesional atopic dermatitis skin is characterized by broad terminal differentiation defects and variable immune abnormalities.	J Allergy Clin Immunol.
atopic dermatitis	BCL6	Expression profiling by array, count, 3 development stage, 2 disease state, 11 individual sets	GPL570	GSE32924 Samples	Nonlesional atopic dermatitis skin is characterized by broad terminal differentiation defects and variable immune abnormalities.	J Allergy Clin Immunol.
atopic dermatitis	STX16	Expression profiling by array, count, 3 development stage, 2 disease state, 11 individual sets	GPL570	GSE32924 Samples	Nonlesional atopic dermatitis skin is characterized by broad terminal differentiation defects and variable immune abnormalities.	J Allergy Clin Immunol.
atopic dermatitis	ALOXE3	Expression profiling by array, count, 3 development stage, 2 disease state, 11 individual sets	GPL570	GSE32924 Samples	Nonlesional atopic dermatitis skin is characterized by broad terminal differentiation defects and variable immune abnormalities.	J Allergy Clin Immunol.
atopic dermatitis	ATP2C2	Expression profiling by array, count, 3 development stage, 2 disease state, 11 individual sets	GPL570	GSE32924 Samples	Nonlesional atopic dermatitis skin is characterized by broad terminal differentiation defects and variable immune abnormalities.	J Allergy Clin Immunol.
atopic dermatitis	C3ORF52	Expression profiling by array, count, 3 development stage, 2 disease state, 11 individual sets	GPL570	GSE32924 Samples	Nonlesional atopic dermatitis skin is characterized by broad terminal differentiation defects and variable immune abnormalities.	J Allergy Clin Immunol.
atopic dermatitis	SOCS5	Expression profiling by array, count, 3 development stage, 2 disease state, 11 individual sets	GPL570	GSE32924 Samples	Nonlesional atopic dermatitis skin is characterized by broad terminal differentiation defects and variable immune abnormalities.	J Allergy Clin Immunol.
atopic dermatitis	NIPBL	Expression profiling by array, count, 3 development stage, 2 disease state, 11 individual sets	GPL570	GSE32924 Samples	Nonlesional atopic dermatitis skin is characterized by broad terminal differentiation defects and variable immune abnormalities.	J Allergy Clin Immunol.
atopic dermatitis	USP34	Expression profiling by array, count, 3 development stage, 2 disease state, 11 individual sets	GPL570	GSE32924 Samples	Nonlesional atopic dermatitis skin is characterized by broad terminal differentiation defects and variable immune abnormalities.	J Allergy Clin Immunol.
atopic dermatitis	DCTN4	Expression profiling by array, count, 3 development stage, 2 disease state, 11 individual sets	GPL570	GSE32924 Samples	Nonlesional atopic dermatitis skin is characterized by broad terminal differentiation defects and variable immune abnormalities.	J Allergy Clin Immunol.
atopic dermatitis	PSEN1	Expression profiling by array, count, 3 development stage, 2 disease state, 11 individual sets	GPL570	GSE32924 Samples	Nonlesional atopic dermatitis skin is characterized by broad terminal differentiation defects and variable immune abnormalities.	J Allergy Clin Immunol.
atopic dermatitis	APOOL	Expression profiling by array, count, 3 development stage, 2 disease state, 11 individual sets	GPL570	GSE32924 Samples	Nonlesional atopic dermatitis skin is characterized by broad terminal differentiation defects and variable immune abnormalities.	J Allergy Clin Immunol.
atopic dermatitis	EML4	Expression profiling by array, count, 3 development stage, 2 disease state, 11 individual sets	GPL570	GSE32924 Samples	Nonlesional atopic dermatitis skin is characterized by broad terminal differentiation defects and variable immune abnormalities.	J Allergy Clin Immunol.
atopic dermatitis	USP15	Expression profiling by array, count, 3 development stage, 2 disease state, 11 individual sets	GPL570	GSE32924 Samples	Nonlesional atopic dermatitis skin is characterized by broad terminal differentiation defects and variable immune abnormalities.	J Allergy Clin Immunol.
atopic dermatitis	BICD2	Expression profiling by array, count, 3 development stage, 2 disease state, 11 individual sets	GPL570	GSE32924 Samples	Nonlesional atopic dermatitis skin is characterized by broad terminal differentiation defects and variable immune abnormalities.	J Allergy Clin Immunol.
atopic dermatitis	AAGAB	Expression profiling by array, count, 3 development stage, 2 disease state, 11 individual sets	GPL570	GSE32924 Samples	Nonlesional atopic dermatitis skin is characterized by broad terminal differentiation defects and variable immune abnormalities.	J Allergy Clin Immunol.
atopic dermatitis	SRGAP2P1	Expression profiling by array, count, 3 development stage, 2 disease state, 11 individual sets	GPL570	GSE32924 Samples	Nonlesional atopic dermatitis skin is characterized by broad terminal differentiation defects and variable immune abnormalities.	J Allergy Clin Immunol.
atopic dermatitis	LIMS1	Expression profiling by array, count, 3 development stage, 2 disease state, 11 individual sets	GPL570	GSE32924 Samples	Nonlesional atopic dermatitis skin is characterized by broad terminal differentiation defects and variable immune abnormalities.	J Allergy Clin Immunol.
atopic dermatitis	NAV3	Expression profiling by array, count, 3 development stage, 2 disease state, 11 individual sets	GPL570	GSE32924 Samples	Nonlesional atopic dermatitis skin is characterized by broad terminal differentiation defects and variable immune abnormalities.	J Allergy Clin Immunol.
atopic dermatitis	RAPGEF6	Expression profiling by array, count, 3 development stage, 2 disease state, 11 individual sets	GPL570	GSE32924 Samples	Nonlesional atopic dermatitis skin is characterized by broad terminal differentiation defects and variable immune abnormalities.	J Allergy Clin Immunol.
atopic dermatitis	TSPAN5	Expression profiling by array, count, 3 development stage, 2 disease state, 11 individual sets	GPL570	GSE32924 Samples	Nonlesional atopic dermatitis skin is characterized by broad terminal differentiation defects and variable immune abnormalities.	J Allergy Clin Immunol.
atopic dermatitis	ALOX12	Expression profiling by array, count, 3 development stage, 2 disease state, 11 individual sets	GPL570	GSE32924 Samples	Nonlesional atopic dermatitis skin is characterized by broad terminal differentiation defects and variable immune abnormalities.	J Allergy Clin Immunol.
atopic dermatitis	SLC1A6	Expression profiling by array, count, 3 development stage, 2 disease state, 11 individual sets	GPL570	GSE32924 Samples	Nonlesional atopic dermatitis skin is characterized by broad terminal differentiation defects and variable immune abnormalities.	J Allergy Clin Immunol.
atopic dermatitis	PRNP	Expression profiling by array, count, 3 development stage, 2 disease state, 11 individual sets	GPL570	GSE32924 Samples	Nonlesional atopic dermatitis skin is characterized by broad terminal differentiation defects and variable immune abnormalities.	J Allergy Clin Immunol.
atopic dermatitis	PTPN11	Expression profiling by array, count, 3 development stage, 2 disease state, 11 individual sets	GPL570	GSE32924 Samples	Nonlesional atopic dermatitis skin is characterized by broad terminal differentiation defects and variable immune abnormalities.	J Allergy Clin Immunol.
atopic dermatitis	NA	Expression profiling by array, count, 3 development stage, 2 disease state, 11 individual sets	GPL570	GSE32924 Samples	Nonlesional atopic dermatitis skin is characterized by broad terminal differentiation defects and variable immune abnormalities.	J Allergy Clin Immunol.
atopic dermatitis	RAB5A	Expression profiling by array, count, 3 development stage, 2 disease state, 11 individual sets	GPL570	GSE32924 Samples	Nonlesional atopic dermatitis skin is characterized by broad terminal differentiation defects and variable immune abnormalities.	J Allergy Clin Immunol.
atopic dermatitis	NOTCH2	Expression profiling by array, count, 3 development stage, 2 disease state, 11 individual sets	GPL570	GSE32924 Samples	Nonlesional atopic dermatitis skin is characterized by broad terminal differentiation defects and variable immune abnormalities.	J Allergy Clin Immunol.
atopic dermatitis	PALMD	Expression profiling by array, count, 3 development stage, 2 disease state, 11 individual sets	GPL570	GSE32924 Samples	Nonlesional atopic dermatitis skin is characterized by broad terminal differentiation defects and variable immune abnormalities.	J Allergy Clin Immunol.
atopic dermatitis	IGFBP5	Expression profiling by array, count, 3 development stage, 2 disease state, 11 individual sets	GPL570	GSE32924 Samples	Nonlesional atopic dermatitis skin is characterized by broad terminal differentiation defects and variable immune abnormalities.	J Allergy Clin Immunol.

atopic dermatitis	LOC647979	Expression profiling by array, count, 3 development stage, 2 disease state, 11 individual sets	GPL570	GSE32924 Samples	Nonlesional atopic dermatitis skin is characterized by broad terminal differentiation defects and variable immune abnormalities.	J Allergy Clin Immunol.
atopic dermatitis	TMED2	Expression profiling by array, count, 3 development stage, 2 disease state, 11 individual sets	GPL570	GSE32924 Samples	Nonlesional atopic dermatitis skin is characterized by broad terminal differentiation defects and variable immune abnormalities.	J Allergy Clin Immunol.
atopic dermatitis	TMED2	Expression profiling by array, count, 3 development stage, 2 disease state, 11 individual sets	GPL570	GSE32924 Samples	Nonlesional atopic dermatitis skin is characterized by broad terminal differentiation defects and variable immune abnormalities.	J Allergy Clin Immunol.
atopic dermatitis	EZR	Expression profiling by array, count, 3 development stage, 2 disease state, 11 individual sets	GPL570	GSE32924 Samples	Nonlesional atopic dermatitis skin is characterized by broad terminal differentiation defects and variable immune abnormalities.	J Allergy Clin Immunol.
atopic dermatitis	RFWD2	Expression profiling by array, count, 3 development stage, 2 disease state, 11 individual sets	GPL570	GSE32924 Samples	Nonlesional atopic dermatitis skin is characterized by broad terminal differentiation defects and variable immune abnormalities.	J Allergy Clin Immunol.
atopic dermatitis	UBE2J1	Expression profiling by array, count, 3 development stage, 2 disease state, 11 individual sets	GPL570	GSE32924 Samples	Nonlesional atopic dermatitis skin is characterized by broad terminal differentiation defects and variable immune abnormalities.	J Allergy Clin Immunol.
atopic dermatitis	DCTD	Expression profiling by array, count, 3 development stage, 2 disease state, 11 individual sets	GPL570	GSE32924 Samples	Nonlesional atopic dermatitis skin is characterized by broad terminal differentiation defects and variable immune abnormalities.	J Allergy Clin Immunol.
atopic dermatitis	UBE2J1	Expression profiling by array, count, 3 development stage, 2 disease state, 11 individual sets	GPL570	GSE32924 Samples	Nonlesional atopic dermatitis skin is characterized by broad terminal differentiation defects and variable immune abnormalities.	J Allergy Clin Immunol.
atopic dermatitis	MED6	Expression profiling by array, count, 3 development stage, 2 disease state, 11 individual sets	GPL570	GSE32924 Samples	Nonlesional atopic dermatitis skin is characterized by broad terminal differentiation defects and variable immune abnormalities.	J Allergy Clin Immunol.
atopic dermatitis	PPP2R5C	Expression profiling by array, count, 3 development stage, 2 disease state, 11 individual sets	GPL570	GSE32924 Samples	Nonlesional atopic dermatitis skin is characterized by broad terminal differentiation defects and variable immune abnormalities.	J Allergy Clin Immunol.
atopic dermatitis	CSNK1A1	Expression profiling by array, count, 3 development stage, 2 disease state, 11 individual sets	GPL570	GSE32924 Samples	Nonlesional atopic dermatitis skin is characterized by broad terminal differentiation defects and variable immune abnormalities.	J Allergy Clin Immunol.
atopic dermatitis	MAN2A1	Expression profiling by array, count, 3 development stage, 2 disease state, 11 individual sets	GPL570	GSE32924 Samples	Nonlesional atopic dermatitis skin is characterized by broad terminal differentiation defects and variable immune abnormalities.	J Allergy Clin Immunol.
atopic dermatitis	RIOK3	Expression profiling by array, count, 3 development stage, 2 disease state, 11 individual sets	GPL570	GSE32924 Samples	Nonlesional atopic dermatitis skin is characterized by broad terminal differentiation defects and variable immune abnormalities.	J Allergy Clin Immunol.
atopic dermatitis	VTA1	Expression profiling by array, count, 3 development stage, 2 disease state, 11 individual sets	GPL570	GSE32924 Samples	Nonlesional atopic dermatitis skin is characterized by broad terminal differentiation defects and variable immune abnormalities.	J Allergy Clin Immunol.
atopic dermatitis	PRKDC	Expression profiling by array, count, 3 development stage, 2 disease state, 11 individual sets	GPL570	GSE32924 Samples	Nonlesional atopic dermatitis skin is characterized by broad terminal differentiation defects and variable immune abnormalities.	J Allergy Clin Immunol.
atopic dermatitis	UBXN4	Expression profiling by array, count, 3 development stage, 2 disease state, 11 individual sets	GPL570	GSE32924 Samples	Nonlesional atopic dermatitis skin is characterized by broad terminal differentiation defects and variable immune abnormalities.	J Allergy Clin Immunol.
atopic dermatitis	PRPF4B	Expression profiling by array, count, 3 development stage, 2 disease state, 11 individual sets	GPL570	GSE32924 Samples	Nonlesional atopic dermatitis skin is characterized by broad terminal differentiation defects and variable immune abnormalities.	J Allergy Clin Immunol.
atopic dermatitis	MED30	Expression profiling by array, count, 3 development stage, 2 disease state, 11 individual sets	GPL570	GSE32924 Samples	Nonlesional atopic dermatitis skin is characterized by broad terminal differentiation defects and variable immune abnormalities.	J Allergy Clin Immunol.
atopic dermatitis	CASP1	Expression profiling by array, count, 3 development stage, 2 disease state, 11 individual sets	GPL570	GSE32924 Samples	Nonlesional atopic dermatitis skin is characterized by broad terminal differentiation defects and variable immune abnormalities.	J Allergy Clin Immunol.
atopic dermatitis	PLEKHF2	Expression profiling by array, count, 3 development stage, 2 disease state, 11 individual sets	GPL570	GSE32924 Samples	Nonlesional atopic dermatitis skin is characterized by broad terminal differentiation defects and variable immune abnormalities.	J Allergy Clin Immunol.
atopic dermatitis	PAPOLA	Expression profiling by array, count, 3 development stage, 2 disease state, 11 individual sets	GPL570	GSE32924 Samples	Nonlesional atopic dermatitis skin is characterized by broad terminal differentiation defects and variable immune abnormalities.	J Allergy Clin Immunol.
atopic dermatitis	GPR177	Expression profiling by array, count, 3 development stage, 2 disease state, 11 individual sets	GPL570	GSE32924 Samples	Nonlesional atopic dermatitis skin is characterized by broad terminal differentiation defects and variable immune abnormalities.	J Allergy Clin Immunol.
atopic dermatitis	MED6	Expression profiling by array, count, 3 development stage, 2 disease state, 11 individual sets	GPL570	GSE32924 Samples	Nonlesional atopic dermatitis skin is characterized by broad terminal differentiation defects and variable immune abnormalities.	J Allergy Clin Immunol.
atopic dermatitis	WBP11	Expression profiling by array, count, 3 development stage, 2 disease state, 11 individual sets	GPL570	GSE32924 Samples	Nonlesional atopic dermatitis skin is characterized by broad terminal differentiation defects and variable immune abnormalities.	J Allergy Clin Immunol.
atopic dermatitis	PGCP	Expression profiling by array, count, 3 development stage, 2 disease state, 11 individual sets	GPL570	GSE32924 Samples	Nonlesional atopic dermatitis skin is characterized by broad terminal differentiation defects and variable immune abnormalities.	J Allergy Clin Immunol.
atopic dermatitis	C10ORF18	Expression profiling by array, count, 3 development stage, 2 disease state, 11 individual sets	GPL570	GSE32924 Samples	Nonlesional atopic dermatitis skin is characterized by broad terminal differentiation defects and variable immune abnormalities.	J Allergy Clin Immunol.
atopic dermatitis	BMP2	Expression profiling by array, count, 3 development stage, 2 disease state, 11 individual sets	GPL570	GSE32924 Samples	Nonlesional atopic dermatitis skin is characterized by broad terminal differentiation defects and variable immune abnormalities.	J Allergy Clin Immunol.
atopic dermatitis	CTNND1	Expression profiling by array, count, 3 development stage, 2 disease state, 11 individual sets	GPL570	GSE32924 Samples	Nonlesional atopic dermatitis skin is characterized by broad terminal differentiation defects and variable immune abnormalities.	J Allergy Clin Immunol.
atopic dermatitis	EPB41L3	Expression profiling by array, count, 3 development stage, 2 disease state, 11 individual sets	GPL570	GSE32924 Samples	Nonlesional atopic dermatitis skin is characterized by broad terminal differentiation defects and variable immune abnormalities.	J Allergy Clin Immunol.
atopic dermatitis	FYN	Expression profiling by array, count, 3 development stage, 2 disease state, 11 individual sets	GPL570	GSE32924 Samples	Nonlesional atopic dermatitis skin is characterized by broad terminal differentiation defects and variable immune abnormalities.	J Allergy Clin Immunol.
atopic dermatitis	DKK2	Expression profiling by array, count, 3 development stage, 2 disease state, 11 individual sets	GPL570	GSE32924 Samples	Nonlesional atopic dermatitis skin is characterized by broad terminal differentiation defects and variable immune abnormalities.	J Allergy Clin Immunol.
atopic dermatitis	NA	Expression profiling by array, count, 3 development stage, 2 disease state, 11 individual sets	GPL570	GSE32924 Samples	Nonlesional atopic dermatitis skin is characterized by broad terminal differentiation defects and variable immune abnormalities.	J Allergy Clin Immunol.
atopic dermatitis	SVEP1	Expression profiling by array, count, 3 development stage, 2 disease state, 11 individual sets	GPL570	GSE32924 Samples	Nonlesional atopic dermatitis skin is characterized by broad terminal differentiation defects and variable immune abnormalities.	J Allergy Clin Immunol.
atopic dermatitis	NA	Expression profiling by array, count, 3 development stage, 2 disease state, 11 individual sets	GPL570	GSE32924 Samples	Nonlesional atopic dermatitis skin is characterized by broad terminal differentiation defects and variable immune abnormalities.	J Allergy Clin Immunol.
atopic dermatitis	EGR1	Expression profiling by array, count, 3 development stage, 2 disease state, 11 individual sets	GPL570	GSE32924 Samples	Nonlesional atopic dermatitis skin is characterized by broad terminal differentiation defects and variable immune abnormalities.	J Allergy Clin Immunol.
atopic dermatitis	MALAT1	Expression profiling by array, count, 3 development stage, 2 disease state, 11 individual sets	GPL570	GSE32924 Samples	Nonlesional atopic dermatitis skin is characterized by broad terminal differentiation defects and variable immune abnormalities.	J Allergy Clin Immunol.
atopic dermatitis	NCOA1	Expression profiling by array, count, 3 development stage, 2 disease state, 11 individual sets	GPL570	GSE32924 Samples	Nonlesional atopic dermatitis skin is characterized by broad terminal differentiation defects and variable immune abnormalities.	J Allergy Clin Immunol.
atopic dermatitis	ATF6	Expression profiling by array, count, 3 development stage, 2 disease state, 11 individual sets	GPL570	GSE32924 Samples	Nonlesional atopic dermatitis skin is characterized by broad terminal differentiation defects and variable immune abnormalities.	J Allergy Clin Immunol.
atopic dermatitis	C20ORF30	Expression profiling by array, count, 3 development stage, 2 disease state, 11 individual sets	GPL570	GSE32924 Samples	Nonlesional atopic dermatitis skin is characterized by broad terminal differentiation defects and variable immune abnormalities.	J Allergy Clin Immunol.
atopic dermatitis	EID1	Expression profiling by array, count, 3 development stage, 2 disease state, 11 individual sets	GPL570	GSE32924 Samples	Nonlesional atopic dermatitis skin is characterized by broad terminal differentiation defects and variable immune abnormalities.	J Allergy Clin Immunol.
atopic dermatitis	TUG1	Expression profiling by array, count, 3 development stage, 2 disease state, 11 individual sets	GPL570	GSE32924 Samples	Nonlesional atopic dermatitis skin is characterized by broad terminal differentiation defects and variable immune abnormalities.	J Allergy Clin Immunol.

atopic dermatitis	FIBIN	Expression profiling by array, count, 3 development stage, 2 disease state, 11 individual sets	GPL570	GSE32924 Samples	Nonlesional atopic dermatitis skin is characterized by broad terminal differentiation defects and variable immune abnormalities.	J Allergy Clin Immunol.
atopic dermatitis	CPE	Expression profiling by array, count, 3 development stage, 2 disease state, 11 individual sets	GPL570	GSE32924 Samples	Nonlesional atopic dermatitis skin is characterized by broad terminal differentiation defects and variable immune abnormalities.	J Allergy Clin Immunol.
atopic dermatitis	ALDH1A1	Expression profiling by array, count, 3 development stage, 2 disease state, 11 individual sets	GPL570	GSE32924 Samples	Nonlesional atopic dermatitis skin is characterized by broad terminal differentiation defects and variable immune abnormalities.	J Allergy Clin Immunol.
atopic dermatitis	PSAPL1	Expression profiling by array, count, 3 development stage, 2 disease state, 11 individual sets	GPL570	GSE32924 Samples	Nonlesional atopic dermatitis skin is characterized by broad terminal differentiation defects and variable immune abnormalities.	J Allergy Clin Immunol.
atopic dermatitis	CARD18	Expression profiling by array, count, 3 development stage, 2 disease state, 11 individual sets	GPL570	GSE32924 Samples	Nonlesional atopic dermatitis skin is characterized by broad terminal differentiation defects and variable immune abnormalities.	J Allergy Clin Immunol.
atopic dermatitis	ABHD12B	Expression profiling by array, count, 3 development stage, 2 disease state, 11 individual sets	GPL570	GSE32924 Samples	Nonlesional atopic dermatitis skin is characterized by broad terminal differentiation defects and variable immune abnormalities.	J Allergy Clin Immunol.
atopic dermatitis	OGN	Expression profiling by array, count, 3 development stage, 2 disease state, 11 individual sets	GPL570	GSE32924 Samples	Nonlesional atopic dermatitis skin is characterized by broad terminal differentiation defects and variable immune abnormalities.	J Allergy Clin Immunol.
atopic dermatitis	NA	Expression profiling by array, count, 3 development stage, 2 disease state, 11 individual sets	GPL570	GSE32924 Samples	Nonlesional atopic dermatitis skin is characterized by broad terminal differentiation defects and variable immune abnormalities.	J Allergy Clin Immunol.
atopic dermatitis	ZNF512	Expression profiling by array, count, 3 development stage, 2 disease state, 11 individual sets	GPL570	GSE32924 Samples	Nonlesional atopic dermatitis skin is characterized by broad terminal differentiation defects and variable immune abnormalities.	J Allergy Clin Immunol.
atopic dermatitis	NBR1	Expression profiling by array, count, 3 development stage, 2 disease state, 11 individual sets	GPL570	GSE32924 Samples	Nonlesional atopic dermatitis skin is characterized by broad terminal differentiation defects and variable immune abnormalities.	J Allergy Clin Immunol.
atopic dermatitis	CRYZL1	Expression profiling by array, count, 3 development stage, 2 disease state, 11 individual sets	GPL570	GSE32924 Samples	Nonlesional atopic dermatitis skin is characterized by broad terminal differentiation defects and variable immune abnormalities.	J Allergy Clin Immunol.
atopic dermatitis	ZC3H7A	Expression profiling by array, count, 3 development stage, 2 disease state, 11 individual sets	GPL570	GSE32924 Samples	Nonlesional atopic dermatitis skin is characterized by broad terminal differentiation defects and variable immune abnormalities.	J Allergy Clin Immunol.
atopic dermatitis	USP47	Expression profiling by array, count, 3 development stage, 2 disease state, 11 individual sets	GPL570	GSE32924 Samples	Nonlesional atopic dermatitis skin is characterized by broad terminal differentiation defects and variable immune abnormalities.	J Allergy Clin Immunol.
atopic dermatitis	KIAA0776	Expression profiling by array, count, 3 development stage, 2 disease state, 11 individual sets	GPL570	GSE32924 Samples	Nonlesional atopic dermatitis skin is characterized by broad terminal differentiation defects and variable immune abnormalities.	J Allergy Clin Immunol.
atopic dermatitis	ARIH1	Expression profiling by array, count, 3 development stage, 2 disease state, 11 individual sets	GPL570	GSE32924 Samples	Nonlesional atopic dermatitis skin is characterized by broad terminal differentiation defects and variable immune abnormalities.	J Allergy Clin Immunol.
atopic dermatitis	ZXDC	Expression profiling by array, count, 3 development stage, 2 disease state, 11 individual sets	GPL570	GSE32924 Samples	Nonlesional atopic dermatitis skin is characterized by broad terminal differentiation defects and variable immune abnormalities.	J Allergy Clin Immunol.
atopic dermatitis	MLF1IP	Expression profiling by array, count, 3 development stage, 2 disease state, 11 individual sets	GPL570	GSE32924 Samples	Nonlesional atopic dermatitis skin is characterized by broad terminal differentiation defects and variable immune abnormalities.	J Allergy Clin Immunol.
atopic dermatitis	NA	Expression profiling by array, count, 3 development stage, 2 disease state, 11 individual sets	GPL570	GSE32924 Samples	Nonlesional atopic dermatitis skin is characterized by broad terminal differentiation defects and variable immune abnormalities.	J Allergy Clin Immunol.
atopic dermatitis	IGDCC4	Expression profiling by array, count, 3 development stage, 2 disease state, 11 individual sets	GPL570	GSE32924 Samples	Nonlesional atopic dermatitis skin is characterized by broad terminal differentiation defects and variable immune abnormalities.	J Allergy Clin Immunol.
atopic dermatitis	LRRC17	Expression profiling by array, count, 3 development stage, 2 disease state, 11 individual sets	GPL570	GSE32924 Samples	Nonlesional atopic dermatitis skin is characterized by broad terminal differentiation defects and variable immune abnormalities.	J Allergy Clin Immunol.
atopic dermatitis	CYBRD1	Expression profiling by array, count, 3 development stage, 2 disease state, 11 individual sets	GPL570	GSE32924 Samples	Nonlesional atopic dermatitis skin is characterized by broad terminal differentiation defects and variable immune abnormalities.	J Allergy Clin Immunol.
atopic dermatitis	COL8A2	Expression profiling by array, count, 3 development stage, 2 disease state, 11 individual sets	GPL570	GSE32924 Samples	Nonlesional atopic dermatitis skin is characterized by broad terminal differentiation defects and variable immune abnormalities.	J Allergy Clin Immunol.
atopic dermatitis	NA	Expression profiling by array, count, 3 development stage, 2 disease state, 11 individual sets	GPL570	GSE32924 Samples	Nonlesional atopic dermatitis skin is characterized by broad terminal differentiation defects and variable immune abnormalities.	J Allergy Clin Immunol.
atopic dermatitis	DDX42	Expression profiling by array, count, 3 development stage, 2 disease state, 11 individual sets	GPL570	GSE32924 Samples	Nonlesional atopic dermatitis skin is characterized by broad terminal differentiation defects and variable immune abnormalities.	J Allergy Clin Immunol.
atopic dermatitis	CCDC109A	Expression profiling by array, count, 3 development stage, 2 disease state, 11 individual sets	GPL570	GSE32924 Samples	Nonlesional atopic dermatitis skin is characterized by broad terminal differentiation defects and variable immune abnormalities.	J Allergy Clin Immunol.
atopic dermatitis	AP3B1	Expression profiling by array, count, 3 development stage, 2 disease state, 11 individual sets	GPL570	GSE32924 Samples	Nonlesional atopic dermatitis skin is characterized by broad terminal differentiation defects and variable immune abnormalities.	J Allergy Clin Immunol.
atopic dermatitis	SLC33A1	Expression profiling by array, count, 3 development stage, 2 disease state, 11 individual sets	GPL570	GSE32924 Samples	Nonlesional atopic dermatitis skin is characterized by broad terminal differentiation defects and variable immune abnormalities.	J Allergy Clin Immunol.
atopic dermatitis	LRCH1	Expression profiling by array, count, 3 development stage, 2 disease state, 11 individual sets	GPL570	GSE32924 Samples	Nonlesional atopic dermatitis skin is characterized by broad terminal differentiation defects and variable immune abnormalities.	J Allergy Clin Immunol.
atopic dermatitis	MLLT10	Expression profiling by array, count, 3 development stage, 2 disease state, 11 individual sets	GPL570	GSE32924 Samples	Nonlesional atopic dermatitis skin is characterized by broad terminal differentiation defects and variable immune abnormalities.	J Allergy Clin Immunol.
atopic dermatitis	CTSO	Expression profiling by array, count, 3 development stage, 2 disease state, 11 individual sets	GPL570	GSE32924 Samples	Nonlesional atopic dermatitis skin is characterized by broad terminal differentiation defects and variable immune abnormalities.	J Allergy Clin Immunol.
atopic dermatitis	PDE8A	Expression profiling by array, count, 3 development stage, 2 disease state, 11 individual sets	GPL570	GSE32924 Samples	Nonlesional atopic dermatitis skin is characterized by broad terminal differentiation defects and variable immune abnormalities.	J Allergy Clin Immunol.
atopic dermatitis	NF1	Expression profiling by array, count, 3 development stage, 2 disease state, 11 individual sets	GPL570	GSE32924 Samples	Nonlesional atopic dermatitis skin is characterized by broad terminal differentiation defects and variable immune abnormalities.	J Allergy Clin Immunol.
atopic dermatitis	M6PR	Expression profiling by array, count, 3 development stage, 2 disease state, 11 individual sets	GPL570	GSE32924 Samples	Nonlesional atopic dermatitis skin is characterized by broad terminal differentiation defects and variable immune abnormalities.	J Allergy Clin Immunol.
atopic dermatitis	MON1B	Expression profiling by array, count, 3 development stage, 2 disease state, 11 individual sets	GPL570	GSE32924 Samples	Nonlesional atopic dermatitis skin is characterized by broad terminal differentiation defects and variable immune abnormalities.	J Allergy Clin Immunol.
atopic dermatitis	RICS	Expression profiling by array, count, 3 development stage, 2 disease state, 11 individual sets	GPL570	GSE32924 Samples	Nonlesional atopic dermatitis skin is characterized by broad terminal differentiation defects and variable immune abnormalities.	J Allergy Clin Immunol.
atopic dermatitis	UBE4B	Expression profiling by array, count, 3 development stage, 2 disease state, 11 individual sets	GPL570	GSE32924 Samples	Nonlesional atopic dermatitis skin is characterized by broad terminal differentiation defects and variable immune abnormalities.	J Allergy Clin Immunol.
atopic dermatitis	ARID1B	Expression profiling by array, count, 3 development stage, 2 disease state, 11 individual sets	GPL570	GSE32924 Samples	Nonlesional atopic dermatitis skin is characterized by broad terminal differentiation defects and variable immune abnormalities.	J Allergy Clin Immunol.
atopic dermatitis	EPHA4	Expression profiling by array, count, 3 development stage, 2 disease state, 11 individual sets	GPL570	GSE32924 Samples	Nonlesional atopic dermatitis skin is characterized by broad terminal differentiation defects and variable immune abnormalities.	J Allergy Clin Immunol.
atopic dermatitis	COBL	Expression profiling by array, count, 3 development stage, 2 disease state, 11 individual sets	GPL570	GSE32924 Samples	Nonlesional atopic dermatitis skin is characterized by broad terminal differentiation defects and variable immune abnormalities.	J Allergy Clin Immunol.
atopic dermatitis	SLITRK6	Expression profiling by array, count, 3 development stage, 2 disease state, 11 individual sets	GPL570	GSE32924 Samples	Nonlesional atopic dermatitis skin is characterized by broad terminal differentiation defects and variable immune abnormalities.	J Allergy Clin Immunol.
atopic dermatitis	NAP1L2	Expression profiling by array, count, 3 development stage, 2 disease state, 11 individual sets	GPL570	GSE32924 Samples	Nonlesional atopic dermatitis skin is characterized by broad terminal differentiation defects and variable immune abnormalities.	J Allergy Clin Immunol.

atopic dermatitis	NA	Expression profiling by array, count, 3 development stage, 2 disease state, 11 individual sets	GPL570	GSE32924 Samples	Nonlesional atopic dermatitis skin is characterized by broad terminal differentiation defects and variable immune abnormalities.	J Allergy Clin Immunol.
atopic dermatitis	ALG10B	Expression profiling by array, count, 3 development stage, 2 disease state, 11 individual sets	GPL570	GSE32924 Samples	Nonlesional atopic dermatitis skin is characterized by broad terminal differentiation defects and variable immune abnormalities.	J Allergy Clin Immunol.
atopic dermatitis	LOC730102	Expression profiling by array, count, 3 development stage, 2 disease state, 11 individual sets	GPL570	GSE32924 Samples	Nonlesional atopic dermatitis skin is characterized by broad terminal differentiation defects and variable immune abnormalities.	J Allergy Clin Immunol.
atopic dermatitis	MED23	Expression profiling by array, count, 3 development stage, 2 disease state, 11 individual sets	GPL570	GSE32924 Samples	Nonlesional atopic dermatitis skin is characterized by broad terminal differentiation defects and variable immune abnormalities.	J Allergy Clin Immunol.
atopic dermatitis	ZDHHC9	Expression profiling by array, count, 3 development stage, 2 disease state, 11 individual sets	GPL570	GSE32924 Samples	Nonlesional atopic dermatitis skin is characterized by broad terminal differentiation defects and variable immune abnormalities.	J Allergy Clin Immunol.
atopic dermatitis	SAV1	Expression profiling by array, count, 3 development stage, 2 disease state, 11 individual sets	GPL570	GSE32924 Samples	Nonlesional atopic dermatitis skin is characterized by broad terminal differentiation defects and variable immune abnormalities.	J Allergy Clin Immunol.
atopic dermatitis	NAP1L3	Expression profiling by array, count, 3 development stage, 2 disease state, 11 individual sets	GPL570	GSE32924 Samples	Nonlesional atopic dermatitis skin is characterized by broad terminal differentiation defects and variable immune abnormalities.	J Allergy Clin Immunol.
atopic dermatitis	MACROD2	Expression profiling by array, count, 3 development stage, 2 disease state, 11 individual sets	GPL570	GSE32924 Samples	Nonlesional atopic dermatitis skin is characterized by broad terminal differentiation defects and variable immune abnormalities.	J Allergy Clin Immunol.
atopic dermatitis	C14ORF132	Expression profiling by array, count, 3 development stage, 2 disease state, 11 individual sets	GPL570	GSE32924 Samples	Nonlesional atopic dermatitis skin is characterized by broad terminal differentiation defects and variable immune abnormalities.	J Allergy Clin Immunol.
atopic dermatitis	NA	Expression profiling by array, count, 3 development stage, 2 disease state, 11 individual sets	GPL570	GSE32924 Samples	Nonlesional atopic dermatitis skin is characterized by broad terminal differentiation defects and variable immune abnormalities.	J Allergy Clin Immunol.
atopic dermatitis	BCHE	Expression profiling by array, count, 3 development stage, 2 disease state, 11 individual sets	GPL570	GSE32924 Samples	Nonlesional atopic dermatitis skin is characterized by broad terminal differentiation defects and variable immune abnormalities.	J Allergy Clin Immunol.
atopic dermatitis	SEC63	Expression profiling by array, count, 3 development stage, 2 disease state, 11 individual sets	GPL570	GSE32924 Samples	Nonlesional atopic dermatitis skin is characterized by broad terminal differentiation defects and variable immune abnormalities.	J Allergy Clin Immunol.
atopic dermatitis	DHX36	Expression profiling by array, count, 3 development stage, 2 disease state, 11 individual sets	GPL570	GSE32924 Samples	Nonlesional atopic dermatitis skin is characterized by broad terminal differentiation defects and variable immune abnormalities.	J Allergy Clin Immunol.
atopic dermatitis	NA	Expression profiling by array, count, 3 development stage, 2 disease state, 11 individual sets	GPL570	GSE32924 Samples	Nonlesional atopic dermatitis skin is characterized by broad terminal differentiation defects and variable immune abnormalities.	J Allergy Clin Immunol.
atopic dermatitis	SFRS2IP	Expression profiling by array, count, 3 development stage, 2 disease state, 11 individual sets	GPL570	GSE32924 Samples	Nonlesional atopic dermatitis skin is characterized by broad terminal differentiation defects and variable immune abnormalities.	J Allergy Clin Immunol.
atopic dermatitis	MYO6	Expression profiling by array, count, 3 development stage, 2 disease state, 11 individual sets	GPL570	GSE32924 Samples	Nonlesional atopic dermatitis skin is characterized by broad terminal differentiation defects and variable immune abnormalities.	J Allergy Clin Immunol.
atopic dermatitis	GNAS	Expression profiling by array, count, 3 development stage, 2 disease state, 11 individual sets	GPL570	GSE32924 Samples	Nonlesional atopic dermatitis skin is characterized by broad terminal differentiation defects and variable immune abnormalities.	J Allergy Clin Immunol.
atopic dermatitis	USP16	Expression profiling by array, count, 3 development stage, 2 disease state, 11 individual sets	GPL570	GSE32924 Samples	Nonlesional atopic dermatitis skin is characterized by broad terminal differentiation defects and variable immune abnormalities.	J Allergy Clin Immunol.
atopic dermatitis	ATF1	Expression profiling by array, count, 3 development stage, 2 disease state, 11 individual sets	GPL570	GSE32924 Samples	Nonlesional atopic dermatitis skin is characterized by broad terminal differentiation defects and variable immune abnormalities.	J Allergy Clin Immunol.
atopic dermatitis	PRKCH	Expression profiling by array, count, 3 development stage, 2 disease state, 11 individual sets	GPL570	GSE32924 Samples	Nonlesional atopic dermatitis skin is characterized by broad terminal differentiation defects and variable immune abnormalities.	J Allergy Clin Immunol.
atopic dermatitis	PLDN	Expression profiling by array, count, 3 development stage, 2 disease state, 11 individual sets	GPL570	GSE32924 Samples	Nonlesional atopic dermatitis skin is characterized by broad terminal differentiation defects and variable immune abnormalities.	J Allergy Clin Immunol.
atopic dermatitis	ZW10	Expression profiling by array, count, 3 development stage, 2 disease state, 11 individual sets	GPL570	GSE32924 Samples	Nonlesional atopic dermatitis skin is characterized by broad terminal differentiation defects and variable immune abnormalities.	J Allergy Clin Immunol.
atopic dermatitis	CTDSPL2	Expression profiling by array, count, 3 development stage, 2 disease state, 11 individual sets	GPL570	GSE32924 Samples	Nonlesional atopic dermatitis skin is characterized by broad terminal differentiation defects and variable immune abnormalities.	J Allergy Clin Immunol.
atopic dermatitis	MAP4K4	Expression profiling by array, count, 3 development stage, 2 disease state, 11 individual sets	GPL570	GSE32924 Samples	Nonlesional atopic dermatitis skin is characterized by broad terminal differentiation defects and variable immune abnormalities.	J Allergy Clin Immunol.
atopic dermatitis	SRPK2	Expression profiling by array, count, 3 development stage, 2 disease state, 11 individual sets	GPL570	GSE32924 Samples	Nonlesional atopic dermatitis skin is characterized by broad terminal differentiation defects and variable immune abnormalities.	J Allergy Clin Immunol.
atopic dermatitis	CXORF26	Expression profiling by array, count, 3 development stage, 2 disease state, 11 individual sets	GPL570	GSE32924 Samples	Nonlesional atopic dermatitis skin is characterized by broad terminal differentiation defects and variable immune abnormalities.	J Allergy Clin Immunol.
atopic dermatitis	ANKHD1	Expression profiling by array, count, 3 development stage, 2 disease state, 11 individual sets	GPL570	GSE32924 Samples	Nonlesional atopic dermatitis skin is characterized by broad terminal differentiation defects and variable immune abnormalities.	J Allergy Clin Immunol.
atopic dermatitis	C16ORF87	Expression profiling by array, count, 3 development stage, 2 disease state, 11 individual sets	GPL570	GSE32924 Samples	Nonlesional atopic dermatitis skin is characterized by broad terminal differentiation defects and variable immune abnormalities.	J Allergy Clin Immunol.
atopic dermatitis	NA	Expression profiling by array, count, 3 development stage, 2 disease state, 11 individual sets	GPL570	GSE32924 Samples	Nonlesional atopic dermatitis skin is characterized by broad terminal differentiation defects and variable immune abnormalities.	J Allergy Clin Immunol.
atopic dermatitis	GAPVD1	Expression profiling by array, count, 3 development stage, 2 disease state, 11 individual sets	GPL570	GSE32924 Samples	Nonlesional atopic dermatitis skin is characterized by broad terminal differentiation defects and variable immune abnormalities.	J Allergy Clin Immunol.
atopic dermatitis	RICTOR	Expression profiling by array, count, 3 development stage, 2 disease state, 11 individual sets	GPL570	GSE32924 Samples	Nonlesional atopic dermatitis skin is characterized by broad terminal differentiation defects and variable immune abnormalities.	J Allergy Clin Immunol.
atopic dermatitis	ZNF638	Expression profiling by array, count, 3 development stage, 2 disease state, 11 individual sets	GPL570	GSE32924 Samples	Nonlesional atopic dermatitis skin is characterized by broad terminal differentiation defects and variable immune abnormalities.	J Allergy Clin Immunol.
atopic dermatitis	PLEKHG1	Expression profiling by array, count, 3 development stage, 2 disease state, 11 individual sets	GPL570	GSE32924 Samples	Nonlesional atopic dermatitis skin is characterized by broad terminal differentiation defects and variable immune abnormalities.	J Allergy Clin Immunol.
atopic dermatitis	ZMYM6	Expression profiling by array, count, 3 development stage, 2 disease state, 11 individual sets	GPL570	GSE32924 Samples	Nonlesional atopic dermatitis skin is characterized by broad terminal differentiation defects and variable immune abnormalities.	J Allergy Clin Immunol.
atopic dermatitis	JAK2	Expression profiling by array, count, 3 development stage, 2 disease state, 11 individual sets	GPL570	GSE32924 Samples	Nonlesional atopic dermatitis skin is characterized by broad terminal differentiation defects and variable immune abnormalities.	J Allergy Clin Immunol.
atopic dermatitis	CAMK2D	Expression profiling by array, count, 3 development stage, 2 disease state, 11 individual sets	GPL570	GSE32924 Samples	Nonlesional atopic dermatitis skin is characterized by broad terminal differentiation defects and variable immune abnormalities.	J Allergy Clin Immunol.
atopic dermatitis	CREBL2	Expression profiling by array, count, 3 development stage, 2 disease state, 11 individual sets	GPL570	GSE32924 Samples	Nonlesional atopic dermatitis skin is characterized by broad terminal differentiation defects and variable immune abnormalities.	J Allergy Clin Immunol.
atopic dermatitis	PAFAH1B1	Expression profiling by array, count, 3 development stage, 2 disease state, 11 individual sets	GPL570	GSE32924 Samples	Nonlesional atopic dermatitis skin is characterized by broad terminal differentiation defects and variable immune abnormalities.	J Allergy Clin Immunol.
atopic dermatitis	ARFGEF1	Expression profiling by array, count, 3 development stage, 2 disease state, 11 individual sets	GPL570	GSE32924 Samples	Nonlesional atopic dermatitis skin is characterized by broad terminal differentiation defects and variable immune abnormalities.	J Allergy Clin Immunol.
atopic dermatitis	PTPLAD1	Expression profiling by array, count, 3 development stage, 2 disease state, 11 individual sets	GPL570	GSE32924 Samples	Nonlesional atopic dermatitis skin is characterized by broad terminal differentiation defects and variable immune abnormalities.	J Allergy Clin Immunol.
atopic dermatitis	FAM115A	Expression profiling by array, count, 3 development stage, 2 disease state, 11 individual sets	GPL570	GSE32924 Samples	Nonlesional atopic dermatitis skin is characterized by broad terminal differentiation defects and variable immune abnormalities.	J Allergy Clin Immunol.

atopic dermatitis	FGFR2	Expression profiling by array, count, 3 development stage, 2 disease state, 11 individual sets	GPL570	GSE32924 Samples	Nonlesional atopic dermatitis skin is characterized by broad terminal differentiation defects and variable immune abnormalities.	J Allergy Clin Immunol.
atopic dermatitis	PALLD	Expression profiling by array, count, 3 development stage, 2 disease state, 11 individual sets	GPL570	GSE32924 Samples	Nonlesional atopic dermatitis skin is characterized by broad terminal differentiation defects and variable immune abnormalities.	J Allergy Clin Immunol.
atopic dermatitis	GNPDA2	Expression profiling by array, count, 3 development stage, 2 disease state, 11 individual sets	GPL570	GSE32924 Samples	Nonlesional atopic dermatitis skin is characterized by broad terminal differentiation defects and variable immune abnormalities.	J Allergy Clin Immunol.
atopic dermatitis	WAC	Expression profiling by array, count, 3 development stage, 2 disease state, 11 individual sets	GPL570	GSE32924 Samples	Nonlesional atopic dermatitis skin is characterized by broad terminal differentiation defects and variable immune abnormalities.	J Allergy Clin Immunol.
atopic dermatitis	MAP3K7IP2	Expression profiling by array, count, 3 development stage, 2 disease state, 11 individual sets	GPL570	GSE32924 Samples	Nonlesional atopic dermatitis skin is characterized by broad terminal differentiation defects and variable immune abnormalities.	J Allergy Clin Immunol.
atopic dermatitis	BCOR	Expression profiling by array, count, 3 development stage, 2 disease state, 11 individual sets	GPL570	GSE32924 Samples	Nonlesional atopic dermatitis skin is characterized by broad terminal differentiation defects and variable immune abnormalities.	J Allergy Clin Immunol.
atopic dermatitis	RANBP9	Expression profiling by array, count, 3 development stage, 2 disease state, 11 individual sets	GPL570	GSE32924 Samples	Nonlesional atopic dermatitis skin is characterized by broad terminal differentiation defects and variable immune abnormalities.	J Allergy Clin Immunol.
atopic dermatitis	EPB41L3	Expression profiling by array, count, 3 development stage, 2 disease state, 11 individual sets	GPL570	GSE32924 Samples	Nonlesional atopic dermatitis skin is characterized by broad terminal differentiation defects and variable immune abnormalities.	J Allergy Clin Immunol.
atopic dermatitis	NA	Expression profiling by array, count, 3 development stage, 2 disease state, 11 individual sets	GPL570	GSE32924 Samples	Nonlesional atopic dermatitis skin is characterized by broad terminal differentiation defects and variable immune abnormalities.	J Allergy Clin Immunol.
atopic dermatitis	TSPAN5	Expression profiling by array, count, 3 development stage, 2 disease state, 11 individual sets	GPL570	GSE32924 Samples	Nonlesional atopic dermatitis skin is characterized by broad terminal differentiation defects and variable immune abnormalities.	J Allergy Clin Immunol.
atopic dermatitis	WAPAL	Expression profiling by array, count, 3 development stage, 2 disease state, 11 individual sets	GPL570	GSE32924 Samples	Nonlesional atopic dermatitis skin is characterized by broad terminal differentiation defects and variable immune abnormalities.	J Allergy Clin Immunol.
atopic dermatitis	C3ORF38	Expression profiling by array, count, 3 development stage, 2 disease state, 11 individual sets	GPL570	GSE32924 Samples	Nonlesional atopic dermatitis skin is characterized by broad terminal differentiation defects and variable immune abnormalities.	J Allergy Clin Immunol.
atopic dermatitis	MTHFD2L	Expression profiling by array, count, 3 development stage, 2 disease state, 11 individual sets	GPL570	GSE32924 Samples	Nonlesional atopic dermatitis skin is characterized by broad terminal differentiation defects and variable immune abnormalities.	J Allergy Clin Immunol.
atopic dermatitis	CSNK2A1	Expression profiling by array, count, 3 development stage, 2 disease state, 11 individual sets	GPL570	GSE32924 Samples	Nonlesional atopic dermatitis skin is characterized by broad terminal differentiation defects and variable immune abnormalities.	J Allergy Clin Immunol.
atopic dermatitis	ATG5	Expression profiling by array, count, 3 development stage, 2 disease state, 11 individual sets	GPL570	GSE32924 Samples	Nonlesional atopic dermatitis skin is characterized by broad terminal differentiation defects and variable immune abnormalities.	J Allergy Clin Immunol.
atopic dermatitis	SLC39A2	Expression profiling by array, count, 3 development stage, 2 disease state, 11 individual sets	GPL570	GSE32924 Samples	Nonlesional atopic dermatitis skin is characterized by broad terminal differentiation defects and variable immune abnormalities.	J Allergy Clin Immunol.
atopic dermatitis	GAS7	Expression profiling by array, count, 3 development stage, 2 disease state, 11 individual sets	GPL570	GSE32924 Samples	Nonlesional atopic dermatitis skin is characterized by broad terminal differentiation defects and variable immune abnormalities.	J Allergy Clin Immunol.
atopic dermatitis	NA	Expression profiling by array, count, 3 development stage, 2 disease state, 11 individual sets	GPL570	GSE32924 Samples	Nonlesional atopic dermatitis skin is characterized by broad terminal differentiation defects and variable immune abnormalities.	J Allergy Clin Immunol.
atopic dermatitis	NA	Expression profiling by array, count, 3 development stage, 2 disease state, 11 individual sets	GPL570	GSE32924 Samples	Nonlesional atopic dermatitis skin is characterized by broad terminal differentiation defects and variable immune abnormalities.	J Allergy Clin Immunol.
atopic dermatitis	CNTN1	Expression profiling by array, count, 3 development stage, 2 disease state, 11 individual sets	GPL570	GSE32924 Samples	Nonlesional atopic dermatitis skin is characterized by broad terminal differentiation defects and variable immune abnormalities.	J Allergy Clin Immunol.
atopic dermatitis	ABHD6	Expression profiling by array, count, 3 development stage, 2 disease state, 11 individual sets	GPL570	GSE32924 Samples	Nonlesional atopic dermatitis skin is characterized by broad terminal differentiation defects and variable immune abnormalities.	J Allergy Clin Immunol.
atopic dermatitis	C10ORF57	Expression profiling by array, count, 3 development stage, 2 disease state, 11 individual sets	GPL570	GSE32924 Samples	Nonlesional atopic dermatitis skin is characterized by broad terminal differentiation defects and variable immune abnormalities.	J Allergy Clin Immunol.
atopic dermatitis	RIT1	Expression profiling by array, count, 3 development stage, 2 disease state, 11 individual sets	GPL570	GSE32924 Samples	Nonlesional atopic dermatitis skin is characterized by broad terminal differentiation defects and variable immune abnormalities.	J Allergy Clin Immunol.
atopic dermatitis	GRHL1	Expression profiling by array, count, 3 development stage, 2 disease state, 11 individual sets	GPL570	GSE32924 Samples	Nonlesional atopic dermatitis skin is characterized by broad terminal differentiation defects and variable immune abnormalities.	J Allergy Clin Immunol.
atopic dermatitis	MUC15	Expression profiling by array, count, 3 development stage, 2 disease state, 11 individual sets	GPL570	GSE32924 Samples	Nonlesional atopic dermatitis skin is characterized by broad terminal differentiation defects and variable immune abnormalities.	J Allergy Clin Immunol.
atopic dermatitis	OTUB2	Expression profiling by array, count, 3 development stage, 2 disease state, 11 individual sets	GPL570	GSE32924 Samples	Nonlesional atopic dermatitis skin is characterized by broad terminal differentiation defects and variable immune abnormalities.	J Allergy Clin Immunol.
atopic dermatitis	NA	Expression profiling by array, count, 3 development stage, 2 disease state, 11 individual sets	GPL570	GSE32924 Samples	Nonlesional atopic dermatitis skin is characterized by broad terminal differentiation defects and variable immune abnormalities.	J Allergy Clin Immunol.
atopic dermatitis	SPRR4	Expression profiling by array, count, 3 development stage, 2 disease state, 11 individual sets	GPL570	GSE32924 Samples	Nonlesional atopic dermatitis skin is characterized by broad terminal differentiation defects and variable immune abnormalities.	J Allergy Clin Immunol.
atopic dermatitis	NA	Expression profiling by array, count, 3 development stage, 2 disease state, 11 individual sets	GPL570	GSE32924 Samples	Nonlesional atopic dermatitis skin is characterized by broad terminal differentiation defects and variable immune abnormalities.	J Allergy Clin Immunol.
atopic dermatitis	GPLD1	Expression profiling by array, count, 3 development stage, 2 disease state, 11 individual sets	GPL570	GSE32924 Samples	Nonlesional atopic dermatitis skin is characterized by broad terminal differentiation defects and variable immune abnormalities.	J Allergy Clin Immunol.
atopic dermatitis	TRAM1L1	Expression profiling by array, count, 3 development stage, 2 disease state, 11 individual sets	GPL570	GSE32924 Samples	Nonlesional atopic dermatitis skin is characterized by broad terminal differentiation defects and variable immune abnormalities.	J Allergy Clin Immunol.
atopic dermatitis	TUB	Expression profiling by array, count, 3 development stage, 2 disease state, 11 individual sets	GPL570	GSE32924 Samples	Nonlesional atopic dermatitis skin is characterized by broad terminal differentiation defects and variable immune abnormalities.	J Allergy Clin Immunol.
atopic dermatitis	NA	Expression profiling by array, count, 3 development stage, 2 disease state, 11 individual sets	GPL570	GSE32924 Samples	Nonlesional atopic dermatitis skin is characterized by broad terminal differentiation defects and variable immune abnormalities.	J Allergy Clin Immunol.
atopic dermatitis	SMARCA1	Expression profiling by array, count, 3 development stage, 2 disease state, 11 individual sets	GPL570	GSE32924 Samples	Nonlesional atopic dermatitis skin is characterized by broad terminal differentiation defects and variable immune abnormalities.	J Allergy Clin Immunol.
atopic dermatitis	RTN4	Expression profiling by array, count, 3 development stage, 2 disease state, 11 individual sets	GPL570	GSE32924 Samples	Nonlesional atopic dermatitis skin is characterized by broad terminal differentiation defects and variable immune abnormalities.	J Allergy Clin Immunol.
atopic dermatitis	NPIPL3	Expression profiling by array, count, 3 development stage, 2 disease state, 11 individual sets	GPL570	GSE32924 Samples	Nonlesional atopic dermatitis skin is characterized by broad terminal differentiation defects and variable immune abnormalities.	J Allergy Clin Immunol.
atopic dermatitis	C15ORF59	Expression profiling by array, count, 3 development stage, 2 disease state, 11 individual sets	GPL570	GSE32924 Samples	Nonlesional atopic dermatitis skin is characterized by broad terminal differentiation defects and variable immune abnormalities.	J Allergy Clin Immunol.
atopic dermatitis	TIMP3	Expression profiling by array, count, 3 development stage, 2 disease state, 11 individual sets	GPL570	GSE32924 Samples	Nonlesional atopic dermatitis skin is characterized by broad terminal differentiation defects and variable immune abnormalities.	J Allergy Clin Immunol.
atopic dermatitis	NEGR1	Expression profiling by array, count, 3 development stage, 2 disease state, 11 individual sets	GPL570	GSE32924 Samples	Nonlesional atopic dermatitis skin is characterized by broad terminal differentiation defects and variable immune abnormalities.	J Allergy Clin Immunol.
atopic dermatitis	EPB41L2	Expression profiling by array, count, 3 development stage, 2 disease state, 11 individual sets	GPL570	GSE32924 Samples	Nonlesional atopic dermatitis skin is characterized by broad terminal differentiation defects and variable immune abnormalities.	J Allergy Clin Immunol.
atopic dermatitis	AGTR1	Expression profiling by array, count, 3 development stage, 2 disease state, 11 individual sets	GPL570	GSE32924 Samples	Nonlesional atopic dermatitis skin is characterized by broad terminal differentiation defects and variable immune abnormalities.	J Allergy Clin Immunol.

atopic dermatitis	MMP27	Expression profiling by array, count, 3 development stage, 2 disease state, 11 individual sets	GPL570	GSE32924 Samples	Nonlesional atopic dermatitis skin is characterized by broad terminal differentiation defects and variable immune abnormalities.	J Allergy Clin Immunol.
atopic dermatitis	OMD	Expression profiling by array, count, 3 development stage, 2 disease state, 11 individual sets	GPL570	GSE32924 Samples	Nonlesional atopic dermatitis skin is characterized by broad terminal differentiation defects and variable immune abnormalities.	J Allergy Clin Immunol.
atopic dermatitis	LOX	Expression profiling by array, count, 3 development stage, 2 disease state, 11 individual sets	GPL570	GSE32924 Samples	Nonlesional atopic dermatitis skin is characterized by broad terminal differentiation defects and variable immune abnormalities.	J Allergy Clin Immunol.
atopic dermatitis	CCNG2	Expression profiling by array, count, 3 development stage, 2 disease state, 11 individual sets	GPL570	GSE32924 Samples	Nonlesional atopic dermatitis skin is characterized by broad terminal differentiation defects and variable immune abnormalities.	J Allergy Clin Immunol.
atopic dermatitis	ARL4C	Expression profiling by array, count, 3 development stage, 2 disease state, 11 individual sets	GPL570	GSE32924 Samples	Nonlesional atopic dermatitis skin is characterized by broad terminal differentiation defects and variable immune abnormalities.	J Allergy Clin Immunol.
atopic dermatitis	TIMP2	Expression profiling by array, count, 3 development stage, 2 disease state, 11 individual sets	GPL570	GSE32924 Samples	Nonlesional atopic dermatitis skin is characterized by broad terminal differentiation defects and variable immune abnormalities.	J Allergy Clin Immunol.
atopic dermatitis	TMEM200A	Expression profiling by array, count, 3 development stage, 2 disease state, 11 individual sets	GPL570	GSE32924 Samples	Nonlesional atopic dermatitis skin is characterized by broad terminal differentiation defects and variable immune abnormalities.	J Allergy Clin Immunol.
atopic dermatitis	PRKCB	Expression profiling by array, count, 3 development stage, 2 disease state, 11 individual sets	GPL570	GSE32924 Samples	Nonlesional atopic dermatitis skin is characterized by broad terminal differentiation defects and variable immune abnormalities.	J Allergy Clin Immunol.
atopic dermatitis	NA	Expression profiling by array, count, 3 development stage, 2 disease state, 11 individual sets	GPL570	GSE32924 Samples	Nonlesional atopic dermatitis skin is characterized by broad terminal differentiation defects and variable immune abnormalities.	J Allergy Clin Immunol.
atopic dermatitis	CMAH	Expression profiling by array, count, 3 development stage, 2 disease state, 11 individual sets	GPL570	GSE32924 Samples	Nonlesional atopic dermatitis skin is characterized by broad terminal differentiation defects and variable immune abnormalities.	J Allergy Clin Immunol.
atopic dermatitis	CCDC80	Expression profiling by array, count, 3 development stage, 2 disease state, 11 individual sets	GPL570	GSE32924 Samples	Nonlesional atopic dermatitis skin is characterized by broad terminal differentiation defects and variable immune abnormalities.	J Allergy Clin Immunol.
atopic dermatitis	OMD	Expression profiling by array, count, 3 development stage, 2 disease state, 11 individual sets	GPL570	GSE32924 Samples	Nonlesional atopic dermatitis skin is characterized by broad terminal differentiation defects and variable immune abnormalities.	J Allergy Clin Immunol.
atopic dermatitis	IGSF10	Expression profiling by array, count, 3 development stage, 2 disease state, 11 individual sets	GPL570	GSE32924 Samples	Nonlesional atopic dermatitis skin is characterized by broad terminal differentiation defects and variable immune abnormalities.	J Allergy Clin Immunol.
atopic dermatitis	ANGPTL1	Expression profiling by array, count, 3 development stage, 2 disease state, 11 individual sets	GPL570	GSE32924 Samples	Nonlesional atopic dermatitis skin is characterized by broad terminal differentiation defects and variable immune abnormalities.	J Allergy Clin Immunol.
atopic dermatitis	CP	Expression profiling by array, count, 3 development stage, 2 disease state, 11 individual sets	GPL570	GSE32924 Samples	Nonlesional atopic dermatitis skin is characterized by broad terminal differentiation defects and variable immune abnormalities.	J Allergy Clin Immunol.
atopic dermatitis	SLC25A13	Expression profiling by array, count, 3 development stage, 2 disease state, 11 individual sets	GPL570	GSE32924 Samples	Nonlesional atopic dermatitis skin is characterized by broad terminal differentiation defects and variable immune abnormalities.	J Allergy Clin Immunol.
atopic dermatitis	SETD5	Expression profiling by array, count, 3 development stage, 2 disease state, 11 individual sets	GPL570	GSE32924 Samples	Nonlesional atopic dermatitis skin is characterized by broad terminal differentiation defects and variable immune abnormalities.	J Allergy Clin Immunol.
atopic dermatitis	SFRS4	Expression profiling by array, count, 3 development stage, 2 disease state, 11 individual sets	GPL570	GSE32924 Samples	Nonlesional atopic dermatitis skin is characterized by broad terminal differentiation defects and variable immune abnormalities.	J Allergy Clin Immunol.
atopic dermatitis	PVRL4	Expression profiling by array, count, 3 development stage, 2 disease state, 11 individual sets	GPL570	GSE32924 Samples	Nonlesional atopic dermatitis skin is characterized by broad terminal differentiation defects and variable immune abnormalities.	J Allergy Clin Immunol.
atopic dermatitis	FBXW7	Expression profiling by array, count, 3 development stage, 2 disease state, 11 individual sets	GPL570	GSE32924 Samples	Nonlesional atopic dermatitis skin is characterized by broad terminal differentiation defects and variable immune abnormalities.	J Allergy Clin Immunol.
atopic dermatitis	MCOLN3	Expression profiling by array, count, 3 development stage, 2 disease state, 11 individual sets	GPL570	GSE32924 Samples	Nonlesional atopic dermatitis skin is characterized by broad terminal differentiation defects and variable immune abnormalities.	J Allergy Clin Immunol.
atopic dermatitis	AGPAT9	Expression profiling by array, count, 3 development stage, 2 disease state, 11 individual sets	GPL570	GSE32924 Samples	Nonlesional atopic dermatitis skin is characterized by broad terminal differentiation defects and variable immune abnormalities.	J Allergy Clin Immunol.
atopic dermatitis	CX3CR1	Expression profiling by array, count, 3 development stage, 2 disease state, 11 individual sets	GPL570	GSE32924 Samples	Nonlesional atopic dermatitis skin is characterized by broad terminal differentiation defects and variable immune abnormalities.	J Allergy Clin Immunol.
atopic dermatitis	CYP39A1	Expression profiling by array, count, 3 development stage, 2 disease state, 11 individual sets	GPL570	GSE32924 Samples	Nonlesional atopic dermatitis skin is characterized by broad terminal differentiation defects and variable immune abnormalities.	J Allergy Clin Immunol.
atopic dermatitis	NA	Expression profiling by array, count, 3 development stage, 2 disease state, 11 individual sets	GPL570	GSE32924 Samples	Nonlesional atopic dermatitis skin is characterized by broad terminal differentiation defects and variable immune abnormalities.	J Allergy Clin Immunol.
atopic dermatitis	IL7	Expression profiling by array, count, 3 development stage, 2 disease state, 11 individual sets	GPL570	GSE32924 Samples	Nonlesional atopic dermatitis skin is characterized by broad terminal differentiation defects and variable immune abnormalities.	J Allergy Clin Immunol.
atopic dermatitis	PRKCB	Expression profiling by array, count, 3 development stage, 2 disease state, 11 individual sets	GPL570	GSE32924 Samples	Nonlesional atopic dermatitis skin is characterized by broad terminal differentiation defects and variable immune abnormalities.	J Allergy Clin Immunol.
atopic dermatitis	OTUD1	Expression profiling by array, count, 3 development stage, 2 disease state, 11 individual sets	GPL570	GSE32924 Samples	Nonlesional atopic dermatitis skin is characterized by broad terminal differentiation defects and variable immune abnormalities.	J Allergy Clin Immunol.
atopic dermatitis	SCIN	Expression profiling by array, count, 3 development stage, 2 disease state, 11 individual sets	GPL570	GSE32924 Samples	Nonlesional atopic dermatitis skin is characterized by broad terminal differentiation defects and variable immune abnormalities.	J Allergy Clin Immunol.
atopic dermatitis	FLRT3	Expression profiling by array, count, 3 development stage, 2 disease state, 11 individual sets	GPL570	GSE32924 Samples	Nonlesional atopic dermatitis skin is characterized by broad terminal differentiation defects and variable immune abnormalities.	J Allergy Clin Immunol.
atopic dermatitis	SLC16A10	Expression profiling by array, count, 3 development stage, 2 disease state, 11 individual sets	GPL570	GSE32924 Samples	Nonlesional atopic dermatitis skin is characterized by broad terminal differentiation defects and variable immune abnormalities.	J Allergy Clin Immunol.
atopic dermatitis	PRRX1	Expression profiling by array, count, 3 development stage, 2 disease state, 11 individual sets	GPL570	GSE32924 Samples	Nonlesional atopic dermatitis skin is characterized by broad terminal differentiation defects and variable immune abnormalities.	J Allergy Clin Immunol.
atopic dermatitis	PICALM	Expression profiling by array, count, 3 development stage, 2 disease state, 11 individual sets	GPL570	GSE32924 Samples	Nonlesional atopic dermatitis skin is characterized by broad terminal differentiation defects and variable immune abnormalities.	J Allergy Clin Immunol.
atopic dermatitis	STS	Expression profiling by array, count, 3 development stage, 2 disease state, 11 individual sets	GPL570	GSE32924 Samples	Nonlesional atopic dermatitis skin is characterized by broad terminal differentiation defects and variable immune abnormalities.	J Allergy Clin Immunol.
atopic dermatitis	FBN1	Expression profiling by array, count, 3 development stage, 2 disease state, 11 individual sets	GPL570	GSE32924 Samples	Nonlesional atopic dermatitis skin is characterized by broad terminal differentiation defects and variable immune abnormalities.	J Allergy Clin Immunol.
atopic dermatitis	C1S	Expression profiling by array, count, 3 development stage, 2 disease state, 11 individual sets	GPL570	GSE32924 Samples	Nonlesional atopic dermatitis skin is characterized by broad terminal differentiation defects and variable immune abnormalities.	J Allergy Clin Immunol.
atopic dermatitis	PDGFRL	Expression profiling by array, count, 3 development stage, 2 disease state, 11 individual sets	GPL570	GSE32924 Samples	Nonlesional atopic dermatitis skin is characterized by broad terminal differentiation defects and variable immune abnormalities.	J Allergy Clin Immunol.
atopic dermatitis	IL1F7	Expression profiling by array, count, 3 development stage, 2 disease state, 11 individual sets	GPL570	GSE32924 Samples	Nonlesional atopic dermatitis skin is characterized by broad terminal differentiation defects and variable immune abnormalities.	J Allergy Clin Immunol.
atopic dermatitis	PSORS1C2	Expression profiling by array, count, 3 development stage, 2 disease state, 11 individual sets	GPL570	GSE32924 Samples	Nonlesional atopic dermatitis skin is characterized by broad terminal differentiation defects and variable immune abnormalities.	J Allergy Clin Immunol.
atopic dermatitis	LOC100130476	Expression profiling by array, count, 3 development stage, 2 disease state, 11 individual sets	GPL570	GSE32924 Samples	Nonlesional atopic dermatitis skin is characterized by broad terminal differentiation defects and variable immune abnormalities.	J Allergy Clin Immunol.
atopic dermatitis	GREM1	Expression profiling by array, count, 3 development stage, 2 disease state, 11 individual sets	GPL570	GSE32924 Samples	Nonlesional atopic dermatitis skin is characterized by broad terminal differentiation defects and variable immune abnormalities.	J Allergy Clin Immunol.

atopic dermatitis	CORIN	Expression profiling by array, count, 3 development stage, 2 disease state, 11 individual sets	GPL570	GSE32924 Samples	Nonlesional atopic dermatitis skin is characterized by broad terminal differentiation defects and variable immune abnormalities.	J Allergy Clin Immunol.
atopic dermatitis	C17ORF48	Expression profiling by array, count, 3 development stage, 2 disease state, 11 individual sets	GPL570	GSE32924 Samples	Nonlesional atopic dermatitis skin is characterized by broad terminal differentiation defects and variable immune abnormalities.	J Allergy Clin Immunol.
atopic dermatitis	ZNF134	Expression profiling by array, count, 3 development stage, 2 disease state, 11 individual sets	GPL570	GSE32924 Samples	Nonlesional atopic dermatitis skin is characterized by broad terminal differentiation defects and variable immune abnormalities.	J Allergy Clin Immunol.
atopic dermatitis	LPAR1	Expression profiling by array, count, 3 development stage, 2 disease state, 11 individual sets	GPL570	GSE32924 Samples	Nonlesional atopic dermatitis skin is characterized by broad terminal differentiation defects and variable immune abnormalities.	J Allergy Clin Immunol.
atopic dermatitis	HSF2	Expression profiling by array, count, 3 development stage, 2 disease state, 11 individual sets	GPL570	GSE32924 Samples	Nonlesional atopic dermatitis skin is characterized by broad terminal differentiation defects and variable immune abnormalities.	J Allergy Clin Immunol.
atopic dermatitis	HDGFRP3	Expression profiling by array, count, 3 development stage, 2 disease state, 11 individual sets	GPL570	GSE32924 Samples	Nonlesional atopic dermatitis skin is characterized by broad terminal differentiation defects and variable immune abnormalities.	J Allergy Clin Immunol.
atopic dermatitis	GLT8D2	Expression profiling by array, count, 3 development stage, 2 disease state, 11 individual sets	GPL570	GSE32924 Samples	Nonlesional atopic dermatitis skin is characterized by broad terminal differentiation defects and variable immune abnormalities.	J Allergy Clin Immunol.
atopic dermatitis	LPIN2	Expression profiling by array, count, 3 development stage, 2 disease state, 11 individual sets	GPL570	GSE32924 Samples	Nonlesional atopic dermatitis skin is characterized by broad terminal differentiation defects and variable immune abnormalities.	J Allergy Clin Immunol.
atopic dermatitis	IGFBP5	Expression profiling by array, count, 3 development stage, 2 disease state, 11 individual sets	GPL570	GSE32924 Samples	Nonlesional atopic dermatitis skin is characterized by broad terminal differentiation defects and variable immune abnormalities.	J Allergy Clin Immunol.
atopic dermatitis	FAM38B	Expression profiling by array, count, 3 development stage, 2 disease state, 11 individual sets	GPL570	GSE32924 Samples	Nonlesional atopic dermatitis skin is characterized by broad terminal differentiation defects and variable immune abnormalities.	J Allergy Clin Immunol.
atopic dermatitis	NA	Expression profiling by array, count, 3 development stage, 2 disease state, 11 individual sets	GPL570	GSE32924 Samples	Nonlesional atopic dermatitis skin is characterized by broad terminal differentiation defects and variable immune abnormalities.	J Allergy Clin Immunol.
atopic dermatitis	IGF2R	Expression profiling by array, count, 3 development stage, 2 disease state, 11 individual sets	GPL570	GSE32924 Samples	Nonlesional atopic dermatitis skin is characterized by broad terminal differentiation defects and variable immune abnormalities.	J Allergy Clin Immunol.
atopic dermatitis	MAX	Expression profiling by array, count, 3 development stage, 2 disease state, 11 individual sets	GPL570	GSE32924 Samples	Nonlesional atopic dermatitis skin is characterized by broad terminal differentiation defects and variable immune abnormalities.	J Allergy Clin Immunol.
atopic dermatitis	NA	Expression profiling by array, count, 3 development stage, 2 disease state, 11 individual sets	GPL570	GSE32924 Samples	Nonlesional atopic dermatitis skin is characterized by broad terminal differentiation defects and variable immune abnormalities.	J Allergy Clin Immunol.
atopic dermatitis	MCOLN3	Expression profiling by array, count, 3 development stage, 2 disease state, 11 individual sets	GPL570	GSE32924 Samples	Nonlesional atopic dermatitis skin is characterized by broad terminal differentiation defects and variable immune abnormalities.	J Allergy Clin Immunol.
atopic dermatitis	NA	Expression profiling by array, count, 3 development stage, 2 disease state, 11 individual sets	GPL570	GSE32924 Samples	Nonlesional atopic dermatitis skin is characterized by broad terminal differentiation defects and variable immune abnormalities.	J Allergy Clin Immunol.
atopic dermatitis	SNAP23	Expression profiling by array, count, 3 development stage, 2 disease state, 11 individual sets	GPL570	GSE32924 Samples	Nonlesional atopic dermatitis skin is characterized by broad terminal differentiation defects and variable immune abnormalities.	J Allergy Clin Immunol.
atopic dermatitis	FLI1	Expression profiling by array, count, 3 development stage, 2 disease state, 11 individual sets	GPL570	GSE32924 Samples	Nonlesional atopic dermatitis skin is characterized by broad terminal differentiation defects and variable immune abnormalities.	J Allergy Clin Immunol.
atopic dermatitis	ANP32A	Expression profiling by array, count, 3 development stage, 2 disease state, 11 individual sets	GPL570	GSE32924 Samples	Nonlesional atopic dermatitis skin is characterized by broad terminal differentiation defects and variable immune abnormalities.	J Allergy Clin Immunol.
atopic dermatitis	KLHL20	Expression profiling by array, count, 3 development stage, 2 disease state, 11 individual sets	GPL570	GSE32924 Samples	Nonlesional atopic dermatitis skin is characterized by broad terminal differentiation defects and variable immune abnormalities.	J Allergy Clin Immunol.
atopic dermatitis	ANKFY1	Expression profiling by array, count, 3 development stage, 2 disease state, 11 individual sets	GPL570	GSE32924 Samples	Nonlesional atopic dermatitis skin is characterized by broad terminal differentiation defects and variable immune abnormalities.	J Allergy Clin Immunol.
atopic dermatitis	XPO7	Expression profiling by array, count, 3 development stage, 2 disease state, 11 individual sets	GPL570	GSE32924 Samples	Nonlesional atopic dermatitis skin is characterized by broad terminal differentiation defects and variable immune abnormalities.	J Allergy Clin Immunol.
atopic dermatitis	NA	Expression profiling by array, count, 3 development stage, 2 disease state, 11 individual sets	GPL570	GSE32924 Samples	Nonlesional atopic dermatitis skin is characterized by broad terminal differentiation defects and variable immune abnormalities.	J Allergy Clin Immunol.
atopic dermatitis	FAM115A	Expression profiling by array, count, 3 development stage, 2 disease state, 11 individual sets	GPL570	GSE32924 Samples	Nonlesional atopic dermatitis skin is characterized by broad terminal differentiation defects and variable immune abnormalities.	J Allergy Clin Immunol.
atopic dermatitis	DCAF7	Expression profiling by array, count, 3 development stage, 2 disease state, 11 individual sets	GPL570	GSE32924 Samples	Nonlesional atopic dermatitis skin is characterized by broad terminal differentiation defects and variable immune abnormalities.	J Allergy Clin Immunol.
atopic dermatitis	KDELR3	Expression profiling by array, count, 3 development stage, 2 disease state, 11 individual sets	GPL570	GSE32924 Samples	Nonlesional atopic dermatitis skin is characterized by broad terminal differentiation defects and variable immune abnormalities.	J Allergy Clin Immunol.
atopic dermatitis	KLF7	Expression profiling by array, count, 3 development stage, 2 disease state, 11 individual sets	GPL570	GSE32924 Samples	Nonlesional atopic dermatitis skin is characterized by broad terminal differentiation defects and variable immune abnormalities.	J Allergy Clin Immunol.
atopic dermatitis	ERG	Expression profiling by array, count, 3 development stage, 2 disease state, 11 individual sets	GPL570	GSE32924 Samples	Nonlesional atopic dermatitis skin is characterized by broad terminal differentiation defects and variable immune abnormalities.	J Allergy Clin Immunol.
atopic dermatitis	NA	Expression profiling by array, count, 3 development stage, 2 disease state, 11 individual sets	GPL570	GSE32924 Samples	Nonlesional atopic dermatitis skin is characterized by broad terminal differentiation defects and variable immune abnormalities.	J Allergy Clin Immunol.
atopic dermatitis	LPCAT2	Expression profiling by array, count, 3 development stage, 2 disease state, 11 individual sets	GPL570	GSE32924 Samples	Nonlesional atopic dermatitis skin is characterized by broad terminal differentiation defects and variable immune abnormalities.	J Allergy Clin Immunol.
atopic dermatitis	C3ORF38	Expression profiling by array, count, 3 development stage, 2 disease state, 11 individual sets	GPL570	GSE32924 Samples	Nonlesional atopic dermatitis skin is characterized by broad terminal differentiation defects and variable immune abnormalities.	J Allergy Clin Immunol.
atopic dermatitis	NA	Expression profiling by array, count, 3 development stage, 2 disease state, 11 individual sets	GPL570	GSE32924 Samples	Nonlesional atopic dermatitis skin is characterized by broad terminal differentiation defects and variable immune abnormalities.	J Allergy Clin Immunol.
atopic dermatitis	PRSS12	Expression profiling by array, count, 3 development stage, 2 disease state, 11 individual sets	GPL570	GSE32924 Samples	Nonlesional atopic dermatitis skin is characterized by broad terminal differentiation defects and variable immune abnormalities.	J Allergy Clin Immunol.
atopic dermatitis	GAN	Expression profiling by array, count, 3 development stage, 2 disease state, 11 individual sets	GPL570	GSE32924 Samples	Nonlesional atopic dermatitis skin is characterized by broad terminal differentiation defects and variable immune abnormalities.	J Allergy Clin Immunol.
atopic dermatitis	ESYT3	Expression profiling by array, count, 3 development stage, 2 disease state, 11 individual sets	GPL570	GSE32924 Samples	Nonlesional atopic dermatitis skin is characterized by broad terminal differentiation defects and variable immune abnormalities.	J Allergy Clin Immunol.
atopic dermatitis	PTPN21	Expression profiling by array, count, 3 development stage, 2 disease state, 11 individual sets	GPL570	GSE32924 Samples	Nonlesional atopic dermatitis skin is characterized by broad terminal differentiation defects and variable immune abnormalities.	J Allergy Clin Immunol.
atopic dermatitis	NA	Expression profiling by array, count, 3 development stage, 2 disease state, 11 individual sets	GPL570	GSE32924 Samples	Nonlesional atopic dermatitis skin is characterized by broad terminal differentiation defects and variable immune abnormalities.	J Allergy Clin Immunol.
atopic dermatitis	EFHC2	Expression profiling by array, count, 3 development stage, 2 disease state, 11 individual sets	GPL570	GSE32924 Samples	Nonlesional atopic dermatitis skin is characterized by broad terminal differentiation defects and variable immune abnormalities.	J Allergy Clin Immunol.
atopic dermatitis	NA	Expression profiling by array, count, 3 development stage, 2 disease state, 11 individual sets	GPL570	GSE32924 Samples	Nonlesional atopic dermatitis skin is characterized by broad terminal differentiation defects and variable immune abnormalities.	J Allergy Clin Immunol.
atopic dermatitis	PLEKHA8	Expression profiling by array, count, 3 development stage, 2 disease state, 11 individual sets	GPL570	GSE32924 Samples	Nonlesional atopic dermatitis skin is characterized by broad terminal differentiation defects and variable immune abnormalities.	J Allergy Clin Immunol.
atopic dermatitis	NA	Expression profiling by array, count, 3 development stage, 2 disease state, 11 individual sets	GPL570	GSE32924 Samples	Nonlesional atopic dermatitis skin is characterized by broad terminal differentiation defects and variable immune abnormalities.	J Allergy Clin Immunol.

atopic dermatitis	CORIN	Expression profiling by array, count, 3 development stage, 2 disease state, 11 individual sets	GPL570	GSE32924 Samples	Nonlesional atopic dermatitis skin is characterized by broad terminal differentiation defects and variable immune abnormalities.	J Allergy Clin Immunol.
atopic dermatitis	NA	Expression profiling by array, count, 3 development stage, 2 disease state, 11 individual sets	GPL570	GSE32924 Samples	Nonlesional atopic dermatitis skin is characterized by broad terminal differentiation defects and variable immune abnormalities.	J Allergy Clin Immunol.
atopic dermatitis	ABCA9	Expression profiling by array, count, 3 development stage, 2 disease state, 11 individual sets	GPL570	GSE32924 Samples	Nonlesional atopic dermatitis skin is characterized by broad terminal differentiation defects and variable immune abnormalities.	J Allergy Clin Immunol.
atopic dermatitis	FLJ32063	Expression profiling by array, count, 3 development stage, 2 disease state, 11 individual sets	GPL570	GSE32924 Samples	Nonlesional atopic dermatitis skin is characterized by broad terminal differentiation defects and variable immune abnormalities.	J Allergy Clin Immunol.
atopic dermatitis	CNKSR2	Expression profiling by array, count, 3 development stage, 2 disease state, 11 individual sets	GPL570	GSE32924 Samples	Nonlesional atopic dermatitis skin is characterized by broad terminal differentiation defects and variable immune abnormalities.	J Allergy Clin Immunol.
atopic dermatitis	NA	Expression profiling by array, count, 3 development stage, 2 disease state, 11 individual sets	GPL570	GSE32924 Samples	Nonlesional atopic dermatitis skin is characterized by broad terminal differentiation defects and variable immune abnormalities.	J Allergy Clin Immunol.
atopic dermatitis	FNDC1	Expression profiling by array, count, 3 development stage, 2 disease state, 11 individual sets	GPL570	GSE32924 Samples	Nonlesional atopic dermatitis skin is characterized by broad terminal differentiation defects and variable immune abnormalities.	J Allergy Clin Immunol.
atopic dermatitis	MPDZ	Expression profiling by array, count, 3 development stage, 2 disease state, 11 individual sets	GPL570	GSE32924 Samples	Nonlesional atopic dermatitis skin is characterized by broad terminal differentiation defects and variable immune abnormalities.	J Allergy Clin Immunol.
atopic dermatitis	LSG1	Expression profiling by array, count, 3 development stage, 2 disease state, 11 individual sets	GPL570	GSE32924 Samples	Nonlesional atopic dermatitis skin is characterized by broad terminal differentiation defects and variable immune abnormalities.	J Allergy Clin Immunol.
atopic dermatitis	RBL2	Expression profiling by array, count, 3 development stage, 2 disease state, 11 individual sets	GPL570	GSE32924 Samples	Nonlesional atopic dermatitis skin is characterized by broad terminal differentiation defects and variable immune abnormalities.	J Allergy Clin Immunol.
atopic dermatitis	RIF1	Expression profiling by array, count, 3 development stage, 2 disease state, 11 individual sets	GPL570	GSE32924 Samples	Nonlesional atopic dermatitis skin is characterized by broad terminal differentiation defects and variable immune abnormalities.	J Allergy Clin Immunol.
atopic dermatitis	SGSM2	Expression profiling by array, count, 3 development stage, 2 disease state, 11 individual sets	GPL570	GSE32924 Samples	Nonlesional atopic dermatitis skin is characterized by broad terminal differentiation defects and variable immune abnormalities.	J Allergy Clin Immunol.
atopic dermatitis	DDX17	Expression profiling by array, count, 3 development stage, 2 disease state, 11 individual sets	GPL570	GSE32924 Samples	Nonlesional atopic dermatitis skin is characterized by broad terminal differentiation defects and variable immune abnormalities.	J Allergy Clin Immunol.
atopic dermatitis	CHD9	Expression profiling by array, count, 3 development stage, 2 disease state, 11 individual sets	GPL570	GSE32924 Samples	Nonlesional atopic dermatitis skin is characterized by broad terminal differentiation defects and variable immune abnormalities.	J Allergy Clin Immunol.
atopic dermatitis	NA	Expression profiling by array, count, 3 development stage, 2 disease state, 11 individual sets	GPL570	GSE32924 Samples	Nonlesional atopic dermatitis skin is characterized by broad terminal differentiation defects and variable immune abnormalities.	J Allergy Clin Immunol.
atopic dermatitis	MALAT1	Expression profiling by array, count, 3 development stage, 2 disease state, 11 individual sets	GPL570	GSE32924 Samples	Nonlesional atopic dermatitis skin is characterized by broad terminal differentiation defects and variable immune abnormalities.	J Allergy Clin Immunol.
atopic dermatitis	COCH	Expression profiling by array, count, 3 development stage, 2 disease state, 11 individual sets	GPL570	GSE32924 Samples	Nonlesional atopic dermatitis skin is characterized by broad terminal differentiation defects and variable immune abnormalities.	J Allergy Clin Immunol.
atopic dermatitis	ZNF513	Expression profiling by array, count, 3 development stage, 2 disease state, 11 individual sets	GPL570	GSE32924 Samples	Nonlesional atopic dermatitis skin is characterized by broad terminal differentiation defects and variable immune abnormalities.	J Allergy Clin Immunol.
atopic dermatitis	TMEM161A	Expression profiling by array, count, 3 development stage, 2 disease state, 11 individual sets	GPL570	GSE32924 Samples	Nonlesional atopic dermatitis skin is characterized by broad terminal differentiation defects and variable immune abnormalities.	J Allergy Clin Immunol.
atopic dermatitis	ZFYVE19	Expression profiling by array, count, 3 development stage, 2 disease state, 11 individual sets	GPL570	GSE32924 Samples	Nonlesional atopic dermatitis skin is characterized by broad terminal differentiation defects and variable immune abnormalities.	J Allergy Clin Immunol.
atopic dermatitis	IPO13	Expression profiling by array, count, 3 development stage, 2 disease state, 11 individual sets	GPL570	GSE32924 Samples	Nonlesional atopic dermatitis skin is characterized by broad terminal differentiation defects and variable immune abnormalities.	J Allergy Clin Immunol.
atopic dermatitis	GRK6	Expression profiling by array, count, 3 development stage, 2 disease state, 11 individual sets	GPL570	GSE32924 Samples	Nonlesional atopic dermatitis skin is characterized by broad terminal differentiation defects and variable immune abnormalities.	J Allergy Clin Immunol.
atopic dermatitis	VWA1	Expression profiling by array, count, 3 development stage, 2 disease state, 11 individual sets	GPL570	GSE32924 Samples	Nonlesional atopic dermatitis skin is characterized by broad terminal differentiation defects and variable immune abnormalities.	J Allergy Clin Immunol.
atopic dermatitis	CORO1B	Expression profiling by array, count, 3 development stage, 2 disease state, 11 individual sets	GPL570	GSE32924 Samples	Nonlesional atopic dermatitis skin is characterized by broad terminal differentiation defects and variable immune abnormalities.	J Allergy Clin Immunol.
atopic dermatitis	MON1A	Expression profiling by array, count, 3 development stage, 2 disease state, 11 individual sets	GPL570	GSE32924 Samples	Nonlesional atopic dermatitis skin is characterized by broad terminal differentiation defects and variable immune abnormalities.	J Allergy Clin Immunol.
atopic dermatitis	NDOR1	Expression profiling by array, count, 3 development stage, 2 disease state, 11 individual sets	GPL570	GSE32924 Samples	Nonlesional atopic dermatitis skin is characterized by broad terminal differentiation defects and variable immune abnormalities.	J Allergy Clin Immunol.
atopic dermatitis	RASSF7	Expression profiling by array, count, 3 development stage, 2 disease state, 11 individual sets	GPL570	GSE32924 Samples	Nonlesional atopic dermatitis skin is characterized by broad terminal differentiation defects and variable immune abnormalities.	J Allergy Clin Immunol.
atopic dermatitis	SLC37A4	Expression profiling by array, count, 3 development stage, 2 disease state, 11 individual sets	GPL570	GSE32924 Samples	Nonlesional atopic dermatitis skin is characterized by broad terminal differentiation defects and variable immune abnormalities.	J Allergy Clin Immunol.
atopic dermatitis	ENDOG	Expression profiling by array, count, 3 development stage, 2 disease state, 11 individual sets	GPL570	GSE32924 Samples	Nonlesional atopic dermatitis skin is characterized by broad terminal differentiation defects and variable immune abnormalities.	J Allergy Clin Immunol.
atopic dermatitis	PUSL1	Expression profiling by array, count, 3 development stage, 2 disease state, 11 individual sets	GPL570	GSE32924 Samples	Nonlesional atopic dermatitis skin is characterized by broad terminal differentiation defects and variable immune abnormalities.	J Allergy Clin Immunol.
atopic dermatitis	OGFOD2	Expression profiling by array, count, 3 development stage, 2 disease state, 11 individual sets	GPL570	GSE32924 Samples	Nonlesional atopic dermatitis skin is characterized by broad terminal differentiation defects and variable immune abnormalities.	J Allergy Clin Immunol.
atopic dermatitis	LYNX1	Expression profiling by array, count, 3 development stage, 2 disease state, 11 individual sets	GPL570	GSE32924 Samples	Nonlesional atopic dermatitis skin is characterized by broad terminal differentiation defects and variable immune abnormalities.	J Allergy Clin Immunol.
atopic dermatitis	MED16	Expression profiling by array, count, 3 development stage, 2 disease state, 11 individual sets	GPL570	GSE32924 Samples	Nonlesional atopic dermatitis skin is characterized by broad terminal differentiation defects and variable immune abnormalities.	J Allergy Clin Immunol.
atopic dermatitis	RAB4B	Expression profiling by array, count, 3 development stage, 2 disease state, 11 individual sets	GPL570	GSE32924 Samples	Nonlesional atopic dermatitis skin is characterized by broad terminal differentiation defects and variable immune abnormalities.	J Allergy Clin Immunol.
atopic dermatitis	ZNF444	Expression profiling by array, count, 3 development stage, 2 disease state, 11 individual sets	GPL570	GSE32924 Samples	Nonlesional atopic dermatitis skin is characterized by broad terminal differentiation defects and variable immune abnormalities.	J Allergy Clin Immunol.
atopic dermatitis	TELO2	Expression profiling by array, count, 3 development stage, 2 disease state, 11 individual sets	GPL570	GSE32924 Samples	Nonlesional atopic dermatitis skin is characterized by broad terminal differentiation defects and variable immune abnormalities.	J Allergy Clin Immunol.
atopic dermatitis	LMF1	Expression profiling by array, count, 3 development stage, 2 disease state, 11 individual sets	GPL570	GSE32924 Samples	Nonlesional atopic dermatitis skin is characterized by broad terminal differentiation defects and variable immune abnormalities.	J Allergy Clin Immunol.
atopic dermatitis	RNPEPL1	Expression profiling by array, count, 3 development stage, 2 disease state, 11 individual sets	GPL570	GSE32924 Samples	Nonlesional atopic dermatitis skin is characterized by broad terminal differentiation defects and variable immune abnormalities.	J Allergy Clin Immunol.
atopic dermatitis	POLD1	Expression profiling by array, count, 3 development stage, 2 disease state, 11 individual sets	GPL570	GSE32924 Samples	Nonlesional atopic dermatitis skin is characterized by broad terminal differentiation defects and variable immune abnormalities.	J Allergy Clin Immunol.
atopic dermatitis	RAB4B	Expression profiling by array, count, 3 development stage, 2 disease state, 11 individual sets	GPL570	GSE32924 Samples	Nonlesional atopic dermatitis skin is characterized by broad terminal differentiation defects and variable immune abnormalities.	J Allergy Clin Immunol.
atopic dermatitis	DGCR14	Expression profiling by array, count, 3 development stage, 2 disease state, 11 individual sets	GPL570	GSE32924 Samples	Nonlesional atopic dermatitis skin is characterized by broad terminal differentiation defects and variable immune abnormalities.	J Allergy Clin Immunol.

atopic dermatitis	ATG4D	Expression profiling by array, count, 3 development stage, 2 disease state, 11 individual sets	GPL570	GSE32924 Samples	Nonlesional atopic dermatitis skin is characterized by broad terminal differentiation defects and variable immune abnormalities.	J Allergy Clin Immunol.
atopic dermatitis	CYHR1	Expression profiling by array, count, 3 development stage, 2 disease state, 11 individual sets	GPL570	GSE32924 Samples	Nonlesional atopic dermatitis skin is characterized by broad terminal differentiation defects and variable immune abnormalities.	J Allergy Clin Immunol.
atopic dermatitis	RAVER1	Expression profiling by array, count, 3 development stage, 2 disease state, 11 individual sets	GPL570	GSE32924 Samples	Nonlesional atopic dermatitis skin is characterized by broad terminal differentiation defects and variable immune abnormalities.	J Allergy Clin Immunol.
atopic dermatitis	C4ORF48	Expression profiling by array, count, 3 development stage, 2 disease state, 11 individual sets	GPL570	GSE32924 Samples	Nonlesional atopic dermatitis skin is characterized by broad terminal differentiation defects and variable immune abnormalities.	J Allergy Clin Immunol.
atopic dermatitis	GSDMD	Expression profiling by array, count, 3 development stage, 2 disease state, 11 individual sets	GPL570	GSE32924 Samples	Nonlesional atopic dermatitis skin is characterized by broad terminal differentiation defects and variable immune abnormalities.	J Allergy Clin Immunol.
atopic dermatitis	HSD17B1	Expression profiling by array, count, 3 development stage, 2 disease state, 11 individual sets	GPL570	GSE32924 Samples	Nonlesional atopic dermatitis skin is characterized by broad terminal differentiation defects and variable immune abnormalities.	J Allergy Clin Immunol.
atopic dermatitis	RPUSD3	Expression profiling by array, count, 3 development stage, 2 disease state, 11 individual sets	GPL570	GSE32924 Samples	Nonlesional atopic dermatitis skin is characterized by broad terminal differentiation defects and variable immune abnormalities.	J Allergy Clin Immunol.
atopic dermatitis	PCYT2	Expression profiling by array, count, 3 development stage, 2 disease state, 11 individual sets	GPL570	GSE32924 Samples	Nonlesional atopic dermatitis skin is characterized by broad terminal differentiation defects and variable immune abnormalities.	J Allergy Clin Immunol.
atopic dermatitis	FAM173A	Expression profiling by array, count, 3 development stage, 2 disease state, 11 individual sets	GPL570	GSE32924 Samples	Nonlesional atopic dermatitis skin is characterized by broad terminal differentiation defects and variable immune abnormalities.	J Allergy Clin Immunol.
atopic dermatitis	LOC100133039	Expression profiling by array, count, 3 development stage, 2 disease state, 11 individual sets	GPL570	GSE32924 Samples	Nonlesional atopic dermatitis skin is characterized by broad terminal differentiation defects and variable immune abnormalities.	J Allergy Clin Immunol.
atopic dermatitis	PPFIA3	Expression profiling by array, count, 3 development stage, 2 disease state, 11 individual sets	GPL570	GSE32924 Samples	Nonlesional atopic dermatitis skin is characterized by broad terminal differentiation defects and variable immune abnormalities.	J Allergy Clin Immunol.
atopic dermatitis	CLCN7	Expression profiling by array, count, 3 development stage, 2 disease state, 11 individual sets	GPL570	GSE32924 Samples	Nonlesional atopic dermatitis skin is characterized by broad terminal differentiation defects and variable immune abnormalities.	J Allergy Clin Immunol.
atopic dermatitis	THOC4	Expression profiling by array, count, 3 development stage, 2 disease state, 11 individual sets	GPL570	GSE32924 Samples	Nonlesional atopic dermatitis skin is characterized by broad terminal differentiation defects and variable immune abnormalities.	J Allergy Clin Immunol.
atopic dermatitis	ATP13A2	Expression profiling by array, count, 3 development stage, 2 disease state, 11 individual sets	GPL570	GSE32924 Samples	Nonlesional atopic dermatitis skin is characterized by broad terminal differentiation defects and variable immune abnormalities.	J Allergy Clin Immunol.
atopic dermatitis	FASTK	Expression profiling by array, count, 3 development stage, 2 disease state, 11 individual sets	GPL570	GSE32924 Samples	Nonlesional atopic dermatitis skin is characterized by broad terminal differentiation defects and variable immune abnormalities.	J Allergy Clin Immunol.
atopic dermatitis	TBC1D5	Expression profiling by array, count, 3 development stage, 2 disease state, 11 individual sets	GPL570	GSE32924 Samples	Nonlesional atopic dermatitis skin is characterized by broad terminal differentiation defects and variable immune abnormalities.	J Allergy Clin Immunol.
atopic dermatitis	DHRS13	Expression profiling by array, count, 3 development stage, 2 disease state, 11 individual sets	GPL570	GSE32924 Samples	Nonlesional atopic dermatitis skin is characterized by broad terminal differentiation defects and variable immune abnormalities.	J Allergy Clin Immunol.
atopic dermatitis	COMTD1	Expression profiling by array, count, 3 development stage, 2 disease state, 11 individual sets	GPL570	GSE32924 Samples	Nonlesional atopic dermatitis skin is characterized by broad terminal differentiation defects and variable immune abnormalities.	J Allergy Clin Immunol.
atopic dermatitis	MTG1	Expression profiling by array, count, 3 development stage, 2 disease state, 11 individual sets	GPL570	GSE32924 Samples	Nonlesional atopic dermatitis skin is characterized by broad terminal differentiation defects and variable immune abnormalities.	J Allergy Clin Immunol.
atopic dermatitis	NA	Expression profiling by array, count, 3 development stage, 2 disease state, 11 individual sets	GPL570	GSE32924 Samples	Nonlesional atopic dermatitis skin is characterized by broad terminal differentiation defects and variable immune abnormalities.	J Allergy Clin Immunol.
atopic dermatitis	ITGB6	Expression profiling by array, count, 3 development stage, 2 disease state, 11 individual sets	GPL570	GSE32924 Samples	Nonlesional atopic dermatitis skin is characterized by broad terminal differentiation defects and variable immune abnormalities.	J Allergy Clin Immunol.
atopic dermatitis	PTP4A3	Expression profiling by array, count, 3 development stage, 2 disease state, 11 individual sets	GPL570	GSE32924 Samples	Nonlesional atopic dermatitis skin is characterized by broad terminal differentiation defects and variable immune abnormalities.	J Allergy Clin Immunol.
atopic dermatitis	C1QTNF1	Expression profiling by array, count, 3 development stage, 2 disease state, 11 individual sets	GPL570	GSE32924 Samples	Nonlesional atopic dermatitis skin is characterized by broad terminal differentiation defects and variable immune abnormalities.	J Allergy Clin Immunol.
atopic dermatitis	NCEH1	Expression profiling by array, count, 3 development stage, 2 disease state, 11 individual sets	GPL570	GSE32924 Samples	Nonlesional atopic dermatitis skin is characterized by broad terminal differentiation defects and variable immune abnormalities.	J Allergy Clin Immunol.
atopic dermatitis	NA	Expression profiling by array, count, 3 development stage, 2 disease state, 11 individual sets	GPL570	GSE32924 Samples	Nonlesional atopic dermatitis skin is characterized by broad terminal differentiation defects and variable immune abnormalities.	J Allergy Clin Immunol.
atopic dermatitis	XRCC5	Expression profiling by array, count, 3 development stage, 2 disease state, 11 individual sets	GPL570	GSE32924 Samples	Nonlesional atopic dermatitis skin is characterized by broad terminal differentiation defects and variable immune abnormalities.	J Allergy Clin Immunol.
atopic dermatitis	N4BP2L2	Expression profiling by array, count, 3 development stage, 2 disease state, 11 individual sets	GPL570	GSE32924 Samples	Nonlesional atopic dermatitis skin is characterized by broad terminal differentiation defects and variable immune abnormalities.	J Allergy Clin Immunol.
atopic dermatitis	NA	Expression profiling by array, count, 3 development stage, 2 disease state, 11 individual sets	GPL570	GSE32924 Samples	Nonlesional atopic dermatitis skin is characterized by broad terminal differentiation defects and variable immune abnormalities.	J Allergy Clin Immunol.
atopic dermatitis	DAPK2	Expression profiling by array, count, 3 development stage, 2 disease state, 11 individual sets	GPL570	GSE32924 Samples	Nonlesional atopic dermatitis skin is characterized by broad terminal differentiation defects and variable immune abnormalities.	J Allergy Clin Immunol.
atopic dermatitis	ATF7IP	Expression profiling by array, count, 3 development stage, 2 disease state, 11 individual sets	GPL570	GSE32924 Samples	Nonlesional atopic dermatitis skin is characterized by broad terminal differentiation defects and variable immune abnormalities.	J Allergy Clin Immunol.
atopic dermatitis	NA	Expression profiling by array, count, 3 development stage, 2 disease state, 11 individual sets	GPL570	GSE32924 Samples	Nonlesional atopic dermatitis skin is characterized by broad terminal differentiation defects and variable immune abnormalities.	J Allergy Clin Immunol.
atopic dermatitis	TMEM8A	Expression profiling by array, count, 3 development stage, 2 disease state, 11 individual sets	GPL570	GSE32924 Samples	Nonlesional atopic dermatitis skin is characterized by broad terminal differentiation defects and variable immune abnormalities.	J Allergy Clin Immunol.
atopic dermatitis	FBXO2	Expression profiling by array, count, 3 development stage, 2 disease state, 11 individual sets	GPL570	GSE32924 Samples	Nonlesional atopic dermatitis skin is characterized by broad terminal differentiation defects and variable immune abnormalities.	J Allergy Clin Immunol.
atopic dermatitis	NECAB3	Expression profiling by array, count, 3 development stage, 2 disease state, 11 individual sets	GPL570	GSE32924 Samples	Nonlesional atopic dermatitis skin is characterized by broad terminal differentiation defects and variable immune abnormalities.	J Allergy Clin Immunol.
atopic dermatitis	C16ORF14	Expression profiling by array, count, 3 development stage, 2 disease state, 11 individual sets	GPL570	GSE32924 Samples	Nonlesional atopic dermatitis skin is characterized by broad terminal differentiation defects and variable immune abnormalities.	J Allergy Clin Immunol.
atopic dermatitis	CX3CL1	Expression profiling by array, count, 3 development stage, 2 disease state, 11 individual sets	GPL570	GSE32924 Samples	Nonlesional atopic dermatitis skin is characterized by broad terminal differentiation defects and variable immune abnormalities.	J Allergy Clin Immunol.
atopic dermatitis	TCOF1	Expression profiling by array, count, 3 development stage, 2 disease state, 11 individual sets	GPL570	GSE32924 Samples	Nonlesional atopic dermatitis skin is characterized by broad terminal differentiation defects and variable immune abnormalities.	J Allergy Clin Immunol.
atopic dermatitis	C16ORF48	Expression profiling by array, count, 3 development stage, 2 disease state, 11 individual sets	GPL570	GSE32924 Samples	Nonlesional atopic dermatitis skin is characterized by broad terminal differentiation defects and variable immune abnormalities.	J Allergy Clin Immunol.
atopic dermatitis	NA	Expression profiling by array, count, 3 development stage, 2 disease state, 11 individual sets	GPL570	GSE32924 Samples	Nonlesional atopic dermatitis skin is characterized by broad terminal differentiation defects and variable immune abnormalities.	J Allergy Clin Immunol.
atopic dermatitis	NA	Expression profiling by array, count, 3 development stage, 2 disease state, 11 individual sets	GPL570	GSE32924 Samples	Nonlesional atopic dermatitis skin is characterized by broad terminal differentiation defects and variable immune abnormalities.	J Allergy Clin Immunol.
atopic dermatitis	CSPG4	Expression profiling by array, count, 3 development stage, 2 disease state, 11 individual sets	GPL570	GSE32924 Samples	Nonlesional atopic dermatitis skin is characterized by broad terminal differentiation defects and variable immune abnormalities.	J Allergy Clin Immunol.

atopic dermatitis	BAT2	Expression profiling by array, count, 3 development stage, 2 disease state, 11 individual sets	GPL570	GSE32924 Samples	Nonlesional atopic dermatitis skin is characterized by broad terminal differentiation defects and variable immune abnormalities.	J Allergy Clin Immunol.
atopic dermatitis	SH2B1	Expression profiling by array, count, 3 development stage, 2 disease state, 11 individual sets	GPL570	GSE32924 Samples	Nonlesional atopic dermatitis skin is characterized by broad terminal differentiation defects and variable immune abnormalities.	J Allergy Clin Immunol.
atopic dermatitis	SH2B1	Expression profiling by array, count, 3 development stage, 2 disease state, 11 individual sets	GPL570	GSE32924 Samples	Nonlesional atopic dermatitis skin is characterized by broad terminal differentiation defects and variable immune abnormalities.	J Allergy Clin Immunol.
atopic dermatitis	NELF	Expression profiling by array, count, 3 development stage, 2 disease state, 11 individual sets	GPL570	GSE32924 Samples	Nonlesional atopic dermatitis skin is characterized by broad terminal differentiation defects and variable immune abnormalities.	J Allergy Clin Immunol.
atopic dermatitis	C6ORF136	Expression profiling by array, count, 3 development stage, 2 disease state, 11 individual sets	GPL570	GSE32924 Samples	Nonlesional atopic dermatitis skin is characterized by broad terminal differentiation defects and variable immune abnormalities.	J Allergy Clin Immunol.
atopic dermatitis	TP53I13	Expression profiling by array, count, 3 development stage, 2 disease state, 11 individual sets	GPL570	GSE32924 Samples	Nonlesional atopic dermatitis skin is characterized by broad terminal differentiation defects and variable immune abnormalities.	J Allergy Clin Immunol.
atopic dermatitis	COL27A1	Expression profiling by array, count, 3 development stage, 2 disease state, 11 individual sets	GPL570	GSE32924 Samples	Nonlesional atopic dermatitis skin is characterized by broad terminal differentiation defects and variable immune abnormalities.	J Allergy Clin Immunol.
atopic dermatitis	ABCA7	Expression profiling by array, count, 3 development stage, 2 disease state, 11 individual sets	GPL570	GSE32924 Samples	Nonlesional atopic dermatitis skin is characterized by broad terminal differentiation defects and variable immune abnormalities.	J Allergy Clin Immunol.
atopic dermatitis	CSPG4	Expression profiling by array, count, 3 development stage, 2 disease state, 11 individual sets	GPL570	GSE32924 Samples	Nonlesional atopic dermatitis skin is characterized by broad terminal differentiation defects and variable immune abnormalities.	J Allergy Clin Immunol.
atopic dermatitis	C17ORF70	Expression profiling by array, count, 3 development stage, 2 disease state, 11 individual sets	GPL570	GSE32924 Samples	Nonlesional atopic dermatitis skin is characterized by broad terminal differentiation defects and variable immune abnormalities.	J Allergy Clin Immunol.
atopic dermatitis	NA	Expression profiling by array, count, 3 development stage, 2 disease state, 11 individual sets	GPL570	GSE32924 Samples	Nonlesional atopic dermatitis skin is characterized by broad terminal differentiation defects and variable immune abnormalities.	J Allergy Clin Immunol.
atopic dermatitis	MAP3K11	Expression profiling by array, count, 3 development stage, 2 disease state, 11 individual sets	GPL570	GSE32924 Samples	Nonlesional atopic dermatitis skin is characterized by broad terminal differentiation defects and variable immune abnormalities.	J Allergy Clin Immunol.
atopic dermatitis	TMEM120A	Expression profiling by array, count, 3 development stage, 2 disease state, 11 individual sets	GPL570	GSE32924 Samples	Nonlesional atopic dermatitis skin is characterized by broad terminal differentiation defects and variable immune abnormalities.	J Allergy Clin Immunol.
atopic dermatitis	C1ORF86	Expression profiling by array, count, 3 development stage, 2 disease state, 11 individual sets	GPL570	GSE32924 Samples	Nonlesional atopic dermatitis skin is characterized by broad terminal differentiation defects and variable immune abnormalities.	J Allergy Clin Immunol.
atopic dermatitis	MICALL2	Expression profiling by array, count, 3 development stage, 2 disease state, 11 individual sets	GPL570	GSE32924 Samples	Nonlesional atopic dermatitis skin is characterized by broad terminal differentiation defects and variable immune abnormalities.	J Allergy Clin Immunol.
atopic dermatitis	ACAP3	Expression profiling by array, count, 3 development stage, 2 disease state, 11 individual sets	GPL570	GSE32924 Samples	Nonlesional atopic dermatitis skin is characterized by broad terminal differentiation defects and variable immune abnormalities.	J Allergy Clin Immunol.
atopic dermatitis	IRX4	Expression profiling by array, count, 3 development stage, 2 disease state, 11 individual sets	GPL570	GSE32924 Samples	Nonlesional atopic dermatitis skin is characterized by broad terminal differentiation defects and variable immune abnormalities.	J Allergy Clin Immunol.
atopic dermatitis	C16ORF7	Expression profiling by array, count, 3 development stage, 2 disease state, 11 individual sets	GPL570	GSE32924 Samples	Nonlesional atopic dermatitis skin is characterized by broad terminal differentiation defects and variable immune abnormalities.	J Allergy Clin Immunol.
atopic dermatitis	LPHN1	Expression profiling by array, count, 3 development stage, 2 disease state, 11 individual sets	GPL570	GSE32924 Samples	Nonlesional atopic dermatitis skin is characterized by broad terminal differentiation defects and variable immune abnormalities.	J Allergy Clin Immunol.
atopic dermatitis	NA	Expression profiling by array, count, 3 development stage, 2 disease state, 11 individual sets	GPL570	GSE32924 Samples	Nonlesional atopic dermatitis skin is characterized by broad terminal differentiation defects and variable immune abnormalities.	J Allergy Clin Immunol.
atopic dermatitis	TLR5	Expression profiling by array, count, 3 development stage, 2 disease state, 11 individual sets	GPL570	GSE32924 Samples	Nonlesional atopic dermatitis skin is characterized by broad terminal differentiation defects and variable immune abnormalities.	J Allergy Clin Immunol.
atopic dermatitis	PABPC1L	Expression profiling by array, count, 3 development stage, 2 disease state, 11 individual sets	GPL570	GSE32924 Samples	Nonlesional atopic dermatitis skin is characterized by broad terminal differentiation defects and variable immune abnormalities.	J Allergy Clin Immunol.
atopic dermatitis	LOC646014	Expression profiling by array, count, 3 development stage, 2 disease state, 11 individual sets	GPL570	GSE32924 Samples	Nonlesional atopic dermatitis skin is characterized by broad terminal differentiation defects and variable immune abnormalities.	J Allergy Clin Immunol.
atopic dermatitis	ATHL1	Expression profiling by array, count, 3 development stage, 2 disease state, 11 individual sets	GPL570	GSE32924 Samples	Nonlesional atopic dermatitis skin is characterized by broad terminal differentiation defects and variable immune abnormalities.	J Allergy Clin Immunol.
atopic dermatitis	C9ORF86	Expression profiling by array, count, 3 development stage, 2 disease state, 11 individual sets	GPL570	GSE32924 Samples	Nonlesional atopic dermatitis skin is characterized by broad terminal differentiation defects and variable immune abnormalities.	J Allergy Clin Immunol.
atopic dermatitis	SH3PXD2A	Expression profiling by array, count, 3 development stage, 2 disease state, 11 individual sets	GPL570	GSE32924 Samples	Nonlesional atopic dermatitis skin is characterized by broad terminal differentiation defects and variable immune abnormalities.	J Allergy Clin Immunol.
atopic dermatitis	NFIC	Expression profiling by array, count, 3 development stage, 2 disease state, 11 individual sets	GPL570	GSE32924 Samples	Nonlesional atopic dermatitis skin is characterized by broad terminal differentiation defects and variable immune abnormalities.	J Allergy Clin Immunol.
atopic dermatitis	C10ORF18	Expression profiling by array, count, 3 development stage, 2 disease state, 11 individual sets	GPL570	GSE32924 Samples	Nonlesional atopic dermatitis skin is characterized by broad terminal differentiation defects and variable immune abnormalities.	J Allergy Clin Immunol.
atopic dermatitis	SF3B1	Expression profiling by array, count, 3 development stage, 2 disease state, 11 individual sets	GPL570	GSE32924 Samples	Nonlesional atopic dermatitis skin is characterized by broad terminal differentiation defects and variable immune abnormalities.	J Allergy Clin Immunol.
atopic dermatitis	NA	Expression profiling by array, count, 3 development stage, 2 disease state, 11 individual sets	GPL570	GSE32924 Samples	Nonlesional atopic dermatitis skin is characterized by broad terminal differentiation defects and variable immune abnormalities.	J Allergy Clin Immunol.
atopic dermatitis	NA	Expression profiling by array, count, 3 development stage, 2 disease state, 11 individual sets	GPL570	GSE32924 Samples	Nonlesional atopic dermatitis skin is characterized by broad terminal differentiation defects and variable immune abnormalities.	J Allergy Clin Immunol.
atopic dermatitis	NA	Expression profiling by array, count, 3 development stage, 2 disease state, 11 individual sets	GPL570	GSE32924 Samples	Nonlesional atopic dermatitis skin is characterized by broad terminal differentiation defects and variable immune abnormalities.	J Allergy Clin Immunol.
atopic dermatitis	ZNF785	Expression profiling by array, count, 3 development stage, 2 disease state, 11 individual sets	GPL570	GSE32924 Samples	Nonlesional atopic dermatitis skin is characterized by broad terminal differentiation defects and variable immune abnormalities.	J Allergy Clin Immunol.
atopic dermatitis	ZNF785	Expression profiling by array, count, 3 development stage, 2 disease state, 11 individual sets	GPL570	GSE32924 Samples	Nonlesional atopic dermatitis skin is characterized by broad terminal differentiation defects and variable immune abnormalities.	J Allergy Clin Immunol.
atopic dermatitis	NA	Expression profiling by array, count, 3 development stage, 2 disease state, 11 individual sets	GPL570	GSE32924 Samples	Nonlesional atopic dermatitis skin is characterized by broad terminal differentiation defects and variable immune abnormalities.	J Allergy Clin Immunol.
atopic dermatitis	RSBN1	Expression profiling by array, count, 3 development stage, 2 disease state, 11 individual sets	GPL570	GSE32924 Samples	Nonlesional atopic dermatitis skin is characterized by broad terminal differentiation defects and variable immune abnormalities.	J Allergy Clin Immunol.
atopic dermatitis	NA	Expression profiling by array, count, 3 development stage, 2 disease state, 11 individual sets	GPL570	GSE32924 Samples	Nonlesional atopic dermatitis skin is characterized by broad terminal differentiation defects and variable immune abnormalities.	J Allergy Clin Immunol.
atopic dermatitis	NA	Expression profiling by array, count, 3 development stage, 2 disease state, 11 individual sets	GPL570	GSE32924 Samples	Nonlesional atopic dermatitis skin is characterized by broad terminal differentiation defects and variable immune abnormalities.	J Allergy Clin Immunol.
atopic dermatitis	LRRFIP1	Expression profiling by array, count, 3 development stage, 2 disease state, 11 individual sets	GPL570	GSE32924 Samples	Nonlesional atopic dermatitis skin is characterized by broad terminal differentiation defects and variable immune abnormalities.	J Allergy Clin Immunol.
atopic dermatitis	AGXT2L2	Expression profiling by array, count, 3 development stage, 2 disease state, 11 individual sets	GPL570	GSE32924 Samples	Nonlesional atopic dermatitis skin is characterized by broad terminal differentiation defects and variable immune abnormalities.	J Allergy Clin Immunol.
atopic dermatitis	USP31	Expression profiling by array, count, 3 development stage, 2 disease state, 11 individual sets	GPL570	GSE32924 Samples	Nonlesional atopic dermatitis skin is characterized by broad terminal differentiation defects and variable immune abnormalities.	J Allergy Clin Immunol.

atopic dermatitis	NA	Expression profiling by array, count, 3 development stage, 2 disease state, 11 individual sets	GPL570	GSE32924 Samples	Nonlesional atopic dermatitis skin is characterized by broad terminal differentiation defects and variable immune abnormalities.	J Allergy Clin Immunol.
atopic dermatitis	NAMPT	Expression profiling by array, count, 3 development stage, 2 disease state, 11 individual sets	GPL570	GSE32924 Samples	Nonlesional atopic dermatitis skin is characterized by broad terminal differentiation defects and variable immune abnormalities.	J Allergy Clin Immunol.
atopic dermatitis	NA	Expression profiling by array, count, 3 development stage, 2 disease state, 11 individual sets	GPL570	GSE32924 Samples	Nonlesional atopic dermatitis skin is characterized by broad terminal differentiation defects and variable immune abnormalities.	J Allergy Clin Immunol.
atopic dermatitis	NA	Expression profiling by array, count, 3 development stage, 2 disease state, 11 individual sets	GPL570	GSE32924 Samples	Nonlesional atopic dermatitis skin is characterized by broad terminal differentiation defects and variable immune abnormalities.	J Allergy Clin Immunol.
atopic dermatitis	PXDN	Expression profiling by array, count, 3 development stage, 2 disease state, 11 individual sets	GPL570	GSE32924 Samples	Nonlesional atopic dermatitis skin is characterized by broad terminal differentiation defects and variable immune abnormalities.	J Allergy Clin Immunol.
atopic dermatitis	PDXK	Expression profiling by array, count, 3 development stage, 2 disease state, 11 individual sets	GPL570	GSE32924 Samples	Nonlesional atopic dermatitis skin is characterized by broad terminal differentiation defects and variable immune abnormalities.	J Allergy Clin Immunol.
atopic dermatitis	ANGPTL4	Expression profiling by array, count, 3 development stage, 2 disease state, 11 individual sets	GPL570	GSE32924 Samples	Nonlesional atopic dermatitis skin is characterized by broad terminal differentiation defects and variable immune abnormalities.	J Allergy Clin Immunol.
atopic dermatitis	GPX2	Expression profiling by array, count, 3 development stage, 2 disease state, 11 individual sets	GPL570	GSE32924 Samples	Nonlesional atopic dermatitis skin is characterized by broad terminal differentiation defects and variable immune abnormalities.	J Allergy Clin Immunol.
atopic dermatitis	PI15	Expression profiling by array, count, 3 development stage, 2 disease state, 11 individual sets	GPL570	GSE32924 Samples	Nonlesional atopic dermatitis skin is characterized by broad terminal differentiation defects and variable immune abnormalities.	J Allergy Clin Immunol.
atopic dermatitis	PSENEN	Expression profiling by array, count, 3 development stage, 2 disease state, 11 individual sets	GPL570	GSE32924 Samples	Nonlesional atopic dermatitis skin is characterized by broad terminal differentiation defects and variable immune abnormalities.	J Allergy Clin Immunol.
atopic dermatitis	THOC4	Expression profiling by array, count, 3 development stage, 2 disease state, 11 individual sets	GPL570	GSE32924 Samples	Nonlesional atopic dermatitis skin is characterized by broad terminal differentiation defects and variable immune abnormalities.	J Allergy Clin Immunol.
atopic dermatitis	HES4	Expression profiling by array, count, 3 development stage, 2 disease state, 11 individual sets	GPL570	GSE32924 Samples	Nonlesional atopic dermatitis skin is characterized by broad terminal differentiation defects and variable immune abnormalities.	J Allergy Clin Immunol.
atopic dermatitis	ODF3B	Expression profiling by array, count, 3 development stage, 2 disease state, 11 individual sets	GPL570	GSE32924 Samples	Nonlesional atopic dermatitis skin is characterized by broad terminal differentiation defects and variable immune abnormalities.	J Allergy Clin Immunol.
atopic dermatitis	TYMP	Expression profiling by array, count, 3 development stage, 2 disease state, 11 individual sets	GPL570	GSE32924 Samples	Nonlesional atopic dermatitis skin is characterized by broad terminal differentiation defects and variable immune abnormalities.	J Allergy Clin Immunol.
atopic dermatitis	TNFRSF12A	Expression profiling by array, count, 3 development stage, 2 disease state, 11 individual sets	GPL570	GSE32924 Samples	Nonlesional atopic dermatitis skin is characterized by broad terminal differentiation defects and variable immune abnormalities.	J Allergy Clin Immunol.
atopic dermatitis	FLAD1	Expression profiling by array, count, 3 development stage, 2 disease state, 11 individual sets	GPL570	GSE32924 Samples	Nonlesional atopic dermatitis skin is characterized by broad terminal differentiation defects and variable immune abnormalities.	J Allergy Clin Immunol.
atopic dermatitis	HEATR7A	Expression profiling by array, count, 3 development stage, 2 disease state, 11 individual sets	GPL570	GSE32924 Samples	Nonlesional atopic dermatitis skin is characterized by broad terminal differentiation defects and variable immune abnormalities.	J Allergy Clin Immunol.
atopic dermatitis	NOC4L	Expression profiling by array, count, 3 development stage, 2 disease state, 11 individual sets	GPL570	GSE32924 Samples	Nonlesional atopic dermatitis skin is characterized by broad terminal differentiation defects and variable immune abnormalities.	J Allergy Clin Immunol.
atopic dermatitis	GLTPD1	Expression profiling by array, count, 3 development stage, 2 disease state, 11 individual sets	GPL570	GSE32924 Samples	Nonlesional atopic dermatitis skin is characterized by broad terminal differentiation defects and variable immune abnormalities.	J Allergy Clin Immunol.
atopic dermatitis	LRCH4	Expression profiling by array, count, 3 development stage, 2 disease state, 11 individual sets	GPL570	GSE32924 Samples	Nonlesional atopic dermatitis skin is characterized by broad terminal differentiation defects and variable immune abnormalities.	J Allergy Clin Immunol.
atopic dermatitis	STOML1	Expression profiling by array, count, 3 development stage, 2 disease state, 11 individual sets	GPL570	GSE32924 Samples	Nonlesional atopic dermatitis skin is characterized by broad terminal differentiation defects and variable immune abnormalities.	J Allergy Clin Immunol.
atopic dermatitis	FARSA	Expression profiling by array, count, 3 development stage, 2 disease state, 11 individual sets	GPL570	GSE32924 Samples	Nonlesional atopic dermatitis skin is characterized by broad terminal differentiation defects and variable immune abnormalities.	J Allergy Clin Immunol.
atopic dermatitis	KRI1	Expression profiling by array, count, 3 development stage, 2 disease state, 11 individual sets	GPL570	GSE32924 Samples	Nonlesional atopic dermatitis skin is characterized by broad terminal differentiation defects and variable immune abnormalities.	J Allergy Clin Immunol.
atopic dermatitis	NA	Expression profiling by array, count, 3 development stage, 2 disease state, 11 individual sets	GPL570	GSE32924 Samples	Nonlesional atopic dermatitis skin is characterized by broad terminal differentiation defects and variable immune abnormalities.	J Allergy Clin Immunol.
atopic dermatitis	PML	Expression profiling by array, count, 3 development stage, 2 disease state, 11 individual sets	GPL570	GSE32924 Samples	Nonlesional atopic dermatitis skin is characterized by broad terminal differentiation defects and variable immune abnormalities.	J Allergy Clin Immunol.
atopic dermatitis	SLC5A6	Expression profiling by array, count, 3 development stage, 2 disease state, 11 individual sets	GPL570	GSE32924 Samples	Nonlesional atopic dermatitis skin is characterized by broad terminal differentiation defects and variable immune abnormalities.	J Allergy Clin Immunol.
atopic dermatitis	NA	Expression profiling by array, count, 3 development stage, 2 disease state, 11 individual sets	GPL570	GSE32924 Samples	Nonlesional atopic dermatitis skin is characterized by broad terminal differentiation defects and variable immune abnormalities.	J Allergy Clin Immunol.
atopic dermatitis	CKS2	Expression profiling by array, count, 3 development stage, 2 disease state, 11 individual sets	GPL570	GSE32924 Samples	Nonlesional atopic dermatitis skin is characterized by broad terminal differentiation defects and variable immune abnormalities.	J Allergy Clin Immunol.
atopic dermatitis	NCAPG	Expression profiling by array, count, 3 development stage, 2 disease state, 11 individual sets	GPL570	GSE32924 Samples	Nonlesional atopic dermatitis skin is characterized by broad terminal differentiation defects and variable immune abnormalities.	J Allergy Clin Immunol.
atopic dermatitis	KIF18B	Expression profiling by array, count, 3 development stage, 2 disease state, 11 individual sets	GPL570	GSE32924 Samples	Nonlesional atopic dermatitis skin is characterized by broad terminal differentiation defects and variable immune abnormalities.	J Allergy Clin Immunol.
atopic dermatitis	WNT5A	Expression profiling by array, count, 3 development stage, 2 disease state, 11 individual sets	GPL570	GSE32924 Samples	Nonlesional atopic dermatitis skin is characterized by broad terminal differentiation defects and variable immune abnormalities.	J Allergy Clin Immunol.
atopic dermatitis	FANCI	Expression profiling by array, count, 3 development stage, 2 disease state, 11 individual sets	GPL570	GSE32924 Samples	Nonlesional atopic dermatitis skin is characterized by broad terminal differentiation defects and variable immune abnormalities.	J Allergy Clin Immunol.
atopic dermatitis	IRF7	Expression profiling by array, count, 3 development stage, 2 disease state, 11 individual sets	GPL570	GSE32924 Samples	Nonlesional atopic dermatitis skin is characterized by broad terminal differentiation defects and variable immune abnormalities.	J Allergy Clin Immunol.
atopic dermatitis	NA	Expression profiling by array, count, 3 development stage, 2 disease state, 11 individual sets	GPL570	GSE32924 Samples	Nonlesional atopic dermatitis skin is characterized by broad terminal differentiation defects and variable immune abnormalities.	J Allergy Clin Immunol.
atopic dermatitis	IFI35	Expression profiling by array, count, 3 development stage, 2 disease state, 11 individual sets	GPL570	GSE32924 Samples	Nonlesional atopic dermatitis skin is characterized by broad terminal differentiation defects and variable immune abnormalities.	J Allergy Clin Immunol.
atopic dermatitis	CLDN7	Expression profiling by array, count, 3 development stage, 2 disease state, 11 individual sets	GPL570	GSE32924 Samples	Nonlesional atopic dermatitis skin is characterized by broad terminal differentiation defects and variable immune abnormalities.	J Allergy Clin Immunol.
atopic dermatitis	ETV1	Expression profiling by array, count, 3 development stage, 2 disease state, 11 individual sets	GPL570	GSE32924 Samples	Nonlesional atopic dermatitis skin is characterized by broad terminal differentiation defects and variable immune abnormalities.	J Allergy Clin Immunol.
atopic dermatitis	NA	Expression profiling by array, count, 3 development stage, 2 disease state, 11 individual sets	GPL570	GSE32924 Samples	Nonlesional atopic dermatitis skin is characterized by broad terminal differentiation defects and variable immune abnormalities.	J Allergy Clin Immunol.
atopic dermatitis	TBRG1	Expression profiling by array, count, 3 development stage, 2 disease state, 11 individual sets	GPL570	GSE32924 Samples	Nonlesional atopic dermatitis skin is characterized by broad terminal differentiation defects and variable immune abnormalities.	J Allergy Clin Immunol.
atopic dermatitis	TOB2	Expression profiling by array, count, 3 development stage, 2 disease state, 11 individual sets	GPL570	GSE32924 Samples	Nonlesional atopic dermatitis skin is characterized by broad terminal differentiation defects and variable immune abnormalities.	J Allergy Clin Immunol.
atopic dermatitis	LOC283788	Expression profiling by array, count, 3 development stage, 2 disease state, 11 individual sets	GPL570	GSE32924 Samples	Nonlesional atopic dermatitis skin is characterized by broad terminal differentiation defects and variable immune abnormalities.	J Allergy Clin Immunol.

atopic dermatitis	RAMP3	Expression profiling by array, count, 3 development stage, 2 disease state, 11 individual sets	GPL570	GSE32924 Samples	Nonlesional atopic dermatitis skin is characterized by broad terminal differentiation defects and variable immune abnormalities.	J Allergy Clin Immunol.
atopic dermatitis	URG4	Expression profiling by array, count, 3 development stage, 2 disease state, 11 individual sets	GPL570	GSE32924 Samples	Nonlesional atopic dermatitis skin is characterized by broad terminal differentiation defects and variable immune abnormalities.	J Allergy Clin Immunol.
atopic dermatitis	MZF1	Expression profiling by array, count, 3 development stage, 2 disease state, 11 individual sets	GPL570	GSE32924 Samples	Nonlesional atopic dermatitis skin is characterized by broad terminal differentiation defects and variable immune abnormalities.	J Allergy Clin Immunol.
atopic dermatitis	LZTR1	Expression profiling by array, count, 3 development stage, 2 disease state, 11 individual sets	GPL570	GSE32924 Samples	Nonlesional atopic dermatitis skin is characterized by broad terminal differentiation defects and variable immune abnormalities.	J Allergy Clin Immunol.
atopic dermatitis	ENTPD6	Expression profiling by array, count, 3 development stage, 2 disease state, 11 individual sets	GPL570	GSE32924 Samples	Nonlesional atopic dermatitis skin is characterized by broad terminal differentiation defects and variable immune abnormalities.	J Allergy Clin Immunol.
atopic dermatitis	ANAPC2	Expression profiling by array, count, 3 development stage, 2 disease state, 11 individual sets	GPL570	GSE32924 Samples	Nonlesional atopic dermatitis skin is characterized by broad terminal differentiation defects and variable immune abnormalities.	J Allergy Clin Immunol.
atopic dermatitis	C19ORF60	Expression profiling by array, count, 3 development stage, 2 disease state, 11 individual sets	GPL570	GSE32924 Samples	Nonlesional atopic dermatitis skin is characterized by broad terminal differentiation defects and variable immune abnormalities.	J Allergy Clin Immunol.
atopic dermatitis	COL5A3	Expression profiling by array, count, 3 development stage, 2 disease state, 11 individual sets	GPL570	GSE32924 Samples	Nonlesional atopic dermatitis skin is characterized by broad terminal differentiation defects and variable immune abnormalities.	J Allergy Clin Immunol.
atopic dermatitis	SORBS3	Expression profiling by array, count, 3 development stage, 2 disease state, 11 individual sets	GPL570	GSE32924 Samples	Nonlesional atopic dermatitis skin is characterized by broad terminal differentiation defects and variable immune abnormalities.	J Allergy Clin Immunol.
atopic dermatitis	C16ORF13	Expression profiling by array, count, 3 development stage, 2 disease state, 11 individual sets	GPL570	GSE32924 Samples	Nonlesional atopic dermatitis skin is characterized by broad terminal differentiation defects and variable immune abnormalities.	J Allergy Clin Immunol.
atopic dermatitis	ELMO3	Expression profiling by array, count, 3 development stage, 2 disease state, 11 individual sets	GPL570	GSE32924 Samples	Nonlesional atopic dermatitis skin is characterized by broad terminal differentiation defects and variable immune abnormalities.	J Allergy Clin Immunol.
atopic dermatitis	TNFRSF14	Expression profiling by array, count, 3 development stage, 2 disease state, 11 individual sets	GPL570	GSE32924 Samples	Nonlesional atopic dermatitis skin is characterized by broad terminal differentiation defects and variable immune abnormalities.	J Allergy Clin Immunol.
atopic dermatitis	C7ORF43	Expression profiling by array, count, 3 development stage, 2 disease state, 11 individual sets	GPL570	GSE32924 Samples	Nonlesional atopic dermatitis skin is characterized by broad terminal differentiation defects and variable immune abnormalities.	J Allergy Clin Immunol.
atopic dermatitis	TMC6	Expression profiling by array, count, 3 development stage, 2 disease state, 11 individual sets	GPL570	GSE32924 Samples	Nonlesional atopic dermatitis skin is characterized by broad terminal differentiation defects and variable immune abnormalities.	J Allergy Clin Immunol.
atopic dermatitis	PRR5	Expression profiling by array, count, 3 development stage, 2 disease state, 11 individual sets	GPL570	GSE32924 Samples	Nonlesional atopic dermatitis skin is characterized by broad terminal differentiation defects and variable immune abnormalities.	J Allergy Clin Immunol.
atopic dermatitis	CYB561	Expression profiling by array, count, 3 development stage, 2 disease state, 11 individual sets	GPL570	GSE32924 Samples	Nonlesional atopic dermatitis skin is characterized by broad terminal differentiation defects and variable immune abnormalities.	J Allergy Clin Immunol.
atopic dermatitis	ZFPM1	Expression profiling by array, count, 3 development stage, 2 disease state, 11 individual sets	GPL570	GSE32924 Samples	Nonlesional atopic dermatitis skin is characterized by broad terminal differentiation defects and variable immune abnormalities.	J Allergy Clin Immunol.
atopic dermatitis	ZNF777	Expression profiling by array, count, 3 development stage, 2 disease state, 11 individual sets	GPL570	GSE32924 Samples	Nonlesional atopic dermatitis skin is characterized by broad terminal differentiation defects and variable immune abnormalities.	J Allergy Clin Immunol.
atopic dermatitis	C19ORF48	Expression profiling by array, count, 3 development stage, 2 disease state, 11 individual sets	GPL570	GSE32924 Samples	Nonlesional atopic dermatitis skin is characterized by broad terminal differentiation defects and variable immune abnormalities.	J Allergy Clin Immunol.
atopic dermatitis	NUDT22	Expression profiling by array, count, 3 development stage, 2 disease state, 11 individual sets	GPL570	GSE32924 Samples	Nonlesional atopic dermatitis skin is characterized by broad terminal differentiation defects and variable immune abnormalities.	J Allergy Clin Immunol.
atopic dermatitis	YIF1B	Expression profiling by array, count, 3 development stage, 2 disease state, 11 individual sets	GPL570	GSE32924 Samples	Nonlesional atopic dermatitis skin is characterized by broad terminal differentiation defects and variable immune abnormalities.	J Allergy Clin Immunol.
atopic dermatitis	CDK2AP2	Expression profiling by array, count, 3 development stage, 2 disease state, 11 individual sets	GPL570	GSE32924 Samples	Nonlesional atopic dermatitis skin is characterized by broad terminal differentiation defects and variable immune abnormalities.	J Allergy Clin Immunol.
atopic dermatitis	PARP10	Expression profiling by array, count, 3 development stage, 2 disease state, 11 individual sets	GPL570	GSE32924 Samples	Nonlesional atopic dermatitis skin is characterized by broad terminal differentiation defects and variable immune abnormalities.	J Allergy Clin Immunol.
atopic dermatitis	NA	Expression profiling by array, count, 3 development stage, 2 disease state, 11 individual sets	GPL570	GSE32924 Samples	Nonlesional atopic dermatitis skin is characterized by broad terminal differentiation defects and variable immune abnormalities.	J Allergy Clin Immunol.
atopic dermatitis	RTKN	Expression profiling by array, count, 3 development stage, 2 disease state, 11 individual sets	GPL570	GSE32924 Samples	Nonlesional atopic dermatitis skin is characterized by broad terminal differentiation defects and variable immune abnormalities.	J Allergy Clin Immunol.
atopic dermatitis	NADSYN1	Expression profiling by array, count, 3 development stage, 2 disease state, 11 individual sets	GPL570	GSE32924 Samples	Nonlesional atopic dermatitis skin is characterized by broad terminal differentiation defects and variable immune abnormalities.	J Allergy Clin Immunol.
atopic dermatitis	ECSIT	Expression profiling by array, count, 3 development stage, 2 disease state, 11 individual sets	GPL570	GSE32924 Samples	Nonlesional atopic dermatitis skin is characterized by broad terminal differentiation defects and variable immune abnormalities.	J Allergy Clin Immunol.
atopic dermatitis	SCAND1	Expression profiling by array, count, 3 development stage, 2 disease state, 11 individual sets	GPL570	GSE32924 Samples	Nonlesional atopic dermatitis skin is characterized by broad terminal differentiation defects and variable immune abnormalities.	J Allergy Clin Immunol.
atopic dermatitis	MFSD10	Expression profiling by array, count, 3 development stage, 2 disease state, 11 individual sets	GPL570	GSE32924 Samples	Nonlesional atopic dermatitis skin is characterized by broad terminal differentiation defects and variable immune abnormalities.	J Allergy Clin Immunol.
atopic dermatitis	C9ORF142	Expression profiling by array, count, 3 development stage, 2 disease state, 11 individual sets	GPL570	GSE32924 Samples	Nonlesional atopic dermatitis skin is characterized by broad terminal differentiation defects and variable immune abnormalities.	J Allergy Clin Immunol.
atopic dermatitis	ARRDC1	Expression profiling by array, count, 3 development stage, 2 disease state, 11 individual sets	GPL570	GSE32924 Samples	Nonlesional atopic dermatitis skin is characterized by broad terminal differentiation defects and variable immune abnormalities.	J Allergy Clin Immunol.
atopic dermatitis	NA	Expression profiling by array, count, 3 development stage, 2 disease state, 11 individual sets	GPL570	GSE32924 Samples	Nonlesional atopic dermatitis skin is characterized by broad terminal differentiation defects and variable immune abnormalities.	J Allergy Clin Immunol.
atopic dermatitis	DNM2	Expression profiling by array, count, 3 development stage, 2 disease state, 11 individual sets	GPL570	GSE32924 Samples	Nonlesional atopic dermatitis skin is characterized by broad terminal differentiation defects and variable immune abnormalities.	J Allergy Clin Immunol.
atopic dermatitis	SAPS1	Expression profiling by array, count, 3 development stage, 2 disease state, 11 individual sets	GPL570	GSE32924 Samples	Nonlesional atopic dermatitis skin is characterized by broad terminal differentiation defects and variable immune abnormalities.	J Allergy Clin Immunol.
atopic dermatitis	CDC42EP1	Expression profiling by array, count, 3 development stage, 2 disease state, 11 individual sets	GPL570	GSE32924 Samples	Nonlesional atopic dermatitis skin is characterized by broad terminal differentiation defects and variable immune abnormalities.	J Allergy Clin Immunol.
atopic dermatitis	NA	Expression profiling by array, count, 3 development stage, 2 disease state, 11 individual sets	GPL570	GSE32924 Samples	Nonlesional atopic dermatitis skin is characterized by broad terminal differentiation defects and variable immune abnormalities.	J Allergy Clin Immunol.
atopic dermatitis	ASXL1	Expression profiling by array, count, 3 development stage, 2 disease state, 11 individual sets	GPL570	GSE32924 Samples	Nonlesional atopic dermatitis skin is characterized by broad terminal differentiation defects and variable immune abnormalities.	J Allergy Clin Immunol.
atopic dermatitis	NA	Expression profiling by array, count, 3 development stage, 2 disease state, 11 individual sets	GPL570	GSE32924 Samples	Nonlesional atopic dermatitis skin is characterized by broad terminal differentiation defects and variable immune abnormalities.	J Allergy Clin Immunol.
atopic dermatitis	DICER1	Expression profiling by array, count, 3 development stage, 2 disease state, 11 individual sets	GPL570	GSE32924 Samples	Nonlesional atopic dermatitis skin is characterized by broad terminal differentiation defects and variable immune abnormalities.	J Allergy Clin Immunol.
atopic dermatitis	NA	Expression profiling by array, count, 3 development stage, 2 disease state, 11 individual sets	GPL570	GSE32924 Samples	Nonlesional atopic dermatitis skin is characterized by broad terminal differentiation defects and variable immune abnormalities.	J Allergy Clin Immunol.
atopic dermatitis	PTER	Expression profiling by array, count, 3 development stage, 2 disease state, 11 individual sets	GPL570	GSE32924 Samples	Nonlesional atopic dermatitis skin is characterized by broad terminal differentiation defects and variable immune abnormalities.	J Allergy Clin Immunol.

atopic dermatitis	TRNP1	Expression profiling by array, count, 3 development stage, 2 disease state, 11 individual sets	GPL570	GSE32924 Samples	Nonlesional atopic dermatitis skin is characterized by broad terminal differentiation defects and variable immune abnormalities.	J Allergy Clin Immunol.
atopic dermatitis	GART	Expression profiling by array, count, 3 development stage, 2 disease state, 11 individual sets	GPL570	GSE32924 Samples	Nonlesional atopic dermatitis skin is characterized by broad terminal differentiation defects and variable immune abnormalities.	J Allergy Clin Immunol.
atopic dermatitis	NA	Expression profiling by array, count, 3 development stage, 2 disease state, 11 individual sets	GPL570	GSE32924 Samples	Nonlesional atopic dermatitis skin is characterized by broad terminal differentiation defects and variable immune abnormalities.	J Allergy Clin Immunol.
atopic dermatitis	C7ORF44	Expression profiling by array, count, 3 development stage, 2 disease state, 11 individual sets	GPL570	GSE32924 Samples	Nonlesional atopic dermatitis skin is characterized by broad terminal differentiation defects and variable immune abnormalities.	J Allergy Clin Immunol.
atopic dermatitis	SNRPA1	Expression profiling by array, count, 3 development stage, 2 disease state, 11 individual sets	GPL570	GSE32924 Samples	Nonlesional atopic dermatitis skin is characterized by broad terminal differentiation defects and variable immune abnormalities.	J Allergy Clin Immunol.
atopic dermatitis	ARFGAP1	Expression profiling by array, count, 3 development stage, 2 disease state, 11 individual sets	GPL570	GSE32924 Samples	Nonlesional atopic dermatitis skin is characterized by broad terminal differentiation defects and variable immune abnormalities.	J Allergy Clin Immunol.
atopic dermatitis	AKAP13	Expression profiling by array, count, 3 development stage, 2 disease state, 11 individual sets	GPL570	GSE32924 Samples	Nonlesional atopic dermatitis skin is characterized by broad terminal differentiation defects and variable immune abnormalities.	J Allergy Clin Immunol.
atopic dermatitis	DPP7	Expression profiling by array, count, 3 development stage, 2 disease state, 11 individual sets	GPL570	GSE32924 Samples	Nonlesional atopic dermatitis skin is characterized by broad terminal differentiation defects and variable immune abnormalities.	J Allergy Clin Immunol.
atopic dermatitis	CDK10	Expression profiling by array, count, 3 development stage, 2 disease state, 11 individual sets	GPL570	GSE32924 Samples	Nonlesional atopic dermatitis skin is characterized by broad terminal differentiation defects and variable immune abnormalities.	J Allergy Clin Immunol.
atopic dermatitis	GTPBP8	Expression profiling by array, count, 3 development stage, 2 disease state, 11 individual sets	GPL570	GSE32924 Samples	Nonlesional atopic dermatitis skin is characterized by broad terminal differentiation defects and variable immune abnormalities.	J Allergy Clin Immunol.
atopic dermatitis	USP42	Expression profiling by array, count, 3 development stage, 2 disease state, 11 individual sets	GPL570	GSE32924 Samples	Nonlesional atopic dermatitis skin is characterized by broad terminal differentiation defects and variable immune abnormalities.	J Allergy Clin Immunol.
atopic dermatitis	ALS2CL	Expression profiling by array, count, 3 development stage, 2 disease state, 11 individual sets	GPL570	GSE32924 Samples	Nonlesional atopic dermatitis skin is characterized by broad terminal differentiation defects and variable immune abnormalities.	J Allergy Clin Immunol.
atopic dermatitis	FAM160B2	Expression profiling by array, count, 3 development stage, 2 disease state, 11 individual sets	GPL570	GSE32924 Samples	Nonlesional atopic dermatitis skin is characterized by broad terminal differentiation defects and variable immune abnormalities.	J Allergy Clin Immunol.
atopic dermatitis	MBD3	Expression profiling by array, count, 3 development stage, 2 disease state, 11 individual sets	GPL570	GSE32924 Samples	Nonlesional atopic dermatitis skin is characterized by broad terminal differentiation defects and variable immune abnormalities.	J Allergy Clin Immunol.
atopic dermatitis	FAM113A	Expression profiling by array, count, 3 development stage, 2 disease state, 11 individual sets	GPL570	GSE32924 Samples	Nonlesional atopic dermatitis skin is characterized by broad terminal differentiation defects and variable immune abnormalities.	J Allergy Clin Immunol.
atopic dermatitis	WDR34	Expression profiling by array, count, 3 development stage, 2 disease state, 11 individual sets	GPL570	GSE32924 Samples	Nonlesional atopic dermatitis skin is characterized by broad terminal differentiation defects and variable immune abnormalities.	J Allergy Clin Immunol.
atopic dermatitis	ATAD3A	Expression profiling by array, count, 3 development stage, 2 disease state, 11 individual sets	GPL570	GSE32924 Samples	Nonlesional atopic dermatitis skin is characterized by broad terminal differentiation defects and variable immune abnormalities.	J Allergy Clin Immunol.
atopic dermatitis	TCIRG1	Expression profiling by array, count, 3 development stage, 2 disease state, 11 individual sets	GPL570	GSE32924 Samples	Nonlesional atopic dermatitis skin is characterized by broad terminal differentiation defects and variable immune abnormalities.	J Allergy Clin Immunol.
atopic dermatitis	DGKZ	Expression profiling by array, count, 3 development stage, 2 disease state, 11 individual sets	GPL570	GSE32924 Samples	Nonlesional atopic dermatitis skin is characterized by broad terminal differentiation defects and variable immune abnormalities.	J Allergy Clin Immunol.
atopic dermatitis	CDK10	Expression profiling by array, count, 3 development stage, 2 disease state, 11 individual sets	GPL570	GSE32924 Samples	Nonlesional atopic dermatitis skin is characterized by broad terminal differentiation defects and variable immune abnormalities.	J Allergy Clin Immunol.
atopic dermatitis	ECE1	Expression profiling by array, count, 3 development stage, 2 disease state, 11 individual sets	GPL570	GSE32924 Samples	Nonlesional atopic dermatitis skin is characterized by broad terminal differentiation defects and variable immune abnormalities.	J Allergy Clin Immunol.
atopic dermatitis	NAPEPLD	Expression profiling by array, count, 3 development stage, 2 disease state, 11 individual sets	GPL570	GSE32924 Samples	Nonlesional atopic dermatitis skin is characterized by broad terminal differentiation defects and variable immune abnormalities.	J Allergy Clin Immunol.
atopic dermatitis	NA	Expression profiling by array, count, 3 development stage, 2 disease state, 11 individual sets	GPL570	GSE32924 Samples	Nonlesional atopic dermatitis skin is characterized by broad terminal differentiation defects and variable immune abnormalities.	J Allergy Clin Immunol.
atopic dermatitis	RGS14	Expression profiling by array, count, 3 development stage, 2 disease state, 11 individual sets	GPL570	GSE32924 Samples	Nonlesional atopic dermatitis skin is characterized by broad terminal differentiation defects and variable immune abnormalities.	J Allergy Clin Immunol.
atopic dermatitis	LINS1	Expression profiling by array, count, 3 development stage, 2 disease state, 11 individual sets	GPL570	GSE32924 Samples	Nonlesional atopic dermatitis skin is characterized by broad terminal differentiation defects and variable immune abnormalities.	J Allergy Clin Immunol.
atopic dermatitis	ILVBL	Expression profiling by array, count, 3 development stage, 2 disease state, 11 individual sets	GPL570	GSE32924 Samples	Nonlesional atopic dermatitis skin is characterized by broad terminal differentiation defects and variable immune abnormalities.	J Allergy Clin Immunol.
atopic dermatitis	BCKDHA	Expression profiling by array, count, 3 development stage, 2 disease state, 11 individual sets	GPL570	GSE32924 Samples	Nonlesional atopic dermatitis skin is characterized by broad terminal differentiation defects and variable immune abnormalities.	J Allergy Clin Immunol.
atopic dermatitis	MRPL12	Expression profiling by array, count, 3 development stage, 2 disease state, 11 individual sets	GPL570	GSE32924 Samples	Nonlesional atopic dermatitis skin is characterized by broad terminal differentiation defects and variable immune abnormalities.	J Allergy Clin Immunol.
atopic dermatitis	TIPRL	Expression profiling by array, count, 3 development stage, 2 disease state, 11 individual sets	GPL570	GSE32924 Samples	Nonlesional atopic dermatitis skin is characterized by broad terminal differentiation defects and variable immune abnormalities.	J Allergy Clin Immunol.
atopic dermatitis	NA	Expression profiling by array, count, 3 development stage, 2 disease state, 11 individual sets	GPL570	GSE32924 Samples	Nonlesional atopic dermatitis skin is characterized by broad terminal differentiation defects and variable immune abnormalities.	J Allergy Clin Immunol.
atopic dermatitis	NA	Expression profiling by array, count, 3 development stage, 2 disease state, 11 individual sets	GPL570	GSE32924 Samples	Nonlesional atopic dermatitis skin is characterized by broad terminal differentiation defects and variable immune abnormalities.	J Allergy Clin Immunol.
atopic dermatitis	NA	Expression profiling by array, count, 3 development stage, 2 disease state, 11 individual sets	GPL570	GSE32924 Samples	Nonlesional atopic dermatitis skin is characterized by broad terminal differentiation defects and variable immune abnormalities.	J Allergy Clin Immunol.
atopic dermatitis	FOXP1	Expression profiling by array, count, 3 development stage, 2 disease state, 11 individual sets	GPL570	GSE32924 Samples	Nonlesional atopic dermatitis skin is characterized by broad terminal differentiation defects and variable immune abnormalities.	J Allergy Clin Immunol.
atopic dermatitis	S100A10	Expression profiling by array, count, 3 development stage, 2 disease state, 11 individual sets	GPL570	GSE32924 Samples	Nonlesional atopic dermatitis skin is characterized by broad terminal differentiation defects and variable immune abnormalities.	J Allergy Clin Immunol.
atopic dermatitis	ZGPAT	Expression profiling by array, count, 3 development stage, 2 disease state, 11 individual sets	GPL570	GSE32924 Samples	Nonlesional atopic dermatitis skin is characterized by broad terminal differentiation defects and variable immune abnormalities.	J Allergy Clin Immunol.
atopic dermatitis	NA	Expression profiling by array, count, 3 development stage, 2 disease state, 11 individual sets	GPL570	GSE32924 Samples	Nonlesional atopic dermatitis skin is characterized by broad terminal differentiation defects and variable immune abnormalities.	J Allergy Clin Immunol.
atopic dermatitis	TMEM129	Expression profiling by array, count, 3 development stage, 2 disease state, 11 individual sets	GPL570	GSE32924 Samples	Nonlesional atopic dermatitis skin is characterized by broad terminal differentiation defects and variable immune abnormalities.	J Allergy Clin Immunol.
atopic dermatitis	WWP2	Expression profiling by array, count, 3 development stage, 2 disease state, 11 individual sets	GPL570	GSE32924 Samples	Nonlesional atopic dermatitis skin is characterized by broad terminal differentiation defects and variable immune abnormalities.	J Allergy Clin Immunol.
atopic dermatitis	ZNF444	Expression profiling by array, count, 3 development stage, 2 disease state, 11 individual sets	GPL570	GSE32924 Samples	Nonlesional atopic dermatitis skin is characterized by broad terminal differentiation defects and variable immune abnormalities.	J Allergy Clin Immunol.
atopic dermatitis	SLC25A23	Expression profiling by array, count, 3 development stage, 2 disease state, 11 individual sets	GPL570	GSE32924 Samples	Nonlesional atopic dermatitis skin is characterized by broad terminal differentiation defects and variable immune abnormalities.	J Allergy Clin Immunol.
atopic dermatitis	TESK1	Expression profiling by array, count, 3 development stage, 2 disease state, 11 individual sets	GPL570	GSE32924 Samples	Nonlesional atopic dermatitis skin is characterized by broad terminal differentiation defects and variable immune abnormalities.	J Allergy Clin Immunol.

atopic dermatitis	TFEB	Expression profiling by array, count, 3 development stage, 2 disease state, 11 individual sets	GPL570	GSE32924 Samples	Nonlesional atopic dermatitis skin is characterized by broad terminal differentiation defects and variable immune abnormalities.	J Allergy Clin Immunol.
atopic dermatitis	RNF145	Expression profiling by array, count, 3 development stage, 2 disease state, 11 individual sets	GPL570	GSE32924 Samples	Nonlesional atopic dermatitis skin is characterized by broad terminal differentiation defects and variable immune abnormalities.	J Allergy Clin Immunol.
atopic dermatitis	NDE1	Expression profiling by array, count, 3 development stage, 2 disease state, 11 individual sets	GPL570	GSE32924 Samples	Nonlesional atopic dermatitis skin is characterized by broad terminal differentiation defects and variable immune abnormalities.	J Allergy Clin Immunol.
atopic dermatitis	ATG16L2	Expression profiling by array, count, 3 development stage, 2 disease state, 11 individual sets	GPL570	GSE32924 Samples	Nonlesional atopic dermatitis skin is characterized by broad terminal differentiation defects and variable immune abnormalities.	J Allergy Clin Immunol.
atopic dermatitis	MST1R	Expression profiling by array, count, 3 development stage, 2 disease state, 11 individual sets	GPL570	GSE32924 Samples	Nonlesional atopic dermatitis skin is characterized by broad terminal differentiation defects and variable immune abnormalities.	J Allergy Clin Immunol.
atopic dermatitis	TRIM13	Expression profiling by array, count, 3 development stage, 2 disease state, 11 individual sets	GPL570	GSE32924 Samples	Nonlesional atopic dermatitis skin is characterized by broad terminal differentiation defects and variable immune abnormalities.	J Allergy Clin Immunol.
atopic dermatitis	MALAT1	Expression profiling by array, count, 3 development stage, 2 disease state, 11 individual sets	GPL570	GSE32924 Samples	Nonlesional atopic dermatitis skin is characterized by broad terminal differentiation defects and variable immune abnormalities.	J Allergy Clin Immunol.
atopic dermatitis	TMED4	Expression profiling by array, count, 3 development stage, 2 disease state, 11 individual sets	GPL570	GSE32924 Samples	Nonlesional atopic dermatitis skin is characterized by broad terminal differentiation defects and variable immune abnormalities.	J Allergy Clin Immunol.
atopic dermatitis	NME4	Expression profiling by array, count, 3 development stage, 2 disease state, 11 individual sets	GPL570	GSE32924 Samples	Nonlesional atopic dermatitis skin is characterized by broad terminal differentiation defects and variable immune abnormalities.	J Allergy Clin Immunol.
atopic dermatitis	ATP2A3	Expression profiling by array, count, 3 development stage, 2 disease state, 11 individual sets	GPL570	GSE32924 Samples	Nonlesional atopic dermatitis skin is characterized by broad terminal differentiation defects and variable immune abnormalities.	J Allergy Clin Immunol.
atopic dermatitis	KCNN4	Expression profiling by array, count, 3 development stage, 2 disease state, 11 individual sets	GPL570	GSE32924 Samples	Nonlesional atopic dermatitis skin is characterized by broad terminal differentiation defects and variable immune abnormalities.	J Allergy Clin Immunol.
atopic dermatitis	ACY1	Expression profiling by array, count, 3 development stage, 2 disease state, 11 individual sets	GPL570	GSE32924 Samples	Nonlesional atopic dermatitis skin is characterized by broad terminal differentiation defects and variable immune abnormalities.	J Allergy Clin Immunol.
atopic dermatitis	NA	Expression profiling by array, count, 3 development stage, 2 disease state, 11 individual sets	GPL570	GSE32924 Samples	Nonlesional atopic dermatitis skin is characterized by broad terminal differentiation defects and variable immune abnormalities.	J Allergy Clin Immunol.
atopic dermatitis	PHLDA1	Expression profiling by array, count, 3 development stage, 2 disease state, 11 individual sets	GPL570	GSE32924 Samples	Nonlesional atopic dermatitis skin is characterized by broad terminal differentiation defects and variable immune abnormalities.	J Allergy Clin Immunol.
atopic dermatitis	INF2	Expression profiling by array, count, 3 development stage, 2 disease state, 11 individual sets	GPL570	GSE32924 Samples	Nonlesional atopic dermatitis skin is characterized by broad terminal differentiation defects and variable immune abnormalities.	J Allergy Clin Immunol.
atopic dermatitis	RTN2	Expression profiling by array, count, 3 development stage, 2 disease state, 11 individual sets	GPL570	GSE32924 Samples	Nonlesional atopic dermatitis skin is characterized by broad terminal differentiation defects and variable immune abnormalities.	J Allergy Clin Immunol.
atopic dermatitis	LRFN4	Expression profiling by array, count, 3 development stage, 2 disease state, 11 individual sets	GPL570	GSE32924 Samples	Nonlesional atopic dermatitis skin is characterized by broad terminal differentiation defects and variable immune abnormalities.	J Allergy Clin Immunol.
atopic dermatitis	LMF1	Expression profiling by array, count, 3 development stage, 2 disease state, 11 individual sets	GPL570	GSE32924 Samples	Nonlesional atopic dermatitis skin is characterized by broad terminal differentiation defects and variable immune abnormalities.	J Allergy Clin Immunol.
atopic dermatitis	RSBN1L	Expression profiling by array, count, 3 development stage, 2 disease state, 11 individual sets	GPL570	GSE32924 Samples	Nonlesional atopic dermatitis skin is characterized by broad terminal differentiation defects and variable immune abnormalities.	J Allergy Clin Immunol.
atopic dermatitis	PELI3	Expression profiling by array, count, 3 development stage, 2 disease state, 11 individual sets	GPL570	GSE32924 Samples	Nonlesional atopic dermatitis skin is characterized by broad terminal differentiation defects and variable immune abnormalities.	J Allergy Clin Immunol.
atopic dermatitis	SF3A2	Expression profiling by array, count, 3 development stage, 2 disease state, 11 individual sets	GPL570	GSE32924 Samples	Nonlesional atopic dermatitis skin is characterized by broad terminal differentiation defects and variable immune abnormalities.	J Allergy Clin Immunol.
atopic dermatitis	NA	Expression profiling by array, count, 3 development stage, 2 disease state, 11 individual sets	GPL570	GSE32924 Samples	Nonlesional atopic dermatitis skin is characterized by broad terminal differentiation defects and variable immune abnormalities.	J Allergy Clin Immunol.
atopic dermatitis	NA	Expression profiling by array, count, 3 development stage, 2 disease state, 11 individual sets	GPL570	GSE32924 Samples	Nonlesional atopic dermatitis skin is characterized by broad terminal differentiation defects and variable immune abnormalities.	J Allergy Clin Immunol.
atopic dermatitis	FNDC3B	Expression profiling by array, count, 3 development stage, 2 disease state, 11 individual sets	GPL570	GSE32924 Samples	Nonlesional atopic dermatitis skin is characterized by broad terminal differentiation defects and variable immune abnormalities.	J Allergy Clin Immunol.
atopic dermatitis	RER1	Expression profiling by array, count, 3 development stage, 2 disease state, 11 individual sets	GPL570	GSE32924 Samples	Nonlesional atopic dermatitis skin is characterized by broad terminal differentiation defects and variable immune abnormalities.	J Allergy Clin Immunol.
atopic dermatitis	ZNF692	Expression profiling by array, count, 3 development stage, 2 disease state, 11 individual sets	GPL570	GSE32924 Samples	Nonlesional atopic dermatitis skin is characterized by broad terminal differentiation defects and variable immune abnormalities.	J Allergy Clin Immunol.
atopic dermatitis	MAN2C1	Expression profiling by array, count, 3 development stage, 2 disease state, 11 individual sets	GPL570	GSE32924 Samples	Nonlesional atopic dermatitis skin is characterized by broad terminal differentiation defects and variable immune abnormalities.	J Allergy Clin Immunol.
atopic dermatitis	RP3-402G11.5	Expression profiling by array, count, 3 development stage, 2 disease state, 11 individual sets	GPL570	GSE32924 Samples	Nonlesional atopic dermatitis skin is characterized by broad terminal differentiation defects and variable immune abnormalities.	J Allergy Clin Immunol.
atopic dermatitis	POMGNT1	Expression profiling by array, count, 3 development stage, 2 disease state, 11 individual sets	GPL570	GSE32924 Samples	Nonlesional atopic dermatitis skin is characterized by broad terminal differentiation defects and variable immune abnormalities.	J Allergy Clin Immunol.
atopic dermatitis	NUCKS1	Expression profiling by array, count, 3 development stage, 2 disease state, 11 individual sets	GPL570	GSE32924 Samples	Nonlesional atopic dermatitis skin is characterized by broad terminal differentiation defects and variable immune abnormalities.	J Allergy Clin Immunol.
atopic dermatitis	BAT2	Expression profiling by array, count, 3 development stage, 2 disease state, 11 individual sets	GPL570	GSE32924 Samples	Nonlesional atopic dermatitis skin is characterized by broad terminal differentiation defects and variable immune abnormalities.	J Allergy Clin Immunol.
atopic dermatitis	PLVAP	Expression profiling by array, count, 3 development stage, 2 disease state, 11 individual sets	GPL570	GSE32924 Samples	Nonlesional atopic dermatitis skin is characterized by broad terminal differentiation defects and variable immune abnormalities.	J Allergy Clin Immunol.
atopic dermatitis	CTSD	Expression profiling by array, count, 3 development stage, 2 disease state, 11 individual sets	GPL570	GSE32924 Samples	Nonlesional atopic dermatitis skin is characterized by broad terminal differentiation defects and variable immune abnormalities.	J Allergy Clin Immunol.
atopic dermatitis	CYBA	Expression profiling by array, count, 3 development stage, 2 disease state, 11 individual sets	GPL570	GSE32924 Samples	Nonlesional atopic dermatitis skin is characterized by broad terminal differentiation defects and variable immune abnormalities.	J Allergy Clin Immunol.
atopic dermatitis	C19ORF22	Expression profiling by array, count, 3 development stage, 2 disease state, 11 individual sets	GPL570	GSE32924 Samples	Nonlesional atopic dermatitis skin is characterized by broad terminal differentiation defects and variable immune abnormalities.	J Allergy Clin Immunol.
atopic dermatitis	ZG16B	Expression profiling by array, count, 3 development stage, 2 disease state, 11 individual sets	GPL570	GSE32924 Samples	Nonlesional atopic dermatitis skin is characterized by broad terminal differentiation defects and variable immune abnormalities.	J Allergy Clin Immunol.
atopic dermatitis	C16ORF42	Expression profiling by array, count, 3 development stage, 2 disease state, 11 individual sets	GPL570	GSE32924 Samples	Nonlesional atopic dermatitis skin is characterized by broad terminal differentiation defects and variable immune abnormalities.	J Allergy Clin Immunol.
atopic dermatitis	STRA13	Expression profiling by array, count, 3 development stage, 2 disease state, 11 individual sets	GPL570	GSE32924 Samples	Nonlesional atopic dermatitis skin is characterized by broad terminal differentiation defects and variable immune abnormalities.	J Allergy Clin Immunol.
atopic dermatitis	H2AFX	Expression profiling by array, count, 3 development stage, 2 disease state, 11 individual sets	GPL570	GSE32924 Samples	Nonlesional atopic dermatitis skin is characterized by broad terminal differentiation defects and variable immune abnormalities.	J Allergy Clin Immunol.
atopic dermatitis	TYMP	Expression profiling by array, count, 3 development stage, 2 disease state, 11 individual sets	GPL570	GSE32924 Samples	Nonlesional atopic dermatitis skin is characterized by broad terminal differentiation defects and variable immune abnormalities.	J Allergy Clin Immunol.
atopic dermatitis	SCO2	Expression profiling by array, count, 3 development stage, 2 disease state, 11 individual sets	GPL570	GSE32924 Samples	Nonlesional atopic dermatitis skin is characterized by broad terminal differentiation defects and variable immune abnormalities.	J Allergy Clin Immunol.

atopic dermatitis	EHF	Expression profiling by array, count, 3 development stage, 2 disease state, 11 individual sets	GPL570	GSE32924 Samples	Nonlesional atopic dermatitis skin is characterized by broad terminal differentiation defects and variable immune abnormalities.	J Allergy Clin Immunol.
atopic dermatitis	PSMB10	Expression profiling by array, count, 3 development stage, 2 disease state, 11 individual sets	GPL570	GSE32924 Samples	Nonlesional atopic dermatitis skin is characterized by broad terminal differentiation defects and variable immune abnormalities.	J Allergy Clin Immunol.
atopic dermatitis	WNT5A	Expression profiling by array, count, 3 development stage, 2 disease state, 11 individual sets	GPL570	GSE32924 Samples	Nonlesional atopic dermatitis skin is characterized by broad terminal differentiation defects and variable immune abnormalities.	J Allergy Clin Immunol.
atopic dermatitis	F12	Expression profiling by array, count, 3 development stage, 2 disease state, 11 individual sets	GPL570	GSE32924 Samples	Nonlesional atopic dermatitis skin is characterized by broad terminal differentiation defects and variable immune abnormalities.	J Allergy Clin Immunol.
atopic dermatitis	NOSIP	Expression profiling by array, count, 3 development stage, 2 disease state, 11 individual sets	GPL570	GSE32924 Samples	Nonlesional atopic dermatitis skin is characterized by broad terminal differentiation defects and variable immune abnormalities.	J Allergy Clin Immunol.
atopic dermatitis	CDC42EP5	Expression profiling by array, count, 3 development stage, 2 disease state, 11 individual sets	GPL570	GSE32924 Samples	Nonlesional atopic dermatitis skin is characterized by broad terminal differentiation defects and variable immune abnormalities.	J Allergy Clin Immunol.
atopic dermatitis	COL5A3	Expression profiling by array, count, 3 development stage, 2 disease state, 11 individual sets	GPL570	GSE32924 Samples	Nonlesional atopic dermatitis skin is characterized by broad terminal differentiation defects and variable immune abnormalities.	J Allergy Clin Immunol.
atopic dermatitis	ZBTB7B	Expression profiling by array, count, 3 development stage, 2 disease state, 11 individual sets	GPL570	GSE32924 Samples	Nonlesional atopic dermatitis skin is characterized by broad terminal differentiation defects and variable immune abnormalities.	J Allergy Clin Immunol.
atopic dermatitis	LTB4R	Expression profiling by array, count, 3 development stage, 2 disease state, 11 individual sets	GPL570	GSE32924 Samples	Nonlesional atopic dermatitis skin is characterized by broad terminal differentiation defects and variable immune abnormalities.	J Allergy Clin Immunol.
atopic dermatitis	KIFC2	Expression profiling by array, count, 3 development stage, 2 disease state, 11 individual sets	GPL570	GSE32924 Samples	Nonlesional atopic dermatitis skin is characterized by broad terminal differentiation defects and variable immune abnormalities.	J Allergy Clin Immunol.
atopic dermatitis	MZF1	Expression profiling by array, count, 3 development stage, 2 disease state, 11 individual sets	GPL570	GSE32924 Samples	Nonlesional atopic dermatitis skin is characterized by broad terminal differentiation defects and variable immune abnormalities.	J Allergy Clin Immunol.
atopic dermatitis	RUNX3	Expression profiling by array, count, 3 development stage, 2 disease state, 11 individual sets	GPL570	GSE32924 Samples	Nonlesional atopic dermatitis skin is characterized by broad terminal differentiation defects and variable immune abnormalities.	J Allergy Clin Immunol.
atopic dermatitis	PDXK	Expression profiling by array, count, 3 development stage, 2 disease state, 11 individual sets	GPL570	GSE32924 Samples	Nonlesional atopic dermatitis skin is characterized by broad terminal differentiation defects and variable immune abnormalities.	J Allergy Clin Immunol.
atopic dermatitis	SATB1	Expression profiling by array, count, 3 development stage, 2 disease state, 11 individual sets	GPL570	GSE32924 Samples	Nonlesional atopic dermatitis skin is characterized by broad terminal differentiation defects and variable immune abnormalities.	J Allergy Clin Immunol.
atopic dermatitis	LUC7L	Expression profiling by array, count, 3 development stage, 2 disease state, 11 individual sets	GPL570	GSE32924 Samples	Nonlesional atopic dermatitis skin is characterized by broad terminal differentiation defects and variable immune abnormalities.	J Allergy Clin Immunol.
atopic dermatitis	CTBP2	Expression profiling by array, count, 3 development stage, 2 disease state, 11 individual sets	GPL570	GSE32924 Samples	Nonlesional atopic dermatitis skin is characterized by broad terminal differentiation defects and variable immune abnormalities.	J Allergy Clin Immunol.
atopic dermatitis	NA	Expression profiling by array, count, 3 development stage, 2 disease state, 11 individual sets	GPL570	GSE32924 Samples	Nonlesional atopic dermatitis skin is characterized by broad terminal differentiation defects and variable immune abnormalities.	J Allergy Clin Immunol.
atopic dermatitis	AKAP13	Expression profiling by array, count, 3 development stage, 2 disease state, 11 individual sets	GPL570	GSE32924 Samples	Nonlesional atopic dermatitis skin is characterized by broad terminal differentiation defects and variable immune abnormalities.	J Allergy Clin Immunol.
atopic dermatitis	WASH3P	Expression profiling by array, count, 3 development stage, 2 disease state, 11 individual sets	GPL570	GSE32924 Samples	Nonlesional atopic dermatitis skin is characterized by broad terminal differentiation defects and variable immune abnormalities.	J Allergy Clin Immunol.
atopic dermatitis	AURKAIP1	Expression profiling by array, count, 3 development stage, 2 disease state, 11 individual sets	GPL570	GSE32924 Samples	Nonlesional atopic dermatitis skin is characterized by broad terminal differentiation defects and variable immune abnormalities.	J Allergy Clin Immunol.
atopic dermatitis	ANPEP	Expression profiling by array, count, 3 development stage, 2 disease state, 11 individual sets	GPL570	GSE32924 Samples	Nonlesional atopic dermatitis skin is characterized by broad terminal differentiation defects and variable immune abnormalities.	J Allergy Clin Immunol.
atopic dermatitis	CARD10	Expression profiling by array, count, 3 development stage, 2 disease state, 11 individual sets	GPL570	GSE32924 Samples	Nonlesional atopic dermatitis skin is characterized by broad terminal differentiation defects and variable immune abnormalities.	J Allergy Clin Immunol.
atopic dermatitis	SEC61A1	Expression profiling by array, count, 3 development stage, 2 disease state, 11 individual sets	GPL570	GSE32924 Samples	Nonlesional atopic dermatitis skin is characterized by broad terminal differentiation defects and variable immune abnormalities.	J Allergy Clin Immunol.
atopic dermatitis	FMNL2	Expression profiling by array, count, 3 development stage, 2 disease state, 11 individual sets	GPL570	GSE32924 Samples	Nonlesional atopic dermatitis skin is characterized by broad terminal differentiation defects and variable immune abnormalities.	J Allergy Clin Immunol.
atopic dermatitis	BCS1L	Expression profiling by array, count, 3 development stage, 2 disease state, 11 individual sets	GPL570	GSE32924 Samples	Nonlesional atopic dermatitis skin is characterized by broad terminal differentiation defects and variable immune abnormalities.	J Allergy Clin Immunol.
atopic dermatitis	MED16	Expression profiling by array, count, 3 development stage, 2 disease state, 11 individual sets	GPL570	GSE32924 Samples	Nonlesional atopic dermatitis skin is characterized by broad terminal differentiation defects and variable immune abnormalities.	J Allergy Clin Immunol.
atopic dermatitis	MGC2752	Expression profiling by array, count, 3 development stage, 2 disease state, 11 individual sets	GPL570	GSE32924 Samples	Nonlesional atopic dermatitis skin is characterized by broad terminal differentiation defects and variable immune abnormalities.	J Allergy Clin Immunol.
atopic dermatitis	NDUFS7	Expression profiling by array, count, 3 development stage, 2 disease state, 11 individual sets	GPL570	GSE32924 Samples	Nonlesional atopic dermatitis skin is characterized by broad terminal differentiation defects and variable immune abnormalities.	J Allergy Clin Immunol.
atopic dermatitis	AK1	Expression profiling by array, count, 3 development stage, 2 disease state, 11 individual sets	GPL570	GSE32924 Samples	Nonlesional atopic dermatitis skin is characterized by broad terminal differentiation defects and variable immune abnormalities.	J Allergy Clin Immunol.
atopic dermatitis	RUNX1	Expression profiling by array, count, 3 development stage, 2 disease state, 11 individual sets	GPL570	GSE32924 Samples	Nonlesional atopic dermatitis skin is characterized by broad terminal differentiation defects and variable immune abnormalities.	J Allergy Clin Immunol.
atopic dermatitis	NADSYN1	Expression profiling by array, count, 3 development stage, 2 disease state, 11 individual sets	GPL570	GSE32924 Samples	Nonlesional atopic dermatitis skin is characterized by broad terminal differentiation defects and variable immune abnormalities.	J Allergy Clin Immunol.
atopic dermatitis	SREBF1	Expression profiling by array, count, 3 development stage, 2 disease state, 11 individual sets	GPL570	GSE32924 Samples	Nonlesional atopic dermatitis skin is characterized by broad terminal differentiation defects and variable immune abnormalities.	J Allergy Clin Immunol.
atopic dermatitis	SLC4A11	Expression profiling by array, count, 3 development stage, 2 disease state, 11 individual sets	GPL570	GSE32924 Samples	Nonlesional atopic dermatitis skin is characterized by broad terminal differentiation defects and variable immune abnormalities.	J Allergy Clin Immunol.
atopic dermatitis	NA	Expression profiling by array, count, 3 development stage, 2 disease state, 11 individual sets	GPL570	GSE32924 Samples	Nonlesional atopic dermatitis skin is characterized by broad terminal differentiation defects and variable immune abnormalities.	J Allergy Clin Immunol.
atopic dermatitis	SMARCA4	Expression profiling by array, count, 3 development stage, 2 disease state, 11 individual sets	GPL570	GSE32924 Samples	Nonlesional atopic dermatitis skin is characterized by broad terminal differentiation defects and variable immune abnormalities.	J Allergy Clin Immunol.
atopic dermatitis	SF3A2	Expression profiling by array, count, 3 development stage, 2 disease state, 11 individual sets	GPL570	GSE32924 Samples	Nonlesional atopic dermatitis skin is characterized by broad terminal differentiation defects and variable immune abnormalities.	J Allergy Clin Immunol.
atopic dermatitis	SCAND1	Expression profiling by array, count, 3 development stage, 2 disease state, 11 individual sets	GPL570	GSE32924 Samples	Nonlesional atopic dermatitis skin is characterized by broad terminal differentiation defects and variable immune abnormalities.	J Allergy Clin Immunol.
atopic dermatitis	SCAND1	Expression profiling by array, count, 3 development stage, 2 disease state, 11 individual sets	GPL570	GSE32924 Samples	Nonlesional atopic dermatitis skin is characterized by broad terminal differentiation defects and variable immune abnormalities.	J Allergy Clin Immunol.
atopic dermatitis	CLCN7	Expression profiling by array, count, 3 development stage, 2 disease state, 11 individual sets	GPL570	GSE32924 Samples	Nonlesional atopic dermatitis skin is characterized by broad terminal differentiation defects and variable immune abnormalities.	J Allergy Clin Immunol.
atopic dermatitis	HGS	Expression profiling by array, count, 3 development stage, 2 disease state, 11 individual sets	GPL570	GSE32924 Samples	Nonlesional atopic dermatitis skin is characterized by broad terminal differentiation defects and variable immune abnormalities.	J Allergy Clin Immunol.
atopic dermatitis	RABEP2	Expression profiling by array, count, 3 development stage, 2 disease state, 11 individual sets	GPL570	GSE32924 Samples	Nonlesional atopic dermatitis skin is characterized by broad terminal differentiation defects and variable immune abnormalities.	J Allergy Clin Immunol.

atopic dermatitis	RP3-402G11.5	Expression profiling by array, count, 3 development stage, 2 disease state, 11 individual sets	GPL570	GSE32924 Samples	Nonlesional atopic dermatitis skin is characterized by broad terminal differentiation defects and variable immune abnormalities.	J Allergy Clin Immunol.
atopic dermatitis	IRF3	Expression profiling by array, count, 3 development stage, 2 disease state, 11 individual sets	GPL570	GSE32924 Samples	Nonlesional atopic dermatitis skin is characterized by broad terminal differentiation defects and variable immune abnormalities.	J Allergy Clin Immunol.
atopic dermatitis	NA	Expression profiling by array, count, 3 development stage, 2 disease state, 11 individual sets	GPL570	GSE32924 Samples	Nonlesional atopic dermatitis skin is characterized by broad terminal differentiation defects and variable immune abnormalities.	J Allergy Clin Immunol.
atopic dermatitis	MED13	Expression profiling by array, count, 3 development stage, 2 disease state, 11 individual sets	GPL570	GSE32924 Samples	Nonlesional atopic dermatitis skin is characterized by broad terminal differentiation defects and variable immune abnormalities.	J Allergy Clin Immunol.
atopic dermatitis	EDEM3	Expression profiling by array, count, 3 development stage, 2 disease state, 11 individual sets	GPL570	GSE32924 Samples	Nonlesional atopic dermatitis skin is characterized by broad terminal differentiation defects and variable immune abnormalities.	J Allergy Clin Immunol.
atopic dermatitis	ECHDC3	Expression profiling by array, count, 3 development stage, 2 disease state, 11 individual sets	GPL570	GSE32924 Samples	Nonlesional atopic dermatitis skin is characterized by broad terminal differentiation defects and variable immune abnormalities.	J Allergy Clin Immunol.
atopic dermatitis	ALKBH7	Expression profiling by array, count, 3 development stage, 2 disease state, 11 individual sets	GPL570	GSE32924 Samples	Nonlesional atopic dermatitis skin is characterized by broad terminal differentiation defects and variable immune abnormalities.	J Allergy Clin Immunol.
atopic dermatitis	EHD2	Expression profiling by array, count, 3 development stage, 2 disease state, 11 individual sets	GPL570	GSE32924 Samples	Nonlesional atopic dermatitis skin is characterized by broad terminal differentiation defects and variable immune abnormalities.	J Allergy Clin Immunol.
atopic dermatitis	EHD2	Expression profiling by array, count, 3 development stage, 2 disease state, 11 individual sets	GPL570	GSE32924 Samples	Nonlesional atopic dermatitis skin is characterized by broad terminal differentiation defects and variable immune abnormalities.	J Allergy Clin Immunol.
atopic dermatitis	RBM47	Expression profiling by array, count, 3 development stage, 2 disease state, 11 individual sets	GPL570	GSE32924 Samples	Nonlesional atopic dermatitis skin is characterized by broad terminal differentiation defects and variable immune abnormalities.	J Allergy Clin Immunol.
atopic dermatitis	ILVBL	Expression profiling by array, count, 3 development stage, 2 disease state, 11 individual sets	GPL570	GSE32924 Samples	Nonlesional atopic dermatitis skin is characterized by broad terminal differentiation defects and variable immune abnormalities.	J Allergy Clin Immunol.
atopic dermatitis	MAP2K7	Expression profiling by array, count, 3 development stage, 2 disease state, 11 individual sets	GPL570	GSE32924 Samples	Nonlesional atopic dermatitis skin is characterized by broad terminal differentiation defects and variable immune abnormalities.	J Allergy Clin Immunol.
atopic dermatitis	MSI2	Expression profiling by array, count, 3 development stage, 2 disease state, 11 individual sets	GPL570	GSE32924 Samples	Nonlesional atopic dermatitis skin is characterized by broad terminal differentiation defects and variable immune abnormalities.	J Allergy Clin Immunol.
atopic dermatitis	NA	Expression profiling by array, count, 3 development stage, 2 disease state, 11 individual sets	GPL570	GSE32924 Samples	Nonlesional atopic dermatitis skin is characterized by broad terminal differentiation defects and variable immune abnormalities.	J Allergy Clin Immunol.
atopic dermatitis	SCRN2	Expression profiling by array, count, 3 development stage, 2 disease state, 11 individual sets	GPL570	GSE32924 Samples	Nonlesional atopic dermatitis skin is characterized by broad terminal differentiation defects and variable immune abnormalities.	J Allergy Clin Immunol.
atopic dermatitis	CPSF3L	Expression profiling by array, count, 3 development stage, 2 disease state, 11 individual sets	GPL570	GSE32924 Samples	Nonlesional atopic dermatitis skin is characterized by broad terminal differentiation defects and variable immune abnormalities.	J Allergy Clin Immunol.
atopic dermatitis	CAPN1	Expression profiling by array, count, 3 development stage, 2 disease state, 11 individual sets	GPL570	GSE32924 Samples	Nonlesional atopic dermatitis skin is characterized by broad terminal differentiation defects and variable immune abnormalities.	J Allergy Clin Immunol.
atopic dermatitis	BAIAP2	Expression profiling by array, count, 3 development stage, 2 disease state, 11 individual sets	GPL570	GSE32924 Samples	Nonlesional atopic dermatitis skin is characterized by broad terminal differentiation defects and variable immune abnormalities.	J Allergy Clin Immunol.
atopic dermatitis	C19ORF6	Expression profiling by array, count, 3 development stage, 2 disease state, 11 individual sets	GPL570	GSE32924 Samples	Nonlesional atopic dermatitis skin is characterized by broad terminal differentiation defects and variable immune abnormalities.	J Allergy Clin Immunol.
atopic dermatitis	NA	Expression profiling by array, count, 3 development stage, 2 disease state, 11 individual sets	GPL570	GSE32924 Samples	Nonlesional atopic dermatitis skin is characterized by broad terminal differentiation defects and variable immune abnormalities.	J Allergy Clin Immunol.
atopic dermatitis	NA	Expression profiling by array, count, 3 development stage, 2 disease state, 11 individual sets	GPL570	GSE32924 Samples	Nonlesional atopic dermatitis skin is characterized by broad terminal differentiation defects and variable immune abnormalities.	J Allergy Clin Immunol.
atopic dermatitis	PLEKHB2	Expression profiling by array, count, 3 development stage, 2 disease state, 11 individual sets	GPL570	GSE32924 Samples	Nonlesional atopic dermatitis skin is characterized by broad terminal differentiation defects and variable immune abnormalities.	J Allergy Clin Immunol.
atopic dermatitis	NA	Expression profiling by array, count, 3 development stage, 2 disease state, 11 individual sets	GPL570	GSE32924 Samples	Nonlesional atopic dermatitis skin is characterized by broad terminal differentiation defects and variable immune abnormalities.	J Allergy Clin Immunol.
atopic dermatitis	ERAP1	Expression profiling by array, count, 3 development stage, 2 disease state, 11 individual sets	GPL570	GSE32924 Samples	Nonlesional atopic dermatitis skin is characterized by broad terminal differentiation defects and variable immune abnormalities.	J Allergy Clin Immunol.
atopic dermatitis	HNRNPF	Expression profiling by array, count, 3 development stage, 2 disease state, 11 individual sets	GPL570	GSE32924 Samples	Nonlesional atopic dermatitis skin is characterized by broad terminal differentiation defects and variable immune abnormalities.	J Allergy Clin Immunol.
atopic dermatitis	SRP72	Expression profiling by array, count, 3 development stage, 2 disease state, 11 individual sets	GPL570	GSE32924 Samples	Nonlesional atopic dermatitis skin is characterized by broad terminal differentiation defects and variable immune abnormalities.	J Allergy Clin Immunol.
atopic dermatitis	APPL2	Expression profiling by array, count, 3 development stage, 2 disease state, 11 individual sets	GPL570	GSE32924 Samples	Nonlesional atopic dermatitis skin is characterized by broad terminal differentiation defects and variable immune abnormalities.	J Allergy Clin Immunol.
atopic dermatitis	NA	Expression profiling by array, count, 3 development stage, 2 disease state, 11 individual sets	GPL570	GSE32924 Samples	Nonlesional atopic dermatitis skin is characterized by broad terminal differentiation defects and variable immune abnormalities.	J Allergy Clin Immunol.
atopic dermatitis	FAM120A	Expression profiling by array, count, 3 development stage, 2 disease state, 11 individual sets	GPL570	GSE32924 Samples	Nonlesional atopic dermatitis skin is characterized by broad terminal differentiation defects and variable immune abnormalities.	J Allergy Clin Immunol.
atopic dermatitis	SLC39A6	Expression profiling by array, count, 3 development stage, 2 disease state, 11 individual sets	GPL570	GSE32924 Samples	Nonlesional atopic dermatitis skin is characterized by broad terminal differentiation defects and variable immune abnormalities.	J Allergy Clin Immunol.
atopic dermatitis	BICD2	Expression profiling by array, count, 3 development stage, 2 disease state, 11 individual sets	GPL570	GSE32924 Samples	Nonlesional atopic dermatitis skin is characterized by broad terminal differentiation defects and variable immune abnormalities.	J Allergy Clin Immunol.
atopic dermatitis	CHIC2	Expression profiling by array, count, 3 development stage, 2 disease state, 11 individual sets	GPL570	GSE32924 Samples	Nonlesional atopic dermatitis skin is characterized by broad terminal differentiation defects and variable immune abnormalities.	J Allergy Clin Immunol.
atopic dermatitis	CSNK1A1	Expression profiling by array, count, 3 development stage, 2 disease state, 11 individual sets	GPL570	GSE32924 Samples	Nonlesional atopic dermatitis skin is characterized by broad terminal differentiation defects and variable immune abnormalities.	J Allergy Clin Immunol.
atopic dermatitis	GNAI3	Expression profiling by array, count, 3 development stage, 2 disease state, 11 individual sets	GPL570	GSE32924 Samples	Nonlesional atopic dermatitis skin is characterized by broad terminal differentiation defects and variable immune abnormalities.	J Allergy Clin Immunol.
atopic dermatitis	TPD52	Expression profiling by array, count, 3 development stage, 2 disease state, 11 individual sets	GPL570	GSE32924 Samples	Nonlesional atopic dermatitis skin is characterized by broad terminal differentiation defects and variable immune abnormalities.	J Allergy Clin Immunol.
atopic dermatitis	ESRP1	Expression profiling by array, count, 3 development stage, 2 disease state, 11 individual sets	GPL570	GSE32924 Samples	Nonlesional atopic dermatitis skin is characterized by broad terminal differentiation defects and variable immune abnormalities.	J Allergy Clin Immunol.
atopic dermatitis	CCDC90A	Expression profiling by array, count, 3 development stage, 2 disease state, 11 individual sets	GPL570	GSE32924 Samples	Nonlesional atopic dermatitis skin is characterized by broad terminal differentiation defects and variable immune abnormalities.	J Allergy Clin Immunol.
atopic dermatitis	BACH1	Expression profiling by array, count, 3 development stage, 2 disease state, 11 individual sets	GPL570	GSE32924 Samples	Nonlesional atopic dermatitis skin is characterized by broad terminal differentiation defects and variable immune abnormalities.	J Allergy Clin Immunol.
atopic dermatitis	SRGAP2P1	Expression profiling by array, count, 3 development stage, 2 disease state, 11 individual sets	GPL570	GSE32924 Samples	Nonlesional atopic dermatitis skin is characterized by broad terminal differentiation defects and variable immune abnormalities.	J Allergy Clin Immunol.
atopic dermatitis	HPGD	Expression profiling by array, count, 3 development stage, 2 disease state, 11 individual sets	GPL570	GSE32924 Samples	Nonlesional atopic dermatitis skin is characterized by broad terminal differentiation defects and variable immune abnormalities.	J Allergy Clin Immunol.
atopic dermatitis	HPGD	Expression profiling by array, count, 3 development stage, 2 disease state, 11 individual sets	GPL570	GSE32924 Samples	Nonlesional atopic dermatitis skin is characterized by broad terminal differentiation defects and variable immune abnormalities.	J Allergy Clin Immunol.

atopic dermatitis	HPGD	Expression profiling by array, count, 3 development stage, 2 disease state, 11 individual sets	GPL570	GSE32924 Samples	Nonlesional atopic dermatitis skin is characterized by broad terminal differentiation defects and variable immune abnormalities.	J Allergy Clin Immunol.
atopic dermatitis	RORA	Expression profiling by array, count, 3 development stage, 2 disease state, 11 individual sets	GPL570	GSE32924 Samples	Nonlesional atopic dermatitis skin is characterized by broad terminal differentiation defects and variable immune abnormalities.	J Allergy Clin Immunol.
atopic dermatitis	RAB11FIP1	Expression profiling by array, count, 3 development stage, 2 disease state, 11 individual sets	GPL570	GSE32924 Samples	Nonlesional atopic dermatitis skin is characterized by broad terminal differentiation defects and variable immune abnormalities.	J Allergy Clin Immunol.
atopic dermatitis	RORA	Expression profiling by array, count, 3 development stage, 2 disease state, 11 individual sets	GPL570	GSE32924 Samples	Nonlesional atopic dermatitis skin is characterized by broad terminal differentiation defects and variable immune abnormalities.	J Allergy Clin Immunol.
atopic dermatitis	BPIL2	Expression profiling by array, count, 3 development stage, 2 disease state, 11 individual sets	GPL570	GSE32924 Samples	Nonlesional atopic dermatitis skin is characterized by broad terminal differentiation defects and variable immune abnormalities.	J Allergy Clin Immunol.
atopic dermatitis	38782 MEMBRANE-ASSOCIATED RING FINGER (C3HC4) 7 10.09	Expression profiling by array, count, 3 development stage, 2 disease state, 11 individual sets	GPL570	GSE32924 Samples	Nonlesional atopic dermatitis skin is characterized by broad terminal differentiation defects and variable immune abnormalities.	J Allergy Clin Immunol.
atopic dermatitis	RAB14	Expression profiling by array, count, 3 development stage, 2 disease state, 11 individual sets	GPL570	GSE32924 Samples	Nonlesional atopic dermatitis skin is characterized by broad terminal differentiation defects and variable immune abnormalities.	J Allergy Clin Immunol.
atopic dermatitis	OCIAD1	Expression profiling by array, count, 3 development stage, 2 disease state, 11 individual sets	GPL570	GSE32924 Samples	Nonlesional atopic dermatitis skin is characterized by broad terminal differentiation defects and variable immune abnormalities.	J Allergy Clin Immunol.
atopic dermatitis	RBMS1	Expression profiling by array, count, 3 development stage, 2 disease state, 11 individual sets	GPL570	GSE32924 Samples	Nonlesional atopic dermatitis skin is characterized by broad terminal differentiation defects and variable immune abnormalities.	J Allergy Clin Immunol.
atopic dermatitis	SYPL1	Expression profiling by array, count, 3 development stage, 2 disease state, 11 individual sets	GPL570	GSE32924 Samples	Nonlesional atopic dermatitis skin is characterized by broad terminal differentiation defects and variable immune abnormalities.	J Allergy Clin Immunol.
atopic dermatitis	VAMP3	Expression profiling by array, count, 3 development stage, 2 disease state, 11 individual sets	GPL570	GSE32924 Samples	Nonlesional atopic dermatitis skin is characterized by broad terminal differentiation defects and variable immune abnormalities.	J Allergy Clin Immunol.
atopic dermatitis	POLR2K	Expression profiling by array, count, 3 development stage, 2 disease state, 11 individual sets	GPL570	GSE32924 Samples	Nonlesional atopic dermatitis skin is characterized by broad terminal differentiation defects and variable immune abnormalities.	J Allergy Clin Immunol.
atopic dermatitis	HSP90B1	Expression profiling by array, count, 3 development stage, 2 disease state, 11 individual sets	GPL570	GSE32924 Samples	Nonlesional atopic dermatitis skin is characterized by broad terminal differentiation defects and variable immune abnormalities.	J Allergy Clin Immunol.
atopic dermatitis	LAPTM4B	Expression profiling by array, count, 3 development stage, 2 disease state, 11 individual sets	GPL570	GSE32924 Samples	Nonlesional atopic dermatitis skin is characterized by broad terminal differentiation defects and variable immune abnormalities.	J Allergy Clin Immunol.
atopic dermatitis	OTUD1	Expression profiling by array, count, 3 development stage, 2 disease state, 11 individual sets	GPL570	GSE32924 Samples	Nonlesional atopic dermatitis skin is characterized by broad terminal differentiation defects and variable immune abnormalities.	J Allergy Clin Immunol.
atopic dermatitis	NDFIP2	Expression profiling by array, count, 3 development stage, 2 disease state, 11 individual sets	GPL570	GSE32924 Samples	Nonlesional atopic dermatitis skin is characterized by broad terminal differentiation defects and variable immune abnormalities.	J Allergy Clin Immunol.
atopic dermatitis	JUB	Expression profiling by array, count, 3 development stage, 2 disease state, 11 individual sets	GPL570	GSE32924 Samples	Nonlesional atopic dermatitis skin is characterized by broad terminal differentiation defects and variable immune abnormalities.	J Allergy Clin Immunol.
atopic dermatitis	RIOK3	Expression profiling by array, count, 3 development stage, 2 disease state, 11 individual sets	GPL570	GSE32924 Samples	Nonlesional atopic dermatitis skin is characterized by broad terminal differentiation defects and variable immune abnormalities.	J Allergy Clin Immunol.
atopic dermatitis	COL17A1	Expression profiling by array, count, 3 development stage, 2 disease state, 11 individual sets	GPL570	GSE32924 Samples	Nonlesional atopic dermatitis skin is characterized by broad terminal differentiation defects and variable immune abnormalities.	J Allergy Clin Immunol.
atopic dermatitis	NA	Expression profiling by array, count, 3 development stage, 2 disease state, 11 individual sets	GPL570	GSE32924 Samples	Nonlesional atopic dermatitis skin is characterized by broad terminal differentiation defects and variable immune abnormalities.	J Allergy Clin Immunol.
atopic dermatitis	MUC15	Expression profiling by array, count, 3 development stage, 2 disease state, 11 individual sets	GPL570	GSE32924 Samples	Nonlesional atopic dermatitis skin is characterized by broad terminal differentiation defects and variable immune abnormalities.	J Allergy Clin Immunol.
atopic dermatitis	CSNK2A2	Expression profiling by array, count, 3 development stage, 2 disease state, 11 individual sets	GPL570	GSE32924 Samples	Nonlesional atopic dermatitis skin is characterized by broad terminal differentiation defects and variable immune abnormalities.	J Allergy Clin Immunol.
atopic dermatitis	NA	Expression profiling by array, count, 3 development stage, 2 disease state, 11 individual sets	GPL570	GSE32924 Samples	Nonlesional atopic dermatitis skin is characterized by broad terminal differentiation defects and variable immune abnormalities.	J Allergy Clin Immunol.
atopic dermatitis	GPR115	Expression profiling by array, count, 3 development stage, 2 disease state, 11 individual sets	GPL570	GSE32924 Samples	Nonlesional atopic dermatitis skin is characterized by broad terminal differentiation defects and variable immune abnormalities.	J Allergy Clin Immunol.
atopic dermatitis	NDFIP2	Expression profiling by array, count, 3 development stage, 2 disease state, 11 individual sets	GPL570	GSE32924 Samples	Nonlesional atopic dermatitis skin is characterized by broad terminal differentiation defects and variable immune abnormalities.	J Allergy Clin Immunol.
atopic dermatitis	NA	Expression profiling by array, count, 3 development stage, 2 disease state, 11 individual sets	GPL570	GSE32924 Samples	Nonlesional atopic dermatitis skin is characterized by broad terminal differentiation defects and variable immune abnormalities.	J Allergy Clin Immunol.
atopic dermatitis	IDE	Expression profiling by array, count, 3 development stage, 2 disease state, 11 individual sets	GPL570	GSE32924 Samples	Nonlesional atopic dermatitis skin is characterized by broad terminal differentiation defects and variable immune abnormalities.	J Allergy Clin Immunol.
atopic dermatitis	YOD1	Expression profiling by array, count, 3 development stage, 2 disease state, 11 individual sets	GPL570	GSE32924 Samples	Nonlesional atopic dermatitis skin is characterized by broad terminal differentiation defects and variable immune abnormalities.	J Allergy Clin Immunol.
atopic dermatitis	AADACL2	Expression profiling by array, count, 3 development stage, 2 disease state, 11 individual sets	GPL570	GSE32924 Samples	Nonlesional atopic dermatitis skin is characterized by broad terminal differentiation defects and variable immune abnormalities.	J Allergy Clin Immunol.
atopic dermatitis	RORA	Expression profiling by array, count, 3 development stage, 2 disease state, 11 individual sets	GPL570	GSE32924 Samples	Nonlesional atopic dermatitis skin is characterized by broad terminal differentiation defects and variable immune abnormalities.	J Allergy Clin Immunol.
atopic dermatitis	ELOVL7	Expression profiling by array, count, 3 development stage, 2 disease state, 11 individual sets	GPL570	GSE32924 Samples	Nonlesional atopic dermatitis skin is characterized by broad terminal differentiation defects and variable immune abnormalities.	J Allergy Clin Immunol.
atopic dermatitis	ARL5A	Expression profiling by array, count, 3 development stage, 2 disease state, 11 individual sets	GPL570	GSE32924 Samples	Nonlesional atopic dermatitis skin is characterized by broad terminal differentiation defects and variable immune abnormalities.	J Allergy Clin Immunol.
atopic dermatitis	KRT80	Expression profiling by array, count, 3 development stage, 2 disease state, 11 individual sets	GPL570	GSE32924 Samples	Nonlesional atopic dermatitis skin is characterized by broad terminal differentiation defects and variable immune abnormalities.	J Allergy Clin Immunol.
atopic dermatitis	SERPINB2	Expression profiling by array, count, 3 development stage, 2 disease state, 11 individual sets	GPL570	GSE32924 Samples	Nonlesional atopic dermatitis skin is characterized by broad terminal differentiation defects and variable immune abnormalities.	J Allergy Clin Immunol.
atopic dermatitis	XG	Expression profiling by array, count, 3 development stage, 2 disease state, 11 individual sets	GPL570	GSE32924 Samples	Nonlesional atopic dermatitis skin is characterized by broad terminal differentiation defects and variable immune abnormalities.	J Allergy Clin Immunol.
atopic dermatitis	EMB	Expression profiling by array, count, 3 development stage, 2 disease state, 11 individual sets	GPL570	GSE32924 Samples	Nonlesional atopic dermatitis skin is characterized by broad terminal differentiation defects and variable immune abnormalities.	J Allergy Clin Immunol.
atopic dermatitis	BLMH	Expression profiling by array, count, 3 development stage, 2 disease state, 11 individual sets	GPL570	GSE32924 Samples	Nonlesional atopic dermatitis skin is characterized by broad terminal differentiation defects and variable immune abnormalities.	J Allergy Clin Immunol.
atopic dermatitis	CTSL2	Expression profiling by array, count, 3 development stage, 2 disease state, 11 individual sets	GPL570	GSE32924 Samples	Nonlesional atopic dermatitis skin is characterized by broad terminal differentiation defects and variable immune abnormalities.	J Allergy Clin Immunol.
atopic dermatitis	SCEL	Expression profiling by array, count, 3 development stage, 2 disease state, 11 individual sets	GPL570	GSE32924 Samples	Nonlesional atopic dermatitis skin is characterized by broad terminal differentiation defects and variable immune abnormalities.	J Allergy Clin Immunol.
atopic dermatitis	ELMOD1	Expression profiling by array, count, 3 development stage, 2 disease state, 11 individual sets	GPL570	GSE32924 Samples	Nonlesional atopic dermatitis skin is characterized by broad terminal differentiation defects and variable immune abnormalities.	J Allergy Clin Immunol.

atopic dermatitis	C4ORF3	Expression profiling by array, count, 3 development stage, 2 disease state, 11 individual sets	GPL570	GSE32924 Samples	Nonlesional atopic dermatitis skin is characterized by broad terminal differentiation defects and variable immune abnormalities.	J Allergy Clin Immunol.
atopic dermatitis	ARIH1	Expression profiling by array, count, 3 development stage, 2 disease state, 11 individual sets	GPL570	GSE32924 Samples	Nonlesional atopic dermatitis skin is characterized by broad terminal differentiation defects and variable immune abnormalities.	J Allergy Clin Immunol.
atopic dermatitis	SNX2	Expression profiling by array, count, 3 development stage, 2 disease state, 11 individual sets	GPL570	GSE32924 Samples	Nonlesional atopic dermatitis skin is characterized by broad terminal differentiation defects and variable immune abnormalities.	J Allergy Clin Immunol.
atopic dermatitis	PCNP	Expression profiling by array, count, 3 development stage, 2 disease state, 11 individual sets	GPL570	GSE32924 Samples	Nonlesional atopic dermatitis skin is characterized by broad terminal differentiation defects and variable immune abnormalities.	J Allergy Clin Immunol.
atopic dermatitis	YAP1	Expression profiling by array, count, 3 development stage, 2 disease state, 11 individual sets	GPL570	GSE32924 Samples	Nonlesional atopic dermatitis skin is characterized by broad terminal differentiation defects and variable immune abnormalities.	J Allergy Clin Immunol.
atopic dermatitis	FOXN3	Expression profiling by array, count, 3 development stage, 2 disease state, 11 individual sets	GPL570	GSE32924 Samples	Nonlesional atopic dermatitis skin is characterized by broad terminal differentiation defects and variable immune abnormalities.	J Allergy Clin Immunol.
atopic dermatitis	C1ORF96	Expression profiling by array, count, 3 development stage, 2 disease state, 11 individual sets	GPL570	GSE32924 Samples	Nonlesional atopic dermatitis skin is characterized by broad terminal differentiation defects and variable immune abnormalities.	J Allergy Clin Immunol.
atopic dermatitis	PTPN21	Expression profiling by array, count, 3 development stage, 2 disease state, 11 individual sets	GPL570	GSE32924 Samples	Nonlesional atopic dermatitis skin is characterized by broad terminal differentiation defects and variable immune abnormalities.	J Allergy Clin Immunol.
atopic dermatitis	FGFR2	Expression profiling by array, count, 3 development stage, 2 disease state, 11 individual sets	GPL570	GSE32924 Samples	Nonlesional atopic dermatitis skin is characterized by broad terminal differentiation defects and variable immune abnormalities.	J Allergy Clin Immunol.
atopic dermatitis	C12ORF29	Expression profiling by array, count, 3 development stage, 2 disease state, 11 individual sets	GPL570	GSE32924 Samples	Nonlesional atopic dermatitis skin is characterized by broad terminal differentiation defects and variable immune abnormalities.	J Allergy Clin Immunol.
atopic dermatitis	CSNK2A2	Expression profiling by array, count, 3 development stage, 2 disease state, 11 individual sets	GPL570	GSE32924 Samples	Nonlesional atopic dermatitis skin is characterized by broad terminal differentiation defects and variable immune abnormalities.	J Allergy Clin Immunol.
atopic dermatitis	PARD6G	Expression profiling by array, count, 3 development stage, 2 disease state, 11 individual sets	GPL570	GSE32924 Samples	Nonlesional atopic dermatitis skin is characterized by broad terminal differentiation defects and variable immune abnormalities.	J Allergy Clin Immunol.
atopic dermatitis	F2R	Expression profiling by array, count, 3 development stage, 2 disease state, 11 individual sets	GPL570	GSE32924 Samples	Nonlesional atopic dermatitis skin is characterized by broad terminal differentiation defects and variable immune abnormalities.	J Allergy Clin Immunol.
atopic dermatitis	CYP26B1	Expression profiling by array, count, 3 development stage, 2 disease state, 11 individual sets	GPL570	GSE32924 Samples	Nonlesional atopic dermatitis skin is characterized by broad terminal differentiation defects and variable immune abnormalities.	J Allergy Clin Immunol.
atopic dermatitis	ATP6AP2	Expression profiling by array, count, 3 development stage, 2 disease state, 11 individual sets	GPL570	GSE32924 Samples	Nonlesional atopic dermatitis skin is characterized by broad terminal differentiation defects and variable immune abnormalities.	J Allergy Clin Immunol.
atopic dermatitis	RAB1A	Expression profiling by array, count, 3 development stage, 2 disease state, 11 individual sets	GPL570	GSE32924 Samples	Nonlesional atopic dermatitis skin is characterized by broad terminal differentiation defects and variable immune abnormalities.	J Allergy Clin Immunol.
atopic dermatitis	SLC35F5	Expression profiling by array, count, 3 development stage, 2 disease state, 11 individual sets	GPL570	GSE32924 Samples	Nonlesional atopic dermatitis skin is characterized by broad terminal differentiation defects and variable immune abnormalities.	J Allergy Clin Immunol.
atopic dermatitis	UBL3	Expression profiling by array, count, 3 development stage, 2 disease state, 11 individual sets	GPL570	GSE32924 Samples	Nonlesional atopic dermatitis skin is characterized by broad terminal differentiation defects and variable immune abnormalities.	J Allergy Clin Immunol.
atopic dermatitis	CITED2	Expression profiling by array, count, 3 development stage, 2 disease state, 11 individual sets	GPL570	GSE32924 Samples	Nonlesional atopic dermatitis skin is characterized by broad terminal differentiation defects and variable immune abnormalities.	J Allergy Clin Immunol.
atopic dermatitis	NA	Expression profiling by array, count, 3 development stage, 2 disease state, 11 individual sets	GPL570	GSE32924 Samples	Nonlesional atopic dermatitis skin is characterized by broad terminal differentiation defects and variable immune abnormalities.	J Allergy Clin Immunol.
atopic dermatitis	ALDH3A2	Expression profiling by array, count, 3 development stage, 2 disease state, 11 individual sets	GPL570	GSE32924 Samples	Nonlesional atopic dermatitis skin is characterized by broad terminal differentiation defects and variable immune abnormalities.	J Allergy Clin Immunol.
atopic dermatitis	EGR3	Expression profiling by array, count, 3 development stage, 2 disease state, 11 individual sets	GPL570	GSE32924 Samples	Nonlesional atopic dermatitis skin is characterized by broad terminal differentiation defects and variable immune abnormalities.	J Allergy Clin Immunol.
atopic dermatitis	BNIP3L	Expression profiling by array, count, 3 development stage, 2 disease state, 11 individual sets	GPL570	GSE32924 Samples	Nonlesional atopic dermatitis skin is characterized by broad terminal differentiation defects and variable immune abnormalities.	J Allergy Clin Immunol.
atopic dermatitis	PALLD	Expression profiling by array, count, 3 development stage, 2 disease state, 11 individual sets	GPL570	GSE32924 Samples	Nonlesional atopic dermatitis skin is characterized by broad terminal differentiation defects and variable immune abnormalities.	J Allergy Clin Immunol.
atopic dermatitis	NA	Expression profiling by array, count, 3 development stage, 2 disease state, 11 individual sets	GPL570	GSE32924 Samples	Nonlesional atopic dermatitis skin is characterized by broad terminal differentiation defects and variable immune abnormalities.	J Allergy Clin Immunol.
atopic dermatitis	SCEL	Expression profiling by array, count, 3 development stage, 2 disease state, 11 individual sets	GPL570	GSE32924 Samples	Nonlesional atopic dermatitis skin is characterized by broad terminal differentiation defects and variable immune abnormalities.	J Allergy Clin Immunol.
atopic dermatitis	YWHAZ	Expression profiling by array, count, 3 development stage, 2 disease state, 11 individual sets	GPL570	GSE32924 Samples	Nonlesional atopic dermatitis skin is characterized by broad terminal differentiation defects and variable immune abnormalities.	J Allergy Clin Immunol.
atopic dermatitis	HSP90AB1	Expression profiling by array, count, 3 development stage, 2 disease state, 11 individual sets	GPL570	GSE32924 Samples	Nonlesional atopic dermatitis skin is characterized by broad terminal differentiation defects and variable immune abnormalities.	J Allergy Clin Immunol.
atopic dermatitis	KRT23	Expression profiling by array, count, 3 development stage, 2 disease state, 11 individual sets	GPL570	GSE32924 Samples	Nonlesional atopic dermatitis skin is characterized by broad terminal differentiation defects and variable immune abnormalities.	J Allergy Clin Immunol.
atopic dermatitis	CHMP4C	Expression profiling by array, count, 3 development stage, 2 disease state, 11 individual sets	GPL570	GSE32924 Samples	Nonlesional atopic dermatitis skin is characterized by broad terminal differentiation defects and variable immune abnormalities.	J Allergy Clin Immunol.
atopic dermatitis	ELOVL4	Expression profiling by array, count, 3 development stage, 2 disease state, 11 individual sets	GPL570	GSE32924 Samples	Nonlesional atopic dermatitis skin is characterized by broad terminal differentiation defects and variable immune abnormalities.	J Allergy Clin Immunol.
atopic dermatitis	HMGCR	Expression profiling by array, count, 3 development stage, 2 disease state, 11 individual sets	GPL570	GSE32924 Samples	Nonlesional atopic dermatitis skin is characterized by broad terminal differentiation defects and variable immune abnormalities.	J Allergy Clin Immunol.
atopic dermatitis	MALAT1	Expression profiling by array, count, 3 development stage, 2 disease state, 11 individual sets	GPL570	GSE32924 Samples	Nonlesional atopic dermatitis skin is characterized by broad terminal differentiation defects and variable immune abnormalities.	J Allergy Clin Immunol.
atopic dermatitis	CHURC1	Expression profiling by array, count, 3 development stage, 2 disease state, 11 individual sets	GPL570	GSE32924 Samples	Nonlesional atopic dermatitis skin is characterized by broad terminal differentiation defects and variable immune abnormalities.	J Allergy Clin Immunol.
atopic dermatitis	F3	Expression profiling by array, count, 3 development stage, 2 disease state, 11 individual sets	GPL570	GSE32924 Samples	Nonlesional atopic dermatitis skin is characterized by broad terminal differentiation defects and variable immune abnormalities.	J Allergy Clin Immunol.
atopic dermatitis	TXNIP	Expression profiling by array, count, 3 development stage, 2 disease state, 11 individual sets	GPL570	GSE32924 Samples	Nonlesional atopic dermatitis skin is characterized by broad terminal differentiation defects and variable immune abnormalities.	J Allergy Clin Immunol.
atopic dermatitis	PTGDS	Expression profiling by array, count, 3 development stage, 2 disease state, 11 individual sets	GPL570	GSE32924 Samples	Nonlesional atopic dermatitis skin is characterized by broad terminal differentiation defects and variable immune abnormalities.	J Allergy Clin Immunol.
atopic dermatitis	OLFML3	Expression profiling by array, count, 3 development stage, 2 disease state, 11 individual sets	GPL570	GSE32924 Samples	Nonlesional atopic dermatitis skin is characterized by broad terminal differentiation defects and variable immune abnormalities.	J Allergy Clin Immunol.
atopic dermatitis	OSR2	Expression profiling by array, count, 3 development stage, 2 disease state, 11 individual sets	GPL570	GSE32924 Samples	Nonlesional atopic dermatitis skin is characterized by broad terminal differentiation defects and variable immune abnormalities.	J Allergy Clin Immunol.
atopic dermatitis	DCLK1	Expression profiling by array, count, 3 development stage, 2 disease state, 11 individual sets	GPL570	GSE32924 Samples	Nonlesional atopic dermatitis skin is characterized by broad terminal differentiation defects and variable immune abnormalities.	J Allergy Clin Immunol.
atopic dermatitis	CD55	Expression profiling by array, count, 3 development stage, 2 disease state, 11 individual sets	GPL570	GSE32924 Samples	Nonlesional atopic dermatitis skin is characterized by broad terminal differentiation defects and variable immune abnormalities.	J Allergy Clin Immunol.

atopic dermatitis	CD55	Expression profiling by array, count, 3 development stage, 2 disease state, 11 individual sets	GPL570	GSE32924 Samples	Nonlesional atopic dermatitis skin is characterized by broad terminal differentiation defects and variable immune abnormalities.	J Allergy Clin Immunol.
atopic dermatitis	FBLN1	Expression profiling by array, count, 3 development stage, 2 disease state, 11 individual sets	GPL570	GSE32924 Samples	Nonlesional atopic dermatitis skin is characterized by broad terminal differentiation defects and variable immune abnormalities.	J Allergy Clin Immunol.
atopic dermatitis	LOX	Expression profiling by array, count, 3 development stage, 2 disease state, 11 individual sets	GPL570	GSE32924 Samples	Nonlesional atopic dermatitis skin is characterized by broad terminal differentiation defects and variable immune abnormalities.	J Allergy Clin Immunol.
atopic dermatitis	AGTR1	Expression profiling by array, count, 3 development stage, 2 disease state, 11 individual sets	GPL570	GSE32924 Samples	Nonlesional atopic dermatitis skin is characterized by broad terminal differentiation defects and variable immune abnormalities.	J Allergy Clin Immunol.
atopic dermatitis	C5ORF46	Expression profiling by array, count, 3 development stage, 2 disease state, 11 individual sets	GPL570	GSE32924 Samples	Nonlesional atopic dermatitis skin is characterized by broad terminal differentiation defects and variable immune abnormalities.	J Allergy Clin Immunol.
atopic dermatitis	NBPF10	Expression profiling by array, count, 3 development stage, 2 disease state, 11 individual sets	GPL570	GSE32924 Samples	Nonlesional atopic dermatitis skin is characterized by broad terminal differentiation defects and variable immune abnormalities.	J Allergy Clin Immunol.
atopic dermatitis	UBXN4	Expression profiling by array, count, 3 development stage, 2 disease state, 11 individual sets	GPL570	GSE32924 Samples	Nonlesional atopic dermatitis skin is characterized by broad terminal differentiation defects and variable immune abnormalities.	J Allergy Clin Immunol.
atopic dermatitis	CCDC80	Expression profiling by array, count, 3 development stage, 2 disease state, 11 individual sets	GPL570	GSE32924 Samples	Nonlesional atopic dermatitis skin is characterized by broad terminal differentiation defects and variable immune abnormalities.	J Allergy Clin Immunol.
atopic dermatitis	SFRS2IP	Expression profiling by array, count, 3 development stage, 2 disease state, 11 individual sets	GPL570	GSE32924 Samples	Nonlesional atopic dermatitis skin is characterized by broad terminal differentiation defects and variable immune abnormalities.	J Allergy Clin Immunol.
atopic dermatitis	QSER1	Expression profiling by array, count, 3 development stage, 2 disease state, 11 individual sets	GPL570	GSE32924 Samples	Nonlesional atopic dermatitis skin is characterized by broad terminal differentiation defects and variable immune abnormalities.	J Allergy Clin Immunol.
atopic dermatitis	MALAT1	Expression profiling by array, count, 3 development stage, 2 disease state, 11 individual sets	GPL570	GSE32924 Samples	Nonlesional atopic dermatitis skin is characterized by broad terminal differentiation defects and variable immune abnormalities.	J Allergy Clin Immunol.
atopic dermatitis	WIF1	Expression profiling by array, count, 3 development stage, 2 disease state, 11 individual sets	GPL570	GSE32924 Samples	Nonlesional atopic dermatitis skin is characterized by broad terminal differentiation defects and variable immune abnormalities.	J Allergy Clin Immunol.
atopic dermatitis	FRMD6	Expression profiling by array, count, 3 development stage, 2 disease state, 11 individual sets	GPL570	GSE32924 Samples	Nonlesional atopic dermatitis skin is characterized by broad terminal differentiation defects and variable immune abnormalities.	J Allergy Clin Immunol.
atopic dermatitis	SLC30A1	Expression profiling by array, count, 3 development stage, 2 disease state, 11 individual sets	GPL570	GSE32924 Samples	Nonlesional atopic dermatitis skin is characterized by broad terminal differentiation defects and variable immune abnormalities.	J Allergy Clin Immunol.
atopic dermatitis	CMTM6	Expression profiling by array, count, 3 development stage, 2 disease state, 11 individual sets	GPL570	GSE32924 Samples	Nonlesional atopic dermatitis skin is characterized by broad terminal differentiation defects and variable immune abnormalities.	J Allergy Clin Immunol.
atopic dermatitis	CAPRIN1	Expression profiling by array, count, 3 development stage, 2 disease state, 11 individual sets	GPL570	GSE32924 Samples	Nonlesional atopic dermatitis skin is characterized by broad terminal differentiation defects and variable immune abnormalities.	J Allergy Clin Immunol.
atopic dermatitis	UCHL3	Expression profiling by array, count, 3 development stage, 2 disease state, 11 individual sets	GPL570	GSE32924 Samples	Nonlesional atopic dermatitis skin is characterized by broad terminal differentiation defects and variable immune abnormalities.	J Allergy Clin Immunol.
atopic dermatitis	EMP2	Expression profiling by array, count, 3 development stage, 2 disease state, 11 individual sets	GPL570	GSE32924 Samples	Nonlesional atopic dermatitis skin is characterized by broad terminal differentiation defects and variable immune abnormalities.	J Allergy Clin Immunol.
atopic dermatitis	RAD23B	Expression profiling by array, count, 3 development stage, 2 disease state, 11 individual sets	GPL570	GSE32924 Samples	Nonlesional atopic dermatitis skin is characterized by broad terminal differentiation defects and variable immune abnormalities.	J Allergy Clin Immunol.
atopic dermatitis	SPINK5	Expression profiling by array, count, 3 development stage, 2 disease state, 11 individual sets	GPL570	GSE32924 Samples	Nonlesional atopic dermatitis skin is characterized by broad terminal differentiation defects and variable immune abnormalities.	J Allergy Clin Immunol.
atopic dermatitis	CTSK	Expression profiling by array, count, 3 development stage, 2 disease state, 11 individual sets	GPL570	GSE32924 Samples	Nonlesional atopic dermatitis skin is characterized by broad terminal differentiation defects and variable immune abnormalities.	J Allergy Clin Immunol.
atopic dermatitis	CD55	Expression profiling by array, count, 3 development stage, 2 disease state, 11 individual sets	GPL570	GSE32924 Samples	Nonlesional atopic dermatitis skin is characterized by broad terminal differentiation defects and variable immune abnormalities.	J Allergy Clin Immunol.
atopic dermatitis	RAC1	Expression profiling by array, count, 3 development stage, 2 disease state, 11 individual sets	GPL570	GSE32924 Samples	Nonlesional atopic dermatitis skin is characterized by broad terminal differentiation defects and variable immune abnormalities.	J Allergy Clin Immunol.
atopic dermatitis	RHOA	Expression profiling by array, count, 3 development stage, 2 disease state, 11 individual sets	GPL570	GSE32924 Samples	Nonlesional atopic dermatitis skin is characterized by broad terminal differentiation defects and variable immune abnormalities.	J Allergy Clin Immunol.
atopic dermatitis	GRHL1	Expression profiling by array, count, 3 development stage, 2 disease state, 11 individual sets	GPL570	GSE32924 Samples	Nonlesional atopic dermatitis skin is characterized by broad terminal differentiation defects and variable immune abnormalities.	J Allergy Clin Immunol.
atopic dermatitis	ZNF750	Expression profiling by array, count, 3 development stage, 2 disease state, 11 individual sets	GPL570	GSE32924 Samples	Nonlesional atopic dermatitis skin is characterized by broad terminal differentiation defects and variable immune abnormalities.	J Allergy Clin Immunol.
atopic dermatitis	HSPC159	Expression profiling by array, count, 3 development stage, 2 disease state, 11 individual sets	GPL570	GSE32924 Samples	Nonlesional atopic dermatitis skin is characterized by broad terminal differentiation defects and variable immune abnormalities.	J Allergy Clin Immunol.
atopic dermatitis	MAL2	Expression profiling by array, count, 3 development stage, 2 disease state, 11 individual sets	GPL570	GSE32924 Samples	Nonlesional atopic dermatitis skin is characterized by broad terminal differentiation defects and variable immune abnormalities.	J Allergy Clin Immunol.
atopic dermatitis	SERPINB5	Expression profiling by array, count, 3 development stage, 2 disease state, 11 individual sets	GPL570	GSE32924 Samples	Nonlesional atopic dermatitis skin is characterized by broad terminal differentiation defects and variable immune abnormalities.	J Allergy Clin Immunol.
atopic dermatitis	HOPX	Expression profiling by array, count, 3 development stage, 2 disease state, 11 individual sets	GPL570	GSE32924 Samples	Nonlesional atopic dermatitis skin is characterized by broad terminal differentiation defects and variable immune abnormalities.	J Allergy Clin Immunol.
atopic dermatitis	DSG1	Expression profiling by array, count, 3 development stage, 2 disease state, 11 individual sets	GPL570	GSE32924 Samples	Nonlesional atopic dermatitis skin is characterized by broad terminal differentiation defects and variable immune abnormalities.	J Allergy Clin Immunol.
atopic dermatitis	DEGS1	Expression profiling by array, count, 3 development stage, 2 disease state, 11 individual sets	GPL570	GSE32924 Samples	Nonlesional atopic dermatitis skin is characterized by broad terminal differentiation defects and variable immune abnormalities.	J Allergy Clin Immunol.
atopic dermatitis	FLG	Expression profiling by array, count, 3 development stage, 2 disease state, 11 individual sets	GPL570	GSE32924 Samples	Nonlesional atopic dermatitis skin is characterized by broad terminal differentiation defects and variable immune abnormalities.	J Allergy Clin Immunol.
atopic dermatitis	SFRP2	Expression profiling by array, count, 3 development stage, 2 disease state, 11 individual sets	GPL570	GSE32924 Samples	Nonlesional atopic dermatitis skin is characterized by broad terminal differentiation defects and variable immune abnormalities.	J Allergy Clin Immunol.
atopic dermatitis	PPP1R14C	Expression profiling by array, count, 3 development stage, 2 disease state, 11 individual sets	GPL570	GSE32924 Samples	Nonlesional atopic dermatitis skin is characterized by broad terminal differentiation defects and variable immune abnormalities.	J Allergy Clin Immunol.
atopic dermatitis	GNAI1	Expression profiling by array, count, 3 development stage, 2 disease state, 11 individual sets	GPL570	GSE32924 Samples	Nonlesional atopic dermatitis skin is characterized by broad terminal differentiation defects and variable immune abnormalities.	J Allergy Clin Immunol.
atopic dermatitis	GATM	Expression profiling by array, count, 3 development stage, 2 disease state, 11 individual sets	GPL570	GSE32924 Samples	Nonlesional atopic dermatitis skin is characterized by broad terminal differentiation defects and variable immune abnormalities.	J Allergy Clin Immunol.
atopic dermatitis	FBLN1	Expression profiling by array, count, 3 development stage, 2 disease state, 11 individual sets	GPL570	GSE32924 Samples	Nonlesional atopic dermatitis skin is characterized by broad terminal differentiation defects and variable immune abnormalities.	J Allergy Clin Immunol.
atopic dermatitis	NA	Expression profiling by array, count, 3 development stage, 2 disease state, 11 individual sets	GPL570	GSE32924 Samples	Nonlesional atopic dermatitis skin is characterized by broad terminal differentiation defects and variable immune abnormalities.	J Allergy Clin Immunol.
atopic dermatitis	FBLN1	Expression profiling by array, count, 3 development stage, 2 disease state, 11 individual sets	GPL570	GSE32924 Samples	Nonlesional atopic dermatitis skin is characterized by broad terminal differentiation defects and variable immune abnormalities.	J Allergy Clin Immunol.
atopic dermatitis	LEPR	Expression profiling by array, count, 3 development stage, 2 disease state, 11 individual sets	GPL570	GSE32924 Samples	Nonlesional atopic dermatitis skin is characterized by broad terminal differentiation defects and variable immune abnormalities.	J Allergy Clin Immunol.

atopic dermatitis	CALD1	Expression profiling by array, count, 3 development stage, 2 disease state, 11 individual sets	GPL570	GSE32924 Samples	Nonlesional atopic dermatitis skin is characterized by broad terminal differentiation defects and variable immune abnormalities.	J Allergy Clin Immunol.
atopic dermatitis	TIMP2	Expression profiling by array, count, 3 development stage, 2 disease state, 11 individual sets	GPL570	GSE32924 Samples	Nonlesional atopic dermatitis skin is characterized by broad terminal differentiation defects and variable immune abnormalities.	J Allergy Clin Immunol.
atopic dermatitis	RORA	Expression profiling by array, count, 3 development stage, 2 disease state, 11 individual sets	GPL570	GSE32924 Samples	Nonlesional atopic dermatitis skin is characterized by broad terminal differentiation defects and variable immune abnormalities.	J Allergy Clin Immunol.
atopic dermatitis	CLDN1	Expression profiling by array, count, 3 development stage, 2 disease state, 11 individual sets	GPL570	GSE32924 Samples	Nonlesional atopic dermatitis skin is characterized by broad terminal differentiation defects and variable immune abnormalities.	J Allergy Clin Immunol.
atopic dermatitis	SCEL	Expression profiling by array, count, 3 development stage, 2 disease state, 11 individual sets	GPL570	GSE32924 Samples	Nonlesional atopic dermatitis skin is characterized by broad terminal differentiation defects and variable immune abnormalities.	J Allergy Clin Immunol.
atopic dermatitis	MALAT1	Expression profiling by array, count, 3 development stage, 2 disease state, 11 individual sets	GPL570	GSE32924 Samples	Nonlesional atopic dermatitis skin is characterized by broad terminal differentiation defects and variable immune abnormalities.	J Allergy Clin Immunol.
atopic dermatitis	NA	Expression profiling by array, count, 3 development stage, 2 disease state, 11 individual sets	GPL570	GSE32924 Samples	Nonlesional atopic dermatitis skin is characterized by broad terminal differentiation defects and variable immune abnormalities.	J Allergy Clin Immunol.
atopic dermatitis	HBB	Expression profiling by array, count, 3 development stage, 2 disease state, 11 individual sets	GPL570	GSE32924 Samples	Nonlesional atopic dermatitis skin is characterized by broad terminal differentiation defects and variable immune abnormalities.	J Allergy Clin Immunol.
atopic dermatitis	HBB	Expression profiling by array, count, 3 development stage, 2 disease state, 11 individual sets	GPL570	GSE32924 Samples	Nonlesional atopic dermatitis skin is characterized by broad terminal differentiation defects and variable immune abnormalities.	J Allergy Clin Immunol.
atopic dermatitis	HBB	Expression profiling by array, count, 3 development stage, 2 disease state, 11 individual sets	GPL570	GSE32924 Samples	Nonlesional atopic dermatitis skin is characterized by broad terminal differentiation defects and variable immune abnormalities.	J Allergy Clin Immunol.
atopic dermatitis	NA	Expression profiling by array, count, 3 development stage, 2 disease state, 11 individual sets	GPL570	GSE32924 Samples	Nonlesional atopic dermatitis skin is characterized by broad terminal differentiation defects and variable immune abnormalities.	J Allergy Clin Immunol.
atopic dermatitis	NFATC2IP	Expression profiling by array, count, 3 development stage, 2 disease state, 11 individual sets	GPL570	GSE32924 Samples	Nonlesional atopic dermatitis skin is characterized by broad terminal differentiation defects and variable immune abnormalities.	J Allergy Clin Immunol.
atopic dermatitis	C9ORF3	Expression profiling by array, count, 3 development stage, 2 disease state, 11 individual sets	GPL570	GSE32924 Samples	Nonlesional atopic dermatitis skin is characterized by broad terminal differentiation defects and variable immune abnormalities.	J Allergy Clin Immunol.
atopic dermatitis	NA	Expression profiling by array, count, 3 development stage, 2 disease state, 11 individual sets	GPL570	GSE32924 Samples	Nonlesional atopic dermatitis skin is characterized by broad terminal differentiation defects and variable immune abnormalities.	J Allergy Clin Immunol.
atopic dermatitis	WASH3P	Expression profiling by array, count, 3 development stage, 2 disease state, 11 individual sets	GPL570	GSE32924 Samples	Nonlesional atopic dermatitis skin is characterized by broad terminal differentiation defects and variable immune abnormalities.	J Allergy Clin Immunol.
atopic dermatitis	HSPG2	Expression profiling by array, count, 3 development stage, 2 disease state, 11 individual sets	GPL570	GSE32924 Samples	Nonlesional atopic dermatitis skin is characterized by broad terminal differentiation defects and variable immune abnormalities.	J Allergy Clin Immunol.
atopic dermatitis	CFLAR	Expression profiling by array, count, 3 development stage, 2 disease state, 11 individual sets	GPL570	GSE32924 Samples	Nonlesional atopic dermatitis skin is characterized by broad terminal differentiation defects and variable immune abnormalities.	J Allergy Clin Immunol.
atopic dermatitis	ANKRD9	Expression profiling by array, count, 3 development stage, 2 disease state, 11 individual sets	GPL570	GSE32924 Samples	Nonlesional atopic dermatitis skin is characterized by broad terminal differentiation defects and variable immune abnormalities.	J Allergy Clin Immunol.
atopic dermatitis	TNS4	Expression profiling by array, count, 3 development stage, 2 disease state, 11 individual sets	GPL570	GSE32924 Samples	Nonlesional atopic dermatitis skin is characterized by broad terminal differentiation defects and variable immune abnormalities.	J Allergy Clin Immunol.
atopic dermatitis	DICER1	Expression profiling by array, count, 3 development stage, 2 disease state, 11 individual sets	GPL570	GSE32924 Samples	Nonlesional atopic dermatitis skin is characterized by broad terminal differentiation defects and variable immune abnormalities.	J Allergy Clin Immunol.
atopic dermatitis	NA	Expression profiling by array, count, 3 development stage, 2 disease state, 11 individual sets	GPL570	GSE32924 Samples	Nonlesional atopic dermatitis skin is characterized by broad terminal differentiation defects and variable immune abnormalities.	J Allergy Clin Immunol.
atopic dermatitis	MBNL1	Expression profiling by array, count, 3 development stage, 2 disease state, 11 individual sets	GPL570	GSE32924 Samples	Nonlesional atopic dermatitis skin is characterized by broad terminal differentiation defects and variable immune abnormalities.	J Allergy Clin Immunol.
atopic dermatitis	LOC641298	Expression profiling by array, count, 3 development stage, 2 disease state, 11 individual sets	GPL570	GSE32924 Samples	Nonlesional atopic dermatitis skin is characterized by broad terminal differentiation defects and variable immune abnormalities.	J Allergy Clin Immunol.
atopic dermatitis	NA	Expression profiling by array, count, 3 development stage, 2 disease state, 11 individual sets	GPL570	GSE32924 Samples	Nonlesional atopic dermatitis skin is characterized by broad terminal differentiation defects and variable immune abnormalities.	J Allergy Clin Immunol.
atopic dermatitis	DCN	Expression profiling by array, count, 3 development stage, 2 disease state, 11 individual sets	GPL570	GSE32924 Samples	Nonlesional atopic dermatitis skin is characterized by broad terminal differentiation defects and variable immune abnormalities.	J Allergy Clin Immunol.
atopic dermatitis	DCN	Expression profiling by array, count, 3 development stage, 2 disease state, 11 individual sets	GPL570	GSE32924 Samples	Nonlesional atopic dermatitis skin is characterized by broad terminal differentiation defects and variable immune abnormalities.	J Allergy Clin Immunol.
atopic dermatitis	TMEM45A	Expression profiling by array, count, 3 development stage, 2 disease state, 11 individual sets	GPL570	GSE32924 Samples	Nonlesional atopic dermatitis skin is characterized by broad terminal differentiation defects and variable immune abnormalities.	J Allergy Clin Immunol.
atopic dermatitis	KRT2	Expression profiling by array, count, 3 development stage, 2 disease state, 11 individual sets	GPL570	GSE32924 Samples	Nonlesional atopic dermatitis skin is characterized by broad terminal differentiation defects and variable immune abnormalities.	J Allergy Clin Immunol.
atopic dermatitis	FLG2	Expression profiling by array, count, 3 development stage, 2 disease state, 11 individual sets	GPL570	GSE32924 Samples	Nonlesional atopic dermatitis skin is characterized by broad terminal differentiation defects and variable immune abnormalities.	J Allergy Clin Immunol.
atopic dermatitis	KRT16	Expression profiling by array, count, 3 development stage, 2 disease state, 11 individual sets	GPL570	GSE32924 Samples	Nonlesional atopic dermatitis skin is characterized by broad terminal differentiation defects and variable immune abnormalities.	J Allergy Clin Immunol.
atopic dermatitis	IDE	Expression profiling by array, count, 3 development stage, 2 disease state, 11 individual sets	GPL570	GSE32924 Samples	Nonlesional atopic dermatitis skin is characterized by broad terminal differentiation defects and variable immune abnormalities.	J Allergy Clin Immunol.
atopic dermatitis	ABCA12	Expression profiling by array, count, 3 development stage, 2 disease state, 11 individual sets	GPL570	GSE32924 Samples	Nonlesional atopic dermatitis skin is characterized by broad terminal differentiation defects and variable immune abnormalities.	J Allergy Clin Immunol.
atopic dermatitis	TPRG1	Expression profiling by array, count, 3 development stage, 2 disease state, 11 individual sets	GPL570	GSE32924 Samples	Nonlesional atopic dermatitis skin is characterized by broad terminal differentiation defects and variable immune abnormalities.	J Allergy Clin Immunol.
atopic dermatitis	KLK5	Expression profiling by array, count, 3 development stage, 2 disease state, 11 individual sets	GPL570	GSE32924 Samples	Nonlesional atopic dermatitis skin is characterized by broad terminal differentiation defects and variable immune abnormalities.	J Allergy Clin Immunol.
atopic dermatitis	POF1B	Expression profiling by array, count, 3 development stage, 2 disease state, 11 individual sets	GPL570	GSE32924 Samples	Nonlesional atopic dermatitis skin is characterized by broad terminal differentiation defects and variable immune abnormalities.	J Allergy Clin Immunol.
atopic dermatitis	KLK7	Expression profiling by array, count, 3 development stage, 2 disease state, 11 individual sets	GPL570	GSE32924 Samples	Nonlesional atopic dermatitis skin is characterized by broad terminal differentiation defects and variable immune abnormalities.	J Allergy Clin Immunol.
atopic dermatitis	LY6G6C	Expression profiling by array, count, 3 development stage, 2 disease state, 11 individual sets	GPL570	GSE32924 Samples	Nonlesional atopic dermatitis skin is characterized by broad terminal differentiation defects and variable immune abnormalities.	J Allergy Clin Immunol.
atopic dermatitis	CDSN	Expression profiling by array, count, 3 development stage, 2 disease state, 11 individual sets	GPL570	GSE32924 Samples	Nonlesional atopic dermatitis skin is characterized by broad terminal differentiation defects and variable immune abnormalities.	J Allergy Clin Immunol.
atopic dermatitis	SLURP1	Expression profiling by array, count, 3 development stage, 2 disease state, 11 individual sets	GPL570	GSE32924 Samples	Nonlesional atopic dermatitis skin is characterized by broad terminal differentiation defects and variable immune abnormalities.	J Allergy Clin Immunol.
atopic dermatitis	SERPINA12	Expression profiling by array, count, 3 development stage, 2 disease state, 11 individual sets	GPL570	GSE32924 Samples	Nonlesional atopic dermatitis skin is characterized by broad terminal differentiation defects and variable immune abnormalities.	J Allergy Clin Immunol.
atopic dermatitis	C1ORF68	Expression profiling by array, count, 3 development stage, 2 disease state, 11 individual sets	GPL570	GSE32924 Samples	Nonlesional atopic dermatitis skin is characterized by broad terminal differentiation defects and variable immune abnormalities.	J Allergy Clin Immunol.

atopic dermatitis	EREG	Expression profiling by array, count, 3 development stage, 2 disease state, 11 individual sets	GPL570	GSE32924 Samples	Nonlesional atopic dermatitis skin is characterized by broad terminal differentiation defects and variable immune abnormalities.	J Allergy Clin Immunol.
atopic dermatitis	SERPINB7	Expression profiling by array, count, 3 development stage, 2 disease state, 11 individual sets	GPL570	GSE32924 Samples	Nonlesional atopic dermatitis skin is characterized by broad terminal differentiation defects and variable immune abnormalities.	J Allergy Clin Immunol.
atopic dermatitis	LOR	Expression profiling by array, count, 3 development stage, 2 disease state, 11 individual sets	GPL570	GSE32924 Samples	Nonlesional atopic dermatitis skin is characterized by broad terminal differentiation defects and variable immune abnormalities.	J Allergy Clin Immunol.
atopic dermatitis	LCE1B	Expression profiling by array, count, 3 development stage, 2 disease state, 11 individual sets	GPL570	GSE32924 Samples	Nonlesional atopic dermatitis skin is characterized by broad terminal differentiation defects and variable immune abnormalities.	J Allergy Clin Immunol.
atopic dermatitis	CST6	Expression profiling by array, count, 3 development stage, 2 disease state, 11 individual sets	GPL570	GSE32924 Samples	Nonlesional atopic dermatitis skin is characterized by broad terminal differentiation defects and variable immune abnormalities.	J Allergy Clin Immunol.
atopic dermatitis	ASPRV1	Expression profiling by array, count, 3 development stage, 2 disease state, 11 individual sets	GPL570	GSE32924 Samples	Nonlesional atopic dermatitis skin is characterized by broad terminal differentiation defects and variable immune abnormalities.	J Allergy Clin Immunol.
atopic dermatitis	LCE2B	Expression profiling by array, count, 3 development stage, 2 disease state, 11 individual sets	GPL570	GSE32924 Samples	Nonlesional atopic dermatitis skin is characterized by broad terminal differentiation defects and variable immune abnormalities.	J Allergy Clin Immunol.
atopic dermatitis	COL6A6	Expression profiling by array, count, 3 development stage, 2 disease state, 11 individual sets	GPL570	GSE32924 Samples	Nonlesional atopic dermatitis skin is characterized by broad terminal differentiation defects and variable immune abnormalities.	J Allergy Clin Immunol.
atopic dermatitis	LTF	Expression profiling by array, count, 3 development stage, 2 disease state, 11 individual sets	GPL570	GSE32924 Samples	Nonlesional atopic dermatitis skin is characterized by broad terminal differentiation defects and variable immune abnormalities.	J Allergy Clin Immunol.
atopic dermatitis	LZTS1	Expression profiling by array, count, 3 development stage, 2 disease state, 11 individual sets	GPL570	GSE32924 Samples	Nonlesional atopic dermatitis skin is characterized by broad terminal differentiation defects and variable immune abnormalities.	J Allergy Clin Immunol.
atopic dermatitis	CLN6	Expression profiling by array, count, 3 development stage, 2 disease state, 11 individual sets	GPL570	GSE32924 Samples	Nonlesional atopic dermatitis skin is characterized by broad terminal differentiation defects and variable immune abnormalities.	J Allergy Clin Immunol.
atopic dermatitis	ATAD3B	Expression profiling by array, count, 3 development stage, 2 disease state, 11 individual sets	GPL570	GSE32924 Samples	Nonlesional atopic dermatitis skin is characterized by broad terminal differentiation defects and variable immune abnormalities.	J Allergy Clin Immunol.
atopic dermatitis	PRMT7	Expression profiling by array, count, 3 development stage, 2 disease state, 11 individual sets	GPL570	GSE32924 Samples	Nonlesional atopic dermatitis skin is characterized by broad terminal differentiation defects and variable immune abnormalities.	J Allergy Clin Immunol.
atopic dermatitis	SPR	Expression profiling by array, count, 3 development stage, 2 disease state, 11 individual sets	GPL570	GSE32924 Samples	Nonlesional atopic dermatitis skin is characterized by broad terminal differentiation defects and variable immune abnormalities.	J Allergy Clin Immunol.
atopic dermatitis	TMEM160	Expression profiling by array, count, 3 development stage, 2 disease state, 11 individual sets	GPL570	GSE32924 Samples	Nonlesional atopic dermatitis skin is characterized by broad terminal differentiation defects and variable immune abnormalities.	J Allergy Clin Immunol.
atopic dermatitis	NCLN	Expression profiling by array, count, 3 development stage, 2 disease state, 11 individual sets	GPL570	GSE32924 Samples	Nonlesional atopic dermatitis skin is characterized by broad terminal differentiation defects and variable immune abnormalities.	J Allergy Clin Immunol.
atopic dermatitis	NA	Expression profiling by array, count, 3 development stage, 2 disease state, 11 individual sets	GPL570	GSE32924 Samples	Nonlesional atopic dermatitis skin is characterized by broad terminal differentiation defects and variable immune abnormalities.	J Allergy Clin Immunol.
atopic dermatitis	RECQL4	Expression profiling by array, count, 3 development stage, 2 disease state, 11 individual sets	GPL570	GSE32924 Samples	Nonlesional atopic dermatitis skin is characterized by broad terminal differentiation defects and variable immune abnormalities.	J Allergy Clin Immunol.
atopic dermatitis	CDT1	Expression profiling by array, count, 3 development stage, 2 disease state, 11 individual sets	GPL570	GSE32924 Samples	Nonlesional atopic dermatitis skin is characterized by broad terminal differentiation defects and variable immune abnormalities.	J Allergy Clin Immunol.
atopic dermatitis	MAP3K14	Expression profiling by array, count, 3 development stage, 2 disease state, 11 individual sets	GPL570	GSE32924 Samples	Nonlesional atopic dermatitis skin is characterized by broad terminal differentiation defects and variable immune abnormalities.	J Allergy Clin Immunol.
atopic dermatitis	MECOM	Expression profiling by array, count, 3 development stage, 2 disease state, 11 individual sets	GPL570	GSE32924 Samples	Nonlesional atopic dermatitis skin is characterized by broad terminal differentiation defects and variable immune abnormalities.	J Allergy Clin Immunol.
atopic dermatitis	PGF	Expression profiling by array, count, 3 development stage, 2 disease state, 11 individual sets	GPL570	GSE32924 Samples	Nonlesional atopic dermatitis skin is characterized by broad terminal differentiation defects and variable immune abnormalities.	J Allergy Clin Immunol.
atopic dermatitis	ALDH4A1	Expression profiling by array, count, 3 development stage, 2 disease state, 11 individual sets	GPL570	GSE32924 Samples	Nonlesional atopic dermatitis skin is characterized by broad terminal differentiation defects and variable immune abnormalities.	J Allergy Clin Immunol.
atopic dermatitis	PRR5	Expression profiling by array, count, 3 development stage, 2 disease state, 11 individual sets	GPL570	GSE32924 Samples	Nonlesional atopic dermatitis skin is characterized by broad terminal differentiation defects and variable immune abnormalities.	J Allergy Clin Immunol.
atopic dermatitis	PPP2R3B	Expression profiling by array, count, 3 development stage, 2 disease state, 11 individual sets	GPL570	GSE32924 Samples	Nonlesional atopic dermatitis skin is characterized by broad terminal differentiation defects and variable immune abnormalities.	J Allergy Clin Immunol.
atopic dermatitis	LOC728431	Expression profiling by array, count, 3 development stage, 2 disease state, 11 individual sets	GPL570	GSE32924 Samples	Nonlesional atopic dermatitis skin is characterized by broad terminal differentiation defects and variable immune abnormalities.	J Allergy Clin Immunol.
atopic dermatitis	CDK10	Expression profiling by array, count, 3 development stage, 2 disease state, 11 individual sets	GPL570	GSE32924 Samples	Nonlesional atopic dermatitis skin is characterized by broad terminal differentiation defects and variable immune abnormalities.	J Allergy Clin Immunol.
atopic dermatitis	EPN1	Expression profiling by array, count, 3 development stage, 2 disease state, 11 individual sets	GPL570	GSE32924 Samples	Nonlesional atopic dermatitis skin is characterized by broad terminal differentiation defects and variable immune abnormalities.	J Allergy Clin Immunol.
atopic dermatitis	SH3BP2	Expression profiling by array, count, 3 development stage, 2 disease state, 11 individual sets	GPL570	GSE32924 Samples	Nonlesional atopic dermatitis skin is characterized by broad terminal differentiation defects and variable immune abnormalities.	J Allergy Clin Immunol.
atopic dermatitis	METTL1	Expression profiling by array, count, 3 development stage, 2 disease state, 11 individual sets	GPL570	GSE32924 Samples	Nonlesional atopic dermatitis skin is characterized by broad terminal differentiation defects and variable immune abnormalities.	J Allergy Clin Immunol.
atopic dermatitis	SNHG3	Expression profiling by array, count, 3 development stage, 2 disease state, 11 individual sets	GPL570	GSE32924 Samples	Nonlesional atopic dermatitis skin is characterized by broad terminal differentiation defects and variable immune abnormalities.	J Allergy Clin Immunol.
atopic dermatitis	NAPEPLD	Expression profiling by array, count, 3 development stage, 2 disease state, 11 individual sets	GPL570	GSE32924 Samples	Nonlesional atopic dermatitis skin is characterized by broad terminal differentiation defects and variable immune abnormalities.	J Allergy Clin Immunol.
atopic dermatitis	MYO10	Expression profiling by array, count, 3 development stage, 2 disease state, 11 individual sets	GPL570	GSE32924 Samples	Nonlesional atopic dermatitis skin is characterized by broad terminal differentiation defects and variable immune abnormalities.	J Allergy Clin Immunol.
atopic dermatitis	ARMC6	Expression profiling by array, count, 3 development stage, 2 disease state, 11 individual sets	GPL570	GSE32924 Samples	Nonlesional atopic dermatitis skin is characterized by broad terminal differentiation defects and variable immune abnormalities.	J Allergy Clin Immunol.
atopic dermatitis	NA	Expression profiling by array, count, 3 development stage, 2 disease state, 11 individual sets	GPL570	GSE32924 Samples	Nonlesional atopic dermatitis skin is characterized by broad terminal differentiation defects and variable immune abnormalities.	J Allergy Clin Immunol.
atopic dermatitis	MAP6D1	Expression profiling by array, count, 3 development stage, 2 disease state, 11 individual sets	GPL570	GSE32924 Samples	Nonlesional atopic dermatitis skin is characterized by broad terminal differentiation defects and variable immune abnormalities.	J Allergy Clin Immunol.
atopic dermatitis	PAOX	Expression profiling by array, count, 3 development stage, 2 disease state, 11 individual sets	GPL570	GSE32924 Samples	Nonlesional atopic dermatitis skin is characterized by broad terminal differentiation defects and variable immune abnormalities.	J Allergy Clin Immunol.
atopic dermatitis	LRRC68	Expression profiling by array, count, 3 development stage, 2 disease state, 11 individual sets	GPL570	GSE32924 Samples	Nonlesional atopic dermatitis skin is characterized by broad terminal differentiation defects and variable immune abnormalities.	J Allergy Clin Immunol.
atopic dermatitis	FAM53A	Expression profiling by array, count, 3 development stage, 2 disease state, 11 individual sets	GPL570	GSE32924 Samples	Nonlesional atopic dermatitis skin is characterized by broad terminal differentiation defects and variable immune abnormalities.	J Allergy Clin Immunol.
atopic dermatitis	CORO1B	Expression profiling by array, count, 3 development stage, 2 disease state, 11 individual sets	GPL570	GSE32924 Samples	Nonlesional atopic dermatitis skin is characterized by broad terminal differentiation defects and variable immune abnormalities.	J Allergy Clin Immunol.
atopic dermatitis	FBXL19	Expression profiling by array, count, 3 development stage, 2 disease state, 11 individual sets	GPL570	GSE32924 Samples	Nonlesional atopic dermatitis skin is characterized by broad terminal differentiation defects and variable immune abnormalities.	J Allergy Clin Immunol.

atopic dermatitis	NRTN	Expression profiling by array, count, 3 development stage, 2 disease state, 11 individual sets	GPL570	GSE32924 Samples	Nonlesional atopic dermatitis skin is characterized by broad terminal differentiation defects and variable immune abnormalities.	J Allergy Clin Immunol.
atopic dermatitis	CBLC	Expression profiling by array, count, 3 development stage, 2 disease state, 11 individual sets	GPL570	GSE32924 Samples	Nonlesional atopic dermatitis skin is characterized by broad terminal differentiation defects and variable immune abnormalities.	J Allergy Clin Immunol.
atopic dermatitis	FDPSL2A	Expression profiling by array, count, 3 development stage, 2 disease state, 11 individual sets	GPL570	GSE32924 Samples	Nonlesional atopic dermatitis skin is characterized by broad terminal differentiation defects and variable immune abnormalities.	J Allergy Clin Immunol.
atopic dermatitis	GOLGA7B	Expression profiling by array, count, 3 development stage, 2 disease state, 11 individual sets	GPL570	GSE32924 Samples	Nonlesional atopic dermatitis skin is characterized by broad terminal differentiation defects and variable immune abnormalities.	J Allergy Clin Immunol.
atopic dermatitis	PRKXP1	Expression profiling by array, count, 3 development stage, 2 disease state, 11 individual sets	GPL570	GSE32924 Samples	Nonlesional atopic dermatitis skin is characterized by broad terminal differentiation defects and variable immune abnormalities.	J Allergy Clin Immunol.
atopic dermatitis	NA	Expression profiling by array, count, 3 development stage, 2 disease state, 11 individual sets	GPL570	GSE32924 Samples	Nonlesional atopic dermatitis skin is characterized by broad terminal differentiation defects and variable immune abnormalities.	J Allergy Clin Immunol.
atopic dermatitis	C6ORF141	Expression profiling by array, count, 3 development stage, 2 disease state, 11 individual sets	GPL570	GSE32924 Samples	Nonlesional atopic dermatitis skin is characterized by broad terminal differentiation defects and variable immune abnormalities.	J Allergy Clin Immunol.
atopic dermatitis	CCNB1	Expression profiling by array, count, 3 development stage, 2 disease state, 11 individual sets	GPL570	GSE32924 Samples	Nonlesional atopic dermatitis skin is characterized by broad terminal differentiation defects and variable immune abnormalities.	J Allergy Clin Immunol.
atopic dermatitis	CDC20	Expression profiling by array, count, 3 development stage, 2 disease state, 11 individual sets	GPL570	GSE32924 Samples	Nonlesional atopic dermatitis skin is characterized by broad terminal differentiation defects and variable immune abnormalities.	J Allergy Clin Immunol.
atopic dermatitis	AURKA	Expression profiling by array, count, 3 development stage, 2 disease state, 11 individual sets	GPL570	GSE32924 Samples	Nonlesional atopic dermatitis skin is characterized by broad terminal differentiation defects and variable immune abnormalities.	J Allergy Clin Immunol.
atopic dermatitis	NEK2	Expression profiling by array, count, 3 development stage, 2 disease state, 11 individual sets	GPL570	GSE32924 Samples	Nonlesional atopic dermatitis skin is characterized by broad terminal differentiation defects and variable immune abnormalities.	J Allergy Clin Immunol.
atopic dermatitis	NDC80	Expression profiling by array, count, 3 development stage, 2 disease state, 11 individual sets	GPL570	GSE32924 Samples	Nonlesional atopic dermatitis skin is characterized by broad terminal differentiation defects and variable immune abnormalities.	J Allergy Clin Immunol.
atopic dermatitis	CRLF1	Expression profiling by array, count, 3 development stage, 2 disease state, 11 individual sets	GPL570	GSE32924 Samples	Nonlesional atopic dermatitis skin is characterized by broad terminal differentiation defects and variable immune abnormalities.	J Allergy Clin Immunol.
atopic dermatitis	AK2	Expression profiling by array, count, 3 development stage, 2 disease state, 11 individual sets	GPL570	GSE32924 Samples	Nonlesional atopic dermatitis skin is characterized by broad terminal differentiation defects and variable immune abnormalities.	J Allergy Clin Immunol.
atopic dermatitis	LOC728806	Expression profiling by array, count, 3 development stage, 2 disease state, 11 individual sets	GPL570	GSE32924 Samples	Nonlesional atopic dermatitis skin is characterized by broad terminal differentiation defects and variable immune abnormalities.	J Allergy Clin Immunol.
atopic dermatitis	NA	Expression profiling by array, count, 3 development stage, 2 disease state, 11 individual sets	GPL570	GSE32924 Samples	Nonlesional atopic dermatitis skin is characterized by broad terminal differentiation defects and variable immune abnormalities.	J Allergy Clin Immunol.
atopic dermatitis	DKFZP434B20	Expression profiling by array, count, 3 development stage, 2 disease state, 11 individual sets	GPL570	GSE32924 Samples	Nonlesional atopic dermatitis skin is characterized by broad terminal differentiation defects and variable immune abnormalities.	J Allergy Clin Immunol.
atopic dermatitis	NA	Expression profiling by array, count, 3 development stage, 2 disease state, 11 individual sets	GPL570	GSE32924 Samples	Nonlesional atopic dermatitis skin is characterized by broad terminal differentiation defects and variable immune abnormalities.	J Allergy Clin Immunol.
atopic dermatitis	LOC654433	Expression profiling by array, count, 3 development stage, 2 disease state, 11 individual sets	GPL570	GSE32924 Samples	Nonlesional atopic dermatitis skin is characterized by broad terminal differentiation defects and variable immune abnormalities.	J Allergy Clin Immunol.
atopic dermatitis	MGC16385	Expression profiling by array, count, 3 development stage, 2 disease state, 11 individual sets	GPL570	GSE32924 Samples	Nonlesional atopic dermatitis skin is characterized by broad terminal differentiation defects and variable immune abnormalities.	J Allergy Clin Immunol.
atopic dermatitis	NTHL1	Expression profiling by array, count, 3 development stage, 2 disease state, 11 individual sets	GPL570	GSE32924 Samples	Nonlesional atopic dermatitis skin is characterized by broad terminal differentiation defects and variable immune abnormalities.	J Allergy Clin Immunol.
atopic dermatitis	NAT14	Expression profiling by array, count, 3 development stage, 2 disease state, 11 individual sets	GPL570	GSE32924 Samples	Nonlesional atopic dermatitis skin is characterized by broad terminal differentiation defects and variable immune abnormalities.	J Allergy Clin Immunol.
atopic dermatitis	TMCO4	Expression profiling by array, count, 3 development stage, 2 disease state, 11 individual sets	GPL570	GSE32924 Samples	Nonlesional atopic dermatitis skin is characterized by broad terminal differentiation defects and variable immune abnormalities.	J Allergy Clin Immunol.
atopic dermatitis	MLLT3	Expression profiling by array, count, 3 development stage, 2 disease state, 11 individual sets	GPL570	GSE32924 Samples	Nonlesional atopic dermatitis skin is characterized by broad terminal differentiation defects and variable immune abnormalities.	J Allergy Clin Immunol.
atopic dermatitis	C16ORF14	Expression profiling by array, count, 3 development stage, 2 disease state, 11 individual sets	GPL570	GSE32924 Samples	Nonlesional atopic dermatitis skin is characterized by broad terminal differentiation defects and variable immune abnormalities.	J Allergy Clin Immunol.
atopic dermatitis	SIGIRR	Expression profiling by array, count, 3 development stage, 2 disease state, 11 individual sets	GPL570	GSE32924 Samples	Nonlesional atopic dermatitis skin is characterized by broad terminal differentiation defects and variable immune abnormalities.	J Allergy Clin Immunol.
atopic dermatitis	GPRC5C	Expression profiling by array, count, 3 development stage, 2 disease state, 11 individual sets	GPL570	GSE32924 Samples	Nonlesional atopic dermatitis skin is characterized by broad terminal differentiation defects and variable immune abnormalities.	J Allergy Clin Immunol.
atopic dermatitis	CD320	Expression profiling by array, count, 3 development stage, 2 disease state, 11 individual sets	GPL570	GSE32924 Samples	Nonlesional atopic dermatitis skin is characterized by broad terminal differentiation defects and variable immune abnormalities.	J Allergy Clin Immunol.
atopic dermatitis	FLJ45482	Expression profiling by array, count, 3 development stage, 2 disease state, 11 individual sets	GPL570	GSE32924 Samples	Nonlesional atopic dermatitis skin is characterized by broad terminal differentiation defects and variable immune abnormalities.	J Allergy Clin Immunol.
atopic dermatitis	DUSP8	Expression profiling by array, count, 3 development stage, 2 disease state, 11 individual sets	GPL570	GSE32924 Samples	Nonlesional atopic dermatitis skin is characterized by broad terminal differentiation defects and variable immune abnormalities.	J Allergy Clin Immunol.
atopic dermatitis	C16ORF35	Expression profiling by array, count, 3 development stage, 2 disease state, 11 individual sets	GPL570	GSE32924 Samples	Nonlesional atopic dermatitis skin is characterized by broad terminal differentiation defects and variable immune abnormalities.	J Allergy Clin Immunol.
atopic dermatitis	NA	Expression profiling by array, count, 3 development stage, 2 disease state, 11 individual sets	GPL570	GSE32924 Samples	Nonlesional atopic dermatitis skin is characterized by broad terminal differentiation defects and variable immune abnormalities.	J Allergy Clin Immunol.
atopic dermatitis	NA	Expression profiling by array, count, 3 development stage, 2 disease state, 11 individual sets	GPL570	GSE32924 Samples	Nonlesional atopic dermatitis skin is characterized by broad terminal differentiation defects and variable immune abnormalities.	J Allergy Clin Immunol.
atopic dermatitis	BRF1	Expression profiling by array, count, 3 development stage, 2 disease state, 11 individual sets	GPL570	GSE32924 Samples	Nonlesional atopic dermatitis skin is characterized by broad terminal differentiation defects and variable immune abnormalities.	J Allergy Clin Immunol.
atopic dermatitis	NA	Expression profiling by array, count, 3 development stage, 2 disease state, 11 individual sets	GPL570	GSE32924 Samples	Nonlesional atopic dermatitis skin is characterized by broad terminal differentiation defects and variable immune abnormalities.	J Allergy Clin Immunol.
atopic dermatitis	FKRP	Expression profiling by array, count, 3 development stage, 2 disease state, 11 individual sets	GPL570	GSE32924 Samples	Nonlesional atopic dermatitis skin is characterized by broad terminal differentiation defects and variable immune abnormalities.	J Allergy Clin Immunol.
atopic dermatitis	KLHL17	Expression profiling by array, count, 3 development stage, 2 disease state, 11 individual sets	GPL570	GSE32924 Samples	Nonlesional atopic dermatitis skin is characterized by broad terminal differentiation defects and variable immune abnormalities.	J Allergy Clin Immunol.
atopic dermatitis	HEXDC	Expression profiling by array, count, 3 development stage, 2 disease state, 11 individual sets	GPL570	GSE32924 Samples	Nonlesional atopic dermatitis skin is characterized by broad terminal differentiation defects and variable immune abnormalities.	J Allergy Clin Immunol.
atopic dermatitis	CRELD1	Expression profiling by array, count, 3 development stage, 2 disease state, 11 individual sets	GPL570	GSE32924 Samples	Nonlesional atopic dermatitis skin is characterized by broad terminal differentiation defects and variable immune abnormalities.	J Allergy Clin Immunol.
atopic dermatitis	NA	Expression profiling by array, count, 3 development stage, 2 disease state, 11 individual sets	GPL570	GSE32924 Samples	Nonlesional atopic dermatitis skin is characterized by broad terminal differentiation defects and variable immune abnormalities.	J Allergy Clin Immunol.
atopic dermatitis	TAZ	Expression profiling by array, count, 3 development stage, 2 disease state, 11 individual sets	GPL570	GSE32924 Samples	Nonlesional atopic dermatitis skin is characterized by broad terminal differentiation defects and variable immune abnormalities.	J Allergy Clin Immunol.

atopic dermatitis	ZBTB48	Expression profiling by array, count, 3 development stage, 2 disease state, 11 individual sets	GPL570	GSE32924 Samples	Nonlesional atopic dermatitis skin is characterized by broad terminal differentiation defects and variable immune abnormalities.	J Allergy Clin Immunol.
atopic dermatitis	NUFIP1	Expression profiling by array, count, 3 development stage, 2 disease state, 11 individual sets	GPL570	GSE32924 Samples	Nonlesional atopic dermatitis skin is characterized by broad terminal differentiation defects and variable immune abnormalities.	J Allergy Clin Immunol.
atopic dermatitis	PHKG2	Expression profiling by array, count, 3 development stage, 2 disease state, 11 individual sets	GPL570	GSE32924 Samples	Nonlesional atopic dermatitis skin is characterized by broad terminal differentiation defects and variable immune abnormalities.	J Allergy Clin Immunol.
atopic dermatitis	NA	Expression profiling by array, count, 3 development stage, 2 disease state, 11 individual sets	GPL570	GSE32924 Samples	Nonlesional atopic dermatitis skin is characterized by broad terminal differentiation defects and variable immune abnormalities.	J Allergy Clin Immunol.
atopic dermatitis	DOT1L	Expression profiling by array, count, 3 development stage, 2 disease state, 11 individual sets	GPL570	GSE32924 Samples	Nonlesional atopic dermatitis skin is characterized by broad terminal differentiation defects and variable immune abnormalities.	J Allergy Clin Immunol.
atopic dermatitis	LOC442075	Expression profiling by array, count, 3 development stage, 2 disease state, 11 individual sets	GPL570	GSE32924 Samples	Nonlesional atopic dermatitis skin is characterized by broad terminal differentiation defects and variable immune abnormalities.	J Allergy Clin Immunol.
atopic dermatitis	NA	Expression profiling by array, count, 3 development stage, 2 disease state, 11 individual sets	GPL570	GSE32924 Samples	Nonlesional atopic dermatitis skin is characterized by broad terminal differentiation defects and variable immune abnormalities.	J Allergy Clin Immunol.
atopic dermatitis	NA	Expression profiling by array, count, 3 development stage, 2 disease state, 11 individual sets	GPL570	GSE32924 Samples	Nonlesional atopic dermatitis skin is characterized by broad terminal differentiation defects and variable immune abnormalities.	J Allergy Clin Immunol.
atopic dermatitis	MUDENG	Expression profiling by array, count, 3 development stage, 2 disease state, 11 individual sets	GPL570	GSE32924 Samples	Nonlesional atopic dermatitis skin is characterized by broad terminal differentiation defects and variable immune abnormalities.	J Allergy Clin Immunol.
atopic dermatitis	C19ORF60	Expression profiling by array, count, 3 development stage, 2 disease state, 11 individual sets	GPL570	GSE32924 Samples	Nonlesional atopic dermatitis skin is characterized by broad terminal differentiation defects and variable immune abnormalities.	J Allergy Clin Immunol.
atopic dermatitis	B3GAT3	Expression profiling by array, count, 3 development stage, 2 disease state, 11 individual sets	GPL570	GSE32924 Samples	Nonlesional atopic dermatitis skin is characterized by broad terminal differentiation defects and variable immune abnormalities.	J Allergy Clin Immunol.
atopic dermatitis	NA	Expression profiling by array, count, 3 development stage, 2 disease state, 11 individual sets	GPL570	GSE32924 Samples	Nonlesional atopic dermatitis skin is characterized by broad terminal differentiation defects and variable immune abnormalities.	J Allergy Clin Immunol.
atopic dermatitis	NA	Expression profiling by array, count, 3 development stage, 2 disease state, 11 individual sets	GPL570	GSE32924 Samples	Nonlesional atopic dermatitis skin is characterized by broad terminal differentiation defects and variable immune abnormalities.	J Allergy Clin Immunol.
atopic dermatitis	NA	Expression profiling by array, count, 3 development stage, 2 disease state, 11 individual sets	GPL570	GSE32924 Samples	Nonlesional atopic dermatitis skin is characterized by broad terminal differentiation defects and variable immune abnormalities.	J Allergy Clin Immunol.
atopic dermatitis	FGD3	Expression profiling by array, count, 3 development stage, 2 disease state, 11 individual sets	GPL570	GSE32924 Samples	Nonlesional atopic dermatitis skin is characterized by broad terminal differentiation defects and variable immune abnormalities.	J Allergy Clin Immunol.
atopic dermatitis	ANKRD13A	Expression profiling by array, count, 3 development stage, 2 disease state, 11 individual sets	GPL570	GSE32924 Samples	Nonlesional atopic dermatitis skin is characterized by broad terminal differentiation defects and variable immune abnormalities.	J Allergy Clin Immunol.
atopic dermatitis	BRD4	Expression profiling by array, count, 3 development stage, 2 disease state, 11 individual sets	GPL570	GSE32924 Samples	Nonlesional atopic dermatitis skin is characterized by broad terminal differentiation defects and variable immune abnormalities.	J Allergy Clin Immunol.
atopic dermatitis	NA	Expression profiling by array, count, 3 development stage, 2 disease state, 11 individual sets	GPL570	GSE32924 Samples	Nonlesional atopic dermatitis skin is characterized by broad terminal differentiation defects and variable immune abnormalities.	J Allergy Clin Immunol.
atopic dermatitis	SNX27	Expression profiling by array, count, 3 development stage, 2 disease state, 11 individual sets	GPL570	GSE32924 Samples	Nonlesional atopic dermatitis skin is characterized by broad terminal differentiation defects and variable immune abnormalities.	J Allergy Clin Immunol.
atopic dermatitis	NA	Expression profiling by array, count, 3 development stage, 2 disease state, 11 individual sets	GPL570	GSE32924 Samples	Nonlesional atopic dermatitis skin is characterized by broad terminal differentiation defects and variable immune abnormalities.	J Allergy Clin Immunol.
atopic dermatitis	NA	Expression profiling by array, count, 3 development stage, 2 disease state, 11 individual sets	GPL570	GSE32924 Samples	Nonlesional atopic dermatitis skin is characterized by broad terminal differentiation defects and variable immune abnormalities.	J Allergy Clin Immunol.
atopic dermatitis	KIAA1618	Expression profiling by array, count, 3 development stage, 2 disease state, 11 individual sets	GPL570	GSE32924 Samples	Nonlesional atopic dermatitis skin is characterized by broad terminal differentiation defects and variable immune abnormalities.	J Allergy Clin Immunol.
atopic dermatitis	NA	Expression profiling by array, count, 3 development stage, 2 disease state, 11 individual sets	GPL570	GSE32924 Samples	Nonlesional atopic dermatitis skin is characterized by broad terminal differentiation defects and variable immune abnormalities.	J Allergy Clin Immunol.
atopic dermatitis	IDS	Expression profiling by array, count, 3 development stage, 2 disease state, 11 individual sets	GPL570	GSE32924 Samples	Nonlesional atopic dermatitis skin is characterized by broad terminal differentiation defects and variable immune abnormalities.	J Allergy Clin Immunol.
atopic dermatitis	CNIH4	Expression profiling by array, count, 3 development stage, 2 disease state, 11 individual sets	GPL570	GSE32924 Samples	Nonlesional atopic dermatitis skin is characterized by broad terminal differentiation defects and variable immune abnormalities.	J Allergy Clin Immunol.
atopic dermatitis	NA	Expression profiling by array, count, 3 development stage, 2 disease state, 11 individual sets	GPL570	GSE32924 Samples	Nonlesional atopic dermatitis skin is characterized by broad terminal differentiation defects and variable immune abnormalities.	J Allergy Clin Immunol.
atopic dermatitis	ZNF628	Expression profiling by array, count, 3 development stage, 2 disease state, 11 individual sets	GPL570	GSE32924 Samples	Nonlesional atopic dermatitis skin is characterized by broad terminal differentiation defects and variable immune abnormalities.	J Allergy Clin Immunol.
atopic dermatitis	NA	Expression profiling by array, count, 3 development stage, 2 disease state, 11 individual sets	GPL570	GSE32924 Samples	Nonlesional atopic dermatitis skin is characterized by broad terminal differentiation defects and variable immune abnormalities.	J Allergy Clin Immunol.
atopic dermatitis	TPCN2	Expression profiling by array, count, 3 development stage, 2 disease state, 11 individual sets	GPL570	GSE32924 Samples	Nonlesional atopic dermatitis skin is characterized by broad terminal differentiation defects and variable immune abnormalities.	J Allergy Clin Immunol.
atopic dermatitis	SIRT2	Expression profiling by array, count, 3 development stage, 2 disease state, 11 individual sets	GPL570	GSE32924 Samples	Nonlesional atopic dermatitis skin is characterized by broad terminal differentiation defects and variable immune abnormalities.	J Allergy Clin Immunol.
atopic dermatitis	NUP210	Expression profiling by array, count, 3 development stage, 2 disease state, 11 individual sets	GPL570	GSE32924 Samples	Nonlesional atopic dermatitis skin is characterized by broad terminal differentiation defects and variable immune abnormalities.	J Allergy Clin Immunol.
atopic dermatitis	NA	Expression profiling by array, count, 3 development stage, 2 disease state, 11 individual sets	GPL570	GSE32924 Samples	Nonlesional atopic dermatitis skin is characterized by broad terminal differentiation defects and variable immune abnormalities.	J Allergy Clin Immunol.
atopic dermatitis	PSRC1	Expression profiling by array, count, 3 development stage, 2 disease state, 11 individual sets	GPL570	GSE32924 Samples	Nonlesional atopic dermatitis skin is characterized by broad terminal differentiation defects and variable immune abnormalities.	J Allergy Clin Immunol.
atopic dermatitis	GTSE1	Expression profiling by array, count, 3 development stage, 2 disease state, 11 individual sets	GPL570	GSE32924 Samples	Nonlesional atopic dermatitis skin is characterized by broad terminal differentiation defects and variable immune abnormalities.	J Allergy Clin Immunol.
atopic dermatitis	ZBTB1	Expression profiling by array, count, 3 development stage, 2 disease state, 11 individual sets	GPL570	GSE32924 Samples	Nonlesional atopic dermatitis skin is characterized by broad terminal differentiation defects and variable immune abnormalities.	J Allergy Clin Immunol.
atopic dermatitis	NA	Expression profiling by array, count, 3 development stage, 2 disease state, 11 individual sets	GPL570	GSE32924 Samples	Nonlesional atopic dermatitis skin is characterized by broad terminal differentiation defects and variable immune abnormalities.	J Allergy Clin Immunol.
atopic dermatitis	SAP30BP	Expression profiling by array, count, 3 development stage, 2 disease state, 11 individual sets	GPL570	GSE32924 Samples	Nonlesional atopic dermatitis skin is characterized by broad terminal differentiation defects and variable immune abnormalities.	J Allergy Clin Immunol.
atopic dermatitis	FOXP4	Expression profiling by array, count, 3 development stage, 2 disease state, 11 individual sets	GPL570	GSE32924 Samples	Nonlesional atopic dermatitis skin is characterized by broad terminal differentiation defects and variable immune abnormalities.	J Allergy Clin Immunol.
atopic dermatitis	GCAT	Expression profiling by array, count, 3 development stage, 2 disease state, 11 individual sets	GPL570	GSE32924 Samples	Nonlesional atopic dermatitis skin is characterized by broad terminal differentiation defects and variable immune abnormalities.	J Allergy Clin Immunol.
atopic dermatitis	CHDH	Expression profiling by array, count, 3 development stage, 2 disease state, 11 individual sets	GPL570	GSE32924 Samples	Nonlesional atopic dermatitis skin is characterized by broad terminal differentiation defects and variable immune abnormalities.	J Allergy Clin Immunol.
atopic dermatitis	WWC1	Expression profiling by array, count, 3 development stage, 2 disease state, 11 individual sets	GPL570	GSE32924 Samples	Nonlesional atopic dermatitis skin is characterized by broad terminal differentiation defects and variable immune abnormalities.	J Allergy Clin Immunol.

atopic dermatitis	TBX2	Expression profiling by array, count, 3 development stage, 2 disease state, 11 individual sets	GPL570	GSE32924 Samples	Nonlesional atopic dermatitis skin is characterized by broad terminal differentiation defects and variable immune abnormalities.	J Allergy Clin Immunol.
atopic dermatitis	NA	Expression profiling by array, count, 3 development stage, 2 disease state, 11 individual sets	GPL570	GSE32924 Samples	Nonlesional atopic dermatitis skin is characterized by broad terminal differentiation defects and variable immune abnormalities.	J Allergy Clin Immunol.
atopic dermatitis	NA	Expression profiling by array, count, 3 development stage, 2 disease state, 11 individual sets	GPL570	GSE32924 Samples	Nonlesional atopic dermatitis skin is characterized by broad terminal differentiation defects and variable immune abnormalities.	J Allergy Clin Immunol.
atopic dermatitis	MC1R	Expression profiling by array, count, 3 development stage, 2 disease state, 11 individual sets	GPL570	GSE32924 Samples	Nonlesional atopic dermatitis skin is characterized by broad terminal differentiation defects and variable immune abnormalities.	J Allergy Clin Immunol.
atopic dermatitis	GGT1	Expression profiling by array, count, 3 development stage, 2 disease state, 11 individual sets	GPL570	GSE32924 Samples	Nonlesional atopic dermatitis skin is characterized by broad terminal differentiation defects and variable immune abnormalities.	J Allergy Clin Immunol.
atopic dermatitis	OPLAH	Expression profiling by array, count, 3 development stage, 2 disease state, 11 individual sets	GPL570	GSE32924 Samples	Nonlesional atopic dermatitis skin is characterized by broad terminal differentiation defects and variable immune abnormalities.	J Allergy Clin Immunol.
atopic dermatitis	C9ORF140	Expression profiling by array, count, 3 development stage, 2 disease state, 11 individual sets	GPL570	GSE32924 Samples	Nonlesional atopic dermatitis skin is characterized by broad terminal differentiation defects and variable immune abnormalities.	J Allergy Clin Immunol.
atopic dermatitis	PSKH1	Expression profiling by array, count, 3 development stage, 2 disease state, 11 individual sets	GPL570	GSE32924 Samples	Nonlesional atopic dermatitis skin is characterized by broad terminal differentiation defects and variable immune abnormalities.	J Allergy Clin Immunol.
atopic dermatitis	LOC284801	Expression profiling by array, count, 3 development stage, 2 disease state, 11 individual sets	GPL570	GSE32924 Samples	Nonlesional atopic dermatitis skin is characterized by broad terminal differentiation defects and variable immune abnormalities.	J Allergy Clin Immunol.
atopic dermatitis	HIPK2	Expression profiling by array, count, 3 development stage, 2 disease state, 11 individual sets	GPL570	GSE32924 Samples	Nonlesional atopic dermatitis skin is characterized by broad terminal differentiation defects and variable immune abnormalities.	J Allergy Clin Immunol.
atopic dermatitis	NA	Expression profiling by array, count, 3 development stage, 2 disease state, 11 individual sets	GPL570	GSE32924 Samples	Nonlesional atopic dermatitis skin is characterized by broad terminal differentiation defects and variable immune abnormalities.	J Allergy Clin Immunol.
atopic dermatitis	TRPM1	Expression profiling by array, count, 3 development stage, 2 disease state, 11 individual sets	GPL570	GSE32924 Samples	Nonlesional atopic dermatitis skin is characterized by broad terminal differentiation defects and variable immune abnormalities.	J Allergy Clin Immunol.
atopic dermatitis	TRPM1	Expression profiling by array, count, 3 development stage, 2 disease state, 11 individual sets	GPL570	GSE32924 Samples	Nonlesional atopic dermatitis skin is characterized by broad terminal differentiation defects and variable immune abnormalities.	J Allergy Clin Immunol.
atopic dermatitis	NA	Expression profiling by array, count, 3 development stage, 2 disease state, 11 individual sets	GPL570	GSE32924 Samples	Nonlesional atopic dermatitis skin is characterized by broad terminal differentiation defects and variable immune abnormalities.	J Allergy Clin Immunol.
atopic dermatitis	NA	Expression profiling by array, count, 3 development stage, 2 disease state, 11 individual sets	GPL570	GSE32924 Samples	Nonlesional atopic dermatitis skin is characterized by broad terminal differentiation defects and variable immune abnormalities.	J Allergy Clin Immunol.
atopic dermatitis	KIF14	Expression profiling by array, count, 3 development stage, 2 disease state, 11 individual sets	GPL570	GSE32924 Samples	Nonlesional atopic dermatitis skin is characterized by broad terminal differentiation defects and variable immune abnormalities.	J Allergy Clin Immunol.
atopic dermatitis	FOXM1	Expression profiling by array, count, 3 development stage, 2 disease state, 11 individual sets	GPL570	GSE32924 Samples	Nonlesional atopic dermatitis skin is characterized by broad terminal differentiation defects and variable immune abnormalities.	J Allergy Clin Immunol.
atopic dermatitis	NA	Expression profiling by array, count, 3 development stage, 2 disease state, 11 individual sets	GPL570	GSE32924 Samples	Nonlesional atopic dermatitis skin is characterized by broad terminal differentiation defects and variable immune abnormalities.	J Allergy Clin Immunol.
atopic dermatitis	GFER	Expression profiling by array, count, 3 development stage, 2 disease state, 11 individual sets	GPL570	GSE32924 Samples	Nonlesional atopic dermatitis skin is characterized by broad terminal differentiation defects and variable immune abnormalities.	J Allergy Clin Immunol.
atopic dermatitis	NA	Expression profiling by array, count, 3 development stage, 2 disease state, 11 individual sets	GPL570	GSE32924 Samples	Nonlesional atopic dermatitis skin is characterized by broad terminal differentiation defects and variable immune abnormalities.	J Allergy Clin Immunol.
atopic dermatitis	NA	Expression profiling by array, count, 3 development stage, 2 disease state, 11 individual sets	GPL570	GSE32924 Samples	Nonlesional atopic dermatitis skin is characterized by broad terminal differentiation defects and variable immune abnormalities.	J Allergy Clin Immunol.
atopic dermatitis	C1ORF69	Expression profiling by array, count, 3 development stage, 2 disease state, 11 individual sets	GPL570	GSE32924 Samples	Nonlesional atopic dermatitis skin is characterized by broad terminal differentiation defects and variable immune abnormalities.	J Allergy Clin Immunol.
atopic dermatitis	NA	Expression profiling by array, count, 3 development stage, 2 disease state, 11 individual sets	GPL570	GSE32924 Samples	Nonlesional atopic dermatitis skin is characterized by broad terminal differentiation defects and variable immune abnormalities.	J Allergy Clin Immunol.
atopic dermatitis	ARFGAP1	Expression profiling by array, count, 3 development stage, 2 disease state, 11 individual sets	GPL570	GSE32924 Samples	Nonlesional atopic dermatitis skin is characterized by broad terminal differentiation defects and variable immune abnormalities.	J Allergy Clin Immunol.
atopic dermatitis	NA	Expression profiling by array, count, 3 development stage, 2 disease state, 11 individual sets	GPL570	GSE32924 Samples	Nonlesional atopic dermatitis skin is characterized by broad terminal differentiation defects and variable immune abnormalities.	J Allergy Clin Immunol.
atopic dermatitis	C19ORF20	Expression profiling by array, count, 3 development stage, 2 disease state, 11 individual sets	GPL570	GSE32924 Samples	Nonlesional atopic dermatitis skin is characterized by broad terminal differentiation defects and variable immune abnormalities.	J Allergy Clin Immunol.
atopic dermatitis	PTP4A3	Expression profiling by array, count, 3 development stage, 2 disease state, 11 individual sets	GPL570	GSE32924 Samples	Nonlesional atopic dermatitis skin is characterized by broad terminal differentiation defects and variable immune abnormalities.	J Allergy Clin Immunol.
atopic dermatitis	NA	Expression profiling by array, count, 3 development stage, 2 disease state, 11 individual sets	GPL570	GSE32924 Samples	Nonlesional atopic dermatitis skin is characterized by broad terminal differentiation defects and variable immune abnormalities.	J Allergy Clin Immunol.
atopic dermatitis	NA	Expression profiling by array, count, 3 development stage, 2 disease state, 11 individual sets	GPL570	GSE32924 Samples	Nonlesional atopic dermatitis skin is characterized by broad terminal differentiation defects and variable immune abnormalities.	J Allergy Clin Immunol.
atopic dermatitis	SFRS15	Expression profiling by array, count, 3 development stage, 2 disease state, 11 individual sets	GPL570	GSE32924 Samples	Nonlesional atopic dermatitis skin is characterized by broad terminal differentiation defects and variable immune abnormalities.	J Allergy Clin Immunol.
atopic dermatitis	STAG1	Expression profiling by array, count, 3 development stage, 2 disease state, 11 individual sets	GPL570	GSE32924 Samples	Nonlesional atopic dermatitis skin is characterized by broad terminal differentiation defects and variable immune abnormalities.	J Allergy Clin Immunol.
atopic dermatitis	NA	Expression profiling by array, count, 3 development stage, 2 disease state, 11 individual sets	GPL570	GSE32924 Samples	Nonlesional atopic dermatitis skin is characterized by broad terminal differentiation defects and variable immune abnormalities.	J Allergy Clin Immunol.
atopic dermatitis	CORO6	Expression profiling by array, count, 3 development stage, 2 disease state, 11 individual sets	GPL570	GSE32924 Samples	Nonlesional atopic dermatitis skin is characterized by broad terminal differentiation defects and variable immune abnormalities.	J Allergy Clin Immunol.
atopic dermatitis	SLC38A9	Expression profiling by array, count, 3 development stage, 2 disease state, 11 individual sets	GPL570	GSE32924 Samples	Nonlesional atopic dermatitis skin is characterized by broad terminal differentiation defects and variable immune abnormalities.	J Allergy Clin Immunol.
atopic dermatitis	NA	Expression profiling by array, count, 3 development stage, 2 disease state, 11 individual sets	GPL570	GSE32924 Samples	Nonlesional atopic dermatitis skin is characterized by broad terminal differentiation defects and variable immune abnormalities.	J Allergy Clin Immunol.
atopic dermatitis	NA	Expression profiling by array, count, 3 development stage, 2 disease state, 11 individual sets	GPL570	GSE32924 Samples	Nonlesional atopic dermatitis skin is characterized by broad terminal differentiation defects and variable immune abnormalities.	J Allergy Clin Immunol.
atopic dermatitis	NA	Expression profiling by array, count, 3 development stage, 2 disease state, 11 individual sets	GPL570	GSE32924 Samples	Nonlesional atopic dermatitis skin is characterized by broad terminal differentiation defects and variable immune abnormalities.	J Allergy Clin Immunol.
atopic dermatitis	PTGR1	Expression profiling by array, count, 3 development stage, 2 disease state, 11 individual sets	GPL570	GSE32924 Samples	Nonlesional atopic dermatitis skin is characterized by broad terminal differentiation defects and variable immune abnormalities.	J Allergy Clin Immunol.
atopic dermatitis	CTTN	Expression profiling by array, count, 3 development stage, 2 disease state, 11 individual sets	GPL570	GSE32924 Samples	Nonlesional atopic dermatitis skin is characterized by broad terminal differentiation defects and variable immune abnormalities.	J Allergy Clin Immunol.
atopic dermatitis	NA	Expression profiling by array, count, 3 development stage, 2 disease state, 11 individual sets	GPL570	GSE32924 Samples	Nonlesional atopic dermatitis skin is characterized by broad terminal differentiation defects and variable immune abnormalities.	J Allergy Clin Immunol.
atopic dermatitis	NA	Expression profiling by array, count, 3 development stage, 2 disease state, 11 individual sets	GPL570	GSE32924 Samples	Nonlesional atopic dermatitis skin is characterized by broad terminal differentiation defects and variable immune abnormalities.	J Allergy Clin Immunol.

atopic dermatitis	NA	Expression profiling by array, count, 3 development stage, 2 disease state, 11 individual sets	GPL570	GSE32924 Samples	Nonlesional atopic dermatitis skin is characterized by broad terminal differentiation defects and variable immune abnormalities.	J Allergy Clin Immunol.
atopic dermatitis	NA	Expression profiling by array, count, 3 development stage, 2 disease state, 11 individual sets	GPL570	GSE32924 Samples	Nonlesional atopic dermatitis skin is characterized by broad terminal differentiation defects and variable immune abnormalities.	J Allergy Clin Immunol.
atopic dermatitis	NA	Expression profiling by array, count, 3 development stage, 2 disease state, 11 individual sets	GPL570	GSE32924 Samples	Nonlesional atopic dermatitis skin is characterized by broad terminal differentiation defects and variable immune abnormalities.	J Allergy Clin Immunol.
atopic dermatitis	GRB10	Expression profiling by array, count, 3 development stage, 2 disease state, 11 individual sets	GPL570	GSE32924 Samples	Nonlesional atopic dermatitis skin is characterized by broad terminal differentiation defects and variable immune abnormalities.	J Allergy Clin Immunol.
atopic dermatitis	NA	Expression profiling by array, count, 3 development stage, 2 disease state, 11 individual sets	GPL570	GSE32924 Samples	Nonlesional atopic dermatitis skin is characterized by broad terminal differentiation defects and variable immune abnormalities.	J Allergy Clin Immunol.
atopic dermatitis	ZNF37B	Expression profiling by array, count, 3 development stage, 2 disease state, 11 individual sets	GPL570	GSE32924 Samples	Nonlesional atopic dermatitis skin is characterized by broad terminal differentiation defects and variable immune abnormalities.	J Allergy Clin Immunol.
atopic dermatitis	RBM6	Expression profiling by array, count, 3 development stage, 2 disease state, 11 individual sets	GPL570	GSE32924 Samples	Nonlesional atopic dermatitis skin is characterized by broad terminal differentiation defects and variable immune abnormalities.	J Allergy Clin Immunol.
atopic dermatitis	TULP4	Expression profiling by array, count, 3 development stage, 2 disease state, 11 individual sets	GPL570	GSE32924 Samples	Nonlesional atopic dermatitis skin is characterized by broad terminal differentiation defects and variable immune abnormalities.	J Allergy Clin Immunol.
atopic dermatitis	NA	Expression profiling by array, count, 3 development stage, 2 disease state, 11 individual sets	GPL570	GSE32924 Samples	Nonlesional atopic dermatitis skin is characterized by broad terminal differentiation defects and variable immune abnormalities.	J Allergy Clin Immunol.
atopic dermatitis	CASZ1	Expression profiling by array, count, 3 development stage, 2 disease state, 11 individual sets	GPL570	GSE32924 Samples	Nonlesional atopic dermatitis skin is characterized by broad terminal differentiation defects and variable immune abnormalities.	J Allergy Clin Immunol.
atopic dermatitis	DENND1B	Expression profiling by array, count, 3 development stage, 2 disease state, 11 individual sets	GPL570	GSE32924 Samples	Nonlesional atopic dermatitis skin is characterized by broad terminal differentiation defects and variable immune abnormalities.	J Allergy Clin Immunol.
atopic dermatitis	HELLS	Expression profiling by array, count, 3 development stage, 2 disease state, 11 individual sets	GPL570	GSE32924 Samples	Nonlesional atopic dermatitis skin is characterized by broad terminal differentiation defects and variable immune abnormalities.	J Allergy Clin Immunol.
atopic dermatitis	USP36	Expression profiling by array, count, 3 development stage, 2 disease state, 11 individual sets	GPL570	GSE32924 Samples	Nonlesional atopic dermatitis skin is characterized by broad terminal differentiation defects and variable immune abnormalities.	J Allergy Clin Immunol.
atopic dermatitis	NA	Expression profiling by array, count, 3 development stage, 2 disease state, 11 individual sets	GPL570	GSE32924 Samples	Nonlesional atopic dermatitis skin is characterized by broad terminal differentiation defects and variable immune abnormalities.	J Allergy Clin Immunol.
atopic dermatitis	NA	Expression profiling by array, count, 3 development stage, 2 disease state, 11 individual sets	GPL570	GSE32924 Samples	Nonlesional atopic dermatitis skin is characterized by broad terminal differentiation defects and variable immune abnormalities.	J Allergy Clin Immunol.
atopic dermatitis	SOX4	Expression profiling by array, count, 3 development stage, 2 disease state, 11 individual sets	GPL570	GSE32924 Samples	Nonlesional atopic dermatitis skin is characterized by broad terminal differentiation defects and variable immune abnormalities.	J Allergy Clin Immunol.
atopic dermatitis	RASSF4	Expression profiling by array, count, 3 development stage, 2 disease state, 11 individual sets	GPL570	GSE32924 Samples	Nonlesional atopic dermatitis skin is characterized by broad terminal differentiation defects and variable immune abnormalities.	J Allergy Clin Immunol.
atopic dermatitis	EIF3C	Expression profiling by array, count, 3 development stage, 2 disease state, 11 individual sets	GPL570	GSE32924 Samples	Nonlesional atopic dermatitis skin is characterized by broad terminal differentiation defects and variable immune abnormalities.	J Allergy Clin Immunol.
atopic dermatitis	NA	Expression profiling by array, count, 3 development stage, 2 disease state, 11 individual sets	GPL570	GSE32924 Samples	Nonlesional atopic dermatitis skin is characterized by broad terminal differentiation defects and variable immune abnormalities.	J Allergy Clin Immunol.
atopic dermatitis	GGNBP2	Expression profiling by array, count, 3 development stage, 2 disease state, 11 individual sets	GPL570	GSE32924 Samples	Nonlesional atopic dermatitis skin is characterized by broad terminal differentiation defects and variable immune abnormalities.	J Allergy Clin Immunol.
atopic dermatitis	C5ORF28	Expression profiling by array, count, 3 development stage, 2 disease state, 11 individual sets	GPL570	GSE32924 Samples	Nonlesional atopic dermatitis skin is characterized by broad terminal differentiation defects and variable immune abnormalities.	J Allergy Clin Immunol.
atopic dermatitis	NA	Expression profiling by array, count, 3 development stage, 2 disease state, 11 individual sets	GPL570	GSE32924 Samples	Nonlesional atopic dermatitis skin is characterized by broad terminal differentiation defects and variable immune abnormalities.	J Allergy Clin Immunol.
atopic dermatitis	PSIP1	Expression profiling by array, count, 3 development stage, 2 disease state, 11 individual sets	GPL570	GSE32924 Samples	Nonlesional atopic dermatitis skin is characterized by broad terminal differentiation defects and variable immune abnormalities.	J Allergy Clin Immunol.
atopic dermatitis	LOC440288	Expression profiling by array, count, 3 development stage, 2 disease state, 11 individual sets	GPL570	GSE32924 Samples	Nonlesional atopic dermatitis skin is characterized by broad terminal differentiation defects and variable immune abnormalities.	J Allergy Clin Immunol.
atopic dermatitis	SARS2	Expression profiling by array, count, 3 development stage, 2 disease state, 11 individual sets	GPL570	GSE32924 Samples	Nonlesional atopic dermatitis skin is characterized by broad terminal differentiation defects and variable immune abnormalities.	J Allergy Clin Immunol.
atopic dermatitis	SLC19A1	Expression profiling by array, count, 3 development stage, 2 disease state, 11 individual sets	GPL570	GSE32924 Samples	Nonlesional atopic dermatitis skin is characterized by broad terminal differentiation defects and variable immune abnormalities.	J Allergy Clin Immunol.
atopic dermatitis	PPCDC	Expression profiling by array, count, 3 development stage, 2 disease state, 11 individual sets	GPL570	GSE32924 Samples	Nonlesional atopic dermatitis skin is characterized by broad terminal differentiation defects and variable immune abnormalities.	J Allergy Clin Immunol.
atopic dermatitis	STX4	Expression profiling by array, count, 3 development stage, 2 disease state, 11 individual sets	GPL570	GSE32924 Samples	Nonlesional atopic dermatitis skin is characterized by broad terminal differentiation defects and variable immune abnormalities.	J Allergy Clin Immunol.
atopic dermatitis	KLHL22	Expression profiling by array, count, 3 development stage, 2 disease state, 11 individual sets	GPL570	GSE32924 Samples	Nonlesional atopic dermatitis skin is characterized by broad terminal differentiation defects and variable immune abnormalities.	J Allergy Clin Immunol.
atopic dermatitis	C19ORF28	Expression profiling by array, count, 3 development stage, 2 disease state, 11 individual sets	GPL570	GSE32924 Samples	Nonlesional atopic dermatitis skin is characterized by broad terminal differentiation defects and variable immune abnormalities.	J Allergy Clin Immunol.
atopic dermatitis	NA	Expression profiling by array, count, 3 development stage, 2 disease state, 11 individual sets	GPL570	GSE32924 Samples	Nonlesional atopic dermatitis skin is characterized by broad terminal differentiation defects and variable immune abnormalities.	J Allergy Clin Immunol.
atopic dermatitis	CHAF1A	Expression profiling by array, count, 3 development stage, 2 disease state, 11 individual sets	GPL570	GSE32924 Samples	Nonlesional atopic dermatitis skin is characterized by broad terminal differentiation defects and variable immune abnormalities.	J Allergy Clin Immunol.
atopic dermatitis	TUBGCP6	Expression profiling by array, count, 3 development stage, 2 disease state, 11 individual sets	GPL570	GSE32924 Samples	Nonlesional atopic dermatitis skin is characterized by broad terminal differentiation defects and variable immune abnormalities.	J Allergy Clin Immunol.
atopic dermatitis	NA	Expression profiling by array, count, 3 development stage, 2 disease state, 11 individual sets	GPL570	GSE32924 Samples	Nonlesional atopic dermatitis skin is characterized by broad terminal differentiation defects and variable immune abnormalities.	J Allergy Clin Immunol.
atopic dermatitis	FBXL6	Expression profiling by array, count, 3 development stage, 2 disease state, 11 individual sets	GPL570	GSE32924 Samples	Nonlesional atopic dermatitis skin is characterized by broad terminal differentiation defects and variable immune abnormalities.	J Allergy Clin Immunol.
atopic dermatitis	C3ORF62	Expression profiling by array, count, 3 development stage, 2 disease state, 11 individual sets	GPL570	GSE32924 Samples	Nonlesional atopic dermatitis skin is characterized by broad terminal differentiation defects and variable immune abnormalities.	J Allergy Clin Immunol.
atopic dermatitis	CCDC142	Expression profiling by array, count, 3 development stage, 2 disease state, 11 individual sets	GPL570	GSE32924 Samples	Nonlesional atopic dermatitis skin is characterized by broad terminal differentiation defects and variable immune abnormalities.	J Allergy Clin Immunol.
atopic dermatitis	LOC100131096	Expression profiling by array, count, 3 development stage, 2 disease state, 11 individual sets	GPL570	GSE32924 Samples	Nonlesional atopic dermatitis skin is characterized by broad terminal differentiation defects and variable immune abnormalities.	J Allergy Clin Immunol.
atopic dermatitis	ABCC5	Expression profiling by array, count, 3 development stage, 2 disease state, 11 individual sets	GPL570	GSE32924 Samples	Nonlesional atopic dermatitis skin is characterized by broad terminal differentiation defects and variable immune abnormalities.	J Allergy Clin Immunol.
atopic dermatitis	HSF4	Expression profiling by array, count, 3 development stage, 2 disease state, 11 individual sets	GPL570	GSE32924 Samples	Nonlesional atopic dermatitis skin is characterized by broad terminal differentiation defects and variable immune abnormalities.	J Allergy Clin Immunol.
atopic dermatitis	C1ORF159	Expression profiling by array, count, 3 development stage, 2 disease state, 11 individual sets	GPL570	GSE32924 Samples	Nonlesional atopic dermatitis skin is characterized by broad terminal differentiation defects and variable immune abnormalities.	J Allergy Clin Immunol.

atopic dermatitis	TGIF2	Expression profiling by array, count, 3 development stage, 2 disease state, 11 individual sets	GPL570	GSE32924 Samples	Nonlesional atopic dermatitis skin is characterized by broad terminal differentiation defects and variable immune abnormalities.	J Allergy Clin Immunol.
atopic dermatitis	CCDC61	Expression profiling by array, count, 3 development stage, 2 disease state, 11 individual sets	GPL570	GSE32924 Samples	Nonlesional atopic dermatitis skin is characterized by broad terminal differentiation defects and variable immune abnormalities.	J Allergy Clin Immunol.
atopic dermatitis	NA	Expression profiling by array, count, 3 development stage, 2 disease state, 11 individual sets	GPL570	GSE32924 Samples	Nonlesional atopic dermatitis skin is characterized by broad terminal differentiation defects and variable immune abnormalities.	J Allergy Clin Immunol.
atopic dermatitis	RNASEH2C	Expression profiling by array, count, 3 development stage, 2 disease state, 11 individual sets	GPL570	GSE32924 Samples	Nonlesional atopic dermatitis skin is characterized by broad terminal differentiation defects and variable immune abnormalities.	J Allergy Clin Immunol.
atopic dermatitis	DGCR8	Expression profiling by array, count, 3 development stage, 2 disease state, 11 individual sets	GPL570	GSE32924 Samples	Nonlesional atopic dermatitis skin is characterized by broad terminal differentiation defects and variable immune abnormalities.	J Allergy Clin Immunol.
atopic dermatitis	ABCB8	Expression profiling by array, count, 3 development stage, 2 disease state, 11 individual sets	GPL570	GSE32924 Samples	Nonlesional atopic dermatitis skin is characterized by broad terminal differentiation defects and variable immune abnormalities.	J Allergy Clin Immunol.
atopic dermatitis	ROGDI	Expression profiling by array, count, 3 development stage, 2 disease state, 11 individual sets	GPL570	GSE32924 Samples	Nonlesional atopic dermatitis skin is characterized by broad terminal differentiation defects and variable immune abnormalities.	J Allergy Clin Immunol.
atopic dermatitis	ZBTB42	Expression profiling by array, count, 3 development stage, 2 disease state, 11 individual sets	GPL570	GSE32924 Samples	Nonlesional atopic dermatitis skin is characterized by broad terminal differentiation defects and variable immune abnormalities.	J Allergy Clin Immunol.
atopic dermatitis	RAB15	Expression profiling by array, count, 3 development stage, 2 disease state, 11 individual sets	GPL570	GSE32924 Samples	Nonlesional atopic dermatitis skin is characterized by broad terminal differentiation defects and variable immune abnormalities.	J Allergy Clin Immunol.
atopic dermatitis	RAB15	Expression profiling by array, count, 3 development stage, 2 disease state, 11 individual sets	GPL570	GSE32924 Samples	Nonlesional atopic dermatitis skin is characterized by broad terminal differentiation defects and variable immune abnormalities.	J Allergy Clin Immunol.
atopic dermatitis	NA	Expression profiling by array, count, 3 development stage, 2 disease state, 11 individual sets	GPL570	GSE32924 Samples	Nonlesional atopic dermatitis skin is characterized by broad terminal differentiation defects and variable immune abnormalities.	J Allergy Clin Immunol.
atopic dermatitis	OSTM1	Expression profiling by array, count, 3 development stage, 2 disease state, 11 individual sets	GPL570	GSE32924 Samples	Nonlesional atopic dermatitis skin is characterized by broad terminal differentiation defects and variable immune abnormalities.	J Allergy Clin Immunol.
atopic dermatitis	NA	Expression profiling by array, count, 3 development stage, 2 disease state, 11 individual sets	GPL570	GSE32924 Samples	Nonlesional atopic dermatitis skin is characterized by broad terminal differentiation defects and variable immune abnormalities.	J Allergy Clin Immunol.
atopic dermatitis	NA	Expression profiling by array, count, 3 development stage, 2 disease state, 11 individual sets	GPL570	GSE32924 Samples	Nonlesional atopic dermatitis skin is characterized by broad terminal differentiation defects and variable immune abnormalities.	J Allergy Clin Immunol.
atopic dermatitis	NA	Expression profiling by array, count, 3 development stage, 2 disease state, 11 individual sets	GPL570	GSE32924 Samples	Nonlesional atopic dermatitis skin is characterized by broad terminal differentiation defects and variable immune abnormalities.	J Allergy Clin Immunol.
atopic dermatitis	FLJ00049	Expression profiling by array, count, 3 development stage, 2 disease state, 11 individual sets	GPL570	GSE32924 Samples	Nonlesional atopic dermatitis skin is characterized by broad terminal differentiation defects and variable immune abnormalities.	J Allergy Clin Immunol.
atopic dermatitis	NA	Expression profiling by array, count, 3 development stage, 2 disease state, 11 individual sets	GPL570	GSE32924 Samples	Nonlesional atopic dermatitis skin is characterized by broad terminal differentiation defects and variable immune abnormalities.	J Allergy Clin Immunol.
atopic dermatitis	NA	Expression profiling by array, count, 3 development stage, 2 disease state, 11 individual sets	GPL570	GSE32924 Samples	Nonlesional atopic dermatitis skin is characterized by broad terminal differentiation defects and variable immune abnormalities.	J Allergy Clin Immunol.
atopic dermatitis	ODF3B	Expression profiling by array, count, 3 development stage, 2 disease state, 11 individual sets	GPL570	GSE32924 Samples	Nonlesional atopic dermatitis skin is characterized by broad terminal differentiation defects and variable immune abnormalities.	J Allergy Clin Immunol.
atopic dermatitis	NA	Expression profiling by array, count, 3 development stage, 2 disease state, 11 individual sets	GPL570	GSE32924 Samples	Nonlesional atopic dermatitis skin is characterized by broad terminal differentiation defects and variable immune abnormalities.	J Allergy Clin Immunol.
atopic dermatitis	ITCH	Expression profiling by array, count, 3 development stage, 2 disease state, 11 individual sets	GPL570	GSE32924 Samples	Nonlesional atopic dermatitis skin is characterized by broad terminal differentiation defects and variable immune abnormalities.	J Allergy Clin Immunol.
atopic dermatitis	EGR1	Expression profiling by array, count, 3 development stage, 2 disease state, 11 individual sets	GPL570	GSE32924 Samples	Nonlesional atopic dermatitis skin is characterized by broad terminal differentiation defects and variable immune abnormalities.	J Allergy Clin Immunol.
atopic dermatitis	MRTO4	Expression profiling by array, count, 3 development stage, 2 disease state, 11 individual sets	GPL570	GSE32924 Samples	Nonlesional atopic dermatitis skin is characterized by broad terminal differentiation defects and variable immune abnormalities.	J Allergy Clin Immunol.
atopic dermatitis	GGT1	Expression profiling by array, count, 3 development stage, 2 disease state, 11 individual sets	GPL570	GSE32924 Samples	Nonlesional atopic dermatitis skin is characterized by broad terminal differentiation defects and variable immune abnormalities.	J Allergy Clin Immunol.
atopic dermatitis	GGT1	Expression profiling by array, count, 3 development stage, 2 disease state, 11 individual sets	GPL570	GSE32924 Samples	Nonlesional atopic dermatitis skin is characterized by broad terminal differentiation defects and variable immune abnormalities.	J Allergy Clin Immunol.
atopic dermatitis	ELF3	Expression profiling by array, count, 3 development stage, 2 disease state, 11 individual sets	GPL570	GSE32924 Samples	Nonlesional atopic dermatitis skin is characterized by broad terminal differentiation defects and variable immune abnormalities.	J Allergy Clin Immunol.
atopic dermatitis	PHLDA1	Expression profiling by array, count, 3 development stage, 2 disease state, 11 individual sets	GPL570	GSE32924 Samples	Nonlesional atopic dermatitis skin is characterized by broad terminal differentiation defects and variable immune abnormalities.	J Allergy Clin Immunol.
atopic dermatitis	ABP1	Expression profiling by array, count, 3 development stage, 2 disease state, 11 individual sets	GPL570	GSE32924 Samples	Nonlesional atopic dermatitis skin is characterized by broad terminal differentiation defects and variable immune abnormalities.	J Allergy Clin Immunol.
atopic dermatitis	ZNF618	Expression profiling by array, count, 3 development stage, 2 disease state, 11 individual sets	GPL570	GSE32924 Samples	Nonlesional atopic dermatitis skin is characterized by broad terminal differentiation defects and variable immune abnormalities.	J Allergy Clin Immunol.
atopic dermatitis	NA	Expression profiling by array, count, 3 development stage, 2 disease state, 11 individual sets	GPL570	GSE32924 Samples	Nonlesional atopic dermatitis skin is characterized by broad terminal differentiation defects and variable immune abnormalities.	J Allergy Clin Immunol.
atopic dermatitis	NA	Expression profiling by array, count, 3 development stage, 2 disease state, 11 individual sets	GPL570	GSE32924 Samples	Nonlesional atopic dermatitis skin is characterized by broad terminal differentiation defects and variable immune abnormalities.	J Allergy Clin Immunol.
atopic dermatitis	PDK3	Expression profiling by array, count, 3 development stage, 2 disease state, 11 individual sets	GPL570	GSE32924 Samples	Nonlesional atopic dermatitis skin is characterized by broad terminal differentiation defects and variable immune abnormalities.	J Allergy Clin Immunol.
atopic dermatitis	PTCD2	Expression profiling by array, count, 3 development stage, 2 disease state, 11 individual sets	GPL570	GSE32924 Samples	Nonlesional atopic dermatitis skin is characterized by broad terminal differentiation defects and variable immune abnormalities.	J Allergy Clin Immunol.
atopic dermatitis	NA	Expression profiling by array, count, 3 development stage, 2 disease state, 11 individual sets	GPL570	GSE32924 Samples	Nonlesional atopic dermatitis skin is characterized by broad terminal differentiation defects and variable immune abnormalities.	J Allergy Clin Immunol.
atopic dermatitis	CD9	Expression profiling by array, count, 3 development stage, 2 disease state, 11 individual sets	GPL570	GSE32924 Samples	Nonlesional atopic dermatitis skin is characterized by broad terminal differentiation defects and variable immune abnormalities.	J Allergy Clin Immunol.
atopic dermatitis	NA	Expression profiling by array, count, 3 development stage, 2 disease state, 11 individual sets	GPL570	GSE32924 Samples	Nonlesional atopic dermatitis skin is characterized by broad terminal differentiation defects and variable immune abnormalities.	J Allergy Clin Immunol.
atopic dermatitis	NA	Expression profiling by array, count, 3 development stage, 2 disease state, 11 individual sets	GPL570	GSE32924 Samples	Nonlesional atopic dermatitis skin is characterized by broad terminal differentiation defects and variable immune abnormalities.	J Allergy Clin Immunol.
atopic dermatitis	GPRC5A	Expression profiling by array, count, 3 development stage, 2 disease state, 11 individual sets	GPL570	GSE32924 Samples	Nonlesional atopic dermatitis skin is characterized by broad terminal differentiation defects and variable immune abnormalities.	J Allergy Clin Immunol.
atopic dermatitis	NA	Expression profiling by array, count, 3 development stage, 2 disease state, 11 individual sets	GPL570	GSE32924 Samples	Nonlesional atopic dermatitis skin is characterized by broad terminal differentiation defects and variable immune abnormalities.	J Allergy Clin Immunol.
atopic dermatitis	NA	Expression profiling by array, count, 3 development stage, 2 disease state, 11 individual sets	GPL570	GSE32924 Samples	Nonlesional atopic dermatitis skin is characterized by broad terminal differentiation defects and variable immune abnormalities.	J Allergy Clin Immunol.
atopic dermatitis	STXBP3	Expression profiling by array, count, 3 development stage, 2 disease state, 11 individual sets	GPL570	GSE32924 Samples	Nonlesional atopic dermatitis skin is characterized by broad terminal differentiation defects and variable immune abnormalities.	J Allergy Clin Immunol.

atopic dermatitis	NA	Expression profiling by array, count, 3 development stage, 2 disease state, 11 individual sets	GPL570	GSE32924 Samples	Nonlesional atopic dermatitis skin is characterized by broad terminal differentiation defects and variable immune abnormalities.	J Allergy Clin Immunol.
atopic dermatitis	NA	Expression profiling by array, count, 3 development stage, 2 disease state, 11 individual sets	GPL570	GSE32924 Samples	Nonlesional atopic dermatitis skin is characterized by broad terminal differentiation defects and variable immune abnormalities.	J Allergy Clin Immunol.
atopic dermatitis	SLC1A4	Expression profiling by array, count, 3 development stage, 2 disease state, 11 individual sets	GPL570	GSE32924 Samples	Nonlesional atopic dermatitis skin is characterized by broad terminal differentiation defects and variable immune abnormalities.	J Allergy Clin Immunol.
atopic dermatitis	NA	Expression profiling by array, count, 3 development stage, 2 disease state, 11 individual sets	GPL570	GSE32924 Samples	Nonlesional atopic dermatitis skin is characterized by broad terminal differentiation defects and variable immune abnormalities.	J Allergy Clin Immunol.
atopic dermatitis	NA	Expression profiling by array, count, 3 development stage, 2 disease state, 11 individual sets	GPL570	GSE32924 Samples	Nonlesional atopic dermatitis skin is characterized by broad terminal differentiation defects and variable immune abnormalities.	J Allergy Clin Immunol.
atopic dermatitis	CEL	Expression profiling by array, count, 3 development stage, 2 disease state, 11 individual sets	GPL570	GSE32924 Samples	Nonlesional atopic dermatitis skin is characterized by broad terminal differentiation defects and variable immune abnormalities.	J Allergy Clin Immunol.
atopic dermatitis	NA	Expression profiling by array, count, 3 development stage, 2 disease state, 11 individual sets	GPL570	GSE32924 Samples	Nonlesional atopic dermatitis skin is characterized by broad terminal differentiation defects and variable immune abnormalities.	J Allergy Clin Immunol.
atopic dermatitis	AASDH	Expression profiling by array, count, 3 development stage, 2 disease state, 11 individual sets	GPL570	GSE32924 Samples	Nonlesional atopic dermatitis skin is characterized by broad terminal differentiation defects and variable immune abnormalities.	J Allergy Clin Immunol.
atopic dermatitis	SMC5	Expression profiling by array, count, 3 development stage, 2 disease state, 11 individual sets	GPL570	GSE32924 Samples	Nonlesional atopic dermatitis skin is characterized by broad terminal differentiation defects and variable immune abnormalities.	J Allergy Clin Immunol.
atopic dermatitis	NUPL1	Expression profiling by array, count, 3 development stage, 2 disease state, 11 individual sets	GPL570	GSE32924 Samples	Nonlesional atopic dermatitis skin is characterized by broad terminal differentiation defects and variable immune abnormalities.	J Allergy Clin Immunol.
atopic dermatitis	VPS8	Expression profiling by array, count, 3 development stage, 2 disease state, 11 individual sets	GPL570	GSE32924 Samples	Nonlesional atopic dermatitis skin is characterized by broad terminal differentiation defects and variable immune abnormalities.	J Allergy Clin Immunol.
atopic dermatitis	CDC42BPA	Expression profiling by array, count, 3 development stage, 2 disease state, 11 individual sets	GPL570	GSE32924 Samples	Nonlesional atopic dermatitis skin is characterized by broad terminal differentiation defects and variable immune abnormalities.	J Allergy Clin Immunol.
atopic dermatitis	CALD1	Expression profiling by array, count, 3 development stage, 2 disease state, 11 individual sets	GPL570	GSE32924 Samples	Nonlesional atopic dermatitis skin is characterized by broad terminal differentiation defects and variable immune abnormalities.	J Allergy Clin Immunol.
atopic dermatitis	NA	Expression profiling by array, count, 3 development stage, 2 disease state, 11 individual sets	GPL570	GSE32924 Samples	Nonlesional atopic dermatitis skin is characterized by broad terminal differentiation defects and variable immune abnormalities.	J Allergy Clin Immunol.
atopic dermatitis	SPEN	Expression profiling by array, count, 3 development stage, 2 disease state, 11 individual sets	GPL570	GSE32924 Samples	Nonlesional atopic dermatitis skin is characterized by broad terminal differentiation defects and variable immune abnormalities.	J Allergy Clin Immunol.
atopic dermatitis	TOP1	Expression profiling by array, count, 3 development stage, 2 disease state, 11 individual sets	GPL570	GSE32924 Samples	Nonlesional atopic dermatitis skin is characterized by broad terminal differentiation defects and variable immune abnormalities.	J Allergy Clin Immunol.
atopic dermatitis	MEX3D	Expression profiling by array, count, 3 development stage, 2 disease state, 11 individual sets	GPL570	GSE32924 Samples	Nonlesional atopic dermatitis skin is characterized by broad terminal differentiation defects and variable immune abnormalities.	J Allergy Clin Immunol.
atopic dermatitis	YTHDC1	Expression profiling by array, count, 3 development stage, 2 disease state, 11 individual sets	GPL570	GSE32924 Samples	Nonlesional atopic dermatitis skin is characterized by broad terminal differentiation defects and variable immune abnormalities.	J Allergy Clin Immunol.
atopic dermatitis	CAND1	Expression profiling by array, count, 3 development stage, 2 disease state, 11 individual sets	GPL570	GSE32924 Samples	Nonlesional atopic dermatitis skin is characterized by broad terminal differentiation defects and variable immune abnormalities.	J Allergy Clin Immunol.
atopic dermatitis	RSRC1	Expression profiling by array, count, 3 development stage, 2 disease state, 11 individual sets	GPL570	GSE32924 Samples	Nonlesional atopic dermatitis skin is characterized by broad terminal differentiation defects and variable immune abnormalities.	J Allergy Clin Immunol.
atopic dermatitis	KLHDC10	Expression profiling by array, count, 3 development stage, 2 disease state, 11 individual sets	GPL570	GSE32924 Samples	Nonlesional atopic dermatitis skin is characterized by broad terminal differentiation defects and variable immune abnormalities.	J Allergy Clin Immunol.
atopic dermatitis	RBM8A	Expression profiling by array, count, 3 development stage, 2 disease state, 11 individual sets	GPL570	GSE32924 Samples	Nonlesional atopic dermatitis skin is characterized by broad terminal differentiation defects and variable immune abnormalities.	J Allergy Clin Immunol.
atopic dermatitis	SMG1	Expression profiling by array, count, 3 development stage, 2 disease state, 11 individual sets	GPL570	GSE32924 Samples	Nonlesional atopic dermatitis skin is characterized by broad terminal differentiation defects and variable immune abnormalities.	J Allergy Clin Immunol.
atopic dermatitis	ATRX	Expression profiling by array, count, 3 development stage, 2 disease state, 11 individual sets	GPL570	GSE32924 Samples	Nonlesional atopic dermatitis skin is characterized by broad terminal differentiation defects and variable immune abnormalities.	J Allergy Clin Immunol.
atopic dermatitis	EFNB2	Expression profiling by array, count, 3 development stage, 2 disease state, 11 individual sets	GPL570	GSE32924 Samples	Nonlesional atopic dermatitis skin is characterized by broad terminal differentiation defects and variable immune abnormalities.	J Allergy Clin Immunol.
atopic dermatitis	LRP8	Expression profiling by array, count, 3 development stage, 2 disease state, 11 individual sets	GPL570	GSE32924 Samples	Nonlesional atopic dermatitis skin is characterized by broad terminal differentiation defects and variable immune abnormalities.	J Allergy Clin Immunol.
atopic dermatitis	KLHL5	Expression profiling by array, count, 3 development stage, 2 disease state, 11 individual sets	GPL570	GSE32924 Samples	Nonlesional atopic dermatitis skin is characterized by broad terminal differentiation defects and variable immune abnormalities.	J Allergy Clin Immunol.
atopic dermatitis	FOXK2	Expression profiling by array, count, 3 development stage, 2 disease state, 11 individual sets	GPL570	GSE32924 Samples	Nonlesional atopic dermatitis skin is characterized by broad terminal differentiation defects and variable immune abnormalities.	J Allergy Clin Immunol.
atopic dermatitis	TBRG4	Expression profiling by array, count, 3 development stage, 2 disease state, 11 individual sets	GPL570	GSE32924 Samples	Nonlesional atopic dermatitis skin is characterized by broad terminal differentiation defects and variable immune abnormalities.	J Allergy Clin Immunol.
atopic dermatitis	MTG1	Expression profiling by array, count, 3 development stage, 2 disease state, 11 individual sets	GPL570	GSE32924 Samples	Nonlesional atopic dermatitis skin is characterized by broad terminal differentiation defects and variable immune abnormalities.	J Allergy Clin Immunol.
atopic dermatitis	ITIH4	Expression profiling by array, count, 3 development stage, 2 disease state, 11 individual sets	GPL570	GSE32924 Samples	Nonlesional atopic dermatitis skin is characterized by broad terminal differentiation defects and variable immune abnormalities.	J Allergy Clin Immunol.
atopic dermatitis	ATF7IP	Expression profiling by array, count, 3 development stage, 2 disease state, 11 individual sets	GPL570	GSE32924 Samples	Nonlesional atopic dermatitis skin is characterized by broad terminal differentiation defects and variable immune abnormalities.	J Allergy Clin Immunol.
atopic dermatitis	NA	Expression profiling by array, count, 3 development stage, 2 disease state, 11 individual sets	GPL570	GSE32924 Samples	Nonlesional atopic dermatitis skin is characterized by broad terminal differentiation defects and variable immune abnormalities.	J Allergy Clin Immunol.
atopic dermatitis	NA	Expression profiling by array, count, 3 development stage, 2 disease state, 11 individual sets	GPL570	GSE32924 Samples	Nonlesional atopic dermatitis skin is characterized by broad terminal differentiation defects and variable immune abnormalities.	J Allergy Clin Immunol.
atopic dermatitis	NA	Expression profiling by array, count, 3 development stage, 2 disease state, 11 individual sets	GPL570	GSE32924 Samples	Nonlesional atopic dermatitis skin is characterized by broad terminal differentiation defects and variable immune abnormalities.	J Allergy Clin Immunol.
atopic dermatitis	AKAP13	Expression profiling by array, count, 3 development stage, 2 disease state, 11 individual sets	GPL570	GSE32924 Samples	Nonlesional atopic dermatitis skin is characterized by broad terminal differentiation defects and variable immune abnormalities.	J Allergy Clin Immunol.
atopic dermatitis	BGLAP	Expression profiling by array, count, 3 development stage, 2 disease state, 11 individual sets	GPL570	GSE32924 Samples	Nonlesional atopic dermatitis skin is characterized by broad terminal differentiation defects and variable immune abnormalities.	J Allergy Clin Immunol.
atopic dermatitis	CD44	Expression profiling by array, count, 3 development stage, 2 disease state, 11 individual sets	GPL570	GSE32924 Samples	Nonlesional atopic dermatitis skin is characterized by broad terminal differentiation defects and variable immune abnormalities.	J Allergy Clin Immunol.
atopic dermatitis	NA	Expression profiling by array, count, 3 development stage, 2 disease state, 11 individual sets	GPL570	GSE32924 Samples	Nonlesional atopic dermatitis skin is characterized by broad terminal differentiation defects and variable immune abnormalities.	J Allergy Clin Immunol.
atopic dermatitis	GJC1	Expression profiling by array, count, 3 development stage, 2 disease state, 11 individual sets	GPL570	GSE32924 Samples	Nonlesional atopic dermatitis skin is characterized by broad terminal differentiation defects and variable immune abnormalities.	J Allergy Clin Immunol.
atopic dermatitis	CCL26	Expression profiling by array, count, 3 development stage, 2 disease state, 11 individual sets	GPL570	GSE32924 Samples	Nonlesional atopic dermatitis skin is characterized by broad terminal differentiation defects and variable immune abnormalities.	J Allergy Clin Immunol.

atopic dermatitis	CCL17	Expression profiling by array, count, 3 development stage, 2 disease state, 11 individual sets	GPL570	GSE32924 Samples	Nonlesional atopic dermatitis skin is characterized by broad terminal differentiation defects and variable immune abnormalities.	J Allergy Clin Immunol.
atopic dermatitis	FAM175A	Expression profiling by array, count, 3 development stage, 2 disease state, 11 individual sets	GPL570	GSE32924 Samples	Nonlesional atopic dermatitis skin is characterized by broad terminal differentiation defects and variable immune abnormalities.	J Allergy Clin Immunol.
atopic dermatitis	ZNF227	Expression profiling by array, count, 3 development stage, 2 disease state, 11 individual sets	GPL570	GSE32924 Samples	Nonlesional atopic dermatitis skin is characterized by broad terminal differentiation defects and variable immune abnormalities.	J Allergy Clin Immunol.
atopic dermatitis	NKAP	Expression profiling by array, count, 3 development stage, 2 disease state, 11 individual sets	GPL570	GSE32924 Samples	Nonlesional atopic dermatitis skin is characterized by broad terminal differentiation defects and variable immune abnormalities.	J Allergy Clin Immunol.
atopic dermatitis	RNF180	Expression profiling by array, count, 3 development stage, 2 disease state, 11 individual sets	GPL570	GSE32924 Samples	Nonlesional atopic dermatitis skin is characterized by broad terminal differentiation defects and variable immune abnormalities.	J Allergy Clin Immunol.
atopic dermatitis	ZNF160	Expression profiling by array, count, 3 development stage, 2 disease state, 11 individual sets	GPL570	GSE32924 Samples	Nonlesional atopic dermatitis skin is characterized by broad terminal differentiation defects and variable immune abnormalities.	J Allergy Clin Immunol.
atopic dermatitis	FRY	Expression profiling by array, count, 3 development stage, 2 disease state, 11 individual sets	GPL570	GSE32924 Samples	Nonlesional atopic dermatitis skin is characterized by broad terminal differentiation defects and variable immune abnormalities.	J Allergy Clin Immunol.
atopic dermatitis	CFI	Expression profiling by array, count, 3 development stage, 2 disease state, 11 individual sets	GPL570	GSE32924 Samples	Nonlesional atopic dermatitis skin is characterized by broad terminal differentiation defects and variable immune abnormalities.	J Allergy Clin Immunol.
atopic dermatitis	NA	Expression profiling by array, count, 3 development stage, 2 disease state, 11 individual sets	GPL570	GSE32924 Samples	Nonlesional atopic dermatitis skin is characterized by broad terminal differentiation defects and variable immune abnormalities.	J Allergy Clin Immunol.
atopic dermatitis	NA	Expression profiling by array, count, 3 development stage, 2 disease state, 11 individual sets	GPL570	GSE32924 Samples	Nonlesional atopic dermatitis skin is characterized by broad terminal differentiation defects and variable immune abnormalities.	J Allergy Clin Immunol.
atopic dermatitis	KLHL24	Expression profiling by array, count, 3 development stage, 2 disease state, 11 individual sets	GPL570	GSE32924 Samples	Nonlesional atopic dermatitis skin is characterized by broad terminal differentiation defects and variable immune abnormalities.	J Allergy Clin Immunol.
atopic dermatitis	NA	Expression profiling by array, count, 3 development stage, 2 disease state, 11 individual sets	GPL570	GSE32924 Samples	Nonlesional atopic dermatitis skin is characterized by broad terminal differentiation defects and variable immune abnormalities.	J Allergy Clin Immunol.
atopic dermatitis	CCDC126	Expression profiling by array, count, 3 development stage, 2 disease state, 11 individual sets	GPL570	GSE32924 Samples	Nonlesional atopic dermatitis skin is characterized by broad terminal differentiation defects and variable immune abnormalities.	J Allergy Clin Immunol.
atopic dermatitis	GPR98	Expression profiling by array, count, 3 development stage, 2 disease state, 11 individual sets	GPL570	GSE32924 Samples	Nonlesional atopic dermatitis skin is characterized by broad terminal differentiation defects and variable immune abnormalities.	J Allergy Clin Immunol.
atopic dermatitis	TTC3	Expression profiling by array, count, 3 development stage, 2 disease state, 11 individual sets	GPL570	GSE32924 Samples	Nonlesional atopic dermatitis skin is characterized by broad terminal differentiation defects and variable immune abnormalities.	J Allergy Clin Immunol.
atopic dermatitis	PRKAB1	Expression profiling by array, count, 3 development stage, 2 disease state, 11 individual sets	GPL570	GSE32924 Samples	Nonlesional atopic dermatitis skin is characterized by broad terminal differentiation defects and variable immune abnormalities.	J Allergy Clin Immunol.
atopic dermatitis	C3ORF17	Expression profiling by array, count, 3 development stage, 2 disease state, 11 individual sets	GPL570	GSE32924 Samples	Nonlesional atopic dermatitis skin is characterized by broad terminal differentiation defects and variable immune abnormalities.	J Allergy Clin Immunol.
atopic dermatitis	SLFN5	Expression profiling by array, count, 3 development stage, 2 disease state, 11 individual sets	GPL570	GSE32924 Samples	Nonlesional atopic dermatitis skin is characterized by broad terminal differentiation defects and variable immune abnormalities.	J Allergy Clin Immunol.
atopic dermatitis	GM2A	Expression profiling by array, count, 3 development stage, 2 disease state, 11 individual sets	GPL570	GSE32924 Samples	Nonlesional atopic dermatitis skin is characterized by broad terminal differentiation defects and variable immune abnormalities.	J Allergy Clin Immunol.
atopic dermatitis	IQGAP1	Expression profiling by array, count, 3 development stage, 2 disease state, 11 individual sets	GPL570	GSE32924 Samples	Nonlesional atopic dermatitis skin is characterized by broad terminal differentiation defects and variable immune abnormalities.	J Allergy Clin Immunol.
atopic dermatitis	TPM1	Expression profiling by array, count, 3 development stage, 2 disease state, 11 individual sets	GPL570	GSE32924 Samples	Nonlesional atopic dermatitis skin is characterized by broad terminal differentiation defects and variable immune abnormalities.	J Allergy Clin Immunol.
atopic dermatitis	NA	Expression profiling by array, count, 3 development stage, 2 disease state, 11 individual sets	GPL570	GSE32924 Samples	Nonlesional atopic dermatitis skin is characterized by broad terminal differentiation defects and variable immune abnormalities.	J Allergy Clin Immunol.
atopic dermatitis	PRKCB	Expression profiling by array, count, 3 development stage, 2 disease state, 11 individual sets	GPL570	GSE32924 Samples	Nonlesional atopic dermatitis skin is characterized by broad terminal differentiation defects and variable immune abnormalities.	J Allergy Clin Immunol.
atopic dermatitis	MEIS1	Expression profiling by array, count, 3 development stage, 2 disease state, 11 individual sets	GPL570	GSE32924 Samples	Nonlesional atopic dermatitis skin is characterized by broad terminal differentiation defects and variable immune abnormalities.	J Allergy Clin Immunol.
atopic dermatitis	NA	Expression profiling by array, count, 3 development stage, 2 disease state, 11 individual sets	GPL570	GSE32924 Samples	Nonlesional atopic dermatitis skin is characterized by broad terminal differentiation defects and variable immune abnormalities.	J Allergy Clin Immunol.
atopic dermatitis	NA	Expression profiling by array, count, 3 development stage, 2 disease state, 11 individual sets	GPL570	GSE32924 Samples	Nonlesional atopic dermatitis skin is characterized by broad terminal differentiation defects and variable immune abnormalities.	J Allergy Clin Immunol.
atopic dermatitis	USP31	Expression profiling by array, count, 3 development stage, 2 disease state, 11 individual sets	GPL570	GSE32924 Samples	Nonlesional atopic dermatitis skin is characterized by broad terminal differentiation defects and variable immune abnormalities.	J Allergy Clin Immunol.
atopic dermatitis	GLCCI1	Expression profiling by array, count, 3 development stage, 2 disease state, 11 individual sets	GPL570	GSE32924 Samples	Nonlesional atopic dermatitis skin is characterized by broad terminal differentiation defects and variable immune abnormalities.	J Allergy Clin Immunol.
atopic dermatitis	XG	Expression profiling by array, count, 3 development stage, 2 disease state, 11 individual sets	GPL570	GSE32924 Samples	Nonlesional atopic dermatitis skin is characterized by broad terminal differentiation defects and variable immune abnormalities.	J Allergy Clin Immunol.
atopic dermatitis	SDPR	Expression profiling by array, count, 3 development stage, 2 disease state, 11 individual sets	GPL570	GSE32924 Samples	Nonlesional atopic dermatitis skin is characterized by broad terminal differentiation defects and variable immune abnormalities.	J Allergy Clin Immunol.
atopic dermatitis	PTGFR	Expression profiling by array, count, 3 development stage, 2 disease state, 11 individual sets	GPL570	GSE32924 Samples	Nonlesional atopic dermatitis skin is characterized by broad terminal differentiation defects and variable immune abnormalities.	J Allergy Clin Immunol.
atopic dermatitis	RPP30	Expression profiling by array, count, 3 development stage, 2 disease state, 11 individual sets	GPL570	GSE32924 Samples	Nonlesional atopic dermatitis skin is characterized by broad terminal differentiation defects and variable immune abnormalities.	J Allergy Clin Immunol.
atopic dermatitis	DPYSL3	Expression profiling by array, count, 3 development stage, 2 disease state, 11 individual sets	GPL570	GSE32924 Samples	Nonlesional atopic dermatitis skin is characterized by broad terminal differentiation defects and variable immune abnormalities.	J Allergy Clin Immunol.
atopic dermatitis	LSS	Expression profiling by array, count, 3 development stage, 2 disease state, 11 individual sets	GPL570	GSE32924 Samples	Nonlesional atopic dermatitis skin is characterized by broad terminal differentiation defects and variable immune abnormalities.	J Allergy Clin Immunol.
atopic dermatitis	SMARCA2	Expression profiling by array, count, 3 development stage, 2 disease state, 11 individual sets	GPL570	GSE32924 Samples	Nonlesional atopic dermatitis skin is characterized by broad terminal differentiation defects and variable immune abnormalities.	J Allergy Clin Immunol.
atopic dermatitis	MFN2	Expression profiling by array, count, 3 development stage, 2 disease state, 11 individual sets	GPL570	GSE32924 Samples	Nonlesional atopic dermatitis skin is characterized by broad terminal differentiation defects and variable immune abnormalities.	J Allergy Clin Immunol.
atopic dermatitis	UBE4B	Expression profiling by array, count, 3 development stage, 2 disease state, 11 individual sets	GPL570	GSE32924 Samples	Nonlesional atopic dermatitis skin is characterized by broad terminal differentiation defects and variable immune abnormalities.	J Allergy Clin Immunol.
atopic dermatitis	UFM1	Expression profiling by array, count, 3 development stage, 2 disease state, 11 individual sets	GPL570	GSE32924 Samples	Nonlesional atopic dermatitis skin is characterized by broad terminal differentiation defects and variable immune abnormalities.	J Allergy Clin Immunol.
atopic dermatitis	PPP3R1	Expression profiling by array, count, 3 development stage, 2 disease state, 11 individual sets	GPL570	GSE32924 Samples	Nonlesional atopic dermatitis skin is characterized by broad terminal differentiation defects and variable immune abnormalities.	J Allergy Clin Immunol.
atopic dermatitis	GLE1	Expression profiling by array, count, 3 development stage, 2 disease state, 11 individual sets	GPL570	GSE32924 Samples	Nonlesional atopic dermatitis skin is characterized by broad terminal differentiation defects and variable immune abnormalities.	J Allergy Clin Immunol.
atopic dermatitis	CREBZF	Expression profiling by array, count, 3 development stage, 2 disease state, 11 individual sets	GPL570	GSE32924 Samples	Nonlesional atopic dermatitis skin is characterized by broad terminal differentiation defects and variable immune abnormalities.	J Allergy Clin Immunol.

atopic dermatitis	ARFIP1	Expression profiling by array, count, 3 development stage, 2 disease state, 11 individual sets	GPL570	GSE32924 Samples	Nonlesional atopic dermatitis skin is characterized by broad terminal differentiation defects and variable immune abnormalities.	J Allergy Clin Immunol.
atopic dermatitis	ZBTB6	Expression profiling by array, count, 3 development stage, 2 disease state, 11 individual sets	GPL570	GSE32924 Samples	Nonlesional atopic dermatitis skin is characterized by broad terminal differentiation defects and variable immune abnormalities.	J Allergy Clin Immunol.
atopic dermatitis	LRP12	Expression profiling by array, count, 3 development stage, 2 disease state, 11 individual sets	GPL570	GSE32924 Samples	Nonlesional atopic dermatitis skin is characterized by broad terminal differentiation defects and variable immune abnormalities.	J Allergy Clin Immunol.
atopic dermatitis	SNIP1	Expression profiling by array, count, 3 development stage, 2 disease state, 11 individual sets	GPL570	GSE32924 Samples	Nonlesional atopic dermatitis skin is characterized by broad terminal differentiation defects and variable immune abnormalities.	J Allergy Clin Immunol.
atopic dermatitis	RNFT1	Expression profiling by array, count, 3 development stage, 2 disease state, 11 individual sets	GPL570	GSE32924 Samples	Nonlesional atopic dermatitis skin is characterized by broad terminal differentiation defects and variable immune abnormalities.	J Allergy Clin Immunol.
atopic dermatitis	SGPP1	Expression profiling by array, count, 3 development stage, 2 disease state, 11 individual sets	GPL570	GSE32924 Samples	Nonlesional atopic dermatitis skin is characterized by broad terminal differentiation defects and variable immune abnormalities.	J Allergy Clin Immunol.
atopic dermatitis	PCGF5	Expression profiling by array, count, 3 development stage, 2 disease state, 11 individual sets	GPL570	GSE32924 Samples	Nonlesional atopic dermatitis skin is characterized by broad terminal differentiation defects and variable immune abnormalities.	J Allergy Clin Immunol.
atopic dermatitis	ADAM9	Expression profiling by array, count, 3 development stage, 2 disease state, 11 individual sets	GPL570	GSE32924 Samples	Nonlesional atopic dermatitis skin is characterized by broad terminal differentiation defects and variable immune abnormalities.	J Allergy Clin Immunol.
atopic dermatitis	WEE1	Expression profiling by array, count, 3 development stage, 2 disease state, 11 individual sets	GPL570	GSE32924 Samples	Nonlesional atopic dermatitis skin is characterized by broad terminal differentiation defects and variable immune abnormalities.	J Allergy Clin Immunol.
atopic dermatitis	EIF4E3	Expression profiling by array, count, 3 development stage, 2 disease state, 11 individual sets	GPL570	GSE32924 Samples	Nonlesional atopic dermatitis skin is characterized by broad terminal differentiation defects and variable immune abnormalities.	J Allergy Clin Immunol.
atopic dermatitis	COBLL1	Expression profiling by array, count, 3 development stage, 2 disease state, 11 individual sets	GPL570	GSE32924 Samples	Nonlesional atopic dermatitis skin is characterized by broad terminal differentiation defects and variable immune abnormalities.	J Allergy Clin Immunol.
atopic dermatitis	NT5E	Expression profiling by array, count, 3 development stage, 2 disease state, 11 individual sets	GPL570	GSE32924 Samples	Nonlesional atopic dermatitis skin is characterized by broad terminal differentiation defects and variable immune abnormalities.	J Allergy Clin Immunol.
atopic dermatitis	PVRL3	Expression profiling by array, count, 3 development stage, 2 disease state, 11 individual sets	GPL570	GSE32924 Samples	Nonlesional atopic dermatitis skin is characterized by broad terminal differentiation defects and variable immune abnormalities.	J Allergy Clin Immunol.
atopic dermatitis	ANGPTL2	Expression profiling by array, count, 3 development stage, 2 disease state, 11 individual sets	GPL570	GSE32924 Samples	Nonlesional atopic dermatitis skin is characterized by broad terminal differentiation defects and variable immune abnormalities.	J Allergy Clin Immunol.
atopic dermatitis	HHEX	Expression profiling by array, count, 3 development stage, 2 disease state, 11 individual sets	GPL570	GSE32924 Samples	Nonlesional atopic dermatitis skin is characterized by broad terminal differentiation defects and variable immune abnormalities.	J Allergy Clin Immunol.
atopic dermatitis	TUBGCP3	Expression profiling by array, count, 3 development stage, 2 disease state, 11 individual sets	GPL570	GSE32924 Samples	Nonlesional atopic dermatitis skin is characterized by broad terminal differentiation defects and variable immune abnormalities.	J Allergy Clin Immunol.
atopic dermatitis	RHOB	Expression profiling by array, count, 3 development stage, 2 disease state, 11 individual sets	GPL570	GSE32924 Samples	Nonlesional atopic dermatitis skin is characterized by broad terminal differentiation defects and variable immune abnormalities.	J Allergy Clin Immunol.
atopic dermatitis	ARPP19	Expression profiling by array, count, 3 development stage, 2 disease state, 11 individual sets	GPL570	GSE32924 Samples	Nonlesional atopic dermatitis skin is characterized by broad terminal differentiation defects and variable immune abnormalities.	J Allergy Clin Immunol.
atopic dermatitis	APC	Expression profiling by array, count, 3 development stage, 2 disease state, 11 individual sets	GPL570	GSE32924 Samples	Nonlesional atopic dermatitis skin is characterized by broad terminal differentiation defects and variable immune abnormalities.	J Allergy Clin Immunol.
atopic dermatitis	DNAJB4	Expression profiling by array, count, 3 development stage, 2 disease state, 11 individual sets	GPL570	GSE32924 Samples	Nonlesional atopic dermatitis skin is characterized by broad terminal differentiation defects and variable immune abnormalities.	J Allergy Clin Immunol.
atopic dermatitis	TOR1AIP1	Expression profiling by array, count, 3 development stage, 2 disease state, 11 individual sets	GPL570	GSE32924 Samples	Nonlesional atopic dermatitis skin is characterized by broad terminal differentiation defects and variable immune abnormalities.	J Allergy Clin Immunol.
atopic dermatitis	UTRN	Expression profiling by array, count, 3 development stage, 2 disease state, 11 individual sets	GPL570	GSE32924 Samples	Nonlesional atopic dermatitis skin is characterized by broad terminal differentiation defects and variable immune abnormalities.	J Allergy Clin Immunol.
atopic dermatitis	LMBR1	Expression profiling by array, count, 3 development stage, 2 disease state, 11 individual sets	GPL570	GSE32924 Samples	Nonlesional atopic dermatitis skin is characterized by broad terminal differentiation defects and variable immune abnormalities.	J Allergy Clin Immunol.
atopic dermatitis	ORC5L	Expression profiling by array, count, 3 development stage, 2 disease state, 11 individual sets	GPL570	GSE32924 Samples	Nonlesional atopic dermatitis skin is characterized by broad terminal differentiation defects and variable immune abnormalities.	J Allergy Clin Immunol.
atopic dermatitis	GPHN	Expression profiling by array, count, 3 development stage, 2 disease state, 11 individual sets	GPL570	GSE32924 Samples	Nonlesional atopic dermatitis skin is characterized by broad terminal differentiation defects and variable immune abnormalities.	J Allergy Clin Immunol.
atopic dermatitis	THUMPD3	Expression profiling by array, count, 3 development stage, 2 disease state, 11 individual sets	GPL570	GSE32924 Samples	Nonlesional atopic dermatitis skin is characterized by broad terminal differentiation defects and variable immune abnormalities.	J Allergy Clin Immunol.
atopic dermatitis	NXT2	Expression profiling by array, count, 3 development stage, 2 disease state, 11 individual sets	GPL570	GSE32924 Samples	Nonlesional atopic dermatitis skin is characterized by broad terminal differentiation defects and variable immune abnormalities.	J Allergy Clin Immunol.
atopic dermatitis	CAB39	Expression profiling by array, count, 3 development stage, 2 disease state, 11 individual sets	GPL570	GSE32924 Samples	Nonlesional atopic dermatitis skin is characterized by broad terminal differentiation defects and variable immune abnormalities.	J Allergy Clin Immunol.
atopic dermatitis	MKRN2	Expression profiling by array, count, 3 development stage, 2 disease state, 11 individual sets	GPL570	GSE32924 Samples	Nonlesional atopic dermatitis skin is characterized by broad terminal differentiation defects and variable immune abnormalities.	J Allergy Clin Immunol.
atopic dermatitis	CDC5L	Expression profiling by array, count, 3 development stage, 2 disease state, 11 individual sets	GPL570	GSE32924 Samples	Nonlesional atopic dermatitis skin is characterized by broad terminal differentiation defects and variable immune abnormalities.	J Allergy Clin Immunol.
atopic dermatitis	MSH6	Expression profiling by array, count, 3 development stage, 2 disease state, 11 individual sets	GPL570	GSE32924 Samples	Nonlesional atopic dermatitis skin is characterized by broad terminal differentiation defects and variable immune abnormalities.	J Allergy Clin Immunol.
atopic dermatitis	TANK	Expression profiling by array, count, 3 development stage, 2 disease state, 11 individual sets	GPL570	GSE32924 Samples	Nonlesional atopic dermatitis skin is characterized by broad terminal differentiation defects and variable immune abnormalities.	J Allergy Clin Immunol.
atopic dermatitis	ALG13	Expression profiling by array, count, 3 development stage, 2 disease state, 11 individual sets	GPL570	GSE32924 Samples	Nonlesional atopic dermatitis skin is characterized by broad terminal differentiation defects and variable immune abnormalities.	J Allergy Clin Immunol.
atopic dermatitis	DUSP5	Expression profiling by array, count, 3 development stage, 2 disease state, 11 individual sets	GPL570	GSE32924 Samples	Nonlesional atopic dermatitis skin is characterized by broad terminal differentiation defects and variable immune abnormalities.	J Allergy Clin Immunol.
atopic dermatitis	API5	Expression profiling by array, count, 3 development stage, 2 disease state, 11 individual sets	GPL570	GSE32924 Samples	Nonlesional atopic dermatitis skin is characterized by broad terminal differentiation defects and variable immune abnormalities.	J Allergy Clin Immunol.
atopic dermatitis	KPNA4	Expression profiling by array, count, 3 development stage, 2 disease state, 11 individual sets	GPL570	GSE32924 Samples	Nonlesional atopic dermatitis skin is characterized by broad terminal differentiation defects and variable immune abnormalities.	J Allergy Clin Immunol.
atopic dermatitis	ACTR2	Expression profiling by array, count, 3 development stage, 2 disease state, 11 individual sets	GPL570	GSE32924 Samples	Nonlesional atopic dermatitis skin is characterized by broad terminal differentiation defects and variable immune abnormalities.	J Allergy Clin Immunol.
atopic dermatitis	HSPA4	Expression profiling by array, count, 3 development stage, 2 disease state, 11 individual sets	GPL570	GSE32924 Samples	Nonlesional atopic dermatitis skin is characterized by broad terminal differentiation defects and variable immune abnormalities.	J Allergy Clin Immunol.
atopic dermatitis	SPAG9	Expression profiling by array, count, 3 development stage, 2 disease state, 11 individual sets	GPL570	GSE32924 Samples	Nonlesional atopic dermatitis skin is characterized by broad terminal differentiation defects and variable immune abnormalities.	J Allergy Clin Immunol.
atopic dermatitis	KPNA1	Expression profiling by array, count, 3 development stage, 2 disease state, 11 individual sets	GPL570	GSE32924 Samples	Nonlesional atopic dermatitis skin is characterized by broad terminal differentiation defects and variable immune abnormalities.	J Allergy Clin Immunol.
atopic dermatitis	USP30	Expression profiling by array, count, 3 development stage, 2 disease state, 11 individual sets	GPL570	GSE32924 Samples	Nonlesional atopic dermatitis skin is characterized by broad terminal differentiation defects and variable immune abnormalities.	J Allergy Clin Immunol.

atopic dermatitis	TET3	Expression profiling by array, count, 3 development stage, 2 disease state, 11 individual sets	GPL570	GSE32924 Samples	Nonlesional atopic dermatitis skin is characterized by broad terminal differentiation defects and variable immune abnormalities.	J Allergy Clin Immunol.
atopic dermatitis	UGCG	Expression profiling by array, count, 3 development stage, 2 disease state, 11 individual sets	GPL570	GSE32924 Samples	Nonlesional atopic dermatitis skin is characterized by broad terminal differentiation defects and variable immune abnormalities.	J Allergy Clin Immunol.
atopic dermatitis	IL1RN	Expression profiling by array, count, 3 development stage, 2 disease state, 11 individual sets	GPL570	GSE32924 Samples	Nonlesional atopic dermatitis skin is characterized by broad terminal differentiation defects and variable immune abnormalities.	J Allergy Clin Immunol.
atopic dermatitis	CSDA	Expression profiling by array, count, 3 development stage, 2 disease state, 11 individual sets	GPL570	GSE32924 Samples	Nonlesional atopic dermatitis skin is characterized by broad terminal differentiation defects and variable immune abnormalities.	J Allergy Clin Immunol.
atopic dermatitis	CPZ	Expression profiling by array, count, 3 development stage, 2 disease state, 11 individual sets	GPL570	GSE32924 Samples	Nonlesional atopic dermatitis skin is characterized by broad terminal differentiation defects and variable immune abnormalities.	J Allergy Clin Immunol.
atopic dermatitis	IGFL2	Expression profiling by array, count, 3 development stage, 2 disease state, 11 individual sets	GPL570	GSE32924 Samples	Nonlesional atopic dermatitis skin is characterized by broad terminal differentiation defects and variable immune abnormalities.	J Allergy Clin Immunol.
atopic dermatitis	AQP9	Expression profiling by array, count, 3 development stage, 2 disease state, 11 individual sets	GPL570	GSE32924 Samples	Nonlesional atopic dermatitis skin is characterized by broad terminal differentiation defects and variable immune abnormalities.	J Allergy Clin Immunol.
atopic dermatitis	RNASE7	Expression profiling by array, count, 3 development stage, 2 disease state, 11 individual sets	GPL570	GSE32924 Samples	Nonlesional atopic dermatitis skin is characterized by broad terminal differentiation defects and variable immune abnormalities.	J Allergy Clin Immunol.
atopic dermatitis	SLC1A6	Expression profiling by array, count, 3 development stage, 2 disease state, 11 individual sets	GPL570	GSE32924 Samples	Nonlesional atopic dermatitis skin is characterized by broad terminal differentiation defects and variable immune abnormalities.	J Allergy Clin Immunol.
atopic dermatitis	ANXA9	Expression profiling by array, count, 3 development stage, 2 disease state, 11 individual sets	GPL570	GSE32924 Samples	Nonlesional atopic dermatitis skin is characterized by broad terminal differentiation defects and variable immune abnormalities.	J Allergy Clin Immunol.
atopic dermatitis	LCE1E	Expression profiling by array, count, 3 development stage, 2 disease state, 11 individual sets	GPL570	GSE32924 Samples	Nonlesional atopic dermatitis skin is characterized by broad terminal differentiation defects and variable immune abnormalities.	J Allergy Clin Immunol.
atopic dermatitis	SLC46A2	Expression profiling by array, count, 3 development stage, 2 disease state, 11 individual sets	GPL570	GSE32924 Samples	Nonlesional atopic dermatitis skin is characterized by broad terminal differentiation defects and variable immune abnormalities.	J Allergy Clin Immunol.
atopic dermatitis	AADAC	Expression profiling by array, count, 3 development stage, 2 disease state, 11 individual sets	GPL570	GSE32924 Samples	Nonlesional atopic dermatitis skin is characterized by broad terminal differentiation defects and variable immune abnormalities.	J Allergy Clin Immunol.
atopic dermatitis	PSG4	Expression profiling by array, count, 3 development stage, 2 disease state, 11 individual sets	GPL570	GSE32924 Samples	Nonlesional atopic dermatitis skin is characterized by broad terminal differentiation defects and variable immune abnormalities.	J Allergy Clin Immunol.
atopic dermatitis	OGN	Expression profiling by array, count, 3 development stage, 2 disease state, 11 individual sets	GPL570	GSE32924 Samples	Nonlesional atopic dermatitis skin is characterized by broad terminal differentiation defects and variable immune abnormalities.	J Allergy Clin Immunol.
atopic dermatitis	NA	Expression profiling by array, count, 3 development stage, 2 disease state, 11 individual sets	GPL570	GSE32924 Samples	Nonlesional atopic dermatitis skin is characterized by broad terminal differentiation defects and variable immune abnormalities.	J Allergy Clin Immunol.
atopic dermatitis	TNKS2	Expression profiling by array, count, 3 development stage, 2 disease state, 11 individual sets	GPL570	GSE32924 Samples	Nonlesional atopic dermatitis skin is characterized by broad terminal differentiation defects and variable immune abnormalities.	J Allergy Clin Immunol.
atopic dermatitis	NA	Expression profiling by array, count, 3 development stage, 2 disease state, 11 individual sets	GPL570	GSE32924 Samples	Nonlesional atopic dermatitis skin is characterized by broad terminal differentiation defects and variable immune abnormalities.	J Allergy Clin Immunol.
atopic dermatitis	RNF146	Expression profiling by array, count, 3 development stage, 2 disease state, 11 individual sets	GPL570	GSE32924 Samples	Nonlesional atopic dermatitis skin is characterized by broad terminal differentiation defects and variable immune abnormalities.	J Allergy Clin Immunol.
atopic dermatitis	PXK	Expression profiling by array, count, 3 development stage, 2 disease state, 11 individual sets	GPL570	GSE32924 Samples	Nonlesional atopic dermatitis skin is characterized by broad terminal differentiation defects and variable immune abnormalities.	J Allergy Clin Immunol.
atopic dermatitis	SGCG	Expression profiling by array, count, 3 development stage, 2 disease state, 11 individual sets	GPL570	GSE32924 Samples	Nonlesional atopic dermatitis skin is characterized by broad terminal differentiation defects and variable immune abnormalities.	J Allergy Clin Immunol.
atopic dermatitis	IL17D	Expression profiling by array, count, 3 development stage, 2 disease state, 11 individual sets	GPL570	GSE32924 Samples	Nonlesional atopic dermatitis skin is characterized by broad terminal differentiation defects and variable immune abnormalities.	J Allergy Clin Immunol.
atopic dermatitis	LRRC2	Expression profiling by array, count, 3 development stage, 2 disease state, 11 individual sets	GPL570	GSE32924 Samples	Nonlesional atopic dermatitis skin is characterized by broad terminal differentiation defects and variable immune abnormalities.	J Allergy Clin Immunol.
atopic dermatitis	SLC18A2	Expression profiling by array, count, 3 development stage, 2 disease state, 11 individual sets	GPL570	GSE32924 Samples	Nonlesional atopic dermatitis skin is characterized by broad terminal differentiation defects and variable immune abnormalities.	J Allergy Clin Immunol.
atopic dermatitis	HPGDS	Expression profiling by array, count, 3 development stage, 2 disease state, 11 individual sets	GPL570	GSE32924 Samples	Nonlesional atopic dermatitis skin is characterized by broad terminal differentiation defects and variable immune abnormalities.	J Allergy Clin Immunol.
atopic dermatitis	IGSF10	Expression profiling by array, count, 3 development stage, 2 disease state, 11 individual sets	GPL570	GSE32924 Samples	Nonlesional atopic dermatitis skin is characterized by broad terminal differentiation defects and variable immune abnormalities.	J Allergy Clin Immunol.
atopic dermatitis	FAM70A	Expression profiling by array, count, 3 development stage, 2 disease state, 11 individual sets	GPL570	GSE32924 Samples	Nonlesional atopic dermatitis skin is characterized by broad terminal differentiation defects and variable immune abnormalities.	J Allergy Clin Immunol.
atopic dermatitis	PNN	Expression profiling by array, count, 3 development stage, 2 disease state, 11 individual sets	GPL570	GSE32924 Samples	Nonlesional atopic dermatitis skin is characterized by broad terminal differentiation defects and variable immune abnormalities.	J Allergy Clin Immunol.
atopic dermatitis	CHD2	Expression profiling by array, count, 3 development stage, 2 disease state, 11 individual sets	GPL570	GSE32924 Samples	Nonlesional atopic dermatitis skin is characterized by broad terminal differentiation defects and variable immune abnormalities.	J Allergy Clin Immunol.
atopic dermatitis	CHD2	Expression profiling by array, count, 3 development stage, 2 disease state, 11 individual sets	GPL570	GSE32924 Samples	Nonlesional atopic dermatitis skin is characterized by broad terminal differentiation defects and variable immune abnormalities.	J Allergy Clin Immunol.
atopic dermatitis	NA	Expression profiling by array, count, 3 development stage, 2 disease state, 11 individual sets	GPL570	GSE32924 Samples	Nonlesional atopic dermatitis skin is characterized by broad terminal differentiation defects and variable immune abnormalities.	J Allergy Clin Immunol.
atopic dermatitis	RGS22	Expression profiling by array, count, 3 development stage, 2 disease state, 11 individual sets	GPL570	GSE32924 Samples	Nonlesional atopic dermatitis skin is characterized by broad terminal differentiation defects and variable immune abnormalities.	J Allergy Clin Immunol.
atopic dermatitis	ESR1	Expression profiling by array, count, 3 development stage, 2 disease state, 11 individual sets	GPL570	GSE32924 Samples	Nonlesional atopic dermatitis skin is characterized by broad terminal differentiation defects and variable immune abnormalities.	J Allergy Clin Immunol.
atopic dermatitis	SVEP1	Expression profiling by array, count, 3 development stage, 2 disease state, 11 individual sets	GPL570	GSE32924 Samples	Nonlesional atopic dermatitis skin is characterized by broad terminal differentiation defects and variable immune abnormalities.	J Allergy Clin Immunol.
atopic dermatitis	CLDN23	Expression profiling by array, count, 3 development stage, 2 disease state, 11 individual sets	GPL570	GSE32924 Samples	Nonlesional atopic dermatitis skin is characterized by broad terminal differentiation defects and variable immune abnormalities.	J Allergy Clin Immunol.
atopic dermatitis	CYP2J2	Expression profiling by array, count, 3 development stage, 2 disease state, 11 individual sets	GPL570	GSE32924 Samples	Nonlesional atopic dermatitis skin is characterized by broad terminal differentiation defects and variable immune abnormalities.	J Allergy Clin Immunol.
atopic dermatitis	CTNNA1	Expression profiling by array, count, 3 development stage, 2 disease state, 11 individual sets	GPL570	GSE32924 Samples	Nonlesional atopic dermatitis skin is characterized by broad terminal differentiation defects and variable immune abnormalities.	J Allergy Clin Immunol.
atopic dermatitis	VAV3	Expression profiling by array, count, 3 development stage, 2 disease state, 11 individual sets	GPL570	GSE32924 Samples	Nonlesional atopic dermatitis skin is characterized by broad terminal differentiation defects and variable immune abnormalities.	J Allergy Clin Immunol.
atopic dermatitis	NA	Expression profiling by array, count, 3 development stage, 2 disease state, 11 individual sets	GPL570	GSE32924 Samples	Nonlesional atopic dermatitis skin is characterized by broad terminal differentiation defects and variable immune abnormalities.	J Allergy Clin Immunol.
atopic dermatitis	YWHAE	Expression profiling by array, count, 3 development stage, 2 disease state, 11 individual sets	GPL570	GSE32924 Samples	Nonlesional atopic dermatitis skin is characterized by broad terminal differentiation defects and variable immune abnormalities.	J Allergy Clin Immunol.
atopic dermatitis	LAMP1	Expression profiling by array, count, 3 development stage, 2 disease state, 11 individual sets	GPL570	GSE32924 Samples	Nonlesional atopic dermatitis skin is characterized by broad terminal differentiation defects and variable immune abnormalities.	J Allergy Clin Immunol.

atopic dermatitis	MS4A2	Expression profiling by array, count, 3 development stage, 2 disease state, 11 individual sets	GPL570	GSE32924 Samples	Nonlesional atopic dermatitis skin is characterized by broad terminal differentiation defects and variable immune abnormalities.	J Allergy Clin Immunol.
atopic dermatitis	RGS13	Expression profiling by array, count, 3 development stage, 2 disease state, 11 individual sets	GPL570	GSE32924 Samples	Nonlesional atopic dermatitis skin is characterized by broad terminal differentiation defects and variable immune abnormalities.	J Allergy Clin Immunol.
atopic dermatitis	MMRN1	Expression profiling by array, count, 3 development stage, 2 disease state, 11 individual sets	GPL570	GSE32924 Samples	Nonlesional atopic dermatitis skin is characterized by broad terminal differentiation defects and variable immune abnormalities.	J Allergy Clin Immunol.
atopic dermatitis	LYVE1	Expression profiling by array, count, 3 development stage, 2 disease state, 11 individual sets	GPL570	GSE32924 Samples	Nonlesional atopic dermatitis skin is characterized by broad terminal differentiation defects and variable immune abnormalities.	J Allergy Clin Immunol.
atopic dermatitis	GPR34	Expression profiling by array, count, 3 development stage, 2 disease state, 11 individual sets	GPL570	GSE32924 Samples	Nonlesional atopic dermatitis skin is characterized by broad terminal differentiation defects and variable immune abnormalities.	J Allergy Clin Immunol.
atopic dermatitis	LYVE1	Expression profiling by array, count, 3 development stage, 2 disease state, 11 individual sets	GPL570	GSE32924 Samples	Nonlesional atopic dermatitis skin is characterized by broad terminal differentiation defects and variable immune abnormalities.	J Allergy Clin Immunol.
atopic dermatitis	CP	Expression profiling by array, count, 3 development stage, 2 disease state, 11 individual sets	GPL570	GSE32924 Samples	Nonlesional atopic dermatitis skin is characterized by broad terminal differentiation defects and variable immune abnormalities.	J Allergy Clin Immunol.
atopic dermatitis	GREM1	Expression profiling by array, count, 3 development stage, 2 disease state, 11 individual sets	GPL570	GSE32924 Samples	Nonlesional atopic dermatitis skin is characterized by broad terminal differentiation defects and variable immune abnormalities.	J Allergy Clin Immunol.
atopic dermatitis	IGJ	Expression profiling by array, count, 3 development stage, 2 disease state, 11 individual sets	GPL570	GSE32924 Samples	Nonlesional atopic dermatitis skin is characterized by broad terminal differentiation defects and variable immune abnormalities.	J Allergy Clin Immunol.
atopic dermatitis	LIMA1	Expression profiling by array, count, 3 development stage, 2 disease state, 11 individual sets	GPL570	GSE32924 Samples	Nonlesional atopic dermatitis skin is characterized by broad terminal differentiation defects and variable immune abnormalities.	J Allergy Clin Immunol.
atopic dermatitis	POMGNT1	Expression profiling by array, count, 3 development stage, 2 disease state, 11 individual sets	GPL570	GSE32924 Samples	Nonlesional atopic dermatitis skin is characterized by broad terminal differentiation defects and variable immune abnormalities.	J Allergy Clin Immunol.
atopic dermatitis	SYNE2	Expression profiling by array, count, 3 development stage, 2 disease state, 11 individual sets	GPL570	GSE32924 Samples	Nonlesional atopic dermatitis skin is characterized by broad terminal differentiation defects and variable immune abnormalities.	J Allergy Clin Immunol.
atopic dermatitis	NA	Expression profiling by array, count, 3 development stage, 2 disease state, 11 individual sets	GPL570	GSE32924 Samples	Nonlesional atopic dermatitis skin is characterized by broad terminal differentiation defects and variable immune abnormalities.	J Allergy Clin Immunol.
atopic dermatitis	FBXW11	Expression profiling by array, count, 3 development stage, 2 disease state, 11 individual sets	GPL570	GSE32924 Samples	Nonlesional atopic dermatitis skin is characterized by broad terminal differentiation defects and variable immune abnormalities.	J Allergy Clin Immunol.
atopic dermatitis	OGDH	Expression profiling by array, count, 3 development stage, 2 disease state, 11 individual sets	GPL570	GSE32924 Samples	Nonlesional atopic dermatitis skin is characterized by broad terminal differentiation defects and variable immune abnormalities.	J Allergy Clin Immunol.
atopic dermatitis	PSD4	Expression profiling by array, count, 3 development stage, 2 disease state, 11 individual sets	GPL570	GSE32924 Samples	Nonlesional atopic dermatitis skin is characterized by broad terminal differentiation defects and variable immune abnormalities.	J Allergy Clin Immunol.
atopic dermatitis	KBTBD4	Expression profiling by array, count, 3 development stage, 2 disease state, 11 individual sets	GPL570	GSE32924 Samples	Nonlesional atopic dermatitis skin is characterized by broad terminal differentiation defects and variable immune abnormalities.	J Allergy Clin Immunol.
atopic dermatitis	SRPR	Expression profiling by array, count, 3 development stage, 2 disease state, 11 individual sets	GPL570	GSE32924 Samples	Nonlesional atopic dermatitis skin is characterized by broad terminal differentiation defects and variable immune abnormalities.	J Allergy Clin Immunol.
atopic dermatitis	VARS	Expression profiling by array, count, 3 development stage, 2 disease state, 11 individual sets	GPL570	GSE32924 Samples	Nonlesional atopic dermatitis skin is characterized by broad terminal differentiation defects and variable immune abnormalities.	J Allergy Clin Immunol.
atopic dermatitis	ZEB1	Expression profiling by array, count, 3 development stage, 2 disease state, 11 individual sets	GPL570	GSE32924 Samples	Nonlesional atopic dermatitis skin is characterized by broad terminal differentiation defects and variable immune abnormalities.	J Allergy Clin Immunol.
atopic dermatitis	DSTYK	Expression profiling by array, count, 3 development stage, 2 disease state, 11 individual sets	GPL570	GSE32924 Samples	Nonlesional atopic dermatitis skin is characterized by broad terminal differentiation defects and variable immune abnormalities.	J Allergy Clin Immunol.
atopic dermatitis	ANKRD11	Expression profiling by array, count, 3 development stage, 2 disease state, 11 individual sets	GPL570	GSE32924 Samples	Nonlesional atopic dermatitis skin is characterized by broad terminal differentiation defects and variable immune abnormalities.	J Allergy Clin Immunol.
atopic dermatitis	IKBKB	Expression profiling by array, count, 3 development stage, 2 disease state, 11 individual sets	GPL570	GSE32924 Samples	Nonlesional atopic dermatitis skin is characterized by broad terminal differentiation defects and variable immune abnormalities.	J Allergy Clin Immunol.
atopic dermatitis	MYH11	Expression profiling by array, count, 3 development stage, 2 disease state, 11 individual sets	GPL570	GSE32924 Samples	Nonlesional atopic dermatitis skin is characterized by broad terminal differentiation defects and variable immune abnormalities.	J Allergy Clin Immunol.
atopic dermatitis	PPIA	Expression profiling by array, count, 3 development stage, 2 disease state, 11 individual sets	GPL570	GSE32924 Samples	Nonlesional atopic dermatitis skin is characterized by broad terminal differentiation defects and variable immune abnormalities.	J Allergy Clin Immunol.
atopic dermatitis	HEATR2	Expression profiling by array, count, 3 development stage, 2 disease state, 11 individual sets	GPL570	GSE32924 Samples	Nonlesional atopic dermatitis skin is characterized by broad terminal differentiation defects and variable immune abnormalities.	J Allergy Clin Immunol.
atopic dermatitis	ERLIN1	Expression profiling by array, count, 3 development stage, 2 disease state, 11 individual sets	GPL570	GSE32924 Samples	Nonlesional atopic dermatitis skin is characterized by broad terminal differentiation defects and variable immune abnormalities.	J Allergy Clin Immunol.
atopic dermatitis	IFT57	Expression profiling by array, count, 3 development stage, 2 disease state, 11 individual sets	GPL570	GSE32924 Samples	Nonlesional atopic dermatitis skin is characterized by broad terminal differentiation defects and variable immune abnormalities.	J Allergy Clin Immunol.
atopic dermatitis	PRUNE2	Expression profiling by array, count, 3 development stage, 2 disease state, 11 individual sets	GPL570	GSE32924 Samples	Nonlesional atopic dermatitis skin is characterized by broad terminal differentiation defects and variable immune abnormalities.	J Allergy Clin Immunol.
atopic dermatitis	UBE2W	Expression profiling by array, count, 3 development stage, 2 disease state, 11 individual sets	GPL570	GSE32924 Samples	Nonlesional atopic dermatitis skin is characterized by broad terminal differentiation defects and variable immune abnormalities.	J Allergy Clin Immunol.
atopic dermatitis	ZNF567	Expression profiling by array, count, 3 development stage, 2 disease state, 11 individual sets	GPL570	GSE32924 Samples	Nonlesional atopic dermatitis skin is characterized by broad terminal differentiation defects and variable immune abnormalities.	J Allergy Clin Immunol.
atopic dermatitis	TRIM4	Expression profiling by array, count, 3 development stage, 2 disease state, 11 individual sets	GPL570	GSE32924 Samples	Nonlesional atopic dermatitis skin is characterized by broad terminal differentiation defects and variable immune abnormalities.	J Allergy Clin Immunol.
atopic dermatitis	NA	Expression profiling by array, count, 3 development stage, 2 disease state, 11 individual sets	GPL570	GSE32924 Samples	Nonlesional atopic dermatitis skin is characterized by broad terminal differentiation defects and variable immune abnormalities.	J Allergy Clin Immunol.
atopic dermatitis	ZNF567	Expression profiling by array, count, 3 development stage, 2 disease state, 11 individual sets	GPL570	GSE32924 Samples	Nonlesional atopic dermatitis skin is characterized by broad terminal differentiation defects and variable immune abnormalities.	J Allergy Clin Immunol.
atopic dermatitis	LRRC28	Expression profiling by array, count, 3 development stage, 2 disease state, 11 individual sets	GPL570	GSE32924 Samples	Nonlesional atopic dermatitis skin is characterized by broad terminal differentiation defects and variable immune abnormalities.	J Allergy Clin Immunol.
atopic dermatitis	FIP1L1	Expression profiling by array, count, 3 development stage, 2 disease state, 11 individual sets	GPL570	GSE32924 Samples	Nonlesional atopic dermatitis skin is characterized by broad terminal differentiation defects and variable immune abnormalities.	J Allergy Clin Immunol.
atopic dermatitis	PCDHB6	Expression profiling by array, count, 3 development stage, 2 disease state, 11 individual sets	GPL570	GSE32924 Samples	Nonlesional atopic dermatitis skin is characterized by broad terminal differentiation defects and variable immune abnormalities.	J Allergy Clin Immunol.
atopic dermatitis	XPNPEP2	Expression profiling by array, count, 3 development stage, 2 disease state, 11 individual sets	GPL570	GSE32924 Samples	Nonlesional atopic dermatitis skin is characterized by broad terminal differentiation defects and variable immune abnormalities.	J Allergy Clin Immunol.
atopic dermatitis	JAK1	Expression profiling by array, count, 3 development stage, 2 disease state, 11 individual sets	GPL570	GSE32924 Samples	Nonlesional atopic dermatitis skin is characterized by broad terminal differentiation defects and variable immune abnormalities.	J Allergy Clin Immunol.
atopic dermatitis	TBL1XR1	Expression profiling by array, count, 3 development stage, 2 disease state, 11 individual sets	GPL570	GSE32924 Samples	Nonlesional atopic dermatitis skin is characterized by broad terminal differentiation defects and variable immune abnormalities.	J Allergy Clin Immunol.
atopic dermatitis	NA	Expression profiling by array, count, 3 development stage, 2 disease state, 11 individual sets	GPL570	GSE32924 Samples	Nonlesional atopic dermatitis skin is characterized by broad terminal differentiation defects and variable immune abnormalities.	J Allergy Clin Immunol.

atopic dermatitis	STK4	Expression profiling by array, count, 3 development stage, 2 disease state, 11 individual sets	GPL570	GSE32924 Samples	Nonlesional atopic dermatitis skin is characterized by broad terminal differentiation defects and variable immune abnormalities.	J Allergy Clin Immunol.
atopic dermatitis	NHSL1	Expression profiling by array, count, 3 development stage, 2 disease state, 11 individual sets	GPL570	GSE32924 Samples	Nonlesional atopic dermatitis skin is characterized by broad terminal differentiation defects and variable immune abnormalities.	J Allergy Clin Immunol.
atopic dermatitis	RASSF5	Expression profiling by array, count, 3 development stage, 2 disease state, 11 individual sets	GPL570	GSE32924 Samples	Nonlesional atopic dermatitis skin is characterized by broad terminal differentiation defects and variable immune abnormalities.	J Allergy Clin Immunol.
atopic dermatitis	GAS7	Expression profiling by array, count, 3 development stage, 2 disease state, 11 individual sets	GPL570	GSE32924 Samples	Nonlesional atopic dermatitis skin is characterized by broad terminal differentiation defects and variable immune abnormalities.	J Allergy Clin Immunol.
atopic dermatitis	NA	Expression profiling by array, count, 3 development stage, 2 disease state, 11 individual sets	GPL570	GSE32924 Samples	Nonlesional atopic dermatitis skin is characterized by broad terminal differentiation defects and variable immune abnormalities.	J Allergy Clin Immunol.
atopic dermatitis	HPGD	Expression profiling by array, count, 3 development stage, 2 disease state, 11 individual sets	GPL570	GSE32924 Samples	Nonlesional atopic dermatitis skin is characterized by broad terminal differentiation defects and variable immune abnormalities.	J Allergy Clin Immunol.
atopic dermatitis	ARHGAP18	Expression profiling by array, count, 3 development stage, 2 disease state, 11 individual sets	GPL570	GSE32924 Samples	Nonlesional atopic dermatitis skin is characterized by broad terminal differentiation defects and variable immune abnormalities.	J Allergy Clin Immunol.
atopic dermatitis	SGEF	Expression profiling by array, count, 3 development stage, 2 disease state, 11 individual sets	GPL570	GSE32924 Samples	Nonlesional atopic dermatitis skin is characterized by broad terminal differentiation defects and variable immune abnormalities.	J Allergy Clin Immunol.
atopic dermatitis	NA	Expression profiling by array, count, 3 development stage, 2 disease state, 11 individual sets	GPL570	GSE32924 Samples	Nonlesional atopic dermatitis skin is characterized by broad terminal differentiation defects and variable immune abnormalities.	J Allergy Clin Immunol.
atopic dermatitis	KIAA2026	Expression profiling by array, count, 3 development stage, 2 disease state, 11 individual sets	GPL570	GSE32924 Samples	Nonlesional atopic dermatitis skin is characterized by broad terminal differentiation defects and variable immune abnormalities.	J Allergy Clin Immunol.
atopic dermatitis	PSEN1	Expression profiling by array, count, 3 development stage, 2 disease state, 11 individual sets	GPL570	GSE32924 Samples	Nonlesional atopic dermatitis skin is characterized by broad terminal differentiation defects and variable immune abnormalities.	J Allergy Clin Immunol.
atopic dermatitis	MUTED	Expression profiling by array, count, 3 development stage, 2 disease state, 11 individual sets	GPL570	GSE32924 Samples	Nonlesional atopic dermatitis skin is characterized by broad terminal differentiation defects and variable immune abnormalities.	J Allergy Clin Immunol.
atopic dermatitis	ZCCHC7	Expression profiling by array, count, 3 development stage, 2 disease state, 11 individual sets	GPL570	GSE32924 Samples	Nonlesional atopic dermatitis skin is characterized by broad terminal differentiation defects and variable immune abnormalities.	J Allergy Clin Immunol.
atopic dermatitis	KIAA1324L	Expression profiling by array, count, 3 development stage, 2 disease state, 11 individual sets	GPL570	GSE32924 Samples	Nonlesional atopic dermatitis skin is characterized by broad terminal differentiation defects and variable immune abnormalities.	J Allergy Clin Immunol.
atopic dermatitis	BMPR2	Expression profiling by array, count, 3 development stage, 2 disease state, 11 individual sets	GPL570	GSE32924 Samples	Nonlesional atopic dermatitis skin is characterized by broad terminal differentiation defects and variable immune abnormalities.	J Allergy Clin Immunol.
atopic dermatitis	SVEP1	Expression profiling by array, count, 3 development stage, 2 disease state, 11 individual sets	GPL570	GSE32924 Samples	Nonlesional atopic dermatitis skin is characterized by broad terminal differentiation defects and variable immune abnormalities.	J Allergy Clin Immunol.
atopic dermatitis	N4BP2L1	Expression profiling by array, count, 3 development stage, 2 disease state, 11 individual sets	GPL570	GSE32924 Samples	Nonlesional atopic dermatitis skin is characterized by broad terminal differentiation defects and variable immune abnormalities.	J Allergy Clin Immunol.
atopic dermatitis	CEP120	Expression profiling by array, count, 3 development stage, 2 disease state, 11 individual sets	GPL570	GSE32924 Samples	Nonlesional atopic dermatitis skin is characterized by broad terminal differentiation defects and variable immune abnormalities.	J Allergy Clin Immunol.
atopic dermatitis	LRFN5	Expression profiling by array, count, 3 development stage, 2 disease state, 11 individual sets	GPL570	GSE32924 Samples	Nonlesional atopic dermatitis skin is characterized by broad terminal differentiation defects and variable immune abnormalities.	J Allergy Clin Immunol.
atopic dermatitis	C4ORF12	Expression profiling by array, count, 3 development stage, 2 disease state, 11 individual sets	GPL570	GSE32924 Samples	Nonlesional atopic dermatitis skin is characterized by broad terminal differentiation defects and variable immune abnormalities.	J Allergy Clin Immunol.
atopic dermatitis	RHOJ	Expression profiling by array, count, 3 development stage, 2 disease state, 11 individual sets	GPL570	GSE32924 Samples	Nonlesional atopic dermatitis skin is characterized by broad terminal differentiation defects and variable immune abnormalities.	J Allergy Clin Immunol.
atopic dermatitis	FIBIN	Expression profiling by array, count, 3 development stage, 2 disease state, 11 individual sets	GPL570	GSE32924 Samples	Nonlesional atopic dermatitis skin is characterized by broad terminal differentiation defects and variable immune abnormalities.	J Allergy Clin Immunol.
atopic dermatitis	SPINK1	Expression profiling by array, count, 3 development stage, 2 disease state, 11 individual sets	GPL570	GSE32924 Samples	Nonlesional atopic dermatitis skin is characterized by broad terminal differentiation defects and variable immune abnormalities.	J Allergy Clin Immunol.
atopic dermatitis	ZNF24	Expression profiling by array, count, 3 development stage, 2 disease state, 11 individual sets	GPL570	GSE32924 Samples	Nonlesional atopic dermatitis skin is characterized by broad terminal differentiation defects and variable immune abnormalities.	J Allergy Clin Immunol.
atopic dermatitis	SNAP23	Expression profiling by array, count, 3 development stage, 2 disease state, 11 individual sets	GPL570	GSE32924 Samples	Nonlesional atopic dermatitis skin is characterized by broad terminal differentiation defects and variable immune abnormalities.	J Allergy Clin Immunol.
atopic dermatitis	MTMR2	Expression profiling by array, count, 3 development stage, 2 disease state, 11 individual sets	GPL570	GSE32924 Samples	Nonlesional atopic dermatitis skin is characterized by broad terminal differentiation defects and variable immune abnormalities.	J Allergy Clin Immunol.
atopic dermatitis	PDGFC	Expression profiling by array, count, 3 development stage, 2 disease state, 11 individual sets	GPL570	GSE32924 Samples	Nonlesional atopic dermatitis skin is characterized by broad terminal differentiation defects and variable immune abnormalities.	J Allergy Clin Immunol.
atopic dermatitis	CASK	Expression profiling by array, count, 3 development stage, 2 disease state, 11 individual sets	GPL570	GSE32924 Samples	Nonlesional atopic dermatitis skin is characterized by broad terminal differentiation defects and variable immune abnormalities.	J Allergy Clin Immunol.
atopic dermatitis	RAB6A	Expression profiling by array, count, 3 development stage, 2 disease state, 11 individual sets	GPL570	GSE32924 Samples	Nonlesional atopic dermatitis skin is characterized by broad terminal differentiation defects and variable immune abnormalities.	J Allergy Clin Immunol.
atopic dermatitis	SLAIN2	Expression profiling by array, count, 3 development stage, 2 disease state, 11 individual sets	GPL570	GSE32924 Samples	Nonlesional atopic dermatitis skin is characterized by broad terminal differentiation defects and variable immune abnormalities.	J Allergy Clin Immunol.
atopic dermatitis	SPON1	Expression profiling by array, count, 3 development stage, 2 disease state, 11 individual sets	GPL570	GSE32924 Samples	Nonlesional atopic dermatitis skin is characterized by broad terminal differentiation defects and variable immune abnormalities.	J Allergy Clin Immunol.
atopic dermatitis	PPP1R3B	Expression profiling by array, count, 3 development stage, 2 disease state, 11 individual sets	GPL570	GSE32924 Samples	Nonlesional atopic dermatitis skin is characterized by broad terminal differentiation defects and variable immune abnormalities.	J Allergy Clin Immunol.
atopic dermatitis	STX16	Expression profiling by array, count, 3 development stage, 2 disease state, 11 individual sets	GPL570	GSE32924 Samples	Nonlesional atopic dermatitis skin is characterized by broad terminal differentiation defects and variable immune abnormalities.	J Allergy Clin Immunol.
atopic dermatitis	IRF6	Expression profiling by array, count, 3 development stage, 2 disease state, 11 individual sets	GPL570	GSE32924 Samples	Nonlesional atopic dermatitis skin is characterized by broad terminal differentiation defects and variable immune abnormalities.	J Allergy Clin Immunol.
atopic dermatitis	INPP5A	Expression profiling by array, count, 3 development stage, 2 disease state, 11 individual sets	GPL570	GSE32924 Samples	Nonlesional atopic dermatitis skin is characterized by broad terminal differentiation defects and variable immune abnormalities.	J Allergy Clin Immunol.
atopic dermatitis	HSP90B1	Expression profiling by array, count, 3 development stage, 2 disease state, 11 individual sets	GPL570	GSE32924 Samples	Nonlesional atopic dermatitis skin is characterized by broad terminal differentiation defects and variable immune abnormalities.	J Allergy Clin Immunol.
atopic dermatitis	C15ORF24	Expression profiling by array, count, 3 development stage, 2 disease state, 11 individual sets	GPL570	GSE32924 Samples	Nonlesional atopic dermatitis skin is characterized by broad terminal differentiation defects and variable immune abnormalities.	J Allergy Clin Immunol.
atopic dermatitis	UBASH3B	Expression profiling by array, count, 3 development stage, 2 disease state, 11 individual sets	GPL570	GSE32924 Samples	Nonlesional atopic dermatitis skin is characterized by broad terminal differentiation defects and variable immune abnormalities.	J Allergy Clin Immunol.
atopic dermatitis	VPS53	Expression profiling by array, count, 3 development stage, 2 disease state, 11 individual sets	GPL570	GSE32924 Samples	Nonlesional atopic dermatitis skin is characterized by broad terminal differentiation defects and variable immune abnormalities.	J Allergy Clin Immunol.
atopic dermatitis	PCIF1	Expression profiling by array, count, 3 development stage, 2 disease state, 11 individual sets	GPL570	GSE32924 Samples	Nonlesional atopic dermatitis skin is characterized by broad terminal differentiation defects and variable immune abnormalities.	J Allergy Clin Immunol.
atopic dermatitis	ZNF638	Expression profiling by array, count, 3 development stage, 2 disease state, 11 individual sets	GPL570	GSE32924 Samples	Nonlesional atopic dermatitis skin is characterized by broad terminal differentiation defects and variable immune abnormalities.	J Allergy Clin Immunol.

atopic dermatitis	CARS	Expression profiling by array, count, 3 development stage, 2 disease state, 11 individual sets	GPL570	GSE32924 Samples	Nonlesional atopic dermatitis skin is characterized by broad terminal differentiation defects and variable immune abnormalities.	J Allergy Clin Immunol.
atopic dermatitis	GAS7	Expression profiling by array, count, 3 development stage, 2 disease state, 11 individual sets	GPL570	GSE32924 Samples	Nonlesional atopic dermatitis skin is characterized by broad terminal differentiation defects and variable immune abnormalities.	J Allergy Clin Immunol.
atopic dermatitis	FCGR2C	Expression profiling by array, count, 3 development stage, 2 disease state, 11 individual sets	GPL570	GSE32924 Samples	Nonlesional atopic dermatitis skin is characterized by broad terminal differentiation defects and variable immune abnormalities.	J Allergy Clin Immunol.
atopic dermatitis	CLN8	Expression profiling by array, count, 3 development stage, 2 disease state, 11 individual sets	GPL570	GSE32924 Samples	Nonlesional atopic dermatitis skin is characterized by broad terminal differentiation defects and variable immune abnormalities.	J Allergy Clin Immunol.
atopic dermatitis	CLIP1	Expression profiling by array, count, 3 development stage, 2 disease state, 11 individual sets	GPL570	GSE32924 Samples	Nonlesional atopic dermatitis skin is characterized by broad terminal differentiation defects and variable immune abnormalities.	J Allergy Clin Immunol.
atopic dermatitis	KBTBD2	Expression profiling by array, count, 3 development stage, 2 disease state, 11 individual sets	GPL570	GSE32924 Samples	Nonlesional atopic dermatitis skin is characterized by broad terminal differentiation defects and variable immune abnormalities.	J Allergy Clin Immunol.
atopic dermatitis	GNG12	Expression profiling by array, count, 3 development stage, 2 disease state, 11 individual sets	GPL570	GSE32924 Samples	Nonlesional atopic dermatitis skin is characterized by broad terminal differentiation defects and variable immune abnormalities.	J Allergy Clin Immunol.
atopic dermatitis	NUSAP1	Expression profiling by array, count, 3 development stage, 2 disease state, 11 individual sets	GPL570	GSE32924 Samples	Nonlesional atopic dermatitis skin is characterized by broad terminal differentiation defects and variable immune abnormalities.	J Allergy Clin Immunol.
atopic dermatitis	UBASH3B	Expression profiling by array, count, 3 development stage, 2 disease state, 11 individual sets	GPL570	GSE32924 Samples	Nonlesional atopic dermatitis skin is characterized by broad terminal differentiation defects and variable immune abnormalities.	J Allergy Clin Immunol.
atopic dermatitis	ZNF655	Expression profiling by array, count, 3 development stage, 2 disease state, 11 individual sets	GPL570	GSE32924 Samples	Nonlesional atopic dermatitis skin is characterized by broad terminal differentiation defects and variable immune abnormalities.	J Allergy Clin Immunol.
atopic dermatitis	C13ORF31	Expression profiling by array, count, 3 development stage, 2 disease state, 11 individual sets	GPL570	GSE32924 Samples	Nonlesional atopic dermatitis skin is characterized by broad terminal differentiation defects and variable immune abnormalities.	J Allergy Clin Immunol.
atopic dermatitis	ETV7	Expression profiling by array, count, 3 development stage, 2 disease state, 11 individual sets	GPL570	GSE32924 Samples	Nonlesional atopic dermatitis skin is characterized by broad terminal differentiation defects and variable immune abnormalities.	J Allergy Clin Immunol.
atopic dermatitis	CYP39A1	Expression profiling by array, count, 3 development stage, 2 disease state, 11 individual sets	GPL570	GSE32924 Samples	Nonlesional atopic dermatitis skin is characterized by broad terminal differentiation defects and variable immune abnormalities.	J Allergy Clin Immunol.
atopic dermatitis	SLAIN2	Expression profiling by array, count, 3 development stage, 2 disease state, 11 individual sets	GPL570	GSE32924 Samples	Nonlesional atopic dermatitis skin is characterized by broad terminal differentiation defects and variable immune abnormalities.	J Allergy Clin Immunol.
atopic dermatitis	BCLAF1	Expression profiling by array, count, 3 development stage, 2 disease state, 11 individual sets	GPL570	GSE32924 Samples	Nonlesional atopic dermatitis skin is characterized by broad terminal differentiation defects and variable immune abnormalities.	J Allergy Clin Immunol.
atopic dermatitis	SLAIN2	Expression profiling by array, count, 3 development stage, 2 disease state, 11 individual sets	GPL570	GSE32924 Samples	Nonlesional atopic dermatitis skin is characterized by broad terminal differentiation defects and variable immune abnormalities.	J Allergy Clin Immunol.
atopic dermatitis	UBE2H	Expression profiling by array, count, 3 development stage, 2 disease state, 11 individual sets	GPL570	GSE32924 Samples	Nonlesional atopic dermatitis skin is characterized by broad terminal differentiation defects and variable immune abnormalities.	J Allergy Clin Immunol.
atopic dermatitis	CTTN	Expression profiling by array, count, 3 development stage, 2 disease state, 11 individual sets	GPL570	GSE32924 Samples	Nonlesional atopic dermatitis skin is characterized by broad terminal differentiation defects and variable immune abnormalities.	J Allergy Clin Immunol.
atopic dermatitis	CUGBP1	Expression profiling by array, count, 3 development stage, 2 disease state, 11 individual sets	GPL570	GSE32924 Samples	Nonlesional atopic dermatitis skin is characterized by broad terminal differentiation defects and variable immune abnormalities.	J Allergy Clin Immunol.
atopic dermatitis	PTPRA	Expression profiling by array, count, 3 development stage, 2 disease state, 11 individual sets	GPL570	GSE32924 Samples	Nonlesional atopic dermatitis skin is characterized by broad terminal differentiation defects and variable immune abnormalities.	J Allergy Clin Immunol.
atopic dermatitis	XGPY2	Expression profiling by array, count, 3 development stage, 2 disease state, 11 individual sets	GPL570	GSE32924 Samples	Nonlesional atopic dermatitis skin is characterized by broad terminal differentiation defects and variable immune abnormalities.	J Allergy Clin Immunol.
atopic dermatitis	ORMDL1	Expression profiling by array, count, 3 development stage, 2 disease state, 11 individual sets	GPL570	GSE32924 Samples	Nonlesional atopic dermatitis skin is characterized by broad terminal differentiation defects and variable immune abnormalities.	J Allergy Clin Immunol.
atopic dermatitis	NA	Expression profiling by array, count, 3 development stage, 2 disease state, 11 individual sets	GPL570	GSE32924 Samples	Nonlesional atopic dermatitis skin is characterized by broad terminal differentiation defects and variable immune abnormalities.	J Allergy Clin Immunol.
atopic dermatitis	SFRS5	Expression profiling by array, count, 3 development stage, 2 disease state, 11 individual sets	GPL570	GSE32924 Samples	Nonlesional atopic dermatitis skin is characterized by broad terminal differentiation defects and variable immune abnormalities.	J Allergy Clin Immunol.
atopic dermatitis	HDLBP	Expression profiling by array, count, 3 development stage, 2 disease state, 11 individual sets	GPL570	GSE32924 Samples	Nonlesional atopic dermatitis skin is characterized by broad terminal differentiation defects and variable immune abnormalities.	J Allergy Clin Immunol.
atopic dermatitis	FAM190B	Expression profiling by array, count, 3 development stage, 2 disease state, 11 individual sets	GPL570	GSE32924 Samples	Nonlesional atopic dermatitis skin is characterized by broad terminal differentiation defects and variable immune abnormalities.	J Allergy Clin Immunol.
atopic dermatitis	GALNT12	Expression profiling by array, count, 3 development stage, 2 disease state, 11 individual sets	GPL570	GSE32924 Samples	Nonlesional atopic dermatitis skin is characterized by broad terminal differentiation defects and variable immune abnormalities.	J Allergy Clin Immunol.
atopic dermatitis	NAV3	Expression profiling by array, count, 3 development stage, 2 disease state, 11 individual sets	GPL570	GSE32924 Samples	Nonlesional atopic dermatitis skin is characterized by broad terminal differentiation defects and variable immune abnormalities.	J Allergy Clin Immunol.
atopic dermatitis	MACC1	Expression profiling by array, count, 3 development stage, 2 disease state, 11 individual sets	GPL570	GSE32924 Samples	Nonlesional atopic dermatitis skin is characterized by broad terminal differentiation defects and variable immune abnormalities.	J Allergy Clin Immunol.
atopic dermatitis	MAP7	Expression profiling by array, count, 3 development stage, 2 disease state, 11 individual sets	GPL570	GSE32924 Samples	Nonlesional atopic dermatitis skin is characterized by broad terminal differentiation defects and variable immune abnormalities.	J Allergy Clin Immunol.
atopic dermatitis	RHEB	Expression profiling by array, count, 3 development stage, 2 disease state, 11 individual sets	GPL570	GSE32924 Samples	Nonlesional atopic dermatitis skin is characterized by broad terminal differentiation defects and variable immune abnormalities.	J Allergy Clin Immunol.
atopic dermatitis	RAB2A	Expression profiling by array, count, 3 development stage, 2 disease state, 11 individual sets	GPL570	GSE32924 Samples	Nonlesional atopic dermatitis skin is characterized by broad terminal differentiation defects and variable immune abnormalities.	J Allergy Clin Immunol.
atopic dermatitis	C16ORF63	Expression profiling by array, count, 3 development stage, 2 disease state, 11 individual sets	GPL570	GSE32924 Samples	Nonlesional atopic dermatitis skin is characterized by broad terminal differentiation defects and variable immune abnormalities.	J Allergy Clin Immunol.
atopic dermatitis	METTL9	Expression profiling by array, count, 3 development stage, 2 disease state, 11 individual sets	GPL570	GSE32924 Samples	Nonlesional atopic dermatitis skin is characterized by broad terminal differentiation defects and variable immune abnormalities.	J Allergy Clin Immunol.
atopic dermatitis	SPIRE1	Expression profiling by array, count, 3 development stage, 2 disease state, 11 individual sets	GPL570	GSE32924 Samples	Nonlesional atopic dermatitis skin is characterized by broad terminal differentiation defects and variable immune abnormalities.	J Allergy Clin Immunol.
atopic dermatitis	RPAP3	Expression profiling by array, count, 3 development stage, 2 disease state, 11 individual sets	GPL570	GSE32924 Samples	Nonlesional atopic dermatitis skin is characterized by broad terminal differentiation defects and variable immune abnormalities.	J Allergy Clin Immunol.
atopic dermatitis	MTPAP	Expression profiling by array, count, 3 development stage, 2 disease state, 11 individual sets	GPL570	GSE32924 Samples	Nonlesional atopic dermatitis skin is characterized by broad terminal differentiation defects and variable immune abnormalities.	J Allergy Clin Immunol.
atopic dermatitis	BRCC3	Expression profiling by array, count, 3 development stage, 2 disease state, 11 individual sets	GPL570	GSE32924 Samples	Nonlesional atopic dermatitis skin is characterized by broad terminal differentiation defects and variable immune abnormalities.	J Allergy Clin Immunol.
atopic dermatitis	RWDD2B	Expression profiling by array, count, 3 development stage, 2 disease state, 11 individual sets	GPL570	GSE32924 Samples	Nonlesional atopic dermatitis skin is characterized by broad terminal differentiation defects and variable immune abnormalities.	J Allergy Clin Immunol.
atopic dermatitis	ARNTL	Expression profiling by array, count, 3 development stage, 2 disease state, 11 individual sets	GPL570	GSE32924 Samples	Nonlesional atopic dermatitis skin is characterized by broad terminal differentiation defects and variable immune abnormalities.	J Allergy Clin Immunol.
atopic dermatitis	XKRX	Expression profiling by array, count, 3 development stage, 2 disease state, 11 individual sets	GPL570	GSE32924 Samples	Nonlesional atopic dermatitis skin is characterized by broad terminal differentiation defects and variable immune abnormalities.	J Allergy Clin Immunol.

atopic dermatitis	PTPN21	Expression profiling by array, count, 3 development stage, 2 disease state, 11 individual sets	GPL570	GSE32924 Samples	Nonlesional atopic dermatitis skin is characterized by broad terminal differentiation defects and variable immune abnormalities.	J Allergy Clin Immunol.
atopic dermatitis	ACOT8	Expression profiling by array, count, 3 development stage, 2 disease state, 11 individual sets	GPL570	GSE32924 Samples	Nonlesional atopic dermatitis skin is characterized by broad terminal differentiation defects and variable immune abnormalities.	J Allergy Clin Immunol.
atopic dermatitis	OCLN	Expression profiling by array, count, 3 development stage, 2 disease state, 11 individual sets	GPL570	GSE32924 Samples	Nonlesional atopic dermatitis skin is characterized by broad terminal differentiation defects and variable immune abnormalities.	J Allergy Clin Immunol.
atopic dermatitis	NA	Expression profiling by array, count, 3 development stage, 2 disease state, 11 individual sets	GPL570	GSE32924 Samples	Nonlesional atopic dermatitis skin is characterized by broad terminal differentiation defects and variable immune abnormalities.	J Allergy Clin Immunol.
atopic dermatitis	CYP2C18	Expression profiling by array, count, 3 development stage, 2 disease state, 11 individual sets	GPL570	GSE32924 Samples	Nonlesional atopic dermatitis skin is characterized by broad terminal differentiation defects and variable immune abnormalities.	J Allergy Clin Immunol.
atopic dermatitis	RNASE7	Expression profiling by array, count, 3 development stage, 2 disease state, 11 individual sets	GPL570	GSE32924 Samples	Nonlesional atopic dermatitis skin is characterized by broad terminal differentiation defects and variable immune abnormalities.	J Allergy Clin Immunol.
atopic dermatitis	ACER1	Expression profiling by array, count, 3 development stage, 2 disease state, 11 individual sets	GPL570	GSE32924 Samples	Nonlesional atopic dermatitis skin is characterized by broad terminal differentiation defects and variable immune abnormalities.	J Allergy Clin Immunol.
atopic dermatitis	F2RL1	Expression profiling by array, count, 3 development stage, 2 disease state, 11 individual sets	GPL570	GSE32924 Samples	Nonlesional atopic dermatitis skin is characterized by broad terminal differentiation defects and variable immune abnormalities.	J Allergy Clin Immunol.
atopic dermatitis	CDV3	Expression profiling by array, count, 3 development stage, 2 disease state, 11 individual sets	GPL570	GSE32924 Samples	Nonlesional atopic dermatitis skin is characterized by broad terminal differentiation defects and variable immune abnormalities.	J Allergy Clin Immunol.
atopic dermatitis	MTMR1	Expression profiling by array, count, 3 development stage, 2 disease state, 11 individual sets	GPL570	GSE32924 Samples	Nonlesional atopic dermatitis skin is characterized by broad terminal differentiation defects and variable immune abnormalities.	J Allergy Clin Immunol.
atopic dermatitis	EZR	Expression profiling by array, count, 3 development stage, 2 disease state, 11 individual sets	GPL570	GSE32924 Samples	Nonlesional atopic dermatitis skin is characterized by broad terminal differentiation defects and variable immune abnormalities.	J Allergy Clin Immunol.
atopic dermatitis	NA	Expression profiling by array, count, 3 development stage, 2 disease state, 11 individual sets	GPL570	GSE32924 Samples	Nonlesional atopic dermatitis skin is characterized by broad terminal differentiation defects and variable immune abnormalities.	J Allergy Clin Immunol.
atopic dermatitis	SQLE	Expression profiling by array, count, 3 development stage, 2 disease state, 11 individual sets	GPL570	GSE32924 Samples	Nonlesional atopic dermatitis skin is characterized by broad terminal differentiation defects and variable immune abnormalities.	J Allergy Clin Immunol.
atopic dermatitis	RNASE7	Expression profiling by array, count, 3 development stage, 2 disease state, 11 individual sets	GPL570	GSE32924 Samples	Nonlesional atopic dermatitis skin is characterized by broad terminal differentiation defects and variable immune abnormalities.	J Allergy Clin Immunol.
atopic dermatitis	ELMO2	Expression profiling by array, count, 3 development stage, 2 disease state, 11 individual sets	GPL570	GSE32924 Samples	Nonlesional atopic dermatitis skin is characterized by broad terminal differentiation defects and variable immune abnormalities.	J Allergy Clin Immunol.
atopic dermatitis	PDE8A	Expression profiling by array, count, 3 development stage, 2 disease state, 11 individual sets	GPL570	GSE32924 Samples	Nonlesional atopic dermatitis skin is characterized by broad terminal differentiation defects and variable immune abnormalities.	J Allergy Clin Immunol.
atopic dermatitis	API5	Expression profiling by array, count, 3 development stage, 2 disease state, 11 individual sets	GPL570	GSE32924 Samples	Nonlesional atopic dermatitis skin is characterized by broad terminal differentiation defects and variable immune abnormalities.	J Allergy Clin Immunol.
atopic dermatitis	FAM175B	Expression profiling by array, count, 3 development stage, 2 disease state, 11 individual sets	GPL570	GSE32924 Samples	Nonlesional atopic dermatitis skin is characterized by broad terminal differentiation defects and variable immune abnormalities.	J Allergy Clin Immunol.
atopic dermatitis	ARFGEF1	Expression profiling by array, count, 3 development stage, 2 disease state, 11 individual sets	GPL570	GSE32924 Samples	Nonlesional atopic dermatitis skin is characterized by broad terminal differentiation defects and variable immune abnormalities.	J Allergy Clin Immunol.
atopic dermatitis	ANKRD13C	Expression profiling by array, count, 3 development stage, 2 disease state, 11 individual sets	GPL570	GSE32924 Samples	Nonlesional atopic dermatitis skin is characterized by broad terminal differentiation defects and variable immune abnormalities.	J Allergy Clin Immunol.
atopic dermatitis	ARIH1	Expression profiling by array, count, 3 development stage, 2 disease state, 11 individual sets	GPL570	GSE32924 Samples	Nonlesional atopic dermatitis skin is characterized by broad terminal differentiation defects and variable immune abnormalities.	J Allergy Clin Immunol.
atopic dermatitis	OSBPL11	Expression profiling by array, count, 3 development stage, 2 disease state, 11 individual sets	GPL570	GSE32924 Samples	Nonlesional atopic dermatitis skin is characterized by broad terminal differentiation defects and variable immune abnormalities.	J Allergy Clin Immunol.
atopic dermatitis	RPRD1A	Expression profiling by array, count, 3 development stage, 2 disease state, 11 individual sets	GPL570	GSE32924 Samples	Nonlesional atopic dermatitis skin is characterized by broad terminal differentiation defects and variable immune abnormalities.	J Allergy Clin Immunol.
atopic dermatitis	MED28	Expression profiling by array, count, 3 development stage, 2 disease state, 11 individual sets	GPL570	GSE32924 Samples	Nonlesional atopic dermatitis skin is characterized by broad terminal differentiation defects and variable immune abnormalities.	J Allergy Clin Immunol.
atopic dermatitis	KLRAQ1	Expression profiling by array, count, 3 development stage, 2 disease state, 11 individual sets	GPL570	GSE32924 Samples	Nonlesional atopic dermatitis skin is characterized by broad terminal differentiation defects and variable immune abnormalities.	J Allergy Clin Immunol.
atopic dermatitis	TFG	Expression profiling by array, count, 3 development stage, 2 disease state, 11 individual sets	GPL570	GSE32924 Samples	Nonlesional atopic dermatitis skin is characterized by broad terminal differentiation defects and variable immune abnormalities.	J Allergy Clin Immunol.
atopic dermatitis	C9ORF125	Expression profiling by array, count, 3 development stage, 2 disease state, 11 individual sets	GPL570	GSE32924 Samples	Nonlesional atopic dermatitis skin is characterized by broad terminal differentiation defects and variable immune abnormalities.	J Allergy Clin Immunol.
atopic dermatitis	NA	Expression profiling by array, count, 3 development stage, 2 disease state, 11 individual sets	GPL570	GSE32924 Samples	Nonlesional atopic dermatitis skin is characterized by broad terminal differentiation defects and variable immune abnormalities.	J Allergy Clin Immunol.
atopic dermatitis	TMEM68	Expression profiling by array, count, 3 development stage, 2 disease state, 11 individual sets	GPL570	GSE32924 Samples	Nonlesional atopic dermatitis skin is characterized by broad terminal differentiation defects and variable immune abnormalities.	J Allergy Clin Immunol.
atopic dermatitis	USP2	Expression profiling by array, count, 3 development stage, 2 disease state, 11 individual sets	GPL570	GSE32924 Samples	Nonlesional atopic dermatitis skin is characterized by broad terminal differentiation defects and variable immune abnormalities.	J Allergy Clin Immunol.
atopic dermatitis	DNAJA4	Expression profiling by array, count, 3 development stage, 2 disease state, 11 individual sets	GPL570	GSE32924 Samples	Nonlesional atopic dermatitis skin is characterized by broad terminal differentiation defects and variable immune abnormalities.	J Allergy Clin Immunol.
atopic dermatitis	GFM2	Expression profiling by array, count, 3 development stage, 2 disease state, 11 individual sets	GPL570	GSE32924 Samples	Nonlesional atopic dermatitis skin is characterized by broad terminal differentiation defects and variable immune abnormalities.	J Allergy Clin Immunol.
atopic dermatitis	CALU	Expression profiling by array, count, 3 development stage, 2 disease state, 11 individual sets	GPL570	GSE32924 Samples	Nonlesional atopic dermatitis skin is characterized by broad terminal differentiation defects and variable immune abnormalities.	J Allergy Clin Immunol.
atopic dermatitis	NID1	Expression profiling by array, count, 3 development stage, 2 disease state, 11 individual sets	GPL570	GSE32924 Samples	Nonlesional atopic dermatitis skin is characterized by broad terminal differentiation defects and variable immune abnormalities.	J Allergy Clin Immunol.
atopic dermatitis	PTPN12	Expression profiling by array, count, 3 development stage, 2 disease state, 11 individual sets	GPL570	GSE32924 Samples	Nonlesional atopic dermatitis skin is characterized by broad terminal differentiation defects and variable immune abnormalities.	J Allergy Clin Immunol.
atopic dermatitis	ATRN	Expression profiling by array, count, 3 development stage, 2 disease state, 11 individual sets	GPL570	GSE32924 Samples	Nonlesional atopic dermatitis skin is characterized by broad terminal differentiation defects and variable immune abnormalities.	J Allergy Clin Immunol.
atopic dermatitis	GTF2I	Expression profiling by array, count, 3 development stage, 2 disease state, 11 individual sets	GPL570	GSE32924 Samples	Nonlesional atopic dermatitis skin is characterized by broad terminal differentiation defects and variable immune abnormalities.	J Allergy Clin Immunol.
atopic dermatitis	SMARCC1	Expression profiling by array, count, 3 development stage, 2 disease state, 11 individual sets	GPL570	GSE32924 Samples	Nonlesional atopic dermatitis skin is characterized by broad terminal differentiation defects and variable immune abnormalities.	J Allergy Clin Immunol.
atopic dermatitis	ANAPC5	Expression profiling by array, count, 3 development stage, 2 disease state, 11 individual sets	GPL570	GSE32924 Samples	Nonlesional atopic dermatitis skin is characterized by broad terminal differentiation defects and variable immune abnormalities.	J Allergy Clin Immunol.
atopic dermatitis	NA	Expression profiling by array, count, 3 development stage, 2 disease state, 11 individual sets	GPL570	GSE32924 Samples	Nonlesional atopic dermatitis skin is characterized by broad terminal differentiation defects and variable immune abnormalities.	J Allergy Clin Immunol.
atopic dermatitis	MAPKAP1	Expression profiling by array, count, 3 development stage, 2 disease state, 11 individual sets	GPL570	GSE32924 Samples	Nonlesional atopic dermatitis skin is characterized by broad terminal differentiation defects and variable immune abnormalities.	J Allergy Clin Immunol.

atopic dermatitis	RCAN1	Expression profiling by array, count, 3 development stage, 2 disease state, 11 individual sets	GPL570	GSE32924 Samples	Nonlesional atopic dermatitis skin is characterized by broad terminal differentiation defects and variable immune abnormalities.	J Allergy Clin Immunol.
atopic dermatitis	TOX4	Expression profiling by array, count, 3 development stage, 2 disease state, 11 individual sets	GPL570	GSE32924 Samples	Nonlesional atopic dermatitis skin is characterized by broad terminal differentiation defects and variable immune abnormalities.	J Allergy Clin Immunol.
atopic dermatitis	DHX9	Expression profiling by array, count, 3 development stage, 2 disease state, 11 individual sets	GPL570	GSE32924 Samples	Nonlesional atopic dermatitis skin is characterized by broad terminal differentiation defects and variable immune abnormalities.	J Allergy Clin Immunol.
atopic dermatitis	SLC39A6	Expression profiling by array, count, 3 development stage, 2 disease state, 11 individual sets	GPL570	GSE32924 Samples	Nonlesional atopic dermatitis skin is characterized by broad terminal differentiation defects and variable immune abnormalities.	J Allergy Clin Immunol.
atopic dermatitis	IL13RA1	Expression profiling by array, count, 3 development stage, 2 disease state, 11 individual sets	GPL570	GSE32924 Samples	Nonlesional atopic dermatitis skin is characterized by broad terminal differentiation defects and variable immune abnormalities.	J Allergy Clin Immunol.
atopic dermatitis	VPS35	Expression profiling by array, count, 3 development stage, 2 disease state, 11 individual sets	GPL570	GSE32924 Samples	Nonlesional atopic dermatitis skin is characterized by broad terminal differentiation defects and variable immune abnormalities.	J Allergy Clin Immunol.
atopic dermatitis	MFSD6	Expression profiling by array, count, 3 development stage, 2 disease state, 11 individual sets	GPL570	GSE32924 Samples	Nonlesional atopic dermatitis skin is characterized by broad terminal differentiation defects and variable immune abnormalities.	J Allergy Clin Immunol.
atopic dermatitis	TJP1	Expression profiling by array, count, 3 development stage, 2 disease state, 11 individual sets	GPL570	GSE32924 Samples	Nonlesional atopic dermatitis skin is characterized by broad terminal differentiation defects and variable immune abnormalities.	J Allergy Clin Immunol.
atopic dermatitis	CTTNBP2NL	Expression profiling by array, count, 3 development stage, 2 disease state, 11 individual sets	GPL570	GSE32924 Samples	Nonlesional atopic dermatitis skin is characterized by broad terminal differentiation defects and variable immune abnormalities.	J Allergy Clin Immunol.
atopic dermatitis	BTBD11	Expression profiling by array, count, 3 development stage, 2 disease state, 11 individual sets	GPL570	GSE32924 Samples	Nonlesional atopic dermatitis skin is characterized by broad terminal differentiation defects and variable immune abnormalities.	J Allergy Clin Immunol.
atopic dermatitis	KLHL7	Expression profiling by array, count, 3 development stage, 2 disease state, 11 individual sets	GPL570	GSE32924 Samples	Nonlesional atopic dermatitis skin is characterized by broad terminal differentiation defects and variable immune abnormalities.	J Allergy Clin Immunol.
atopic dermatitis	LGALS8	Expression profiling by array, count, 3 development stage, 2 disease state, 11 individual sets	GPL570	GSE32924 Samples	Nonlesional atopic dermatitis skin is characterized by broad terminal differentiation defects and variable immune abnormalities.	J Allergy Clin Immunol.
atopic dermatitis	ALOXE3	Expression profiling by array, count, 3 development stage, 2 disease state, 11 individual sets	GPL570	GSE32924 Samples	Nonlesional atopic dermatitis skin is characterized by broad terminal differentiation defects and variable immune abnormalities.	J Allergy Clin Immunol.
atopic dermatitis	PLD1	Expression profiling by array, count, 3 development stage, 2 disease state, 11 individual sets	GPL570	GSE32924 Samples	Nonlesional atopic dermatitis skin is characterized by broad terminal differentiation defects and variable immune abnormalities.	J Allergy Clin Immunol.
atopic dermatitis	PCSK6	Expression profiling by array, count, 3 development stage, 2 disease state, 11 individual sets	GPL570	GSE32924 Samples	Nonlesional atopic dermatitis skin is characterized by broad terminal differentiation defects and variable immune abnormalities.	J Allergy Clin Immunol.
atopic dermatitis	DLX5	Expression profiling by array, count, 3 development stage, 2 disease state, 11 individual sets	GPL570	GSE32924 Samples	Nonlesional atopic dermatitis skin is characterized by broad terminal differentiation defects and variable immune abnormalities.	J Allergy Clin Immunol.
atopic dermatitis	RHBDL2	Expression profiling by array, count, 3 development stage, 2 disease state, 11 individual sets	GPL570	GSE32924 Samples	Nonlesional atopic dermatitis skin is characterized by broad terminal differentiation defects and variable immune abnormalities.	J Allergy Clin Immunol.
atopic dermatitis	RHBDL2	Expression profiling by array, count, 3 development stage, 2 disease state, 11 individual sets	GPL570	GSE32924 Samples	Nonlesional atopic dermatitis skin is characterized by broad terminal differentiation defects and variable immune abnormalities.	J Allergy Clin Immunol.
atopic dermatitis	HSPC159	Expression profiling by array, count, 3 development stage, 2 disease state, 11 individual sets	GPL570	GSE32924 Samples	Nonlesional atopic dermatitis skin is characterized by broad terminal differentiation defects and variable immune abnormalities.	J Allergy Clin Immunol.
atopic dermatitis	MPP7	Expression profiling by array, count, 3 development stage, 2 disease state, 11 individual sets	GPL570	GSE32924 Samples	Nonlesional atopic dermatitis skin is characterized by broad terminal differentiation defects and variable immune abnormalities.	J Allergy Clin Immunol.
atopic dermatitis	IDE	Expression profiling by array, count, 3 development stage, 2 disease state, 11 individual sets	GPL570	GSE32924 Samples	Nonlesional atopic dermatitis skin is characterized by broad terminal differentiation defects and variable immune abnormalities.	J Allergy Clin Immunol.
atopic dermatitis	PTGS1	Expression profiling by array, count, 3 development stage, 2 disease state, 11 individual sets	GPL570	GSE32924 Samples	Nonlesional atopic dermatitis skin is characterized by broad terminal differentiation defects and variable immune abnormalities.	J Allergy Clin Immunol.
atopic dermatitis	C1ORF46	Expression profiling by array, count, 3 development stage, 2 disease state, 11 individual sets	GPL570	GSE32924 Samples	Nonlesional atopic dermatitis skin is characterized by broad terminal differentiation defects and variable immune abnormalities.	J Allergy Clin Immunol.
atopic dermatitis	WFDC12	Expression profiling by array, count, 3 development stage, 2 disease state, 11 individual sets	GPL570	GSE32924 Samples	Nonlesional atopic dermatitis skin is characterized by broad terminal differentiation defects and variable immune abnormalities.	J Allergy Clin Immunol.
atopic dermatitis	CDSN	Expression profiling by array, count, 3 development stage, 2 disease state, 11 individual sets	GPL570	GSE32924 Samples	Nonlesional atopic dermatitis skin is characterized by broad terminal differentiation defects and variable immune abnormalities.	J Allergy Clin Immunol.
atopic dermatitis	POF1B	Expression profiling by array, count, 3 development stage, 2 disease state, 11 individual sets	GPL570	GSE32924 Samples	Nonlesional atopic dermatitis skin is characterized by broad terminal differentiation defects and variable immune abnormalities.	J Allergy Clin Immunol.
atopic dermatitis	CRCT1	Expression profiling by array, count, 3 development stage, 2 disease state, 11 individual sets	GPL570	GSE32924 Samples	Nonlesional atopic dermatitis skin is characterized by broad terminal differentiation defects and variable immune abnormalities.	J Allergy Clin Immunol.
atopic dermatitis	ANXA9	Expression profiling by array, count, 3 development stage, 2 disease state, 11 individual sets	GPL570	GSE32924 Samples	Nonlesional atopic dermatitis skin is characterized by broad terminal differentiation defects and variable immune abnormalities.	J Allergy Clin Immunol.
atopic dermatitis	IL1F7	Expression profiling by array, count, 3 development stage, 2 disease state, 11 individual sets	GPL570	GSE32924 Samples	Nonlesional atopic dermatitis skin is characterized by broad terminal differentiation defects and variable immune abnormalities.	J Allergy Clin Immunol.
atopic dermatitis	BTC	Expression profiling by array, count, 3 development stage, 2 disease state, 11 individual sets	GPL570	GSE32924 Samples	Nonlesional atopic dermatitis skin is characterized by broad terminal differentiation defects and variable immune abnormalities.	J Allergy Clin Immunol.
atopic dermatitis	MAGI1	Expression profiling by array, count, 3 development stage, 2 disease state, 11 individual sets	GPL570	GSE32924 Samples	Nonlesional atopic dermatitis skin is characterized by broad terminal differentiation defects and variable immune abnormalities.	J Allergy Clin Immunol.
atopic dermatitis	GTDC1	Expression profiling by array, count, 3 development stage, 2 disease state, 11 individual sets	GPL570	GSE32924 Samples	Nonlesional atopic dermatitis skin is characterized by broad terminal differentiation defects and variable immune abnormalities.	J Allergy Clin Immunol.
atopic dermatitis	CDCP1	Expression profiling by array, count, 3 development stage, 2 disease state, 11 individual sets	GPL570	GSE32924 Samples	Nonlesional atopic dermatitis skin is characterized by broad terminal differentiation defects and variable immune abnormalities.	J Allergy Clin Immunol.
atopic dermatitis	TFE3	Expression profiling by array, count, 3 development stage, 2 disease state, 11 individual sets	GPL570	GSE32924 Samples	Nonlesional atopic dermatitis skin is characterized by broad terminal differentiation defects and variable immune abnormalities.	J Allergy Clin Immunol.
atopic dermatitis	BCL2L2	Expression profiling by array, count, 3 development stage, 2 disease state, 11 individual sets	GPL570	GSE32924 Samples	Nonlesional atopic dermatitis skin is characterized by broad terminal differentiation defects and variable immune abnormalities.	J Allergy Clin Immunol.
atopic dermatitis	EIF4EBP2	Expression profiling by array, count, 3 development stage, 2 disease state, 11 individual sets	GPL570	GSE32924 Samples	Nonlesional atopic dermatitis skin is characterized by broad terminal differentiation defects and variable immune abnormalities.	J Allergy Clin Immunol.
atopic dermatitis	B3GALNT2	Expression profiling by array, count, 3 development stage, 2 disease state, 11 individual sets	GPL570	GSE32924 Samples	Nonlesional atopic dermatitis skin is characterized by broad terminal differentiation defects and variable immune abnormalities.	J Allergy Clin Immunol.
atopic dermatitis	NA	Expression profiling by array, count, 3 development stage, 2 disease state, 11 individual sets	GPL570	GSE32924 Samples	Nonlesional atopic dermatitis skin is characterized by broad terminal differentiation defects and variable immune abnormalities.	J Allergy Clin Immunol.
atopic dermatitis	EXOSC6	Expression profiling by array, count, 3 development stage, 2 disease state, 11 individual sets	GPL570	GSE32924 Samples	Nonlesional atopic dermatitis skin is characterized by broad terminal differentiation defects and variable immune abnormalities.	J Allergy Clin Immunol.
atopic dermatitis	TP63	Expression profiling by array, count, 3 development stage, 2 disease state, 11 individual sets	GPL570	GSE32924 Samples	Nonlesional atopic dermatitis skin is characterized by broad terminal differentiation defects and variable immune abnormalities.	J Allergy Clin Immunol.
atopic dermatitis	C1ORF174	Expression profiling by array, count, 3 development stage, 2 disease state, 11 individual sets	GPL570	GSE32924 Samples	Nonlesional atopic dermatitis skin is characterized by broad terminal differentiation defects and variable immune abnormalities.	J Allergy Clin Immunol.

atopic dermatitis	MMP14	Expression profiling by array, count, 3 development stage, 2 disease state, 11 individual sets	GPL570	GSE32924 Samples	Nonlesional atopic dermatitis skin is characterized by broad terminal differentiation defects and variable immune abnormalities.	J Allergy Clin Immunol.
atopic dermatitis	EPAS1	Expression profiling by array, count, 3 development stage, 2 disease state, 11 individual sets	GPL570	GSE32924 Samples	Nonlesional atopic dermatitis skin is characterized by broad terminal differentiation defects and variable immune abnormalities.	J Allergy Clin Immunol.
atopic dermatitis	TP53	Expression profiling by array, count, 3 development stage, 2 disease state, 11 individual sets	GPL570	GSE32924 Samples	Nonlesional atopic dermatitis skin is characterized by broad terminal differentiation defects and variable immune abnormalities.	J Allergy Clin Immunol.
atopic dermatitis	GAS1	Expression profiling by array, count, 3 development stage, 2 disease state, 11 individual sets	GPL570	GSE32924 Samples	Nonlesional atopic dermatitis skin is characterized by broad terminal differentiation defects and variable immune abnormalities.	J Allergy Clin Immunol.
atopic dermatitis	ARHGEF7	Expression profiling by array, count, 3 development stage, 2 disease state, 11 individual sets	GPL570	GSE32924 Samples	Nonlesional atopic dermatitis skin is characterized by broad terminal differentiation defects and variable immune abnormalities.	J Allergy Clin Immunol.
atopic dermatitis	SUPT16H	Expression profiling by array, count, 3 development stage, 2 disease state, 11 individual sets	GPL570	GSE32924 Samples	Nonlesional atopic dermatitis skin is characterized by broad terminal differentiation defects and variable immune abnormalities.	J Allergy Clin Immunol.
atopic dermatitis	EXOC4	Expression profiling by array, count, 3 development stage, 2 disease state, 11 individual sets	GPL570	GSE32924 Samples	Nonlesional atopic dermatitis skin is characterized by broad terminal differentiation defects and variable immune abnormalities.	J Allergy Clin Immunol.
atopic dermatitis	NSMAF	Expression profiling by array, count, 3 development stage, 2 disease state, 11 individual sets	GPL570	GSE32924 Samples	Nonlesional atopic dermatitis skin is characterized by broad terminal differentiation defects and variable immune abnormalities.	J Allergy Clin Immunol.
atopic dermatitis	ABHD2	Expression profiling by array, count, 3 development stage, 2 disease state, 11 individual sets	GPL570	GSE32924 Samples	Nonlesional atopic dermatitis skin is characterized by broad terminal differentiation defects and variable immune abnormalities.	J Allergy Clin Immunol.
atopic dermatitis	ORMDL3	Expression profiling by array, count, 3 development stage, 2 disease state, 11 individual sets	GPL570	GSE32924 Samples	Nonlesional atopic dermatitis skin is characterized by broad terminal differentiation defects and variable immune abnormalities.	J Allergy Clin Immunol.
atopic dermatitis	ARHGAP29	Expression profiling by array, count, 3 development stage, 2 disease state, 11 individual sets	GPL570	GSE32924 Samples	Nonlesional atopic dermatitis skin is characterized by broad terminal differentiation defects and variable immune abnormalities.	J Allergy Clin Immunol.
atopic dermatitis	ABCC9	Expression profiling by array, count, 3 development stage, 2 disease state, 11 individual sets	GPL570	GSE32924 Samples	Nonlesional atopic dermatitis skin is characterized by broad terminal differentiation defects and variable immune abnormalities.	J Allergy Clin Immunol.
atopic dermatitis	UBASH3B	Expression profiling by array, count, 3 development stage, 2 disease state, 11 individual sets	GPL570	GSE32924 Samples	Nonlesional atopic dermatitis skin is characterized by broad terminal differentiation defects and variable immune abnormalities.	J Allergy Clin Immunol.
atopic dermatitis	PLEKHA2	Expression profiling by array, count, 3 development stage, 2 disease state, 11 individual sets	GPL570	GSE32924 Samples	Nonlesional atopic dermatitis skin is characterized by broad terminal differentiation defects and variable immune abnormalities.	J Allergy Clin Immunol.
atopic dermatitis	SFRS12	Expression profiling by array, count, 3 development stage, 2 disease state, 11 individual sets	GPL570	GSE32924 Samples	Nonlesional atopic dermatitis skin is characterized by broad terminal differentiation defects and variable immune abnormalities.	J Allergy Clin Immunol.
atopic dermatitis	NA	Expression profiling by array, count, 3 development stage, 2 disease state, 11 individual sets	GPL570	GSE32924 Samples	Nonlesional atopic dermatitis skin is characterized by broad terminal differentiation defects and variable immune abnormalities.	J Allergy Clin Immunol.
atopic dermatitis	PRDM6	Expression profiling by array, count, 3 development stage, 2 disease state, 11 individual sets	GPL570	GSE32924 Samples	Nonlesional atopic dermatitis skin is characterized by broad terminal differentiation defects and variable immune abnormalities.	J Allergy Clin Immunol.
atopic dermatitis	ARG1	Expression profiling by array, count, 3 development stage, 2 disease state, 11 individual sets	GPL570	GSE32924 Samples	Nonlesional atopic dermatitis skin is characterized by broad terminal differentiation defects and variable immune abnormalities.	J Allergy Clin Immunol.
atopic dermatitis	COPA	Expression profiling by array, count, 3 development stage, 2 disease state, 11 individual sets	GPL570	GSE32924 Samples	Nonlesional atopic dermatitis skin is characterized by broad terminal differentiation defects and variable immune abnormalities.	J Allergy Clin Immunol.
atopic dermatitis	TNPO2	Expression profiling by array, count, 3 development stage, 2 disease state, 11 individual sets	GPL570	GSE32924 Samples	Nonlesional atopic dermatitis skin is characterized by broad terminal differentiation defects and variable immune abnormalities.	J Allergy Clin Immunol.
atopic dermatitis	DHX9	Expression profiling by array, count, 3 development stage, 2 disease state, 11 individual sets	GPL570	GSE32924 Samples	Nonlesional atopic dermatitis skin is characterized by broad terminal differentiation defects and variable immune abnormalities.	J Allergy Clin Immunol.
atopic dermatitis	KCTD20	Expression profiling by array, count, 3 development stage, 2 disease state, 11 individual sets	GPL570	GSE32924 Samples	Nonlesional atopic dermatitis skin is characterized by broad terminal differentiation defects and variable immune abnormalities.	J Allergy Clin Immunol.
atopic dermatitis	PDXDC1	Expression profiling by array, count, 3 development stage, 2 disease state, 11 individual sets	GPL570	GSE32924 Samples	Nonlesional atopic dermatitis skin is characterized by broad terminal differentiation defects and variable immune abnormalities.	J Allergy Clin Immunol.
atopic dermatitis	TMEM86A	Expression profiling by array, count, 3 development stage, 2 disease state, 11 individual sets	GPL570	GSE32924 Samples	Nonlesional atopic dermatitis skin is characterized by broad terminal differentiation defects and variable immune abnormalities.	J Allergy Clin Immunol.
atopic dermatitis	C13ORF36	Expression profiling by array, count, 3 development stage, 2 disease state, 11 individual sets	GPL570	GSE32924 Samples	Nonlesional atopic dermatitis skin is characterized by broad terminal differentiation defects and variable immune abnormalities.	J Allergy Clin Immunol.
atopic dermatitis	CNOT6L	Expression profiling by array, count, 3 development stage, 2 disease state, 11 individual sets	GPL570	GSE32924 Samples	Nonlesional atopic dermatitis skin is characterized by broad terminal differentiation defects and variable immune abnormalities.	J Allergy Clin Immunol.
atopic dermatitis	TFAP2C	Expression profiling by array, count, 3 development stage, 2 disease state, 11 individual sets	GPL570	GSE32924 Samples	Nonlesional atopic dermatitis skin is characterized by broad terminal differentiation defects and variable immune abnormalities.	J Allergy Clin Immunol.
atopic dermatitis	SLC6A9	Expression profiling by array, count, 3 development stage, 2 disease state, 11 individual sets	GPL570	GSE32924 Samples	Nonlesional atopic dermatitis skin is characterized by broad terminal differentiation defects and variable immune abnormalities.	J Allergy Clin Immunol.
atopic dermatitis	LUZP6	Expression profiling by array, count, 3 development stage, 2 disease state, 11 individual sets	GPL570	GSE32924 Samples	Nonlesional atopic dermatitis skin is characterized by broad terminal differentiation defects and variable immune abnormalities.	J Allergy Clin Immunol.
atopic dermatitis	LYRM5	Expression profiling by array, count, 3 development stage, 2 disease state, 11 individual sets	GPL570	GSE32924 Samples	Nonlesional atopic dermatitis skin is characterized by broad terminal differentiation defects and variable immune abnormalities.	J Allergy Clin Immunol.
atopic dermatitis	GATM	Expression profiling by array, count, 3 development stage, 2 disease state, 11 individual sets	GPL570	GSE32924 Samples	Nonlesional atopic dermatitis skin is characterized by broad terminal differentiation defects and variable immune abnormalities.	J Allergy Clin Immunol.
atopic dermatitis	TNFRSF19	Expression profiling by array, count, 3 development stage, 2 disease state, 11 individual sets	GPL570	GSE32924 Samples	Nonlesional atopic dermatitis skin is characterized by broad terminal differentiation defects and variable immune abnormalities.	J Allergy Clin Immunol.
atopic dermatitis	TNFRSF19	Expression profiling by array, count, 3 development stage, 2 disease state, 11 individual sets	GPL570	GSE32924 Samples	Nonlesional atopic dermatitis skin is characterized by broad terminal differentiation defects and variable immune abnormalities.	J Allergy Clin Immunol.
atopic dermatitis	IL1RN	Expression profiling by array, count, 3 development stage, 2 disease state, 11 individual sets	GPL570	GSE32924 Samples	Nonlesional atopic dermatitis skin is characterized by broad terminal differentiation defects and variable immune abnormalities.	J Allergy Clin Immunol.
atopic dermatitis	ZNF770	Expression profiling by array, count, 3 development stage, 2 disease state, 11 individual sets	GPL570	GSE32924 Samples	Nonlesional atopic dermatitis skin is characterized by broad terminal differentiation defects and variable immune abnormalities.	J Allergy Clin Immunol.
atopic dermatitis	LOC284242	Expression profiling by array, count, 3 development stage, 2 disease state, 11 individual sets	GPL570	GSE32924 Samples	Nonlesional atopic dermatitis skin is characterized by broad terminal differentiation defects and variable immune abnormalities.	J Allergy Clin Immunol.
atopic dermatitis	NA	Expression profiling by array, count, 3 development stage, 2 disease state, 11 individual sets	GPL570	GSE32924 Samples	Nonlesional atopic dermatitis skin is characterized by broad terminal differentiation defects and variable immune abnormalities.	J Allergy Clin Immunol.
atopic dermatitis	ACTR2	Expression profiling by array, count, 3 development stage, 2 disease state, 11 individual sets	GPL570	GSE32924 Samples	Nonlesional atopic dermatitis skin is characterized by broad terminal differentiation defects and variable immune abnormalities.	J Allergy Clin Immunol.
atopic dermatitis	ACTR2	Expression profiling by array, count, 3 development stage, 2 disease state, 11 individual sets	GPL570	GSE32924 Samples	Nonlesional atopic dermatitis skin is characterized by broad terminal differentiation defects and variable immune abnormalities.	J Allergy Clin Immunol.
atopic dermatitis	GM2A	Expression profiling by array, count, 3 development stage, 2 disease state, 11 individual sets	GPL570	GSE32924 Samples	Nonlesional atopic dermatitis skin is characterized by broad terminal differentiation defects and variable immune abnormalities.	J Allergy Clin Immunol.
atopic dermatitis	GM2A	Expression profiling by array, count, 3 development stage, 2 disease state, 11 individual sets	GPL570	GSE32924 Samples	Nonlesional atopic dermatitis skin is characterized by broad terminal differentiation defects and variable immune abnormalities.	J Allergy Clin Immunol.

atopic dermatitis	CASP14	Expression profiling by array, count, 3 development stage, 2 disease state, 11 individual sets	GPL570	GSE32924 Samples	Nonlesional atopic dermatitis skin is characterized by broad terminal differentiation defects and variable immune abnormalities.	J Allergy Clin Immunol.
atopic dermatitis	SERPINB12	Expression profiling by array, count, 3 development stage, 2 disease state, 11 individual sets	GPL570	GSE32924 Samples	Nonlesional atopic dermatitis skin is characterized by broad terminal differentiation defects and variable immune abnormalities.	J Allergy Clin Immunol.
atopic dermatitis	MAP3K1	Expression profiling by array, count, 3 development stage, 2 disease state, 11 individual sets	GPL570	GSE32924 Samples	Nonlesional atopic dermatitis skin is characterized by broad terminal differentiation defects and variable immune abnormalities.	J Allergy Clin Immunol.
atopic dermatitis	CCDC123	Expression profiling by array, count, 3 development stage, 2 disease state, 11 individual sets	GPL570	GSE32924 Samples	Nonlesional atopic dermatitis skin is characterized by broad terminal differentiation defects and variable immune abnormalities.	J Allergy Clin Immunol.
atopic dermatitis	NA	Expression profiling by array, count, 3 development stage, 2 disease state, 11 individual sets	GPL570	GSE32924 Samples	Nonlesional atopic dermatitis skin is characterized by broad terminal differentiation defects and variable immune abnormalities.	J Allergy Clin Immunol.
atopic dermatitis	MCOLN3	Expression profiling by array, count, 3 development stage, 2 disease state, 11 individual sets	GPL570	GSE32924 Samples	Nonlesional atopic dermatitis skin is characterized by broad terminal differentiation defects and variable immune abnormalities.	J Allergy Clin Immunol.
atopic dermatitis	NA	Expression profiling by array, count, 3 development stage, 2 disease state, 11 individual sets	GPL570	GSE32924 Samples	Nonlesional atopic dermatitis skin is characterized by broad terminal differentiation defects and variable immune abnormalities.	J Allergy Clin Immunol.
atopic dermatitis	FMNL2	Expression profiling by array, count, 3 development stage, 2 disease state, 11 individual sets	GPL570	GSE32924 Samples	Nonlesional atopic dermatitis skin is characterized by broad terminal differentiation defects and variable immune abnormalities.	J Allergy Clin Immunol.
atopic dermatitis	PPFIBP1	Expression profiling by array, count, 3 development stage, 2 disease state, 11 individual sets	GPL570	GSE32924 Samples	Nonlesional atopic dermatitis skin is characterized by broad terminal differentiation defects and variable immune abnormalities.	J Allergy Clin Immunol.
atopic dermatitis	SMCHD1	Expression profiling by array, count, 3 development stage, 2 disease state, 11 individual sets	GPL570	GSE32924 Samples	Nonlesional atopic dermatitis skin is characterized by broad terminal differentiation defects and variable immune abnormalities.	J Allergy Clin Immunol.
atopic dermatitis	LARP1B	Expression profiling by array, count, 3 development stage, 2 disease state, 11 individual sets	GPL570	GSE32924 Samples	Nonlesional atopic dermatitis skin is characterized by broad terminal differentiation defects and variable immune abnormalities.	J Allergy Clin Immunol.
atopic dermatitis	NA	Expression profiling by array, count, 3 development stage, 2 disease state, 11 individual sets	GPL570	GSE32924 Samples	Nonlesional atopic dermatitis skin is characterized by broad terminal differentiation defects and variable immune abnormalities.	J Allergy Clin Immunol.
atopic dermatitis	PIK3R1	Expression profiling by array, count, 3 development stage, 2 disease state, 11 individual sets	GPL570	GSE32924 Samples	Nonlesional atopic dermatitis skin is characterized by broad terminal differentiation defects and variable immune abnormalities.	J Allergy Clin Immunol.
atopic dermatitis	NA	Expression profiling by array, count, 3 development stage, 2 disease state, 11 individual sets	GPL570	GSE32924 Samples	Nonlesional atopic dermatitis skin is characterized by broad terminal differentiation defects and variable immune abnormalities.	J Allergy Clin Immunol.
atopic dermatitis	HECTD1	Expression profiling by array, count, 3 development stage, 2 disease state, 11 individual sets	GPL570	GSE32924 Samples	Nonlesional atopic dermatitis skin is characterized by broad terminal differentiation defects and variable immune abnormalities.	J Allergy Clin Immunol.
atopic dermatitis	NA	Expression profiling by array, count, 3 development stage, 2 disease state, 11 individual sets	GPL570	GSE32924 Samples	Nonlesional atopic dermatitis skin is characterized by broad terminal differentiation defects and variable immune abnormalities.	J Allergy Clin Immunol.
atopic dermatitis	NA	Expression profiling by array, count, 3 development stage, 2 disease state, 11 individual sets	GPL570	GSE32924 Samples	Nonlesional atopic dermatitis skin is characterized by broad terminal differentiation defects and variable immune abnormalities.	J Allergy Clin Immunol.
atopic dermatitis	MRVI1	Expression profiling by array, count, 3 development stage, 2 disease state, 11 individual sets	GPL570	GSE32924 Samples	Nonlesional atopic dermatitis skin is characterized by broad terminal differentiation defects and variable immune abnormalities.	J Allergy Clin Immunol.
atopic dermatitis	BOD1L	Expression profiling by array, count, 3 development stage, 2 disease state, 11 individual sets	GPL570	GSE32924 Samples	Nonlesional atopic dermatitis skin is characterized by broad terminal differentiation defects and variable immune abnormalities.	J Allergy Clin Immunol.
atopic dermatitis	NA	Expression profiling by array, count, 3 development stage, 2 disease state, 11 individual sets	GPL570	GSE32924 Samples	Nonlesional atopic dermatitis skin is characterized by broad terminal differentiation defects and variable immune abnormalities.	J Allergy Clin Immunol.
atopic dermatitis	LOC149832	Expression profiling by array, count, 3 development stage, 2 disease state, 11 individual sets	GPL570	GSE32924 Samples	Nonlesional atopic dermatitis skin is characterized by broad terminal differentiation defects and variable immune abnormalities.	J Allergy Clin Immunol.
atopic dermatitis	ADAMTSL3	Expression profiling by array, count, 3 development stage, 2 disease state, 11 individual sets	GPL570	GSE32924 Samples	Nonlesional atopic dermatitis skin is characterized by broad terminal differentiation defects and variable immune abnormalities.	J Allergy Clin Immunol.
atopic dermatitis	TAC1	Expression profiling by array, count, 3 development stage, 2 disease state, 11 individual sets	GPL570	GSE32924 Samples	Nonlesional atopic dermatitis skin is characterized by broad terminal differentiation defects and variable immune abnormalities.	J Allergy Clin Immunol.
atopic dermatitis	NA	Expression profiling by array, count, 3 development stage, 2 disease state, 11 individual sets	GPL570	GSE32924 Samples	Nonlesional atopic dermatitis skin is characterized by broad terminal differentiation defects and variable immune abnormalities.	J Allergy Clin Immunol.
atopic dermatitis	NA	Expression profiling by array, count, 3 development stage, 2 disease state, 11 individual sets	GPL570	GSE32924 Samples	Nonlesional atopic dermatitis skin is characterized by broad terminal differentiation defects and variable immune abnormalities.	J Allergy Clin Immunol.
atopic dermatitis	FAM108B1	Expression profiling by array, count, 3 development stage, 2 disease state, 11 individual sets	GPL570	GSE32924 Samples	Nonlesional atopic dermatitis skin is characterized by broad terminal differentiation defects and variable immune abnormalities.	J Allergy Clin Immunol.
atopic dermatitis	ZNF711	Expression profiling by array, count, 3 development stage, 2 disease state, 11 individual sets	GPL570	GSE32924 Samples	Nonlesional atopic dermatitis skin is characterized by broad terminal differentiation defects and variable immune abnormalities.	J Allergy Clin Immunol.
atopic dermatitis	ANKFN1	Expression profiling by array, count, 3 development stage, 2 disease state, 11 individual sets	GPL570	GSE32924 Samples	Nonlesional atopic dermatitis skin is characterized by broad terminal differentiation defects and variable immune abnormalities.	J Allergy Clin Immunol.
atopic dermatitis	IL1F10	Expression profiling by array, count, 3 development stage, 2 disease state, 11 individual sets	GPL570	GSE32924 Samples	Nonlesional atopic dermatitis skin is characterized by broad terminal differentiation defects and variable immune abnormalities.	J Allergy Clin Immunol.
atopic dermatitis	CLDN23	Expression profiling by array, count, 3 development stage, 2 disease state, 11 individual sets	GPL570	GSE32924 Samples	Nonlesional atopic dermatitis skin is characterized by broad terminal differentiation defects and variable immune abnormalities.	J Allergy Clin Immunol.
atopic dermatitis	HECTD1	Expression profiling by array, count, 3 development stage, 2 disease state, 11 individual sets	GPL570	GSE32924 Samples	Nonlesional atopic dermatitis skin is characterized by broad terminal differentiation defects and variable immune abnormalities.	J Allergy Clin Immunol.
atopic dermatitis	CYP2C9	Expression profiling by array, count, 3 development stage, 2 disease state, 11 individual sets	GPL570	GSE32924 Samples	Nonlesional atopic dermatitis skin is characterized by broad terminal differentiation defects and variable immune abnormalities.	J Allergy Clin Immunol.
atopic dermatitis	XK	Expression profiling by array, count, 3 development stage, 2 disease state, 11 individual sets	GPL570	GSE32924 Samples	Nonlesional atopic dermatitis skin is characterized by broad terminal differentiation defects and variable immune abnormalities.	J Allergy Clin Immunol.
atopic dermatitis	PLEKHA5	Expression profiling by array, count, 3 development stage, 2 disease state, 11 individual sets	GPL570	GSE32924 Samples	Nonlesional atopic dermatitis skin is characterized by broad terminal differentiation defects and variable immune abnormalities.	J Allergy Clin Immunol.
atopic dermatitis	KANK1	Expression profiling by array, count, 3 development stage, 2 disease state, 11 individual sets	GPL570	GSE32924 Samples	Nonlesional atopic dermatitis skin is characterized by broad terminal differentiation defects and variable immune abnormalities.	J Allergy Clin Immunol.
atopic dermatitis	NARG1	Expression profiling by array, count, 3 development stage, 2 disease state, 11 individual sets	GPL570	GSE32924 Samples	Nonlesional atopic dermatitis skin is characterized by broad terminal differentiation defects and variable immune abnormalities.	J Allergy Clin Immunol.
atopic dermatitis	CP	Expression profiling by array, count, 3 development stage, 2 disease state, 11 individual sets	GPL570	GSE32924 Samples	Nonlesional atopic dermatitis skin is characterized by broad terminal differentiation defects and variable immune abnormalities.	J Allergy Clin Immunol.
atopic dermatitis	NA	Expression profiling by array, count, 3 development stage, 2 disease state, 11 individual sets	GPL570	GSE32924 Samples	Nonlesional atopic dermatitis skin is characterized by broad terminal differentiation defects and variable immune abnormalities.	J Allergy Clin Immunol.
atopic dermatitis	UGGT2	Expression profiling by array, count, 3 development stage, 2 disease state, 11 individual sets	GPL570	GSE32924 Samples	Nonlesional atopic dermatitis skin is characterized by broad terminal differentiation defects and variable immune abnormalities.	J Allergy Clin Immunol.
atopic dermatitis	SYNE1	Expression profiling by array, count, 3 development stage, 2 disease state, 11 individual sets	GPL570	GSE32924 Samples	Nonlesional atopic dermatitis skin is characterized by broad terminal differentiation defects and variable immune abnormalities.	J Allergy Clin Immunol.
atopic dermatitis	SEPP1	Expression profiling by array, count, 3 development stage, 2 disease state, 11 individual sets	GPL570	GSE32924 Samples	Nonlesional atopic dermatitis skin is characterized by broad terminal differentiation defects and variable immune abnormalities.	J Allergy Clin Immunol.

atopic dermatitis	REL	Expression profiling by array, count, 3 development stage, 2 disease state, 11 individual sets	GPL570	GSE32924 Samples	Nonlesional atopic dermatitis skin is characterized by broad terminal differentiation defects and variable immune abnormalities.	J Allergy Clin Immunol.
atopic dermatitis	MALAT1	Expression profiling by array, count, 3 development stage, 2 disease state, 11 individual sets	GPL570	GSE32924 Samples	Nonlesional atopic dermatitis skin is characterized by broad terminal differentiation defects and variable immune abnormalities.	J Allergy Clin Immunol.
atopic dermatitis	CHD9	Expression profiling by array, count, 3 development stage, 2 disease state, 11 individual sets	GPL570	GSE32924 Samples	Nonlesional atopic dermatitis skin is characterized by broad terminal differentiation defects and variable immune abnormalities.	J Allergy Clin Immunol.
atopic dermatitis	CLINT1	Expression profiling by array, count, 3 development stage, 2 disease state, 11 individual sets	GPL570	GSE32924 Samples	Nonlesional atopic dermatitis skin is characterized by broad terminal differentiation defects and variable immune abnormalities.	J Allergy Clin Immunol.
atopic dermatitis	WAC	Expression profiling by array, count, 3 development stage, 2 disease state, 11 individual sets	GPL570	GSE32924 Samples	Nonlesional atopic dermatitis skin is characterized by broad terminal differentiation defects and variable immune abnormalities.	J Allergy Clin Immunol.
atopic dermatitis	EEA1	Expression profiling by array, count, 3 development stage, 2 disease state, 11 individual sets	GPL570	GSE32924 Samples	Nonlesional atopic dermatitis skin is characterized by broad terminal differentiation defects and variable immune abnormalities.	J Allergy Clin Immunol.
atopic dermatitis	NA	Expression profiling by array, count, 3 development stage, 2 disease state, 11 individual sets	GPL570	GSE32924 Samples	Nonlesional atopic dermatitis skin is characterized by broad terminal differentiation defects and variable immune abnormalities.	J Allergy Clin Immunol.
atopic dermatitis	NA	Expression profiling by array, count, 3 development stage, 2 disease state, 11 individual sets	GPL570	GSE32924 Samples	Nonlesional atopic dermatitis skin is characterized by broad terminal differentiation defects and variable immune abnormalities.	J Allergy Clin Immunol.
atopic dermatitis	HHATL	Expression profiling by array, count, 3 development stage, 2 disease state, 11 individual sets	GPL570	GSE32924 Samples	Nonlesional atopic dermatitis skin is characterized by broad terminal differentiation defects and variable immune abnormalities.	J Allergy Clin Immunol.
atopic dermatitis	SLC44A5	Expression profiling by array, count, 3 development stage, 2 disease state, 11 individual sets	GPL570	GSE32924 Samples	Nonlesional atopic dermatitis skin is characterized by broad terminal differentiation defects and variable immune abnormalities.	J Allergy Clin Immunol.
atopic dermatitis	UPK1A	Expression profiling by array, count, 3 development stage, 2 disease state, 11 individual sets	GPL570	GSE32924 Samples	Nonlesional atopic dermatitis skin is characterized by broad terminal differentiation defects and variable immune abnormalities.	J Allergy Clin Immunol.
atopic dermatitis	PSG1	Expression profiling by array, count, 3 development stage, 2 disease state, 11 individual sets	GPL570	GSE32924 Samples	Nonlesional atopic dermatitis skin is characterized by broad terminal differentiation defects and variable immune abnormalities.	J Allergy Clin Immunol.
atopic dermatitis	PSG7	Expression profiling by array, count, 3 development stage, 2 disease state, 11 individual sets	GPL570	GSE32924 Samples	Nonlesional atopic dermatitis skin is characterized by broad terminal differentiation defects and variable immune abnormalities.	J Allergy Clin Immunol.
atopic dermatitis	DDX17	Expression profiling by array, count, 3 development stage, 2 disease state, 11 individual sets	GPL570	GSE32924 Samples	Nonlesional atopic dermatitis skin is characterized by broad terminal differentiation defects and variable immune abnormalities.	J Allergy Clin Immunol.
atopic dermatitis	KCTD4	Expression profiling by array, count, 3 development stage, 2 disease state, 11 individual sets	GPL570	GSE32924 Samples	Nonlesional atopic dermatitis skin is characterized by broad terminal differentiation defects and variable immune abnormalities.	J Allergy Clin Immunol.
atopic dermatitis	NA	Expression profiling by array, count, 3 development stage, 2 disease state, 11 individual sets	GPL570	GSE32924 Samples	Nonlesional atopic dermatitis skin is characterized by broad terminal differentiation defects and variable immune abnormalities.	J Allergy Clin Immunol.
atopic dermatitis	GPR115	Expression profiling by array, count, 3 development stage, 2 disease state, 11 individual sets	GPL570	GSE32924 Samples	Nonlesional atopic dermatitis skin is characterized by broad terminal differentiation defects and variable immune abnormalities.	J Allergy Clin Immunol.
atopic dermatitis	PTPN21	Expression profiling by array, count, 3 development stage, 2 disease state, 11 individual sets	GPL570	GSE32924 Samples	Nonlesional atopic dermatitis skin is characterized by broad terminal differentiation defects and variable immune abnormalities.	J Allergy Clin Immunol.
atopic dermatitis	LCE1E	Expression profiling by array, count, 3 development stage, 2 disease state, 11 individual sets	GPL570	GSE32924 Samples	Nonlesional atopic dermatitis skin is characterized by broad terminal differentiation defects and variable immune abnormalities.	J Allergy Clin Immunol.
atopic dermatitis	BTC	Expression profiling by array, count, 3 development stage, 2 disease state, 11 individual sets	GPL570	GSE32924 Samples	Nonlesional atopic dermatitis skin is characterized by broad terminal differentiation defects and variable immune abnormalities.	J Allergy Clin Immunol.
atopic dermatitis	BMP7	Expression profiling by array, count, 3 development stage, 2 disease state, 11 individual sets	GPL570	GSE32924 Samples	Nonlesional atopic dermatitis skin is characterized by broad terminal differentiation defects and variable immune abnormalities.	J Allergy Clin Immunol.
atopic dermatitis	NA	Expression profiling by array, count, 3 development stage, 2 disease state, 11 individual sets	GPL570	GSE32924 Samples	Nonlesional atopic dermatitis skin is characterized by broad terminal differentiation defects and variable immune abnormalities.	J Allergy Clin Immunol.
atopic dermatitis	ATP6V1A	Expression profiling by array, count, 3 development stage, 2 disease state, 11 individual sets	GPL570	GSE32924 Samples	Nonlesional atopic dermatitis skin is characterized by broad terminal differentiation defects and variable immune abnormalities.	J Allergy Clin Immunol.
atopic dermatitis	AHNAK	Expression profiling by array, count, 3 development stage, 2 disease state, 11 individual sets	GPL570	GSE32924 Samples	Nonlesional atopic dermatitis skin is characterized by broad terminal differentiation defects and variable immune abnormalities.	J Allergy Clin Immunol.
atopic dermatitis	NA	Expression profiling by array, count, 3 development stage, 2 disease state, 11 individual sets	GPL570	GSE32924 Samples	Nonlesional atopic dermatitis skin is characterized by broad terminal differentiation defects and variable immune abnormalities.	J Allergy Clin Immunol.
atopic dermatitis	SP8	Expression profiling by array, count, 3 development stage, 2 disease state, 11 individual sets	GPL570	GSE32924 Samples	Nonlesional atopic dermatitis skin is characterized by broad terminal differentiation defects and variable immune abnormalities.	J Allergy Clin Immunol.
atopic dermatitis	AGR3	Expression profiling by array, count, 3 development stage, 2 disease state, 11 individual sets	GPL570	GSE32924 Samples	Nonlesional atopic dermatitis skin is characterized by broad terminal differentiation defects and variable immune abnormalities.	J Allergy Clin Immunol.
atopic dermatitis	ABCA13	Expression profiling by array, count, 3 development stage, 2 disease state, 11 individual sets	GPL570	GSE32924 Samples	Nonlesional atopic dermatitis skin is characterized by broad terminal differentiation defects and variable immune abnormalities.	J Allergy Clin Immunol.
atopic dermatitis	ABCA13	Expression profiling by array, count, 3 development stage, 2 disease state, 11 individual sets	GPL570	GSE32924 Samples	Nonlesional atopic dermatitis skin is characterized by broad terminal differentiation defects and variable immune abnormalities.	J Allergy Clin Immunol.
atopic dermatitis	MSMB	Expression profiling by array, count, 3 development stage, 2 disease state, 11 individual sets	GPL570	GSE32924 Samples	Nonlesional atopic dermatitis skin is characterized by broad terminal differentiation defects and variable immune abnormalities.	J Allergy Clin Immunol.
atopic dermatitis	MSMB	Expression profiling by array, count, 3 development stage, 2 disease state, 11 individual sets	GPL570	GSE32924 Samples	Nonlesional atopic dermatitis skin is characterized by broad terminal differentiation defects and variable immune abnormalities.	J Allergy Clin Immunol.
atopic dermatitis	ARPC5	Expression profiling by array, count, 3 development stage, 2 disease state, 11 individual sets	GPL570	GSE32924 Samples	Nonlesional atopic dermatitis skin is characterized by broad terminal differentiation defects and variable immune abnormalities.	J Allergy Clin Immunol.
atopic dermatitis	CCT2	Expression profiling by array, count, 3 development stage, 2 disease state, 11 individual sets	GPL570	GSE32924 Samples	Nonlesional atopic dermatitis skin is characterized by broad terminal differentiation defects and variable immune abnormalities.	J Allergy Clin Immunol.
atopic dermatitis	VAMP3	Expression profiling by array, count, 3 development stage, 2 disease state, 11 individual sets	GPL570	GSE32924 Samples	Nonlesional atopic dermatitis skin is characterized by broad terminal differentiation defects and variable immune abnormalities.	J Allergy Clin Immunol.
atopic dermatitis	DAZAP2	Expression profiling by array, count, 3 development stage, 2 disease state, 11 individual sets	GPL570	GSE32924 Samples	Nonlesional atopic dermatitis skin is characterized by broad terminal differentiation defects and variable immune abnormalities.	J Allergy Clin Immunol.
atopic dermatitis	NAT13	Expression profiling by array, count, 3 development stage, 2 disease state, 11 individual sets	GPL570	GSE32924 Samples	Nonlesional atopic dermatitis skin is characterized by broad terminal differentiation defects and variable immune abnormalities.	J Allergy Clin Immunol.
atopic dermatitis	SCOC	Expression profiling by array, count, 3 development stage, 2 disease state, 11 individual sets	GPL570	GSE32924 Samples	Nonlesional atopic dermatitis skin is characterized by broad terminal differentiation defects and variable immune abnormalities.	J Allergy Clin Immunol.
atopic dermatitis	GDE1	Expression profiling by array, count, 3 development stage, 2 disease state, 11 individual sets	GPL570	GSE32924 Samples	Nonlesional atopic dermatitis skin is characterized by broad terminal differentiation defects and variable immune abnormalities.	J Allergy Clin Immunol.
atopic dermatitis	NA	Expression profiling by array, count, 3 development stage, 2 disease state, 11 individual sets	GPL570	GSE32924 Samples	Nonlesional atopic dermatitis skin is characterized by broad terminal differentiation defects and variable immune abnormalities.	J Allergy Clin Immunol.
atopic dermatitis	ASAH1	Expression profiling by array, count, 3 development stage, 2 disease state, 11 individual sets	GPL570	GSE32924 Samples	Nonlesional atopic dermatitis skin is characterized by broad terminal differentiation defects and variable immune abnormalities.	J Allergy Clin Immunol.
atopic dermatitis	CAT	Expression profiling by array, count, 3 development stage, 2 disease state, 11 individual sets	GPL570	GSE32924 Samples	Nonlesional atopic dermatitis skin is characterized by broad terminal differentiation defects and variable immune abnormalities.	J Allergy Clin Immunol.

atopic dermatitis	HLF	Expression profiling by array, count, 3 development stage, 2 disease state, 11 individual sets	GPL570	GSE32924 Samples	Nonlesional atopic dermatitis skin is characterized by broad terminal differentiation defects and variable immune abnormalities.	J Allergy Clin Immunol.
atopic dermatitis	GGTA1	Expression profiling by array, count, 3 development stage, 2 disease state, 11 individual sets	GPL570	GSE32924 Samples	Nonlesional atopic dermatitis skin is characterized by broad terminal differentiation defects and variable immune abnormalities.	J Allergy Clin Immunol.
atopic dermatitis	NA	Expression profiling by array, count, 3 development stage, 2 disease state, 11 individual sets	GPL570	GSE32924 Samples	Nonlesional atopic dermatitis skin is characterized by broad terminal differentiation defects and variable immune abnormalities.	J Allergy Clin Immunol.
atopic dermatitis	SGPL1	Expression profiling by array, count, 3 development stage, 2 disease state, 11 individual sets	GPL570	GSE32924 Samples	Nonlesional atopic dermatitis skin is characterized by broad terminal differentiation defects and variable immune abnormalities.	J Allergy Clin Immunol.
atopic dermatitis	RMND5A	Expression profiling by array, count, 3 development stage, 2 disease state, 11 individual sets	GPL570	GSE32924 Samples	Nonlesional atopic dermatitis skin is characterized by broad terminal differentiation defects and variable immune abnormalities.	J Allergy Clin Immunol.
atopic dermatitis	HSDL2	Expression profiling by array, count, 3 development stage, 2 disease state, 11 individual sets	GPL570	GSE32924 Samples	Nonlesional atopic dermatitis skin is characterized by broad terminal differentiation defects and variable immune abnormalities.	J Allergy Clin Immunol.
atopic dermatitis	GNS	Expression profiling by array, count, 3 development stage, 2 disease state, 11 individual sets	GPL570	GSE32924 Samples	Nonlesional atopic dermatitis skin is characterized by broad terminal differentiation defects and variable immune abnormalities.	J Allergy Clin Immunol.
atopic dermatitis	AIDA	Expression profiling by array, count, 3 development stage, 2 disease state, 11 individual sets	GPL570	GSE32924 Samples	Nonlesional atopic dermatitis skin is characterized by broad terminal differentiation defects and variable immune abnormalities.	J Allergy Clin Immunol.
atopic dermatitis	TSPAN3	Expression profiling by array, count, 3 development stage, 2 disease state, 11 individual sets	GPL570	GSE32924 Samples	Nonlesional atopic dermatitis skin is characterized by broad terminal differentiation defects and variable immune abnormalities.	J Allergy Clin Immunol.
atopic dermatitis	TRAM1	Expression profiling by array, count, 3 development stage, 2 disease state, 11 individual sets	GPL570	GSE32924 Samples	Nonlesional atopic dermatitis skin is characterized by broad terminal differentiation defects and variable immune abnormalities.	J Allergy Clin Immunol.
atopic dermatitis	CNOT8	Expression profiling by array, count, 3 development stage, 2 disease state, 11 individual sets	GPL570	GSE32924 Samples	Nonlesional atopic dermatitis skin is characterized by broad terminal differentiation defects and variable immune abnormalities.	J Allergy Clin Immunol.
atopic dermatitis	FAM107B	Expression profiling by array, count, 3 development stage, 2 disease state, 11 individual sets	GPL570	GSE32924 Samples	Nonlesional atopic dermatitis skin is characterized by broad terminal differentiation defects and variable immune abnormalities.	J Allergy Clin Immunol.
atopic dermatitis	FAM107B	Expression profiling by array, count, 3 development stage, 2 disease state, 11 individual sets	GPL570	GSE32924 Samples	Nonlesional atopic dermatitis skin is characterized by broad terminal differentiation defects and variable immune abnormalities.	J Allergy Clin Immunol.
atopic dermatitis	SH3KBP1	Expression profiling by array, count, 3 development stage, 2 disease state, 11 individual sets	GPL570	GSE32924 Samples	Nonlesional atopic dermatitis skin is characterized by broad terminal differentiation defects and variable immune abnormalities.	J Allergy Clin Immunol.
atopic dermatitis	SMOC2	Expression profiling by array, count, 3 development stage, 2 disease state, 11 individual sets	GPL570	GSE32924 Samples	Nonlesional atopic dermatitis skin is characterized by broad terminal differentiation defects and variable immune abnormalities.	J Allergy Clin Immunol.
atopic dermatitis	MALAT1	Expression profiling by array, count, 3 development stage, 2 disease state, 11 individual sets	GPL570	GSE32924 Samples	Nonlesional atopic dermatitis skin is characterized by broad terminal differentiation defects and variable immune abnormalities.	J Allergy Clin Immunol.
atopic dermatitis	GALNT1	Expression profiling by array, count, 3 development stage, 2 disease state, 11 individual sets	GPL570	GSE32924 Samples	Nonlesional atopic dermatitis skin is characterized by broad terminal differentiation defects and variable immune abnormalities.	J Allergy Clin Immunol.
atopic dermatitis	PARD3	Expression profiling by array, count, 3 development stage, 2 disease state, 11 individual sets	GPL570	GSE32924 Samples	Nonlesional atopic dermatitis skin is characterized by broad terminal differentiation defects and variable immune abnormalities.	J Allergy Clin Immunol.
atopic dermatitis	GALNT1	Expression profiling by array, count, 3 development stage, 2 disease state, 11 individual sets	GPL570	GSE32924 Samples	Nonlesional atopic dermatitis skin is characterized by broad terminal differentiation defects and variable immune abnormalities.	J Allergy Clin Immunol.
atopic dermatitis	HADHA	Expression profiling by array, count, 3 development stage, 2 disease state, 11 individual sets	GPL570	GSE32924 Samples	Nonlesional atopic dermatitis skin is characterized by broad terminal differentiation defects and variable immune abnormalities.	J Allergy Clin Immunol.
atopic dermatitis	TCF4	Expression profiling by array, count, 3 development stage, 2 disease state, 11 individual sets	GPL570	GSE32924 Samples	Nonlesional atopic dermatitis skin is characterized by broad terminal differentiation defects and variable immune abnormalities.	J Allergy Clin Immunol.
atopic dermatitis	RAPGEF4	Expression profiling by array, count, 3 development stage, 2 disease state, 11 individual sets	GPL570	GSE32924 Samples	Nonlesional atopic dermatitis skin is characterized by broad terminal differentiation defects and variable immune abnormalities.	J Allergy Clin Immunol.
atopic dermatitis	ALDH3A2	Expression profiling by array, count, 3 development stage, 2 disease state, 11 individual sets	GPL570	GSE32924 Samples	Nonlesional atopic dermatitis skin is characterized by broad terminal differentiation defects and variable immune abnormalities.	J Allergy Clin Immunol.
atopic dermatitis	NUCKS1	Expression profiling by array, count, 3 development stage, 2 disease state, 11 individual sets	GPL570	GSE32924 Samples	Nonlesional atopic dermatitis skin is characterized by broad terminal differentiation defects and variable immune abnormalities.	J Allergy Clin Immunol.
atopic dermatitis	KLF6	Expression profiling by array, count, 3 development stage, 2 disease state, 11 individual sets	GPL570	GSE32924 Samples	Nonlesional atopic dermatitis skin is characterized by broad terminal differentiation defects and variable immune abnormalities.	J Allergy Clin Immunol.
atopic dermatitis	COLEC12	Expression profiling by array, count, 3 development stage, 2 disease state, 11 individual sets	GPL570	GSE32924 Samples	Nonlesional atopic dermatitis skin is characterized by broad terminal differentiation defects and variable immune abnormalities.	J Allergy Clin Immunol.
atopic dermatitis	OLFML1	Expression profiling by array, count, 3 development stage, 2 disease state, 11 individual sets	GPL570	GSE32924 Samples	Nonlesional atopic dermatitis skin is characterized by broad terminal differentiation defects and variable immune abnormalities.	J Allergy Clin Immunol.
atopic dermatitis	RNASE4	Expression profiling by array, count, 3 development stage, 2 disease state, 11 individual sets	GPL570	GSE32924 Samples	Nonlesional atopic dermatitis skin is characterized by broad terminal differentiation defects and variable immune abnormalities.	J Allergy Clin Immunol.
atopic dermatitis	FBLN5	Expression profiling by array, count, 3 development stage, 2 disease state, 11 individual sets	GPL570	GSE32924 Samples	Nonlesional atopic dermatitis skin is characterized by broad terminal differentiation defects and variable immune abnormalities.	J Allergy Clin Immunol.
atopic dermatitis	CRTAP	Expression profiling by array, count, 3 development stage, 2 disease state, 11 individual sets	GPL570	GSE32924 Samples	Nonlesional atopic dermatitis skin is characterized by broad terminal differentiation defects and variable immune abnormalities.	J Allergy Clin Immunol.
atopic dermatitis	LPAR1	Expression profiling by array, count, 3 development stage, 2 disease state, 11 individual sets	GPL570	GSE32924 Samples	Nonlesional atopic dermatitis skin is characterized by broad terminal differentiation defects and variable immune abnormalities.	J Allergy Clin Immunol.
atopic dermatitis	PSMD10	Expression profiling by array, count, 3 development stage, 2 disease state, 11 individual sets	GPL570	GSE32924 Samples	Nonlesional atopic dermatitis skin is characterized by broad terminal differentiation defects and variable immune abnormalities.	J Allergy Clin Immunol.
atopic dermatitis	FBXO8	Expression profiling by array, count, 3 development stage, 2 disease state, 11 individual sets	GPL570	GSE32924 Samples	Nonlesional atopic dermatitis skin is characterized by broad terminal differentiation defects and variable immune abnormalities.	J Allergy Clin Immunol.
atopic dermatitis	SSBP2	Expression profiling by array, count, 3 development stage, 2 disease state, 11 individual sets	GPL570	GSE32924 Samples	Nonlesional atopic dermatitis skin is characterized by broad terminal differentiation defects and variable immune abnormalities.	J Allergy Clin Immunol.
atopic dermatitis	NCK1	Expression profiling by array, count, 3 development stage, 2 disease state, 11 individual sets	GPL570	GSE32924 Samples	Nonlesional atopic dermatitis skin is characterized by broad terminal differentiation defects and variable immune abnormalities.	J Allergy Clin Immunol.
atopic dermatitis	TMEM50B	Expression profiling by array, count, 3 development stage, 2 disease state, 11 individual sets	GPL570	GSE32924 Samples	Nonlesional atopic dermatitis skin is characterized by broad terminal differentiation defects and variable immune abnormalities.	J Allergy Clin Immunol.
atopic dermatitis	WDR26	Expression profiling by array, count, 3 development stage, 2 disease state, 11 individual sets	GPL570	GSE32924 Samples	Nonlesional atopic dermatitis skin is characterized by broad terminal differentiation defects and variable immune abnormalities.	J Allergy Clin Immunol.
atopic dermatitis	MAPRE2	Expression profiling by array, count, 3 development stage, 2 disease state, 11 individual sets	GPL570	GSE32924 Samples	Nonlesional atopic dermatitis skin is characterized by broad terminal differentiation defects and variable immune abnormalities.	J Allergy Clin Immunol.
atopic dermatitis	ZFYVE21	Expression profiling by array, count, 3 development stage, 2 disease state, 11 individual sets	GPL570	GSE32924 Samples	Nonlesional atopic dermatitis skin is characterized by broad terminal differentiation defects and variable immune abnormalities.	J Allergy Clin Immunol.
atopic dermatitis	RPRD1A	Expression profiling by array, count, 3 development stage, 2 disease state, 11 individual sets	GPL570	GSE32924 Samples	Nonlesional atopic dermatitis skin is characterized by broad terminal differentiation defects and variable immune abnormalities.	J Allergy Clin Immunol.
atopic dermatitis	38969	Expression profiling by array, count, 3 development stage, 2 disease state, 11 individual sets	GPL570	GSE32924 Samples	Nonlesional atopic dermatitis skin is characterized by broad terminal differentiation defects and variable immune abnormalities.	J Allergy Clin Immunol.

atopic dermatitis	PTPN11	Expression profiling by array, count, 3 development stage, 2 disease state, 11 individual sets	GPL570	GSE32924 Samples	Nonlesional atopic dermatitis skin is characterized by broad terminal differentiation defects and variable immune abnormalities.	J Allergy Clin Immunol.
atopic dermatitis	PEA15	Expression profiling by array, count, 3 development stage, 2 disease state, 11 individual sets	GPL570	GSE32924 Samples	Nonlesional atopic dermatitis skin is characterized by broad terminal differentiation defects and variable immune abnormalities.	J Allergy Clin Immunol.
atopic dermatitis	ROCK1	Expression profiling by array, count, 3 development stage, 2 disease state, 11 individual sets	GPL570	GSE32924 Samples	Nonlesional atopic dermatitis skin is characterized by broad terminal differentiation defects and variable immune abnormalities.	J Allergy Clin Immunol.
atopic dermatitis	PIGY	Expression profiling by array, count, 3 development stage, 2 disease state, 11 individual sets	GPL570	GSE32924 Samples	Nonlesional atopic dermatitis skin is characterized by broad terminal differentiation defects and variable immune abnormalities.	J Allergy Clin Immunol.
atopic dermatitis	C20ORF108	Expression profiling by array, count, 3 development stage, 2 disease state, 11 individual sets	GPL570	GSE32924 Samples	Nonlesional atopic dermatitis skin is characterized by broad terminal differentiation defects and variable immune abnormalities.	J Allergy Clin Immunol.
atopic dermatitis	38961	Expression profiling by array, count, 3 development stage, 2 disease state, 11 individual sets	GPL570	GSE32924 Samples	Nonlesional atopic dermatitis skin is characterized by broad terminal differentiation defects and variable immune abnormalities.	J Allergy Clin Immunol.
atopic dermatitis	SLC11A2	Expression profiling by array, count, 3 development stage, 2 disease state, 11 individual sets	GPL570	GSE32924 Samples	Nonlesional atopic dermatitis skin is characterized by broad terminal differentiation defects and variable immune abnormalities.	J Allergy Clin Immunol.
atopic dermatitis	ERMP1	Expression profiling by array, count, 3 development stage, 2 disease state, 11 individual sets	GPL570	GSE32924 Samples	Nonlesional atopic dermatitis skin is characterized by broad terminal differentiation defects and variable immune abnormalities.	J Allergy Clin Immunol.
atopic dermatitis	SCCPDH	Expression profiling by array, count, 3 development stage, 2 disease state, 11 individual sets	GPL570	GSE32924 Samples	Nonlesional atopic dermatitis skin is characterized by broad terminal differentiation defects and variable immune abnormalities.	J Allergy Clin Immunol.
atopic dermatitis	CDV3	Expression profiling by array, count, 3 development stage, 2 disease state, 11 individual sets	GPL570	GSE32924 Samples	Nonlesional atopic dermatitis skin is characterized by broad terminal differentiation defects and variable immune abnormalities.	J Allergy Clin Immunol.
atopic dermatitis	SETD8	Expression profiling by array, count, 3 development stage, 2 disease state, 11 individual sets	GPL570	GSE32924 Samples	Nonlesional atopic dermatitis skin is characterized by broad terminal differentiation defects and variable immune abnormalities.	J Allergy Clin Immunol.
atopic dermatitis	TPM4	Expression profiling by array, count, 3 development stage, 2 disease state, 11 individual sets	GPL570	GSE32924 Samples	Nonlesional atopic dermatitis skin is characterized by broad terminal differentiation defects and variable immune abnormalities.	J Allergy Clin Immunol.
atopic dermatitis	NA	Expression profiling by array, count, 3 development stage, 2 disease state, 11 individual sets	GPL570	GSE32924 Samples	Nonlesional atopic dermatitis skin is characterized by broad terminal differentiation defects and variable immune abnormalities.	J Allergy Clin Immunol.
atopic dermatitis	CDYL	Expression profiling by array, count, 3 development stage, 2 disease state, 11 individual sets	GPL570	GSE32924 Samples	Nonlesional atopic dermatitis skin is characterized by broad terminal differentiation defects and variable immune abnormalities.	J Allergy Clin Immunol.
atopic dermatitis	C19ORF2	Expression profiling by array, count, 3 development stage, 2 disease state, 11 individual sets	GPL570	GSE32924 Samples	Nonlesional atopic dermatitis skin is characterized by broad terminal differentiation defects and variable immune abnormalities.	J Allergy Clin Immunol.
atopic dermatitis	CLIP1	Expression profiling by array, count, 3 development stage, 2 disease state, 11 individual sets	GPL570	GSE32924 Samples	Nonlesional atopic dermatitis skin is characterized by broad terminal differentiation defects and variable immune abnormalities.	J Allergy Clin Immunol.
atopic dermatitis	RANBP9	Expression profiling by array, count, 3 development stage, 2 disease state, 11 individual sets	GPL570	GSE32924 Samples	Nonlesional atopic dermatitis skin is characterized by broad terminal differentiation defects and variable immune abnormalities.	J Allergy Clin Immunol.
atopic dermatitis	NRBF2	Expression profiling by array, count, 3 development stage, 2 disease state, 11 individual sets	GPL570	GSE32924 Samples	Nonlesional atopic dermatitis skin is characterized by broad terminal differentiation defects and variable immune abnormalities.	J Allergy Clin Immunol.
atopic dermatitis	LYPLA1	Expression profiling by array, count, 3 development stage, 2 disease state, 11 individual sets	GPL570	GSE32924 Samples	Nonlesional atopic dermatitis skin is characterized by broad terminal differentiation defects and variable immune abnormalities.	J Allergy Clin Immunol.
atopic dermatitis	JAK1	Expression profiling by array, count, 3 development stage, 2 disease state, 11 individual sets	GPL570	GSE32924 Samples	Nonlesional atopic dermatitis skin is characterized by broad terminal differentiation defects and variable immune abnormalities.	J Allergy Clin Immunol.
atopic dermatitis	CITED2	Expression profiling by array, count, 3 development stage, 2 disease state, 11 individual sets	GPL570	GSE32924 Samples	Nonlesional atopic dermatitis skin is characterized by broad terminal differentiation defects and variable immune abnormalities.	J Allergy Clin Immunol.
atopic dermatitis	PSAT1	Expression profiling by array, count, 3 development stage, 2 disease state, 11 individual sets	GPL570	GSE32924 Samples	Nonlesional atopic dermatitis skin is characterized by broad terminal differentiation defects and variable immune abnormalities.	J Allergy Clin Immunol.
atopic dermatitis	CLDN1	Expression profiling by array, count, 3 development stage, 2 disease state, 11 individual sets	GPL570	GSE32924 Samples	Nonlesional atopic dermatitis skin is characterized by broad terminal differentiation defects and variable immune abnormalities.	J Allergy Clin Immunol.
atopic dermatitis	KLF4	Expression profiling by array, count, 3 development stage, 2 disease state, 11 individual sets	GPL570	GSE32924 Samples	Nonlesional atopic dermatitis skin is characterized by broad terminal differentiation defects and variable immune abnormalities.	J Allergy Clin Immunol.
atopic dermatitis	C10ORF57	Expression profiling by array, count, 3 development stage, 2 disease state, 11 individual sets	GPL570	GSE32924 Samples	Nonlesional atopic dermatitis skin is characterized by broad terminal differentiation defects and variable immune abnormalities.	J Allergy Clin Immunol.
atopic dermatitis	GLRX	Expression profiling by array, count, 3 development stage, 2 disease state, 11 individual sets	GPL570	GSE32924 Samples	Nonlesional atopic dermatitis skin is characterized by broad terminal differentiation defects and variable immune abnormalities.	J Allergy Clin Immunol.
atopic dermatitis	SYNE1	Expression profiling by array, count, 3 development stage, 2 disease state, 11 individual sets	GPL570	GSE32924 Samples	Nonlesional atopic dermatitis skin is characterized by broad terminal differentiation defects and variable immune abnormalities.	J Allergy Clin Immunol.
atopic dermatitis	CMAH	Expression profiling by array, count, 3 development stage, 2 disease state, 11 individual sets	GPL570	GSE32924 Samples	Nonlesional atopic dermatitis skin is characterized by broad terminal differentiation defects and variable immune abnormalities.	J Allergy Clin Immunol.
atopic dermatitis	SGEF	Expression profiling by array, count, 3 development stage, 2 disease state, 11 individual sets	GPL570	GSE32924 Samples	Nonlesional atopic dermatitis skin is characterized by broad terminal differentiation defects and variable immune abnormalities.	J Allergy Clin Immunol.
atopic dermatitis	NA	Expression profiling by array, count, 3 development stage, 2 disease state, 11 individual sets	GPL570	GSE32924 Samples	Nonlesional atopic dermatitis skin is characterized by broad terminal differentiation defects and variable immune abnormalities.	J Allergy Clin Immunol.
atopic dermatitis	PLP1	Expression profiling by array, count, 3 development stage, 2 disease state, 11 individual sets	GPL570	GSE32924 Samples	Nonlesional atopic dermatitis skin is characterized by broad terminal differentiation defects and variable immune abnormalities.	J Allergy Clin Immunol.
atopic dermatitis	RAB11FIP1	Expression profiling by array, count, 3 development stage, 2 disease state, 11 individual sets	GPL570	GSE32924 Samples	Nonlesional atopic dermatitis skin is characterized by broad terminal differentiation defects and variable immune abnormalities.	J Allergy Clin Immunol.
atopic dermatitis	GTF2F2	Expression profiling by array, count, 3 development stage, 2 disease state, 11 individual sets	GPL570	GSE32924 Samples	Nonlesional atopic dermatitis skin is characterized by broad terminal differentiation defects and variable immune abnormalities.	J Allergy Clin Immunol.
atopic dermatitis	NA	Expression profiling by array, count, 3 development stage, 2 disease state, 11 individual sets	GPL570	GSE32924 Samples	Nonlesional atopic dermatitis skin is characterized by broad terminal differentiation defects and variable immune abnormalities.	J Allergy Clin Immunol.
atopic dermatitis	LOC100049716	Expression profiling by array, count, 3 development stage, 2 disease state, 11 individual sets	GPL570	GSE32924 Samples	Nonlesional atopic dermatitis skin is characterized by broad terminal differentiation defects and variable immune abnormalities.	J Allergy Clin Immunol.
atopic dermatitis	TMEM99	Expression profiling by array, count, 3 development stage, 2 disease state, 11 individual sets	GPL570	GSE32924 Samples	Nonlesional atopic dermatitis skin is characterized by broad terminal differentiation defects and variable immune abnormalities.	J Allergy Clin Immunol.
atopic dermatitis	CYP39A1	Expression profiling by array, count, 3 development stage, 2 disease state, 11 individual sets	GPL570	GSE32924 Samples	Nonlesional atopic dermatitis skin is characterized by broad terminal differentiation defects and variable immune abnormalities.	J Allergy Clin Immunol.
atopic dermatitis	TRAK1	Expression profiling by array, count, 3 development stage, 2 disease state, 11 individual sets	GPL570	GSE32924 Samples	Nonlesional atopic dermatitis skin is characterized by broad terminal differentiation defects and variable immune abnormalities.	J Allergy Clin Immunol.
atopic dermatitis	MOSPD2	Expression profiling by array, count, 3 development stage, 2 disease state, 11 individual sets	GPL570	GSE32924 Samples	Nonlesional atopic dermatitis skin is characterized by broad terminal differentiation defects and variable immune abnormalities.	J Allergy Clin Immunol.
atopic dermatitis	FAM171B	Expression profiling by array, count, 3 development stage, 2 disease state, 11 individual sets	GPL570	GSE32924 Samples	Nonlesional atopic dermatitis skin is characterized by broad terminal differentiation defects and variable immune abnormalities.	J Allergy Clin Immunol.
atopic dermatitis	HLF	Expression profiling by array, count, 3 development stage, 2 disease state, 11 individual sets	GPL570	GSE32924 Samples	Nonlesional atopic dermatitis skin is characterized by broad terminal differentiation defects and variable immune abnormalities.	J Allergy Clin Immunol.

atopic dermatitis	KDM5C	Expression profiling by array, count, 3 development stage, 2 disease state, 11 individual sets	GPL570	GSE32924 Samples	Nonlesional atopic dermatitis skin is characterized by broad terminal differentiation defects and variable immune abnormalities.	J Allergy Clin Immunol.
atopic dermatitis	SGEF	Expression profiling by array, count, 3 development stage, 2 disease state, 11 individual sets	GPL570	GSE32924 Samples	Nonlesional atopic dermatitis skin is characterized by broad terminal differentiation defects and variable immune abnormalities.	J Allergy Clin Immunol.
atopic dermatitis	NPIPL1	Expression profiling by array, count, 3 development stage, 2 disease state, 11 individual sets	GPL570	GSE32924 Samples	Nonlesional atopic dermatitis skin is characterized by broad terminal differentiation defects and variable immune abnormalities.	J Allergy Clin Immunol.
atopic dermatitis	UST	Expression profiling by array, count, 3 development stage, 2 disease state, 11 individual sets	GPL570	GSE32924 Samples	Nonlesional atopic dermatitis skin is characterized by broad terminal differentiation defects and variable immune abnormalities.	J Allergy Clin Immunol.
atopic dermatitis	TAF15	Expression profiling by array, count, 3 development stage, 2 disease state, 11 individual sets	GPL570	GSE32924 Samples	Nonlesional atopic dermatitis skin is characterized by broad terminal differentiation defects and variable immune abnormalities.	J Allergy Clin Immunol.
atopic dermatitis	ATP2B1	Expression profiling by array, count, 3 development stage, 2 disease state, 11 individual sets	GPL570	GSE32924 Samples	Nonlesional atopic dermatitis skin is characterized by broad terminal differentiation defects and variable immune abnormalities.	J Allergy Clin Immunol.
atopic dermatitis	FGFR2	Expression profiling by array, count, 3 development stage, 2 disease state, 11 individual sets	GPL570	GSE32924 Samples	Nonlesional atopic dermatitis skin is characterized by broad terminal differentiation defects and variable immune abnormalities.	J Allergy Clin Immunol.
atopic dermatitis	CNTN1	Expression profiling by array, count, 3 development stage, 2 disease state, 11 individual sets	GPL570	GSE32924 Samples	Nonlesional atopic dermatitis skin is characterized by broad terminal differentiation defects and variable immune abnormalities.	J Allergy Clin Immunol.
atopic dermatitis	38778	Expression profiling by array, count, 3 development stage, 2 disease state, 11 individual sets	GPL570	GSE32924 Samples	Nonlesional atopic dermatitis skin is characterized by broad terminal differentiation defects and variable immune abnormalities.	J Allergy Clin Immunol.
atopic dermatitis	SPTLC3	Expression profiling by array, count, 3 development stage, 2 disease state, 11 individual sets	GPL570	GSE32924 Samples	Nonlesional atopic dermatitis skin is characterized by broad terminal differentiation defects and variable immune abnormalities.	J Allergy Clin Immunol.
atopic dermatitis	RORA	Expression profiling by array, count, 3 development stage, 2 disease state, 11 individual sets	GPL570	GSE32924 Samples	Nonlesional atopic dermatitis skin is characterized by broad terminal differentiation defects and variable immune abnormalities.	J Allergy Clin Immunol.
atopic dermatitis	SPAG1	Expression profiling by array, count, 3 development stage, 2 disease state, 11 individual sets	GPL570	GSE32924 Samples	Nonlesional atopic dermatitis skin is characterized by broad terminal differentiation defects and variable immune abnormalities.	J Allergy Clin Immunol.
atopic dermatitis	ABHD5	Expression profiling by array, count, 3 development stage, 2 disease state, 11 individual sets	GPL570	GSE32924 Samples	Nonlesional atopic dermatitis skin is characterized by broad terminal differentiation defects and variable immune abnormalities.	J Allergy Clin Immunol.
atopic dermatitis	CHP2	Expression profiling by array, count, 3 development stage, 2 disease state, 11 individual sets	GPL570	GSE32924 Samples	Nonlesional atopic dermatitis skin is characterized by broad terminal differentiation defects and variable immune abnormalities.	J Allergy Clin Immunol.
atopic dermatitis	NA	Expression profiling by array, count, 3 development stage, 2 disease state, 11 individual sets	GPL570	GSE32924 Samples	Nonlesional atopic dermatitis skin is characterized by broad terminal differentiation defects and variable immune abnormalities.	J Allergy Clin Immunol.
atopic dermatitis	CLINT1	Expression profiling by array, count, 3 development stage, 2 disease state, 11 individual sets	GPL570	GSE32924 Samples	Nonlesional atopic dermatitis skin is characterized by broad terminal differentiation defects and variable immune abnormalities.	J Allergy Clin Immunol.
atopic dermatitis	ZRANB1	Expression profiling by array, count, 3 development stage, 2 disease state, 11 individual sets	GPL570	GSE32924 Samples	Nonlesional atopic dermatitis skin is characterized by broad terminal differentiation defects and variable immune abnormalities.	J Allergy Clin Immunol.
atopic dermatitis	USP38	Expression profiling by array, count, 3 development stage, 2 disease state, 11 individual sets	GPL570	GSE32924 Samples	Nonlesional atopic dermatitis skin is characterized by broad terminal differentiation defects and variable immune abnormalities.	J Allergy Clin Immunol.
atopic dermatitis	SIAH2	Expression profiling by array, count, 3 development stage, 2 disease state, 11 individual sets	GPL570	GSE32924 Samples	Nonlesional atopic dermatitis skin is characterized by broad terminal differentiation defects and variable immune abnormalities.	J Allergy Clin Immunol.
atopic dermatitis	USP46	Expression profiling by array, count, 3 development stage, 2 disease state, 11 individual sets	GPL570	GSE32924 Samples	Nonlesional atopic dermatitis skin is characterized by broad terminal differentiation defects and variable immune abnormalities.	J Allergy Clin Immunol.
atopic dermatitis	TPM4	Expression profiling by array, count, 3 development stage, 2 disease state, 11 individual sets	GPL570	GSE32924 Samples	Nonlesional atopic dermatitis skin is characterized by broad terminal differentiation defects and variable immune abnormalities.	J Allergy Clin Immunol.
atopic dermatitis	TPM4	Expression profiling by array, count, 3 development stage, 2 disease state, 11 individual sets	GPL570	GSE32924 Samples	Nonlesional atopic dermatitis skin is characterized by broad terminal differentiation defects and variable immune abnormalities.	J Allergy Clin Immunol.
atopic dermatitis	MTFR1	Expression profiling by array, count, 3 development stage, 2 disease state, 11 individual sets	GPL570	GSE32924 Samples	Nonlesional atopic dermatitis skin is characterized by broad terminal differentiation defects and variable immune abnormalities.	J Allergy Clin Immunol.
atopic dermatitis	MAP7	Expression profiling by array, count, 3 development stage, 2 disease state, 11 individual sets	GPL570	GSE32924 Samples	Nonlesional atopic dermatitis skin is characterized by broad terminal differentiation defects and variable immune abnormalities.	J Allergy Clin Immunol.
atopic dermatitis	XRN2	Expression profiling by array, count, 3 development stage, 2 disease state, 11 individual sets	GPL570	GSE32924 Samples	Nonlesional atopic dermatitis skin is characterized by broad terminal differentiation defects and variable immune abnormalities.	J Allergy Clin Immunol.
atopic dermatitis	FYTTD1	Expression profiling by array, count, 3 development stage, 2 disease state, 11 individual sets	GPL570	GSE32924 Samples	Nonlesional atopic dermatitis skin is characterized by broad terminal differentiation defects and variable immune abnormalities.	J Allergy Clin Immunol.
atopic dermatitis	TMEM9B	Expression profiling by array, count, 3 development stage, 2 disease state, 11 individual sets	GPL570	GSE32924 Samples	Nonlesional atopic dermatitis skin is characterized by broad terminal differentiation defects and variable immune abnormalities.	J Allergy Clin Immunol.
atopic dermatitis	KLF3	Expression profiling by array, count, 3 development stage, 2 disease state, 11 individual sets	GPL570	GSE32924 Samples	Nonlesional atopic dermatitis skin is characterized by broad terminal differentiation defects and variable immune abnormalities.	J Allergy Clin Immunol.
atopic dermatitis	AGA	Expression profiling by array, count, 3 development stage, 2 disease state, 11 individual sets	GPL570	GSE32924 Samples	Nonlesional atopic dermatitis skin is characterized by broad terminal differentiation defects and variable immune abnormalities.	J Allergy Clin Immunol.
atopic dermatitis	SCAMP1	Expression profiling by array, count, 3 development stage, 2 disease state, 11 individual sets	GPL570	GSE32924 Samples	Nonlesional atopic dermatitis skin is characterized by broad terminal differentiation defects and variable immune abnormalities.	J Allergy Clin Immunol.
atopic dermatitis	HIST2H2BE	Expression profiling by array, count, 3 development stage, 2 disease state, 11 individual sets	GPL570	GSE32924 Samples	Nonlesional atopic dermatitis skin is characterized by broad terminal differentiation defects and variable immune abnormalities.	J Allergy Clin Immunol.
atopic dermatitis	PNRC2	Expression profiling by array, count, 3 development stage, 2 disease state, 11 individual sets	GPL570	GSE32924 Samples	Nonlesional atopic dermatitis skin is characterized by broad terminal differentiation defects and variable immune abnormalities.	J Allergy Clin Immunol.
atopic dermatitis	CAPN7	Expression profiling by array, count, 3 development stage, 2 disease state, 11 individual sets	GPL570	GSE32924 Samples	Nonlesional atopic dermatitis skin is characterized by broad terminal differentiation defects and variable immune abnormalities.	J Allergy Clin Immunol.
atopic dermatitis	AKTIP	Expression profiling by array, count, 3 development stage, 2 disease state, 11 individual sets	GPL570	GSE32924 Samples	Nonlesional atopic dermatitis skin is characterized by broad terminal differentiation defects and variable immune abnormalities.	J Allergy Clin Immunol.
atopic dermatitis	GOLGA7	Expression profiling by array, count, 3 development stage, 2 disease state, 11 individual sets	GPL570	GSE32924 Samples	Nonlesional atopic dermatitis skin is characterized by broad terminal differentiation defects and variable immune abnormalities.	J Allergy Clin Immunol.
atopic dermatitis	ZNF277	Expression profiling by array, count, 3 development stage, 2 disease state, 11 individual sets	GPL570	GSE32924 Samples	Nonlesional atopic dermatitis skin is characterized by broad terminal differentiation defects and variable immune abnormalities.	J Allergy Clin Immunol.
atopic dermatitis	RDX	Expression profiling by array, count, 3 development stage, 2 disease state, 11 individual sets	GPL570	GSE32924 Samples	Nonlesional atopic dermatitis skin is characterized by broad terminal differentiation defects and variable immune abnormalities.	J Allergy Clin Immunol.
atopic dermatitis	APPL1	Expression profiling by array, count, 3 development stage, 2 disease state, 11 individual sets	GPL570	GSE32924 Samples	Nonlesional atopic dermatitis skin is characterized by broad terminal differentiation defects and variable immune abnormalities.	J Allergy Clin Immunol.
atopic dermatitis	RIT1	Expression profiling by array, count, 3 development stage, 2 disease state, 11 individual sets	GPL570	GSE32924 Samples	Nonlesional atopic dermatitis skin is characterized by broad terminal differentiation defects and variable immune abnormalities.	J Allergy Clin Immunol.
atopic dermatitis	FXR1	Expression profiling by array, count, 3 development stage, 2 disease state, 11 individual sets	GPL570	GSE32924 Samples	Nonlesional atopic dermatitis skin is characterized by broad terminal differentiation defects and variable immune abnormalities.	J Allergy Clin Immunol.
atopic dermatitis	CCPG1	Expression profiling by array, count, 3 development stage, 2 disease state, 11 individual sets	GPL570	GSE32924 Samples	Nonlesional atopic dermatitis skin is characterized by broad terminal differentiation defects and variable immune abnormalities.	J Allergy Clin Immunol.

atopic dermatitis	DNAJB9	Expression profiling by array, count, 3 development stage, 2 disease state, 11 individual sets	GPL570	GSE32924 Samples	Nonlesional atopic dermatitis skin is characterized by broad terminal differentiation defects and variable immune abnormalities.	J Allergy Clin Immunol.
atopic dermatitis	TLE1	Expression profiling by array, count, 3 development stage, 2 disease state, 11 individual sets	GPL570	GSE32924 Samples	Nonlesional atopic dermatitis skin is characterized by broad terminal differentiation defects and variable immune abnormalities.	J Allergy Clin Immunol.
atopic dermatitis	SPIRE1	Expression profiling by array, count, 3 development stage, 2 disease state, 11 individual sets	GPL570	GSE32924 Samples	Nonlesional atopic dermatitis skin is characterized by broad terminal differentiation defects and variable immune abnormalities.	J Allergy Clin Immunol.
atopic dermatitis	NA	Expression profiling by array, count, 3 development stage, 2 disease state, 11 individual sets	GPL570	GSE32924 Samples	Nonlesional atopic dermatitis skin is characterized by broad terminal differentiation defects and variable immune abnormalities.	J Allergy Clin Immunol.
atopic dermatitis	TMEM43	Expression profiling by array, count, 3 development stage, 2 disease state, 11 individual sets	GPL570	GSE32924 Samples	Nonlesional atopic dermatitis skin is characterized by broad terminal differentiation defects and variable immune abnormalities.	J Allergy Clin Immunol.
atopic dermatitis	PICALM	Expression profiling by array, count, 3 development stage, 2 disease state, 11 individual sets	GPL570	GSE32924 Samples	Nonlesional atopic dermatitis skin is characterized by broad terminal differentiation defects and variable immune abnormalities.	J Allergy Clin Immunol.
atopic dermatitis	BCL6	Expression profiling by array, count, 3 development stage, 2 disease state, 11 individual sets	GPL570	GSE32924 Samples	Nonlesional atopic dermatitis skin is characterized by broad terminal differentiation defects and variable immune abnormalities.	J Allergy Clin Immunol.
atopic dermatitis	ALOXE3	Expression profiling by array, count, 3 development stage, 2 disease state, 11 individual sets	GPL570	GSE32924 Samples	Nonlesional atopic dermatitis skin is characterized by broad terminal differentiation defects and variable immune abnormalities.	J Allergy Clin Immunol.
atopic dermatitis	ATP2C2	Expression profiling by array, count, 3 development stage, 2 disease state, 11 individual sets	GPL570	GSE32924 Samples	Nonlesional atopic dermatitis skin is characterized by broad terminal differentiation defects and variable immune abnormalities.	J Allergy Clin Immunol.
atopic dermatitis	C3ORF52	Expression profiling by array, count, 3 development stage, 2 disease state, 11 individual sets	GPL570	GSE32924 Samples	Nonlesional atopic dermatitis skin is characterized by broad terminal differentiation defects and variable immune abnormalities.	J Allergy Clin Immunol.
atopic dermatitis	USP34	Expression profiling by array, count, 3 development stage, 2 disease state, 11 individual sets	GPL570	GSE32924 Samples	Nonlesional atopic dermatitis skin is characterized by broad terminal differentiation defects and variable immune abnormalities.	J Allergy Clin Immunol.
atopic dermatitis	OSBP	Expression profiling by array, count, 3 development stage, 2 disease state, 11 individual sets	GPL570	GSE32924 Samples	Nonlesional atopic dermatitis skin is characterized by broad terminal differentiation defects and variable immune abnormalities.	J Allergy Clin Immunol.
atopic dermatitis	DCTN4	Expression profiling by array, count, 3 development stage, 2 disease state, 11 individual sets	GPL570	GSE32924 Samples	Nonlesional atopic dermatitis skin is characterized by broad terminal differentiation defects and variable immune abnormalities.	J Allergy Clin Immunol.
atopic dermatitis	PSEN1	Expression profiling by array, count, 3 development stage, 2 disease state, 11 individual sets	GPL570	GSE32924 Samples	Nonlesional atopic dermatitis skin is characterized by broad terminal differentiation defects and variable immune abnormalities.	J Allergy Clin Immunol.
atopic dermatitis	APOOL	Expression profiling by array, count, 3 development stage, 2 disease state, 11 individual sets	GPL570	GSE32924 Samples	Nonlesional atopic dermatitis skin is characterized by broad terminal differentiation defects and variable immune abnormalities.	J Allergy Clin Immunol.
atopic dermatitis	EML4	Expression profiling by array, count, 3 development stage, 2 disease state, 11 individual sets	GPL570	GSE32924 Samples	Nonlesional atopic dermatitis skin is characterized by broad terminal differentiation defects and variable immune abnormalities.	J Allergy Clin Immunol.
atopic dermatitis	USP15	Expression profiling by array, count, 3 development stage, 2 disease state, 11 individual sets	GPL570	GSE32924 Samples	Nonlesional atopic dermatitis skin is characterized by broad terminal differentiation defects and variable immune abnormalities.	J Allergy Clin Immunol.
atopic dermatitis	BICD2	Expression profiling by array, count, 3 development stage, 2 disease state, 11 individual sets	GPL570	GSE32924 Samples	Nonlesional atopic dermatitis skin is characterized by broad terminal differentiation defects and variable immune abnormalities.	J Allergy Clin Immunol.
atopic dermatitis	AAGAB	Expression profiling by array, count, 3 development stage, 2 disease state, 11 individual sets	GPL570	GSE32924 Samples	Nonlesional atopic dermatitis skin is characterized by broad terminal differentiation defects and variable immune abnormalities.	J Allergy Clin Immunol.
atopic dermatitis	SRGAP2P1	Expression profiling by array, count, 3 development stage, 2 disease state, 11 individual sets	GPL570	GSE32924 Samples	Nonlesional atopic dermatitis skin is characterized by broad terminal differentiation defects and variable immune abnormalities.	J Allergy Clin Immunol.
atopic dermatitis	LIMS1	Expression profiling by array, count, 3 development stage, 2 disease state, 11 individual sets	GPL570	GSE32924 Samples	Nonlesional atopic dermatitis skin is characterized by broad terminal differentiation defects and variable immune abnormalities.	J Allergy Clin Immunol.
atopic dermatitis	NAV3	Expression profiling by array, count, 3 development stage, 2 disease state, 11 individual sets	GPL570	GSE32924 Samples	Nonlesional atopic dermatitis skin is characterized by broad terminal differentiation defects and variable immune abnormalities.	J Allergy Clin Immunol.
atopic dermatitis	RAPGEF6	Expression profiling by array, count, 3 development stage, 2 disease state, 11 individual sets	GPL570	GSE32924 Samples	Nonlesional atopic dermatitis skin is characterized by broad terminal differentiation defects and variable immune abnormalities.	J Allergy Clin Immunol.
atopic dermatitis	TSPAN5	Expression profiling by array, count, 3 development stage, 2 disease state, 11 individual sets	GPL570	GSE32924 Samples	Nonlesional atopic dermatitis skin is characterized by broad terminal differentiation defects and variable immune abnormalities.	J Allergy Clin Immunol.
atopic dermatitis	ALOX12	Expression profiling by array, count, 3 development stage, 2 disease state, 11 individual sets	GPL570	GSE32924 Samples	Nonlesional atopic dermatitis skin is characterized by broad terminal differentiation defects and variable immune abnormalities.	J Allergy Clin Immunol.
atopic dermatitis	SLC1A6	Expression profiling by array, count, 3 development stage, 2 disease state, 11 individual sets	GPL570	GSE32924 Samples	Nonlesional atopic dermatitis skin is characterized by broad terminal differentiation defects and variable immune abnormalities.	J Allergy Clin Immunol.
atopic dermatitis	MYO5B	Expression profiling by array, count, 3 development stage, 2 disease state, 11 individual sets	GPL570	GSE32924 Samples	Nonlesional atopic dermatitis skin is characterized by broad terminal differentiation defects and variable immune abnormalities.	J Allergy Clin Immunol.
atopic dermatitis	PRNP	Expression profiling by array, count, 3 development stage, 2 disease state, 11 individual sets	GPL570	GSE32924 Samples	Nonlesional atopic dermatitis skin is characterized by broad terminal differentiation defects and variable immune abnormalities.	J Allergy Clin Immunol.
atopic dermatitis	PTPN11	Expression profiling by array, count, 3 development stage, 2 disease state, 11 individual sets	GPL570	GSE32924 Samples	Nonlesional atopic dermatitis skin is characterized by broad terminal differentiation defects and variable immune abnormalities.	J Allergy Clin Immunol.
atopic dermatitis	NA	Expression profiling by array, count, 3 development stage, 2 disease state, 11 individual sets	GPL570	GSE32924 Samples	Nonlesional atopic dermatitis skin is characterized by broad terminal differentiation defects and variable immune abnormalities.	J Allergy Clin Immunol.
atopic dermatitis	RAB5A	Expression profiling by array, count, 3 development stage, 2 disease state, 11 individual sets	GPL570	GSE32924 Samples	Nonlesional atopic dermatitis skin is characterized by broad terminal differentiation defects and variable immune abnormalities.	J Allergy Clin Immunol.
atopic dermatitis	NOTCH2	Expression profiling by array, count, 3 development stage, 2 disease state, 11 individual sets	GPL570	GSE32924 Samples	Nonlesional atopic dermatitis skin is characterized by broad terminal differentiation defects and variable immune abnormalities.	J Allergy Clin Immunol.
atopic dermatitis	PALMD	Expression profiling by array, count, 3 development stage, 2 disease state, 11 individual sets	GPL570	GSE32924 Samples	Nonlesional atopic dermatitis skin is characterized by broad terminal differentiation defects and variable immune abnormalities.	J Allergy Clin Immunol.
atopic dermatitis	IGFBP5	Expression profiling by array, count, 3 development stage, 2 disease state, 11 individual sets	GPL570	GSE32924 Samples	Nonlesional atopic dermatitis skin is characterized by broad terminal differentiation defects and variable immune abnormalities.	J Allergy Clin Immunol.
atopic dermatitis	LOC647979	Expression profiling by array, count, 3 development stage, 2 disease state, 11 individual sets	GPL570	GSE32924 Samples	Nonlesional atopic dermatitis skin is characterized by broad terminal differentiation defects and variable immune abnormalities.	J Allergy Clin Immunol.
atopic dermatitis	TMED2	Expression profiling by array, count, 3 development stage, 2 disease state, 11 individual sets	GPL570	GSE32924 Samples	Nonlesional atopic dermatitis skin is characterized by broad terminal differentiation defects and variable immune abnormalities.	J Allergy Clin Immunol.
atopic dermatitis	TMED2	Expression profiling by array, count, 3 development stage, 2 disease state, 11 individual sets	GPL570	GSE32924 Samples	Nonlesional atopic dermatitis skin is characterized by broad terminal differentiation defects and variable immune abnormalities.	J Allergy Clin Immunol.
atopic dermatitis	EZR	Expression profiling by array, count, 3 development stage, 2 disease state, 11 individual sets	GPL570	GSE32924 Samples	Nonlesional atopic dermatitis skin is characterized by broad terminal differentiation defects and variable immune abnormalities.	J Allergy Clin Immunol.
atopic dermatitis	RFWD2	Expression profiling by array, count, 3 development stage, 2 disease state, 11 individual sets	GPL570	GSE32924 Samples	Nonlesional atopic dermatitis skin is characterized by broad terminal differentiation defects and variable immune abnormalities.	J Allergy Clin Immunol.
atopic dermatitis	UBE2J1	Expression profiling by array, count, 3 development stage, 2 disease state, 11 individual sets	GPL570	GSE32924 Samples	Nonlesional atopic dermatitis skin is characterized by broad terminal differentiation defects and variable immune abnormalities.	J Allergy Clin Immunol.
atopic dermatitis	DCTD	Expression profiling by array, count, 3 development stage, 2 disease state, 11 individual sets	GPL570	GSE32924 Samples	Nonlesional atopic dermatitis skin is characterized by broad terminal differentiation defects and variable immune abnormalities.	J Allergy Clin Immunol.

atopic dermatitis	CSNK1A1	Expression profiling by array, count, 3 development stage, 2 disease state, 11 individual sets	GPL570	GSE32924 Samples	Nonlesional atopic dermatitis skin is characterized by broad terminal differentiation defects and variable immune abnormalities.	J Allergy Clin Immunol.
atopic dermatitis	UBXN4	Expression profiling by array, count, 3 development stage, 2 disease state, 11 individual sets	GPL570	GSE32924 Samples	Nonlesional atopic dermatitis skin is characterized by broad terminal differentiation defects and variable immune abnormalities.	J Allergy Clin Immunol.
atopic dermatitis	PRPF4B	Expression profiling by array, count, 3 development stage, 2 disease state, 11 individual sets	GPL570	GSE32924 Samples	Nonlesional atopic dermatitis skin is characterized by broad terminal differentiation defects and variable immune abnormalities.	J Allergy Clin Immunol.
atopic dermatitis	MED30	Expression profiling by array, count, 3 development stage, 2 disease state, 11 individual sets	GPL570	GSE32924 Samples	Nonlesional atopic dermatitis skin is characterized by broad terminal differentiation defects and variable immune abnormalities.	J Allergy Clin Immunol.
atopic dermatitis	PLEKHF2	Expression profiling by array, count, 3 development stage, 2 disease state, 11 individual sets	GPL570	GSE32924 Samples	Nonlesional atopic dermatitis skin is characterized by broad terminal differentiation defects and variable immune abnormalities.	J Allergy Clin Immunol.
atopic dermatitis	PAPOLA	Expression profiling by array, count, 3 development stage, 2 disease state, 11 individual sets	GPL570	GSE32924 Samples	Nonlesional atopic dermatitis skin is characterized by broad terminal differentiation defects and variable immune abnormalities.	J Allergy Clin Immunol.
atopic dermatitis	GPR177	Expression profiling by array, count, 3 development stage, 2 disease state, 11 individual sets	GPL570	GSE32924 Samples	Nonlesional atopic dermatitis skin is characterized by broad terminal differentiation defects and variable immune abnormalities.	J Allergy Clin Immunol.
atopic dermatitis	MED6	Expression profiling by array, count, 3 development stage, 2 disease state, 11 individual sets	GPL570	GSE32924 Samples	Nonlesional atopic dermatitis skin is characterized by broad terminal differentiation defects and variable immune abnormalities.	J Allergy Clin Immunol.
atopic dermatitis	WBP11	Expression profiling by array, count, 3 development stage, 2 disease state, 11 individual sets	GPL570	GSE32924 Samples	Nonlesional atopic dermatitis skin is characterized by broad terminal differentiation defects and variable immune abnormalities.	J Allergy Clin Immunol.
atopic dermatitis	PGCP	Expression profiling by array, count, 3 development stage, 2 disease state, 11 individual sets	GPL570	GSE32924 Samples	Nonlesional atopic dermatitis skin is characterized by broad terminal differentiation defects and variable immune abnormalities.	J Allergy Clin Immunol.
atopic dermatitis	C10ORF18	Expression profiling by array, count, 3 development stage, 2 disease state, 11 individual sets	GPL570	GSE32924 Samples	Nonlesional atopic dermatitis skin is characterized by broad terminal differentiation defects and variable immune abnormalities.	J Allergy Clin Immunol.
atopic dermatitis	BMP2	Expression profiling by array, count, 3 development stage, 2 disease state, 11 individual sets	GPL570	GSE32924 Samples	Nonlesional atopic dermatitis skin is characterized by broad terminal differentiation defects and variable immune abnormalities.	J Allergy Clin Immunol.
atopic dermatitis	CTNND1	Expression profiling by array, count, 3 development stage, 2 disease state, 11 individual sets	GPL570	GSE32924 Samples	Nonlesional atopic dermatitis skin is characterized by broad terminal differentiation defects and variable immune abnormalities.	J Allergy Clin Immunol.
atopic dermatitis	EPB41L3	Expression profiling by array, count, 3 development stage, 2 disease state, 11 individual sets	GPL570	GSE32924 Samples	Nonlesional atopic dermatitis skin is characterized by broad terminal differentiation defects and variable immune abnormalities.	J Allergy Clin Immunol.
atopic dermatitis	FYN	Expression profiling by array, count, 3 development stage, 2 disease state, 11 individual sets	GPL570	GSE32924 Samples	Nonlesional atopic dermatitis skin is characterized by broad terminal differentiation defects and variable immune abnormalities.	J Allergy Clin Immunol.
atopic dermatitis	EMCN	Expression profiling by array, count, 3 development stage, 2 disease state, 11 individual sets	GPL570	GSE32924 Samples	Nonlesional atopic dermatitis skin is characterized by broad terminal differentiation defects and variable immune abnormalities.	J Allergy Clin Immunol.
atopic dermatitis	LHFP	Expression profiling by array, count, 3 development stage, 2 disease state, 11 individual sets	GPL570	GSE32924 Samples	Nonlesional atopic dermatitis skin is characterized by broad terminal differentiation defects and variable immune abnormalities.	J Allergy Clin Immunol.
atopic dermatitis	ATP6V0E1	Expression profiling by array, count, 3 development stage, 2 disease state, 11 individual sets	GPL570	GSE32924 Samples	Nonlesional atopic dermatitis skin is characterized by broad terminal differentiation defects and variable immune abnormalities.	J Allergy Clin Immunol.
atopic dermatitis	DKK2	Expression profiling by array, count, 3 development stage, 2 disease state, 11 individual sets	GPL570	GSE32924 Samples	Nonlesional atopic dermatitis skin is characterized by broad terminal differentiation defects and variable immune abnormalities.	J Allergy Clin Immunol.
atopic dermatitis	NA	Expression profiling by array, count, 3 development stage, 2 disease state, 11 individual sets	GPL570	GSE32924 Samples	Nonlesional atopic dermatitis skin is characterized by broad terminal differentiation defects and variable immune abnormalities.	J Allergy Clin Immunol.
atopic dermatitis	SVEP1	Expression profiling by array, count, 3 development stage, 2 disease state, 11 individual sets	GPL570	GSE32924 Samples	Nonlesional atopic dermatitis skin is characterized by broad terminal differentiation defects and variable immune abnormalities.	J Allergy Clin Immunol.
atopic dermatitis	NA	Expression profiling by array, count, 3 development stage, 2 disease state, 11 individual sets	GPL570	GSE32924 Samples	Nonlesional atopic dermatitis skin is characterized by broad terminal differentiation defects and variable immune abnormalities.	J Allergy Clin Immunol.
atopic dermatitis	EGR1	Expression profiling by array, count, 3 development stage, 2 disease state, 11 individual sets	GPL570	GSE32924 Samples	Nonlesional atopic dermatitis skin is characterized by broad terminal differentiation defects and variable immune abnormalities.	J Allergy Clin Immunol.
atopic dermatitis	MALAT1	Expression profiling by array, count, 3 development stage, 2 disease state, 11 individual sets	GPL570	GSE32924 Samples	Nonlesional atopic dermatitis skin is characterized by broad terminal differentiation defects and variable immune abnormalities.	J Allergy Clin Immunol.
atopic dermatitis	PSD3	Expression profiling by array, count, 3 development stage, 2 disease state, 11 individual sets	GPL570	GSE32924 Samples	Nonlesional atopic dermatitis skin is characterized by broad terminal differentiation defects and variable immune abnormalities.	J Allergy Clin Immunol.
atopic dermatitis	NA	Expression profiling by array, count, 3 development stage, 2 disease state, 11 individual sets	GPL570	GSE32924 Samples	Nonlesional atopic dermatitis skin is characterized by broad terminal differentiation defects and variable immune abnormalities.	J Allergy Clin Immunol.
atopic dermatitis	PTK2	Expression profiling by array, count, 3 development stage, 2 disease state, 11 individual sets	GPL570	GSE32924 Samples	Nonlesional atopic dermatitis skin is characterized by broad terminal differentiation defects and variable immune abnormalities.	J Allergy Clin Immunol.
atopic dermatitis	NA	Expression profiling by array, count, 3 development stage, 2 disease state, 11 individual sets	GPL570	GSE32924 Samples	Nonlesional atopic dermatitis skin is characterized by broad terminal differentiation defects and variable immune abnormalities.	J Allergy Clin Immunol.
atopic dermatitis	MTUS1	Expression profiling by array, count, 3 development stage, 2 disease state, 11 individual sets	GPL570	GSE32924 Samples	Nonlesional atopic dermatitis skin is characterized by broad terminal differentiation defects and variable immune abnormalities.	J Allergy Clin Immunol.
atopic dermatitis	FOXO1	Expression profiling by array, count, 3 development stage, 2 disease state, 11 individual sets	GPL570	GSE32924 Samples	Nonlesional atopic dermatitis skin is characterized by broad terminal differentiation defects and variable immune abnormalities.	J Allergy Clin Immunol.
atopic dermatitis	MID2	Expression profiling by array, count, 3 development stage, 2 disease state, 11 individual sets	GPL570	GSE32924 Samples	Nonlesional atopic dermatitis skin is characterized by broad terminal differentiation defects and variable immune abnormalities.	J Allergy Clin Immunol.
atopic dermatitis	NCOA1	Expression profiling by array, count, 3 development stage, 2 disease state, 11 individual sets	GPL570	GSE32924 Samples	Nonlesional atopic dermatitis skin is characterized by broad terminal differentiation defects and variable immune abnormalities.	J Allergy Clin Immunol.
atopic dermatitis	ATF6	Expression profiling by array, count, 3 development stage, 2 disease state, 11 individual sets	GPL570	GSE32924 Samples	Nonlesional atopic dermatitis skin is characterized by broad terminal differentiation defects and variable immune abnormalities.	J Allergy Clin Immunol.
atopic dermatitis	PCDHB14	Expression profiling by array, count, 3 development stage, 2 disease state, 11 individual sets	GPL570	GSE32924 Samples	Nonlesional atopic dermatitis skin is characterized by broad terminal differentiation defects and variable immune abnormalities.	J Allergy Clin Immunol.
atopic dermatitis	EMX2	Expression profiling by array, count, 3 development stage, 2 disease state, 11 individual sets	GPL570	GSE32924 Samples	Nonlesional atopic dermatitis skin is characterized by broad terminal differentiation defects and variable immune abnormalities.	J Allergy Clin Immunol.
atopic dermatitis	C14ORF132	Expression profiling by array, count, 3 development stage, 2 disease state, 11 individual sets	GPL570	GSE32924 Samples	Nonlesional atopic dermatitis skin is characterized by broad terminal differentiation defects and variable immune abnormalities.	J Allergy Clin Immunol.
atopic dermatitis	SNTB1	Expression profiling by array, count, 3 development stage, 2 disease state, 11 individual sets	GPL570	GSE32924 Samples	Nonlesional atopic dermatitis skin is characterized by broad terminal differentiation defects and variable immune abnormalities.	J Allergy Clin Immunol.
atopic dermatitis	SVIP	Expression profiling by array, count, 3 development stage, 2 disease state, 11 individual sets	GPL570	GSE32924 Samples	Nonlesional atopic dermatitis skin is characterized by broad terminal differentiation defects and variable immune abnormalities.	J Allergy Clin Immunol.
atopic dermatitis	JAZF1	Expression profiling by array, count, 3 development stage, 2 disease state, 11 individual sets	GPL570	GSE32924 Samples	Nonlesional atopic dermatitis skin is characterized by broad terminal differentiation defects and variable immune abnormalities.	J Allergy Clin Immunol.
atopic dermatitis	SDC2	Expression profiling by array, count, 3 development stage, 2 disease state, 11 individual sets	GPL570	GSE32924 Samples	Nonlesional atopic dermatitis skin is characterized by broad terminal differentiation defects and variable immune abnormalities.	J Allergy Clin Immunol.
atopic dermatitis	PID1	Expression profiling by array, count, 3 development stage, 2 disease state, 11 individual sets	GPL570	GSE32924 Samples	Nonlesional atopic dermatitis skin is characterized by broad terminal differentiation defects and variable immune abnormalities.	J Allergy Clin Immunol.

atopic dermatitis	LAMA2	Expression profiling by array, count, 3 development stage, 2 disease state, 11 individual sets	GPL570	GSE32924 Samples	Nonlesional atopic dermatitis skin is characterized by broad terminal differentiation defects and variable immune abnormalities.	J Allergy Clin Immunol.
atopic dermatitis	C20ORF30	Expression profiling by array, count, 3 development stage, 2 disease state, 11 individual sets	GPL570	GSE32924 Samples	Nonlesional atopic dermatitis skin is characterized by broad terminal differentiation defects and variable immune abnormalities.	J Allergy Clin Immunol.
atopic dermatitis	TFPI	Expression profiling by array, count, 3 development stage, 2 disease state, 11 individual sets	GPL570	GSE32924 Samples	Nonlesional atopic dermatitis skin is characterized by broad terminal differentiation defects and variable immune abnormalities.	J Allergy Clin Immunol.
atopic dermatitis	EID1	Expression profiling by array, count, 3 development stage, 2 disease state, 11 individual sets	GPL570	GSE32924 Samples	Nonlesional atopic dermatitis skin is characterized by broad terminal differentiation defects and variable immune abnormalities.	J Allergy Clin Immunol.
atopic dermatitis	TUG1	Expression profiling by array, count, 3 development stage, 2 disease state, 11 individual sets	GPL570	GSE32924 Samples	Nonlesional atopic dermatitis skin is characterized by broad terminal differentiation defects and variable immune abnormalities.	J Allergy Clin Immunol.
atopic dermatitis	TCEAL3	Expression profiling by array, count, 3 development stage, 2 disease state, 11 individual sets	GPL570	GSE32924 Samples	Nonlesional atopic dermatitis skin is characterized by broad terminal differentiation defects and variable immune abnormalities.	J Allergy Clin Immunol.
atopic dermatitis	ANP32A	Expression profiling by array, count, 3 development stage, 2 disease state, 11 individual sets	GPL570	GSE32924 Samples	Nonlesional atopic dermatitis skin is characterized by broad terminal differentiation defects and variable immune abnormalities.	J Allergy Clin Immunol.
atopic dermatitis	TCF7L2	Expression profiling by array, count, 3 development stage, 2 disease state, 11 individual sets	GPL570	GSE32924 Samples	Nonlesional atopic dermatitis skin is characterized by broad terminal differentiation defects and variable immune abnormalities.	J Allergy Clin Immunol.
atopic dermatitis	SMARCA2	Expression profiling by array, count, 3 development stage, 2 disease state, 11 individual sets	GPL570	GSE32924 Samples	Nonlesional atopic dermatitis skin is characterized by broad terminal differentiation defects and variable immune abnormalities.	J Allergy Clin Immunol.
atopic dermatitis	SCD5	Expression profiling by array, count, 3 development stage, 2 disease state, 11 individual sets	GPL570	GSE32924 Samples	Nonlesional atopic dermatitis skin is characterized by broad terminal differentiation defects and variable immune abnormalities.	J Allergy Clin Immunol.
atopic dermatitis	CALD1	Expression profiling by array, count, 3 development stage, 2 disease state, 11 individual sets	GPL570	GSE32924 Samples	Nonlesional atopic dermatitis skin is characterized by broad terminal differentiation defects and variable immune abnormalities.	J Allergy Clin Immunol.
atopic dermatitis	FIBIN	Expression profiling by array, count, 3 development stage, 2 disease state, 11 individual sets	GPL570	GSE32924 Samples	Nonlesional atopic dermatitis skin is characterized by broad terminal differentiation defects and variable immune abnormalities.	J Allergy Clin Immunol.
atopic dermatitis	CPE	Expression profiling by array, count, 3 development stage, 2 disease state, 11 individual sets	GPL570	GSE32924 Samples	Nonlesional atopic dermatitis skin is characterized by broad terminal differentiation defects and variable immune abnormalities.	J Allergy Clin Immunol.
atopic dermatitis	ALDH1A1	Expression profiling by array, count, 3 development stage, 2 disease state, 11 individual sets	GPL570	GSE32924 Samples	Nonlesional atopic dermatitis skin is characterized by broad terminal differentiation defects and variable immune abnormalities.	J Allergy Clin Immunol.
atopic dermatitis	PSAPL1	Expression profiling by array, count, 3 development stage, 2 disease state, 11 individual sets	GPL570	GSE32924 Samples	Nonlesional atopic dermatitis skin is characterized by broad terminal differentiation defects and variable immune abnormalities.	J Allergy Clin Immunol.
atopic dermatitis	CARD18	Expression profiling by array, count, 3 development stage, 2 disease state, 11 individual sets	GPL570	GSE32924 Samples	Nonlesional atopic dermatitis skin is characterized by broad terminal differentiation defects and variable immune abnormalities.	J Allergy Clin Immunol.
atopic dermatitis	ABHD12B	Expression profiling by array, count, 3 development stage, 2 disease state, 11 individual sets	GPL570	GSE32924 Samples	Nonlesional atopic dermatitis skin is characterized by broad terminal differentiation defects and variable immune abnormalities.	J Allergy Clin Immunol.
atopic dermatitis	OGN	Expression profiling by array, count, 3 development stage, 2 disease state, 11 individual sets	GPL570	GSE32924 Samples	Nonlesional atopic dermatitis skin is characterized by broad terminal differentiation defects and variable immune abnormalities.	J Allergy Clin Immunol.
atopic dermatitis	ZNF148	Expression profiling by array, count, 3 development stage, 2 disease state, 11 individual sets	GPL570	GSE32924 Samples	Nonlesional atopic dermatitis skin is characterized by broad terminal differentiation defects and variable immune abnormalities.	J Allergy Clin Immunol.
atopic dermatitis	SIPA1L1	Expression profiling by array, count, 3 development stage, 2 disease state, 11 individual sets	GPL570	GSE32924 Samples	Nonlesional atopic dermatitis skin is characterized by broad terminal differentiation defects and variable immune abnormalities.	J Allergy Clin Immunol.
atopic dermatitis	PAIP2B	Expression profiling by array, count, 3 development stage, 2 disease state, 11 individual sets	GPL570	GSE32924 Samples	Nonlesional atopic dermatitis skin is characterized by broad terminal differentiation defects and variable immune abnormalities.	J Allergy Clin Immunol.
atopic dermatitis	KDM5A	Expression profiling by array, count, 3 development stage, 2 disease state, 11 individual sets	GPL570	GSE32924 Samples	Nonlesional atopic dermatitis skin is characterized by broad terminal differentiation defects and variable immune abnormalities.	J Allergy Clin Immunol.
atopic dermatitis	EPHX2	Expression profiling by array, count, 3 development stage, 2 disease state, 11 individual sets	GPL570	GSE32924 Samples	Nonlesional atopic dermatitis skin is characterized by broad terminal differentiation defects and variable immune abnormalities.	J Allergy Clin Immunol.
atopic dermatitis	DST	Expression profiling by array, count, 3 development stage, 2 disease state, 11 individual sets	GPL570	GSE32924 Samples	Nonlesional atopic dermatitis skin is characterized by broad terminal differentiation defects and variable immune abnormalities.	J Allergy Clin Immunol.
atopic dermatitis	NA	Expression profiling by array, count, 3 development stage, 2 disease state, 11 individual sets	GPL570	GSE32924 Samples	Nonlesional atopic dermatitis skin is characterized by broad terminal differentiation defects and variable immune abnormalities.	J Allergy Clin Immunol.
atopic dermatitis	STK38	Expression profiling by array, count, 3 development stage, 2 disease state, 11 individual sets	GPL570	GSE32924 Samples	Nonlesional atopic dermatitis skin is characterized by broad terminal differentiation defects and variable immune abnormalities.	J Allergy Clin Immunol.
atopic dermatitis	ZNF512	Expression profiling by array, count, 3 development stage, 2 disease state, 11 individual sets	GPL570	GSE32924 Samples	Nonlesional atopic dermatitis skin is characterized by broad terminal differentiation defects and variable immune abnormalities.	J Allergy Clin Immunol.
atopic dermatitis	NBR1	Expression profiling by array, count, 3 development stage, 2 disease state, 11 individual sets	GPL570	GSE32924 Samples	Nonlesional atopic dermatitis skin is characterized by broad terminal differentiation defects and variable immune abnormalities.	J Allergy Clin Immunol.
atopic dermatitis	CRYZL1	Expression profiling by array, count, 3 development stage, 2 disease state, 11 individual sets	GPL570	GSE32924 Samples	Nonlesional atopic dermatitis skin is characterized by broad terminal differentiation defects and variable immune abnormalities.	J Allergy Clin Immunol.
atopic dermatitis	ZC3H7A	Expression profiling by array, count, 3 development stage, 2 disease state, 11 individual sets	GPL570	GSE32924 Samples	Nonlesional atopic dermatitis skin is characterized by broad terminal differentiation defects and variable immune abnormalities.	J Allergy Clin Immunol.
atopic dermatitis	PNPLA8	Expression profiling by array, count, 3 development stage, 2 disease state, 11 individual sets	GPL570	GSE32924 Samples	Nonlesional atopic dermatitis skin is characterized by broad terminal differentiation defects and variable immune abnormalities.	J Allergy Clin Immunol.
atopic dermatitis	USP47	Expression profiling by array, count, 3 development stage, 2 disease state, 11 individual sets	GPL570	GSE32924 Samples	Nonlesional atopic dermatitis skin is characterized by broad terminal differentiation defects and variable immune abnormalities.	J Allergy Clin Immunol.
atopic dermatitis	KIAA0776	Expression profiling by array, count, 3 development stage, 2 disease state, 11 individual sets	GPL570	GSE32924 Samples	Nonlesional atopic dermatitis skin is characterized by broad terminal differentiation defects and variable immune abnormalities.	J Allergy Clin Immunol.
atopic dermatitis	ARIH1	Expression profiling by array, count, 3 development stage, 2 disease state, 11 individual sets	GPL570	GSE32924 Samples	Nonlesional atopic dermatitis skin is characterized by broad terminal differentiation defects and variable immune abnormalities.	J Allergy Clin Immunol.
atopic dermatitis	PLXNA2	Expression profiling by array, count, 3 development stage, 2 disease state, 11 individual sets	GPL570	GSE32924 Samples	Nonlesional atopic dermatitis skin is characterized by broad terminal differentiation defects and variable immune abnormalities.	J Allergy Clin Immunol.
atopic dermatitis	CRIPAK	Expression profiling by array, count, 3 development stage, 2 disease state, 11 individual sets	GPL570	GSE32924 Samples	Nonlesional atopic dermatitis skin is characterized by broad terminal differentiation defects and variable immune abnormalities.	J Allergy Clin Immunol.
atopic dermatitis	BAT1	Expression profiling by array, count, 3 development stage, 2 disease state, 11 individual sets	GPL570	GSE32924 Samples	Nonlesional atopic dermatitis skin is characterized by broad terminal differentiation defects and variable immune abnormalities.	J Allergy Clin Immunol.
atopic dermatitis	NA	Expression profiling by array, count, 3 development stage, 2 disease state, 11 individual sets	GPL570	GSE32924 Samples	Nonlesional atopic dermatitis skin is characterized by broad terminal differentiation defects and variable immune abnormalities.	J Allergy Clin Immunol.
atopic dermatitis	NA	Expression profiling by array, count, 3 development stage, 2 disease state, 11 individual sets	GPL570	GSE32924 Samples	Nonlesional atopic dermatitis skin is characterized by broad terminal differentiation defects and variable immune abnormalities.	J Allergy Clin Immunol.
atopic dermatitis	NA	Expression profiling by array, count, 3 development stage, 2 disease state, 11 individual sets	GPL570	GSE32924 Samples	Nonlesional atopic dermatitis skin is characterized by broad terminal differentiation defects and variable immune abnormalities.	J Allergy Clin Immunol.
atopic dermatitis	IGDCC4	Expression profiling by array, count, 3 development stage, 2 disease state, 11 individual sets	GPL570	GSE32924 Samples	Nonlesional atopic dermatitis skin is characterized by broad terminal differentiation defects and variable immune abnormalities.	J Allergy Clin Immunol.

atopic dermatitis	LRRC17	Expression profiling by array, count, 3 development stage, 2 disease state, 11 individual sets	GPL570	GSE32924 Samples	Nonlesional atopic dermatitis skin is characterized by broad terminal differentiation defects and variable immune abnormalities.	J Allergy Clin Immunol.
atopic dermatitis	ZEB1	Expression profiling by array, count, 3 development stage, 2 disease state, 11 individual sets	GPL570	GSE32924 Samples	Nonlesional atopic dermatitis skin is characterized by broad terminal differentiation defects and variable immune abnormalities.	J Allergy Clin Immunol.
atopic dermatitis	PI16	Expression profiling by array, count, 3 development stage, 2 disease state, 11 individual sets	GPL570	GSE32924 Samples	Nonlesional atopic dermatitis skin is characterized by broad terminal differentiation defects and variable immune abnormalities.	J Allergy Clin Immunol.
atopic dermatitis	CYBRD1	Expression profiling by array, count, 3 development stage, 2 disease state, 11 individual sets	GPL570	GSE32924 Samples	Nonlesional atopic dermatitis skin is characterized by broad terminal differentiation defects and variable immune abnormalities.	J Allergy Clin Immunol.
atopic dermatitis	COL8A2	Expression profiling by array, count, 3 development stage, 2 disease state, 11 individual sets	GPL570	GSE32924 Samples	Nonlesional atopic dermatitis skin is characterized by broad terminal differentiation defects and variable immune abnormalities.	J Allergy Clin Immunol.
atopic dermatitis	NA	Expression profiling by array, count, 3 development stage, 2 disease state, 11 individual sets	GPL570	GSE32924 Samples	Nonlesional atopic dermatitis skin is characterized by broad terminal differentiation defects and variable immune abnormalities.	J Allergy Clin Immunol.
atopic dermatitis	DDX42	Expression profiling by array, count, 3 development stage, 2 disease state, 11 individual sets	GPL570	GSE32924 Samples	Nonlesional atopic dermatitis skin is characterized by broad terminal differentiation defects and variable immune abnormalities.	J Allergy Clin Immunol.
atopic dermatitis	CCDC109A	Expression profiling by array, count, 3 development stage, 2 disease state, 11 individual sets	GPL570	GSE32924 Samples	Nonlesional atopic dermatitis skin is characterized by broad terminal differentiation defects and variable immune abnormalities.	J Allergy Clin Immunol.
atopic dermatitis	AP3B1	Expression profiling by array, count, 3 development stage, 2 disease state, 11 individual sets	GPL570	GSE32924 Samples	Nonlesional atopic dermatitis skin is characterized by broad terminal differentiation defects and variable immune abnormalities.	J Allergy Clin Immunol.
atopic dermatitis	LRCH1	Expression profiling by array, count, 3 development stage, 2 disease state, 11 individual sets	GPL570	GSE32924 Samples	Nonlesional atopic dermatitis skin is characterized by broad terminal differentiation defects and variable immune abnormalities.	J Allergy Clin Immunol.
atopic dermatitis	MLLT10	Expression profiling by array, count, 3 development stage, 2 disease state, 11 individual sets	GPL570	GSE32924 Samples	Nonlesional atopic dermatitis skin is characterized by broad terminal differentiation defects and variable immune abnormalities.	J Allergy Clin Immunol.
atopic dermatitis	ZMYND11	Expression profiling by array, count, 3 development stage, 2 disease state, 11 individual sets	GPL570	GSE32924 Samples	Nonlesional atopic dermatitis skin is characterized by broad terminal differentiation defects and variable immune abnormalities.	J Allergy Clin Immunol.
atopic dermatitis	TMCC1	Expression profiling by array, count, 3 development stage, 2 disease state, 11 individual sets	GPL570	GSE32924 Samples	Nonlesional atopic dermatitis skin is characterized by broad terminal differentiation defects and variable immune abnormalities.	J Allergy Clin Immunol.
atopic dermatitis	CTSO	Expression profiling by array, count, 3 development stage, 2 disease state, 11 individual sets	GPL570	GSE32924 Samples	Nonlesional atopic dermatitis skin is characterized by broad terminal differentiation defects and variable immune abnormalities.	J Allergy Clin Immunol.
atopic dermatitis	ZNF436	Expression profiling by array, count, 3 development stage, 2 disease state, 11 individual sets	GPL570	GSE32924 Samples	Nonlesional atopic dermatitis skin is characterized by broad terminal differentiation defects and variable immune abnormalities.	J Allergy Clin Immunol.
atopic dermatitis	NA	Expression profiling by array, count, 3 development stage, 2 disease state, 11 individual sets	GPL570	GSE32924 Samples	Nonlesional atopic dermatitis skin is characterized by broad terminal differentiation defects and variable immune abnormalities.	J Allergy Clin Immunol.
atopic dermatitis	NF1	Expression profiling by array, count, 3 development stage, 2 disease state, 11 individual sets	GPL570	GSE32924 Samples	Nonlesional atopic dermatitis skin is characterized by broad terminal differentiation defects and variable immune abnormalities.	J Allergy Clin Immunol.
atopic dermatitis	UBE4B	Expression profiling by array, count, 3 development stage, 2 disease state, 11 individual sets	GPL570	GSE32924 Samples	Nonlesional atopic dermatitis skin is characterized by broad terminal differentiation defects and variable immune abnormalities.	J Allergy Clin Immunol.
atopic dermatitis	ARID1B	Expression profiling by array, count, 3 development stage, 2 disease state, 11 individual sets	GPL570	GSE32924 Samples	Nonlesional atopic dermatitis skin is characterized by broad terminal differentiation defects and variable immune abnormalities.	J Allergy Clin Immunol.
atopic dermatitis	EPHA4	Expression profiling by array, count, 3 development stage, 2 disease state, 11 individual sets	GPL570	GSE32924 Samples	Nonlesional atopic dermatitis skin is characterized by broad terminal differentiation defects and variable immune abnormalities.	J Allergy Clin Immunol.
atopic dermatitis	KCNMB4	Expression profiling by array, count, 3 development stage, 2 disease state, 11 individual sets	GPL570	GSE32924 Samples	Nonlesional atopic dermatitis skin is characterized by broad terminal differentiation defects and variable immune abnormalities.	J Allergy Clin Immunol.
atopic dermatitis	COBL	Expression profiling by array, count, 3 development stage, 2 disease state, 11 individual sets	GPL570	GSE32924 Samples	Nonlesional atopic dermatitis skin is characterized by broad terminal differentiation defects and variable immune abnormalities.	J Allergy Clin Immunol.
atopic dermatitis	SLITRK6	Expression profiling by array, count, 3 development stage, 2 disease state, 11 individual sets	GPL570	GSE32924 Samples	Nonlesional atopic dermatitis skin is characterized by broad terminal differentiation defects and variable immune abnormalities.	J Allergy Clin Immunol.
atopic dermatitis	NAP1L2	Expression profiling by array, count, 3 development stage, 2 disease state, 11 individual sets	GPL570	GSE32924 Samples	Nonlesional atopic dermatitis skin is characterized by broad terminal differentiation defects and variable immune abnormalities.	J Allergy Clin Immunol.
atopic dermatitis	NA	Expression profiling by array, count, 3 development stage, 2 disease state, 11 individual sets	GPL570	GSE32924 Samples	Nonlesional atopic dermatitis skin is characterized by broad terminal differentiation defects and variable immune abnormalities.	J Allergy Clin Immunol.
atopic dermatitis	DHRS12	Expression profiling by array, count, 3 development stage, 2 disease state, 11 individual sets	GPL570	GSE32924 Samples	Nonlesional atopic dermatitis skin is characterized by broad terminal differentiation defects and variable immune abnormalities.	J Allergy Clin Immunol.
atopic dermatitis	EPB41L4B	Expression profiling by array, count, 3 development stage, 2 disease state, 11 individual sets	GPL570	GSE32924 Samples	Nonlesional atopic dermatitis skin is characterized by broad terminal differentiation defects and variable immune abnormalities.	J Allergy Clin Immunol.
atopic dermatitis	NA	Expression profiling by array, count, 3 development stage, 2 disease state, 11 individual sets	GPL570	GSE32924 Samples	Nonlesional atopic dermatitis skin is characterized by broad terminal differentiation defects and variable immune abnormalities.	J Allergy Clin Immunol.
atopic dermatitis	ALG10B	Expression profiling by array, count, 3 development stage, 2 disease state, 11 individual sets	GPL570	GSE32924 Samples	Nonlesional atopic dermatitis skin is characterized by broad terminal differentiation defects and variable immune abnormalities.	J Allergy Clin Immunol.
atopic dermatitis	TPR	Expression profiling by array, count, 3 development stage, 2 disease state, 11 individual sets	GPL570	GSE32924 Samples	Nonlesional atopic dermatitis skin is characterized by broad terminal differentiation defects and variable immune abnormalities.	J Allergy Clin Immunol.
atopic dermatitis	LOC730102	Expression profiling by array, count, 3 development stage, 2 disease state, 11 individual sets	GPL570	GSE32924 Samples	Nonlesional atopic dermatitis skin is characterized by broad terminal differentiation defects and variable immune abnormalities.	J Allergy Clin Immunol.
atopic dermatitis	PRKAB2	Expression profiling by array, count, 3 development stage, 2 disease state, 11 individual sets	GPL570	GSE32924 Samples	Nonlesional atopic dermatitis skin is characterized by broad terminal differentiation defects and variable immune abnormalities.	J Allergy Clin Immunol.
atopic dermatitis	KDM5A	Expression profiling by array, count, 3 development stage, 2 disease state, 11 individual sets	GPL570	GSE32924 Samples	Nonlesional atopic dermatitis skin is characterized by broad terminal differentiation defects and variable immune abnormalities.	J Allergy Clin Immunol.
atopic dermatitis	NMNAT3	Expression profiling by array, count, 3 development stage, 2 disease state, 11 individual sets	GPL570	GSE32924 Samples	Nonlesional atopic dermatitis skin is characterized by broad terminal differentiation defects and variable immune abnormalities.	J Allergy Clin Immunol.
atopic dermatitis	HNRNPU	Expression profiling by array, count, 3 development stage, 2 disease state, 11 individual sets	GPL570	GSE32924 Samples	Nonlesional atopic dermatitis skin is characterized by broad terminal differentiation defects and variable immune abnormalities.	J Allergy Clin Immunol.
atopic dermatitis	GSK3B	Expression profiling by array, count, 3 development stage, 2 disease state, 11 individual sets	GPL570	GSE32924 Samples	Nonlesional atopic dermatitis skin is characterized by broad terminal differentiation defects and variable immune abnormalities.	J Allergy Clin Immunol.
atopic dermatitis	MED23	Expression profiling by array, count, 3 development stage, 2 disease state, 11 individual sets	GPL570	GSE32924 Samples	Nonlesional atopic dermatitis skin is characterized by broad terminal differentiation defects and variable immune abnormalities.	J Allergy Clin Immunol.
atopic dermatitis	NPR2	Expression profiling by array, count, 3 development stage, 2 disease state, 11 individual sets	GPL570	GSE32924 Samples	Nonlesional atopic dermatitis skin is characterized by broad terminal differentiation defects and variable immune abnormalities.	J Allergy Clin Immunol.
atopic dermatitis	ZDHHC9	Expression profiling by array, count, 3 development stage, 2 disease state, 11 individual sets	GPL570	GSE32924 Samples	Nonlesional atopic dermatitis skin is characterized by broad terminal differentiation defects and variable immune abnormalities.	J Allergy Clin Immunol.
atopic dermatitis	SAV1	Expression profiling by array, count, 3 development stage, 2 disease state, 11 individual sets	GPL570	GSE32924 Samples	Nonlesional atopic dermatitis skin is characterized by broad terminal differentiation defects and variable immune abnormalities.	J Allergy Clin Immunol.
atopic dermatitis	NFIB	Expression profiling by array, count, 3 development stage, 2 disease state, 11 individual sets	GPL570	GSE32924 Samples	Nonlesional atopic dermatitis skin is characterized by broad terminal differentiation defects and variable immune abnormalities.	J Allergy Clin Immunol.

atopic dermatitis	LAMA2	Expression profiling by array, count, 3 development stage, 2 disease state, 11 individual sets	GPL570	GSE32924 Samples	Nonlesional atopic dermatitis skin is characterized by broad terminal differentiation defects and variable immune abnormalities.	J Allergy Clin Immunol.
atopic dermatitis	NA	Expression profiling by array, count, 3 development stage, 2 disease state, 11 individual sets	GPL570	GSE32924 Samples	Nonlesional atopic dermatitis skin is characterized by broad terminal differentiation defects and variable immune abnormalities.	J Allergy Clin Immunol.
atopic dermatitis	NAP1L3	Expression profiling by array, count, 3 development stage, 2 disease state, 11 individual sets	GPL570	GSE32924 Samples	Nonlesional atopic dermatitis skin is characterized by broad terminal differentiation defects and variable immune abnormalities.	J Allergy Clin Immunol.
atopic dermatitis	ABCA6	Expression profiling by array, count, 3 development stage, 2 disease state, 11 individual sets	GPL570	GSE32924 Samples	Nonlesional atopic dermatitis skin is characterized by broad terminal differentiation defects and variable immune abnormalities.	J Allergy Clin Immunol.
atopic dermatitis	NA	Expression profiling by array, count, 3 development stage, 2 disease state, 11 individual sets	GPL570	GSE32924 Samples	Nonlesional atopic dermatitis skin is characterized by broad terminal differentiation defects and variable immune abnormalities.	J Allergy Clin Immunol.
atopic dermatitis	MACROD2	Expression profiling by array, count, 3 development stage, 2 disease state, 11 individual sets	GPL570	GSE32924 Samples	Nonlesional atopic dermatitis skin is characterized by broad terminal differentiation defects and variable immune abnormalities.	J Allergy Clin Immunol.
atopic dermatitis	C14ORF132	Expression profiling by array, count, 3 development stage, 2 disease state, 11 individual sets	GPL570	GSE32924 Samples	Nonlesional atopic dermatitis skin is characterized by broad terminal differentiation defects and variable immune abnormalities.	J Allergy Clin Immunol.
atopic dermatitis	NA	Expression profiling by array, count, 3 development stage, 2 disease state, 11 individual sets	GPL570	GSE32924 Samples	Nonlesional atopic dermatitis skin is characterized by broad terminal differentiation defects and variable immune abnormalities.	J Allergy Clin Immunol.
atopic dermatitis	BCHE	Expression profiling by array, count, 3 development stage, 2 disease state, 11 individual sets	GPL570	GSE32924 Samples	Nonlesional atopic dermatitis skin is characterized by broad terminal differentiation defects and variable immune abnormalities.	J Allergy Clin Immunol.
atopic dermatitis	SEC63	Expression profiling by array, count, 3 development stage, 2 disease state, 11 individual sets	GPL570	GSE32924 Samples	Nonlesional atopic dermatitis skin is characterized by broad terminal differentiation defects and variable immune abnormalities.	J Allergy Clin Immunol.
atopic dermatitis	DHX36	Expression profiling by array, count, 3 development stage, 2 disease state, 11 individual sets	GPL570	GSE32924 Samples	Nonlesional atopic dermatitis skin is characterized by broad terminal differentiation defects and variable immune abnormalities.	J Allergy Clin Immunol.
atopic dermatitis	NA	Expression profiling by array, count, 3 development stage, 2 disease state, 11 individual sets	GPL570	GSE32924 Samples	Nonlesional atopic dermatitis skin is characterized by broad terminal differentiation defects and variable immune abnormalities.	J Allergy Clin Immunol.
atopic dermatitis	SFRS2IP	Expression profiling by array, count, 3 development stage, 2 disease state, 11 individual sets	GPL570	GSE32924 Samples	Nonlesional atopic dermatitis skin is characterized by broad terminal differentiation defects and variable immune abnormalities.	J Allergy Clin Immunol.
atopic dermatitis	MYO6	Expression profiling by array, count, 3 development stage, 2 disease state, 11 individual sets	GPL570	GSE32924 Samples	Nonlesional atopic dermatitis skin is characterized by broad terminal differentiation defects and variable immune abnormalities.	J Allergy Clin Immunol.
atopic dermatitis	GNAS	Expression profiling by array, count, 3 development stage, 2 disease state, 11 individual sets	GPL570	GSE32924 Samples	Nonlesional atopic dermatitis skin is characterized by broad terminal differentiation defects and variable immune abnormalities.	J Allergy Clin Immunol.
atopic dermatitis	USP16	Expression profiling by array, count, 3 development stage, 2 disease state, 11 individual sets	GPL570	GSE32924 Samples	Nonlesional atopic dermatitis skin is characterized by broad terminal differentiation defects and variable immune abnormalities.	J Allergy Clin Immunol.
atopic dermatitis	PRKCH	Expression profiling by array, count, 3 development stage, 2 disease state, 11 individual sets	GPL570	GSE32924 Samples	Nonlesional atopic dermatitis skin is characterized by broad terminal differentiation defects and variable immune abnormalities.	J Allergy Clin Immunol.
atopic dermatitis	PLDN	Expression profiling by array, count, 3 development stage, 2 disease state, 11 individual sets	GPL570	GSE32924 Samples	Nonlesional atopic dermatitis skin is characterized by broad terminal differentiation defects and variable immune abnormalities.	J Allergy Clin Immunol.
atopic dermatitis	MAP4K4	Expression profiling by array, count, 3 development stage, 2 disease state, 11 individual sets	GPL570	GSE32924 Samples	Nonlesional atopic dermatitis skin is characterized by broad terminal differentiation defects and variable immune abnormalities.	J Allergy Clin Immunol.
atopic dermatitis	SRPK2	Expression profiling by array, count, 3 development stage, 2 disease state, 11 individual sets	GPL570	GSE32924 Samples	Nonlesional atopic dermatitis skin is characterized by broad terminal differentiation defects and variable immune abnormalities.	J Allergy Clin Immunol.
atopic dermatitis	NA	Expression profiling by array, count, 3 development stage, 2 disease state, 11 individual sets	GPL570	GSE32924 Samples	Nonlesional atopic dermatitis skin is characterized by broad terminal differentiation defects and variable immune abnormalities.	J Allergy Clin Immunol.
atopic dermatitis	CXORF26	Expression profiling by array, count, 3 development stage, 2 disease state, 11 individual sets	GPL570	GSE32924 Samples	Nonlesional atopic dermatitis skin is characterized by broad terminal differentiation defects and variable immune abnormalities.	J Allergy Clin Immunol.
atopic dermatitis	ANKHD1	Expression profiling by array, count, 3 development stage, 2 disease state, 11 individual sets	GPL570	GSE32924 Samples	Nonlesional atopic dermatitis skin is characterized by broad terminal differentiation defects and variable immune abnormalities.	J Allergy Clin Immunol.
atopic dermatitis	GAPVD1	Expression profiling by array, count, 3 development stage, 2 disease state, 11 individual sets	GPL570	GSE32924 Samples	Nonlesional atopic dermatitis skin is characterized by broad terminal differentiation defects and variable immune abnormalities.	J Allergy Clin Immunol.
atopic dermatitis	RICTOR	Expression profiling by array, count, 3 development stage, 2 disease state, 11 individual sets	GPL570	GSE32924 Samples	Nonlesional atopic dermatitis skin is characterized by broad terminal differentiation defects and variable immune abnormalities.	J Allergy Clin Immunol.
atopic dermatitis	PLEKHG1	Expression profiling by array, count, 3 development stage, 2 disease state, 11 individual sets	GPL570	GSE32924 Samples	Nonlesional atopic dermatitis skin is characterized by broad terminal differentiation defects and variable immune abnormalities.	J Allergy Clin Immunol.
atopic dermatitis	ZMYM6	Expression profiling by array, count, 3 development stage, 2 disease state, 11 individual sets	GPL570	GSE32924 Samples	Nonlesional atopic dermatitis skin is characterized by broad terminal differentiation defects and variable immune abnormalities.	J Allergy Clin Immunol.
atopic dermatitis	JAK2	Expression profiling by array, count, 3 development stage, 2 disease state, 11 individual sets	GPL570	GSE32924 Samples	Nonlesional atopic dermatitis skin is characterized by broad terminal differentiation defects and variable immune abnormalities.	J Allergy Clin Immunol.
atopic dermatitis	CAMK2D	Expression profiling by array, count, 3 development stage, 2 disease state, 11 individual sets	GPL570	GSE32924 Samples	Nonlesional atopic dermatitis skin is characterized by broad terminal differentiation defects and variable immune abnormalities.	J Allergy Clin Immunol.
atopic dermatitis	CREBL2	Expression profiling by array, count, 3 development stage, 2 disease state, 11 individual sets	GPL570	GSE32924 Samples	Nonlesional atopic dermatitis skin is characterized by broad terminal differentiation defects and variable immune abnormalities.	J Allergy Clin Immunol.
atopic dermatitis	PAFAH1B1	Expression profiling by array, count, 3 development stage, 2 disease state, 11 individual sets	GPL570	GSE32924 Samples	Nonlesional atopic dermatitis skin is characterized by broad terminal differentiation defects and variable immune abnormalities.	J Allergy Clin Immunol.
atopic dermatitis	ARFGEF1	Expression profiling by array, count, 3 development stage, 2 disease state, 11 individual sets	GPL570	GSE32924 Samples	Nonlesional atopic dermatitis skin is characterized by broad terminal differentiation defects and variable immune abnormalities.	J Allergy Clin Immunol.
atopic dermatitis	PTPLAD1	Expression profiling by array, count, 3 development stage, 2 disease state, 11 individual sets	GPL570	GSE32924 Samples	Nonlesional atopic dermatitis skin is characterized by broad terminal differentiation defects and variable immune abnormalities.	J Allergy Clin Immunol.
atopic dermatitis	FAM115A	Expression profiling by array, count, 3 development stage, 2 disease state, 11 individual sets	GPL570	GSE32924 Samples	Nonlesional atopic dermatitis skin is characterized by broad terminal differentiation defects and variable immune abnormalities.	J Allergy Clin Immunol.
atopic dermatitis	FGFR2	Expression profiling by array, count, 3 development stage, 2 disease state, 11 individual sets	GPL570	GSE32924 Samples	Nonlesional atopic dermatitis skin is characterized by broad terminal differentiation defects and variable immune abnormalities.	J Allergy Clin Immunol.
atopic dermatitis	PALLD	Expression profiling by array, count, 3 development stage, 2 disease state, 11 individual sets	GPL570	GSE32924 Samples	Nonlesional atopic dermatitis skin is characterized by broad terminal differentiation defects and variable immune abnormalities.	J Allergy Clin Immunol.
atopic dermatitis	GNPDA2	Expression profiling by array, count, 3 development stage, 2 disease state, 11 individual sets	GPL570	GSE32924 Samples	Nonlesional atopic dermatitis skin is characterized by broad terminal differentiation defects and variable immune abnormalities.	J Allergy Clin Immunol.
atopic dermatitis	WAC	Expression profiling by array, count, 3 development stage, 2 disease state, 11 individual sets	GPL570	GSE32924 Samples	Nonlesional atopic dermatitis skin is characterized by broad terminal differentiation defects and variable immune abnormalities.	J Allergy Clin Immunol.
atopic dermatitis	MAP3K7IP2	Expression profiling by array, count, 3 development stage, 2 disease state, 11 individual sets	GPL570	GSE32924 Samples	Nonlesional atopic dermatitis skin is characterized by broad terminal differentiation defects and variable immune abnormalities.	J Allergy Clin Immunol.
atopic dermatitis	BCOR	Expression profiling by array, count, 3 development stage, 2 disease state, 11 individual sets	GPL570	GSE32924 Samples	Nonlesional atopic dermatitis skin is characterized by broad terminal differentiation defects and variable immune abnormalities.	J Allergy Clin Immunol.
atopic dermatitis	RANBP9	Expression profiling by array, count, 3 development stage, 2 disease state, 11 individual sets	GPL570	GSE32924 Samples	Nonlesional atopic dermatitis skin is characterized by broad terminal differentiation defects and variable immune abnormalities.	J Allergy Clin Immunol.

atopic dermatitis	EPB41L3	Expression profiling by array, count, 3 development stage, 2 disease state, 11 individual sets	GPL570	GSE32924 Samples	Nonlesional atopic dermatitis skin is characterized by broad terminal differentiation defects and variable immune abnormalities.	J Allergy Clin Immunol.
atopic dermatitis	NA	Expression profiling by array, count, 3 development stage, 2 disease state, 11 individual sets	GPL570	GSE32924 Samples	Nonlesional atopic dermatitis skin is characterized by broad terminal differentiation defects and variable immune abnormalities.	J Allergy Clin Immunol.
atopic dermatitis	TSPAN5	Expression profiling by array, count, 3 development stage, 2 disease state, 11 individual sets	GPL570	GSE32924 Samples	Nonlesional atopic dermatitis skin is characterized by broad terminal differentiation defects and variable immune abnormalities.	J Allergy Clin Immunol.
atopic dermatitis	MTHFD2L	Expression profiling by array, count, 3 development stage, 2 disease state, 11 individual sets	GPL570	GSE32924 Samples	Nonlesional atopic dermatitis skin is characterized by broad terminal differentiation defects and variable immune abnormalities.	J Allergy Clin Immunol.
atopic dermatitis	ATG5	Expression profiling by array, count, 3 development stage, 2 disease state, 11 individual sets	GPL570	GSE32924 Samples	Nonlesional atopic dermatitis skin is characterized by broad terminal differentiation defects and variable immune abnormalities.	J Allergy Clin Immunol.
atopic dermatitis	SLC39A2	Expression profiling by array, count, 3 development stage, 2 disease state, 11 individual sets	GPL570	GSE32924 Samples	Nonlesional atopic dermatitis skin is characterized by broad terminal differentiation defects and variable immune abnormalities.	J Allergy Clin Immunol.
atopic dermatitis	GAS7	Expression profiling by array, count, 3 development stage, 2 disease state, 11 individual sets	GPL570	GSE32924 Samples	Nonlesional atopic dermatitis skin is characterized by broad terminal differentiation defects and variable immune abnormalities.	J Allergy Clin Immunol.
atopic dermatitis	NA	Expression profiling by array, count, 3 development stage, 2 disease state, 11 individual sets	GPL570	GSE32924 Samples	Nonlesional atopic dermatitis skin is characterized by broad terminal differentiation defects and variable immune abnormalities.	J Allergy Clin Immunol.
atopic dermatitis	NA	Expression profiling by array, count, 3 development stage, 2 disease state, 11 individual sets	GPL570	GSE32924 Samples	Nonlesional atopic dermatitis skin is characterized by broad terminal differentiation defects and variable immune abnormalities.	J Allergy Clin Immunol.
atopic dermatitis	CNTN1	Expression profiling by array, count, 3 development stage, 2 disease state, 11 individual sets	GPL570	GSE32924 Samples	Nonlesional atopic dermatitis skin is characterized by broad terminal differentiation defects and variable immune abnormalities.	J Allergy Clin Immunol.
atopic dermatitis	ABHD6	Expression profiling by array, count, 3 development stage, 2 disease state, 11 individual sets	GPL570	GSE32924 Samples	Nonlesional atopic dermatitis skin is characterized by broad terminal differentiation defects and variable immune abnormalities.	J Allergy Clin Immunol.
atopic dermatitis	C10ORF57	Expression profiling by array, count, 3 development stage, 2 disease state, 11 individual sets	GPL570	GSE32924 Samples	Nonlesional atopic dermatitis skin is characterized by broad terminal differentiation defects and variable immune abnormalities.	J Allergy Clin Immunol.
atopic dermatitis	RIT1	Expression profiling by array, count, 3 development stage, 2 disease state, 11 individual sets	GPL570	GSE32924 Samples	Nonlesional atopic dermatitis skin is characterized by broad terminal differentiation defects and variable immune abnormalities.	J Allergy Clin Immunol.
atopic dermatitis	GRHL1	Expression profiling by array, count, 3 development stage, 2 disease state, 11 individual sets	GPL570	GSE32924 Samples	Nonlesional atopic dermatitis skin is characterized by broad terminal differentiation defects and variable immune abnormalities.	J Allergy Clin Immunol.
atopic dermatitis	MUC15	Expression profiling by array, count, 3 development stage, 2 disease state, 11 individual sets	GPL570	GSE32924 Samples	Nonlesional atopic dermatitis skin is characterized by broad terminal differentiation defects and variable immune abnormalities.	J Allergy Clin Immunol.
atopic dermatitis	OTUB2	Expression profiling by array, count, 3 development stage, 2 disease state, 11 individual sets	GPL570	GSE32924 Samples	Nonlesional atopic dermatitis skin is characterized by broad terminal differentiation defects and variable immune abnormalities.	J Allergy Clin Immunol.
atopic dermatitis	NA	Expression profiling by array, count, 3 development stage, 2 disease state, 11 individual sets	GPL570	GSE32924 Samples	Nonlesional atopic dermatitis skin is characterized by broad terminal differentiation defects and variable immune abnormalities.	J Allergy Clin Immunol.
atopic dermatitis	SPRR4	Expression profiling by array, count, 3 development stage, 2 disease state, 11 individual sets	GPL570	GSE32924 Samples	Nonlesional atopic dermatitis skin is characterized by broad terminal differentiation defects and variable immune abnormalities.	J Allergy Clin Immunol.
atopic dermatitis	NA	Expression profiling by array, count, 3 development stage, 2 disease state, 11 individual sets	GPL570	GSE32924 Samples	Nonlesional atopic dermatitis skin is characterized by broad terminal differentiation defects and variable immune abnormalities.	J Allergy Clin Immunol.
atopic dermatitis	GPLD1	Expression profiling by array, count, 3 development stage, 2 disease state, 11 individual sets	GPL570	GSE32924 Samples	Nonlesional atopic dermatitis skin is characterized by broad terminal differentiation defects and variable immune abnormalities.	J Allergy Clin Immunol.
atopic dermatitis	SH3KBP1	Expression profiling by array, count, 3 development stage, 2 disease state, 11 individual sets	GPL570	GSE32924 Samples	Nonlesional atopic dermatitis skin is characterized by broad terminal differentiation defects and variable immune abnormalities.	J Allergy Clin Immunol.
atopic dermatitis	KIAA1324L	Expression profiling by array, count, 3 development stage, 2 disease state, 11 individual sets	GPL570	GSE32924 Samples	Nonlesional atopic dermatitis skin is characterized by broad terminal differentiation defects and variable immune abnormalities.	J Allergy Clin Immunol.
atopic dermatitis	ARHGAP26	Expression profiling by array, count, 3 development stage, 2 disease state, 11 individual sets	GPL570	GSE32924 Samples	Nonlesional atopic dermatitis skin is characterized by broad terminal differentiation defects and variable immune abnormalities.	J Allergy Clin Immunol.
atopic dermatitis	ANG	Expression profiling by array, count, 3 development stage, 2 disease state, 11 individual sets	GPL570	GSE32924 Samples	Nonlesional atopic dermatitis skin is characterized by broad terminal differentiation defects and variable immune abnormalities.	J Allergy Clin Immunol.
atopic dermatitis	BEX5	Expression profiling by array, count, 3 development stage, 2 disease state, 11 individual sets	GPL570	GSE32924 Samples	Nonlesional atopic dermatitis skin is characterized by broad terminal differentiation defects and variable immune abnormalities.	J Allergy Clin Immunol.
atopic dermatitis	TRAM1L1	Expression profiling by array, count, 3 development stage, 2 disease state, 11 individual sets	GPL570	GSE32924 Samples	Nonlesional atopic dermatitis skin is characterized by broad terminal differentiation defects and variable immune abnormalities.	J Allergy Clin Immunol.
atopic dermatitis	TUB	Expression profiling by array, count, 3 development stage, 2 disease state, 11 individual sets	GPL570	GSE32924 Samples	Nonlesional atopic dermatitis skin is characterized by broad terminal differentiation defects and variable immune abnormalities.	J Allergy Clin Immunol.
atopic dermatitis	NA	Expression profiling by array, count, 3 development stage, 2 disease state, 11 individual sets	GPL570	GSE32924 Samples	Nonlesional atopic dermatitis skin is characterized by broad terminal differentiation defects and variable immune abnormalities.	J Allergy Clin Immunol.
atopic dermatitis	SMARCA1	Expression profiling by array, count, 3 development stage, 2 disease state, 11 individual sets	GPL570	GSE32924 Samples	Nonlesional atopic dermatitis skin is characterized by broad terminal differentiation defects and variable immune abnormalities.	J Allergy Clin Immunol.
atopic dermatitis	RTN4	Expression profiling by array, count, 3 development stage, 2 disease state, 11 individual sets	GPL570	GSE32924 Samples	Nonlesional atopic dermatitis skin is characterized by broad terminal differentiation defects and variable immune abnormalities.	J Allergy Clin Immunol.
atopic dermatitis	NPIPL3	Expression profiling by array, count, 3 development stage, 2 disease state, 11 individual sets	GPL570	GSE32924 Samples	Nonlesional atopic dermatitis skin is characterized by broad terminal differentiation defects and variable immune abnormalities.	J Allergy Clin Immunol.
atopic dermatitis	C15ORF59	Expression profiling by array, count, 3 development stage, 2 disease state, 11 individual sets	GPL570	GSE32924 Samples	Nonlesional atopic dermatitis skin is characterized by broad terminal differentiation defects and variable immune abnormalities.	J Allergy Clin Immunol.
atopic dermatitis	TIMP3	Expression profiling by array, count, 3 development stage, 2 disease state, 11 individual sets	GPL570	GSE32924 Samples	Nonlesional atopic dermatitis skin is characterized by broad terminal differentiation defects and variable immune abnormalities.	J Allergy Clin Immunol.
atopic dermatitis	NEGR1	Expression profiling by array, count, 3 development stage, 2 disease state, 11 individual sets	GPL570	GSE32924 Samples	Nonlesional atopic dermatitis skin is characterized by broad terminal differentiation defects and variable immune abnormalities.	J Allergy Clin Immunol.
atopic dermatitis	EPB41L2	Expression profiling by array, count, 3 development stage, 2 disease state, 11 individual sets	GPL570	GSE32924 Samples	Nonlesional atopic dermatitis skin is characterized by broad terminal differentiation defects and variable immune abnormalities.	J Allergy Clin Immunol.
atopic dermatitis	AGTR1	Expression profiling by array, count, 3 development stage, 2 disease state, 11 individual sets	GPL570	GSE32924 Samples	Nonlesional atopic dermatitis skin is characterized by broad terminal differentiation defects and variable immune abnormalities.	J Allergy Clin Immunol.
atopic dermatitis	MMP27	Expression profiling by array, count, 3 development stage, 2 disease state, 11 individual sets	GPL570	GSE32924 Samples	Nonlesional atopic dermatitis skin is characterized by broad terminal differentiation defects and variable immune abnormalities.	J Allergy Clin Immunol.
atopic dermatitis	OMD	Expression profiling by array, count, 3 development stage, 2 disease state, 11 individual sets	GPL570	GSE32924 Samples	Nonlesional atopic dermatitis skin is characterized by broad terminal differentiation defects and variable immune abnormalities.	J Allergy Clin Immunol.
atopic dermatitis	LOX	Expression profiling by array, count, 3 development stage, 2 disease state, 11 individual sets	GPL570	GSE32924 Samples	Nonlesional atopic dermatitis skin is characterized by broad terminal differentiation defects and variable immune abnormalities.	J Allergy Clin Immunol.
atopic dermatitis	CCNG2	Expression profiling by array, count, 3 development stage, 2 disease state, 11 individual sets	GPL570	GSE32924 Samples	Nonlesional atopic dermatitis skin is characterized by broad terminal differentiation defects and variable immune abnormalities.	J Allergy Clin Immunol.
atopic dermatitis	ARL4C	Expression profiling by array, count, 3 development stage, 2 disease state, 11 individual sets	GPL570	GSE32924 Samples	Nonlesional atopic dermatitis skin is characterized by broad terminal differentiation defects and variable immune abnormalities.	J Allergy Clin Immunol.

atopic dermatitis	TIMP2	Expression profiling by array, count, 3 development stage, 2 disease state, 11 individual sets	GPL570	GSE32924 Samples	Nonlesional atopic dermatitis skin is characterized by broad terminal differentiation defects and variable immune abnormalities.	J Allergy Clin Immunol.
atopic dermatitis	TMEM200A	Expression profiling by array, count, 3 development stage, 2 disease state, 11 individual sets	GPL570	GSE32924 Samples	Nonlesional atopic dermatitis skin is characterized by broad terminal differentiation defects and variable immune abnormalities.	J Allergy Clin Immunol.
atopic dermatitis	PRKCB	Expression profiling by array, count, 3 development stage, 2 disease state, 11 individual sets	GPL570	GSE32924 Samples	Nonlesional atopic dermatitis skin is characterized by broad terminal differentiation defects and variable immune abnormalities.	J Allergy Clin Immunol.
atopic dermatitis	NA	Expression profiling by array, count, 3 development stage, 2 disease state, 11 individual sets	GPL570	GSE32924 Samples	Nonlesional atopic dermatitis skin is characterized by broad terminal differentiation defects and variable immune abnormalities.	J Allergy Clin Immunol.
atopic dermatitis	CMAH	Expression profiling by array, count, 3 development stage, 2 disease state, 11 individual sets	GPL570	GSE32924 Samples	Nonlesional atopic dermatitis skin is characterized by broad terminal differentiation defects and variable immune abnormalities.	J Allergy Clin Immunol.
atopic dermatitis	CCDC80	Expression profiling by array, count, 3 development stage, 2 disease state, 11 individual sets	GPL570	GSE32924 Samples	Nonlesional atopic dermatitis skin is characterized by broad terminal differentiation defects and variable immune abnormalities.	J Allergy Clin Immunol.
atopic dermatitis	OMD	Expression profiling by array, count, 3 development stage, 2 disease state, 11 individual sets	GPL570	GSE32924 Samples	Nonlesional atopic dermatitis skin is characterized by broad terminal differentiation defects and variable immune abnormalities.	J Allergy Clin Immunol.
atopic dermatitis	IGSF10	Expression profiling by array, count, 3 development stage, 2 disease state, 11 individual sets	GPL570	GSE32924 Samples	Nonlesional atopic dermatitis skin is characterized by broad terminal differentiation defects and variable immune abnormalities.	J Allergy Clin Immunol.
atopic dermatitis	ANGPTL1	Expression profiling by array, count, 3 development stage, 2 disease state, 11 individual sets	GPL570	GSE32924 Samples	Nonlesional atopic dermatitis skin is characterized by broad terminal differentiation defects and variable immune abnormalities.	J Allergy Clin Immunol.
atopic dermatitis	CP	Expression profiling by array, count, 3 development stage, 2 disease state, 11 individual sets	GPL570	GSE32924 Samples	Nonlesional atopic dermatitis skin is characterized by broad terminal differentiation defects and variable immune abnormalities.	J Allergy Clin Immunol.
atopic dermatitis	PVRL4	Expression profiling by array, count, 3 development stage, 2 disease state, 11 individual sets	GPL570	GSE32924 Samples	Nonlesional atopic dermatitis skin is characterized by broad terminal differentiation defects and variable immune abnormalities.	J Allergy Clin Immunol.
atopic dermatitis	FBXW7	Expression profiling by array, count, 3 development stage, 2 disease state, 11 individual sets	GPL570	GSE32924 Samples	Nonlesional atopic dermatitis skin is characterized by broad terminal differentiation defects and variable immune abnormalities.	J Allergy Clin Immunol.
atopic dermatitis	MCOLN3	Expression profiling by array, count, 3 development stage, 2 disease state, 11 individual sets	GPL570	GSE32924 Samples	Nonlesional atopic dermatitis skin is characterized by broad terminal differentiation defects and variable immune abnormalities.	J Allergy Clin Immunol.
atopic dermatitis	AGPAT9	Expression profiling by array, count, 3 development stage, 2 disease state, 11 individual sets	GPL570	GSE32924 Samples	Nonlesional atopic dermatitis skin is characterized by broad terminal differentiation defects and variable immune abnormalities.	J Allergy Clin Immunol.
atopic dermatitis	CX3CR1	Expression profiling by array, count, 3 development stage, 2 disease state, 11 individual sets	GPL570	GSE32924 Samples	Nonlesional atopic dermatitis skin is characterized by broad terminal differentiation defects and variable immune abnormalities.	J Allergy Clin Immunol.
atopic dermatitis	CYP39A1	Expression profiling by array, count, 3 development stage, 2 disease state, 11 individual sets	GPL570	GSE32924 Samples	Nonlesional atopic dermatitis skin is characterized by broad terminal differentiation defects and variable immune abnormalities.	J Allergy Clin Immunol.
atopic dermatitis	NA	Expression profiling by array, count, 3 development stage, 2 disease state, 11 individual sets	GPL570	GSE32924 Samples	Nonlesional atopic dermatitis skin is characterized by broad terminal differentiation defects and variable immune abnormalities.	J Allergy Clin Immunol.
atopic dermatitis	IL7	Expression profiling by array, count, 3 development stage, 2 disease state, 11 individual sets	GPL570	GSE32924 Samples	Nonlesional atopic dermatitis skin is characterized by broad terminal differentiation defects and variable immune abnormalities.	J Allergy Clin Immunol.
atopic dermatitis	PRKCB	Expression profiling by array, count, 3 development stage, 2 disease state, 11 individual sets	GPL570	GSE32924 Samples	Nonlesional atopic dermatitis skin is characterized by broad terminal differentiation defects and variable immune abnormalities.	J Allergy Clin Immunol.
atopic dermatitis	OTUD1	Expression profiling by array, count, 3 development stage, 2 disease state, 11 individual sets	GPL570	GSE32924 Samples	Nonlesional atopic dermatitis skin is characterized by broad terminal differentiation defects and variable immune abnormalities.	J Allergy Clin Immunol.
atopic dermatitis	SCIN	Expression profiling by array, count, 3 development stage, 2 disease state, 11 individual sets	GPL570	GSE32924 Samples	Nonlesional atopic dermatitis skin is characterized by broad terminal differentiation defects and variable immune abnormalities.	J Allergy Clin Immunol.
atopic dermatitis	FLRT3	Expression profiling by array, count, 3 development stage, 2 disease state, 11 individual sets	GPL570	GSE32924 Samples	Nonlesional atopic dermatitis skin is characterized by broad terminal differentiation defects and variable immune abnormalities.	J Allergy Clin Immunol.
atopic dermatitis	SLC16A10	Expression profiling by array, count, 3 development stage, 2 disease state, 11 individual sets	GPL570	GSE32924 Samples	Nonlesional atopic dermatitis skin is characterized by broad terminal differentiation defects and variable immune abnormalities.	J Allergy Clin Immunol.
atopic dermatitis	PRRX1	Expression profiling by array, count, 3 development stage, 2 disease state, 11 individual sets	GPL570	GSE32924 Samples	Nonlesional atopic dermatitis skin is characterized by broad terminal differentiation defects and variable immune abnormalities.	J Allergy Clin Immunol.
atopic dermatitis	PICALM	Expression profiling by array, count, 3 development stage, 2 disease state, 11 individual sets	GPL570	GSE32924 Samples	Nonlesional atopic dermatitis skin is characterized by broad terminal differentiation defects and variable immune abnormalities.	J Allergy Clin Immunol.
atopic dermatitis	STS	Expression profiling by array, count, 3 development stage, 2 disease state, 11 individual sets	GPL570	GSE32924 Samples	Nonlesional atopic dermatitis skin is characterized by broad terminal differentiation defects and variable immune abnormalities.	J Allergy Clin Immunol.
atopic dermatitis	FBN1	Expression profiling by array, count, 3 development stage, 2 disease state, 11 individual sets	GPL570	GSE32924 Samples	Nonlesional atopic dermatitis skin is characterized by broad terminal differentiation defects and variable immune abnormalities.	J Allergy Clin Immunol.
atopic dermatitis	C1S	Expression profiling by array, count, 3 development stage, 2 disease state, 11 individual sets	GPL570	GSE32924 Samples	Nonlesional atopic dermatitis skin is characterized by broad terminal differentiation defects and variable immune abnormalities.	J Allergy Clin Immunol.
atopic dermatitis	PDGFRL	Expression profiling by array, count, 3 development stage, 2 disease state, 11 individual sets	GPL570	GSE32924 Samples	Nonlesional atopic dermatitis skin is characterized by broad terminal differentiation defects and variable immune abnormalities.	J Allergy Clin Immunol.
atopic dermatitis	IL1F7	Expression profiling by array, count, 3 development stage, 2 disease state, 11 individual sets	GPL570	GSE32924 Samples	Nonlesional atopic dermatitis skin is characterized by broad terminal differentiation defects and variable immune abnormalities.	J Allergy Clin Immunol.
atopic dermatitis	PSORS1C2	Expression profiling by array, count, 3 development stage, 2 disease state, 11 individual sets	GPL570	GSE32924 Samples	Nonlesional atopic dermatitis skin is characterized by broad terminal differentiation defects and variable immune abnormalities.	J Allergy Clin Immunol.
atopic dermatitis	LOC100130476	Expression profiling by array, count, 3 development stage, 2 disease state, 11 individual sets	GPL570	GSE32924 Samples	Nonlesional atopic dermatitis skin is characterized by broad terminal differentiation defects and variable immune abnormalities.	J Allergy Clin Immunol.
atopic dermatitis	GREM1	Expression profiling by array, count, 3 development stage, 2 disease state, 11 individual sets	GPL570	GSE32924 Samples	Nonlesional atopic dermatitis skin is characterized by broad terminal differentiation defects and variable immune abnormalities.	J Allergy Clin Immunol.
atopic dermatitis	CORIN	Expression profiling by array, count, 3 development stage, 2 disease state, 11 individual sets	GPL570	GSE32924 Samples	Nonlesional atopic dermatitis skin is characterized by broad terminal differentiation defects and variable immune abnormalities.	J Allergy Clin Immunol.
atopic dermatitis	C7ORF64	Expression profiling by array, count, 3 development stage, 2 disease state, 11 individual sets	GPL570	GSE32924 Samples	Nonlesional atopic dermatitis skin is characterized by broad terminal differentiation defects and variable immune abnormalities.	J Allergy Clin Immunol.
atopic dermatitis	C17ORF48	Expression profiling by array, count, 3 development stage, 2 disease state, 11 individual sets	GPL570	GSE32924 Samples	Nonlesional atopic dermatitis skin is characterized by broad terminal differentiation defects and variable immune abnormalities.	J Allergy Clin Immunol.
atopic dermatitis	ZNF134	Expression profiling by array, count, 3 development stage, 2 disease state, 11 individual sets	GPL570	GSE32924 Samples	Nonlesional atopic dermatitis skin is characterized by broad terminal differentiation defects and variable immune abnormalities.	J Allergy Clin Immunol.
atopic dermatitis	HSF2	Expression profiling by array, count, 3 development stage, 2 disease state, 11 individual sets	GPL570	GSE32924 Samples	Nonlesional atopic dermatitis skin is characterized by broad terminal differentiation defects and variable immune abnormalities.	J Allergy Clin Immunol.
atopic dermatitis	HDGFRP3	Expression profiling by array, count, 3 development stage, 2 disease state, 11 individual sets	GPL570	GSE32924 Samples	Nonlesional atopic dermatitis skin is characterized by broad terminal differentiation defects and variable immune abnormalities.	J Allergy Clin Immunol.
atopic dermatitis	GLT8D2	Expression profiling by array, count, 3 development stage, 2 disease state, 11 individual sets	GPL570	GSE32924 Samples	Nonlesional atopic dermatitis skin is characterized by broad terminal differentiation defects and variable immune abnormalities.	J Allergy Clin Immunol.
atopic dermatitis	SCCPDH	Expression profiling by array, count, 3 development stage, 2 disease state, 11 individual sets	GPL570	GSE32924 Samples	Nonlesional atopic dermatitis skin is characterized by broad terminal differentiation defects and variable immune abnormalities.	J Allergy Clin Immunol.

atopic dermatitis	LPIN2	Expression profiling by array, count, 3 development stage, 2 disease state, 11 individual sets	GPL570	GSE32924 Samples	Nonlesional atopic dermatitis skin is characterized by broad terminal differentiation defects and variable immune abnormalities.	J Allergy Clin Immunol.
atopic dermatitis	NA	Expression profiling by array, count, 3 development stage, 2 disease state, 11 individual sets	GPL570	GSE32924 Samples	Nonlesional atopic dermatitis skin is characterized by broad terminal differentiation defects and variable immune abnormalities.	J Allergy Clin Immunol.
atopic dermatitis	IGFBP5	Expression profiling by array, count, 3 development stage, 2 disease state, 11 individual sets	GPL570	GSE32924 Samples	Nonlesional atopic dermatitis skin is characterized by broad terminal differentiation defects and variable immune abnormalities.	J Allergy Clin Immunol.
atopic dermatitis	IGFBP5	Expression profiling by array, count, 3 development stage, 2 disease state, 11 individual sets	GPL570	GSE32924 Samples	Nonlesional atopic dermatitis skin is characterized by broad terminal differentiation defects and variable immune abnormalities.	J Allergy Clin Immunol.
atopic dermatitis	FAM38B	Expression profiling by array, count, 3 development stage, 2 disease state, 11 individual sets	GPL570	GSE32924 Samples	Nonlesional atopic dermatitis skin is characterized by broad terminal differentiation defects and variable immune abnormalities.	J Allergy Clin Immunol.
atopic dermatitis	NA	Expression profiling by array, count, 3 development stage, 2 disease state, 11 individual sets	GPL570	GSE32924 Samples	Nonlesional atopic dermatitis skin is characterized by broad terminal differentiation defects and variable immune abnormalities.	J Allergy Clin Immunol.
atopic dermatitis	PKP4	Expression profiling by array, count, 3 development stage, 2 disease state, 11 individual sets	GPL570	GSE32924 Samples	Nonlesional atopic dermatitis skin is characterized by broad terminal differentiation defects and variable immune abnormalities.	J Allergy Clin Immunol.
atopic dermatitis	IGF2R	Expression profiling by array, count, 3 development stage, 2 disease state, 11 individual sets	GPL570	GSE32924 Samples	Nonlesional atopic dermatitis skin is characterized by broad terminal differentiation defects and variable immune abnormalities.	J Allergy Clin Immunol.
atopic dermatitis	MAX	Expression profiling by array, count, 3 development stage, 2 disease state, 11 individual sets	GPL570	GSE32924 Samples	Nonlesional atopic dermatitis skin is characterized by broad terminal differentiation defects and variable immune abnormalities.	J Allergy Clin Immunol.
atopic dermatitis	NA	Expression profiling by array, count, 3 development stage, 2 disease state, 11 individual sets	GPL570	GSE32924 Samples	Nonlesional atopic dermatitis skin is characterized by broad terminal differentiation defects and variable immune abnormalities.	J Allergy Clin Immunol.
atopic dermatitis	USP46	Expression profiling by array, count, 3 development stage, 2 disease state, 11 individual sets	GPL570	GSE32924 Samples	Nonlesional atopic dermatitis skin is characterized by broad terminal differentiation defects and variable immune abnormalities.	J Allergy Clin Immunol.
atopic dermatitis	FOXD1	Expression profiling by array, count, 3 development stage, 2 disease state, 11 individual sets	GPL570	GSE32924 Samples	Nonlesional atopic dermatitis skin is characterized by broad terminal differentiation defects and variable immune abnormalities.	J Allergy Clin Immunol.
atopic dermatitis	NA	Expression profiling by array, count, 3 development stage, 2 disease state, 11 individual sets	GPL570	GSE32924 Samples	Nonlesional atopic dermatitis skin is characterized by broad terminal differentiation defects and variable immune abnormalities.	J Allergy Clin Immunol.
atopic dermatitis	NA	Expression profiling by array, count, 3 development stage, 2 disease state, 11 individual sets	GPL570	GSE32924 Samples	Nonlesional atopic dermatitis skin is characterized by broad terminal differentiation defects and variable immune abnormalities.	J Allergy Clin Immunol.
atopic dermatitis	MCOLN3	Expression profiling by array, count, 3 development stage, 2 disease state, 11 individual sets	GPL570	GSE32924 Samples	Nonlesional atopic dermatitis skin is characterized by broad terminal differentiation defects and variable immune abnormalities.	J Allergy Clin Immunol.
atopic dermatitis	TMEM159	Expression profiling by array, count, 3 development stage, 2 disease state, 11 individual sets	GPL570	GSE32924 Samples	Nonlesional atopic dermatitis skin is characterized by broad terminal differentiation defects and variable immune abnormalities.	J Allergy Clin Immunol.
atopic dermatitis	SNAP23	Expression profiling by array, count, 3 development stage, 2 disease state, 11 individual sets	GPL570	GSE32924 Samples	Nonlesional atopic dermatitis skin is characterized by broad terminal differentiation defects and variable immune abnormalities.	J Allergy Clin Immunol.
atopic dermatitis	FLI1	Expression profiling by array, count, 3 development stage, 2 disease state, 11 individual sets	GPL570	GSE32924 Samples	Nonlesional atopic dermatitis skin is characterized by broad terminal differentiation defects and variable immune abnormalities.	J Allergy Clin Immunol.
atopic dermatitis	ANP32A	Expression profiling by array, count, 3 development stage, 2 disease state, 11 individual sets	GPL570	GSE32924 Samples	Nonlesional atopic dermatitis skin is characterized by broad terminal differentiation defects and variable immune abnormalities.	J Allergy Clin Immunol.
atopic dermatitis	ARNT	Expression profiling by array, count, 3 development stage, 2 disease state, 11 individual sets	GPL570	GSE32924 Samples	Nonlesional atopic dermatitis skin is characterized by broad terminal differentiation defects and variable immune abnormalities.	J Allergy Clin Immunol.
atopic dermatitis	ANKFY1	Expression profiling by array, count, 3 development stage, 2 disease state, 11 individual sets	GPL570	GSE32924 Samples	Nonlesional atopic dermatitis skin is characterized by broad terminal differentiation defects and variable immune abnormalities.	J Allergy Clin Immunol.
atopic dermatitis	XPO7	Expression profiling by array, count, 3 development stage, 2 disease state, 11 individual sets	GPL570	GSE32924 Samples	Nonlesional atopic dermatitis skin is characterized by broad terminal differentiation defects and variable immune abnormalities.	J Allergy Clin Immunol.
atopic dermatitis	NA	Expression profiling by array, count, 3 development stage, 2 disease state, 11 individual sets	GPL570	GSE32924 Samples	Nonlesional atopic dermatitis skin is characterized by broad terminal differentiation defects and variable immune abnormalities.	J Allergy Clin Immunol.
atopic dermatitis	NA	Expression profiling by array, count, 3 development stage, 2 disease state, 11 individual sets	GPL570	GSE32924 Samples	Nonlesional atopic dermatitis skin is characterized by broad terminal differentiation defects and variable immune abnormalities.	J Allergy Clin Immunol.
atopic dermatitis	FAM115A	Expression profiling by array, count, 3 development stage, 2 disease state, 11 individual sets	GPL570	GSE32924 Samples	Nonlesional atopic dermatitis skin is characterized by broad terminal differentiation defects and variable immune abnormalities.	J Allergy Clin Immunol.
atopic dermatitis	IGFBP5	Expression profiling by array, count, 3 development stage, 2 disease state, 11 individual sets	GPL570	GSE32924 Samples	Nonlesional atopic dermatitis skin is characterized by broad terminal differentiation defects and variable immune abnormalities.	J Allergy Clin Immunol.
atopic dermatitis	KDELR3	Expression profiling by array, count, 3 development stage, 2 disease state, 11 individual sets	GPL570	GSE32924 Samples	Nonlesional atopic dermatitis skin is characterized by broad terminal differentiation defects and variable immune abnormalities.	J Allergy Clin Immunol.
atopic dermatitis	KLF7	Expression profiling by array, count, 3 development stage, 2 disease state, 11 individual sets	GPL570	GSE32924 Samples	Nonlesional atopic dermatitis skin is characterized by broad terminal differentiation defects and variable immune abnormalities.	J Allergy Clin Immunol.
atopic dermatitis	NA	Expression profiling by array, count, 3 development stage, 2 disease state, 11 individual sets	GPL570	GSE32924 Samples	Nonlesional atopic dermatitis skin is characterized by broad terminal differentiation defects and variable immune abnormalities.	J Allergy Clin Immunol.
atopic dermatitis	ERG	Expression profiling by array, count, 3 development stage, 2 disease state, 11 individual sets	GPL570	GSE32924 Samples	Nonlesional atopic dermatitis skin is characterized by broad terminal differentiation defects and variable immune abnormalities.	J Allergy Clin Immunol.
atopic dermatitis	SCIN	Expression profiling by array, count, 3 development stage, 2 disease state, 11 individual sets	GPL570	GSE32924 Samples	Nonlesional atopic dermatitis skin is characterized by broad terminal differentiation defects and variable immune abnormalities.	J Allergy Clin Immunol.
atopic dermatitis	KAZALD1	Expression profiling by array, count, 3 development stage, 2 disease state, 11 individual sets	GPL570	GSE32924 Samples	Nonlesional atopic dermatitis skin is characterized by broad terminal differentiation defects and variable immune abnormalities.	J Allergy Clin Immunol.
atopic dermatitis	NA	Expression profiling by array, count, 3 development stage, 2 disease state, 11 individual sets	GPL570	GSE32924 Samples	Nonlesional atopic dermatitis skin is characterized by broad terminal differentiation defects and variable immune abnormalities.	J Allergy Clin Immunol.
atopic dermatitis	LPCAT2	Expression profiling by array, count, 3 development stage, 2 disease state, 11 individual sets	GPL570	GSE32924 Samples	Nonlesional atopic dermatitis skin is characterized by broad terminal differentiation defects and variable immune abnormalities.	J Allergy Clin Immunol.
atopic dermatitis	NA	Expression profiling by array, count, 3 development stage, 2 disease state, 11 individual sets	GPL570	GSE32924 Samples	Nonlesional atopic dermatitis skin is characterized by broad terminal differentiation defects and variable immune abnormalities.	J Allergy Clin Immunol.
atopic dermatitis	NA	Expression profiling by array, count, 3 development stage, 2 disease state, 11 individual sets	GPL570	GSE32924 Samples	Nonlesional atopic dermatitis skin is characterized by broad terminal differentiation defects and variable immune abnormalities.	J Allergy Clin Immunol.
atopic dermatitis	PRSS12	Expression profiling by array, count, 3 development stage, 2 disease state, 11 individual sets	GPL570	GSE32924 Samples	Nonlesional atopic dermatitis skin is characterized by broad terminal differentiation defects and variable immune abnormalities.	J Allergy Clin Immunol.
atopic dermatitis	GAN	Expression profiling by array, count, 3 development stage, 2 disease state, 11 individual sets	GPL570	GSE32924 Samples	Nonlesional atopic dermatitis skin is characterized by broad terminal differentiation defects and variable immune abnormalities.	J Allergy Clin Immunol.
atopic dermatitis	ESYT3	Expression profiling by array, count, 3 development stage, 2 disease state, 11 individual sets	GPL570	GSE32924 Samples	Nonlesional atopic dermatitis skin is characterized by broad terminal differentiation defects and variable immune abnormalities.	J Allergy Clin Immunol.
atopic dermatitis	PTPN21	Expression profiling by array, count, 3 development stage, 2 disease state, 11 individual sets	GPL570	GSE32924 Samples	Nonlesional atopic dermatitis skin is characterized by broad terminal differentiation defects and variable immune abnormalities.	J Allergy Clin Immunol.
atopic dermatitis	MYH14	Expression profiling by array, count, 3 development stage, 2 disease state, 11 individual sets	GPL570	GSE32924 Samples	Nonlesional atopic dermatitis skin is characterized by broad terminal differentiation defects and variable immune abnormalities.	J Allergy Clin Immunol.

atopic dermatitis	MYH14	Expression profiling by array, count, 3 development stage, 2 disease state, 11 individual sets	GPL570	GSE32924 Samples	Nonlesional atopic dermatitis skin is characterized by broad terminal differentiation defects and variable immune abnormalities.	J Allergy Clin Immunol.
atopic dermatitis	EFHC2	Expression profiling by array, count, 3 development stage, 2 disease state, 11 individual sets	GPL570	GSE32924 Samples	Nonlesional atopic dermatitis skin is characterized by broad terminal differentiation defects and variable immune abnormalities.	J Allergy Clin Immunol.
atopic dermatitis	WNT4	Expression profiling by array, count, 3 development stage, 2 disease state, 11 individual sets	GPL570	GSE32924 Samples	Nonlesional atopic dermatitis skin is characterized by broad terminal differentiation defects and variable immune abnormalities.	J Allergy Clin Immunol.
atopic dermatitis	NA	Expression profiling by array, count, 3 development stage, 2 disease state, 11 individual sets	GPL570	GSE32924 Samples	Nonlesional atopic dermatitis skin is characterized by broad terminal differentiation defects and variable immune abnormalities.	J Allergy Clin Immunol.
atopic dermatitis	PLEKHA8	Expression profiling by array, count, 3 development stage, 2 disease state, 11 individual sets	GPL570	GSE32924 Samples	Nonlesional atopic dermatitis skin is characterized by broad terminal differentiation defects and variable immune abnormalities.	J Allergy Clin Immunol.
atopic dermatitis	NA	Expression profiling by array, count, 3 development stage, 2 disease state, 11 individual sets	GPL570	GSE32924 Samples	Nonlesional atopic dermatitis skin is characterized by broad terminal differentiation defects and variable immune abnormalities.	J Allergy Clin Immunol.
atopic dermatitis	CORIN	Expression profiling by array, count, 3 development stage, 2 disease state, 11 individual sets	GPL570	GSE32924 Samples	Nonlesional atopic dermatitis skin is characterized by broad terminal differentiation defects and variable immune abnormalities.	J Allergy Clin Immunol.
atopic dermatitis	NA	Expression profiling by array, count, 3 development stage, 2 disease state, 11 individual sets	GPL570	GSE32924 Samples	Nonlesional atopic dermatitis skin is characterized by broad terminal differentiation defects and variable immune abnormalities.	J Allergy Clin Immunol.
atopic dermatitis	ABCA9	Expression profiling by array, count, 3 development stage, 2 disease state, 11 individual sets	GPL570	GSE32924 Samples	Nonlesional atopic dermatitis skin is characterized by broad terminal differentiation defects and variable immune abnormalities.	J Allergy Clin Immunol.
atopic dermatitis	FLJ32063	Expression profiling by array, count, 3 development stage, 2 disease state, 11 individual sets	GPL570	GSE32924 Samples	Nonlesional atopic dermatitis skin is characterized by broad terminal differentiation defects and variable immune abnormalities.	J Allergy Clin Immunol.
atopic dermatitis	CNKSR2	Expression profiling by array, count, 3 development stage, 2 disease state, 11 individual sets	GPL570	GSE32924 Samples	Nonlesional atopic dermatitis skin is characterized by broad terminal differentiation defects and variable immune abnormalities.	J Allergy Clin Immunol.
atopic dermatitis	NA	Expression profiling by array, count, 3 development stage, 2 disease state, 11 individual sets	GPL570	GSE32924 Samples	Nonlesional atopic dermatitis skin is characterized by broad terminal differentiation defects and variable immune abnormalities.	J Allergy Clin Immunol.
atopic dermatitis	FNDC1	Expression profiling by array, count, 3 development stage, 2 disease state, 11 individual sets	GPL570	GSE32924 Samples	Nonlesional atopic dermatitis skin is characterized by broad terminal differentiation defects and variable immune abnormalities.	J Allergy Clin Immunol.
atopic dermatitis	PCDHB10	Expression profiling by array, count, 3 development stage, 2 disease state, 11 individual sets	GPL570	GSE32924 Samples	Nonlesional atopic dermatitis skin is characterized by broad terminal differentiation defects and variable immune abnormalities.	J Allergy Clin Immunol.
atopic dermatitis	MPDZ	Expression profiling by array, count, 3 development stage, 2 disease state, 11 individual sets	GPL570	GSE32924 Samples	Nonlesional atopic dermatitis skin is characterized by broad terminal differentiation defects and variable immune abnormalities.	J Allergy Clin Immunol.
atopic dermatitis	ABHD2	Expression profiling by array, count, 3 development stage, 2 disease state, 11 individual sets	GPL570	GSE32924 Samples	Nonlesional atopic dermatitis skin is characterized by broad terminal differentiation defects and variable immune abnormalities.	J Allergy Clin Immunol.
atopic dermatitis	LSG1	Expression profiling by array, count, 3 development stage, 2 disease state, 11 individual sets	GPL570	GSE32924 Samples	Nonlesional atopic dermatitis skin is characterized by broad terminal differentiation defects and variable immune abnormalities.	J Allergy Clin Immunol.
atopic dermatitis	RIF1	Expression profiling by array, count, 3 development stage, 2 disease state, 11 individual sets	GPL570	GSE32924 Samples	Nonlesional atopic dermatitis skin is characterized by broad terminal differentiation defects and variable immune abnormalities.	J Allergy Clin Immunol.
atopic dermatitis	SGSM2	Expression profiling by array, count, 3 development stage, 2 disease state, 11 individual sets	GPL570	GSE32924 Samples	Nonlesional atopic dermatitis skin is characterized by broad terminal differentiation defects and variable immune abnormalities.	J Allergy Clin Immunol.
atopic dermatitis	DDX17	Expression profiling by array, count, 3 development stage, 2 disease state, 11 individual sets	GPL570	GSE32924 Samples	Nonlesional atopic dermatitis skin is characterized by broad terminal differentiation defects and variable immune abnormalities.	J Allergy Clin Immunol.
atopic dermatitis	CHD9	Expression profiling by array, count, 3 development stage, 2 disease state, 11 individual sets	GPL570	GSE32924 Samples	Nonlesional atopic dermatitis skin is characterized by broad terminal differentiation defects and variable immune abnormalities.	J Allergy Clin Immunol.
atopic dermatitis	NA	Expression profiling by array, count, 3 development stage, 2 disease state, 11 individual sets	GPL570	GSE32924 Samples	Nonlesional atopic dermatitis skin is characterized by broad terminal differentiation defects and variable immune abnormalities.	J Allergy Clin Immunol.
atopic dermatitis	MALAT1	Expression profiling by array, count, 3 development stage, 2 disease state, 11 individual sets	GPL570	GSE32924 Samples	Nonlesional atopic dermatitis skin is characterized by broad terminal differentiation defects and variable immune abnormalities.	J Allergy Clin Immunol.
atopic dermatitis	COCH	Expression profiling by array, count, 3 development stage, 2 disease state, 11 individual sets	GPL570	GSE32924 Samples	Nonlesional atopic dermatitis skin is characterized by broad terminal differentiation defects and variable immune abnormalities.	J Allergy Clin Immunol.
atopic dermatitis	ZNF513	Expression profiling by array, count, 3 development stage, 2 disease state, 11 individual sets	GPL570	GSE32924 Samples	Nonlesional atopic dermatitis skin is characterized by broad terminal differentiation defects and variable immune abnormalities.	J Allergy Clin Immunol.
atopic dermatitis	RHBDD3	Expression profiling by array, count, 3 development stage, 2 disease state, 11 individual sets	GPL570	GSE32924 Samples	Nonlesional atopic dermatitis skin is characterized by broad terminal differentiation defects and variable immune abnormalities.	J Allergy Clin Immunol.
atopic dermatitis	TMEM161A	Expression profiling by array, count, 3 development stage, 2 disease state, 11 individual sets	GPL570	GSE32924 Samples	Nonlesional atopic dermatitis skin is characterized by broad terminal differentiation defects and variable immune abnormalities.	J Allergy Clin Immunol.
atopic dermatitis	TMUB1	Expression profiling by array, count, 3 development stage, 2 disease state, 11 individual sets	GPL570	GSE32924 Samples	Nonlesional atopic dermatitis skin is characterized by broad terminal differentiation defects and variable immune abnormalities.	J Allergy Clin Immunol.
atopic dermatitis	IPO13	Expression profiling by array, count, 3 development stage, 2 disease state, 11 individual sets	GPL570	GSE32924 Samples	Nonlesional atopic dermatitis skin is characterized by broad terminal differentiation defects and variable immune abnormalities.	J Allergy Clin Immunol.
atopic dermatitis	GRK6	Expression profiling by array, count, 3 development stage, 2 disease state, 11 individual sets	GPL570	GSE32924 Samples	Nonlesional atopic dermatitis skin is characterized by broad terminal differentiation defects and variable immune abnormalities.	J Allergy Clin Immunol.
atopic dermatitis	VWA1	Expression profiling by array, count, 3 development stage, 2 disease state, 11 individual sets	GPL570	GSE32924 Samples	Nonlesional atopic dermatitis skin is characterized by broad terminal differentiation defects and variable immune abnormalities.	J Allergy Clin Immunol.
atopic dermatitis	CORO1B	Expression profiling by array, count, 3 development stage, 2 disease state, 11 individual sets	GPL570	GSE32924 Samples	Nonlesional atopic dermatitis skin is characterized by broad terminal differentiation defects and variable immune abnormalities.	J Allergy Clin Immunol.
atopic dermatitis	MON1A	Expression profiling by array, count, 3 development stage, 2 disease state, 11 individual sets	GPL570	GSE32924 Samples	Nonlesional atopic dermatitis skin is characterized by broad terminal differentiation defects and variable immune abnormalities.	J Allergy Clin Immunol.
atopic dermatitis	NDOR1	Expression profiling by array, count, 3 development stage, 2 disease state, 11 individual sets	GPL570	GSE32924 Samples	Nonlesional atopic dermatitis skin is characterized by broad terminal differentiation defects and variable immune abnormalities.	J Allergy Clin Immunol.
atopic dermatitis	RASSF7	Expression profiling by array, count, 3 development stage, 2 disease state, 11 individual sets	GPL570	GSE32924 Samples	Nonlesional atopic dermatitis skin is characterized by broad terminal differentiation defects and variable immune abnormalities.	J Allergy Clin Immunol.
atopic dermatitis	SLC37A4	Expression profiling by array, count, 3 development stage, 2 disease state, 11 individual sets	GPL570	GSE32924 Samples	Nonlesional atopic dermatitis skin is characterized by broad terminal differentiation defects and variable immune abnormalities.	J Allergy Clin Immunol.
atopic dermatitis	PACSIN3	Expression profiling by array, count, 3 development stage, 2 disease state, 11 individual sets	GPL570	GSE32924 Samples	Nonlesional atopic dermatitis skin is characterized by broad terminal differentiation defects and variable immune abnormalities.	J Allergy Clin Immunol.
atopic dermatitis	NA	Expression profiling by array, count, 3 development stage, 2 disease state, 11 individual sets	GPL570	GSE32924 Samples	Nonlesional atopic dermatitis skin is characterized by broad terminal differentiation defects and variable immune abnormalities.	J Allergy Clin Immunol.
atopic dermatitis	APEX2	Expression profiling by array, count, 3 development stage, 2 disease state, 11 individual sets	GPL570	GSE32924 Samples	Nonlesional atopic dermatitis skin is characterized by broad terminal differentiation defects and variable immune abnormalities.	J Allergy Clin Immunol.
atopic dermatitis	FDXR	Expression profiling by array, count, 3 development stage, 2 disease state, 11 individual sets	GPL570	GSE32924 Samples	Nonlesional atopic dermatitis skin is characterized by broad terminal differentiation defects and variable immune abnormalities.	J Allergy Clin Immunol.
atopic dermatitis	PPAN	Expression profiling by array, count, 3 development stage, 2 disease state, 11 individual sets	GPL570	GSE32924 Samples	Nonlesional atopic dermatitis skin is characterized by broad terminal differentiation defects and variable immune abnormalities.	J Allergy Clin Immunol.

atopic dermatitis	WDR4	Expression profiling by array, count, 3 development stage, 2 disease state, 11 individual sets	GPL570	GSE32924 Samples	Nonlesional atopic dermatitis skin is characterized by broad terminal differentiation defects and variable immune abnormalities.	J Allergy Clin Immunol.
atopic dermatitis	ENDOG	Expression profiling by array, count, 3 development stage, 2 disease state, 11 individual sets	GPL570	GSE32924 Samples	Nonlesional atopic dermatitis skin is characterized by broad terminal differentiation defects and variable immune abnormalities.	J Allergy Clin Immunol.
atopic dermatitis	PUSL1	Expression profiling by array, count, 3 development stage, 2 disease state, 11 individual sets	GPL570	GSE32924 Samples	Nonlesional atopic dermatitis skin is characterized by broad terminal differentiation defects and variable immune abnormalities.	J Allergy Clin Immunol.
atopic dermatitis	SMPD2	Expression profiling by array, count, 3 development stage, 2 disease state, 11 individual sets	GPL570	GSE32924 Samples	Nonlesional atopic dermatitis skin is characterized by broad terminal differentiation defects and variable immune abnormalities.	J Allergy Clin Immunol.
atopic dermatitis	NA	Expression profiling by array, count, 3 development stage, 2 disease state, 11 individual sets	GPL570	GSE32924 Samples	Nonlesional atopic dermatitis skin is characterized by broad terminal differentiation defects and variable immune abnormalities.	J Allergy Clin Immunol.
atopic dermatitis	ICAM3	Expression profiling by array, count, 3 development stage, 2 disease state, 11 individual sets	GPL570	GSE32924 Samples	Nonlesional atopic dermatitis skin is characterized by broad terminal differentiation defects and variable immune abnormalities.	J Allergy Clin Immunol.
atopic dermatitis	GPR172A	Expression profiling by array, count, 3 development stage, 2 disease state, 11 individual sets	GPL570	GSE32924 Samples	Nonlesional atopic dermatitis skin is characterized by broad terminal differentiation defects and variable immune abnormalities.	J Allergy Clin Immunol.
atopic dermatitis	OGFOD2	Expression profiling by array, count, 3 development stage, 2 disease state, 11 individual sets	GPL570	GSE32924 Samples	Nonlesional atopic dermatitis skin is characterized by broad terminal differentiation defects and variable immune abnormalities.	J Allergy Clin Immunol.
atopic dermatitis	UBE2S	Expression profiling by array, count, 3 development stage, 2 disease state, 11 individual sets	GPL570	GSE32924 Samples	Nonlesional atopic dermatitis skin is characterized by broad terminal differentiation defects and variable immune abnormalities.	J Allergy Clin Immunol.
atopic dermatitis	MED16	Expression profiling by array, count, 3 development stage, 2 disease state, 11 individual sets	GPL570	GSE32924 Samples	Nonlesional atopic dermatitis skin is characterized by broad terminal differentiation defects and variable immune abnormalities.	J Allergy Clin Immunol.
atopic dermatitis	RAB4B	Expression profiling by array, count, 3 development stage, 2 disease state, 11 individual sets	GPL570	GSE32924 Samples	Nonlesional atopic dermatitis skin is characterized by broad terminal differentiation defects and variable immune abnormalities.	J Allergy Clin Immunol.
atopic dermatitis	TELO2	Expression profiling by array, count, 3 development stage, 2 disease state, 11 individual sets	GPL570	GSE32924 Samples	Nonlesional atopic dermatitis skin is characterized by broad terminal differentiation defects and variable immune abnormalities.	J Allergy Clin Immunol.
atopic dermatitis	LMF1	Expression profiling by array, count, 3 development stage, 2 disease state, 11 individual sets	GPL570	GSE32924 Samples	Nonlesional atopic dermatitis skin is characterized by broad terminal differentiation defects and variable immune abnormalities.	J Allergy Clin Immunol.
atopic dermatitis	RNPEPL1	Expression profiling by array, count, 3 development stage, 2 disease state, 11 individual sets	GPL570	GSE32924 Samples	Nonlesional atopic dermatitis skin is characterized by broad terminal differentiation defects and variable immune abnormalities.	J Allergy Clin Immunol.
atopic dermatitis	ZBTB17	Expression profiling by array, count, 3 development stage, 2 disease state, 11 individual sets	GPL570	GSE32924 Samples	Nonlesional atopic dermatitis skin is characterized by broad terminal differentiation defects and variable immune abnormalities.	J Allergy Clin Immunol.
atopic dermatitis	POLD1	Expression profiling by array, count, 3 development stage, 2 disease state, 11 individual sets	GPL570	GSE32924 Samples	Nonlesional atopic dermatitis skin is characterized by broad terminal differentiation defects and variable immune abnormalities.	J Allergy Clin Immunol.
atopic dermatitis	RAB4B	Expression profiling by array, count, 3 development stage, 2 disease state, 11 individual sets	GPL570	GSE32924 Samples	Nonlesional atopic dermatitis skin is characterized by broad terminal differentiation defects and variable immune abnormalities.	J Allergy Clin Immunol.
atopic dermatitis	DGCR14	Expression profiling by array, count, 3 development stage, 2 disease state, 11 individual sets	GPL570	GSE32924 Samples	Nonlesional atopic dermatitis skin is characterized by broad terminal differentiation defects and variable immune abnormalities.	J Allergy Clin Immunol.
atopic dermatitis	C17ORF56	Expression profiling by array, count, 3 development stage, 2 disease state, 11 individual sets	GPL570	GSE32924 Samples	Nonlesional atopic dermatitis skin is characterized by broad terminal differentiation defects and variable immune abnormalities.	J Allergy Clin Immunol.
atopic dermatitis	ATG4D	Expression profiling by array, count, 3 development stage, 2 disease state, 11 individual sets	GPL570	GSE32924 Samples	Nonlesional atopic dermatitis skin is characterized by broad terminal differentiation defects and variable immune abnormalities.	J Allergy Clin Immunol.
atopic dermatitis	CYHR1	Expression profiling by array, count, 3 development stage, 2 disease state, 11 individual sets	GPL570	GSE32924 Samples	Nonlesional atopic dermatitis skin is characterized by broad terminal differentiation defects and variable immune abnormalities.	J Allergy Clin Immunol.
atopic dermatitis	RAVER1	Expression profiling by array, count, 3 development stage, 2 disease state, 11 individual sets	GPL570	GSE32924 Samples	Nonlesional atopic dermatitis skin is characterized by broad terminal differentiation defects and variable immune abnormalities.	J Allergy Clin Immunol.
atopic dermatitis	SLC12A4	Expression profiling by array, count, 3 development stage, 2 disease state, 11 individual sets	GPL570	GSE32924 Samples	Nonlesional atopic dermatitis skin is characterized by broad terminal differentiation defects and variable immune abnormalities.	J Allergy Clin Immunol.
atopic dermatitis	PNPLA6	Expression profiling by array, count, 3 development stage, 2 disease state, 11 individual sets	GPL570	GSE32924 Samples	Nonlesional atopic dermatitis skin is characterized by broad terminal differentiation defects and variable immune abnormalities.	J Allergy Clin Immunol.
atopic dermatitis	C4ORF48	Expression profiling by array, count, 3 development stage, 2 disease state, 11 individual sets	GPL570	GSE32924 Samples	Nonlesional atopic dermatitis skin is characterized by broad terminal differentiation defects and variable immune abnormalities.	J Allergy Clin Immunol.
atopic dermatitis	GSDMD	Expression profiling by array, count, 3 development stage, 2 disease state, 11 individual sets	GPL570	GSE32924 Samples	Nonlesional atopic dermatitis skin is characterized by broad terminal differentiation defects and variable immune abnormalities.	J Allergy Clin Immunol.
atopic dermatitis	HSD17B1	Expression profiling by array, count, 3 development stage, 2 disease state, 11 individual sets	GPL570	GSE32924 Samples	Nonlesional atopic dermatitis skin is characterized by broad terminal differentiation defects and variable immune abnormalities.	J Allergy Clin Immunol.
atopic dermatitis	RPUSD3	Expression profiling by array, count, 3 development stage, 2 disease state, 11 individual sets	GPL570	GSE32924 Samples	Nonlesional atopic dermatitis skin is characterized by broad terminal differentiation defects and variable immune abnormalities.	J Allergy Clin Immunol.
atopic dermatitis	FAM173A	Expression profiling by array, count, 3 development stage, 2 disease state, 11 individual sets	GPL570	GSE32924 Samples	Nonlesional atopic dermatitis skin is characterized by broad terminal differentiation defects and variable immune abnormalities.	J Allergy Clin Immunol.
atopic dermatitis	LOC100133039	Expression profiling by array, count, 3 development stage, 2 disease state, 11 individual sets	GPL570	GSE32924 Samples	Nonlesional atopic dermatitis skin is characterized by broad terminal differentiation defects and variable immune abnormalities.	J Allergy Clin Immunol.
atopic dermatitis	PPFIA3	Expression profiling by array, count, 3 development stage, 2 disease state, 11 individual sets	GPL570	GSE32924 Samples	Nonlesional atopic dermatitis skin is characterized by broad terminal differentiation defects and variable immune abnormalities.	J Allergy Clin Immunol.
atopic dermatitis	CLCN7	Expression profiling by array, count, 3 development stage, 2 disease state, 11 individual sets	GPL570	GSE32924 Samples	Nonlesional atopic dermatitis skin is characterized by broad terminal differentiation defects and variable immune abnormalities.	J Allergy Clin Immunol.
atopic dermatitis	THOC4	Expression profiling by array, count, 3 development stage, 2 disease state, 11 individual sets	GPL570	GSE32924 Samples	Nonlesional atopic dermatitis skin is characterized by broad terminal differentiation defects and variable immune abnormalities.	J Allergy Clin Immunol.
atopic dermatitis	ATP13A2	Expression profiling by array, count, 3 development stage, 2 disease state, 11 individual sets	GPL570	GSE32924 Samples	Nonlesional atopic dermatitis skin is characterized by broad terminal differentiation defects and variable immune abnormalities.	J Allergy Clin Immunol.
atopic dermatitis	FASTK	Expression profiling by array, count, 3 development stage, 2 disease state, 11 individual sets	GPL570	GSE32924 Samples	Nonlesional atopic dermatitis skin is characterized by broad terminal differentiation defects and variable immune abnormalities.	J Allergy Clin Immunol.
atopic dermatitis	TBC1D5	Expression profiling by array, count, 3 development stage, 2 disease state, 11 individual sets	GPL570	GSE32924 Samples	Nonlesional atopic dermatitis skin is characterized by broad terminal differentiation defects and variable immune abnormalities.	J Allergy Clin Immunol.
atopic dermatitis	DHRS13	Expression profiling by array, count, 3 development stage, 2 disease state, 11 individual sets	GPL570	GSE32924 Samples	Nonlesional atopic dermatitis skin is characterized by broad terminal differentiation defects and variable immune abnormalities.	J Allergy Clin Immunol.
atopic dermatitis	COMTD1	Expression profiling by array, count, 3 development stage, 2 disease state, 11 individual sets	GPL570	GSE32924 Samples	Nonlesional atopic dermatitis skin is characterized by broad terminal differentiation defects and variable immune abnormalities.	J Allergy Clin Immunol.
atopic dermatitis	MTG1	Expression profiling by array, count, 3 development stage, 2 disease state, 11 individual sets	GPL570	GSE32924 Samples	Nonlesional atopic dermatitis skin is characterized by broad terminal differentiation defects and variable immune abnormalities.	J Allergy Clin Immunol.
atopic dermatitis	NA	Expression profiling by array, count, 3 development stage, 2 disease state, 11 individual sets	GPL570	GSE32924 Samples	Nonlesional atopic dermatitis skin is characterized by broad terminal differentiation defects and variable immune abnormalities.	J Allergy Clin Immunol.
atopic dermatitis	ITGB6	Expression profiling by array, count, 3 development stage, 2 disease state, 11 individual sets	GPL570	GSE32924 Samples	Nonlesional atopic dermatitis skin is characterized by broad terminal differentiation defects and variable immune abnormalities.	J Allergy Clin Immunol.

atopic dermatitis	NA	Expression profiling by array, count, 3 development stage, 2 disease state, 11 individual sets	GPL570	GSE32924 Samples	Nonlesional atopic dermatitis skin is characterized by broad terminal differentiation defects and variable immune abnormalities.	J Allergy Clin Immunol.
atopic dermatitis	PTP4A3	Expression profiling by array, count, 3 development stage, 2 disease state, 11 individual sets	GPL570	GSE32924 Samples	Nonlesional atopic dermatitis skin is characterized by broad terminal differentiation defects and variable immune abnormalities.	J Allergy Clin Immunol.
atopic dermatitis	C1QTNF1	Expression profiling by array, count, 3 development stage, 2 disease state, 11 individual sets	GPL570	GSE32924 Samples	Nonlesional atopic dermatitis skin is characterized by broad terminal differentiation defects and variable immune abnormalities.	J Allergy Clin Immunol.
atopic dermatitis	NA	Expression profiling by array, count, 3 development stage, 2 disease state, 11 individual sets	GPL570	GSE32924 Samples	Nonlesional atopic dermatitis skin is characterized by broad terminal differentiation defects and variable immune abnormalities.	J Allergy Clin Immunol.
atopic dermatitis	XRCC5	Expression profiling by array, count, 3 development stage, 2 disease state, 11 individual sets	GPL570	GSE32924 Samples	Nonlesional atopic dermatitis skin is characterized by broad terminal differentiation defects and variable immune abnormalities.	J Allergy Clin Immunol.
atopic dermatitis	NA	Expression profiling by array, count, 3 development stage, 2 disease state, 11 individual sets	GPL570	GSE32924 Samples	Nonlesional atopic dermatitis skin is characterized by broad terminal differentiation defects and variable immune abnormalities.	J Allergy Clin Immunol.
atopic dermatitis	N4BP2L2	Expression profiling by array, count, 3 development stage, 2 disease state, 11 individual sets	GPL570	GSE32924 Samples	Nonlesional atopic dermatitis skin is characterized by broad terminal differentiation defects and variable immune abnormalities.	J Allergy Clin Immunol.
atopic dermatitis	SUZ12	Expression profiling by array, count, 3 development stage, 2 disease state, 11 individual sets	GPL570	GSE32924 Samples	Nonlesional atopic dermatitis skin is characterized by broad terminal differentiation defects and variable immune abnormalities.	J Allergy Clin Immunol.
atopic dermatitis	NA	Expression profiling by array, count, 3 development stage, 2 disease state, 11 individual sets	GPL570	GSE32924 Samples	Nonlesional atopic dermatitis skin is characterized by broad terminal differentiation defects and variable immune abnormalities.	J Allergy Clin Immunol.
atopic dermatitis	NA	Expression profiling by array, count, 3 development stage, 2 disease state, 11 individual sets	GPL570	GSE32924 Samples	Nonlesional atopic dermatitis skin is characterized by broad terminal differentiation defects and variable immune abnormalities.	J Allergy Clin Immunol.
atopic dermatitis	DAPK2	Expression profiling by array, count, 3 development stage, 2 disease state, 11 individual sets	GPL570	GSE32924 Samples	Nonlesional atopic dermatitis skin is characterized by broad terminal differentiation defects and variable immune abnormalities.	J Allergy Clin Immunol.
atopic dermatitis	NA	Expression profiling by array, count, 3 development stage, 2 disease state, 11 individual sets	GPL570	GSE32924 Samples	Nonlesional atopic dermatitis skin is characterized by broad terminal differentiation defects and variable immune abnormalities.	J Allergy Clin Immunol.
atopic dermatitis	NA	Expression profiling by array, count, 3 development stage, 2 disease state, 11 individual sets	GPL570	GSE32924 Samples	Nonlesional atopic dermatitis skin is characterized by broad terminal differentiation defects and variable immune abnormalities.	J Allergy Clin Immunol.
atopic dermatitis	NA	Expression profiling by array, count, 3 development stage, 2 disease state, 11 individual sets	GPL570	GSE32924 Samples	Nonlesional atopic dermatitis skin is characterized by broad terminal differentiation defects and variable immune abnormalities.	J Allergy Clin Immunol.
atopic dermatitis	NA	Expression profiling by array, count, 3 development stage, 2 disease state, 11 individual sets	GPL570	GSE32924 Samples	Nonlesional atopic dermatitis skin is characterized by broad terminal differentiation defects and variable immune abnormalities.	J Allergy Clin Immunol.
atopic dermatitis	ATF7IP	Expression profiling by array, count, 3 development stage, 2 disease state, 11 individual sets	GPL570	GSE32924 Samples	Nonlesional atopic dermatitis skin is characterized by broad terminal differentiation defects and variable immune abnormalities.	J Allergy Clin Immunol.
atopic dermatitis	NA	Expression profiling by array, count, 3 development stage, 2 disease state, 11 individual sets	GPL570	GSE32924 Samples	Nonlesional atopic dermatitis skin is characterized by broad terminal differentiation defects and variable immune abnormalities.	J Allergy Clin Immunol.
atopic dermatitis	TMEM8A	Expression profiling by array, count, 3 development stage, 2 disease state, 11 individual sets	GPL570	GSE32924 Samples	Nonlesional atopic dermatitis skin is characterized by broad terminal differentiation defects and variable immune abnormalities.	J Allergy Clin Immunol.
atopic dermatitis	NECAB3	Expression profiling by array, count, 3 development stage, 2 disease state, 11 individual sets	GPL570	GSE32924 Samples	Nonlesional atopic dermatitis skin is characterized by broad terminal differentiation defects and variable immune abnormalities.	J Allergy Clin Immunol.
atopic dermatitis	C16ORF14	Expression profiling by array, count, 3 development stage, 2 disease state, 11 individual sets	GPL570	GSE32924 Samples	Nonlesional atopic dermatitis skin is characterized by broad terminal differentiation defects and variable immune abnormalities.	J Allergy Clin Immunol.
atopic dermatitis	TCOF1	Expression profiling by array, count, 3 development stage, 2 disease state, 11 individual sets	GPL570	GSE32924 Samples	Nonlesional atopic dermatitis skin is characterized by broad terminal differentiation defects and variable immune abnormalities.	J Allergy Clin Immunol.
atopic dermatitis	NA	Expression profiling by array, count, 3 development stage, 2 disease state, 11 individual sets	GPL570	GSE32924 Samples	Nonlesional atopic dermatitis skin is characterized by broad terminal differentiation defects and variable immune abnormalities.	J Allergy Clin Immunol.
atopic dermatitis	NA	Expression profiling by array, count, 3 development stage, 2 disease state, 11 individual sets	GPL570	GSE32924 Samples	Nonlesional atopic dermatitis skin is characterized by broad terminal differentiation defects and variable immune abnormalities.	J Allergy Clin Immunol.
atopic dermatitis	CSPG4	Expression profiling by array, count, 3 development stage, 2 disease state, 11 individual sets	GPL570	GSE32924 Samples	Nonlesional atopic dermatitis skin is characterized by broad terminal differentiation defects and variable immune abnormalities.	J Allergy Clin Immunol.
atopic dermatitis	BAT2	Expression profiling by array, count, 3 development stage, 2 disease state, 11 individual sets	GPL570	GSE32924 Samples	Nonlesional atopic dermatitis skin is characterized by broad terminal differentiation defects and variable immune abnormalities.	J Allergy Clin Immunol.
atopic dermatitis	SH2B1	Expression profiling by array, count, 3 development stage, 2 disease state, 11 individual sets	GPL570	GSE32924 Samples	Nonlesional atopic dermatitis skin is characterized by broad terminal differentiation defects and variable immune abnormalities.	J Allergy Clin Immunol.
atopic dermatitis	SH2B1	Expression profiling by array, count, 3 development stage, 2 disease state, 11 individual sets	GPL570	GSE32924 Samples	Nonlesional atopic dermatitis skin is characterized by broad terminal differentiation defects and variable immune abnormalities.	J Allergy Clin Immunol.
atopic dermatitis	C6ORF136	Expression profiling by array, count, 3 development stage, 2 disease state, 11 individual sets	GPL570	GSE32924 Samples	Nonlesional atopic dermatitis skin is characterized by broad terminal differentiation defects and variable immune abnormalities.	J Allergy Clin Immunol.
atopic dermatitis	TP53I13	Expression profiling by array, count, 3 development stage, 2 disease state, 11 individual sets	GPL570	GSE32924 Samples	Nonlesional atopic dermatitis skin is characterized by broad terminal differentiation defects and variable immune abnormalities.	J Allergy Clin Immunol.
atopic dermatitis	NA	Expression profiling by array, count, 3 development stage, 2 disease state, 11 individual sets	GPL570	GSE32924 Samples	Nonlesional atopic dermatitis skin is characterized by broad terminal differentiation defects and variable immune abnormalities.	J Allergy Clin Immunol.
atopic dermatitis	NA	Expression profiling by array, count, 3 development stage, 2 disease state, 11 individual sets	GPL570	GSE32924 Samples	Nonlesional atopic dermatitis skin is characterized by broad terminal differentiation defects and variable immune abnormalities.	J Allergy Clin Immunol.
atopic dermatitis	NA	Expression profiling by array, count, 3 development stage, 2 disease state, 11 individual sets	GPL570	GSE32924 Samples	Nonlesional atopic dermatitis skin is characterized by broad terminal differentiation defects and variable immune abnormalities.	J Allergy Clin Immunol.
atopic dermatitis	COL27A1	Expression profiling by array, count, 3 development stage, 2 disease state, 11 individual sets	GPL570	GSE32924 Samples	Nonlesional atopic dermatitis skin is characterized by broad terminal differentiation defects and variable immune abnormalities.	J Allergy Clin Immunol.
atopic dermatitis	ABCA7	Expression profiling by array, count, 3 development stage, 2 disease state, 11 individual sets	GPL570	GSE32924 Samples	Nonlesional atopic dermatitis skin is characterized by broad terminal differentiation defects and variable immune abnormalities.	J Allergy Clin Immunol.
atopic dermatitis	CSPG4	Expression profiling by array, count, 3 development stage, 2 disease state, 11 individual sets	GPL570	GSE32924 Samples	Nonlesional atopic dermatitis skin is characterized by broad terminal differentiation defects and variable immune abnormalities.	J Allergy Clin Immunol.
atopic dermatitis	C17ORF70	Expression profiling by array, count, 3 development stage, 2 disease state, 11 individual sets	GPL570	GSE32924 Samples	Nonlesional atopic dermatitis skin is characterized by broad terminal differentiation defects and variable immune abnormalities.	J Allergy Clin Immunol.
atopic dermatitis	SKIV2L	Expression profiling by array, count, 3 development stage, 2 disease state, 11 individual sets	GPL570	GSE32924 Samples	Nonlesional atopic dermatitis skin is characterized by broad terminal differentiation defects and variable immune abnormalities.	J Allergy Clin Immunol.
atopic dermatitis	NA	Expression profiling by array, count, 3 development stage, 2 disease state, 11 individual sets	GPL570	GSE32924 Samples	Nonlesional atopic dermatitis skin is characterized by broad terminal differentiation defects and variable immune abnormalities.	J Allergy Clin Immunol.
atopic dermatitis	MAP3K11	Expression profiling by array, count, 3 development stage, 2 disease state, 11 individual sets	GPL570	GSE32924 Samples	Nonlesional atopic dermatitis skin is characterized by broad terminal differentiation defects and variable immune abnormalities.	J Allergy Clin Immunol.
atopic dermatitis	C1ORF86	Expression profiling by array, count, 3 development stage, 2 disease state, 11 individual sets	GPL570	GSE32924 Samples	Nonlesional atopic dermatitis skin is characterized by broad terminal differentiation defects and variable immune abnormalities.	J Allergy Clin Immunol.
atopic dermatitis	MICALL2	Expression profiling by array, count, 3 development stage, 2 disease state, 11 individual sets	GPL570	GSE32924 Samples	Nonlesional atopic dermatitis skin is characterized by broad terminal differentiation defects and variable immune abnormalities.	J Allergy Clin Immunol.

atopic dermatitis	ACAP3	Expression profiling by array, count, 3 development stage, 2 disease state, 11 individual sets	GPL570	GSE32924 Samples	Nonlesional atopic dermatitis skin is characterized by broad terminal differentiation defects and variable immune abnormalities.	J Allergy Clin Immunol.
atopic dermatitis	IRX4	Expression profiling by array, count, 3 development stage, 2 disease state, 11 individual sets	GPL570	GSE32924 Samples	Nonlesional atopic dermatitis skin is characterized by broad terminal differentiation defects and variable immune abnormalities.	J Allergy Clin Immunol.
atopic dermatitis	C16ORF7	Expression profiling by array, count, 3 development stage, 2 disease state, 11 individual sets	GPL570	GSE32924 Samples	Nonlesional atopic dermatitis skin is characterized by broad terminal differentiation defects and variable immune abnormalities.	J Allergy Clin Immunol.
atopic dermatitis	LPHN1	Expression profiling by array, count, 3 development stage, 2 disease state, 11 individual sets	GPL570	GSE32924 Samples	Nonlesional atopic dermatitis skin is characterized by broad terminal differentiation defects and variable immune abnormalities.	J Allergy Clin Immunol.
atopic dermatitis	PAQR6	Expression profiling by array, count, 3 development stage, 2 disease state, 11 individual sets	GPL570	GSE32924 Samples	Nonlesional atopic dermatitis skin is characterized by broad terminal differentiation defects and variable immune abnormalities.	J Allergy Clin Immunol.
atopic dermatitis	NA	Expression profiling by array, count, 3 development stage, 2 disease state, 11 individual sets	GPL570	GSE32924 Samples	Nonlesional atopic dermatitis skin is characterized by broad terminal differentiation defects and variable immune abnormalities.	J Allergy Clin Immunol.
atopic dermatitis	LOC646014	Expression profiling by array, count, 3 development stage, 2 disease state, 11 individual sets	GPL570	GSE32924 Samples	Nonlesional atopic dermatitis skin is characterized by broad terminal differentiation defects and variable immune abnormalities.	J Allergy Clin Immunol.
atopic dermatitis	NA	Expression profiling by array, count, 3 development stage, 2 disease state, 11 individual sets	GPL570	GSE32924 Samples	Nonlesional atopic dermatitis skin is characterized by broad terminal differentiation defects and variable immune abnormalities.	J Allergy Clin Immunol.
atopic dermatitis	ATHL1	Expression profiling by array, count, 3 development stage, 2 disease state, 11 individual sets	GPL570	GSE32924 Samples	Nonlesional atopic dermatitis skin is characterized by broad terminal differentiation defects and variable immune abnormalities.	J Allergy Clin Immunol.
atopic dermatitis	HNRNPL	Expression profiling by array, count, 3 development stage, 2 disease state, 11 individual sets	GPL570	GSE32924 Samples	Nonlesional atopic dermatitis skin is characterized by broad terminal differentiation defects and variable immune abnormalities.	J Allergy Clin Immunol.
atopic dermatitis	C9ORF86	Expression profiling by array, count, 3 development stage, 2 disease state, 11 individual sets	GPL570	GSE32924 Samples	Nonlesional atopic dermatitis skin is characterized by broad terminal differentiation defects and variable immune abnormalities.	J Allergy Clin Immunol.
atopic dermatitis	SH3PXD2A	Expression profiling by array, count, 3 development stage, 2 disease state, 11 individual sets	GPL570	GSE32924 Samples	Nonlesional atopic dermatitis skin is characterized by broad terminal differentiation defects and variable immune abnormalities.	J Allergy Clin Immunol.
atopic dermatitis	NFIC	Expression profiling by array, count, 3 development stage, 2 disease state, 11 individual sets	GPL570	GSE32924 Samples	Nonlesional atopic dermatitis skin is characterized by broad terminal differentiation defects and variable immune abnormalities.	J Allergy Clin Immunol.
atopic dermatitis	C10ORF18	Expression profiling by array, count, 3 development stage, 2 disease state, 11 individual sets	GPL570	GSE32924 Samples	Nonlesional atopic dermatitis skin is characterized by broad terminal differentiation defects and variable immune abnormalities.	J Allergy Clin Immunol.
atopic dermatitis	SF3B1	Expression profiling by array, count, 3 development stage, 2 disease state, 11 individual sets	GPL570	GSE32924 Samples	Nonlesional atopic dermatitis skin is characterized by broad terminal differentiation defects and variable immune abnormalities.	J Allergy Clin Immunol.
atopic dermatitis	NA	Expression profiling by array, count, 3 development stage, 2 disease state, 11 individual sets	GPL570	GSE32924 Samples	Nonlesional atopic dermatitis skin is characterized by broad terminal differentiation defects and variable immune abnormalities.	J Allergy Clin Immunol.
atopic dermatitis	NA	Expression profiling by array, count, 3 development stage, 2 disease state, 11 individual sets	GPL570	GSE32924 Samples	Nonlesional atopic dermatitis skin is characterized by broad terminal differentiation defects and variable immune abnormalities.	J Allergy Clin Immunol.
atopic dermatitis	NA	Expression profiling by array, count, 3 development stage, 2 disease state, 11 individual sets	GPL570	GSE32924 Samples	Nonlesional atopic dermatitis skin is characterized by broad terminal differentiation defects and variable immune abnormalities.	J Allergy Clin Immunol.
atopic dermatitis	ZNF785	Expression profiling by array, count, 3 development stage, 2 disease state, 11 individual sets	GPL570	GSE32924 Samples	Nonlesional atopic dermatitis skin is characterized by broad terminal differentiation defects and variable immune abnormalities.	J Allergy Clin Immunol.
atopic dermatitis	ZNF785	Expression profiling by array, count, 3 development stage, 2 disease state, 11 individual sets	GPL570	GSE32924 Samples	Nonlesional atopic dermatitis skin is characterized by broad terminal differentiation defects and variable immune abnormalities.	J Allergy Clin Immunol.
atopic dermatitis	NA	Expression profiling by array, count, 3 development stage, 2 disease state, 11 individual sets	GPL570	GSE32924 Samples	Nonlesional atopic dermatitis skin is characterized by broad terminal differentiation defects and variable immune abnormalities.	J Allergy Clin Immunol.
atopic dermatitis	RSBN1	Expression profiling by array, count, 3 development stage, 2 disease state, 11 individual sets	GPL570	GSE32924 Samples	Nonlesional atopic dermatitis skin is characterized by broad terminal differentiation defects and variable immune abnormalities.	J Allergy Clin Immunol.
atopic dermatitis	NA	Expression profiling by array, count, 3 development stage, 2 disease state, 11 individual sets	GPL570	GSE32924 Samples	Nonlesional atopic dermatitis skin is characterized by broad terminal differentiation defects and variable immune abnormalities.	J Allergy Clin Immunol.
atopic dermatitis	NA	Expression profiling by array, count, 3 development stage, 2 disease state, 11 individual sets	GPL570	GSE32924 Samples	Nonlesional atopic dermatitis skin is characterized by broad terminal differentiation defects and variable immune abnormalities.	J Allergy Clin Immunol.
atopic dermatitis	LRRFIP1	Expression profiling by array, count, 3 development stage, 2 disease state, 11 individual sets	GPL570	GSE32924 Samples	Nonlesional atopic dermatitis skin is characterized by broad terminal differentiation defects and variable immune abnormalities.	J Allergy Clin Immunol.
atopic dermatitis	UBXN7	Expression profiling by array, count, 3 development stage, 2 disease state, 11 individual sets	GPL570	GSE32924 Samples	Nonlesional atopic dermatitis skin is characterized by broad terminal differentiation defects and variable immune abnormalities.	J Allergy Clin Immunol.
atopic dermatitis	AGXT2L2	Expression profiling by array, count, 3 development stage, 2 disease state, 11 individual sets	GPL570	GSE32924 Samples	Nonlesional atopic dermatitis skin is characterized by broad terminal differentiation defects and variable immune abnormalities.	J Allergy Clin Immunol.
atopic dermatitis	USP31	Expression profiling by array, count, 3 development stage, 2 disease state, 11 individual sets	GPL570	GSE32924 Samples	Nonlesional atopic dermatitis skin is characterized by broad terminal differentiation defects and variable immune abnormalities.	J Allergy Clin Immunol.
atopic dermatitis	NA	Expression profiling by array, count, 3 development stage, 2 disease state, 11 individual sets	GPL570	GSE32924 Samples	Nonlesional atopic dermatitis skin is characterized by broad terminal differentiation defects and variable immune abnormalities.	J Allergy Clin Immunol.
atopic dermatitis	NAMPT	Expression profiling by array, count, 3 development stage, 2 disease state, 11 individual sets	GPL570	GSE32924 Samples	Nonlesional atopic dermatitis skin is characterized by broad terminal differentiation defects and variable immune abnormalities.	J Allergy Clin Immunol.
atopic dermatitis	NA	Expression profiling by array, count, 3 development stage, 2 disease state, 11 individual sets	GPL570	GSE32924 Samples	Nonlesional atopic dermatitis skin is characterized by broad terminal differentiation defects and variable immune abnormalities.	J Allergy Clin Immunol.
atopic dermatitis	NA	Expression profiling by array, count, 3 development stage, 2 disease state, 11 individual sets	GPL570	GSE32924 Samples	Nonlesional atopic dermatitis skin is characterized by broad terminal differentiation defects and variable immune abnormalities.	J Allergy Clin Immunol.
atopic dermatitis	PXDN	Expression profiling by array, count, 3 development stage, 2 disease state, 11 individual sets	GPL570	GSE32924 Samples	Nonlesional atopic dermatitis skin is characterized by broad terminal differentiation defects and variable immune abnormalities.	J Allergy Clin Immunol.
atopic dermatitis	PDXK	Expression profiling by array, count, 3 development stage, 2 disease state, 11 individual sets	GPL570	GSE32924 Samples	Nonlesional atopic dermatitis skin is characterized by broad terminal differentiation defects and variable immune abnormalities.	J Allergy Clin Immunol.
atopic dermatitis	ANGPTL4	Expression profiling by array, count, 3 development stage, 2 disease state, 11 individual sets	GPL570	GSE32924 Samples	Nonlesional atopic dermatitis skin is characterized by broad terminal differentiation defects and variable immune abnormalities.	J Allergy Clin Immunol.
atopic dermatitis	GPX2	Expression profiling by array, count, 3 development stage, 2 disease state, 11 individual sets	GPL570	GSE32924 Samples	Nonlesional atopic dermatitis skin is characterized by broad terminal differentiation defects and variable immune abnormalities.	J Allergy Clin Immunol.
atopic dermatitis	PI15	Expression profiling by array, count, 3 development stage, 2 disease state, 11 individual sets	GPL570	GSE32924 Samples	Nonlesional atopic dermatitis skin is characterized by broad terminal differentiation defects and variable immune abnormalities.	J Allergy Clin Immunol.
atopic dermatitis	PSENEN	Expression profiling by array, count, 3 development stage, 2 disease state, 11 individual sets	GPL570	GSE32924 Samples	Nonlesional atopic dermatitis skin is characterized by broad terminal differentiation defects and variable immune abnormalities.	J Allergy Clin Immunol.
atopic dermatitis	THOC4	Expression profiling by array, count, 3 development stage, 2 disease state, 11 individual sets	GPL570	GSE32924 Samples	Nonlesional atopic dermatitis skin is characterized by broad terminal differentiation defects and variable immune abnormalities.	J Allergy Clin Immunol.
atopic dermatitis	HES4	Expression profiling by array, count, 3 development stage, 2 disease state, 11 individual sets	GPL570	GSE32924 Samples	Nonlesional atopic dermatitis skin is characterized by broad terminal differentiation defects and variable immune abnormalities.	J Allergy Clin Immunol.
atopic dermatitis	ODF3B	Expression profiling by array, count, 3 development stage, 2 disease state, 11 individual sets	GPL570	GSE32924 Samples	Nonlesional atopic dermatitis skin is characterized by broad terminal differentiation defects and variable immune abnormalities.	J Allergy Clin Immunol.

atopic dermatitis	TYMP	Expression profiling by array, count, 3 development stage, 2 disease state, 11 individual sets	GPL570	GSE32924 Samples	Nonlesional atopic dermatitis skin is characterized by broad terminal differentiation defects and variable immune abnormalities.	J Allergy Clin Immunol.
atopic dermatitis	TNFRSF12A	Expression profiling by array, count, 3 development stage, 2 disease state, 11 individual sets	GPL570	GSE32924 Samples	Nonlesional atopic dermatitis skin is characterized by broad terminal differentiation defects and variable immune abnormalities.	J Allergy Clin Immunol.
atopic dermatitis	NOC4L	Expression profiling by array, count, 3 development stage, 2 disease state, 11 individual sets	GPL570	GSE32924 Samples	Nonlesional atopic dermatitis skin is characterized by broad terminal differentiation defects and variable immune abnormalities.	J Allergy Clin Immunol.
atopic dermatitis	GPR172A	Expression profiling by array, count, 3 development stage, 2 disease state, 11 individual sets	GPL570	GSE32924 Samples	Nonlesional atopic dermatitis skin is characterized by broad terminal differentiation defects and variable immune abnormalities.	J Allergy Clin Immunol.
atopic dermatitis	LRCH4	Expression profiling by array, count, 3 development stage, 2 disease state, 11 individual sets	GPL570	GSE32924 Samples	Nonlesional atopic dermatitis skin is characterized by broad terminal differentiation defects and variable immune abnormalities.	J Allergy Clin Immunol.
atopic dermatitis	STOML1	Expression profiling by array, count, 3 development stage, 2 disease state, 11 individual sets	GPL570	GSE32924 Samples	Nonlesional atopic dermatitis skin is characterized by broad terminal differentiation defects and variable immune abnormalities.	J Allergy Clin Immunol.
atopic dermatitis	FARSA	Expression profiling by array, count, 3 development stage, 2 disease state, 11 individual sets	GPL570	GSE32924 Samples	Nonlesional atopic dermatitis skin is characterized by broad terminal differentiation defects and variable immune abnormalities.	J Allergy Clin Immunol.
atopic dermatitis	MTG1	Expression profiling by array, count, 3 development stage, 2 disease state, 11 individual sets	GPL570	GSE32924 Samples	Nonlesional atopic dermatitis skin is characterized by broad terminal differentiation defects and variable immune abnormalities.	J Allergy Clin Immunol.
atopic dermatitis	PNKP	Expression profiling by array, count, 3 development stage, 2 disease state, 11 individual sets	GPL570	GSE32924 Samples	Nonlesional atopic dermatitis skin is characterized by broad terminal differentiation defects and variable immune abnormalities.	J Allergy Clin Immunol.
atopic dermatitis	ITPKC	Expression profiling by array, count, 3 development stage, 2 disease state, 11 individual sets	GPL570	GSE32924 Samples	Nonlesional atopic dermatitis skin is characterized by broad terminal differentiation defects and variable immune abnormalities.	J Allergy Clin Immunol.
atopic dermatitis	GRWD1	Expression profiling by array, count, 3 development stage, 2 disease state, 11 individual sets	GPL570	GSE32924 Samples	Nonlesional atopic dermatitis skin is characterized by broad terminal differentiation defects and variable immune abnormalities.	J Allergy Clin Immunol.
atopic dermatitis	NA	Expression profiling by array, count, 3 development stage, 2 disease state, 11 individual sets	GPL570	GSE32924 Samples	Nonlesional atopic dermatitis skin is characterized by broad terminal differentiation defects and variable immune abnormalities.	J Allergy Clin Immunol.
atopic dermatitis	PLCB3	Expression profiling by array, count, 3 development stage, 2 disease state, 11 individual sets	GPL570	GSE32924 Samples	Nonlesional atopic dermatitis skin is characterized by broad terminal differentiation defects and variable immune abnormalities.	J Allergy Clin Immunol.
atopic dermatitis	COG7	Expression profiling by array, count, 3 development stage, 2 disease state, 11 individual sets	GPL570	GSE32924 Samples	Nonlesional atopic dermatitis skin is characterized by broad terminal differentiation defects and variable immune abnormalities.	J Allergy Clin Immunol.
atopic dermatitis	KRI1	Expression profiling by array, count, 3 development stage, 2 disease state, 11 individual sets	GPL570	GSE32924 Samples	Nonlesional atopic dermatitis skin is characterized by broad terminal differentiation defects and variable immune abnormalities.	J Allergy Clin Immunol.
atopic dermatitis	NA	Expression profiling by array, count, 3 development stage, 2 disease state, 11 individual sets	GPL570	GSE32924 Samples	Nonlesional atopic dermatitis skin is characterized by broad terminal differentiation defects and variable immune abnormalities.	J Allergy Clin Immunol.
atopic dermatitis	PML	Expression profiling by array, count, 3 development stage, 2 disease state, 11 individual sets	GPL570	GSE32924 Samples	Nonlesional atopic dermatitis skin is characterized by broad terminal differentiation defects and variable immune abnormalities.	J Allergy Clin Immunol.
atopic dermatitis	SLC5A6	Expression profiling by array, count, 3 development stage, 2 disease state, 11 individual sets	GPL570	GSE32924 Samples	Nonlesional atopic dermatitis skin is characterized by broad terminal differentiation defects and variable immune abnormalities.	J Allergy Clin Immunol.
atopic dermatitis	E2F3	Expression profiling by array, count, 3 development stage, 2 disease state, 11 individual sets	GPL570	GSE32924 Samples	Nonlesional atopic dermatitis skin is characterized by broad terminal differentiation defects and variable immune abnormalities.	J Allergy Clin Immunol.
atopic dermatitis	EXOSC4	Expression profiling by array, count, 3 development stage, 2 disease state, 11 individual sets	GPL570	GSE32924 Samples	Nonlesional atopic dermatitis skin is characterized by broad terminal differentiation defects and variable immune abnormalities.	J Allergy Clin Immunol.
atopic dermatitis	NA	Expression profiling by array, count, 3 development stage, 2 disease state, 11 individual sets	GPL570	GSE32924 Samples	Nonlesional atopic dermatitis skin is characterized by broad terminal differentiation defects and variable immune abnormalities.	J Allergy Clin Immunol.
atopic dermatitis	BCL3	Expression profiling by array, count, 3 development stage, 2 disease state, 11 individual sets	GPL570	GSE32924 Samples	Nonlesional atopic dermatitis skin is characterized by broad terminal differentiation defects and variable immune abnormalities.	J Allergy Clin Immunol.
atopic dermatitis	PAQR4	Expression profiling by array, count, 3 development stage, 2 disease state, 11 individual sets	GPL570	GSE32924 Samples	Nonlesional atopic dermatitis skin is characterized by broad terminal differentiation defects and variable immune abnormalities.	J Allergy Clin Immunol.
atopic dermatitis	CISH	Expression profiling by array, count, 3 development stage, 2 disease state, 11 individual sets	GPL570	GSE32924 Samples	Nonlesional atopic dermatitis skin is characterized by broad terminal differentiation defects and variable immune abnormalities.	J Allergy Clin Immunol.
atopic dermatitis	LOC153346	Expression profiling by array, count, 3 development stage, 2 disease state, 11 individual sets	GPL570	GSE32924 Samples	Nonlesional atopic dermatitis skin is characterized by broad terminal differentiation defects and variable immune abnormalities.	J Allergy Clin Immunol.
atopic dermatitis	ORC6L	Expression profiling by array, count, 3 development stage, 2 disease state, 11 individual sets	GPL570	GSE32924 Samples	Nonlesional atopic dermatitis skin is characterized by broad terminal differentiation defects and variable immune abnormalities.	J Allergy Clin Immunol.
atopic dermatitis	UBE2C	Expression profiling by array, count, 3 development stage, 2 disease state, 11 individual sets	GPL570	GSE32924 Samples	Nonlesional atopic dermatitis skin is characterized by broad terminal differentiation defects and variable immune abnormalities.	J Allergy Clin Immunol.
atopic dermatitis	CKS2	Expression profiling by array, count, 3 development stage, 2 disease state, 11 individual sets	GPL570	GSE32924 Samples	Nonlesional atopic dermatitis skin is characterized by broad terminal differentiation defects and variable immune abnormalities.	J Allergy Clin Immunol.
atopic dermatitis	NCAPG	Expression profiling by array, count, 3 development stage, 2 disease state, 11 individual sets	GPL570	GSE32924 Samples	Nonlesional atopic dermatitis skin is characterized by broad terminal differentiation defects and variable immune abnormalities.	J Allergy Clin Immunol.
atopic dermatitis	KIF18B	Expression profiling by array, count, 3 development stage, 2 disease state, 11 individual sets	GPL570	GSE32924 Samples	Nonlesional atopic dermatitis skin is characterized by broad terminal differentiation defects and variable immune abnormalities.	J Allergy Clin Immunol.
atopic dermatitis	WNT5A	Expression profiling by array, count, 3 development stage, 2 disease state, 11 individual sets	GPL570	GSE32924 Samples	Nonlesional atopic dermatitis skin is characterized by broad terminal differentiation defects and variable immune abnormalities.	J Allergy Clin Immunol.
atopic dermatitis	FANCI	Expression profiling by array, count, 3 development stage, 2 disease state, 11 individual sets	GPL570	GSE32924 Samples	Nonlesional atopic dermatitis skin is characterized by broad terminal differentiation defects and variable immune abnormalities.	J Allergy Clin Immunol.
atopic dermatitis	FANCD2	Expression profiling by array, count, 3 development stage, 2 disease state, 11 individual sets	GPL570	GSE32924 Samples	Nonlesional atopic dermatitis skin is characterized by broad terminal differentiation defects and variable immune abnormalities.	J Allergy Clin Immunol.
atopic dermatitis	CXCR4	Expression profiling by array, count, 3 development stage, 2 disease state, 11 individual sets	GPL570	GSE32924 Samples	Nonlesional atopic dermatitis skin is characterized by broad terminal differentiation defects and variable immune abnormalities.	J Allergy Clin Immunol.
atopic dermatitis	IRF7	Expression profiling by array, count, 3 development stage, 2 disease state, 11 individual sets	GPL570	GSE32924 Samples	Nonlesional atopic dermatitis skin is characterized by broad terminal differentiation defects and variable immune abnormalities.	J Allergy Clin Immunol.
atopic dermatitis	HTRA3	Expression profiling by array, count, 3 development stage, 2 disease state, 11 individual sets	GPL570	GSE32924 Samples	Nonlesional atopic dermatitis skin is characterized by broad terminal differentiation defects and variable immune abnormalities.	J Allergy Clin Immunol.
atopic dermatitis	NA	Expression profiling by array, count, 3 development stage, 2 disease state, 11 individual sets	GPL570	GSE32924 Samples	Nonlesional atopic dermatitis skin is characterized by broad terminal differentiation defects and variable immune abnormalities.	J Allergy Clin Immunol.
atopic dermatitis	IFI35	Expression profiling by array, count, 3 development stage, 2 disease state, 11 individual sets	GPL570	GSE32924 Samples	Nonlesional atopic dermatitis skin is characterized by broad terminal differentiation defects and variable immune abnormalities.	J Allergy Clin Immunol.
atopic dermatitis	CLDN7	Expression profiling by array, count, 3 development stage, 2 disease state, 11 individual sets	GPL570	GSE32924 Samples	Nonlesional atopic dermatitis skin is characterized by broad terminal differentiation defects and variable immune abnormalities.	J Allergy Clin Immunol.
atopic dermatitis	ETV1	Expression profiling by array, count, 3 development stage, 2 disease state, 11 individual sets	GPL570	GSE32924 Samples	Nonlesional atopic dermatitis skin is characterized by broad terminal differentiation defects and variable immune abnormalities.	J Allergy Clin Immunol.
atopic dermatitis	NA	Expression profiling by array, count, 3 development stage, 2 disease state, 11 individual sets	GPL570	GSE32924 Samples	Nonlesional atopic dermatitis skin is characterized by broad terminal differentiation defects and variable immune abnormalities.	J Allergy Clin Immunol.

atopic dermatitis	TBRG1	Expression profiling by array, count, 3 development stage, 2 disease state, 11 individual sets	GPL570	GSE32924 Samples	Nonlesional atopic dermatitis skin is characterized by broad terminal differentiation defects and variable immune abnormalities.	J Allergy Clin Immunol.
atopic dermatitis	TOB2	Expression profiling by array, count, 3 development stage, 2 disease state, 11 individual sets	GPL570	GSE32924 Samples	Nonlesional atopic dermatitis skin is characterized by broad terminal differentiation defects and variable immune abnormalities.	J Allergy Clin Immunol.
atopic dermatitis	LOC283788	Expression profiling by array, count, 3 development stage, 2 disease state, 11 individual sets	GPL570	GSE32924 Samples	Nonlesional atopic dermatitis skin is characterized by broad terminal differentiation defects and variable immune abnormalities.	J Allergy Clin Immunol.
atopic dermatitis	RAMP3	Expression profiling by array, count, 3 development stage, 2 disease state, 11 individual sets	GPL570	GSE32924 Samples	Nonlesional atopic dermatitis skin is characterized by broad terminal differentiation defects and variable immune abnormalities.	J Allergy Clin Immunol.
atopic dermatitis	DEF6	Expression profiling by array, count, 3 development stage, 2 disease state, 11 individual sets	GPL570	GSE32924 Samples	Nonlesional atopic dermatitis skin is characterized by broad terminal differentiation defects and variable immune abnormalities.	J Allergy Clin Immunol.
atopic dermatitis	RGS12	Expression profiling by array, count, 3 development stage, 2 disease state, 11 individual sets	GPL570	GSE32924 Samples	Nonlesional atopic dermatitis skin is characterized by broad terminal differentiation defects and variable immune abnormalities.	J Allergy Clin Immunol.
atopic dermatitis	ELMO3	Expression profiling by array, count, 3 development stage, 2 disease state, 11 individual sets	GPL570	GSE32924 Samples	Nonlesional atopic dermatitis skin is characterized by broad terminal differentiation defects and variable immune abnormalities.	J Allergy Clin Immunol.
atopic dermatitis	PLEKHJ1	Expression profiling by array, count, 3 development stage, 2 disease state, 11 individual sets	GPL570	GSE32924 Samples	Nonlesional atopic dermatitis skin is characterized by broad terminal differentiation defects and variable immune abnormalities.	J Allergy Clin Immunol.
atopic dermatitis	TNFRSF14	Expression profiling by array, count, 3 development stage, 2 disease state, 11 individual sets	GPL570	GSE32924 Samples	Nonlesional atopic dermatitis skin is characterized by broad terminal differentiation defects and variable immune abnormalities.	J Allergy Clin Immunol.
atopic dermatitis	C7ORF43	Expression profiling by array, count, 3 development stage, 2 disease state, 11 individual sets	GPL570	GSE32924 Samples	Nonlesional atopic dermatitis skin is characterized by broad terminal differentiation defects and variable immune abnormalities.	J Allergy Clin Immunol.
atopic dermatitis	TMC6	Expression profiling by array, count, 3 development stage, 2 disease state, 11 individual sets	GPL570	GSE32924 Samples	Nonlesional atopic dermatitis skin is characterized by broad terminal differentiation defects and variable immune abnormalities.	J Allergy Clin Immunol.
atopic dermatitis	PRR5	Expression profiling by array, count, 3 development stage, 2 disease state, 11 individual sets	GPL570	GSE32924 Samples	Nonlesional atopic dermatitis skin is characterized by broad terminal differentiation defects and variable immune abnormalities.	J Allergy Clin Immunol.
atopic dermatitis	CYB561	Expression profiling by array, count, 3 development stage, 2 disease state, 11 individual sets	GPL570	GSE32924 Samples	Nonlesional atopic dermatitis skin is characterized by broad terminal differentiation defects and variable immune abnormalities.	J Allergy Clin Immunol.
atopic dermatitis	C9ORF86	Expression profiling by array, count, 3 development stage, 2 disease state, 11 individual sets	GPL570	GSE32924 Samples	Nonlesional atopic dermatitis skin is characterized by broad terminal differentiation defects and variable immune abnormalities.	J Allergy Clin Immunol.
atopic dermatitis	ZFPM1	Expression profiling by array, count, 3 development stage, 2 disease state, 11 individual sets	GPL570	GSE32924 Samples	Nonlesional atopic dermatitis skin is characterized by broad terminal differentiation defects and variable immune abnormalities.	J Allergy Clin Immunol.
atopic dermatitis	ZNF777	Expression profiling by array, count, 3 development stage, 2 disease state, 11 individual sets	GPL570	GSE32924 Samples	Nonlesional atopic dermatitis skin is characterized by broad terminal differentiation defects and variable immune abnormalities.	J Allergy Clin Immunol.
atopic dermatitis	C19ORF48	Expression profiling by array, count, 3 development stage, 2 disease state, 11 individual sets	GPL570	GSE32924 Samples	Nonlesional atopic dermatitis skin is characterized by broad terminal differentiation defects and variable immune abnormalities.	J Allergy Clin Immunol.
atopic dermatitis	IFRD2	Expression profiling by array, count, 3 development stage, 2 disease state, 11 individual sets	GPL570	GSE32924 Samples	Nonlesional atopic dermatitis skin is characterized by broad terminal differentiation defects and variable immune abnormalities.	J Allergy Clin Immunol.
atopic dermatitis	NUDT22	Expression profiling by array, count, 3 development stage, 2 disease state, 11 individual sets	GPL570	GSE32924 Samples	Nonlesional atopic dermatitis skin is characterized by broad terminal differentiation defects and variable immune abnormalities.	J Allergy Clin Immunol.
atopic dermatitis	YIF1B	Expression profiling by array, count, 3 development stage, 2 disease state, 11 individual sets	GPL570	GSE32924 Samples	Nonlesional atopic dermatitis skin is characterized by broad terminal differentiation defects and variable immune abnormalities.	J Allergy Clin Immunol.
atopic dermatitis	CDK2AP2	Expression profiling by array, count, 3 development stage, 2 disease state, 11 individual sets	GPL570	GSE32924 Samples	Nonlesional atopic dermatitis skin is characterized by broad terminal differentiation defects and variable immune abnormalities.	J Allergy Clin Immunol.
atopic dermatitis	LSR	Expression profiling by array, count, 3 development stage, 2 disease state, 11 individual sets	GPL570	GSE32924 Samples	Nonlesional atopic dermatitis skin is characterized by broad terminal differentiation defects and variable immune abnormalities.	J Allergy Clin Immunol.
atopic dermatitis	TNIP2	Expression profiling by array, count, 3 development stage, 2 disease state, 11 individual sets	GPL570	GSE32924 Samples	Nonlesional atopic dermatitis skin is characterized by broad terminal differentiation defects and variable immune abnormalities.	J Allergy Clin Immunol.
atopic dermatitis	TSPAN17	Expression profiling by array, count, 3 development stage, 2 disease state, 11 individual sets	GPL570	GSE32924 Samples	Nonlesional atopic dermatitis skin is characterized by broad terminal differentiation defects and variable immune abnormalities.	J Allergy Clin Immunol.
atopic dermatitis	FBXW5	Expression profiling by array, count, 3 development stage, 2 disease state, 11 individual sets	GPL570	GSE32924 Samples	Nonlesional atopic dermatitis skin is characterized by broad terminal differentiation defects and variable immune abnormalities.	J Allergy Clin Immunol.
atopic dermatitis	PARP10	Expression profiling by array, count, 3 development stage, 2 disease state, 11 individual sets	GPL570	GSE32924 Samples	Nonlesional atopic dermatitis skin is characterized by broad terminal differentiation defects and variable immune abnormalities.	J Allergy Clin Immunol.
atopic dermatitis	NA	Expression profiling by array, count, 3 development stage, 2 disease state, 11 individual sets	GPL570	GSE32924 Samples	Nonlesional atopic dermatitis skin is characterized by broad terminal differentiation defects and variable immune abnormalities.	J Allergy Clin Immunol.
atopic dermatitis	RTKN	Expression profiling by array, count, 3 development stage, 2 disease state, 11 individual sets	GPL570	GSE32924 Samples	Nonlesional atopic dermatitis skin is characterized by broad terminal differentiation defects and variable immune abnormalities.	J Allergy Clin Immunol.
atopic dermatitis	NADSYN1	Expression profiling by array, count, 3 development stage, 2 disease state, 11 individual sets	GPL570	GSE32924 Samples	Nonlesional atopic dermatitis skin is characterized by broad terminal differentiation defects and variable immune abnormalities.	J Allergy Clin Immunol.
atopic dermatitis	EIF5A	Expression profiling by array, count, 3 development stage, 2 disease state, 11 individual sets	GPL570	GSE32924 Samples	Nonlesional atopic dermatitis skin is characterized by broad terminal differentiation defects and variable immune abnormalities.	J Allergy Clin Immunol.
atopic dermatitis	ECSIT	Expression profiling by array, count, 3 development stage, 2 disease state, 11 individual sets	GPL570	GSE32924 Samples	Nonlesional atopic dermatitis skin is characterized by broad terminal differentiation defects and variable immune abnormalities.	J Allergy Clin Immunol.
atopic dermatitis	SCAND1	Expression profiling by array, count, 3 development stage, 2 disease state, 11 individual sets	GPL570	GSE32924 Samples	Nonlesional atopic dermatitis skin is characterized by broad terminal differentiation defects and variable immune abnormalities.	J Allergy Clin Immunol.
atopic dermatitis	MFSD10	Expression profiling by array, count, 3 development stage, 2 disease state, 11 individual sets	GPL570	GSE32924 Samples	Nonlesional atopic dermatitis skin is characterized by broad terminal differentiation defects and variable immune abnormalities.	J Allergy Clin Immunol.
atopic dermatitis	C9ORF142	Expression profiling by array, count, 3 development stage, 2 disease state, 11 individual sets	GPL570	GSE32924 Samples	Nonlesional atopic dermatitis skin is characterized by broad terminal differentiation defects and variable immune abnormalities.	J Allergy Clin Immunol.
atopic dermatitis	SEMA3F	Expression profiling by array, count, 3 development stage, 2 disease state, 11 individual sets	GPL570	GSE32924 Samples	Nonlesional atopic dermatitis skin is characterized by broad terminal differentiation defects and variable immune abnormalities.	J Allergy Clin Immunol.
atopic dermatitis	MIB2	Expression profiling by array, count, 3 development stage, 2 disease state, 11 individual sets	GPL570	GSE32924 Samples	Nonlesional atopic dermatitis skin is characterized by broad terminal differentiation defects and variable immune abnormalities.	J Allergy Clin Immunol.
atopic dermatitis	LMF2	Expression profiling by array, count, 3 development stage, 2 disease state, 11 individual sets	GPL570	GSE32924 Samples	Nonlesional atopic dermatitis skin is characterized by broad terminal differentiation defects and variable immune abnormalities.	J Allergy Clin Immunol.
atopic dermatitis	ARRDC1	Expression profiling by array, count, 3 development stage, 2 disease state, 11 individual sets	GPL570	GSE32924 Samples	Nonlesional atopic dermatitis skin is characterized by broad terminal differentiation defects and variable immune abnormalities.	J Allergy Clin Immunol.
atopic dermatitis	NA	Expression profiling by array, count, 3 development stage, 2 disease state, 11 individual sets	GPL570	GSE32924 Samples	Nonlesional atopic dermatitis skin is characterized by broad terminal differentiation defects and variable immune abnormalities.	J Allergy Clin Immunol.
atopic dermatitis	DNM2	Expression profiling by array, count, 3 development stage, 2 disease state, 11 individual sets	GPL570	GSE32924 Samples	Nonlesional atopic dermatitis skin is characterized by broad terminal differentiation defects and variable immune abnormalities.	J Allergy Clin Immunol.
atopic dermatitis	SAPS1	Expression profiling by array, count, 3 development stage, 2 disease state, 11 individual sets	GPL570	GSE32924 Samples	Nonlesional atopic dermatitis skin is characterized by broad terminal differentiation defects and variable immune abnormalities.	J Allergy Clin Immunol.

atopic dermatitis	CDC42EP1	Expression profiling by array, count, 3 development stage, 2 disease state, 11 individual sets	GPL570	GSE32924 Samples	Nonlesional atopic dermatitis skin is characterized by broad terminal differentiation defects and variable immune abnormalities.	J Allergy Clin Immunol.
atopic dermatitis	NA	Expression profiling by array, count, 3 development stage, 2 disease state, 11 individual sets	GPL570	GSE32924 Samples	Nonlesional atopic dermatitis skin is characterized by broad terminal differentiation defects and variable immune abnormalities.	J Allergy Clin Immunol.
atopic dermatitis	ASXL1	Expression profiling by array, count, 3 development stage, 2 disease state, 11 individual sets	GPL570	GSE32924 Samples	Nonlesional atopic dermatitis skin is characterized by broad terminal differentiation defects and variable immune abnormalities.	J Allergy Clin Immunol.
atopic dermatitis	TNFRSF10A	Expression profiling by array, count, 3 development stage, 2 disease state, 11 individual sets	GPL570	GSE32924 Samples	Nonlesional atopic dermatitis skin is characterized by broad terminal differentiation defects and variable immune abnormalities.	J Allergy Clin Immunol.
atopic dermatitis	NA	Expression profiling by array, count, 3 development stage, 2 disease state, 11 individual sets	GPL570	GSE32924 Samples	Nonlesional atopic dermatitis skin is characterized by broad terminal differentiation defects and variable immune abnormalities.	J Allergy Clin Immunol.
atopic dermatitis	NA	Expression profiling by array, count, 3 development stage, 2 disease state, 11 individual sets	GPL570	GSE32924 Samples	Nonlesional atopic dermatitis skin is characterized by broad terminal differentiation defects and variable immune abnormalities.	J Allergy Clin Immunol.
atopic dermatitis	DICER1	Expression profiling by array, count, 3 development stage, 2 disease state, 11 individual sets	GPL570	GSE32924 Samples	Nonlesional atopic dermatitis skin is characterized by broad terminal differentiation defects and variable immune abnormalities.	J Allergy Clin Immunol.
atopic dermatitis	ASXL1	Expression profiling by array, count, 3 development stage, 2 disease state, 11 individual sets	GPL570	GSE32924 Samples	Nonlesional atopic dermatitis skin is characterized by broad terminal differentiation defects and variable immune abnormalities.	J Allergy Clin Immunol.
atopic dermatitis	NA	Expression profiling by array, count, 3 development stage, 2 disease state, 11 individual sets	GPL570	GSE32924 Samples	Nonlesional atopic dermatitis skin is characterized by broad terminal differentiation defects and variable immune abnormalities.	J Allergy Clin Immunol.
atopic dermatitis	NA	Expression profiling by array, count, 3 development stage, 2 disease state, 11 individual sets	GPL570	GSE32924 Samples	Nonlesional atopic dermatitis skin is characterized by broad terminal differentiation defects and variable immune abnormalities.	J Allergy Clin Immunol.
atopic dermatitis	RAB7L1	Expression profiling by array, count, 3 development stage, 2 disease state, 11 individual sets	GPL570	GSE32924 Samples	Nonlesional atopic dermatitis skin is characterized by broad terminal differentiation defects and variable immune abnormalities.	J Allergy Clin Immunol.
atopic dermatitis	PRO2852	Expression profiling by array, count, 3 development stage, 2 disease state, 11 individual sets	GPL570	GSE32924 Samples	Nonlesional atopic dermatitis skin is characterized by broad terminal differentiation defects and variable immune abnormalities.	J Allergy Clin Immunol.
atopic dermatitis	SLC35A3	Expression profiling by array, count, 3 development stage, 2 disease state, 11 individual sets	GPL570	GSE32924 Samples	Nonlesional atopic dermatitis skin is characterized by broad terminal differentiation defects and variable immune abnormalities.	J Allergy Clin Immunol.
atopic dermatitis	NA	Expression profiling by array, count, 3 development stage, 2 disease state, 11 individual sets	GPL570	GSE32924 Samples	Nonlesional atopic dermatitis skin is characterized by broad terminal differentiation defects and variable immune abnormalities.	J Allergy Clin Immunol.
atopic dermatitis	TP53I3	Expression profiling by array, count, 3 development stage, 2 disease state, 11 individual sets	GPL570	GSE32924 Samples	Nonlesional atopic dermatitis skin is characterized by broad terminal differentiation defects and variable immune abnormalities.	J Allergy Clin Immunol.
atopic dermatitis	GART	Expression profiling by array, count, 3 development stage, 2 disease state, 11 individual sets	GPL570	GSE32924 Samples	Nonlesional atopic dermatitis skin is characterized by broad terminal differentiation defects and variable immune abnormalities.	J Allergy Clin Immunol.
atopic dermatitis	NA	Expression profiling by array, count, 3 development stage, 2 disease state, 11 individual sets	GPL570	GSE32924 Samples	Nonlesional atopic dermatitis skin is characterized by broad terminal differentiation defects and variable immune abnormalities.	J Allergy Clin Immunol.
atopic dermatitis	MMP28	Expression profiling by array, count, 3 development stage, 2 disease state, 11 individual sets	GPL570	GSE32924 Samples	Nonlesional atopic dermatitis skin is characterized by broad terminal differentiation defects and variable immune abnormalities.	J Allergy Clin Immunol.
atopic dermatitis	GEN1	Expression profiling by array, count, 3 development stage, 2 disease state, 11 individual sets	GPL570	GSE32924 Samples	Nonlesional atopic dermatitis skin is characterized by broad terminal differentiation defects and variable immune abnormalities.	J Allergy Clin Immunol.
atopic dermatitis	DLEU2	Expression profiling by array, count, 3 development stage, 2 disease state, 11 individual sets	GPL570	GSE32924 Samples	Nonlesional atopic dermatitis skin is characterized by broad terminal differentiation defects and variable immune abnormalities.	J Allergy Clin Immunol.
atopic dermatitis	TPCN2	Expression profiling by array, count, 3 development stage, 2 disease state, 11 individual sets	GPL570	GSE32924 Samples	Nonlesional atopic dermatitis skin is characterized by broad terminal differentiation defects and variable immune abnormalities.	J Allergy Clin Immunol.
atopic dermatitis	NKTR	Expression profiling by array, count, 3 development stage, 2 disease state, 11 individual sets	GPL570	GSE32924 Samples	Nonlesional atopic dermatitis skin is characterized by broad terminal differentiation defects and variable immune abnormalities.	J Allergy Clin Immunol.
atopic dermatitis	C7ORF44	Expression profiling by array, count, 3 development stage, 2 disease state, 11 individual sets	GPL570	GSE32924 Samples	Nonlesional atopic dermatitis skin is characterized by broad terminal differentiation defects and variable immune abnormalities.	J Allergy Clin Immunol.
atopic dermatitis	SNRPA1	Expression profiling by array, count, 3 development stage, 2 disease state, 11 individual sets	GPL570	GSE32924 Samples	Nonlesional atopic dermatitis skin is characterized by broad terminal differentiation defects and variable immune abnormalities.	J Allergy Clin Immunol.
atopic dermatitis	ARFGAP1	Expression profiling by array, count, 3 development stage, 2 disease state, 11 individual sets	GPL570	GSE32924 Samples	Nonlesional atopic dermatitis skin is characterized by broad terminal differentiation defects and variable immune abnormalities.	J Allergy Clin Immunol.
atopic dermatitis	AKAP13	Expression profiling by array, count, 3 development stage, 2 disease state, 11 individual sets	GPL570	GSE32924 Samples	Nonlesional atopic dermatitis skin is characterized by broad terminal differentiation defects and variable immune abnormalities.	J Allergy Clin Immunol.
atopic dermatitis	CDK10	Expression profiling by array, count, 3 development stage, 2 disease state, 11 individual sets	GPL570	GSE32924 Samples	Nonlesional atopic dermatitis skin is characterized by broad terminal differentiation defects and variable immune abnormalities.	J Allergy Clin Immunol.
atopic dermatitis	CHD7	Expression profiling by array, count, 3 development stage, 2 disease state, 11 individual sets	GPL570	GSE32924 Samples	Nonlesional atopic dermatitis skin is characterized by broad terminal differentiation defects and variable immune abnormalities.	J Allergy Clin Immunol.
atopic dermatitis	GTPBP8	Expression profiling by array, count, 3 development stage, 2 disease state, 11 individual sets	GPL570	GSE32924 Samples	Nonlesional atopic dermatitis skin is characterized by broad terminal differentiation defects and variable immune abnormalities.	J Allergy Clin Immunol.
atopic dermatitis	EXOC3	Expression profiling by array, count, 3 development stage, 2 disease state, 11 individual sets	GPL570	GSE32924 Samples	Nonlesional atopic dermatitis skin is characterized by broad terminal differentiation defects and variable immune abnormalities.	J Allergy Clin Immunol.
atopic dermatitis	ALS2CL	Expression profiling by array, count, 3 development stage, 2 disease state, 11 individual sets	GPL570	GSE32924 Samples	Nonlesional atopic dermatitis skin is characterized by broad terminal differentiation defects and variable immune abnormalities.	J Allergy Clin Immunol.
atopic dermatitis	FAM160B2	Expression profiling by array, count, 3 development stage, 2 disease state, 11 individual sets	GPL570	GSE32924 Samples	Nonlesional atopic dermatitis skin is characterized by broad terminal differentiation defects and variable immune abnormalities.	J Allergy Clin Immunol.
atopic dermatitis	MBD3	Expression profiling by array, count, 3 development stage, 2 disease state, 11 individual sets	GPL570	GSE32924 Samples	Nonlesional atopic dermatitis skin is characterized by broad terminal differentiation defects and variable immune abnormalities.	J Allergy Clin Immunol.
atopic dermatitis	FAM113A	Expression profiling by array, count, 3 development stage, 2 disease state, 11 individual sets	GPL570	GSE32924 Samples	Nonlesional atopic dermatitis skin is characterized by broad terminal differentiation defects and variable immune abnormalities.	J Allergy Clin Immunol.
atopic dermatitis	WDR34	Expression profiling by array, count, 3 development stage, 2 disease state, 11 individual sets	GPL570	GSE32924 Samples	Nonlesional atopic dermatitis skin is characterized by broad terminal differentiation defects and variable immune abnormalities.	J Allergy Clin Immunol.
atopic dermatitis	ATAD3A	Expression profiling by array, count, 3 development stage, 2 disease state, 11 individual sets	GPL570	GSE32924 Samples	Nonlesional atopic dermatitis skin is characterized by broad terminal differentiation defects and variable immune abnormalities.	J Allergy Clin Immunol.
atopic dermatitis	TCIRG1	Expression profiling by array, count, 3 development stage, 2 disease state, 11 individual sets	GPL570	GSE32924 Samples	Nonlesional atopic dermatitis skin is characterized by broad terminal differentiation defects and variable immune abnormalities.	J Allergy Clin Immunol.
atopic dermatitis	DGKZ	Expression profiling by array, count, 3 development stage, 2 disease state, 11 individual sets	GPL570	GSE32924 Samples	Nonlesional atopic dermatitis skin is characterized by broad terminal differentiation defects and variable immune abnormalities.	J Allergy Clin Immunol.
atopic dermatitis	CDK10	Expression profiling by array, count, 3 development stage, 2 disease state, 11 individual sets	GPL570	GSE32924 Samples	Nonlesional atopic dermatitis skin is characterized by broad terminal differentiation defects and variable immune abnormalities.	J Allergy Clin Immunol.
atopic dermatitis	ECE1	Expression profiling by array, count, 3 development stage, 2 disease state, 11 individual sets	GPL570	GSE32924 Samples	Nonlesional atopic dermatitis skin is characterized by broad terminal differentiation defects and variable immune abnormalities.	J Allergy Clin Immunol.
atopic dermatitis	NAPEPLD	Expression profiling by array, count, 3 development stage, 2 disease state, 11 individual sets	GPL570	GSE32924 Samples	Nonlesional atopic dermatitis skin is characterized by broad terminal differentiation defects and variable immune abnormalities.	J Allergy Clin Immunol.

atopic dermatitis	NA	Expression profiling by array, count, 3 development stage, 2 disease state, 11 individual sets	GPL570	GSE32924 Samples	Nonlesional atopic dermatitis skin is characterized by broad terminal differentiation defects and variable immune abnormalities.	J Allergy Clin Immunol.
atopic dermatitis	NA	Expression profiling by array, count, 3 development stage, 2 disease state, 11 individual sets	GPL570	GSE32924 Samples	Nonlesional atopic dermatitis skin is characterized by broad terminal differentiation defects and variable immune abnormalities.	J Allergy Clin Immunol.
atopic dermatitis	NA	Expression profiling by array, count, 3 development stage, 2 disease state, 11 individual sets	GPL570	GSE32924 Samples	Nonlesional atopic dermatitis skin is characterized by broad terminal differentiation defects and variable immune abnormalities.	J Allergy Clin Immunol.
atopic dermatitis	TCAG7.907	Expression profiling by array, count, 3 development stage, 2 disease state, 11 individual sets	GPL570	GSE32924 Samples	Nonlesional atopic dermatitis skin is characterized by broad terminal differentiation defects and variable immune abnormalities.	J Allergy Clin Immunol.
atopic dermatitis	NA	Expression profiling by array, count, 3 development stage, 2 disease state, 11 individual sets	GPL570	GSE32924 Samples	Nonlesional atopic dermatitis skin is characterized by broad terminal differentiation defects and variable immune abnormalities.	J Allergy Clin Immunol.
atopic dermatitis	BFAR	Expression profiling by array, count, 3 development stage, 2 disease state, 11 individual sets	GPL570	GSE32924 Samples	Nonlesional atopic dermatitis skin is characterized by broad terminal differentiation defects and variable immune abnormalities.	J Allergy Clin Immunol.
atopic dermatitis	RNF213	Expression profiling by array, count, 3 development stage, 2 disease state, 11 individual sets	GPL570	GSE32924 Samples	Nonlesional atopic dermatitis skin is characterized by broad terminal differentiation defects and variable immune abnormalities.	J Allergy Clin Immunol.
atopic dermatitis	RHBDF2	Expression profiling by array, count, 3 development stage, 2 disease state, 11 individual sets	GPL570	GSE32924 Samples	Nonlesional atopic dermatitis skin is characterized by broad terminal differentiation defects and variable immune abnormalities.	J Allergy Clin Immunol.
atopic dermatitis	RGS14	Expression profiling by array, count, 3 development stage, 2 disease state, 11 individual sets	GPL570	GSE32924 Samples	Nonlesional atopic dermatitis skin is characterized by broad terminal differentiation defects and variable immune abnormalities.	J Allergy Clin Immunol.
atopic dermatitis	LINS1	Expression profiling by array, count, 3 development stage, 2 disease state, 11 individual sets	GPL570	GSE32924 Samples	Nonlesional atopic dermatitis skin is characterized by broad terminal differentiation defects and variable immune abnormalities.	J Allergy Clin Immunol.
atopic dermatitis	ILVBL	Expression profiling by array, count, 3 development stage, 2 disease state, 11 individual sets	GPL570	GSE32924 Samples	Nonlesional atopic dermatitis skin is characterized by broad terminal differentiation defects and variable immune abnormalities.	J Allergy Clin Immunol.
atopic dermatitis	BCKDHA	Expression profiling by array, count, 3 development stage, 2 disease state, 11 individual sets	GPL570	GSE32924 Samples	Nonlesional atopic dermatitis skin is characterized by broad terminal differentiation defects and variable immune abnormalities.	J Allergy Clin Immunol.
atopic dermatitis	MRPL12	Expression profiling by array, count, 3 development stage, 2 disease state, 11 individual sets	GPL570	GSE32924 Samples	Nonlesional atopic dermatitis skin is characterized by broad terminal differentiation defects and variable immune abnormalities.	J Allergy Clin Immunol.
atopic dermatitis	EIF5A	Expression profiling by array, count, 3 development stage, 2 disease state, 11 individual sets	GPL570	GSE32924 Samples	Nonlesional atopic dermatitis skin is characterized by broad terminal differentiation defects and variable immune abnormalities.	J Allergy Clin Immunol.
atopic dermatitis	SRA1	Expression profiling by array, count, 3 development stage, 2 disease state, 11 individual sets	GPL570	GSE32924 Samples	Nonlesional atopic dermatitis skin is characterized by broad terminal differentiation defects and variable immune abnormalities.	J Allergy Clin Immunol.
atopic dermatitis	TIPRL	Expression profiling by array, count, 3 development stage, 2 disease state, 11 individual sets	GPL570	GSE32924 Samples	Nonlesional atopic dermatitis skin is characterized by broad terminal differentiation defects and variable immune abnormalities.	J Allergy Clin Immunol.
atopic dermatitis	NA	Expression profiling by array, count, 3 development stage, 2 disease state, 11 individual sets	GPL570	GSE32924 Samples	Nonlesional atopic dermatitis skin is characterized by broad terminal differentiation defects and variable immune abnormalities.	J Allergy Clin Immunol.
atopic dermatitis	NA	Expression profiling by array, count, 3 development stage, 2 disease state, 11 individual sets	GPL570	GSE32924 Samples	Nonlesional atopic dermatitis skin is characterized by broad terminal differentiation defects and variable immune abnormalities.	J Allergy Clin Immunol.
atopic dermatitis	NA	Expression profiling by array, count, 3 development stage, 2 disease state, 11 individual sets	GPL570	GSE32924 Samples	Nonlesional atopic dermatitis skin is characterized by broad terminal differentiation defects and variable immune abnormalities.	J Allergy Clin Immunol.
atopic dermatitis	RNF213	Expression profiling by array, count, 3 development stage, 2 disease state, 11 individual sets	GPL570	GSE32924 Samples	Nonlesional atopic dermatitis skin is characterized by broad terminal differentiation defects and variable immune abnormalities.	J Allergy Clin Immunol.
atopic dermatitis	FOXP1	Expression profiling by array, count, 3 development stage, 2 disease state, 11 individual sets	GPL570	GSE32924 Samples	Nonlesional atopic dermatitis skin is characterized by broad terminal differentiation defects and variable immune abnormalities.	J Allergy Clin Immunol.
atopic dermatitis	S100A10	Expression profiling by array, count, 3 development stage, 2 disease state, 11 individual sets	GPL570	GSE32924 Samples	Nonlesional atopic dermatitis skin is characterized by broad terminal differentiation defects and variable immune abnormalities.	J Allergy Clin Immunol.
atopic dermatitis	NA	Expression profiling by array, count, 3 development stage, 2 disease state, 11 individual sets	GPL570	GSE32924 Samples	Nonlesional atopic dermatitis skin is characterized by broad terminal differentiation defects and variable immune abnormalities.	J Allergy Clin Immunol.
atopic dermatitis	RSC1A1	Expression profiling by array, count, 3 development stage, 2 disease state, 11 individual sets	GPL570	GSE32924 Samples	Nonlesional atopic dermatitis skin is characterized by broad terminal differentiation defects and variable immune abnormalities.	J Allergy Clin Immunol.
atopic dermatitis	ZGPAT	Expression profiling by array, count, 3 development stage, 2 disease state, 11 individual sets	GPL570	GSE32924 Samples	Nonlesional atopic dermatitis skin is characterized by broad terminal differentiation defects and variable immune abnormalities.	J Allergy Clin Immunol.
atopic dermatitis	NA	Expression profiling by array, count, 3 development stage, 2 disease state, 11 individual sets	GPL570	GSE32924 Samples	Nonlesional atopic dermatitis skin is characterized by broad terminal differentiation defects and variable immune abnormalities.	J Allergy Clin Immunol.
atopic dermatitis	SLC25A23	Expression profiling by array, count, 3 development stage, 2 disease state, 11 individual sets	GPL570	GSE32924 Samples	Nonlesional atopic dermatitis skin is characterized by broad terminal differentiation defects and variable immune abnormalities.	J Allergy Clin Immunol.
atopic dermatitis	TFEB	Expression profiling by array, count, 3 development stage, 2 disease state, 11 individual sets	GPL570	GSE32924 Samples	Nonlesional atopic dermatitis skin is characterized by broad terminal differentiation defects and variable immune abnormalities.	J Allergy Clin Immunol.
atopic dermatitis	RNF145	Expression profiling by array, count, 3 development stage, 2 disease state, 11 individual sets	GPL570	GSE32924 Samples	Nonlesional atopic dermatitis skin is characterized by broad terminal differentiation defects and variable immune abnormalities.	J Allergy Clin Immunol.
atopic dermatitis	NDE1	Expression profiling by array, count, 3 development stage, 2 disease state, 11 individual sets	GPL570	GSE32924 Samples	Nonlesional atopic dermatitis skin is characterized by broad terminal differentiation defects and variable immune abnormalities.	J Allergy Clin Immunol.
atopic dermatitis	MST1R	Expression profiling by array, count, 3 development stage, 2 disease state, 11 individual sets	GPL570	GSE32924 Samples	Nonlesional atopic dermatitis skin is characterized by broad terminal differentiation defects and variable immune abnormalities.	J Allergy Clin Immunol.
atopic dermatitis	TRIM13	Expression profiling by array, count, 3 development stage, 2 disease state, 11 individual sets	GPL570	GSE32924 Samples	Nonlesional atopic dermatitis skin is characterized by broad terminal differentiation defects and variable immune abnormalities.	J Allergy Clin Immunol.
atopic dermatitis	MALAT1	Expression profiling by array, count, 3 development stage, 2 disease state, 11 individual sets	GPL570	GSE32924 Samples	Nonlesional atopic dermatitis skin is characterized by broad terminal differentiation defects and variable immune abnormalities.	J Allergy Clin Immunol.
atopic dermatitis	TMED4	Expression profiling by array, count, 3 development stage, 2 disease state, 11 individual sets	GPL570	GSE32924 Samples	Nonlesional atopic dermatitis skin is characterized by broad terminal differentiation defects and variable immune abnormalities.	J Allergy Clin Immunol.
atopic dermatitis	ACY1	Expression profiling by array, count, 3 development stage, 2 disease state, 11 individual sets	GPL570	GSE32924 Samples	Nonlesional atopic dermatitis skin is characterized by broad terminal differentiation defects and variable immune abnormalities.	J Allergy Clin Immunol.
atopic dermatitis	RSBN1L	Expression profiling by array, count, 3 development stage, 2 disease state, 11 individual sets	GPL570	GSE32924 Samples	Nonlesional atopic dermatitis skin is characterized by broad terminal differentiation defects and variable immune abnormalities.	J Allergy Clin Immunol.
atopic dermatitis	SF3A2	Expression profiling by array, count, 3 development stage, 2 disease state, 11 individual sets	GPL570	GSE32924 Samples	Nonlesional atopic dermatitis skin is characterized by broad terminal differentiation defects and variable immune abnormalities.	J Allergy Clin Immunol.
atopic dermatitis	NA	Expression profiling by array, count, 3 development stage, 2 disease state, 11 individual sets	GPL570	GSE32924 Samples	Nonlesional atopic dermatitis skin is characterized by broad terminal differentiation defects and variable immune abnormalities.	J Allergy Clin Immunol.
atopic dermatitis	NA	Expression profiling by array, count, 3 development stage, 2 disease state, 11 individual sets	GPL570	GSE32924 Samples	Nonlesional atopic dermatitis skin is characterized by broad terminal differentiation defects and variable immune abnormalities.	J Allergy Clin Immunol.
atopic dermatitis	FNDC3B	Expression profiling by array, count, 3 development stage, 2 disease state, 11 individual sets	GPL570	GSE32924 Samples	Nonlesional atopic dermatitis skin is characterized by broad terminal differentiation defects and variable immune abnormalities.	J Allergy Clin Immunol.
atopic dermatitis	RER1	Expression profiling by array, count, 3 development stage, 2 disease state, 11 individual sets	GPL570	GSE32924 Samples	Nonlesional atopic dermatitis skin is characterized by broad terminal differentiation defects and variable immune abnormalities.	J Allergy Clin Immunol.

atopic dermatitis	MAN2C1	Expression profiling by array, count, 3 development stage, 2 disease state, 11 individual sets	GPL570	GSE32924 Samples	Nonlesional atopic dermatitis skin is characterized by broad terminal differentiation defects and variable immune abnormalities.	J Allergy Clin Immunol.
atopic dermatitis	RP3-402G11.5	Expression profiling by array, count, 3 development stage, 2 disease state, 11 individual sets	GPL570	GSE32924 Samples	Nonlesional atopic dermatitis skin is characterized by broad terminal differentiation defects and variable immune abnormalities.	J Allergy Clin Immunol.
atopic dermatitis	POMGNT1	Expression profiling by array, count, 3 development stage, 2 disease state, 11 individual sets	GPL570	GSE32924 Samples	Nonlesional atopic dermatitis skin is characterized by broad terminal differentiation defects and variable immune abnormalities.	J Allergy Clin Immunol.
atopic dermatitis	NUCKS1	Expression profiling by array, count, 3 development stage, 2 disease state, 11 individual sets	GPL570	GSE32924 Samples	Nonlesional atopic dermatitis skin is characterized by broad terminal differentiation defects and variable immune abnormalities.	J Allergy Clin Immunol.
atopic dermatitis	BAT2	Expression profiling by array, count, 3 development stage, 2 disease state, 11 individual sets	GPL570	GSE32924 Samples	Nonlesional atopic dermatitis skin is characterized by broad terminal differentiation defects and variable immune abnormalities.	J Allergy Clin Immunol.
atopic dermatitis	PLVAP	Expression profiling by array, count, 3 development stage, 2 disease state, 11 individual sets	GPL570	GSE32924 Samples	Nonlesional atopic dermatitis skin is characterized by broad terminal differentiation defects and variable immune abnormalities.	J Allergy Clin Immunol.
atopic dermatitis	CTSD	Expression profiling by array, count, 3 development stage, 2 disease state, 11 individual sets	GPL570	GSE32924 Samples	Nonlesional atopic dermatitis skin is characterized by broad terminal differentiation defects and variable immune abnormalities.	J Allergy Clin Immunol.
atopic dermatitis	CYBA	Expression profiling by array, count, 3 development stage, 2 disease state, 11 individual sets	GPL570	GSE32924 Samples	Nonlesional atopic dermatitis skin is characterized by broad terminal differentiation defects and variable immune abnormalities.	J Allergy Clin Immunol.
atopic dermatitis	C19ORF22	Expression profiling by array, count, 3 development stage, 2 disease state, 11 individual sets	GPL570	GSE32924 Samples	Nonlesional atopic dermatitis skin is characterized by broad terminal differentiation defects and variable immune abnormalities.	J Allergy Clin Immunol.
atopic dermatitis	ZG16B	Expression profiling by array, count, 3 development stage, 2 disease state, 11 individual sets	GPL570	GSE32924 Samples	Nonlesional atopic dermatitis skin is characterized by broad terminal differentiation defects and variable immune abnormalities.	J Allergy Clin Immunol.
atopic dermatitis	STARD10	Expression profiling by array, count, 3 development stage, 2 disease state, 11 individual sets	GPL570	GSE32924 Samples	Nonlesional atopic dermatitis skin is characterized by broad terminal differentiation defects and variable immune abnormalities.	J Allergy Clin Immunol.
atopic dermatitis	SCRIB	Expression profiling by array, count, 3 development stage, 2 disease state, 11 individual sets	GPL570	GSE32924 Samples	Nonlesional atopic dermatitis skin is characterized by broad terminal differentiation defects and variable immune abnormalities.	J Allergy Clin Immunol.
atopic dermatitis	RANGAP1	Expression profiling by array, count, 3 development stage, 2 disease state, 11 individual sets	GPL570	GSE32924 Samples	Nonlesional atopic dermatitis skin is characterized by broad terminal differentiation defects and variable immune abnormalities.	J Allergy Clin Immunol.
atopic dermatitis	C16ORF42	Expression profiling by array, count, 3 development stage, 2 disease state, 11 individual sets	GPL570	GSE32924 Samples	Nonlesional atopic dermatitis skin is characterized by broad terminal differentiation defects and variable immune abnormalities.	J Allergy Clin Immunol.
atopic dermatitis	STRA13	Expression profiling by array, count, 3 development stage, 2 disease state, 11 individual sets	GPL570	GSE32924 Samples	Nonlesional atopic dermatitis skin is characterized by broad terminal differentiation defects and variable immune abnormalities.	J Allergy Clin Immunol.
atopic dermatitis	ARD1A	Expression profiling by array, count, 3 development stage, 2 disease state, 11 individual sets	GPL570	GSE32924 Samples	Nonlesional atopic dermatitis skin is characterized by broad terminal differentiation defects and variable immune abnormalities.	J Allergy Clin Immunol.
atopic dermatitis	C17ORF62	Expression profiling by array, count, 3 development stage, 2 disease state, 11 individual sets	GPL570	GSE32924 Samples	Nonlesional atopic dermatitis skin is characterized by broad terminal differentiation defects and variable immune abnormalities.	J Allergy Clin Immunol.
atopic dermatitis	TNIP2	Expression profiling by array, count, 3 development stage, 2 disease state, 11 individual sets	GPL570	GSE32924 Samples	Nonlesional atopic dermatitis skin is characterized by broad terminal differentiation defects and variable immune abnormalities.	J Allergy Clin Immunol.
atopic dermatitis	SNHG12	Expression profiling by array, count, 3 development stage, 2 disease state, 11 individual sets	GPL570	GSE32924 Samples	Nonlesional atopic dermatitis skin is characterized by broad terminal differentiation defects and variable immune abnormalities.	J Allergy Clin Immunol.
atopic dermatitis	GBAP	Expression profiling by array, count, 3 development stage, 2 disease state, 11 individual sets	GPL570	GSE32924 Samples	Nonlesional atopic dermatitis skin is characterized by broad terminal differentiation defects and variable immune abnormalities.	J Allergy Clin Immunol.
atopic dermatitis	SAC3D1	Expression profiling by array, count, 3 development stage, 2 disease state, 11 individual sets	GPL570	GSE32924 Samples	Nonlesional atopic dermatitis skin is characterized by broad terminal differentiation defects and variable immune abnormalities.	J Allergy Clin Immunol.
atopic dermatitis	PARP12	Expression profiling by array, count, 3 development stage, 2 disease state, 11 individual sets	GPL570	GSE32924 Samples	Nonlesional atopic dermatitis skin is characterized by broad terminal differentiation defects and variable immune abnormalities.	J Allergy Clin Immunol.
atopic dermatitis	IRF9	Expression profiling by array, count, 3 development stage, 2 disease state, 11 individual sets	GPL570	GSE32924 Samples	Nonlesional atopic dermatitis skin is characterized by broad terminal differentiation defects and variable immune abnormalities.	J Allergy Clin Immunol.
atopic dermatitis	MVP	Expression profiling by array, count, 3 development stage, 2 disease state, 11 individual sets	GPL570	GSE32924 Samples	Nonlesional atopic dermatitis skin is characterized by broad terminal differentiation defects and variable immune abnormalities.	J Allergy Clin Immunol.
atopic dermatitis	VSNL1	Expression profiling by array, count, 3 development stage, 2 disease state, 11 individual sets	GPL570	GSE32924 Samples	Nonlesional atopic dermatitis skin is characterized by broad terminal differentiation defects and variable immune abnormalities.	J Allergy Clin Immunol.
atopic dermatitis	CANT1	Expression profiling by array, count, 3 development stage, 2 disease state, 11 individual sets	GPL570	GSE32924 Samples	Nonlesional atopic dermatitis skin is characterized by broad terminal differentiation defects and variable immune abnormalities.	J Allergy Clin Immunol.
atopic dermatitis	DTYMK	Expression profiling by array, count, 3 development stage, 2 disease state, 11 individual sets	GPL570	GSE32924 Samples	Nonlesional atopic dermatitis skin is characterized by broad terminal differentiation defects and variable immune abnormalities.	J Allergy Clin Immunol.
atopic dermatitis	TRIM47	Expression profiling by array, count, 3 development stage, 2 disease state, 11 individual sets	GPL570	GSE32924 Samples	Nonlesional atopic dermatitis skin is characterized by broad terminal differentiation defects and variable immune abnormalities.	J Allergy Clin Immunol.
atopic dermatitis	CDC25B	Expression profiling by array, count, 3 development stage, 2 disease state, 11 individual sets	GPL570	GSE32924 Samples	Nonlesional atopic dermatitis skin is characterized by broad terminal differentiation defects and variable immune abnormalities.	J Allergy Clin Immunol.
atopic dermatitis	NA	Expression profiling by array, count, 3 development stage, 2 disease state, 11 individual sets	GPL570	GSE32924 Samples	Nonlesional atopic dermatitis skin is characterized by broad terminal differentiation defects and variable immune abnormalities.	J Allergy Clin Immunol.
atopic dermatitis	MCM5	Expression profiling by array, count, 3 development stage, 2 disease state, 11 individual sets	GPL570	GSE32924 Samples	Nonlesional atopic dermatitis skin is characterized by broad terminal differentiation defects and variable immune abnormalities.	J Allergy Clin Immunol.
atopic dermatitis	H2AFX	Expression profiling by array, count, 3 development stage, 2 disease state, 11 individual sets	GPL570	GSE32924 Samples	Nonlesional atopic dermatitis skin is characterized by broad terminal differentiation defects and variable immune abnormalities.	J Allergy Clin Immunol.
atopic dermatitis	TYMP	Expression profiling by array, count, 3 development stage, 2 disease state, 11 individual sets	GPL570	GSE32924 Samples	Nonlesional atopic dermatitis skin is characterized by broad terminal differentiation defects and variable immune abnormalities.	J Allergy Clin Immunol.
atopic dermatitis	SCO2	Expression profiling by array, count, 3 development stage, 2 disease state, 11 individual sets	GPL570	GSE32924 Samples	Nonlesional atopic dermatitis skin is characterized by broad terminal differentiation defects and variable immune abnormalities.	J Allergy Clin Immunol.
atopic dermatitis	EHF	Expression profiling by array, count, 3 development stage, 2 disease state, 11 individual sets	GPL570	GSE32924 Samples	Nonlesional atopic dermatitis skin is characterized by broad terminal differentiation defects and variable immune abnormalities.	J Allergy Clin Immunol.
atopic dermatitis	NA	Expression profiling by array, count, 3 development stage, 2 disease state, 11 individual sets	GPL570	GSE32924 Samples	Nonlesional atopic dermatitis skin is characterized by broad terminal differentiation defects and variable immune abnormalities.	J Allergy Clin Immunol.
atopic dermatitis	TNPO1	Expression profiling by array, count, 3 development stage, 2 disease state, 11 individual sets	GPL570	GSE32924 Samples	Nonlesional atopic dermatitis skin is characterized by broad terminal differentiation defects and variable immune abnormalities.	J Allergy Clin Immunol.
atopic dermatitis	APRT	Expression profiling by array, count, 3 development stage, 2 disease state, 11 individual sets	GPL570	GSE32924 Samples	Nonlesional atopic dermatitis skin is characterized by broad terminal differentiation defects and variable immune abnormalities.	J Allergy Clin Immunol.
atopic dermatitis	RPS6KB2	Expression profiling by array, count, 3 development stage, 2 disease state, 11 individual sets	GPL570	GSE32924 Samples	Nonlesional atopic dermatitis skin is characterized by broad terminal differentiation defects and variable immune abnormalities.	J Allergy Clin Immunol.
atopic dermatitis	PSMB10	Expression profiling by array, count, 3 development stage, 2 disease state, 11 individual sets	GPL570	GSE32924 Samples	Nonlesional atopic dermatitis skin is characterized by broad terminal differentiation defects and variable immune abnormalities.	J Allergy Clin Immunol.
atopic dermatitis	WNT5A	Expression profiling by array, count, 3 development stage, 2 disease state, 11 individual sets	GPL570	GSE32924 Samples	Nonlesional atopic dermatitis skin is characterized by broad terminal differentiation defects and variable immune abnormalities.	J Allergy Clin Immunol.

atopic dermatitis	F12	Expression profiling by array, count, 3 development stage, 2 disease state, 11 individual sets	GPL570	GSE32924 Samples	Nonlesional atopic dermatitis skin is characterized by broad terminal differentiation defects and variable immune abnormalities.	J Allergy Clin Immunol.
atopic dermatitis	REEP4	Expression profiling by array, count, 3 development stage, 2 disease state, 11 individual sets	GPL570	GSE32924 Samples	Nonlesional atopic dermatitis skin is characterized by broad terminal differentiation defects and variable immune abnormalities.	J Allergy Clin Immunol.
atopic dermatitis	NOSIP	Expression profiling by array, count, 3 development stage, 2 disease state, 11 individual sets	GPL570	GSE32924 Samples	Nonlesional atopic dermatitis skin is characterized by broad terminal differentiation defects and variable immune abnormalities.	J Allergy Clin Immunol.
atopic dermatitis	CDC42EP5	Expression profiling by array, count, 3 development stage, 2 disease state, 11 individual sets	GPL570	GSE32924 Samples	Nonlesional atopic dermatitis skin is characterized by broad terminal differentiation defects and variable immune abnormalities.	J Allergy Clin Immunol.
atopic dermatitis	ZBTB7B	Expression profiling by array, count, 3 development stage, 2 disease state, 11 individual sets	GPL570	GSE32924 Samples	Nonlesional atopic dermatitis skin is characterized by broad terminal differentiation defects and variable immune abnormalities.	J Allergy Clin Immunol.
atopic dermatitis	LTB4R	Expression profiling by array, count, 3 development stage, 2 disease state, 11 individual sets	GPL570	GSE32924 Samples	Nonlesional atopic dermatitis skin is characterized by broad terminal differentiation defects and variable immune abnormalities.	J Allergy Clin Immunol.
atopic dermatitis	ZFP36L2	Expression profiling by array, count, 3 development stage, 2 disease state, 11 individual sets	GPL570	GSE32924 Samples	Nonlesional atopic dermatitis skin is characterized by broad terminal differentiation defects and variable immune abnormalities.	J Allergy Clin Immunol.
atopic dermatitis	NA	Expression profiling by array, count, 3 development stage, 2 disease state, 11 individual sets	GPL570	GSE32924 Samples	Nonlesional atopic dermatitis skin is characterized by broad terminal differentiation defects and variable immune abnormalities.	J Allergy Clin Immunol.
atopic dermatitis	MZF1	Expression profiling by array, count, 3 development stage, 2 disease state, 11 individual sets	GPL570	GSE32924 Samples	Nonlesional atopic dermatitis skin is characterized by broad terminal differentiation defects and variable immune abnormalities.	J Allergy Clin Immunol.
atopic dermatitis	CTBP2	Expression profiling by array, count, 3 development stage, 2 disease state, 11 individual sets	GPL570	GSE32924 Samples	Nonlesional atopic dermatitis skin is characterized by broad terminal differentiation defects and variable immune abnormalities.	J Allergy Clin Immunol.
atopic dermatitis	AKAP13	Expression profiling by array, count, 3 development stage, 2 disease state, 11 individual sets	GPL570	GSE32924 Samples	Nonlesional atopic dermatitis skin is characterized by broad terminal differentiation defects and variable immune abnormalities.	J Allergy Clin Immunol.
atopic dermatitis	WASH3P	Expression profiling by array, count, 3 development stage, 2 disease state, 11 individual sets	GPL570	GSE32924 Samples	Nonlesional atopic dermatitis skin is characterized by broad terminal differentiation defects and variable immune abnormalities.	J Allergy Clin Immunol.
atopic dermatitis	NA	Expression profiling by array, count, 3 development stage, 2 disease state, 11 individual sets	GPL570	GSE32924 Samples	Nonlesional atopic dermatitis skin is characterized by broad terminal differentiation defects and variable immune abnormalities.	J Allergy Clin Immunol.
atopic dermatitis	NA	Expression profiling by array, count, 3 development stage, 2 disease state, 11 individual sets	GPL570	GSE32924 Samples	Nonlesional atopic dermatitis skin is characterized by broad terminal differentiation defects and variable immune abnormalities.	J Allergy Clin Immunol.
atopic dermatitis	NA	Expression profiling by array, count, 3 development stage, 2 disease state, 11 individual sets	GPL570	GSE32924 Samples	Nonlesional atopic dermatitis skin is characterized by broad terminal differentiation defects and variable immune abnormalities.	J Allergy Clin Immunol.
atopic dermatitis	TNFRSF1A	Expression profiling by array, count, 3 development stage, 2 disease state, 11 individual sets	GPL570	GSE32924 Samples	Nonlesional atopic dermatitis skin is characterized by broad terminal differentiation defects and variable immune abnormalities.	J Allergy Clin Immunol.
atopic dermatitis	ARPC1B	Expression profiling by array, count, 3 development stage, 2 disease state, 11 individual sets	GPL570	GSE32924 Samples	Nonlesional atopic dermatitis skin is characterized by broad terminal differentiation defects and variable immune abnormalities.	J Allergy Clin Immunol.
atopic dermatitis	NA	Expression profiling by array, count, 3 development stage, 2 disease state, 11 individual sets	GPL570	GSE32924 Samples	Nonlesional atopic dermatitis skin is characterized by broad terminal differentiation defects and variable immune abnormalities.	J Allergy Clin Immunol.
atopic dermatitis	NAPRT1	Expression profiling by array, count, 3 development stage, 2 disease state, 11 individual sets	GPL570	GSE32924 Samples	Nonlesional atopic dermatitis skin is characterized by broad terminal differentiation defects and variable immune abnormalities.	J Allergy Clin Immunol.
atopic dermatitis	TAPBP	Expression profiling by array, count, 3 development stage, 2 disease state, 11 individual sets	GPL570	GSE32924 Samples	Nonlesional atopic dermatitis skin is characterized by broad terminal differentiation defects and variable immune abnormalities.	J Allergy Clin Immunol.
atopic dermatitis	CSNK1A1	Expression profiling by array, count, 3 development stage, 2 disease state, 11 individual sets	GPL570	GSE32924 Samples	Nonlesional atopic dermatitis skin is characterized by broad terminal differentiation defects and variable immune abnormalities.	J Allergy Clin Immunol.
atopic dermatitis	PLDN	Expression profiling by array, count, 3 development stage, 2 disease state, 11 individual sets	GPL570	GSE32924 Samples	Nonlesional atopic dermatitis skin is characterized by broad terminal differentiation defects and variable immune abnormalities.	J Allergy Clin Immunol.
atopic dermatitis	NA	Expression profiling by array, count, 3 development stage, 2 disease state, 11 individual sets	GPL570	GSE32924 Samples	Nonlesional atopic dermatitis skin is characterized by broad terminal differentiation defects and variable immune abnormalities.	J Allergy Clin Immunol.
atopic dermatitis	AURKAIP1	Expression profiling by array, count, 3 development stage, 2 disease state, 11 individual sets	GPL570	GSE32924 Samples	Nonlesional atopic dermatitis skin is characterized by broad terminal differentiation defects and variable immune abnormalities.	J Allergy Clin Immunol.
atopic dermatitis	FAM129B	Expression profiling by array, count, 3 development stage, 2 disease state, 11 individual sets	GPL570	GSE32924 Samples	Nonlesional atopic dermatitis skin is characterized by broad terminal differentiation defects and variable immune abnormalities.	J Allergy Clin Immunol.
atopic dermatitis	YARS	Expression profiling by array, count, 3 development stage, 2 disease state, 11 individual sets	GPL570	GSE32924 Samples	Nonlesional atopic dermatitis skin is characterized by broad terminal differentiation defects and variable immune abnormalities.	J Allergy Clin Immunol.
atopic dermatitis	NA	Expression profiling by array, count, 3 development stage, 2 disease state, 11 individual sets	GPL570	GSE32924 Samples	Nonlesional atopic dermatitis skin is characterized by broad terminal differentiation defects and variable immune abnormalities.	J Allergy Clin Immunol.
atopic dermatitis	SNHG12	Expression profiling by array, count, 3 development stage, 2 disease state, 11 individual sets	GPL570	GSE32924 Samples	Nonlesional atopic dermatitis skin is characterized by broad terminal differentiation defects and variable immune abnormalities.	J Allergy Clin Immunol.
atopic dermatitis	DLEU2	Expression profiling by array, count, 3 development stage, 2 disease state, 11 individual sets	GPL570	GSE32924 Samples	Nonlesional atopic dermatitis skin is characterized by broad terminal differentiation defects and variable immune abnormalities.	J Allergy Clin Immunol.
atopic dermatitis	ESRP2	Expression profiling by array, count, 3 development stage, 2 disease state, 11 individual sets	GPL570	GSE32924 Samples	Nonlesional atopic dermatitis skin is characterized by broad terminal differentiation defects and variable immune abnormalities.	J Allergy Clin Immunol.
atopic dermatitis	ANPEP	Expression profiling by array, count, 3 development stage, 2 disease state, 11 individual sets	GPL570	GSE32924 Samples	Nonlesional atopic dermatitis skin is characterized by broad terminal differentiation defects and variable immune abnormalities.	J Allergy Clin Immunol.
atopic dermatitis	CARD10	Expression profiling by array, count, 3 development stage, 2 disease state, 11 individual sets	GPL570	GSE32924 Samples	Nonlesional atopic dermatitis skin is characterized by broad terminal differentiation defects and variable immune abnormalities.	J Allergy Clin Immunol.
atopic dermatitis	SEC61A1	Expression profiling by array, count, 3 development stage, 2 disease state, 11 individual sets	GPL570	GSE32924 Samples	Nonlesional atopic dermatitis skin is characterized by broad terminal differentiation defects and variable immune abnormalities.	J Allergy Clin Immunol.
atopic dermatitis	FMNL2	Expression profiling by array, count, 3 development stage, 2 disease state, 11 individual sets	GPL570	GSE32924 Samples	Nonlesional atopic dermatitis skin is characterized by broad terminal differentiation defects and variable immune abnormalities.	J Allergy Clin Immunol.
atopic dermatitis	FASTKD1	Expression profiling by array, count, 3 development stage, 2 disease state, 11 individual sets	GPL570	GSE32924 Samples	Nonlesional atopic dermatitis skin is characterized by broad terminal differentiation defects and variable immune abnormalities.	J Allergy Clin Immunol.
atopic dermatitis	MED16	Expression profiling by array, count, 3 development stage, 2 disease state, 11 individual sets	GPL570	GSE32924 Samples	Nonlesional atopic dermatitis skin is characterized by broad terminal differentiation defects and variable immune abnormalities.	J Allergy Clin Immunol.
atopic dermatitis	MICAL1	Expression profiling by array, count, 3 development stage, 2 disease state, 11 individual sets	GPL570	GSE32924 Samples	Nonlesional atopic dermatitis skin is characterized by broad terminal differentiation defects and variable immune abnormalities.	J Allergy Clin Immunol.
atopic dermatitis	MGC2752	Expression profiling by array, count, 3 development stage, 2 disease state, 11 individual sets	GPL570	GSE32924 Samples	Nonlesional atopic dermatitis skin is characterized by broad terminal differentiation defects and variable immune abnormalities.	J Allergy Clin Immunol.
atopic dermatitis	NDUFS7	Expression profiling by array, count, 3 development stage, 2 disease state, 11 individual sets	GPL570	GSE32924 Samples	Nonlesional atopic dermatitis skin is characterized by broad terminal differentiation defects and variable immune abnormalities.	J Allergy Clin Immunol.
atopic dermatitis	AK1	Expression profiling by array, count, 3 development stage, 2 disease state, 11 individual sets	GPL570	GSE32924 Samples	Nonlesional atopic dermatitis skin is characterized by broad terminal differentiation defects and variable immune abnormalities.	J Allergy Clin Immunol.
atopic dermatitis	NADSYN1	Expression profiling by array, count, 3 development stage, 2 disease state, 11 individual sets	GPL570	GSE32924 Samples	Nonlesional atopic dermatitis skin is characterized by broad terminal differentiation defects and variable immune abnormalities.	J Allergy Clin Immunol.

atopic dermatitis	SLC4A11	Expression profiling by array, count, 3 development stage, 2 disease state, 11 individual sets	GPL570	GSE32924 Samples	Nonlesional atopic dermatitis skin is characterized by broad terminal differentiation defects and variable immune abnormalities.	J Allergy Clin Immunol.
atopic dermatitis	NA	Expression profiling by array, count, 3 development stage, 2 disease state, 11 individual sets	GPL570	GSE32924 Samples	Nonlesional atopic dermatitis skin is characterized by broad terminal differentiation defects and variable immune abnormalities.	J Allergy Clin Immunol.
atopic dermatitis	SMARCA4	Expression profiling by array, count, 3 development stage, 2 disease state, 11 individual sets	GPL570	GSE32924 Samples	Nonlesional atopic dermatitis skin is characterized by broad terminal differentiation defects and variable immune abnormalities.	J Allergy Clin Immunol.
atopic dermatitis	SF3A2	Expression profiling by array, count, 3 development stage, 2 disease state, 11 individual sets	GPL570	GSE32924 Samples	Nonlesional atopic dermatitis skin is characterized by broad terminal differentiation defects and variable immune abnormalities.	J Allergy Clin Immunol.
atopic dermatitis	NA	Expression profiling by array, count, 3 development stage, 2 disease state, 11 individual sets	GPL570	GSE32924 Samples	Nonlesional atopic dermatitis skin is characterized by broad terminal differentiation defects and variable immune abnormalities.	J Allergy Clin Immunol.
atopic dermatitis	ACER3	Expression profiling by array, count, 3 development stage, 2 disease state, 11 individual sets	GPL570	GSE32924 Samples	Nonlesional atopic dermatitis skin is characterized by broad terminal differentiation defects and variable immune abnormalities.	J Allergy Clin Immunol.
atopic dermatitis	SCAND1	Expression profiling by array, count, 3 development stage, 2 disease state, 11 individual sets	GPL570	GSE32924 Samples	Nonlesional atopic dermatitis skin is characterized by broad terminal differentiation defects and variable immune abnormalities.	J Allergy Clin Immunol.
atopic dermatitis	SCAND1	Expression profiling by array, count, 3 development stage, 2 disease state, 11 individual sets	GPL570	GSE32924 Samples	Nonlesional atopic dermatitis skin is characterized by broad terminal differentiation defects and variable immune abnormalities.	J Allergy Clin Immunol.
atopic dermatitis	CLCN7	Expression profiling by array, count, 3 development stage, 2 disease state, 11 individual sets	GPL570	GSE32924 Samples	Nonlesional atopic dermatitis skin is characterized by broad terminal differentiation defects and variable immune abnormalities.	J Allergy Clin Immunol.
atopic dermatitis	HGS	Expression profiling by array, count, 3 development stage, 2 disease state, 11 individual sets	GPL570	GSE32924 Samples	Nonlesional atopic dermatitis skin is characterized by broad terminal differentiation defects and variable immune abnormalities.	J Allergy Clin Immunol.
atopic dermatitis	RABEP2	Expression profiling by array, count, 3 development stage, 2 disease state, 11 individual sets	GPL570	GSE32924 Samples	Nonlesional atopic dermatitis skin is characterized by broad terminal differentiation defects and variable immune abnormalities.	J Allergy Clin Immunol.
atopic dermatitis	RP3-402G11.5	Expression profiling by array, count, 3 development stage, 2 disease state, 11 individual sets	GPL570	GSE32924 Samples	Nonlesional atopic dermatitis skin is characterized by broad terminal differentiation defects and variable immune abnormalities.	J Allergy Clin Immunol.
atopic dermatitis	NA	Expression profiling by array, count, 3 development stage, 2 disease state, 11 individual sets	GPL570	GSE32924 Samples	Nonlesional atopic dermatitis skin is characterized by broad terminal differentiation defects and variable immune abnormalities.	J Allergy Clin Immunol.
atopic dermatitis	MED13	Expression profiling by array, count, 3 development stage, 2 disease state, 11 individual sets	GPL570	GSE32924 Samples	Nonlesional atopic dermatitis skin is characterized by broad terminal differentiation defects and variable immune abnormalities.	J Allergy Clin Immunol.
atopic dermatitis	EDEM3	Expression profiling by array, count, 3 development stage, 2 disease state, 11 individual sets	GPL570	GSE32924 Samples	Nonlesional atopic dermatitis skin is characterized by broad terminal differentiation defects and variable immune abnormalities.	J Allergy Clin Immunol.
atopic dermatitis	RBM47	Expression profiling by array, count, 3 development stage, 2 disease state, 11 individual sets	GPL570	GSE32924 Samples	Nonlesional atopic dermatitis skin is characterized by broad terminal differentiation defects and variable immune abnormalities.	J Allergy Clin Immunol.
atopic dermatitis	ILVBL	Expression profiling by array, count, 3 development stage, 2 disease state, 11 individual sets	GPL570	GSE32924 Samples	Nonlesional atopic dermatitis skin is characterized by broad terminal differentiation defects and variable immune abnormalities.	J Allergy Clin Immunol.
atopic dermatitis	MAP2K7	Expression profiling by array, count, 3 development stage, 2 disease state, 11 individual sets	GPL570	GSE32924 Samples	Nonlesional atopic dermatitis skin is characterized by broad terminal differentiation defects and variable immune abnormalities.	J Allergy Clin Immunol.
atopic dermatitis	MSI2	Expression profiling by array, count, 3 development stage, 2 disease state, 11 individual sets	GPL570	GSE32924 Samples	Nonlesional atopic dermatitis skin is characterized by broad terminal differentiation defects and variable immune abnormalities.	J Allergy Clin Immunol.
atopic dermatitis	NA	Expression profiling by array, count, 3 development stage, 2 disease state, 11 individual sets	GPL570	GSE32924 Samples	Nonlesional atopic dermatitis skin is characterized by broad terminal differentiation defects and variable immune abnormalities.	J Allergy Clin Immunol.
atopic dermatitis	F11R	Expression profiling by array, count, 3 development stage, 2 disease state, 11 individual sets	GPL570	GSE32924 Samples	Nonlesional atopic dermatitis skin is characterized by broad terminal differentiation defects and variable immune abnormalities.	J Allergy Clin Immunol.
atopic dermatitis	C19ORF22	Expression profiling by array, count, 3 development stage, 2 disease state, 11 individual sets	GPL570	GSE32924 Samples	Nonlesional atopic dermatitis skin is characterized by broad terminal differentiation defects and variable immune abnormalities.	J Allergy Clin Immunol.
atopic dermatitis	CDC37	Expression profiling by array, count, 3 development stage, 2 disease state, 11 individual sets	GPL570	GSE32924 Samples	Nonlesional atopic dermatitis skin is characterized by broad terminal differentiation defects and variable immune abnormalities.	J Allergy Clin Immunol.
atopic dermatitis	CAPN1	Expression profiling by array, count, 3 development stage, 2 disease state, 11 individual sets	GPL570	GSE32924 Samples	Nonlesional atopic dermatitis skin is characterized by broad terminal differentiation defects and variable immune abnormalities.	J Allergy Clin Immunol.
atopic dermatitis	BAIAP2	Expression profiling by array, count, 3 development stage, 2 disease state, 11 individual sets	GPL570	GSE32924 Samples	Nonlesional atopic dermatitis skin is characterized by broad terminal differentiation defects and variable immune abnormalities.	J Allergy Clin Immunol.
atopic dermatitis	C19ORF6	Expression profiling by array, count, 3 development stage, 2 disease state, 11 individual sets	GPL570	GSE32924 Samples	Nonlesional atopic dermatitis skin is characterized by broad terminal differentiation defects and variable immune abnormalities.	J Allergy Clin Immunol.
atopic dermatitis	NA	Expression profiling by array, count, 3 development stage, 2 disease state, 11 individual sets	GPL570	GSE32924 Samples	Nonlesional atopic dermatitis skin is characterized by broad terminal differentiation defects and variable immune abnormalities.	J Allergy Clin Immunol.
atopic dermatitis	NA	Expression profiling by array, count, 3 development stage, 2 disease state, 11 individual sets	GPL570	GSE32924 Samples	Nonlesional atopic dermatitis skin is characterized by broad terminal differentiation defects and variable immune abnormalities.	J Allergy Clin Immunol.
atopic dermatitis	PLEKHB2	Expression profiling by array, count, 3 development stage, 2 disease state, 11 individual sets	GPL570	GSE32924 Samples	Nonlesional atopic dermatitis skin is characterized by broad terminal differentiation defects and variable immune abnormalities.	J Allergy Clin Immunol.
atopic dermatitis	NA	Expression profiling by array, count, 3 development stage, 2 disease state, 11 individual sets	GPL570	GSE32924 Samples	Nonlesional atopic dermatitis skin is characterized by broad terminal differentiation defects and variable immune abnormalities.	J Allergy Clin Immunol.
atopic dermatitis	ERAP1	Expression profiling by array, count, 3 development stage, 2 disease state, 11 individual sets	GPL570	GSE32924 Samples	Nonlesional atopic dermatitis skin is characterized by broad terminal differentiation defects and variable immune abnormalities.	J Allergy Clin Immunol.
atopic dermatitis	NA	Expression profiling by array, count, 3 development stage, 2 disease state, 11 individual sets	GPL570	GSE32924 Samples	Nonlesional atopic dermatitis skin is characterized by broad terminal differentiation defects and variable immune abnormalities.	J Allergy Clin Immunol.
atopic dermatitis	BICD2	Expression profiling by array, count, 3 development stage, 2 disease state, 11 individual sets	GPL570	GSE32924 Samples	Nonlesional atopic dermatitis skin is characterized by broad terminal differentiation defects and variable immune abnormalities.	J Allergy Clin Immunol.
atopic dermatitis	CHIC2	Expression profiling by array, count, 3 development stage, 2 disease state, 11 individual sets	GPL570	GSE32924 Samples	Nonlesional atopic dermatitis skin is characterized by broad terminal differentiation defects and variable immune abnormalities.	J Allergy Clin Immunol.
atopic dermatitis	CSNK1A1	Expression profiling by array, count, 3 development stage, 2 disease state, 11 individual sets	GPL570	GSE32924 Samples	Nonlesional atopic dermatitis skin is characterized by broad terminal differentiation defects and variable immune abnormalities.	J Allergy Clin Immunol.
atopic dermatitis	GNAI3	Expression profiling by array, count, 3 development stage, 2 disease state, 11 individual sets	GPL570	GSE32924 Samples	Nonlesional atopic dermatitis skin is characterized by broad terminal differentiation defects and variable immune abnormalities.	J Allergy Clin Immunol.
atopic dermatitis	TPD52	Expression profiling by array, count, 3 development stage, 2 disease state, 11 individual sets	GPL570	GSE32924 Samples	Nonlesional atopic dermatitis skin is characterized by broad terminal differentiation defects and variable immune abnormalities.	J Allergy Clin Immunol.
atopic dermatitis	CCDC90A	Expression profiling by array, count, 3 development stage, 2 disease state, 11 individual sets	GPL570	GSE32924 Samples	Nonlesional atopic dermatitis skin is characterized by broad terminal differentiation defects and variable immune abnormalities.	J Allergy Clin Immunol.
atopic dermatitis	BACH1	Expression profiling by array, count, 3 development stage, 2 disease state, 11 individual sets	GPL570	GSE32924 Samples	Nonlesional atopic dermatitis skin is characterized by broad terminal differentiation defects and variable immune abnormalities.	J Allergy Clin Immunol.
atopic dermatitis	SRGAP2P1	Expression profiling by array, count, 3 development stage, 2 disease state, 11 individual sets	GPL570	GSE32924 Samples	Nonlesional atopic dermatitis skin is characterized by broad terminal differentiation defects and variable immune abnormalities.	J Allergy Clin Immunol.
atopic dermatitis	HPGD	Expression profiling by array, count, 3 development stage, 2 disease state, 11 individual sets	GPL570	GSE32924 Samples	Nonlesional atopic dermatitis skin is characterized by broad terminal differentiation defects and variable immune abnormalities.	J Allergy Clin Immunol.

atopic dermatitis	HPGD	Expression profiling by array, count, 3 development stage, 2 disease state, 11 individual sets	GPL570	GSE32924 Samples	Nonlesional atopic dermatitis skin is characterized by broad terminal differentiation defects and variable immune abnormalities.	J Allergy Clin Immunol.
atopic dermatitis	HPGD	Expression profiling by array, count, 3 development stage, 2 disease state, 11 individual sets	GPL570	GSE32924 Samples	Nonlesional atopic dermatitis skin is characterized by broad terminal differentiation defects and variable immune abnormalities.	J Allergy Clin Immunol.
atopic dermatitis	RORA	Expression profiling by array, count, 3 development stage, 2 disease state, 11 individual sets	GPL570	GSE32924 Samples	Nonlesional atopic dermatitis skin is characterized by broad terminal differentiation defects and variable immune abnormalities.	J Allergy Clin Immunol.
atopic dermatitis	RAB11FIP1	Expression profiling by array, count, 3 development stage, 2 disease state, 11 individual sets	GPL570	GSE32924 Samples	Nonlesional atopic dermatitis skin is characterized by broad terminal differentiation defects and variable immune abnormalities.	J Allergy Clin Immunol.
atopic dermatitis	RORA	Expression profiling by array, count, 3 development stage, 2 disease state, 11 individual sets	GPL570	GSE32924 Samples	Nonlesional atopic dermatitis skin is characterized by broad terminal differentiation defects and variable immune abnormalities.	J Allergy Clin Immunol.
atopic dermatitis	BPIL2	Expression profiling by array, count, 3 development stage, 2 disease state, 11 individual sets	GPL570	GSE32924 Samples	Nonlesional atopic dermatitis skin is characterized by broad terminal differentiation defects and variable immune abnormalities.	J Allergy Clin Immunol.
atopic dermatitis	RAB14	Expression profiling by array, count, 3 development stage, 2 disease state, 11 individual sets	GPL570	GSE32924 Samples	Nonlesional atopic dermatitis skin is characterized by broad terminal differentiation defects and variable immune abnormalities.	J Allergy Clin Immunol.
atopic dermatitis	C1ORF43	Expression profiling by array, count, 3 development stage, 2 disease state, 11 individual sets	GPL570	GSE32924 Samples	Nonlesional atopic dermatitis skin is characterized by broad terminal differentiation defects and variable immune abnormalities.	J Allergy Clin Immunol.
atopic dermatitis	OCIAD1	Expression profiling by array, count, 3 development stage, 2 disease state, 11 individual sets	GPL570	GSE32924 Samples	Nonlesional atopic dermatitis skin is characterized by broad terminal differentiation defects and variable immune abnormalities.	J Allergy Clin Immunol.
atopic dermatitis	UBL3	Expression profiling by array, count, 3 development stage, 2 disease state, 11 individual sets	GPL570	GSE32924 Samples	Nonlesional atopic dermatitis skin is characterized by broad terminal differentiation defects and variable immune abnormalities.	J Allergy Clin Immunol.
atopic dermatitis	SYPL1	Expression profiling by array, count, 3 development stage, 2 disease state, 11 individual sets	GPL570	GSE32924 Samples	Nonlesional atopic dermatitis skin is characterized by broad terminal differentiation defects and variable immune abnormalities.	J Allergy Clin Immunol.
atopic dermatitis	VAMP3	Expression profiling by array, count, 3 development stage, 2 disease state, 11 individual sets	GPL570	GSE32924 Samples	Nonlesional atopic dermatitis skin is characterized by broad terminal differentiation defects and variable immune abnormalities.	J Allergy Clin Immunol.
atopic dermatitis	POLR2K	Expression profiling by array, count, 3 development stage, 2 disease state, 11 individual sets	GPL570	GSE32924 Samples	Nonlesional atopic dermatitis skin is characterized by broad terminal differentiation defects and variable immune abnormalities.	J Allergy Clin Immunol.
atopic dermatitis	HSP90B1	Expression profiling by array, count, 3 development stage, 2 disease state, 11 individual sets	GPL570	GSE32924 Samples	Nonlesional atopic dermatitis skin is characterized by broad terminal differentiation defects and variable immune abnormalities.	J Allergy Clin Immunol.
atopic dermatitis	LAPTM4B	Expression profiling by array, count, 3 development stage, 2 disease state, 11 individual sets	GPL570	GSE32924 Samples	Nonlesional atopic dermatitis skin is characterized by broad terminal differentiation defects and variable immune abnormalities.	J Allergy Clin Immunol.
atopic dermatitis	OTUD1	Expression profiling by array, count, 3 development stage, 2 disease state, 11 individual sets	GPL570	GSE32924 Samples	Nonlesional atopic dermatitis skin is characterized by broad terminal differentiation defects and variable immune abnormalities.	J Allergy Clin Immunol.
atopic dermatitis	NDFIP2	Expression profiling by array, count, 3 development stage, 2 disease state, 11 individual sets	GPL570	GSE32924 Samples	Nonlesional atopic dermatitis skin is characterized by broad terminal differentiation defects and variable immune abnormalities.	J Allergy Clin Immunol.
atopic dermatitis	COL17A1	Expression profiling by array, count, 3 development stage, 2 disease state, 11 individual sets	GPL570	GSE32924 Samples	Nonlesional atopic dermatitis skin is characterized by broad terminal differentiation defects and variable immune abnormalities.	J Allergy Clin Immunol.
atopic dermatitis	NA	Expression profiling by array, count, 3 development stage, 2 disease state, 11 individual sets	GPL570	GSE32924 Samples	Nonlesional atopic dermatitis skin is characterized by broad terminal differentiation defects and variable immune abnormalities.	J Allergy Clin Immunol.
atopic dermatitis	MUC15	Expression profiling by array, count, 3 development stage, 2 disease state, 11 individual sets	GPL570	GSE32924 Samples	Nonlesional atopic dermatitis skin is characterized by broad terminal differentiation defects and variable immune abnormalities.	J Allergy Clin Immunol.
atopic dermatitis	SLC31A2	Expression profiling by array, count, 3 development stage, 2 disease state, 11 individual sets	GPL570	GSE32924 Samples	Nonlesional atopic dermatitis skin is characterized by broad terminal differentiation defects and variable immune abnormalities.	J Allergy Clin Immunol.
atopic dermatitis	NA	Expression profiling by array, count, 3 development stage, 2 disease state, 11 individual sets	GPL570	GSE32924 Samples	Nonlesional atopic dermatitis skin is characterized by broad terminal differentiation defects and variable immune abnormalities.	J Allergy Clin Immunol.
atopic dermatitis	GPR115	Expression profiling by array, count, 3 development stage, 2 disease state, 11 individual sets	GPL570	GSE32924 Samples	Nonlesional atopic dermatitis skin is characterized by broad terminal differentiation defects and variable immune abnormalities.	J Allergy Clin Immunol.
atopic dermatitis	NDFIP2	Expression profiling by array, count, 3 development stage, 2 disease state, 11 individual sets	GPL570	GSE32924 Samples	Nonlesional atopic dermatitis skin is characterized by broad terminal differentiation defects and variable immune abnormalities.	J Allergy Clin Immunol.
atopic dermatitis	NA	Expression profiling by array, count, 3 development stage, 2 disease state, 11 individual sets	GPL570	GSE32924 Samples	Nonlesional atopic dermatitis skin is characterized by broad terminal differentiation defects and variable immune abnormalities.	J Allergy Clin Immunol.
atopic dermatitis	IDE	Expression profiling by array, count, 3 development stage, 2 disease state, 11 individual sets	GPL570	GSE32924 Samples	Nonlesional atopic dermatitis skin is characterized by broad terminal differentiation defects and variable immune abnormalities.	J Allergy Clin Immunol.
atopic dermatitis	YOD1	Expression profiling by array, count, 3 development stage, 2 disease state, 11 individual sets	GPL570	GSE32924 Samples	Nonlesional atopic dermatitis skin is characterized by broad terminal differentiation defects and variable immune abnormalities.	J Allergy Clin Immunol.
atopic dermatitis	AADACL2	Expression profiling by array, count, 3 development stage, 2 disease state, 11 individual sets	GPL570	GSE32924 Samples	Nonlesional atopic dermatitis skin is characterized by broad terminal differentiation defects and variable immune abnormalities.	J Allergy Clin Immunol.
atopic dermatitis	RORA	Expression profiling by array, count, 3 development stage, 2 disease state, 11 individual sets	GPL570	GSE32924 Samples	Nonlesional atopic dermatitis skin is characterized by broad terminal differentiation defects and variable immune abnormalities.	J Allergy Clin Immunol.
atopic dermatitis	ELOVL7	Expression profiling by array, count, 3 development stage, 2 disease state, 11 individual sets	GPL570	GSE32924 Samples	Nonlesional atopic dermatitis skin is characterized by broad terminal differentiation defects and variable immune abnormalities.	J Allergy Clin Immunol.
atopic dermatitis	ARL5A	Expression profiling by array, count, 3 development stage, 2 disease state, 11 individual sets	GPL570	GSE32924 Samples	Nonlesional atopic dermatitis skin is characterized by broad terminal differentiation defects and variable immune abnormalities.	J Allergy Clin Immunol.
atopic dermatitis	KRT80	Expression profiling by array, count, 3 development stage, 2 disease state, 11 individual sets	GPL570	GSE32924 Samples	Nonlesional atopic dermatitis skin is characterized by broad terminal differentiation defects and variable immune abnormalities.	J Allergy Clin Immunol.
atopic dermatitis	SERPINB2	Expression profiling by array, count, 3 development stage, 2 disease state, 11 individual sets	GPL570	GSE32924 Samples	Nonlesional atopic dermatitis skin is characterized by broad terminal differentiation defects and variable immune abnormalities.	J Allergy Clin Immunol.
atopic dermatitis	XG	Expression profiling by array, count, 3 development stage, 2 disease state, 11 individual sets	GPL570	GSE32924 Samples	Nonlesional atopic dermatitis skin is characterized by broad terminal differentiation defects and variable immune abnormalities.	J Allergy Clin Immunol.
atopic dermatitis	EMB	Expression profiling by array, count, 3 development stage, 2 disease state, 11 individual sets	GPL570	GSE32924 Samples	Nonlesional atopic dermatitis skin is characterized by broad terminal differentiation defects and variable immune abnormalities.	J Allergy Clin Immunol.
atopic dermatitis	BLMH	Expression profiling by array, count, 3 development stage, 2 disease state, 11 individual sets	GPL570	GSE32924 Samples	Nonlesional atopic dermatitis skin is characterized by broad terminal differentiation defects and variable immune abnormalities.	J Allergy Clin Immunol.
atopic dermatitis	CTSL2	Expression profiling by array, count, 3 development stage, 2 disease state, 11 individual sets	GPL570	GSE32924 Samples	Nonlesional atopic dermatitis skin is characterized by broad terminal differentiation defects and variable immune abnormalities.	J Allergy Clin Immunol.
atopic dermatitis	SCEL	Expression profiling by array, count, 3 development stage, 2 disease state, 11 individual sets	GPL570	GSE32924 Samples	Nonlesional atopic dermatitis skin is characterized by broad terminal differentiation defects and variable immune abnormalities.	J Allergy Clin Immunol.
atopic dermatitis	ELMOD1	Expression profiling by array, count, 3 development stage, 2 disease state, 11 individual sets	GPL570	GSE32924 Samples	Nonlesional atopic dermatitis skin is characterized by broad terminal differentiation defects and variable immune abnormalities.	J Allergy Clin Immunol.
atopic dermatitis	LMBRD1	Expression profiling by array, count, 3 development stage, 2 disease state, 11 individual sets	GPL570	GSE32924 Samples	Nonlesional atopic dermatitis skin is characterized by broad terminal differentiation defects and variable immune abnormalities.	J Allergy Clin Immunol.
atopic dermatitis	C4ORF3	Expression profiling by array, count, 3 development stage, 2 disease state, 11 individual sets	GPL570	GSE32924 Samples	Nonlesional atopic dermatitis skin is characterized by broad terminal differentiation defects and variable immune abnormalities.	J Allergy Clin Immunol.

atopic dermatitis	ARIH1	Expression profiling by array, count, 3 development stage, 2 disease state, 11 individual sets	GPL570	GSE32924 Samples	Nonlesional atopic dermatitis skin is characterized by broad terminal differentiation defects and variable immune abnormalities.	J Allergy Clin Immunol.
atopic dermatitis	IDS	Expression profiling by array, count, 3 development stage, 2 disease state, 11 individual sets	GPL570	GSE32924 Samples	Nonlesional atopic dermatitis skin is characterized by broad terminal differentiation defects and variable immune abnormalities.	J Allergy Clin Immunol.
atopic dermatitis	NA	Expression profiling by array, count, 3 development stage, 2 disease state, 11 individual sets	GPL570	GSE32924 Samples	Nonlesional atopic dermatitis skin is characterized by broad terminal differentiation defects and variable immune abnormalities.	J Allergy Clin Immunol.
atopic dermatitis	PCNP	Expression profiling by array, count, 3 development stage, 2 disease state, 11 individual sets	GPL570	GSE32924 Samples	Nonlesional atopic dermatitis skin is characterized by broad terminal differentiation defects and variable immune abnormalities.	J Allergy Clin Immunol.
atopic dermatitis	SWAP70	Expression profiling by array, count, 3 development stage, 2 disease state, 11 individual sets	GPL570	GSE32924 Samples	Nonlesional atopic dermatitis skin is characterized by broad terminal differentiation defects and variable immune abnormalities.	J Allergy Clin Immunol.
atopic dermatitis	FOXN3	Expression profiling by array, count, 3 development stage, 2 disease state, 11 individual sets	GPL570	GSE32924 Samples	Nonlesional atopic dermatitis skin is characterized by broad terminal differentiation defects and variable immune abnormalities.	J Allergy Clin Immunol.
atopic dermatitis	C1ORF96	Expression profiling by array, count, 3 development stage, 2 disease state, 11 individual sets	GPL570	GSE32924 Samples	Nonlesional atopic dermatitis skin is characterized by broad terminal differentiation defects and variable immune abnormalities.	J Allergy Clin Immunol.
atopic dermatitis	PTPN21	Expression profiling by array, count, 3 development stage, 2 disease state, 11 individual sets	GPL570	GSE32924 Samples	Nonlesional atopic dermatitis skin is characterized by broad terminal differentiation defects and variable immune abnormalities.	J Allergy Clin Immunol.
atopic dermatitis	MEGF9	Expression profiling by array, count, 3 development stage, 2 disease state, 11 individual sets	GPL570	GSE32924 Samples	Nonlesional atopic dermatitis skin is characterized by broad terminal differentiation defects and variable immune abnormalities.	J Allergy Clin Immunol.
atopic dermatitis	FGFR2	Expression profiling by array, count, 3 development stage, 2 disease state, 11 individual sets	GPL570	GSE32924 Samples	Nonlesional atopic dermatitis skin is characterized by broad terminal differentiation defects and variable immune abnormalities.	J Allergy Clin Immunol.
atopic dermatitis	GALNT1	Expression profiling by array, count, 3 development stage, 2 disease state, 11 individual sets	GPL570	GSE32924 Samples	Nonlesional atopic dermatitis skin is characterized by broad terminal differentiation defects and variable immune abnormalities.	J Allergy Clin Immunol.
atopic dermatitis	C12ORF29	Expression profiling by array, count, 3 development stage, 2 disease state, 11 individual sets	GPL570	GSE32924 Samples	Nonlesional atopic dermatitis skin is characterized by broad terminal differentiation defects and variable immune abnormalities.	J Allergy Clin Immunol.
atopic dermatitis	AHNAK	Expression profiling by array, count, 3 development stage, 2 disease state, 11 individual sets	GPL570	GSE32924 Samples	Nonlesional atopic dermatitis skin is characterized by broad terminal differentiation defects and variable immune abnormalities.	J Allergy Clin Immunol.
atopic dermatitis	CYP26B1	Expression profiling by array, count, 3 development stage, 2 disease state, 11 individual sets	GPL570	GSE32924 Samples	Nonlesional atopic dermatitis skin is characterized by broad terminal differentiation defects and variable immune abnormalities.	J Allergy Clin Immunol.
atopic dermatitis	ATP6AP2	Expression profiling by array, count, 3 development stage, 2 disease state, 11 individual sets	GPL570	GSE32924 Samples	Nonlesional atopic dermatitis skin is characterized by broad terminal differentiation defects and variable immune abnormalities.	J Allergy Clin Immunol.
atopic dermatitis	MAP1LC3B	Expression profiling by array, count, 3 development stage, 2 disease state, 11 individual sets	GPL570	GSE32924 Samples	Nonlesional atopic dermatitis skin is characterized by broad terminal differentiation defects and variable immune abnormalities.	J Allergy Clin Immunol.
atopic dermatitis	RAB1A	Expression profiling by array, count, 3 development stage, 2 disease state, 11 individual sets	GPL570	GSE32924 Samples	Nonlesional atopic dermatitis skin is characterized by broad terminal differentiation defects and variable immune abnormalities.	J Allergy Clin Immunol.
atopic dermatitis	NA	Expression profiling by array, count, 3 development stage, 2 disease state, 11 individual sets	GPL570	GSE32924 Samples	Nonlesional atopic dermatitis skin is characterized by broad terminal differentiation defects and variable immune abnormalities.	J Allergy Clin Immunol.
atopic dermatitis	SLC35F5	Expression profiling by array, count, 3 development stage, 2 disease state, 11 individual sets	GPL570	GSE32924 Samples	Nonlesional atopic dermatitis skin is characterized by broad terminal differentiation defects and variable immune abnormalities.	J Allergy Clin Immunol.
atopic dermatitis	UBL3	Expression profiling by array, count, 3 development stage, 2 disease state, 11 individual sets	GPL570	GSE32924 Samples	Nonlesional atopic dermatitis skin is characterized by broad terminal differentiation defects and variable immune abnormalities.	J Allergy Clin Immunol.
atopic dermatitis	CITED2	Expression profiling by array, count, 3 development stage, 2 disease state, 11 individual sets	GPL570	GSE32924 Samples	Nonlesional atopic dermatitis skin is characterized by broad terminal differentiation defects and variable immune abnormalities.	J Allergy Clin Immunol.
atopic dermatitis	NA	Expression profiling by array, count, 3 development stage, 2 disease state, 11 individual sets	GPL570	GSE32924 Samples	Nonlesional atopic dermatitis skin is characterized by broad terminal differentiation defects and variable immune abnormalities.	J Allergy Clin Immunol.
atopic dermatitis	ALDH3A2	Expression profiling by array, count, 3 development stage, 2 disease state, 11 individual sets	GPL570	GSE32924 Samples	Nonlesional atopic dermatitis skin is characterized by broad terminal differentiation defects and variable immune abnormalities.	J Allergy Clin Immunol.
atopic dermatitis	NA	Expression profiling by array, count, 3 development stage, 2 disease state, 11 individual sets	GPL570	GSE32924 Samples	Nonlesional atopic dermatitis skin is characterized by broad terminal differentiation defects and variable immune abnormalities.	J Allergy Clin Immunol.
atopic dermatitis	EGR3	Expression profiling by array, count, 3 development stage, 2 disease state, 11 individual sets	GPL570	GSE32924 Samples	Nonlesional atopic dermatitis skin is characterized by broad terminal differentiation defects and variable immune abnormalities.	J Allergy Clin Immunol.
atopic dermatitis	BNIP3L	Expression profiling by array, count, 3 development stage, 2 disease state, 11 individual sets	GPL570	GSE32924 Samples	Nonlesional atopic dermatitis skin is characterized by broad terminal differentiation defects and variable immune abnormalities.	J Allergy Clin Immunol.
atopic dermatitis	PALLD	Expression profiling by array, count, 3 development stage, 2 disease state, 11 individual sets	GPL570	GSE32924 Samples	Nonlesional atopic dermatitis skin is characterized by broad terminal differentiation defects and variable immune abnormalities.	J Allergy Clin Immunol.
atopic dermatitis	NEBL	Expression profiling by array, count, 3 development stage, 2 disease state, 11 individual sets	GPL570	GSE32924 Samples	Nonlesional atopic dermatitis skin is characterized by broad terminal differentiation defects and variable immune abnormalities.	J Allergy Clin Immunol.
atopic dermatitis	PDZD2	Expression profiling by array, count, 3 development stage, 2 disease state, 11 individual sets	GPL570	GSE32924 Samples	Nonlesional atopic dermatitis skin is characterized by broad terminal differentiation defects and variable immune abnormalities.	J Allergy Clin Immunol.
atopic dermatitis	NA	Expression profiling by array, count, 3 development stage, 2 disease state, 11 individual sets	GPL570	GSE32924 Samples	Nonlesional atopic dermatitis skin is characterized by broad terminal differentiation defects and variable immune abnormalities.	J Allergy Clin Immunol.
atopic dermatitis	SCEL	Expression profiling by array, count, 3 development stage, 2 disease state, 11 individual sets	GPL570	GSE32924 Samples	Nonlesional atopic dermatitis skin is characterized by broad terminal differentiation defects and variable immune abnormalities.	J Allergy Clin Immunol.
atopic dermatitis	YWHAZ	Expression profiling by array, count, 3 development stage, 2 disease state, 11 individual sets	GPL570	GSE32924 Samples	Nonlesional atopic dermatitis skin is characterized by broad terminal differentiation defects and variable immune abnormalities.	J Allergy Clin Immunol.
atopic dermatitis	HSP90AB1	Expression profiling by array, count, 3 development stage, 2 disease state, 11 individual sets	GPL570	GSE32924 Samples	Nonlesional atopic dermatitis skin is characterized by broad terminal differentiation defects and variable immune abnormalities.	J Allergy Clin Immunol.
atopic dermatitis	KRT23	Expression profiling by array, count, 3 development stage, 2 disease state, 11 individual sets	GPL570	GSE32924 Samples	Nonlesional atopic dermatitis skin is characterized by broad terminal differentiation defects and variable immune abnormalities.	J Allergy Clin Immunol.
atopic dermatitis	CHMP4C	Expression profiling by array, count, 3 development stage, 2 disease state, 11 individual sets	GPL570	GSE32924 Samples	Nonlesional atopic dermatitis skin is characterized by broad terminal differentiation defects and variable immune abnormalities.	J Allergy Clin Immunol.
atopic dermatitis	ELOVL4	Expression profiling by array, count, 3 development stage, 2 disease state, 11 individual sets	GPL570	GSE32924 Samples	Nonlesional atopic dermatitis skin is characterized by broad terminal differentiation defects and variable immune abnormalities.	J Allergy Clin Immunol.
atopic dermatitis	HMGCR	Expression profiling by array, count, 3 development stage, 2 disease state, 11 individual sets	GPL570	GSE32924 Samples	Nonlesional atopic dermatitis skin is characterized by broad terminal differentiation defects and variable immune abnormalities.	J Allergy Clin Immunol.
atopic dermatitis	MALAT1	Expression profiling by array, count, 3 development stage, 2 disease state, 11 individual sets	GPL570	GSE32924 Samples	Nonlesional atopic dermatitis skin is characterized by broad terminal differentiation defects and variable immune abnormalities.	J Allergy Clin Immunol.
atopic dermatitis	CHURC1	Expression profiling by array, count, 3 development stage, 2 disease state, 11 individual sets	GPL570	GSE32924 Samples	Nonlesional atopic dermatitis skin is characterized by broad terminal differentiation defects and variable immune abnormalities.	J Allergy Clin Immunol.
atopic dermatitis	NRP1	Expression profiling by array, count, 3 development stage, 2 disease state, 11 individual sets	GPL570	GSE32924 Samples	Nonlesional atopic dermatitis skin is characterized by broad terminal differentiation defects and variable immune abnormalities.	J Allergy Clin Immunol.
atopic dermatitis	NEGR1	Expression profiling by array, count, 3 development stage, 2 disease state, 11 individual sets	GPL570	GSE32924 Samples	Nonlesional atopic dermatitis skin is characterized by broad terminal differentiation defects and variable immune abnormalities.	J Allergy Clin Immunol.

atopic dermatitis	FAM85A	Expression profiling by array, count, 3 development stage, 2 disease state, 11 individual sets	GPL570	GSE32924 Samples	Nonlesional atopic dermatitis skin is characterized by broad terminal differentiation defects and variable immune abnormalities.	J Allergy Clin Immunol.
atopic dermatitis	LNX1	Expression profiling by array, count, 3 development stage, 2 disease state, 11 individual sets	GPL570	GSE32924 Samples	Nonlesional atopic dermatitis skin is characterized by broad terminal differentiation defects and variable immune abnormalities.	J Allergy Clin Immunol.
atopic dermatitis	F3	Expression profiling by array, count, 3 development stage, 2 disease state, 11 individual sets	GPL570	GSE32924 Samples	Nonlesional atopic dermatitis skin is characterized by broad terminal differentiation defects and variable immune abnormalities.	J Allergy Clin Immunol.
atopic dermatitis	TXNIP	Expression profiling by array, count, 3 development stage, 2 disease state, 11 individual sets	GPL570	GSE32924 Samples	Nonlesional atopic dermatitis skin is characterized by broad terminal differentiation defects and variable immune abnormalities.	J Allergy Clin Immunol.
atopic dermatitis	PTGDS	Expression profiling by array, count, 3 development stage, 2 disease state, 11 individual sets	GPL570	GSE32924 Samples	Nonlesional atopic dermatitis skin is characterized by broad terminal differentiation defects and variable immune abnormalities.	J Allergy Clin Immunol.
atopic dermatitis	OLFML3	Expression profiling by array, count, 3 development stage, 2 disease state, 11 individual sets	GPL570	GSE32924 Samples	Nonlesional atopic dermatitis skin is characterized by broad terminal differentiation defects and variable immune abnormalities.	J Allergy Clin Immunol.
atopic dermatitis	OSR2	Expression profiling by array, count, 3 development stage, 2 disease state, 11 individual sets	GPL570	GSE32924 Samples	Nonlesional atopic dermatitis skin is characterized by broad terminal differentiation defects and variable immune abnormalities.	J Allergy Clin Immunol.
atopic dermatitis	DCLK1	Expression profiling by array, count, 3 development stage, 2 disease state, 11 individual sets	GPL570	GSE32924 Samples	Nonlesional atopic dermatitis skin is characterized by broad terminal differentiation defects and variable immune abnormalities.	J Allergy Clin Immunol.
atopic dermatitis	CD55	Expression profiling by array, count, 3 development stage, 2 disease state, 11 individual sets	GPL570	GSE32924 Samples	Nonlesional atopic dermatitis skin is characterized by broad terminal differentiation defects and variable immune abnormalities.	J Allergy Clin Immunol.
atopic dermatitis	CD55	Expression profiling by array, count, 3 development stage, 2 disease state, 11 individual sets	GPL570	GSE32924 Samples	Nonlesional atopic dermatitis skin is characterized by broad terminal differentiation defects and variable immune abnormalities.	J Allergy Clin Immunol.
atopic dermatitis	FBLN1	Expression profiling by array, count, 3 development stage, 2 disease state, 11 individual sets	GPL570	GSE32924 Samples	Nonlesional atopic dermatitis skin is characterized by broad terminal differentiation defects and variable immune abnormalities.	J Allergy Clin Immunol.
atopic dermatitis	LOX	Expression profiling by array, count, 3 development stage, 2 disease state, 11 individual sets	GPL570	GSE32924 Samples	Nonlesional atopic dermatitis skin is characterized by broad terminal differentiation defects and variable immune abnormalities.	J Allergy Clin Immunol.
atopic dermatitis	AGTR1	Expression profiling by array, count, 3 development stage, 2 disease state, 11 individual sets	GPL570	GSE32924 Samples	Nonlesional atopic dermatitis skin is characterized by broad terminal differentiation defects and variable immune abnormalities.	J Allergy Clin Immunol.
atopic dermatitis	C5ORF46	Expression profiling by array, count, 3 development stage, 2 disease state, 11 individual sets	GPL570	GSE32924 Samples	Nonlesional atopic dermatitis skin is characterized by broad terminal differentiation defects and variable immune abnormalities.	J Allergy Clin Immunol.
atopic dermatitis	NBPF10	Expression profiling by array, count, 3 development stage, 2 disease state, 11 individual sets	GPL570	GSE32924 Samples	Nonlesional atopic dermatitis skin is characterized by broad terminal differentiation defects and variable immune abnormalities.	J Allergy Clin Immunol.
atopic dermatitis	UBXN4	Expression profiling by array, count, 3 development stage, 2 disease state, 11 individual sets	GPL570	GSE32924 Samples	Nonlesional atopic dermatitis skin is characterized by broad terminal differentiation defects and variable immune abnormalities.	J Allergy Clin Immunol.
atopic dermatitis	CCDC80	Expression profiling by array, count, 3 development stage, 2 disease state, 11 individual sets	GPL570	GSE32924 Samples	Nonlesional atopic dermatitis skin is characterized by broad terminal differentiation defects and variable immune abnormalities.	J Allergy Clin Immunol.
atopic dermatitis	SFRS2IP	Expression profiling by array, count, 3 development stage, 2 disease state, 11 individual sets	GPL570	GSE32924 Samples	Nonlesional atopic dermatitis skin is characterized by broad terminal differentiation defects and variable immune abnormalities.	J Allergy Clin Immunol.
atopic dermatitis	QSER1	Expression profiling by array, count, 3 development stage, 2 disease state, 11 individual sets	GPL570	GSE32924 Samples	Nonlesional atopic dermatitis skin is characterized by broad terminal differentiation defects and variable immune abnormalities.	J Allergy Clin Immunol.
atopic dermatitis	MALAT1	Expression profiling by array, count, 3 development stage, 2 disease state, 11 individual sets	GPL570	GSE32924 Samples	Nonlesional atopic dermatitis skin is characterized by broad terminal differentiation defects and variable immune abnormalities.	J Allergy Clin Immunol.
atopic dermatitis	WIF1	Expression profiling by array, count, 3 development stage, 2 disease state, 11 individual sets	GPL570	GSE32924 Samples	Nonlesional atopic dermatitis skin is characterized by broad terminal differentiation defects and variable immune abnormalities.	J Allergy Clin Immunol.
atopic dermatitis	FRMD6	Expression profiling by array, count, 3 development stage, 2 disease state, 11 individual sets	GPL570	GSE32924 Samples	Nonlesional atopic dermatitis skin is characterized by broad terminal differentiation defects and variable immune abnormalities.	J Allergy Clin Immunol.
atopic dermatitis	CMTM6	Expression profiling by array, count, 3 development stage, 2 disease state, 11 individual sets	GPL570	GSE32924 Samples	Nonlesional atopic dermatitis skin is characterized by broad terminal differentiation defects and variable immune abnormalities.	J Allergy Clin Immunol.
atopic dermatitis	CAPRIN1	Expression profiling by array, count, 3 development stage, 2 disease state, 11 individual sets	GPL570	GSE32924 Samples	Nonlesional atopic dermatitis skin is characterized by broad terminal differentiation defects and variable immune abnormalities.	J Allergy Clin Immunol.
atopic dermatitis	RAD23B	Expression profiling by array, count, 3 development stage, 2 disease state, 11 individual sets	GPL570	GSE32924 Samples	Nonlesional atopic dermatitis skin is characterized by broad terminal differentiation defects and variable immune abnormalities.	J Allergy Clin Immunol.
atopic dermatitis	SPINK5	Expression profiling by array, count, 3 development stage, 2 disease state, 11 individual sets	GPL570	GSE32924 Samples	Nonlesional atopic dermatitis skin is characterized by broad terminal differentiation defects and variable immune abnormalities.	J Allergy Clin Immunol.
atopic dermatitis	CTSK	Expression profiling by array, count, 3 development stage, 2 disease state, 11 individual sets	GPL570	GSE32924 Samples	Nonlesional atopic dermatitis skin is characterized by broad terminal differentiation defects and variable immune abnormalities.	J Allergy Clin Immunol.
atopic dermatitis	CD55	Expression profiling by array, count, 3 development stage, 2 disease state, 11 individual sets	GPL570	GSE32924 Samples	Nonlesional atopic dermatitis skin is characterized by broad terminal differentiation defects and variable immune abnormalities.	J Allergy Clin Immunol.
atopic dermatitis	GNAI1	Expression profiling by array, count, 3 development stage, 2 disease state, 11 individual sets	GPL570	GSE32924 Samples	Nonlesional atopic dermatitis skin is characterized by broad terminal differentiation defects and variable immune abnormalities.	J Allergy Clin Immunol.
atopic dermatitis	RAC1	Expression profiling by array, count, 3 development stage, 2 disease state, 11 individual sets	GPL570	GSE32924 Samples	Nonlesional atopic dermatitis skin is characterized by broad terminal differentiation defects and variable immune abnormalities.	J Allergy Clin Immunol.
atopic dermatitis	RHOA	Expression profiling by array, count, 3 development stage, 2 disease state, 11 individual sets	GPL570	GSE32924 Samples	Nonlesional atopic dermatitis skin is characterized by broad terminal differentiation defects and variable immune abnormalities.	J Allergy Clin Immunol.
atopic dermatitis	CAT	Expression profiling by array, count, 3 development stage, 2 disease state, 11 individual sets	GPL570	GSE32924 Samples	Nonlesional atopic dermatitis skin is characterized by broad terminal differentiation defects and variable immune abnormalities.	J Allergy Clin Immunol.
atopic dermatitis	ZNF750	Expression profiling by array, count, 3 development stage, 2 disease state, 11 individual sets	GPL570	GSE32924 Samples	Nonlesional atopic dermatitis skin is characterized by broad terminal differentiation defects and variable immune abnormalities.	J Allergy Clin Immunol.
atopic dermatitis	HSPC159	Expression profiling by array, count, 3 development stage, 2 disease state, 11 individual sets	GPL570	GSE32924 Samples	Nonlesional atopic dermatitis skin is characterized by broad terminal differentiation defects and variable immune abnormalities.	J Allergy Clin Immunol.
atopic dermatitis	MAL2	Expression profiling by array, count, 3 development stage, 2 disease state, 11 individual sets	GPL570	GSE32924 Samples	Nonlesional atopic dermatitis skin is characterized by broad terminal differentiation defects and variable immune abnormalities.	J Allergy Clin Immunol.
atopic dermatitis	HOPX	Expression profiling by array, count, 3 development stage, 2 disease state, 11 individual sets	GPL570	GSE32924 Samples	Nonlesional atopic dermatitis skin is characterized by broad terminal differentiation defects and variable immune abnormalities.	J Allergy Clin Immunol.
atopic dermatitis	DSG1	Expression profiling by array, count, 3 development stage, 2 disease state, 11 individual sets	GPL570	GSE32924 Samples	Nonlesional atopic dermatitis skin is characterized by broad terminal differentiation defects and variable immune abnormalities.	J Allergy Clin Immunol.
atopic dermatitis	DEGS1	Expression profiling by array, count, 3 development stage, 2 disease state, 11 individual sets	GPL570	GSE32924 Samples	Nonlesional atopic dermatitis skin is characterized by broad terminal differentiation defects and variable immune abnormalities.	J Allergy Clin Immunol.
atopic dermatitis	FLG	Expression profiling by array, count, 3 development stage, 2 disease state, 11 individual sets	GPL570	GSE32924 Samples	Nonlesional atopic dermatitis skin is characterized by broad terminal differentiation defects and variable immune abnormalities.	J Allergy Clin Immunol.
atopic dermatitis	SFRP2	Expression profiling by array, count, 3 development stage, 2 disease state, 11 individual sets	GPL570	GSE32924 Samples	Nonlesional atopic dermatitis skin is characterized by broad terminal differentiation defects and variable immune abnormalities.	J Allergy Clin Immunol.
atopic dermatitis	PPP1R14C	Expression profiling by array, count, 3 development stage, 2 disease state, 11 individual sets	GPL570	GSE32924 Samples	Nonlesional atopic dermatitis skin is characterized by broad terminal differentiation defects and variable immune abnormalities.	J Allergy Clin Immunol.

atopic dermatitis	GATM	Expression profiling by array, count, 3 development stage, 2 disease state, 11 individual sets	GPL570	GSE32924 Samples	Nonlesional atopic dermatitis skin is characterized by broad terminal differentiation defects and variable immune abnormalities.	J Allergy Clin Immunol.
atopic dermatitis	FBLN1	Expression profiling by array, count, 3 development stage, 2 disease state, 11 individual sets	GPL570	GSE32924 Samples	Nonlesional atopic dermatitis skin is characterized by broad terminal differentiation defects and variable immune abnormalities.	J Allergy Clin Immunol.
atopic dermatitis	CCDC80	Expression profiling by array, count, 3 development stage, 2 disease state, 11 individual sets	GPL570	GSE32924 Samples	Nonlesional atopic dermatitis skin is characterized by broad terminal differentiation defects and variable immune abnormalities.	J Allergy Clin Immunol.
atopic dermatitis	NA	Expression profiling by array, count, 3 development stage, 2 disease state, 11 individual sets	GPL570	GSE32924 Samples	Nonlesional atopic dermatitis skin is characterized by broad terminal differentiation defects and variable immune abnormalities.	J Allergy Clin Immunol.
atopic dermatitis	FBLN1	Expression profiling by array, count, 3 development stage, 2 disease state, 11 individual sets	GPL570	GSE32924 Samples	Nonlesional atopic dermatitis skin is characterized by broad terminal differentiation defects and variable immune abnormalities.	J Allergy Clin Immunol.
atopic dermatitis	LEPR	Expression profiling by array, count, 3 development stage, 2 disease state, 11 individual sets	GPL570	GSE32924 Samples	Nonlesional atopic dermatitis skin is characterized by broad terminal differentiation defects and variable immune abnormalities.	J Allergy Clin Immunol.
atopic dermatitis	H3F3B	Expression profiling by array, count, 3 development stage, 2 disease state, 11 individual sets	GPL570	GSE32924 Samples	Nonlesional atopic dermatitis skin is characterized by broad terminal differentiation defects and variable immune abnormalities.	J Allergy Clin Immunol.
atopic dermatitis	CALD1	Expression profiling by array, count, 3 development stage, 2 disease state, 11 individual sets	GPL570	GSE32924 Samples	Nonlesional atopic dermatitis skin is characterized by broad terminal differentiation defects and variable immune abnormalities.	J Allergy Clin Immunol.
atopic dermatitis	APP	Expression profiling by array, count, 3 development stage, 2 disease state, 11 individual sets	GPL570	GSE32924 Samples	Nonlesional atopic dermatitis skin is characterized by broad terminal differentiation defects and variable immune abnormalities.	J Allergy Clin Immunol.
atopic dermatitis	TIMP2	Expression profiling by array, count, 3 development stage, 2 disease state, 11 individual sets	GPL570	GSE32924 Samples	Nonlesional atopic dermatitis skin is characterized by broad terminal differentiation defects and variable immune abnormalities.	J Allergy Clin Immunol.
atopic dermatitis	CYBRD1	Expression profiling by array, count, 3 development stage, 2 disease state, 11 individual sets	GPL570	GSE32924 Samples	Nonlesional atopic dermatitis skin is characterized by broad terminal differentiation defects and variable immune abnormalities.	J Allergy Clin Immunol.
atopic dermatitis	PPAP2B	Expression profiling by array, count, 3 development stage, 2 disease state, 11 individual sets	GPL570	GSE32924 Samples	Nonlesional atopic dermatitis skin is characterized by broad terminal differentiation defects and variable immune abnormalities.	J Allergy Clin Immunol.
atopic dermatitis	RORA	Expression profiling by array, count, 3 development stage, 2 disease state, 11 individual sets	GPL570	GSE32924 Samples	Nonlesional atopic dermatitis skin is characterized by broad terminal differentiation defects and variable immune abnormalities.	J Allergy Clin Immunol.
atopic dermatitis	PGRMC2	Expression profiling by array, count, 3 development stage, 2 disease state, 11 individual sets	GPL570	GSE32924 Samples	Nonlesional atopic dermatitis skin is characterized by broad terminal differentiation defects and variable immune abnormalities.	J Allergy Clin Immunol.
atopic dermatitis	SSFA2	Expression profiling by array, count, 3 development stage, 2 disease state, 11 individual sets	GPL570	GSE32924 Samples	Nonlesional atopic dermatitis skin is characterized by broad terminal differentiation defects and variable immune abnormalities.	J Allergy Clin Immunol.
atopic dermatitis	CLDN1	Expression profiling by array, count, 3 development stage, 2 disease state, 11 individual sets	GPL570	GSE32924 Samples	Nonlesional atopic dermatitis skin is characterized by broad terminal differentiation defects and variable immune abnormalities.	J Allergy Clin Immunol.
atopic dermatitis	SCEL	Expression profiling by array, count, 3 development stage, 2 disease state, 11 individual sets	GPL570	GSE32924 Samples	Nonlesional atopic dermatitis skin is characterized by broad terminal differentiation defects and variable immune abnormalities.	J Allergy Clin Immunol.
atopic dermatitis	MALAT1	Expression profiling by array, count, 3 development stage, 2 disease state, 11 individual sets	GPL570	GSE32924 Samples	Nonlesional atopic dermatitis skin is characterized by broad terminal differentiation defects and variable immune abnormalities.	J Allergy Clin Immunol.
atopic dermatitis	NA	Expression profiling by array, count, 3 development stage, 2 disease state, 11 individual sets	GPL570	GSE32924 Samples	Nonlesional atopic dermatitis skin is characterized by broad terminal differentiation defects and variable immune abnormalities.	J Allergy Clin Immunol.
atopic dermatitis	HBB	Expression profiling by array, count, 3 development stage, 2 disease state, 11 individual sets	GPL570	GSE32924 Samples	Nonlesional atopic dermatitis skin is characterized by broad terminal differentiation defects and variable immune abnormalities.	J Allergy Clin Immunol.
atopic dermatitis	HBB	Expression profiling by array, count, 3 development stage, 2 disease state, 11 individual sets	GPL570	GSE32924 Samples	Nonlesional atopic dermatitis skin is characterized by broad terminal differentiation defects and variable immune abnormalities.	J Allergy Clin Immunol.
atopic dermatitis	HBB	Expression profiling by array, count, 3 development stage, 2 disease state, 11 individual sets	GPL570	GSE32924 Samples	Nonlesional atopic dermatitis skin is characterized by broad terminal differentiation defects and variable immune abnormalities.	J Allergy Clin Immunol.
atopic dermatitis	NA	Expression profiling by array, count, 3 development stage, 2 disease state, 11 individual sets	GPL570	GSE32924 Samples	Nonlesional atopic dermatitis skin is characterized by broad terminal differentiation defects and variable immune abnormalities.	J Allergy Clin Immunol.
atopic dermatitis	KPNA2	Expression profiling by array, count, 3 development stage, 2 disease state, 11 individual sets	GPL570	GSE32924 Samples	Nonlesional atopic dermatitis skin is characterized by broad terminal differentiation defects and variable immune abnormalities.	J Allergy Clin Immunol.
atopic dermatitis	PSME2	Expression profiling by array, count, 3 development stage, 2 disease state, 11 individual sets	GPL570	GSE32924 Samples	Nonlesional atopic dermatitis skin is characterized by broad terminal differentiation defects and variable immune abnormalities.	J Allergy Clin Immunol.
atopic dermatitis	TNFSF10	Expression profiling by array, count, 3 development stage, 2 disease state, 11 individual sets	GPL570	GSE32924 Samples	Nonlesional atopic dermatitis skin is characterized by broad terminal differentiation defects and variable immune abnormalities.	J Allergy Clin Immunol.
atopic dermatitis	TNC	Expression profiling by array, count, 3 development stage, 2 disease state, 11 individual sets	GPL570	GSE32924 Samples	Nonlesional atopic dermatitis skin is characterized by broad terminal differentiation defects and variable immune abnormalities.	J Allergy Clin Immunol.
atopic dermatitis	NFATC2IP	Expression profiling by array, count, 3 development stage, 2 disease state, 11 individual sets	GPL570	GSE32924 Samples	Nonlesional atopic dermatitis skin is characterized by broad terminal differentiation defects and variable immune abnormalities.	J Allergy Clin Immunol.
atopic dermatitis	SEMA3F	Expression profiling by array, count, 3 development stage, 2 disease state, 11 individual sets	GPL570	GSE32924 Samples	Nonlesional atopic dermatitis skin is characterized by broad terminal differentiation defects and variable immune abnormalities.	J Allergy Clin Immunol.
atopic dermatitis	C9ORF3	Expression profiling by array, count, 3 development stage, 2 disease state, 11 individual sets	GPL570	GSE32924 Samples	Nonlesional atopic dermatitis skin is characterized by broad terminal differentiation defects and variable immune abnormalities.	J Allergy Clin Immunol.
atopic dermatitis	CFLAR	Expression profiling by array, count, 3 development stage, 2 disease state, 11 individual sets	GPL570	GSE32924 Samples	Nonlesional atopic dermatitis skin is characterized by broad terminal differentiation defects and variable immune abnormalities.	J Allergy Clin Immunol.
atopic dermatitis	ANKRD9	Expression profiling by array, count, 3 development stage, 2 disease state, 11 individual sets	GPL570	GSE32924 Samples	Nonlesional atopic dermatitis skin is characterized by broad terminal differentiation defects and variable immune abnormalities.	J Allergy Clin Immunol.
atopic dermatitis	ITGB4	Expression profiling by array, count, 3 development stage, 2 disease state, 11 individual sets	GPL570	GSE32924 Samples	Nonlesional atopic dermatitis skin is characterized by broad terminal differentiation defects and variable immune abnormalities.	J Allergy Clin Immunol.
atopic dermatitis	TNS4	Expression profiling by array, count, 3 development stage, 2 disease state, 11 individual sets	GPL570	GSE32924 Samples	Nonlesional atopic dermatitis skin is characterized by broad terminal differentiation defects and variable immune abnormalities.	J Allergy Clin Immunol.
atopic dermatitis	DICER1	Expression profiling by array, count, 3 development stage, 2 disease state, 11 individual sets	GPL570	GSE32924 Samples	Nonlesional atopic dermatitis skin is characterized by broad terminal differentiation defects and variable immune abnormalities.	J Allergy Clin Immunol.
atopic dermatitis	SGK1	Expression profiling by array, count, 3 development stage, 2 disease state, 11 individual sets	GPL570	GSE32924 Samples	Nonlesional atopic dermatitis skin is characterized by broad terminal differentiation defects and variable immune abnormalities.	J Allergy Clin Immunol.
atopic dermatitis	PPIB	Expression profiling by array, count, 3 development stage, 2 disease state, 11 individual sets	GPL570	GSE32924 Samples	Nonlesional atopic dermatitis skin is characterized by broad terminal differentiation defects and variable immune abnormalities.	J Allergy Clin Immunol.
atopic dermatitis	NA	Expression profiling by array, count, 3 development stage, 2 disease state, 11 individual sets	GPL570	GSE32924 Samples	Nonlesional atopic dermatitis skin is characterized by broad terminal differentiation defects and variable immune abnormalities.	J Allergy Clin Immunol.
atopic dermatitis	MBNL1	Expression profiling by array, count, 3 development stage, 2 disease state, 11 individual sets	GPL570	GSE32924 Samples	Nonlesional atopic dermatitis skin is characterized by broad terminal differentiation defects and variable immune abnormalities.	J Allergy Clin Immunol.
atopic dermatitis	LOC641298	Expression profiling by array, count, 3 development stage, 2 disease state, 11 individual sets	GPL570	GSE32924 Samples	Nonlesional atopic dermatitis skin is characterized by broad terminal differentiation defects and variable immune abnormalities.	J Allergy Clin Immunol.
atopic dermatitis	S100A2	Expression profiling by array, count, 3 development stage, 2 disease state, 11 individual sets	GPL570	GSE32924 Samples	Nonlesional atopic dermatitis skin is characterized by broad terminal differentiation defects and variable immune abnormalities.	J Allergy Clin Immunol.

atopic dermatitis	DCN	Expression profiling by array, count, 3 development stage, 2 disease state, 11 individual sets	GPL570	GSE32924 Samples	Nonlesional atopic dermatitis skin is characterized by broad terminal differentiation defects and variable immune abnormalities.	J Allergy Clin Immunol.
atopic dermatitis	DCN	Expression profiling by array, count, 3 development stage, 2 disease state, 11 individual sets	GPL570	GSE32924 Samples	Nonlesional atopic dermatitis skin is characterized by broad terminal differentiation defects and variable immune abnormalities.	J Allergy Clin Immunol.
atopic dermatitis	TIMP2	Expression profiling by array, count, 3 development stage, 2 disease state, 11 individual sets	GPL570	GSE32924 Samples	Nonlesional atopic dermatitis skin is characterized by broad terminal differentiation defects and variable immune abnormalities.	J Allergy Clin Immunol.
atopic dermatitis	DCN	Expression profiling by array, count, 3 development stage, 2 disease state, 11 individual sets	GPL570	GSE32924 Samples	Nonlesional atopic dermatitis skin is characterized by broad terminal differentiation defects and variable immune abnormalities.	J Allergy Clin Immunol.
atopic dermatitis	DCN	Expression profiling by array, count, 3 development stage, 2 disease state, 11 individual sets	GPL570	GSE32924 Samples	Nonlesional atopic dermatitis skin is characterized by broad terminal differentiation defects and variable immune abnormalities.	J Allergy Clin Immunol.
atopic dermatitis	TMEM45A	Expression profiling by array, count, 3 development stage, 2 disease state, 11 individual sets	GPL570	GSE32924 Samples	Nonlesional atopic dermatitis skin is characterized by broad terminal differentiation defects and variable immune abnormalities.	J Allergy Clin Immunol.
atopic dermatitis	FLG2	Expression profiling by array, count, 3 development stage, 2 disease state, 11 individual sets	GPL570	GSE32924 Samples	Nonlesional atopic dermatitis skin is characterized by broad terminal differentiation defects and variable immune abnormalities.	J Allergy Clin Immunol.
atopic dermatitis	KRT16	Expression profiling by array, count, 3 development stage, 2 disease state, 11 individual sets	GPL570	GSE32924 Samples	Nonlesional atopic dermatitis skin is characterized by broad terminal differentiation defects and variable immune abnormalities.	J Allergy Clin Immunol.
atopic dermatitis	IDE	Expression profiling by array, count, 3 development stage, 2 disease state, 11 individual sets	GPL570	GSE32924 Samples	Nonlesional atopic dermatitis skin is characterized by broad terminal differentiation defects and variable immune abnormalities.	J Allergy Clin Immunol.
atopic dermatitis	ABCA12	Expression profiling by array, count, 3 development stage, 2 disease state, 11 individual sets	GPL570	GSE32924 Samples	Nonlesional atopic dermatitis skin is characterized by broad terminal differentiation defects and variable immune abnormalities.	J Allergy Clin Immunol.
atopic dermatitis	TPRG1	Expression profiling by array, count, 3 development stage, 2 disease state, 11 individual sets	GPL570	GSE32924 Samples	Nonlesional atopic dermatitis skin is characterized by broad terminal differentiation defects and variable immune abnormalities.	J Allergy Clin Immunol.
atopic dermatitis	KLK5	Expression profiling by array, count, 3 development stage, 2 disease state, 11 individual sets	GPL570	GSE32924 Samples	Nonlesional atopic dermatitis skin is characterized by broad terminal differentiation defects and variable immune abnormalities.	J Allergy Clin Immunol.
atopic dermatitis	POF1B	Expression profiling by array, count, 3 development stage, 2 disease state, 11 individual sets	GPL570	GSE32924 Samples	Nonlesional atopic dermatitis skin is characterized by broad terminal differentiation defects and variable immune abnormalities.	J Allergy Clin Immunol.
atopic dermatitis	KLK7	Expression profiling by array, count, 3 development stage, 2 disease state, 11 individual sets	GPL570	GSE32924 Samples	Nonlesional atopic dermatitis skin is characterized by broad terminal differentiation defects and variable immune abnormalities.	J Allergy Clin Immunol.
atopic dermatitis	LY6G6C	Expression profiling by array, count, 3 development stage, 2 disease state, 11 individual sets	GPL570	GSE32924 Samples	Nonlesional atopic dermatitis skin is characterized by broad terminal differentiation defects and variable immune abnormalities.	J Allergy Clin Immunol.
atopic dermatitis	CDSN	Expression profiling by array, count, 3 development stage, 2 disease state, 11 individual sets	GPL570	GSE32924 Samples	Nonlesional atopic dermatitis skin is characterized by broad terminal differentiation defects and variable immune abnormalities.	J Allergy Clin Immunol.
atopic dermatitis	SLURP1	Expression profiling by array, count, 3 development stage, 2 disease state, 11 individual sets	GPL570	GSE32924 Samples	Nonlesional atopic dermatitis skin is characterized by broad terminal differentiation defects and variable immune abnormalities.	J Allergy Clin Immunol.
atopic dermatitis	SERPINA12	Expression profiling by array, count, 3 development stage, 2 disease state, 11 individual sets	GPL570	GSE32924 Samples	Nonlesional atopic dermatitis skin is characterized by broad terminal differentiation defects and variable immune abnormalities.	J Allergy Clin Immunol.
atopic dermatitis	C1ORF68	Expression profiling by array, count, 3 development stage, 2 disease state, 11 individual sets	GPL570	GSE32924 Samples	Nonlesional atopic dermatitis skin is characterized by broad terminal differentiation defects and variable immune abnormalities.	J Allergy Clin Immunol.
atopic dermatitis	EREG	Expression profiling by array, count, 3 development stage, 2 disease state, 11 individual sets	GPL570	GSE32924 Samples	Nonlesional atopic dermatitis skin is characterized by broad terminal differentiation defects and variable immune abnormalities.	J Allergy Clin Immunol.
atopic dermatitis	SERPINB7	Expression profiling by array, count, 3 development stage, 2 disease state, 11 individual sets	GPL570	GSE32924 Samples	Nonlesional atopic dermatitis skin is characterized by broad terminal differentiation defects and variable immune abnormalities.	J Allergy Clin Immunol.
atopic dermatitis	LOR	Expression profiling by array, count, 3 development stage, 2 disease state, 11 individual sets	GPL570	GSE32924 Samples	Nonlesional atopic dermatitis skin is characterized by broad terminal differentiation defects and variable immune abnormalities.	J Allergy Clin Immunol.
atopic dermatitis	LCE1B	Expression profiling by array, count, 3 development stage, 2 disease state, 11 individual sets	GPL570	GSE32924 Samples	Nonlesional atopic dermatitis skin is characterized by broad terminal differentiation defects and variable immune abnormalities.	J Allergy Clin Immunol.
atopic dermatitis	CST6	Expression profiling by array, count, 3 development stage, 2 disease state, 11 individual sets	GPL570	GSE32924 Samples	Nonlesional atopic dermatitis skin is characterized by broad terminal differentiation defects and variable immune abnormalities.	J Allergy Clin Immunol.
atopic dermatitis	ASPRV1	Expression profiling by array, count, 3 development stage, 2 disease state, 11 individual sets	GPL570	GSE32924 Samples	Nonlesional atopic dermatitis skin is characterized by broad terminal differentiation defects and variable immune abnormalities.	J Allergy Clin Immunol.
atopic dermatitis	LCE2B	Expression profiling by array, count, 3 development stage, 2 disease state, 11 individual sets	GPL570	GSE32924 Samples	Nonlesional atopic dermatitis skin is characterized by broad terminal differentiation defects and variable immune abnormalities.	J Allergy Clin Immunol.
atopic dermatitis	COL6A6	Expression profiling by array, count, 3 development stage, 2 disease state, 11 individual sets	GPL570	GSE32924 Samples	Nonlesional atopic dermatitis skin is characterized by broad terminal differentiation defects and variable immune abnormalities.	J Allergy Clin Immunol.
atopic dermatitis	LTF	Expression profiling by array, count, 3 development stage, 2 disease state, 11 individual sets	GPL570	GSE32924 Samples	Nonlesional atopic dermatitis skin is characterized by broad terminal differentiation defects and variable immune abnormalities.	J Allergy Clin Immunol.
atopic dermatitis	SLC38A7	Expression profiling by array, count, 3 development stage, 2 disease state, 11 individual sets	GPL570	GSE32924 Samples	Nonlesional atopic dermatitis skin is characterized by broad terminal differentiation defects and variable immune abnormalities.	J Allergy Clin Immunol.
atopic dermatitis	KPTN	Expression profiling by array, count, 3 development stage, 2 disease state, 11 individual sets	GPL570	GSE32924 Samples	Nonlesional atopic dermatitis skin is characterized by broad terminal differentiation defects and variable immune abnormalities.	J Allergy Clin Immunol.
atopic dermatitis	LZTS1	Expression profiling by array, count, 3 development stage, 2 disease state, 11 individual sets	GPL570	GSE32924 Samples	Nonlesional atopic dermatitis skin is characterized by broad terminal differentiation defects and variable immune abnormalities.	J Allergy Clin Immunol.
atopic dermatitis	CLN6	Expression profiling by array, count, 3 development stage, 2 disease state, 11 individual sets	GPL570	GSE32924 Samples	Nonlesional atopic dermatitis skin is characterized by broad terminal differentiation defects and variable immune abnormalities.	J Allergy Clin Immunol.
atopic dermatitis	ATAD3B	Expression profiling by array, count, 3 development stage, 2 disease state, 11 individual sets	GPL570	GSE32924 Samples	Nonlesional atopic dermatitis skin is characterized by broad terminal differentiation defects and variable immune abnormalities.	J Allergy Clin Immunol.
atopic dermatitis	PRMT7	Expression profiling by array, count, 3 development stage, 2 disease state, 11 individual sets	GPL570	GSE32924 Samples	Nonlesional atopic dermatitis skin is characterized by broad terminal differentiation defects and variable immune abnormalities.	J Allergy Clin Immunol.
atopic dermatitis	SPR	Expression profiling by array, count, 3 development stage, 2 disease state, 11 individual sets	GPL570	GSE32924 Samples	Nonlesional atopic dermatitis skin is characterized by broad terminal differentiation defects and variable immune abnormalities.	J Allergy Clin Immunol.
atopic dermatitis	TMEM160	Expression profiling by array, count, 3 development stage, 2 disease state, 11 individual sets	GPL570	GSE32924 Samples	Nonlesional atopic dermatitis skin is characterized by broad terminal differentiation defects and variable immune abnormalities.	J Allergy Clin Immunol.
atopic dermatitis	ZDHHC12	Expression profiling by array, count, 3 development stage, 2 disease state, 11 individual sets	GPL570	GSE32924 Samples	Nonlesional atopic dermatitis skin is characterized by broad terminal differentiation defects and variable immune abnormalities.	J Allergy Clin Immunol.
atopic dermatitis	NCLN	Expression profiling by array, count, 3 development stage, 2 disease state, 11 individual sets	GPL570	GSE32924 Samples	Nonlesional atopic dermatitis skin is characterized by broad terminal differentiation defects and variable immune abnormalities.	J Allergy Clin Immunol.
atopic dermatitis	NA	Expression profiling by array, count, 3 development stage, 2 disease state, 11 individual sets	GPL570	GSE32924 Samples	Nonlesional atopic dermatitis skin is characterized by broad terminal differentiation defects and variable immune abnormalities.	J Allergy Clin Immunol.
atopic dermatitis	PVT1	Expression profiling by array, count, 3 development stage, 2 disease state, 11 individual sets	GPL570	GSE32924 Samples	Nonlesional atopic dermatitis skin is characterized by broad terminal differentiation defects and variable immune abnormalities.	J Allergy Clin Immunol.
atopic dermatitis	SERTAD4	Expression profiling by array, count, 3 development stage, 2 disease state, 11 individual sets	GPL570	GSE32924 Samples	Nonlesional atopic dermatitis skin is characterized by broad terminal differentiation defects and variable immune abnormalities.	J Allergy Clin Immunol.

atopic dermatitis	GRK6	Expression profiling by array, count, 3 development stage, 2 disease state, 11 individual sets	GPL570	GSE32924 Samples	Nonlesional atopic dermatitis skin is characterized by broad terminal differentiation defects and variable immune abnormalities.	J Allergy Clin Immunol.
atopic dermatitis	PITPNC1	Expression profiling by array, count, 3 development stage, 2 disease state, 11 individual sets	GPL570	GSE32924 Samples	Nonlesional atopic dermatitis skin is characterized by broad terminal differentiation defects and variable immune abnormalities.	J Allergy Clin Immunol.
atopic dermatitis	PAFAH1B3	Expression profiling by array, count, 3 development stage, 2 disease state, 11 individual sets	GPL570	GSE32924 Samples	Nonlesional atopic dermatitis skin is characterized by broad terminal differentiation defects and variable immune abnormalities.	J Allergy Clin Immunol.
atopic dermatitis	RPS6KA1	Expression profiling by array, count, 3 development stage, 2 disease state, 11 individual sets	GPL570	GSE32924 Samples	Nonlesional atopic dermatitis skin is characterized by broad terminal differentiation defects and variable immune abnormalities.	J Allergy Clin Immunol.
atopic dermatitis	RECQL4	Expression profiling by array, count, 3 development stage, 2 disease state, 11 individual sets	GPL570	GSE32924 Samples	Nonlesional atopic dermatitis skin is characterized by broad terminal differentiation defects and variable immune abnormalities.	J Allergy Clin Immunol.
atopic dermatitis	CDT1	Expression profiling by array, count, 3 development stage, 2 disease state, 11 individual sets	GPL570	GSE32924 Samples	Nonlesional atopic dermatitis skin is characterized by broad terminal differentiation defects and variable immune abnormalities.	J Allergy Clin Immunol.
atopic dermatitis	CISH	Expression profiling by array, count, 3 development stage, 2 disease state, 11 individual sets	GPL570	GSE32924 Samples	Nonlesional atopic dermatitis skin is characterized by broad terminal differentiation defects and variable immune abnormalities.	J Allergy Clin Immunol.
atopic dermatitis	MIR17HG	Expression profiling by array, count, 3 development stage, 2 disease state, 11 individual sets	GPL570	GSE32924 Samples	Nonlesional atopic dermatitis skin is characterized by broad terminal differentiation defects and variable immune abnormalities.	J Allergy Clin Immunol.
atopic dermatitis	SOCS1	Expression profiling by array, count, 3 development stage, 2 disease state, 11 individual sets	GPL570	GSE32924 Samples	Nonlesional atopic dermatitis skin is characterized by broad terminal differentiation defects and variable immune abnormalities.	J Allergy Clin Immunol.
atopic dermatitis	MAP3K14	Expression profiling by array, count, 3 development stage, 2 disease state, 11 individual sets	GPL570	GSE32924 Samples	Nonlesional atopic dermatitis skin is characterized by broad terminal differentiation defects and variable immune abnormalities.	J Allergy Clin Immunol.
atopic dermatitis	MECOM	Expression profiling by array, count, 3 development stage, 2 disease state, 11 individual sets	GPL570	GSE32924 Samples	Nonlesional atopic dermatitis skin is characterized by broad terminal differentiation defects and variable immune abnormalities.	J Allergy Clin Immunol.
atopic dermatitis	PGF	Expression profiling by array, count, 3 development stage, 2 disease state, 11 individual sets	GPL570	GSE32924 Samples	Nonlesional atopic dermatitis skin is characterized by broad terminal differentiation defects and variable immune abnormalities.	J Allergy Clin Immunol.
atopic dermatitis	ALDH4A1	Expression profiling by array, count, 3 development stage, 2 disease state, 11 individual sets	GPL570	GSE32924 Samples	Nonlesional atopic dermatitis skin is characterized by broad terminal differentiation defects and variable immune abnormalities.	J Allergy Clin Immunol.
atopic dermatitis	PRR5	Expression profiling by array, count, 3 development stage, 2 disease state, 11 individual sets	GPL570	GSE32924 Samples	Nonlesional atopic dermatitis skin is characterized by broad terminal differentiation defects and variable immune abnormalities.	J Allergy Clin Immunol.
atopic dermatitis	PPP2R3B	Expression profiling by array, count, 3 development stage, 2 disease state, 11 individual sets	GPL570	GSE32924 Samples	Nonlesional atopic dermatitis skin is characterized by broad terminal differentiation defects and variable immune abnormalities.	J Allergy Clin Immunol.
atopic dermatitis	LOC728431	Expression profiling by array, count, 3 development stage, 2 disease state, 11 individual sets	GPL570	GSE32924 Samples	Nonlesional atopic dermatitis skin is characterized by broad terminal differentiation defects and variable immune abnormalities.	J Allergy Clin Immunol.
atopic dermatitis	CDK10	Expression profiling by array, count, 3 development stage, 2 disease state, 11 individual sets	GPL570	GSE32924 Samples	Nonlesional atopic dermatitis skin is characterized by broad terminal differentiation defects and variable immune abnormalities.	J Allergy Clin Immunol.
atopic dermatitis	EPN1	Expression profiling by array, count, 3 development stage, 2 disease state, 11 individual sets	GPL570	GSE32924 Samples	Nonlesional atopic dermatitis skin is characterized by broad terminal differentiation defects and variable immune abnormalities.	J Allergy Clin Immunol.
atopic dermatitis	TGIF1	Expression profiling by array, count, 3 development stage, 2 disease state, 11 individual sets	GPL570	GSE32924 Samples	Nonlesional atopic dermatitis skin is characterized by broad terminal differentiation defects and variable immune abnormalities.	J Allergy Clin Immunol.
atopic dermatitis	GMIP	Expression profiling by array, count, 3 development stage, 2 disease state, 11 individual sets	GPL570	GSE32924 Samples	Nonlesional atopic dermatitis skin is characterized by broad terminal differentiation defects and variable immune abnormalities.	J Allergy Clin Immunol.
atopic dermatitis	SH3BP2	Expression profiling by array, count, 3 development stage, 2 disease state, 11 individual sets	GPL570	GSE32924 Samples	Nonlesional atopic dermatitis skin is characterized by broad terminal differentiation defects and variable immune abnormalities.	J Allergy Clin Immunol.
atopic dermatitis	METTL1	Expression profiling by array, count, 3 development stage, 2 disease state, 11 individual sets	GPL570	GSE32924 Samples	Nonlesional atopic dermatitis skin is characterized by broad terminal differentiation defects and variable immune abnormalities.	J Allergy Clin Immunol.
atopic dermatitis	SBNO1	Expression profiling by array, count, 3 development stage, 2 disease state, 11 individual sets	GPL570	GSE32924 Samples	Nonlesional atopic dermatitis skin is characterized by broad terminal differentiation defects and variable immune abnormalities.	J Allergy Clin Immunol.
atopic dermatitis	UBA6	Expression profiling by array, count, 3 development stage, 2 disease state, 11 individual sets	GPL570	GSE32924 Samples	Nonlesional atopic dermatitis skin is characterized by broad terminal differentiation defects and variable immune abnormalities.	J Allergy Clin Immunol.
atopic dermatitis	DCLRE1C	Expression profiling by array, count, 3 development stage, 2 disease state, 11 individual sets	GPL570	GSE32924 Samples	Nonlesional atopic dermatitis skin is characterized by broad terminal differentiation defects and variable immune abnormalities.	J Allergy Clin Immunol.
atopic dermatitis	SNHG3	Expression profiling by array, count, 3 development stage, 2 disease state, 11 individual sets	GPL570	GSE32924 Samples	Nonlesional atopic dermatitis skin is characterized by broad terminal differentiation defects and variable immune abnormalities.	J Allergy Clin Immunol.
atopic dermatitis	NAPEPLD	Expression profiling by array, count, 3 development stage, 2 disease state, 11 individual sets	GPL570	GSE32924 Samples	Nonlesional atopic dermatitis skin is characterized by broad terminal differentiation defects and variable immune abnormalities.	J Allergy Clin Immunol.
atopic dermatitis	MYO10	Expression profiling by array, count, 3 development stage, 2 disease state, 11 individual sets	GPL570	GSE32924 Samples	Nonlesional atopic dermatitis skin is characterized by broad terminal differentiation defects and variable immune abnormalities.	J Allergy Clin Immunol.
atopic dermatitis	ARMC6	Expression profiling by array, count, 3 development stage, 2 disease state, 11 individual sets	GPL570	GSE32924 Samples	Nonlesional atopic dermatitis skin is characterized by broad terminal differentiation defects and variable immune abnormalities.	J Allergy Clin Immunol.
atopic dermatitis	NA	Expression profiling by array, count, 3 development stage, 2 disease state, 11 individual sets	GPL570	GSE32924 Samples	Nonlesional atopic dermatitis skin is characterized by broad terminal differentiation defects and variable immune abnormalities.	J Allergy Clin Immunol.
atopic dermatitis	MAP6D1	Expression profiling by array, count, 3 development stage, 2 disease state, 11 individual sets	GPL570	GSE32924 Samples	Nonlesional atopic dermatitis skin is characterized by broad terminal differentiation defects and variable immune abnormalities.	J Allergy Clin Immunol.
atopic dermatitis	MATK	Expression profiling by array, count, 3 development stage, 2 disease state, 11 individual sets	GPL570	GSE32924 Samples	Nonlesional atopic dermatitis skin is characterized by broad terminal differentiation defects and variable immune abnormalities.	J Allergy Clin Immunol.
atopic dermatitis	PAOX	Expression profiling by array, count, 3 development stage, 2 disease state, 11 individual sets	GPL570	GSE32924 Samples	Nonlesional atopic dermatitis skin is characterized by broad terminal differentiation defects and variable immune abnormalities.	J Allergy Clin Immunol.
atopic dermatitis	FAM53A	Expression profiling by array, count, 3 development stage, 2 disease state, 11 individual sets	GPL570	GSE32924 Samples	Nonlesional atopic dermatitis skin is characterized by broad terminal differentiation defects and variable immune abnormalities.	J Allergy Clin Immunol.
atopic dermatitis	CORO1B	Expression profiling by array, count, 3 development stage, 2 disease state, 11 individual sets	GPL570	GSE32924 Samples	Nonlesional atopic dermatitis skin is characterized by broad terminal differentiation defects and variable immune abnormalities.	J Allergy Clin Immunol.
atopic dermatitis	FBXL19	Expression profiling by array, count, 3 development stage, 2 disease state, 11 individual sets	GPL570	GSE32924 Samples	Nonlesional atopic dermatitis skin is characterized by broad terminal differentiation defects and variable immune abnormalities.	J Allergy Clin Immunol.
atopic dermatitis	NRTN	Expression profiling by array, count, 3 development stage, 2 disease state, 11 individual sets	GPL570	GSE32924 Samples	Nonlesional atopic dermatitis skin is characterized by broad terminal differentiation defects and variable immune abnormalities.	J Allergy Clin Immunol.
atopic dermatitis	CBLC	Expression profiling by array, count, 3 development stage, 2 disease state, 11 individual sets	GPL570	GSE32924 Samples	Nonlesional atopic dermatitis skin is characterized by broad terminal differentiation defects and variable immune abnormalities.	J Allergy Clin Immunol.
atopic dermatitis	FDPSL2A	Expression profiling by array, count, 3 development stage, 2 disease state, 11 individual sets	GPL570	GSE32924 Samples	Nonlesional atopic dermatitis skin is characterized by broad terminal differentiation defects and variable immune abnormalities.	J Allergy Clin Immunol.
atopic dermatitis	GOLGA7B	Expression profiling by array, count, 3 development stage, 2 disease state, 11 individual sets	GPL570	GSE32924 Samples	Nonlesional atopic dermatitis skin is characterized by broad terminal differentiation defects and variable immune abnormalities.	J Allergy Clin Immunol.
atopic dermatitis	PRKXP1	Expression profiling by array, count, 3 development stage, 2 disease state, 11 individual sets	GPL570	GSE32924 Samples	Nonlesional atopic dermatitis skin is characterized by broad terminal differentiation defects and variable immune abnormalities.	J Allergy Clin Immunol.

atopic dermatitis	NA	Expression profiling by array, count, 3 development stage, 2 disease state, 11 individual sets	GPL570	GSE32924 Samples	Nonlesional atopic dermatitis skin is characterized by broad terminal differentiation defects and variable immune abnormalities.	J Allergy Clin Immunol.
atopic dermatitis	C6ORF141	Expression profiling by array, count, 3 development stage, 2 disease state, 11 individual sets	GPL570	GSE32924 Samples	Nonlesional atopic dermatitis skin is characterized by broad terminal differentiation defects and variable immune abnormalities.	J Allergy Clin Immunol.
atopic dermatitis	CCNB1	Expression profiling by array, count, 3 development stage, 2 disease state, 11 individual sets	GPL570	GSE32924 Samples	Nonlesional atopic dermatitis skin is characterized by broad terminal differentiation defects and variable immune abnormalities.	J Allergy Clin Immunol.
atopic dermatitis	CDC20	Expression profiling by array, count, 3 development stage, 2 disease state, 11 individual sets	GPL570	GSE32924 Samples	Nonlesional atopic dermatitis skin is characterized by broad terminal differentiation defects and variable immune abnormalities.	J Allergy Clin Immunol.
atopic dermatitis	AURKA	Expression profiling by array, count, 3 development stage, 2 disease state, 11 individual sets	GPL570	GSE32924 Samples	Nonlesional atopic dermatitis skin is characterized by broad terminal differentiation defects and variable immune abnormalities.	J Allergy Clin Immunol.
atopic dermatitis	NEK2	Expression profiling by array, count, 3 development stage, 2 disease state, 11 individual sets	GPL570	GSE32924 Samples	Nonlesional atopic dermatitis skin is characterized by broad terminal differentiation defects and variable immune abnormalities.	J Allergy Clin Immunol.
atopic dermatitis	NDC80	Expression profiling by array, count, 3 development stage, 2 disease state, 11 individual sets	GPL570	GSE32924 Samples	Nonlesional atopic dermatitis skin is characterized by broad terminal differentiation defects and variable immune abnormalities.	J Allergy Clin Immunol.
atopic dermatitis	TIMELESS	Expression profiling by array, count, 3 development stage, 2 disease state, 11 individual sets	GPL570	GSE32924 Samples	Nonlesional atopic dermatitis skin is characterized by broad terminal differentiation defects and variable immune abnormalities.	J Allergy Clin Immunol.
atopic dermatitis	CRLF1	Expression profiling by array, count, 3 development stage, 2 disease state, 11 individual sets	GPL570	GSE32924 Samples	Nonlesional atopic dermatitis skin is characterized by broad terminal differentiation defects and variable immune abnormalities.	J Allergy Clin Immunol.
atopic dermatitis	AK2	Expression profiling by array, count, 3 development stage, 2 disease state, 11 individual sets	GPL570	GSE32924 Samples	Nonlesional atopic dermatitis skin is characterized by broad terminal differentiation defects and variable immune abnormalities.	J Allergy Clin Immunol.
atopic dermatitis	FAM120AOS	Expression profiling by array, count, 3 development stage, 2 disease state, 11 individual sets	GPL570	GSE32924 Samples	Nonlesional atopic dermatitis skin is characterized by broad terminal differentiation defects and variable immune abnormalities.	J Allergy Clin Immunol.
atopic dermatitis	LOC728806	Expression profiling by array, count, 3 development stage, 2 disease state, 11 individual sets	GPL570	GSE32924 Samples	Nonlesional atopic dermatitis skin is characterized by broad terminal differentiation defects and variable immune abnormalities.	J Allergy Clin Immunol.
atopic dermatitis	NA	Expression profiling by array, count, 3 development stage, 2 disease state, 11 individual sets	GPL570	GSE32924 Samples	Nonlesional atopic dermatitis skin is characterized by broad terminal differentiation defects and variable immune abnormalities.	J Allergy Clin Immunol.
atopic dermatitis	DKFZP434B20	Expression profiling by array, count, 3 development stage, 2 disease state, 11 individual sets	GPL570	GSE32924 Samples	Nonlesional atopic dermatitis skin is characterized by broad terminal differentiation defects and variable immune abnormalities.	J Allergy Clin Immunol.
atopic dermatitis	NA	Expression profiling by array, count, 3 development stage, 2 disease state, 11 individual sets	GPL570	GSE32924 Samples	Nonlesional atopic dermatitis skin is characterized by broad terminal differentiation defects and variable immune abnormalities.	J Allergy Clin Immunol.
atopic dermatitis	LOC654433	Expression profiling by array, count, 3 development stage, 2 disease state, 11 individual sets	GPL570	GSE32924 Samples	Nonlesional atopic dermatitis skin is characterized by broad terminal differentiation defects and variable immune abnormalities.	J Allergy Clin Immunol.
atopic dermatitis	MGC16385	Expression profiling by array, count, 3 development stage, 2 disease state, 11 individual sets	GPL570	GSE32924 Samples	Nonlesional atopic dermatitis skin is characterized by broad terminal differentiation defects and variable immune abnormalities.	J Allergy Clin Immunol.
atopic dermatitis	CD320	Expression profiling by array, count, 3 development stage, 2 disease state, 11 individual sets	GPL570	GSE32924 Samples	Nonlesional atopic dermatitis skin is characterized by broad terminal differentiation defects and variable immune abnormalities.	J Allergy Clin Immunol.
atopic dermatitis	FLJ45482	Expression profiling by array, count, 3 development stage, 2 disease state, 11 individual sets	GPL570	GSE32924 Samples	Nonlesional atopic dermatitis skin is characterized by broad terminal differentiation defects and variable immune abnormalities.	J Allergy Clin Immunol.
atopic dermatitis	BRF1	Expression profiling by array, count, 3 development stage, 2 disease state, 11 individual sets	GPL570	GSE32924 Samples	Nonlesional atopic dermatitis skin is characterized by broad terminal differentiation defects and variable immune abnormalities.	J Allergy Clin Immunol.
atopic dermatitis	NA	Expression profiling by array, count, 3 development stage, 2 disease state, 11 individual sets	GPL570	GSE32924 Samples	Nonlesional atopic dermatitis skin is characterized by broad terminal differentiation defects and variable immune abnormalities.	J Allergy Clin Immunol.
atopic dermatitis	FGR	Expression profiling by array, count, 3 development stage, 2 disease state, 11 individual sets	GPL570	GSE32924 Samples	Nonlesional atopic dermatitis skin is characterized by broad terminal differentiation defects and variable immune abnormalities.	J Allergy Clin Immunol.
atopic dermatitis	FKRP	Expression profiling by array, count, 3 development stage, 2 disease state, 11 individual sets	GPL570	GSE32924 Samples	Nonlesional atopic dermatitis skin is characterized by broad terminal differentiation defects and variable immune abnormalities.	J Allergy Clin Immunol.
atopic dermatitis	KLHL17	Expression profiling by array, count, 3 development stage, 2 disease state, 11 individual sets	GPL570	GSE32924 Samples	Nonlesional atopic dermatitis skin is characterized by broad terminal differentiation defects and variable immune abnormalities.	J Allergy Clin Immunol.
atopic dermatitis	HEXDC	Expression profiling by array, count, 3 development stage, 2 disease state, 11 individual sets	GPL570	GSE32924 Samples	Nonlesional atopic dermatitis skin is characterized by broad terminal differentiation defects and variable immune abnormalities.	J Allergy Clin Immunol.
atopic dermatitis	TAZ	Expression profiling by array, count, 3 development stage, 2 disease state, 11 individual sets	GPL570	GSE32924 Samples	Nonlesional atopic dermatitis skin is characterized by broad terminal differentiation defects and variable immune abnormalities.	J Allergy Clin Immunol.
atopic dermatitis	ZBTB48	Expression profiling by array, count, 3 development stage, 2 disease state, 11 individual sets	GPL570	GSE32924 Samples	Nonlesional atopic dermatitis skin is characterized by broad terminal differentiation defects and variable immune abnormalities.	J Allergy Clin Immunol.
atopic dermatitis	NUFIP1	Expression profiling by array, count, 3 development stage, 2 disease state, 11 individual sets	GPL570	GSE32924 Samples	Nonlesional atopic dermatitis skin is characterized by broad terminal differentiation defects and variable immune abnormalities.	J Allergy Clin Immunol.
atopic dermatitis	PHKG2	Expression profiling by array, count, 3 development stage, 2 disease state, 11 individual sets	GPL570	GSE32924 Samples	Nonlesional atopic dermatitis skin is characterized by broad terminal differentiation defects and variable immune abnormalities.	J Allergy Clin Immunol.
atopic dermatitis	NA	Expression profiling by array, count, 3 development stage, 2 disease state, 11 individual sets	GPL570	GSE32924 Samples	Nonlesional atopic dermatitis skin is characterized by broad terminal differentiation defects and variable immune abnormalities.	J Allergy Clin Immunol.
atopic dermatitis	KDM6B	Expression profiling by array, count, 3 development stage, 2 disease state, 11 individual sets	GPL570	GSE32924 Samples	Nonlesional atopic dermatitis skin is characterized by broad terminal differentiation defects and variable immune abnormalities.	J Allergy Clin Immunol.
atopic dermatitis	DOT1L	Expression profiling by array, count, 3 development stage, 2 disease state, 11 individual sets	GPL570	GSE32924 Samples	Nonlesional atopic dermatitis skin is characterized by broad terminal differentiation defects and variable immune abnormalities.	J Allergy Clin Immunol.
atopic dermatitis	LOC442075	Expression profiling by array, count, 3 development stage, 2 disease state, 11 individual sets	GPL570	GSE32924 Samples	Nonlesional atopic dermatitis skin is characterized by broad terminal differentiation defects and variable immune abnormalities.	J Allergy Clin Immunol.
atopic dermatitis	PPIL2	Expression profiling by array, count, 3 development stage, 2 disease state, 11 individual sets	GPL570	GSE32924 Samples	Nonlesional atopic dermatitis skin is characterized by broad terminal differentiation defects and variable immune abnormalities.	J Allergy Clin Immunol.
atopic dermatitis	NA	Expression profiling by array, count, 3 development stage, 2 disease state, 11 individual sets	GPL570	GSE32924 Samples	Nonlesional atopic dermatitis skin is characterized by broad terminal differentiation defects and variable immune abnormalities.	J Allergy Clin Immunol.
atopic dermatitis	ZBTB43	Expression profiling by array, count, 3 development stage, 2 disease state, 11 individual sets	GPL570	GSE32924 Samples	Nonlesional atopic dermatitis skin is characterized by broad terminal differentiation defects and variable immune abnormalities.	J Allergy Clin Immunol.
atopic dermatitis	NA	Expression profiling by array, count, 3 development stage, 2 disease state, 11 individual sets	GPL570	GSE32924 Samples	Nonlesional atopic dermatitis skin is characterized by broad terminal differentiation defects and variable immune abnormalities.	J Allergy Clin Immunol.
atopic dermatitis	MUDENG	Expression profiling by array, count, 3 development stage, 2 disease state, 11 individual sets	GPL570	GSE32924 Samples	Nonlesional atopic dermatitis skin is characterized by broad terminal differentiation defects and variable immune abnormalities.	J Allergy Clin Immunol.
atopic dermatitis	NA	Expression profiling by array, count, 3 development stage, 2 disease state, 11 individual sets	GPL570	GSE32924 Samples	Nonlesional atopic dermatitis skin is characterized by broad terminal differentiation defects and variable immune abnormalities.	J Allergy Clin Immunol.
atopic dermatitis	NA	Expression profiling by array, count, 3 development stage, 2 disease state, 11 individual sets	GPL570	GSE32924 Samples	Nonlesional atopic dermatitis skin is characterized by broad terminal differentiation defects and variable immune abnormalities.	J Allergy Clin Immunol.
atopic dermatitis	NA	Expression profiling by array, count, 3 development stage, 2 disease state, 11 individual sets	GPL570	GSE32924 Samples	Nonlesional atopic dermatitis skin is characterized by broad terminal differentiation defects and variable immune abnormalities.	J Allergy Clin Immunol.

atopic dermatitis	NA	Expression profiling by array, count, 3 development stage, 2 disease state, 11 individual sets	GPL570	GSE32924 Samples	Nonlesional atopic dermatitis skin is characterized by broad terminal differentiation defects and variable immune abnormalities.	J Allergy Clin Immunol.
atopic dermatitis	NA	Expression profiling by array, count, 3 development stage, 2 disease state, 11 individual sets	GPL570	GSE32924 Samples	Nonlesional atopic dermatitis skin is characterized by broad terminal differentiation defects and variable immune abnormalities.	J Allergy Clin Immunol.
atopic dermatitis	NA	Expression profiling by array, count, 3 development stage, 2 disease state, 11 individual sets	GPL570	GSE32924 Samples	Nonlesional atopic dermatitis skin is characterized by broad terminal differentiation defects and variable immune abnormalities.	J Allergy Clin Immunol.
atopic dermatitis	FGD3	Expression profiling by array, count, 3 development stage, 2 disease state, 11 individual sets	GPL570	GSE32924 Samples	Nonlesional atopic dermatitis skin is characterized by broad terminal differentiation defects and variable immune abnormalities.	J Allergy Clin Immunol.
atopic dermatitis	ANKRD13A	Expression profiling by array, count, 3 development stage, 2 disease state, 11 individual sets	GPL570	GSE32924 Samples	Nonlesional atopic dermatitis skin is characterized by broad terminal differentiation defects and variable immune abnormalities.	J Allergy Clin Immunol.
atopic dermatitis	BRD4	Expression profiling by array, count, 3 development stage, 2 disease state, 11 individual sets	GPL570	GSE32924 Samples	Nonlesional atopic dermatitis skin is characterized by broad terminal differentiation defects and variable immune abnormalities.	J Allergy Clin Immunol.
atopic dermatitis	NA	Expression profiling by array, count, 3 development stage, 2 disease state, 11 individual sets	GPL570	GSE32924 Samples	Nonlesional atopic dermatitis skin is characterized by broad terminal differentiation defects and variable immune abnormalities.	J Allergy Clin Immunol.
atopic dermatitis	SNX27	Expression profiling by array, count, 3 development stage, 2 disease state, 11 individual sets	GPL570	GSE32924 Samples	Nonlesional atopic dermatitis skin is characterized by broad terminal differentiation defects and variable immune abnormalities.	J Allergy Clin Immunol.
atopic dermatitis	NA	Expression profiling by array, count, 3 development stage, 2 disease state, 11 individual sets	GPL570	GSE32924 Samples	Nonlesional atopic dermatitis skin is characterized by broad terminal differentiation defects and variable immune abnormalities.	J Allergy Clin Immunol.
atopic dermatitis	KIAA1618	Expression profiling by array, count, 3 development stage, 2 disease state, 11 individual sets	GPL570	GSE32924 Samples	Nonlesional atopic dermatitis skin is characterized by broad terminal differentiation defects and variable immune abnormalities.	J Allergy Clin Immunol.
atopic dermatitis	NA	Expression profiling by array, count, 3 development stage, 2 disease state, 11 individual sets	GPL570	GSE32924 Samples	Nonlesional atopic dermatitis skin is characterized by broad terminal differentiation defects and variable immune abnormalities.	J Allergy Clin Immunol.
atopic dermatitis	IDS	Expression profiling by array, count, 3 development stage, 2 disease state, 11 individual sets	GPL570	GSE32924 Samples	Nonlesional atopic dermatitis skin is characterized by broad terminal differentiation defects and variable immune abnormalities.	J Allergy Clin Immunol.
atopic dermatitis	CNIH4	Expression profiling by array, count, 3 development stage, 2 disease state, 11 individual sets	GPL570	GSE32924 Samples	Nonlesional atopic dermatitis skin is characterized by broad terminal differentiation defects and variable immune abnormalities.	J Allergy Clin Immunol.
atopic dermatitis	NA	Expression profiling by array, count, 3 development stage, 2 disease state, 11 individual sets	GPL570	GSE32924 Samples	Nonlesional atopic dermatitis skin is characterized by broad terminal differentiation defects and variable immune abnormalities.	J Allergy Clin Immunol.
atopic dermatitis	NA	Expression profiling by array, count, 3 development stage, 2 disease state, 11 individual sets	GPL570	GSE32924 Samples	Nonlesional atopic dermatitis skin is characterized by broad terminal differentiation defects and variable immune abnormalities.	J Allergy Clin Immunol.
atopic dermatitis	NA	Expression profiling by array, count, 3 development stage, 2 disease state, 11 individual sets	GPL570	GSE32924 Samples	Nonlesional atopic dermatitis skin is characterized by broad terminal differentiation defects and variable immune abnormalities.	J Allergy Clin Immunol.
atopic dermatitis	KDM6B	Expression profiling by array, count, 3 development stage, 2 disease state, 11 individual sets	GPL570	GSE32924 Samples	Nonlesional atopic dermatitis skin is characterized by broad terminal differentiation defects and variable immune abnormalities.	J Allergy Clin Immunol.
atopic dermatitis	ZNF628	Expression profiling by array, count, 3 development stage, 2 disease state, 11 individual sets	GPL570	GSE32924 Samples	Nonlesional atopic dermatitis skin is characterized by broad terminal differentiation defects and variable immune abnormalities.	J Allergy Clin Immunol.
atopic dermatitis	IL18BP	Expression profiling by array, count, 3 development stage, 2 disease state, 11 individual sets	GPL570	GSE32924 Samples	Nonlesional atopic dermatitis skin is characterized by broad terminal differentiation defects and variable immune abnormalities.	J Allergy Clin Immunol.
atopic dermatitis	KCNAB2	Expression profiling by array, count, 3 development stage, 2 disease state, 11 individual sets	GPL570	GSE32924 Samples	Nonlesional atopic dermatitis skin is characterized by broad terminal differentiation defects and variable immune abnormalities.	J Allergy Clin Immunol.
atopic dermatitis	NA	Expression profiling by array, count, 3 development stage, 2 disease state, 11 individual sets	GPL570	GSE32924 Samples	Nonlesional atopic dermatitis skin is characterized by broad terminal differentiation defects and variable immune abnormalities.	J Allergy Clin Immunol.
atopic dermatitis	TPCN2	Expression profiling by array, count, 3 development stage, 2 disease state, 11 individual sets	GPL570	GSE32924 Samples	Nonlesional atopic dermatitis skin is characterized by broad terminal differentiation defects and variable immune abnormalities.	J Allergy Clin Immunol.
atopic dermatitis	SIRT2	Expression profiling by array, count, 3 development stage, 2 disease state, 11 individual sets	GPL570	GSE32924 Samples	Nonlesional atopic dermatitis skin is characterized by broad terminal differentiation defects and variable immune abnormalities.	J Allergy Clin Immunol.
atopic dermatitis	NUP210	Expression profiling by array, count, 3 development stage, 2 disease state, 11 individual sets	GPL570	GSE32924 Samples	Nonlesional atopic dermatitis skin is characterized by broad terminal differentiation defects and variable immune abnormalities.	J Allergy Clin Immunol.
atopic dermatitis	NA	Expression profiling by array, count, 3 development stage, 2 disease state, 11 individual sets	GPL570	GSE32924 Samples	Nonlesional atopic dermatitis skin is characterized by broad terminal differentiation defects and variable immune abnormalities.	J Allergy Clin Immunol.
atopic dermatitis	PSRC1	Expression profiling by array, count, 3 development stage, 2 disease state, 11 individual sets	GPL570	GSE32924 Samples	Nonlesional atopic dermatitis skin is characterized by broad terminal differentiation defects and variable immune abnormalities.	J Allergy Clin Immunol.
atopic dermatitis	GTSE1	Expression profiling by array, count, 3 development stage, 2 disease state, 11 individual sets	GPL570	GSE32924 Samples	Nonlesional atopic dermatitis skin is characterized by broad terminal differentiation defects and variable immune abnormalities.	J Allergy Clin Immunol.
atopic dermatitis	ZBTB1	Expression profiling by array, count, 3 development stage, 2 disease state, 11 individual sets	GPL570	GSE32924 Samples	Nonlesional atopic dermatitis skin is characterized by broad terminal differentiation defects and variable immune abnormalities.	J Allergy Clin Immunol.
atopic dermatitis	NA	Expression profiling by array, count, 3 development stage, 2 disease state, 11 individual sets	GPL570	GSE32924 Samples	Nonlesional atopic dermatitis skin is characterized by broad terminal differentiation defects and variable immune abnormalities.	J Allergy Clin Immunol.
atopic dermatitis	SAP30BP	Expression profiling by array, count, 3 development stage, 2 disease state, 11 individual sets	GPL570	GSE32924 Samples	Nonlesional atopic dermatitis skin is characterized by broad terminal differentiation defects and variable immune abnormalities.	J Allergy Clin Immunol.
atopic dermatitis	GCAT	Expression profiling by array, count, 3 development stage, 2 disease state, 11 individual sets	GPL570	GSE32924 Samples	Nonlesional atopic dermatitis skin is characterized by broad terminal differentiation defects and variable immune abnormalities.	J Allergy Clin Immunol.
atopic dermatitis	NA	Expression profiling by array, count, 3 development stage, 2 disease state, 11 individual sets	GPL570	GSE32924 Samples	Nonlesional atopic dermatitis skin is characterized by broad terminal differentiation defects and variable immune abnormalities.	J Allergy Clin Immunol.
atopic dermatitis	MC1R	Expression profiling by array, count, 3 development stage, 2 disease state, 11 individual sets	GPL570	GSE32924 Samples	Nonlesional atopic dermatitis skin is characterized by broad terminal differentiation defects and variable immune abnormalities.	J Allergy Clin Immunol.
atopic dermatitis	GGT1	Expression profiling by array, count, 3 development stage, 2 disease state, 11 individual sets	GPL570	GSE32924 Samples	Nonlesional atopic dermatitis skin is characterized by broad terminal differentiation defects and variable immune abnormalities.	J Allergy Clin Immunol.
atopic dermatitis	OPLAH	Expression profiling by array, count, 3 development stage, 2 disease state, 11 individual sets	GPL570	GSE32924 Samples	Nonlesional atopic dermatitis skin is characterized by broad terminal differentiation defects and variable immune abnormalities.	J Allergy Clin Immunol.
atopic dermatitis	C9ORF140	Expression profiling by array, count, 3 development stage, 2 disease state, 11 individual sets	GPL570	GSE32924 Samples	Nonlesional atopic dermatitis skin is characterized by broad terminal differentiation defects and variable immune abnormalities.	J Allergy Clin Immunol.
atopic dermatitis	PSKH1	Expression profiling by array, count, 3 development stage, 2 disease state, 11 individual sets	GPL570	GSE32924 Samples	Nonlesional atopic dermatitis skin is characterized by broad terminal differentiation defects and variable immune abnormalities.	J Allergy Clin Immunol.
atopic dermatitis	LOC284801	Expression profiling by array, count, 3 development stage, 2 disease state, 11 individual sets	GPL570	GSE32924 Samples	Nonlesional atopic dermatitis skin is characterized by broad terminal differentiation defects and variable immune abnormalities.	J Allergy Clin Immunol.
atopic dermatitis	HIPK2	Expression profiling by array, count, 3 development stage, 2 disease state, 11 individual sets	GPL570	GSE32924 Samples	Nonlesional atopic dermatitis skin is characterized by broad terminal differentiation defects and variable immune abnormalities.	J Allergy Clin Immunol.
atopic dermatitis	NA	Expression profiling by array, count, 3 development stage, 2 disease state, 11 individual sets	GPL570	GSE32924 Samples	Nonlesional atopic dermatitis skin is characterized by broad terminal differentiation defects and variable immune abnormalities.	J Allergy Clin Immunol.
atopic dermatitis	TRPM1	Expression profiling by array, count, 3 development stage, 2 disease state, 11 individual sets	GPL570	GSE32924 Samples	Nonlesional atopic dermatitis skin is characterized by broad terminal differentiation defects and variable immune abnormalities.	J Allergy Clin Immunol.

atopic dermatitis	TRPM1	Expression profiling by array, count, 3 development stage, 2 disease state, 11 individual sets	GPL570	GSE32924 Samples	Nonlesional atopic dermatitis skin is characterized by broad terminal differentiation defects and variable immune abnormalities.	J Allergy Clin Immunol.
atopic dermatitis	NA	Expression profiling by array, count, 3 development stage, 2 disease state, 11 individual sets	GPL570	GSE32924 Samples	Nonlesional atopic dermatitis skin is characterized by broad terminal differentiation defects and variable immune abnormalities.	J Allergy Clin Immunol.
atopic dermatitis	NA	Expression profiling by array, count, 3 development stage, 2 disease state, 11 individual sets	GPL570	GSE32924 Samples	Nonlesional atopic dermatitis skin is characterized by broad terminal differentiation defects and variable immune abnormalities.	J Allergy Clin Immunol.
atopic dermatitis	TK1	Expression profiling by array, count, 3 development stage, 2 disease state, 11 individual sets	GPL570	GSE32924 Samples	Nonlesional atopic dermatitis skin is characterized by broad terminal differentiation defects and variable immune abnormalities.	J Allergy Clin Immunol.
atopic dermatitis	TTK	Expression profiling by array, count, 3 development stage, 2 disease state, 11 individual sets	GPL570	GSE32924 Samples	Nonlesional atopic dermatitis skin is characterized by broad terminal differentiation defects and variable immune abnormalities.	J Allergy Clin Immunol.
atopic dermatitis	NCAPG	Expression profiling by array, count, 3 development stage, 2 disease state, 11 individual sets	GPL570	GSE32924 Samples	Nonlesional atopic dermatitis skin is characterized by broad terminal differentiation defects and variable immune abnormalities.	J Allergy Clin Immunol.
atopic dermatitis	IQGAP3	Expression profiling by array, count, 3 development stage, 2 disease state, 11 individual sets	GPL570	GSE32924 Samples	Nonlesional atopic dermatitis skin is characterized by broad terminal differentiation defects and variable immune abnormalities.	J Allergy Clin Immunol.
atopic dermatitis	KIF14	Expression profiling by array, count, 3 development stage, 2 disease state, 11 individual sets	GPL570	GSE32924 Samples	Nonlesional atopic dermatitis skin is characterized by broad terminal differentiation defects and variable immune abnormalities.	J Allergy Clin Immunol.
atopic dermatitis	MKI67	Expression profiling by array, count, 3 development stage, 2 disease state, 11 individual sets	GPL570	GSE32924 Samples	Nonlesional atopic dermatitis skin is characterized by broad terminal differentiation defects and variable immune abnormalities.	J Allergy Clin Immunol.
atopic dermatitis	ZNF668	Expression profiling by array, count, 3 development stage, 2 disease state, 11 individual sets	GPL570	GSE32924 Samples	Nonlesional atopic dermatitis skin is characterized by broad terminal differentiation defects and variable immune abnormalities.	J Allergy Clin Immunol.
atopic dermatitis	AURKB	Expression profiling by array, count, 3 development stage, 2 disease state, 11 individual sets	GPL570	GSE32924 Samples	Nonlesional atopic dermatitis skin is characterized by broad terminal differentiation defects and variable immune abnormalities.	J Allergy Clin Immunol.
atopic dermatitis	FOXM1	Expression profiling by array, count, 3 development stage, 2 disease state, 11 individual sets	GPL570	GSE32924 Samples	Nonlesional atopic dermatitis skin is characterized by broad terminal differentiation defects and variable immune abnormalities.	J Allergy Clin Immunol.
atopic dermatitis	NA	Expression profiling by array, count, 3 development stage, 2 disease state, 11 individual sets	GPL570	GSE32924 Samples	Nonlesional atopic dermatitis skin is characterized by broad terminal differentiation defects and variable immune abnormalities.	J Allergy Clin Immunol.
atopic dermatitis	GFER	Expression profiling by array, count, 3 development stage, 2 disease state, 11 individual sets	GPL570	GSE32924 Samples	Nonlesional atopic dermatitis skin is characterized by broad terminal differentiation defects and variable immune abnormalities.	J Allergy Clin Immunol.
atopic dermatitis	TPI1	Expression profiling by array, count, 3 development stage, 2 disease state, 11 individual sets	GPL570	GSE32924 Samples	Nonlesional atopic dermatitis skin is characterized by broad terminal differentiation defects and variable immune abnormalities.	J Allergy Clin Immunol.
atopic dermatitis	FANCA	Expression profiling by array, count, 3 development stage, 2 disease state, 11 individual sets	GPL570	GSE32924 Samples	Nonlesional atopic dermatitis skin is characterized by broad terminal differentiation defects and variable immune abnormalities.	J Allergy Clin Immunol.
atopic dermatitis	GALE	Expression profiling by array, count, 3 development stage, 2 disease state, 11 individual sets	GPL570	GSE32924 Samples	Nonlesional atopic dermatitis skin is characterized by broad terminal differentiation defects and variable immune abnormalities.	J Allergy Clin Immunol.
atopic dermatitis	NFKBIE	Expression profiling by array, count, 3 development stage, 2 disease state, 11 individual sets	GPL570	GSE32924 Samples	Nonlesional atopic dermatitis skin is characterized by broad terminal differentiation defects and variable immune abnormalities.	J Allergy Clin Immunol.
atopic dermatitis	IL27RA	Expression profiling by array, count, 3 development stage, 2 disease state, 11 individual sets	GPL570	GSE32924 Samples	Nonlesional atopic dermatitis skin is characterized by broad terminal differentiation defects and variable immune abnormalities.	J Allergy Clin Immunol.
atopic dermatitis	NA	Expression profiling by array, count, 3 development stage, 2 disease state, 11 individual sets	GPL570	GSE32924 Samples	Nonlesional atopic dermatitis skin is characterized by broad terminal differentiation defects and variable immune abnormalities.	J Allergy Clin Immunol.
atopic dermatitis	NA	Expression profiling by array, count, 3 development stage, 2 disease state, 11 individual sets	GPL570	GSE32924 Samples	Nonlesional atopic dermatitis skin is characterized by broad terminal differentiation defects and variable immune abnormalities.	J Allergy Clin Immunol.
atopic dermatitis	C1ORF69	Expression profiling by array, count, 3 development stage, 2 disease state, 11 individual sets	GPL570	GSE32924 Samples	Nonlesional atopic dermatitis skin is characterized by broad terminal differentiation defects and variable immune abnormalities.	J Allergy Clin Immunol.
atopic dermatitis	STYK1	Expression profiling by array, count, 3 development stage, 2 disease state, 11 individual sets	GPL570	GSE32924 Samples	Nonlesional atopic dermatitis skin is characterized by broad terminal differentiation defects and variable immune abnormalities.	J Allergy Clin Immunol.
atopic dermatitis	NA	Expression profiling by array, count, 3 development stage, 2 disease state, 11 individual sets	GPL570	GSE32924 Samples	Nonlesional atopic dermatitis skin is characterized by broad terminal differentiation defects and variable immune abnormalities.	J Allergy Clin Immunol.
atopic dermatitis	ARFGAP1	Expression profiling by array, count, 3 development stage, 2 disease state, 11 individual sets	GPL570	GSE32924 Samples	Nonlesional atopic dermatitis skin is characterized by broad terminal differentiation defects and variable immune abnormalities.	J Allergy Clin Immunol.
atopic dermatitis	NA	Expression profiling by array, count, 3 development stage, 2 disease state, 11 individual sets	GPL570	GSE32924 Samples	Nonlesional atopic dermatitis skin is characterized by broad terminal differentiation defects and variable immune abnormalities.	J Allergy Clin Immunol.
atopic dermatitis	C19ORF20	Expression profiling by array, count, 3 development stage, 2 disease state, 11 individual sets	GPL570	GSE32924 Samples	Nonlesional atopic dermatitis skin is characterized by broad terminal differentiation defects and variable immune abnormalities.	J Allergy Clin Immunol.
atopic dermatitis	PTP4A3	Expression profiling by array, count, 3 development stage, 2 disease state, 11 individual sets	GPL570	GSE32924 Samples	Nonlesional atopic dermatitis skin is characterized by broad terminal differentiation defects and variable immune abnormalities.	J Allergy Clin Immunol.
atopic dermatitis	NA	Expression profiling by array, count, 3 development stage, 2 disease state, 11 individual sets	GPL570	GSE32924 Samples	Nonlesional atopic dermatitis skin is characterized by broad terminal differentiation defects and variable immune abnormalities.	J Allergy Clin Immunol.
atopic dermatitis	NA	Expression profiling by array, count, 3 development stage, 2 disease state, 11 individual sets	GPL570	GSE32924 Samples	Nonlesional atopic dermatitis skin is characterized by broad terminal differentiation defects and variable immune abnormalities.	J Allergy Clin Immunol.
atopic dermatitis	SFRS15	Expression profiling by array, count, 3 development stage, 2 disease state, 11 individual sets	GPL570	GSE32924 Samples	Nonlesional atopic dermatitis skin is characterized by broad terminal differentiation defects and variable immune abnormalities.	J Allergy Clin Immunol.
atopic dermatitis	STAG1	Expression profiling by array, count, 3 development stage, 2 disease state, 11 individual sets	GPL570	GSE32924 Samples	Nonlesional atopic dermatitis skin is characterized by broad terminal differentiation defects and variable immune abnormalities.	J Allergy Clin Immunol.
atopic dermatitis	NA	Expression profiling by array, count, 3 development stage, 2 disease state, 11 individual sets	GPL570	GSE32924 Samples	Nonlesional atopic dermatitis skin is characterized by broad terminal differentiation defects and variable immune abnormalities.	J Allergy Clin Immunol.
atopic dermatitis	NA	Expression profiling by array, count, 3 development stage, 2 disease state, 11 individual sets	GPL570	GSE32924 Samples	Nonlesional atopic dermatitis skin is characterized by broad terminal differentiation defects and variable immune abnormalities.	J Allergy Clin Immunol.
atopic dermatitis	NA	Expression profiling by array, count, 3 development stage, 2 disease state, 11 individual sets	GPL570	GSE32924 Samples	Nonlesional atopic dermatitis skin is characterized by broad terminal differentiation defects and variable immune abnormalities.	J Allergy Clin Immunol.
atopic dermatitis	CORO6	Expression profiling by array, count, 3 development stage, 2 disease state, 11 individual sets	GPL570	GSE32924 Samples	Nonlesional atopic dermatitis skin is characterized by broad terminal differentiation defects and variable immune abnormalities.	J Allergy Clin Immunol.
atopic dermatitis	SLC38A9	Expression profiling by array, count, 3 development stage, 2 disease state, 11 individual sets	GPL570	GSE32924 Samples	Nonlesional atopic dermatitis skin is characterized by broad terminal differentiation defects and variable immune abnormalities.	J Allergy Clin Immunol.
atopic dermatitis	NA	Expression profiling by array, count, 3 development stage, 2 disease state, 11 individual sets	GPL570	GSE32924 Samples	Nonlesional atopic dermatitis skin is characterized by broad terminal differentiation defects and variable immune abnormalities.	J Allergy Clin Immunol.
atopic dermatitis	NA	Expression profiling by array, count, 3 development stage, 2 disease state, 11 individual sets	GPL570	GSE32924 Samples	Nonlesional atopic dermatitis skin is characterized by broad terminal differentiation defects and variable immune abnormalities.	J Allergy Clin Immunol.
atopic dermatitis	NA	Expression profiling by array, count, 3 development stage, 2 disease state, 11 individual sets	GPL570	GSE32924 Samples	Nonlesional atopic dermatitis skin is characterized by broad terminal differentiation defects and variable immune abnormalities.	J Allergy Clin Immunol.
atopic dermatitis	PTGR1	Expression profiling by array, count, 3 development stage, 2 disease state, 11 individual sets	GPL570	GSE32924 Samples	Nonlesional atopic dermatitis skin is characterized by broad terminal differentiation defects and variable immune abnormalities.	J Allergy Clin Immunol.

atopic dermatitis	CTTN	Expression profiling by array, count, 3 development stage, 2 disease state, 11 individual sets	GPL570	GSE32924 Samples	Nonlesional atopic dermatitis skin is characterized by broad terminal differentiation defects and variable immune abnormalities.	J Allergy Clin Immunol.
atopic dermatitis	NA	Expression profiling by array, count, 3 development stage, 2 disease state, 11 individual sets	GPL570	GSE32924 Samples	Nonlesional atopic dermatitis skin is characterized by broad terminal differentiation defects and variable immune abnormalities.	J Allergy Clin Immunol.
atopic dermatitis	NA	Expression profiling by array, count, 3 development stage, 2 disease state, 11 individual sets	GPL570	GSE32924 Samples	Nonlesional atopic dermatitis skin is characterized by broad terminal differentiation defects and variable immune abnormalities.	J Allergy Clin Immunol.
atopic dermatitis	NA	Expression profiling by array, count, 3 development stage, 2 disease state, 11 individual sets	GPL570	GSE32924 Samples	Nonlesional atopic dermatitis skin is characterized by broad terminal differentiation defects and variable immune abnormalities.	J Allergy Clin Immunol.
atopic dermatitis	NA	Expression profiling by array, count, 3 development stage, 2 disease state, 11 individual sets	GPL570	GSE32924 Samples	Nonlesional atopic dermatitis skin is characterized by broad terminal differentiation defects and variable immune abnormalities.	J Allergy Clin Immunol.
atopic dermatitis	NA	Expression profiling by array, count, 3 development stage, 2 disease state, 11 individual sets	GPL570	GSE32924 Samples	Nonlesional atopic dermatitis skin is characterized by broad terminal differentiation defects and variable immune abnormalities.	J Allergy Clin Immunol.
atopic dermatitis	GRB10	Expression profiling by array, count, 3 development stage, 2 disease state, 11 individual sets	GPL570	GSE32924 Samples	Nonlesional atopic dermatitis skin is characterized by broad terminal differentiation defects and variable immune abnormalities.	J Allergy Clin Immunol.
atopic dermatitis	NA	Expression profiling by array, count, 3 development stage, 2 disease state, 11 individual sets	GPL570	GSE32924 Samples	Nonlesional atopic dermatitis skin is characterized by broad terminal differentiation defects and variable immune abnormalities.	J Allergy Clin Immunol.
atopic dermatitis	ZNF37B	Expression profiling by array, count, 3 development stage, 2 disease state, 11 individual sets	GPL570	GSE32924 Samples	Nonlesional atopic dermatitis skin is characterized by broad terminal differentiation defects and variable immune abnormalities.	J Allergy Clin Immunol.
atopic dermatitis	RBM6	Expression profiling by array, count, 3 development stage, 2 disease state, 11 individual sets	GPL570	GSE32924 Samples	Nonlesional atopic dermatitis skin is characterized by broad terminal differentiation defects and variable immune abnormalities.	J Allergy Clin Immunol.
atopic dermatitis	TULP4	Expression profiling by array, count, 3 development stage, 2 disease state, 11 individual sets	GPL570	GSE32924 Samples	Nonlesional atopic dermatitis skin is characterized by broad terminal differentiation defects and variable immune abnormalities.	J Allergy Clin Immunol.
atopic dermatitis	NA	Expression profiling by array, count, 3 development stage, 2 disease state, 11 individual sets	GPL570	GSE32924 Samples	Nonlesional atopic dermatitis skin is characterized by broad terminal differentiation defects and variable immune abnormalities.	J Allergy Clin Immunol.
atopic dermatitis	CASZ1	Expression profiling by array, count, 3 development stage, 2 disease state, 11 individual sets	GPL570	GSE32924 Samples	Nonlesional atopic dermatitis skin is characterized by broad terminal differentiation defects and variable immune abnormalities.	J Allergy Clin Immunol.
atopic dermatitis	DENND1B	Expression profiling by array, count, 3 development stage, 2 disease state, 11 individual sets	GPL570	GSE32924 Samples	Nonlesional atopic dermatitis skin is characterized by broad terminal differentiation defects and variable immune abnormalities.	J Allergy Clin Immunol.
atopic dermatitis	HELLS	Expression profiling by array, count, 3 development stage, 2 disease state, 11 individual sets	GPL570	GSE32924 Samples	Nonlesional atopic dermatitis skin is characterized by broad terminal differentiation defects and variable immune abnormalities.	J Allergy Clin Immunol.
atopic dermatitis	USP36	Expression profiling by array, count, 3 development stage, 2 disease state, 11 individual sets	GPL570	GSE32924 Samples	Nonlesional atopic dermatitis skin is characterized by broad terminal differentiation defects and variable immune abnormalities.	J Allergy Clin Immunol.
atopic dermatitis	NA	Expression profiling by array, count, 3 development stage, 2 disease state, 11 individual sets	GPL570	GSE32924 Samples	Nonlesional atopic dermatitis skin is characterized by broad terminal differentiation defects and variable immune abnormalities.	J Allergy Clin Immunol.
atopic dermatitis	NA	Expression profiling by array, count, 3 development stage, 2 disease state, 11 individual sets	GPL570	GSE32924 Samples	Nonlesional atopic dermatitis skin is characterized by broad terminal differentiation defects and variable immune abnormalities.	J Allergy Clin Immunol.
atopic dermatitis	SOX4	Expression profiling by array, count, 3 development stage, 2 disease state, 11 individual sets	GPL570	GSE32924 Samples	Nonlesional atopic dermatitis skin is characterized by broad terminal differentiation defects and variable immune abnormalities.	J Allergy Clin Immunol.
atopic dermatitis	RASSF4	Expression profiling by array, count, 3 development stage, 2 disease state, 11 individual sets	GPL570	GSE32924 Samples	Nonlesional atopic dermatitis skin is characterized by broad terminal differentiation defects and variable immune abnormalities.	J Allergy Clin Immunol.
atopic dermatitis	EIF3C	Expression profiling by array, count, 3 development stage, 2 disease state, 11 individual sets	GPL570	GSE32924 Samples	Nonlesional atopic dermatitis skin is characterized by broad terminal differentiation defects and variable immune abnormalities.	J Allergy Clin Immunol.
atopic dermatitis	NA	Expression profiling by array, count, 3 development stage, 2 disease state, 11 individual sets	GPL570	GSE32924 Samples	Nonlesional atopic dermatitis skin is characterized by broad terminal differentiation defects and variable immune abnormalities.	J Allergy Clin Immunol.
atopic dermatitis	GGNBP2	Expression profiling by array, count, 3 development stage, 2 disease state, 11 individual sets	GPL570	GSE32924 Samples	Nonlesional atopic dermatitis skin is characterized by broad terminal differentiation defects and variable immune abnormalities.	J Allergy Clin Immunol.
atopic dermatitis	C5ORF28	Expression profiling by array, count, 3 development stage, 2 disease state, 11 individual sets	GPL570	GSE32924 Samples	Nonlesional atopic dermatitis skin is characterized by broad terminal differentiation defects and variable immune abnormalities.	J Allergy Clin Immunol.
atopic dermatitis	NA	Expression profiling by array, count, 3 development stage, 2 disease state, 11 individual sets	GPL570	GSE32924 Samples	Nonlesional atopic dermatitis skin is characterized by broad terminal differentiation defects and variable immune abnormalities.	J Allergy Clin Immunol.
atopic dermatitis	NA	Expression profiling by array, count, 3 development stage, 2 disease state, 11 individual sets	GPL570	GSE32924 Samples	Nonlesional atopic dermatitis skin is characterized by broad terminal differentiation defects and variable immune abnormalities.	J Allergy Clin Immunol.
atopic dermatitis	TCEA1	Expression profiling by array, count, 3 development stage, 2 disease state, 11 individual sets	GPL570	GSE32924 Samples	Nonlesional atopic dermatitis skin is characterized by broad terminal differentiation defects and variable immune abnormalities.	J Allergy Clin Immunol.
atopic dermatitis	PSIP1	Expression profiling by array, count, 3 development stage, 2 disease state, 11 individual sets	GPL570	GSE32924 Samples	Nonlesional atopic dermatitis skin is characterized by broad terminal differentiation defects and variable immune abnormalities.	J Allergy Clin Immunol.
atopic dermatitis	TROAP	Expression profiling by array, count, 3 development stage, 2 disease state, 11 individual sets	GPL570	GSE32924 Samples	Nonlesional atopic dermatitis skin is characterized by broad terminal differentiation defects and variable immune abnormalities.	J Allergy Clin Immunol.
atopic dermatitis	LOC440288	Expression profiling by array, count, 3 development stage, 2 disease state, 11 individual sets	GPL570	GSE32924 Samples	Nonlesional atopic dermatitis skin is characterized by broad terminal differentiation defects and variable immune abnormalities.	J Allergy Clin Immunol.
atopic dermatitis	UBE2CBP	Expression profiling by array, count, 3 development stage, 2 disease state, 11 individual sets	GPL570	GSE32924 Samples	Nonlesional atopic dermatitis skin is characterized by broad terminal differentiation defects and variable immune abnormalities.	J Allergy Clin Immunol.
atopic dermatitis	POLQ	Expression profiling by array, count, 3 development stage, 2 disease state, 11 individual sets	GPL570	GSE32924 Samples	Nonlesional atopic dermatitis skin is characterized by broad terminal differentiation defects and variable immune abnormalities.	J Allergy Clin Immunol.
atopic dermatitis	SARS2	Expression profiling by array, count, 3 development stage, 2 disease state, 11 individual sets	GPL570	GSE32924 Samples	Nonlesional atopic dermatitis skin is characterized by broad terminal differentiation defects and variable immune abnormalities.	J Allergy Clin Immunol.
atopic dermatitis	MTP18	Expression profiling by array, count, 3 development stage, 2 disease state, 11 individual sets	GPL570	GSE32924 Samples	Nonlesional atopic dermatitis skin is characterized by broad terminal differentiation defects and variable immune abnormalities.	J Allergy Clin Immunol.
atopic dermatitis	C16ORF35	Expression profiling by array, count, 3 development stage, 2 disease state, 11 individual sets	GPL570	GSE32924 Samples	Nonlesional atopic dermatitis skin is characterized by broad terminal differentiation defects and variable immune abnormalities.	J Allergy Clin Immunol.
atopic dermatitis	SLC19A1	Expression profiling by array, count, 3 development stage, 2 disease state, 11 individual sets	GPL570	GSE32924 Samples	Nonlesional atopic dermatitis skin is characterized by broad terminal differentiation defects and variable immune abnormalities.	J Allergy Clin Immunol.
atopic dermatitis	RABEP2	Expression profiling by array, count, 3 development stage, 2 disease state, 11 individual sets	GPL570	GSE32924 Samples	Nonlesional atopic dermatitis skin is characterized by broad terminal differentiation defects and variable immune abnormalities.	J Allergy Clin Immunol.
atopic dermatitis	STX4	Expression profiling by array, count, 3 development stage, 2 disease state, 11 individual sets	GPL570	GSE32924 Samples	Nonlesional atopic dermatitis skin is characterized by broad terminal differentiation defects and variable immune abnormalities.	J Allergy Clin Immunol.
atopic dermatitis	PPP2R5B	Expression profiling by array, count, 3 development stage, 2 disease state, 11 individual sets	GPL570	GSE32924 Samples	Nonlesional atopic dermatitis skin is characterized by broad terminal differentiation defects and variable immune abnormalities.	J Allergy Clin Immunol.
atopic dermatitis	USP35	Expression profiling by array, count, 3 development stage, 2 disease state, 11 individual sets	GPL570	GSE32924 Samples	Nonlesional atopic dermatitis skin is characterized by broad terminal differentiation defects and variable immune abnormalities.	J Allergy Clin Immunol.
atopic dermatitis	C19ORF28	Expression profiling by array, count, 3 development stage, 2 disease state, 11 individual sets	GPL570	GSE32924 Samples	Nonlesional atopic dermatitis skin is characterized by broad terminal differentiation defects and variable immune abnormalities.	J Allergy Clin Immunol.

atopic dermatitis	NA	Expression profiling by array, count, 3 development stage, 2 disease state, 11 individual sets	GPL570	GSE32924 Samples	Nonlesional atopic dermatitis skin is characterized by broad terminal differentiation defects and variable immune abnormalities.	J Allergy Clin Immunol.
atopic dermatitis	CHAF1A	Expression profiling by array, count, 3 development stage, 2 disease state, 11 individual sets	GPL570	GSE32924 Samples	Nonlesional atopic dermatitis skin is characterized by broad terminal differentiation defects and variable immune abnormalities.	J Allergy Clin Immunol.
atopic dermatitis	TUBGCP6	Expression profiling by array, count, 3 development stage, 2 disease state, 11 individual sets	GPL570	GSE32924 Samples	Nonlesional atopic dermatitis skin is characterized by broad terminal differentiation defects and variable immune abnormalities.	J Allergy Clin Immunol.
atopic dermatitis	NA	Expression profiling by array, count, 3 development stage, 2 disease state, 11 individual sets	GPL570	GSE32924 Samples	Nonlesional atopic dermatitis skin is characterized by broad terminal differentiation defects and variable immune abnormalities.	J Allergy Clin Immunol.
atopic dermatitis	FBXL6	Expression profiling by array, count, 3 development stage, 2 disease state, 11 individual sets	GPL570	GSE32924 Samples	Nonlesional atopic dermatitis skin is characterized by broad terminal differentiation defects and variable immune abnormalities.	J Allergy Clin Immunol.
atopic dermatitis	C3ORF62	Expression profiling by array, count, 3 development stage, 2 disease state, 11 individual sets	GPL570	GSE32924 Samples	Nonlesional atopic dermatitis skin is characterized by broad terminal differentiation defects and variable immune abnormalities.	J Allergy Clin Immunol.
atopic dermatitis	CCDC142	Expression profiling by array, count, 3 development stage, 2 disease state, 11 individual sets	GPL570	GSE32924 Samples	Nonlesional atopic dermatitis skin is characterized by broad terminal differentiation defects and variable immune abnormalities.	J Allergy Clin Immunol.
atopic dermatitis	TP73	Expression profiling by array, count, 3 development stage, 2 disease state, 11 individual sets	GPL570	GSE32924 Samples	Nonlesional atopic dermatitis skin is characterized by broad terminal differentiation defects and variable immune abnormalities.	J Allergy Clin Immunol.
atopic dermatitis	LOC100131096	Expression profiling by array, count, 3 development stage, 2 disease state, 11 individual sets	GPL570	GSE32924 Samples	Nonlesional atopic dermatitis skin is characterized by broad terminal differentiation defects and variable immune abnormalities.	J Allergy Clin Immunol.
atopic dermatitis	ABCC5	Expression profiling by array, count, 3 development stage, 2 disease state, 11 individual sets	GPL570	GSE32924 Samples	Nonlesional atopic dermatitis skin is characterized by broad terminal differentiation defects and variable immune abnormalities.	J Allergy Clin Immunol.
atopic dermatitis	FOXK2	Expression profiling by array, count, 3 development stage, 2 disease state, 11 individual sets	GPL570	GSE32924 Samples	Nonlesional atopic dermatitis skin is characterized by broad terminal differentiation defects and variable immune abnormalities.	J Allergy Clin Immunol.
atopic dermatitis	C1ORF159	Expression profiling by array, count, 3 development stage, 2 disease state, 11 individual sets	GPL570	GSE32924 Samples	Nonlesional atopic dermatitis skin is characterized by broad terminal differentiation defects and variable immune abnormalities.	J Allergy Clin Immunol.
atopic dermatitis	TGIF2	Expression profiling by array, count, 3 development stage, 2 disease state, 11 individual sets	GPL570	GSE32924 Samples	Nonlesional atopic dermatitis skin is characterized by broad terminal differentiation defects and variable immune abnormalities.	J Allergy Clin Immunol.
atopic dermatitis	CCDC61	Expression profiling by array, count, 3 development stage, 2 disease state, 11 individual sets	GPL570	GSE32924 Samples	Nonlesional atopic dermatitis skin is characterized by broad terminal differentiation defects and variable immune abnormalities.	J Allergy Clin Immunol.
atopic dermatitis	DGCR8	Expression profiling by array, count, 3 development stage, 2 disease state, 11 individual sets	GPL570	GSE32924 Samples	Nonlesional atopic dermatitis skin is characterized by broad terminal differentiation defects and variable immune abnormalities.	J Allergy Clin Immunol.
atopic dermatitis	NA	Expression profiling by array, count, 3 development stage, 2 disease state, 11 individual sets	GPL570	GSE32924 Samples	Nonlesional atopic dermatitis skin is characterized by broad terminal differentiation defects and variable immune abnormalities.	J Allergy Clin Immunol.
atopic dermatitis	ROGDI	Expression profiling by array, count, 3 development stage, 2 disease state, 11 individual sets	GPL570	GSE32924 Samples	Nonlesional atopic dermatitis skin is characterized by broad terminal differentiation defects and variable immune abnormalities.	J Allergy Clin Immunol.
atopic dermatitis	NA	Expression profiling by array, count, 3 development stage, 2 disease state, 11 individual sets	GPL570	GSE32924 Samples	Nonlesional atopic dermatitis skin is characterized by broad terminal differentiation defects and variable immune abnormalities.	J Allergy Clin Immunol.
atopic dermatitis	ZBTB42	Expression profiling by array, count, 3 development stage, 2 disease state, 11 individual sets	GPL570	GSE32924 Samples	Nonlesional atopic dermatitis skin is characterized by broad terminal differentiation defects and variable immune abnormalities.	J Allergy Clin Immunol.
atopic dermatitis	RAB15	Expression profiling by array, count, 3 development stage, 2 disease state, 11 individual sets	GPL570	GSE32924 Samples	Nonlesional atopic dermatitis skin is characterized by broad terminal differentiation defects and variable immune abnormalities.	J Allergy Clin Immunol.
atopic dermatitis	RAB15	Expression profiling by array, count, 3 development stage, 2 disease state, 11 individual sets	GPL570	GSE32924 Samples	Nonlesional atopic dermatitis skin is characterized by broad terminal differentiation defects and variable immune abnormalities.	J Allergy Clin Immunol.
atopic dermatitis	TIMM50	Expression profiling by array, count, 3 development stage, 2 disease state, 11 individual sets	GPL570	GSE32924 Samples	Nonlesional atopic dermatitis skin is characterized by broad terminal differentiation defects and variable immune abnormalities.	J Allergy Clin Immunol.
atopic dermatitis	FBXO31	Expression profiling by array, count, 3 development stage, 2 disease state, 11 individual sets	GPL570	GSE32924 Samples	Nonlesional atopic dermatitis skin is characterized by broad terminal differentiation defects and variable immune abnormalities.	J Allergy Clin Immunol.
atopic dermatitis	RPS6KA4	Expression profiling by array, count, 3 development stage, 2 disease state, 11 individual sets	GPL570	GSE32924 Samples	Nonlesional atopic dermatitis skin is characterized by broad terminal differentiation defects and variable immune abnormalities.	J Allergy Clin Immunol.
atopic dermatitis	NA	Expression profiling by array, count, 3 development stage, 2 disease state, 11 individual sets	GPL570	GSE32924 Samples	Nonlesional atopic dermatitis skin is characterized by broad terminal differentiation defects and variable immune abnormalities.	J Allergy Clin Immunol.
atopic dermatitis	OSTM1	Expression profiling by array, count, 3 development stage, 2 disease state, 11 individual sets	GPL570	GSE32924 Samples	Nonlesional atopic dermatitis skin is characterized by broad terminal differentiation defects and variable immune abnormalities.	J Allergy Clin Immunol.
atopic dermatitis	NA	Expression profiling by array, count, 3 development stage, 2 disease state, 11 individual sets	GPL570	GSE32924 Samples	Nonlesional atopic dermatitis skin is characterized by broad terminal differentiation defects and variable immune abnormalities.	J Allergy Clin Immunol.
atopic dermatitis	NA	Expression profiling by array, count, 3 development stage, 2 disease state, 11 individual sets	GPL570	GSE32924 Samples	Nonlesional atopic dermatitis skin is characterized by broad terminal differentiation defects and variable immune abnormalities.	J Allergy Clin Immunol.
atopic dermatitis	NA	Expression profiling by array, count, 3 development stage, 2 disease state, 11 individual sets	GPL570	GSE32924 Samples	Nonlesional atopic dermatitis skin is characterized by broad terminal differentiation defects and variable immune abnormalities.	J Allergy Clin Immunol.
atopic dermatitis	FLJ00049	Expression profiling by array, count, 3 development stage, 2 disease state, 11 individual sets	GPL570	GSE32924 Samples	Nonlesional atopic dermatitis skin is characterized by broad terminal differentiation defects and variable immune abnormalities.	J Allergy Clin Immunol.
atopic dermatitis	NA	Expression profiling by array, count, 3 development stage, 2 disease state, 11 individual sets	GPL570	GSE32924 Samples	Nonlesional atopic dermatitis skin is characterized by broad terminal differentiation defects and variable immune abnormalities.	J Allergy Clin Immunol.
atopic dermatitis	NA	Expression profiling by array, count, 3 development stage, 2 disease state, 11 individual sets	GPL570	GSE32924 Samples	Nonlesional atopic dermatitis skin is characterized by broad terminal differentiation defects and variable immune abnormalities.	J Allergy Clin Immunol.
atopic dermatitis	ODF3B	Expression profiling by array, count, 3 development stage, 2 disease state, 11 individual sets	GPL570	GSE32924 Samples	Nonlesional atopic dermatitis skin is characterized by broad terminal differentiation defects and variable immune abnormalities.	J Allergy Clin Immunol.
atopic dermatitis	NA	Expression profiling by array, count, 3 development stage, 2 disease state, 11 individual sets	GPL570	GSE32924 Samples	Nonlesional atopic dermatitis skin is characterized by broad terminal differentiation defects and variable immune abnormalities.	J Allergy Clin Immunol.
atopic dermatitis	ITCH	Expression profiling by array, count, 3 development stage, 2 disease state, 11 individual sets	GPL570	GSE32924 Samples	Nonlesional atopic dermatitis skin is characterized by broad terminal differentiation defects and variable immune abnormalities.	J Allergy Clin Immunol.
atopic dermatitis	EGR1	Expression profiling by array, count, 3 development stage, 2 disease state, 11 individual sets	GPL570	GSE32924 Samples	Nonlesional atopic dermatitis skin is characterized by broad terminal differentiation defects and variable immune abnormalities.	J Allergy Clin Immunol.
atopic dermatitis	MRTO4	Expression profiling by array, count, 3 development stage, 2 disease state, 11 individual sets	GPL570	GSE32924 Samples	Nonlesional atopic dermatitis skin is characterized by broad terminal differentiation defects and variable immune abnormalities.	J Allergy Clin Immunol.
atopic dermatitis	GGT1	Expression profiling by array, count, 3 development stage, 2 disease state, 11 individual sets	GPL570	GSE32924 Samples	Nonlesional atopic dermatitis skin is characterized by broad terminal differentiation defects and variable immune abnormalities.	J Allergy Clin Immunol.
atopic dermatitis	GGT1	Expression profiling by array, count, 3 development stage, 2 disease state, 11 individual sets	GPL570	GSE32924 Samples	Nonlesional atopic dermatitis skin is characterized by broad terminal differentiation defects and variable immune abnormalities.	J Allergy Clin Immunol.
atopic dermatitis	ELF3	Expression profiling by array, count, 3 development stage, 2 disease state, 11 individual sets	GPL570	GSE32924 Samples	Nonlesional atopic dermatitis skin is characterized by broad terminal differentiation defects and variable immune abnormalities.	J Allergy Clin Immunol.
atopic dermatitis	PHLDA1	Expression profiling by array, count, 3 development stage, 2 disease state, 11 individual sets	GPL570	GSE32924 Samples	Nonlesional atopic dermatitis skin is characterized by broad terminal differentiation defects and variable immune abnormalities.	J Allergy Clin Immunol.

atopic dermatitis	ABP1	Expression profiling by array, count, 3 development stage, 2 disease state, 11 individual sets	GPL570	GSE32924 Samples	Nonlesional atopic dermatitis skin is characterized by broad terminal differentiation defects and variable immune abnormalities.	J Allergy Clin Immunol.
atopic dermatitis	ZNF618	Expression profiling by array, count, 3 development stage, 2 disease state, 11 individual sets	GPL570	GSE32924 Samples	Nonlesional atopic dermatitis skin is characterized by broad terminal differentiation defects and variable immune abnormalities.	J Allergy Clin Immunol.
atopic dermatitis	NA	Expression profiling by array, count, 3 development stage, 2 disease state, 11 individual sets	GPL570	GSE32924 Samples	Nonlesional atopic dermatitis skin is characterized by broad terminal differentiation defects and variable immune abnormalities.	J Allergy Clin Immunol.
atopic dermatitis	PTCD2	Expression profiling by array, count, 3 development stage, 2 disease state, 11 individual sets	GPL570	GSE32924 Samples	Nonlesional atopic dermatitis skin is characterized by broad terminal differentiation defects and variable immune abnormalities.	J Allergy Clin Immunol.
atopic dermatitis	NA	Expression profiling by array, count, 3 development stage, 2 disease state, 11 individual sets	GPL570	GSE32924 Samples	Nonlesional atopic dermatitis skin is characterized by broad terminal differentiation defects and variable immune abnormalities.	J Allergy Clin Immunol.
atopic dermatitis	CD9	Expression profiling by array, count, 3 development stage, 2 disease state, 11 individual sets	GPL570	GSE32924 Samples	Nonlesional atopic dermatitis skin is characterized by broad terminal differentiation defects and variable immune abnormalities.	J Allergy Clin Immunol.
atopic dermatitis	NA	Expression profiling by array, count, 3 development stage, 2 disease state, 11 individual sets	GPL570	GSE32924 Samples	Nonlesional atopic dermatitis skin is characterized by broad terminal differentiation defects and variable immune abnormalities.	J Allergy Clin Immunol.
atopic dermatitis	NA	Expression profiling by array, count, 3 development stage, 2 disease state, 11 individual sets	GPL570	GSE32924 Samples	Nonlesional atopic dermatitis skin is characterized by broad terminal differentiation defects and variable immune abnormalities.	J Allergy Clin Immunol.
atopic dermatitis	GPRC5A	Expression profiling by array, count, 3 development stage, 2 disease state, 11 individual sets	GPL570	GSE32924 Samples	Nonlesional atopic dermatitis skin is characterized by broad terminal differentiation defects and variable immune abnormalities.	J Allergy Clin Immunol.
atopic dermatitis	SNORA28	Expression profiling by array, count, 3 development stage, 2 disease state, 11 individual sets	GPL570	GSE32924 Samples	Nonlesional atopic dermatitis skin is characterized by broad terminal differentiation defects and variable immune abnormalities.	J Allergy Clin Immunol.
atopic dermatitis	MED26	Expression profiling by array, count, 3 development stage, 2 disease state, 11 individual sets	GPL570	GSE32924 Samples	Nonlesional atopic dermatitis skin is characterized by broad terminal differentiation defects and variable immune abnormalities.	J Allergy Clin Immunol.
atopic dermatitis	NA	Expression profiling by array, count, 3 development stage, 2 disease state, 11 individual sets	GPL570	GSE32924 Samples	Nonlesional atopic dermatitis skin is characterized by broad terminal differentiation defects and variable immune abnormalities.	J Allergy Clin Immunol.
atopic dermatitis	NA	Expression profiling by array, count, 3 development stage, 2 disease state, 11 individual sets	GPL570	GSE32924 Samples	Nonlesional atopic dermatitis skin is characterized by broad terminal differentiation defects and variable immune abnormalities.	J Allergy Clin Immunol.
atopic dermatitis	STXBP3	Expression profiling by array, count, 3 development stage, 2 disease state, 11 individual sets	GPL570	GSE32924 Samples	Nonlesional atopic dermatitis skin is characterized by broad terminal differentiation defects and variable immune abnormalities.	J Allergy Clin Immunol.
atopic dermatitis	NA	Expression profiling by array, count, 3 development stage, 2 disease state, 11 individual sets	GPL570	GSE32924 Samples	Nonlesional atopic dermatitis skin is characterized by broad terminal differentiation defects and variable immune abnormalities.	J Allergy Clin Immunol.
atopic dermatitis	NA	Expression profiling by array, count, 3 development stage, 2 disease state, 11 individual sets	GPL570	GSE32924 Samples	Nonlesional atopic dermatitis skin is characterized by broad terminal differentiation defects and variable immune abnormalities.	J Allergy Clin Immunol.
atopic dermatitis	NA	Expression profiling by array, count, 3 development stage, 2 disease state, 11 individual sets	GPL570	GSE32924 Samples	Nonlesional atopic dermatitis skin is characterized by broad terminal differentiation defects and variable immune abnormalities.	J Allergy Clin Immunol.
atopic dermatitis	LOC100131601	Expression profiling by array, count, 3 development stage, 2 disease state, 11 individual sets	GPL570	GSE32924 Samples	Nonlesional atopic dermatitis skin is characterized by broad terminal differentiation defects and variable immune abnormalities.	J Allergy Clin Immunol.
atopic dermatitis	SLC1A4	Expression profiling by array, count, 3 development stage, 2 disease state, 11 individual sets	GPL570	GSE32924 Samples	Nonlesional atopic dermatitis skin is characterized by broad terminal differentiation defects and variable immune abnormalities.	J Allergy Clin Immunol.
atopic dermatitis	NA	Expression profiling by array, count, 3 development stage, 2 disease state, 11 individual sets	GPL570	GSE32924 Samples	Nonlesional atopic dermatitis skin is characterized by broad terminal differentiation defects and variable immune abnormalities.	J Allergy Clin Immunol.
atopic dermatitis	NA	Expression profiling by array, count, 3 development stage, 2 disease state, 11 individual sets	GPL570	GSE32924 Samples	Nonlesional atopic dermatitis skin is characterized by broad terminal differentiation defects and variable immune abnormalities.	J Allergy Clin Immunol.
atopic dermatitis	CEL	Expression profiling by array, count, 3 development stage, 2 disease state, 11 individual sets	GPL570	GSE32924 Samples	Nonlesional atopic dermatitis skin is characterized by broad terminal differentiation defects and variable immune abnormalities.	J Allergy Clin Immunol.
atopic dermatitis	NA	Expression profiling by array, count, 3 development stage, 2 disease state, 11 individual sets	GPL570	GSE32924 Samples	Nonlesional atopic dermatitis skin is characterized by broad terminal differentiation defects and variable immune abnormalities.	J Allergy Clin Immunol.
atopic dermatitis	AASDH	Expression profiling by array, count, 3 development stage, 2 disease state, 11 individual sets	GPL570	GSE32924 Samples	Nonlesional atopic dermatitis skin is characterized by broad terminal differentiation defects and variable immune abnormalities.	J Allergy Clin Immunol.
atopic dermatitis	SMC5	Expression profiling by array, count, 3 development stage, 2 disease state, 11 individual sets	GPL570	GSE32924 Samples	Nonlesional atopic dermatitis skin is characterized by broad terminal differentiation defects and variable immune abnormalities.	J Allergy Clin Immunol.
atopic dermatitis	NUPL1	Expression profiling by array, count, 3 development stage, 2 disease state, 11 individual sets	GPL570	GSE32924 Samples	Nonlesional atopic dermatitis skin is characterized by broad terminal differentiation defects and variable immune abnormalities.	J Allergy Clin Immunol.
atopic dermatitis	VPS8	Expression profiling by array, count, 3 development stage, 2 disease state, 11 individual sets	GPL570	GSE32924 Samples	Nonlesional atopic dermatitis skin is characterized by broad terminal differentiation defects and variable immune abnormalities.	J Allergy Clin Immunol.
atopic dermatitis	CDC42BPA	Expression profiling by array, count, 3 development stage, 2 disease state, 11 individual sets	GPL570	GSE32924 Samples	Nonlesional atopic dermatitis skin is characterized by broad terminal differentiation defects and variable immune abnormalities.	J Allergy Clin Immunol.
atopic dermatitis	CALD1	Expression profiling by array, count, 3 development stage, 2 disease state, 11 individual sets	GPL570	GSE32924 Samples	Nonlesional atopic dermatitis skin is characterized by broad terminal differentiation defects and variable immune abnormalities.	J Allergy Clin Immunol.
atopic dermatitis	NA	Expression profiling by array, count, 3 development stage, 2 disease state, 11 individual sets	GPL570	GSE32924 Samples	Nonlesional atopic dermatitis skin is characterized by broad terminal differentiation defects and variable immune abnormalities.	J Allergy Clin Immunol.
atopic dermatitis	SPEN	Expression profiling by array, count, 3 development stage, 2 disease state, 11 individual sets	GPL570	GSE32924 Samples	Nonlesional atopic dermatitis skin is characterized by broad terminal differentiation defects and variable immune abnormalities.	J Allergy Clin Immunol.
atopic dermatitis	TOP1	Expression profiling by array, count, 3 development stage, 2 disease state, 11 individual sets	GPL570	GSE32924 Samples	Nonlesional atopic dermatitis skin is characterized by broad terminal differentiation defects and variable immune abnormalities.	J Allergy Clin Immunol.
atopic dermatitis	MEX3D	Expression profiling by array, count, 3 development stage, 2 disease state, 11 individual sets	GPL570	GSE32924 Samples	Nonlesional atopic dermatitis skin is characterized by broad terminal differentiation defects and variable immune abnormalities.	J Allergy Clin Immunol.
atopic dermatitis	YTHDC1	Expression profiling by array, count, 3 development stage, 2 disease state, 11 individual sets	GPL570	GSE32924 Samples	Nonlesional atopic dermatitis skin is characterized by broad terminal differentiation defects and variable immune abnormalities.	J Allergy Clin Immunol.
atopic dermatitis	NA	Expression profiling by array, count, 3 development stage, 2 disease state, 11 individual sets	GPL570	GSE32924 Samples	Nonlesional atopic dermatitis skin is characterized by broad terminal differentiation defects and variable immune abnormalities.	J Allergy Clin Immunol.
atopic dermatitis	RSRC1	Expression profiling by array, count, 3 development stage, 2 disease state, 11 individual sets	GPL570	GSE32924 Samples	Nonlesional atopic dermatitis skin is characterized by broad terminal differentiation defects and variable immune abnormalities.	J Allergy Clin Immunol.
atopic dermatitis	KLHDC10	Expression profiling by array, count, 3 development stage, 2 disease state, 11 individual sets	GPL570	GSE32924 Samples	Nonlesional atopic dermatitis skin is characterized by broad terminal differentiation defects and variable immune abnormalities.	J Allergy Clin Immunol.
atopic dermatitis	RBM8A	Expression profiling by array, count, 3 development stage, 2 disease state, 11 individual sets	GPL570	GSE32924 Samples	Nonlesional atopic dermatitis skin is characterized by broad terminal differentiation defects and variable immune abnormalities.	J Allergy Clin Immunol.
atopic dermatitis	SMG1	Expression profiling by array, count, 3 development stage, 2 disease state, 11 individual sets	GPL570	GSE32924 Samples	Nonlesional atopic dermatitis skin is characterized by broad terminal differentiation defects and variable immune abnormalities.	J Allergy Clin Immunol.
atopic dermatitis	ATRX	Expression profiling by array, count, 3 development stage, 2 disease state, 11 individual sets	GPL570	GSE32924 Samples	Nonlesional atopic dermatitis skin is characterized by broad terminal differentiation defects and variable immune abnormalities.	J Allergy Clin Immunol.
atopic dermatitis	EFNB2	Expression profiling by array, count, 3 development stage, 2 disease state, 11 individual sets	GPL570	GSE32924 Samples	Nonlesional atopic dermatitis skin is characterized by broad terminal differentiation defects and variable immune abnormalities.	J Allergy Clin Immunol.

atopic dermatitis	LRP8	Expression profiling by array, count, 3 development stage, 2 disease state, 11 individual sets	GPL570	GSE32924 Samples	Nonlesional atopic dermatitis skin is characterized by broad terminal differentiation defects and variable immune abnormalities.	J Allergy Clin Immunol.
atopic dermatitis	SLC38A5	Expression profiling by array, count, 3 development stage, 2 disease state, 11 individual sets	GPL570	GSE32924 Samples	Nonlesional atopic dermatitis skin is characterized by broad terminal differentiation defects and variable immune abnormalities.	J Allergy Clin Immunol.
atopic dermatitis	BRCA2	Expression profiling by array, count, 3 development stage, 2 disease state, 11 individual sets	GPL570	GSE32924 Samples	Nonlesional atopic dermatitis skin is characterized by broad terminal differentiation defects and variable immune abnormalities.	J Allergy Clin Immunol.
atopic dermatitis	KLHL5	Expression profiling by array, count, 3 development stage, 2 disease state, 11 individual sets	GPL570	GSE32924 Samples	Nonlesional atopic dermatitis skin is characterized by broad terminal differentiation defects and variable immune abnormalities.	J Allergy Clin Immunol.
atopic dermatitis	NFKB2	Expression profiling by array, count, 3 development stage, 2 disease state, 11 individual sets	GPL570	GSE32924 Samples	Nonlesional atopic dermatitis skin is characterized by broad terminal differentiation defects and variable immune abnormalities.	J Allergy Clin Immunol.
atopic dermatitis	FOXK2	Expression profiling by array, count, 3 development stage, 2 disease state, 11 individual sets	GPL570	GSE32924 Samples	Nonlesional atopic dermatitis skin is characterized by broad terminal differentiation defects and variable immune abnormalities.	J Allergy Clin Immunol.
atopic dermatitis	ACOT11	Expression profiling by array, count, 3 development stage, 2 disease state, 11 individual sets	GPL570	GSE32924 Samples	Nonlesional atopic dermatitis skin is characterized by broad terminal differentiation defects and variable immune abnormalities.	J Allergy Clin Immunol.
atopic dermatitis	PBX2	Expression profiling by array, count, 3 development stage, 2 disease state, 11 individual sets	GPL570	GSE32924 Samples	Nonlesional atopic dermatitis skin is characterized by broad terminal differentiation defects and variable immune abnormalities.	J Allergy Clin Immunol.
atopic dermatitis	TAF6L	Expression profiling by array, count, 3 development stage, 2 disease state, 11 individual sets	GPL570	GSE32924 Samples	Nonlesional atopic dermatitis skin is characterized by broad terminal differentiation defects and variable immune abnormalities.	J Allergy Clin Immunol.
atopic dermatitis	PPFIA3	Expression profiling by array, count, 3 development stage, 2 disease state, 11 individual sets	GPL570	GSE32924 Samples	Nonlesional atopic dermatitis skin is characterized by broad terminal differentiation defects and variable immune abnormalities.	J Allergy Clin Immunol.
atopic dermatitis	NCRNA00202	Expression profiling by array, count, 3 development stage, 2 disease state, 11 individual sets	GPL570	GSE32924 Samples	Nonlesional atopic dermatitis skin is characterized by broad terminal differentiation defects and variable immune abnormalities.	J Allergy Clin Immunol.
atopic dermatitis	ZNF814	Expression profiling by array, count, 3 development stage, 2 disease state, 11 individual sets	GPL570	GSE32924 Samples	Nonlesional atopic dermatitis skin is characterized by broad terminal differentiation defects and variable immune abnormalities.	J Allergy Clin Immunol.
atopic dermatitis	NA	Expression profiling by array, count, 3 development stage, 2 disease state, 11 individual sets	GPL570	GSE32924 Samples	Nonlesional atopic dermatitis skin is characterized by broad terminal differentiation defects and variable immune abnormalities.	J Allergy Clin Immunol.
atopic dermatitis	NA	Expression profiling by array, count, 3 development stage, 2 disease state, 11 individual sets	GPL570	GSE32924 Samples	Nonlesional atopic dermatitis skin is characterized by broad terminal differentiation defects and variable immune abnormalities.	J Allergy Clin Immunol.
atopic dermatitis	ATF7IP	Expression profiling by array, count, 3 development stage, 2 disease state, 11 individual sets	GPL570	GSE32924 Samples	Nonlesional atopic dermatitis skin is characterized by broad terminal differentiation defects and variable immune abnormalities.	J Allergy Clin Immunol.
atopic dermatitis	NA	Expression profiling by array, count, 3 development stage, 2 disease state, 11 individual sets	GPL570	GSE32924 Samples	Nonlesional atopic dermatitis skin is characterized by broad terminal differentiation defects and variable immune abnormalities.	J Allergy Clin Immunol.
atopic dermatitis	NGLY1	Expression profiling by array, count, 3 development stage, 2 disease state, 11 individual sets	GPL570	GSE32924 Samples	Nonlesional atopic dermatitis skin is characterized by broad terminal differentiation defects and variable immune abnormalities.	J Allergy Clin Immunol.
atopic dermatitis	NA	Expression profiling by array, count, 3 development stage, 2 disease state, 11 individual sets	GPL570	GSE32924 Samples	Nonlesional atopic dermatitis skin is characterized by broad terminal differentiation defects and variable immune abnormalities.	J Allergy Clin Immunol.
atopic dermatitis	AKAP13	Expression profiling by array, count, 3 development stage, 2 disease state, 11 individual sets	GPL570	GSE32924 Samples	Nonlesional atopic dermatitis skin is characterized by broad terminal differentiation defects and variable immune abnormalities.	J Allergy Clin Immunol.
atopic dermatitis	CD44	Expression profiling by array, count, 3 development stage, 2 disease state, 11 individual sets	GPL570	GSE32924 Samples	Nonlesional atopic dermatitis skin is characterized by broad terminal differentiation defects and variable immune abnormalities.	J Allergy Clin Immunol.
atopic dermatitis	SEL1L	Expression profiling by array, count, 3 development stage, 2 disease state, 11 individual sets	GPL570	GSE32924 Samples	Nonlesional atopic dermatitis skin is characterized by broad terminal differentiation defects and variable immune abnormalities.	J Allergy Clin Immunol.
atopic dermatitis	NA	Expression profiling by array, count, 3 development stage, 2 disease state, 11 individual sets	GPL570	GSE32924 Samples	Nonlesional atopic dermatitis skin is characterized by broad terminal differentiation defects and variable immune abnormalities.	J Allergy Clin Immunol.
atopic dermatitis	GJC1	Expression profiling by array, count, 3 development stage, 2 disease state, 11 individual sets	GPL570	GSE32924 Samples	Nonlesional atopic dermatitis skin is characterized by broad terminal differentiation defects and variable immune abnormalities.	J Allergy Clin Immunol.
atopic dermatitis	CCL26	Expression profiling by array, count, 3 development stage, 2 disease state, 11 individual sets	GPL570	GSE32924 Samples	Nonlesional atopic dermatitis skin is characterized by broad terminal differentiation defects and variable immune abnormalities.	J Allergy Clin Immunol.
atopic dermatitis	CCL17	Expression profiling by array, count, 3 development stage, 2 disease state, 11 individual sets	GPL570	GSE32924 Samples	Nonlesional atopic dermatitis skin is characterized by broad terminal differentiation defects and variable immune abnormalities.	J Allergy Clin Immunol.
atopic dermatitis	FAM175A	Expression profiling by array, count, 3 development stage, 2 disease state, 11 individual sets	GPL570	GSE32924 Samples	Nonlesional atopic dermatitis skin is characterized by broad terminal differentiation defects and variable immune abnormalities.	J Allergy Clin Immunol.
atopic dermatitis	ZNF227	Expression profiling by array, count, 3 development stage, 2 disease state, 11 individual sets	GPL570	GSE32924 Samples	Nonlesional atopic dermatitis skin is characterized by broad terminal differentiation defects and variable immune abnormalities.	J Allergy Clin Immunol.
atopic dermatitis	GLRB	Expression profiling by array, count, 3 development stage, 2 disease state, 11 individual sets	GPL570	GSE32924 Samples	Nonlesional atopic dermatitis skin is characterized by broad terminal differentiation defects and variable immune abnormalities.	J Allergy Clin Immunol.
atopic dermatitis	NKAP	Expression profiling by array, count, 3 development stage, 2 disease state, 11 individual sets	GPL570	GSE32924 Samples	Nonlesional atopic dermatitis skin is characterized by broad terminal differentiation defects and variable immune abnormalities.	J Allergy Clin Immunol.
atopic dermatitis	STX17	Expression profiling by array, count, 3 development stage, 2 disease state, 11 individual sets	GPL570	GSE32924 Samples	Nonlesional atopic dermatitis skin is characterized by broad terminal differentiation defects and variable immune abnormalities.	J Allergy Clin Immunol.
atopic dermatitis	RNF180	Expression profiling by array, count, 3 development stage, 2 disease state, 11 individual sets	GPL570	GSE32924 Samples	Nonlesional atopic dermatitis skin is characterized by broad terminal differentiation defects and variable immune abnormalities.	J Allergy Clin Immunol.
atopic dermatitis	ZNF160	Expression profiling by array, count, 3 development stage, 2 disease state, 11 individual sets	GPL570	GSE32924 Samples	Nonlesional atopic dermatitis skin is characterized by broad terminal differentiation defects and variable immune abnormalities.	J Allergy Clin Immunol.
atopic dermatitis	FRY	Expression profiling by array, count, 3 development stage, 2 disease state, 11 individual sets	GPL570	GSE32924 Samples	Nonlesional atopic dermatitis skin is characterized by broad terminal differentiation defects and variable immune abnormalities.	J Allergy Clin Immunol.
atopic dermatitis	PLCL2	Expression profiling by array, count, 3 development stage, 2 disease state, 11 individual sets	GPL570	GSE32924 Samples	Nonlesional atopic dermatitis skin is characterized by broad terminal differentiation defects and variable immune abnormalities.	J Allergy Clin Immunol.
atopic dermatitis	CFI	Expression profiling by array, count, 3 development stage, 2 disease state, 11 individual sets	GPL570	GSE32924 Samples	Nonlesional atopic dermatitis skin is characterized by broad terminal differentiation defects and variable immune abnormalities.	J Allergy Clin Immunol.
atopic dermatitis	NA	Expression profiling by array, count, 3 development stage, 2 disease state, 11 individual sets	GPL570	GSE32924 Samples	Nonlesional atopic dermatitis skin is characterized by broad terminal differentiation defects and variable immune abnormalities.	J Allergy Clin Immunol.
atopic dermatitis	NA	Expression profiling by array, count, 3 development stage, 2 disease state, 11 individual sets	GPL570	GSE32924 Samples	Nonlesional atopic dermatitis skin is characterized by broad terminal differentiation defects and variable immune abnormalities.	J Allergy Clin Immunol.
atopic dermatitis	KLHL24	Expression profiling by array, count, 3 development stage, 2 disease state, 11 individual sets	GPL570	GSE32924 Samples	Nonlesional atopic dermatitis skin is characterized by broad terminal differentiation defects and variable immune abnormalities.	J Allergy Clin Immunol.
atopic dermatitis	PALMD	Expression profiling by array, count, 3 development stage, 2 disease state, 11 individual sets	GPL570	GSE32924 Samples	Nonlesional atopic dermatitis skin is characterized by broad terminal differentiation defects and variable immune abnormalities.	J Allergy Clin Immunol.
atopic dermatitis	NA	Expression profiling by array, count, 3 development stage, 2 disease state, 11 individual sets	GPL570	GSE32924 Samples	Nonlesional atopic dermatitis skin is characterized by broad terminal differentiation defects and variable immune abnormalities.	J Allergy Clin Immunol.
atopic dermatitis	CDADC1	Expression profiling by array, count, 3 development stage, 2 disease state, 11 individual sets	GPL570	GSE32924 Samples	Nonlesional atopic dermatitis skin is characterized by broad terminal differentiation defects and variable immune abnormalities.	J Allergy Clin Immunol.

atopic dermatitis	VPS41	Expression profiling by array, count, 3 development stage, 2 disease state, 11 individual sets	GPL570	GSE32924 Samples	Nonlesional atopic dermatitis skin is characterized by broad terminal differentiation defects and variable immune abnormalities.	J Allergy Clin Immunol.
atopic dermatitis	ZNF148	Expression profiling by array, count, 3 development stage, 2 disease state, 11 individual sets	GPL570	GSE32924 Samples	Nonlesional atopic dermatitis skin is characterized by broad terminal differentiation defects and variable immune abnormalities.	J Allergy Clin Immunol.
atopic dermatitis	CCDC126	Expression profiling by array, count, 3 development stage, 2 disease state, 11 individual sets	GPL570	GSE32924 Samples	Nonlesional atopic dermatitis skin is characterized by broad terminal differentiation defects and variable immune abnormalities.	J Allergy Clin Immunol.
atopic dermatitis	GPR98	Expression profiling by array, count, 3 development stage, 2 disease state, 11 individual sets	GPL570	GSE32924 Samples	Nonlesional atopic dermatitis skin is characterized by broad terminal differentiation defects and variable immune abnormalities.	J Allergy Clin Immunol.
atopic dermatitis	PDE4DIP	Expression profiling by array, count, 3 development stage, 2 disease state, 11 individual sets	GPL570	GSE32924 Samples	Nonlesional atopic dermatitis skin is characterized by broad terminal differentiation defects and variable immune abnormalities.	J Allergy Clin Immunol.
atopic dermatitis	TTC3	Expression profiling by array, count, 3 development stage, 2 disease state, 11 individual sets	GPL570	GSE32924 Samples	Nonlesional atopic dermatitis skin is characterized by broad terminal differentiation defects and variable immune abnormalities.	J Allergy Clin Immunol.
atopic dermatitis	PRKAB1	Expression profiling by array, count, 3 development stage, 2 disease state, 11 individual sets	GPL570	GSE32924 Samples	Nonlesional atopic dermatitis skin is characterized by broad terminal differentiation defects and variable immune abnormalities.	J Allergy Clin Immunol.
atopic dermatitis	C3ORF17	Expression profiling by array, count, 3 development stage, 2 disease state, 11 individual sets	GPL570	GSE32924 Samples	Nonlesional atopic dermatitis skin is characterized by broad terminal differentiation defects and variable immune abnormalities.	J Allergy Clin Immunol.
atopic dermatitis	GM2A	Expression profiling by array, count, 3 development stage, 2 disease state, 11 individual sets	GPL570	GSE32924 Samples	Nonlesional atopic dermatitis skin is characterized by broad terminal differentiation defects and variable immune abnormalities.	J Allergy Clin Immunol.
atopic dermatitis	IQGAP1	Expression profiling by array, count, 3 development stage, 2 disease state, 11 individual sets	GPL570	GSE32924 Samples	Nonlesional atopic dermatitis skin is characterized by broad terminal differentiation defects and variable immune abnormalities.	J Allergy Clin Immunol.
atopic dermatitis	TPM1	Expression profiling by array, count, 3 development stage, 2 disease state, 11 individual sets	GPL570	GSE32924 Samples	Nonlesional atopic dermatitis skin is characterized by broad terminal differentiation defects and variable immune abnormalities.	J Allergy Clin Immunol.
atopic dermatitis	NA	Expression profiling by array, count, 3 development stage, 2 disease state, 11 individual sets	GPL570	GSE32924 Samples	Nonlesional atopic dermatitis skin is characterized by broad terminal differentiation defects and variable immune abnormalities.	J Allergy Clin Immunol.
atopic dermatitis	FLJ33630	Expression profiling by array, count, 3 development stage, 2 disease state, 11 individual sets	GPL570	GSE32924 Samples	Nonlesional atopic dermatitis skin is characterized by broad terminal differentiation defects and variable immune abnormalities.	J Allergy Clin Immunol.
atopic dermatitis	PRKCB	Expression profiling by array, count, 3 development stage, 2 disease state, 11 individual sets	GPL570	GSE32924 Samples	Nonlesional atopic dermatitis skin is characterized by broad terminal differentiation defects and variable immune abnormalities.	J Allergy Clin Immunol.
atopic dermatitis	MEIS1	Expression profiling by array, count, 3 development stage, 2 disease state, 11 individual sets	GPL570	GSE32924 Samples	Nonlesional atopic dermatitis skin is characterized by broad terminal differentiation defects and variable immune abnormalities.	J Allergy Clin Immunol.
atopic dermatitis	NA	Expression profiling by array, count, 3 development stage, 2 disease state, 11 individual sets	GPL570	GSE32924 Samples	Nonlesional atopic dermatitis skin is characterized by broad terminal differentiation defects and variable immune abnormalities.	J Allergy Clin Immunol.
atopic dermatitis	NA	Expression profiling by array, count, 3 development stage, 2 disease state, 11 individual sets	GPL570	GSE32924 Samples	Nonlesional atopic dermatitis skin is characterized by broad terminal differentiation defects and variable immune abnormalities.	J Allergy Clin Immunol.
atopic dermatitis	USP31	Expression profiling by array, count, 3 development stage, 2 disease state, 11 individual sets	GPL570	GSE32924 Samples	Nonlesional atopic dermatitis skin is characterized by broad terminal differentiation defects and variable immune abnormalities.	J Allergy Clin Immunol.
atopic dermatitis	GLCCI1	Expression profiling by array, count, 3 development stage, 2 disease state, 11 individual sets	GPL570	GSE32924 Samples	Nonlesional atopic dermatitis skin is characterized by broad terminal differentiation defects and variable immune abnormalities.	J Allergy Clin Immunol.
atopic dermatitis	XG	Expression profiling by array, count, 3 development stage, 2 disease state, 11 individual sets	GPL570	GSE32924 Samples	Nonlesional atopic dermatitis skin is characterized by broad terminal differentiation defects and variable immune abnormalities.	J Allergy Clin Immunol.
atopic dermatitis	SDPR	Expression profiling by array, count, 3 development stage, 2 disease state, 11 individual sets	GPL570	GSE32924 Samples	Nonlesional atopic dermatitis skin is characterized by broad terminal differentiation defects and variable immune abnormalities.	J Allergy Clin Immunol.
atopic dermatitis	PTGFR	Expression profiling by array, count, 3 development stage, 2 disease state, 11 individual sets	GPL570	GSE32924 Samples	Nonlesional atopic dermatitis skin is characterized by broad terminal differentiation defects and variable immune abnormalities.	J Allergy Clin Immunol.
atopic dermatitis	RPP30	Expression profiling by array, count, 3 development stage, 2 disease state, 11 individual sets	GPL570	GSE32924 Samples	Nonlesional atopic dermatitis skin is characterized by broad terminal differentiation defects and variable immune abnormalities.	J Allergy Clin Immunol.
atopic dermatitis	ZNF148	Expression profiling by array, count, 3 development stage, 2 disease state, 11 individual sets	GPL570	GSE32924 Samples	Nonlesional atopic dermatitis skin is characterized by broad terminal differentiation defects and variable immune abnormalities.	J Allergy Clin Immunol.
atopic dermatitis	DPYSL3	Expression profiling by array, count, 3 development stage, 2 disease state, 11 individual sets	GPL570	GSE32924 Samples	Nonlesional atopic dermatitis skin is characterized by broad terminal differentiation defects and variable immune abnormalities.	J Allergy Clin Immunol.
atopic dermatitis	LSS	Expression profiling by array, count, 3 development stage, 2 disease state, 11 individual sets	GPL570	GSE32924 Samples	Nonlesional atopic dermatitis skin is characterized by broad terminal differentiation defects and variable immune abnormalities.	J Allergy Clin Immunol.
atopic dermatitis	SMARCA2	Expression profiling by array, count, 3 development stage, 2 disease state, 11 individual sets	GPL570	GSE32924 Samples	Nonlesional atopic dermatitis skin is characterized by broad terminal differentiation defects and variable immune abnormalities.	J Allergy Clin Immunol.
atopic dermatitis	MFN2	Expression profiling by array, count, 3 development stage, 2 disease state, 11 individual sets	GPL570	GSE32924 Samples	Nonlesional atopic dermatitis skin is characterized by broad terminal differentiation defects and variable immune abnormalities.	J Allergy Clin Immunol.
atopic dermatitis	UBE4B	Expression profiling by array, count, 3 development stage, 2 disease state, 11 individual sets	GPL570	GSE32924 Samples	Nonlesional atopic dermatitis skin is characterized by broad terminal differentiation defects and variable immune abnormalities.	J Allergy Clin Immunol.
atopic dermatitis	UFM1	Expression profiling by array, count, 3 development stage, 2 disease state, 11 individual sets	GPL570	GSE32924 Samples	Nonlesional atopic dermatitis skin is characterized by broad terminal differentiation defects and variable immune abnormalities.	J Allergy Clin Immunol.
atopic dermatitis	PPP3R1	Expression profiling by array, count, 3 development stage, 2 disease state, 11 individual sets	GPL570	GSE32924 Samples	Nonlesional atopic dermatitis skin is characterized by broad terminal differentiation defects and variable immune abnormalities.	J Allergy Clin Immunol.
atopic dermatitis	GLE1	Expression profiling by array, count, 3 development stage, 2 disease state, 11 individual sets	GPL570	GSE32924 Samples	Nonlesional atopic dermatitis skin is characterized by broad terminal differentiation defects and variable immune abnormalities.	J Allergy Clin Immunol.
atopic dermatitis	CREBZF	Expression profiling by array, count, 3 development stage, 2 disease state, 11 individual sets	GPL570	GSE32924 Samples	Nonlesional atopic dermatitis skin is characterized by broad terminal differentiation defects and variable immune abnormalities.	J Allergy Clin Immunol.
atopic dermatitis	ARFIP1	Expression profiling by array, count, 3 development stage, 2 disease state, 11 individual sets	GPL570	GSE32924 Samples	Nonlesional atopic dermatitis skin is characterized by broad terminal differentiation defects and variable immune abnormalities.	J Allergy Clin Immunol.
atopic dermatitis	ZBTB6	Expression profiling by array, count, 3 development stage, 2 disease state, 11 individual sets	GPL570	GSE32924 Samples	Nonlesional atopic dermatitis skin is characterized by broad terminal differentiation defects and variable immune abnormalities.	J Allergy Clin Immunol.
atopic dermatitis	LRP12	Expression profiling by array, count, 3 development stage, 2 disease state, 11 individual sets	GPL570	GSE32924 Samples	Nonlesional atopic dermatitis skin is characterized by broad terminal differentiation defects and variable immune abnormalities.	J Allergy Clin Immunol.
atopic dermatitis	SGPP1	Expression profiling by array, count, 3 development stage, 2 disease state, 11 individual sets	GPL570	GSE32924 Samples	Nonlesional atopic dermatitis skin is characterized by broad terminal differentiation defects and variable immune abnormalities.	J Allergy Clin Immunol.
atopic dermatitis	PCGF5	Expression profiling by array, count, 3 development stage, 2 disease state, 11 individual sets	GPL570	GSE32924 Samples	Nonlesional atopic dermatitis skin is characterized by broad terminal differentiation defects and variable immune abnormalities.	J Allergy Clin Immunol.
atopic dermatitis	ADAM9	Expression profiling by array, count, 3 development stage, 2 disease state, 11 individual sets	GPL570	GSE32924 Samples	Nonlesional atopic dermatitis skin is characterized by broad terminal differentiation defects and variable immune abnormalities.	J Allergy Clin Immunol.
atopic dermatitis	WEE1	Expression profiling by array, count, 3 development stage, 2 disease state, 11 individual sets	GPL570	GSE32924 Samples	Nonlesional atopic dermatitis skin is characterized by broad terminal differentiation defects and variable immune abnormalities.	J Allergy Clin Immunol.
atopic dermatitis	EIF4E3	Expression profiling by array, count, 3 development stage, 2 disease state, 11 individual sets	GPL570	GSE32924 Samples	Nonlesional atopic dermatitis skin is characterized by broad terminal differentiation defects and variable immune abnormalities.	J Allergy Clin Immunol.

atopic dermatitis	COBLL1	Expression profiling by array, count, 3 development stage, 2 disease state, 11 individual sets	GPL570	GSE32924 Samples	Nonlesional atopic dermatitis skin is characterized by broad terminal differentiation defects and variable immune abnormalities.	J Allergy Clin Immunol.
atopic dermatitis	NT5E	Expression profiling by array, count, 3 development stage, 2 disease state, 11 individual sets	GPL570	GSE32924 Samples	Nonlesional atopic dermatitis skin is characterized by broad terminal differentiation defects and variable immune abnormalities.	J Allergy Clin Immunol.
atopic dermatitis	PVRL3	Expression profiling by array, count, 3 development stage, 2 disease state, 11 individual sets	GPL570	GSE32924 Samples	Nonlesional atopic dermatitis skin is characterized by broad terminal differentiation defects and variable immune abnormalities.	J Allergy Clin Immunol.
atopic dermatitis	ANGPTL2	Expression profiling by array, count, 3 development stage, 2 disease state, 11 individual sets	GPL570	GSE32924 Samples	Nonlesional atopic dermatitis skin is characterized by broad terminal differentiation defects and variable immune abnormalities.	J Allergy Clin Immunol.
atopic dermatitis	HHEX	Expression profiling by array, count, 3 development stage, 2 disease state, 11 individual sets	GPL570	GSE32924 Samples	Nonlesional atopic dermatitis skin is characterized by broad terminal differentiation defects and variable immune abnormalities.	J Allergy Clin Immunol.
atopic dermatitis	TUBGCP3	Expression profiling by array, count, 3 development stage, 2 disease state, 11 individual sets	GPL570	GSE32924 Samples	Nonlesional atopic dermatitis skin is characterized by broad terminal differentiation defects and variable immune abnormalities.	J Allergy Clin Immunol.
atopic dermatitis	RHOB	Expression profiling by array, count, 3 development stage, 2 disease state, 11 individual sets	GPL570	GSE32924 Samples	Nonlesional atopic dermatitis skin is characterized by broad terminal differentiation defects and variable immune abnormalities.	J Allergy Clin Immunol.
atopic dermatitis	ARPP19	Expression profiling by array, count, 3 development stage, 2 disease state, 11 individual sets	GPL570	GSE32924 Samples	Nonlesional atopic dermatitis skin is characterized by broad terminal differentiation defects and variable immune abnormalities.	J Allergy Clin Immunol.
atopic dermatitis	APC	Expression profiling by array, count, 3 development stage, 2 disease state, 11 individual sets	GPL570	GSE32924 Samples	Nonlesional atopic dermatitis skin is characterized by broad terminal differentiation defects and variable immune abnormalities.	J Allergy Clin Immunol.
atopic dermatitis	DNAJB4	Expression profiling by array, count, 3 development stage, 2 disease state, 11 individual sets	GPL570	GSE32924 Samples	Nonlesional atopic dermatitis skin is characterized by broad terminal differentiation defects and variable immune abnormalities.	J Allergy Clin Immunol.
atopic dermatitis	TOR1AIP1	Expression profiling by array, count, 3 development stage, 2 disease state, 11 individual sets	GPL570	GSE32924 Samples	Nonlesional atopic dermatitis skin is characterized by broad terminal differentiation defects and variable immune abnormalities.	J Allergy Clin Immunol.
atopic dermatitis	UTRN	Expression profiling by array, count, 3 development stage, 2 disease state, 11 individual sets	GPL570	GSE32924 Samples	Nonlesional atopic dermatitis skin is characterized by broad terminal differentiation defects and variable immune abnormalities.	J Allergy Clin Immunol.
atopic dermatitis	LMBR1	Expression profiling by array, count, 3 development stage, 2 disease state, 11 individual sets	GPL570	GSE32924 Samples	Nonlesional atopic dermatitis skin is characterized by broad terminal differentiation defects and variable immune abnormalities.	J Allergy Clin Immunol.
atopic dermatitis	ORC5L	Expression profiling by array, count, 3 development stage, 2 disease state, 11 individual sets	GPL570	GSE32924 Samples	Nonlesional atopic dermatitis skin is characterized by broad terminal differentiation defects and variable immune abnormalities.	J Allergy Clin Immunol.
atopic dermatitis	GPHN	Expression profiling by array, count, 3 development stage, 2 disease state, 11 individual sets	GPL570	GSE32924 Samples	Nonlesional atopic dermatitis skin is characterized by broad terminal differentiation defects and variable immune abnormalities.	J Allergy Clin Immunol.
atopic dermatitis	THUMPD3	Expression profiling by array, count, 3 development stage, 2 disease state, 11 individual sets	GPL570	GSE32924 Samples	Nonlesional atopic dermatitis skin is characterized by broad terminal differentiation defects and variable immune abnormalities.	J Allergy Clin Immunol.
atopic dermatitis	NXT2	Expression profiling by array, count, 3 development stage, 2 disease state, 11 individual sets	GPL570	GSE32924 Samples	Nonlesional atopic dermatitis skin is characterized by broad terminal differentiation defects and variable immune abnormalities.	J Allergy Clin Immunol.
atopic dermatitis	CAB39	Expression profiling by array, count, 3 development stage, 2 disease state, 11 individual sets	GPL570	GSE32924 Samples	Nonlesional atopic dermatitis skin is characterized by broad terminal differentiation defects and variable immune abnormalities.	J Allergy Clin Immunol.
atopic dermatitis	MKRN2	Expression profiling by array, count, 3 development stage, 2 disease state, 11 individual sets	GPL570	GSE32924 Samples	Nonlesional atopic dermatitis skin is characterized by broad terminal differentiation defects and variable immune abnormalities.	J Allergy Clin Immunol.
atopic dermatitis	CDC5L	Expression profiling by array, count, 3 development stage, 2 disease state, 11 individual sets	GPL570	GSE32924 Samples	Nonlesional atopic dermatitis skin is characterized by broad terminal differentiation defects and variable immune abnormalities.	J Allergy Clin Immunol.
atopic dermatitis	MSH6	Expression profiling by array, count, 3 development stage, 2 disease state, 11 individual sets	GPL570	GSE32924 Samples	Nonlesional atopic dermatitis skin is characterized by broad terminal differentiation defects and variable immune abnormalities.	J Allergy Clin Immunol.
atopic dermatitis	TANK	Expression profiling by array, count, 3 development stage, 2 disease state, 11 individual sets	GPL570	GSE32924 Samples	Nonlesional atopic dermatitis skin is characterized by broad terminal differentiation defects and variable immune abnormalities.	J Allergy Clin Immunol.
atopic dermatitis	ALG13	Expression profiling by array, count, 3 development stage, 2 disease state, 11 individual sets	GPL570	GSE32924 Samples	Nonlesional atopic dermatitis skin is characterized by broad terminal differentiation defects and variable immune abnormalities.	J Allergy Clin Immunol.
atopic dermatitis	DUSP5	Expression profiling by array, count, 3 development stage, 2 disease state, 11 individual sets	GPL570	GSE32924 Samples	Nonlesional atopic dermatitis skin is characterized by broad terminal differentiation defects and variable immune abnormalities.	J Allergy Clin Immunol.
atopic dermatitis	API5	Expression profiling by array, count, 3 development stage, 2 disease state, 11 individual sets	GPL570	GSE32924 Samples	Nonlesional atopic dermatitis skin is characterized by broad terminal differentiation defects and variable immune abnormalities.	J Allergy Clin Immunol.
atopic dermatitis	KPNA4	Expression profiling by array, count, 3 development stage, 2 disease state, 11 individual sets	GPL570	GSE32924 Samples	Nonlesional atopic dermatitis skin is characterized by broad terminal differentiation defects and variable immune abnormalities.	J Allergy Clin Immunol.
atopic dermatitis	ACTR2	Expression profiling by array, count, 3 development stage, 2 disease state, 11 individual sets	GPL570	GSE32924 Samples	Nonlesional atopic dermatitis skin is characterized by broad terminal differentiation defects and variable immune abnormalities.	J Allergy Clin Immunol.
atopic dermatitis	SPAG9	Expression profiling by array, count, 3 development stage, 2 disease state, 11 individual sets	GPL570	GSE32924 Samples	Nonlesional atopic dermatitis skin is characterized by broad terminal differentiation defects and variable immune abnormalities.	J Allergy Clin Immunol.
atopic dermatitis	KPNA1	Expression profiling by array, count, 3 development stage, 2 disease state, 11 individual sets	GPL570	GSE32924 Samples	Nonlesional atopic dermatitis skin is characterized by broad terminal differentiation defects and variable immune abnormalities.	J Allergy Clin Immunol.
atopic dermatitis	TET3	Expression profiling by array, count, 3 development stage, 2 disease state, 11 individual sets	GPL570	GSE32924 Samples	Nonlesional atopic dermatitis skin is characterized by broad terminal differentiation defects and variable immune abnormalities.	J Allergy Clin Immunol.
atopic dermatitis	UGCG	Expression profiling by array, count, 3 development stage, 2 disease state, 11 individual sets	GPL570	GSE32924 Samples	Nonlesional atopic dermatitis skin is characterized by broad terminal differentiation defects and variable immune abnormalities.	J Allergy Clin Immunol.
atopic dermatitis	IL1RN	Expression profiling by array, count, 3 development stage, 2 disease state, 11 individual sets	GPL570	GSE32924 Samples	Nonlesional atopic dermatitis skin is characterized by broad terminal differentiation defects and variable immune abnormalities.	J Allergy Clin Immunol.
atopic dermatitis	CSDA	Expression profiling by array, count, 3 development stage, 2 disease state, 11 individual sets	GPL570	GSE32924 Samples	Nonlesional atopic dermatitis skin is characterized by broad terminal differentiation defects and variable immune abnormalities.	J Allergy Clin Immunol.
atopic dermatitis	CPZ	Expression profiling by array, count, 3 development stage, 2 disease state, 11 individual sets	GPL570	GSE32924 Samples	Nonlesional atopic dermatitis skin is characterized by broad terminal differentiation defects and variable immune abnormalities.	J Allergy Clin Immunol.
atopic dermatitis	IGFL2	Expression profiling by array, count, 3 development stage, 2 disease state, 11 individual sets	GPL570	GSE32924 Samples	Nonlesional atopic dermatitis skin is characterized by broad terminal differentiation defects and variable immune abnormalities.	J Allergy Clin Immunol.
atopic dermatitis	AQP9	Expression profiling by array, count, 3 development stage, 2 disease state, 11 individual sets	GPL570	GSE32924 Samples	Nonlesional atopic dermatitis skin is characterized by broad terminal differentiation defects and variable immune abnormalities.	J Allergy Clin Immunol.
atopic dermatitis	RNASE7	Expression profiling by array, count, 3 development stage, 2 disease state, 11 individual sets	GPL570	GSE32924 Samples	Nonlesional atopic dermatitis skin is characterized by broad terminal differentiation defects and variable immune abnormalities.	J Allergy Clin Immunol.
atopic dermatitis	SLC1A6	Expression profiling by array, count, 3 development stage, 2 disease state, 11 individual sets	GPL570	GSE32924 Samples	Nonlesional atopic dermatitis skin is characterized by broad terminal differentiation defects and variable immune abnormalities.	J Allergy Clin Immunol.
atopic dermatitis	ANXA9	Expression profiling by array, count, 3 development stage, 2 disease state, 11 individual sets	GPL570	GSE32924 Samples	Nonlesional atopic dermatitis skin is characterized by broad terminal differentiation defects and variable immune abnormalities.	J Allergy Clin Immunol.
atopic dermatitis	LCE1E	Expression profiling by array, count, 3 development stage, 2 disease state, 11 individual sets	GPL570	GSE32924 Samples	Nonlesional atopic dermatitis skin is characterized by broad terminal differentiation defects and variable immune abnormalities.	J Allergy Clin Immunol.
atopic dermatitis	SLC46A2	Expression profiling by array, count, 3 development stage, 2 disease state, 11 individual sets	GPL570	GSE32924 Samples	Nonlesional atopic dermatitis skin is characterized by broad terminal differentiation defects and variable immune abnormalities.	J Allergy Clin Immunol.

atopic dermatitis	AADAC	Expression profiling by array, count, 3 development stage, 2 disease state, 11 individual sets	GPL570	GSE32924 Samples	Nonlesional atopic dermatitis skin is characterized by broad terminal differentiation defects and variable immune abnormalities.	J Allergy Clin Immunol.
atopic dermatitis	PSG4	Expression profiling by array, count, 3 development stage, 2 disease state, 11 individual sets	GPL570	GSE32924 Samples	Nonlesional atopic dermatitis skin is characterized by broad terminal differentiation defects and variable immune abnormalities.	J Allergy Clin Immunol.
atopic dermatitis	OGN	Expression profiling by array, count, 3 development stage, 2 disease state, 11 individual sets	GPL570	GSE32924 Samples	Nonlesional atopic dermatitis skin is characterized by broad terminal differentiation defects and variable immune abnormalities.	J Allergy Clin Immunol.
atopic dermatitis	NA	Expression profiling by array, count, 3 development stage, 2 disease state, 11 individual sets	GPL570	GSE32924 Samples	Nonlesional atopic dermatitis skin is characterized by broad terminal differentiation defects and variable immune abnormalities.	J Allergy Clin Immunol.
atopic dermatitis	TNKS2	Expression profiling by array, count, 3 development stage, 2 disease state, 11 individual sets	GPL570	GSE32924 Samples	Nonlesional atopic dermatitis skin is characterized by broad terminal differentiation defects and variable immune abnormalities.	J Allergy Clin Immunol.
atopic dermatitis	NA	Expression profiling by array, count, 3 development stage, 2 disease state, 11 individual sets	GPL570	GSE32924 Samples	Nonlesional atopic dermatitis skin is characterized by broad terminal differentiation defects and variable immune abnormalities.	J Allergy Clin Immunol.
atopic dermatitis	RNF146	Expression profiling by array, count, 3 development stage, 2 disease state, 11 individual sets	GPL570	GSE32924 Samples	Nonlesional atopic dermatitis skin is characterized by broad terminal differentiation defects and variable immune abnormalities.	J Allergy Clin Immunol.
atopic dermatitis	PXK	Expression profiling by array, count, 3 development stage, 2 disease state, 11 individual sets	GPL570	GSE32924 Samples	Nonlesional atopic dermatitis skin is characterized by broad terminal differentiation defects and variable immune abnormalities.	J Allergy Clin Immunol.
atopic dermatitis	SGCG	Expression profiling by array, count, 3 development stage, 2 disease state, 11 individual sets	GPL570	GSE32924 Samples	Nonlesional atopic dermatitis skin is characterized by broad terminal differentiation defects and variable immune abnormalities.	J Allergy Clin Immunol.
atopic dermatitis	IL17D	Expression profiling by array, count, 3 development stage, 2 disease state, 11 individual sets	GPL570	GSE32924 Samples	Nonlesional atopic dermatitis skin is characterized by broad terminal differentiation defects and variable immune abnormalities.	J Allergy Clin Immunol.
atopic dermatitis	LRRC2	Expression profiling by array, count, 3 development stage, 2 disease state, 11 individual sets	GPL570	GSE32924 Samples	Nonlesional atopic dermatitis skin is characterized by broad terminal differentiation defects and variable immune abnormalities.	J Allergy Clin Immunol.
atopic dermatitis	SLC18A2	Expression profiling by array, count, 3 development stage, 2 disease state, 11 individual sets	GPL570	GSE32924 Samples	Nonlesional atopic dermatitis skin is characterized by broad terminal differentiation defects and variable immune abnormalities.	J Allergy Clin Immunol.
atopic dermatitis	HPGDS	Expression profiling by array, count, 3 development stage, 2 disease state, 11 individual sets	GPL570	GSE32924 Samples	Nonlesional atopic dermatitis skin is characterized by broad terminal differentiation defects and variable immune abnormalities.	J Allergy Clin Immunol.
atopic dermatitis	IGSF10	Expression profiling by array, count, 3 development stage, 2 disease state, 11 individual sets	GPL570	GSE32924 Samples	Nonlesional atopic dermatitis skin is characterized by broad terminal differentiation defects and variable immune abnormalities.	J Allergy Clin Immunol.
atopic dermatitis	FAM70A	Expression profiling by array, count, 3 development stage, 2 disease state, 11 individual sets	GPL570	GSE32924 Samples	Nonlesional atopic dermatitis skin is characterized by broad terminal differentiation defects and variable immune abnormalities.	J Allergy Clin Immunol.
atopic dermatitis	PNN	Expression profiling by array, count, 3 development stage, 2 disease state, 11 individual sets	GPL570	GSE32924 Samples	Nonlesional atopic dermatitis skin is characterized by broad terminal differentiation defects and variable immune abnormalities.	J Allergy Clin Immunol.
atopic dermatitis	NA	Expression profiling by array, count, 3 development stage, 2 disease state, 11 individual sets	GPL570	GSE32924 Samples	Nonlesional atopic dermatitis skin is characterized by broad terminal differentiation defects and variable immune abnormalities.	J Allergy Clin Immunol.
atopic dermatitis	RGS22	Expression profiling by array, count, 3 development stage, 2 disease state, 11 individual sets	GPL570	GSE32924 Samples	Nonlesional atopic dermatitis skin is characterized by broad terminal differentiation defects and variable immune abnormalities.	J Allergy Clin Immunol.
atopic dermatitis	ESR1	Expression profiling by array, count, 3 development stage, 2 disease state, 11 individual sets	GPL570	GSE32924 Samples	Nonlesional atopic dermatitis skin is characterized by broad terminal differentiation defects and variable immune abnormalities.	J Allergy Clin Immunol.
atopic dermatitis	SVEP1	Expression profiling by array, count, 3 development stage, 2 disease state, 11 individual sets	GPL570	GSE32924 Samples	Nonlesional atopic dermatitis skin is characterized by broad terminal differentiation defects and variable immune abnormalities.	J Allergy Clin Immunol.
atopic dermatitis	CLDN23	Expression profiling by array, count, 3 development stage, 2 disease state, 11 individual sets	GPL570	GSE32924 Samples	Nonlesional atopic dermatitis skin is characterized by broad terminal differentiation defects and variable immune abnormalities.	J Allergy Clin Immunol.
atopic dermatitis	CYP2J2	Expression profiling by array, count, 3 development stage, 2 disease state, 11 individual sets	GPL570	GSE32924 Samples	Nonlesional atopic dermatitis skin is characterized by broad terminal differentiation defects and variable immune abnormalities.	J Allergy Clin Immunol.
atopic dermatitis	CTNNA1	Expression profiling by array, count, 3 development stage, 2 disease state, 11 individual sets	GPL570	GSE32924 Samples	Nonlesional atopic dermatitis skin is characterized by broad terminal differentiation defects and variable immune abnormalities.	J Allergy Clin Immunol.
atopic dermatitis	VAV3	Expression profiling by array, count, 3 development stage, 2 disease state, 11 individual sets	GPL570	GSE32924 Samples	Nonlesional atopic dermatitis skin is characterized by broad terminal differentiation defects and variable immune abnormalities.	J Allergy Clin Immunol.
atopic dermatitis	NA	Expression profiling by array, count, 3 development stage, 2 disease state, 11 individual sets	GPL570	GSE32924 Samples	Nonlesional atopic dermatitis skin is characterized by broad terminal differentiation defects and variable immune abnormalities.	J Allergy Clin Immunol.
atopic dermatitis	YWHAE	Expression profiling by array, count, 3 development stage, 2 disease state, 11 individual sets	GPL570	GSE32924 Samples	Nonlesional atopic dermatitis skin is characterized by broad terminal differentiation defects and variable immune abnormalities.	J Allergy Clin Immunol.
atopic dermatitis	LAMP1	Expression profiling by array, count, 3 development stage, 2 disease state, 11 individual sets	GPL570	GSE32924 Samples	Nonlesional atopic dermatitis skin is characterized by broad terminal differentiation defects and variable immune abnormalities.	J Allergy Clin Immunol.
atopic dermatitis	MS4A2	Expression profiling by array, count, 3 development stage, 2 disease state, 11 individual sets	GPL570	GSE32924 Samples	Nonlesional atopic dermatitis skin is characterized by broad terminal differentiation defects and variable immune abnormalities.	J Allergy Clin Immunol.
atopic dermatitis	RGS13	Expression profiling by array, count, 3 development stage, 2 disease state, 11 individual sets	GPL570	GSE32924 Samples	Nonlesional atopic dermatitis skin is characterized by broad terminal differentiation defects and variable immune abnormalities.	J Allergy Clin Immunol.
atopic dermatitis	MMRN1	Expression profiling by array, count, 3 development stage, 2 disease state, 11 individual sets	GPL570	GSE32924 Samples	Nonlesional atopic dermatitis skin is characterized by broad terminal differentiation defects and variable immune abnormalities.	J Allergy Clin Immunol.
atopic dermatitis	LYVE1	Expression profiling by array, count, 3 development stage, 2 disease state, 11 individual sets	GPL570	GSE32924 Samples	Nonlesional atopic dermatitis skin is characterized by broad terminal differentiation defects and variable immune abnormalities.	J Allergy Clin Immunol.
atopic dermatitis	GPR34	Expression profiling by array, count, 3 development stage, 2 disease state, 11 individual sets	GPL570	GSE32924 Samples	Nonlesional atopic dermatitis skin is characterized by broad terminal differentiation defects and variable immune abnormalities.	J Allergy Clin Immunol.
atopic dermatitis	LYVE1	Expression profiling by array, count, 3 development stage, 2 disease state, 11 individual sets	GPL570	GSE32924 Samples	Nonlesional atopic dermatitis skin is characterized by broad terminal differentiation defects and variable immune abnormalities.	J Allergy Clin Immunol.
atopic dermatitis	CP	Expression profiling by array, count, 3 development stage, 2 disease state, 11 individual sets	GPL570	GSE32924 Samples	Nonlesional atopic dermatitis skin is characterized by broad terminal differentiation defects and variable immune abnormalities.	J Allergy Clin Immunol.
atopic dermatitis	GREM1	Expression profiling by array, count, 3 development stage, 2 disease state, 11 individual sets	GPL570	GSE32924 Samples	Nonlesional atopic dermatitis skin is characterized by broad terminal differentiation defects and variable immune abnormalities.	J Allergy Clin Immunol.
atopic dermatitis	IGJ	Expression profiling by array, count, 3 development stage, 2 disease state, 11 individual sets	GPL570	GSE32924 Samples	Nonlesional atopic dermatitis skin is characterized by broad terminal differentiation defects and variable immune abnormalities.	J Allergy Clin Immunol.
atopic dermatitis	LIMA1	Expression profiling by array, count, 3 development stage, 2 disease state, 11 individual sets	GPL570	GSE32924 Samples	Nonlesional atopic dermatitis skin is characterized by broad terminal differentiation defects and variable immune abnormalities.	J Allergy Clin Immunol.
atopic dermatitis	POMGNT1	Expression profiling by array, count, 3 development stage, 2 disease state, 11 individual sets	GPL570	GSE32924 Samples	Nonlesional atopic dermatitis skin is characterized by broad terminal differentiation defects and variable immune abnormalities.	J Allergy Clin Immunol.
atopic dermatitis	SYNE2	Expression profiling by array, count, 3 development stage, 2 disease state, 11 individual sets	GPL570	GSE32924 Samples	Nonlesional atopic dermatitis skin is characterized by broad terminal differentiation defects and variable immune abnormalities.	J Allergy Clin Immunol.
atopic dermatitis	NA	Expression profiling by array, count, 3 development stage, 2 disease state, 11 individual sets	GPL570	GSE32924 Samples	Nonlesional atopic dermatitis skin is characterized by broad terminal differentiation defects and variable immune abnormalities.	J Allergy Clin Immunol.
atopic dermatitis	FBXW11	Expression profiling by array, count, 3 development stage, 2 disease state, 11 individual sets	GPL570	GSE32924 Samples	Nonlesional atopic dermatitis skin is characterized by broad terminal differentiation defects and variable immune abnormalities.	J Allergy Clin Immunol.

atopic dermatitis	PSD4	Expression profiling by array, count, 3 development stage, 2 disease state, 11 individual sets	GPL570	GSE32924 Samples	Nonlesional atopic dermatitis skin is characterized by broad terminal differentiation defects and variable immune abnormalities.	J Allergy Clin Immunol.
atopic dermatitis	KBTBD4	Expression profiling by array, count, 3 development stage, 2 disease state, 11 individual sets	GPL570	GSE32924 Samples	Nonlesional atopic dermatitis skin is characterized by broad terminal differentiation defects and variable immune abnormalities.	J Allergy Clin Immunol.
atopic dermatitis	SRPR	Expression profiling by array, count, 3 development stage, 2 disease state, 11 individual sets	GPL570	GSE32924 Samples	Nonlesional atopic dermatitis skin is characterized by broad terminal differentiation defects and variable immune abnormalities.	J Allergy Clin Immunol.
atopic dermatitis	ZEB1	Expression profiling by array, count, 3 development stage, 2 disease state, 11 individual sets	GPL570	GSE32924 Samples	Nonlesional atopic dermatitis skin is characterized by broad terminal differentiation defects and variable immune abnormalities.	J Allergy Clin Immunol.
atopic dermatitis	DSTYK	Expression profiling by array, count, 3 development stage, 2 disease state, 11 individual sets	GPL570	GSE32924 Samples	Nonlesional atopic dermatitis skin is characterized by broad terminal differentiation defects and variable immune abnormalities.	J Allergy Clin Immunol.
atopic dermatitis	ANKRD11	Expression profiling by array, count, 3 development stage, 2 disease state, 11 individual sets	GPL570	GSE32924 Samples	Nonlesional atopic dermatitis skin is characterized by broad terminal differentiation defects and variable immune abnormalities.	J Allergy Clin Immunol.
atopic dermatitis	MYH11	Expression profiling by array, count, 3 development stage, 2 disease state, 11 individual sets	GPL570	GSE32924 Samples	Nonlesional atopic dermatitis skin is characterized by broad terminal differentiation defects and variable immune abnormalities.	J Allergy Clin Immunol.
atopic dermatitis	PPIA	Expression profiling by array, count, 3 development stage, 2 disease state, 11 individual sets	GPL570	GSE32924 Samples	Nonlesional atopic dermatitis skin is characterized by broad terminal differentiation defects and variable immune abnormalities.	J Allergy Clin Immunol.
atopic dermatitis	ERLIN1	Expression profiling by array, count, 3 development stage, 2 disease state, 11 individual sets	GPL570	GSE32924 Samples	Nonlesional atopic dermatitis skin is characterized by broad terminal differentiation defects and variable immune abnormalities.	J Allergy Clin Immunol.
atopic dermatitis	IFT57	Expression profiling by array, count, 3 development stage, 2 disease state, 11 individual sets	GPL570	GSE32924 Samples	Nonlesional atopic dermatitis skin is characterized by broad terminal differentiation defects and variable immune abnormalities.	J Allergy Clin Immunol.
atopic dermatitis	SLC8A1	Expression profiling by array, count, 3 development stage, 2 disease state, 11 individual sets	GPL570	GSE32924 Samples	Nonlesional atopic dermatitis skin is characterized by broad terminal differentiation defects and variable immune abnormalities.	J Allergy Clin Immunol.
atopic dermatitis	PRUNE2	Expression profiling by array, count, 3 development stage, 2 disease state, 11 individual sets	GPL570	GSE32924 Samples	Nonlesional atopic dermatitis skin is characterized by broad terminal differentiation defects and variable immune abnormalities.	J Allergy Clin Immunol.
atopic dermatitis	UBE2W	Expression profiling by array, count, 3 development stage, 2 disease state, 11 individual sets	GPL570	GSE32924 Samples	Nonlesional atopic dermatitis skin is characterized by broad terminal differentiation defects and variable immune abnormalities.	J Allergy Clin Immunol.
atopic dermatitis	ZNF567	Expression profiling by array, count, 3 development stage, 2 disease state, 11 individual sets	GPL570	GSE32924 Samples	Nonlesional atopic dermatitis skin is characterized by broad terminal differentiation defects and variable immune abnormalities.	J Allergy Clin Immunol.
atopic dermatitis	TRIM4	Expression profiling by array, count, 3 development stage, 2 disease state, 11 individual sets	GPL570	GSE32924 Samples	Nonlesional atopic dermatitis skin is characterized by broad terminal differentiation defects and variable immune abnormalities.	J Allergy Clin Immunol.
atopic dermatitis	NA	Expression profiling by array, count, 3 development stage, 2 disease state, 11 individual sets	GPL570	GSE32924 Samples	Nonlesional atopic dermatitis skin is characterized by broad terminal differentiation defects and variable immune abnormalities.	J Allergy Clin Immunol.
atopic dermatitis	ZNF567	Expression profiling by array, count, 3 development stage, 2 disease state, 11 individual sets	GPL570	GSE32924 Samples	Nonlesional atopic dermatitis skin is characterized by broad terminal differentiation defects and variable immune abnormalities.	J Allergy Clin Immunol.
atopic dermatitis	LRRC28	Expression profiling by array, count, 3 development stage, 2 disease state, 11 individual sets	GPL570	GSE32924 Samples	Nonlesional atopic dermatitis skin is characterized by broad terminal differentiation defects and variable immune abnormalities.	J Allergy Clin Immunol.
atopic dermatitis	FIP1L1	Expression profiling by array, count, 3 development stage, 2 disease state, 11 individual sets	GPL570	GSE32924 Samples	Nonlesional atopic dermatitis skin is characterized by broad terminal differentiation defects and variable immune abnormalities.	J Allergy Clin Immunol.
atopic dermatitis	GIPC2	Expression profiling by array, count, 3 development stage, 2 disease state, 11 individual sets	GPL570	GSE32924 Samples	Nonlesional atopic dermatitis skin is characterized by broad terminal differentiation defects and variable immune abnormalities.	J Allergy Clin Immunol.
atopic dermatitis	PCDHB6	Expression profiling by array, count, 3 development stage, 2 disease state, 11 individual sets	GPL570	GSE32924 Samples	Nonlesional atopic dermatitis skin is characterized by broad terminal differentiation defects and variable immune abnormalities.	J Allergy Clin Immunol.
atopic dermatitis	XPNPEP2	Expression profiling by array, count, 3 development stage, 2 disease state, 11 individual sets	GPL570	GSE32924 Samples	Nonlesional atopic dermatitis skin is characterized by broad terminal differentiation defects and variable immune abnormalities.	J Allergy Clin Immunol.
atopic dermatitis	JAK1	Expression profiling by array, count, 3 development stage, 2 disease state, 11 individual sets	GPL570	GSE32924 Samples	Nonlesional atopic dermatitis skin is characterized by broad terminal differentiation defects and variable immune abnormalities.	J Allergy Clin Immunol.
atopic dermatitis	TBL1XR1	Expression profiling by array, count, 3 development stage, 2 disease state, 11 individual sets	GPL570	GSE32924 Samples	Nonlesional atopic dermatitis skin is characterized by broad terminal differentiation defects and variable immune abnormalities.	J Allergy Clin Immunol.
atopic dermatitis	NA	Expression profiling by array, count, 3 development stage, 2 disease state, 11 individual sets	GPL570	GSE32924 Samples	Nonlesional atopic dermatitis skin is characterized by broad terminal differentiation defects and variable immune abnormalities.	J Allergy Clin Immunol.
atopic dermatitis	STK4	Expression profiling by array, count, 3 development stage, 2 disease state, 11 individual sets	GPL570	GSE32924 Samples	Nonlesional atopic dermatitis skin is characterized by broad terminal differentiation defects and variable immune abnormalities.	J Allergy Clin Immunol.
atopic dermatitis	NHSL1	Expression profiling by array, count, 3 development stage, 2 disease state, 11 individual sets	GPL570	GSE32924 Samples	Nonlesional atopic dermatitis skin is characterized by broad terminal differentiation defects and variable immune abnormalities.	J Allergy Clin Immunol.
atopic dermatitis	RASSF5	Expression profiling by array, count, 3 development stage, 2 disease state, 11 individual sets	GPL570	GSE32924 Samples	Nonlesional atopic dermatitis skin is characterized by broad terminal differentiation defects and variable immune abnormalities.	J Allergy Clin Immunol.
atopic dermatitis	GAS7	Expression profiling by array, count, 3 development stage, 2 disease state, 11 individual sets	GPL570	GSE32924 Samples	Nonlesional atopic dermatitis skin is characterized by broad terminal differentiation defects and variable immune abnormalities.	J Allergy Clin Immunol.
atopic dermatitis	NA	Expression profiling by array, count, 3 development stage, 2 disease state, 11 individual sets	GPL570	GSE32924 Samples	Nonlesional atopic dermatitis skin is characterized by broad terminal differentiation defects and variable immune abnormalities.	J Allergy Clin Immunol.
atopic dermatitis	HPGD	Expression profiling by array, count, 3 development stage, 2 disease state, 11 individual sets	GPL570	GSE32924 Samples	Nonlesional atopic dermatitis skin is characterized by broad terminal differentiation defects and variable immune abnormalities.	J Allergy Clin Immunol.
atopic dermatitis	ARHGAP18	Expression profiling by array, count, 3 development stage, 2 disease state, 11 individual sets	GPL570	GSE32924 Samples	Nonlesional atopic dermatitis skin is characterized by broad terminal differentiation defects and variable immune abnormalities.	J Allergy Clin Immunol.
atopic dermatitis	SGEF	Expression profiling by array, count, 3 development stage, 2 disease state, 11 individual sets	GPL570	GSE32924 Samples	Nonlesional atopic dermatitis skin is characterized by broad terminal differentiation defects and variable immune abnormalities.	J Allergy Clin Immunol.
atopic dermatitis	NA	Expression profiling by array, count, 3 development stage, 2 disease state, 11 individual sets	GPL570	GSE32924 Samples	Nonlesional atopic dermatitis skin is characterized by broad terminal differentiation defects and variable immune abnormalities.	J Allergy Clin Immunol.
atopic dermatitis	KIAA2026	Expression profiling by array, count, 3 development stage, 2 disease state, 11 individual sets	GPL570	GSE32924 Samples	Nonlesional atopic dermatitis skin is characterized by broad terminal differentiation defects and variable immune abnormalities.	J Allergy Clin Immunol.
atopic dermatitis	PSEN1	Expression profiling by array, count, 3 development stage, 2 disease state, 11 individual sets	GPL570	GSE32924 Samples	Nonlesional atopic dermatitis skin is characterized by broad terminal differentiation defects and variable immune abnormalities.	J Allergy Clin Immunol.
atopic dermatitis	MUTED	Expression profiling by array, count, 3 development stage, 2 disease state, 11 individual sets	GPL570	GSE32924 Samples	Nonlesional atopic dermatitis skin is characterized by broad terminal differentiation defects and variable immune abnormalities.	J Allergy Clin Immunol.
atopic dermatitis	ZCCHC7	Expression profiling by array, count, 3 development stage, 2 disease state, 11 individual sets	GPL570	GSE32924 Samples	Nonlesional atopic dermatitis skin is characterized by broad terminal differentiation defects and variable immune abnormalities.	J Allergy Clin Immunol.
atopic dermatitis	CALD1	Expression profiling by array, count, 3 development stage, 2 disease state, 11 individual sets	GPL570	GSE32924 Samples	Nonlesional atopic dermatitis skin is characterized by broad terminal differentiation defects and variable immune abnormalities.	J Allergy Clin Immunol.
atopic dermatitis	KIAA1324L	Expression profiling by array, count, 3 development stage, 2 disease state, 11 individual sets	GPL570	GSE32924 Samples	Nonlesional atopic dermatitis skin is characterized by broad terminal differentiation defects and variable immune abnormalities.	J Allergy Clin Immunol.
atopic dermatitis	BMPR2	Expression profiling by array, count, 3 development stage, 2 disease state, 11 individual sets	GPL570	GSE32924 Samples	Nonlesional atopic dermatitis skin is characterized by broad terminal differentiation defects and variable immune abnormalities.	J Allergy Clin Immunol.

atopic dermatitis	NEK3	Expression profiling by array, count, 3 development stage, 2 disease state, 11 individual sets	GPL570	GSE32924 Samples	Nonlesional atopic dermatitis skin is characterized by broad terminal differentiation defects and variable immune abnormalities.	J Allergy Clin Immunol.
atopic dermatitis	NA	Expression profiling by array, count, 3 development stage, 2 disease state, 11 individual sets	GPL570	GSE32924 Samples	Nonlesional atopic dermatitis skin is characterized by broad terminal differentiation defects and variable immune abnormalities.	J Allergy Clin Immunol.
atopic dermatitis	KDM5A	Expression profiling by array, count, 3 development stage, 2 disease state, 11 individual sets	GPL570	GSE32924 Samples	Nonlesional atopic dermatitis skin is characterized by broad terminal differentiation defects and variable immune abnormalities.	J Allergy Clin Immunol.
atopic dermatitis	NA	Expression profiling by array, count, 3 development stage, 2 disease state, 11 individual sets	GPL570	GSE32924 Samples	Nonlesional atopic dermatitis skin is characterized by broad terminal differentiation defects and variable immune abnormalities.	J Allergy Clin Immunol.
atopic dermatitis	SVEP1	Expression profiling by array, count, 3 development stage, 2 disease state, 11 individual sets	GPL570	GSE32924 Samples	Nonlesional atopic dermatitis skin is characterized by broad terminal differentiation defects and variable immune abnormalities.	J Allergy Clin Immunol.
atopic dermatitis	N4BP2L1	Expression profiling by array, count, 3 development stage, 2 disease state, 11 individual sets	GPL570	GSE32924 Samples	Nonlesional atopic dermatitis skin is characterized by broad terminal differentiation defects and variable immune abnormalities.	J Allergy Clin Immunol.
atopic dermatitis	CEP120	Expression profiling by array, count, 3 development stage, 2 disease state, 11 individual sets	GPL570	GSE32924 Samples	Nonlesional atopic dermatitis skin is characterized by broad terminal differentiation defects and variable immune abnormalities.	J Allergy Clin Immunol.
atopic dermatitis	LRFN5	Expression profiling by array, count, 3 development stage, 2 disease state, 11 individual sets	GPL570	GSE32924 Samples	Nonlesional atopic dermatitis skin is characterized by broad terminal differentiation defects and variable immune abnormalities.	J Allergy Clin Immunol.
atopic dermatitis	C4ORF12	Expression profiling by array, count, 3 development stage, 2 disease state, 11 individual sets	GPL570	GSE32924 Samples	Nonlesional atopic dermatitis skin is characterized by broad terminal differentiation defects and variable immune abnormalities.	J Allergy Clin Immunol.
atopic dermatitis	TCEAL7	Expression profiling by array, count, 3 development stage, 2 disease state, 11 individual sets	GPL570	GSE32924 Samples	Nonlesional atopic dermatitis skin is characterized by broad terminal differentiation defects and variable immune abnormalities.	J Allergy Clin Immunol.
atopic dermatitis	LMO4	Expression profiling by array, count, 3 development stage, 2 disease state, 11 individual sets	GPL570	GSE32924 Samples	Nonlesional atopic dermatitis skin is characterized by broad terminal differentiation defects and variable immune abnormalities.	J Allergy Clin Immunol.
atopic dermatitis	RHOJ	Expression profiling by array, count, 3 development stage, 2 disease state, 11 individual sets	GPL570	GSE32924 Samples	Nonlesional atopic dermatitis skin is characterized by broad terminal differentiation defects and variable immune abnormalities.	J Allergy Clin Immunol.
atopic dermatitis	FIBIN	Expression profiling by array, count, 3 development stage, 2 disease state, 11 individual sets	GPL570	GSE32924 Samples	Nonlesional atopic dermatitis skin is characterized by broad terminal differentiation defects and variable immune abnormalities.	J Allergy Clin Immunol.
atopic dermatitis	C1QTNF7	Expression profiling by array, count, 3 development stage, 2 disease state, 11 individual sets	GPL570	GSE32924 Samples	Nonlesional atopic dermatitis skin is characterized by broad terminal differentiation defects and variable immune abnormalities.	J Allergy Clin Immunol.
atopic dermatitis	SPINK1	Expression profiling by array, count, 3 development stage, 2 disease state, 11 individual sets	GPL570	GSE32924 Samples	Nonlesional atopic dermatitis skin is characterized by broad terminal differentiation defects and variable immune abnormalities.	J Allergy Clin Immunol.
atopic dermatitis	ZNF24	Expression profiling by array, count, 3 development stage, 2 disease state, 11 individual sets	GPL570	GSE32924 Samples	Nonlesional atopic dermatitis skin is characterized by broad terminal differentiation defects and variable immune abnormalities.	J Allergy Clin Immunol.
atopic dermatitis	SNAP23	Expression profiling by array, count, 3 development stage, 2 disease state, 11 individual sets	GPL570	GSE32924 Samples	Nonlesional atopic dermatitis skin is characterized by broad terminal differentiation defects and variable immune abnormalities.	J Allergy Clin Immunol.
atopic dermatitis	MTMR2	Expression profiling by array, count, 3 development stage, 2 disease state, 11 individual sets	GPL570	GSE32924 Samples	Nonlesional atopic dermatitis skin is characterized by broad terminal differentiation defects and variable immune abnormalities.	J Allergy Clin Immunol.
atopic dermatitis	PDGFC	Expression profiling by array, count, 3 development stage, 2 disease state, 11 individual sets	GPL570	GSE32924 Samples	Nonlesional atopic dermatitis skin is characterized by broad terminal differentiation defects and variable immune abnormalities.	J Allergy Clin Immunol.
atopic dermatitis	CASK	Expression profiling by array, count, 3 development stage, 2 disease state, 11 individual sets	GPL570	GSE32924 Samples	Nonlesional atopic dermatitis skin is characterized by broad terminal differentiation defects and variable immune abnormalities.	J Allergy Clin Immunol.
atopic dermatitis	RAB6A	Expression profiling by array, count, 3 development stage, 2 disease state, 11 individual sets	GPL570	GSE32924 Samples	Nonlesional atopic dermatitis skin is characterized by broad terminal differentiation defects and variable immune abnormalities.	J Allergy Clin Immunol.
atopic dermatitis	SLAIN2	Expression profiling by array, count, 3 development stage, 2 disease state, 11 individual sets	GPL570	GSE32924 Samples	Nonlesional atopic dermatitis skin is characterized by broad terminal differentiation defects and variable immune abnormalities.	J Allergy Clin Immunol.
atopic dermatitis	SPON1	Expression profiling by array, count, 3 development stage, 2 disease state, 11 individual sets	GPL570	GSE32924 Samples	Nonlesional atopic dermatitis skin is characterized by broad terminal differentiation defects and variable immune abnormalities.	J Allergy Clin Immunol.
atopic dermatitis	PPP1R3B	Expression profiling by array, count, 3 development stage, 2 disease state, 11 individual sets	GPL570	GSE32924 Samples	Nonlesional atopic dermatitis skin is characterized by broad terminal differentiation defects and variable immune abnormalities.	J Allergy Clin Immunol.
atopic dermatitis	STX16	Expression profiling by array, count, 3 development stage, 2 disease state, 11 individual sets	GPL570	GSE32924 Samples	Nonlesional atopic dermatitis skin is characterized by broad terminal differentiation defects and variable immune abnormalities.	J Allergy Clin Immunol.
atopic dermatitis	IRF6	Expression profiling by array, count, 3 development stage, 2 disease state, 11 individual sets	GPL570	GSE32924 Samples	Nonlesional atopic dermatitis skin is characterized by broad terminal differentiation defects and variable immune abnormalities.	J Allergy Clin Immunol.
atopic dermatitis	INPP5A	Expression profiling by array, count, 3 development stage, 2 disease state, 11 individual sets	GPL570	GSE32924 Samples	Nonlesional atopic dermatitis skin is characterized by broad terminal differentiation defects and variable immune abnormalities.	J Allergy Clin Immunol.
atopic dermatitis	HSP90B1	Expression profiling by array, count, 3 development stage, 2 disease state, 11 individual sets	GPL570	GSE32924 Samples	Nonlesional atopic dermatitis skin is characterized by broad terminal differentiation defects and variable immune abnormalities.	J Allergy Clin Immunol.
atopic dermatitis	C15ORF24	Expression profiling by array, count, 3 development stage, 2 disease state, 11 individual sets	GPL570	GSE32924 Samples	Nonlesional atopic dermatitis skin is characterized by broad terminal differentiation defects and variable immune abnormalities.	J Allergy Clin Immunol.
atopic dermatitis	BPTF	Expression profiling by array, count, 3 development stage, 2 disease state, 11 individual sets	GPL570	GSE32924 Samples	Nonlesional atopic dermatitis skin is characterized by broad terminal differentiation defects and variable immune abnormalities.	J Allergy Clin Immunol.
atopic dermatitis	UBASH3B	Expression profiling by array, count, 3 development stage, 2 disease state, 11 individual sets	GPL570	GSE32924 Samples	Nonlesional atopic dermatitis skin is characterized by broad terminal differentiation defects and variable immune abnormalities.	J Allergy Clin Immunol.
atopic dermatitis	PCIF1	Expression profiling by array, count, 3 development stage, 2 disease state, 11 individual sets	GPL570	GSE32924 Samples	Nonlesional atopic dermatitis skin is characterized by broad terminal differentiation defects and variable immune abnormalities.	J Allergy Clin Immunol.
atopic dermatitis	CARS	Expression profiling by array, count, 3 development stage, 2 disease state, 11 individual sets	GPL570	GSE32924 Samples	Nonlesional atopic dermatitis skin is characterized by broad terminal differentiation defects and variable immune abnormalities.	J Allergy Clin Immunol.
atopic dermatitis	GAS7	Expression profiling by array, count, 3 development stage, 2 disease state, 11 individual sets	GPL570	GSE32924 Samples	Nonlesional atopic dermatitis skin is characterized by broad terminal differentiation defects and variable immune abnormalities.	J Allergy Clin Immunol.
atopic dermatitis	FCGR2C	Expression profiling by array, count, 3 development stage, 2 disease state, 11 individual sets	GPL570	GSE32924 Samples	Nonlesional atopic dermatitis skin is characterized by broad terminal differentiation defects and variable immune abnormalities.	J Allergy Clin Immunol.
atopic dermatitis	CLN8	Expression profiling by array, count, 3 development stage, 2 disease state, 11 individual sets	GPL570	GSE32924 Samples	Nonlesional atopic dermatitis skin is characterized by broad terminal differentiation defects and variable immune abnormalities.	J Allergy Clin Immunol.
atopic dermatitis	CLIP1	Expression profiling by array, count, 3 development stage, 2 disease state, 11 individual sets	GPL570	GSE32924 Samples	Nonlesional atopic dermatitis skin is characterized by broad terminal differentiation defects and variable immune abnormalities.	J Allergy Clin Immunol.
atopic dermatitis	UBASH3B	Expression profiling by array, count, 3 development stage, 2 disease state, 11 individual sets	GPL570	GSE32924 Samples	Nonlesional atopic dermatitis skin is characterized by broad terminal differentiation defects and variable immune abnormalities.	J Allergy Clin Immunol.
atopic dermatitis	C13ORF31	Expression profiling by array, count, 3 development stage, 2 disease state, 11 individual sets	GPL570	GSE32924 Samples	Nonlesional atopic dermatitis skin is characterized by broad terminal differentiation defects and variable immune abnormalities.	J Allergy Clin Immunol.
atopic dermatitis	ETV7	Expression profiling by array, count, 3 development stage, 2 disease state, 11 individual sets	GPL570	GSE32924 Samples	Nonlesional atopic dermatitis skin is characterized by broad terminal differentiation defects and variable immune abnormalities.	J Allergy Clin Immunol.
atopic dermatitis	CYP39A1	Expression profiling by array, count, 3 development stage, 2 disease state, 11 individual sets	GPL570	GSE32924 Samples	Nonlesional atopic dermatitis skin is characterized by broad terminal differentiation defects and variable immune abnormalities.	J Allergy Clin Immunol.

atopic dermatitis	SLAIN2	Expression profiling by array, count, 3 development stage, 2 disease state, 11 individual sets	GPL570	GSE32924 Samples	Nonlesional atopic dermatitis skin is characterized by broad terminal differentiation defects and variable immune abnormalities.	J Allergy Clin Immunol.
atopic dermatitis	SLAIN2	Expression profiling by array, count, 3 development stage, 2 disease state, 11 individual sets	GPL570	GSE32924 Samples	Nonlesional atopic dermatitis skin is characterized by broad terminal differentiation defects and variable immune abnormalities.	J Allergy Clin Immunol.
atopic dermatitis	CUGBP1	Expression profiling by array, count, 3 development stage, 2 disease state, 11 individual sets	GPL570	GSE32924 Samples	Nonlesional atopic dermatitis skin is characterized by broad terminal differentiation defects and variable immune abnormalities.	J Allergy Clin Immunol.
atopic dermatitis	PTPRA	Expression profiling by array, count, 3 development stage, 2 disease state, 11 individual sets	GPL570	GSE32924 Samples	Nonlesional atopic dermatitis skin is characterized by broad terminal differentiation defects and variable immune abnormalities.	J Allergy Clin Immunol.
atopic dermatitis	XGPY2	Expression profiling by array, count, 3 development stage, 2 disease state, 11 individual sets	GPL570	GSE32924 Samples	Nonlesional atopic dermatitis skin is characterized by broad terminal differentiation defects and variable immune abnormalities.	J Allergy Clin Immunol.
atopic dermatitis	ORMDL1	Expression profiling by array, count, 3 development stage, 2 disease state, 11 individual sets	GPL570	GSE32924 Samples	Nonlesional atopic dermatitis skin is characterized by broad terminal differentiation defects and variable immune abnormalities.	J Allergy Clin Immunol.
atopic dermatitis	NA	Expression profiling by array, count, 3 development stage, 2 disease state, 11 individual sets	GPL570	GSE32924 Samples	Nonlesional atopic dermatitis skin is characterized by broad terminal differentiation defects and variable immune abnormalities.	J Allergy Clin Immunol.
atopic dermatitis	SFRS5	Expression profiling by array, count, 3 development stage, 2 disease state, 11 individual sets	GPL570	GSE32924 Samples	Nonlesional atopic dermatitis skin is characterized by broad terminal differentiation defects and variable immune abnormalities.	J Allergy Clin Immunol.
atopic dermatitis	HDLBP	Expression profiling by array, count, 3 development stage, 2 disease state, 11 individual sets	GPL570	GSE32924 Samples	Nonlesional atopic dermatitis skin is characterized by broad terminal differentiation defects and variable immune abnormalities.	J Allergy Clin Immunol.
atopic dermatitis	FAM190B	Expression profiling by array, count, 3 development stage, 2 disease state, 11 individual sets	GPL570	GSE32924 Samples	Nonlesional atopic dermatitis skin is characterized by broad terminal differentiation defects and variable immune abnormalities.	J Allergy Clin Immunol.
atopic dermatitis	GALNT12	Expression profiling by array, count, 3 development stage, 2 disease state, 11 individual sets	GPL570	GSE32924 Samples	Nonlesional atopic dermatitis skin is characterized by broad terminal differentiation defects and variable immune abnormalities.	J Allergy Clin Immunol.
atopic dermatitis	NAV3	Expression profiling by array, count, 3 development stage, 2 disease state, 11 individual sets	GPL570	GSE32924 Samples	Nonlesional atopic dermatitis skin is characterized by broad terminal differentiation defects and variable immune abnormalities.	J Allergy Clin Immunol.
atopic dermatitis	MACC1	Expression profiling by array, count, 3 development stage, 2 disease state, 11 individual sets	GPL570	GSE32924 Samples	Nonlesional atopic dermatitis skin is characterized by broad terminal differentiation defects and variable immune abnormalities.	J Allergy Clin Immunol.
atopic dermatitis	MAP7	Expression profiling by array, count, 3 development stage, 2 disease state, 11 individual sets	GPL570	GSE32924 Samples	Nonlesional atopic dermatitis skin is characterized by broad terminal differentiation defects and variable immune abnormalities.	J Allergy Clin Immunol.
atopic dermatitis	RHEB	Expression profiling by array, count, 3 development stage, 2 disease state, 11 individual sets	GPL570	GSE32924 Samples	Nonlesional atopic dermatitis skin is characterized by broad terminal differentiation defects and variable immune abnormalities.	J Allergy Clin Immunol.
atopic dermatitis	RAB2A	Expression profiling by array, count, 3 development stage, 2 disease state, 11 individual sets	GPL570	GSE32924 Samples	Nonlesional atopic dermatitis skin is characterized by broad terminal differentiation defects and variable immune abnormalities.	J Allergy Clin Immunol.
atopic dermatitis	C16ORF63	Expression profiling by array, count, 3 development stage, 2 disease state, 11 individual sets	GPL570	GSE32924 Samples	Nonlesional atopic dermatitis skin is characterized by broad terminal differentiation defects and variable immune abnormalities.	J Allergy Clin Immunol.
atopic dermatitis	METTL9	Expression profiling by array, count, 3 development stage, 2 disease state, 11 individual sets	GPL570	GSE32924 Samples	Nonlesional atopic dermatitis skin is characterized by broad terminal differentiation defects and variable immune abnormalities.	J Allergy Clin Immunol.
atopic dermatitis	SPIRE1	Expression profiling by array, count, 3 development stage, 2 disease state, 11 individual sets	GPL570	GSE32924 Samples	Nonlesional atopic dermatitis skin is characterized by broad terminal differentiation defects and variable immune abnormalities.	J Allergy Clin Immunol.
atopic dermatitis	RPAP3	Expression profiling by array, count, 3 development stage, 2 disease state, 11 individual sets	GPL570	GSE32924 Samples	Nonlesional atopic dermatitis skin is characterized by broad terminal differentiation defects and variable immune abnormalities.	J Allergy Clin Immunol.
atopic dermatitis	BRCC3	Expression profiling by array, count, 3 development stage, 2 disease state, 11 individual sets	GPL570	GSE32924 Samples	Nonlesional atopic dermatitis skin is characterized by broad terminal differentiation defects and variable immune abnormalities.	J Allergy Clin Immunol.
atopic dermatitis	RWDD2B	Expression profiling by array, count, 3 development stage, 2 disease state, 11 individual sets	GPL570	GSE32924 Samples	Nonlesional atopic dermatitis skin is characterized by broad terminal differentiation defects and variable immune abnormalities.	J Allergy Clin Immunol.
atopic dermatitis	ARNTL	Expression profiling by array, count, 3 development stage, 2 disease state, 11 individual sets	GPL570	GSE32924 Samples	Nonlesional atopic dermatitis skin is characterized by broad terminal differentiation defects and variable immune abnormalities.	J Allergy Clin Immunol.
atopic dermatitis	XKRX	Expression profiling by array, count, 3 development stage, 2 disease state, 11 individual sets	GPL570	GSE32924 Samples	Nonlesional atopic dermatitis skin is characterized by broad terminal differentiation defects and variable immune abnormalities.	J Allergy Clin Immunol.
atopic dermatitis	PTPN21	Expression profiling by array, count, 3 development stage, 2 disease state, 11 individual sets	GPL570	GSE32924 Samples	Nonlesional atopic dermatitis skin is characterized by broad terminal differentiation defects and variable immune abnormalities.	J Allergy Clin Immunol.
atopic dermatitis	ACOT8	Expression profiling by array, count, 3 development stage, 2 disease state, 11 individual sets	GPL570	GSE32924 Samples	Nonlesional atopic dermatitis skin is characterized by broad terminal differentiation defects and variable immune abnormalities.	J Allergy Clin Immunol.
atopic dermatitis	OCLN	Expression profiling by array, count, 3 development stage, 2 disease state, 11 individual sets	GPL570	GSE32924 Samples	Nonlesional atopic dermatitis skin is characterized by broad terminal differentiation defects and variable immune abnormalities.	J Allergy Clin Immunol.
atopic dermatitis	NA	Expression profiling by array, count, 3 development stage, 2 disease state, 11 individual sets	GPL570	GSE32924 Samples	Nonlesional atopic dermatitis skin is characterized by broad terminal differentiation defects and variable immune abnormalities.	J Allergy Clin Immunol.
atopic dermatitis	CYP2C18	Expression profiling by array, count, 3 development stage, 2 disease state, 11 individual sets	GPL570	GSE32924 Samples	Nonlesional atopic dermatitis skin is characterized by broad terminal differentiation defects and variable immune abnormalities.	J Allergy Clin Immunol.
atopic dermatitis	RNASE7	Expression profiling by array, count, 3 development stage, 2 disease state, 11 individual sets	GPL570	GSE32924 Samples	Nonlesional atopic dermatitis skin is characterized by broad terminal differentiation defects and variable immune abnormalities.	J Allergy Clin Immunol.
atopic dermatitis	ACER1	Expression profiling by array, count, 3 development stage, 2 disease state, 11 individual sets	GPL570	GSE32924 Samples	Nonlesional atopic dermatitis skin is characterized by broad terminal differentiation defects and variable immune abnormalities.	J Allergy Clin Immunol.
atopic dermatitis	F2RL1	Expression profiling by array, count, 3 development stage, 2 disease state, 11 individual sets	GPL570	GSE32924 Samples	Nonlesional atopic dermatitis skin is characterized by broad terminal differentiation defects and variable immune abnormalities.	J Allergy Clin Immunol.
atopic dermatitis	CDV3	Expression profiling by array, count, 3 development stage, 2 disease state, 11 individual sets	GPL570	GSE32924 Samples	Nonlesional atopic dermatitis skin is characterized by broad terminal differentiation defects and variable immune abnormalities.	J Allergy Clin Immunol.
atopic dermatitis	MTMR1	Expression profiling by array, count, 3 development stage, 2 disease state, 11 individual sets	GPL570	GSE32924 Samples	Nonlesional atopic dermatitis skin is characterized by broad terminal differentiation defects and variable immune abnormalities.	J Allergy Clin Immunol.
atopic dermatitis	EZR	Expression profiling by array, count, 3 development stage, 2 disease state, 11 individual sets	GPL570	GSE32924 Samples	Nonlesional atopic dermatitis skin is characterized by broad terminal differentiation defects and variable immune abnormalities.	J Allergy Clin Immunol.
atopic dermatitis	NA	Expression profiling by array, count, 3 development stage, 2 disease state, 11 individual sets	GPL570	GSE32924 Samples	Nonlesional atopic dermatitis skin is characterized by broad terminal differentiation defects and variable immune abnormalities.	J Allergy Clin Immunol.
atopic dermatitis	SQLE	Expression profiling by array, count, 3 development stage, 2 disease state, 11 individual sets	GPL570	GSE32924 Samples	Nonlesional atopic dermatitis skin is characterized by broad terminal differentiation defects and variable immune abnormalities.	J Allergy Clin Immunol.
atopic dermatitis	RNASE7	Expression profiling by array, count, 3 development stage, 2 disease state, 11 individual sets	GPL570	GSE32924 Samples	Nonlesional atopic dermatitis skin is characterized by broad terminal differentiation defects and variable immune abnormalities.	J Allergy Clin Immunol.
atopic dermatitis	ELMO2	Expression profiling by array, count, 3 development stage, 2 disease state, 11 individual sets	GPL570	GSE32924 Samples	Nonlesional atopic dermatitis skin is characterized by broad terminal differentiation defects and variable immune abnormalities.	J Allergy Clin Immunol.
atopic dermatitis	ABHD10	Expression profiling by array, count, 3 development stage, 2 disease state, 11 individual sets	GPL570	GSE32924 Samples	Nonlesional atopic dermatitis skin is characterized by broad terminal differentiation defects and variable immune abnormalities.	J Allergy Clin Immunol.
atopic dermatitis	MIER3	Expression profiling by array, count, 3 development stage, 2 disease state, 11 individual sets	GPL570	GSE32924 Samples	Nonlesional atopic dermatitis skin is characterized by broad terminal differentiation defects and variable immune abnormalities.	J Allergy Clin Immunol.

atopic dermatitis	FAM175B	Expression profiling by array, count, 3 development stage, 2 disease state, 11 individual sets	GPL570	GSE32924 Samples	Nonlesional atopic dermatitis skin is characterized by broad terminal differentiation defects and variable immune abnormalities.	J Allergy Clin Immunol.
atopic dermatitis	ARFGEF1	Expression profiling by array, count, 3 development stage, 2 disease state, 11 individual sets	GPL570	GSE32924 Samples	Nonlesional atopic dermatitis skin is characterized by broad terminal differentiation defects and variable immune abnormalities.	J Allergy Clin Immunol.
atopic dermatitis	ARIH1	Expression profiling by array, count, 3 development stage, 2 disease state, 11 individual sets	GPL570	GSE32924 Samples	Nonlesional atopic dermatitis skin is characterized by broad terminal differentiation defects and variable immune abnormalities.	J Allergy Clin Immunol.
atopic dermatitis	RPRD1A	Expression profiling by array, count, 3 development stage, 2 disease state, 11 individual sets	GPL570	GSE32924 Samples	Nonlesional atopic dermatitis skin is characterized by broad terminal differentiation defects and variable immune abnormalities.	J Allergy Clin Immunol.
atopic dermatitis	MED28	Expression profiling by array, count, 3 development stage, 2 disease state, 11 individual sets	GPL570	GSE32924 Samples	Nonlesional atopic dermatitis skin is characterized by broad terminal differentiation defects and variable immune abnormalities.	J Allergy Clin Immunol.
atopic dermatitis	KLRAQ1	Expression profiling by array, count, 3 development stage, 2 disease state, 11 individual sets	GPL570	GSE32924 Samples	Nonlesional atopic dermatitis skin is characterized by broad terminal differentiation defects and variable immune abnormalities.	J Allergy Clin Immunol.
atopic dermatitis	TFG	Expression profiling by array, count, 3 development stage, 2 disease state, 11 individual sets	GPL570	GSE32924 Samples	Nonlesional atopic dermatitis skin is characterized by broad terminal differentiation defects and variable immune abnormalities.	J Allergy Clin Immunol.
atopic dermatitis	ATP5S	Expression profiling by array, count, 3 development stage, 2 disease state, 11 individual sets	GPL570	GSE32924 Samples	Nonlesional atopic dermatitis skin is characterized by broad terminal differentiation defects and variable immune abnormalities.	J Allergy Clin Immunol.
atopic dermatitis	C9ORF125	Expression profiling by array, count, 3 development stage, 2 disease state, 11 individual sets	GPL570	GSE32924 Samples	Nonlesional atopic dermatitis skin is characterized by broad terminal differentiation defects and variable immune abnormalities.	J Allergy Clin Immunol.
atopic dermatitis	ALS2	Expression profiling by array, count, 3 development stage, 2 disease state, 11 individual sets	GPL570	GSE32924 Samples	Nonlesional atopic dermatitis skin is characterized by broad terminal differentiation defects and variable immune abnormalities.	J Allergy Clin Immunol.
atopic dermatitis	NA	Expression profiling by array, count, 3 development stage, 2 disease state, 11 individual sets	GPL570	GSE32924 Samples	Nonlesional atopic dermatitis skin is characterized by broad terminal differentiation defects and variable immune abnormalities.	J Allergy Clin Immunol.
atopic dermatitis	TMEM68	Expression profiling by array, count, 3 development stage, 2 disease state, 11 individual sets	GPL570	GSE32924 Samples	Nonlesional atopic dermatitis skin is characterized by broad terminal differentiation defects and variable immune abnormalities.	J Allergy Clin Immunol.
atopic dermatitis	USP2	Expression profiling by array, count, 3 development stage, 2 disease state, 11 individual sets	GPL570	GSE32924 Samples	Nonlesional atopic dermatitis skin is characterized by broad terminal differentiation defects and variable immune abnormalities.	J Allergy Clin Immunol.
atopic dermatitis	DNAJA4	Expression profiling by array, count, 3 development stage, 2 disease state, 11 individual sets	GPL570	GSE32924 Samples	Nonlesional atopic dermatitis skin is characterized by broad terminal differentiation defects and variable immune abnormalities.	J Allergy Clin Immunol.
atopic dermatitis	GFM2	Expression profiling by array, count, 3 development stage, 2 disease state, 11 individual sets	GPL570	GSE32924 Samples	Nonlesional atopic dermatitis skin is characterized by broad terminal differentiation defects and variable immune abnormalities.	J Allergy Clin Immunol.
atopic dermatitis	CALU	Expression profiling by array, count, 3 development stage, 2 disease state, 11 individual sets	GPL570	GSE32924 Samples	Nonlesional atopic dermatitis skin is characterized by broad terminal differentiation defects and variable immune abnormalities.	J Allergy Clin Immunol.
atopic dermatitis	NID1	Expression profiling by array, count, 3 development stage, 2 disease state, 11 individual sets	GPL570	GSE32924 Samples	Nonlesional atopic dermatitis skin is characterized by broad terminal differentiation defects and variable immune abnormalities.	J Allergy Clin Immunol.
atopic dermatitis	PTPN12	Expression profiling by array, count, 3 development stage, 2 disease state, 11 individual sets	GPL570	GSE32924 Samples	Nonlesional atopic dermatitis skin is characterized by broad terminal differentiation defects and variable immune abnormalities.	J Allergy Clin Immunol.
atopic dermatitis	ATRN	Expression profiling by array, count, 3 development stage, 2 disease state, 11 individual sets	GPL570	GSE32924 Samples	Nonlesional atopic dermatitis skin is characterized by broad terminal differentiation defects and variable immune abnormalities.	J Allergy Clin Immunol.
atopic dermatitis	GTF2I	Expression profiling by array, count, 3 development stage, 2 disease state, 11 individual sets	GPL570	GSE32924 Samples	Nonlesional atopic dermatitis skin is characterized by broad terminal differentiation defects and variable immune abnormalities.	J Allergy Clin Immunol.
atopic dermatitis	SMARCC1	Expression profiling by array, count, 3 development stage, 2 disease state, 11 individual sets	GPL570	GSE32924 Samples	Nonlesional atopic dermatitis skin is characterized by broad terminal differentiation defects and variable immune abnormalities.	J Allergy Clin Immunol.
atopic dermatitis	ANAPC5	Expression profiling by array, count, 3 development stage, 2 disease state, 11 individual sets	GPL570	GSE32924 Samples	Nonlesional atopic dermatitis skin is characterized by broad terminal differentiation defects and variable immune abnormalities.	J Allergy Clin Immunol.
atopic dermatitis	NA	Expression profiling by array, count, 3 development stage, 2 disease state, 11 individual sets	GPL570	GSE32924 Samples	Nonlesional atopic dermatitis skin is characterized by broad terminal differentiation defects and variable immune abnormalities.	J Allergy Clin Immunol.
atopic dermatitis	MAPKAP1	Expression profiling by array, count, 3 development stage, 2 disease state, 11 individual sets	GPL570	GSE32924 Samples	Nonlesional atopic dermatitis skin is characterized by broad terminal differentiation defects and variable immune abnormalities.	J Allergy Clin Immunol.
atopic dermatitis	RCAN1	Expression profiling by array, count, 3 development stage, 2 disease state, 11 individual sets	GPL570	GSE32924 Samples	Nonlesional atopic dermatitis skin is characterized by broad terminal differentiation defects and variable immune abnormalities.	J Allergy Clin Immunol.
atopic dermatitis	TOX4	Expression profiling by array, count, 3 development stage, 2 disease state, 11 individual sets	GPL570	GSE32924 Samples	Nonlesional atopic dermatitis skin is characterized by broad terminal differentiation defects and variable immune abnormalities.	J Allergy Clin Immunol.
atopic dermatitis	DHX9	Expression profiling by array, count, 3 development stage, 2 disease state, 11 individual sets	GPL570	GSE32924 Samples	Nonlesional atopic dermatitis skin is characterized by broad terminal differentiation defects and variable immune abnormalities.	J Allergy Clin Immunol.
atopic dermatitis	SLC39A6	Expression profiling by array, count, 3 development stage, 2 disease state, 11 individual sets	GPL570	GSE32924 Samples	Nonlesional atopic dermatitis skin is characterized by broad terminal differentiation defects and variable immune abnormalities.	J Allergy Clin Immunol.
atopic dermatitis	IL13RA1	Expression profiling by array, count, 3 development stage, 2 disease state, 11 individual sets	GPL570	GSE32924 Samples	Nonlesional atopic dermatitis skin is characterized by broad terminal differentiation defects and variable immune abnormalities.	J Allergy Clin Immunol.
atopic dermatitis	VPS35	Expression profiling by array, count, 3 development stage, 2 disease state, 11 individual sets	GPL570	GSE32924 Samples	Nonlesional atopic dermatitis skin is characterized by broad terminal differentiation defects and variable immune abnormalities.	J Allergy Clin Immunol.
atopic dermatitis	MFSD6	Expression profiling by array, count, 3 development stage, 2 disease state, 11 individual sets	GPL570	GSE32924 Samples	Nonlesional atopic dermatitis skin is characterized by broad terminal differentiation defects and variable immune abnormalities.	J Allergy Clin Immunol.
atopic dermatitis	TJP1	Expression profiling by array, count, 3 development stage, 2 disease state, 11 individual sets	GPL570	GSE32924 Samples	Nonlesional atopic dermatitis skin is characterized by broad terminal differentiation defects and variable immune abnormalities.	J Allergy Clin Immunol.
atopic dermatitis	CTTNBP2NL	Expression profiling by array, count, 3 development stage, 2 disease state, 11 individual sets	GPL570	GSE32924 Samples	Nonlesional atopic dermatitis skin is characterized by broad terminal differentiation defects and variable immune abnormalities.	J Allergy Clin Immunol.
atopic dermatitis	BTBD11	Expression profiling by array, count, 3 development stage, 2 disease state, 11 individual sets	GPL570	GSE32924 Samples	Nonlesional atopic dermatitis skin is characterized by broad terminal differentiation defects and variable immune abnormalities.	J Allergy Clin Immunol.
atopic dermatitis	KLHL7	Expression profiling by array, count, 3 development stage, 2 disease state, 11 individual sets	GPL570	GSE32924 Samples	Nonlesional atopic dermatitis skin is characterized by broad terminal differentiation defects and variable immune abnormalities.	J Allergy Clin Immunol.
atopic dermatitis	LGALS8	Expression profiling by array, count, 3 development stage, 2 disease state, 11 individual sets	GPL570	GSE32924 Samples	Nonlesional atopic dermatitis skin is characterized by broad terminal differentiation defects and variable immune abnormalities.	J Allergy Clin Immunol.
atopic dermatitis	ALOXE3	Expression profiling by array, count, 3 development stage, 2 disease state, 11 individual sets	GPL570	GSE32924 Samples	Nonlesional atopic dermatitis skin is characterized by broad terminal differentiation defects and variable immune abnormalities.	J Allergy Clin Immunol.
atopic dermatitis	PLD1	Expression profiling by array, count, 3 development stage, 2 disease state, 11 individual sets	GPL570	GSE32924 Samples	Nonlesional atopic dermatitis skin is characterized by broad terminal differentiation defects and variable immune abnormalities.	J Allergy Clin Immunol.
atopic dermatitis	PCSK6	Expression profiling by array, count, 3 development stage, 2 disease state, 11 individual sets	GPL570	GSE32924 Samples	Nonlesional atopic dermatitis skin is characterized by broad terminal differentiation defects and variable immune abnormalities.	J Allergy Clin Immunol.
atopic dermatitis	DLX5	Expression profiling by array, count, 3 development stage, 2 disease state, 11 individual sets	GPL570	GSE32924 Samples	Nonlesional atopic dermatitis skin is characterized by broad terminal differentiation defects and variable immune abnormalities.	J Allergy Clin Immunol.
atopic dermatitis	RHBDL2	Expression profiling by array, count, 3 development stage, 2 disease state, 11 individual sets	GPL570	GSE32924 Samples	Nonlesional atopic dermatitis skin is characterized by broad terminal differentiation defects and variable immune abnormalities.	J Allergy Clin Immunol.

atopic dermatitis	RHBDL2	Expression profiling by array, count, 3 development stage, 2 disease state, 11 individual sets	GPL570	GSE32924 Samples	Nonlesional atopic dermatitis skin is characterized by broad terminal differentiation defects and variable immune abnormalities.	J Allergy Clin Immunol.
atopic dermatitis	HSPC159	Expression profiling by array, count, 3 development stage, 2 disease state, 11 individual sets	GPL570	GSE32924 Samples	Nonlesional atopic dermatitis skin is characterized by broad terminal differentiation defects and variable immune abnormalities.	J Allergy Clin Immunol.
atopic dermatitis	MPP7	Expression profiling by array, count, 3 development stage, 2 disease state, 11 individual sets	GPL570	GSE32924 Samples	Nonlesional atopic dermatitis skin is characterized by broad terminal differentiation defects and variable immune abnormalities.	J Allergy Clin Immunol.
atopic dermatitis	IDE	Expression profiling by array, count, 3 development stage, 2 disease state, 11 individual sets	GPL570	GSE32924 Samples	Nonlesional atopic dermatitis skin is characterized by broad terminal differentiation defects and variable immune abnormalities.	J Allergy Clin Immunol.
atopic dermatitis	PTGS1	Expression profiling by array, count, 3 development stage, 2 disease state, 11 individual sets	GPL570	GSE32924 Samples	Nonlesional atopic dermatitis skin is characterized by broad terminal differentiation defects and variable immune abnormalities.	J Allergy Clin Immunol.
atopic dermatitis	C1ORF46	Expression profiling by array, count, 3 development stage, 2 disease state, 11 individual sets	GPL570	GSE32924 Samples	Nonlesional atopic dermatitis skin is characterized by broad terminal differentiation defects and variable immune abnormalities.	J Allergy Clin Immunol.
atopic dermatitis	WFDC12	Expression profiling by array, count, 3 development stage, 2 disease state, 11 individual sets	GPL570	GSE32924 Samples	Nonlesional atopic dermatitis skin is characterized by broad terminal differentiation defects and variable immune abnormalities.	J Allergy Clin Immunol.
atopic dermatitis	CDSN	Expression profiling by array, count, 3 development stage, 2 disease state, 11 individual sets	GPL570	GSE32924 Samples	Nonlesional atopic dermatitis skin is characterized by broad terminal differentiation defects and variable immune abnormalities.	J Allergy Clin Immunol.
atopic dermatitis	POF1B	Expression profiling by array, count, 3 development stage, 2 disease state, 11 individual sets	GPL570	GSE32924 Samples	Nonlesional atopic dermatitis skin is characterized by broad terminal differentiation defects and variable immune abnormalities.	J Allergy Clin Immunol.
atopic dermatitis	CRCT1	Expression profiling by array, count, 3 development stage, 2 disease state, 11 individual sets	GPL570	GSE32924 Samples	Nonlesional atopic dermatitis skin is characterized by broad terminal differentiation defects and variable immune abnormalities.	J Allergy Clin Immunol.
atopic dermatitis	ANXA9	Expression profiling by array, count, 3 development stage, 2 disease state, 11 individual sets	GPL570	GSE32924 Samples	Nonlesional atopic dermatitis skin is characterized by broad terminal differentiation defects and variable immune abnormalities.	J Allergy Clin Immunol.
atopic dermatitis	IL1F7	Expression profiling by array, count, 3 development stage, 2 disease state, 11 individual sets	GPL570	GSE32924 Samples	Nonlesional atopic dermatitis skin is characterized by broad terminal differentiation defects and variable immune abnormalities.	J Allergy Clin Immunol.
atopic dermatitis	BTC	Expression profiling by array, count, 3 development stage, 2 disease state, 11 individual sets	GPL570	GSE32924 Samples	Nonlesional atopic dermatitis skin is characterized by broad terminal differentiation defects and variable immune abnormalities.	J Allergy Clin Immunol.
atopic dermatitis	GTDC1	Expression profiling by array, count, 3 development stage, 2 disease state, 11 individual sets	GPL570	GSE32924 Samples	Nonlesional atopic dermatitis skin is characterized by broad terminal differentiation defects and variable immune abnormalities.	J Allergy Clin Immunol.
atopic dermatitis	CDCP1	Expression profiling by array, count, 3 development stage, 2 disease state, 11 individual sets	GPL570	GSE32924 Samples	Nonlesional atopic dermatitis skin is characterized by broad terminal differentiation defects and variable immune abnormalities.	J Allergy Clin Immunol.
atopic dermatitis	TFE3	Expression profiling by array, count, 3 development stage, 2 disease state, 11 individual sets	GPL570	GSE32924 Samples	Nonlesional atopic dermatitis skin is characterized by broad terminal differentiation defects and variable immune abnormalities.	J Allergy Clin Immunol.
atopic dermatitis	BCL2L2	Expression profiling by array, count, 3 development stage, 2 disease state, 11 individual sets	GPL570	GSE32924 Samples	Nonlesional atopic dermatitis skin is characterized by broad terminal differentiation defects and variable immune abnormalities.	J Allergy Clin Immunol.
atopic dermatitis	EIF4EBP2	Expression profiling by array, count, 3 development stage, 2 disease state, 11 individual sets	GPL570	GSE32924 Samples	Nonlesional atopic dermatitis skin is characterized by broad terminal differentiation defects and variable immune abnormalities.	J Allergy Clin Immunol.
atopic dermatitis	B3GALNT2	Expression profiling by array, count, 3 development stage, 2 disease state, 11 individual sets	GPL570	GSE32924 Samples	Nonlesional atopic dermatitis skin is characterized by broad terminal differentiation defects and variable immune abnormalities.	J Allergy Clin Immunol.
atopic dermatitis	NA	Expression profiling by array, count, 3 development stage, 2 disease state, 11 individual sets	GPL570	GSE32924 Samples	Nonlesional atopic dermatitis skin is characterized by broad terminal differentiation defects and variable immune abnormalities.	J Allergy Clin Immunol.
atopic dermatitis	EXOSC6	Expression profiling by array, count, 3 development stage, 2 disease state, 11 individual sets	GPL570	GSE32924 Samples	Nonlesional atopic dermatitis skin is characterized by broad terminal differentiation defects and variable immune abnormalities.	J Allergy Clin Immunol.
atopic dermatitis	TP63	Expression profiling by array, count, 3 development stage, 2 disease state, 11 individual sets	GPL570	GSE32924 Samples	Nonlesional atopic dermatitis skin is characterized by broad terminal differentiation defects and variable immune abnormalities.	J Allergy Clin Immunol.
atopic dermatitis	C1ORF174	Expression profiling by array, count, 3 development stage, 2 disease state, 11 individual sets	GPL570	GSE32924 Samples	Nonlesional atopic dermatitis skin is characterized by broad terminal differentiation defects and variable immune abnormalities.	J Allergy Clin Immunol.
atopic dermatitis	MMP14	Expression profiling by array, count, 3 development stage, 2 disease state, 11 individual sets	GPL570	GSE32924 Samples	Nonlesional atopic dermatitis skin is characterized by broad terminal differentiation defects and variable immune abnormalities.	J Allergy Clin Immunol.
atopic dermatitis	EPAS1	Expression profiling by array, count, 3 development stage, 2 disease state, 11 individual sets	GPL570	GSE32924 Samples	Nonlesional atopic dermatitis skin is characterized by broad terminal differentiation defects and variable immune abnormalities.	J Allergy Clin Immunol.
atopic dermatitis	TP53	Expression profiling by array, count, 3 development stage, 2 disease state, 11 individual sets	GPL570	GSE32924 Samples	Nonlesional atopic dermatitis skin is characterized by broad terminal differentiation defects and variable immune abnormalities.	J Allergy Clin Immunol.
atopic dermatitis	GAS1	Expression profiling by array, count, 3 development stage, 2 disease state, 11 individual sets	GPL570	GSE32924 Samples	Nonlesional atopic dermatitis skin is characterized by broad terminal differentiation defects and variable immune abnormalities.	J Allergy Clin Immunol.
atopic dermatitis	ARHGEF7	Expression profiling by array, count, 3 development stage, 2 disease state, 11 individual sets	GPL570	GSE32924 Samples	Nonlesional atopic dermatitis skin is characterized by broad terminal differentiation defects and variable immune abnormalities.	J Allergy Clin Immunol.
atopic dermatitis	SUPT16H	Expression profiling by array, count, 3 development stage, 2 disease state, 11 individual sets	GPL570	GSE32924 Samples	Nonlesional atopic dermatitis skin is characterized by broad terminal differentiation defects and variable immune abnormalities.	J Allergy Clin Immunol.
atopic dermatitis	EXOC4	Expression profiling by array, count, 3 development stage, 2 disease state, 11 individual sets	GPL570	GSE32924 Samples	Nonlesional atopic dermatitis skin is characterized by broad terminal differentiation defects and variable immune abnormalities.	J Allergy Clin Immunol.
atopic dermatitis	NSMAF	Expression profiling by array, count, 3 development stage, 2 disease state, 11 individual sets	GPL570	GSE32924 Samples	Nonlesional atopic dermatitis skin is characterized by broad terminal differentiation defects and variable immune abnormalities.	J Allergy Clin Immunol.
atopic dermatitis	ABHD2	Expression profiling by array, count, 3 development stage, 2 disease state, 11 individual sets	GPL570	GSE32924 Samples	Nonlesional atopic dermatitis skin is characterized by broad terminal differentiation defects and variable immune abnormalities.	J Allergy Clin Immunol.
atopic dermatitis	ORMDL3	Expression profiling by array, count, 3 development stage, 2 disease state, 11 individual sets	GPL570	GSE32924 Samples	Nonlesional atopic dermatitis skin is characterized by broad terminal differentiation defects and variable immune abnormalities.	J Allergy Clin Immunol.
atopic dermatitis	ARHGAP29	Expression profiling by array, count, 3 development stage, 2 disease state, 11 individual sets	GPL570	GSE32924 Samples	Nonlesional atopic dermatitis skin is characterized by broad terminal differentiation defects and variable immune abnormalities.	J Allergy Clin Immunol.
atopic dermatitis	ABCC9	Expression profiling by array, count, 3 development stage, 2 disease state, 11 individual sets	GPL570	GSE32924 Samples	Nonlesional atopic dermatitis skin is characterized by broad terminal differentiation defects and variable immune abnormalities.	J Allergy Clin Immunol.
atopic dermatitis	UBASH3B	Expression profiling by array, count, 3 development stage, 2 disease state, 11 individual sets	GPL570	GSE32924 Samples	Nonlesional atopic dermatitis skin is characterized by broad terminal differentiation defects and variable immune abnormalities.	J Allergy Clin Immunol.
atopic dermatitis	PLEKHA2	Expression profiling by array, count, 3 development stage, 2 disease state, 11 individual sets	GPL570	GSE32924 Samples	Nonlesional atopic dermatitis skin is characterized by broad terminal differentiation defects and variable immune abnormalities.	J Allergy Clin Immunol.
atopic dermatitis	SFRS12	Expression profiling by array, count, 3 development stage, 2 disease state, 11 individual sets	GPL570	GSE32924 Samples	Nonlesional atopic dermatitis skin is characterized by broad terminal differentiation defects and variable immune abnormalities.	J Allergy Clin Immunol.
atopic dermatitis	NA	Expression profiling by array, count, 3 development stage, 2 disease state, 11 individual sets	GPL570	GSE32924 Samples	Nonlesional atopic dermatitis skin is characterized by broad terminal differentiation defects and variable immune abnormalities.	J Allergy Clin Immunol.
atopic dermatitis	MYH10	Expression profiling by array, count, 3 development stage, 2 disease state, 11 individual sets	GPL570	GSE32924 Samples	Nonlesional atopic dermatitis skin is characterized by broad terminal differentiation defects and variable immune abnormalities.	J Allergy Clin Immunol.
atopic dermatitis	PRDM6	Expression profiling by array, count, 3 development stage, 2 disease state, 11 individual sets	GPL570	GSE32924 Samples	Nonlesional atopic dermatitis skin is characterized by broad terminal differentiation defects and variable immune abnormalities.	J Allergy Clin Immunol.

atopic dermatitis	ARG1	Expression profiling by array, count, 3 development stage, 2 disease state, 11 individual sets	GPL570	GSE32924 Samples	Nonlesional atopic dermatitis skin is characterized by broad terminal differentiation defects and variable immune abnormalities.	J Allergy Clin Immunol.
atopic dermatitis	COPA	Expression profiling by array, count, 3 development stage, 2 disease state, 11 individual sets	GPL570	GSE32924 Samples	Nonlesional atopic dermatitis skin is characterized by broad terminal differentiation defects and variable immune abnormalities.	J Allergy Clin Immunol.
atopic dermatitis	TNPO2	Expression profiling by array, count, 3 development stage, 2 disease state, 11 individual sets	GPL570	GSE32924 Samples	Nonlesional atopic dermatitis skin is characterized by broad terminal differentiation defects and variable immune abnormalities.	J Allergy Clin Immunol.
atopic dermatitis	DHX9	Expression profiling by array, count, 3 development stage, 2 disease state, 11 individual sets	GPL570	GSE32924 Samples	Nonlesional atopic dermatitis skin is characterized by broad terminal differentiation defects and variable immune abnormalities.	J Allergy Clin Immunol.
atopic dermatitis	KCTD20	Expression profiling by array, count, 3 development stage, 2 disease state, 11 individual sets	GPL570	GSE32924 Samples	Nonlesional atopic dermatitis skin is characterized by broad terminal differentiation defects and variable immune abnormalities.	J Allergy Clin Immunol.
atopic dermatitis	PDXDC1	Expression profiling by array, count, 3 development stage, 2 disease state, 11 individual sets	GPL570	GSE32924 Samples	Nonlesional atopic dermatitis skin is characterized by broad terminal differentiation defects and variable immune abnormalities.	J Allergy Clin Immunol.
atopic dermatitis	TMEM86A	Expression profiling by array, count, 3 development stage, 2 disease state, 11 individual sets	GPL570	GSE32924 Samples	Nonlesional atopic dermatitis skin is characterized by broad terminal differentiation defects and variable immune abnormalities.	J Allergy Clin Immunol.
atopic dermatitis	CNOT6L	Expression profiling by array, count, 3 development stage, 2 disease state, 11 individual sets	GPL570	GSE32924 Samples	Nonlesional atopic dermatitis skin is characterized by broad terminal differentiation defects and variable immune abnormalities.	J Allergy Clin Immunol.
atopic dermatitis	TFAP2C	Expression profiling by array, count, 3 development stage, 2 disease state, 11 individual sets	GPL570	GSE32924 Samples	Nonlesional atopic dermatitis skin is characterized by broad terminal differentiation defects and variable immune abnormalities.	J Allergy Clin Immunol.
atopic dermatitis	SLC6A9	Expression profiling by array, count, 3 development stage, 2 disease state, 11 individual sets	GPL570	GSE32924 Samples	Nonlesional atopic dermatitis skin is characterized by broad terminal differentiation defects and variable immune abnormalities.	J Allergy Clin Immunol.
atopic dermatitis	LUZP6	Expression profiling by array, count, 3 development stage, 2 disease state, 11 individual sets	GPL570	GSE32924 Samples	Nonlesional atopic dermatitis skin is characterized by broad terminal differentiation defects and variable immune abnormalities.	J Allergy Clin Immunol.
atopic dermatitis	LYRM5	Expression profiling by array, count, 3 development stage, 2 disease state, 11 individual sets	GPL570	GSE32924 Samples	Nonlesional atopic dermatitis skin is characterized by broad terminal differentiation defects and variable immune abnormalities.	J Allergy Clin Immunol.
atopic dermatitis	GATM	Expression profiling by array, count, 3 development stage, 2 disease state, 11 individual sets	GPL570	GSE32924 Samples	Nonlesional atopic dermatitis skin is characterized by broad terminal differentiation defects and variable immune abnormalities.	J Allergy Clin Immunol.
atopic dermatitis	TNFRSF19	Expression profiling by array, count, 3 development stage, 2 disease state, 11 individual sets	GPL570	GSE32924 Samples	Nonlesional atopic dermatitis skin is characterized by broad terminal differentiation defects and variable immune abnormalities.	J Allergy Clin Immunol.
atopic dermatitis	IL1RN	Expression profiling by array, count, 3 development stage, 2 disease state, 11 individual sets	GPL570	GSE32924 Samples	Nonlesional atopic dermatitis skin is characterized by broad terminal differentiation defects and variable immune abnormalities.	J Allergy Clin Immunol.
atopic dermatitis	ZNF770	Expression profiling by array, count, 3 development stage, 2 disease state, 11 individual sets	GPL570	GSE32924 Samples	Nonlesional atopic dermatitis skin is characterized by broad terminal differentiation defects and variable immune abnormalities.	J Allergy Clin Immunol.
atopic dermatitis	LOC284242	Expression profiling by array, count, 3 development stage, 2 disease state, 11 individual sets	GPL570	GSE32924 Samples	Nonlesional atopic dermatitis skin is characterized by broad terminal differentiation defects and variable immune abnormalities.	J Allergy Clin Immunol.
atopic dermatitis	NA	Expression profiling by array, count, 3 development stage, 2 disease state, 11 individual sets	GPL570	GSE32924 Samples	Nonlesional atopic dermatitis skin is characterized by broad terminal differentiation defects and variable immune abnormalities.	J Allergy Clin Immunol.
atopic dermatitis	ACTR2	Expression profiling by array, count, 3 development stage, 2 disease state, 11 individual sets	GPL570	GSE32924 Samples	Nonlesional atopic dermatitis skin is characterized by broad terminal differentiation defects and variable immune abnormalities.	J Allergy Clin Immunol.
atopic dermatitis	ACTR2	Expression profiling by array, count, 3 development stage, 2 disease state, 11 individual sets	GPL570	GSE32924 Samples	Nonlesional atopic dermatitis skin is characterized by broad terminal differentiation defects and variable immune abnormalities.	J Allergy Clin Immunol.
atopic dermatitis	GM2A	Expression profiling by array, count, 3 development stage, 2 disease state, 11 individual sets	GPL570	GSE32924 Samples	Nonlesional atopic dermatitis skin is characterized by broad terminal differentiation defects and variable immune abnormalities.	J Allergy Clin Immunol.
atopic dermatitis	GM2A	Expression profiling by array, count, 3 development stage, 2 disease state, 11 individual sets	GPL570	GSE32924 Samples	Nonlesional atopic dermatitis skin is characterized by broad terminal differentiation defects and variable immune abnormalities.	J Allergy Clin Immunol.
atopic dermatitis	CASP14	Expression profiling by array, count, 3 development stage, 2 disease state, 11 individual sets	GPL570	GSE32924 Samples	Nonlesional atopic dermatitis skin is characterized by broad terminal differentiation defects and variable immune abnormalities.	J Allergy Clin Immunol.
atopic dermatitis	SERPINB12	Expression profiling by array, count, 3 development stage, 2 disease state, 11 individual sets	GPL570	GSE32924 Samples	Nonlesional atopic dermatitis skin is characterized by broad terminal differentiation defects and variable immune abnormalities.	J Allergy Clin Immunol.
atopic dermatitis	MAP3K1	Expression profiling by array, count, 3 development stage, 2 disease state, 11 individual sets	GPL570	GSE32924 Samples	Nonlesional atopic dermatitis skin is characterized by broad terminal differentiation defects and variable immune abnormalities.	J Allergy Clin Immunol.
atopic dermatitis	CCDC123	Expression profiling by array, count, 3 development stage, 2 disease state, 11 individual sets	GPL570	GSE32924 Samples	Nonlesional atopic dermatitis skin is characterized by broad terminal differentiation defects and variable immune abnormalities.	J Allergy Clin Immunol.
atopic dermatitis	NA	Expression profiling by array, count, 3 development stage, 2 disease state, 11 individual sets	GPL570	GSE32924 Samples	Nonlesional atopic dermatitis skin is characterized by broad terminal differentiation defects and variable immune abnormalities.	J Allergy Clin Immunol.
atopic dermatitis	MCOLN3	Expression profiling by array, count, 3 development stage, 2 disease state, 11 individual sets	GPL570	GSE32924 Samples	Nonlesional atopic dermatitis skin is characterized by broad terminal differentiation defects and variable immune abnormalities.	J Allergy Clin Immunol.
atopic dermatitis	NA	Expression profiling by array, count, 3 development stage, 2 disease state, 11 individual sets	GPL570	GSE32924 Samples	Nonlesional atopic dermatitis skin is characterized by broad terminal differentiation defects and variable immune abnormalities.	J Allergy Clin Immunol.
atopic dermatitis	FMNL2	Expression profiling by array, count, 3 development stage, 2 disease state, 11 individual sets	GPL570	GSE32924 Samples	Nonlesional atopic dermatitis skin is characterized by broad terminal differentiation defects and variable immune abnormalities.	J Allergy Clin Immunol.
atopic dermatitis	PPFIBP1	Expression profiling by array, count, 3 development stage, 2 disease state, 11 individual sets	GPL570	GSE32924 Samples	Nonlesional atopic dermatitis skin is characterized by broad terminal differentiation defects and variable immune abnormalities.	J Allergy Clin Immunol.
atopic dermatitis	SMCHD1	Expression profiling by array, count, 3 development stage, 2 disease state, 11 individual sets	GPL570	GSE32924 Samples	Nonlesional atopic dermatitis skin is characterized by broad terminal differentiation defects and variable immune abnormalities.	J Allergy Clin Immunol.
atopic dermatitis	LARP1B	Expression profiling by array, count, 3 development stage, 2 disease state, 11 individual sets	GPL570	GSE32924 Samples	Nonlesional atopic dermatitis skin is characterized by broad terminal differentiation defects and variable immune abnormalities.	J Allergy Clin Immunol.
atopic dermatitis	TBL1X	Expression profiling by array, count, 3 development stage, 2 disease state, 11 individual sets	GPL570	GSE32924 Samples	Nonlesional atopic dermatitis skin is characterized by broad terminal differentiation defects and variable immune abnormalities.	J Allergy Clin Immunol.
atopic dermatitis	NA	Expression profiling by array, count, 3 development stage, 2 disease state, 11 individual sets	GPL570	GSE32924 Samples	Nonlesional atopic dermatitis skin is characterized by broad terminal differentiation defects and variable immune abnormalities.	J Allergy Clin Immunol.
atopic dermatitis	PIK3R1	Expression profiling by array, count, 3 development stage, 2 disease state, 11 individual sets	GPL570	GSE32924 Samples	Nonlesional atopic dermatitis skin is characterized by broad terminal differentiation defects and variable immune abnormalities.	J Allergy Clin Immunol.
atopic dermatitis	NA	Expression profiling by array, count, 3 development stage, 2 disease state, 11 individual sets	GPL570	GSE32924 Samples	Nonlesional atopic dermatitis skin is characterized by broad terminal differentiation defects and variable immune abnormalities.	J Allergy Clin Immunol.
atopic dermatitis	HECTD1	Expression profiling by array, count, 3 development stage, 2 disease state, 11 individual sets	GPL570	GSE32924 Samples	Nonlesional atopic dermatitis skin is characterized by broad terminal differentiation defects and variable immune abnormalities.	J Allergy Clin Immunol.
atopic dermatitis	NA	Expression profiling by array, count, 3 development stage, 2 disease state, 11 individual sets	GPL570	GSE32924 Samples	Nonlesional atopic dermatitis skin is characterized by broad terminal differentiation defects and variable immune abnormalities.	J Allergy Clin Immunol.
atopic dermatitis	NA	Expression profiling by array, count, 3 development stage, 2 disease state, 11 individual sets	GPL570	GSE32924 Samples	Nonlesional atopic dermatitis skin is characterized by broad terminal differentiation defects and variable immune abnormalities.	J Allergy Clin Immunol.
atopic dermatitis	MRVI1	Expression profiling by array, count, 3 development stage, 2 disease state, 11 individual sets	GPL570	GSE32924 Samples	Nonlesional atopic dermatitis skin is characterized by broad terminal differentiation defects and variable immune abnormalities.	J Allergy Clin Immunol.

atopic dermatitis	BOD1L	Expression profiling by array, count, 3 development stage, 2 disease state, 11 individual sets	GPL570	GSE32924 Samples	Nonlesional atopic dermatitis skin is characterized by broad terminal differentiation defects and variable immune abnormalities.	J Allergy Clin Immunol.
atopic dermatitis	ZNF681	Expression profiling by array, count, 3 development stage, 2 disease state, 11 individual sets	GPL570	GSE32924 Samples	Nonlesional atopic dermatitis skin is characterized by broad terminal differentiation defects and variable immune abnormalities.	J Allergy Clin Immunol.
atopic dermatitis	NA	Expression profiling by array, count, 3 development stage, 2 disease state, 11 individual sets	GPL570	GSE32924 Samples	Nonlesional atopic dermatitis skin is characterized by broad terminal differentiation defects and variable immune abnormalities.	J Allergy Clin Immunol.
atopic dermatitis	LOC149832	Expression profiling by array, count, 3 development stage, 2 disease state, 11 individual sets	GPL570	GSE32924 Samples	Nonlesional atopic dermatitis skin is characterized by broad terminal differentiation defects and variable immune abnormalities.	J Allergy Clin Immunol.
atopic dermatitis	ADAMTSL3	Expression profiling by array, count, 3 development stage, 2 disease state, 11 individual sets	GPL570	GSE32924 Samples	Nonlesional atopic dermatitis skin is characterized by broad terminal differentiation defects and variable immune abnormalities.	J Allergy Clin Immunol.
atopic dermatitis	TAC1	Expression profiling by array, count, 3 development stage, 2 disease state, 11 individual sets	GPL570	GSE32924 Samples	Nonlesional atopic dermatitis skin is characterized by broad terminal differentiation defects and variable immune abnormalities.	J Allergy Clin Immunol.
atopic dermatitis	NA	Expression profiling by array, count, 3 development stage, 2 disease state, 11 individual sets	GPL570	GSE32924 Samples	Nonlesional atopic dermatitis skin is characterized by broad terminal differentiation defects and variable immune abnormalities.	J Allergy Clin Immunol.
atopic dermatitis	NA	Expression profiling by array, count, 3 development stage, 2 disease state, 11 individual sets	GPL570	GSE32924 Samples	Nonlesional atopic dermatitis skin is characterized by broad terminal differentiation defects and variable immune abnormalities.	J Allergy Clin Immunol.
atopic dermatitis	FAM108B1	Expression profiling by array, count, 3 development stage, 2 disease state, 11 individual sets	GPL570	GSE32924 Samples	Nonlesional atopic dermatitis skin is characterized by broad terminal differentiation defects and variable immune abnormalities.	J Allergy Clin Immunol.
atopic dermatitis	ZNF711	Expression profiling by array, count, 3 development stage, 2 disease state, 11 individual sets	GPL570	GSE32924 Samples	Nonlesional atopic dermatitis skin is characterized by broad terminal differentiation defects and variable immune abnormalities.	J Allergy Clin Immunol.
atopic dermatitis	ANKFN1	Expression profiling by array, count, 3 development stage, 2 disease state, 11 individual sets	GPL570	GSE32924 Samples	Nonlesional atopic dermatitis skin is characterized by broad terminal differentiation defects and variable immune abnormalities.	J Allergy Clin Immunol.
atopic dermatitis	IL1F10	Expression profiling by array, count, 3 development stage, 2 disease state, 11 individual sets	GPL570	GSE32924 Samples	Nonlesional atopic dermatitis skin is characterized by broad terminal differentiation defects and variable immune abnormalities.	J Allergy Clin Immunol.
atopic dermatitis	CLDN23	Expression profiling by array, count, 3 development stage, 2 disease state, 11 individual sets	GPL570	GSE32924 Samples	Nonlesional atopic dermatitis skin is characterized by broad terminal differentiation defects and variable immune abnormalities.	J Allergy Clin Immunol.
atopic dermatitis	HECTD1	Expression profiling by array, count, 3 development stage, 2 disease state, 11 individual sets	GPL570	GSE32924 Samples	Nonlesional atopic dermatitis skin is characterized by broad terminal differentiation defects and variable immune abnormalities.	J Allergy Clin Immunol.
atopic dermatitis	CYP2C9	Expression profiling by array, count, 3 development stage, 2 disease state, 11 individual sets	GPL570	GSE32924 Samples	Nonlesional atopic dermatitis skin is characterized by broad terminal differentiation defects and variable immune abnormalities.	J Allergy Clin Immunol.
atopic dermatitis	XK	Expression profiling by array, count, 3 development stage, 2 disease state, 11 individual sets	GPL570	GSE32924 Samples	Nonlesional atopic dermatitis skin is characterized by broad terminal differentiation defects and variable immune abnormalities.	J Allergy Clin Immunol.
atopic dermatitis	PLEKHA5	Expression profiling by array, count, 3 development stage, 2 disease state, 11 individual sets	GPL570	GSE32924 Samples	Nonlesional atopic dermatitis skin is characterized by broad terminal differentiation defects and variable immune abnormalities.	J Allergy Clin Immunol.
atopic dermatitis	SIGLEC6	Expression profiling by array, count, 3 development stage, 2 disease state, 11 individual sets	GPL570	GSE32924 Samples	Nonlesional atopic dermatitis skin is characterized by broad terminal differentiation defects and variable immune abnormalities.	J Allergy Clin Immunol.
atopic dermatitis	RGS13	Expression profiling by array, count, 3 development stage, 2 disease state, 11 individual sets	GPL570	GSE32924 Samples	Nonlesional atopic dermatitis skin is characterized by broad terminal differentiation defects and variable immune abnormalities.	J Allergy Clin Immunol.
atopic dermatitis	NA	Expression profiling by array, count, 3 development stage, 2 disease state, 11 individual sets	GPL570	GSE32924 Samples	Nonlesional atopic dermatitis skin is characterized by broad terminal differentiation defects and variable immune abnormalities.	J Allergy Clin Immunol.
atopic dermatitis	KANK1	Expression profiling by array, count, 3 development stage, 2 disease state, 11 individual sets	GPL570	GSE32924 Samples	Nonlesional atopic dermatitis skin is characterized by broad terminal differentiation defects and variable immune abnormalities.	J Allergy Clin Immunol.
atopic dermatitis	NARG1	Expression profiling by array, count, 3 development stage, 2 disease state, 11 individual sets	GPL570	GSE32924 Samples	Nonlesional atopic dermatitis skin is characterized by broad terminal differentiation defects and variable immune abnormalities.	J Allergy Clin Immunol.
atopic dermatitis	CP	Expression profiling by array, count, 3 development stage, 2 disease state, 11 individual sets	GPL570	GSE32924 Samples	Nonlesional atopic dermatitis skin is characterized by broad terminal differentiation defects and variable immune abnormalities.	J Allergy Clin Immunol.
atopic dermatitis	NA	Expression profiling by array, count, 3 development stage, 2 disease state, 11 individual sets	GPL570	GSE32924 Samples	Nonlesional atopic dermatitis skin is characterized by broad terminal differentiation defects and variable immune abnormalities.	J Allergy Clin Immunol.
atopic dermatitis	LOC728730	Expression profiling by array, count, 3 development stage, 2 disease state, 11 individual sets	GPL570	GSE32924 Samples	Nonlesional atopic dermatitis skin is characterized by broad terminal differentiation defects and variable immune abnormalities.	J Allergy Clin Immunol.
atopic dermatitis	UGGT2	Expression profiling by array, count, 3 development stage, 2 disease state, 11 individual sets	GPL570	GSE32924 Samples	Nonlesional atopic dermatitis skin is characterized by broad terminal differentiation defects and variable immune abnormalities.	J Allergy Clin Immunol.
atopic dermatitis	SYNE1	Expression profiling by array, count, 3 development stage, 2 disease state, 11 individual sets	GPL570	GSE32924 Samples	Nonlesional atopic dermatitis skin is characterized by broad terminal differentiation defects and variable immune abnormalities.	J Allergy Clin Immunol.
atopic dermatitis	SEPP1	Expression profiling by array, count, 3 development stage, 2 disease state, 11 individual sets	GPL570	GSE32924 Samples	Nonlesional atopic dermatitis skin is characterized by broad terminal differentiation defects and variable immune abnormalities.	J Allergy Clin Immunol.
atopic dermatitis	MALAT1	Expression profiling by array, count, 3 development stage, 2 disease state, 11 individual sets	GPL570	GSE32924 Samples	Nonlesional atopic dermatitis skin is characterized by broad terminal differentiation defects and variable immune abnormalities.	J Allergy Clin Immunol.
atopic dermatitis	CHD9	Expression profiling by array, count, 3 development stage, 2 disease state, 11 individual sets	GPL570	GSE32924 Samples	Nonlesional atopic dermatitis skin is characterized by broad terminal differentiation defects and variable immune abnormalities.	J Allergy Clin Immunol.
atopic dermatitis	CLINT1	Expression profiling by array, count, 3 development stage, 2 disease state, 11 individual sets	GPL570	GSE32924 Samples	Nonlesional atopic dermatitis skin is characterized by broad terminal differentiation defects and variable immune abnormalities.	J Allergy Clin Immunol.
atopic dermatitis	WAC	Expression profiling by array, count, 3 development stage, 2 disease state, 11 individual sets	GPL570	GSE32924 Samples	Nonlesional atopic dermatitis skin is characterized by broad terminal differentiation defects and variable immune abnormalities.	J Allergy Clin Immunol.
atopic dermatitis	EEA1	Expression profiling by array, count, 3 development stage, 2 disease state, 11 individual sets	GPL570	GSE32924 Samples	Nonlesional atopic dermatitis skin is characterized by broad terminal differentiation defects and variable immune abnormalities.	J Allergy Clin Immunol.
atopic dermatitis	NA	Expression profiling by array, count, 3 development stage, 2 disease state, 11 individual sets	GPL570	GSE32924 Samples	Nonlesional atopic dermatitis skin is characterized by broad terminal differentiation defects and variable immune abnormalities.	J Allergy Clin Immunol.
atopic dermatitis	NA	Expression profiling by array, count, 3 development stage, 2 disease state, 11 individual sets	GPL570	GSE32924 Samples	Nonlesional atopic dermatitis skin is characterized by broad terminal differentiation defects and variable immune abnormalities.	J Allergy Clin Immunol.
atopic dermatitis	HHATL	Expression profiling by array, count, 3 development stage, 2 disease state, 11 individual sets	GPL570	GSE32924 Samples	Nonlesional atopic dermatitis skin is characterized by broad terminal differentiation defects and variable immune abnormalities.	J Allergy Clin Immunol.
atopic dermatitis	SLC44A5	Expression profiling by array, count, 3 development stage, 2 disease state, 11 individual sets	GPL570	GSE32924 Samples	Nonlesional atopic dermatitis skin is characterized by broad terminal differentiation defects and variable immune abnormalities.	J Allergy Clin Immunol.
atopic dermatitis	UPK1A	Expression profiling by array, count, 3 development stage, 2 disease state, 11 individual sets	GPL570	GSE32924 Samples	Nonlesional atopic dermatitis skin is characterized by broad terminal differentiation defects and variable immune abnormalities.	J Allergy Clin Immunol.
atopic dermatitis	PSG1	Expression profiling by array, count, 3 development stage, 2 disease state, 11 individual sets	GPL570	GSE32924 Samples	Nonlesional atopic dermatitis skin is characterized by broad terminal differentiation defects and variable immune abnormalities.	J Allergy Clin Immunol.
atopic dermatitis	PSG7	Expression profiling by array, count, 3 development stage, 2 disease state, 11 individual sets	GPL570	GSE32924 Samples	Nonlesional atopic dermatitis skin is characterized by broad terminal differentiation defects and variable immune abnormalities.	J Allergy Clin Immunol.
atopic dermatitis	DDX17	Expression profiling by array, count, 3 development stage, 2 disease state, 11 individual sets	GPL570	GSE32924 Samples	Nonlesional atopic dermatitis skin is characterized by broad terminal differentiation defects and variable immune abnormalities.	J Allergy Clin Immunol.

atopic dermatitis	KCTD4	Expression profiling by array, count, 3 development stage, 2 disease state, 11 individual sets	GPL570	GSE32924 Samples	Nonlesional atopic dermatitis skin is characterized by broad terminal differentiation defects and variable immune abnormalities.	J Allergy Clin Immunol.
atopic dermatitis	NA	Expression profiling by array, count, 3 development stage, 2 disease state, 11 individual sets	GPL570	GSE32924 Samples	Nonlesional atopic dermatitis skin is characterized by broad terminal differentiation defects and variable immune abnormalities.	J Allergy Clin Immunol.
atopic dermatitis	GPR115	Expression profiling by array, count, 3 development stage, 2 disease state, 11 individual sets	GPL570	GSE32924 Samples	Nonlesional atopic dermatitis skin is characterized by broad terminal differentiation defects and variable immune abnormalities.	J Allergy Clin Immunol.
atopic dermatitis	PTPN21	Expression profiling by array, count, 3 development stage, 2 disease state, 11 individual sets	GPL570	GSE32924 Samples	Nonlesional atopic dermatitis skin is characterized by broad terminal differentiation defects and variable immune abnormalities.	J Allergy Clin Immunol.
atopic dermatitis	LCE1E	Expression profiling by array, count, 3 development stage, 2 disease state, 11 individual sets	GPL570	GSE32924 Samples	Nonlesional atopic dermatitis skin is characterized by broad terminal differentiation defects and variable immune abnormalities.	J Allergy Clin Immunol.
atopic dermatitis	BTC	Expression profiling by array, count, 3 development stage, 2 disease state, 11 individual sets	GPL570	GSE32924 Samples	Nonlesional atopic dermatitis skin is characterized by broad terminal differentiation defects and variable immune abnormalities.	J Allergy Clin Immunol.
atopic dermatitis	BMP7	Expression profiling by array, count, 3 development stage, 2 disease state, 11 individual sets	GPL570	GSE32924 Samples	Nonlesional atopic dermatitis skin is characterized by broad terminal differentiation defects and variable immune abnormalities.	J Allergy Clin Immunol.
atopic dermatitis	NA	Expression profiling by array, count, 3 development stage, 2 disease state, 11 individual sets	GPL570	GSE32924 Samples	Nonlesional atopic dermatitis skin is characterized by broad terminal differentiation defects and variable immune abnormalities.	J Allergy Clin Immunol.
atopic dermatitis	ATP6V1A	Expression profiling by array, count, 3 development stage, 2 disease state, 11 individual sets	GPL570	GSE32924 Samples	Nonlesional atopic dermatitis skin is characterized by broad terminal differentiation defects and variable immune abnormalities.	J Allergy Clin Immunol.
atopic dermatitis	AHNAK	Expression profiling by array, count, 3 development stage, 2 disease state, 11 individual sets	GPL570	GSE32924 Samples	Nonlesional atopic dermatitis skin is characterized by broad terminal differentiation defects and variable immune abnormalities.	J Allergy Clin Immunol.
atopic dermatitis	NA	Expression profiling by array, count, 3 development stage, 2 disease state, 11 individual sets	GPL570	GSE32924 Samples	Nonlesional atopic dermatitis skin is characterized by broad terminal differentiation defects and variable immune abnormalities.	J Allergy Clin Immunol.
atopic dermatitis	SP8	Expression profiling by array, count, 3 development stage, 2 disease state, 11 individual sets	GPL570	GSE32924 Samples	Nonlesional atopic dermatitis skin is characterized by broad terminal differentiation defects and variable immune abnormalities.	J Allergy Clin Immunol.
atopic dermatitis	AGR3	Expression profiling by array, count, 3 development stage, 2 disease state, 11 individual sets	GPL570	GSE32924 Samples	Nonlesional atopic dermatitis skin is characterized by broad terminal differentiation defects and variable immune abnormalities.	J Allergy Clin Immunol.
atopic dermatitis	ABCA13	Expression profiling by array, count, 3 development stage, 2 disease state, 11 individual sets	GPL570	GSE32924 Samples	Nonlesional atopic dermatitis skin is characterized by broad terminal differentiation defects and variable immune abnormalities.	J Allergy Clin Immunol.
atopic dermatitis	ABCA13	Expression profiling by array, count, 3 development stage, 2 disease state, 11 individual sets	GPL570	GSE32924 Samples	Nonlesional atopic dermatitis skin is characterized by broad terminal differentiation defects and variable immune abnormalities.	J Allergy Clin Immunol.
atopic dermatitis	MSMB	Expression profiling by array, count, 3 development stage, 2 disease state, 11 individual sets	GPL570	GSE32924 Samples	Nonlesional atopic dermatitis skin is characterized by broad terminal differentiation defects and variable immune abnormalities.	J Allergy Clin Immunol.
atopic dermatitis	MSMB	Expression profiling by array, count, 3 development stage, 2 disease state, 11 individual sets	GPL570	GSE32924 Samples	Nonlesional atopic dermatitis skin is characterized by broad terminal differentiation defects and variable immune abnormalities.	J Allergy Clin Immunol.
Atopic dermatitis	ABCA1	Expression profiling by array	GPL96	GSE5667	Early cutaneous gene transcription changes in adult atopic dermatitis and potential clinical implications	Exp Dermatol.
Atopic dermatitis	APM1	Expression profiling by array	GPL96	GSE5667	Early cutaneous gene transcription changes in adult atopic dermatitis and potential clinical implications	Exp Dermatol.
Atopic dermatitis	CRN	Expression profiling by array	GPL96	GSE5667	Early cutaneous gene transcription changes in adult atopic dermatitis and potential clinical implications	Exp Dermatol.
Atopic dermatitis	FABP4	Expression profiling by array	GPL96	GSE5667	Early cutaneous gene transcription changes in adult atopic dermatitis and potential clinical implications	Exp Dermatol.
Atopic dermatitis	GPD1	Expression profiling by array	GPL96	GSE5667	Early cutaneous gene transcription changes in adult atopic dermatitis and potential clinical implications	Exp Dermatol.
Atopic dermatitis	HMGCS2	Expression profiling by array	GPL96	GSE5667	Early cutaneous gene transcription changes in adult atopic dermatitis and potential clinical implications	Exp Dermatol.
Atopic dermatitis	LEPR	Expression profiling by array	GPL96	GSE5667	Early cutaneous gene transcription changes in adult atopic dermatitis and potential clinical implications	Exp Dermatol.
Atopic dermatitis	LPL	Expression profiling by array	GPL96	GSE5667	Early cutaneous gene transcription changes in adult atopic dermatitis and potential clinical implications	Exp Dermatol.
Atopic dermatitis	PDK4	Expression profiling by array	GPL96	GSE5667	Early cutaneous gene transcription changes in adult atopic dermatitis and potential clinical implications	Exp Dermatol.
Atopic dermatitis	PLIN	Expression profiling by array	GPL96	GSE5667	Early cutaneous gene transcription changes in adult atopic dermatitis and potential clinical implications	Exp Dermatol.
Atopic dermatitis	UBE2I	Expression profiling by array	GPL96	GSE5667	Early cutaneous gene transcription changes in adult atopic dermatitis and potential clinical implications	Exp Dermatol.
Atopic dermatitis	PPARA4	Expression profiling by array	GPL96	GSE5667	Early cutaneous gene transcription changes in adult atopic dermatitis and potential clinical implications	Exp Dermatol.
Atopic dermatitis	PPARG	Expression profiling by array	GPL96	GSE5667	Early cutaneous gene transcription changes in adult atopic dermatitis and potential clinical implications	Exp Dermatol.
Atopic dermatitis	PPARGC1A	Expression profiling by array	GPL96	GSE5667	Early cutaneous gene transcription changes in adult atopic dermatitis and potential clinical implications	Exp Dermatol.
atopic dermatitis	ELAFIN				High Expression Levels of Keratinocyte Antimicrobial Proteins in Psoriasis Compared with Atopic Dermatitis	J invest Dermatol.
atopic dermatitis	HBD1				High Expression Levels of Keratinocyte Antimicrobial Proteins in Psoriasis Compared with Atopic Dermatitis	J invest Dermatol.
atopic dermatitis	HBD2				High Expression Levels of Keratinocyte Antimicrobial Proteins in Psoriasis Compared with Atopic Dermatitis	J invest Dermatol.
atopic dermatitis	HBD3				High Expression Levels of Keratinocyte Antimicrobial Proteins in Psoriasis Compared with Atopic Dermatitis	J invest Dermatol.
atopic dermatitis	K10				High Expression Levels of Keratinocyte Antimicrobial Proteins in Psoriasis Compared with Atopic Dermatitis	J invest Dermatol.
atopic dermatitis	K16				High Expression Levels of Keratinocyte Antimicrobial Proteins in Psoriasis Compared with Atopic Dermatitis	J invest Dermatol.
atopic dermatitis	K17				High Expression Levels of Keratinocyte Antimicrobial Proteins in Psoriasis Compared with Atopic Dermatitis	J invest Dermatol.
atopic dermatitis	LL37				High Expression Levels of Keratinocyte Antimicrobial Proteins in Psoriasis Compared with Atopic Dermatitis	J invest Dermatol.
atopic dermatitis	MRP14				High Expression Levels of Keratinocyte Antimicrobial Proteins in Psoriasis Compared with Atopic Dermatitis	J invest Dermatol.
atopic dermatitis	MRP8				High Expression Levels of Keratinocyte Antimicrobial Proteins in Psoriasis Compared with Atopic Dermatitis	J invest Dermatol.
atopic dermatitis	TLR2				High Expression Levels of Keratinocyte Antimicrobial Proteins in Psoriasis Compared with Atopic Dermatitis	J invest Dermatol.
atopic dermatitis	TLR3				High Expression Levels of Keratinocyte Antimicrobial Proteins in Psoriasis Compared with Atopic Dermatitis	J invest Dermatol.
Atopic Eczema	CRISP3	Expression profiling by array	GPL7191	GSE12511	Global Expression Profiling in Atopic Eczema Reveals Reciprocal Expression of Inflammatory and Lipid Genes	Plos One
Atopic Eczema	PTGIS	Expression profiling by array	GPL7191	GSE12511	Global Expression Profiling in Atopic Eczema Reveals Reciprocal Expression of Inflammatory and Lipid Genes	Plos One
Atopic Eczema	CTSK	Expression profiling by array	GPL7191	GSE12511	Global Expression Profiling in Atopic Eczema Reveals Reciprocal Expression of Inflammatory and Lipid Genes	Plos One
Atopic Eczema	COL6A3	Expression profiling by array	GPL7191	GSE12511	Global Expression Profiling in Atopic Eczema Reveals Reciprocal Expression of Inflammatory and Lipid Genes	Plos One
Atopic Eczema	CTSG	Expression profiling by array	GPL7191	GSE12511	Global Expression Profiling in Atopic Eczema Reveals Reciprocal Expression of Inflammatory and Lipid Genes	Plos One
Atopic Eczema	MAPKAPK3	Expression profiling by array	GPL7191	GSE12511	Global Expression Profiling in Atopic Eczema Reveals Reciprocal Expression of Inflammatory and Lipid Genes	Plos One
Atopic Eczema	NCF4	Expression profiling by array	GPL7191	GSE12511	Global Expression Profiling in Atopic Eczema Reveals Reciprocal Expression of Inflammatory and Lipid Genes	Plos One
Atopic Eczema	CD1C	Expression profiling by array	GPL7191	GSE12511	Global Expression Profiling in Atopic Eczema Reveals Reciprocal Expression of Inflammatory and Lipid Genes	Plos One
Atopic Eczema	SHANK2	Expression profiling by array	GPL7191	GSE12511	Global Expression Profiling in Atopic Eczema Reveals Reciprocal Expression of Inflammatory and Lipid Genes	Plos One
Atopic Eczema	COL6A2	Expression profiling by array	GPL7191	GSE12511	Global Expression Profiling in Atopic Eczema Reveals Reciprocal Expression of Inflammatory and Lipid Genes	Plos One

Atopic Eczema	MYBPC1	Expression profiling by array	GPL7191	GSE12511	Global Expression Profiling in Atopic Eczema Reveals Reciprocal Expression of Inflammatory and Lipid Genes	Plos One
Atopic Eczema	C1R	Expression profiling by array	GPL7191	GSE12511	Global Expression Profiling in Atopic Eczema Reveals Reciprocal Expression of Inflammatory and Lipid Genes	Plos One
Atopic Eczema	AEBP1	Expression profiling by array	GPL7191	GSE12511	Global Expression Profiling in Atopic Eczema Reveals Reciprocal Expression of Inflammatory and Lipid Genes	Plos One
Atopic Eczema	PTGER3	Expression profiling by array	GPL7191	GSE12511	Global Expression Profiling in Atopic Eczema Reveals Reciprocal Expression of Inflammatory and Lipid Genes	Plos One
Atopic Eczema	CCL18	Expression profiling by array	GPL7191	GSE12511	Global Expression Profiling in Atopic Eczema Reveals Reciprocal Expression of Inflammatory and Lipid Genes	Plos One
Atopic Eczema	CDSN	Expression profiling by array	GPL7191	GSE12511	Global Expression Profiling in Atopic Eczema Reveals Reciprocal Expression of Inflammatory and Lipid Genes	Plos One
Atopic Eczema	NBL1	Expression profiling by array	GPL7191	GSE12511	Global Expression Profiling in Atopic Eczema Reveals Reciprocal Expression of Inflammatory and Lipid Genes	Plos One
Atopic Eczema	COL6A1	Expression profiling by array	GPL7191	GSE12511	Global Expression Profiling in Atopic Eczema Reveals Reciprocal Expression of Inflammatory and Lipid Genes	Plos One
Atopic Eczema	C1ORF42	Expression profiling by array	GPL7191	GSE12511	Global Expression Profiling in Atopic Eczema Reveals Reciprocal Expression of Inflammatory and Lipid Genes	Plos One
Atopic Eczema	EMILIN2	Expression profiling by array	GPL7191	GSE12511	Global Expression Profiling in Atopic Eczema Reveals Reciprocal Expression of Inflammatory and Lipid Genes	Plos One
Atopic Eczema	FGF12	Expression profiling by array	GPL7191	GSE12511	Global Expression Profiling in Atopic Eczema Reveals Reciprocal Expression of Inflammatory and Lipid Genes	Plos One
Atopic Eczema	TNFRSF14	Expression profiling by array	GPL7191	GSE12511	Global Expression Profiling in Atopic Eczema Reveals Reciprocal Expression of Inflammatory and Lipid Genes	Plos One
Atopic Eczema	C1S	Expression profiling by array	GPL7191	GSE12511	Global Expression Profiling in Atopic Eczema Reveals Reciprocal Expression of Inflammatory and Lipid Genes	Plos One
Atopic Eczema	PTGES	Expression profiling by array	GPL7191	GSE12511	Global Expression Profiling in Atopic Eczema Reveals Reciprocal Expression of Inflammatory and Lipid Genes	Plos One
Atopic Eczema	TRAK2	Expression profiling by array	GPL7191	GSE12511	Global Expression Profiling in Atopic Eczema Reveals Reciprocal Expression of Inflammatory and Lipid Genes	Plos One
Atopic Eczema	NRM	Expression profiling by array	GPL7191	GSE12511	Global Expression Profiling in Atopic Eczema Reveals Reciprocal Expression of Inflammatory and Lipid Genes	Plos One
Atopic Eczema	GLTP	Expression profiling by array	GPL7191	GSE12511	Global Expression Profiling in Atopic Eczema Reveals Reciprocal Expression of Inflammatory and Lipid Genes	Plos One
Atopic Eczema	ACSL3	Expression profiling by array	GPL7191	GSE12511	Global Expression Profiling in Atopic Eczema Reveals Reciprocal Expression of Inflammatory and Lipid Genes	Plos One
Atopic Eczema	NDST1	Expression profiling by array	GPL7191	GSE12511	Global Expression Profiling in Atopic Eczema Reveals Reciprocal Expression of Inflammatory and Lipid Genes	Plos One
Atopic Eczema	TIMP2	Expression profiling by array	GPL7191	GSE12511	Global Expression Profiling in Atopic Eczema Reveals Reciprocal Expression of Inflammatory and Lipid Genes	Plos One
Atopic Eczema	PROS1	Expression profiling by array	GPL7191	GSE12511	Global Expression Profiling in Atopic Eczema Reveals Reciprocal Expression of Inflammatory and Lipid Genes	Plos One
Atopic Eczema	FAM89A	Expression profiling by array	GPL7191	GSE12511	Global Expression Profiling in Atopic Eczema Reveals Reciprocal Expression of Inflammatory and Lipid Genes	Plos One
Atopic Eczema	IDE	Expression profiling by array	GPL7191	GSE12511	Global Expression Profiling in Atopic Eczema Reveals Reciprocal Expression of Inflammatory and Lipid Genes	Plos One
Atopic Eczema	CORO1A	Expression profiling by array	GPL7191	GSE12511	Global Expression Profiling in Atopic Eczema Reveals Reciprocal Expression of Inflammatory and Lipid Genes	Plos One
Atopic Eczema	RNASE6	Expression profiling by array	GPL7191	GSE12511	Global Expression Profiling in Atopic Eczema Reveals Reciprocal Expression of Inflammatory and Lipid Genes	Plos One
Atopic Eczema	CASP5	Expression profiling by array	GPL7191	GSE12511	Global Expression Profiling in Atopic Eczema Reveals Reciprocal Expression of Inflammatory and Lipid Genes	Plos One
Atopic Eczema	FBN1	Expression profiling by array	GPL7191	GSE12511	Global Expression Profiling in Atopic Eczema Reveals Reciprocal Expression of Inflammatory and Lipid Genes	Plos One
Atopic Eczema	CECR1	Expression profiling by array	GPL7191	GSE12511	Global Expression Profiling in Atopic Eczema Reveals Reciprocal Expression of Inflammatory and Lipid Genes	Plos One
Atopic Eczema	PSMB8	Expression profiling by array	GPL7191	GSE12511	Global Expression Profiling in Atopic Eczema Reveals Reciprocal Expression of Inflammatory and Lipid Genes	Plos One
Atopic Eczema	DAB2	Expression profiling by array	GPL7191	GSE12511	Global Expression Profiling in Atopic Eczema Reveals Reciprocal Expression of Inflammatory and Lipid Genes	Plos One
Atopic Eczema	THBD	Expression profiling by array	GPL7191	GSE12511	Global Expression Profiling in Atopic Eczema Reveals Reciprocal Expression of Inflammatory and Lipid Genes	Plos One
Atopic Eczema	LOX	Expression profiling by array	GPL7191	GSE12511	Global Expression Profiling in Atopic Eczema Reveals Reciprocal Expression of Inflammatory and Lipid Genes	Plos One
Atopic Eczema	SOX9	Expression profiling by array	GPL7191	GSE12511	Global Expression Profiling in Atopic Eczema Reveals Reciprocal Expression of Inflammatory and Lipid Genes	Plos One
Atopic Eczema	IL10RA	Expression profiling by array	GPL7191	GSE12511	Global Expression Profiling in Atopic Eczema Reveals Reciprocal Expression of Inflammatory and Lipid Genes	Plos One
Atopic Eczema	DNER	Expression profiling by array	GPL7191	GSE12511	Global Expression Profiling in Atopic Eczema Reveals Reciprocal Expression of Inflammatory and Lipid Genes	Plos One
Atopic Eczema	KIAA1324	Expression profiling by array	GPL7191	GSE12511	Global Expression Profiling in Atopic Eczema Reveals Reciprocal Expression of Inflammatory and Lipid Genes	Plos One
Atopic Eczema	ITGB2	Expression profiling by array	GPL7191	GSE12511	Global Expression Profiling in Atopic Eczema Reveals Reciprocal Expression of Inflammatory and Lipid Genes	Plos One
Atopic Eczema	IFITM1	Expression profiling by array	GPL7191	GSE12511	Global Expression Profiling in Atopic Eczema Reveals Reciprocal Expression of Inflammatory and Lipid Genes	Plos One
Atopic Eczema	TGM1	Expression profiling by array	GPL7191	GSE12511	Global Expression Profiling in Atopic Eczema Reveals Reciprocal Expression of Inflammatory and Lipid Genes	Plos One
Atopic Eczema	LOC642851	Expression profiling by array	GPL7191	GSE12511	Global Expression Profiling in Atopic Eczema Reveals Reciprocal Expression of Inflammatory and Lipid Genes	Plos One
Atopic Eczema	PMP22	Expression profiling by array	GPL7191	GSE12511	Global Expression Profiling in Atopic Eczema Reveals Reciprocal Expression of Inflammatory and Lipid Genes	Plos One
Atopic Eczema	IL4R	Expression profiling by array	GPL7191	GSE12511	Global Expression Profiling in Atopic Eczema Reveals Reciprocal Expression of Inflammatory and Lipid Genes	Plos One
Atopic Eczema	SLC12A2	Expression profiling by array	GPL7191	GSE12511	Global Expression Profiling in Atopic Eczema Reveals Reciprocal Expression of Inflammatory and Lipid Genes	Plos One
Atopic Eczema	ARRB2	Expression profiling by array	GPL7191	GSE12511	Global Expression Profiling in Atopic Eczema Reveals Reciprocal Expression of Inflammatory and Lipid Genes	Plos One
Atopic Eczema	PPARGC1A	Expression profiling by array	GPL7191	GSE12511	Global Expression Profiling in Atopic Eczema Reveals Reciprocal Expression of Inflammatory and Lipid Genes	Plos One
Atopic Eczema	STK10	Expression profiling by array	GPL7191	GSE12511	Global Expression Profiling in Atopic Eczema Reveals Reciprocal Expression of Inflammatory and Lipid Genes	Plos One
Atopic Eczema	ZMAT1	Expression profiling by array	GPL7191	GSE12511	Global Expression Profiling in Atopic Eczema Reveals Reciprocal Expression of Inflammatory and Lipid Genes	Plos One
Atopic Eczema	GPR109A	Expression profiling by array	GPL7191	GSE12511	Global Expression Profiling in Atopic Eczema Reveals Reciprocal Expression of Inflammatory and Lipid Genes	Plos One
Atopic Eczema	HAO2	Expression profiling by array	GPL7191	GSE12511	Global Expression Profiling in Atopic Eczema Reveals Reciprocal Expression of Inflammatory and Lipid Genes	Plos One
Atopic Eczema	RPRC1	Expression profiling by array	GPL7191	GSE12511	Global Expression Profiling in Atopic Eczema Reveals Reciprocal Expression of Inflammatory and Lipid Genes	Plos One
Atopic Eczema	RAB31	Expression profiling by array	GPL7191	GSE12511	Global Expression Profiling in Atopic Eczema Reveals Reciprocal Expression of Inflammatory and Lipid Genes	Plos One
Atopic Eczema	MAPK8	Expression profiling by array	GPL7191	GSE12511	Global Expression Profiling in Atopic Eczema Reveals Reciprocal Expression of Inflammatory and Lipid Genes	Plos One
Atopic Eczema	IFITM2	Expression profiling by array	GPL7191	GSE12511	Global Expression Profiling in Atopic Eczema Reveals Reciprocal Expression of Inflammatory and Lipid Genes	Plos One
Atopic Eczema	SERPINB2	Expression profiling by array	GPL7191	GSE12511	Global Expression Profiling in Atopic Eczema Reveals Reciprocal Expression of Inflammatory and Lipid Genes	Plos One
Atopic Eczema	APRT	Expression profiling by array	GPL7191	GSE12511	Global Expression Profiling in Atopic Eczema Reveals Reciprocal Expression of Inflammatory and Lipid Genes	Plos One
Atopic Eczema	KIAA1949	Expression profiling by array	GPL7191	GSE12511	Global Expression Profiling in Atopic Eczema Reveals Reciprocal Expression of Inflammatory and Lipid Genes	Plos One
Atopic Eczema	TIMP1	Expression profiling by array	GPL7191	GSE12511	Global Expression Profiling in Atopic Eczema Reveals Reciprocal Expression of Inflammatory and Lipid Genes	Plos One
Atopic Eczema	SOX8	Expression profiling by array	GPL7191	GSE12511	Global Expression Profiling in Atopic Eczema Reveals Reciprocal Expression of Inflammatory and Lipid Genes	Plos One
Atopic Eczema	THY1	Expression profiling by array	GPL7191	GSE12511	Global Expression Profiling in Atopic Eczema Reveals Reciprocal Expression of Inflammatory and Lipid Genes	Plos One
Atopic Eczema	FYN	Expression profiling by array	GPL7191	GSE12511	Global Expression Profiling in Atopic Eczema Reveals Reciprocal Expression of Inflammatory and Lipid Genes	Plos One
Atopic Eczema	SERPING1	Expression profiling by array	GPL7191	GSE12511	Global Expression Profiling in Atopic Eczema Reveals Reciprocal Expression of Inflammatory and Lipid Genes	Plos One
Atopic Eczema	MYO5C	Expression profiling by array	GPL7191	GSE12511	Global Expression Profiling in Atopic Eczema Reveals Reciprocal Expression of Inflammatory and Lipid Genes	Plos One

Atopic Eczema	ITGA4	Expression profiling by array	GPL7191	GSE12511	Global Expression Profiling in Atopic Eczema Reveals Reciprocal Expression of Inflammatory and Lipid Genes	Plos One
Atopic Eczema	KLF4	Expression profiling by array	GPL7191	GSE12511	Global Expression Profiling in Atopic Eczema Reveals Reciprocal Expression of Inflammatory and Lipid Genes	Plos One
Atopic Eczema	LYZ	Expression profiling by array	GPL7191	GSE12511	Global Expression Profiling in Atopic Eczema Reveals Reciprocal Expression of Inflammatory and Lipid Genes	Plos One
Atopic Eczema	BST2	Expression profiling by array	GPL7191	GSE12511	Global Expression Profiling in Atopic Eczema Reveals Reciprocal Expression of Inflammatory and Lipid Genes	Plos One
Atopic Eczema	LOC645638	Expression profiling by array	GPL7191	GSE12511	Global Expression Profiling in Atopic Eczema Reveals Reciprocal Expression of Inflammatory and Lipid Genes	Plos One
Atopic Eczema	PDGFRL	Expression profiling by array	GPL7191	GSE12511	Global Expression Profiling in Atopic Eczema Reveals Reciprocal Expression of Inflammatory and Lipid Genes	Plos One
Atopic Eczema	LOC257407	Expression profiling by array	GPL7191	GSE12511	Global Expression Profiling in Atopic Eczema Reveals Reciprocal Expression of Inflammatory and Lipid Genes	Plos One
Atopic Eczema	HLA-F	Expression profiling by array	GPL7191	GSE12511	Global Expression Profiling in Atopic Eczema Reveals Reciprocal Expression of Inflammatory and Lipid Genes	Plos One
Atopic Eczema	PLEKHQ1	Expression profiling by array	GPL7191	GSE12511	Global Expression Profiling in Atopic Eczema Reveals Reciprocal Expression of Inflammatory and Lipid Genes	Plos One
Atopic Eczema	TMEM56	Expression profiling by array	GPL7191	GSE12511	Global Expression Profiling in Atopic Eczema Reveals Reciprocal Expression of Inflammatory and Lipid Genes	Plos One
Atopic Eczema	PLA2R1	Expression profiling by array	GPL7191	GSE12511	Global Expression Profiling in Atopic Eczema Reveals Reciprocal Expression of Inflammatory and Lipid Genes	Plos One
Atopic Eczema	CD44	Expression profiling by array	GPL7191	GSE12511	Global Expression Profiling in Atopic Eczema Reveals Reciprocal Expression of Inflammatory and Lipid Genes	Plos One
Atopic Eczema	RAB32	Expression profiling by array	GPL7191	GSE12511	Global Expression Profiling in Atopic Eczema Reveals Reciprocal Expression of Inflammatory and Lipid Genes	Plos One
Atopic Eczema	HUNK	Expression profiling by array	GPL7191	GSE12511	Global Expression Profiling in Atopic Eczema Reveals Reciprocal Expression of Inflammatory and Lipid Genes	Plos One
Atopic Eczema	SEPT11	Expression profiling by array	GPL7191	GSE12511	Global Expression Profiling in Atopic Eczema Reveals Reciprocal Expression of Inflammatory and Lipid Genes	Plos One
Atopic Eczema	DPH1	Expression profiling by array	GPL7191	GSE12511	Global Expression Profiling in Atopic Eczema Reveals Reciprocal Expression of Inflammatory and Lipid Genes	Plos One
Atopic Eczema	ZAP70	Expression profiling by array	GPL7191	GSE12511	Global Expression Profiling in Atopic Eczema Reveals Reciprocal Expression of Inflammatory and Lipid Genes	Plos One
Atopic Eczema	LOC340061	Expression profiling by array	GPL7191	GSE12511	Global Expression Profiling in Atopic Eczema Reveals Reciprocal Expression of Inflammatory and Lipid Genes	Plos One
Atopic Eczema	NCF1	Expression profiling by array	GPL7191	GSE12511	Global Expression Profiling in Atopic Eczema Reveals Reciprocal Expression of Inflammatory and Lipid Genes	Plos One
Atopic Eczema	PLCB4	Expression profiling by array	GPL7191	GSE12511	Global Expression Profiling in Atopic Eczema Reveals Reciprocal Expression of Inflammatory and Lipid Genes	Plos One
Atopic Eczema	SMPD2	Expression profiling by array	GPL7191	GSE12511	Global Expression Profiling in Atopic Eczema Reveals Reciprocal Expression of Inflammatory and Lipid Genes	Plos One
Atopic Eczema	APBB1IP	Expression profiling by array	GPL7191	GSE12511	Global Expression Profiling in Atopic Eczema Reveals Reciprocal Expression of Inflammatory and Lipid Genes	Plos One
Atopic Eczema	ARPC1B	Expression profiling by array	GPL7191	GSE12511	Global Expression Profiling in Atopic Eczema Reveals Reciprocal Expression of Inflammatory and Lipid Genes	Plos One
Atopic Eczema	CD52	Expression profiling by array	GPL7191	GSE12511	Global Expression Profiling in Atopic Eczema Reveals Reciprocal Expression of Inflammatory and Lipid Genes	Plos One
Atopic Eczema	MMP2	Expression profiling by array	GPL7191	GSE12511	Global Expression Profiling in Atopic Eczema Reveals Reciprocal Expression of Inflammatory and Lipid Genes	Plos One
Atopic Eczema	SOCS3	Expression profiling by array	GPL7191	GSE12511	Global Expression Profiling in Atopic Eczema Reveals Reciprocal Expression of Inflammatory and Lipid Genes	Plos One
Atopic Eczema	ZNFX1	Expression profiling by array	GPL7191	GSE12511	Global Expression Profiling in Atopic Eczema Reveals Reciprocal Expression of Inflammatory and Lipid Genes	Plos One
Atopic Eczema	TNIP2	Expression profiling by array	GPL7191	GSE12511	Global Expression Profiling in Atopic Eczema Reveals Reciprocal Expression of Inflammatory and Lipid Genes	Plos One
Atopic Eczema	MYST2	Expression profiling by array	GPL7191	GSE12511	Global Expression Profiling in Atopic Eczema Reveals Reciprocal Expression of Inflammatory and Lipid Genes	Plos One
Atopic Eczema	CLEC10A	Expression profiling by array	GPL7191	GSE12511	Global Expression Profiling in Atopic Eczema Reveals Reciprocal Expression of Inflammatory and Lipid Genes	Plos One
Atopic Eczema	COX7B	Expression profiling by array	GPL7191	GSE12511	Global Expression Profiling in Atopic Eczema Reveals Reciprocal Expression of Inflammatory and Lipid Genes	Plos One
Atopic Eczema	GNB4	Expression profiling by array	GPL7191	GSE12511	Global Expression Profiling in Atopic Eczema Reveals Reciprocal Expression of Inflammatory and Lipid Genes	Plos One
Atopic Eczema	HIRIP3	Expression profiling by array	GPL7191	GSE12511	Global Expression Profiling in Atopic Eczema Reveals Reciprocal Expression of Inflammatory and Lipid Genes	Plos One
Atopic Eczema	EMB	Expression profiling by array	GPL7191	GSE12511	Global Expression Profiling in Atopic Eczema Reveals Reciprocal Expression of Inflammatory and Lipid Genes	Plos One
Atopic Eczema	MAP4	Expression profiling by array	GPL7191	GSE12511	Global Expression Profiling in Atopic Eczema Reveals Reciprocal Expression of Inflammatory and Lipid Genes	Plos One
Atopic Eczema	FADS1	Expression profiling by array	GPL7191	GSE12511	Global Expression Profiling in Atopic Eczema Reveals Reciprocal Expression of Inflammatory and Lipid Genes	Plos One
Atopic Eczema	FOXC1	Expression profiling by array	GPL7191	GSE12511	Global Expression Profiling in Atopic Eczema Reveals Reciprocal Expression of Inflammatory and Lipid Genes	Plos One
Atopic Eczema	GMFG	Expression profiling by array	GPL7191	GSE12511	Global Expression Profiling in Atopic Eczema Reveals Reciprocal Expression of Inflammatory and Lipid Genes	Plos One
Atopic Eczema	LOC653198	Expression profiling by array	GPL7191	GSE12511	Global Expression Profiling in Atopic Eczema Reveals Reciprocal Expression of Inflammatory and Lipid Genes	Plos One
Atopic Eczema	CFHR1	Expression profiling by array	GPL7191	GSE12511	Global Expression Profiling in Atopic Eczema Reveals Reciprocal Expression of Inflammatory and Lipid Genes	Plos One
Atopic Eczema	FAM84B	Expression profiling by array	GPL7191	GSE12511	Global Expression Profiling in Atopic Eczema Reveals Reciprocal Expression of Inflammatory and Lipid Genes	Plos One
Atopic Eczema	CD99	Expression profiling by array	GPL7191	GSE12511	Global Expression Profiling in Atopic Eczema Reveals Reciprocal Expression of Inflammatory and Lipid Genes	Plos One
Atopic Eczema	PLCL2	Expression profiling by array	GPL7191	GSE12511	Global Expression Profiling in Atopic Eczema Reveals Reciprocal Expression of Inflammatory and Lipid Genes	Plos One
Atopic Eczema	HLA-DRB1	Expression profiling by array	GPL7191	GSE12511	Global Expression Profiling in Atopic Eczema Reveals Reciprocal Expression of Inflammatory and Lipid Genes	Plos One
Atopic Eczema	OAS3	Expression profiling by array	GPL7191	GSE12511	Global Expression Profiling in Atopic Eczema Reveals Reciprocal Expression of Inflammatory and Lipid Genes	Plos One
Atopic Eczema	SELPLG	Expression profiling by array	GPL7191	GSE12511	Global Expression Profiling in Atopic Eczema Reveals Reciprocal Expression of Inflammatory and Lipid Genes	Plos One
Atopic Eczema	JUNB	Expression profiling by array	GPL7191	GSE12511	Global Expression Profiling in Atopic Eczema Reveals Reciprocal Expression of Inflammatory and Lipid Genes	Plos One
Atopic Eczema	TUBB	Expression profiling by array	GPL7191	GSE12511	Global Expression Profiling in Atopic Eczema Reveals Reciprocal Expression of Inflammatory and Lipid Genes	Plos One
Atopic Eczema	C7	Expression profiling by array	GPL7191	GSE12511	Global Expression Profiling in Atopic Eczema Reveals Reciprocal Expression of Inflammatory and Lipid Genes	Plos One
Atopic Eczema	FOXA1	Expression profiling by array	GPL7191	GSE12511	Global Expression Profiling in Atopic Eczema Reveals Reciprocal Expression of Inflammatory and Lipid Genes	Plos One
Atopic Eczema	KCNAB2	Expression profiling by array	GPL7191	GSE12511	Global Expression Profiling in Atopic Eczema Reveals Reciprocal Expression of Inflammatory and Lipid Genes	Plos One
Atopic Eczema	ACACA	Expression profiling by array	GPL7191	GSE12511	Global Expression Profiling in Atopic Eczema Reveals Reciprocal Expression of Inflammatory and Lipid Genes	Plos One
Atopic Eczema	PER3	Expression profiling by array	GPL7191	GSE12511	Global Expression Profiling in Atopic Eczema Reveals Reciprocal Expression of Inflammatory and Lipid Genes	Plos One
Atopic Eczema	RAC2	Expression profiling by array	GPL7191	GSE12511	Global Expression Profiling in Atopic Eczema Reveals Reciprocal Expression of Inflammatory and Lipid Genes	Plos One
Atopic Eczema	SCARA3	Expression profiling by array	GPL7191	GSE12511	Global Expression Profiling in Atopic Eczema Reveals Reciprocal Expression of Inflammatory and Lipid Genes	Plos One
Atopic Eczema	POSTN	Expression profiling by array	GPL7191	GSE12511	Global Expression Profiling in Atopic Eczema Reveals Reciprocal Expression of Inflammatory and Lipid Genes	Plos One
Atopic Eczema	LCK	Expression profiling by array	GPL7191	GSE12511	Global Expression Profiling in Atopic Eczema Reveals Reciprocal Expression of Inflammatory and Lipid Genes	Plos One
Atopic Eczema	PCCB	Expression profiling by array	GPL7191	GSE12511	Global Expression Profiling in Atopic Eczema Reveals Reciprocal Expression of Inflammatory and Lipid Genes	Plos One
Atopic Eczema	THAP11	Expression profiling by array	GPL7191	GSE12511	Global Expression Profiling in Atopic Eczema Reveals Reciprocal Expression of Inflammatory and Lipid Genes	Plos One
Atopic Eczema	MYADM	Expression profiling by array	GPL7191	GSE12511	Global Expression Profiling in Atopic Eczema Reveals Reciprocal Expression of Inflammatory and Lipid Genes	Plos One
Atopic Eczema	CD5	Expression profiling by array	GPL7191	GSE12511	Global Expression Profiling in Atopic Eczema Reveals Reciprocal Expression of Inflammatory and Lipid Genes	Plos One
Atopic Eczema	TAPBP	Expression profiling by array	GPL7191	GSE12511	Global Expression Profiling in Atopic Eczema Reveals Reciprocal Expression of Inflammatory and Lipid Genes	Plos One

Atopic Eczema	DOCK2	Expression profiling by array	GPL7191	GSE12511	Global Expression Profiling in Atopic Eczema Reveals Reciprocal Expression of Inflammatory and Lipid Genes	Plos One
Atopic Eczema	PPP1R1B	Expression profiling by array	GPL7191	GSE12511	Global Expression Profiling in Atopic Eczema Reveals Reciprocal Expression of Inflammatory and Lipid Genes	Plos One
Atopic Eczema	UBE2L6	Expression profiling by array	GPL7191	GSE12511	Global Expression Profiling in Atopic Eczema Reveals Reciprocal Expression of Inflammatory and Lipid Genes	Plos One
Atopic Eczema	WDTC1	Expression profiling by array	GPL7191	GSE12511	Global Expression Profiling in Atopic Eczema Reveals Reciprocal Expression of Inflammatory and Lipid Genes	Plos One
Atopic Eczema	SH3BGRL3	Expression profiling by array	GPL7191	GSE12511	Global Expression Profiling in Atopic Eczema Reveals Reciprocal Expression of Inflammatory and Lipid Genes	Plos One
Atopic Eczema	SEMG1	Expression profiling by array	GPL7191	GSE12511	Global Expression Profiling in Atopic Eczema Reveals Reciprocal Expression of Inflammatory and Lipid Genes	Plos One
Atopic Eczema	VTCN1	Expression profiling by array	GPL7191	GSE12511	Global Expression Profiling in Atopic Eczema Reveals Reciprocal Expression of Inflammatory and Lipid Genes	Plos One
Atopic Eczema	TBX3	Expression profiling by array	GPL7191	GSE12511	Global Expression Profiling in Atopic Eczema Reveals Reciprocal Expression of Inflammatory and Lipid Genes	Plos One
Atopic Eczema	ELMOD1	Expression profiling by array	GPL7191	GSE12511	Global Expression Profiling in Atopic Eczema Reveals Reciprocal Expression of Inflammatory and Lipid Genes	Plos One
Atopic Eczema	DEFB1	Expression profiling by array	GPL7191	GSE12511	Global Expression Profiling in Atopic Eczema Reveals Reciprocal Expression of Inflammatory and Lipid Genes	Plos One
Atopic Eczema	ME1	Expression profiling by array	GPL7191	GSE12511	Global Expression Profiling in Atopic Eczema Reveals Reciprocal Expression of Inflammatory and Lipid Genes	Plos One
Atopic Eczema	CD59	Expression profiling by array	GPL7191	GSE12511	Global Expression Profiling in Atopic Eczema Reveals Reciprocal Expression of Inflammatory and Lipid Genes	Plos One
Atopic Eczema	C10ORF26	Expression profiling by array	GPL7191	GSE12511	Global Expression Profiling in Atopic Eczema Reveals Reciprocal Expression of Inflammatory and Lipid Genes	Plos One
Atopic Eczema	RHOF	Expression profiling by array	GPL7191	GSE12511	Global Expression Profiling in Atopic Eczema Reveals Reciprocal Expression of Inflammatory and Lipid Genes	Plos One
Atopic Eczema	NCALD	Expression profiling by array	GPL7191	GSE12511	Global Expression Profiling in Atopic Eczema Reveals Reciprocal Expression of Inflammatory and Lipid Genes	Plos One
Atopic Eczema	ZNF187	Expression profiling by array	GPL7191	GSE12511	Global Expression Profiling in Atopic Eczema Reveals Reciprocal Expression of Inflammatory and Lipid Genes	Plos One
Atopic Eczema	MGST1	Expression profiling by array	GPL7191	GSE12511	Global Expression Profiling in Atopic Eczema Reveals Reciprocal Expression of Inflammatory and Lipid Genes	Plos One
Atopic Eczema	C1ORF38	Expression profiling by array	GPL7191	GSE12511	Global Expression Profiling in Atopic Eczema Reveals Reciprocal Expression of Inflammatory and Lipid Genes	Plos One
Atopic Eczema	PIP	Expression profiling by array	GPL7191	GSE12511	Global Expression Profiling in Atopic Eczema Reveals Reciprocal Expression of Inflammatory and Lipid Genes	Plos One
Atopic Eczema	CD34	Expression profiling by array	GPL7191	GSE12511	Global Expression Profiling in Atopic Eczema Reveals Reciprocal Expression of Inflammatory and Lipid Genes	Plos One
Atopic Eczema	GAS1	Expression profiling by array	GPL7191	GSE12511	Global Expression Profiling in Atopic Eczema Reveals Reciprocal Expression of Inflammatory and Lipid Genes	Plos One
Atopic Eczema	LCP2	Expression profiling by array	GPL7191	GSE12511	Global Expression Profiling in Atopic Eczema Reveals Reciprocal Expression of Inflammatory and Lipid Genes	Plos One
Atopic Eczema	INHBB	Expression profiling by array	GPL7191	GSE12511	Global Expression Profiling in Atopic Eczema Reveals Reciprocal Expression of Inflammatory and Lipid Genes	Plos One
Atopic Eczema	HLA-E	Expression profiling by array	GPL7191	GSE12511	Global Expression Profiling in Atopic Eczema Reveals Reciprocal Expression of Inflammatory and Lipid Genes	Plos One
Atopic Eczema	FBLN1	Expression profiling by array	GPL7191	GSE12511	Global Expression Profiling in Atopic Eczema Reveals Reciprocal Expression of Inflammatory and Lipid Genes	Plos One
Atopic Eczema	AI091487	Expression profiling by array	GPL7191	GSE12511	Global Expression Profiling in Atopic Eczema Reveals Reciprocal Expression of Inflammatory and Lipid Genes	Plos One
Atopic Eczema	PYHIN1	Expression profiling by array	GPL7191	GSE12511	Global Expression Profiling in Atopic Eczema Reveals Reciprocal Expression of Inflammatory and Lipid Genes	Plos One
Atopic Eczema	KLK8	Expression profiling by array	GPL7191	GSE12511	Global Expression Profiling in Atopic Eczema Reveals Reciprocal Expression of Inflammatory and Lipid Genes	Plos One
Atopic Eczema	TUBA1	Expression profiling by array	GPL7191	GSE12511	Global Expression Profiling in Atopic Eczema Reveals Reciprocal Expression of Inflammatory and Lipid Genes	Plos One
Atopic Eczema	PEA15	Expression profiling by array	GPL7191	GSE12511	Global Expression Profiling in Atopic Eczema Reveals Reciprocal Expression of Inflammatory and Lipid Genes	Plos One
Atopic Eczema	NDRG1	Expression profiling by array	GPL7191	GSE12511	Global Expression Profiling in Atopic Eczema Reveals Reciprocal Expression of Inflammatory and Lipid Genes	Plos One
Atopic Eczema	FLJ25084	Expression profiling by array	GPL7191	GSE12511	Global Expression Profiling in Atopic Eczema Reveals Reciprocal Expression of Inflammatory and Lipid Genes	Plos One
Atopic Eczema	KRT17	Expression profiling by array	GPL7191	GSE12511	Global Expression Profiling in Atopic Eczema Reveals Reciprocal Expression of Inflammatory and Lipid Genes	Plos One
Atopic Eczema	S100A10	Expression profiling by array	GPL7191	GSE12511	Global Expression Profiling in Atopic Eczema Reveals Reciprocal Expression of Inflammatory and Lipid Genes	Plos One
Atopic Eczema	CTSC	Expression profiling by array	GPL7191	GSE12511	Global Expression Profiling in Atopic Eczema Reveals Reciprocal Expression of Inflammatory and Lipid Genes	Plos One
Atopic Eczema	GRN	Expression profiling by array	GPL7191	GSE12511	Global Expression Profiling in Atopic Eczema Reveals Reciprocal Expression of Inflammatory and Lipid Genes	Plos One
Atopic Eczema	VAV1	Expression profiling by array	GPL7191	GSE12511	Global Expression Profiling in Atopic Eczema Reveals Reciprocal Expression of Inflammatory and Lipid Genes	Plos One
Atopic Eczema	ERBB3	Expression profiling by array	GPL7191	GSE12511	Global Expression Profiling in Atopic Eczema Reveals Reciprocal Expression of Inflammatory and Lipid Genes	Plos One
Atopic Eczema	TRIP6	Expression profiling by array	GPL7191	GSE12511	Global Expression Profiling in Atopic Eczema Reveals Reciprocal Expression of Inflammatory and Lipid Genes	Plos One
Atopic Eczema	TRIM55	Expression profiling by array	GPL7191	GSE12511	Global Expression Profiling in Atopic Eczema Reveals Reciprocal Expression of Inflammatory and Lipid Genes	Plos One
Atopic Eczema	ADAM19	Expression profiling by array	GPL7191	GSE12511	Global Expression Profiling in Atopic Eczema Reveals Reciprocal Expression of Inflammatory and Lipid Genes	Plos One
Atopic Eczema	CSNK2A2	Expression profiling by array	GPL7191	GSE12511	Global Expression Profiling in Atopic Eczema Reveals Reciprocal Expression of Inflammatory and Lipid Genes	Plos One
Atopic Eczema	NCF2	Expression profiling by array	GPL7191	GSE12511	Global Expression Profiling in Atopic Eczema Reveals Reciprocal Expression of Inflammatory and Lipid Genes	Plos One
Atopic Eczema	GREM1	Expression profiling by array	GPL7191	GSE12511	Global Expression Profiling in Atopic Eczema Reveals Reciprocal Expression of Inflammatory and Lipid Genes	Plos One
Atopic Eczema	CCR5	Expression profiling by array	GPL7191	GSE12511	Global Expression Profiling in Atopic Eczema Reveals Reciprocal Expression of Inflammatory and Lipid Genes	Plos One
Atopic Eczema	ZNF511	Expression profiling by array	GPL7191	GSE12511	Global Expression Profiling in Atopic Eczema Reveals Reciprocal Expression of Inflammatory and Lipid Genes	Plos One
Atopic Eczema	PLEKHB1	Expression profiling by array	GPL7191	GSE12511	Global Expression Profiling in Atopic Eczema Reveals Reciprocal Expression of Inflammatory and Lipid Genes	Plos One
Atopic Eczema	MLC1	Expression profiling by array	GPL7191	GSE12511	Global Expression Profiling in Atopic Eczema Reveals Reciprocal Expression of Inflammatory and Lipid Genes	Plos One
Atopic Eczema	SHRM	Expression profiling by array	GPL7191	GSE12511	Global Expression Profiling in Atopic Eczema Reveals Reciprocal Expression of Inflammatory and Lipid Genes	Plos One
Atopic Eczema	AI276654	Expression profiling by array	GPL7191	GSE12511	Global Expression Profiling in Atopic Eczema Reveals Reciprocal Expression of Inflammatory and Lipid Genes	Plos One
Atopic Eczema	SYT11	Expression profiling by array	GPL7191	GSE12511	Global Expression Profiling in Atopic Eczema Reveals Reciprocal Expression of Inflammatory and Lipid Genes	Plos One
Atopic Eczema	KRTAP10-11	Expression profiling by array	GPL7191	GSE12511	Global Expression Profiling in Atopic Eczema Reveals Reciprocal Expression of Inflammatory and Lipid Genes	Plos One
Atopic Eczema	SLC30A10	Expression profiling by array	GPL7191	GSE12511	Global Expression Profiling in Atopic Eczema Reveals Reciprocal Expression of Inflammatory and Lipid Genes	Plos One
Atopic Eczema	CLDN8	Expression profiling by array	GPL7191	GSE12511	Global Expression Profiling in Atopic Eczema Reveals Reciprocal Expression of Inflammatory and Lipid Genes	Plos One
Atopic Eczema	FLJ21127	Expression profiling by array	GPL7191	GSE12511	Global Expression Profiling in Atopic Eczema Reveals Reciprocal Expression of Inflammatory and Lipid Genes	Plos One
Atopic Eczema	BASP1	Expression profiling by array	GPL7191	GSE12511	Global Expression Profiling in Atopic Eczema Reveals Reciprocal Expression of Inflammatory and Lipid Genes	Plos One
Atopic Eczema	MLPH	Expression profiling by array	GPL7191	GSE12511	Global Expression Profiling in Atopic Eczema Reveals Reciprocal Expression of Inflammatory and Lipid Genes	Plos One
Atopic Eczema	AQP3	Expression profiling by array	GPL7191	GSE12511	Global Expression Profiling in Atopic Eczema Reveals Reciprocal Expression of Inflammatory and Lipid Genes	Plos One
Atopic Eczema	SCGB2A1	Expression profiling by array	GPL7191	GSE12511	Global Expression Profiling in Atopic Eczema Reveals Reciprocal Expression of Inflammatory and Lipid Genes	Plos One
Atopic Eczema	HPSE	Expression profiling by array	GPL7191	GSE12511	Global Expression Profiling in Atopic Eczema Reveals Reciprocal Expression of Inflammatory and Lipid Genes	Plos One
Atopic Eczema	ZBTB5	Expression profiling by array	GPL7191	GSE12511	Global Expression Profiling in Atopic Eczema Reveals Reciprocal Expression of Inflammatory and Lipid Genes	Plos One
Atopic Eczema	C1ORF34	Expression profiling by array	GPL7191	GSE12511	Global Expression Profiling in Atopic Eczema Reveals Reciprocal Expression of Inflammatory and Lipid Genes	Plos One

Atopic Eczema	KCNH2	Expression profiling by array	GPL7191	GSE12511	Global Expression Profiling in Atopic Eczema Reveals Reciprocal Expression of Inflammatory and Lipid Genes	Plos One
Atopic Eczema	FADS2	Expression profiling by array	GPL7191	GSE12511	Global Expression Profiling in Atopic Eczema Reveals Reciprocal Expression of Inflammatory and Lipid Genes	Plos One
Atopic Eczema	TUBB3	Expression profiling by array	GPL7191	GSE12511	Global Expression Profiling in Atopic Eczema Reveals Reciprocal Expression of Inflammatory and Lipid Genes	Plos One
Atopic Eczema	ABHD5	Expression profiling by array	GPL7191	GSE12511	Global Expression Profiling in Atopic Eczema Reveals Reciprocal Expression of Inflammatory and Lipid Genes	Plos One
Atopic Eczema	AI338101	Expression profiling by array	GPL7191	GSE12511	Global Expression Profiling in Atopic Eczema Reveals Reciprocal Expression of Inflammatory and Lipid Genes	Plos One
Atopic Eczema	ZNF506	Expression profiling by array	GPL7191	GSE12511	Global Expression Profiling in Atopic Eczema Reveals Reciprocal Expression of Inflammatory and Lipid Genes	Plos One
Atopic Eczema	SORBS2	Expression profiling by array	GPL7191	GSE12511	Global Expression Profiling in Atopic Eczema Reveals Reciprocal Expression of Inflammatory and Lipid Genes	Plos One
Atopic Eczema	ACSS2	Expression profiling by array	GPL7191	GSE12511	Global Expression Profiling in Atopic Eczema Reveals Reciprocal Expression of Inflammatory and Lipid Genes	Plos One
Atopic Eczema	ZNF204	Expression profiling by array	GPL7191	GSE12511	Global Expression Profiling in Atopic Eczema Reveals Reciprocal Expression of Inflammatory and Lipid Genes	Plos One
Atopic Eczema	MRAS	Expression profiling by array	GPL7191	GSE12511	Global Expression Profiling in Atopic Eczema Reveals Reciprocal Expression of Inflammatory and Lipid Genes	Plos One
Atopic Eczema	CKAP1	Expression profiling by array	GPL7191	GSE12511	Global Expression Profiling in Atopic Eczema Reveals Reciprocal Expression of Inflammatory and Lipid Genes	Plos One
Atopic Eczema	ZNF592	Expression profiling by array	GPL7191	GSE12511	Global Expression Profiling in Atopic Eczema Reveals Reciprocal Expression of Inflammatory and Lipid Genes	Plos One
Atopic Eczema	LOC442609	Expression profiling by array	GPL7191	GSE12511	Global Expression Profiling in Atopic Eczema Reveals Reciprocal Expression of Inflammatory and Lipid Genes	Plos One
Atopic Eczema	C1ORF115	Expression profiling by array	GPL7191	GSE12511	Global Expression Profiling in Atopic Eczema Reveals Reciprocal Expression of Inflammatory and Lipid Genes	Plos One
Atopic Eczema	DDX54	Expression profiling by array	GPL7191	GSE12511	Global Expression Profiling in Atopic Eczema Reveals Reciprocal Expression of Inflammatory and Lipid Genes	Plos One
Atopic Eczema	PLSCR3	Expression profiling by array	GPL7191	GSE12511	Global Expression Profiling in Atopic Eczema Reveals Reciprocal Expression of Inflammatory and Lipid Genes	Plos One
Atopic Eczema	DPP8	Expression profiling by array	GPL7191	GSE12511	Global Expression Profiling in Atopic Eczema Reveals Reciprocal Expression of Inflammatory and Lipid Genes	Plos One
Atopic Eczema	CD3D	Expression profiling by array	GPL7191	GSE12511	Global Expression Profiling in Atopic Eczema Reveals Reciprocal Expression of Inflammatory and Lipid Genes	Plos One
Atopic Eczema	CFC1	Expression profiling by array	GPL7191	GSE12511	Global Expression Profiling in Atopic Eczema Reveals Reciprocal Expression of Inflammatory and Lipid Genes	Plos One
Atopic Eczema	VASN	Expression profiling by array	GPL7191	GSE12511	Global Expression Profiling in Atopic Eczema Reveals Reciprocal Expression of Inflammatory and Lipid Genes	Plos One
Atopic Eczema	SNX19	Expression profiling by array	GPL7191	GSE12511	Global Expression Profiling in Atopic Eczema Reveals Reciprocal Expression of Inflammatory and Lipid Genes	Plos One
Atopic Eczema	EIF5B	Expression profiling by array	GPL7191	GSE12511	Global Expression Profiling in Atopic Eczema Reveals Reciprocal Expression of Inflammatory and Lipid Genes	Plos One
Atopic Eczema	TUBB6	Expression profiling by array	GPL7191	GSE12511	Global Expression Profiling in Atopic Eczema Reveals Reciprocal Expression of Inflammatory and Lipid Genes	Plos One
Atopic Eczema	PRKD1	Expression profiling by array	GPL7191	GSE12511	Global Expression Profiling in Atopic Eczema Reveals Reciprocal Expression of Inflammatory and Lipid Genes	Plos One
Atopic Eczema	VSIG4	Expression profiling by array	GPL7191	GSE12511	Global Expression Profiling in Atopic Eczema Reveals Reciprocal Expression of Inflammatory and Lipid Genes	Plos One
Atopic Eczema	ORF1-FL49	Expression profiling by array	GPL7191	GSE12511	Global Expression Profiling in Atopic Eczema Reveals Reciprocal Expression of Inflammatory and Lipid Genes	Plos One
Atopic Eczema	SLURP1	Expression profiling by array	GPL7191	GSE12511	Global Expression Profiling in Atopic Eczema Reveals Reciprocal Expression of Inflammatory and Lipid Genes	Plos One
Atopic Eczema	SLC43A3	Expression profiling by array	GPL7191	GSE12511	Global Expression Profiling in Atopic Eczema Reveals Reciprocal Expression of Inflammatory and Lipid Genes	Plos One
Atopic Eczema	ST3GAL6	Expression profiling by array	GPL7191	GSE12511	Global Expression Profiling in Atopic Eczema Reveals Reciprocal Expression of Inflammatory and Lipid Genes	Plos One
Atopic Eczema	ACSL1	Expression profiling by array	GPL7191	GSE12511	Global Expression Profiling in Atopic Eczema Reveals Reciprocal Expression of Inflammatory and Lipid Genes	Plos One
Atopic Eczema	TRIM25	Expression profiling by array	GPL7191	GSE12511	Global Expression Profiling in Atopic Eczema Reveals Reciprocal Expression of Inflammatory and Lipid Genes	Plos One
Atopic Eczema	STK39	Expression profiling by array	GPL7191	GSE12511	Global Expression Profiling in Atopic Eczema Reveals Reciprocal Expression of Inflammatory and Lipid Genes	Plos One
Atopic Eczema	FBXW8	Expression profiling by array	GPL7191	GSE12511	Global Expression Profiling in Atopic Eczema Reveals Reciprocal Expression of Inflammatory and Lipid Genes	Plos One
Atopic Eczema	TGM4	Expression profiling by array	GPL7191	GSE12511	Global Expression Profiling in Atopic Eczema Reveals Reciprocal Expression of Inflammatory and Lipid Genes	Plos One
Atopic Eczema	AVEN	Expression profiling by array	GPL7191	GSE12511	Global Expression Profiling in Atopic Eczema Reveals Reciprocal Expression of Inflammatory and Lipid Genes	Plos One
Atopic Eczema	IRF1	Expression profiling by array	GPL7191	GSE12511	Global Expression Profiling in Atopic Eczema Reveals Reciprocal Expression of Inflammatory and Lipid Genes	Plos One
Atopic Eczema	RNH1	Expression profiling by array	GPL7191	GSE12511	Global Expression Profiling in Atopic Eczema Reveals Reciprocal Expression of Inflammatory and Lipid Genes	Plos One
Atopic Eczema	MLSTD1	Expression profiling by array	GPL7191	GSE12511	Global Expression Profiling in Atopic Eczema Reveals Reciprocal Expression of Inflammatory and Lipid Genes	Plos One
Atopic Eczema	MAP3K6	Expression profiling by array	GPL7191	GSE12511	Global Expression Profiling in Atopic Eczema Reveals Reciprocal Expression of Inflammatory and Lipid Genes	Plos One
Atopic Eczema	XKR6	Expression profiling by array	GPL7191	GSE12511	Global Expression Profiling in Atopic Eczema Reveals Reciprocal Expression of Inflammatory and Lipid Genes	Plos One
Atopic Eczema	SDC2	Expression profiling by array	GPL7191	GSE12511	Global Expression Profiling in Atopic Eczema Reveals Reciprocal Expression of Inflammatory and Lipid Genes	Plos One
Atopic Eczema	CAMK1	Expression profiling by array	GPL7191	GSE12511	Global Expression Profiling in Atopic Eczema Reveals Reciprocal Expression of Inflammatory and Lipid Genes	Plos One
Atopic Eczema	PPM1F	Expression profiling by array	GPL7191	GSE12511	Global Expression Profiling in Atopic Eczema Reveals Reciprocal Expression of Inflammatory and Lipid Genes	Plos One
Atopic Eczema	TRAFD1	Expression profiling by array	GPL7191	GSE12511	Global Expression Profiling in Atopic Eczema Reveals Reciprocal Expression of Inflammatory and Lipid Genes	Plos One
Atopic Eczema	FLJ25037	Expression profiling by array	GPL7191	GSE12511	Global Expression Profiling in Atopic Eczema Reveals Reciprocal Expression of Inflammatory and Lipid Genes	Plos One
Atopic Eczema	AI301734	Expression profiling by array	GPL7191	GSE12511	Global Expression Profiling in Atopic Eczema Reveals Reciprocal Expression of Inflammatory and Lipid Genes	Plos One
Atopic Eczema	EXOC3	Expression profiling by array	GPL7191	GSE12511	Global Expression Profiling in Atopic Eczema Reveals Reciprocal Expression of Inflammatory and Lipid Genes	Plos One
Atopic Eczema	GPR176	Expression profiling by array	GPL7191	GSE12511	Global Expression Profiling in Atopic Eczema Reveals Reciprocal Expression of Inflammatory and Lipid Genes	Plos One
Atopic Eczema	AA224758	Expression profiling by array	GPL7191	GSE12511	Global Expression Profiling in Atopic Eczema Reveals Reciprocal Expression of Inflammatory and Lipid Genes	Plos One
Atopic Eczema	MOBKL2A	Expression profiling by array	GPL7191	GSE12511	Global Expression Profiling in Atopic Eczema Reveals Reciprocal Expression of Inflammatory and Lipid Genes	Plos One
Atopic Eczema	AI871056	Expression profiling by array	GPL7191	GSE12511	Global Expression Profiling in Atopic Eczema Reveals Reciprocal Expression of Inflammatory and Lipid Genes	Plos One
Atopic Eczema	SOLH	Expression profiling by array	GPL7191	GSE12511	Global Expression Profiling in Atopic Eczema Reveals Reciprocal Expression of Inflammatory and Lipid Genes	Plos One
Atopic Eczema	MYD88	Expression profiling by array	GPL7191	GSE12511	Global Expression Profiling in Atopic Eczema Reveals Reciprocal Expression of Inflammatory and Lipid Genes	Plos One
Atopic Eczema	ARRB1	Expression profiling by array	GPL7191	GSE12511	Global Expression Profiling in Atopic Eczema Reveals Reciprocal Expression of Inflammatory and Lipid Genes	Plos One
Atopic Eczema	CHMP4C	Expression profiling by array	GPL7191	GSE12511	Global Expression Profiling in Atopic Eczema Reveals Reciprocal Expression of Inflammatory and Lipid Genes	Plos One
Atopic Eczema	FLJ37543	Expression profiling by array	GPL7191	GSE12511	Global Expression Profiling in Atopic Eczema Reveals Reciprocal Expression of Inflammatory and Lipid Genes	Plos One
Atopic Eczema	DUSP14	Expression profiling by array	GPL7191	GSE12511	Global Expression Profiling in Atopic Eczema Reveals Reciprocal Expression of Inflammatory and Lipid Genes	Plos One
Atopic Eczema	NSUN7	Expression profiling by array	GPL7191	GSE12511	Global Expression Profiling in Atopic Eczema Reveals Reciprocal Expression of Inflammatory and Lipid Genes	Plos One
Atopic Eczema	GPSM3	Expression profiling by array	GPL7191	GSE12511	Global Expression Profiling in Atopic Eczema Reveals Reciprocal Expression of Inflammatory and Lipid Genes	Plos One
Atopic Eczema	PIM1	Expression profiling by array	GPL7191	GSE12511	Global Expression Profiling in Atopic Eczema Reveals Reciprocal Expression of Inflammatory and Lipid Genes	Plos One
Atopic Eczema	RAGE	Expression profiling by array	GPL7191	GSE12511	Global Expression Profiling in Atopic Eczema Reveals Reciprocal Expression of Inflammatory and Lipid Genes	Plos One
Atopic Eczema	CALM3	Expression profiling by array	GPL7191	GSE12511	Global Expression Profiling in Atopic Eczema Reveals Reciprocal Expression of Inflammatory and Lipid Genes	Plos One

Atopic Eczema	HMGCS1	Expression profiling by array	GPL7191	GSE12511	Global Expression Profiling in Atopic Eczema Reveals Reciprocal Expression of Inflammatory and Lipid Genes	Plos One
Atopic Eczema	CSF1R	Expression profiling by array	GPL7191	GSE12511	Global Expression Profiling in Atopic Eczema Reveals Reciprocal Expression of Inflammatory and Lipid Genes	Plos One
Atopic Eczema	ZNF626	Expression profiling by array	GPL7191	GSE12511	Global Expression Profiling in Atopic Eczema Reveals Reciprocal Expression of Inflammatory and Lipid Genes	Plos One
Atopic Eczema	MS4A4A	Expression profiling by array	GPL7191	GSE12511	Global Expression Profiling in Atopic Eczema Reveals Reciprocal Expression of Inflammatory and Lipid Genes	Plos One
Atopic Eczema	ATP2B2	Expression profiling by array	GPL7191	GSE12511	Global Expression Profiling in Atopic Eczema Reveals Reciprocal Expression of Inflammatory and Lipid Genes	Plos One
Atopic Eczema	PTPLA	Expression profiling by array	GPL7191	GSE12511	Global Expression Profiling in Atopic Eczema Reveals Reciprocal Expression of Inflammatory and Lipid Genes	Plos One
Atopic Eczema	DUSP1	Expression profiling by array	GPL7191	GSE12511	Global Expression Profiling in Atopic Eczema Reveals Reciprocal Expression of Inflammatory and Lipid Genes	Plos One
Atopic Eczema	CYP4F8	Expression profiling by array	GPL7191	GSE12511	Global Expression Profiling in Atopic Eczema Reveals Reciprocal Expression of Inflammatory and Lipid Genes	Plos One
Atopic Eczema	CP	Expression profiling by array	GPL7191	GSE12511	Global Expression Profiling in Atopic Eczema Reveals Reciprocal Expression of Inflammatory and Lipid Genes	Plos One
Atopic Eczema	C9ORF91	Expression profiling by array	GPL7191	GSE12511	Global Expression Profiling in Atopic Eczema Reveals Reciprocal Expression of Inflammatory and Lipid Genes	Plos One
Atopic Eczema	KIAA0174	Expression profiling by array	GPL7191	GSE12511	Global Expression Profiling in Atopic Eczema Reveals Reciprocal Expression of Inflammatory and Lipid Genes	Plos One
Atopic Eczema	MAP4K1	Expression profiling by array	GPL7191	GSE12511	Global Expression Profiling in Atopic Eczema Reveals Reciprocal Expression of Inflammatory and Lipid Genes	Plos One
Atopic Eczema	CADPS2	Expression profiling by array	GPL7191	GSE12511	Global Expression Profiling in Atopic Eczema Reveals Reciprocal Expression of Inflammatory and Lipid Genes	Plos One
Atopic Eczema	FCGRT	Expression profiling by array	GPL7191	GSE12511	Global Expression Profiling in Atopic Eczema Reveals Reciprocal Expression of Inflammatory and Lipid Genes	Plos One
Atopic Eczema	PRSS1	Expression profiling by array	GPL7191	GSE12511	Global Expression Profiling in Atopic Eczema Reveals Reciprocal Expression of Inflammatory and Lipid Genes	Plos One
Atopic Eczema	ATPAF2	Expression profiling by array	GPL7191	GSE12511	Global Expression Profiling in Atopic Eczema Reveals Reciprocal Expression of Inflammatory and Lipid Genes	Plos One
Atopic Eczema	C19ORF6	Expression profiling by array	GPL7191	GSE12511	Global Expression Profiling in Atopic Eczema Reveals Reciprocal Expression of Inflammatory and Lipid Genes	Plos One
Atopic Eczema	CD46	Expression profiling by array	GPL7191	GSE12511	Global Expression Profiling in Atopic Eczema Reveals Reciprocal Expression of Inflammatory and Lipid Genes	Plos One
Atopic Eczema	ITK	Expression profiling by array	GPL7191	GSE12511	Global Expression Profiling in Atopic Eczema Reveals Reciprocal Expression of Inflammatory and Lipid Genes	Plos One
Atopic Eczema	BMPR1A	Expression profiling by array	GPL7191	GSE12511	Global Expression Profiling in Atopic Eczema Reveals Reciprocal Expression of Inflammatory and Lipid Genes	Plos One
Atopic Eczema	CDC25B	Expression profiling by array	GPL7191	GSE12511	Global Expression Profiling in Atopic Eczema Reveals Reciprocal Expression of Inflammatory and Lipid Genes	Plos One
Atopic Eczema	LRRC56	Expression profiling by array	GPL7191	GSE12511	Global Expression Profiling in Atopic Eczema Reveals Reciprocal Expression of Inflammatory and Lipid Genes	Plos One
Atopic Eczema	FRZB	Expression profiling by array	GPL7191	GSE12511	Global Expression Profiling in Atopic Eczema Reveals Reciprocal Expression of Inflammatory and Lipid Genes	Plos One
Atopic Eczema	ARSB	Expression profiling by array	GPL7191	GSE12511	Global Expression Profiling in Atopic Eczema Reveals Reciprocal Expression of Inflammatory and Lipid Genes	Plos One
Atopic Eczema	AI351836	Expression profiling by array	GPL7191	GSE12511	Global Expression Profiling in Atopic Eczema Reveals Reciprocal Expression of Inflammatory and Lipid Genes	Plos One
Atopic Eczema	MUC5B	Expression profiling by array	GPL7191	GSE12511	Global Expression Profiling in Atopic Eczema Reveals Reciprocal Expression of Inflammatory and Lipid Genes	Plos One
Atopic Eczema	TUBA3	Expression profiling by array	GPL7191	GSE12511	Global Expression Profiling in Atopic Eczema Reveals Reciprocal Expression of Inflammatory and Lipid Genes	Plos One
Atopic Eczema	SLPI	Expression profiling by array	GPL7191	GSE12511	Global Expression Profiling in Atopic Eczema Reveals Reciprocal Expression of Inflammatory and Lipid Genes	Plos One
Atopic Eczema	AI128614	Expression profiling by array	GPL7191	GSE12511	Global Expression Profiling in Atopic Eczema Reveals Reciprocal Expression of Inflammatory and Lipid Genes	Plos One
Atopic Eczema	MARVELD2	Expression profiling by array	GPL7191	GSE12511	Global Expression Profiling in Atopic Eczema Reveals Reciprocal Expression of Inflammatory and Lipid Genes	Plos One
Atopic Eczema	ACADM	Expression profiling by array	GPL7191	GSE12511	Global Expression Profiling in Atopic Eczema Reveals Reciprocal Expression of Inflammatory and Lipid Genes	Plos One
Atopic Eczema	NBEA	Expression profiling by array	GPL7191	GSE12511	Global Expression Profiling in Atopic Eczema Reveals Reciprocal Expression of Inflammatory and Lipid Genes	Plos One
Atopic Eczema	TATDN3	Expression profiling by array	GPL7191	GSE12511	Global Expression Profiling in Atopic Eczema Reveals Reciprocal Expression of Inflammatory and Lipid Genes	Plos One
Atopic Eczema	OLFM1	Expression profiling by array	GPL7191	GSE12511	Global Expression Profiling in Atopic Eczema Reveals Reciprocal Expression of Inflammatory and Lipid Genes	Plos One
Atopic Eczema	FBLN5	Expression profiling by array	GPL7191	GSE12511	Global Expression Profiling in Atopic Eczema Reveals Reciprocal Expression of Inflammatory and Lipid Genes	Plos One
Atopic Eczema	H87106	Expression profiling by array	GPL7191	GSE12511	Global Expression Profiling in Atopic Eczema Reveals Reciprocal Expression of Inflammatory and Lipid Genes	Plos One
Atopic Eczema	PREX1	Expression profiling by array	GPL7191	GSE12511	Global Expression Profiling in Atopic Eczema Reveals Reciprocal Expression of Inflammatory and Lipid Genes	Plos One
Atopic Eczema	MGC7036	Expression profiling by array	GPL7191	GSE12511	Global Expression Profiling in Atopic Eczema Reveals Reciprocal Expression of Inflammatory and Lipid Genes	Plos One
Atopic Eczema	CYP51A1	Expression profiling by array	GPL7191	GSE12511	Global Expression Profiling in Atopic Eczema Reveals Reciprocal Expression of Inflammatory and Lipid Genes	Plos One
Atopic Eczema	GRAMD1C	Expression profiling by array	GPL7191	GSE12511	Global Expression Profiling in Atopic Eczema Reveals Reciprocal Expression of Inflammatory and Lipid Genes	Plos One
Atopic Eczema	NOB1	Expression profiling by array	GPL7191	GSE12511	Global Expression Profiling in Atopic Eczema Reveals Reciprocal Expression of Inflammatory and Lipid Genes	Plos One
Atopic Eczema	POR	Expression profiling by array	GPL7191	GSE12511	Global Expression Profiling in Atopic Eczema Reveals Reciprocal Expression of Inflammatory and Lipid Genes	Plos One
Atopic Eczema	CYBRD1	Expression profiling by array	GPL7191	GSE12511	Global Expression Profiling in Atopic Eczema Reveals Reciprocal Expression of Inflammatory and Lipid Genes	Plos One
Atopic Eczema	C1ORF21	Expression profiling by array	GPL7191	GSE12511	Global Expression Profiling in Atopic Eczema Reveals Reciprocal Expression of Inflammatory and Lipid Genes	Plos One
Atopic Eczema	RABAC1	Expression profiling by array	GPL7191	GSE12511	Global Expression Profiling in Atopic Eczema Reveals Reciprocal Expression of Inflammatory and Lipid Genes	Plos One
Atopic Eczema	PRDX4	Expression profiling by array	GPL7191	GSE12511	Global Expression Profiling in Atopic Eczema Reveals Reciprocal Expression of Inflammatory and Lipid Genes	Plos One
Atopic Eczema	SGK2	Expression profiling by array	GPL7191	GSE12511	Global Expression Profiling in Atopic Eczema Reveals Reciprocal Expression of Inflammatory and Lipid Genes	Plos One
Atopic Eczema	CYP4F2	Expression profiling by array	GPL7191	GSE12511	Global Expression Profiling in Atopic Eczema Reveals Reciprocal Expression of Inflammatory and Lipid Genes	Plos One
Atopic Eczema	MPG	Expression profiling by array	GPL7191	GSE12511	Global Expression Profiling in Atopic Eczema Reveals Reciprocal Expression of Inflammatory and Lipid Genes	Plos One
Atopic Eczema	C9ORF150	Expression profiling by array	GPL7191	GSE12511	Global Expression Profiling in Atopic Eczema Reveals Reciprocal Expression of Inflammatory and Lipid Genes	Plos One
Atopic Eczema	TMEM54	Expression profiling by array	GPL7191	GSE12511	Global Expression Profiling in Atopic Eczema Reveals Reciprocal Expression of Inflammatory and Lipid Genes	Plos One
Atopic Eczema	ALDH1L1	Expression profiling by array	GPL7191	GSE12511	Global Expression Profiling in Atopic Eczema Reveals Reciprocal Expression of Inflammatory and Lipid Genes	Plos One
Atopic Eczema	TUBG1	Expression profiling by array	GPL7191	GSE12511	Global Expression Profiling in Atopic Eczema Reveals Reciprocal Expression of Inflammatory and Lipid Genes	Plos One
Atopic Eczema	LOC643008	Expression profiling by array	GPL7191	GSE12511	Global Expression Profiling in Atopic Eczema Reveals Reciprocal Expression of Inflammatory and Lipid Genes	Plos One
Atopic Eczema	PDIA3	Expression profiling by array	GPL7191	GSE12511	Global Expression Profiling in Atopic Eczema Reveals Reciprocal Expression of Inflammatory and Lipid Genes	Plos One
Atopic Eczema	PLEC1	Expression profiling by array	GPL7191	GSE12511	Global Expression Profiling in Atopic Eczema Reveals Reciprocal Expression of Inflammatory and Lipid Genes	Plos One
Atopic Eczema	OCA2	Expression profiling by array	GPL7191	GSE12511	Global Expression Profiling in Atopic Eczema Reveals Reciprocal Expression of Inflammatory and Lipid Genes	Plos One
Atopic Eczema	WSB1	Expression profiling by array	GPL7191	GSE12511	Global Expression Profiling in Atopic Eczema Reveals Reciprocal Expression of Inflammatory and Lipid Genes	Plos One
Atopic Eczema	FAM46C	Expression profiling by array	GPL7191	GSE12511	Global Expression Profiling in Atopic Eczema Reveals Reciprocal Expression of Inflammatory and Lipid Genes	Plos One
Atopic Eczema	FAM96B	Expression profiling by array	GPL7191	GSE12511	Global Expression Profiling in Atopic Eczema Reveals Reciprocal Expression of Inflammatory and Lipid Genes	Plos One
Atopic Eczema	ZNF710	Expression profiling by array	GPL7191	GSE12511	Global Expression Profiling in Atopic Eczema Reveals Reciprocal Expression of Inflammatory and Lipid Genes	Plos One
Atopic Eczema	AA621402	Expression profiling by array	GPL7191	GSE12511	Global Expression Profiling in Atopic Eczema Reveals Reciprocal Expression of Inflammatory and Lipid Genes	Plos One

Atopic Eczema	FLOT2	Expression profiling by array	GPL7191	GSE12511	Global Expression Profiling in Atopic Eczema Reveals Reciprocal Expression of Inflammatory and Lipid Genes	Plos One
Atopic Eczema	AA670190	Expression profiling by array	GPL7191	GSE12511	Global Expression Profiling in Atopic Eczema Reveals Reciprocal Expression of Inflammatory and Lipid Genes	Plos One
Atopic Eczema	DPP4	Expression profiling by array	GPL7191	GSE12511	Global Expression Profiling in Atopic Eczema Reveals Reciprocal Expression of Inflammatory and Lipid Genes	Plos One
Atopic Eczema	N64033	Expression profiling by array	GPL7191	GSE12511	Global Expression Profiling in Atopic Eczema Reveals Reciprocal Expression of Inflammatory and Lipid Genes	Plos One
Atopic Eczema	GNAI2	Expression profiling by array	GPL7191	GSE12511	Global Expression Profiling in Atopic Eczema Reveals Reciprocal Expression of Inflammatory and Lipid Genes	Plos One
Atopic Eczema	C14ORF92	Expression profiling by array	GPL7191	GSE12511	Global Expression Profiling in Atopic Eczema Reveals Reciprocal Expression of Inflammatory and Lipid Genes	Plos One
Atopic Eczema	ELF3	Expression profiling by array	GPL7191	GSE12511	Global Expression Profiling in Atopic Eczema Reveals Reciprocal Expression of Inflammatory and Lipid Genes	Plos One
Atopic Eczema	KIF13A	Expression profiling by array	GPL7191	GSE12511	Global Expression Profiling in Atopic Eczema Reveals Reciprocal Expression of Inflammatory and Lipid Genes	Plos One
Atopic Eczema	CA2	Expression profiling by array	GPL7191	GSE12511	Global Expression Profiling in Atopic Eczema Reveals Reciprocal Expression of Inflammatory and Lipid Genes	Plos One
Atopic Eczema	ARG1	Expression profiling by array	GPL7191	GSE12511	Global Expression Profiling in Atopic Eczema Reveals Reciprocal Expression of Inflammatory and Lipid Genes	Plos One
Atopic Eczema	C12ORF10	Expression profiling by array	GPL7191	GSE12511	Global Expression Profiling in Atopic Eczema Reveals Reciprocal Expression of Inflammatory and Lipid Genes	Plos One
Atopic Eczema	CTDP1	Expression profiling by array	GPL7191	GSE12511	Global Expression Profiling in Atopic Eczema Reveals Reciprocal Expression of Inflammatory and Lipid Genes	Plos One
Atopic Eczema	CD6	Expression profiling by array	GPL7191	GSE12511	Global Expression Profiling in Atopic Eczema Reveals Reciprocal Expression of Inflammatory and Lipid Genes	Plos One
Atopic Eczema	EPSTI1	Expression profiling by array	GPL7191	GSE12511	Global Expression Profiling in Atopic Eczema Reveals Reciprocal Expression of Inflammatory and Lipid Genes	Plos One
Atopic Eczema	SPRR4	Expression profiling by array	GPL7191	GSE12511	Global Expression Profiling in Atopic Eczema Reveals Reciprocal Expression of Inflammatory and Lipid Genes	Plos One
Atopic Eczema	TSTA3	Expression profiling by array	GPL7191	GSE12511	Global Expression Profiling in Atopic Eczema Reveals Reciprocal Expression of Inflammatory and Lipid Genes	Plos One
Atopic Eczema	UQCRB	Expression profiling by array	GPL7191	GSE12511	Global Expression Profiling in Atopic Eczema Reveals Reciprocal Expression of Inflammatory and Lipid Genes	Plos One
Atopic Eczema	INPP5D	Expression profiling by array	GPL7191	GSE12511	Global Expression Profiling in Atopic Eczema Reveals Reciprocal Expression of Inflammatory and Lipid Genes	Plos One
Atopic Eczema	FLT3LG	Expression profiling by array	GPL7191	GSE12511	Global Expression Profiling in Atopic Eczema Reveals Reciprocal Expression of Inflammatory and Lipid Genes	Plos One
Atopic Eczema	SSR4	Expression profiling by array	GPL7191	GSE12511	Global Expression Profiling in Atopic Eczema Reveals Reciprocal Expression of Inflammatory and Lipid Genes	Plos One
Atopic Eczema	JOSD1	Expression profiling by array	GPL7191	GSE12511	Global Expression Profiling in Atopic Eczema Reveals Reciprocal Expression of Inflammatory and Lipid Genes	Plos One
Atopic Eczema	TINF2	Expression profiling by array	GPL7191	GSE12511	Global Expression Profiling in Atopic Eczema Reveals Reciprocal Expression of Inflammatory and Lipid Genes	Plos One
Atopic Eczema	MSN	Expression profiling by array	GPL7191	GSE12511	Global Expression Profiling in Atopic Eczema Reveals Reciprocal Expression of Inflammatory and Lipid Genes	Plos One
Atopic Eczema	AI091654	Expression profiling by array	GPL7191	GSE12511	Global Expression Profiling in Atopic Eczema Reveals Reciprocal Expression of Inflammatory and Lipid Genes	Plos One
Atopic Eczema	CSK	Expression profiling by array	GPL7191	GSE12511	Global Expression Profiling in Atopic Eczema Reveals Reciprocal Expression of Inflammatory and Lipid Genes	Plos One
Atopic Eczema	ITGB8	Expression profiling by array	GPL7191	GSE12511	Global Expression Profiling in Atopic Eczema Reveals Reciprocal Expression of Inflammatory and Lipid Genes	Plos One
Atopic Eczema	LOC388323	Expression profiling by array	GPL7191	GSE12511	Global Expression Profiling in Atopic Eczema Reveals Reciprocal Expression of Inflammatory and Lipid Genes	Plos One
Atopic Eczema	PREPL	Expression profiling by array	GPL7191	GSE12511	Global Expression Profiling in Atopic Eczema Reveals Reciprocal Expression of Inflammatory and Lipid Genes	Plos One
Atopic Eczema	MKRN1	Expression profiling by array	GPL7191	GSE12511	Global Expression Profiling in Atopic Eczema Reveals Reciprocal Expression of Inflammatory and Lipid Genes	Plos One
Atopic Eczema	GBP2	Expression profiling by array	GPL7191	GSE12511	Global Expression Profiling in Atopic Eczema Reveals Reciprocal Expression of Inflammatory and Lipid Genes	Plos One
Atopic Eczema	COG4	Expression profiling by array	GPL7191	GSE12511	Global Expression Profiling in Atopic Eczema Reveals Reciprocal Expression of Inflammatory and Lipid Genes	Plos One
Atopic Eczema	TSPAN33	Expression profiling by array	GPL7191	GSE12511	Global Expression Profiling in Atopic Eczema Reveals Reciprocal Expression of Inflammatory and Lipid Genes	Plos One
Atopic Eczema	PSMC4	Expression profiling by array	GPL7191	GSE12511	Global Expression Profiling in Atopic Eczema Reveals Reciprocal Expression of Inflammatory and Lipid Genes	Plos One
Atopic Eczema	EIF3S3	Expression profiling by array	GPL7191	GSE12511	Global Expression Profiling in Atopic Eczema Reveals Reciprocal Expression of Inflammatory and Lipid Genes	Plos One
Atopic Eczema	ZNF428	Expression profiling by array	GPL7191	GSE12511	Global Expression Profiling in Atopic Eczema Reveals Reciprocal Expression of Inflammatory and Lipid Genes	Plos One
Atopic Eczema	RBPSUH	Expression profiling by array	GPL7191	GSE12511	Global Expression Profiling in Atopic Eczema Reveals Reciprocal Expression of Inflammatory and Lipid Genes	Plos One
Atopic Eczema	ACP6	Expression profiling by array	GPL7191	GSE12511	Global Expression Profiling in Atopic Eczema Reveals Reciprocal Expression of Inflammatory and Lipid Genes	Plos One
Atopic Eczema	PDE6A	Expression profiling by array	GPL7191	GSE12511	Global Expression Profiling in Atopic Eczema Reveals Reciprocal Expression of Inflammatory and Lipid Genes	Plos One
Atopic Eczema	GPC4	Expression profiling by array	GPL7191	GSE12511	Global Expression Profiling in Atopic Eczema Reveals Reciprocal Expression of Inflammatory and Lipid Genes	Plos One
Atopic Eczema	LOC400128	Expression profiling by array	GPL7191	GSE12511	Global Expression Profiling in Atopic Eczema Reveals Reciprocal Expression of Inflammatory and Lipid Genes	Plos One
Atopic Eczema	TBC1D9	Expression profiling by array	GPL7191	GSE12511	Global Expression Profiling in Atopic Eczema Reveals Reciprocal Expression of Inflammatory and Lipid Genes	Plos One
Atopic Eczema	SLC2A1	Expression profiling by array	GPL7191	GSE12511	Global Expression Profiling in Atopic Eczema Reveals Reciprocal Expression of Inflammatory and Lipid Genes	Plos One
Atopic Eczema	UBE2D3	Expression profiling by array	GPL7191	GSE12511	Global Expression Profiling in Atopic Eczema Reveals Reciprocal Expression of Inflammatory and Lipid Genes	Plos One
Atopic Eczema	PTPRC	Expression profiling by array	GPL7191	GSE12511	Global Expression Profiling in Atopic Eczema Reveals Reciprocal Expression of Inflammatory and Lipid Genes	Plos One
Atopic Eczema	EIF3S7	Expression profiling by array	GPL7191	GSE12511	Global Expression Profiling in Atopic Eczema Reveals Reciprocal Expression of Inflammatory and Lipid Genes	Plos One
Atopic Eczema	INSIG1	Expression profiling by array	GPL7191	GSE12511	Global Expression Profiling in Atopic Eczema Reveals Reciprocal Expression of Inflammatory and Lipid Genes	Plos One
Atopic Eczema	PON3	Expression profiling by array	GPL7191	GSE12511	Global Expression Profiling in Atopic Eczema Reveals Reciprocal Expression of Inflammatory and Lipid Genes	Plos One
Atopic Eczema	CIP29	Expression profiling by array	GPL7191	GSE12511	Global Expression Profiling in Atopic Eczema Reveals Reciprocal Expression of Inflammatory and Lipid Genes	Plos One
Atopic Eczema	MEOX1	Expression profiling by array	GPL7191	GSE12511	Global Expression Profiling in Atopic Eczema Reveals Reciprocal Expression of Inflammatory and Lipid Genes	Plos One
Atopic Eczema	CLDN15	Expression profiling by array	GPL7191	GSE12511	Global Expression Profiling in Atopic Eczema Reveals Reciprocal Expression of Inflammatory and Lipid Genes	Plos One
Atopic Eczema	C1QB	Expression profiling by array	GPL7191	GSE12511	Global Expression Profiling in Atopic Eczema Reveals Reciprocal Expression of Inflammatory and Lipid Genes	Plos One
Atopic Eczema	LIPH	Expression profiling by array	GPL7191	GSE12511	Global Expression Profiling in Atopic Eczema Reveals Reciprocal Expression of Inflammatory and Lipid Genes	Plos One
Atopic Eczema	TIA1	Expression profiling by array	GPL7191	GSE12511	Global Expression Profiling in Atopic Eczema Reveals Reciprocal Expression of Inflammatory and Lipid Genes	Plos One
Atopic Eczema	CSPG4	Expression profiling by array	GPL7191	GSE12511	Global Expression Profiling in Atopic Eczema Reveals Reciprocal Expression of Inflammatory and Lipid Genes	Plos One
Atopic Eczema	ALOX15B	Expression profiling by array	GPL7191	GSE12511	Global Expression Profiling in Atopic Eczema Reveals Reciprocal Expression of Inflammatory and Lipid Genes	Plos One
Atopic Eczema	ANKRD6	Expression profiling by array	GPL7191	GSE12511	Global Expression Profiling in Atopic Eczema Reveals Reciprocal Expression of Inflammatory and Lipid Genes	Plos One
Atopic Eczema	ATP2B4	Expression profiling by array	GPL7191	GSE12511	Global Expression Profiling in Atopic Eczema Reveals Reciprocal Expression of Inflammatory and Lipid Genes	Plos One
Atopic Eczema	MCF2L2	Expression profiling by array	GPL7191	GSE12511	Global Expression Profiling in Atopic Eczema Reveals Reciprocal Expression of Inflammatory and Lipid Genes	Plos One
Atopic Eczema	LOC400948	Expression profiling by array	GPL7191	GSE12511	Global Expression Profiling in Atopic Eczema Reveals Reciprocal Expression of Inflammatory and Lipid Genes	Plos One
Atopic Eczema	UBIAD1	Expression profiling by array	GPL7191	GSE12511	Global Expression Profiling in Atopic Eczema Reveals Reciprocal Expression of Inflammatory and Lipid Genes	Plos One
Atopic Eczema	ELOVL5	Expression profiling by array	GPL7191	GSE12511	Global Expression Profiling in Atopic Eczema Reveals Reciprocal Expression of Inflammatory and Lipid Genes	Plos One
Atopic Eczema	MFAP5	Expression profiling by array	GPL7191	GSE12511	Global Expression Profiling in Atopic Eczema Reveals Reciprocal Expression of Inflammatory and Lipid Genes	Plos One

Atopic Eczema	PRR5	Expression profiling by array	GPL7191	GSE12511	Global Expression Profiling in Atopic Eczema Reveals Reciprocal Expression of Inflammatory and Lipid Genes	Plos One
Atopic Eczema	EXOSC7	Expression profiling by array	GPL7191	GSE12511	Global Expression Profiling in Atopic Eczema Reveals Reciprocal Expression of Inflammatory and Lipid Genes	Plos One
Atopic Eczema	HN1	Expression profiling by array	GPL7191	GSE12511	Global Expression Profiling in Atopic Eczema Reveals Reciprocal Expression of Inflammatory and Lipid Genes	Plos One
Atopic Eczema	PLEKHA4	Expression profiling by array	GPL7191	GSE12511	Global Expression Profiling in Atopic Eczema Reveals Reciprocal Expression of Inflammatory and Lipid Genes	Plos One
Atopic Eczema	FAU	Expression profiling by array	GPL7191	GSE12511	Global Expression Profiling in Atopic Eczema Reveals Reciprocal Expression of Inflammatory and Lipid Genes	Plos One
Atopic Eczema	AATF	Expression profiling by array	GPL7191	GSE12511	Global Expression Profiling in Atopic Eczema Reveals Reciprocal Expression of Inflammatory and Lipid Genes	Plos One
Atopic Eczema	DHX8	Expression profiling by array	GPL7191	GSE12511	Global Expression Profiling in Atopic Eczema Reveals Reciprocal Expression of Inflammatory and Lipid Genes	Plos One
Atopic Eczema	PDZD6	Expression profiling by array	GPL7191	GSE12511	Global Expression Profiling in Atopic Eczema Reveals Reciprocal Expression of Inflammatory and Lipid Genes	Plos One
Atopic Eczema	WISP2	Expression profiling by array	GPL7191	GSE12511	Global Expression Profiling in Atopic Eczema Reveals Reciprocal Expression of Inflammatory and Lipid Genes	Plos One
Atopic Eczema	AA975259	Expression profiling by array	GPL7191	GSE12511	Global Expression Profiling in Atopic Eczema Reveals Reciprocal Expression of Inflammatory and Lipid Genes	Plos One
Atopic Eczema	MYO6	Expression profiling by array	GPL7191	GSE12511	Global Expression Profiling in Atopic Eczema Reveals Reciprocal Expression of Inflammatory and Lipid Genes	Plos One
Atopic Eczema	CD300A	Expression profiling by array	GPL7191	GSE12511	Global Expression Profiling in Atopic Eczema Reveals Reciprocal Expression of Inflammatory and Lipid Genes	Plos One
Atopic Eczema	GATM	Expression profiling by array	GPL7191	GSE12511	Global Expression Profiling in Atopic Eczema Reveals Reciprocal Expression of Inflammatory and Lipid Genes	Plos One
Atopic Eczema	TOB1	Expression profiling by array	GPL7191	GSE12511	Global Expression Profiling in Atopic Eczema Reveals Reciprocal Expression of Inflammatory and Lipid Genes	Plos One
Atopic Eczema	TMEM97	Expression profiling by array	GPL7191	GSE12511	Global Expression Profiling in Atopic Eczema Reveals Reciprocal Expression of Inflammatory and Lipid Genes	Plos One
Atopic Eczema	CD1B	Expression profiling by array	GPL7191	GSE12511	Global Expression Profiling in Atopic Eczema Reveals Reciprocal Expression of Inflammatory and Lipid Genes	Plos One
Atopic Eczema	ELF4	Expression profiling by array	GPL7191	GSE12511	Global Expression Profiling in Atopic Eczema Reveals Reciprocal Expression of Inflammatory and Lipid Genes	Plos One
Atopic Eczema	RCSD1	Expression profiling by array	GPL7191	GSE12511	Global Expression Profiling in Atopic Eczema Reveals Reciprocal Expression of Inflammatory and Lipid Genes	Plos One
Atopic Eczema	CASP4	Expression profiling by array	GPL7191	GSE12511	Global Expression Profiling in Atopic Eczema Reveals Reciprocal Expression of Inflammatory and Lipid Genes	Plos One
Atopic Eczema	CXCL12	Expression profiling by array	GPL7191	GSE12511	Global Expression Profiling in Atopic Eczema Reveals Reciprocal Expression of Inflammatory and Lipid Genes	Plos One
Atopic Eczema	DMKN	Expression profiling by array	GPL7191	GSE12511	Global Expression Profiling in Atopic Eczema Reveals Reciprocal Expression of Inflammatory and Lipid Genes	Plos One
Atopic Eczema	IL2RG	Expression profiling by array	GPL7191	GSE12511	Global Expression Profiling in Atopic Eczema Reveals Reciprocal Expression of Inflammatory and Lipid Genes	Plos One
Atopic Eczema	C2ORF18	Expression profiling by array	GPL7191	GSE12511	Global Expression Profiling in Atopic Eczema Reveals Reciprocal Expression of Inflammatory and Lipid Genes	Plos One
Atopic Eczema	GSTP1	Expression profiling by array	GPL7191	GSE12511	Global Expression Profiling in Atopic Eczema Reveals Reciprocal Expression of Inflammatory and Lipid Genes	Plos One
Atopic Eczema	TNFAIP3	Expression profiling by array	GPL7191	GSE12511	Global Expression Profiling in Atopic Eczema Reveals Reciprocal Expression of Inflammatory and Lipid Genes	Plos One
Atopic Eczema	FTL	Expression profiling by array	GPL7191	GSE12511	Global Expression Profiling in Atopic Eczema Reveals Reciprocal Expression of Inflammatory and Lipid Genes	Plos One
Atopic Eczema	NFKBIE	Expression profiling by array	GPL7191	GSE12511	Global Expression Profiling in Atopic Eczema Reveals Reciprocal Expression of Inflammatory and Lipid Genes	Plos One
Atopic Eczema	AA991624	Expression profiling by array	GPL7191	GSE12511	Global Expression Profiling in Atopic Eczema Reveals Reciprocal Expression of Inflammatory and Lipid Genes	Plos One
Atopic Eczema	CNTNAP1	Expression profiling by array	GPL7191	GSE12511	Global Expression Profiling in Atopic Eczema Reveals Reciprocal Expression of Inflammatory and Lipid Genes	Plos One
Atopic Eczema	PIAS1	Expression profiling by array	GPL7191	GSE12511	Global Expression Profiling in Atopic Eczema Reveals Reciprocal Expression of Inflammatory and Lipid Genes	Plos One
Atopic Eczema	MAP3K14	Expression profiling by array	GPL7191	GSE12511	Global Expression Profiling in Atopic Eczema Reveals Reciprocal Expression of Inflammatory and Lipid Genes	Plos One
Atopic Eczema	C10ORF99	Expression profiling by array	GPL7191	GSE12511	Global Expression Profiling in Atopic Eczema Reveals Reciprocal Expression of Inflammatory and Lipid Genes	Plos One
Atopic Eczema	ELK3	Expression profiling by array	GPL7191	GSE12511	Global Expression Profiling in Atopic Eczema Reveals Reciprocal Expression of Inflammatory and Lipid Genes	Plos One
Atopic Eczema	ZNF539	Expression profiling by array	GPL7191	GSE12511	Global Expression Profiling in Atopic Eczema Reveals Reciprocal Expression of Inflammatory and Lipid Genes	Plos One
Atopic Eczema	ZP1	Expression profiling by array	GPL7191	GSE12511	Global Expression Profiling in Atopic Eczema Reveals Reciprocal Expression of Inflammatory and Lipid Genes	Plos One
Atopic Eczema	TAP1	Expression profiling by array	GPL7191	GSE12511	Global Expression Profiling in Atopic Eczema Reveals Reciprocal Expression of Inflammatory and Lipid Genes	Plos One
Atopic Eczema	LRRC39	Expression profiling by array	GPL7191	GSE12511	Global Expression Profiling in Atopic Eczema Reveals Reciprocal Expression of Inflammatory and Lipid Genes	Plos One
Atopic Eczema	BCAT2	Expression profiling by array	GPL7191	GSE12511	Global Expression Profiling in Atopic Eczema Reveals Reciprocal Expression of Inflammatory and Lipid Genes	Plos One
Atopic Eczema	PIP5K1B	Expression profiling by array	GPL7191	GSE12511	Global Expression Profiling in Atopic Eczema Reveals Reciprocal Expression of Inflammatory and Lipid Genes	Plos One
Atopic Eczema	CD33	Expression profiling by array	GPL7191	GSE12511	Global Expression Profiling in Atopic Eczema Reveals Reciprocal Expression of Inflammatory and Lipid Genes	Plos One
Atopic Eczema	IL12A	Expression profiling by array	GPL7191	GSE12511	Global Expression Profiling in Atopic Eczema Reveals Reciprocal Expression of Inflammatory and Lipid Genes	Plos One
Atopic Eczema	EHD2	Expression profiling by array	GPL7191	GSE12511	Global Expression Profiling in Atopic Eczema Reveals Reciprocal Expression of Inflammatory and Lipid Genes	Plos One
Atopic Eczema	PET112L	Expression profiling by array	GPL7191	GSE12511	Global Expression Profiling in Atopic Eczema Reveals Reciprocal Expression of Inflammatory and Lipid Genes	Plos One
Atopic Eczema	MYCBP	Expression profiling by array	GPL7191	GSE12511	Global Expression Profiling in Atopic Eczema Reveals Reciprocal Expression of Inflammatory and Lipid Genes	Plos One
Atopic Eczema	CXCL1	Expression profiling by array	GPL7191	GSE12511	Global Expression Profiling in Atopic Eczema Reveals Reciprocal Expression of Inflammatory and Lipid Genes	Plos One
Atopic Eczema	FEZ2	Expression profiling by array	GPL7191	GSE12511	Global Expression Profiling in Atopic Eczema Reveals Reciprocal Expression of Inflammatory and Lipid Genes	Plos One
Atopic Eczema	SNX8	Expression profiling by array	GPL7191	GSE12511	Global Expression Profiling in Atopic Eczema Reveals Reciprocal Expression of Inflammatory and Lipid Genes	Plos One
Atopic Eczema	MAN1A1	Expression profiling by array	GPL7191	GSE12511	Global Expression Profiling in Atopic Eczema Reveals Reciprocal Expression of Inflammatory and Lipid Genes	Plos One
Atopic Eczema	ENC1	Expression profiling by array	GPL7191	GSE12511	Global Expression Profiling in Atopic Eczema Reveals Reciprocal Expression of Inflammatory and Lipid Genes	Plos One
Atopic Eczema	ELL2	Expression profiling by array	GPL7191	GSE12511	Global Expression Profiling in Atopic Eczema Reveals Reciprocal Expression of Inflammatory and Lipid Genes	Plos One
Atopic Eczema	ARHGAP4	Expression profiling by array	GPL7191	GSE12511	Global Expression Profiling in Atopic Eczema Reveals Reciprocal Expression of Inflammatory and Lipid Genes	Plos One
Atopic Eczema	NSDHL	Expression profiling by array	GPL7191	GSE12511	Global Expression Profiling in Atopic Eczema Reveals Reciprocal Expression of Inflammatory and Lipid Genes	Plos One
Atopic Eczema	MMP17	Expression profiling by array	GPL7191	GSE12511	Global Expression Profiling in Atopic Eczema Reveals Reciprocal Expression of Inflammatory and Lipid Genes	Plos One
Atopic Eczema	CEBPB	Expression profiling by array	GPL7191	GSE12511	Global Expression Profiling in Atopic Eczema Reveals Reciprocal Expression of Inflammatory and Lipid Genes	Plos One
Atopic Eczema	PLEKHM2	Expression profiling by array	GPL7191	GSE12511	Global Expression Profiling in Atopic Eczema Reveals Reciprocal Expression of Inflammatory and Lipid Genes	Plos One
Atopic Eczema	TRPV2	Expression profiling by array	GPL7191	GSE12511	Global Expression Profiling in Atopic Eczema Reveals Reciprocal Expression of Inflammatory and Lipid Genes	Plos One
Atopic Eczema	CCDC34	Expression profiling by array	GPL7191	GSE12511	Global Expression Profiling in Atopic Eczema Reveals Reciprocal Expression of Inflammatory and Lipid Genes	Plos One
Atopic Eczema	PLEKHO1	Expression profiling by array	GPL7191	GSE12511	Global Expression Profiling in Atopic Eczema Reveals Reciprocal Expression of Inflammatory and Lipid Genes	Plos One
Atopic Eczema	VGLL3	Expression profiling by array	GPL7191	GSE12511	Global Expression Profiling in Atopic Eczema Reveals Reciprocal Expression of Inflammatory and Lipid Genes	Plos One
Atopic Eczema	CLEC3B	Expression profiling by array	GPL7191	GSE12511	Global Expression Profiling in Atopic Eczema Reveals Reciprocal Expression of Inflammatory and Lipid Genes	Plos One
Atopic Eczema	SHROOM2	Expression profiling by array	GPL7191	GSE12511	Global Expression Profiling in Atopic Eczema Reveals Reciprocal Expression of Inflammatory and Lipid Genes	Plos One
Atopic Eczema	CPXM2	Expression profiling by array	GPL7191	GSE12511	Global Expression Profiling in Atopic Eczema Reveals Reciprocal Expression of Inflammatory and Lipid Genes	Plos One

Atopic Eczema	PDCD4	Expression profiling by array	GPL7191	GSE12511	Global Expression Profiling in Atopic Eczema Reveals Reciprocal Expression of Inflammatory and Lipid Genes	Plos One
Atopic Eczema	FN1	Expression profiling by array	GPL7191	GSE12511	Global Expression Profiling in Atopic Eczema Reveals Reciprocal Expression of Inflammatory and Lipid Genes	Plos One
Atopic Eczema	LOC643980	Expression profiling by array	GPL7191	GSE12511	Global Expression Profiling in Atopic Eczema Reveals Reciprocal Expression of Inflammatory and Lipid Genes	Plos One
Atopic Eczema	IRF7	Expression profiling by array	GPL7191	GSE12511	Global Expression Profiling in Atopic Eczema Reveals Reciprocal Expression of Inflammatory and Lipid Genes	Plos One
Atopic Eczema	GABRP	Expression profiling by array	GPL7191	GSE12511	Global Expression Profiling in Atopic Eczema Reveals Reciprocal Expression of Inflammatory and Lipid Genes	Plos One
Atopic Eczema	LY6G6C	Expression profiling by array	GPL7191	GSE12511	Global Expression Profiling in Atopic Eczema Reveals Reciprocal Expression of Inflammatory and Lipid Genes	Plos One
Atopic Eczema	SEMA6D	Expression profiling by array	GPL7191	GSE12511	Global Expression Profiling in Atopic Eczema Reveals Reciprocal Expression of Inflammatory and Lipid Genes	Plos One
Atopic Eczema	ZNF256	Expression profiling by array	GPL7191	GSE12511	Global Expression Profiling in Atopic Eczema Reveals Reciprocal Expression of Inflammatory and Lipid Genes	Plos One
Atopic Eczema	ANXA11	Expression profiling by array	GPL7191	GSE12511	Global Expression Profiling in Atopic Eczema Reveals Reciprocal Expression of Inflammatory and Lipid Genes	Plos One
Atopic Eczema	F10	Expression profiling by array	GPL7191	GSE12511	Global Expression Profiling in Atopic Eczema Reveals Reciprocal Expression of Inflammatory and Lipid Genes	Plos One
Atopic Eczema	ZNF83	Expression profiling by array	GPL7191	GSE12511	Global Expression Profiling in Atopic Eczema Reveals Reciprocal Expression of Inflammatory and Lipid Genes	Plos One
Atopic Eczema	ACOX2	Expression profiling by array	GPL7191	GSE12511	Global Expression Profiling in Atopic Eczema Reveals Reciprocal Expression of Inflammatory and Lipid Genes	Plos One
Atopic Eczema	ZCCHC6	Expression profiling by array	GPL7191	GSE12511	Global Expression Profiling in Atopic Eczema Reveals Reciprocal Expression of Inflammatory and Lipid Genes	Plos One
Atopic Eczema	SLC44A1	Expression profiling by array	GPL7191	GSE12511	Global Expression Profiling in Atopic Eczema Reveals Reciprocal Expression of Inflammatory and Lipid Genes	Plos One
Atopic Eczema	LOC125893	Expression profiling by array	GPL7191	GSE12511	Global Expression Profiling in Atopic Eczema Reveals Reciprocal Expression of Inflammatory and Lipid Genes	Plos One
Atopic Eczema	PYGM	Expression profiling by array	GPL7191	GSE12511	Global Expression Profiling in Atopic Eczema Reveals Reciprocal Expression of Inflammatory and Lipid Genes	Plos One
Atopic Eczema	CEBPG	Expression profiling by array	GPL7191	GSE12511	Global Expression Profiling in Atopic Eczema Reveals Reciprocal Expression of Inflammatory and Lipid Genes	Plos One
Atopic Eczema	C3AR1	Expression profiling by array	GPL7191	GSE12511	Global Expression Profiling in Atopic Eczema Reveals Reciprocal Expression of Inflammatory and Lipid Genes	Plos One
Atopic Eczema	CDIPT	Expression profiling by array	GPL7191	GSE12511	Global Expression Profiling in Atopic Eczema Reveals Reciprocal Expression of Inflammatory and Lipid Genes	Plos One
Atopic Eczema	FAM46A	Expression profiling by array	GPL7191	GSE12511	Global Expression Profiling in Atopic Eczema Reveals Reciprocal Expression of Inflammatory and Lipid Genes	Plos One
Atopic Eczema	GABARAP	Expression profiling by array	GPL7191	GSE12511	Global Expression Profiling in Atopic Eczema Reveals Reciprocal Expression of Inflammatory and Lipid Genes	Plos One
Atopic Eczema	PPGB	Expression profiling by array	GPL7191	GSE12511	Global Expression Profiling in Atopic Eczema Reveals Reciprocal Expression of Inflammatory and Lipid Genes	Plos One
Atopic Eczema	CTPS	Expression profiling by array	GPL7191	GSE12511	Global Expression Profiling in Atopic Eczema Reveals Reciprocal Expression of Inflammatory and Lipid Genes	Plos One
Atopic Eczema	CDKN2D	Expression profiling by array	GPL7191	GSE12511	Global Expression Profiling in Atopic Eczema Reveals Reciprocal Expression of Inflammatory and Lipid Genes	Plos One
Atopic Eczema	TSEN34	Expression profiling by array	GPL7191	GSE12511	Global Expression Profiling in Atopic Eczema Reveals Reciprocal Expression of Inflammatory and Lipid Genes	Plos One
Atopic Eczema	ZNF664	Expression profiling by array	GPL7191	GSE12511	Global Expression Profiling in Atopic Eczema Reveals Reciprocal Expression of Inflammatory and Lipid Genes	Plos One
Atopic Eczema	LOC642775	Expression profiling by array	GPL7191	GSE12511	Global Expression Profiling in Atopic Eczema Reveals Reciprocal Expression of Inflammatory and Lipid Genes	Plos One
Atopic Eczema	MPP7	Expression profiling by array	GPL7191	GSE12511	Global Expression Profiling in Atopic Eczema Reveals Reciprocal Expression of Inflammatory and Lipid Genes	Plos One
Atopic Eczema	LGALS9	Expression profiling by array	GPL7191	GSE12511	Global Expression Profiling in Atopic Eczema Reveals Reciprocal Expression of Inflammatory and Lipid Genes	Plos One
Atopic Eczema	CSF2RB	Expression profiling by array	GPL7191	GSE12511	Global Expression Profiling in Atopic Eczema Reveals Reciprocal Expression of Inflammatory and Lipid Genes	Plos One
Atopic Eczema	NEK4	Expression profiling by array	GPL7191	GSE12511	Global Expression Profiling in Atopic Eczema Reveals Reciprocal Expression of Inflammatory and Lipid Genes	Plos One
Atopic Eczema	PEX11A	Expression profiling by array	GPL7191	GSE12511	Global Expression Profiling in Atopic Eczema Reveals Reciprocal Expression of Inflammatory and Lipid Genes	Plos One
Atopic Eczema	LOC440341	Expression profiling by array	GPL7191	GSE12511	Global Expression Profiling in Atopic Eczema Reveals Reciprocal Expression of Inflammatory and Lipid Genes	Plos One
Atopic Eczema	NOTCH4	Expression profiling by array	GPL7191	GSE12511	Global Expression Profiling in Atopic Eczema Reveals Reciprocal Expression of Inflammatory and Lipid Genes	Plos One
Atopic Eczema	R19275	Expression profiling by array	GPL7191	GSE12511	Global Expression Profiling in Atopic Eczema Reveals Reciprocal Expression of Inflammatory and Lipid Genes	Plos One
Atopic Eczema	SNRK	Expression profiling by array	GPL7191	GSE12511	Global Expression Profiling in Atopic Eczema Reveals Reciprocal Expression of Inflammatory and Lipid Genes	Plos One
Atopic Eczema	MS4A6A	Expression profiling by array	GPL7191	GSE12511	Global Expression Profiling in Atopic Eczema Reveals Reciprocal Expression of Inflammatory and Lipid Genes	Plos One
Atopic Eczema	PPIG	Expression profiling by array	GPL7191	GSE12511	Global Expression Profiling in Atopic Eczema Reveals Reciprocal Expression of Inflammatory and Lipid Genes	Plos One
Atopic Eczema	EIF3S4	Expression profiling by array	GPL7191	GSE12511	Global Expression Profiling in Atopic Eczema Reveals Reciprocal Expression of Inflammatory and Lipid Genes	Plos One
Atopic Eczema	FBP1	Expression profiling by array	GPL7191	GSE12511	Global Expression Profiling in Atopic Eczema Reveals Reciprocal Expression of Inflammatory and Lipid Genes	Plos One
Atopic Eczema	SH3RF1	Expression profiling by array	GPL7191	GSE12511	Global Expression Profiling in Atopic Eczema Reveals Reciprocal Expression of Inflammatory and Lipid Genes	Plos One
Atopic Eczema	SRA1	Expression profiling by array	GPL7191	GSE12511	Global Expression Profiling in Atopic Eczema Reveals Reciprocal Expression of Inflammatory and Lipid Genes	Plos One
Atopic Eczema	LOC644488	Expression profiling by array	GPL7191	GSE12511	Global Expression Profiling in Atopic Eczema Reveals Reciprocal Expression of Inflammatory and Lipid Genes	Plos One
Atopic Eczema	CSNK2B	Expression profiling by array	GPL7191	GSE12511	Global Expression Profiling in Atopic Eczema Reveals Reciprocal Expression of Inflammatory and Lipid Genes	Plos One
Atopic Eczema	FAM83D	Expression profiling by array	GPL7191	GSE12511	Global Expression Profiling in Atopic Eczema Reveals Reciprocal Expression of Inflammatory and Lipid Genes	Plos One
Atopic Eczema	ADAMTS14	Expression profiling by array	GPL7191	GSE12511	Global Expression Profiling in Atopic Eczema Reveals Reciprocal Expression of Inflammatory and Lipid Genes	Plos One
Atopic Eczema	BCKDHB	Expression profiling by array	GPL7191	GSE12511	Global Expression Profiling in Atopic Eczema Reveals Reciprocal Expression of Inflammatory and Lipid Genes	Plos One
Atopic Eczema	ARL1	Expression profiling by array	GPL7191	GSE12511	Global Expression Profiling in Atopic Eczema Reveals Reciprocal Expression of Inflammatory and Lipid Genes	Plos One
Atopic Eczema	SYTL2	Expression profiling by array	GPL7191	GSE12511	Global Expression Profiling in Atopic Eczema Reveals Reciprocal Expression of Inflammatory and Lipid Genes	Plos One
Atopic Eczema	FNBP1	Expression profiling by array	GPL7191	GSE12511	Global Expression Profiling in Atopic Eczema Reveals Reciprocal Expression of Inflammatory and Lipid Genes	Plos One
Atopic Eczema	CCR2	Expression profiling by array	GPL7191	GSE12511	Global Expression Profiling in Atopic Eczema Reveals Reciprocal Expression of Inflammatory and Lipid Genes	Plos One
Atopic Eczema	LTBP2	Expression profiling by array	GPL7191	GSE12511	Global Expression Profiling in Atopic Eczema Reveals Reciprocal Expression of Inflammatory and Lipid Genes	Plos One
Atopic Eczema	AA552851	Expression profiling by array	GPL7191	GSE12511	Global Expression Profiling in Atopic Eczema Reveals Reciprocal Expression of Inflammatory and Lipid Genes	Plos One
Atopic Eczema	RGS16	Expression profiling by array	GPL7191	GSE12511	Global Expression Profiling in Atopic Eczema Reveals Reciprocal Expression of Inflammatory and Lipid Genes	Plos One
Atopic Eczema	TBC1D2B	Expression profiling by array	GPL7191	GSE12511	Global Expression Profiling in Atopic Eczema Reveals Reciprocal Expression of Inflammatory and Lipid Genes	Plos One
Atopic Eczema	LAT	Expression profiling by array	GPL7191	GSE12511	Global Expression Profiling in Atopic Eczema Reveals Reciprocal Expression of Inflammatory and Lipid Genes	Plos One
Atopic Eczema	LSP1	Expression profiling by array	GPL7191	GSE12511	Global Expression Profiling in Atopic Eczema Reveals Reciprocal Expression of Inflammatory and Lipid Genes	Plos One
Atopic Eczema	C15ORF20	Expression profiling by array	GPL7191	GSE12511	Global Expression Profiling in Atopic Eczema Reveals Reciprocal Expression of Inflammatory and Lipid Genes	Plos One
Atopic Eczema	MGC9850	Expression profiling by array	GPL7191	GSE12511	Global Expression Profiling in Atopic Eczema Reveals Reciprocal Expression of Inflammatory and Lipid Genes	Plos One
Atopic Eczema	TTC21B	Expression profiling by array	GPL7191	GSE12511	Global Expression Profiling in Atopic Eczema Reveals Reciprocal Expression of Inflammatory and Lipid Genes	Plos One
Atopic Eczema	KIAA1245	Expression profiling by array	GPL7191	GSE12511	Global Expression Profiling in Atopic Eczema Reveals Reciprocal Expression of Inflammatory and Lipid Genes	Plos One
Atopic Eczema	IGFBP3	Expression profiling by array	GPL7191	GSE12511	Global Expression Profiling in Atopic Eczema Reveals Reciprocal Expression of Inflammatory and Lipid Genes	Plos One

Atopic Eczema	FGR	Expression profiling by array	GPL7191	GSE12511	Global Expression Profiling in Atopic Eczema Reveals Reciprocal Expression of Inflammatory and Lipid Genes	Plos One
Atopic Eczema	COMP	Expression profiling by array	GPL7191	GSE12511	Global Expression Profiling in Atopic Eczema Reveals Reciprocal Expression of Inflammatory and Lipid Genes	Plos One
Atopic Eczema	FCER2	Expression profiling by array	GPL7191	GSE12511	Global Expression Profiling in Atopic Eczema Reveals Reciprocal Expression of Inflammatory and Lipid Genes	Plos One
Atopic Eczema	UNQ338	Expression profiling by array	GPL7191	GSE12511	Global Expression Profiling in Atopic Eczema Reveals Reciprocal Expression of Inflammatory and Lipid Genes	Plos One
Atopic Eczema	C13ORF1	Expression profiling by array	GPL7191	GSE12511	Global Expression Profiling in Atopic Eczema Reveals Reciprocal Expression of Inflammatory and Lipid Genes	Plos One
Atopic Eczema	FCGBP	Expression profiling by array	GPL7191	GSE12511	Global Expression Profiling in Atopic Eczema Reveals Reciprocal Expression of Inflammatory and Lipid Genes	Plos One
Atopic Eczema	TMEM25	Expression profiling by array	GPL7191	GSE12511	Global Expression Profiling in Atopic Eczema Reveals Reciprocal Expression of Inflammatory and Lipid Genes	Plos One
Atopic Eczema	DYNLL1	Expression profiling by array	GPL7191	GSE12511	Global Expression Profiling in Atopic Eczema Reveals Reciprocal Expression of Inflammatory and Lipid Genes	Plos One
Atopic Eczema	TARDBP	Expression profiling by array	GPL7191	GSE12511	Global Expression Profiling in Atopic Eczema Reveals Reciprocal Expression of Inflammatory and Lipid Genes	Plos One
Atopic Eczema	UXT	Expression profiling by array	GPL7191	GSE12511	Global Expression Profiling in Atopic Eczema Reveals Reciprocal Expression of Inflammatory and Lipid Genes	Plos One
Atopic Eczema	C1QC	Expression profiling by array	GPL7191	GSE12511	Global Expression Profiling in Atopic Eczema Reveals Reciprocal Expression of Inflammatory and Lipid Genes	Plos One
Atopic Eczema	CYP4B1	Expression profiling by array	GPL7191	GSE12511	Global Expression Profiling in Atopic Eczema Reveals Reciprocal Expression of Inflammatory and Lipid Genes	Plos One
Atopic Eczema	ASGR2	Expression profiling by array	GPL7191	GSE12511	Global Expression Profiling in Atopic Eczema Reveals Reciprocal Expression of Inflammatory and Lipid Genes	Plos One
Atopic Eczema	CACNA1D	Expression profiling by array	GPL7191	GSE12511	Global Expression Profiling in Atopic Eczema Reveals Reciprocal Expression of Inflammatory and Lipid Genes	Plos One
Atopic Eczema	ACAD8	Expression profiling by array	GPL7191	GSE12511	Global Expression Profiling in Atopic Eczema Reveals Reciprocal Expression of Inflammatory and Lipid Genes	Plos One
Atopic Eczema	TYROBP	Expression profiling by array	GPL7191	GSE12511	Global Expression Profiling in Atopic Eczema Reveals Reciprocal Expression of Inflammatory and Lipid Genes	Plos One
Atopic Eczema	NMI	Expression profiling by array	GPL7191	GSE12511	Global Expression Profiling in Atopic Eczema Reveals Reciprocal Expression of Inflammatory and Lipid Genes	Plos One
Atopic Eczema	C1ORF45	Expression profiling by array	GPL7191	GSE12511	Global Expression Profiling in Atopic Eczema Reveals Reciprocal Expression of Inflammatory and Lipid Genes	Plos One
Atopic Eczema	BRP44L	Expression profiling by array	GPL7191	GSE12511	Global Expression Profiling in Atopic Eczema Reveals Reciprocal Expression of Inflammatory and Lipid Genes	Plos One
Atopic Eczema	HLA-DPB1	Expression profiling by array	GPL7191	GSE12511	Global Expression Profiling in Atopic Eczema Reveals Reciprocal Expression of Inflammatory and Lipid Genes	Plos One
Atopic Eczema	ALDH6A1	Expression profiling by array	GPL7191	GSE12511	Global Expression Profiling in Atopic Eczema Reveals Reciprocal Expression of Inflammatory and Lipid Genes	Plos One
Atopic Eczema	MAPK7	Expression profiling by array	GPL7191	GSE12511	Global Expression Profiling in Atopic Eczema Reveals Reciprocal Expression of Inflammatory and Lipid Genes	Plos One
Atopic Eczema	ZNF540	Expression profiling by array	GPL7191	GSE12511	Global Expression Profiling in Atopic Eczema Reveals Reciprocal Expression of Inflammatory and Lipid Genes	Plos One
Atopic Eczema	ABCF3	Expression profiling by array	GPL7191	GSE12511	Global Expression Profiling in Atopic Eczema Reveals Reciprocal Expression of Inflammatory and Lipid Genes	Plos One
Atopic Eczema	CRISPLD1	Expression profiling by array	GPL7191	GSE12511	Global Expression Profiling in Atopic Eczema Reveals Reciprocal Expression of Inflammatory and Lipid Genes	Plos One
Atopic Eczema	CFB	Expression profiling by array	GPL7191	GSE12511	Global Expression Profiling in Atopic Eczema Reveals Reciprocal Expression of Inflammatory and Lipid Genes	Plos One
Atopic Eczema	GLT25D1	Expression profiling by array	GPL7191	GSE12511	Global Expression Profiling in Atopic Eczema Reveals Reciprocal Expression of Inflammatory and Lipid Genes	Plos One
Atopic Eczema	SLC10A3	Expression profiling by array	GPL7191	GSE12511	Global Expression Profiling in Atopic Eczema Reveals Reciprocal Expression of Inflammatory and Lipid Genes	Plos One
Atopic Eczema	NUP62	Expression profiling by array	GPL7191	GSE12511	Global Expression Profiling in Atopic Eczema Reveals Reciprocal Expression of Inflammatory and Lipid Genes	Plos One
Atopic Eczema	CYFIP2	Expression profiling by array	GPL7191	GSE12511	Global Expression Profiling in Atopic Eczema Reveals Reciprocal Expression of Inflammatory and Lipid Genes	Plos One
Atopic Eczema	C17ORF27	Expression profiling by array	GPL7191	GSE12511	Global Expression Profiling in Atopic Eczema Reveals Reciprocal Expression of Inflammatory and Lipid Genes	Plos One
Atopic Eczema	TMPRSS2	Expression profiling by array	GPL7191	GSE12511	Global Expression Profiling in Atopic Eczema Reveals Reciprocal Expression of Inflammatory and Lipid Genes	Plos One
Atopic Eczema	AMMECR1	Expression profiling by array	GPL7191	GSE12511	Global Expression Profiling in Atopic Eczema Reveals Reciprocal Expression of Inflammatory and Lipid Genes	Plos One
Atopic Eczema	ATP1A2	Expression profiling by array	GPL7191	GSE12511	Global Expression Profiling in Atopic Eczema Reveals Reciprocal Expression of Inflammatory and Lipid Genes	Plos One
Atopic Eczema	DDA1	Expression profiling by array	GPL7191	GSE12511	Global Expression Profiling in Atopic Eczema Reveals Reciprocal Expression of Inflammatory and Lipid Genes	Plos One
Atopic Eczema	C18ORF49	Expression profiling by array	GPL7191	GSE12511	Global Expression Profiling in Atopic Eczema Reveals Reciprocal Expression of Inflammatory and Lipid Genes	Plos One
Atopic Eczema	ECHDC1	Expression profiling by array	GPL7191	GSE12511	Global Expression Profiling in Atopic Eczema Reveals Reciprocal Expression of Inflammatory and Lipid Genes	Plos One
Atopic Eczema	LOC145783	Expression profiling by array	GPL7191	GSE12511	Global Expression Profiling in Atopic Eczema Reveals Reciprocal Expression of Inflammatory and Lipid Genes	Plos One
Atopic Eczema	GPR143	Expression profiling by array	GPL7191	GSE12511	Global Expression Profiling in Atopic Eczema Reveals Reciprocal Expression of Inflammatory and Lipid Genes	Plos One
Atopic Eczema	SYNJ2	Expression profiling by array	GPL7191	GSE12511	Global Expression Profiling in Atopic Eczema Reveals Reciprocal Expression of Inflammatory and Lipid Genes	Plos One
Atopic Eczema	IGSF4B	Expression profiling by array	GPL7191	GSE12511	Global Expression Profiling in Atopic Eczema Reveals Reciprocal Expression of Inflammatory and Lipid Genes	Plos One
Atopic Eczema	FRAS1	Expression profiling by array	GPL7191	GSE12511	Global Expression Profiling in Atopic Eczema Reveals Reciprocal Expression of Inflammatory and Lipid Genes	Plos One
Atopic Eczema	IRAK1	Expression profiling by array	GPL7191	GSE12511	Global Expression Profiling in Atopic Eczema Reveals Reciprocal Expression of Inflammatory and Lipid Genes	Plos One
Atopic Eczema	HDDC2	Expression profiling by array	GPL7191	GSE12511	Global Expression Profiling in Atopic Eczema Reveals Reciprocal Expression of Inflammatory and Lipid Genes	Plos One
Atopic Eczema	YIF1A	Expression profiling by array	GPL7191	GSE12511	Global Expression Profiling in Atopic Eczema Reveals Reciprocal Expression of Inflammatory and Lipid Genes	Plos One
Atopic Eczema	FCHO1	Expression profiling by array	GPL7191	GSE12511	Global Expression Profiling in Atopic Eczema Reveals Reciprocal Expression of Inflammatory and Lipid Genes	Plos One
Atopic Eczema	AA971543	Expression profiling by array	GPL7191	GSE12511	Global Expression Profiling in Atopic Eczema Reveals Reciprocal Expression of Inflammatory and Lipid Genes	Plos One
Atopic Eczema	TMSB10	Expression profiling by array	GPL7191	GSE12511	Global Expression Profiling in Atopic Eczema Reveals Reciprocal Expression of Inflammatory and Lipid Genes	Plos One
Atopic Eczema	RGS20	Expression profiling by array	GPL7191	GSE12511	Global Expression Profiling in Atopic Eczema Reveals Reciprocal Expression of Inflammatory and Lipid Genes	Plos One
Atopic Eczema	NFXL1	Expression profiling by array	GPL7191	GSE12511	Global Expression Profiling in Atopic Eczema Reveals Reciprocal Expression of Inflammatory and Lipid Genes	Plos One
Atopic Eczema	CD36	Expression profiling by array	GPL7191	GSE12511	Global Expression Profiling in Atopic Eczema Reveals Reciprocal Expression of Inflammatory and Lipid Genes	Plos One
Atopic Eczema	GPD1	Expression profiling by array	GPL7191	GSE12511	Global Expression Profiling in Atopic Eczema Reveals Reciprocal Expression of Inflammatory and Lipid Genes	Plos One
Atopic Eczema	TNFRSF12A	Expression profiling by array	GPL7191	GSE12511	Global Expression Profiling in Atopic Eczema Reveals Reciprocal Expression of Inflammatory and Lipid Genes	Plos One
Atopic Eczema	TAP2	Expression profiling by array	GPL7191	GSE12511	Global Expression Profiling in Atopic Eczema Reveals Reciprocal Expression of Inflammatory and Lipid Genes	Plos One
Atopic Eczema	CALML5	Expression profiling by array	GPL7191	GSE12511	Global Expression Profiling in Atopic Eczema Reveals Reciprocal Expression of Inflammatory and Lipid Genes	Plos One
Atopic Eczema	PHC2	Expression profiling by array	GPL7191	GSE12511	Global Expression Profiling in Atopic Eczema Reveals Reciprocal Expression of Inflammatory and Lipid Genes	Plos One
Atopic Eczema	CCL2	Expression profiling by array	GPL7191	GSE12511	Global Expression Profiling in Atopic Eczema Reveals Reciprocal Expression of Inflammatory and Lipid Genes	Plos One
Atopic Eczema	INPP1	Expression profiling by array	GPL7191	GSE12511	Global Expression Profiling in Atopic Eczema Reveals Reciprocal Expression of Inflammatory and Lipid Genes	Plos One
Atopic Eczema	TBC1D22A	Expression profiling by array	GPL7191	GSE12511	Global Expression Profiling in Atopic Eczema Reveals Reciprocal Expression of Inflammatory and Lipid Genes	Plos One
Atopic Eczema	BLMH	Expression profiling by array	GPL7191	GSE12511	Global Expression Profiling in Atopic Eczema Reveals Reciprocal Expression of Inflammatory and Lipid Genes	Plos One
Atopic Eczema	CXORF40B	Expression profiling by array	GPL7191	GSE12511	Global Expression Profiling in Atopic Eczema Reveals Reciprocal Expression of Inflammatory and Lipid Genes	Plos One
Atopic Eczema	AKR1B10	Expression profiling by array	GPL7191	GSE12511	Global Expression Profiling in Atopic Eczema Reveals Reciprocal Expression of Inflammatory and Lipid Genes	Plos One

Atopic Eczema	HLA-A	Expression profiling by array	GPL7191	GSE12511	Global Expression Profiling in Atopic Eczema Reveals Reciprocal Expression of Inflammatory and Lipid Genes	Plos One
Atopic Eczema	AI344419	Expression profiling by array	GPL7191	GSE12511	Global Expression Profiling in Atopic Eczema Reveals Reciprocal Expression of Inflammatory and Lipid Genes	Plos One
Atopic Eczema	TNFRSF1A	Expression profiling by array	GPL7191	GSE12511	Global Expression Profiling in Atopic Eczema Reveals Reciprocal Expression of Inflammatory and Lipid Genes	Plos One
Atopic Eczema	AKAP13	Expression profiling by array	GPL7191	GSE12511	Global Expression Profiling in Atopic Eczema Reveals Reciprocal Expression of Inflammatory and Lipid Genes	Plos One
Atopic Eczema	DOPEY1	Expression profiling by array	GPL7191	GSE12511	Global Expression Profiling in Atopic Eczema Reveals Reciprocal Expression of Inflammatory and Lipid Genes	Plos One
Atopic Eczema	KIAA0232	Expression profiling by array	GPL7191	GSE12511	Global Expression Profiling in Atopic Eczema Reveals Reciprocal Expression of Inflammatory and Lipid Genes	Plos One
Atopic Eczema	DIRAS3	Expression profiling by array	GPL7191	GSE12511	Global Expression Profiling in Atopic Eczema Reveals Reciprocal Expression of Inflammatory and Lipid Genes	Plos One
Atopic Eczema	TPP1	Expression profiling by array	GPL7191	GSE12511	Global Expression Profiling in Atopic Eczema Reveals Reciprocal Expression of Inflammatory and Lipid Genes	Plos One
Atopic Eczema	DOPEY2	Expression profiling by array	GPL7191	GSE12511	Global Expression Profiling in Atopic Eczema Reveals Reciprocal Expression of Inflammatory and Lipid Genes	Plos One
Atopic Eczema	MAP1A	Expression profiling by array	GPL7191	GSE12511	Global Expression Profiling in Atopic Eczema Reveals Reciprocal Expression of Inflammatory and Lipid Genes	Plos One
Atopic Eczema	NTHL1	Expression profiling by array	GPL7191	GSE12511	Global Expression Profiling in Atopic Eczema Reveals Reciprocal Expression of Inflammatory and Lipid Genes	Plos One
Atopic Eczema	SLAMF1	Expression profiling by array	GPL7191	GSE12511	Global Expression Profiling in Atopic Eczema Reveals Reciprocal Expression of Inflammatory and Lipid Genes	Plos One
Atopic Eczema	WDFY4	Expression profiling by array	GPL7191	GSE12511	Global Expression Profiling in Atopic Eczema Reveals Reciprocal Expression of Inflammatory and Lipid Genes	Plos One
Atopic Eczema	TEAD1	Expression profiling by array	GPL7191	GSE12511	Global Expression Profiling in Atopic Eczema Reveals Reciprocal Expression of Inflammatory and Lipid Genes	Plos One
Atopic Eczema	CHD3	Expression profiling by array	GPL7191	GSE12511	Global Expression Profiling in Atopic Eczema Reveals Reciprocal Expression of Inflammatory and Lipid Genes	Plos One
Atopic Eczema	DGAT1	Expression profiling by array	GPL7191	GSE12511	Global Expression Profiling in Atopic Eczema Reveals Reciprocal Expression of Inflammatory and Lipid Genes	Plos One
Atopic Eczema	PAG1	Expression profiling by array	GPL7191	GSE12511	Global Expression Profiling in Atopic Eczema Reveals Reciprocal Expression of Inflammatory and Lipid Genes	Plos One
Atopic Eczema	PTP4A3	Expression profiling by array	GPL7191	GSE12511	Global Expression Profiling in Atopic Eczema Reveals Reciprocal Expression of Inflammatory and Lipid Genes	Plos One
Atopic Eczema	ELL	Expression profiling by array	GPL7191	GSE12511	Global Expression Profiling in Atopic Eczema Reveals Reciprocal Expression of Inflammatory and Lipid Genes	Plos One
Atopic Eczema	N45230	Expression profiling by array	GPL7191	GSE12511	Global Expression Profiling in Atopic Eczema Reveals Reciprocal Expression of Inflammatory and Lipid Genes	Plos One
Atopic Eczema	SUSD1	Expression profiling by array	GPL7191	GSE12511	Global Expression Profiling in Atopic Eczema Reveals Reciprocal Expression of Inflammatory and Lipid Genes	Plos One
Atopic Eczema	CD163	Expression profiling by array	GPL7191	GSE12511	Global Expression Profiling in Atopic Eczema Reveals Reciprocal Expression of Inflammatory and Lipid Genes	Plos One
Atopic Eczema	OPRS1	Expression profiling by array	GPL7191	GSE12511	Global Expression Profiling in Atopic Eczema Reveals Reciprocal Expression of Inflammatory and Lipid Genes	Plos One
Atopic Eczema	GBP1	Expression profiling by array	GPL7191	GSE12511	Global Expression Profiling in Atopic Eczema Reveals Reciprocal Expression of Inflammatory and Lipid Genes	Plos One
Atopic Eczema	ROM1	Expression profiling by array	GPL7191	GSE12511	Global Expression Profiling in Atopic Eczema Reveals Reciprocal Expression of Inflammatory and Lipid Genes	Plos One
Atopic Eczema	DUSP22	Expression profiling by array	GPL7191	GSE12511	Global Expression Profiling in Atopic Eczema Reveals Reciprocal Expression of Inflammatory and Lipid Genes	Plos One
Atopic Eczema	CGI-38	Expression profiling by array	GPL7191	GSE12511	Global Expression Profiling in Atopic Eczema Reveals Reciprocal Expression of Inflammatory and Lipid Genes	Plos One
Atopic Eczema	MEGF10	Expression profiling by array	GPL7191	GSE12511	Global Expression Profiling in Atopic Eczema Reveals Reciprocal Expression of Inflammatory and Lipid Genes	Plos One
Atopic Eczema	SEC13L1	Expression profiling by array	GPL7191	GSE12511	Global Expression Profiling in Atopic Eczema Reveals Reciprocal Expression of Inflammatory and Lipid Genes	Plos One
Atopic Eczema	CTLA4	Expression profiling by array	GPL7191	GSE12511	Global Expression Profiling in Atopic Eczema Reveals Reciprocal Expression of Inflammatory and Lipid Genes	Plos One
Atopic Eczema	FAM59B	Expression profiling by array	GPL7191	GSE12511	Global Expression Profiling in Atopic Eczema Reveals Reciprocal Expression of Inflammatory and Lipid Genes	Plos One
Atopic Eczema	COL15A1	Expression profiling by array	GPL7191	GSE12511	Global Expression Profiling in Atopic Eczema Reveals Reciprocal Expression of Inflammatory and Lipid Genes	Plos One
Atopic Eczema	EML1	Expression profiling by array	GPL7191	GSE12511	Global Expression Profiling in Atopic Eczema Reveals Reciprocal Expression of Inflammatory and Lipid Genes	Plos One
Atopic Eczema	RNF20	Expression profiling by array	GPL7191	GSE12511	Global Expression Profiling in Atopic Eczema Reveals Reciprocal Expression of Inflammatory and Lipid Genes	Plos One
Atopic Eczema	NFKBIA	Expression profiling by array	GPL7191	GSE12511	Global Expression Profiling in Atopic Eczema Reveals Reciprocal Expression of Inflammatory and Lipid Genes	Plos One
Atopic Eczema	UBE2D4	Expression profiling by array	GPL7191	GSE12511	Global Expression Profiling in Atopic Eczema Reveals Reciprocal Expression of Inflammatory and Lipid Genes	Plos One
Atopic Eczema	ANTXR2	Expression profiling by array	GPL7191	GSE12511	Global Expression Profiling in Atopic Eczema Reveals Reciprocal Expression of Inflammatory and Lipid Genes	Plos One
Atopic Eczema	C1ORF187	Expression profiling by array	GPL7191	GSE12511	Global Expression Profiling in Atopic Eczema Reveals Reciprocal Expression of Inflammatory and Lipid Genes	Plos One
Atopic Eczema	PANX1	Expression profiling by array	GPL7191	GSE12511	Global Expression Profiling in Atopic Eczema Reveals Reciprocal Expression of Inflammatory and Lipid Genes	Plos One
Atopic Eczema	EVI2B	Expression profiling by array	GPL7191	GSE12511	Global Expression Profiling in Atopic Eczema Reveals Reciprocal Expression of Inflammatory and Lipid Genes	Plos One
Atopic Eczema	FAM50A	Expression profiling by array	GPL7191	GSE12511	Global Expression Profiling in Atopic Eczema Reveals Reciprocal Expression of Inflammatory and Lipid Genes	Plos One
Atopic Eczema	CAMK2N1	Expression profiling by array	GPL7191	GSE12511	Global Expression Profiling in Atopic Eczema Reveals Reciprocal Expression of Inflammatory and Lipid Genes	Plos One
Atopic Eczema	FCER1G	Expression profiling by array	GPL7191	GSE12511	Global Expression Profiling in Atopic Eczema Reveals Reciprocal Expression of Inflammatory and Lipid Genes	Plos One
Atopic Eczema	GPX4	Expression profiling by array	GPL7191	GSE12511	Global Expression Profiling in Atopic Eczema Reveals Reciprocal Expression of Inflammatory and Lipid Genes	Plos One
Atopic Eczema	CEACAM6	Expression profiling by array	GPL7191	GSE12511	Global Expression Profiling in Atopic Eczema Reveals Reciprocal Expression of Inflammatory and Lipid Genes	Plos One
Atopic Eczema	PRDM10	Expression profiling by array	GPL7191	GSE12511	Global Expression Profiling in Atopic Eczema Reveals Reciprocal Expression of Inflammatory and Lipid Genes	Plos One
Atopic Eczema	AA702728	Expression profiling by array	GPL7191	GSE12511	Global Expression Profiling in Atopic Eczema Reveals Reciprocal Expression of Inflammatory and Lipid Genes	Plos One
Atopic Eczema	GBA	Expression profiling by array	GPL7191	GSE12511	Global Expression Profiling in Atopic Eczema Reveals Reciprocal Expression of Inflammatory and Lipid Genes	Plos One
Atopic Eczema	NR2C2	Expression profiling by array	GPL7191	GSE12511	Global Expression Profiling in Atopic Eczema Reveals Reciprocal Expression of Inflammatory and Lipid Genes	Plos One
Atopic Eczema	FAM3D	Expression profiling by array	GPL7191	GSE12511	Global Expression Profiling in Atopic Eczema Reveals Reciprocal Expression of Inflammatory and Lipid Genes	Plos One
Atopic Eczema	PHC3	Expression profiling by array	GPL7191	GSE12511	Global Expression Profiling in Atopic Eczema Reveals Reciprocal Expression of Inflammatory and Lipid Genes	Plos One
Atopic Eczema	UGCG	Expression profiling by array	GPL7191	GSE12511	Global Expression Profiling in Atopic Eczema Reveals Reciprocal Expression of Inflammatory and Lipid Genes	Plos One
Atopic Eczema	PLXNA2	Expression profiling by array	GPL7191	GSE12511	Global Expression Profiling in Atopic Eczema Reveals Reciprocal Expression of Inflammatory and Lipid Genes	Plos One
Atopic Eczema	EDG1	Expression profiling by array	GPL7191	GSE12511	Global Expression Profiling in Atopic Eczema Reveals Reciprocal Expression of Inflammatory and Lipid Genes	Plos One
Atopic Eczema	RGPD5	Expression profiling by array	GPL7191	GSE12511	Global Expression Profiling in Atopic Eczema Reveals Reciprocal Expression of Inflammatory and Lipid Genes	Plos One
Atopic Eczema	FLJ20273	Expression profiling by array	GPL7191	GSE12511	Global Expression Profiling in Atopic Eczema Reveals Reciprocal Expression of Inflammatory and Lipid Genes	Plos One
Atopic Eczema	ZC3H14	Expression profiling by array	GPL7191	GSE12511	Global Expression Profiling in Atopic Eczema Reveals Reciprocal Expression of Inflammatory and Lipid Genes	Plos One
Atopic Eczema	RIC8A	Expression profiling by array	GPL7191	GSE12511	Global Expression Profiling in Atopic Eczema Reveals Reciprocal Expression of Inflammatory and Lipid Genes	Plos One
Atopic Eczema	RAC1	Expression profiling by array	GPL7191	GSE12511	Global Expression Profiling in Atopic Eczema Reveals Reciprocal Expression of Inflammatory and Lipid Genes	Plos One
Atopic Eczema	FAM84A	Expression profiling by array	GPL7191	GSE12511	Global Expression Profiling in Atopic Eczema Reveals Reciprocal Expression of Inflammatory and Lipid Genes	Plos One
Atopic Eczema	ALDH3B1	Expression profiling by array	GPL7191	GSE12511	Global Expression Profiling in Atopic Eczema Reveals Reciprocal Expression of Inflammatory and Lipid Genes	Plos One
Atopic Eczema	FOXI1	Expression profiling by array	GPL7191	GSE12511	Global Expression Profiling in Atopic Eczema Reveals Reciprocal Expression of Inflammatory and Lipid Genes	Plos One

Atopic Eczema	IER2	Expression profiling by array	GPL7191	GSE12511	Global Expression Profiling in Atopic Eczema Reveals Reciprocal Expression of Inflammatory and Lipid Genes	Plos One
Atopic Eczema	DDR1	Expression profiling by array	GPL7191	GSE12511	Global Expression Profiling in Atopic Eczema Reveals Reciprocal Expression of Inflammatory and Lipid Genes	Plos One
Atopic Eczema	B4GALT4	Expression profiling by array	GPL7191	GSE12511	Global Expression Profiling in Atopic Eczema Reveals Reciprocal Expression of Inflammatory and Lipid Genes	Plos One
Atopic Eczema	APBA3	Expression profiling by array	GPL7191	GSE12511	Global Expression Profiling in Atopic Eczema Reveals Reciprocal Expression of Inflammatory and Lipid Genes	Plos One
Atopic Eczema	SMA3	Expression profiling by array	GPL7191	GSE12511	Global Expression Profiling in Atopic Eczema Reveals Reciprocal Expression of Inflammatory and Lipid Genes	Plos One
Atopic Eczema	TRPC3	Expression profiling by array	GPL7191	GSE12511	Global Expression Profiling in Atopic Eczema Reveals Reciprocal Expression of Inflammatory and Lipid Genes	Plos One
Atopic Eczema	TERF1	Expression profiling by array	GPL7191	GSE12511	Global Expression Profiling in Atopic Eczema Reveals Reciprocal Expression of Inflammatory and Lipid Genes	Plos One
Atopic Eczema	TXLNA	Expression profiling by array	GPL7191	GSE12511	Global Expression Profiling in Atopic Eczema Reveals Reciprocal Expression of Inflammatory and Lipid Genes	Plos One
Atopic Eczema	TCEA2	Expression profiling by array	GPL7191	GSE12511	Global Expression Profiling in Atopic Eczema Reveals Reciprocal Expression of Inflammatory and Lipid Genes	Plos One
Atopic Eczema	PXDN	Expression profiling by array	GPL7191	GSE12511	Global Expression Profiling in Atopic Eczema Reveals Reciprocal Expression of Inflammatory and Lipid Genes	Plos One
Atopic Eczema	TFCP2L1	Expression profiling by array	GPL7191	GSE12511	Global Expression Profiling in Atopic Eczema Reveals Reciprocal Expression of Inflammatory and Lipid Genes	Plos One
Atopic Eczema	SBEM	Expression profiling by array	GPL7191	GSE12511	Global Expression Profiling in Atopic Eczema Reveals Reciprocal Expression of Inflammatory and Lipid Genes	Plos One
Atopic Eczema	ZNF595	Expression profiling by array	GPL7191	GSE12511	Global Expression Profiling in Atopic Eczema Reveals Reciprocal Expression of Inflammatory and Lipid Genes	Plos One
Atopic Eczema	TOMM40	Expression profiling by array	GPL7191	GSE12511	Global Expression Profiling in Atopic Eczema Reveals Reciprocal Expression of Inflammatory and Lipid Genes	Plos One
Atopic Eczema	SAMSN1	Expression profiling by array	GPL7191	GSE12511	Global Expression Profiling in Atopic Eczema Reveals Reciprocal Expression of Inflammatory and Lipid Genes	Plos One
Atopic Eczema	HIPK2	Expression profiling by array	GPL7191	GSE12511	Global Expression Profiling in Atopic Eczema Reveals Reciprocal Expression of Inflammatory and Lipid Genes	Plos One
Atopic Eczema	MAN2A1	Expression profiling by array	GPL7191	GSE12511	Global Expression Profiling in Atopic Eczema Reveals Reciprocal Expression of Inflammatory and Lipid Genes	Plos One
Atopic Eczema	CTA-221G9.4	Expression profiling by array	GPL7191	GSE12511	Global Expression Profiling in Atopic Eczema Reveals Reciprocal Expression of Inflammatory and Lipid Genes	Plos One
Atopic Eczema	FIBP	Expression profiling by array	GPL7191	GSE12511	Global Expression Profiling in Atopic Eczema Reveals Reciprocal Expression of Inflammatory and Lipid Genes	Plos One
Atopic Eczema	KIAA1841	Expression profiling by array	GPL7191	GSE12511	Global Expression Profiling in Atopic Eczema Reveals Reciprocal Expression of Inflammatory and Lipid Genes	Plos One
Atopic Eczema	KIAA1409	Expression profiling by array	GPL7191	GSE12511	Global Expression Profiling in Atopic Eczema Reveals Reciprocal Expression of Inflammatory and Lipid Genes	Plos One
Atopic Eczema	SLC24A6	Expression profiling by array	GPL7191	GSE12511	Global Expression Profiling in Atopic Eczema Reveals Reciprocal Expression of Inflammatory and Lipid Genes	Plos One
Atopic Eczema	EIF4E2	Expression profiling by array	GPL7191	GSE12511	Global Expression Profiling in Atopic Eczema Reveals Reciprocal Expression of Inflammatory and Lipid Genes	Plos One
Atopic Eczema	PSCDBP	Expression profiling by array	GPL7191	GSE12511	Global Expression Profiling in Atopic Eczema Reveals Reciprocal Expression of Inflammatory and Lipid Genes	Plos One
Atopic Eczema	TMSB4X	Expression profiling by array	GPL7191	GSE12511	Global Expression Profiling in Atopic Eczema Reveals Reciprocal Expression of Inflammatory and Lipid Genes	Plos One
Atopic Eczema	CPEB4	Expression profiling by array	GPL7191	GSE12511	Global Expression Profiling in Atopic Eczema Reveals Reciprocal Expression of Inflammatory and Lipid Genes	Plos One
Atopic Eczema	EIF2A	Expression profiling by array	GPL7191	GSE12511	Global Expression Profiling in Atopic Eczema Reveals Reciprocal Expression of Inflammatory and Lipid Genes	Plos One
Atopic Eczema	CD55	Expression profiling by array	GPL7191	GSE12511	Global Expression Profiling in Atopic Eczema Reveals Reciprocal Expression of Inflammatory and Lipid Genes	Plos One
Atopic Eczema	MCL1	Expression profiling by array	GPL7191	GSE12511	Global Expression Profiling in Atopic Eczema Reveals Reciprocal Expression of Inflammatory and Lipid Genes	Plos One
Atopic Eczema	AA918191	Expression profiling by array	GPL7191	GSE12511	Global Expression Profiling in Atopic Eczema Reveals Reciprocal Expression of Inflammatory and Lipid Genes	Plos One
Atopic Eczema	C10ORF76	Expression profiling by array	GPL7191	GSE12511	Global Expression Profiling in Atopic Eczema Reveals Reciprocal Expression of Inflammatory and Lipid Genes	Plos One
Atopic Eczema	PCYT1B	Expression profiling by array	GPL7191	GSE12511	Global Expression Profiling in Atopic Eczema Reveals Reciprocal Expression of Inflammatory and Lipid Genes	Plos One
Atopic Eczema	KIAA1881	Expression profiling by array	GPL7191	GSE12511	Global Expression Profiling in Atopic Eczema Reveals Reciprocal Expression of Inflammatory and Lipid Genes	Plos One
Atopic Eczema	GALNT12	Expression profiling by array	GPL7191	GSE12511	Global Expression Profiling in Atopic Eczema Reveals Reciprocal Expression of Inflammatory and Lipid Genes	Plos One
Atopic Eczema	HDLBP	Expression profiling by array	GPL7191	GSE12511	Global Expression Profiling in Atopic Eczema Reveals Reciprocal Expression of Inflammatory and Lipid Genes	Plos One
Atopic Eczema	SHKBP1	Expression profiling by array	GPL7191	GSE12511	Global Expression Profiling in Atopic Eczema Reveals Reciprocal Expression of Inflammatory and Lipid Genes	Plos One
Atopic Eczema	AI002301	Expression profiling by array	GPL7191	GSE12511	Global Expression Profiling in Atopic Eczema Reveals Reciprocal Expression of Inflammatory and Lipid Genes	Plos One
Atopic Eczema	BHLHB3	Expression profiling by array	GPL7191	GSE12511	Global Expression Profiling in Atopic Eczema Reveals Reciprocal Expression of Inflammatory and Lipid Genes	Plos One
Atopic Eczema	CPD	Expression profiling by array	GPL7191	GSE12511	Global Expression Profiling in Atopic Eczema Reveals Reciprocal Expression of Inflammatory and Lipid Genes	Plos One
Atopic Eczema	CENTG1	Expression profiling by array	GPL7191	GSE12511	Global Expression Profiling in Atopic Eczema Reveals Reciprocal Expression of Inflammatory and Lipid Genes	Plos One
Atopic Eczema	LAIR1	Expression profiling by array	GPL7191	GSE12511	Global Expression Profiling in Atopic Eczema Reveals Reciprocal Expression of Inflammatory and Lipid Genes	Plos One
Atopic Eczema	MGC4728	Expression profiling by array	GPL7191	GSE12511	Global Expression Profiling in Atopic Eczema Reveals Reciprocal Expression of Inflammatory and Lipid Genes	Plos One
Atopic Eczema	ZNF185	Expression profiling by array	GPL7191	GSE12511	Global Expression Profiling in Atopic Eczema Reveals Reciprocal Expression of Inflammatory and Lipid Genes	Plos One
Atopic Eczema	F13A1	Expression profiling by array	GPL7191	GSE12511	Global Expression Profiling in Atopic Eczema Reveals Reciprocal Expression of Inflammatory and Lipid Genes	Plos One
Atopic Eczema	FAP	Expression profiling by array	GPL7191	GSE12511	Global Expression Profiling in Atopic Eczema Reveals Reciprocal Expression of Inflammatory and Lipid Genes	Plos One
Atopic Eczema	C1ORF158	Expression profiling by array	GPL7191	GSE12511	Global Expression Profiling in Atopic Eczema Reveals Reciprocal Expression of Inflammatory and Lipid Genes	Plos One
Atopic Eczema	AA570170	Expression profiling by array	GPL7191	GSE12511	Global Expression Profiling in Atopic Eczema Reveals Reciprocal Expression of Inflammatory and Lipid Genes	Plos One
Atopic Eczema	NEDD4L	Expression profiling by array	GPL7191	GSE12511	Global Expression Profiling in Atopic Eczema Reveals Reciprocal Expression of Inflammatory and Lipid Genes	Plos One
Atopic Eczema	MAPK6	Expression profiling by array	GPL7191	GSE12511	Global Expression Profiling in Atopic Eczema Reveals Reciprocal Expression of Inflammatory and Lipid Genes	Plos One
Atopic Eczema	EGF	Expression profiling by array	GPL7191	GSE12511	Global Expression Profiling in Atopic Eczema Reveals Reciprocal Expression of Inflammatory and Lipid Genes	Plos One
Atopic Eczema	DNMT1	Expression profiling by array	GPL7191	GSE12511	Global Expression Profiling in Atopic Eczema Reveals Reciprocal Expression of Inflammatory and Lipid Genes	Plos One
Atopic Eczema	OVOL2	Expression profiling by array	GPL7191	GSE12511	Global Expression Profiling in Atopic Eczema Reveals Reciprocal Expression of Inflammatory and Lipid Genes	Plos One
Atopic Eczema	BAT2D1	Expression profiling by array	GPL7191	GSE12511	Global Expression Profiling in Atopic Eczema Reveals Reciprocal Expression of Inflammatory and Lipid Genes	Plos One
Atopic Eczema	FARSLB	Expression profiling by array	GPL7191	GSE12511	Global Expression Profiling in Atopic Eczema Reveals Reciprocal Expression of Inflammatory and Lipid Genes	Plos One
Atopic Eczema	PREI3	Expression profiling by array	GPL7191	GSE12511	Global Expression Profiling in Atopic Eczema Reveals Reciprocal Expression of Inflammatory and Lipid Genes	Plos One
Atopic Eczema	PRPF31	Expression profiling by array	GPL7191	GSE12511	Global Expression Profiling in Atopic Eczema Reveals Reciprocal Expression of Inflammatory and Lipid Genes	Plos One
Atopic Eczema	PSME2	Expression profiling by array	GPL7191	GSE12511	Global Expression Profiling in Atopic Eczema Reveals Reciprocal Expression of Inflammatory and Lipid Genes	Plos One
Atopic Eczema	FLJ21963	Expression profiling by array	GPL7191	GSE12511	Global Expression Profiling in Atopic Eczema Reveals Reciprocal Expression of Inflammatory and Lipid Genes	Plos One
Atopic Eczema	ZNF672	Expression profiling by array	GPL7191	GSE12511	Global Expression Profiling in Atopic Eczema Reveals Reciprocal Expression of Inflammatory and Lipid Genes	Plos One
Atopic Eczema	TMC6	Expression profiling by array	GPL7191	GSE12511	Global Expression Profiling in Atopic Eczema Reveals Reciprocal Expression of Inflammatory and Lipid Genes	Plos One
Atopic Eczema	H2AFV	Expression profiling by array	GPL7191	GSE12511	Global Expression Profiling in Atopic Eczema Reveals Reciprocal Expression of Inflammatory and Lipid Genes	Plos One
Atopic Eczema	CCM2	Expression profiling by array	GPL7191	GSE12511	Global Expression Profiling in Atopic Eczema Reveals Reciprocal Expression of Inflammatory and Lipid Genes	Plos One

Atopic Eczema	CHMP7	Expression profiling by array	GPL7191	GSE12511	Global Expression Profiling in Atopic Eczema Reveals Reciprocal Expression of Inflammatory and Lipid Genes	Plos One
Atopic Eczema	CCDC47	Expression profiling by array	GPL7191	GSE12511	Global Expression Profiling in Atopic Eczema Reveals Reciprocal Expression of Inflammatory and Lipid Genes	Plos One
Atopic Eczema	DIXDC1	Expression profiling by array	GPL7191	GSE12511	Global Expression Profiling in Atopic Eczema Reveals Reciprocal Expression of Inflammatory and Lipid Genes	Plos One
Atopic Eczema	SNX2	Expression profiling by array	GPL7191	GSE12511	Global Expression Profiling in Atopic Eczema Reveals Reciprocal Expression of Inflammatory and Lipid Genes	Plos One
Atopic Eczema	PANK1	Expression profiling by array	GPL7191	GSE12511	Global Expression Profiling in Atopic Eczema Reveals Reciprocal Expression of Inflammatory and Lipid Genes	Plos One
Atopic Eczema	TRAM2	Expression profiling by array	GPL7191	GSE12511	Global Expression Profiling in Atopic Eczema Reveals Reciprocal Expression of Inflammatory and Lipid Genes	Plos One
Atopic Eczema	MRPL37	Expression profiling by array	GPL7191	GSE12511	Global Expression Profiling in Atopic Eczema Reveals Reciprocal Expression of Inflammatory and Lipid Genes	Plos One
Atopic Eczema	PDGFRA	Expression profiling by array	GPL7191	GSE12511	Global Expression Profiling in Atopic Eczema Reveals Reciprocal Expression of Inflammatory and Lipid Genes	Plos One
Atopic Eczema	PIK3C2B	Expression profiling by array	GPL7191	GSE12511	Global Expression Profiling in Atopic Eczema Reveals Reciprocal Expression of Inflammatory and Lipid Genes	Plos One
Atopic Eczema	AA825954	Expression profiling by array	GPL7191	GSE12511	Global Expression Profiling in Atopic Eczema Reveals Reciprocal Expression of Inflammatory and Lipid Genes	Plos One
Atopic Eczema	USP4	Expression profiling by array	GPL7191	GSE12511	Global Expression Profiling in Atopic Eczema Reveals Reciprocal Expression of Inflammatory and Lipid Genes	Plos One
Atopic Eczema	LGALS3BP	Expression profiling by array	GPL7191	GSE12511	Global Expression Profiling in Atopic Eczema Reveals Reciprocal Expression of Inflammatory and Lipid Genes	Plos One
Atopic Eczema	AZGP1	Expression profiling by array	GPL7191	GSE12511	Global Expression Profiling in Atopic Eczema Reveals Reciprocal Expression of Inflammatory and Lipid Genes	Plos One
Atopic Eczema	JAM3	Expression profiling by array	GPL7191	GSE12511	Global Expression Profiling in Atopic Eczema Reveals Reciprocal Expression of Inflammatory and Lipid Genes	Plos One
Atopic Eczema	CD2	Expression profiling by array	GPL7191	GSE12511	Global Expression Profiling in Atopic Eczema Reveals Reciprocal Expression of Inflammatory and Lipid Genes	Plos One
Atopic Eczema	RPA2	Expression profiling by array	GPL7191	GSE12511	Global Expression Profiling in Atopic Eczema Reveals Reciprocal Expression of Inflammatory and Lipid Genes	Plos One
Atopic Eczema	CDKL1	Expression profiling by array	GPL7191	GSE12511	Global Expression Profiling in Atopic Eczema Reveals Reciprocal Expression of Inflammatory and Lipid Genes	Plos One
Atopic Eczema	PTK2	Expression profiling by array	GPL7191	GSE12511	Global Expression Profiling in Atopic Eczema Reveals Reciprocal Expression of Inflammatory and Lipid Genes	Plos One
Atopic Eczema	SPIN1	Expression profiling by array	GPL7191	GSE12511	Global Expression Profiling in Atopic Eczema Reveals Reciprocal Expression of Inflammatory and Lipid Genes	Plos One
Atopic Eczema	C11ORF24	Expression profiling by array	GPL7191	GSE12511	Global Expression Profiling in Atopic Eczema Reveals Reciprocal Expression of Inflammatory and Lipid Genes	Plos One
Atopic Eczema	TIAM2	Expression profiling by array	GPL7191	GSE12511	Global Expression Profiling in Atopic Eczema Reveals Reciprocal Expression of Inflammatory and Lipid Genes	Plos One
Atopic Eczema	AA922508	Expression profiling by array	GPL7191	GSE12511	Global Expression Profiling in Atopic Eczema Reveals Reciprocal Expression of Inflammatory and Lipid Genes	Plos One
Atopic Eczema	ADAMTS6	Expression profiling by array	GPL7191	GSE12511	Global Expression Profiling in Atopic Eczema Reveals Reciprocal Expression of Inflammatory and Lipid Genes	Plos One
Atopic Eczema	DMGDH	Expression profiling by array	GPL7191	GSE12511	Global Expression Profiling in Atopic Eczema Reveals Reciprocal Expression of Inflammatory and Lipid Genes	Plos One
Atopic Eczema	SSBP4	Expression profiling by array	GPL7191	GSE12511	Global Expression Profiling in Atopic Eczema Reveals Reciprocal Expression of Inflammatory and Lipid Genes	Plos One
Atopic Eczema	SQRDL	Expression profiling by array	GPL7191	GSE12511	Global Expression Profiling in Atopic Eczema Reveals Reciprocal Expression of Inflammatory and Lipid Genes	Plos One
Atopic Eczema	ACLY	Expression profiling by array	GPL7191	GSE12511	Global Expression Profiling in Atopic Eczema Reveals Reciprocal Expression of Inflammatory and Lipid Genes	Plos One
Atopic Eczema	AA581501	Expression profiling by array	GPL7191	GSE12511	Global Expression Profiling in Atopic Eczema Reveals Reciprocal Expression of Inflammatory and Lipid Genes	Plos One
Atopic Eczema	SHMT2	Expression profiling by array	GPL7191	GSE12511	Global Expression Profiling in Atopic Eczema Reveals Reciprocal Expression of Inflammatory and Lipid Genes	Plos One
Atopic Eczema	PPAP2B	Expression profiling by array	GPL7191	GSE12511	Global Expression Profiling in Atopic Eczema Reveals Reciprocal Expression of Inflammatory and Lipid Genes	Plos One
Atopic Eczema	EBI3	Expression profiling by array	GPL7191	GSE12511	Global Expression Profiling in Atopic Eczema Reveals Reciprocal Expression of Inflammatory and Lipid Genes	Plos One
Atopic Eczema	HDHD1A	Expression profiling by array	GPL7191	GSE12511	Global Expression Profiling in Atopic Eczema Reveals Reciprocal Expression of Inflammatory and Lipid Genes	Plos One
Atopic Eczema	C20ORF121	Expression profiling by array	GPL7191	GSE12511	Global Expression Profiling in Atopic Eczema Reveals Reciprocal Expression of Inflammatory and Lipid Genes	Plos One
Atopic Eczema	TRAF1	Expression profiling by array	GPL7191	GSE12511	Global Expression Profiling in Atopic Eczema Reveals Reciprocal Expression of Inflammatory and Lipid Genes	Plos One
Atopic Eczema	LOC283130	Expression profiling by array	GPL7191	GSE12511	Global Expression Profiling in Atopic Eczema Reveals Reciprocal Expression of Inflammatory and Lipid Genes	Plos One
Atopic Eczema	ITPR1	Expression profiling by array	GPL7191	GSE12511	Global Expression Profiling in Atopic Eczema Reveals Reciprocal Expression of Inflammatory and Lipid Genes	Plos One
Atopic Eczema	AI038595	Expression profiling by array	GPL7191	GSE12511	Global Expression Profiling in Atopic Eczema Reveals Reciprocal Expression of Inflammatory and Lipid Genes	Plos One
Atopic Eczema	ZBTB25	Expression profiling by array	GPL7191	GSE12511	Global Expression Profiling in Atopic Eczema Reveals Reciprocal Expression of Inflammatory and Lipid Genes	Plos One
Atopic Eczema	POLR2E	Expression profiling by array	GPL7191	GSE12511	Global Expression Profiling in Atopic Eczema Reveals Reciprocal Expression of Inflammatory and Lipid Genes	Plos One
Atopic Eczema	PSMD9	Expression profiling by array	GPL7191	GSE12511	Global Expression Profiling in Atopic Eczema Reveals Reciprocal Expression of Inflammatory and Lipid Genes	Plos One
Atopic Eczema	AI097617	Expression profiling by array	GPL7191	GSE12511	Global Expression Profiling in Atopic Eczema Reveals Reciprocal Expression of Inflammatory and Lipid Genes	Plos One
Atopic Eczema	CAMK1D	Expression profiling by array	GPL7191	GSE12511	Global Expression Profiling in Atopic Eczema Reveals Reciprocal Expression of Inflammatory and Lipid Genes	Plos One
Atopic Eczema	ANKRD39	Expression profiling by array	GPL7191	GSE12511	Global Expression Profiling in Atopic Eczema Reveals Reciprocal Expression of Inflammatory and Lipid Genes	Plos One
Atopic Eczema	RALA	Expression profiling by array	GPL7191	GSE12511	Global Expression Profiling in Atopic Eczema Reveals Reciprocal Expression of Inflammatory and Lipid Genes	Plos One
Atopic Eczema	MAP2K2	Expression profiling by array	GPL7191	GSE12511	Global Expression Profiling in Atopic Eczema Reveals Reciprocal Expression of Inflammatory and Lipid Genes	Plos One
Atopic Eczema	AA503283	Expression profiling by array	GPL7191	GSE12511	Global Expression Profiling in Atopic Eczema Reveals Reciprocal Expression of Inflammatory and Lipid Genes	Plos One
Atopic Eczema	TYRP1	Expression profiling by array	GPL7191	GSE12511	Global Expression Profiling in Atopic Eczema Reveals Reciprocal Expression of Inflammatory and Lipid Genes	Plos One
Atopic Eczema	DBT	Expression profiling by array	GPL7191	GSE12511	Global Expression Profiling in Atopic Eczema Reveals Reciprocal Expression of Inflammatory and Lipid Genes	Plos One
Atopic Eczema	CD8A	Expression profiling by array	GPL7191	GSE12511	Global Expression Profiling in Atopic Eczema Reveals Reciprocal Expression of Inflammatory and Lipid Genes	Plos One
Atopic Eczema	EDAR	Expression profiling by array	GPL7191	GSE12511	Global Expression Profiling in Atopic Eczema Reveals Reciprocal Expression of Inflammatory and Lipid Genes	Plos One
Atopic Eczema	LOC390927	Expression profiling by array	GPL7191	GSE12511	Global Expression Profiling in Atopic Eczema Reveals Reciprocal Expression of Inflammatory and Lipid Genes	Plos One
Atopic Eczema	OSBP2	Expression profiling by array	GPL7191	GSE12511	Global Expression Profiling in Atopic Eczema Reveals Reciprocal Expression of Inflammatory and Lipid Genes	Plos One
Atopic Eczema	STAT5A	Expression profiling by array	GPL7191	GSE12511	Global Expression Profiling in Atopic Eczema Reveals Reciprocal Expression of Inflammatory and Lipid Genes	Plos One
Atopic Eczema	LOC643564	Expression profiling by array	GPL7191	GSE12511	Global Expression Profiling in Atopic Eczema Reveals Reciprocal Expression of Inflammatory and Lipid Genes	Plos One
Atopic Eczema	NRG3	Expression profiling by array	GPL7191	GSE12511	Global Expression Profiling in Atopic Eczema Reveals Reciprocal Expression of Inflammatory and Lipid Genes	Plos One
Atopic Eczema	DRG1	Expression profiling by array	GPL7191	GSE12511	Global Expression Profiling in Atopic Eczema Reveals Reciprocal Expression of Inflammatory and Lipid Genes	Plos One
Atopic Eczema	PODN	Expression profiling by array	GPL7191	GSE12511	Global Expression Profiling in Atopic Eczema Reveals Reciprocal Expression of Inflammatory and Lipid Genes	Plos One
Atopic Eczema	MYO1E	Expression profiling by array	GPL7191	GSE12511	Global Expression Profiling in Atopic Eczema Reveals Reciprocal Expression of Inflammatory and Lipid Genes	Plos One
Atopic Eczema	TFF3	Expression profiling by array	GPL7191	GSE12511	Global Expression Profiling in Atopic Eczema Reveals Reciprocal Expression of Inflammatory and Lipid Genes	Plos One
Atopic Eczema	IER5	Expression profiling by array	GPL7191	GSE12511	Global Expression Profiling in Atopic Eczema Reveals Reciprocal Expression of Inflammatory and Lipid Genes	Plos One
Atopic Eczema	UBE2L3	Expression profiling by array	GPL7191	GSE12511	Global Expression Profiling in Atopic Eczema Reveals Reciprocal Expression of Inflammatory and Lipid Genes	Plos One
Atopic Eczema	NCK1	Expression profiling by array	GPL7191	GSE12511	Global Expression Profiling in Atopic Eczema Reveals Reciprocal Expression of Inflammatory and Lipid Genes	Plos One

Atopic Eczema	AA489655	Expression profiling by array	GPL7191	GSE12511	Global Expression Profiling in Atopic Eczema Reveals Reciprocal Expression of Inflammatory and Lipid Genes	Plos One
Atopic Eczema	TMEM41A	Expression profiling by array	GPL7191	GSE12511	Global Expression Profiling in Atopic Eczema Reveals Reciprocal Expression of Inflammatory and Lipid Genes	Plos One
Atopic Eczema	SELE	Expression profiling by array	GPL7191	GSE12511	Global Expression Profiling in Atopic Eczema Reveals Reciprocal Expression of Inflammatory and Lipid Genes	Plos One
Atopic Eczema	PNPLA4	Expression profiling by array	GPL7191	GSE12511	Global Expression Profiling in Atopic Eczema Reveals Reciprocal Expression of Inflammatory and Lipid Genes	Plos One
Atopic Eczema	JPH2	Expression profiling by array	GPL7191	GSE12511	Global Expression Profiling in Atopic Eczema Reveals Reciprocal Expression of Inflammatory and Lipid Genes	Plos One
Atopic Eczema	BCAP29	Expression profiling by array	GPL7191	GSE12511	Global Expression Profiling in Atopic Eczema Reveals Reciprocal Expression of Inflammatory and Lipid Genes	Plos One
Atopic Eczema	KIF1C	Expression profiling by array	GPL7191	GSE12511	Global Expression Profiling in Atopic Eczema Reveals Reciprocal Expression of Inflammatory and Lipid Genes	Plos One
Atopic Eczema	PGM5P1	Expression profiling by array	GPL7191	GSE12511	Global Expression Profiling in Atopic Eczema Reveals Reciprocal Expression of Inflammatory and Lipid Genes	Plos One
Atopic Eczema	FCHO2	Expression profiling by array	GPL7191	GSE12511	Global Expression Profiling in Atopic Eczema Reveals Reciprocal Expression of Inflammatory and Lipid Genes	Plos One
Atopic Eczema	REV1L	Expression profiling by array	GPL7191	GSE12511	Global Expression Profiling in Atopic Eczema Reveals Reciprocal Expression of Inflammatory and Lipid Genes	Plos One
Atopic Eczema	GANAB	Expression profiling by array	GPL7191	GSE12511	Global Expression Profiling in Atopic Eczema Reveals Reciprocal Expression of Inflammatory and Lipid Genes	Plos One
Atopic Eczema	MFGE8	Expression profiling by array	GPL7191	GSE12511	Global Expression Profiling in Atopic Eczema Reveals Reciprocal Expression of Inflammatory and Lipid Genes	Plos One
Atopic Eczema	RARRES3	Expression profiling by array	GPL7191	GSE12511	Global Expression Profiling in Atopic Eczema Reveals Reciprocal Expression of Inflammatory and Lipid Genes	Plos One
Atopic Eczema	ZNF260	Expression profiling by array	GPL7191	GSE12511	Global Expression Profiling in Atopic Eczema Reveals Reciprocal Expression of Inflammatory and Lipid Genes	Plos One
Atopic Eczema	COBLL1	Expression profiling by array	GPL7191	GSE12511	Global Expression Profiling in Atopic Eczema Reveals Reciprocal Expression of Inflammatory and Lipid Genes	Plos One
Atopic Eczema	ADORA2B	Expression profiling by array	GPL7191	GSE12511	Global Expression Profiling in Atopic Eczema Reveals Reciprocal Expression of Inflammatory and Lipid Genes	Plos One
Atopic Eczema	ARD1A	Expression profiling by array	GPL7191	GSE12511	Global Expression Profiling in Atopic Eczema Reveals Reciprocal Expression of Inflammatory and Lipid Genes	Plos One
Atopic Eczema	CPSF3L	Expression profiling by array	GPL7191	GSE12511	Global Expression Profiling in Atopic Eczema Reveals Reciprocal Expression of Inflammatory and Lipid Genes	Plos One
Atopic Eczema	EPB41L4B	Expression profiling by array	GPL7191	GSE12511	Global Expression Profiling in Atopic Eczema Reveals Reciprocal Expression of Inflammatory and Lipid Genes	Plos One
Atopic Eczema	DEGS1	Expression profiling by array	GPL7191	GSE12511	Global Expression Profiling in Atopic Eczema Reveals Reciprocal Expression of Inflammatory and Lipid Genes	Plos One
Atopic Eczema	ANXA4	Expression profiling by array	GPL7191	GSE12511	Global Expression Profiling in Atopic Eczema Reveals Reciprocal Expression of Inflammatory and Lipid Genes	Plos One
Atopic Eczema	C2ORF24	Expression profiling by array	GPL7191	GSE12511	Global Expression Profiling in Atopic Eczema Reveals Reciprocal Expression of Inflammatory and Lipid Genes	Plos One
Atopic Eczema	HAAO	Expression profiling by array	GPL7191	GSE12511	Global Expression Profiling in Atopic Eczema Reveals Reciprocal Expression of Inflammatory and Lipid Genes	Plos One
Atopic Eczema	LOH11CR2A	Expression profiling by array	GPL7191	GSE12511	Global Expression Profiling in Atopic Eczema Reveals Reciprocal Expression of Inflammatory and Lipid Genes	Plos One
Atopic Eczema	HOXC6	Expression profiling by array	GPL7191	GSE12511	Global Expression Profiling in Atopic Eczema Reveals Reciprocal Expression of Inflammatory and Lipid Genes	Plos One
Atopic Eczema	NUP210	Expression profiling by array	GPL7191	GSE12511	Global Expression Profiling in Atopic Eczema Reveals Reciprocal Expression of Inflammatory and Lipid Genes	Plos One
Atopic Eczema	LASP1	Expression profiling by array	GPL7191	GSE12511	Global Expression Profiling in Atopic Eczema Reveals Reciprocal Expression of Inflammatory and Lipid Genes	Plos One
Atopic Eczema	TAF1B	Expression profiling by array	GPL7191	GSE12511	Global Expression Profiling in Atopic Eczema Reveals Reciprocal Expression of Inflammatory and Lipid Genes	Plos One
Atopic Eczema	ARHGEF2	Expression profiling by array	GPL7191	GSE12511	Global Expression Profiling in Atopic Eczema Reveals Reciprocal Expression of Inflammatory and Lipid Genes	Plos One
Atopic Eczema	TMEM101	Expression profiling by array	GPL7191	GSE12511	Global Expression Profiling in Atopic Eczema Reveals Reciprocal Expression of Inflammatory and Lipid Genes	Plos One
Atopic Eczema	CRLF1	Expression profiling by array	GPL7191	GSE12511	Global Expression Profiling in Atopic Eczema Reveals Reciprocal Expression of Inflammatory and Lipid Genes	Plos One
Atopic Eczema	ATP10B	Expression profiling by array	GPL7191	GSE12511	Global Expression Profiling in Atopic Eczema Reveals Reciprocal Expression of Inflammatory and Lipid Genes	Plos One
Atopic Eczema	CASC4	Expression profiling by array	GPL7191	GSE12511	Global Expression Profiling in Atopic Eczema Reveals Reciprocal Expression of Inflammatory and Lipid Genes	Plos One
Atopic Eczema	LOC643988	Expression profiling by array	GPL7191	GSE12511	Global Expression Profiling in Atopic Eczema Reveals Reciprocal Expression of Inflammatory and Lipid Genes	Plos One
Atopic Eczema	AGPAT3	Expression profiling by array	GPL7191	GSE12511	Global Expression Profiling in Atopic Eczema Reveals Reciprocal Expression of Inflammatory and Lipid Genes	Plos One
Atopic Eczema	VPS37C	Expression profiling by array	GPL7191	GSE12511	Global Expression Profiling in Atopic Eczema Reveals Reciprocal Expression of Inflammatory and Lipid Genes	Plos One
Atopic Eczema	RPS6KB2	Expression profiling by array	GPL7191	GSE12511	Global Expression Profiling in Atopic Eczema Reveals Reciprocal Expression of Inflammatory and Lipid Genes	Plos One
Atopic Eczema	HMHA1	Expression profiling by array	GPL7191	GSE12511	Global Expression Profiling in Atopic Eczema Reveals Reciprocal Expression of Inflammatory and Lipid Genes	Plos One
Atopic Eczema	MAPK13	Expression profiling by array	GPL7191	GSE12511	Global Expression Profiling in Atopic Eczema Reveals Reciprocal Expression of Inflammatory and Lipid Genes	Plos One
Atopic Eczema	RNF8	Expression profiling by array	GPL7191	GSE12511	Global Expression Profiling in Atopic Eczema Reveals Reciprocal Expression of Inflammatory and Lipid Genes	Plos One
Atopic Eczema	CPA3	Expression profiling by array	GPL7191	GSE12511	Global Expression Profiling in Atopic Eczema Reveals Reciprocal Expression of Inflammatory and Lipid Genes	Plos One
Atopic Eczema	TMEM127	Expression profiling by array	GPL7191	GSE12511	Global Expression Profiling in Atopic Eczema Reveals Reciprocal Expression of Inflammatory and Lipid Genes	Plos One
Atopic Eczema	AA676776	Expression profiling by array	GPL7191	GSE12511	Global Expression Profiling in Atopic Eczema Reveals Reciprocal Expression of Inflammatory and Lipid Genes	Plos One
Atopic Eczema	TMED5	Expression profiling by array	GPL7191	GSE12511	Global Expression Profiling in Atopic Eczema Reveals Reciprocal Expression of Inflammatory and Lipid Genes	Plos One
Atopic Eczema	MAP2K3	Expression profiling by array	GPL7191	GSE12511	Global Expression Profiling in Atopic Eczema Reveals Reciprocal Expression of Inflammatory and Lipid Genes	Plos One
Atopic Eczema	KDELR2	Expression profiling by array	GPL7191	GSE12511	Global Expression Profiling in Atopic Eczema Reveals Reciprocal Expression of Inflammatory and Lipid Genes	Plos One
Atopic Eczema	TRAT1	Expression profiling by array	GPL7191	GSE12511	Global Expression Profiling in Atopic Eczema Reveals Reciprocal Expression of Inflammatory and Lipid Genes	Plos One
Atopic Eczema	DCBLD1	Expression profiling by array	GPL7191	GSE12511	Global Expression Profiling in Atopic Eczema Reveals Reciprocal Expression of Inflammatory and Lipid Genes	Plos One
Atopic Eczema	TNC	Expression profiling by array	GPL7191	GSE12511	Global Expression Profiling in Atopic Eczema Reveals Reciprocal Expression of Inflammatory and Lipid Genes	Plos One
Atopic Eczema	RABEPK	Expression profiling by array	GPL7191	GSE12511	Global Expression Profiling in Atopic Eczema Reveals Reciprocal Expression of Inflammatory and Lipid Genes	Plos One
Atopic Eczema	ARHGAP1	Expression profiling by array	GPL7191	GSE12511	Global Expression Profiling in Atopic Eczema Reveals Reciprocal Expression of Inflammatory and Lipid Genes	Plos One
Atopic Eczema	PEPD	Expression profiling by array	GPL7191	GSE12511	Global Expression Profiling in Atopic Eczema Reveals Reciprocal Expression of Inflammatory and Lipid Genes	Plos One
Atopic Eczema	CCDC9	Expression profiling by array	GPL7191	GSE12511	Global Expression Profiling in Atopic Eczema Reveals Reciprocal Expression of Inflammatory and Lipid Genes	Plos One
Atopic Eczema	KIAA1407	Expression profiling by array	GPL7191	GSE12511	Global Expression Profiling in Atopic Eczema Reveals Reciprocal Expression of Inflammatory and Lipid Genes	Plos One
Atopic Eczema	GCNT2	Expression profiling by array	GPL7191	GSE12511	Global Expression Profiling in Atopic Eczema Reveals Reciprocal Expression of Inflammatory and Lipid Genes	Plos One
Atopic Eczema	SLC15A1	Expression profiling by array	GPL7191	GSE12511	Global Expression Profiling in Atopic Eczema Reveals Reciprocal Expression of Inflammatory and Lipid Genes	Plos One
Atopic Eczema	FBL	Expression profiling by array	GPL7191	GSE12511	Global Expression Profiling in Atopic Eczema Reveals Reciprocal Expression of Inflammatory and Lipid Genes	Plos One
Atopic Eczema	GABPB2	Expression profiling by array	GPL7191	GSE12511	Global Expression Profiling in Atopic Eczema Reveals Reciprocal Expression of Inflammatory and Lipid Genes	Plos One
Atopic Eczema	GYPC	Expression profiling by array	GPL7191	GSE12511	Global Expression Profiling in Atopic Eczema Reveals Reciprocal Expression of Inflammatory and Lipid Genes	Plos One
Atopic Eczema	RANBP2	Expression profiling by array	GPL7191	GSE12511	Global Expression Profiling in Atopic Eczema Reveals Reciprocal Expression of Inflammatory and Lipid Genes	Plos One
Atopic Eczema	TSPAN5	Expression profiling by array	GPL7191	GSE12511	Global Expression Profiling in Atopic Eczema Reveals Reciprocal Expression of Inflammatory and Lipid Genes	Plos One
Atopic Eczema	MYLK	Expression profiling by array	GPL7191	GSE12511	Global Expression Profiling in Atopic Eczema Reveals Reciprocal Expression of Inflammatory and Lipid Genes	Plos One

Atopic Eczema	SLIT3	Expression profiling by array	GPL7191	GSE12511	Global Expression Profiling in Atopic Eczema Reveals Reciprocal Expression of Inflammatory and Lipid Genes	Plos One
Atopic Eczema	CYP11B1	Expression profiling by array	GPL7191	GSE12511	Global Expression Profiling in Atopic Eczema Reveals Reciprocal Expression of Inflammatory and Lipid Genes	Plos One
Atopic Eczema	COMMD8	Expression profiling by array	GPL7191	GSE12511	Global Expression Profiling in Atopic Eczema Reveals Reciprocal Expression of Inflammatory and Lipid Genes	Plos One
Atopic Eczema	TMF1	Expression profiling by array	GPL7191	GSE12511	Global Expression Profiling in Atopic Eczema Reveals Reciprocal Expression of Inflammatory and Lipid Genes	Plos One
Atopic Eczema	TRIM35	Expression profiling by array	GPL7191	GSE12511	Global Expression Profiling in Atopic Eczema Reveals Reciprocal Expression of Inflammatory and Lipid Genes	Plos One
Atopic Eczema	LOH12CR2	Expression profiling by array	GPL7191	GSE12511	Global Expression Profiling in Atopic Eczema Reveals Reciprocal Expression of Inflammatory and Lipid Genes	Plos One
Atopic Eczema	BIRC3	Expression profiling by array	GPL7191	GSE12511	Global Expression Profiling in Atopic Eczema Reveals Reciprocal Expression of Inflammatory and Lipid Genes	Plos One
Atopic Eczema	SLC14A1	Expression profiling by array	GPL7191	GSE12511	Global Expression Profiling in Atopic Eczema Reveals Reciprocal Expression of Inflammatory and Lipid Genes	Plos One
Atopic Eczema	AA746175	Expression profiling by array	GPL7191	GSE12511	Global Expression Profiling in Atopic Eczema Reveals Reciprocal Expression of Inflammatory and Lipid Genes	Plos One
Atopic Eczema	PISD	Expression profiling by array	GPL7191	GSE12511	Global Expression Profiling in Atopic Eczema Reveals Reciprocal Expression of Inflammatory and Lipid Genes	Plos One
Atopic Eczema	MOSPD3	Expression profiling by array	GPL7191	GSE12511	Global Expression Profiling in Atopic Eczema Reveals Reciprocal Expression of Inflammatory and Lipid Genes	Plos One
Atopic Eczema	CYP3A5	Expression profiling by array	GPL7191	GSE12511	Global Expression Profiling in Atopic Eczema Reveals Reciprocal Expression of Inflammatory and Lipid Genes	Plos One
Atopic Eczema	FSTL4	Expression profiling by array	GPL7191	GSE12511	Global Expression Profiling in Atopic Eczema Reveals Reciprocal Expression of Inflammatory and Lipid Genes	Plos One
Atopic Eczema	UNQ1887	Expression profiling by array	GPL7191	GSE12511	Global Expression Profiling in Atopic Eczema Reveals Reciprocal Expression of Inflammatory and Lipid Genes	Plos One
Atopic Eczema	SLC25A37	Expression profiling by array	GPL7191	GSE12511	Global Expression Profiling in Atopic Eczema Reveals Reciprocal Expression of Inflammatory and Lipid Genes	Plos One
Atopic Eczema	SYT17	Expression profiling by array	GPL7191	GSE12511	Global Expression Profiling in Atopic Eczema Reveals Reciprocal Expression of Inflammatory and Lipid Genes	Plos One
Atopic Eczema	REG4	Expression profiling by array	GPL7191	GSE12511	Global Expression Profiling in Atopic Eczema Reveals Reciprocal Expression of Inflammatory and Lipid Genes	Plos One
Atopic Eczema	PHLDA3	Expression profiling by array	GPL7191	GSE12511	Global Expression Profiling in Atopic Eczema Reveals Reciprocal Expression of Inflammatory and Lipid Genes	Plos One
Atopic Eczema	GNG12	Expression profiling by array	GPL7191	GSE12511	Global Expression Profiling in Atopic Eczema Reveals Reciprocal Expression of Inflammatory and Lipid Genes	Plos One
Atopic Eczema	SAC3D1	Expression profiling by array	GPL7191	GSE12511	Global Expression Profiling in Atopic Eczema Reveals Reciprocal Expression of Inflammatory and Lipid Genes	Plos One
Atopic Eczema	GALNTL1	Expression profiling by array	GPL7191	GSE12511	Global Expression Profiling in Atopic Eczema Reveals Reciprocal Expression of Inflammatory and Lipid Genes	Plos One
Atopic Eczema	ANKDD1A	Expression profiling by array	GPL7191	GSE12511	Global Expression Profiling in Atopic Eczema Reveals Reciprocal Expression of Inflammatory and Lipid Genes	Plos One
Atopic Eczema	PLXNA1	Expression profiling by array	GPL7191	GSE12511	Global Expression Profiling in Atopic Eczema Reveals Reciprocal Expression of Inflammatory and Lipid Genes	Plos One
Atopic Eczema	AA777699	Expression profiling by array	GPL7191	GSE12511	Global Expression Profiling in Atopic Eczema Reveals Reciprocal Expression of Inflammatory and Lipid Genes	Plos One
Atopic Eczema	C15ORF39	Expression profiling by array	GPL7191	GSE12511	Global Expression Profiling in Atopic Eczema Reveals Reciprocal Expression of Inflammatory and Lipid Genes	Plos One
Atopic Eczema	RBM38	Expression profiling by array	GPL7191	GSE12511	Global Expression Profiling in Atopic Eczema Reveals Reciprocal Expression of Inflammatory and Lipid Genes	Plos One
Atopic Eczema	CSPG2	Expression profiling by array	GPL7191	GSE12511	Global Expression Profiling in Atopic Eczema Reveals Reciprocal Expression of Inflammatory and Lipid Genes	Plos One
Atopic Eczema	THRA	Expression profiling by array	GPL7191	GSE12511	Global Expression Profiling in Atopic Eczema Reveals Reciprocal Expression of Inflammatory and Lipid Genes	Plos One
Atopic Eczema	POLRMT	Expression profiling by array	GPL7191	GSE12511	Global Expression Profiling in Atopic Eczema Reveals Reciprocal Expression of Inflammatory and Lipid Genes	Plos One
Atopic Eczema	INOC1	Expression profiling by array	GPL7191	GSE12511	Global Expression Profiling in Atopic Eczema Reveals Reciprocal Expression of Inflammatory and Lipid Genes	Plos One
Atopic Eczema	AA702714	Expression profiling by array	GPL7191	GSE12511	Global Expression Profiling in Atopic Eczema Reveals Reciprocal Expression of Inflammatory and Lipid Genes	Plos One
Atopic Eczema	SLC7A2	Expression profiling by array	GPL7191	GSE12511	Global Expression Profiling in Atopic Eczema Reveals Reciprocal Expression of Inflammatory and Lipid Genes	Plos One
Atopic Eczema	GATA3	Expression profiling by array	GPL7191	GSE12511	Global Expression Profiling in Atopic Eczema Reveals Reciprocal Expression of Inflammatory and Lipid Genes	Plos One
Atopic Eczema	ZC3HAV1	Expression profiling by array	GPL7191	GSE12511	Global Expression Profiling in Atopic Eczema Reveals Reciprocal Expression of Inflammatory and Lipid Genes	Plos One
Atopic Eczema	COBL	Expression profiling by array	GPL7191	GSE12511	Global Expression Profiling in Atopic Eczema Reveals Reciprocal Expression of Inflammatory and Lipid Genes	Plos One
Atopic Eczema	LMNB2	Expression profiling by array	GPL7191	GSE12511	Global Expression Profiling in Atopic Eczema Reveals Reciprocal Expression of Inflammatory and Lipid Genes	Plos One
Atopic Eczema	SLC7A11	Expression profiling by array	GPL7191	GSE12511	Global Expression Profiling in Atopic Eczema Reveals Reciprocal Expression of Inflammatory and Lipid Genes	Plos One
Atopic Eczema	BCL2L10	Expression profiling by array	GPL7191	GSE12511	Global Expression Profiling in Atopic Eczema Reveals Reciprocal Expression of Inflammatory and Lipid Genes	Plos One
Atopic Eczema	SORBS1	Expression profiling by array	GPL7191	GSE12511	Global Expression Profiling in Atopic Eczema Reveals Reciprocal Expression of Inflammatory and Lipid Genes	Plos One
Atopic Eczema	PLAUR	Expression profiling by array	GPL7191	GSE12511	Global Expression Profiling in Atopic Eczema Reveals Reciprocal Expression of Inflammatory and Lipid Genes	Plos One
Atopic Eczema	TMEM140	Expression profiling by array	GPL7191	GSE12511	Global Expression Profiling in Atopic Eczema Reveals Reciprocal Expression of Inflammatory and Lipid Genes	Plos One
Atopic Eczema	CDADC1	Expression profiling by array	GPL7191	GSE12511	Global Expression Profiling in Atopic Eczema Reveals Reciprocal Expression of Inflammatory and Lipid Genes	Plos One
Atopic Eczema	ZNF331	Expression profiling by array	GPL7191	GSE12511	Global Expression Profiling in Atopic Eczema Reveals Reciprocal Expression of Inflammatory and Lipid Genes	Plos One
Atopic Eczema	SLFN11	Expression profiling by array	GPL7191	GSE12511	Global Expression Profiling in Atopic Eczema Reveals Reciprocal Expression of Inflammatory and Lipid Genes	Plos One
Atopic Eczema	ICAM3	Expression profiling by array	GPL7191	GSE12511	Global Expression Profiling in Atopic Eczema Reveals Reciprocal Expression of Inflammatory and Lipid Genes	Plos One
Atopic Eczema	HDAC11	Expression profiling by array	GPL7191	GSE12511	Global Expression Profiling in Atopic Eczema Reveals Reciprocal Expression of Inflammatory and Lipid Genes	Plos One
Atopic Eczema	DERL1	Expression profiling by array	GPL7191	GSE12511	Global Expression Profiling in Atopic Eczema Reveals Reciprocal Expression of Inflammatory and Lipid Genes	Plos One
Atopic Eczema	UBL4A	Expression profiling by array	GPL7191	GSE12511	Global Expression Profiling in Atopic Eczema Reveals Reciprocal Expression of Inflammatory and Lipid Genes	Plos One
Atopic Eczema	CLN8	Expression profiling by array	GPL7191	GSE12511	Global Expression Profiling in Atopic Eczema Reveals Reciprocal Expression of Inflammatory and Lipid Genes	Plos One
Atopic Eczema	TUBB2C	Expression profiling by array	GPL7191	GSE12511	Global Expression Profiling in Atopic Eczema Reveals Reciprocal Expression of Inflammatory and Lipid Genes	Plos One
Atopic Eczema	PKLR	Expression profiling by array	GPL7191	GSE12511	Global Expression Profiling in Atopic Eczema Reveals Reciprocal Expression of Inflammatory and Lipid Genes	Plos One
Atopic Eczema	C19ORF30	Expression profiling by array	GPL7191	GSE12511	Global Expression Profiling in Atopic Eczema Reveals Reciprocal Expression of Inflammatory and Lipid Genes	Plos One
Atopic Eczema	SFRS2IP	Expression profiling by array	GPL7191	GSE12511	Global Expression Profiling in Atopic Eczema Reveals Reciprocal Expression of Inflammatory and Lipid Genes	Plos One
Atopic Eczema	MAPBPIP	Expression profiling by array	GPL7191	GSE12511	Global Expression Profiling in Atopic Eczema Reveals Reciprocal Expression of Inflammatory and Lipid Genes	Plos One
Atopic Eczema	FLII	Expression profiling by array	GPL7191	GSE12511	Global Expression Profiling in Atopic Eczema Reveals Reciprocal Expression of Inflammatory and Lipid Genes	Plos One
Atopic Eczema	PCOLCE	Expression profiling by array	GPL7191	GSE12511	Global Expression Profiling in Atopic Eczema Reveals Reciprocal Expression of Inflammatory and Lipid Genes	Plos One
Atopic Eczema	GMPPB	Expression profiling by array	GPL7191	GSE12511	Global Expression Profiling in Atopic Eczema Reveals Reciprocal Expression of Inflammatory and Lipid Genes	Plos One
Atopic Eczema	TAX1BP1	Expression profiling by array	GPL7191	GSE12511	Global Expression Profiling in Atopic Eczema Reveals Reciprocal Expression of Inflammatory and Lipid Genes	Plos One
Atopic Eczema	AI310519	Expression profiling by array	GPL7191	GSE12511	Global Expression Profiling in Atopic Eczema Reveals Reciprocal Expression of Inflammatory and Lipid Genes	Plos One
Atopic Eczema	RP5-821D11.2	Expression profiling by array	GPL7191	GSE12511	Global Expression Profiling in Atopic Eczema Reveals Reciprocal Expression of Inflammatory and Lipid Genes	Plos One
Atopic Eczema	MLLT1	Expression profiling by array	GPL7191	GSE12511	Global Expression Profiling in Atopic Eczema Reveals Reciprocal Expression of Inflammatory and Lipid Genes	Plos One
Atopic Eczema	C9ORF68	Expression profiling by array	GPL7191	GSE12511	Global Expression Profiling in Atopic Eczema Reveals Reciprocal Expression of Inflammatory and Lipid Genes	Plos One

Atopic Eczema	IFRD2	Expression profiling by array	GPL7191	GSE12511	Global Expression Profiling in Atopic Eczema Reveals Reciprocal Expression of Inflammatory and Lipid Genes	Plos One
Atopic Eczema	RPL18	Expression profiling by array	GPL7191	GSE12511	Global Expression Profiling in Atopic Eczema Reveals Reciprocal Expression of Inflammatory and Lipid Genes	Plos One
Atopic Eczema	PSD4	Expression profiling by array	GPL7191	GSE12511	Global Expression Profiling in Atopic Eczema Reveals Reciprocal Expression of Inflammatory and Lipid Genes	Plos One
Atopic Eczema	SLC26A3	Expression profiling by array	GPL7191	GSE12511	Global Expression Profiling in Atopic Eczema Reveals Reciprocal Expression of Inflammatory and Lipid Genes	Plos One
Atopic Eczema	LAMA4	Expression profiling by array	GPL7191	GSE12511	Global Expression Profiling in Atopic Eczema Reveals Reciprocal Expression of Inflammatory and Lipid Genes	Plos One
Atopic Eczema	MAN1C1	Expression profiling by array	GPL7191	GSE12511	Global Expression Profiling in Atopic Eczema Reveals Reciprocal Expression of Inflammatory and Lipid Genes	Plos One
Atopic Eczema	FAM62C	Expression profiling by array	GPL7191	GSE12511	Global Expression Profiling in Atopic Eczema Reveals Reciprocal Expression of Inflammatory and Lipid Genes	Plos One
Atopic Eczema	MAFB	Expression profiling by array	GPL7191	GSE12511	Global Expression Profiling in Atopic Eczema Reveals Reciprocal Expression of Inflammatory and Lipid Genes	Plos One
Atopic Eczema	MRPL28	Expression profiling by array	GPL7191	GSE12511	Global Expression Profiling in Atopic Eczema Reveals Reciprocal Expression of Inflammatory and Lipid Genes	Plos One
Atopic Eczema	HNRPR	Expression profiling by array	GPL7191	GSE12511	Global Expression Profiling in Atopic Eczema Reveals Reciprocal Expression of Inflammatory and Lipid Genes	Plos One
Atopic Eczema	TMEM158	Expression profiling by array	GPL7191	GSE12511	Global Expression Profiling in Atopic Eczema Reveals Reciprocal Expression of Inflammatory and Lipid Genes	Plos One
Atopic Eczema	ZC3H7B	Expression profiling by array	GPL7191	GSE12511	Global Expression Profiling in Atopic Eczema Reveals Reciprocal Expression of Inflammatory and Lipid Genes	Plos One
Atopic Eczema	FOS	Expression profiling by array	GPL7191	GSE12511	Global Expression Profiling in Atopic Eczema Reveals Reciprocal Expression of Inflammatory and Lipid Genes	Plos One
Atopic Eczema	NUDT5	Expression profiling by array	GPL7191	GSE12511	Global Expression Profiling in Atopic Eczema Reveals Reciprocal Expression of Inflammatory and Lipid Genes	Plos One
Atopic Eczema	ATP11A	Expression profiling by array	GPL7191	GSE12511	Global Expression Profiling in Atopic Eczema Reveals Reciprocal Expression of Inflammatory and Lipid Genes	Plos One
Atopic Eczema	FNTA	Expression profiling by array	GPL7191	GSE12511	Global Expression Profiling in Atopic Eczema Reveals Reciprocal Expression of Inflammatory and Lipid Genes	Plos One
Atopic Eczema	RPL14	Expression profiling by array	GPL7191	GSE12511	Global Expression Profiling in Atopic Eczema Reveals Reciprocal Expression of Inflammatory and Lipid Genes	Plos One
Atopic Eczema	PKIB	Expression profiling by array	GPL7191	GSE12511	Global Expression Profiling in Atopic Eczema Reveals Reciprocal Expression of Inflammatory and Lipid Genes	Plos One
Atopic Eczema	TNPO2	Expression profiling by array	GPL7191	GSE12511	Global Expression Profiling in Atopic Eczema Reveals Reciprocal Expression of Inflammatory and Lipid Genes	Plos One
Atopic Eczema	STARD4	Expression profiling by array	GPL7191	GSE12511	Global Expression Profiling in Atopic Eczema Reveals Reciprocal Expression of Inflammatory and Lipid Genes	Plos One
Atopic Eczema	RASSF5	Expression profiling by array	GPL7191	GSE12511	Global Expression Profiling in Atopic Eczema Reveals Reciprocal Expression of Inflammatory and Lipid Genes	Plos One
Atopic Eczema	PDHA1	Expression profiling by array	GPL7191	GSE12511	Global Expression Profiling in Atopic Eczema Reveals Reciprocal Expression of Inflammatory and Lipid Genes	Plos One
Atopic Eczema	DMRT3	Expression profiling by array	GPL7191	GSE12511	Global Expression Profiling in Atopic Eczema Reveals Reciprocal Expression of Inflammatory and Lipid Genes	Plos One
Atopic Eczema	CROP	Expression profiling by array	GPL7191	GSE12511	Global Expression Profiling in Atopic Eczema Reveals Reciprocal Expression of Inflammatory and Lipid Genes	Plos One
Atopic Eczema	BMP1	Expression profiling by array	GPL7191	GSE12511	Global Expression Profiling in Atopic Eczema Reveals Reciprocal Expression of Inflammatory and Lipid Genes	Plos One
Atopic Eczema	NOL12	Expression profiling by array	GPL7191	GSE12511	Global Expression Profiling in Atopic Eczema Reveals Reciprocal Expression of Inflammatory and Lipid Genes	Plos One
Atopic Eczema	SFPQ	Expression profiling by array	GPL7191	GSE12511	Global Expression Profiling in Atopic Eczema Reveals Reciprocal Expression of Inflammatory and Lipid Genes	Plos One
Atopic Eczema	COL5A2	Expression profiling by array	GPL7191	GSE12511	Global Expression Profiling in Atopic Eczema Reveals Reciprocal Expression of Inflammatory and Lipid Genes	Plos One
Atopic Eczema	GFOD2	Expression profiling by array	GPL7191	GSE12511	Global Expression Profiling in Atopic Eczema Reveals Reciprocal Expression of Inflammatory and Lipid Genes	Plos One
Atopic Eczema	KIAA1754	Expression profiling by array	GPL7191	GSE12511	Global Expression Profiling in Atopic Eczema Reveals Reciprocal Expression of Inflammatory and Lipid Genes	Plos One
Atopic Eczema	PTDSS1	Expression profiling by array	GPL7191	GSE12511	Global Expression Profiling in Atopic Eczema Reveals Reciprocal Expression of Inflammatory and Lipid Genes	Plos One
Atopic Eczema	MYST3	Expression profiling by array	GPL7191	GSE12511	Global Expression Profiling in Atopic Eczema Reveals Reciprocal Expression of Inflammatory and Lipid Genes	Plos One
Atopic Eczema	KIAA0256	Expression profiling by array	GPL7191	GSE12511	Global Expression Profiling in Atopic Eczema Reveals Reciprocal Expression of Inflammatory and Lipid Genes	Plos One
Atopic Eczema	DLC1	Expression profiling by array	GPL7191	GSE12511	Global Expression Profiling in Atopic Eczema Reveals Reciprocal Expression of Inflammatory and Lipid Genes	Plos One
Atopic Eczema	LPL	Expression profiling by array	GPL7191	GSE12511	Global Expression Profiling in Atopic Eczema Reveals Reciprocal Expression of Inflammatory and Lipid Genes	Plos One
Atopic Eczema	ALDH5A1	Expression profiling by array	GPL7191	GSE12511	Global Expression Profiling in Atopic Eczema Reveals Reciprocal Expression of Inflammatory and Lipid Genes	Plos One
Atopic Eczema	ZNF688	Expression profiling by array	GPL7191	GSE12511	Global Expression Profiling in Atopic Eczema Reveals Reciprocal Expression of Inflammatory and Lipid Genes	Plos One
Atopic Eczema	AI269958	Expression profiling by array	GPL7191	GSE12511	Global Expression Profiling in Atopic Eczema Reveals Reciprocal Expression of Inflammatory and Lipid Genes	Plos One
Atopic Eczema	STCH	Expression profiling by array	GPL7191	GSE12511	Global Expression Profiling in Atopic Eczema Reveals Reciprocal Expression of Inflammatory and Lipid Genes	Plos One
Atopic Eczema	MGAT1	Expression profiling by array	GPL7191	GSE12511	Global Expression Profiling in Atopic Eczema Reveals Reciprocal Expression of Inflammatory and Lipid Genes	Plos One
Atopic Eczema	GPBP1	Expression profiling by array	GPL7191	GSE12511	Global Expression Profiling in Atopic Eczema Reveals Reciprocal Expression of Inflammatory and Lipid Genes	Plos One
Atopic Eczema	B3GNT2	Expression profiling by array	GPL7191	GSE12511	Global Expression Profiling in Atopic Eczema Reveals Reciprocal Expression of Inflammatory and Lipid Genes	Plos One
Atopic Eczema	CRYAB	Expression profiling by array	GPL7191	GSE12511	Global Expression Profiling in Atopic Eczema Reveals Reciprocal Expression of Inflammatory and Lipid Genes	Plos One
Atopic Eczema	RET	Expression profiling by array	GPL7191	GSE12511	Global Expression Profiling in Atopic Eczema Reveals Reciprocal Expression of Inflammatory and Lipid Genes	Plos One
Atopic Eczema	EEF1D	Expression profiling by array	GPL7191	GSE12511	Global Expression Profiling in Atopic Eczema Reveals Reciprocal Expression of Inflammatory and Lipid Genes	Plos One
Atopic Eczema	MBOAT2	Expression profiling by array	GPL7191	GSE12511	Global Expression Profiling in Atopic Eczema Reveals Reciprocal Expression of Inflammatory and Lipid Genes	Plos One
Atopic Eczema	SEPP1	Expression profiling by array	GPL7191	GSE12511	Global Expression Profiling in Atopic Eczema Reveals Reciprocal Expression of Inflammatory and Lipid Genes	Plos One
Atopic Eczema	LOC146795	Expression profiling by array	GPL7191	GSE12511	Global Expression Profiling in Atopic Eczema Reveals Reciprocal Expression of Inflammatory and Lipid Genes	Plos One
Atopic Eczema	DTNA	Expression profiling by array	GPL7191	GSE12511	Global Expression Profiling in Atopic Eczema Reveals Reciprocal Expression of Inflammatory and Lipid Genes	Plos One
Atopic Eczema	SIDT2	Expression profiling by array	GPL7191	GSE12511	Global Expression Profiling in Atopic Eczema Reveals Reciprocal Expression of Inflammatory and Lipid Genes	Plos One
Atopic Eczema	EPAS1	Expression profiling by array	GPL7191	GSE12511	Global Expression Profiling in Atopic Eczema Reveals Reciprocal Expression of Inflammatory and Lipid Genes	Plos One
Atopic Eczema	MCM4	Expression profiling by array	GPL7191	GSE12511	Global Expression Profiling in Atopic Eczema Reveals Reciprocal Expression of Inflammatory and Lipid Genes	Plos One
Atopic Eczema	PIGF	Expression profiling by array	GPL7191	GSE12511	Global Expression Profiling in Atopic Eczema Reveals Reciprocal Expression of Inflammatory and Lipid Genes	Plos One
Atopic Eczema	LOC349114	Expression profiling by array	GPL7191	GSE12511	Global Expression Profiling in Atopic Eczema Reveals Reciprocal Expression of Inflammatory and Lipid Genes	Plos One
Atopic Eczema	MARCO	Expression profiling by array	GPL7191	GSE12511	Global Expression Profiling in Atopic Eczema Reveals Reciprocal Expression of Inflammatory and Lipid Genes	Plos One
Atopic Eczema	WAS	Expression profiling by array	GPL7191	GSE12511	Global Expression Profiling in Atopic Eczema Reveals Reciprocal Expression of Inflammatory and Lipid Genes	Plos One
Atopic Eczema	KIAA0146	Expression profiling by array	GPL7191	GSE12511	Global Expression Profiling in Atopic Eczema Reveals Reciprocal Expression of Inflammatory and Lipid Genes	Plos One
Atopic Eczema	TXNL2	Expression profiling by array	GPL7191	GSE12511	Global Expression Profiling in Atopic Eczema Reveals Reciprocal Expression of Inflammatory and Lipid Genes	Plos One
Atopic Eczema	PKN2	Expression profiling by array	GPL7191	GSE12511	Global Expression Profiling in Atopic Eczema Reveals Reciprocal Expression of Inflammatory and Lipid Genes	Plos One
Atopic Eczema	GATA2	Expression profiling by array	GPL7191	GSE12511	Global Expression Profiling in Atopic Eczema Reveals Reciprocal Expression of Inflammatory and Lipid Genes	Plos One
Atopic Eczema	RBP5	Expression profiling by array	GPL7191	GSE12511	Global Expression Profiling in Atopic Eczema Reveals Reciprocal Expression of Inflammatory and Lipid Genes	Plos One
Atopic Eczema	LOC402571	Expression profiling by array	GPL7191	GSE12511	Global Expression Profiling in Atopic Eczema Reveals Reciprocal Expression of Inflammatory and Lipid Genes	Plos One

Atopic Eczema	RMND5A	Expression profiling by array	GPL7191	GSE12511	Global Expression Profiling in Atopic Eczema Reveals Reciprocal Expression of Inflammatory and Lipid Genes	Plos One
Atopic Eczema	C4A	Expression profiling by array	GPL7191	GSE12511	Global Expression Profiling in Atopic Eczema Reveals Reciprocal Expression of Inflammatory and Lipid Genes	Plos One
Atopic Eczema	C6ORF130	Expression profiling by array	GPL7191	GSE12511	Global Expression Profiling in Atopic Eczema Reveals Reciprocal Expression of Inflammatory and Lipid Genes	Plos One
Atopic Eczema	PKMYT1	Expression profiling by array	GPL7191	GSE12511	Global Expression Profiling in Atopic Eczema Reveals Reciprocal Expression of Inflammatory and Lipid Genes	Plos One
Atopic Eczema	LOC645626	Expression profiling by array	GPL7191	GSE12511	Global Expression Profiling in Atopic Eczema Reveals Reciprocal Expression of Inflammatory and Lipid Genes	Plos One
Atopic Eczema	BOK	Expression profiling by array	GPL7191	GSE12511	Global Expression Profiling in Atopic Eczema Reveals Reciprocal Expression of Inflammatory and Lipid Genes	Plos One
Atopic Eczema	ASNA1	Expression profiling by array	GPL7191	GSE12511	Global Expression Profiling in Atopic Eczema Reveals Reciprocal Expression of Inflammatory and Lipid Genes	Plos One
Atopic Eczema	CAND1	Expression profiling by array	GPL7191	GSE12511	Global Expression Profiling in Atopic Eczema Reveals Reciprocal Expression of Inflammatory and Lipid Genes	Plos One
Atopic Eczema	LOC54103	Expression profiling by array	GPL7191	GSE12511	Global Expression Profiling in Atopic Eczema Reveals Reciprocal Expression of Inflammatory and Lipid Genes	Plos One
Atopic Eczema	CASR	Expression profiling by array	GPL7191	GSE12511	Global Expression Profiling in Atopic Eczema Reveals Reciprocal Expression of Inflammatory and Lipid Genes	Plos One
Atopic Eczema	PDPN	Expression profiling by array	GPL7191	GSE12511	Global Expression Profiling in Atopic Eczema Reveals Reciprocal Expression of Inflammatory and Lipid Genes	Plos One
Atopic Eczema	LOC389541	Expression profiling by array	GPL7191	GSE12511	Global Expression Profiling in Atopic Eczema Reveals Reciprocal Expression of Inflammatory and Lipid Genes	Plos One
Atopic Eczema	APBA2BP	Expression profiling by array	GPL7191	GSE12511	Global Expression Profiling in Atopic Eczema Reveals Reciprocal Expression of Inflammatory and Lipid Genes	Plos One
Atopic Eczema	BTBD2	Expression profiling by array	GPL7191	GSE12511	Global Expression Profiling in Atopic Eczema Reveals Reciprocal Expression of Inflammatory and Lipid Genes	Plos One
Atopic Eczema	NIP30	Expression profiling by array	GPL7191	GSE12511	Global Expression Profiling in Atopic Eczema Reveals Reciprocal Expression of Inflammatory and Lipid Genes	Plos One
Atopic Eczema	DARC	Expression profiling by array	GPL7191	GSE12511	Global Expression Profiling in Atopic Eczema Reveals Reciprocal Expression of Inflammatory and Lipid Genes	Plos One
Atopic Eczema	SCD5	Expression profiling by array	GPL7191	GSE12511	Global Expression Profiling in Atopic Eczema Reveals Reciprocal Expression of Inflammatory and Lipid Genes	Plos One
Atopic Eczema	C6ORF105	Expression profiling by array	GPL7191	GSE12511	Global Expression Profiling in Atopic Eczema Reveals Reciprocal Expression of Inflammatory and Lipid Genes	Plos One
Atopic Eczema	N73572	Expression profiling by array	GPL7191	GSE12511	Global Expression Profiling in Atopic Eczema Reveals Reciprocal Expression of Inflammatory and Lipid Genes	Plos One
Atopic Eczema	HNRPAB	Expression profiling by array	GPL7191	GSE12511	Global Expression Profiling in Atopic Eczema Reveals Reciprocal Expression of Inflammatory and Lipid Genes	Plos One
Atopic Eczema	LOC400590	Expression profiling by array	GPL7191	GSE12511	Global Expression Profiling in Atopic Eczema Reveals Reciprocal Expression of Inflammatory and Lipid Genes	Plos One
Atopic Eczema	ZDHHC2	Expression profiling by array	GPL7191	GSE12511	Global Expression Profiling in Atopic Eczema Reveals Reciprocal Expression of Inflammatory and Lipid Genes	Plos One
Atopic Eczema	RAB40B	Expression profiling by array	GPL7191	GSE12511	Global Expression Profiling in Atopic Eczema Reveals Reciprocal Expression of Inflammatory and Lipid Genes	Plos One
Atopic Eczema	ARHGAP15	Expression profiling by array	GPL7191	GSE12511	Global Expression Profiling in Atopic Eczema Reveals Reciprocal Expression of Inflammatory and Lipid Genes	Plos One
Atopic Eczema	RRBP1	Expression profiling by array	GPL7191	GSE12511	Global Expression Profiling in Atopic Eczema Reveals Reciprocal Expression of Inflammatory and Lipid Genes	Plos One
Atopic Eczema	MOSPD2	Expression profiling by array	GPL7191	GSE12511	Global Expression Profiling in Atopic Eczema Reveals Reciprocal Expression of Inflammatory and Lipid Genes	Plos One
Atopic Eczema	C11ORF66	Expression profiling by array	GPL7191	GSE12511	Global Expression Profiling in Atopic Eczema Reveals Reciprocal Expression of Inflammatory and Lipid Genes	Plos One
Atopic Eczema	DAP	Expression profiling by array	GPL7191	GSE12511	Global Expression Profiling in Atopic Eczema Reveals Reciprocal Expression of Inflammatory and Lipid Genes	Plos One
Atopic Eczema	GRB2	Expression profiling by array	GPL7191	GSE12511	Global Expression Profiling in Atopic Eczema Reveals Reciprocal Expression of Inflammatory and Lipid Genes	Plos One
Atopic Eczema	CALML3	Expression profiling by array	GPL7191	GSE12511	Global Expression Profiling in Atopic Eczema Reveals Reciprocal Expression of Inflammatory and Lipid Genes	Plos One
Atopic Eczema	ISOC1	Expression profiling by array	GPL7191	GSE12511	Global Expression Profiling in Atopic Eczema Reveals Reciprocal Expression of Inflammatory and Lipid Genes	Plos One
Atopic Eczema	TMEM107	Expression profiling by array	GPL7191	GSE12511	Global Expression Profiling in Atopic Eczema Reveals Reciprocal Expression of Inflammatory and Lipid Genes	Plos One
Atopic Eczema	SFRS1	Expression profiling by array	GPL7191	GSE12511	Global Expression Profiling in Atopic Eczema Reveals Reciprocal Expression of Inflammatory and Lipid Genes	Plos One
Atopic Eczema	RNASEH2A	Expression profiling by array	GPL7191	GSE12511	Global Expression Profiling in Atopic Eczema Reveals Reciprocal Expression of Inflammatory and Lipid Genes	Plos One
Atopic Eczema	CHD4	Expression profiling by array	GPL7191	GSE12511	Global Expression Profiling in Atopic Eczema Reveals Reciprocal Expression of Inflammatory and Lipid Genes	Plos One
Atopic Eczema	MAN2C1	Expression profiling by array	GPL7191	GSE12511	Global Expression Profiling in Atopic Eczema Reveals Reciprocal Expression of Inflammatory and Lipid Genes	Plos One
Atopic Eczema	DAD1	Expression profiling by array	GPL7191	GSE12511	Global Expression Profiling in Atopic Eczema Reveals Reciprocal Expression of Inflammatory and Lipid Genes	Plos One
Atopic Eczema	AKR1C2	Expression profiling by array	GPL7191	GSE12511	Global Expression Profiling in Atopic Eczema Reveals Reciprocal Expression of Inflammatory and Lipid Genes	Plos One
Atopic Eczema	KLRK1	Expression profiling by array	GPL7191	GSE12511	Global Expression Profiling in Atopic Eczema Reveals Reciprocal Expression of Inflammatory and Lipid Genes	Plos One
Atopic Eczema	LOC644450	Expression profiling by array	GPL7191	GSE12511	Global Expression Profiling in Atopic Eczema Reveals Reciprocal Expression of Inflammatory and Lipid Genes	Plos One
Atopic Eczema	FOSL2	Expression profiling by array	GPL7191	GSE12511	Global Expression Profiling in Atopic Eczema Reveals Reciprocal Expression of Inflammatory and Lipid Genes	Plos One
Atopic Eczema	NDP	Expression profiling by array	GPL7191	GSE12511	Global Expression Profiling in Atopic Eczema Reveals Reciprocal Expression of Inflammatory and Lipid Genes	Plos One
Atopic Eczema	DDX49	Expression profiling by array	GPL7191	GSE12511	Global Expression Profiling in Atopic Eczema Reveals Reciprocal Expression of Inflammatory and Lipid Genes	Plos One
Atopic Eczema	CHMP4A	Expression profiling by array	GPL7191	GSE12511	Global Expression Profiling in Atopic Eczema Reveals Reciprocal Expression of Inflammatory and Lipid Genes	Plos One
Atopic Eczema	METTL7B	Expression profiling by array	GPL7191	GSE12511	Global Expression Profiling in Atopic Eczema Reveals Reciprocal Expression of Inflammatory and Lipid Genes	Plos One
Atopic Eczema	TACC1	Expression profiling by array	GPL7191	GSE12511	Global Expression Profiling in Atopic Eczema Reveals Reciprocal Expression of Inflammatory and Lipid Genes	Plos One
Atopic Eczema	C14ORF8	Expression profiling by array	GPL7191	GSE12511	Global Expression Profiling in Atopic Eczema Reveals Reciprocal Expression of Inflammatory and Lipid Genes	Plos One
Atopic Eczema	ZNF10	Expression profiling by array	GPL7191	GSE12511	Global Expression Profiling in Atopic Eczema Reveals Reciprocal Expression of Inflammatory and Lipid Genes	Plos One
Atopic Eczema	AA866057	Expression profiling by array	GPL7191	GSE12511	Global Expression Profiling in Atopic Eczema Reveals Reciprocal Expression of Inflammatory and Lipid Genes	Plos One
Atopic Eczema	AA928058	Expression profiling by array	GPL7191	GSE12511	Global Expression Profiling in Atopic Eczema Reveals Reciprocal Expression of Inflammatory and Lipid Genes	Plos One
Atopic Eczema	SLC1A3	Expression profiling by array	GPL7191	GSE12511	Global Expression Profiling in Atopic Eczema Reveals Reciprocal Expression of Inflammatory and Lipid Genes	Plos One
Atopic Eczema	EPHA5	Expression profiling by array	GPL7191	GSE12511	Global Expression Profiling in Atopic Eczema Reveals Reciprocal Expression of Inflammatory and Lipid Genes	Plos One
Atopic Eczema	SRGAP2	Expression profiling by array	GPL7191	GSE12511	Global Expression Profiling in Atopic Eczema Reveals Reciprocal Expression of Inflammatory and Lipid Genes	Plos One
Atopic Eczema	ERBB4	Expression profiling by array	GPL7191	GSE12511	Global Expression Profiling in Atopic Eczema Reveals Reciprocal Expression of Inflammatory and Lipid Genes	Plos One
Atopic Eczema	C18ORF24	Expression profiling by array	GPL7191	GSE12511	Global Expression Profiling in Atopic Eczema Reveals Reciprocal Expression of Inflammatory and Lipid Genes	Plos One
Atopic Eczema	CILP	Expression profiling by array	GPL7191	GSE12511	Global Expression Profiling in Atopic Eczema Reveals Reciprocal Expression of Inflammatory and Lipid Genes	Plos One
Atopic Eczema	ITGBL1	Expression profiling by array	GPL7191	GSE12511	Global Expression Profiling in Atopic Eczema Reveals Reciprocal Expression of Inflammatory and Lipid Genes	Plos One
Atopic Eczema	DCUN1D4	Expression profiling by array	GPL7191	GSE12511	Global Expression Profiling in Atopic Eczema Reveals Reciprocal Expression of Inflammatory and Lipid Genes	Plos One
Atopic Eczema	ZNF704	Expression profiling by array	GPL7191	GSE12511	Global Expression Profiling in Atopic Eczema Reveals Reciprocal Expression of Inflammatory and Lipid Genes	Plos One
Atopic Eczema	FCGR2B	Expression profiling by array	GPL7191	GSE12511	Global Expression Profiling in Atopic Eczema Reveals Reciprocal Expression of Inflammatory and Lipid Genes	Plos One
Atopic Eczema	AA777417	Expression profiling by array	GPL7191	GSE12511	Global Expression Profiling in Atopic Eczema Reveals Reciprocal Expression of Inflammatory and Lipid Genes	Plos One
Atopic Eczema	ARHGDIA	Expression profiling by array	GPL7191	GSE12511	Global Expression Profiling in Atopic Eczema Reveals Reciprocal Expression of Inflammatory and Lipid Genes	Plos One

Atopic Eczema	BCL2L13	Expression profiling by array	GPL7191	GSE12511	Global Expression Profiling in Atopic Eczema Reveals Reciprocal Expression of Inflammatory and Lipid Genes	Plos One
Atopic Eczema	MECR	Expression profiling by array	GPL7191	GSE12511	Global Expression Profiling in Atopic Eczema Reveals Reciprocal Expression of Inflammatory and Lipid Genes	Plos One
Atopic Eczema	GRK5	Expression profiling by array	GPL7191	GSE12511	Global Expression Profiling in Atopic Eczema Reveals Reciprocal Expression of Inflammatory and Lipid Genes	Plos One
Atopic Eczema	POU6F1	Expression profiling by array	GPL7191	GSE12511	Global Expression Profiling in Atopic Eczema Reveals Reciprocal Expression of Inflammatory and Lipid Genes	Plos One
Atopic Eczema	LRRC16	Expression profiling by array	GPL7191	GSE12511	Global Expression Profiling in Atopic Eczema Reveals Reciprocal Expression of Inflammatory and Lipid Genes	Plos One
Atopic Eczema	AI095492	Expression profiling by array	GPL7191	GSE12511	Global Expression Profiling in Atopic Eczema Reveals Reciprocal Expression of Inflammatory and Lipid Genes	Plos One
Atopic Eczema	PDE4DIP	Expression profiling by array	GPL7191	GSE12511	Global Expression Profiling in Atopic Eczema Reveals Reciprocal Expression of Inflammatory and Lipid Genes	Plos One
Atopic Eczema	EFNB2	Expression profiling by array	GPL7191	GSE12511	Global Expression Profiling in Atopic Eczema Reveals Reciprocal Expression of Inflammatory and Lipid Genes	Plos One
Atopic Eczema	FBXO45	Expression profiling by array	GPL7191	GSE12511	Global Expression Profiling in Atopic Eczema Reveals Reciprocal Expression of Inflammatory and Lipid Genes	Plos One
Atopic Eczema	ARHGEF18	Expression profiling by array	GPL7191	GSE12511	Global Expression Profiling in Atopic Eczema Reveals Reciprocal Expression of Inflammatory and Lipid Genes	Plos One
Atopic Eczema	AA773755	Expression profiling by array	GPL7191	GSE12511	Global Expression Profiling in Atopic Eczema Reveals Reciprocal Expression of Inflammatory and Lipid Genes	Plos One
Atopic Eczema	LOC441212	Expression profiling by array	GPL7191	GSE12511	Global Expression Profiling in Atopic Eczema Reveals Reciprocal Expression of Inflammatory and Lipid Genes	Plos One
Atopic Eczema	COMMD5	Expression profiling by array	GPL7191	GSE12511	Global Expression Profiling in Atopic Eczema Reveals Reciprocal Expression of Inflammatory and Lipid Genes	Plos One
Atopic Eczema	CISH	Expression profiling by array	GPL7191	GSE12511	Global Expression Profiling in Atopic Eczema Reveals Reciprocal Expression of Inflammatory and Lipid Genes	Plos One
Atopic Eczema	MMP19	Expression profiling by array	GPL7191	GSE12511	Global Expression Profiling in Atopic Eczema Reveals Reciprocal Expression of Inflammatory and Lipid Genes	Plos One
Atopic Eczema	NPLOC4	Expression profiling by array	GPL7191	GSE12511	Global Expression Profiling in Atopic Eczema Reveals Reciprocal Expression of Inflammatory and Lipid Genes	Plos One
Atopic Eczema	FAM3C	Expression profiling by array	GPL7191	GSE12511	Global Expression Profiling in Atopic Eczema Reveals Reciprocal Expression of Inflammatory and Lipid Genes	Plos One
Atopic Eczema	PKP1	Expression profiling by array	GPL7191	GSE12511	Global Expression Profiling in Atopic Eczema Reveals Reciprocal Expression of Inflammatory and Lipid Genes	Plos One
Atopic Eczema	NUP214	Expression profiling by array	GPL7191	GSE12511	Global Expression Profiling in Atopic Eczema Reveals Reciprocal Expression of Inflammatory and Lipid Genes	Plos One
Atopic Eczema	SELP	Expression profiling by array	GPL7191	GSE12511	Global Expression Profiling in Atopic Eczema Reveals Reciprocal Expression of Inflammatory and Lipid Genes	Plos One
Atopic Eczema	RPL22L1	Expression profiling by array	GPL7191	GSE12511	Global Expression Profiling in Atopic Eczema Reveals Reciprocal Expression of Inflammatory and Lipid Genes	Plos One
Atopic Eczema	AMDHD1	Expression profiling by array	GPL7191	GSE12511	Global Expression Profiling in Atopic Eczema Reveals Reciprocal Expression of Inflammatory and Lipid Genes	Plos One
Atopic Eczema	YPEL1	Expression profiling by array	GPL7191	GSE12511	Global Expression Profiling in Atopic Eczema Reveals Reciprocal Expression of Inflammatory and Lipid Genes	Plos One
Atopic Eczema	RPS3A	Expression profiling by array	GPL7191	GSE12511	Global Expression Profiling in Atopic Eczema Reveals Reciprocal Expression of Inflammatory and Lipid Genes	Plos One
Atopic Eczema	PLXNC1	Expression profiling by array	GPL7191	GSE12511	Global Expression Profiling in Atopic Eczema Reveals Reciprocal Expression of Inflammatory and Lipid Genes	Plos One
Atopic Eczema	C4ORF12	Expression profiling by array	GPL7191	GSE12511	Global Expression Profiling in Atopic Eczema Reveals Reciprocal Expression of Inflammatory and Lipid Genes	Plos One
Atopic Eczema	NRBP1	Expression profiling by array	GPL7191	GSE12511	Global Expression Profiling in Atopic Eczema Reveals Reciprocal Expression of Inflammatory and Lipid Genes	Plos One
Atopic Eczema	SLC43A1	Expression profiling by array	GPL7191	GSE12511	Global Expression Profiling in Atopic Eczema Reveals Reciprocal Expression of Inflammatory and Lipid Genes	Plos One
Atopic Eczema	RANGAP1	Expression profiling by array	GPL7191	GSE12511	Global Expression Profiling in Atopic Eczema Reveals Reciprocal Expression of Inflammatory and Lipid Genes	Plos One
Atopic Eczema	CCDC55	Expression profiling by array	GPL7191	GSE12511	Global Expression Profiling in Atopic Eczema Reveals Reciprocal Expression of Inflammatory and Lipid Genes	Plos One
Atopic Eczema	TCF12	Expression profiling by array	GPL7191	GSE12511	Global Expression Profiling in Atopic Eczema Reveals Reciprocal Expression of Inflammatory and Lipid Genes	Plos One
Atopic Eczema	GCNT3	Expression profiling by array	GPL7191	GSE12511	Global Expression Profiling in Atopic Eczema Reveals Reciprocal Expression of Inflammatory and Lipid Genes	Plos One
Atopic Eczema	SC4MOL	Expression profiling by array	GPL7191	GSE12511	Global Expression Profiling in Atopic Eczema Reveals Reciprocal Expression of Inflammatory and Lipid Genes	Plos One
Atopic Eczema	CCDC12	Expression profiling by array	GPL7191	GSE12511	Global Expression Profiling in Atopic Eczema Reveals Reciprocal Expression of Inflammatory and Lipid Genes	Plos One
Atopic Eczema	ADRM1	Expression profiling by array	GPL7191	GSE12511	Global Expression Profiling in Atopic Eczema Reveals Reciprocal Expression of Inflammatory and Lipid Genes	Plos One
Atopic Eczema	OS9	Expression profiling by array	GPL7191	GSE12511	Global Expression Profiling in Atopic Eczema Reveals Reciprocal Expression of Inflammatory and Lipid Genes	Plos One
Atopic Eczema	VAV2	Expression profiling by array	GPL7191	GSE12511	Global Expression Profiling in Atopic Eczema Reveals Reciprocal Expression of Inflammatory and Lipid Genes	Plos One
Atopic Eczema	SCARA5	Expression profiling by array	GPL7191	GSE12511	Global Expression Profiling in Atopic Eczema Reveals Reciprocal Expression of Inflammatory and Lipid Genes	Plos One
Atopic Eczema	ZMYM5	Expression profiling by array	GPL7191	GSE12511	Global Expression Profiling in Atopic Eczema Reveals Reciprocal Expression of Inflammatory and Lipid Genes	Plos One
Atopic Eczema	SRXN1	Expression profiling by array	GPL7191	GSE12511	Global Expression Profiling in Atopic Eczema Reveals Reciprocal Expression of Inflammatory and Lipid Genes	Plos One
Atopic Eczema	ATG16L1	Expression profiling by array	GPL7191	GSE12511	Global Expression Profiling in Atopic Eczema Reveals Reciprocal Expression of Inflammatory and Lipid Genes	Plos One
Atopic Eczema	FAM82C	Expression profiling by array	GPL7191	GSE12511	Global Expression Profiling in Atopic Eczema Reveals Reciprocal Expression of Inflammatory and Lipid Genes	Plos One
Atopic Eczema	PSEN2	Expression profiling by array	GPL7191	GSE12511	Global Expression Profiling in Atopic Eczema Reveals Reciprocal Expression of Inflammatory and Lipid Genes	Plos One
Atopic Eczema	TAF5L	Expression profiling by array	GPL7191	GSE12511	Global Expression Profiling in Atopic Eczema Reveals Reciprocal Expression of Inflammatory and Lipid Genes	Plos One
Atopic Eczema	CBR1	Expression profiling by array	GPL7191	GSE12511	Global Expression Profiling in Atopic Eczema Reveals Reciprocal Expression of Inflammatory and Lipid Genes	Plos One
Atopic Eczema	JARID1A	Expression profiling by array	GPL7191	GSE12511	Global Expression Profiling in Atopic Eczema Reveals Reciprocal Expression of Inflammatory and Lipid Genes	Plos One
Atopic Eczema	TFE3	Expression profiling by array	GPL7191	GSE12511	Global Expression Profiling in Atopic Eczema Reveals Reciprocal Expression of Inflammatory and Lipid Genes	Plos One
Atopic Eczema	RPH3AL	Expression profiling by array	GPL7191	GSE12511	Global Expression Profiling in Atopic Eczema Reveals Reciprocal Expression of Inflammatory and Lipid Genes	Plos One
Atopic Eczema	EPHB6	Expression profiling by array	GPL7191	GSE12511	Global Expression Profiling in Atopic Eczema Reveals Reciprocal Expression of Inflammatory and Lipid Genes	Plos One
Atopic Eczema	CDC34	Expression profiling by array	GPL7191	GSE12511	Global Expression Profiling in Atopic Eczema Reveals Reciprocal Expression of Inflammatory and Lipid Genes	Plos One
Atopic Eczema	CLCN3	Expression profiling by array	GPL7191	GSE12511	Global Expression Profiling in Atopic Eczema Reveals Reciprocal Expression of Inflammatory and Lipid Genes	Plos One
Atopic Eczema	PLEKHA5	Expression profiling by array	GPL7191	GSE12511	Global Expression Profiling in Atopic Eczema Reveals Reciprocal Expression of Inflammatory and Lipid Genes	Plos One
Atopic Eczema	POU3F1	Expression profiling by array	GPL7191	GSE12511	Global Expression Profiling in Atopic Eczema Reveals Reciprocal Expression of Inflammatory and Lipid Genes	Plos One
Atopic Eczema	C12ORF44	Expression profiling by array	GPL7191	GSE12511	Global Expression Profiling in Atopic Eczema Reveals Reciprocal Expression of Inflammatory and Lipid Genes	Plos One
Atopic Eczema	C6ORF160	Expression profiling by array	GPL7191	GSE12511	Global Expression Profiling in Atopic Eczema Reveals Reciprocal Expression of Inflammatory and Lipid Genes	Plos One
Atopic Eczema	PCDHA6	Expression profiling by array	GPL7191	GSE12511	Global Expression Profiling in Atopic Eczema Reveals Reciprocal Expression of Inflammatory and Lipid Genes	Plos One
Atopic Eczema	MGC15523	Expression profiling by array	GPL7191	GSE12511	Global Expression Profiling in Atopic Eczema Reveals Reciprocal Expression of Inflammatory and Lipid Genes	Plos One
Atopic Eczema	ATP1A1	Expression profiling by array	GPL7191	GSE12511	Global Expression Profiling in Atopic Eczema Reveals Reciprocal Expression of Inflammatory and Lipid Genes	Plos One
Atopic Eczema	GPR111	Expression profiling by array	GPL7191	GSE12511	Global Expression Profiling in Atopic Eczema Reveals Reciprocal Expression of Inflammatory and Lipid Genes	Plos One
Atopic Eczema	AA676696	Expression profiling by array	GPL7191	GSE12511	Global Expression Profiling in Atopic Eczema Reveals Reciprocal Expression of Inflammatory and Lipid Genes	Plos One
Atopic Eczema	LETMD1	Expression profiling by array	GPL7191	GSE12511	Global Expression Profiling in Atopic Eczema Reveals Reciprocal Expression of Inflammatory and Lipid Genes	Plos One
Atopic Eczema	EIF4A1	Expression profiling by array	GPL7191	GSE12511	Global Expression Profiling in Atopic Eczema Reveals Reciprocal Expression of Inflammatory and Lipid Genes	Plos One

Atopic Eczema	FAM51A1	Expression profiling by array	GPL7191	GSE12511	Global Expression Profiling in Atopic Eczema Reveals Reciprocal Expression of Inflammatory and Lipid Genes	Plos One
Atopic Eczema	BST1	Expression profiling by array	GPL7191	GSE12511	Global Expression Profiling in Atopic Eczema Reveals Reciprocal Expression of Inflammatory and Lipid Genes	Plos One
Atopic Eczema	FSCN1	Expression profiling by array	GPL7191	GSE12511	Global Expression Profiling in Atopic Eczema Reveals Reciprocal Expression of Inflammatory and Lipid Genes	Plos One
Atopic Eczema	USP46	Expression profiling by array	GPL7191	GSE12511	Global Expression Profiling in Atopic Eczema Reveals Reciprocal Expression of Inflammatory and Lipid Genes	Plos One
Atopic Eczema	OAS2	Expression profiling by array	GPL7191	GSE12511	Global Expression Profiling in Atopic Eczema Reveals Reciprocal Expression of Inflammatory and Lipid Genes	Plos One
Atopic Eczema	RPS16	Expression profiling by array	GPL7191	GSE12511	Global Expression Profiling in Atopic Eczema Reveals Reciprocal Expression of Inflammatory and Lipid Genes	Plos One
Atopic Eczema	DLG1	Expression profiling by array	GPL7191	GSE12511	Global Expression Profiling in Atopic Eczema Reveals Reciprocal Expression of Inflammatory and Lipid Genes	Plos One
Atopic Eczema	CTDSP2	Expression profiling by array	GPL7191	GSE12511	Global Expression Profiling in Atopic Eczema Reveals Reciprocal Expression of Inflammatory and Lipid Genes	Plos One
Atopic Eczema	RBM25	Expression profiling by array	GPL7191	GSE12511	Global Expression Profiling in Atopic Eczema Reveals Reciprocal Expression of Inflammatory and Lipid Genes	Plos One
Atopic Eczema	VISA	Expression profiling by array	GPL7191	GSE12511	Global Expression Profiling in Atopic Eczema Reveals Reciprocal Expression of Inflammatory and Lipid Genes	Plos One
Atopic Eczema	WNT7A	Expression profiling by array	GPL7191	GSE12511	Global Expression Profiling in Atopic Eczema Reveals Reciprocal Expression of Inflammatory and Lipid Genes	Plos One
Atopic Eczema	C20ORF3	Expression profiling by array	GPL7191	GSE12511	Global Expression Profiling in Atopic Eczema Reveals Reciprocal Expression of Inflammatory and Lipid Genes	Plos One
Atopic Eczema	RNF187	Expression profiling by array	GPL7191	GSE12511	Global Expression Profiling in Atopic Eczema Reveals Reciprocal Expression of Inflammatory and Lipid Genes	Plos One
Atopic Eczema	LOC653071	Expression profiling by array	GPL7191	GSE12511	Global Expression Profiling in Atopic Eczema Reveals Reciprocal Expression of Inflammatory and Lipid Genes	Plos One
Atopic Eczema	AA628230	Expression profiling by array	GPL7191	GSE12511	Global Expression Profiling in Atopic Eczema Reveals Reciprocal Expression of Inflammatory and Lipid Genes	Plos One
Atopic Eczema	DIO2	Expression profiling by array	GPL7191	GSE12511	Global Expression Profiling in Atopic Eczema Reveals Reciprocal Expression of Inflammatory and Lipid Genes	Plos One
Atopic Eczema	LOC646858	Expression profiling by array	GPL7191	GSE12511	Global Expression Profiling in Atopic Eczema Reveals Reciprocal Expression of Inflammatory and Lipid Genes	Plos One
Atopic Eczema	KNDC1	Expression profiling by array	GPL7191	GSE12511	Global Expression Profiling in Atopic Eczema Reveals Reciprocal Expression of Inflammatory and Lipid Genes	Plos One
Atopic Eczema	UBE2R2	Expression profiling by array	GPL7191	GSE12511	Global Expression Profiling in Atopic Eczema Reveals Reciprocal Expression of Inflammatory and Lipid Genes	Plos One
Atopic Eczema	PARP14	Expression profiling by array	GPL7191	GSE12511	Global Expression Profiling in Atopic Eczema Reveals Reciprocal Expression of Inflammatory and Lipid Genes	Plos One
Atopic Eczema	TTC18	Expression profiling by array	GPL7191	GSE12511	Global Expression Profiling in Atopic Eczema Reveals Reciprocal Expression of Inflammatory and Lipid Genes	Plos One
Atopic Eczema	HOP	Expression profiling by array	GPL7191	GSE12511	Global Expression Profiling in Atopic Eczema Reveals Reciprocal Expression of Inflammatory and Lipid Genes	Plos One
Atopic Eczema	SH3KBP1	Expression profiling by array	GPL7191	GSE12511	Global Expression Profiling in Atopic Eczema Reveals Reciprocal Expression of Inflammatory and Lipid Genes	Plos One
Atopic Eczema	PKN1	Expression profiling by array	GPL7191	GSE12511	Global Expression Profiling in Atopic Eczema Reveals Reciprocal Expression of Inflammatory and Lipid Genes	Plos One
Atopic Eczema	PHLDA2	Expression profiling by array	GPL7191	GSE12511	Global Expression Profiling in Atopic Eczema Reveals Reciprocal Expression of Inflammatory and Lipid Genes	Plos One
Atopic Eczema	HOXD9	Expression profiling by array	GPL7191	GSE12511	Global Expression Profiling in Atopic Eczema Reveals Reciprocal Expression of Inflammatory and Lipid Genes	Plos One
Atopic Eczema	RAB37	Expression profiling by array	GPL7191	GSE12511	Global Expression Profiling in Atopic Eczema Reveals Reciprocal Expression of Inflammatory and Lipid Genes	Plos One
Atopic Eczema	MGC39606	Expression profiling by array	GPL7191	GSE12511	Global Expression Profiling in Atopic Eczema Reveals Reciprocal Expression of Inflammatory and Lipid Genes	Plos One
Atopic Eczema	MAP3K8	Expression profiling by array	GPL7191	GSE12511	Global Expression Profiling in Atopic Eczema Reveals Reciprocal Expression of Inflammatory and Lipid Genes	Plos One
Atopic Eczema	MAGI1	Expression profiling by array	GPL7191	GSE12511	Global Expression Profiling in Atopic Eczema Reveals Reciprocal Expression of Inflammatory and Lipid Genes	Plos One
Atopic Eczema	AA777753	Expression profiling by array	GPL7191	GSE12511	Global Expression Profiling in Atopic Eczema Reveals Reciprocal Expression of Inflammatory and Lipid Genes	Plos One
Atopic Eczema	LOC221442	Expression profiling by array	GPL7191	GSE12511	Global Expression Profiling in Atopic Eczema Reveals Reciprocal Expression of Inflammatory and Lipid Genes	Plos One
Atopic Eczema	PPFIBP2	Expression profiling by array	GPL7191	GSE12511	Global Expression Profiling in Atopic Eczema Reveals Reciprocal Expression of Inflammatory and Lipid Genes	Plos One
Atopic Eczema	PSMD11	Expression profiling by array	GPL7191	GSE12511	Global Expression Profiling in Atopic Eczema Reveals Reciprocal Expression of Inflammatory and Lipid Genes	Plos One
Atopic Eczema	PTD004	Expression profiling by array	GPL7191	GSE12511	Global Expression Profiling in Atopic Eczema Reveals Reciprocal Expression of Inflammatory and Lipid Genes	Plos One
Atopic Eczema	RNF150	Expression profiling by array	GPL7191	GSE12511	Global Expression Profiling in Atopic Eczema Reveals Reciprocal Expression of Inflammatory and Lipid Genes	Plos One
Atopic Eczema	FLJ36031	Expression profiling by array	GPL7191	GSE12511	Global Expression Profiling in Atopic Eczema Reveals Reciprocal Expression of Inflammatory and Lipid Genes	Plos One
Atopic Eczema	C1ORF31	Expression profiling by array	GPL7191	GSE12511	Global Expression Profiling in Atopic Eczema Reveals Reciprocal Expression of Inflammatory and Lipid Genes	Plos One
Atopic Eczema	C14ORF138	Expression profiling by array	GPL7191	GSE12511	Global Expression Profiling in Atopic Eczema Reveals Reciprocal Expression of Inflammatory and Lipid Genes	Plos One
Atopic Eczema	POGZ	Expression profiling by array	GPL7191	GSE12511	Global Expression Profiling in Atopic Eczema Reveals Reciprocal Expression of Inflammatory and Lipid Genes	Plos One
Atopic Eczema	AUP1	Expression profiling by array	GPL7191	GSE12511	Global Expression Profiling in Atopic Eczema Reveals Reciprocal Expression of Inflammatory and Lipid Genes	Plos One
Atopic Eczema	C11ORF17	Expression profiling by array	GPL7191	GSE12511	Global Expression Profiling in Atopic Eczema Reveals Reciprocal Expression of Inflammatory and Lipid Genes	Plos One
Atopic Eczema	NTN4	Expression profiling by array	GPL7191	GSE12511	Global Expression Profiling in Atopic Eczema Reveals Reciprocal Expression of Inflammatory and Lipid Genes	Plos One
Atopic Eczema	PARL	Expression profiling by array	GPL7191	GSE12511	Global Expression Profiling in Atopic Eczema Reveals Reciprocal Expression of Inflammatory and Lipid Genes	Plos One
Atopic Eczema	DGCR14	Expression profiling by array	GPL7191	GSE12511	Global Expression Profiling in Atopic Eczema Reveals Reciprocal Expression of Inflammatory and Lipid Genes	Plos One
Atopic Eczema	SMTN	Expression profiling by array	GPL7191	GSE12511	Global Expression Profiling in Atopic Eczema Reveals Reciprocal Expression of Inflammatory and Lipid Genes	Plos One
Atopic Eczema	CCDC73	Expression profiling by array	GPL7191	GSE12511	Global Expression Profiling in Atopic Eczema Reveals Reciprocal Expression of Inflammatory and Lipid Genes	Plos One
Atopic Eczema	FLOT1	Expression profiling by array	GPL7191	GSE12511	Global Expression Profiling in Atopic Eczema Reveals Reciprocal Expression of Inflammatory and Lipid Genes	Plos One
Atopic Eczema	AUH	Expression profiling by array	GPL7191	GSE12511	Global Expression Profiling in Atopic Eczema Reveals Reciprocal Expression of Inflammatory and Lipid Genes	Plos One
Atopic Eczema	AKR1B1	Expression profiling by array	GPL7191	GSE12511	Global Expression Profiling in Atopic Eczema Reveals Reciprocal Expression of Inflammatory and Lipid Genes	Plos One
Atopic Eczema	CCNB2	Expression profiling by array	GPL7191	GSE12511	Global Expression Profiling in Atopic Eczema Reveals Reciprocal Expression of Inflammatory and Lipid Genes	Plos One
Atopic Eczema	SLC15A2	Expression profiling by array	GPL7191	GSE12511	Global Expression Profiling in Atopic Eczema Reveals Reciprocal Expression of Inflammatory and Lipid Genes	Plos One
Atopic Eczema	GREB1	Expression profiling by array	GPL7191	GSE12511	Global Expression Profiling in Atopic Eczema Reveals Reciprocal Expression of Inflammatory and Lipid Genes	Plos One
Atopic Eczema	MMP9	Expression profiling by array	GPL7191	GSE12511	Global Expression Profiling in Atopic Eczema Reveals Reciprocal Expression of Inflammatory and Lipid Genes	Plos One
Atopic Eczema	FLJ16636	Expression profiling by array	GPL7191	GSE12511	Global Expression Profiling in Atopic Eczema Reveals Reciprocal Expression of Inflammatory and Lipid Genes	Plos One
Atopic Eczema	ITPR2	Expression profiling by array	GPL7191	GSE12511	Global Expression Profiling in Atopic Eczema Reveals Reciprocal Expression of Inflammatory and Lipid Genes	Plos One
Atopic Eczema	STK17B	Expression profiling by array	GPL7191	GSE12511	Global Expression Profiling in Atopic Eczema Reveals Reciprocal Expression of Inflammatory and Lipid Genes	Plos One
Atopic Eczema	IL17RE	Expression profiling by array	GPL7191	GSE12511	Global Expression Profiling in Atopic Eczema Reveals Reciprocal Expression of Inflammatory and Lipid Genes	Plos One
Atopic Eczema	MDFIC	Expression profiling by array	GPL7191	GSE12511	Global Expression Profiling in Atopic Eczema Reveals Reciprocal Expression of Inflammatory and Lipid Genes	Plos One
Atopic Eczema	LOC253981	Expression profiling by array	GPL7191	GSE12511	Global Expression Profiling in Atopic Eczema Reveals Reciprocal Expression of Inflammatory and Lipid Genes	Plos One
Atopic Eczema	PSD3	Expression profiling by array	GPL7191	GSE12511	Global Expression Profiling in Atopic Eczema Reveals Reciprocal Expression of Inflammatory and Lipid Genes	Plos One
Atopic Eczema	W52248	Expression profiling by array	GPL7191	GSE12511	Global Expression Profiling in Atopic Eczema Reveals Reciprocal Expression of Inflammatory and Lipid Genes	Plos One

Atopic Eczema	LOC23117	Expression profiling by array	GPL7191	GSE12511	Global Expression Profiling in Atopic Eczema Reveals Reciprocal Expression of Inflammatory and Lipid Genes	Plos One
Atopic Eczema	AA573663	Expression profiling by array	GPL7191	GSE12511	Global Expression Profiling in Atopic Eczema Reveals Reciprocal Expression of Inflammatory and Lipid Genes	Plos One
Atopic Eczema	WWC1	Expression profiling by array	GPL7191	GSE12511	Global Expression Profiling in Atopic Eczema Reveals Reciprocal Expression of Inflammatory and Lipid Genes	Plos One
Atopic Eczema	DUS3L	Expression profiling by array	GPL7191	GSE12511	Global Expression Profiling in Atopic Eczema Reveals Reciprocal Expression of Inflammatory and Lipid Genes	Plos One
Atopic Eczema	C4ORF22	Expression profiling by array	GPL7191	GSE12511	Global Expression Profiling in Atopic Eczema Reveals Reciprocal Expression of Inflammatory and Lipid Genes	Plos One
Atopic Eczema	C8ORF1	Expression profiling by array	GPL7191	GSE12511	Global Expression Profiling in Atopic Eczema Reveals Reciprocal Expression of Inflammatory and Lipid Genes	Plos One
Atopic Eczema	KIAA0100	Expression profiling by array	GPL7191	GSE12511	Global Expression Profiling in Atopic Eczema Reveals Reciprocal Expression of Inflammatory and Lipid Genes	Plos One
Atopic Eczema	NR3C1	Expression profiling by array	GPL7191	GSE12511	Global Expression Profiling in Atopic Eczema Reveals Reciprocal Expression of Inflammatory and Lipid Genes	Plos One
Atopic Eczema	KRTAP4-7	Expression profiling by array	GPL7191	GSE12511	Global Expression Profiling in Atopic Eczema Reveals Reciprocal Expression of Inflammatory and Lipid Genes	Plos One
Atopic Eczema	ICOS	Expression profiling by array	GPL7191	GSE12511	Global Expression Profiling in Atopic Eczema Reveals Reciprocal Expression of Inflammatory and Lipid Genes	Plos One
Atopic Eczema	BRI3BP	Expression profiling by array	GPL7191	GSE12511	Global Expression Profiling in Atopic Eczema Reveals Reciprocal Expression of Inflammatory and Lipid Genes	Plos One
Atopic Eczema	MMP7	Expression profiling by array	GPL7191	GSE12511	Global Expression Profiling in Atopic Eczema Reveals Reciprocal Expression of Inflammatory and Lipid Genes	Plos One
Atopic Eczema	TBXAS1	Expression profiling by array	GPL7191	GSE12511	Global Expression Profiling in Atopic Eczema Reveals Reciprocal Expression of Inflammatory and Lipid Genes	Plos One
Atopic Eczema	NPHP1	Expression profiling by array	GPL7191	GSE12511	Global Expression Profiling in Atopic Eczema Reveals Reciprocal Expression of Inflammatory and Lipid Genes	Plos One
Atopic Eczema	FXR2	Expression profiling by array	GPL7191	GSE12511	Global Expression Profiling in Atopic Eczema Reveals Reciprocal Expression of Inflammatory and Lipid Genes	Plos One
Atopic Eczema	SLC7A8	Expression profiling by array	GPL7191	GSE12511	Global Expression Profiling in Atopic Eczema Reveals Reciprocal Expression of Inflammatory and Lipid Genes	Plos One
Atopic Eczema	LOC652651	Expression profiling by array	GPL7191	GSE12511	Global Expression Profiling in Atopic Eczema Reveals Reciprocal Expression of Inflammatory and Lipid Genes	Plos One
Atopic Eczema	DOCK11	Expression profiling by array	GPL7191	GSE12511	Global Expression Profiling in Atopic Eczema Reveals Reciprocal Expression of Inflammatory and Lipid Genes	Plos One
Atopic Eczema	LOC399959	Expression profiling by array	GPL7191	GSE12511	Global Expression Profiling in Atopic Eczema Reveals Reciprocal Expression of Inflammatory and Lipid Genes	Plos One
Atopic Eczema	CYP19A1	Expression profiling by array	GPL7191	GSE12511	Global Expression Profiling in Atopic Eczema Reveals Reciprocal Expression of Inflammatory and Lipid Genes	Plos One
Atopic Eczema	MRPL32	Expression profiling by array	GPL7191	GSE12511	Global Expression Profiling in Atopic Eczema Reveals Reciprocal Expression of Inflammatory and Lipid Genes	Plos One
Atopic Eczema	TSC22D1	Expression profiling by array	GPL7191	GSE12511	Global Expression Profiling in Atopic Eczema Reveals Reciprocal Expression of Inflammatory and Lipid Genes	Plos One
Atopic Eczema	ANK3	Expression profiling by array	GPL7191	GSE12511	Global Expression Profiling in Atopic Eczema Reveals Reciprocal Expression of Inflammatory and Lipid Genes	Plos One
Atopic Eczema	CHFR	Expression profiling by array	GPL7191	GSE12511	Global Expression Profiling in Atopic Eczema Reveals Reciprocal Expression of Inflammatory and Lipid Genes	Plos One
Atopic Eczema	G3BP	Expression profiling by array	GPL7191	GSE12511	Global Expression Profiling in Atopic Eczema Reveals Reciprocal Expression of Inflammatory and Lipid Genes	Plos One
Atopic Eczema	N23708	Expression profiling by array	GPL7191	GSE12511	Global Expression Profiling in Atopic Eczema Reveals Reciprocal Expression of Inflammatory and Lipid Genes	Plos One
Atopic Eczema	HLA-DQB1	Expression profiling by array	GPL7191	GSE12511	Global Expression Profiling in Atopic Eczema Reveals Reciprocal Expression of Inflammatory and Lipid Genes	Plos One
Atopic Eczema	TBC1D10B	Expression profiling by array	GPL7191	GSE12511	Global Expression Profiling in Atopic Eczema Reveals Reciprocal Expression of Inflammatory and Lipid Genes	Plos One
Atopic Eczema	LSM1	Expression profiling by array	GPL7191	GSE12511	Global Expression Profiling in Atopic Eczema Reveals Reciprocal Expression of Inflammatory and Lipid Genes	Plos One
Atopic Eczema	RUVBL1	Expression profiling by array	GPL7191	GSE12511	Global Expression Profiling in Atopic Eczema Reveals Reciprocal Expression of Inflammatory and Lipid Genes	Plos One
Atopic Eczema	LOC285500	Expression profiling by array	GPL7191	GSE12511	Global Expression Profiling in Atopic Eczema Reveals Reciprocal Expression of Inflammatory and Lipid Genes	Plos One
Atopic Eczema	CAPS2	Expression profiling by array	GPL7191	GSE12511	Global Expression Profiling in Atopic Eczema Reveals Reciprocal Expression of Inflammatory and Lipid Genes	Plos One
Atopic Eczema	PIN1	Expression profiling by array	GPL7191	GSE12511	Global Expression Profiling in Atopic Eczema Reveals Reciprocal Expression of Inflammatory and Lipid Genes	Plos One
Atopic Eczema	FAM38A	Expression profiling by array	GPL7191	GSE12511	Global Expression Profiling in Atopic Eczema Reveals Reciprocal Expression of Inflammatory and Lipid Genes	Plos One
Atopic Eczema	ITSN2	Expression profiling by array	GPL7191	GSE12511	Global Expression Profiling in Atopic Eczema Reveals Reciprocal Expression of Inflammatory and Lipid Genes	Plos One
Atopic Eczema	DLG5	Expression profiling by array	GPL7191	GSE12511	Global Expression Profiling in Atopic Eczema Reveals Reciprocal Expression of Inflammatory and Lipid Genes	Plos One
Atopic Eczema	LOC400451	Expression profiling by array	GPL7191	GSE12511	Global Expression Profiling in Atopic Eczema Reveals Reciprocal Expression of Inflammatory and Lipid Genes	Plos One
Atopic Eczema	MAOB	Expression profiling by array	GPL7191	GSE12511	Global Expression Profiling in Atopic Eczema Reveals Reciprocal Expression of Inflammatory and Lipid Genes	Plos One
Atopic Eczema	FBXL15	Expression profiling by array	GPL7191	GSE12511	Global Expression Profiling in Atopic Eczema Reveals Reciprocal Expression of Inflammatory and Lipid Genes	Plos One
Atopic Eczema	C6ORF152	Expression profiling by array	GPL7191	GSE12511	Global Expression Profiling in Atopic Eczema Reveals Reciprocal Expression of Inflammatory and Lipid Genes	Plos One
Atopic Eczema	NFATC1	Expression profiling by array	GPL7191	GSE12511	Global Expression Profiling in Atopic Eczema Reveals Reciprocal Expression of Inflammatory and Lipid Genes	Plos One
Atopic Eczema	MGC13017	Expression profiling by array	GPL7191	GSE12511	Global Expression Profiling in Atopic Eczema Reveals Reciprocal Expression of Inflammatory and Lipid Genes	Plos One
Atopic Eczema	FADD	Expression profiling by array	GPL7191	GSE12511	Global Expression Profiling in Atopic Eczema Reveals Reciprocal Expression of Inflammatory and Lipid Genes	Plos One
Atopic Eczema	RUFY2	Expression profiling by array	GPL7191	GSE12511	Global Expression Profiling in Atopic Eczema Reveals Reciprocal Expression of Inflammatory and Lipid Genes	Plos One
Atopic Eczema	SETD7	Expression profiling by array	GPL7191	GSE12511	Global Expression Profiling in Atopic Eczema Reveals Reciprocal Expression of Inflammatory and Lipid Genes	Plos One
Atopic Eczema	AI376502	Expression profiling by array	GPL7191	GSE12511	Global Expression Profiling in Atopic Eczema Reveals Reciprocal Expression of Inflammatory and Lipid Genes	Plos One
Atopic Eczema	CCDC107	Expression profiling by array	GPL7191	GSE12511	Global Expression Profiling in Atopic Eczema Reveals Reciprocal Expression of Inflammatory and Lipid Genes	Plos One
Atopic Eczema	DPT	Expression profiling by array	GPL7191	GSE12511	Global Expression Profiling in Atopic Eczema Reveals Reciprocal Expression of Inflammatory and Lipid Genes	Plos One
Atopic Eczema	ADCY8	Expression profiling by array	GPL7191	GSE12511	Global Expression Profiling in Atopic Eczema Reveals Reciprocal Expression of Inflammatory and Lipid Genes	Plos One
Atopic Eczema	LOC441127	Expression profiling by array	GPL7191	GSE12511	Global Expression Profiling in Atopic Eczema Reveals Reciprocal Expression of Inflammatory and Lipid Genes	Plos One
Atopic Eczema	RECK	Expression profiling by array	GPL7191	GSE12511	Global Expression Profiling in Atopic Eczema Reveals Reciprocal Expression of Inflammatory and Lipid Genes	Plos One
Atopic Eczema	RNF135	Expression profiling by array	GPL7191	GSE12511	Global Expression Profiling in Atopic Eczema Reveals Reciprocal Expression of Inflammatory and Lipid Genes	Plos One
Atopic Eczema	PVRL1	Expression profiling by array	GPL7191	GSE12511	Global Expression Profiling in Atopic Eczema Reveals Reciprocal Expression of Inflammatory and Lipid Genes	Plos One
Atopic Eczema	SLC2A9	Expression profiling by array	GPL7191	GSE12511	Global Expression Profiling in Atopic Eczema Reveals Reciprocal Expression of Inflammatory and Lipid Genes	Plos One
Atopic Eczema	SLC3A2	Expression profiling by array	GPL7191	GSE12511	Global Expression Profiling in Atopic Eczema Reveals Reciprocal Expression of Inflammatory and Lipid Genes	Plos One
Atopic Eczema	TMEM130	Expression profiling by array	GPL7191	GSE12511	Global Expression Profiling in Atopic Eczema Reveals Reciprocal Expression of Inflammatory and Lipid Genes	Plos One
Atopic Eczema	C6ORF108	Expression profiling by array	GPL7191	GSE12511	Global Expression Profiling in Atopic Eczema Reveals Reciprocal Expression of Inflammatory and Lipid Genes	Plos One
Atopic Eczema	SLC35E3	Expression profiling by array	GPL7191	GSE12511	Global Expression Profiling in Atopic Eczema Reveals Reciprocal Expression of Inflammatory and Lipid Genes	Plos One
Atopic Eczema	NDUFA11	Expression profiling by array	GPL7191	GSE12511	Global Expression Profiling in Atopic Eczema Reveals Reciprocal Expression of Inflammatory and Lipid Genes	Plos One
Atopic Eczema	OTUB2	Expression profiling by array	GPL7191	GSE12511	Global Expression Profiling in Atopic Eczema Reveals Reciprocal Expression of Inflammatory and Lipid Genes	Plos One
Atopic Eczema	COX18	Expression profiling by array	GPL7191	GSE12511	Global Expression Profiling in Atopic Eczema Reveals Reciprocal Expression of Inflammatory and Lipid Genes	Plos One
Atopic Eczema	HAPLN3	Expression profiling by array	GPL7191	GSE12511	Global Expression Profiling in Atopic Eczema Reveals Reciprocal Expression of Inflammatory and Lipid Genes	Plos One

Atopic Eczema	SIRT7	Expression profiling by array	GPL7191	GSE12511	Global Expression Profiling in Atopic Eczema Reveals Reciprocal Expression of Inflammatory and Lipid Genes	Plos One
Atopic Eczema	ST3GAL5	Expression profiling by array	GPL7191	GSE12511	Global Expression Profiling in Atopic Eczema Reveals Reciprocal Expression of Inflammatory and Lipid Genes	Plos One
Atopic Eczema	CALCRL	Expression profiling by array	GPL7191	GSE12511	Global Expression Profiling in Atopic Eczema Reveals Reciprocal Expression of Inflammatory and Lipid Genes	Plos One
Atopic Eczema	LOC161527	Expression profiling by array	GPL7191	GSE12511	Global Expression Profiling in Atopic Eczema Reveals Reciprocal Expression of Inflammatory and Lipid Genes	Plos One
Atopic Eczema	VKORC1L1	Expression profiling by array	GPL7191	GSE12511	Global Expression Profiling in Atopic Eczema Reveals Reciprocal Expression of Inflammatory and Lipid Genes	Plos One
Atopic Eczema	ADCK4	Expression profiling by array	GPL7191	GSE12511	Global Expression Profiling in Atopic Eczema Reveals Reciprocal Expression of Inflammatory and Lipid Genes	Plos One
Atopic Eczema	DCTN5	Expression profiling by array	GPL7191	GSE12511	Global Expression Profiling in Atopic Eczema Reveals Reciprocal Expression of Inflammatory and Lipid Genes	Plos One
Atopic Eczema	FBP2	Expression profiling by array	GPL7191	GSE12511	Global Expression Profiling in Atopic Eczema Reveals Reciprocal Expression of Inflammatory and Lipid Genes	Plos One
Atopic Eczema	TMCC1	Expression profiling by array	GPL7191	GSE12511	Global Expression Profiling in Atopic Eczema Reveals Reciprocal Expression of Inflammatory and Lipid Genes	Plos One
Atopic Eczema	USP49	Expression profiling by array	GPL7191	GSE12511	Global Expression Profiling in Atopic Eczema Reveals Reciprocal Expression of Inflammatory and Lipid Genes	Plos One
Atopic Eczema	ARHGAP22	Expression profiling by array	GPL7191	GSE12511	Global Expression Profiling in Atopic Eczema Reveals Reciprocal Expression of Inflammatory and Lipid Genes	Plos One
Atopic Eczema	AA598859	Expression profiling by array	GPL7191	GSE12511	Global Expression Profiling in Atopic Eczema Reveals Reciprocal Expression of Inflammatory and Lipid Genes	Plos One
Atopic Eczema	RBMS1	Expression profiling by array	GPL7191	GSE12511	Global Expression Profiling in Atopic Eczema Reveals Reciprocal Expression of Inflammatory and Lipid Genes	Plos One
Atopic Eczema	RRAGD	Expression profiling by array	GPL7191	GSE12511	Global Expression Profiling in Atopic Eczema Reveals Reciprocal Expression of Inflammatory and Lipid Genes	Plos One
Atopic Eczema	ARPC2	Expression profiling by array	GPL7191	GSE12511	Global Expression Profiling in Atopic Eczema Reveals Reciprocal Expression of Inflammatory and Lipid Genes	Plos One
Atopic Eczema	AKR7A2	Expression profiling by array	GPL7191	GSE12511	Global Expression Profiling in Atopic Eczema Reveals Reciprocal Expression of Inflammatory and Lipid Genes	Plos One
Atopic Eczema	IFT122	Expression profiling by array	GPL7191	GSE12511	Global Expression Profiling in Atopic Eczema Reveals Reciprocal Expression of Inflammatory and Lipid Genes	Plos One
Atopic Eczema	DGAT2	Expression profiling by array	GPL7191	GSE12511	Global Expression Profiling in Atopic Eczema Reveals Reciprocal Expression of Inflammatory and Lipid Genes	Plos One
Atopic Eczema	THOC1	Expression profiling by array	GPL7191	GSE12511	Global Expression Profiling in Atopic Eczema Reveals Reciprocal Expression of Inflammatory and Lipid Genes	Plos One
Atopic Eczema	CASP3	Expression profiling by array	GPL7191	GSE12511	Global Expression Profiling in Atopic Eczema Reveals Reciprocal Expression of Inflammatory and Lipid Genes	Plos One
Atopic Eczema	MIR16	Expression profiling by array	GPL7191	GSE12511	Global Expression Profiling in Atopic Eczema Reveals Reciprocal Expression of Inflammatory and Lipid Genes	Plos One
Atopic Eczema	POLR2J2	Expression profiling by array	GPL7191	GSE12511	Global Expression Profiling in Atopic Eczema Reveals Reciprocal Expression of Inflammatory and Lipid Genes	Plos One
Atopic Eczema	DR1	Expression profiling by array	GPL7191	GSE12511	Global Expression Profiling in Atopic Eczema Reveals Reciprocal Expression of Inflammatory and Lipid Genes	Plos One
Atopic Eczema	LOC401152	Expression profiling by array	GPL7191	GSE12511	Global Expression Profiling in Atopic Eczema Reveals Reciprocal Expression of Inflammatory and Lipid Genes	Plos One
Atopic Eczema	SNRPE	Expression profiling by array	GPL7191	GSE12511	Global Expression Profiling in Atopic Eczema Reveals Reciprocal Expression of Inflammatory and Lipid Genes	Plos One
Atopic Eczema	KIAA1961	Expression profiling by array	GPL7191	GSE12511	Global Expression Profiling in Atopic Eczema Reveals Reciprocal Expression of Inflammatory and Lipid Genes	Plos One
Atopic Eczema	YWHAH	Expression profiling by array	GPL7191	GSE12511	Global Expression Profiling in Atopic Eczema Reveals Reciprocal Expression of Inflammatory and Lipid Genes	Plos One
Atopic Eczema	ZKSCAN1	Expression profiling by array	GPL7191	GSE12511	Global Expression Profiling in Atopic Eczema Reveals Reciprocal Expression of Inflammatory and Lipid Genes	Plos One
Atopic Eczema	ZNF467	Expression profiling by array	GPL7191	GSE12511	Global Expression Profiling in Atopic Eczema Reveals Reciprocal Expression of Inflammatory and Lipid Genes	Plos One
Atopic Eczema	SCUBE3	Expression profiling by array	GPL7191	GSE12511	Global Expression Profiling in Atopic Eczema Reveals Reciprocal Expression of Inflammatory and Lipid Genes	Plos One
Atopic Eczema	CAST1	Expression profiling by array	GPL7191	GSE12511	Global Expression Profiling in Atopic Eczema Reveals Reciprocal Expression of Inflammatory and Lipid Genes	Plos One
Atopic Eczema	NES	Expression profiling by array	GPL7191	GSE12511	Global Expression Profiling in Atopic Eczema Reveals Reciprocal Expression of Inflammatory and Lipid Genes	Plos One
Atopic Eczema	MT1F	Expression profiling by array	GPL7191	GSE12511	Global Expression Profiling in Atopic Eczema Reveals Reciprocal Expression of Inflammatory and Lipid Genes	Plos One
Atopic Eczema	ROBO1	Expression profiling by array	GPL7191	GSE12511	Global Expression Profiling in Atopic Eczema Reveals Reciprocal Expression of Inflammatory and Lipid Genes	Plos One
Atopic Eczema	DICER1	Expression profiling by array	GPL7191	GSE12511	Global Expression Profiling in Atopic Eczema Reveals Reciprocal Expression of Inflammatory and Lipid Genes	Plos One
Atopic Eczema	FOXJ2	Expression profiling by array	GPL7191	GSE12511	Global Expression Profiling in Atopic Eczema Reveals Reciprocal Expression of Inflammatory and Lipid Genes	Plos One
Atopic Eczema	MGC13114	Expression profiling by array	GPL7191	GSE12511	Global Expression Profiling in Atopic Eczema Reveals Reciprocal Expression of Inflammatory and Lipid Genes	Plos One
Atopic Eczema	HCK	Expression profiling by array	GPL7191	GSE12511	Global Expression Profiling in Atopic Eczema Reveals Reciprocal Expression of Inflammatory and Lipid Genes	Plos One
Atopic Eczema	MGC14327	Expression profiling by array	GPL7191	GSE12511	Global Expression Profiling in Atopic Eczema Reveals Reciprocal Expression of Inflammatory and Lipid Genes	Plos One
Atopic Eczema	RALGDS	Expression profiling by array	GPL7191	GSE12511	Global Expression Profiling in Atopic Eczema Reveals Reciprocal Expression of Inflammatory and Lipid Genes	Plos One
Atopic Eczema	STYXL1	Expression profiling by array	GPL7191	GSE12511	Global Expression Profiling in Atopic Eczema Reveals Reciprocal Expression of Inflammatory and Lipid Genes	Plos One
Atopic Eczema	AOX1	Expression profiling by array	GPL7191	GSE12511	Global Expression Profiling in Atopic Eczema Reveals Reciprocal Expression of Inflammatory and Lipid Genes	Plos One
Atopic Eczema	DNAJC4	Expression profiling by array	GPL7191	GSE12511	Global Expression Profiling in Atopic Eczema Reveals Reciprocal Expression of Inflammatory and Lipid Genes	Plos One
Atopic Eczema	SGCD	Expression profiling by array	GPL7191	GSE12511	Global Expression Profiling in Atopic Eczema Reveals Reciprocal Expression of Inflammatory and Lipid Genes	Plos One
Atopic Eczema	IGHG1	Expression profiling by array	GPL7191	GSE12511	Global Expression Profiling in Atopic Eczema Reveals Reciprocal Expression of Inflammatory and Lipid Genes	Plos One
Atopic Eczema	AA680099	Expression profiling by array	GPL7191	GSE12511	Global Expression Profiling in Atopic Eczema Reveals Reciprocal Expression of Inflammatory and Lipid Genes	Plos One
Atopic Eczema	ARHGEF11	Expression profiling by array	GPL7191	GSE12511	Global Expression Profiling in Atopic Eczema Reveals Reciprocal Expression of Inflammatory and Lipid Genes	Plos One
Atopic Eczema	PPP1CA	Expression profiling by array	GPL7191	GSE12511	Global Expression Profiling in Atopic Eczema Reveals Reciprocal Expression of Inflammatory and Lipid Genes	Plos One
Atopic Eczema	TANC2	Expression profiling by array	GPL7191	GSE12511	Global Expression Profiling in Atopic Eczema Reveals Reciprocal Expression of Inflammatory and Lipid Genes	Plos One
Atopic Eczema	WDR33	Expression profiling by array	GPL7191	GSE12511	Global Expression Profiling in Atopic Eczema Reveals Reciprocal Expression of Inflammatory and Lipid Genes	Plos One
Atopic Eczema	AP3S1	Expression profiling by array	GPL7191	GSE12511	Global Expression Profiling in Atopic Eczema Reveals Reciprocal Expression of Inflammatory and Lipid Genes	Plos One
Atopic Eczema	MAL2	Expression profiling by array	GPL7191	GSE12511	Global Expression Profiling in Atopic Eczema Reveals Reciprocal Expression of Inflammatory and Lipid Genes	Plos One
Atopic Eczema	SATB2	Expression profiling by array	GPL7191	GSE12511	Global Expression Profiling in Atopic Eczema Reveals Reciprocal Expression of Inflammatory and Lipid Genes	Plos One
Atopic Eczema	SPAG17	Expression profiling by array	GPL7191	GSE12511	Global Expression Profiling in Atopic Eczema Reveals Reciprocal Expression of Inflammatory and Lipid Genes	Plos One
Atopic Eczema	TMEM144	Expression profiling by array	GPL7191	GSE12511	Global Expression Profiling in Atopic Eczema Reveals Reciprocal Expression of Inflammatory and Lipid Genes	Plos One
Atopic Eczema	HMOX2	Expression profiling by array	GPL7191	GSE12511	Global Expression Profiling in Atopic Eczema Reveals Reciprocal Expression of Inflammatory and Lipid Genes	Plos One
Atopic Eczema	FRG1	Expression profiling by array	GPL7191	GSE12511	Global Expression Profiling in Atopic Eczema Reveals Reciprocal Expression of Inflammatory and Lipid Genes	Plos One
Atopic Eczema	SEMA3C	Expression profiling by array	GPL7191	GSE12511	Global Expression Profiling in Atopic Eczema Reveals Reciprocal Expression of Inflammatory and Lipid Genes	Plos One
Atopic Eczema	R35849	Expression profiling by array	GPL7191	GSE12511	Global Expression Profiling in Atopic Eczema Reveals Reciprocal Expression of Inflammatory and Lipid Genes	Plos One
Atopic Eczema	CDK7	Expression profiling by array	GPL7191	GSE12511	Global Expression Profiling in Atopic Eczema Reveals Reciprocal Expression of Inflammatory and Lipid Genes	Plos One
Atopic Eczema	ATP6V1F	Expression profiling by array	GPL7191	GSE12511	Global Expression Profiling in Atopic Eczema Reveals Reciprocal Expression of Inflammatory and Lipid Genes	Plos One
Atopic Eczema	PDZD8	Expression profiling by array	GPL7191	GSE12511	Global Expression Profiling in Atopic Eczema Reveals Reciprocal Expression of Inflammatory and Lipid Genes	Plos One

Atopic Eczema	DDX28	Expression profiling by array	GPL7191	GSE12511	Global Expression Profiling in Atopic Eczema Reveals Reciprocal Expression of Inflammatory and Lipid Genes	Plos One
Atopic Eczema	RORA	Expression profiling by array	GPL7191	GSE12511	Global Expression Profiling in Atopic Eczema Reveals Reciprocal Expression of Inflammatory and Lipid Genes	Plos One
Atopic Eczema	AA992653	Expression profiling by array	GPL7191	GSE12511	Global Expression Profiling in Atopic Eczema Reveals Reciprocal Expression of Inflammatory and Lipid Genes	Plos One
Atopic Eczema	BAD	Expression profiling by array	GPL7191	GSE12511	Global Expression Profiling in Atopic Eczema Reveals Reciprocal Expression of Inflammatory and Lipid Genes	Plos One
Atopic Eczema	FLJ39378	Expression profiling by array	GPL7191	GSE12511	Global Expression Profiling in Atopic Eczema Reveals Reciprocal Expression of Inflammatory and Lipid Genes	Plos One
Atopic Eczema	AMID	Expression profiling by array	GPL7191	GSE12511	Global Expression Profiling in Atopic Eczema Reveals Reciprocal Expression of Inflammatory and Lipid Genes	Plos One
Atopic Eczema	DNM1	Expression profiling by array	GPL7191	GSE12511	Global Expression Profiling in Atopic Eczema Reveals Reciprocal Expression of Inflammatory and Lipid Genes	Plos One
Atopic Eczema	CDC2L6	Expression profiling by array	GPL7191	GSE12511	Global Expression Profiling in Atopic Eczema Reveals Reciprocal Expression of Inflammatory and Lipid Genes	Plos One
Atopic Eczema	SF1	Expression profiling by array	GPL7191	GSE12511	Global Expression Profiling in Atopic Eczema Reveals Reciprocal Expression of Inflammatory and Lipid Genes	Plos One
Atopic Eczema	CHRNB4	Expression profiling by array	GPL7191	GSE12511	Global Expression Profiling in Atopic Eczema Reveals Reciprocal Expression of Inflammatory and Lipid Genes	Plos One
Atopic Eczema	PCGF5	Expression profiling by array	GPL7191	GSE12511	Global Expression Profiling in Atopic Eczema Reveals Reciprocal Expression of Inflammatory and Lipid Genes	Plos One
Atopic Eczema	TIAL1	Expression profiling by array	GPL7191	GSE12511	Global Expression Profiling in Atopic Eczema Reveals Reciprocal Expression of Inflammatory and Lipid Genes	Plos One
Atopic Eczema	SYNPO2	Expression profiling by array	GPL7191	GSE12511	Global Expression Profiling in Atopic Eczema Reveals Reciprocal Expression of Inflammatory and Lipid Genes	Plos One
Atopic Eczema	LOC646621	Expression profiling by array	GPL7191	GSE12511	Global Expression Profiling in Atopic Eczema Reveals Reciprocal Expression of Inflammatory and Lipid Genes	Plos One
Atopic Eczema	SERTAD4	Expression profiling by array	GPL7191	GSE12511	Global Expression Profiling in Atopic Eczema Reveals Reciprocal Expression of Inflammatory and Lipid Genes	Plos One
Atopic Eczema	LOC653327	Expression profiling by array	GPL7191	GSE12511	Global Expression Profiling in Atopic Eczema Reveals Reciprocal Expression of Inflammatory and Lipid Genes	Plos One
Atopic Eczema	PGRMC1	Expression profiling by array	GPL7191	GSE12511	Global Expression Profiling in Atopic Eczema Reveals Reciprocal Expression of Inflammatory and Lipid Genes	Plos One
Atopic Eczema	KIAA0984	Expression profiling by array	GPL7191	GSE12511	Global Expression Profiling in Atopic Eczema Reveals Reciprocal Expression of Inflammatory and Lipid Genes	Plos One
Atopic Eczema	ZDHHC5	Expression profiling by array	GPL7191	GSE12511	Global Expression Profiling in Atopic Eczema Reveals Reciprocal Expression of Inflammatory and Lipid Genes	Plos One
Atopic Eczema	AMPH	Expression profiling by array	GPL7191	GSE12511	Global Expression Profiling in Atopic Eczema Reveals Reciprocal Expression of Inflammatory and Lipid Genes	Plos One
Atopic Eczema	ZNF451	Expression profiling by array	GPL7191	GSE12511	Global Expression Profiling in Atopic Eczema Reveals Reciprocal Expression of Inflammatory and Lipid Genes	Plos One
Atopic Eczema	CENPC1	Expression profiling by array	GPL7191	GSE12511	Global Expression Profiling in Atopic Eczema Reveals Reciprocal Expression of Inflammatory and Lipid Genes	Plos One
Atopic Eczema	UBE2C	Expression profiling by array	GPL7191	GSE12511	Global Expression Profiling in Atopic Eczema Reveals Reciprocal Expression of Inflammatory and Lipid Genes	Plos One
Atopic Eczema	SLC39A8	Expression profiling by array	GPL7191	GSE12511	Global Expression Profiling in Atopic Eczema Reveals Reciprocal Expression of Inflammatory and Lipid Genes	Plos One
Atopic Eczema	COL9A2	Expression profiling by array	GPL7191	GSE12511	Global Expression Profiling in Atopic Eczema Reveals Reciprocal Expression of Inflammatory and Lipid Genes	Plos One
Atopic Eczema	MNDA	Expression profiling by array	GPL7191	GSE12511	Global Expression Profiling in Atopic Eczema Reveals Reciprocal Expression of Inflammatory and Lipid Genes	Plos One
Atopic Eczema	LOC342892	Expression profiling by array	GPL7191	GSE12511	Global Expression Profiling in Atopic Eczema Reveals Reciprocal Expression of Inflammatory and Lipid Genes	Plos One
Atopic Eczema	LOC441383	Expression profiling by array	GPL7191	GSE12511	Global Expression Profiling in Atopic Eczema Reveals Reciprocal Expression of Inflammatory and Lipid Genes	Plos One
Atopic Eczema	ATF4	Expression profiling by array	GPL7191	GSE12511	Global Expression Profiling in Atopic Eczema Reveals Reciprocal Expression of Inflammatory and Lipid Genes	Plos One
Atopic Eczema	ST5	Expression profiling by array	GPL7191	GSE12511	Global Expression Profiling in Atopic Eczema Reveals Reciprocal Expression of Inflammatory and Lipid Genes	Plos One
Atopic Eczema	SLC26A5	Expression profiling by array	GPL7191	GSE12511	Global Expression Profiling in Atopic Eczema Reveals Reciprocal Expression of Inflammatory and Lipid Genes	Plos One
Atopic Eczema	LGALS1	Expression profiling by array	GPL7191	GSE12511	Global Expression Profiling in Atopic Eczema Reveals Reciprocal Expression of Inflammatory and Lipid Genes	Plos One
Atopic Eczema	KIAA0182	Expression profiling by array	GPL7191	GSE12511	Global Expression Profiling in Atopic Eczema Reveals Reciprocal Expression of Inflammatory and Lipid Genes	Plos One
Atopic Eczema	ENDOG	Expression profiling by array	GPL7191	GSE12511	Global Expression Profiling in Atopic Eczema Reveals Reciprocal Expression of Inflammatory and Lipid Genes	Plos One
Atopic Eczema	C19ORF15	Expression profiling by array	GPL7191	GSE12511	Global Expression Profiling in Atopic Eczema Reveals Reciprocal Expression of Inflammatory and Lipid Genes	Plos One
Atopic Eczema	BRI3	Expression profiling by array	GPL7191	GSE12511	Global Expression Profiling in Atopic Eczema Reveals Reciprocal Expression of Inflammatory and Lipid Genes	Plos One
Atopic Eczema	LOC113230	Expression profiling by array	GPL7191	GSE12511	Global Expression Profiling in Atopic Eczema Reveals Reciprocal Expression of Inflammatory and Lipid Genes	Plos One
Atopic Eczema	LOC285419	Expression profiling by array	GPL7191	GSE12511	Global Expression Profiling in Atopic Eczema Reveals Reciprocal Expression of Inflammatory and Lipid Genes	Plos One
Atopic Eczema	MTX2	Expression profiling by array	GPL7191	GSE12511	Global Expression Profiling in Atopic Eczema Reveals Reciprocal Expression of Inflammatory and Lipid Genes	Plos One
Atopic Eczema	ASTN2	Expression profiling by array	GPL7191	GSE12511	Global Expression Profiling in Atopic Eczema Reveals Reciprocal Expression of Inflammatory and Lipid Genes	Plos One
Atopic Eczema	GINS2	Expression profiling by array	GPL7191	GSE12511	Global Expression Profiling in Atopic Eczema Reveals Reciprocal Expression of Inflammatory and Lipid Genes	Plos One
Atopic Eczema	SSNA1	Expression profiling by array	GPL7191	GSE12511	Global Expression Profiling in Atopic Eczema Reveals Reciprocal Expression of Inflammatory and Lipid Genes	Plos One
Atopic Eczema	SRM	Expression profiling by array	GPL7191	GSE12511	Global Expression Profiling in Atopic Eczema Reveals Reciprocal Expression of Inflammatory and Lipid Genes	Plos One
Atopic Eczema	SLC16A5	Expression profiling by array	GPL7191	GSE12511	Global Expression Profiling in Atopic Eczema Reveals Reciprocal Expression of Inflammatory and Lipid Genes	Plos One
Atopic Eczema	SCC-112	Expression profiling by array	GPL7191	GSE12511	Global Expression Profiling in Atopic Eczema Reveals Reciprocal Expression of Inflammatory and Lipid Genes	Plos One
Atopic Eczema	CECR2	Expression profiling by array	GPL7191	GSE12511	Global Expression Profiling in Atopic Eczema Reveals Reciprocal Expression of Inflammatory and Lipid Genes	Plos One
Atopic Eczema	TMEM23	Expression profiling by array	GPL7191	GSE12511	Global Expression Profiling in Atopic Eczema Reveals Reciprocal Expression of Inflammatory and Lipid Genes	Plos One
Atopic Eczema	MTMR11	Expression profiling by array	GPL7191	GSE12511	Global Expression Profiling in Atopic Eczema Reveals Reciprocal Expression of Inflammatory and Lipid Genes	Plos One
Atopic Eczema	CPNE1	Expression profiling by array	GPL7191	GSE12511	Global Expression Profiling in Atopic Eczema Reveals Reciprocal Expression of Inflammatory and Lipid Genes	Plos One
Atopic Eczema	PSRC1	Expression profiling by array	GPL7191	GSE12511	Global Expression Profiling in Atopic Eczema Reveals Reciprocal Expression of Inflammatory and Lipid Genes	Plos One
Atopic Eczema	C16ORF72	Expression profiling by array	GPL7191	GSE12511	Global Expression Profiling in Atopic Eczema Reveals Reciprocal Expression of Inflammatory and Lipid Genes	Plos One
Atopic Eczema	AA913303	Expression profiling by array	GPL7191	GSE12511	Global Expression Profiling in Atopic Eczema Reveals Reciprocal Expression of Inflammatory and Lipid Genes	Plos One
Atopic Eczema	LAMC1	Expression profiling by array	GPL7191	GSE12511	Global Expression Profiling in Atopic Eczema Reveals Reciprocal Expression of Inflammatory and Lipid Genes	Plos One
Atopic Eczema	GOSR2	Expression profiling by array	GPL7191	GSE12511	Global Expression Profiling in Atopic Eczema Reveals Reciprocal Expression of Inflammatory and Lipid Genes	Plos One
Atopic Eczema	TRAF3IP2	Expression profiling by array	GPL7191	GSE12511	Global Expression Profiling in Atopic Eczema Reveals Reciprocal Expression of Inflammatory and Lipid Genes	Plos One
Atopic Eczema	PAWR	Expression profiling by array	GPL7191	GSE12511	Global Expression Profiling in Atopic Eczema Reveals Reciprocal Expression of Inflammatory and Lipid Genes	Plos One
Atopic Eczema	TGFBR3	Expression profiling by array	GPL7191	GSE12511	Global Expression Profiling in Atopic Eczema Reveals Reciprocal Expression of Inflammatory and Lipid Genes	Plos One
Atopic Eczema	RCN1	Expression profiling by array	GPL7191	GSE12511	Global Expression Profiling in Atopic Eczema Reveals Reciprocal Expression of Inflammatory and Lipid Genes	Plos One
Atopic Eczema	FEZ1	Expression profiling by array	GPL7191	GSE12511	Global Expression Profiling in Atopic Eczema Reveals Reciprocal Expression of Inflammatory and Lipid Genes	Plos One
Atopic Eczema	TRPC2	Expression profiling by array	GPL7191	GSE12511	Global Expression Profiling in Atopic Eczema Reveals Reciprocal Expression of Inflammatory and Lipid Genes	Plos One
Atopic Eczema	MYO5A	Expression profiling by array	GPL7191	GSE12511	Global Expression Profiling in Atopic Eczema Reveals Reciprocal Expression of Inflammatory and Lipid Genes	Plos One
Atopic Eczema	NALP2	Expression profiling by array	GPL7191	GSE12511	Global Expression Profiling in Atopic Eczema Reveals Reciprocal Expression of Inflammatory and Lipid Genes	Plos One

Atopic Eczema	DHTKD1	Expression profiling by array	GPL7191	GSE12511	Global Expression Profiling in Atopic Eczema Reveals Reciprocal Expression of Inflammatory and Lipid Genes	Plos One
Atopic Eczema	MNS1	Expression profiling by array	GPL7191	GSE12511	Global Expression Profiling in Atopic Eczema Reveals Reciprocal Expression of Inflammatory and Lipid Genes	Plos One
Atopic Eczema	FKBP9	Expression profiling by array	GPL7191	GSE12511	Global Expression Profiling in Atopic Eczema Reveals Reciprocal Expression of Inflammatory and Lipid Genes	Plos One
Atopic Eczema	ZNF678	Expression profiling by array	GPL7191	GSE12511	Global Expression Profiling in Atopic Eczema Reveals Reciprocal Expression of Inflammatory and Lipid Genes	Plos One
Atopic Eczema	ARL2	Expression profiling by array	GPL7191	GSE12511	Global Expression Profiling in Atopic Eczema Reveals Reciprocal Expression of Inflammatory and Lipid Genes	Plos One
Atopic Eczema	PSMC3	Expression profiling by array	GPL7191	GSE12511	Global Expression Profiling in Atopic Eczema Reveals Reciprocal Expression of Inflammatory and Lipid Genes	Plos One
Atopic Eczema	KATNAL2	Expression profiling by array	GPL7191	GSE12511	Global Expression Profiling in Atopic Eczema Reveals Reciprocal Expression of Inflammatory and Lipid Genes	Plos One
Atopic Eczema	LIX1L	Expression profiling by array	GPL7191	GSE12511	Global Expression Profiling in Atopic Eczema Reveals Reciprocal Expression of Inflammatory and Lipid Genes	Plos One
Atopic Eczema	TBX19	Expression profiling by array	GPL7191	GSE12511	Global Expression Profiling in Atopic Eczema Reveals Reciprocal Expression of Inflammatory and Lipid Genes	Plos One
Atopic Eczema	MTG1	Expression profiling by array	GPL7191	GSE12511	Global Expression Profiling in Atopic Eczema Reveals Reciprocal Expression of Inflammatory and Lipid Genes	Plos One
Atopic Eczema	FLJ10120	Expression profiling by array	GPL7191	GSE12511	Global Expression Profiling in Atopic Eczema Reveals Reciprocal Expression of Inflammatory and Lipid Genes	Plos One
Atopic Eczema	FBXW9	Expression profiling by array	GPL7191	GSE12511	Global Expression Profiling in Atopic Eczema Reveals Reciprocal Expression of Inflammatory and Lipid Genes	Plos One
Atopic Eczema	ARIH1	Expression profiling by array	GPL7191	GSE12511	Global Expression Profiling in Atopic Eczema Reveals Reciprocal Expression of Inflammatory and Lipid Genes	Plos One
Atopic Eczema	ZNF141	Expression profiling by array	GPL7191	GSE12511	Global Expression Profiling in Atopic Eczema Reveals Reciprocal Expression of Inflammatory and Lipid Genes	Plos One
Atopic Eczema	AHR	Expression profiling by array	GPL7191	GSE12511	Global Expression Profiling in Atopic Eczema Reveals Reciprocal Expression of Inflammatory and Lipid Genes	Plos One
Atopic Eczema	CGN	Expression profiling by array	GPL7191	GSE12511	Global Expression Profiling in Atopic Eczema Reveals Reciprocal Expression of Inflammatory and Lipid Genes	Plos One
Atopic Eczema	ZFYVE27	Expression profiling by array	GPL7191	GSE12511	Global Expression Profiling in Atopic Eczema Reveals Reciprocal Expression of Inflammatory and Lipid Genes	Plos One
Atopic Eczema	AK3L1	Expression profiling by array	GPL7191	GSE12511	Global Expression Profiling in Atopic Eczema Reveals Reciprocal Expression of Inflammatory and Lipid Genes	Plos One
Atopic Eczema	TMPIT	Expression profiling by array	GPL7191	GSE12511	Global Expression Profiling in Atopic Eczema Reveals Reciprocal Expression of Inflammatory and Lipid Genes	Plos One
Atopic Eczema	AGPAT4	Expression profiling by array	GPL7191	GSE12511	Global Expression Profiling in Atopic Eczema Reveals Reciprocal Expression of Inflammatory and Lipid Genes	Plos One
Atopic Eczema	AI216134	Expression profiling by array	GPL7191	GSE12511	Global Expression Profiling in Atopic Eczema Reveals Reciprocal Expression of Inflammatory and Lipid Genes	Plos One
Atopic Eczema	PLB1	Expression profiling by array	GPL7191	GSE12511	Global Expression Profiling in Atopic Eczema Reveals Reciprocal Expression of Inflammatory and Lipid Genes	Plos One
Atopic Eczema	PCSK2	Expression profiling by array	GPL7191	GSE12511	Global Expression Profiling in Atopic Eczema Reveals Reciprocal Expression of Inflammatory and Lipid Genes	Plos One
Atopic Eczema	IVL	Expression profiling by array	GPL7191	GSE12511	Global Expression Profiling in Atopic Eczema Reveals Reciprocal Expression of Inflammatory and Lipid Genes	Plos One
Atopic Eczema	SFRS5	Expression profiling by array	GPL7191	GSE12511	Global Expression Profiling in Atopic Eczema Reveals Reciprocal Expression of Inflammatory and Lipid Genes	Plos One
Atopic Eczema	C21ORF66	Expression profiling by array	GPL7191	GSE12511	Global Expression Profiling in Atopic Eczema Reveals Reciprocal Expression of Inflammatory and Lipid Genes	Plos One
Atopic Eczema	AGPAT1	Expression profiling by array	GPL7191	GSE12511	Global Expression Profiling in Atopic Eczema Reveals Reciprocal Expression of Inflammatory and Lipid Genes	Plos One
Atopic Eczema	RPS6KA1	Expression profiling by array	GPL7191	GSE12511	Global Expression Profiling in Atopic Eczema Reveals Reciprocal Expression of Inflammatory and Lipid Genes	Plos One
Atopic Eczema	CDS1	Expression profiling by array	GPL7191	GSE12511	Global Expression Profiling in Atopic Eczema Reveals Reciprocal Expression of Inflammatory and Lipid Genes	Plos One
Atopic Eczema	SOX10	Expression profiling by array	GPL7191	GSE12511	Global Expression Profiling in Atopic Eczema Reveals Reciprocal Expression of Inflammatory and Lipid Genes	Plos One
Atopic Eczema	IGFBP7	Expression profiling by array	GPL7191	GSE12511	Global Expression Profiling in Atopic Eczema Reveals Reciprocal Expression of Inflammatory and Lipid Genes	Plos One
Atopic Eczema	PTP4A1	Expression profiling by array	GPL7191	GSE12511	Global Expression Profiling in Atopic Eczema Reveals Reciprocal Expression of Inflammatory and Lipid Genes	Plos One
Atopic Eczema	EIF1AY	Expression profiling by array	GPL7191	GSE12511	Global Expression Profiling in Atopic Eczema Reveals Reciprocal Expression of Inflammatory and Lipid Genes	Plos One
Atopic Eczema	ERGIC3	Expression profiling by array	GPL7191	GSE12511	Global Expression Profiling in Atopic Eczema Reveals Reciprocal Expression of Inflammatory and Lipid Genes	Plos One
Atopic Eczema	ROD1	Expression profiling by array	GPL7191	GSE12511	Global Expression Profiling in Atopic Eczema Reveals Reciprocal Expression of Inflammatory and Lipid Genes	Plos One
Atopic Eczema	EBF	Expression profiling by array	GPL7191	GSE12511	Global Expression Profiling in Atopic Eczema Reveals Reciprocal Expression of Inflammatory and Lipid Genes	Plos One
Atopic Eczema	VGLL4	Expression profiling by array	GPL7191	GSE12511	Global Expression Profiling in Atopic Eczema Reveals Reciprocal Expression of Inflammatory and Lipid Genes	Plos One
Atopic Eczema	AA169372	Expression profiling by array	GPL7191	GSE12511	Global Expression Profiling in Atopic Eczema Reveals Reciprocal Expression of Inflammatory and Lipid Genes	Plos One
Atopic Eczema	GPR175	Expression profiling by array	GPL7191	GSE12511	Global Expression Profiling in Atopic Eczema Reveals Reciprocal Expression of Inflammatory and Lipid Genes	Plos One
Atopic Eczema	LOC124512	Expression profiling by array	GPL7191	GSE12511	Global Expression Profiling in Atopic Eczema Reveals Reciprocal Expression of Inflammatory and Lipid Genes	Plos One
Atopic Eczema	ADAMTS2	Expression profiling by array	GPL7191	GSE12511	Global Expression Profiling in Atopic Eczema Reveals Reciprocal Expression of Inflammatory and Lipid Genes	Plos One
Atopic Eczema	HBB	Expression profiling by array	GPL7191	GSE12511	Global Expression Profiling in Atopic Eczema Reveals Reciprocal Expression of Inflammatory and Lipid Genes	Plos One
Atopic Eczema	RP11-50D16.3	Expression profiling by array	GPL7191	GSE12511	Global Expression Profiling in Atopic Eczema Reveals Reciprocal Expression of Inflammatory and Lipid Genes	Plos One
Atopic Eczema	UBE2D2	Expression profiling by array	GPL7191	GSE12511	Global Expression Profiling in Atopic Eczema Reveals Reciprocal Expression of Inflammatory and Lipid Genes	Plos One
Atopic Eczema	CCND2	Expression profiling by array	GPL7191	GSE12511	Global Expression Profiling in Atopic Eczema Reveals Reciprocal Expression of Inflammatory and Lipid Genes	Plos One
Atopic Eczema	AA634469	Expression profiling by array	GPL7191	GSE12511	Global Expression Profiling in Atopic Eczema Reveals Reciprocal Expression of Inflammatory and Lipid Genes	Plos One
Atopic Eczema	TMEM163	Expression profiling by array	GPL7191	GSE12511	Global Expression Profiling in Atopic Eczema Reveals Reciprocal Expression of Inflammatory and Lipid Genes	Plos One
Atopic Eczema	DDX3X	Expression profiling by array	GPL7191	GSE12511	Global Expression Profiling in Atopic Eczema Reveals Reciprocal Expression of Inflammatory and Lipid Genes	Plos One
Atopic Eczema	STAT1	Expression profiling by array	GPL7191	GSE12511	Global Expression Profiling in Atopic Eczema Reveals Reciprocal Expression of Inflammatory and Lipid Genes	Plos One
Atopic Eczema	C6ORF170	Expression profiling by array	GPL7191	GSE12511	Global Expression Profiling in Atopic Eczema Reveals Reciprocal Expression of Inflammatory and Lipid Genes	Plos One
Atopic Eczema	FNTB	Expression profiling by array	GPL7191	GSE12511	Global Expression Profiling in Atopic Eczema Reveals Reciprocal Expression of Inflammatory and Lipid Genes	Plos One
Atopic Eczema	GAL3ST1	Expression profiling by array	GPL7191	GSE12511	Global Expression Profiling in Atopic Eczema Reveals Reciprocal Expression of Inflammatory and Lipid Genes	Plos One
Atopic Eczema	CDC45L	Expression profiling by array	GPL7191	GSE12511	Global Expression Profiling in Atopic Eczema Reveals Reciprocal Expression of Inflammatory and Lipid Genes	Plos One
Atopic Eczema	SDF2L1	Expression profiling by array	GPL7191	GSE12511	Global Expression Profiling in Atopic Eczema Reveals Reciprocal Expression of Inflammatory and Lipid Genes	Plos One
Atopic Eczema	DCTD	Expression profiling by array	GPL7191	GSE12511	Global Expression Profiling in Atopic Eczema Reveals Reciprocal Expression of Inflammatory and Lipid Genes	Plos One
Atopic Eczema	CPEB3	Expression profiling by array	GPL7191	GSE12511	Global Expression Profiling in Atopic Eczema Reveals Reciprocal Expression of Inflammatory and Lipid Genes	Plos One
Atopic Eczema	AA620525	Expression profiling by array	GPL7191	GSE12511	Global Expression Profiling in Atopic Eczema Reveals Reciprocal Expression of Inflammatory and Lipid Genes	Plos One
Atopic Eczema	UBTD1	Expression profiling by array	GPL7191	GSE12511	Global Expression Profiling in Atopic Eczema Reveals Reciprocal Expression of Inflammatory and Lipid Genes	Plos One
Atopic Eczema	TM2D2	Expression profiling by array	GPL7191	GSE12511	Global Expression Profiling in Atopic Eczema Reveals Reciprocal Expression of Inflammatory and Lipid Genes	Plos One
Atopic Eczema	FAM89B	Expression profiling by array	GPL7191	GSE12511	Global Expression Profiling in Atopic Eczema Reveals Reciprocal Expression of Inflammatory and Lipid Genes	Plos One
Atopic Eczema	TATDN2	Expression profiling by array	GPL7191	GSE12511	Global Expression Profiling in Atopic Eczema Reveals Reciprocal Expression of Inflammatory and Lipid Genes	Plos One
Atopic Eczema	SUZ12P	Expression profiling by array	GPL7191	GSE12511	Global Expression Profiling in Atopic Eczema Reveals Reciprocal Expression of Inflammatory and Lipid Genes	Plos One

Atopic Eczema	C8A	Expression profiling by array	GPL7191	GSE12511	Global Expression Profiling in Atopic Eczema Reveals Reciprocal Expression of Inflammatory and Lipid Genes	Plos One
Atopic Eczema	CLDN12	Expression profiling by array	GPL7191	GSE12511	Global Expression Profiling in Atopic Eczema Reveals Reciprocal Expression of Inflammatory and Lipid Genes	Plos One
Atopic Eczema	C17ORF71	Expression profiling by array	GPL7191	GSE12511	Global Expression Profiling in Atopic Eczema Reveals Reciprocal Expression of Inflammatory and Lipid Genes	Plos One
Atopic Eczema	AA757720	Expression profiling by array	GPL7191	GSE12511	Global Expression Profiling in Atopic Eczema Reveals Reciprocal Expression of Inflammatory and Lipid Genes	Plos One
Atopic Eczema	THAP8	Expression profiling by array	GPL7191	GSE12511	Global Expression Profiling in Atopic Eczema Reveals Reciprocal Expression of Inflammatory and Lipid Genes	Plos One
Atopic Eczema	GPC6	Expression profiling by array	GPL7191	GSE12511	Global Expression Profiling in Atopic Eczema Reveals Reciprocal Expression of Inflammatory and Lipid Genes	Plos One
Atopic Eczema	GALNT5	Expression profiling by array	GPL7191	GSE12511	Global Expression Profiling in Atopic Eczema Reveals Reciprocal Expression of Inflammatory and Lipid Genes	Plos One
Atopic Eczema	TTC9C	Expression profiling by array	GPL7191	GSE12511	Global Expression Profiling in Atopic Eczema Reveals Reciprocal Expression of Inflammatory and Lipid Genes	Plos One
Atopic Eczema	BMP2K	Expression profiling by array	GPL7191	GSE12511	Global Expression Profiling in Atopic Eczema Reveals Reciprocal Expression of Inflammatory and Lipid Genes	Plos One
Atopic Eczema	AKAP12	Expression profiling by array	GPL7191	GSE12511	Global Expression Profiling in Atopic Eczema Reveals Reciprocal Expression of Inflammatory and Lipid Genes	Plos One
Atopic Eczema	ARHGAP28	Expression profiling by array	GPL7191	GSE12511	Global Expression Profiling in Atopic Eczema Reveals Reciprocal Expression of Inflammatory and Lipid Genes	Plos One
Atopic Eczema	ETV6	Expression profiling by array	GPL7191	GSE12511	Global Expression Profiling in Atopic Eczema Reveals Reciprocal Expression of Inflammatory and Lipid Genes	Plos One
Atopic Eczema	RPP21	Expression profiling by array	GPL7191	GSE12511	Global Expression Profiling in Atopic Eczema Reveals Reciprocal Expression of Inflammatory and Lipid Genes	Plos One
Atopic Eczema	PRPF6	Expression profiling by array	GPL7191	GSE12511	Global Expression Profiling in Atopic Eczema Reveals Reciprocal Expression of Inflammatory and Lipid Genes	Plos One
Atopic Eczema	MAGI2	Expression profiling by array	GPL7191	GSE12511	Global Expression Profiling in Atopic Eczema Reveals Reciprocal Expression of Inflammatory and Lipid Genes	Plos One
Atopic Eczema	LIFR	Expression profiling by array	GPL7191	GSE12511	Global Expression Profiling in Atopic Eczema Reveals Reciprocal Expression of Inflammatory and Lipid Genes	Plos One
Atopic Eczema	BLVRB	Expression profiling by array	GPL7191	GSE12511	Global Expression Profiling in Atopic Eczema Reveals Reciprocal Expression of Inflammatory and Lipid Genes	Plos One
Atopic Eczema	ATP5I	Expression profiling by array	GPL7191	GSE12511	Global Expression Profiling in Atopic Eczema Reveals Reciprocal Expression of Inflammatory and Lipid Genes	Plos One
Atopic Eczema	C10ORF83	Expression profiling by array	GPL7191	GSE12511	Global Expression Profiling in Atopic Eczema Reveals Reciprocal Expression of Inflammatory and Lipid Genes	Plos One
Atopic Eczema	ATAD2	Expression profiling by array	GPL7191	GSE12511	Global Expression Profiling in Atopic Eczema Reveals Reciprocal Expression of Inflammatory and Lipid Genes	Plos One
Atopic Eczema	CCNC	Expression profiling by array	GPL7191	GSE12511	Global Expression Profiling in Atopic Eczema Reveals Reciprocal Expression of Inflammatory and Lipid Genes	Plos One
Atopic Eczema	NSMCE1	Expression profiling by array	GPL7191	GSE12511	Global Expression Profiling in Atopic Eczema Reveals Reciprocal Expression of Inflammatory and Lipid Genes	Plos One
Atopic Eczema	BNC2	Expression profiling by array	GPL7191	GSE12511	Global Expression Profiling in Atopic Eczema Reveals Reciprocal Expression of Inflammatory and Lipid Genes	Plos One
Atopic Eczema	ADAM17	Expression profiling by array	GPL7191	GSE12511	Global Expression Profiling in Atopic Eczema Reveals Reciprocal Expression of Inflammatory and Lipid Genes	Plos One
Atopic Eczema	SCAMP3	Expression profiling by array	GPL7191	GSE12511	Global Expression Profiling in Atopic Eczema Reveals Reciprocal Expression of Inflammatory and Lipid Genes	Plos One
Atopic Eczema	MGC33302	Expression profiling by array	GPL7191	GSE12511	Global Expression Profiling in Atopic Eczema Reveals Reciprocal Expression of Inflammatory and Lipid Genes	Plos One
Atopic Eczema	BAZ2B	Expression profiling by array	GPL7191	GSE12511	Global Expression Profiling in Atopic Eczema Reveals Reciprocal Expression of Inflammatory and Lipid Genes	Plos One
Atopic Eczema	CTDSP1	Expression profiling by array	GPL7191	GSE12511	Global Expression Profiling in Atopic Eczema Reveals Reciprocal Expression of Inflammatory and Lipid Genes	Plos One
Atopic Eczema	ABI2	Expression profiling by array	GPL7191	GSE12511	Global Expression Profiling in Atopic Eczema Reveals Reciprocal Expression of Inflammatory and Lipid Genes	Plos One
Atopic Eczema	CCDC8	Expression profiling by array	GPL7191	GSE12511	Global Expression Profiling in Atopic Eczema Reveals Reciprocal Expression of Inflammatory and Lipid Genes	Plos One
Atopic Eczema	DTYMK	Expression profiling by array	GPL7191	GSE12511	Global Expression Profiling in Atopic Eczema Reveals Reciprocal Expression of Inflammatory and Lipid Genes	Plos One
Atopic Eczema	CPNE2	Expression profiling by array	GPL7191	GSE12511	Global Expression Profiling in Atopic Eczema Reveals Reciprocal Expression of Inflammatory and Lipid Genes	Plos One
Atopic Eczema	EIF2B2	Expression profiling by array	GPL7191	GSE12511	Global Expression Profiling in Atopic Eczema Reveals Reciprocal Expression of Inflammatory and Lipid Genes	Plos One
Atopic Eczema	ACSS1	Expression profiling by array	GPL7191	GSE12511	Global Expression Profiling in Atopic Eczema Reveals Reciprocal Expression of Inflammatory and Lipid Genes	Plos One
Atopic Eczema	PSMB4	Expression profiling by array	GPL7191	GSE12511	Global Expression Profiling in Atopic Eczema Reveals Reciprocal Expression of Inflammatory and Lipid Genes	Plos One
Atopic Eczema	BRAP	Expression profiling by array	GPL7191	GSE12511	Global Expression Profiling in Atopic Eczema Reveals Reciprocal Expression of Inflammatory and Lipid Genes	Plos One
Atopic Eczema	FVT1	Expression profiling by array	GPL7191	GSE12511	Global Expression Profiling in Atopic Eczema Reveals Reciprocal Expression of Inflammatory and Lipid Genes	Plos One
Atopic Eczema	NKIRAS2	Expression profiling by array	GPL7191	GSE12511	Global Expression Profiling in Atopic Eczema Reveals Reciprocal Expression of Inflammatory and Lipid Genes	Plos One
Atopic Eczema	ARMCX6	Expression profiling by array	GPL7191	GSE12511	Global Expression Profiling in Atopic Eczema Reveals Reciprocal Expression of Inflammatory and Lipid Genes	Plos One
Atopic Eczema	GADD45B	Expression profiling by array	GPL7191	GSE12511	Global Expression Profiling in Atopic Eczema Reveals Reciprocal Expression of Inflammatory and Lipid Genes	Plos One
Atopic Eczema	RGNEF	Expression profiling by array	GPL7191	GSE12511	Global Expression Profiling in Atopic Eczema Reveals Reciprocal Expression of Inflammatory and Lipid Genes	Plos One
Atopic Eczema	PEX14	Expression profiling by array	GPL7191	GSE12511	Global Expression Profiling in Atopic Eczema Reveals Reciprocal Expression of Inflammatory and Lipid Genes	Plos One
Atopic Eczema	TARBP2	Expression profiling by array	GPL7191	GSE12511	Global Expression Profiling in Atopic Eczema Reveals Reciprocal Expression of Inflammatory and Lipid Genes	Plos One
Atopic Eczema	C10ORF116	Expression profiling by array	GPL7191	GSE12511	Global Expression Profiling in Atopic Eczema Reveals Reciprocal Expression of Inflammatory and Lipid Genes	Plos One
Atopic Eczema	IFNG	Expression profiling by array	GPL7191	GSE12511	Global Expression Profiling in Atopic Eczema Reveals Reciprocal Expression of Inflammatory and Lipid Genes	Plos One
Atopic Eczema	MVP	Expression profiling by array	GPL7191	GSE12511	Global Expression Profiling in Atopic Eczema Reveals Reciprocal Expression of Inflammatory and Lipid Genes	Plos One
Atopic Eczema	LOC441601	Expression profiling by array	GPL7191	GSE12511	Global Expression Profiling in Atopic Eczema Reveals Reciprocal Expression of Inflammatory and Lipid Genes	Plos One
Atopic Eczema	H2AFX	Expression profiling by array	GPL7191	GSE12511	Global Expression Profiling in Atopic Eczema Reveals Reciprocal Expression of Inflammatory and Lipid Genes	Plos One
Atopic Eczema	IKBKE	Expression profiling by array	GPL7191	GSE12511	Global Expression Profiling in Atopic Eczema Reveals Reciprocal Expression of Inflammatory and Lipid Genes	Plos One
Atopic Eczema	CDH13	Expression profiling by array	GPL7191	GSE12511	Global Expression Profiling in Atopic Eczema Reveals Reciprocal Expression of Inflammatory and Lipid Genes	Plos One
Atopic Eczema	GGT1	Expression profiling by array	GPL7191	GSE12511	Global Expression Profiling in Atopic Eczema Reveals Reciprocal Expression of Inflammatory and Lipid Genes	Plos One
Atopic Eczema	DDO	Expression profiling by array	GPL7191	GSE12511	Global Expression Profiling in Atopic Eczema Reveals Reciprocal Expression of Inflammatory and Lipid Genes	Plos One
Atopic Eczema	RNASEH1	Expression profiling by array	GPL7191	GSE12511	Global Expression Profiling in Atopic Eczema Reveals Reciprocal Expression of Inflammatory and Lipid Genes	Plos One
Atopic Eczema	ADAMTS7	Expression profiling by array	GPL7191	GSE12511	Global Expression Profiling in Atopic Eczema Reveals Reciprocal Expression of Inflammatory and Lipid Genes	Plos One
Atopic Eczema	LOC144363	Expression profiling by array	GPL7191	GSE12511	Global Expression Profiling in Atopic Eczema Reveals Reciprocal Expression of Inflammatory and Lipid Genes	Plos One
Atopic Eczema	CRISPLD2	Expression profiling by array	GPL7191	GSE12511	Global Expression Profiling in Atopic Eczema Reveals Reciprocal Expression of Inflammatory and Lipid Genes	Plos One
Atopic Eczema	KCTD1	Expression profiling by array	GPL7191	GSE12511	Global Expression Profiling in Atopic Eczema Reveals Reciprocal Expression of Inflammatory and Lipid Genes	Plos One
Atopic Eczema	ASXL1	Expression profiling by array	GPL7191	GSE12511	Global Expression Profiling in Atopic Eczema Reveals Reciprocal Expression of Inflammatory and Lipid Genes	Plos One
Atopic Eczema	AI040814	Expression profiling by array	GPL7191	GSE12511	Global Expression Profiling in Atopic Eczema Reveals Reciprocal Expression of Inflammatory and Lipid Genes	Plos One
Atopic Eczema	CXCR4	Expression profiling by array	GPL7191	GSE12511	Global Expression Profiling in Atopic Eczema Reveals Reciprocal Expression of Inflammatory and Lipid Genes	Plos One
Atopic Eczema	FALZ	Expression profiling by array	GPL7191	GSE12511	Global Expression Profiling in Atopic Eczema Reveals Reciprocal Expression of Inflammatory and Lipid Genes	Plos One
Atopic Eczema	FLJ20152	Expression profiling by array	GPL7191	GSE12511	Global Expression Profiling in Atopic Eczema Reveals Reciprocal Expression of Inflammatory and Lipid Genes	Plos One

Atopic Eczema	ASRGL1	Expression profiling by array	GPL7191	GSE12511	Global Expression Profiling in Atopic Eczema Reveals Reciprocal Expression of Inflammatory and Lipid Genes	Plos One
Atopic Eczema	ST3GAL1	Expression profiling by array	GPL7191	GSE12511	Global Expression Profiling in Atopic Eczema Reveals Reciprocal Expression of Inflammatory and Lipid Genes	Plos One
Atopic Eczema	ZC3H6	Expression profiling by array	GPL7191	GSE12511	Global Expression Profiling in Atopic Eczema Reveals Reciprocal Expression of Inflammatory and Lipid Genes	Plos One
Atopic Eczema	PER2	Expression profiling by array	GPL7191	GSE12511	Global Expression Profiling in Atopic Eczema Reveals Reciprocal Expression of Inflammatory and Lipid Genes	Plos One
Atopic Eczema	PDZK1IP1	Expression profiling by array	GPL7191	GSE12511	Global Expression Profiling in Atopic Eczema Reveals Reciprocal Expression of Inflammatory and Lipid Genes	Plos One
Atopic Eczema	F25965	Expression profiling by array	GPL7191	GSE12511	Global Expression Profiling in Atopic Eczema Reveals Reciprocal Expression of Inflammatory and Lipid Genes	Plos One
Atopic Eczema	STAB2	Expression profiling by array	GPL7191	GSE12511	Global Expression Profiling in Atopic Eczema Reveals Reciprocal Expression of Inflammatory and Lipid Genes	Plos One
Atopic Eczema	COL23A1	Expression profiling by array	GPL7191	GSE12511	Global Expression Profiling in Atopic Eczema Reveals Reciprocal Expression of Inflammatory and Lipid Genes	Plos One
Atopic Eczema	DPYSL3	Expression profiling by array	GPL7191	GSE12511	Global Expression Profiling in Atopic Eczema Reveals Reciprocal Expression of Inflammatory and Lipid Genes	Plos One
Atopic Eczema	RG9MTD3	Expression profiling by array	GPL7191	GSE12511	Global Expression Profiling in Atopic Eczema Reveals Reciprocal Expression of Inflammatory and Lipid Genes	Plos One
Atopic Eczema	SMG5	Expression profiling by array	GPL7191	GSE12511	Global Expression Profiling in Atopic Eczema Reveals Reciprocal Expression of Inflammatory and Lipid Genes	Plos One
Atopic Eczema	GALE	Expression profiling by array	GPL7191	GSE12511	Global Expression Profiling in Atopic Eczema Reveals Reciprocal Expression of Inflammatory and Lipid Genes	Plos One
Atopic Eczema	MCAM	Expression profiling by array	GPL7191	GSE12511	Global Expression Profiling in Atopic Eczema Reveals Reciprocal Expression of Inflammatory and Lipid Genes	Plos One
Atopic Eczema	ALPP	Expression profiling by array	GPL7191	GSE12511	Global Expression Profiling in Atopic Eczema Reveals Reciprocal Expression of Inflammatory and Lipid Genes	Plos One
Atopic Eczema	SLCO4A1	Expression profiling by array	GPL7191	GSE12511	Global Expression Profiling in Atopic Eczema Reveals Reciprocal Expression of Inflammatory and Lipid Genes	Plos One
Atopic Eczema	TMCO4	Expression profiling by array	GPL7191	GSE12511	Global Expression Profiling in Atopic Eczema Reveals Reciprocal Expression of Inflammatory and Lipid Genes	Plos One
Atopic Eczema	TEX264	Expression profiling by array	GPL7191	GSE12511	Global Expression Profiling in Atopic Eczema Reveals Reciprocal Expression of Inflammatory and Lipid Genes	Plos One
Atopic Eczema	MUC1	Expression profiling by array	GPL7191	GSE12511	Global Expression Profiling in Atopic Eczema Reveals Reciprocal Expression of Inflammatory and Lipid Genes	Plos One
Atopic Eczema	ZNF589	Expression profiling by array	GPL7191	GSE12511	Global Expression Profiling in Atopic Eczema Reveals Reciprocal Expression of Inflammatory and Lipid Genes	Plos One
Atopic Eczema	CCDC64	Expression profiling by array	GPL7191	GSE12511	Global Expression Profiling in Atopic Eczema Reveals Reciprocal Expression of Inflammatory and Lipid Genes	Plos One
Atopic Eczema	NMT2	Expression profiling by array	GPL7191	GSE12511	Global Expression Profiling in Atopic Eczema Reveals Reciprocal Expression of Inflammatory and Lipid Genes	Plos One
Atopic Eczema	BMPR2	Expression profiling by array	GPL7191	GSE12511	Global Expression Profiling in Atopic Eczema Reveals Reciprocal Expression of Inflammatory and Lipid Genes	Plos One
Atopic Eczema	C10ORF78	Expression profiling by array	GPL7191	GSE12511	Global Expression Profiling in Atopic Eczema Reveals Reciprocal Expression of Inflammatory and Lipid Genes	Plos One
Atopic Eczema	XPO6	Expression profiling by array	GPL7191	GSE12511	Global Expression Profiling in Atopic Eczema Reveals Reciprocal Expression of Inflammatory and Lipid Genes	Plos One
Atopic Eczema	NRP2	Expression profiling by array	GPL7191	GSE12511	Global Expression Profiling in Atopic Eczema Reveals Reciprocal Expression of Inflammatory and Lipid Genes	Plos One
Atopic Eczema	PAPPA	Expression profiling by array	GPL7191	GSE12511	Global Expression Profiling in Atopic Eczema Reveals Reciprocal Expression of Inflammatory and Lipid Genes	Plos One
Atopic Eczema	DTNB	Expression profiling by array	GPL7191	GSE12511	Global Expression Profiling in Atopic Eczema Reveals Reciprocal Expression of Inflammatory and Lipid Genes	Plos One
Atopic Eczema	PTTG1	Expression profiling by array	GPL7191	GSE12511	Global Expression Profiling in Atopic Eczema Reveals Reciprocal Expression of Inflammatory and Lipid Genes	Plos One
Atopic Eczema	PYDC1	Expression profiling by array	GPL7191	GSE12511	Global Expression Profiling in Atopic Eczema Reveals Reciprocal Expression of Inflammatory and Lipid Genes	Plos One
Atopic Eczema	CYBB	Expression profiling by array	GPL7191	GSE12511	Global Expression Profiling in Atopic Eczema Reveals Reciprocal Expression of Inflammatory and Lipid Genes	Plos One
Atopic Eczema	ACSM2	Expression profiling by array	GPL7191	GSE12511	Global Expression Profiling in Atopic Eczema Reveals Reciprocal Expression of Inflammatory and Lipid Genes	Plos One
Atopic Eczema	GLT28D1	Expression profiling by array	GPL7191	GSE12511	Global Expression Profiling in Atopic Eczema Reveals Reciprocal Expression of Inflammatory and Lipid Genes	Plos One
Atopic Eczema	AQP7	Expression profiling by array	GPL7191	GSE12511	Global Expression Profiling in Atopic Eczema Reveals Reciprocal Expression of Inflammatory and Lipid Genes	Plos One
Atopic Eczema	ARL6IP4	Expression profiling by array	GPL7191	GSE12511	Global Expression Profiling in Atopic Eczema Reveals Reciprocal Expression of Inflammatory and Lipid Genes	Plos One
Atopic Eczema	ARL13B	Expression profiling by array	GPL7191	GSE12511	Global Expression Profiling in Atopic Eczema Reveals Reciprocal Expression of Inflammatory and Lipid Genes	Plos One
Atopic Eczema	KIAA0773	Expression profiling by array	GPL7191	GSE12511	Global Expression Profiling in Atopic Eczema Reveals Reciprocal Expression of Inflammatory and Lipid Genes	Plos One
Atopic Eczema	HD	Expression profiling by array	GPL7191	GSE12511	Global Expression Profiling in Atopic Eczema Reveals Reciprocal Expression of Inflammatory and Lipid Genes	Plos One
Atopic Eczema	CBWD1	Expression profiling by array	GPL7191	GSE12511	Global Expression Profiling in Atopic Eczema Reveals Reciprocal Expression of Inflammatory and Lipid Genes	Plos One
Atopic Eczema	RAB8B	Expression profiling by array	GPL7191	GSE12511	Global Expression Profiling in Atopic Eczema Reveals Reciprocal Expression of Inflammatory and Lipid Genes	Plos One
Atopic Eczema	BTNL9	Expression profiling by array	GPL7191	GSE12511	Global Expression Profiling in Atopic Eczema Reveals Reciprocal Expression of Inflammatory and Lipid Genes	Plos One
Atopic Eczema	ARSA	Expression profiling by array	GPL7191	GSE12511	Global Expression Profiling in Atopic Eczema Reveals Reciprocal Expression of Inflammatory and Lipid Genes	Plos One
Atopic Eczema	TPX2	Expression profiling by array	GPL7191	GSE12511	Global Expression Profiling in Atopic Eczema Reveals Reciprocal Expression of Inflammatory and Lipid Genes	Plos One
Atopic Eczema	AA875853	Expression profiling by array	GPL7191	GSE12511	Global Expression Profiling in Atopic Eczema Reveals Reciprocal Expression of Inflammatory and Lipid Genes	Plos One
Atopic Eczema	DDAH1	Expression profiling by array	GPL7191	GSE12511	Global Expression Profiling in Atopic Eczema Reveals Reciprocal Expression of Inflammatory and Lipid Genes	Plos One
Atopic Eczema	MRPL4	Expression profiling by array	GPL7191	GSE12511	Global Expression Profiling in Atopic Eczema Reveals Reciprocal Expression of Inflammatory and Lipid Genes	Plos One
Atopic Eczema	DZIP3	Expression profiling by array	GPL7191	GSE12511	Global Expression Profiling in Atopic Eczema Reveals Reciprocal Expression of Inflammatory and Lipid Genes	Plos One
Atopic Eczema	ARHGAP24	Expression profiling by array	GPL7191	GSE12511	Global Expression Profiling in Atopic Eczema Reveals Reciprocal Expression of Inflammatory and Lipid Genes	Plos One
Atopic Eczema	KIAA1370	Expression profiling by array	GPL7191	GSE12511	Global Expression Profiling in Atopic Eczema Reveals Reciprocal Expression of Inflammatory and Lipid Genes	Plos One
Atopic Eczema	AA777915	Expression profiling by array	GPL7191	GSE12511	Global Expression Profiling in Atopic Eczema Reveals Reciprocal Expression of Inflammatory and Lipid Genes	Plos One
Atopic Eczema	MRPL27	Expression profiling by array	GPL7191	GSE12511	Global Expression Profiling in Atopic Eczema Reveals Reciprocal Expression of Inflammatory and Lipid Genes	Plos One
Atopic Eczema	ODZ4	Expression profiling by array	GPL7191	GSE12511	Global Expression Profiling in Atopic Eczema Reveals Reciprocal Expression of Inflammatory and Lipid Genes	Plos One
Atopic Eczema	GNA12	Expression profiling by array	GPL7191	GSE12511	Global Expression Profiling in Atopic Eczema Reveals Reciprocal Expression of Inflammatory and Lipid Genes	Plos One
Atopic Eczema	ATP6V0D2	Expression profiling by array	GPL7191	GSE12511	Global Expression Profiling in Atopic Eczema Reveals Reciprocal Expression of Inflammatory and Lipid Genes	Plos One
Atopic Eczema	UNC84A	Expression profiling by array	GPL7191	GSE12511	Global Expression Profiling in Atopic Eczema Reveals Reciprocal Expression of Inflammatory and Lipid Genes	Plos One
Atopic Eczema	KIF1A	Expression profiling by array	GPL7191	GSE12511	Global Expression Profiling in Atopic Eczema Reveals Reciprocal Expression of Inflammatory and Lipid Genes	Plos One
Atopic Eczema	KIAA1107	Expression profiling by array	GPL7191	GSE12511	Global Expression Profiling in Atopic Eczema Reveals Reciprocal Expression of Inflammatory and Lipid Genes	Plos One
Atopic Eczema	GNB1	Expression profiling by array	GPL7191	GSE12511	Global Expression Profiling in Atopic Eczema Reveals Reciprocal Expression of Inflammatory and Lipid Genes	Plos One
Atopic Eczema	GOLPH3	Expression profiling by array	GPL7191	GSE12511	Global Expression Profiling in Atopic Eczema Reveals Reciprocal Expression of Inflammatory and Lipid Genes	Plos One
Atopic Eczema	MT2A	Expression profiling by array	GPL7191	GSE12511	Global Expression Profiling in Atopic Eczema Reveals Reciprocal Expression of Inflammatory and Lipid Genes	Plos One
Atopic Eczema	KCNK2	Expression profiling by array	GPL7191	GSE12511	Global Expression Profiling in Atopic Eczema Reveals Reciprocal Expression of Inflammatory and Lipid Genes	Plos One
Atopic Eczema	FLJ10159	Expression profiling by array	GPL7191	GSE12511	Global Expression Profiling in Atopic Eczema Reveals Reciprocal Expression of Inflammatory and Lipid Genes	Plos One
Atopic Eczema	BXDC1	Expression profiling by array	GPL7191	GSE12511	Global Expression Profiling in Atopic Eczema Reveals Reciprocal Expression of Inflammatory and Lipid Genes	Plos One

Atopic Eczema	MRPS12	Expression profiling by array	GPL7191	GSE12511	Global Expression Profiling in Atopic Eczema Reveals Reciprocal Expression of Inflammatory and Lipid Genes	Plos One
Atopic Eczema	ACVR2B	Expression profiling by array	GPL7191	GSE12511	Global Expression Profiling in Atopic Eczema Reveals Reciprocal Expression of Inflammatory and Lipid Genes	Plos One
Atopic Eczema	ABHD6	Expression profiling by array	GPL7191	GSE12511	Global Expression Profiling in Atopic Eczema Reveals Reciprocal Expression of Inflammatory and Lipid Genes	Plos One
Atopic Eczema	LPIN1	Expression profiling by array	GPL7191	GSE12511	Global Expression Profiling in Atopic Eczema Reveals Reciprocal Expression of Inflammatory and Lipid Genes	Plos One
Atopic Eczema	SLC4A11	Expression profiling by array	GPL7191	GSE12511	Global Expression Profiling in Atopic Eczema Reveals Reciprocal Expression of Inflammatory and Lipid Genes	Plos One
Atopic Eczema	URP2	Expression profiling by array	GPL7191	GSE12511	Global Expression Profiling in Atopic Eczema Reveals Reciprocal Expression of Inflammatory and Lipid Genes	Plos One
Atopic Eczema	SLC26A2	Expression profiling by array	GPL7191	GSE12511	Global Expression Profiling in Atopic Eczema Reveals Reciprocal Expression of Inflammatory and Lipid Genes	Plos One
Atopic Eczema	CLIC1	Expression profiling by array	GPL7191	GSE12511	Global Expression Profiling in Atopic Eczema Reveals Reciprocal Expression of Inflammatory and Lipid Genes	Plos One
Atopic Eczema	GNAI1	Expression profiling by array	GPL7191	GSE12511	Global Expression Profiling in Atopic Eczema Reveals Reciprocal Expression of Inflammatory and Lipid Genes	Plos One
Atopic Eczema	CD53	Expression profiling by array	GPL7191	GSE12511	Global Expression Profiling in Atopic Eczema Reveals Reciprocal Expression of Inflammatory and Lipid Genes	Plos One
Atopic Eczema	ANPEP	Expression profiling by array	GPL7191	GSE12511	Global Expression Profiling in Atopic Eczema Reveals Reciprocal Expression of Inflammatory and Lipid Genes	Plos One
Atopic Eczema	MARCH3	Expression profiling by array	GPL7191	GSE12511	Global Expression Profiling in Atopic Eczema Reveals Reciprocal Expression of Inflammatory and Lipid Genes	Plos One
Atopic Eczema	CRIP1	Expression profiling by array	GPL7191	GSE12511	Global Expression Profiling in Atopic Eczema Reveals Reciprocal Expression of Inflammatory and Lipid Genes	Plos One
Atopic Eczema	CD37	Expression profiling by array	GPL7191	GSE12511	Global Expression Profiling in Atopic Eczema Reveals Reciprocal Expression of Inflammatory and Lipid Genes	Plos One
Atopic Eczema	FBLN2	Expression profiling by array	GPL7191	GSE12511	Global Expression Profiling in Atopic Eczema Reveals Reciprocal Expression of Inflammatory and Lipid Genes	Plos One
Atopic Eczema	ETS2	Expression profiling by array	GPL7191	GSE12511	Global Expression Profiling in Atopic Eczema Reveals Reciprocal Expression of Inflammatory and Lipid Genes	Plos One
Atopic Eczema	MICAL1	Expression profiling by array	GPL7191	GSE12511	Global Expression Profiling in Atopic Eczema Reveals Reciprocal Expression of Inflammatory and Lipid Genes	Plos One
Atopic Eczema	NCLN	Expression profiling by array	GPL7191	GSE12511	Global Expression Profiling in Atopic Eczema Reveals Reciprocal Expression of Inflammatory and Lipid Genes	Plos One
Atopic Eczema	GRB14	Expression profiling by array	GPL7191	GSE12511	Global Expression Profiling in Atopic Eczema Reveals Reciprocal Expression of Inflammatory and Lipid Genes	Plos One
Atopic Eczema	MFI2	Expression profiling by array	GPL7191	GSE12511	Global Expression Profiling in Atopic Eczema Reveals Reciprocal Expression of Inflammatory and Lipid Genes	Plos One
Atopic Eczema	FSTL1	Expression profiling by array	GPL7191	GSE12511	Global Expression Profiling in Atopic Eczema Reveals Reciprocal Expression of Inflammatory and Lipid Genes	Plos One
Atopic Eczema	SH3BGRL2	Expression profiling by array	GPL7191	GSE12511	Global Expression Profiling in Atopic Eczema Reveals Reciprocal Expression of Inflammatory and Lipid Genes	Plos One
Atopic Eczema	EGLN3	Expression profiling by array	GPL7191	GSE12511	Global Expression Profiling in Atopic Eczema Reveals Reciprocal Expression of Inflammatory and Lipid Genes	Plos One
Atopic Eczema	PIAS2	Expression profiling by array	GPL7191	GSE12511	Global Expression Profiling in Atopic Eczema Reveals Reciprocal Expression of Inflammatory and Lipid Genes	Plos One
Atopic Eczema	PRSS3	Expression profiling by array	GPL7191	GSE12511	Global Expression Profiling in Atopic Eczema Reveals Reciprocal Expression of Inflammatory and Lipid Genes	Plos One
Atopic Eczema	PLTP	Expression profiling by array	GPL7191	GSE12511	Global Expression Profiling in Atopic Eczema Reveals Reciprocal Expression of Inflammatory and Lipid Genes	Plos One
Atopic Eczema	SLCO2B1	Expression profiling by array	GPL7191	GSE12511	Global Expression Profiling in Atopic Eczema Reveals Reciprocal Expression of Inflammatory and Lipid Genes	Plos One
Atopic Eczema	LCP1	Expression profiling by array	GPL7191	GSE12511	Global Expression Profiling in Atopic Eczema Reveals Reciprocal Expression of Inflammatory and Lipid Genes	Plos One
Atopic Eczema	MASP1	Expression profiling by array	GPL7191	GSE12511	Global Expression Profiling in Atopic Eczema Reveals Reciprocal Expression of Inflammatory and Lipid Genes	Plos One
Atopic Eczema	TRPS1	Expression profiling by array	GPL7191	GSE12511	Global Expression Profiling in Atopic Eczema Reveals Reciprocal Expression of Inflammatory and Lipid Genes	Plos One
Atopic Eczema	TGM3	Expression profiling by array	GPL7191	GSE12511	Global Expression Profiling in Atopic Eczema Reveals Reciprocal Expression of Inflammatory and Lipid Genes	Plos One
Atopic Eczema	RHOG	Expression profiling by array	GPL7191	GSE12511	Global Expression Profiling in Atopic Eczema Reveals Reciprocal Expression of Inflammatory and Lipid Genes	Plos One
Atopic Eczema	RNASE1	Expression profiling by array	GPL7191	GSE12511	Global Expression Profiling in Atopic Eczema Reveals Reciprocal Expression of Inflammatory and Lipid Genes	Plos One
Atopic Eczema	CLEC2B	Expression profiling by array	GPL7191	GSE12511	Global Expression Profiling in Atopic Eczema Reveals Reciprocal Expression of Inflammatory and Lipid Genes	Plos One
Atopic Eczema	PADI2	Expression profiling by array	GPL7191	GSE12511	Global Expression Profiling in Atopic Eczema Reveals Reciprocal Expression of Inflammatory and Lipid Genes	Plos One
Atopic Eczema	IFI30	Expression profiling by array	GPL7191	GSE12511	Global Expression Profiling in Atopic Eczema Reveals Reciprocal Expression of Inflammatory and Lipid Genes	Plos One
Atopic Eczema	TMEM45B	Expression profiling by array	GPL7191	GSE12511	Global Expression Profiling in Atopic Eczema Reveals Reciprocal Expression of Inflammatory and Lipid Genes	Plos One
Atopic Eczema	PLEKHH1	Expression profiling by array	GPL7191	GSE12511	Global Expression Profiling in Atopic Eczema Reveals Reciprocal Expression of Inflammatory and Lipid Genes	Plos One
Atopic Eczema	MOSC2	Expression profiling by array	GPL7191	GSE12511	Global Expression Profiling in Atopic Eczema Reveals Reciprocal Expression of Inflammatory and Lipid Genes	Plos One
Atopic Eczema	PEX12	Expression profiling by array	GPL7191	GSE12511	Global Expression Profiling in Atopic Eczema Reveals Reciprocal Expression of Inflammatory and Lipid Genes	Plos One
Atopic Eczema	DAZ4	Expression profiling by array	GPL7191	GSE12511	Global Expression Profiling in Atopic Eczema Reveals Reciprocal Expression of Inflammatory and Lipid Genes	Plos One
Atopic Eczema	ASPHD2	Expression profiling by array	GPL7191	GSE12511	Global Expression Profiling in Atopic Eczema Reveals Reciprocal Expression of Inflammatory and Lipid Genes	Plos One
Atopic Eczema	PRC1	Expression profiling by array	GPL7191	GSE12511	Global Expression Profiling in Atopic Eczema Reveals Reciprocal Expression of Inflammatory and Lipid Genes	Plos One
Atopic Eczema	ATAD4	Expression profiling by array	GPL7191	GSE12511	Global Expression Profiling in Atopic Eczema Reveals Reciprocal Expression of Inflammatory and Lipid Genes	Plos One
Atopic Eczema	FNBP1L	Expression profiling by array	GPL7191	GSE12511	Global Expression Profiling in Atopic Eczema Reveals Reciprocal Expression of Inflammatory and Lipid Genes	Plos One
Atopic Eczema	SVEP1	Expression profiling by array	GPL7191	GSE12511	Global Expression Profiling in Atopic Eczema Reveals Reciprocal Expression of Inflammatory and Lipid Genes	Plos One
Atopic Eczema	VMD2	Expression profiling by array	GPL7191	GSE12511	Global Expression Profiling in Atopic Eczema Reveals Reciprocal Expression of Inflammatory and Lipid Genes	Plos One
Atopic Eczema	RER1	Expression profiling by array	GPL7191	GSE12511	Global Expression Profiling in Atopic Eczema Reveals Reciprocal Expression of Inflammatory and Lipid Genes	Plos One
Atopic Eczema	BIN2	Expression profiling by array	GPL7191	GSE12511	Global Expression Profiling in Atopic Eczema Reveals Reciprocal Expression of Inflammatory and Lipid Genes	Plos One
Atopic Eczema	NFE2	Expression profiling by array	GPL7191	GSE12511	Global Expression Profiling in Atopic Eczema Reveals Reciprocal Expression of Inflammatory and Lipid Genes	Plos One
Atopic Eczema	TMPRSS13	Expression profiling by array	GPL7191	GSE12511	Global Expression Profiling in Atopic Eczema Reveals Reciprocal Expression of Inflammatory and Lipid Genes	Plos One
Atopic Eczema	PDGFRB	Expression profiling by array	GPL7191	GSE12511	Global Expression Profiling in Atopic Eczema Reveals Reciprocal Expression of Inflammatory and Lipid Genes	Plos One
Atopic Eczema	DAZ1	Expression profiling by array	GPL7191	GSE12511	Global Expression Profiling in Atopic Eczema Reveals Reciprocal Expression of Inflammatory and Lipid Genes	Plos One
Atopic Eczema	GPR153	Expression profiling by array	GPL7191	GSE12511	Global Expression Profiling in Atopic Eczema Reveals Reciprocal Expression of Inflammatory and Lipid Genes	Plos One
Atopic Eczema	FGL2	Expression profiling by array	GPL7191	GSE12511	Global Expression Profiling in Atopic Eczema Reveals Reciprocal Expression of Inflammatory and Lipid Genes	Plos One
Atopic Eczema	PLOD3	Expression profiling by array	GPL7191	GSE12511	Global Expression Profiling in Atopic Eczema Reveals Reciprocal Expression of Inflammatory and Lipid Genes	Plos One
Atopic Eczema	GTF2H5	Expression profiling by array	GPL7191	GSE12511	Global Expression Profiling in Atopic Eczema Reveals Reciprocal Expression of Inflammatory and Lipid Genes	Plos One
Atopic Eczema	ACOT4	Expression profiling by array	GPL7191	GSE12511	Global Expression Profiling in Atopic Eczema Reveals Reciprocal Expression of Inflammatory and Lipid Genes	Plos One
Atopic Eczema	AADAC	Expression profiling by array	GPL7191	GSE12511	Global Expression Profiling in Atopic Eczema Reveals Reciprocal Expression of Inflammatory and Lipid Genes	Plos One
Atopic Eczema	SLC13A1	Expression profiling by array	GPL7191	GSE12511	Global Expression Profiling in Atopic Eczema Reveals Reciprocal Expression of Inflammatory and Lipid Genes	Plos One
Atopic Eczema	ATP6V1B1	Expression profiling by array	GPL7191	GSE12511	Global Expression Profiling in Atopic Eczema Reveals Reciprocal Expression of Inflammatory and Lipid Genes	Plos One
Atopic Eczema	ARNTL	Expression profiling by array	GPL7191	GSE12511	Global Expression Profiling in Atopic Eczema Reveals Reciprocal Expression of Inflammatory and Lipid Genes	Plos One

Atopic Eczema	FABP7	Expression profiling by array	GPL7191	GSE12511	Global Expression Profiling in Atopic Eczema Reveals Reciprocal Expression of Inflammatory and Lipid Genes	Plos One
Atopic Eczema	TAGLN2	Expression profiling by array	GPL7191	GSE12511	Global Expression Profiling in Atopic Eczema Reveals Reciprocal Expression of Inflammatory and Lipid Genes	Plos One
Atopic Eczema	LOC643790	Expression profiling by array	GPL7191	GSE12511	Global Expression Profiling in Atopic Eczema Reveals Reciprocal Expression of Inflammatory and Lipid Genes	Plos One
Atopic Eczema	SLC39A1	Expression profiling by array	GPL7191	GSE12511	Global Expression Profiling in Atopic Eczema Reveals Reciprocal Expression of Inflammatory and Lipid Genes	Plos One
Atopic Eczema	CDA	Expression profiling by array	GPL7191	GSE12511	Global Expression Profiling in Atopic Eczema Reveals Reciprocal Expression of Inflammatory and Lipid Genes	Plos One
Atopic Eczema	FHL3	Expression profiling by array	GPL7191	GSE12511	Global Expression Profiling in Atopic Eczema Reveals Reciprocal Expression of Inflammatory and Lipid Genes	Plos One
Atopic Eczema	ZNF414	Expression profiling by array	GPL7191	GSE12511	Global Expression Profiling in Atopic Eczema Reveals Reciprocal Expression of Inflammatory and Lipid Genes	Plos One
Atopic Eczema	ALOX5AP	Expression profiling by array	GPL7191	GSE12511	Global Expression Profiling in Atopic Eczema Reveals Reciprocal Expression of Inflammatory and Lipid Genes	Plos One
Atopic Eczema	NLGN2	Expression profiling by array	GPL7191	GSE12511	Global Expression Profiling in Atopic Eczema Reveals Reciprocal Expression of Inflammatory and Lipid Genes	Plos One
Atopic Eczema	MAPK1	Expression profiling by array	GPL7191	GSE12511	Global Expression Profiling in Atopic Eczema Reveals Reciprocal Expression of Inflammatory and Lipid Genes	Plos One
Atopic Eczema	LOC202781	Expression profiling by array	GPL7191	GSE12511	Global Expression Profiling in Atopic Eczema Reveals Reciprocal Expression of Inflammatory and Lipid Genes	Plos One
Atopic Eczema	MAPK8IP2	Expression profiling by array	GPL7191	GSE12511	Global Expression Profiling in Atopic Eczema Reveals Reciprocal Expression of Inflammatory and Lipid Genes	Plos One
Atopic Eczema	GARNL4	Expression profiling by array	GPL7191	GSE12511	Global Expression Profiling in Atopic Eczema Reveals Reciprocal Expression of Inflammatory and Lipid Genes	Plos One
Atopic Eczema	TAX1BP3	Expression profiling by array	GPL7191	GSE12511	Global Expression Profiling in Atopic Eczema Reveals Reciprocal Expression of Inflammatory and Lipid Genes	Plos One
Atopic Eczema	PTK9L	Expression profiling by array	GPL7191	GSE12511	Global Expression Profiling in Atopic Eczema Reveals Reciprocal Expression of Inflammatory and Lipid Genes	Plos One
Atopic Eczema	MDM4	Expression profiling by array	GPL7191	GSE12511	Global Expression Profiling in Atopic Eczema Reveals Reciprocal Expression of Inflammatory and Lipid Genes	Plos One
Atopic Eczema	EPS8L3	Expression profiling by array	GPL7191	GSE12511	Global Expression Profiling in Atopic Eczema Reveals Reciprocal Expression of Inflammatory and Lipid Genes	Plos One
Atopic Eczema	DSG2	Expression profiling by array	GPL7191	GSE12511	Global Expression Profiling in Atopic Eczema Reveals Reciprocal Expression of Inflammatory and Lipid Genes	Plos One
Atopic Eczema	PLCB2	Expression profiling by array	GPL7191	GSE12511	Global Expression Profiling in Atopic Eczema Reveals Reciprocal Expression of Inflammatory and Lipid Genes	Plos One
Atopic Eczema	ELAVL2	Expression profiling by array	GPL7191	GSE12511	Global Expression Profiling in Atopic Eczema Reveals Reciprocal Expression of Inflammatory and Lipid Genes	Plos One
Atopic Eczema	SOAT1	Expression profiling by array	GPL7191	GSE12511	Global Expression Profiling in Atopic Eczema Reveals Reciprocal Expression of Inflammatory and Lipid Genes	Plos One
Atopic Eczema	ITM2C	Expression profiling by array	GPL7191	GSE12511	Global Expression Profiling in Atopic Eczema Reveals Reciprocal Expression of Inflammatory and Lipid Genes	Plos One
Atopic Eczema	CST3	Expression profiling by array	GPL7191	GSE12511	Global Expression Profiling in Atopic Eczema Reveals Reciprocal Expression of Inflammatory and Lipid Genes	Plos One
Atopic Eczema	CAB39L	Expression profiling by array	GPL7191	GSE12511	Global Expression Profiling in Atopic Eczema Reveals Reciprocal Expression of Inflammatory and Lipid Genes	Plos One
Atopic Eczema	CFL1	Expression profiling by array	GPL7191	GSE12511	Global Expression Profiling in Atopic Eczema Reveals Reciprocal Expression of Inflammatory and Lipid Genes	Plos One
Atopic Eczema	ITGB4BP	Expression profiling by array	GPL7191	GSE12511	Global Expression Profiling in Atopic Eczema Reveals Reciprocal Expression of Inflammatory and Lipid Genes	Plos One
Atopic Eczema	FARP1	Expression profiling by array	GPL7191	GSE12511	Global Expression Profiling in Atopic Eczema Reveals Reciprocal Expression of Inflammatory and Lipid Genes	Plos One
Atopic Eczema	S100B	Expression profiling by array	GPL7191	GSE12511	Global Expression Profiling in Atopic Eczema Reveals Reciprocal Expression of Inflammatory and Lipid Genes	Plos One
Atopic Eczema	GFRA1	Expression profiling by array	GPL7191	GSE12511	Global Expression Profiling in Atopic Eczema Reveals Reciprocal Expression of Inflammatory and Lipid Genes	Plos One
Atopic Eczema	PLP2	Expression profiling by array	GPL7191	GSE12511	Global Expression Profiling in Atopic Eczema Reveals Reciprocal Expression of Inflammatory and Lipid Genes	Plos One
Atopic Eczema	STAC2	Expression profiling by array	GPL7191	GSE12511	Global Expression Profiling in Atopic Eczema Reveals Reciprocal Expression of Inflammatory and Lipid Genes	Plos One
Atopic Eczema	CDK6	Expression profiling by array	GPL7191	GSE12511	Global Expression Profiling in Atopic Eczema Reveals Reciprocal Expression of Inflammatory and Lipid Genes	Plos One
Atopic Eczema	RHBDF2	Expression profiling by array	GPL7191	GSE12511	Global Expression Profiling in Atopic Eczema Reveals Reciprocal Expression of Inflammatory and Lipid Genes	Plos One
Atopic Eczema	PRRX1	Expression profiling by array	GPL7191	GSE12511	Global Expression Profiling in Atopic Eczema Reveals Reciprocal Expression of Inflammatory and Lipid Genes	Plos One
Atopic Eczema	NXPH3	Expression profiling by array	GPL7191	GSE12511	Global Expression Profiling in Atopic Eczema Reveals Reciprocal Expression of Inflammatory and Lipid Genes	Plos One
Atopic Eczema	C2	Expression profiling by array	GPL7191	GSE12511	Global Expression Profiling in Atopic Eczema Reveals Reciprocal Expression of Inflammatory and Lipid Genes	Plos One
Atopic Eczema	FA2H	Expression profiling by array	GPL7191	GSE12511	Global Expression Profiling in Atopic Eczema Reveals Reciprocal Expression of Inflammatory and Lipid Genes	Plos One
Atopic Eczema	ADCY7	Expression profiling by array	GPL7191	GSE12511	Global Expression Profiling in Atopic Eczema Reveals Reciprocal Expression of Inflammatory and Lipid Genes	Plos One
Atopic Eczema	SPDEF	Expression profiling by array	GPL7191	GSE12511	Global Expression Profiling in Atopic Eczema Reveals Reciprocal Expression of Inflammatory and Lipid Genes	Plos One
Atopic Eczema	SFRP2	Expression profiling by array	GPL7191	GSE12511	Global Expression Profiling in Atopic Eczema Reveals Reciprocal Expression of Inflammatory and Lipid Genes	Plos One
Atopic Eczema	SAPS1	Expression profiling by array	GPL7191	GSE12511	Global Expression Profiling in Atopic Eczema Reveals Reciprocal Expression of Inflammatory and Lipid Genes	Plos One
Atopic Eczema	AKT1	Expression profiling by array	GPL7191	GSE12511	Global Expression Profiling in Atopic Eczema Reveals Reciprocal Expression of Inflammatory and Lipid Genes	Plos One
Atopic Eczema	SH3BGR	Expression profiling by array	GPL7191	GSE12511	Global Expression Profiling in Atopic Eczema Reveals Reciprocal Expression of Inflammatory and Lipid Genes	Plos One
Atopic Eczema	TYRO3	Expression profiling by array	GPL7191	GSE12511	Global Expression Profiling in Atopic Eczema Reveals Reciprocal Expression of Inflammatory and Lipid Genes	Plos One
Atopic Eczema	ABCG1	Expression profiling by array	GPL7191	GSE12511	Global Expression Profiling in Atopic Eczema Reveals Reciprocal Expression of Inflammatory and Lipid Genes	Plos One
Atopic Eczema	VIM	Expression profiling by array	GPL7191	GSE12511	Global Expression Profiling in Atopic Eczema Reveals Reciprocal Expression of Inflammatory and Lipid Genes	Plos One
Atopic Eczema	IFITM3	Expression profiling by array	GPL7191	GSE12511	Global Expression Profiling in Atopic Eczema Reveals Reciprocal Expression of Inflammatory and Lipid Genes	Plos One
Atopic Eczema	STS-1	Expression profiling by array	GPL7191	GSE12511	Global Expression Profiling in Atopic Eczema Reveals Reciprocal Expression of Inflammatory and Lipid Genes	Plos One
Atopic Eczema	PEF1	Expression profiling by array	GPL7191	GSE12511	Global Expression Profiling in Atopic Eczema Reveals Reciprocal Expression of Inflammatory and Lipid Genes	Plos One
Atopic Eczema	FOXP1	Expression profiling by array	GPL7191	GSE12511	Global Expression Profiling in Atopic Eczema Reveals Reciprocal Expression of Inflammatory and Lipid Genes	Plos One
Atopic Eczema	EIF4G1	Expression profiling by array	GPL7191	GSE12511	Global Expression Profiling in Atopic Eczema Reveals Reciprocal Expression of Inflammatory and Lipid Genes	Plos One
Atopic Eczema	TACSTD1	Expression profiling by array	GPL7191	GSE12511	Global Expression Profiling in Atopic Eczema Reveals Reciprocal Expression of Inflammatory and Lipid Genes	Plos One
Atopic Eczema	SIRT2	Expression profiling by array	GPL7191	GSE12511	Global Expression Profiling in Atopic Eczema Reveals Reciprocal Expression of Inflammatory and Lipid Genes	Plos One
Atopic Eczema	SERPINB7	Expression profiling by array	GPL7191	GSE12511	Global Expression Profiling in Atopic Eczema Reveals Reciprocal Expression of Inflammatory and Lipid Genes	Plos One
Atopic Eczema	LYPD3	Expression profiling by array	GPL7191	GSE12511	Global Expression Profiling in Atopic Eczema Reveals Reciprocal Expression of Inflammatory and Lipid Genes	Plos One
Atopic Eczema	TSPAN4	Expression profiling by array	GPL7191	GSE12511	Global Expression Profiling in Atopic Eczema Reveals Reciprocal Expression of Inflammatory and Lipid Genes	Plos One
Atopic Eczema	C17ORF49	Expression profiling by array	GPL7191	GSE12511	Global Expression Profiling in Atopic Eczema Reveals Reciprocal Expression of Inflammatory and Lipid Genes	Plos One
Atopic Eczema	TMEM142A	Expression profiling by array	GPL7191	GSE12511	Global Expression Profiling in Atopic Eczema Reveals Reciprocal Expression of Inflammatory and Lipid Genes	Plos One
Atopic Eczema	LOC388335	Expression profiling by array	GPL7191	GSE12511	Global Expression Profiling in Atopic Eczema Reveals Reciprocal Expression of Inflammatory and Lipid Genes	Plos One
Atopic Eczema	MTERFD2	Expression profiling by array	GPL7191	GSE12511	Global Expression Profiling in Atopic Eczema Reveals Reciprocal Expression of Inflammatory and Lipid Genes	Plos One
Atopic Eczema	PLEK	Expression profiling by array	GPL7191	GSE12511	Global Expression Profiling in Atopic Eczema Reveals Reciprocal Expression of Inflammatory and Lipid Genes	Plos One
Atopic Eczema	CCL21	Expression profiling by array	GPL7191	GSE12511	Global Expression Profiling in Atopic Eczema Reveals Reciprocal Expression of Inflammatory and Lipid Genes	Plos One

Atopic Eczema	TA-NFKBH	Expression profiling by array	GPL7191	GSE12511	Global Expression Profiling in Atopic Eczema Reveals Reciprocal Expression of Inflammatory and Lipid Genes	Plos One
Atopic Eczema	RAB30	Expression profiling by array	GPL7191	GSE12511	Global Expression Profiling in Atopic Eczema Reveals Reciprocal Expression of Inflammatory and Lipid Genes	Plos One
Atopic Eczema	MUC7	Expression profiling by array	GPL7191	GSE12511	Global Expression Profiling in Atopic Eczema Reveals Reciprocal Expression of Inflammatory and Lipid Genes	Plos One
Atopic Eczema	LY86	Expression profiling by array	GPL7191	GSE12511	Global Expression Profiling in Atopic Eczema Reveals Reciprocal Expression of Inflammatory and Lipid Genes	Plos One
Atopic Eczema	MFNG	Expression profiling by array	GPL7191	GSE12511	Global Expression Profiling in Atopic Eczema Reveals Reciprocal Expression of Inflammatory and Lipid Genes	Plos One
Atopic Eczema	SERTAD1	Expression profiling by array	GPL7191	GSE12511	Global Expression Profiling in Atopic Eczema Reveals Reciprocal Expression of Inflammatory and Lipid Genes	Plos One
Atopic Eczema	C15ORF48	Expression profiling by array	GPL7191	GSE12511	Global Expression Profiling in Atopic Eczema Reveals Reciprocal Expression of Inflammatory and Lipid Genes	Plos One
Atopic Eczema	KIF5A	Expression profiling by array	GPL7191	GSE12511	Global Expression Profiling in Atopic Eczema Reveals Reciprocal Expression of Inflammatory and Lipid Genes	Plos One
Atopic Eczema	FLJ22662	Expression profiling by array	GPL7191	GSE12511	Global Expression Profiling in Atopic Eczema Reveals Reciprocal Expression of Inflammatory and Lipid Genes	Plos One
Atopic Eczema	ISG20	Expression profiling by array	GPL7191	GSE12511	Global Expression Profiling in Atopic Eczema Reveals Reciprocal Expression of Inflammatory and Lipid Genes	Plos One
Atopic Eczema	TEAD4	Expression profiling by array	GPL7191	GSE12511	Global Expression Profiling in Atopic Eczema Reveals Reciprocal Expression of Inflammatory and Lipid Genes	Plos One
Atopic Eczema	SEC14L4	Expression profiling by array	GPL7191	GSE12511	Global Expression Profiling in Atopic Eczema Reveals Reciprocal Expression of Inflammatory and Lipid Genes	Plos One
Atopic Eczema	RHPN2	Expression profiling by array	GPL7191	GSE12511	Global Expression Profiling in Atopic Eczema Reveals Reciprocal Expression of Inflammatory and Lipid Genes	Plos One
Atopic Eczema	C1ORF27	Expression profiling by array	GPL7191	GSE12511	Global Expression Profiling in Atopic Eczema Reveals Reciprocal Expression of Inflammatory and Lipid Genes	Plos One
Atopic Eczema	CTNNBIP1	Expression profiling by array	GPL7191	GSE12511	Global Expression Profiling in Atopic Eczema Reveals Reciprocal Expression of Inflammatory and Lipid Genes	Plos One
Atopic Eczema	GAL	Expression profiling by array	GPL7191	GSE12511	Global Expression Profiling in Atopic Eczema Reveals Reciprocal Expression of Inflammatory and Lipid Genes	Plos One
Atopic Eczema	AP3B2	Expression profiling by array	GPL7191	GSE12511	Global Expression Profiling in Atopic Eczema Reveals Reciprocal Expression of Inflammatory and Lipid Genes	Plos One
Atopic Eczema	ZNF655	Expression profiling by array	GPL7191	GSE12511	Global Expression Profiling in Atopic Eczema Reveals Reciprocal Expression of Inflammatory and Lipid Genes	Plos One
Atopic Eczema	IL1RN	Expression profiling by array	GPL7191	GSE12511	Global Expression Profiling in Atopic Eczema Reveals Reciprocal Expression of Inflammatory and Lipid Genes	Plos One
Atopic Eczema	XLKD1	Expression profiling by array	GPL7191	GSE12511	Global Expression Profiling in Atopic Eczema Reveals Reciprocal Expression of Inflammatory and Lipid Genes	Plos One
Atopic Eczema	CD86	Expression profiling by array	GPL7191	GSE12511	Global Expression Profiling in Atopic Eczema Reveals Reciprocal Expression of Inflammatory and Lipid Genes	Plos One
Atopic Eczema	SPRR2G	Expression profiling by array	GPL7191	GSE12511	Global Expression Profiling in Atopic Eczema Reveals Reciprocal Expression of Inflammatory and Lipid Genes	Plos One
Atopic Eczema	MAF	Expression profiling by array	GPL7191	GSE12511	Global Expression Profiling in Atopic Eczema Reveals Reciprocal Expression of Inflammatory and Lipid Genes	Plos One
Atopic Eczema	NEK6	Expression profiling by array	GPL7191	GSE12511	Global Expression Profiling in Atopic Eczema Reveals Reciprocal Expression of Inflammatory and Lipid Genes	Plos One
Atopic Eczema	TRIM2	Expression profiling by array	GPL7191	GSE12511	Global Expression Profiling in Atopic Eczema Reveals Reciprocal Expression of Inflammatory and Lipid Genes	Plos One
Atopic Eczema	EHD1	Expression profiling by array	GPL7191	GSE12511	Global Expression Profiling in Atopic Eczema Reveals Reciprocal Expression of Inflammatory and Lipid Genes	Plos One
Atopic Eczema	ZDHHC18	Expression profiling by array	GPL7191	GSE12511	Global Expression Profiling in Atopic Eczema Reveals Reciprocal Expression of Inflammatory and Lipid Genes	Plos One
Atopic Eczema	LOC219854	Expression profiling by array	GPL7191	GSE12511	Global Expression Profiling in Atopic Eczema Reveals Reciprocal Expression of Inflammatory and Lipid Genes	Plos One
Atopic Eczema	ZNF706	Expression profiling by array	GPL7191	GSE12511	Global Expression Profiling in Atopic Eczema Reveals Reciprocal Expression of Inflammatory and Lipid Genes	Plos One
Atopic Eczema	TP53I3	Expression profiling by array	GPL7191	GSE12511	Global Expression Profiling in Atopic Eczema Reveals Reciprocal Expression of Inflammatory and Lipid Genes	Plos One
Atopic Eczema	CETN3	Expression profiling by array	GPL7191	GSE12511	Global Expression Profiling in Atopic Eczema Reveals Reciprocal Expression of Inflammatory and Lipid Genes	Plos One
Atopic Eczema	TMEM139	Expression profiling by array	GPL7191	GSE12511	Global Expression Profiling in Atopic Eczema Reveals Reciprocal Expression of Inflammatory and Lipid Genes	Plos One
Atopic Eczema	C1ORF198	Expression profiling by array	GPL7191	GSE12511	Global Expression Profiling in Atopic Eczema Reveals Reciprocal Expression of Inflammatory and Lipid Genes	Plos One
Atopic Eczema	RASGEF1C	Expression profiling by array	GPL7191	GSE12511	Global Expression Profiling in Atopic Eczema Reveals Reciprocal Expression of Inflammatory and Lipid Genes	Plos One
Atopic Eczema	EPHA2	Expression profiling by array	GPL7191	GSE12511	Global Expression Profiling in Atopic Eczema Reveals Reciprocal Expression of Inflammatory and Lipid Genes	Plos One
Atopic Eczema	ZNF117	Expression profiling by array	GPL7191	GSE12511	Global Expression Profiling in Atopic Eczema Reveals Reciprocal Expression of Inflammatory and Lipid Genes	Plos One
Atopic Eczema	PPP1R14C	Expression profiling by array	GPL7191	GSE12511	Global Expression Profiling in Atopic Eczema Reveals Reciprocal Expression of Inflammatory and Lipid Genes	Plos One
Atopic Eczema	BTK	Expression profiling by array	GPL7191	GSE12511	Global Expression Profiling in Atopic Eczema Reveals Reciprocal Expression of Inflammatory and Lipid Genes	Plos One
Atopic Eczema	SELL	Expression profiling by array	GPL7191	GSE12511	Global Expression Profiling in Atopic Eczema Reveals Reciprocal Expression of Inflammatory and Lipid Genes	Plos One
Atopic Eczema	SOSTDC1	Expression profiling by array	GPL7191	GSE12511	Global Expression Profiling in Atopic Eczema Reveals Reciprocal Expression of Inflammatory and Lipid Genes	Plos One
Atopic Eczema	LOC284402	Expression profiling by array	GPL7191	GSE12511	Global Expression Profiling in Atopic Eczema Reveals Reciprocal Expression of Inflammatory and Lipid Genes	Plos One
Atopic Eczema	ACSBG1	Expression profiling by array	GPL7191	GSE12511	Global Expression Profiling in Atopic Eczema Reveals Reciprocal Expression of Inflammatory and Lipid Genes	Plos One
Atopic Eczema	RASSF2	Expression profiling by array	GPL7191	GSE12511	Global Expression Profiling in Atopic Eczema Reveals Reciprocal Expression of Inflammatory and Lipid Genes	Plos One
Atopic Eczema	SERPINF1	Expression profiling by array	GPL7191	GSE12511	Global Expression Profiling in Atopic Eczema Reveals Reciprocal Expression of Inflammatory and Lipid Genes	Plos One
Atopic Eczema	FAM62A	Expression profiling by array	GPL7191	GSE12511	Global Expression Profiling in Atopic Eczema Reveals Reciprocal Expression of Inflammatory and Lipid Genes	Plos One
Atopic Eczema	SLC9A8	Expression profiling by array	GPL7191	GSE12511	Global Expression Profiling in Atopic Eczema Reveals Reciprocal Expression of Inflammatory and Lipid Genes	Plos One
Atopic Eczema	PCSK6	Expression profiling by array	GPL7191	GSE12511	Global Expression Profiling in Atopic Eczema Reveals Reciprocal Expression of Inflammatory and Lipid Genes	Plos One
Atopic Eczema	TCIRG1	Expression profiling by array	GPL7191	GSE12511	Global Expression Profiling in Atopic Eczema Reveals Reciprocal Expression of Inflammatory and Lipid Genes	Plos One
Atopic Eczema	PPP1R3D	Expression profiling by array	GPL7191	GSE12511	Global Expression Profiling in Atopic Eczema Reveals Reciprocal Expression of Inflammatory and Lipid Genes	Plos One
Atopic Eczema	FAM57A	Expression profiling by array	GPL7191	GSE12511	Global Expression Profiling in Atopic Eczema Reveals Reciprocal Expression of Inflammatory and Lipid Genes	Plos One
Atopic Eczema	RGS10	Expression profiling by array	GPL7191	GSE12511	Global Expression Profiling in Atopic Eczema Reveals Reciprocal Expression of Inflammatory and Lipid Genes	Plos One
Atopic Eczema	FAM81A	Expression profiling by array	GPL7191	GSE12511	Global Expression Profiling in Atopic Eczema Reveals Reciprocal Expression of Inflammatory and Lipid Genes	Plos One
Atopic Eczema	PLA2G7	Expression profiling by array	GPL7191	GSE12511	Global Expression Profiling in Atopic Eczema Reveals Reciprocal Expression of Inflammatory and Lipid Genes	Plos One
Atopic Eczema	ACTR1A	Expression profiling by array	GPL7191	GSE12511	Global Expression Profiling in Atopic Eczema Reveals Reciprocal Expression of Inflammatory and Lipid Genes	Plos One
Atopic Eczema	ARHGAP30	Expression profiling by array	GPL7191	GSE12511	Global Expression Profiling in Atopic Eczema Reveals Reciprocal Expression of Inflammatory and Lipid Genes	Plos One
Atopic Eczema	CA6	Expression profiling by array	GPL7191	GSE12511	Global Expression Profiling in Atopic Eczema Reveals Reciprocal Expression of Inflammatory and Lipid Genes	Plos One
Atopic Eczema	TPST1	Expression profiling by array	GPL7191	GSE12511	Global Expression Profiling in Atopic Eczema Reveals Reciprocal Expression of Inflammatory and Lipid Genes	Plos One
Atopic Eczema	SERPINH1	Expression profiling by array	GPL7191	GSE12511	Global Expression Profiling in Atopic Eczema Reveals Reciprocal Expression of Inflammatory and Lipid Genes	Plos One
Atopic Eczema	SCOTIN	Expression profiling by array	GPL7191	GSE12511	Global Expression Profiling in Atopic Eczema Reveals Reciprocal Expression of Inflammatory and Lipid Genes	Plos One
Atopic Eczema	FAM26B	Expression profiling by array	GPL7191	GSE12511	Global Expression Profiling in Atopic Eczema Reveals Reciprocal Expression of Inflammatory and Lipid Genes	Plos One
Atopic Eczema	FAM46B	Expression profiling by array	GPL7191	GSE12511	Global Expression Profiling in Atopic Eczema Reveals Reciprocal Expression of Inflammatory and Lipid Genes	Plos One
Atopic Eczema	MAF1	Expression profiling by array	GPL7191	GSE12511	Global Expression Profiling in Atopic Eczema Reveals Reciprocal Expression of Inflammatory and Lipid Genes	Plos One

Atopic Eczema	KLK3	Expression profiling by array	GPL7191	GSE12511	Global Expression Profiling in Atopic Eczema Reveals Reciprocal Expression of Inflammatory and Lipid Genes	Plos One
Atopic Eczema	ITGAM	Expression profiling by array	GPL7191	GSE12511	Global Expression Profiling in Atopic Eczema Reveals Reciprocal Expression of Inflammatory and Lipid Genes	Plos One
Atopic Eczema	KRT7	Expression profiling by array	GPL7191	GSE12511	Global Expression Profiling in Atopic Eczema Reveals Reciprocal Expression of Inflammatory and Lipid Genes	Plos One
Atopic Eczema	PRCP	Expression profiling by array	GPL7191	GSE12511	Global Expression Profiling in Atopic Eczema Reveals Reciprocal Expression of Inflammatory and Lipid Genes	Plos One
Atopic Eczema	TMEM149	Expression profiling by array	GPL7191	GSE12511	Global Expression Profiling in Atopic Eczema Reveals Reciprocal Expression of Inflammatory and Lipid Genes	Plos One
Atopic Eczema	RASGRP1	Expression profiling by array	GPL7191	GSE12511	Global Expression Profiling in Atopic Eczema Reveals Reciprocal Expression of Inflammatory and Lipid Genes	Plos One
Atopic Eczema	C10ORF7	Expression profiling by array	GPL7191	GSE12511	Global Expression Profiling in Atopic Eczema Reveals Reciprocal Expression of Inflammatory and Lipid Genes	Plos One
Atopic Eczema	FOLR2	Expression profiling by array	GPL7191	GSE12511	Global Expression Profiling in Atopic Eczema Reveals Reciprocal Expression of Inflammatory and Lipid Genes	Plos One
Atopic Eczema	PSMB10	Expression profiling by array	GPL7191	GSE12511	Global Expression Profiling in Atopic Eczema Reveals Reciprocal Expression of Inflammatory and Lipid Genes	Plos One
Atopic Eczema	CD81	Expression profiling by array	GPL7191	GSE12511	Global Expression Profiling in Atopic Eczema Reveals Reciprocal Expression of Inflammatory and Lipid Genes	Plos One
Atopic Eczema	CAPN1	Expression profiling by array	GPL7191	GSE12511	Global Expression Profiling in Atopic Eczema Reveals Reciprocal Expression of Inflammatory and Lipid Genes	Plos One
Atopic Eczema	ZUBR1	Expression profiling by array	GPL7191	GSE12511	Global Expression Profiling in Atopic Eczema Reveals Reciprocal Expression of Inflammatory and Lipid Genes	Plos One
Atopic Eczema	JAK1	Expression profiling by array	GPL7191	GSE12511	Global Expression Profiling in Atopic Eczema Reveals Reciprocal Expression of Inflammatory and Lipid Genes	Plos One
Atopic Eczema	SOCS1	Expression profiling by array	GPL7191	GSE12511	Global Expression Profiling in Atopic Eczema Reveals Reciprocal Expression of Inflammatory and Lipid Genes	Plos One
Atopic Eczema	GAB1	Expression profiling by array	GPL7191	GSE12511	Global Expression Profiling in Atopic Eczema Reveals Reciprocal Expression of Inflammatory and Lipid Genes	Plos One
Atopic Eczema	RNF36	Expression profiling by array	GPL7191	GSE12511	Global Expression Profiling in Atopic Eczema Reveals Reciprocal Expression of Inflammatory and Lipid Genes	Plos One
Atopic Eczema	KLF3	Expression profiling by array	GPL7191	GSE12511	Global Expression Profiling in Atopic Eczema Reveals Reciprocal Expression of Inflammatory and Lipid Genes	Plos One
Atopic Eczema	PHLDA1	Expression profiling by array	GPL7191	GSE12511	Global Expression Profiling in Atopic Eczema Reveals Reciprocal Expression of Inflammatory and Lipid Genes	Plos One
Atopic Eczema	MGC48595	Expression profiling by array	GPL7191	GSE12511	Global Expression Profiling in Atopic Eczema Reveals Reciprocal Expression of Inflammatory and Lipid Genes	Plos One
Atopic Eczema	FBXO32	Expression profiling by array	GPL7191	GSE12511	Global Expression Profiling in Atopic Eczema Reveals Reciprocal Expression of Inflammatory and Lipid Genes	Plos One
Atopic Eczema	RBCK1	Expression profiling by array	GPL7191	GSE12511	Global Expression Profiling in Atopic Eczema Reveals Reciprocal Expression of Inflammatory and Lipid Genes	Plos One
Atopic Eczema	CD48	Expression profiling by array	GPL7191	GSE12511	Global Expression Profiling in Atopic Eczema Reveals Reciprocal Expression of Inflammatory and Lipid Genes	Plos One
Atopic Eczema	SIAH2	Expression profiling by array	GPL7191	GSE12511	Global Expression Profiling in Atopic Eczema Reveals Reciprocal Expression of Inflammatory and Lipid Genes	Plos One
Atopic Eczema	D4S234E	Expression profiling by array	GPL7191	GSE12511	Global Expression Profiling in Atopic Eczema Reveals Reciprocal Expression of Inflammatory and Lipid Genes	Plos One
Atopic Eczema	RHOC	Expression profiling by array	GPL7191	GSE12511	Global Expression Profiling in Atopic Eczema Reveals Reciprocal Expression of Inflammatory and Lipid Genes	Plos One
Atopic Eczema	RPL10L	Expression profiling by array	GPL7191	GSE12511	Global Expression Profiling in Atopic Eczema Reveals Reciprocal Expression of Inflammatory and Lipid Genes	Plos One
Atopic Eczema	NAGA	Expression profiling by array	GPL7191	GSE12511	Global Expression Profiling in Atopic Eczema Reveals Reciprocal Expression of Inflammatory and Lipid Genes	Plos One
Atopic Eczema	PLXND1	Expression profiling by array	GPL7191	GSE12511	Global Expression Profiling in Atopic Eczema Reveals Reciprocal Expression of Inflammatory and Lipid Genes	Plos One
Atopic Eczema	CLDN10	Expression profiling by array	GPL7191	GSE12511	Global Expression Profiling in Atopic Eczema Reveals Reciprocal Expression of Inflammatory and Lipid Genes	Plos One
Atopic Eczema	AMPD2	Expression profiling by array	GPL7191	GSE12511	Global Expression Profiling in Atopic Eczema Reveals Reciprocal Expression of Inflammatory and Lipid Genes	Plos One
Atopic Eczema	SERPINB5	Expression profiling by array	GPL7191	GSE12511	Global Expression Profiling in Atopic Eczema Reveals Reciprocal Expression of Inflammatory and Lipid Genes	Plos One
Atopic Eczema	ZNRD1	Expression profiling by array	GPL7191	GSE12511	Global Expression Profiling in Atopic Eczema Reveals Reciprocal Expression of Inflammatory and Lipid Genes	Plos One
Atopic Eczema	TRAF3IP1	Expression profiling by array	GPL7191	GSE12511	Global Expression Profiling in Atopic Eczema Reveals Reciprocal Expression of Inflammatory and Lipid Genes	Plos One
Atopic Eczema	ZNF138	Expression profiling by array	GPL7191	GSE12511	Global Expression Profiling in Atopic Eczema Reveals Reciprocal Expression of Inflammatory and Lipid Genes	Plos One
Atopic Eczema	GPR55	Expression profiling by array	GPL7191	GSE12511	Global Expression Profiling in Atopic Eczema Reveals Reciprocal Expression of Inflammatory and Lipid Genes	Plos One
Atopic Eczema	FGFR1	Expression profiling by array	GPL7191	GSE12511	Global Expression Profiling in Atopic Eczema Reveals Reciprocal Expression of Inflammatory and Lipid Genes	Plos One
Atopic Eczema	COPA	Expression profiling by array	GPL7191	GSE12511	Global Expression Profiling in Atopic Eczema Reveals Reciprocal Expression of Inflammatory and Lipid Genes	Plos One
Atopic Eczema	CORO2A	Expression profiling by array	GPL7191	GSE12511	Global Expression Profiling in Atopic Eczema Reveals Reciprocal Expression of Inflammatory and Lipid Genes	Plos One
Atopic Eczema	RALGPS1	Expression profiling by array	GPL7191	GSE12511	Global Expression Profiling in Atopic Eczema Reveals Reciprocal Expression of Inflammatory and Lipid Genes	Plos One
Atopic Eczema	C2ORF32	Expression profiling by array	GPL7191	GSE12511	Global Expression Profiling in Atopic Eczema Reveals Reciprocal Expression of Inflammatory and Lipid Genes	Plos One
Atopic Eczema	KLK10	Expression profiling by array	GPL7191	GSE12511	Global Expression Profiling in Atopic Eczema Reveals Reciprocal Expression of Inflammatory and Lipid Genes	Plos One
Atopic Eczema	NAPG	Expression profiling by array	GPL7191	GSE12511	Global Expression Profiling in Atopic Eczema Reveals Reciprocal Expression of Inflammatory and Lipid Genes	Plos One
Atopic Eczema	CCNI	Expression profiling by array	GPL7191	GSE12511	Global Expression Profiling in Atopic Eczema Reveals Reciprocal Expression of Inflammatory and Lipid Genes	Plos One
Atopic Eczema	COPG2	Expression profiling by array	GPL7191	GSE12511	Global Expression Profiling in Atopic Eczema Reveals Reciprocal Expression of Inflammatory and Lipid Genes	Plos One
Atopic Eczema	CDH17	Expression profiling by array	GPL7191	GSE12511	Global Expression Profiling in Atopic Eczema Reveals Reciprocal Expression of Inflammatory and Lipid Genes	Plos One
Atopic Eczema	TMEPAI	Expression profiling by array	GPL7191	GSE12511	Global Expression Profiling in Atopic Eczema Reveals Reciprocal Expression of Inflammatory and Lipid Genes	Plos One
Atopic Eczema	RGS3	Expression profiling by array	GPL7191	GSE12511	Global Expression Profiling in Atopic Eczema Reveals Reciprocal Expression of Inflammatory and Lipid Genes	Plos One
Atopic Eczema	CAPG	Expression profiling by array	GPL7191	GSE12511	Global Expression Profiling in Atopic Eczema Reveals Reciprocal Expression of Inflammatory and Lipid Genes	Plos One
Atopic Eczema	CKMT2	Expression profiling by array	GPL7191	GSE12511	Global Expression Profiling in Atopic Eczema Reveals Reciprocal Expression of Inflammatory and Lipid Genes	Plos One
Atopic Eczema	CTSS	Expression profiling by array	GPL7191	GSE12511	Global Expression Profiling in Atopic Eczema Reveals Reciprocal Expression of Inflammatory and Lipid Genes	Plos One
Atopic Eczema	EPHX1	Expression profiling by array	GPL7191	GSE12511	Global Expression Profiling in Atopic Eczema Reveals Reciprocal Expression of Inflammatory and Lipid Genes	Plos One
Atopic Eczema	FRAP1	Expression profiling by array	GPL7191	GSE12511	Global Expression Profiling in Atopic Eczema Reveals Reciprocal Expression of Inflammatory and Lipid Genes	Plos One
Atopic Eczema	TNRC5	Expression profiling by array	GPL7191	GSE12511	Global Expression Profiling in Atopic Eczema Reveals Reciprocal Expression of Inflammatory and Lipid Genes	Plos One
Atopic Eczema	APH1B	Expression profiling by array	GPL7191	GSE12511	Global Expression Profiling in Atopic Eczema Reveals Reciprocal Expression of Inflammatory and Lipid Genes	Plos One
Atopic Eczema	KLF6	Expression profiling by array	GPL7191	GSE12511	Global Expression Profiling in Atopic Eczema Reveals Reciprocal Expression of Inflammatory and Lipid Genes	Plos One
Atopic Eczema	CTH	Expression profiling by array	GPL7191	GSE12511	Global Expression Profiling in Atopic Eczema Reveals Reciprocal Expression of Inflammatory and Lipid Genes	Plos One
Atopic Eczema	SC5DL	Expression profiling by array	GPL7191	GSE12511	Global Expression Profiling in Atopic Eczema Reveals Reciprocal Expression of Inflammatory and Lipid Genes	Plos One
Atopic Eczema	IL6ST	Expression profiling by array	GPL7191	GSE12511	Global Expression Profiling in Atopic Eczema Reveals Reciprocal Expression of Inflammatory and Lipid Genes	Plos One
Atopic Eczema	FREQ	Expression profiling by array	GPL7191	GSE12511	Global Expression Profiling in Atopic Eczema Reveals Reciprocal Expression of Inflammatory and Lipid Genes	Plos One
Atopic Eczema	ITGAX	Expression profiling by array	GPL7191	GSE12511	Global Expression Profiling in Atopic Eczema Reveals Reciprocal Expression of Inflammatory and Lipid Genes	Plos One
Atopic Eczema	F12	Expression profiling by array	GPL7191	GSE12511	Global Expression Profiling in Atopic Eczema Reveals Reciprocal Expression of Inflammatory and Lipid Genes	Plos One
Atopic Eczema	ADNP	Expression profiling by array	GPL7191	GSE12511	Global Expression Profiling in Atopic Eczema Reveals Reciprocal Expression of Inflammatory and Lipid Genes	Plos One

Atopic Eczema	C3ORF52	Expression profiling by array	GPL7191	GSE12511	Global Expression Profiling in Atopic Eczema Reveals Reciprocal Expression of Inflammatory and Lipid Genes	Plos One
Atopic Eczema	FLJ39575	Expression profiling by array	GPL7191	GSE12511	Global Expression Profiling in Atopic Eczema Reveals Reciprocal Expression of Inflammatory and Lipid Genes	Plos One
Atopic Eczema	ARHGDIB	Expression profiling by array	GPL7191	GSE12511	Global Expression Profiling in Atopic Eczema Reveals Reciprocal Expression of Inflammatory and Lipid Genes	Plos One
Atopic Eczema	AP2A2	Expression profiling by array	GPL7191	GSE12511	Global Expression Profiling in Atopic Eczema Reveals Reciprocal Expression of Inflammatory and Lipid Genes	Plos One
Atopic Eczema	NAV2	Expression profiling by array	GPL7191	GSE12511	Global Expression Profiling in Atopic Eczema Reveals Reciprocal Expression of Inflammatory and Lipid Genes	Plos One
Atopic Eczema	CSNK1A1	Expression profiling by array	GPL7191	GSE12511	Global Expression Profiling in Atopic Eczema Reveals Reciprocal Expression of Inflammatory and Lipid Genes	Plos One
Atopic Eczema	ITGB7	Expression profiling by array	GPL7191	GSE12511	Global Expression Profiling in Atopic Eczema Reveals Reciprocal Expression of Inflammatory and Lipid Genes	Plos One
Atopic Eczema	TRA@	Expression profiling by array	GPL7191	GSE12511	Global Expression Profiling in Atopic Eczema Reveals Reciprocal Expression of Inflammatory and Lipid Genes	Plos One
Atopic Eczema	MAP3K3	Expression profiling by array	GPL7191	GSE12511	Global Expression Profiling in Atopic Eczema Reveals Reciprocal Expression of Inflammatory and Lipid Genes	Plos One
Atopic Eczema	RPLP0	Expression profiling by array	GPL7191	GSE12511	Global Expression Profiling in Atopic Eczema Reveals Reciprocal Expression of Inflammatory and Lipid Genes	Plos One
Atopic Eczema	SLC9A9	Expression profiling by array	GPL7191	GSE12511	Global Expression Profiling in Atopic Eczema Reveals Reciprocal Expression of Inflammatory and Lipid Genes	Plos One
Atopic Eczema	GNE	Expression profiling by array	GPL7191	GSE12511	Global Expression Profiling in Atopic Eczema Reveals Reciprocal Expression of Inflammatory and Lipid Genes	Plos One
Atopic Eczema	GJB7	Expression profiling by array	GPL7191	GSE12511	Global Expression Profiling in Atopic Eczema Reveals Reciprocal Expression of Inflammatory and Lipid Genes	Plos One
Atopic Eczema	JAK3	Expression profiling by array	GPL7191	GSE12511	Global Expression Profiling in Atopic Eczema Reveals Reciprocal Expression of Inflammatory and Lipid Genes	Plos One
Atopic Eczema	ASPN	Expression profiling by array	GPL7191	GSE12511	Global Expression Profiling in Atopic Eczema Reveals Reciprocal Expression of Inflammatory and Lipid Genes	Plos One
Atopic Eczema	SEC24C	Expression profiling by array	GPL7191	GSE12511	Global Expression Profiling in Atopic Eczema Reveals Reciprocal Expression of Inflammatory and Lipid Genes	Plos One
Atopic Eczema	JUP	Expression profiling by array	GPL7191	GSE12511	Global Expression Profiling in Atopic Eczema Reveals Reciprocal Expression of Inflammatory and Lipid Genes	Plos One
Atopic Eczema	KALRN	Expression profiling by array	GPL7191	GSE12511	Global Expression Profiling in Atopic Eczema Reveals Reciprocal Expression of Inflammatory and Lipid Genes	Plos One
Atopic Eczema	LAMB2	Expression profiling by array	GPL7191	GSE12511	Global Expression Profiling in Atopic Eczema Reveals Reciprocal Expression of Inflammatory and Lipid Genes	Plos One
Atopic Eczema	GLS	Expression profiling by array	GPL7191	GSE12511	Global Expression Profiling in Atopic Eczema Reveals Reciprocal Expression of Inflammatory and Lipid Genes	Plos One
Atopic Eczema	SCGB2A2	Expression profiling by array	GPL7191	GSE12511	Global Expression Profiling in Atopic Eczema Reveals Reciprocal Expression of Inflammatory and Lipid Genes	Plos One
Atopic Eczema	THSD4	Expression profiling by array	GPL7191	GSE12511	Global Expression Profiling in Atopic Eczema Reveals Reciprocal Expression of Inflammatory and Lipid Genes	Plos One
Atopic Eczema	GTF2F2	Expression profiling by array	GPL7191	GSE12511	Global Expression Profiling in Atopic Eczema Reveals Reciprocal Expression of Inflammatory and Lipid Genes	Plos One
Atopic Eczema	BTF3	Expression profiling by array	GPL7191	GSE12511	Global Expression Profiling in Atopic Eczema Reveals Reciprocal Expression of Inflammatory and Lipid Genes	Plos One
Atopic Eczema	CTNS	Expression profiling by array	GPL7191	GSE12511	Global Expression Profiling in Atopic Eczema Reveals Reciprocal Expression of Inflammatory and Lipid Genes	Plos One
Atopic Eczema	UBE2Z	Expression profiling by array	GPL7191	GSE12511	Global Expression Profiling in Atopic Eczema Reveals Reciprocal Expression of Inflammatory and Lipid Genes	Plos One
Atopic Eczema	SSBP2	Expression profiling by array	GPL7191	GSE12511	Global Expression Profiling in Atopic Eczema Reveals Reciprocal Expression of Inflammatory and Lipid Genes	Plos One
Atopic Eczema	WBSCR17	Expression profiling by array	GPL7191	GSE12511	Global Expression Profiling in Atopic Eczema Reveals Reciprocal Expression of Inflammatory and Lipid Genes	Plos One
Atopic Eczema	SRPX	Expression profiling by array	GPL7191	GSE12511	Global Expression Profiling in Atopic Eczema Reveals Reciprocal Expression of Inflammatory and Lipid Genes	Plos One
Atopic Eczema	CFH	Expression profiling by array	GPL7191	GSE12511	Global Expression Profiling in Atopic Eczema Reveals Reciprocal Expression of Inflammatory and Lipid Genes	Plos One
Atopic Eczema	EDG2	Expression profiling by array	GPL7191	GSE12511	Global Expression Profiling in Atopic Eczema Reveals Reciprocal Expression of Inflammatory and Lipid Genes	Plos One
Atopic Eczema	RERG	Expression profiling by array	GPL7191	GSE12511	Global Expression Profiling in Atopic Eczema Reveals Reciprocal Expression of Inflammatory and Lipid Genes	Plos One
Atopic Eczema	LOC646504	Expression profiling by array	GPL7191	GSE12511	Global Expression Profiling in Atopic Eczema Reveals Reciprocal Expression of Inflammatory and Lipid Genes	Plos One
Atopic Eczema	PDIA5	Expression profiling by array	GPL7191	GSE12511	Global Expression Profiling in Atopic Eczema Reveals Reciprocal Expression of Inflammatory and Lipid Genes	Plos One
Atopic Eczema	NAGPA	Expression profiling by array	GPL7191	GSE12511	Global Expression Profiling in Atopic Eczema Reveals Reciprocal Expression of Inflammatory and Lipid Genes	Plos One
Atopic Eczema	SCNN1G	Expression profiling by array	GPL7191	GSE12511	Global Expression Profiling in Atopic Eczema Reveals Reciprocal Expression of Inflammatory and Lipid Genes	Plos One
Atopic Eczema	RTN4	Expression profiling by array	GPL7191	GSE12511	Global Expression Profiling in Atopic Eczema Reveals Reciprocal Expression of Inflammatory and Lipid Genes	Plos One
Atopic Eczema	GGT2	Expression profiling by array	GPL7191	GSE12511	Global Expression Profiling in Atopic Eczema Reveals Reciprocal Expression of Inflammatory and Lipid Genes	Plos One
Atopic Eczema	B3GALT5	Expression profiling by array	GPL7191	GSE12511	Global Expression Profiling in Atopic Eczema Reveals Reciprocal Expression of Inflammatory and Lipid Genes	Plos One
Atopic Eczema	NXF4	Expression profiling by array	GPL7191	GSE12511	Global Expression Profiling in Atopic Eczema Reveals Reciprocal Expression of Inflammatory and Lipid Genes	Plos One
Atopic Eczema	ZNF397	Expression profiling by array	GPL7191	GSE12511	Global Expression Profiling in Atopic Eczema Reveals Reciprocal Expression of Inflammatory and Lipid Genes	Plos One
Atopic Eczema	IMPDH2	Expression profiling by array	GPL7191	GSE12511	Global Expression Profiling in Atopic Eczema Reveals Reciprocal Expression of Inflammatory and Lipid Genes	Plos One
Atopic Eczema	FMNL1	Expression profiling by array	GPL7191	GSE12511	Global Expression Profiling in Atopic Eczema Reveals Reciprocal Expression of Inflammatory and Lipid Genes	Plos One
Atopic Eczema	SPIRE2	Expression profiling by array	GPL7191	GSE12511	Global Expression Profiling in Atopic Eczema Reveals Reciprocal Expression of Inflammatory and Lipid Genes	Plos One
Atopic Eczema	CLIC2	Expression profiling by array	GPL7191	GSE12511	Global Expression Profiling in Atopic Eczema Reveals Reciprocal Expression of Inflammatory and Lipid Genes	Plos One
Atopic Eczema	LGALS4	Expression profiling by array	GPL7191	GSE12511	Global Expression Profiling in Atopic Eczema Reveals Reciprocal Expression of Inflammatory and Lipid Genes	Plos One
Atopic Eczema	RXRA	Expression profiling by array	GPL7191	GSE12511	Global Expression Profiling in Atopic Eczema Reveals Reciprocal Expression of Inflammatory and Lipid Genes	Plos One
Atopic Eczema	FAS	Expression profiling by array	GPL7191	GSE12511	Global Expression Profiling in Atopic Eczema Reveals Reciprocal Expression of Inflammatory and Lipid Genes	Plos One
Atopic Eczema	NNAT	Expression profiling by array	GPL7191	GSE12511	Global Expression Profiling in Atopic Eczema Reveals Reciprocal Expression of Inflammatory and Lipid Genes	Plos One
Atopic Eczema	MGC42367	Expression profiling by array	GPL7191	GSE12511	Global Expression Profiling in Atopic Eczema Reveals Reciprocal Expression of Inflammatory and Lipid Genes	Plos One
Atopic Eczema	LMO2	Expression profiling by array	GPL7191	GSE12511	Global Expression Profiling in Atopic Eczema Reveals Reciprocal Expression of Inflammatory and Lipid Genes	Plos One
Atopic Eczema	TXNRD1	Expression profiling by array	GPL7191	GSE12511	Global Expression Profiling in Atopic Eczema Reveals Reciprocal Expression of Inflammatory and Lipid Genes	Plos One
Atopic Eczema	KCTD5	Expression profiling by array	GPL7191	GSE12511	Global Expression Profiling in Atopic Eczema Reveals Reciprocal Expression of Inflammatory and Lipid Genes	Plos One
Atopic Eczema	CD2BP2	Expression profiling by array	GPL7191	GSE12511	Global Expression Profiling in Atopic Eczema Reveals Reciprocal Expression of Inflammatory and Lipid Genes	Plos One
Atopic Eczema	FMO1	Expression profiling by array	GPL7191	GSE12511	Global Expression Profiling in Atopic Eczema Reveals Reciprocal Expression of Inflammatory and Lipid Genes	Plos One
Atopic Eczema	SYN2	Expression profiling by array	GPL7191	GSE12511	Global Expression Profiling in Atopic Eczema Reveals Reciprocal Expression of Inflammatory and Lipid Genes	Plos One
Atopic Eczema	EXT1	Expression profiling by array	GPL7191	GSE12511	Global Expression Profiling in Atopic Eczema Reveals Reciprocal Expression of Inflammatory and Lipid Genes	Plos One
Atopic Eczema	CGNL1	Expression profiling by array	GPL7191	GSE12511	Global Expression Profiling in Atopic Eczema Reveals Reciprocal Expression of Inflammatory and Lipid Genes	Plos One
Atopic Eczema	C1QTNF1	Expression profiling by array	GPL7191	GSE12511	Global Expression Profiling in Atopic Eczema Reveals Reciprocal Expression of Inflammatory and Lipid Genes	Plos One
Atopic Eczema	RAB3B	Expression profiling by array	GPL7191	GSE12511	Global Expression Profiling in Atopic Eczema Reveals Reciprocal Expression of Inflammatory and Lipid Genes	Plos One
Atopic Eczema	TIMM17B	Expression profiling by array	GPL7191	GSE12511	Global Expression Profiling in Atopic Eczema Reveals Reciprocal Expression of Inflammatory and Lipid Genes	Plos One
Atopic Eczema	APOBEC3F	Expression profiling by array	GPL7191	GSE12511	Global Expression Profiling in Atopic Eczema Reveals Reciprocal Expression of Inflammatory and Lipid Genes	Plos One

Atopic Eczema	LOC646652	Expression profiling by array	GPL7191	GSE12511	Global Expression Profiling in Atopic Eczema Reveals Reciprocal Expression of Inflammatory and Lipid Genes	Plos One
Atopic Eczema	PHF19	Expression profiling by array	GPL7191	GSE12511	Global Expression Profiling in Atopic Eczema Reveals Reciprocal Expression of Inflammatory and Lipid Genes	Plos One
Atopic Eczema	SLC25A18	Expression profiling by array	GPL7191	GSE12511	Global Expression Profiling in Atopic Eczema Reveals Reciprocal Expression of Inflammatory and Lipid Genes	Plos One
Atopic Eczema	SEPT9	Expression profiling by array	GPL7191	GSE12511	Global Expression Profiling in Atopic Eczema Reveals Reciprocal Expression of Inflammatory and Lipid Genes	Plos One
Atopic Eczema	P2RX4	Expression profiling by array	GPL7191	GSE12511	Global Expression Profiling in Atopic Eczema Reveals Reciprocal Expression of Inflammatory and Lipid Genes	Plos One
Atopic Eczema	ZNF415	Expression profiling by array	GPL7191	GSE12511	Global Expression Profiling in Atopic Eczema Reveals Reciprocal Expression of Inflammatory and Lipid Genes	Plos One
Atopic Eczema	LY96	Expression profiling by array	GPL7191	GSE12511	Global Expression Profiling in Atopic Eczema Reveals Reciprocal Expression of Inflammatory and Lipid Genes	Plos One
Atopic Eczema	EEF1G	Expression profiling by array	GPL7191	GSE12511	Global Expression Profiling in Atopic Eczema Reveals Reciprocal Expression of Inflammatory and Lipid Genes	Plos One
Atopic Eczema	ALDOC	Expression profiling by array	GPL7191	GSE12511	Global Expression Profiling in Atopic Eczema Reveals Reciprocal Expression of Inflammatory and Lipid Genes	Plos One
Atopic Eczema	BIN3	Expression profiling by array	GPL7191	GSE12511	Global Expression Profiling in Atopic Eczema Reveals Reciprocal Expression of Inflammatory and Lipid Genes	Plos One
Atopic Eczema	ETS1	Expression profiling by array	GPL7191	GSE12511	Global Expression Profiling in Atopic Eczema Reveals Reciprocal Expression of Inflammatory and Lipid Genes	Plos One
Atopic Eczema	LOC130576	Expression profiling by array	GPL7191	GSE12511	Global Expression Profiling in Atopic Eczema Reveals Reciprocal Expression of Inflammatory and Lipid Genes	Plos One
Atopic Eczema	KCNK6	Expression profiling by array	GPL7191	GSE12511	Global Expression Profiling in Atopic Eczema Reveals Reciprocal Expression of Inflammatory and Lipid Genes	Plos One
Atopic Eczema	TNFAIP8L1	Expression profiling by array	GPL7191	GSE12511	Global Expression Profiling in Atopic Eczema Reveals Reciprocal Expression of Inflammatory and Lipid Genes	Plos One
Atopic Eczema	SREBF1	Expression profiling by array	GPL7191	GSE12511	Global Expression Profiling in Atopic Eczema Reveals Reciprocal Expression of Inflammatory and Lipid Genes	Plos One
Atopic Eczema	ZNF480	Expression profiling by array	GPL7191	GSE12511	Global Expression Profiling in Atopic Eczema Reveals Reciprocal Expression of Inflammatory and Lipid Genes	Plos One
Atopic Eczema	RAB33A	Expression profiling by array	GPL7191	GSE12511	Global Expression Profiling in Atopic Eczema Reveals Reciprocal Expression of Inflammatory and Lipid Genes	Plos One
Atopic Eczema	PI16	Expression profiling by array	GPL7191	GSE12511	Global Expression Profiling in Atopic Eczema Reveals Reciprocal Expression of Inflammatory and Lipid Genes	Plos One
Atopic Eczema	EHD4	Expression profiling by array	GPL7191	GSE12511	Global Expression Profiling in Atopic Eczema Reveals Reciprocal Expression of Inflammatory and Lipid Genes	Plos One
Atopic Eczema	PRIC285	Expression profiling by array	GPL7191	GSE12511	Global Expression Profiling in Atopic Eczema Reveals Reciprocal Expression of Inflammatory and Lipid Genes	Plos One
Atopic Eczema	ARHGEF6	Expression profiling by array	GPL7191	GSE12511	Global Expression Profiling in Atopic Eczema Reveals Reciprocal Expression of Inflammatory and Lipid Genes	Plos One
Atopic Eczema	AIP	Expression profiling by array	GPL7191	GSE12511	Global Expression Profiling in Atopic Eczema Reveals Reciprocal Expression of Inflammatory and Lipid Genes	Plos One
Atopic Eczema	LOC389289	Expression profiling by array	GPL7191	GSE12511	Global Expression Profiling in Atopic Eczema Reveals Reciprocal Expression of Inflammatory and Lipid Genes	Plos One
Atopic Eczema	KRIT1	Expression profiling by array	GPL7191	GSE12511	Global Expression Profiling in Atopic Eczema Reveals Reciprocal Expression of Inflammatory and Lipid Genes	Plos One
Atopic Eczema	NPTX2	Expression profiling by array	GPL7191	GSE12511	Global Expression Profiling in Atopic Eczema Reveals Reciprocal Expression of Inflammatory and Lipid Genes	Plos One
Atopic Eczema	JUN	Expression profiling by array	GPL7191	GSE12511	Global Expression Profiling in Atopic Eczema Reveals Reciprocal Expression of Inflammatory and Lipid Genes	Plos One
Atopic Eczema	DLG2	Expression profiling by array	GPL7191	GSE12511	Global Expression Profiling in Atopic Eczema Reveals Reciprocal Expression of Inflammatory and Lipid Genes	Plos One
Atopic Eczema	SLC7A7	Expression profiling by array	GPL7191	GSE12511	Global Expression Profiling in Atopic Eczema Reveals Reciprocal Expression of Inflammatory and Lipid Genes	Plos One
Atopic Eczema	CD97	Expression profiling by array	GPL7191	GSE12511	Global Expression Profiling in Atopic Eczema Reveals Reciprocal Expression of Inflammatory and Lipid Genes	Plos One
Atopic Eczema	LOC283537	Expression profiling by array	GPL7191	GSE12511	Global Expression Profiling in Atopic Eczema Reveals Reciprocal Expression of Inflammatory and Lipid Genes	Plos One
Atopic Eczema	MICA	Expression profiling by array	GPL7191	GSE12511	Global Expression Profiling in Atopic Eczema Reveals Reciprocal Expression of Inflammatory and Lipid Genes	Plos One
Atopic Eczema	CDC42EP1	Expression profiling by array	GPL7191	GSE12511	Global Expression Profiling in Atopic Eczema Reveals Reciprocal Expression of Inflammatory and Lipid Genes	Plos One
Atopic Eczema	ACPP	Expression profiling by array	GPL7191	GSE12511	Global Expression Profiling in Atopic Eczema Reveals Reciprocal Expression of Inflammatory and Lipid Genes	Plos One
Atopic Eczema	AGTR1	Expression profiling by array	GPL7191	GSE12511	Global Expression Profiling in Atopic Eczema Reveals Reciprocal Expression of Inflammatory and Lipid Genes	Plos One
Atopic Eczema	RPL27A	Expression profiling by array	GPL7191	GSE12511	Global Expression Profiling in Atopic Eczema Reveals Reciprocal Expression of Inflammatory and Lipid Genes	Plos One
Atopic Eczema	KLHL18	Expression profiling by array	GPL7191	GSE12511	Global Expression Profiling in Atopic Eczema Reveals Reciprocal Expression of Inflammatory and Lipid Genes	Plos One
Atopic Eczema	LOC158381	Expression profiling by array	GPL7191	GSE12511	Global Expression Profiling in Atopic Eczema Reveals Reciprocal Expression of Inflammatory and Lipid Genes	Plos One
Atopic Eczema	ADAMTS10	Expression profiling by array	GPL7191	GSE12511	Global Expression Profiling in Atopic Eczema Reveals Reciprocal Expression of Inflammatory and Lipid Genes	Plos One
Atopic Eczema	WASPIP	Expression profiling by array	GPL7191	GSE12511	Global Expression Profiling in Atopic Eczema Reveals Reciprocal Expression of Inflammatory and Lipid Genes	Plos One
Atopic Eczema	SMAD3	Expression profiling by array	GPL7191	GSE12511	Global Expression Profiling in Atopic Eczema Reveals Reciprocal Expression of Inflammatory and Lipid Genes	Plos One
Atopic Eczema	FZD4	Expression profiling by array	GPL7191	GSE12511	Global Expression Profiling in Atopic Eczema Reveals Reciprocal Expression of Inflammatory and Lipid Genes	Plos One
Atopic Eczema	MFAP4	Expression profiling by array	GPL7191	GSE12511	Global Expression Profiling in Atopic Eczema Reveals Reciprocal Expression of Inflammatory and Lipid Genes	Plos One
Atopic Eczema	BACE2	Expression profiling by array	GPL7191	GSE12511	Global Expression Profiling in Atopic Eczema Reveals Reciprocal Expression of Inflammatory and Lipid Genes	Plos One
Atopic Eczema	ANKH	Expression profiling by array	GPL7191	GSE12511	Global Expression Profiling in Atopic Eczema Reveals Reciprocal Expression of Inflammatory and Lipid Genes	Plos One
Atopic Eczema	CDK4	Expression profiling by array	GPL7191	GSE12511	Global Expression Profiling in Atopic Eczema Reveals Reciprocal Expression of Inflammatory and Lipid Genes	Plos One
Atopic Eczema	PRDM5	Expression profiling by array	GPL7191	GSE12511	Global Expression Profiling in Atopic Eczema Reveals Reciprocal Expression of Inflammatory and Lipid Genes	Plos One
Atopic Eczema	SFRP1	Expression profiling by array	GPL7191	GSE12511	Global Expression Profiling in Atopic Eczema Reveals Reciprocal Expression of Inflammatory and Lipid Genes	Plos One
Atopic Eczema	SEC61A1	Expression profiling by array	GPL7191	GSE12511	Global Expression Profiling in Atopic Eczema Reveals Reciprocal Expression of Inflammatory and Lipid Genes	Plos One
Atopic Eczema	JMJD2C	Expression profiling by array	GPL7191	GSE12511	Global Expression Profiling in Atopic Eczema Reveals Reciprocal Expression of Inflammatory and Lipid Genes	Plos One
Atopic Eczema	WDR53	Expression profiling by array	GPL7191	GSE12511	Global Expression Profiling in Atopic Eczema Reveals Reciprocal Expression of Inflammatory and Lipid Genes	Plos One
Atopic Eczema	IMPA2	Expression profiling by array	GPL7191	GSE12511	Global Expression Profiling in Atopic Eczema Reveals Reciprocal Expression of Inflammatory and Lipid Genes	Plos One
Atopic Eczema	TTC3	Expression profiling by array	GPL7191	GSE12511	Global Expression Profiling in Atopic Eczema Reveals Reciprocal Expression of Inflammatory and Lipid Genes	Plos One
Atopic Eczema	SERPINA12	Expression profiling by array	GPL7191	GSE12511	Global Expression Profiling in Atopic Eczema Reveals Reciprocal Expression of Inflammatory and Lipid Genes	Plos One
Atopic Eczema	SNX4	Expression profiling by array	GPL7191	GSE12511	Global Expression Profiling in Atopic Eczema Reveals Reciprocal Expression of Inflammatory and Lipid Genes	Plos One
Atopic Eczema	KIAA0409	Expression profiling by array	GPL7191	GSE12511	Global Expression Profiling in Atopic Eczema Reveals Reciprocal Expression of Inflammatory and Lipid Genes	Plos One
Atopic Eczema	MRGPRF	Expression profiling by array	GPL7191	GSE12511	Global Expression Profiling in Atopic Eczema Reveals Reciprocal Expression of Inflammatory and Lipid Genes	Plos One
Atopic Eczema	UTP11L	Expression profiling by array	GPL7191	GSE12511	Global Expression Profiling in Atopic Eczema Reveals Reciprocal Expression of Inflammatory and Lipid Genes	Plos One
Atopic Eczema	ULK4	Expression profiling by array	GPL7191	GSE12511	Global Expression Profiling in Atopic Eczema Reveals Reciprocal Expression of Inflammatory and Lipid Genes	Plos One
Atopic Eczema	RPL10	Expression profiling by array	GPL7191	GSE12511	Global Expression Profiling in Atopic Eczema Reveals Reciprocal Expression of Inflammatory and Lipid Genes	Plos One
Atopic Eczema	C9ORF77	Expression profiling by array	GPL7191	GSE12511	Global Expression Profiling in Atopic Eczema Reveals Reciprocal Expression of Inflammatory and Lipid Genes	Plos One
Atopic Eczema	MS4A2	Expression profiling by array	GPL7191	GSE12511	Global Expression Profiling in Atopic Eczema Reveals Reciprocal Expression of Inflammatory and Lipid Genes	Plos One
Atopic Eczema	CST7	Expression profiling by array	GPL7191	GSE12511	Global Expression Profiling in Atopic Eczema Reveals Reciprocal Expression of Inflammatory and Lipid Genes	Plos One

Atopic Eczema	ANGPTL2	Expression profiling by array	GPL7191	GSE12511	Global Expression Profiling in Atopic Eczema Reveals Reciprocal Expression of Inflammatory and Lipid Genes	Plos One
Atopic Eczema	MRPL48	Expression profiling by array	GPL7191	GSE12511	Global Expression Profiling in Atopic Eczema Reveals Reciprocal Expression of Inflammatory and Lipid Genes	Plos One
Atopic Eczema	C21ORF33	Expression profiling by array	GPL7191	GSE12511	Global Expression Profiling in Atopic Eczema Reveals Reciprocal Expression of Inflammatory and Lipid Genes	Plos One
Atopic Eczema	SEC31L1	Expression profiling by array	GPL7191	GSE12511	Global Expression Profiling in Atopic Eczema Reveals Reciprocal Expression of Inflammatory and Lipid Genes	Plos One
Atopic Eczema	BMP2	Expression profiling by array	GPL7191	GSE12511	Global Expression Profiling in Atopic Eczema Reveals Reciprocal Expression of Inflammatory and Lipid Genes	Plos One
Atopic Eczema	ADORA1	Expression profiling by array	GPL7191	GSE12511	Global Expression Profiling in Atopic Eczema Reveals Reciprocal Expression of Inflammatory and Lipid Genes	Plos One
Atopic Eczema	AQP5	Expression profiling by array	GPL7191	GSE12511	Global Expression Profiling in Atopic Eczema Reveals Reciprocal Expression of Inflammatory and Lipid Genes	Plos One
Atopic Eczema	AFAP	Expression profiling by array	GPL7191	GSE12511	Global Expression Profiling in Atopic Eczema Reveals Reciprocal Expression of Inflammatory and Lipid Genes	Plos One
Atopic Eczema	SLC35D2	Expression profiling by array	GPL7191	GSE12511	Global Expression Profiling in Atopic Eczema Reveals Reciprocal Expression of Inflammatory and Lipid Genes	Plos One
Atopic Eczema	PHF17	Expression profiling by array	GPL7191	GSE12511	Global Expression Profiling in Atopic Eczema Reveals Reciprocal Expression of Inflammatory and Lipid Genes	Plos One
Atopic Eczema	LHFP	Expression profiling by array	GPL7191	GSE12511	Global Expression Profiling in Atopic Eczema Reveals Reciprocal Expression of Inflammatory and Lipid Genes	Plos One
Atopic Eczema	ALOX12	Expression profiling by array	GPL7191	GSE12511	Global Expression Profiling in Atopic Eczema Reveals Reciprocal Expression of Inflammatory and Lipid Genes	Plos One
Atopic Eczema	MLH3	Expression profiling by array	GPL7191	GSE12511	Global Expression Profiling in Atopic Eczema Reveals Reciprocal Expression of Inflammatory and Lipid Genes	Plos One
Atopic Eczema	GGPS1	Expression profiling by array	GPL7191	GSE12511	Global Expression Profiling in Atopic Eczema Reveals Reciprocal Expression of Inflammatory and Lipid Genes	Plos One
Atopic Eczema	FCHSD1	Expression profiling by array	GPL7191	GSE12511	Global Expression Profiling in Atopic Eczema Reveals Reciprocal Expression of Inflammatory and Lipid Genes	Plos One
Atopic Eczema	FLJ42709	Expression profiling by array	GPL7191	GSE12511	Global Expression Profiling in Atopic Eczema Reveals Reciprocal Expression of Inflammatory and Lipid Genes	Plos One
Atopic Eczema	LOC644845	Expression profiling by array	GPL7191	GSE12511	Global Expression Profiling in Atopic Eczema Reveals Reciprocal Expression of Inflammatory and Lipid Genes	Plos One
Atopic Eczema	TUBGCP2	Expression profiling by array	GPL7191	GSE12511	Global Expression Profiling in Atopic Eczema Reveals Reciprocal Expression of Inflammatory and Lipid Genes	Plos One
Atopic Eczema	INSR	Expression profiling by array	GPL7191	GSE12511	Global Expression Profiling in Atopic Eczema Reveals Reciprocal Expression of Inflammatory and Lipid Genes	Plos One
Atopic Eczema	PLAU	Expression profiling by array	GPL7191	GSE12511	Global Expression Profiling in Atopic Eczema Reveals Reciprocal Expression of Inflammatory and Lipid Genes	Plos One
Atopic Eczema	TUBB2B	Expression profiling by array	GPL7191	GSE12511	Global Expression Profiling in Atopic Eczema Reveals Reciprocal Expression of Inflammatory and Lipid Genes	Plos One
Atopic Eczema	NOL6	Expression profiling by array	GPL7191	GSE12511	Global Expression Profiling in Atopic Eczema Reveals Reciprocal Expression of Inflammatory and Lipid Genes	Plos One
Atopic Eczema	PPP4R1	Expression profiling by array	GPL7191	GSE12511	Global Expression Profiling in Atopic Eczema Reveals Reciprocal Expression of Inflammatory and Lipid Genes	Plos One
Atopic Eczema	KLF13	Expression profiling by array	GPL7191	GSE12511	Global Expression Profiling in Atopic Eczema Reveals Reciprocal Expression of Inflammatory and Lipid Genes	Plos One
Atopic Eczema	RALY	Expression profiling by array	GPL7191	GSE12511	Global Expression Profiling in Atopic Eczema Reveals Reciprocal Expression of Inflammatory and Lipid Genes	Plos One
Atopic Eczema	ZNF681	Expression profiling by array	GPL7191	GSE12511	Global Expression Profiling in Atopic Eczema Reveals Reciprocal Expression of Inflammatory and Lipid Genes	Plos One
Atopic Eczema	NUP85	Expression profiling by array	GPL7191	GSE12511	Global Expression Profiling in Atopic Eczema Reveals Reciprocal Expression of Inflammatory and Lipid Genes	Plos One
Atopic Eczema	EXOSC5	Expression profiling by array	GPL7191	GSE12511	Global Expression Profiling in Atopic Eczema Reveals Reciprocal Expression of Inflammatory and Lipid Genes	Plos One
Atopic Eczema	NOD3	Expression profiling by array	GPL7191	GSE12511	Global Expression Profiling in Atopic Eczema Reveals Reciprocal Expression of Inflammatory and Lipid Genes	Plos One
Atopic Eczema	C21ORF56	Expression profiling by array	GPL7191	GSE12511	Global Expression Profiling in Atopic Eczema Reveals Reciprocal Expression of Inflammatory and Lipid Genes	Plos One
Atopic Eczema	SLC7A1	Expression profiling by array	GPL7191	GSE12511	Global Expression Profiling in Atopic Eczema Reveals Reciprocal Expression of Inflammatory and Lipid Genes	Plos One
Atopic Eczema	PX19	Expression profiling by array	GPL7191	GSE12511	Global Expression Profiling in Atopic Eczema Reveals Reciprocal Expression of Inflammatory and Lipid Genes	Plos One
Atopic Eczema	C9ORF61	Expression profiling by array	GPL7191	GSE12511	Global Expression Profiling in Atopic Eczema Reveals Reciprocal Expression of Inflammatory and Lipid Genes	Plos One
Atopic Eczema	RNF113A	Expression profiling by array	GPL7191	GSE12511	Global Expression Profiling in Atopic Eczema Reveals Reciprocal Expression of Inflammatory and Lipid Genes	Plos One
Atopic Eczema	NT5E	Expression profiling by array	GPL7191	GSE12511	Global Expression Profiling in Atopic Eczema Reveals Reciprocal Expression of Inflammatory and Lipid Genes	Plos One
Atopic Eczema	ZNF432	Expression profiling by array	GPL7191	GSE12511	Global Expression Profiling in Atopic Eczema Reveals Reciprocal Expression of Inflammatory and Lipid Genes	Plos One
Atopic Eczema	SH3BP2	Expression profiling by array	GPL7191	GSE12511	Global Expression Profiling in Atopic Eczema Reveals Reciprocal Expression of Inflammatory and Lipid Genes	Plos One
Atopic Eczema	UBADC1	Expression profiling by array	GPL7191	GSE12511	Global Expression Profiling in Atopic Eczema Reveals Reciprocal Expression of Inflammatory and Lipid Genes	Plos One
Atopic Eczema	ATOX1	Expression profiling by array	GPL7191	GSE12511	Global Expression Profiling in Atopic Eczema Reveals Reciprocal Expression of Inflammatory and Lipid Genes	Plos One
Atopic Eczema	WNT10B	Expression profiling by array	GPL7191	GSE12511	Global Expression Profiling in Atopic Eczema Reveals Reciprocal Expression of Inflammatory and Lipid Genes	Plos One
Atopic Eczema	MCM5	Expression profiling by array	GPL7191	GSE12511	Global Expression Profiling in Atopic Eczema Reveals Reciprocal Expression of Inflammatory and Lipid Genes	Plos One
Atopic Eczema	DNAJB1	Expression profiling by array	GPL7191	GSE12511	Global Expression Profiling in Atopic Eczema Reveals Reciprocal Expression of Inflammatory and Lipid Genes	Plos One
Atopic Eczema	NUDT21	Expression profiling by array	GPL7191	GSE12511	Global Expression Profiling in Atopic Eczema Reveals Reciprocal Expression of Inflammatory and Lipid Genes	Plos One
Atopic Eczema	FAM105A	Expression profiling by array	GPL7191	GSE12511	Global Expression Profiling in Atopic Eczema Reveals Reciprocal Expression of Inflammatory and Lipid Genes	Plos One
Atopic Eczema	ABL1	Expression profiling by array	GPL7191	GSE12511	Global Expression Profiling in Atopic Eczema Reveals Reciprocal Expression of Inflammatory and Lipid Genes	Plos One
Atopic Eczema	INTS8	Expression profiling by array	GPL7191	GSE12511	Global Expression Profiling in Atopic Eczema Reveals Reciprocal Expression of Inflammatory and Lipid Genes	Plos One
Atopic Eczema	UBE2I	Expression profiling by array	GPL7191	GSE12511	Global Expression Profiling in Atopic Eczema Reveals Reciprocal Expression of Inflammatory and Lipid Genes	Plos One
Atopic Eczema	TNF	Expression profiling by array	GPL7191	GSE12511	Global Expression Profiling in Atopic Eczema Reveals Reciprocal Expression of Inflammatory and Lipid Genes	Plos One
Atopic Eczema	SGCB	Expression profiling by array	GPL7191	GSE12511	Global Expression Profiling in Atopic Eczema Reveals Reciprocal Expression of Inflammatory and Lipid Genes	Plos One
Atopic Eczema	CITED4	Expression profiling by array	GPL7191	GSE12511	Global Expression Profiling in Atopic Eczema Reveals Reciprocal Expression of Inflammatory and Lipid Genes	Plos One
Atopic Eczema	LOC642255	Expression profiling by array	GPL7191	GSE12511	Global Expression Profiling in Atopic Eczema Reveals Reciprocal Expression of Inflammatory and Lipid Genes	Plos One
Atopic Eczema	ACAA2	Expression profiling by array	GPL7191	GSE12511	Global Expression Profiling in Atopic Eczema Reveals Reciprocal Expression of Inflammatory and Lipid Genes	Plos One
Atopic Eczema	ZNF211	Expression profiling by array	GPL7191	GSE12511	Global Expression Profiling in Atopic Eczema Reveals Reciprocal Expression of Inflammatory and Lipid Genes	Plos One
Atopic Eczema	ACOT8	Expression profiling by array	GPL7191	GSE12511	Global Expression Profiling in Atopic Eczema Reveals Reciprocal Expression of Inflammatory and Lipid Genes	Plos One
Atopic Eczema	SESTD1	Expression profiling by array	GPL7191	GSE12511	Global Expression Profiling in Atopic Eczema Reveals Reciprocal Expression of Inflammatory and Lipid Genes	Plos One
Atopic Eczema	GPR124	Expression profiling by array	GPL7191	GSE12511	Global Expression Profiling in Atopic Eczema Reveals Reciprocal Expression of Inflammatory and Lipid Genes	Plos One
Atopic Eczema	RFX2	Expression profiling by array	GPL7191	GSE12511	Global Expression Profiling in Atopic Eczema Reveals Reciprocal Expression of Inflammatory and Lipid Genes	Plos One
Atopic Eczema	TSPAN8	Expression profiling by array	GPL7191	GSE12511	Global Expression Profiling in Atopic Eczema Reveals Reciprocal Expression of Inflammatory and Lipid Genes	Plos One
Atopic Eczema	ARFGEF2	Expression profiling by array	GPL7191	GSE12511	Global Expression Profiling in Atopic Eczema Reveals Reciprocal Expression of Inflammatory and Lipid Genes	Plos One
Atopic Eczema	SURF5	Expression profiling by array	GPL7191	GSE12511	Global Expression Profiling in Atopic Eczema Reveals Reciprocal Expression of Inflammatory and Lipid Genes	Plos One
Atopic Eczema	TMEM79	Expression profiling by array	GPL7191	GSE12511	Global Expression Profiling in Atopic Eczema Reveals Reciprocal Expression of Inflammatory and Lipid Genes	Plos One
Atopic Eczema	CTSZ	Expression profiling by array	GPL7191	GSE12511	Global Expression Profiling in Atopic Eczema Reveals Reciprocal Expression of Inflammatory and Lipid Genes	Plos One

Atopic Eczema	DSCR1L1	Expression profiling by array	GPL7191	GSE12511	Global Expression Profiling in Atopic Eczema Reveals Reciprocal Expression of Inflammatory and Lipid Genes	Plos One
Atopic Eczema	SLC16A4	Expression profiling by array	GPL7191	GSE12511	Global Expression Profiling in Atopic Eczema Reveals Reciprocal Expression of Inflammatory and Lipid Genes	Plos One
Atopic Eczema	CASP7	Expression profiling by array	GPL7191	GSE12511	Global Expression Profiling in Atopic Eczema Reveals Reciprocal Expression of Inflammatory and Lipid Genes	Plos One
Atopic Eczema	KCNJ10	Expression profiling by array	GPL7191	GSE12511	Global Expression Profiling in Atopic Eczema Reveals Reciprocal Expression of Inflammatory and Lipid Genes	Plos One
Atopic Eczema	FLJ10916	Expression profiling by array	GPL7191	GSE12511	Global Expression Profiling in Atopic Eczema Reveals Reciprocal Expression of Inflammatory and Lipid Genes	Plos One
Atopic Eczema	PPP1R16B	Expression profiling by array	GPL7191	GSE12511	Global Expression Profiling in Atopic Eczema Reveals Reciprocal Expression of Inflammatory and Lipid Genes	Plos One
Atopic Eczema	NPEPPS	Expression profiling by array	GPL7191	GSE12511	Global Expression Profiling in Atopic Eczema Reveals Reciprocal Expression of Inflammatory and Lipid Genes	Plos One
Atopic Eczema	CLIPR-59	Expression profiling by array	GPL7191	GSE12511	Global Expression Profiling in Atopic Eczema Reveals Reciprocal Expression of Inflammatory and Lipid Genes	Plos One
Atopic Eczema	MGC18216	Expression profiling by array	GPL7191	GSE12511	Global Expression Profiling in Atopic Eczema Reveals Reciprocal Expression of Inflammatory and Lipid Genes	Plos One
Atopic Eczema	XTP3TPA	Expression profiling by array	GPL7191	GSE12511	Global Expression Profiling in Atopic Eczema Reveals Reciprocal Expression of Inflammatory and Lipid Genes	Plos One
Atopic Eczema	STOML2	Expression profiling by array	GPL7191	GSE12511	Global Expression Profiling in Atopic Eczema Reveals Reciprocal Expression of Inflammatory and Lipid Genes	Plos One
Atopic Eczema	PDHX	Expression profiling by array	GPL7191	GSE12511	Global Expression Profiling in Atopic Eczema Reveals Reciprocal Expression of Inflammatory and Lipid Genes	Plos One
Atopic Eczema	THBS2	Expression profiling by array	GPL7191	GSE12511	Global Expression Profiling in Atopic Eczema Reveals Reciprocal Expression of Inflammatory and Lipid Genes	Plos One
Atopic Eczema	ITGAL	Expression profiling by array	GPL7191	GSE12511	Global Expression Profiling in Atopic Eczema Reveals Reciprocal Expression of Inflammatory and Lipid Genes	Plos One
Atopic Eczema	SULT1A1	Expression profiling by array	GPL7191	GSE12511	Global Expression Profiling in Atopic Eczema Reveals Reciprocal Expression of Inflammatory and Lipid Genes	Plos One
Atopic Eczema	RCC1	Expression profiling by array	GPL7191	GSE12511	Global Expression Profiling in Atopic Eczema Reveals Reciprocal Expression of Inflammatory and Lipid Genes	Plos One
Atopic Eczema	PPM1K	Expression profiling by array	GPL7191	GSE12511	Global Expression Profiling in Atopic Eczema Reveals Reciprocal Expression of Inflammatory and Lipid Genes	Plos One
Atopic Eczema	POLE4	Expression profiling by array	GPL7191	GSE12511	Global Expression Profiling in Atopic Eczema Reveals Reciprocal Expression of Inflammatory and Lipid Genes	Plos One
Atopic Eczema	MXD1	Expression profiling by array	GPL7191	GSE12511	Global Expression Profiling in Atopic Eczema Reveals Reciprocal Expression of Inflammatory and Lipid Genes	Plos One
Atopic Eczema	ABLIM1	Expression profiling by array	GPL7191	GSE12511	Global Expression Profiling in Atopic Eczema Reveals Reciprocal Expression of Inflammatory and Lipid Genes	Plos One
Atopic Eczema	UBE2Q1	Expression profiling by array	GPL7191	GSE12511	Global Expression Profiling in Atopic Eczema Reveals Reciprocal Expression of Inflammatory and Lipid Genes	Plos One
Atopic Eczema	RPP30	Expression profiling by array	GPL7191	GSE12511	Global Expression Profiling in Atopic Eczema Reveals Reciprocal Expression of Inflammatory and Lipid Genes	Plos One
Atopic Eczema	CDH3	Expression profiling by array	GPL7191	GSE12511	Global Expression Profiling in Atopic Eczema Reveals Reciprocal Expression of Inflammatory and Lipid Genes	Plos One
Atopic Eczema	PPT1	Expression profiling by array	GPL7191	GSE12511	Global Expression Profiling in Atopic Eczema Reveals Reciprocal Expression of Inflammatory and Lipid Genes	Plos One
Atopic Eczema	LTBR	Expression profiling by array	GPL7191	GSE12511	Global Expression Profiling in Atopic Eczema Reveals Reciprocal Expression of Inflammatory and Lipid Genes	Plos One
Atopic Eczema	SUPT3H	Expression profiling by array	GPL7191	GSE12511	Global Expression Profiling in Atopic Eczema Reveals Reciprocal Expression of Inflammatory and Lipid Genes	Plos One
Atopic Eczema	DCPS	Expression profiling by array	GPL7191	GSE12511	Global Expression Profiling in Atopic Eczema Reveals Reciprocal Expression of Inflammatory and Lipid Genes	Plos One
Atopic Eczema	LGR4	Expression profiling by array	GPL7191	GSE12511	Global Expression Profiling in Atopic Eczema Reveals Reciprocal Expression of Inflammatory and Lipid Genes	Plos One
Atopic Eczema	DEDD	Expression profiling by array	GPL7191	GSE12511	Global Expression Profiling in Atopic Eczema Reveals Reciprocal Expression of Inflammatory and Lipid Genes	Plos One
Atopic Eczema	TMEM49	Expression profiling by array	GPL7191	GSE12511	Global Expression Profiling in Atopic Eczema Reveals Reciprocal Expression of Inflammatory and Lipid Genes	Plos One
Atopic Eczema	MGC72075	Expression profiling by array	GPL7191	GSE12511	Global Expression Profiling in Atopic Eczema Reveals Reciprocal Expression of Inflammatory and Lipid Genes	Plos One
Atopic Eczema	L3MBTL4	Expression profiling by array	GPL7191	GSE12511	Global Expression Profiling in Atopic Eczema Reveals Reciprocal Expression of Inflammatory and Lipid Genes	Plos One
Atopic Eczema	TCTE3	Expression profiling by array	GPL7191	GSE12511	Global Expression Profiling in Atopic Eczema Reveals Reciprocal Expression of Inflammatory and Lipid Genes	Plos One
Atopic Eczema	KATNB1	Expression profiling by array	GPL7191	GSE12511	Global Expression Profiling in Atopic Eczema Reveals Reciprocal Expression of Inflammatory and Lipid Genes	Plos One
Atopic Eczema	AMY1A	Expression profiling by array	GPL7191	GSE12511	Global Expression Profiling in Atopic Eczema Reveals Reciprocal Expression of Inflammatory and Lipid Genes	Plos One
Atopic Eczema	EIF4ENIF1	Expression profiling by array	GPL7191	GSE12511	Global Expression Profiling in Atopic Eczema Reveals Reciprocal Expression of Inflammatory and Lipid Genes	Plos One
Atopic Eczema	TP53I11	Expression profiling by array	GPL7191	GSE12511	Global Expression Profiling in Atopic Eczema Reveals Reciprocal Expression of Inflammatory and Lipid Genes	Plos One
Atopic Eczema	AYTL2	Expression profiling by array	GPL7191	GSE12511	Global Expression Profiling in Atopic Eczema Reveals Reciprocal Expression of Inflammatory and Lipid Genes	Plos One
Atopic Eczema	LASS1	Expression profiling by array	GPL7191	GSE12511	Global Expression Profiling in Atopic Eczema Reveals Reciprocal Expression of Inflammatory and Lipid Genes	Plos One
Atopic Eczema	CYP2S1	Expression profiling by array	GPL7191	GSE12511	Global Expression Profiling in Atopic Eczema Reveals Reciprocal Expression of Inflammatory and Lipid Genes	Plos One
Atopic Eczema	RAB34	Expression profiling by array	GPL7191	GSE12511	Global Expression Profiling in Atopic Eczema Reveals Reciprocal Expression of Inflammatory and Lipid Genes	Plos One
Atopic Eczema	EIF1	Expression profiling by array	GPL7191	GSE12511	Global Expression Profiling in Atopic Eczema Reveals Reciprocal Expression of Inflammatory and Lipid Genes	Plos One
Atopic Eczema	PDE9A	Expression profiling by array	GPL7191	GSE12511	Global Expression Profiling in Atopic Eczema Reveals Reciprocal Expression of Inflammatory and Lipid Genes	Plos One
Atopic Eczema	SPRR2A	Expression profiling by array	GPL7191	GSE12511	Global Expression Profiling in Atopic Eczema Reveals Reciprocal Expression of Inflammatory and Lipid Genes	Plos One
Atopic Eczema	SPRYD3	Expression profiling by array	GPL7191	GSE12511	Global Expression Profiling in Atopic Eczema Reveals Reciprocal Expression of Inflammatory and Lipid Genes	Plos One
Atopic Eczema	GBF1	Expression profiling by array	GPL7191	GSE12511	Global Expression Profiling in Atopic Eczema Reveals Reciprocal Expression of Inflammatory and Lipid Genes	Plos One
Atopic Eczema	FAM109A	Expression profiling by array	GPL7191	GSE12511	Global Expression Profiling in Atopic Eczema Reveals Reciprocal Expression of Inflammatory and Lipid Genes	Plos One
Atopic Eczema	ADCK1	Expression profiling by array	GPL7191	GSE12511	Global Expression Profiling in Atopic Eczema Reveals Reciprocal Expression of Inflammatory and Lipid Genes	Plos One
Atopic Eczema	ICMT	Expression profiling by array	GPL7191	GSE12511	Global Expression Profiling in Atopic Eczema Reveals Reciprocal Expression of Inflammatory and Lipid Genes	Plos One
Atopic Eczema	C1ORF2	Expression profiling by array	GPL7191	GSE12511	Global Expression Profiling in Atopic Eczema Reveals Reciprocal Expression of Inflammatory and Lipid Genes	Plos One
Atopic Eczema	CASD1	Expression profiling by array	GPL7191	GSE12511	Global Expression Profiling in Atopic Eczema Reveals Reciprocal Expression of Inflammatory and Lipid Genes	Plos One
Atopic Eczema	TLE3	Expression profiling by array	GPL7191	GSE12511	Global Expression Profiling in Atopic Eczema Reveals Reciprocal Expression of Inflammatory and Lipid Genes	Plos One
Atopic Eczema	TCF4	Expression profiling by array	GPL7191	GSE12511	Global Expression Profiling in Atopic Eczema Reveals Reciprocal Expression of Inflammatory and Lipid Genes	Plos One
Atopic Eczema	ARF1	Expression profiling by array	GPL7191	GSE12511	Global Expression Profiling in Atopic Eczema Reveals Reciprocal Expression of Inflammatory and Lipid Genes	Plos One
Atopic Eczema	DHRS7	Expression profiling by array	GPL7191	GSE12511	Global Expression Profiling in Atopic Eczema Reveals Reciprocal Expression of Inflammatory and Lipid Genes	Plos One
Atopic Eczema	PLCL1	Expression profiling by array	GPL7191	GSE12511	Global Expression Profiling in Atopic Eczema Reveals Reciprocal Expression of Inflammatory and Lipid Genes	Plos One
Atopic Eczema	SETD4	Expression profiling by array	GPL7191	GSE12511	Global Expression Profiling in Atopic Eczema Reveals Reciprocal Expression of Inflammatory and Lipid Genes	Plos One
Atopic Eczema	NDEL1	Expression profiling by array	GPL7191	GSE12511	Global Expression Profiling in Atopic Eczema Reveals Reciprocal Expression of Inflammatory and Lipid Genes	Plos One
Atopic Eczema	NECAP1	Expression profiling by array	GPL7191	GSE12511	Global Expression Profiling in Atopic Eczema Reveals Reciprocal Expression of Inflammatory and Lipid Genes	Plos One
Atopic Eczema	IDUA	Expression profiling by array	GPL7191	GSE12511	Global Expression Profiling in Atopic Eczema Reveals Reciprocal Expression of Inflammatory and Lipid Genes	Plos One
Atopic Eczema	GTDC1	Expression profiling by array	GPL7191	GSE12511	Global Expression Profiling in Atopic Eczema Reveals Reciprocal Expression of Inflammatory and Lipid Genes	Plos One
Atopic Eczema	C17ORF81	Expression profiling by array	GPL7191	GSE12511	Global Expression Profiling in Atopic Eczema Reveals Reciprocal Expression of Inflammatory and Lipid Genes	Plos One

Atopic Eczema	LATS2	Expression profiling by array	GPL7191	GSE12511	Global Expression Profiling in Atopic Eczema Reveals Reciprocal Expression of Inflammatory and Lipid Genes	Plos One
Atopic Eczema	OSBP	Expression profiling by array	GPL7191	GSE12511	Global Expression Profiling in Atopic Eczema Reveals Reciprocal Expression of Inflammatory and Lipid Genes	Plos One
Atopic Eczema	C10ORF4	Expression profiling by array	GPL7191	GSE12511	Global Expression Profiling in Atopic Eczema Reveals Reciprocal Expression of Inflammatory and Lipid Genes	Plos One
Atopic Eczema	ALS2CR12	Expression profiling by array	GPL7191	GSE12511	Global Expression Profiling in Atopic Eczema Reveals Reciprocal Expression of Inflammatory and Lipid Genes	Plos One
Atopic Eczema	PHCA	Expression profiling by array	GPL7191	GSE12511	Global Expression Profiling in Atopic Eczema Reveals Reciprocal Expression of Inflammatory and Lipid Genes	Plos One
Atopic Eczema	RPS24	Expression profiling by array	GPL7191	GSE12511	Global Expression Profiling in Atopic Eczema Reveals Reciprocal Expression of Inflammatory and Lipid Genes	Plos One
Atopic Eczema	SYK	Expression profiling by array	GPL7191	GSE12511	Global Expression Profiling in Atopic Eczema Reveals Reciprocal Expression of Inflammatory and Lipid Genes	Plos One
Atopic Eczema	TPSG1	Expression profiling by array	GPL7191	GSE12511	Global Expression Profiling in Atopic Eczema Reveals Reciprocal Expression of Inflammatory and Lipid Genes	Plos One
Atopic Eczema	RSN	Expression profiling by array	GPL7191	GSE12511	Global Expression Profiling in Atopic Eczema Reveals Reciprocal Expression of Inflammatory and Lipid Genes	Plos One
Atopic Eczema	MYO7A	Expression profiling by array	GPL7191	GSE12511	Global Expression Profiling in Atopic Eczema Reveals Reciprocal Expression of Inflammatory and Lipid Genes	Plos One
Atopic Eczema	TSPAN32	Expression profiling by array	GPL7191	GSE12511	Global Expression Profiling in Atopic Eczema Reveals Reciprocal Expression of Inflammatory and Lipid Genes	Plos One
Atopic Eczema	COL4A1	Expression profiling by array	GPL7191	GSE12511	Global Expression Profiling in Atopic Eczema Reveals Reciprocal Expression of Inflammatory and Lipid Genes	Plos One
Atopic Eczema	C1ORF164	Expression profiling by array	GPL7191	GSE12511	Global Expression Profiling in Atopic Eczema Reveals Reciprocal Expression of Inflammatory and Lipid Genes	Plos One
Atopic Eczema	C22ORF13	Expression profiling by array	GPL7191	GSE12511	Global Expression Profiling in Atopic Eczema Reveals Reciprocal Expression of Inflammatory and Lipid Genes	Plos One
Atopic Eczema	ADPRH	Expression profiling by array	GPL7191	GSE12511	Global Expression Profiling in Atopic Eczema Reveals Reciprocal Expression of Inflammatory and Lipid Genes	Plos One
Atopic Eczema	GRHPR	Expression profiling by array	GPL7191	GSE12511	Global Expression Profiling in Atopic Eczema Reveals Reciprocal Expression of Inflammatory and Lipid Genes	Plos One
Atopic Eczema	CNTN1	Expression profiling by array	GPL7191	GSE12511	Global Expression Profiling in Atopic Eczema Reveals Reciprocal Expression of Inflammatory and Lipid Genes	Plos One
Atopic Eczema	SUGT1	Expression profiling by array	GPL7191	GSE12511	Global Expression Profiling in Atopic Eczema Reveals Reciprocal Expression of Inflammatory and Lipid Genes	Plos One
Atopic Eczema	YARS	Expression profiling by array	GPL7191	GSE12511	Global Expression Profiling in Atopic Eczema Reveals Reciprocal Expression of Inflammatory and Lipid Genes	Plos One
Atopic Eczema	CLCNKA	Expression profiling by array	GPL7191	GSE12511	Global Expression Profiling in Atopic Eczema Reveals Reciprocal Expression of Inflammatory and Lipid Genes	Plos One
Atopic Eczema	KENAE	Expression profiling by array	GPL7191	GSE12511	Global Expression Profiling in Atopic Eczema Reveals Reciprocal Expression of Inflammatory and Lipid Genes	Plos One
Atopic Eczema	RARRES1	Expression profiling by array	GPL7191	GSE12511	Global Expression Profiling in Atopic Eczema Reveals Reciprocal Expression of Inflammatory and Lipid Genes	Plos One
Atopic Eczema	C6ORF71	Expression profiling by array	GPL7191	GSE12511	Global Expression Profiling in Atopic Eczema Reveals Reciprocal Expression of Inflammatory and Lipid Genes	Plos One
Atopic Eczema	APCDD1	Expression profiling by array	GPL7191	GSE12511	Global Expression Profiling in Atopic Eczema Reveals Reciprocal Expression of Inflammatory and Lipid Genes	Plos One
Atopic Eczema	PPAP2A	Expression profiling by array	GPL7191	GSE12511	Global Expression Profiling in Atopic Eczema Reveals Reciprocal Expression of Inflammatory and Lipid Genes	Plos One
Atopic Eczema	CD3E	Expression profiling by array	GPL7191	GSE12511	Global Expression Profiling in Atopic Eczema Reveals Reciprocal Expression of Inflammatory and Lipid Genes	Plos One
Atopic Eczema	NAG	Expression profiling by array	GPL7191	GSE12511	Global Expression Profiling in Atopic Eczema Reveals Reciprocal Expression of Inflammatory and Lipid Genes	Plos One
Atopic Eczema	CD4	Expression profiling by array	GPL7191	GSE12511	Global Expression Profiling in Atopic Eczema Reveals Reciprocal Expression of Inflammatory and Lipid Genes	Plos One
Atopic Eczema	C9ORF125	Expression profiling by array	GPL7191	GSE12511	Global Expression Profiling in Atopic Eczema Reveals Reciprocal Expression of Inflammatory and Lipid Genes	Plos One
Atopic Eczema	TRIM8	Expression profiling by array	GPL7191	GSE12511	Global Expression Profiling in Atopic Eczema Reveals Reciprocal Expression of Inflammatory and Lipid Genes	Plos One
Atopic Eczema	ABCA5	Expression profiling by array	GPL7191	GSE12511	Global Expression Profiling in Atopic Eczema Reveals Reciprocal Expression of Inflammatory and Lipid Genes	Plos One
Atopic Eczema	STEAP4	Expression profiling by array	GPL7191	GSE12511	Global Expression Profiling in Atopic Eczema Reveals Reciprocal Expression of Inflammatory and Lipid Genes	Plos One
Atopic Eczema	C14ORF147	Expression profiling by array	GPL7191	GSE12511	Global Expression Profiling in Atopic Eczema Reveals Reciprocal Expression of Inflammatory and Lipid Genes	Plos One
Atopic Eczema	IL1R1	Expression profiling by array	GPL7191	GSE12511	Global Expression Profiling in Atopic Eczema Reveals Reciprocal Expression of Inflammatory and Lipid Genes	Plos One
Atopic Eczema	BAX	Expression profiling by array	GPL7191	GSE12511	Global Expression Profiling in Atopic Eczema Reveals Reciprocal Expression of Inflammatory and Lipid Genes	Plos One
Atopic Eczema	ZNF207	Expression profiling by array	GPL7191	GSE12511	Global Expression Profiling in Atopic Eczema Reveals Reciprocal Expression of Inflammatory and Lipid Genes	Plos One
Atopic Eczema	PALMD	Expression profiling by array	GPL7191	GSE12511	Global Expression Profiling in Atopic Eczema Reveals Reciprocal Expression of Inflammatory and Lipid Genes	Plos One
Atopic Eczema	UBE2V1	Expression profiling by array	GPL7191	GSE12511	Global Expression Profiling in Atopic Eczema Reveals Reciprocal Expression of Inflammatory and Lipid Genes	Plos One
Atopic Eczema	ERN1	Expression profiling by array	GPL7191	GSE12511	Global Expression Profiling in Atopic Eczema Reveals Reciprocal Expression of Inflammatory and Lipid Genes	Plos One
Atopic Eczema	C11ORF59	Expression profiling by array	GPL7191	GSE12511	Global Expression Profiling in Atopic Eczema Reveals Reciprocal Expression of Inflammatory and Lipid Genes	Plos One
Atopic Eczema	PTPN18	Expression profiling by array	GPL7191	GSE12511	Global Expression Profiling in Atopic Eczema Reveals Reciprocal Expression of Inflammatory and Lipid Genes	Plos One
Atopic Eczema	MICAL-L1	Expression profiling by array	GPL7191	GSE12511	Global Expression Profiling in Atopic Eczema Reveals Reciprocal Expression of Inflammatory and Lipid Genes	Plos One
Atopic Eczema	NELL2	Expression profiling by array	GPL7191	GSE12511	Global Expression Profiling in Atopic Eczema Reveals Reciprocal Expression of Inflammatory and Lipid Genes	Plos One
Atopic Eczema	CHP	Expression profiling by array	GPL7191	GSE12511	Global Expression Profiling in Atopic Eczema Reveals Reciprocal Expression of Inflammatory and Lipid Genes	Plos One
Atopic Eczema	UNC45A	Expression profiling by array	GPL7191	GSE12511	Global Expression Profiling in Atopic Eczema Reveals Reciprocal Expression of Inflammatory and Lipid Genes	Plos One
Atopic Eczema	DST	Expression profiling by array	GPL7191	GSE12511	Global Expression Profiling in Atopic Eczema Reveals Reciprocal Expression of Inflammatory and Lipid Genes	Plos One
Atopic Eczema	RPL21	Expression profiling by array	GPL7191	GSE12511	Global Expression Profiling in Atopic Eczema Reveals Reciprocal Expression of Inflammatory and Lipid Genes	Plos One
Atopic Eczema	CAPZA3	Expression profiling by array	GPL7191	GSE12511	Global Expression Profiling in Atopic Eczema Reveals Reciprocal Expression of Inflammatory and Lipid Genes	Plos One
Atopic Eczema	SCP2	Expression profiling by array	GPL7191	GSE12511	Global Expression Profiling in Atopic Eczema Reveals Reciprocal Expression of Inflammatory and Lipid Genes	Plos One
Atopic Eczema	CTSW	Expression profiling by array	GPL7191	GSE12511	Global Expression Profiling in Atopic Eczema Reveals Reciprocal Expression of Inflammatory and Lipid Genes	Plos One
Atopic Eczema	IL32	Expression profiling by array	GPL7191	GSE12511	Global Expression Profiling in Atopic Eczema Reveals Reciprocal Expression of Inflammatory and Lipid Genes	Plos One
Atopic Eczema	SERPINE1	Expression profiling by array	GPL7191	GSE12511	Global Expression Profiling in Atopic Eczema Reveals Reciprocal Expression of Inflammatory and Lipid Genes	Plos One
Atopic Eczema	FUT8	Expression profiling by array	GPL7191	GSE12511	Global Expression Profiling in Atopic Eczema Reveals Reciprocal Expression of Inflammatory and Lipid Genes	Plos One
Atopic Eczema	PDCD6IP	Expression profiling by array	GPL7191	GSE12511	Global Expression Profiling in Atopic Eczema Reveals Reciprocal Expression of Inflammatory and Lipid Genes	Plos One
Atopic Eczema	VCAM1	Expression profiling by array	GPL7191	GSE12511	Global Expression Profiling in Atopic Eczema Reveals Reciprocal Expression of Inflammatory and Lipid Genes	Plos One
Atopic Eczema	NFATC2IP	Expression profiling by array	GPL7191	GSE12511	Global Expression Profiling in Atopic Eczema Reveals Reciprocal Expression of Inflammatory and Lipid Genes	Plos One
Atopic Eczema	NASP	Expression profiling by array	GPL7191	GSE12511	Global Expression Profiling in Atopic Eczema Reveals Reciprocal Expression of Inflammatory and Lipid Genes	Plos One
Atopic Eczema	ABCA12	Expression profiling by array	GPL7191	GSE12511	Global Expression Profiling in Atopic Eczema Reveals Reciprocal Expression of Inflammatory and Lipid Genes	Plos One
Atopic Eczema	C17ORF75	Expression profiling by array	GPL7191	GSE12511	Global Expression Profiling in Atopic Eczema Reveals Reciprocal Expression of Inflammatory and Lipid Genes	Plos One
Atopic Eczema	MYSM1	Expression profiling by array	GPL7191	GSE12511	Global Expression Profiling in Atopic Eczema Reveals Reciprocal Expression of Inflammatory and Lipid Genes	Plos One
Atopic Eczema	NALP9	Expression profiling by array	GPL7191	GSE12511	Global Expression Profiling in Atopic Eczema Reveals Reciprocal Expression of Inflammatory and Lipid Genes	Plos One
Atopic Eczema	DDHD1	Expression profiling by array	GPL7191	GSE12511	Global Expression Profiling in Atopic Eczema Reveals Reciprocal Expression of Inflammatory and Lipid Genes	Plos One

Atopic Eczema	ARID5B	Expression profiling by array	GPL7191	GSE12511	Global Expression Profiling in Atopic Eczema Reveals Reciprocal Expression of Inflammatory and Lipid Genes	Plos One
Atopic Eczema	TOLLIP	Expression profiling by array	GPL7191	GSE12511	Global Expression Profiling in Atopic Eczema Reveals Reciprocal Expression of Inflammatory and Lipid Genes	Plos One
Atopic Eczema	SRD5A2L	Expression profiling by array	GPL7191	GSE12511	Global Expression Profiling in Atopic Eczema Reveals Reciprocal Expression of Inflammatory and Lipid Genes	Plos One
Atopic Eczema	RAB25	Expression profiling by array	GPL7191	GSE12511	Global Expression Profiling in Atopic Eczema Reveals Reciprocal Expression of Inflammatory and Lipid Genes	Plos One
Atopic Eczema	SUSD4	Expression profiling by array	GPL7191	GSE12511	Global Expression Profiling in Atopic Eczema Reveals Reciprocal Expression of Inflammatory and Lipid Genes	Plos One
Atopic Eczema	CCDC108	Expression profiling by array	GPL7191	GSE12511	Global Expression Profiling in Atopic Eczema Reveals Reciprocal Expression of Inflammatory and Lipid Genes	Plos One
Atopic Eczema	ABCB9	Expression profiling by array	GPL7191	GSE12511	Global Expression Profiling in Atopic Eczema Reveals Reciprocal Expression of Inflammatory and Lipid Genes	Plos One
Atopic Eczema	KRT75	Expression profiling by array	GPL7191	GSE12511	Global Expression Profiling in Atopic Eczema Reveals Reciprocal Expression of Inflammatory and Lipid Genes	Plos One
Atopic Eczema	PECI	Expression profiling by array	GPL7191	GSE12511	Global Expression Profiling in Atopic Eczema Reveals Reciprocal Expression of Inflammatory and Lipid Genes	Plos One
Atopic Eczema	CPXM	Expression profiling by array	GPL7191	GSE12511	Global Expression Profiling in Atopic Eczema Reveals Reciprocal Expression of Inflammatory and Lipid Genes	Plos One
Atopic Eczema	NINJ2	Expression profiling by array	GPL7191	GSE12511	Global Expression Profiling in Atopic Eczema Reveals Reciprocal Expression of Inflammatory and Lipid Genes	Plos One
Atopic Eczema	RAPGEF1	Expression profiling by array	GPL7191	GSE12511	Global Expression Profiling in Atopic Eczema Reveals Reciprocal Expression of Inflammatory and Lipid Genes	Plos One
Atopic Eczema	FSTL5	Expression profiling by array	GPL7191	GSE12511	Global Expression Profiling in Atopic Eczema Reveals Reciprocal Expression of Inflammatory and Lipid Genes	Plos One
Atopic Eczema	TRIM46	Expression profiling by array	GPL7191	GSE12511	Global Expression Profiling in Atopic Eczema Reveals Reciprocal Expression of Inflammatory and Lipid Genes	Plos One
Atopic Eczema	SMAD1	Expression profiling by array	GPL7191	GSE12511	Global Expression Profiling in Atopic Eczema Reveals Reciprocal Expression of Inflammatory and Lipid Genes	Plos One
Atopic Eczema	BAT5	Expression profiling by array	GPL7191	GSE12511	Global Expression Profiling in Atopic Eczema Reveals Reciprocal Expression of Inflammatory and Lipid Genes	Plos One
Atopic Eczema	BCAS1	Expression profiling by array	GPL7191	GSE12511	Global Expression Profiling in Atopic Eczema Reveals Reciprocal Expression of Inflammatory and Lipid Genes	Plos One
Atopic Eczema	CYBASC3	Expression profiling by array	GPL7191	GSE12511	Global Expression Profiling in Atopic Eczema Reveals Reciprocal Expression of Inflammatory and Lipid Genes	Plos One
Atopic Eczema	KCTD17	Expression profiling by array	GPL7191	GSE12511	Global Expression Profiling in Atopic Eczema Reveals Reciprocal Expression of Inflammatory and Lipid Genes	Plos One
Atopic Eczema	KCNMB4	Expression profiling by array	GPL7191	GSE12511	Global Expression Profiling in Atopic Eczema Reveals Reciprocal Expression of Inflammatory and Lipid Genes	Plos One
Atopic Eczema	PRPF39	Expression profiling by array	GPL7191	GSE12511	Global Expression Profiling in Atopic Eczema Reveals Reciprocal Expression of Inflammatory and Lipid Genes	Plos One
Atopic Eczema	MGC2749	Expression profiling by array	GPL7191	GSE12511	Global Expression Profiling in Atopic Eczema Reveals Reciprocal Expression of Inflammatory and Lipid Genes	Plos One
Atopic Eczema	ALCAM	Expression profiling by array	GPL7191	GSE12511	Global Expression Profiling in Atopic Eczema Reveals Reciprocal Expression of Inflammatory and Lipid Genes	Plos One
Atopic Eczema	ZNRF3	Expression profiling by array	GPL7191	GSE12511	Global Expression Profiling in Atopic Eczema Reveals Reciprocal Expression of Inflammatory and Lipid Genes	Plos One
Atopic Eczema	SPTBN5	Expression profiling by array	GPL7191	GSE12511	Global Expression Profiling in Atopic Eczema Reveals Reciprocal Expression of Inflammatory and Lipid Genes	Plos One
Atopic Eczema	NDE1	Expression profiling by array	GPL7191	GSE12511	Global Expression Profiling in Atopic Eczema Reveals Reciprocal Expression of Inflammatory and Lipid Genes	Plos One
Atopic Eczema	GALNT3	Expression profiling by array	GPL7191	GSE12511	Global Expression Profiling in Atopic Eczema Reveals Reciprocal Expression of Inflammatory and Lipid Genes	Plos One
Atopic Eczema	ATP11B	Expression profiling by array	GPL7191	GSE12511	Global Expression Profiling in Atopic Eczema Reveals Reciprocal Expression of Inflammatory and Lipid Genes	Plos One
Atopic Eczema	SMPD1	Expression profiling by array	GPL7191	GSE12511	Global Expression Profiling in Atopic Eczema Reveals Reciprocal Expression of Inflammatory and Lipid Genes	Plos One
Atopic Eczema	ROS1	Expression profiling by array	GPL7191	GSE12511	Global Expression Profiling in Atopic Eczema Reveals Reciprocal Expression of Inflammatory and Lipid Genes	Plos One
Atopic Eczema	E2F4	Expression profiling by array	GPL7191	GSE12511	Global Expression Profiling in Atopic Eczema Reveals Reciprocal Expression of Inflammatory and Lipid Genes	Plos One
Atopic Eczema	STK40	Expression profiling by array	GPL7191	GSE12511	Global Expression Profiling in Atopic Eczema Reveals Reciprocal Expression of Inflammatory and Lipid Genes	Plos One
Atopic Eczema	PVRL3	Expression profiling by array	GPL7191	GSE12511	Global Expression Profiling in Atopic Eczema Reveals Reciprocal Expression of Inflammatory and Lipid Genes	Plos One
Atopic Eczema	TTC17	Expression profiling by array	GPL7191	GSE12511	Global Expression Profiling in Atopic Eczema Reveals Reciprocal Expression of Inflammatory and Lipid Genes	Plos One
Atopic Eczema	SLC41A1	Expression profiling by array	GPL7191	GSE12511	Global Expression Profiling in Atopic Eczema Reveals Reciprocal Expression of Inflammatory and Lipid Genes	Plos One
Atopic Eczema	KIAA0831	Expression profiling by array	GPL7191	GSE12511	Global Expression Profiling in Atopic Eczema Reveals Reciprocal Expression of Inflammatory and Lipid Genes	Plos One
Atopic Eczema	FBXO31	Expression profiling by array	GPL7191	GSE12511	Global Expression Profiling in Atopic Eczema Reveals Reciprocal Expression of Inflammatory and Lipid Genes	Plos One
Atopic Eczema	ZNF659	Expression profiling by array	GPL7191	GSE12511	Global Expression Profiling in Atopic Eczema Reveals Reciprocal Expression of Inflammatory and Lipid Genes	Plos One
Atopic Eczema	MRPL11	Expression profiling by array	GPL7191	GSE12511	Global Expression Profiling in Atopic Eczema Reveals Reciprocal Expression of Inflammatory and Lipid Genes	Plos One
Atopic Eczema	WISP1	Expression profiling by array	GPL7191	GSE12511	Global Expression Profiling in Atopic Eczema Reveals Reciprocal Expression of Inflammatory and Lipid Genes	Plos One
Atopic Eczema	ARL3	Expression profiling by array	GPL7191	GSE12511	Global Expression Profiling in Atopic Eczema Reveals Reciprocal Expression of Inflammatory and Lipid Genes	Plos One
Atopic Eczema	B3GALT4	Expression profiling by array	GPL7191	GSE12511	Global Expression Profiling in Atopic Eczema Reveals Reciprocal Expression of Inflammatory and Lipid Genes	Plos One
Atopic Eczema	SRG	Expression profiling by array	GPL7191	GSE12511	Global Expression Profiling in Atopic Eczema Reveals Reciprocal Expression of Inflammatory and Lipid Genes	Plos One
Atopic Eczema	CTSB	Expression profiling by array	GPL7191	GSE12511	Global Expression Profiling in Atopic Eczema Reveals Reciprocal Expression of Inflammatory and Lipid Genes	Plos One
Atopic Eczema	C17ORF28	Expression profiling by array	GPL7191	GSE12511	Global Expression Profiling in Atopic Eczema Reveals Reciprocal Expression of Inflammatory and Lipid Genes	Plos One
Atopic Eczema	SARS2	Expression profiling by array	GPL7191	GSE12511	Global Expression Profiling in Atopic Eczema Reveals Reciprocal Expression of Inflammatory and Lipid Genes	Plos One
Atopic Eczema	RELA	Expression profiling by array	GPL7191	GSE12511	Global Expression Profiling in Atopic Eczema Reveals Reciprocal Expression of Inflammatory and Lipid Genes	Plos One
Atopic Eczema	LZTS2	Expression profiling by array	GPL7191	GSE12511	Global Expression Profiling in Atopic Eczema Reveals Reciprocal Expression of Inflammatory and Lipid Genes	Plos One
Atopic Eczema	CARHSP1	Expression profiling by array	GPL7191	GSE12511	Global Expression Profiling in Atopic Eczema Reveals Reciprocal Expression of Inflammatory and Lipid Genes	Plos One
Atopic Eczema	PDAP1	Expression profiling by array	GPL7191	GSE12511	Global Expression Profiling in Atopic Eczema Reveals Reciprocal Expression of Inflammatory and Lipid Genes	Plos One
Atopic Eczema	PLCB1	Expression profiling by array	GPL7191	GSE12511	Global Expression Profiling in Atopic Eczema Reveals Reciprocal Expression of Inflammatory and Lipid Genes	Plos One
Atopic Eczema	C9ORF86	Expression profiling by array	GPL7191	GSE12511	Global Expression Profiling in Atopic Eczema Reveals Reciprocal Expression of Inflammatory and Lipid Genes	Plos One
Atopic Eczema	PGBD3	Expression profiling by array	GPL7191	GSE12511	Global Expression Profiling in Atopic Eczema Reveals Reciprocal Expression of Inflammatory and Lipid Genes	Plos One
Atopic Eczema	ACAT2	Expression profiling by array	GPL7191	GSE12511	Global Expression Profiling in Atopic Eczema Reveals Reciprocal Expression of Inflammatory and Lipid Genes	Plos One
Atopic Eczema	NIT2	Expression profiling by array	GPL7191	GSE12511	Global Expression Profiling in Atopic Eczema Reveals Reciprocal Expression of Inflammatory and Lipid Genes	Plos One
Atopic Eczema	BCKDK	Expression profiling by array	GPL7191	GSE12511	Global Expression Profiling in Atopic Eczema Reveals Reciprocal Expression of Inflammatory and Lipid Genes	Plos One
Atopic Eczema	KLK11	Expression profiling by array	GPL7191	GSE12511	Global Expression Profiling in Atopic Eczema Reveals Reciprocal Expression of Inflammatory and Lipid Genes	Plos One
Atopic Eczema	ZAK	Expression profiling by array	GPL7191	GSE12511	Global Expression Profiling in Atopic Eczema Reveals Reciprocal Expression of Inflammatory and Lipid Genes	Plos One
Atopic Eczema	TRSPAP1	Expression profiling by array	GPL7191	GSE12511	Global Expression Profiling in Atopic Eczema Reveals Reciprocal Expression of Inflammatory and Lipid Genes	Plos One
Atopic Eczema	VDP	Expression profiling by array	GPL7191	GSE12511	Global Expression Profiling in Atopic Eczema Reveals Reciprocal Expression of Inflammatory and Lipid Genes	Plos One
Atopic Eczema	DNASE1L3	Expression profiling by array	GPL7191	GSE12511	Global Expression Profiling in Atopic Eczema Reveals Reciprocal Expression of Inflammatory and Lipid Genes	Plos One
Atopic Eczema	SP3	Expression profiling by array	GPL7191	GSE12511	Global Expression Profiling in Atopic Eczema Reveals Reciprocal Expression of Inflammatory and Lipid Genes	Plos One

Atopic Eczema	ZNF213	Expression profiling by array	GPL7191	GSE12511	Global Expression Profiling in Atopic Eczema Reveals Reciprocal Expression of Inflammatory and Lipid Genes	Plos One
Atopic Eczema	LDHD	Expression profiling by array	GPL7191	GSE12511	Global Expression Profiling in Atopic Eczema Reveals Reciprocal Expression of Inflammatory and Lipid Genes	Plos One
Atopic Eczema	GNPDA1	Expression profiling by array	GPL7191	GSE12511	Global Expression Profiling in Atopic Eczema Reveals Reciprocal Expression of Inflammatory and Lipid Genes	Plos One
Atopic Eczema	GIT1	Expression profiling by array	GPL7191	GSE12511	Global Expression Profiling in Atopic Eczema Reveals Reciprocal Expression of Inflammatory and Lipid Genes	Plos One
Atopic Eczema	KRAS	Expression profiling by array	GPL7191	GSE12511	Global Expression Profiling in Atopic Eczema Reveals Reciprocal Expression of Inflammatory and Lipid Genes	Plos One
Atopic Eczema	POLR3D	Expression profiling by array	GPL7191	GSE12511	Global Expression Profiling in Atopic Eczema Reveals Reciprocal Expression of Inflammatory and Lipid Genes	Plos One
Atopic Eczema	LOC642412	Expression profiling by array	GPL7191	GSE12511	Global Expression Profiling in Atopic Eczema Reveals Reciprocal Expression of Inflammatory and Lipid Genes	Plos One
Atopic Eczema	COPZ2	Expression profiling by array	GPL7191	GSE12511	Global Expression Profiling in Atopic Eczema Reveals Reciprocal Expression of Inflammatory and Lipid Genes	Plos One
Atopic Eczema	PMM2	Expression profiling by array	GPL7191	GSE12511	Global Expression Profiling in Atopic Eczema Reveals Reciprocal Expression of Inflammatory and Lipid Genes	Plos One
Atopic Eczema	NEK9	Expression profiling by array	GPL7191	GSE12511	Global Expression Profiling in Atopic Eczema Reveals Reciprocal Expression of Inflammatory and Lipid Genes	Plos One
Atopic Eczema	LONRF2	Expression profiling by array	GPL7191	GSE12511	Global Expression Profiling in Atopic Eczema Reveals Reciprocal Expression of Inflammatory and Lipid Genes	Plos One
Atopic Eczema	C1ORF95	Expression profiling by array	GPL7191	GSE12511	Global Expression Profiling in Atopic Eczema Reveals Reciprocal Expression of Inflammatory and Lipid Genes	Plos One
Atopic Eczema	EPB41L1	Expression profiling by array	GPL7191	GSE12511	Global Expression Profiling in Atopic Eczema Reveals Reciprocal Expression of Inflammatory and Lipid Genes	Plos One
Atopic Eczema	KIAA1276	Expression profiling by array	GPL7191	GSE12511	Global Expression Profiling in Atopic Eczema Reveals Reciprocal Expression of Inflammatory and Lipid Genes	Plos One
Atopic Eczema	GPM6B	Expression profiling by array	GPL7191	GSE12511	Global Expression Profiling in Atopic Eczema Reveals Reciprocal Expression of Inflammatory and Lipid Genes	Plos One
Atopic Eczema	BBS2	Expression profiling by array	GPL7191	GSE12511	Global Expression Profiling in Atopic Eczema Reveals Reciprocal Expression of Inflammatory and Lipid Genes	Plos One
Atopic Eczema	DLX5	Expression profiling by array	GPL7191	GSE12511	Global Expression Profiling in Atopic Eczema Reveals Reciprocal Expression of Inflammatory and Lipid Genes	Plos One
Atopic Eczema	ITFG1	Expression profiling by array	GPL7191	GSE12511	Global Expression Profiling in Atopic Eczema Reveals Reciprocal Expression of Inflammatory and Lipid Genes	Plos One
Atopic Eczema	STAU1	Expression profiling by array	GPL7191	GSE12511	Global Expression Profiling in Atopic Eczema Reveals Reciprocal Expression of Inflammatory and Lipid Genes	Plos One
Atopic Eczema	CAPN13	Expression profiling by array	GPL7191	GSE12511	Global Expression Profiling in Atopic Eczema Reveals Reciprocal Expression of Inflammatory and Lipid Genes	Plos One
Atopic Eczema	LOC147804	Expression profiling by array	GPL7191	GSE12511	Global Expression Profiling in Atopic Eczema Reveals Reciprocal Expression of Inflammatory and Lipid Genes	Plos One
Atopic Eczema	CYP4F3	Expression profiling by array	GPL7191	GSE12511	Global Expression Profiling in Atopic Eczema Reveals Reciprocal Expression of Inflammatory and Lipid Genes	Plos One
Atopic Eczema	CEBPZ	Expression profiling by array	GPL7191	GSE12511	Global Expression Profiling in Atopic Eczema Reveals Reciprocal Expression of Inflammatory and Lipid Genes	Plos One
Atopic Eczema	ACADL	Expression profiling by array	GPL7191	GSE12511	Global Expression Profiling in Atopic Eczema Reveals Reciprocal Expression of Inflammatory and Lipid Genes	Plos One
Atopic Eczema	FBXO18	Expression profiling by array	GPL7191	GSE12511	Global Expression Profiling in Atopic Eczema Reveals Reciprocal Expression of Inflammatory and Lipid Genes	Plos One
Atopic Eczema	NUP153	Expression profiling by array	GPL7191	GSE12511	Global Expression Profiling in Atopic Eczema Reveals Reciprocal Expression of Inflammatory and Lipid Genes	Plos One
Atopic Eczema	SLC27A3	Expression profiling by array	GPL7191	GSE12511	Global Expression Profiling in Atopic Eczema Reveals Reciprocal Expression of Inflammatory and Lipid Genes	Plos One
Atopic Eczema	PPFIBP1	Expression profiling by array	GPL7191	GSE12511	Global Expression Profiling in Atopic Eczema Reveals Reciprocal Expression of Inflammatory and Lipid Genes	Plos One
Atopic Eczema	TMED1	Expression profiling by array	GPL7191	GSE12511	Global Expression Profiling in Atopic Eczema Reveals Reciprocal Expression of Inflammatory and Lipid Genes	Plos One
Atopic Eczema	LOC653750	Expression profiling by array	GPL7191	GSE12511	Global Expression Profiling in Atopic Eczema Reveals Reciprocal Expression of Inflammatory and Lipid Genes	Plos One
Atopic Eczema	C6ORF206	Expression profiling by array	GPL7191	GSE12511	Global Expression Profiling in Atopic Eczema Reveals Reciprocal Expression of Inflammatory and Lipid Genes	Plos One
Atopic Eczema	LYN	Expression profiling by array	GPL7191	GSE12511	Global Expression Profiling in Atopic Eczema Reveals Reciprocal Expression of Inflammatory and Lipid Genes	Plos One
Atopic Eczema	SPON2	Expression profiling by array	GPL7191	GSE12511	Global Expression Profiling in Atopic Eczema Reveals Reciprocal Expression of Inflammatory and Lipid Genes	Plos One
Atopic Eczema	APEH	Expression profiling by array	GPL7191	GSE12511	Global Expression Profiling in Atopic Eczema Reveals Reciprocal Expression of Inflammatory and Lipid Genes	Plos One
Atopic Eczema	NAV1	Expression profiling by array	GPL7191	GSE12511	Global Expression Profiling in Atopic Eczema Reveals Reciprocal Expression of Inflammatory and Lipid Genes	Plos One
Atopic Eczema	UGT1A9	Expression profiling by array	GPL7191	GSE12511	Global Expression Profiling in Atopic Eczema Reveals Reciprocal Expression of Inflammatory and Lipid Genes	Plos One
Atopic Eczema	C18ORF1	Expression profiling by array	GPL7191	GSE12511	Global Expression Profiling in Atopic Eczema Reveals Reciprocal Expression of Inflammatory and Lipid Genes	Plos One
Atopic Eczema	PB1	Expression profiling by array	GPL7191	GSE12511	Global Expression Profiling in Atopic Eczema Reveals Reciprocal Expression of Inflammatory and Lipid Genes	Plos One
Atopic Eczema	PTPN21	Expression profiling by array	GPL7191	GSE12511	Global Expression Profiling in Atopic Eczema Reveals Reciprocal Expression of Inflammatory and Lipid Genes	Plos One
Atopic Eczema	ZBTB7A	Expression profiling by array	GPL7191	GSE12511	Global Expression Profiling in Atopic Eczema Reveals Reciprocal Expression of Inflammatory and Lipid Genes	Plos One
Atopic Eczema	GIT2	Expression profiling by array	GPL7191	GSE12511	Global Expression Profiling in Atopic Eczema Reveals Reciprocal Expression of Inflammatory and Lipid Genes	Plos One
Atopic Eczema	C9ORF46	Expression profiling by array	GPL7191	GSE12511	Global Expression Profiling in Atopic Eczema Reveals Reciprocal Expression of Inflammatory and Lipid Genes	Plos One
Atopic Eczema	NFIA	Expression profiling by array	GPL7191	GSE12511	Global Expression Profiling in Atopic Eczema Reveals Reciprocal Expression of Inflammatory and Lipid Genes	Plos One
Atopic Eczema	ERICH1	Expression profiling by array	GPL7191	GSE12511	Global Expression Profiling in Atopic Eczema Reveals Reciprocal Expression of Inflammatory and Lipid Genes	Plos One
Atopic Eczema	CUTL1	Expression profiling by array	GPL7191	GSE12511	Global Expression Profiling in Atopic Eczema Reveals Reciprocal Expression of Inflammatory and Lipid Genes	Plos One
Atopic Eczema	C6ORF68	Expression profiling by array	GPL7191	GSE12511	Global Expression Profiling in Atopic Eczema Reveals Reciprocal Expression of Inflammatory and Lipid Genes	Plos One
Atopic Eczema	BIC	Expression profiling by array	GPL7191	GSE12511	Global Expression Profiling in Atopic Eczema Reveals Reciprocal Expression of Inflammatory and Lipid Genes	Plos One
Atopic Eczema	ZMYM6	Expression profiling by array	GPL7191	GSE12511	Global Expression Profiling in Atopic Eczema Reveals Reciprocal Expression of Inflammatory and Lipid Genes	Plos One
Atopic Eczema	CDC26	Expression profiling by array	GPL7191	GSE12511	Global Expression Profiling in Atopic Eczema Reveals Reciprocal Expression of Inflammatory and Lipid Genes	Plos One
Atopic Eczema	ARID1B	Expression profiling by array	GPL7191	GSE12511	Global Expression Profiling in Atopic Eczema Reveals Reciprocal Expression of Inflammatory and Lipid Genes	Plos One
Atopic Eczema	LOC401320	Expression profiling by array	GPL7191	GSE12511	Global Expression Profiling in Atopic Eczema Reveals Reciprocal Expression of Inflammatory and Lipid Genes	Plos One
Atopic Eczema	ZNFN1A1	Expression profiling by array	GPL7191	GSE12511	Global Expression Profiling in Atopic Eczema Reveals Reciprocal Expression of Inflammatory and Lipid Genes	Plos One
Atopic Eczema	REEP6	Expression profiling by array	GPL7191	GSE12511	Global Expression Profiling in Atopic Eczema Reveals Reciprocal Expression of Inflammatory and Lipid Genes	Plos One
Atopic Eczema	KIAA0738	Expression profiling by array	GPL7191	GSE12511	Global Expression Profiling in Atopic Eczema Reveals Reciprocal Expression of Inflammatory and Lipid Genes	Plos One
Atopic Eczema	CNN1	Expression profiling by array	GPL7191	GSE12511	Global Expression Profiling in Atopic Eczema Reveals Reciprocal Expression of Inflammatory and Lipid Genes	Plos One
Atopic Eczema	VAMP5	Expression profiling by array	GPL7191	GSE12511	Global Expression Profiling in Atopic Eczema Reveals Reciprocal Expression of Inflammatory and Lipid Genes	Plos One
Atopic Eczema	DEDD2	Expression profiling by array	GPL7191	GSE12511	Global Expression Profiling in Atopic Eczema Reveals Reciprocal Expression of Inflammatory and Lipid Genes	Plos One
Atopic Eczema	ARHGEF1	Expression profiling by array	GPL7191	GSE12511	Global Expression Profiling in Atopic Eczema Reveals Reciprocal Expression of Inflammatory and Lipid Genes	Plos One
Atopic Eczema	RAPGEF3	Expression profiling by array	GPL7191	GSE12511	Global Expression Profiling in Atopic Eczema Reveals Reciprocal Expression of Inflammatory and Lipid Genes	Plos One
Atopic Eczema	UCP2	Expression profiling by array	GPL7191	GSE12511	Global Expression Profiling in Atopic Eczema Reveals Reciprocal Expression of Inflammatory and Lipid Genes	Plos One
Atopic Eczema	GPR98	Expression profiling by array	GPL7191	GSE12511	Global Expression Profiling in Atopic Eczema Reveals Reciprocal Expression of Inflammatory and Lipid Genes	Plos One
Atopic Eczema	TNFAIP8	Expression profiling by array	GPL7191	GSE12511	Global Expression Profiling in Atopic Eczema Reveals Reciprocal Expression of Inflammatory and Lipid Genes	Plos One

Atopic Eczema	F2	Expression profiling by array	GPL7191	GSE12511	Global Expression Profiling in Atopic Eczema Reveals Reciprocal Expression of Inflammatory and Lipid Genes	Plos One
Atopic Eczema	EP300	Expression profiling by array	GPL7191	GSE12511	Global Expression Profiling in Atopic Eczema Reveals Reciprocal Expression of Inflammatory and Lipid Genes	Plos One
Atopic Eczema	SERPINB1	Expression profiling by array	GPL7191	GSE12511	Global Expression Profiling in Atopic Eczema Reveals Reciprocal Expression of Inflammatory and Lipid Genes	Plos One
Atopic Eczema	TMC4	Expression profiling by array	GPL7191	GSE12511	Global Expression Profiling in Atopic Eczema Reveals Reciprocal Expression of Inflammatory and Lipid Genes	Plos One
Atopic Eczema	ZBTB22	Expression profiling by array	GPL7191	GSE12511	Global Expression Profiling in Atopic Eczema Reveals Reciprocal Expression of Inflammatory and Lipid Genes	Plos One
Atopic Eczema	MDS032	Expression profiling by array	GPL7191	GSE12511	Global Expression Profiling in Atopic Eczema Reveals Reciprocal Expression of Inflammatory and Lipid Genes	Plos One
Atopic Eczema	ALDH3A1	Expression profiling by array	GPL7191	GSE12511	Global Expression Profiling in Atopic Eczema Reveals Reciprocal Expression of Inflammatory and Lipid Genes	Plos One
Atopic Eczema	LOC90355	Expression profiling by array	GPL7191	GSE12511	Global Expression Profiling in Atopic Eczema Reveals Reciprocal Expression of Inflammatory and Lipid Genes	Plos One
Atopic Eczema	DDX23	Expression profiling by array	GPL7191	GSE12511	Global Expression Profiling in Atopic Eczema Reveals Reciprocal Expression of Inflammatory and Lipid Genes	Plos One
Atopic Eczema	CHAD	Expression profiling by array	GPL7191	GSE12511	Global Expression Profiling in Atopic Eczema Reveals Reciprocal Expression of Inflammatory and Lipid Genes	Plos One
Atopic Eczema	GALC	Expression profiling by array	GPL7191	GSE12511	Global Expression Profiling in Atopic Eczema Reveals Reciprocal Expression of Inflammatory and Lipid Genes	Plos One
Atopic Eczema	CXX1	Expression profiling by array	GPL7191	GSE12511	Global Expression Profiling in Atopic Eczema Reveals Reciprocal Expression of Inflammatory and Lipid Genes	Plos One
Atopic Eczema	MS4A7	Expression profiling by array	GPL7191	GSE12511	Global Expression Profiling in Atopic Eczema Reveals Reciprocal Expression of Inflammatory and Lipid Genes	Plos One
Atopic Eczema	SLC6A16	Expression profiling by array	GPL7191	GSE12511	Global Expression Profiling in Atopic Eczema Reveals Reciprocal Expression of Inflammatory and Lipid Genes	Plos One
Atopic Eczema	SART1	Expression profiling by array	GPL7191	GSE12511	Global Expression Profiling in Atopic Eczema Reveals Reciprocal Expression of Inflammatory and Lipid Genes	Plos One
Atopic Eczema	TASP1	Expression profiling by array	GPL7191	GSE12511	Global Expression Profiling in Atopic Eczema Reveals Reciprocal Expression of Inflammatory and Lipid Genes	Plos One
Atopic Eczema	TRA2A	Expression profiling by array	GPL7191	GSE12511	Global Expression Profiling in Atopic Eczema Reveals Reciprocal Expression of Inflammatory and Lipid Genes	Plos One
Atopic Eczema	WWP2	Expression profiling by array	GPL7191	GSE12511	Global Expression Profiling in Atopic Eczema Reveals Reciprocal Expression of Inflammatory and Lipid Genes	Plos One
Atopic Eczema	ZFAND2B	Expression profiling by array	GPL7191	GSE12511	Global Expression Profiling in Atopic Eczema Reveals Reciprocal Expression of Inflammatory and Lipid Genes	Plos One
Atopic Eczema	KIAA0040	Expression profiling by array	GPL7191	GSE12511	Global Expression Profiling in Atopic Eczema Reveals Reciprocal Expression of Inflammatory and Lipid Genes	Plos One
Atopic Eczema	BPI	Expression profiling by array	GPL7191	GSE12511	Global Expression Profiling in Atopic Eczema Reveals Reciprocal Expression of Inflammatory and Lipid Genes	Plos One
Atopic Eczema	FAM63A	Expression profiling by array	GPL7191	GSE12511	Global Expression Profiling in Atopic Eczema Reveals Reciprocal Expression of Inflammatory and Lipid Genes	Plos One
Atopic Eczema	IL24	Expression profiling by array	GPL7191	GSE12511	Global Expression Profiling in Atopic Eczema Reveals Reciprocal Expression of Inflammatory and Lipid Genes	Plos One
Atopic Eczema	GNPTAB	Expression profiling by array	GPL7191	GSE12511	Global Expression Profiling in Atopic Eczema Reveals Reciprocal Expression of Inflammatory and Lipid Genes	Plos One
Atopic Eczema	LOC284373	Expression profiling by array	GPL7191	GSE12511	Global Expression Profiling in Atopic Eczema Reveals Reciprocal Expression of Inflammatory and Lipid Genes	Plos One
Atopic Eczema	IMMP2L	Expression profiling by array	GPL7191	GSE12511	Global Expression Profiling in Atopic Eczema Reveals Reciprocal Expression of Inflammatory and Lipid Genes	Plos One
Atopic Eczema	C14ORF72	Expression profiling by array	GPL7191	GSE12511	Global Expression Profiling in Atopic Eczema Reveals Reciprocal Expression of Inflammatory and Lipid Genes	Plos One
Atopic Eczema	KLK6	Expression profiling by array	GPL7191	GSE12511	Global Expression Profiling in Atopic Eczema Reveals Reciprocal Expression of Inflammatory and Lipid Genes	Plos One
Atopic Eczema	LARP5	Expression profiling by array	GPL7191	GSE12511	Global Expression Profiling in Atopic Eczema Reveals Reciprocal Expression of Inflammatory and Lipid Genes	Plos One
Atopic Eczema	TG	Expression profiling by array	GPL7191	GSE12511	Global Expression Profiling in Atopic Eczema Reveals Reciprocal Expression of Inflammatory and Lipid Genes	Plos One
Atopic Eczema	RGL1	Expression profiling by array	GPL7191	GSE12511	Global Expression Profiling in Atopic Eczema Reveals Reciprocal Expression of Inflammatory and Lipid Genes	Plos One
Atopic Eczema	UPF1	Expression profiling by array	GPL7191	GSE12511	Global Expression Profiling in Atopic Eczema Reveals Reciprocal Expression of Inflammatory and Lipid Genes	Plos One
Atopic Eczema	OR2A7	Expression profiling by array	GPL7191	GSE12511	Global Expression Profiling in Atopic Eczema Reveals Reciprocal Expression of Inflammatory and Lipid Genes	Plos One
Atopic Eczema	RAD23B	Expression profiling by array	GPL7191	GSE12511	Global Expression Profiling in Atopic Eczema Reveals Reciprocal Expression of Inflammatory and Lipid Genes	Plos One
Atopic Eczema	UPK1A	Expression profiling by array	GPL7191	GSE12511	Global Expression Profiling in Atopic Eczema Reveals Reciprocal Expression of Inflammatory and Lipid Genes	Plos One
Atopic Eczema	LOC400721	Expression profiling by array	GPL7191	GSE12511	Global Expression Profiling in Atopic Eczema Reveals Reciprocal Expression of Inflammatory and Lipid Genes	Plos One
Atopic Eczema	CDKN3	Expression profiling by array	GPL7191	GSE12511	Global Expression Profiling in Atopic Eczema Reveals Reciprocal Expression of Inflammatory and Lipid Genes	Plos One
Atopic Eczema	ACE2	Expression profiling by array	GPL7191	GSE12511	Global Expression Profiling in Atopic Eczema Reveals Reciprocal Expression of Inflammatory and Lipid Genes	Plos One
Atopic Eczema	LGMN	Expression profiling by array	GPL7191	GSE12511	Global Expression Profiling in Atopic Eczema Reveals Reciprocal Expression of Inflammatory and Lipid Genes	Plos One
Atopic Eczema	FLJ32569	Expression profiling by array	GPL7191	GSE12511	Global Expression Profiling in Atopic Eczema Reveals Reciprocal Expression of Inflammatory and Lipid Genes	Plos One
Atopic Eczema	CPZ	Expression profiling by array	GPL7191	GSE12511	Global Expression Profiling in Atopic Eczema Reveals Reciprocal Expression of Inflammatory and Lipid Genes	Plos One
Atopic Eczema	PRSS23	Expression profiling by array	GPL7191	GSE12511	Global Expression Profiling in Atopic Eczema Reveals Reciprocal Expression of Inflammatory and Lipid Genes	Plos One
Atopic Eczema	KIAA1856	Expression profiling by array	GPL7191	GSE12511	Global Expression Profiling in Atopic Eczema Reveals Reciprocal Expression of Inflammatory and Lipid Genes	Plos One
Atopic Eczema	RP1-93H18.5	Expression profiling by array	GPL7191	GSE12511	Global Expression Profiling in Atopic Eczema Reveals Reciprocal Expression of Inflammatory and Lipid Genes	Plos One
Atopic Eczema	DHCR7	Expression profiling by array	GPL7191	GSE12511	Global Expression Profiling in Atopic Eczema Reveals Reciprocal Expression of Inflammatory and Lipid Genes	Plos One
Atopic Eczema	QKI	Expression profiling by array	GPL7191	GSE12511	Global Expression Profiling in Atopic Eczema Reveals Reciprocal Expression of Inflammatory and Lipid Genes	Plos One
Atopic Eczema	CHI3L2	Expression profiling by array	GPL7191	GSE12511	Global Expression Profiling in Atopic Eczema Reveals Reciprocal Expression of Inflammatory and Lipid Genes	Plos One
Atopic Eczema	CARS	Expression profiling by array	GPL7191	GSE12511	Global Expression Profiling in Atopic Eczema Reveals Reciprocal Expression of Inflammatory and Lipid Genes	Plos One
Atopic Eczema	DHRS10	Expression profiling by array	GPL7191	GSE12511	Global Expression Profiling in Atopic Eczema Reveals Reciprocal Expression of Inflammatory and Lipid Genes	Plos One
Atopic Eczema	FKBP2	Expression profiling by array	GPL7191	GSE12511	Global Expression Profiling in Atopic Eczema Reveals Reciprocal Expression of Inflammatory and Lipid Genes	Plos One
Atopic Eczema	SMC5	Expression profiling by array	GPL7191	GSE12511	Global Expression Profiling in Atopic Eczema Reveals Reciprocal Expression of Inflammatory and Lipid Genes	Plos One
Atopic Eczema	RALBP1	Expression profiling by array	GPL7191	GSE12511	Global Expression Profiling in Atopic Eczema Reveals Reciprocal Expression of Inflammatory and Lipid Genes	Plos One
Atopic Eczema	GPR160	Expression profiling by array	GPL7191	GSE12511	Global Expression Profiling in Atopic Eczema Reveals Reciprocal Expression of Inflammatory and Lipid Genes	Plos One
Atopic Eczema	TMEM109	Expression profiling by array	GPL7191	GSE12511	Global Expression Profiling in Atopic Eczema Reveals Reciprocal Expression of Inflammatory and Lipid Genes	Plos One
Atopic Eczema	LUM	Expression profiling by array	GPL7191	GSE12511	Global Expression Profiling in Atopic Eczema Reveals Reciprocal Expression of Inflammatory and Lipid Genes	Plos One
Atopic Eczema	ZDHHC14	Expression profiling by array	GPL7191	GSE12511	Global Expression Profiling in Atopic Eczema Reveals Reciprocal Expression of Inflammatory and Lipid Genes	Plos One
Atopic Eczema	LAS1L	Expression profiling by array	GPL7191	GSE12511	Global Expression Profiling in Atopic Eczema Reveals Reciprocal Expression of Inflammatory and Lipid Genes	Plos One
Atopic Eczema	ABR	Expression profiling by array	GPL7191	GSE12511	Global Expression Profiling in Atopic Eczema Reveals Reciprocal Expression of Inflammatory and Lipid Genes	Plos One
Atopic Eczema	GBAS	Expression profiling by array	GPL7191	GSE12511	Global Expression Profiling in Atopic Eczema Reveals Reciprocal Expression of Inflammatory and Lipid Genes	Plos One
Atopic Eczema	KRT5	Expression profiling by array	GPL7191	GSE12511	Global Expression Profiling in Atopic Eczema Reveals Reciprocal Expression of Inflammatory and Lipid Genes	Plos One
Atopic Eczema	ABCC11	Expression profiling by array	GPL7191	GSE12511	Global Expression Profiling in Atopic Eczema Reveals Reciprocal Expression of Inflammatory and Lipid Genes	Plos One
Atopic Eczema	GCGR	Expression profiling by array	GPL7191	GSE12511	Global Expression Profiling in Atopic Eczema Reveals Reciprocal Expression of Inflammatory and Lipid Genes	Plos One

Atopic Eczema	ASL	Expression profiling by array	GPL7191	GSE12511	Global Expression Profiling in Atopic Eczema Reveals Reciprocal Expression of Inflammatory and Lipid Genes	Plos One
Atopic Eczema	ARTS-1	Expression profiling by array	GPL7191	GSE12511	Global Expression Profiling in Atopic Eczema Reveals Reciprocal Expression of Inflammatory and Lipid Genes	Plos One
Atopic Eczema	CAPNS2	Expression profiling by array	GPL7191	GSE12511	Global Expression Profiling in Atopic Eczema Reveals Reciprocal Expression of Inflammatory and Lipid Genes	Plos One
Atopic Eczema	GALT	Expression profiling by array	GPL7191	GSE12511	Global Expression Profiling in Atopic Eczema Reveals Reciprocal Expression of Inflammatory and Lipid Genes	Plos One
Atopic Eczema	STXBP5	Expression profiling by array	GPL7191	GSE12511	Global Expression Profiling in Atopic Eczema Reveals Reciprocal Expression of Inflammatory and Lipid Genes	Plos One
Atopic Eczema	C13ORF24	Expression profiling by array	GPL7191	GSE12511	Global Expression Profiling in Atopic Eczema Reveals Reciprocal Expression of Inflammatory and Lipid Genes	Plos One
Atopic Eczema	SCTR	Expression profiling by array	GPL7191	GSE12511	Global Expression Profiling in Atopic Eczema Reveals Reciprocal Expression of Inflammatory and Lipid Genes	Plos One
Atopic Eczema	PON1	Expression profiling by array	GPL7191	GSE12511	Global Expression Profiling in Atopic Eczema Reveals Reciprocal Expression of Inflammatory and Lipid Genes	Plos One
Atopic Eczema	DYSF	Expression profiling by array	GPL7191	GSE12511	Global Expression Profiling in Atopic Eczema Reveals Reciprocal Expression of Inflammatory and Lipid Genes	Plos One
Atopic Eczema	FPRL2	Expression profiling by array	GPL7191	GSE12511	Global Expression Profiling in Atopic Eczema Reveals Reciprocal Expression of Inflammatory and Lipid Genes	Plos One
Atopic Eczema	ZNF521	Expression profiling by array	GPL7191	GSE12511	Global Expression Profiling in Atopic Eczema Reveals Reciprocal Expression of Inflammatory and Lipid Genes	Plos One
Atopic Eczema	FER1L4	Expression profiling by array	GPL7191	GSE12511	Global Expression Profiling in Atopic Eczema Reveals Reciprocal Expression of Inflammatory and Lipid Genes	Plos One
Atopic Eczema	ZNF347	Expression profiling by array	GPL7191	GSE12511	Global Expression Profiling in Atopic Eczema Reveals Reciprocal Expression of Inflammatory and Lipid Genes	Plos One
Atopic Eczema	C10ORF128	Expression profiling by array	GPL7191	GSE12511	Global Expression Profiling in Atopic Eczema Reveals Reciprocal Expression of Inflammatory and Lipid Genes	Plos One
Atopic Eczema	ITCH	Expression profiling by array	GPL7191	GSE12511	Global Expression Profiling in Atopic Eczema Reveals Reciprocal Expression of Inflammatory and Lipid Genes	Plos One
Atopic Eczema	LYPLAL1	Expression profiling by array	GPL7191	GSE12511	Global Expression Profiling in Atopic Eczema Reveals Reciprocal Expression of Inflammatory and Lipid Genes	Plos One
Atopic Eczema	CD47	Expression profiling by array	GPL7191	GSE12511	Global Expression Profiling in Atopic Eczema Reveals Reciprocal Expression of Inflammatory and Lipid Genes	Plos One
Atopic Eczema	RGS18	Expression profiling by array	GPL7191	GSE12511	Global Expression Profiling in Atopic Eczema Reveals Reciprocal Expression of Inflammatory and Lipid Genes	Plos One
Atopic Eczema	GNPAT	Expression profiling by array	GPL7191	GSE12511	Global Expression Profiling in Atopic Eczema Reveals Reciprocal Expression of Inflammatory and Lipid Genes	Plos One
Atopic Eczema	MLN	Expression profiling by array	GPL7191	GSE12511	Global Expression Profiling in Atopic Eczema Reveals Reciprocal Expression of Inflammatory and Lipid Genes	Plos One
Atopic Eczema	RGS6	Expression profiling by array	GPL7191	GSE12511	Global Expression Profiling in Atopic Eczema Reveals Reciprocal Expression of Inflammatory and Lipid Genes	Plos One
Atopic Eczema	VAPA	Expression profiling by array	GPL7191	GSE12511	Global Expression Profiling in Atopic Eczema Reveals Reciprocal Expression of Inflammatory and Lipid Genes	Plos One
Atopic Eczema	C21ORF51	Expression profiling by array	GPL7191	GSE12511	Global Expression Profiling in Atopic Eczema Reveals Reciprocal Expression of Inflammatory and Lipid Genes	Plos One
Atopic Eczema	SPTLC2	Expression profiling by array	GPL7191	GSE12511	Global Expression Profiling in Atopic Eczema Reveals Reciprocal Expression of Inflammatory and Lipid Genes	Plos One
Atopic Eczema	IL1R2	Expression profiling by array	GPL7191	GSE12511	Global Expression Profiling in Atopic Eczema Reveals Reciprocal Expression of Inflammatory and Lipid Genes	Plos One
Atopic Eczema	TNRC6A	Expression profiling by array	GPL7191	GSE12511	Global Expression Profiling in Atopic Eczema Reveals Reciprocal Expression of Inflammatory and Lipid Genes	Plos One
Atopic Eczema	SCAND2	Expression profiling by array	GPL7191	GSE12511	Global Expression Profiling in Atopic Eczema Reveals Reciprocal Expression of Inflammatory and Lipid Genes	Plos One
Atopic Eczema	ITGB1BP1	Expression profiling by array	GPL7191	GSE12511	Global Expression Profiling in Atopic Eczema Reveals Reciprocal Expression of Inflammatory and Lipid Genes	Plos One
Atopic Eczema	LOC401101	Expression profiling by array	GPL7191	GSE12511	Global Expression Profiling in Atopic Eczema Reveals Reciprocal Expression of Inflammatory and Lipid Genes	Plos One
Atopic Eczema	TEAD3	Expression profiling by array	GPL7191	GSE12511	Global Expression Profiling in Atopic Eczema Reveals Reciprocal Expression of Inflammatory and Lipid Genes	Plos One
Atopic Eczema	ETFA	Expression profiling by array	GPL7191	GSE12511	Global Expression Profiling in Atopic Eczema Reveals Reciprocal Expression of Inflammatory and Lipid Genes	Plos One
Atopic Eczema	YWHAZ	Expression profiling by array	GPL7191	GSE12511	Global Expression Profiling in Atopic Eczema Reveals Reciprocal Expression of Inflammatory and Lipid Genes	Plos One
Atopic Eczema	ZNF182	Expression profiling by array	GPL7191	GSE12511	Global Expression Profiling in Atopic Eczema Reveals Reciprocal Expression of Inflammatory and Lipid Genes	Plos One
Atopic Eczema	KIAA1641	Expression profiling by array	GPL7191	GSE12511	Global Expression Profiling in Atopic Eczema Reveals Reciprocal Expression of Inflammatory and Lipid Genes	Plos One
Atopic Eczema	PPARA	Expression profiling by array	GPL7191	GSE12511	Global Expression Profiling in Atopic Eczema Reveals Reciprocal Expression of Inflammatory and Lipid Genes	Plos One
Atopic Eczema	MOSC1	Expression profiling by array	GPL7191	GSE12511	Global Expression Profiling in Atopic Eczema Reveals Reciprocal Expression of Inflammatory and Lipid Genes	Plos One
Atopic Eczema	CCDC82	Expression profiling by array	GPL7191	GSE12511	Global Expression Profiling in Atopic Eczema Reveals Reciprocal Expression of Inflammatory and Lipid Genes	Plos One
Atopic Eczema	VIT	Expression profiling by array	GPL7191	GSE12511	Global Expression Profiling in Atopic Eczema Reveals Reciprocal Expression of Inflammatory and Lipid Genes	Plos One
Atopic Eczema	ADAMDEC1	Expression profiling by array	GPL7191	GSE12511	Global Expression Profiling in Atopic Eczema Reveals Reciprocal Expression of Inflammatory and Lipid Genes	Plos One
Atopic Eczema	PRSS12	Expression profiling by array	GPL7191	GSE12511	Global Expression Profiling in Atopic Eczema Reveals Reciprocal Expression of Inflammatory and Lipid Genes	Plos One
Atopic Eczema	PARD6G	Expression profiling by array	GPL7191	GSE12511	Global Expression Profiling in Atopic Eczema Reveals Reciprocal Expression of Inflammatory and Lipid Genes	Plos One
Atopic Eczema	RPS19	Expression profiling by array	GPL7191	GSE12511	Global Expression Profiling in Atopic Eczema Reveals Reciprocal Expression of Inflammatory and Lipid Genes	Plos One
Atopic Eczema	SEZ6L	Expression profiling by array	GPL7191	GSE12511	Global Expression Profiling in Atopic Eczema Reveals Reciprocal Expression of Inflammatory and Lipid Genes	Plos One
Atopic Eczema	WHDC1L1	Expression profiling by array	GPL7191	GSE12511	Global Expression Profiling in Atopic Eczema Reveals Reciprocal Expression of Inflammatory and Lipid Genes	Plos One
Atopic Eczema	NID2	Expression profiling by array	GPL7191	GSE12511	Global Expression Profiling in Atopic Eczema Reveals Reciprocal Expression of Inflammatory and Lipid Genes	Plos One
Atopic Eczema	CCL19	Expression profiling by array	GPL7191	GSE12511	Global Expression Profiling in Atopic Eczema Reveals Reciprocal Expression of Inflammatory and Lipid Genes	Plos One
Atopic Eczema	MARCH9	Expression profiling by array	GPL7191	GSE12511	Global Expression Profiling in Atopic Eczema Reveals Reciprocal Expression of Inflammatory and Lipid Genes	Plos One
Atopic Eczema	IGSF10	Expression profiling by array	GPL7191	GSE12511	Global Expression Profiling in Atopic Eczema Reveals Reciprocal Expression of Inflammatory and Lipid Genes	Plos One
Atopic Eczema	VHL	Expression profiling by array	GPL7191	GSE12511	Global Expression Profiling in Atopic Eczema Reveals Reciprocal Expression of Inflammatory and Lipid Genes	Plos One
Atopic Eczema	PRKAG1	Expression profiling by array	GPL7191	GSE12511	Global Expression Profiling in Atopic Eczema Reveals Reciprocal Expression of Inflammatory and Lipid Genes	Plos One
Atopic Eczema	STK32C	Expression profiling by array	GPL7191	GSE12511	Global Expression Profiling in Atopic Eczema Reveals Reciprocal Expression of Inflammatory and Lipid Genes	Plos One
Atopic Eczema	GIPC1	Expression profiling by array	GPL7191	GSE12511	Global Expression Profiling in Atopic Eczema Reveals Reciprocal Expression of Inflammatory and Lipid Genes	Plos One
Atopic Eczema	C1QTNF6	Expression profiling by array	GPL7191	GSE12511	Global Expression Profiling in Atopic Eczema Reveals Reciprocal Expression of Inflammatory and Lipid Genes	Plos One
Atopic Eczema	COL5A1	Expression profiling by array	GPL7191	GSE12511	Global Expression Profiling in Atopic Eczema Reveals Reciprocal Expression of Inflammatory and Lipid Genes	Plos One
Atopic Eczema	EIF2B4	Expression profiling by array	GPL7191	GSE12511	Global Expression Profiling in Atopic Eczema Reveals Reciprocal Expression of Inflammatory and Lipid Genes	Plos One
Atopic Eczema	LOC646324	Expression profiling by array	GPL7191	GSE12511	Global Expression Profiling in Atopic Eczema Reveals Reciprocal Expression of Inflammatory and Lipid Genes	Plos One
Atopic Eczema	MGC40579	Expression profiling by array	GPL7191	GSE12511	Global Expression Profiling in Atopic Eczema Reveals Reciprocal Expression of Inflammatory and Lipid Genes	Plos One
Atopic Eczema	MRPS25	Expression profiling by array	GPL7191	GSE12511	Global Expression Profiling in Atopic Eczema Reveals Reciprocal Expression of Inflammatory and Lipid Genes	Plos One
Atopic Eczema	C1ORF150	Expression profiling by array	GPL7191	GSE12511	Global Expression Profiling in Atopic Eczema Reveals Reciprocal Expression of Inflammatory and Lipid Genes	Plos One
Atopic Eczema	LOC94431	Expression profiling by array	GPL7191	GSE12511	Global Expression Profiling in Atopic Eczema Reveals Reciprocal Expression of Inflammatory and Lipid Genes	Plos One
Atopic Eczema	CAPZB	Expression profiling by array	GPL7191	GSE12511	Global Expression Profiling in Atopic Eczema Reveals Reciprocal Expression of Inflammatory and Lipid Genes	Plos One
Atopic Eczema	WDR66	Expression profiling by array	GPL7191	GSE12511	Global Expression Profiling in Atopic Eczema Reveals Reciprocal Expression of Inflammatory and Lipid Genes	Plos One

Atopic Eczema	PEX1	Expression profiling by array	GPL7191	GSE12511	Global Expression Profiling in Atopic Eczema Reveals Reciprocal Expression of Inflammatory and Lipid Genes	Plos One
Atopic Eczema	ABCB11	Expression profiling by array	GPL7191	GSE12511	Global Expression Profiling in Atopic Eczema Reveals Reciprocal Expression of Inflammatory and Lipid Genes	Plos One
Atopic Eczema	C22ORF5	Expression profiling by array	GPL7191	GSE12511	Global Expression Profiling in Atopic Eczema Reveals Reciprocal Expression of Inflammatory and Lipid Genes	Plos One
Atopic Eczema	RNF14	Expression profiling by array	GPL7191	GSE12511	Global Expression Profiling in Atopic Eczema Reveals Reciprocal Expression of Inflammatory and Lipid Genes	Plos One
Atopic Eczema	PLA2G12A	Expression profiling by array	GPL7191	GSE12511	Global Expression Profiling in Atopic Eczema Reveals Reciprocal Expression of Inflammatory and Lipid Genes	Plos One
Atopic Eczema	TOPBP1	Expression profiling by array	GPL7191	GSE12511	Global Expression Profiling in Atopic Eczema Reveals Reciprocal Expression of Inflammatory and Lipid Genes	Plos One
Atopic Eczema	PAXIP1	Expression profiling by array	GPL7191	GSE12511	Global Expression Profiling in Atopic Eczema Reveals Reciprocal Expression of Inflammatory and Lipid Genes	Plos One
Atopic Eczema	ACO1	Expression profiling by array	GPL7191	GSE12511	Global Expression Profiling in Atopic Eczema Reveals Reciprocal Expression of Inflammatory and Lipid Genes	Plos One
Atopic Eczema	CHPT1	Expression profiling by array	GPL7191	GSE12511	Global Expression Profiling in Atopic Eczema Reveals Reciprocal Expression of Inflammatory and Lipid Genes	Plos One
Atopic Eczema	LOC441124	Expression profiling by array	GPL7191	GSE12511	Global Expression Profiling in Atopic Eczema Reveals Reciprocal Expression of Inflammatory and Lipid Genes	Plos One
Atopic Eczema	EIF4EBP2	Expression profiling by array	GPL7191	GSE12511	Global Expression Profiling in Atopic Eczema Reveals Reciprocal Expression of Inflammatory and Lipid Genes	Plos One
Atopic Eczema	LOC643338	Expression profiling by array	GPL7191	GSE12511	Global Expression Profiling in Atopic Eczema Reveals Reciprocal Expression of Inflammatory and Lipid Genes	Plos One
Atopic Eczema	ECE1	Expression profiling by array	GPL7191	GSE12511	Global Expression Profiling in Atopic Eczema Reveals Reciprocal Expression of Inflammatory and Lipid Genes	Plos One
Atopic Eczema	C1ORF106	Expression profiling by array	GPL7191	GSE12511	Global Expression Profiling in Atopic Eczema Reveals Reciprocal Expression of Inflammatory and Lipid Genes	Plos One
Atopic Eczema	TFPI	Expression profiling by array	GPL7191	GSE12511	Global Expression Profiling in Atopic Eczema Reveals Reciprocal Expression of Inflammatory and Lipid Genes	Plos One
Atopic Eczema	RHOH	Expression profiling by array	GPL7191	GSE12511	Global Expression Profiling in Atopic Eczema Reveals Reciprocal Expression of Inflammatory and Lipid Genes	Plos One
Atopic Eczema	ABI3BP	Expression profiling by array	GPL7191	GSE12511	Global Expression Profiling in Atopic Eczema Reveals Reciprocal Expression of Inflammatory and Lipid Genes	Plos One
Atopic Eczema	CD300LG	Expression profiling by array	GPL7191	GSE12511	Global Expression Profiling in Atopic Eczema Reveals Reciprocal Expression of Inflammatory and Lipid Genes	Plos One
Atopic Eczema	C6ORF48	Expression profiling by array	GPL7191	GSE12511	Global Expression Profiling in Atopic Eczema Reveals Reciprocal Expression of Inflammatory and Lipid Genes	Plos One
Atopic Eczema	GPR108	Expression profiling by array	GPL7191	GSE12511	Global Expression Profiling in Atopic Eczema Reveals Reciprocal Expression of Inflammatory and Lipid Genes	Plos One
Atopic Eczema	TRIM56	Expression profiling by array	GPL7191	GSE12511	Global Expression Profiling in Atopic Eczema Reveals Reciprocal Expression of Inflammatory and Lipid Genes	Plos One
Atopic Eczema	TXN2	Expression profiling by array	GPL7191	GSE12511	Global Expression Profiling in Atopic Eczema Reveals Reciprocal Expression of Inflammatory and Lipid Genes	Plos One
Atopic Eczema	CNTROB	Expression profiling by array	GPL7191	GSE12511	Global Expression Profiling in Atopic Eczema Reveals Reciprocal Expression of Inflammatory and Lipid Genes	Plos One
Atopic Eczema	TINAG	Expression profiling by array	GPL7191	GSE12511	Global Expression Profiling in Atopic Eczema Reveals Reciprocal Expression of Inflammatory and Lipid Genes	Plos One
Atopic Eczema	NBR1	Expression profiling by array	GPL7191	GSE12511	Global Expression Profiling in Atopic Eczema Reveals Reciprocal Expression of Inflammatory and Lipid Genes	Plos One
Atopic Eczema	ADRBK2	Expression profiling by array	GPL7191	GSE12511	Global Expression Profiling in Atopic Eczema Reveals Reciprocal Expression of Inflammatory and Lipid Genes	Plos One
Atopic Eczema	TFDP2	Expression profiling by array	GPL7191	GSE12511	Global Expression Profiling in Atopic Eczema Reveals Reciprocal Expression of Inflammatory and Lipid Genes	Plos One
Atopic Eczema	SH3PX3	Expression profiling by array	GPL7191	GSE12511	Global Expression Profiling in Atopic Eczema Reveals Reciprocal Expression of Inflammatory and Lipid Genes	Plos One
Atopic Eczema	LITAF	Expression profiling by array	GPL7191	GSE12511	Global Expression Profiling in Atopic Eczema Reveals Reciprocal Expression of Inflammatory and Lipid Genes	Plos One
Atopic Eczema	MGC52110	Expression profiling by array	GPL7191	GSE12511	Global Expression Profiling in Atopic Eczema Reveals Reciprocal Expression of Inflammatory and Lipid Genes	Plos One
Atopic Eczema	MFSD2	Expression profiling by array	GPL7191	GSE12511	Global Expression Profiling in Atopic Eczema Reveals Reciprocal Expression of Inflammatory and Lipid Genes	Plos One
Atopic Eczema	RBP2	Expression profiling by array	GPL7191	GSE12511	Global Expression Profiling in Atopic Eczema Reveals Reciprocal Expression of Inflammatory and Lipid Genes	Plos One
Atopic Eczema	SH2D1A	Expression profiling by array	GPL7191	GSE12511	Global Expression Profiling in Atopic Eczema Reveals Reciprocal Expression of Inflammatory and Lipid Genes	Plos One
Atopic Eczema	NOD27	Expression profiling by array	GPL7191	GSE12511	Global Expression Profiling in Atopic Eczema Reveals Reciprocal Expression of Inflammatory and Lipid Genes	Plos One
Atopic Eczema	PSMB6	Expression profiling by array	GPL7191	GSE12511	Global Expression Profiling in Atopic Eczema Reveals Reciprocal Expression of Inflammatory and Lipid Genes	Plos One
Atopic Eczema	APAF1	Expression profiling by array	GPL7191	GSE12511	Global Expression Profiling in Atopic Eczema Reveals Reciprocal Expression of Inflammatory and Lipid Genes	Plos One
Atopic Eczema	PAQR8	Expression profiling by array	GPL7191	GSE12511	Global Expression Profiling in Atopic Eczema Reveals Reciprocal Expression of Inflammatory and Lipid Genes	Plos One
Atopic Eczema	C20ORF55	Expression profiling by array	GPL7191	GSE12511	Global Expression Profiling in Atopic Eczema Reveals Reciprocal Expression of Inflammatory and Lipid Genes	Plos One
Atopic Eczema	SCARF2	Expression profiling by array	GPL7191	GSE12511	Global Expression Profiling in Atopic Eczema Reveals Reciprocal Expression of Inflammatory and Lipid Genes	Plos One
Atopic Eczema	CENPN	Expression profiling by array	GPL7191	GSE12511	Global Expression Profiling in Atopic Eczema Reveals Reciprocal Expression of Inflammatory and Lipid Genes	Plos One
Atopic Eczema	EFNB1	Expression profiling by array	GPL7191	GSE12511	Global Expression Profiling in Atopic Eczema Reveals Reciprocal Expression of Inflammatory and Lipid Genes	Plos One
Atopic Eczema	SLC25A21	Expression profiling by array	GPL7191	GSE12511	Global Expression Profiling in Atopic Eczema Reveals Reciprocal Expression of Inflammatory and Lipid Genes	Plos One
Atopic Eczema	ZNF503	Expression profiling by array	GPL7191	GSE12511	Global Expression Profiling in Atopic Eczema Reveals Reciprocal Expression of Inflammatory and Lipid Genes	Plos One
Atopic Eczema	FLJ38482	Expression profiling by array	GPL7191	GSE12511	Global Expression Profiling in Atopic Eczema Reveals Reciprocal Expression of Inflammatory and Lipid Genes	Plos One
Atopic Eczema	BAT4	Expression profiling by array	GPL7191	GSE12511	Global Expression Profiling in Atopic Eczema Reveals Reciprocal Expression of Inflammatory and Lipid Genes	Plos One
Atopic Eczema	COL4A2	Expression profiling by array	GPL7191	GSE12511	Global Expression Profiling in Atopic Eczema Reveals Reciprocal Expression of Inflammatory and Lipid Genes	Plos One
Atopic Eczema	AKAP11	Expression profiling by array	GPL7191	GSE12511	Global Expression Profiling in Atopic Eczema Reveals Reciprocal Expression of Inflammatory and Lipid Genes	Plos One
Atopic Eczema	PLD1	Expression profiling by array	GPL7191	GSE12511	Global Expression Profiling in Atopic Eczema Reveals Reciprocal Expression of Inflammatory and Lipid Genes	Plos One
Atopic Eczema	FLJ20186	Expression profiling by array	GPL7191	GSE12511	Global Expression Profiling in Atopic Eczema Reveals Reciprocal Expression of Inflammatory and Lipid Genes	Plos One
Atopic Eczema	METTL9	Expression profiling by array	GPL7191	GSE12511	Global Expression Profiling in Atopic Eczema Reveals Reciprocal Expression of Inflammatory and Lipid Genes	Plos One
Atopic Eczema	PEX19	Expression profiling by array	GPL7191	GSE12511	Global Expression Profiling in Atopic Eczema Reveals Reciprocal Expression of Inflammatory and Lipid Genes	Plos One
Atopic Eczema	DGKH	Expression profiling by array	GPL7191	GSE12511	Global Expression Profiling in Atopic Eczema Reveals Reciprocal Expression of Inflammatory and Lipid Genes	Plos One
Atopic Eczema	WWTR1	Expression profiling by array	GPL7191	GSE12511	Global Expression Profiling in Atopic Eczema Reveals Reciprocal Expression of Inflammatory and Lipid Genes	Plos One
Atopic Eczema	CTSD	Expression profiling by array	GPL7191	GSE12511	Global Expression Profiling in Atopic Eczema Reveals Reciprocal Expression of Inflammatory and Lipid Genes	Plos One
Atopic Eczema	KIAA0226	Expression profiling by array	GPL7191	GSE12511	Global Expression Profiling in Atopic Eczema Reveals Reciprocal Expression of Inflammatory and Lipid Genes	Plos One
Atopic Eczema	C6ORF50	Expression profiling by array	GPL7191	GSE12511	Global Expression Profiling in Atopic Eczema Reveals Reciprocal Expression of Inflammatory and Lipid Genes	Plos One
Atopic Eczema	IKIP	Expression profiling by array	GPL7191	GSE12511	Global Expression Profiling in Atopic Eczema Reveals Reciprocal Expression of Inflammatory and Lipid Genes	Plos One
Atopic Eczema	DMXL1	Expression profiling by array	GPL7191	GSE12511	Global Expression Profiling in Atopic Eczema Reveals Reciprocal Expression of Inflammatory and Lipid Genes	Plos One
Atopic Eczema	PCDHGC3	Expression profiling by array	GPL7191	GSE12511	Global Expression Profiling in Atopic Eczema Reveals Reciprocal Expression of Inflammatory and Lipid Genes	Plos One
Atopic Eczema	SNF8	Expression profiling by array	GPL7191	GSE12511	Global Expression Profiling in Atopic Eczema Reveals Reciprocal Expression of Inflammatory and Lipid Genes	Plos One
Atopic Eczema	PES1	Expression profiling by array	GPL7191	GSE12511	Global Expression Profiling in Atopic Eczema Reveals Reciprocal Expression of Inflammatory and Lipid Genes	Plos One
Atopic Eczema	PACSIN2	Expression profiling by array	GPL7191	GSE12511	Global Expression Profiling in Atopic Eczema Reveals Reciprocal Expression of Inflammatory and Lipid Genes	Plos One

Atopic Eczema	BEAN	Expression profiling by array	GPL7191	GSE12511	Global Expression Profiling in Atopic Eczema Reveals Reciprocal Expression of Inflammatory and Lipid Genes	Plos One
Atopic Eczema	BLK	Expression profiling by array	GPL7191	GSE12511	Global Expression Profiling in Atopic Eczema Reveals Reciprocal Expression of Inflammatory and Lipid Genes	Plos One
Atopic Eczema	WDR25	Expression profiling by array	GPL7191	GSE12511	Global Expression Profiling in Atopic Eczema Reveals Reciprocal Expression of Inflammatory and Lipid Genes	Plos One
Atopic Eczema	TACSTD2	Expression profiling by array	GPL7191	GSE12511	Global Expression Profiling in Atopic Eczema Reveals Reciprocal Expression of Inflammatory and Lipid Genes	Plos One
Atopic Eczema	FUT1	Expression profiling by array	GPL7191	GSE12511	Global Expression Profiling in Atopic Eczema Reveals Reciprocal Expression of Inflammatory and Lipid Genes	Plos One
Atopic Eczema	TLE1	Expression profiling by array	GPL7191	GSE12511	Global Expression Profiling in Atopic Eczema Reveals Reciprocal Expression of Inflammatory and Lipid Genes	Plos One
Atopic Eczema	IFRD1	Expression profiling by array	GPL7191	GSE12511	Global Expression Profiling in Atopic Eczema Reveals Reciprocal Expression of Inflammatory and Lipid Genes	Plos One
Atopic Eczema	PCMTD1	Expression profiling by array	GPL7191	GSE12511	Global Expression Profiling in Atopic Eczema Reveals Reciprocal Expression of Inflammatory and Lipid Genes	Plos One
Atopic Eczema	PTPRS	Expression profiling by array	GPL7191	GSE12511	Global Expression Profiling in Atopic Eczema Reveals Reciprocal Expression of Inflammatory and Lipid Genes	Plos One
Atopic Eczema	PPAN	Expression profiling by array	GPL7191	GSE12511	Global Expression Profiling in Atopic Eczema Reveals Reciprocal Expression of Inflammatory and Lipid Genes	Plos One
Atopic Eczema	UCK2	Expression profiling by array	GPL7191	GSE12511	Global Expression Profiling in Atopic Eczema Reveals Reciprocal Expression of Inflammatory and Lipid Genes	Plos One
Atopic Eczema	IL17D	Expression profiling by array	GPL7191	GSE12511	Global Expression Profiling in Atopic Eczema Reveals Reciprocal Expression of Inflammatory and Lipid Genes	Plos One
Atopic Eczema	PSCD3	Expression profiling by array	GPL7191	GSE12511	Global Expression Profiling in Atopic Eczema Reveals Reciprocal Expression of Inflammatory and Lipid Genes	Plos One
Atopic Eczema	ZNF462	Expression profiling by array	GPL7191	GSE12511	Global Expression Profiling in Atopic Eczema Reveals Reciprocal Expression of Inflammatory and Lipid Genes	Plos One
Atopic Eczema	COPG	Expression profiling by array	GPL7191	GSE12511	Global Expression Profiling in Atopic Eczema Reveals Reciprocal Expression of Inflammatory and Lipid Genes	Plos One
Atopic Eczema	C2ORF11	Expression profiling by array	GPL7191	GSE12511	Global Expression Profiling in Atopic Eczema Reveals Reciprocal Expression of Inflammatory and Lipid Genes	Plos One
Atopic Eczema	FXC1	Expression profiling by array	GPL7191	GSE12511	Global Expression Profiling in Atopic Eczema Reveals Reciprocal Expression of Inflammatory and Lipid Genes	Plos One
Atopic Eczema	LOC441119	Expression profiling by array	GPL7191	GSE12511	Global Expression Profiling in Atopic Eczema Reveals Reciprocal Expression of Inflammatory and Lipid Genes	Plos One
Atopic Eczema	PARD3	Expression profiling by array	GPL7191	GSE12511	Global Expression Profiling in Atopic Eczema Reveals Reciprocal Expression of Inflammatory and Lipid Genes	Plos One
Atopic Eczema	KIAA1411	Expression profiling by array	GPL7191	GSE12511	Global Expression Profiling in Atopic Eczema Reveals Reciprocal Expression of Inflammatory and Lipid Genes	Plos One
Atopic Eczema	GPNMB	Expression profiling by array	GPL7191	GSE12511	Global Expression Profiling in Atopic Eczema Reveals Reciprocal Expression of Inflammatory and Lipid Genes	Plos One
Atopic Eczema	LRP1	Expression profiling by array	GPL7191	GSE12511	Global Expression Profiling in Atopic Eczema Reveals Reciprocal Expression of Inflammatory and Lipid Genes	Plos One
Atopic Eczema	MAOA	Expression profiling by array	GPL7191	GSE12511	Global Expression Profiling in Atopic Eczema Reveals Reciprocal Expression of Inflammatory and Lipid Genes	Plos One
Atopic Eczema	NOS1AP	Expression profiling by array	GPL7191	GSE12511	Global Expression Profiling in Atopic Eczema Reveals Reciprocal Expression of Inflammatory and Lipid Genes	Plos One
Atopic Eczema	DUSP16	Expression profiling by array	GPL7191	GSE12511	Global Expression Profiling in Atopic Eczema Reveals Reciprocal Expression of Inflammatory and Lipid Genes	Plos One
Atopic Eczema	CCDC96	Expression profiling by array	GPL7191	GSE12511	Global Expression Profiling in Atopic Eczema Reveals Reciprocal Expression of Inflammatory and Lipid Genes	Plos One
Atopic Eczema	CRTAP	Expression profiling by array	GPL7191	GSE12511	Global Expression Profiling in Atopic Eczema Reveals Reciprocal Expression of Inflammatory and Lipid Genes	Plos One
Atopic Eczema	TPSAB1	Expression profiling by array	GPL7191	GSE12511	Global Expression Profiling in Atopic Eczema Reveals Reciprocal Expression of Inflammatory and Lipid Genes	Plos One
Atopic Eczema	BPHL	Expression profiling by array	GPL7191	GSE12511	Global Expression Profiling in Atopic Eczema Reveals Reciprocal Expression of Inflammatory and Lipid Genes	Plos One
Atopic Eczema	TSNARE1	Expression profiling by array	GPL7191	GSE12511	Global Expression Profiling in Atopic Eczema Reveals Reciprocal Expression of Inflammatory and Lipid Genes	Plos One
Atopic Eczema	ARL6IP6	Expression profiling by array	GPL7191	GSE12511	Global Expression Profiling in Atopic Eczema Reveals Reciprocal Expression of Inflammatory and Lipid Genes	Plos One
Atopic Eczema	RYBP	Expression profiling by array	GPL7191	GSE12511	Global Expression Profiling in Atopic Eczema Reveals Reciprocal Expression of Inflammatory and Lipid Genes	Plos One
Atopic Eczema	SLC7A5	Expression profiling by array	GPL7191	GSE12511	Global Expression Profiling in Atopic Eczema Reveals Reciprocal Expression of Inflammatory and Lipid Genes	Plos One
Atopic Eczema	SUSD2	Expression profiling by array	GPL7191	GSE12511	Global Expression Profiling in Atopic Eczema Reveals Reciprocal Expression of Inflammatory and Lipid Genes	Plos One
Atopic Eczema	C8ORF72	Expression profiling by array	GPL7191	GSE12511	Global Expression Profiling in Atopic Eczema Reveals Reciprocal Expression of Inflammatory and Lipid Genes	Plos One
Atopic Eczema	SPIRE1	Expression profiling by array	GPL7191	GSE12511	Global Expression Profiling in Atopic Eczema Reveals Reciprocal Expression of Inflammatory and Lipid Genes	Plos One
Atopic Eczema	GMDS	Expression profiling by array	GPL7191	GSE12511	Global Expression Profiling in Atopic Eczema Reveals Reciprocal Expression of Inflammatory and Lipid Genes	Plos One
Atopic Eczema	GPR156	Expression profiling by array	GPL7191	GSE12511	Global Expression Profiling in Atopic Eczema Reveals Reciprocal Expression of Inflammatory and Lipid Genes	Plos One
Atopic Eczema	CYR61	Expression profiling by array	GPL7191	GSE12511	Global Expression Profiling in Atopic Eczema Reveals Reciprocal Expression of Inflammatory and Lipid Genes	Plos One
Atopic Eczema	FCRLM1	Expression profiling by array	GPL7191	GSE12511	Global Expression Profiling in Atopic Eczema Reveals Reciprocal Expression of Inflammatory and Lipid Genes	Plos One
Atopic Eczema	RNF149	Expression profiling by array	GPL7191	GSE12511	Global Expression Profiling in Atopic Eczema Reveals Reciprocal Expression of Inflammatory and Lipid Genes	Plos One
Atopic Eczema	SHC2	Expression profiling by array	GPL7191	GSE12511	Global Expression Profiling in Atopic Eczema Reveals Reciprocal Expression of Inflammatory and Lipid Genes	Plos One
Atopic Eczema	STOM	Expression profiling by array	GPL7191	GSE12511	Global Expression Profiling in Atopic Eczema Reveals Reciprocal Expression of Inflammatory and Lipid Genes	Plos One
Atopic Eczema	SLC22A9	Expression profiling by array	GPL7191	GSE12511	Global Expression Profiling in Atopic Eczema Reveals Reciprocal Expression of Inflammatory and Lipid Genes	Plos One
Atopic Eczema	CYP39A1	Expression profiling by array	GPL7191	GSE12511	Global Expression Profiling in Atopic Eczema Reveals Reciprocal Expression of Inflammatory and Lipid Genes	Plos One
Atopic Eczema	SMAP1L	Expression profiling by array	GPL7191	GSE12511	Global Expression Profiling in Atopic Eczema Reveals Reciprocal Expression of Inflammatory and Lipid Genes	Plos One
Atopic Eczema	VPS41	Expression profiling by array	GPL7191	GSE12511	Global Expression Profiling in Atopic Eczema Reveals Reciprocal Expression of Inflammatory and Lipid Genes	Plos One
Atopic Eczema	MAP2K1	Expression profiling by array	GPL7191	GSE12511	Global Expression Profiling in Atopic Eczema Reveals Reciprocal Expression of Inflammatory and Lipid Genes	Plos One
Atopic Eczema	BID	Expression profiling by array	GPL7191	GSE12511	Global Expression Profiling in Atopic Eczema Reveals Reciprocal Expression of Inflammatory and Lipid Genes	Plos One
Atopic Eczema	GTL3	Expression profiling by array	GPL7191	GSE12511	Global Expression Profiling in Atopic Eczema Reveals Reciprocal Expression of Inflammatory and Lipid Genes	Plos One
Atopic Eczema	SLC11A2	Expression profiling by array	GPL7191	GSE12511	Global Expression Profiling in Atopic Eczema Reveals Reciprocal Expression of Inflammatory and Lipid Genes	Plos One
Atopic Eczema	TMEM77	Expression profiling by array	GPL7191	GSE12511	Global Expression Profiling in Atopic Eczema Reveals Reciprocal Expression of Inflammatory and Lipid Genes	Plos One
Atopic Eczema	SSX4	Expression profiling by array	GPL7191	GSE12511	Global Expression Profiling in Atopic Eczema Reveals Reciprocal Expression of Inflammatory and Lipid Genes	Plos One
Atopic Eczema	MERTK	Expression profiling by array	GPL7191	GSE12511	Global Expression Profiling in Atopic Eczema Reveals Reciprocal Expression of Inflammatory and Lipid Genes	Plos One
Atopic Eczema	M-RIP	Expression profiling by array	GPL7191	GSE12511	Global Expression Profiling in Atopic Eczema Reveals Reciprocal Expression of Inflammatory and Lipid Genes	Plos One
Atopic Eczema	LDLRAP1	Expression profiling by array	GPL7191	GSE12511	Global Expression Profiling in Atopic Eczema Reveals Reciprocal Expression of Inflammatory and Lipid Genes	Plos One
Atopic Eczema	ELAC2	Expression profiling by array	GPL7191	GSE12511	Global Expression Profiling in Atopic Eczema Reveals Reciprocal Expression of Inflammatory and Lipid Genes	Plos One
Atopic Eczema	LAMB1	Expression profiling by array	GPL7191	GSE12511	Global Expression Profiling in Atopic Eczema Reveals Reciprocal Expression of Inflammatory and Lipid Genes	Plos One
Atopic Eczema	MOV10	Expression profiling by array	GPL7191	GSE12511	Global Expression Profiling in Atopic Eczema Reveals Reciprocal Expression of Inflammatory and Lipid Genes	Plos One
Atopic Eczema	CAMTA1	Expression profiling by array	GPL7191	GSE12511	Global Expression Profiling in Atopic Eczema Reveals Reciprocal Expression of Inflammatory and Lipid Genes	Plos One
Atopic Eczema	SMO	Expression profiling by array	GPL7191	GSE12511	Global Expression Profiling in Atopic Eczema Reveals Reciprocal Expression of Inflammatory and Lipid Genes	Plos One
Atopic Eczema	ELAVL1	Expression profiling by array	GPL7191	GSE12511	Global Expression Profiling in Atopic Eczema Reveals Reciprocal Expression of Inflammatory and Lipid Genes	Plos One

Atopic Eczema	SERPINA1	Expression profiling by array	GPL7191	GSE12511	Global Expression Profiling in Atopic Eczema Reveals Reciprocal Expression of Inflammatory and Lipid Genes	Plos One
Atopic Eczema	C3ORF1	Expression profiling by array	GPL7191	GSE12511	Global Expression Profiling in Atopic Eczema Reveals Reciprocal Expression of Inflammatory and Lipid Genes	Plos One
Atopic Eczema	ATAD3B	Expression profiling by array	GPL7191	GSE12511	Global Expression Profiling in Atopic Eczema Reveals Reciprocal Expression of Inflammatory and Lipid Genes	Plos One
Atopic Eczema	ARPC4	Expression profiling by array	GPL7191	GSE12511	Global Expression Profiling in Atopic Eczema Reveals Reciprocal Expression of Inflammatory and Lipid Genes	Plos One
Atopic Eczema	KIAA1632	Expression profiling by array	GPL7191	GSE12511	Global Expression Profiling in Atopic Eczema Reveals Reciprocal Expression of Inflammatory and Lipid Genes	Plos One
Atopic Eczema	KCNT2	Expression profiling by array	GPL7191	GSE12511	Global Expression Profiling in Atopic Eczema Reveals Reciprocal Expression of Inflammatory and Lipid Genes	Plos One
Atopic Eczema	KAL1	Expression profiling by array	GPL7191	GSE12511	Global Expression Profiling in Atopic Eczema Reveals Reciprocal Expression of Inflammatory and Lipid Genes	Plos One
Atopic Eczema	KIAA1045	Expression profiling by array	GPL7191	GSE12511	Global Expression Profiling in Atopic Eczema Reveals Reciprocal Expression of Inflammatory and Lipid Genes	Plos One
Atopic Eczema	NOPE	Expression profiling by array	GPL7191	GSE12511	Global Expression Profiling in Atopic Eczema Reveals Reciprocal Expression of Inflammatory and Lipid Genes	Plos One
Atopic Eczema	APOBEC3B	Expression profiling by array	GPL7191	GSE12511	Global Expression Profiling in Atopic Eczema Reveals Reciprocal Expression of Inflammatory and Lipid Genes	Plos One
Atopic Eczema	ATP5J2	Expression profiling by array	GPL7191	GSE12511	Global Expression Profiling in Atopic Eczema Reveals Reciprocal Expression of Inflammatory and Lipid Genes	Plos One
Atopic Eczema	TGFBR2	Expression profiling by array	GPL7191	GSE12511	Global Expression Profiling in Atopic Eczema Reveals Reciprocal Expression of Inflammatory and Lipid Genes	Plos One
Atopic Eczema	SLC29A1	Expression profiling by array	GPL7191	GSE12511	Global Expression Profiling in Atopic Eczema Reveals Reciprocal Expression of Inflammatory and Lipid Genes	Plos One
Atopic Eczema	DFNA5	Expression profiling by array	GPL7191	GSE12511	Global Expression Profiling in Atopic Eczema Reveals Reciprocal Expression of Inflammatory and Lipid Genes	Plos One
Atopic Eczema	PLGLB2	Expression profiling by array	GPL7191	GSE12511	Global Expression Profiling in Atopic Eczema Reveals Reciprocal Expression of Inflammatory and Lipid Genes	Plos One
Atopic Eczema	PTPRCAP	Expression profiling by array	GPL7191	GSE12511	Global Expression Profiling in Atopic Eczema Reveals Reciprocal Expression of Inflammatory and Lipid Genes	Plos One
Atopic Eczema	NHSL1	Expression profiling by array	GPL7191	GSE12511	Global Expression Profiling in Atopic Eczema Reveals Reciprocal Expression of Inflammatory and Lipid Genes	Plos One
Atopic Eczema	RALGPS2	Expression profiling by array	GPL7191	GSE12511	Global Expression Profiling in Atopic Eczema Reveals Reciprocal Expression of Inflammatory and Lipid Genes	Plos One
Atopic Eczema	LOC402110	Expression profiling by array	GPL7191	GSE12511	Global Expression Profiling in Atopic Eczema Reveals Reciprocal Expression of Inflammatory and Lipid Genes	Plos One
Atopic Eczema	LOC643774	Expression profiling by array	GPL7191	GSE12511	Global Expression Profiling in Atopic Eczema Reveals Reciprocal Expression of Inflammatory and Lipid Genes	Plos One
Atopic Eczema	ATF5	Expression profiling by array	GPL7191	GSE12511	Global Expression Profiling in Atopic Eczema Reveals Reciprocal Expression of Inflammatory and Lipid Genes	Plos One
Atopic Eczema	KIF23	Expression profiling by array	GPL7191	GSE12511	Global Expression Profiling in Atopic Eczema Reveals Reciprocal Expression of Inflammatory and Lipid Genes	Plos One
Atopic Eczema	MXI1	Expression profiling by array	GPL7191	GSE12511	Global Expression Profiling in Atopic Eczema Reveals Reciprocal Expression of Inflammatory and Lipid Genes	Plos One
Atopic Eczema	PRKCQ	Expression profiling by array	GPL7191	GSE12511	Global Expression Profiling in Atopic Eczema Reveals Reciprocal Expression of Inflammatory and Lipid Genes	Plos One
Atopic Eczema	MAK	Expression profiling by array	GPL7191	GSE12511	Global Expression Profiling in Atopic Eczema Reveals Reciprocal Expression of Inflammatory and Lipid Genes	Plos One
Atopic Eczema	LOC90624	Expression profiling by array	GPL7191	GSE12511	Global Expression Profiling in Atopic Eczema Reveals Reciprocal Expression of Inflammatory and Lipid Genes	Plos One
Atopic Eczema	PRTN3	Expression profiling by array	GPL7191	GSE12511	Global Expression Profiling in Atopic Eczema Reveals Reciprocal Expression of Inflammatory and Lipid Genes	Plos One
Atopic Eczema	C20ORF4	Expression profiling by array	GPL7191	GSE12511	Global Expression Profiling in Atopic Eczema Reveals Reciprocal Expression of Inflammatory and Lipid Genes	Plos One
Atopic Eczema	GALNACT-2	Expression profiling by array	GPL7191	GSE12511	Global Expression Profiling in Atopic Eczema Reveals Reciprocal Expression of Inflammatory and Lipid Genes	Plos One
Atopic Eczema	MAGEA11	Expression profiling by array	GPL7191	GSE12511	Global Expression Profiling in Atopic Eczema Reveals Reciprocal Expression of Inflammatory and Lipid Genes	Plos One
Atopic Eczema	PPFIA3	Expression profiling by array	GPL7191	GSE12511	Global Expression Profiling in Atopic Eczema Reveals Reciprocal Expression of Inflammatory and Lipid Genes	Plos One
Atopic Eczema	RYK	Expression profiling by array	GPL7191	GSE12511	Global Expression Profiling in Atopic Eczema Reveals Reciprocal Expression of Inflammatory and Lipid Genes	Plos One
Atopic Eczema	SPG20	Expression profiling by array	GPL7191	GSE12511	Global Expression Profiling in Atopic Eczema Reveals Reciprocal Expression of Inflammatory and Lipid Genes	Plos One
Atopic Eczema	CEP70	Expression profiling by array	GPL7191	GSE12511	Global Expression Profiling in Atopic Eczema Reveals Reciprocal Expression of Inflammatory and Lipid Genes	Plos One
Atopic Eczema	PIM2	Expression profiling by array	GPL7191	GSE12511	Global Expression Profiling in Atopic Eczema Reveals Reciprocal Expression of Inflammatory and Lipid Genes	Plos One
Atopic Eczema	NFE2L1	Expression profiling by array	GPL7191	GSE12511	Global Expression Profiling in Atopic Eczema Reveals Reciprocal Expression of Inflammatory and Lipid Genes	Plos One
Atopic Eczema	SPON1	Expression profiling by array	GPL7191	GSE12511	Global Expression Profiling in Atopic Eczema Reveals Reciprocal Expression of Inflammatory and Lipid Genes	Plos One
Atopic Eczema	FLJ12529	Expression profiling by array	GPL7191	GSE12511	Global Expression Profiling in Atopic Eczema Reveals Reciprocal Expression of Inflammatory and Lipid Genes	Plos One
Atopic Eczema	RPS8	Expression profiling by array	GPL7191	GSE12511	Global Expression Profiling in Atopic Eczema Reveals Reciprocal Expression of Inflammatory and Lipid Genes	Plos One
Atopic Eczema	COG8	Expression profiling by array	GPL7191	GSE12511	Global Expression Profiling in Atopic Eczema Reveals Reciprocal Expression of Inflammatory and Lipid Genes	Plos One
Atopic Eczema	RPL11	Expression profiling by array	GPL7191	GSE12511	Global Expression Profiling in Atopic Eczema Reveals Reciprocal Expression of Inflammatory and Lipid Genes	Plos One
Atopic Eczema	EBI2	Expression profiling by array	GPL7191	GSE12511	Global Expression Profiling in Atopic Eczema Reveals Reciprocal Expression of Inflammatory and Lipid Genes	Plos One
Atopic Eczema	MAD2L2	Expression profiling by array	GPL7191	GSE12511	Global Expression Profiling in Atopic Eczema Reveals Reciprocal Expression of Inflammatory and Lipid Genes	Plos One
Atopic Eczema	GNPNAT1	Expression profiling by array	GPL7191	GSE12511	Global Expression Profiling in Atopic Eczema Reveals Reciprocal Expression of Inflammatory and Lipid Genes	Plos One
Atopic Eczema	THNSL1	Expression profiling by array	GPL7191	GSE12511	Global Expression Profiling in Atopic Eczema Reveals Reciprocal Expression of Inflammatory and Lipid Genes	Plos One
Atopic Eczema	USP32	Expression profiling by array	GPL7191	GSE12511	Global Expression Profiling in Atopic Eczema Reveals Reciprocal Expression of Inflammatory and Lipid Genes	Plos One
Atopic Eczema	CTDSPL	Expression profiling by array	GPL7191	GSE12511	Global Expression Profiling in Atopic Eczema Reveals Reciprocal Expression of Inflammatory and Lipid Genes	Plos One
Atopic Eczema	C6ORF129	Expression profiling by array	GPL7191	GSE12511	Global Expression Profiling in Atopic Eczema Reveals Reciprocal Expression of Inflammatory and Lipid Genes	Plos One
Atopic Eczema	EIF3S5	Expression profiling by array	GPL7191	GSE12511	Global Expression Profiling in Atopic Eczema Reveals Reciprocal Expression of Inflammatory and Lipid Genes	Plos One
Atopic Eczema	PWCR1	Expression profiling by array	GPL7191	GSE12511	Global Expression Profiling in Atopic Eczema Reveals Reciprocal Expression of Inflammatory and Lipid Genes	Plos One
Atopic Eczema	FAM116A	Expression profiling by array	GPL7191	GSE12511	Global Expression Profiling in Atopic Eczema Reveals Reciprocal Expression of Inflammatory and Lipid Genes	Plos One
Atopic Eczema	PPP1R1A	Expression profiling by array	GPL7191	GSE12511	Global Expression Profiling in Atopic Eczema Reveals Reciprocal Expression of Inflammatory and Lipid Genes	Plos One
Atopic Eczema	RAF1	Expression profiling by array	GPL7191	GSE12511	Global Expression Profiling in Atopic Eczema Reveals Reciprocal Expression of Inflammatory and Lipid Genes	Plos One
Atopic Eczema	TOMM40L	Expression profiling by array	GPL7191	GSE12511	Global Expression Profiling in Atopic Eczema Reveals Reciprocal Expression of Inflammatory and Lipid Genes	Plos One
Atopic Eczema	LOC126208	Expression profiling by array	GPL7191	GSE12511	Global Expression Profiling in Atopic Eczema Reveals Reciprocal Expression of Inflammatory and Lipid Genes	Plos One
Atopic Eczema	MGC75360	Expression profiling by array	GPL7191	GSE12511	Global Expression Profiling in Atopic Eczema Reveals Reciprocal Expression of Inflammatory and Lipid Genes	Plos One
Atopic Eczema	LILRB3	Expression profiling by array	GPL7191	GSE12511	Global Expression Profiling in Atopic Eczema Reveals Reciprocal Expression of Inflammatory and Lipid Genes	Plos One
Atopic Eczema	ATOH8	Expression profiling by array	GPL7191	GSE12511	Global Expression Profiling in Atopic Eczema Reveals Reciprocal Expression of Inflammatory and Lipid Genes	Plos One
Atopic Eczema	ADSL	Expression profiling by array	GPL7191	GSE12511	Global Expression Profiling in Atopic Eczema Reveals Reciprocal Expression of Inflammatory and Lipid Genes	Plos One
Atopic Eczema	MTM1	Expression profiling by array	GPL7191	GSE12511	Global Expression Profiling in Atopic Eczema Reveals Reciprocal Expression of Inflammatory and Lipid Genes	Plos One
Atopic Eczema	TRDN	Expression profiling by array	GPL7191	GSE12511	Global Expression Profiling in Atopic Eczema Reveals Reciprocal Expression of Inflammatory and Lipid Genes	Plos One
Atopic Eczema	ATG9A	Expression profiling by array	GPL7191	GSE12511	Global Expression Profiling in Atopic Eczema Reveals Reciprocal Expression of Inflammatory and Lipid Genes	Plos One

Atopic Eczema	PPP1R9A	Expression profiling by array	GPL7191	GSE12511	Global Expression Profiling in Atopic Eczema Reveals Reciprocal Expression of Inflammatory and Lipid Genes	Plos One
Atopic Eczema	ERO1LB	Expression profiling by array	GPL7191	GSE12511	Global Expression Profiling in Atopic Eczema Reveals Reciprocal Expression of Inflammatory and Lipid Genes	Plos One
Atopic Eczema	KRR1	Expression profiling by array	GPL7191	GSE12511	Global Expression Profiling in Atopic Eczema Reveals Reciprocal Expression of Inflammatory and Lipid Genes	Plos One
Atopic Eczema	AHSA1	Expression profiling by array	GPL7191	GSE12511	Global Expression Profiling in Atopic Eczema Reveals Reciprocal Expression of Inflammatory and Lipid Genes	Plos One
Atopic Eczema	CXORF52	Expression profiling by array	GPL7191	GSE12511	Global Expression Profiling in Atopic Eczema Reveals Reciprocal Expression of Inflammatory and Lipid Genes	Plos One
Atopic Eczema	IGFBP5	Expression profiling by array	GPL7191	GSE12511	Global Expression Profiling in Atopic Eczema Reveals Reciprocal Expression of Inflammatory and Lipid Genes	Plos One
Atopic Eczema	CXCR6	Expression profiling by array	GPL7191	GSE12511	Global Expression Profiling in Atopic Eczema Reveals Reciprocal Expression of Inflammatory and Lipid Genes	Plos One
Atopic Eczema	TSEN2	Expression profiling by array	GPL7191	GSE12511	Global Expression Profiling in Atopic Eczema Reveals Reciprocal Expression of Inflammatory and Lipid Genes	Plos One
Atopic Eczema	POMP	Expression profiling by array	GPL7191	GSE12511	Global Expression Profiling in Atopic Eczema Reveals Reciprocal Expression of Inflammatory and Lipid Genes	Plos One
Atopic Eczema	DECR1	Expression profiling by array	GPL7191	GSE12511	Global Expression Profiling in Atopic Eczema Reveals Reciprocal Expression of Inflammatory and Lipid Genes	Plos One
Atopic Eczema	DOCK6	Expression profiling by array	GPL7191	GSE12511	Global Expression Profiling in Atopic Eczema Reveals Reciprocal Expression of Inflammatory and Lipid Genes	Plos One
Atopic Eczema	PPP2R1B	Expression profiling by array	GPL7191	GSE12511	Global Expression Profiling in Atopic Eczema Reveals Reciprocal Expression of Inflammatory and Lipid Genes	Plos One
Atopic Eczema	TM9SF1	Expression profiling by array	GPL7191	GSE12511	Global Expression Profiling in Atopic Eczema Reveals Reciprocal Expression of Inflammatory and Lipid Genes	Plos One
Atopic Eczema	EIF3S12	Expression profiling by array	GPL7191	GSE12511	Global Expression Profiling in Atopic Eczema Reveals Reciprocal Expression of Inflammatory and Lipid Genes	Plos One
Atopic Eczema	RBBP6	Expression profiling by array	GPL7191	GSE12511	Global Expression Profiling in Atopic Eczema Reveals Reciprocal Expression of Inflammatory and Lipid Genes	Plos One
Atopic Eczema	RAB2	Expression profiling by array	GPL7191	GSE12511	Global Expression Profiling in Atopic Eczema Reveals Reciprocal Expression of Inflammatory and Lipid Genes	Plos One
Atopic Eczema	EPHA1	Expression profiling by array	GPL7191	GSE12511	Global Expression Profiling in Atopic Eczema Reveals Reciprocal Expression of Inflammatory and Lipid Genes	Plos One
Atopic Eczema	CARM1	Expression profiling by array	GPL7191	GSE12511	Global Expression Profiling in Atopic Eczema Reveals Reciprocal Expression of Inflammatory and Lipid Genes	Plos One
Atopic Eczema	MGC11271	Expression profiling by array	GPL7191	GSE12511	Global Expression Profiling in Atopic Eczema Reveals Reciprocal Expression of Inflammatory and Lipid Genes	Plos One
Atopic Eczema	MALL	Expression profiling by array	GPL7191	GSE12511	Global Expression Profiling in Atopic Eczema Reveals Reciprocal Expression of Inflammatory and Lipid Genes	Plos One
Atopic Eczema	SRPK2	Expression profiling by array	GPL7191	GSE12511	Global Expression Profiling in Atopic Eczema Reveals Reciprocal Expression of Inflammatory and Lipid Genes	Plos One
Atopic Eczema	CHI3L1	Expression profiling by array	GPL7191	GSE12511	Global Expression Profiling in Atopic Eczema Reveals Reciprocal Expression of Inflammatory and Lipid Genes	Plos One
Atopic Eczema	CART1	Expression profiling by array	GPL7191	GSE12511	Global Expression Profiling in Atopic Eczema Reveals Reciprocal Expression of Inflammatory and Lipid Genes	Plos One
Atopic Eczema	TTC8	Expression profiling by array	GPL7191	GSE12511	Global Expression Profiling in Atopic Eczema Reveals Reciprocal Expression of Inflammatory and Lipid Genes	Plos One
Atopic Eczema	N4BP1	Expression profiling by array	GPL7191	GSE12511	Global Expression Profiling in Atopic Eczema Reveals Reciprocal Expression of Inflammatory and Lipid Genes	Plos One
Atopic Eczema	GAS2L3	Expression profiling by array	GPL7191	GSE12511	Global Expression Profiling in Atopic Eczema Reveals Reciprocal Expression of Inflammatory and Lipid Genes	Plos One
Atopic Eczema	BTBD15	Expression profiling by array	GPL7191	GSE12511	Global Expression Profiling in Atopic Eczema Reveals Reciprocal Expression of Inflammatory and Lipid Genes	Plos One
Atopic Eczema	AMDHD2	Expression profiling by array	GPL7191	GSE12511	Global Expression Profiling in Atopic Eczema Reveals Reciprocal Expression of Inflammatory and Lipid Genes	Plos One
Atopic Eczema	NXF2	Expression profiling by array	GPL7191	GSE12511	Global Expression Profiling in Atopic Eczema Reveals Reciprocal Expression of Inflammatory and Lipid Genes	Plos One
Atopic Eczema	MGC2463	Expression profiling by array	GPL7191	GSE12511	Global Expression Profiling in Atopic Eczema Reveals Reciprocal Expression of Inflammatory and Lipid Genes	Plos One
Atopic Eczema	TMEM115	Expression profiling by array	GPL7191	GSE12511	Global Expression Profiling in Atopic Eczema Reveals Reciprocal Expression of Inflammatory and Lipid Genes	Plos One
Atopic Eczema	LOC388963	Expression profiling by array	GPL7191	GSE12511	Global Expression Profiling in Atopic Eczema Reveals Reciprocal Expression of Inflammatory and Lipid Genes	Plos One
Atopic Eczema	CIZ1	Expression profiling by array	GPL7191	GSE12511	Global Expression Profiling in Atopic Eczema Reveals Reciprocal Expression of Inflammatory and Lipid Genes	Plos One
Atopic Eczema	C19ORF24	Expression profiling by array	GPL7191	GSE12511	Global Expression Profiling in Atopic Eczema Reveals Reciprocal Expression of Inflammatory and Lipid Genes	Plos One
Atopic Eczema	ZNF577	Expression profiling by array	GPL7191	GSE12511	Global Expression Profiling in Atopic Eczema Reveals Reciprocal Expression of Inflammatory and Lipid Genes	Plos One
Atopic Eczema	RIN2	Expression profiling by array	GPL7191	GSE12511	Global Expression Profiling in Atopic Eczema Reveals Reciprocal Expression of Inflammatory and Lipid Genes	Plos One
Atopic Eczema	MX1	Expression profiling by array	GPL7191	GSE12511	Global Expression Profiling in Atopic Eczema Reveals Reciprocal Expression of Inflammatory and Lipid Genes	Plos One
Atopic Eczema	GFM2	Expression profiling by array	GPL7191	GSE12511	Global Expression Profiling in Atopic Eczema Reveals Reciprocal Expression of Inflammatory and Lipid Genes	Plos One
Atopic Eczema	RAB22A	Expression profiling by array	GPL7191	GSE12511	Global Expression Profiling in Atopic Eczema Reveals Reciprocal Expression of Inflammatory and Lipid Genes	Plos One
Atopic Eczema	SLC36A1	Expression profiling by array	GPL7191	GSE12511	Global Expression Profiling in Atopic Eczema Reveals Reciprocal Expression of Inflammatory and Lipid Genes	Plos One
Atopic Eczema	ATRX	Expression profiling by array	GPL7191	GSE12511	Global Expression Profiling in Atopic Eczema Reveals Reciprocal Expression of Inflammatory and Lipid Genes	Plos One
Atopic Eczema	UGT2B7	Expression profiling by array	GPL7191	GSE12511	Global Expression Profiling in Atopic Eczema Reveals Reciprocal Expression of Inflammatory and Lipid Genes	Plos One
Atopic Eczema	WNK1	Expression profiling by array	GPL7191	GSE12511	Global Expression Profiling in Atopic Eczema Reveals Reciprocal Expression of Inflammatory and Lipid Genes	Plos One
Atopic Eczema	LOC375748	Expression profiling by array	GPL7191	GSE12511	Global Expression Profiling in Atopic Eczema Reveals Reciprocal Expression of Inflammatory and Lipid Genes	Plos One
Atopic Eczema	C12ORF23	Expression profiling by array	GPL7191	GSE12511	Global Expression Profiling in Atopic Eczema Reveals Reciprocal Expression of Inflammatory and Lipid Genes	Plos One
Atopic Eczema	TRAF2	Expression profiling by array	GPL7191	GSE12511	Global Expression Profiling in Atopic Eczema Reveals Reciprocal Expression of Inflammatory and Lipid Genes	Plos One
Atopic Eczema	TTC6	Expression profiling by array	GPL7191	GSE12511	Global Expression Profiling in Atopic Eczema Reveals Reciprocal Expression of Inflammatory and Lipid Genes	Plos One
Atopic Eczema	DNAH5	Expression profiling by array	GPL7191	GSE12511	Global Expression Profiling in Atopic Eczema Reveals Reciprocal Expression of Inflammatory and Lipid Genes	Plos One
Atopic Eczema	FDX1	Expression profiling by array	GPL7191	GSE12511	Global Expression Profiling in Atopic Eczema Reveals Reciprocal Expression of Inflammatory and Lipid Genes	Plos One
Atopic Eczema	RAPGEF2	Expression profiling by array	GPL7191	GSE12511	Global Expression Profiling in Atopic Eczema Reveals Reciprocal Expression of Inflammatory and Lipid Genes	Plos One
Atopic Eczema	CDON	Expression profiling by array	GPL7191	GSE12511	Global Expression Profiling in Atopic Eczema Reveals Reciprocal Expression of Inflammatory and Lipid Genes	Plos One
Atopic Eczema	C4ORF29	Expression profiling by array	GPL7191	GSE12511	Global Expression Profiling in Atopic Eczema Reveals Reciprocal Expression of Inflammatory and Lipid Genes	Plos One
Atopic Eczema	MOBKL2C	Expression profiling by array	GPL7191	GSE12511	Global Expression Profiling in Atopic Eczema Reveals Reciprocal Expression of Inflammatory and Lipid Genes	Plos One
Atopic Eczema	SLC7A13	Expression profiling by array	GPL7191	GSE12511	Global Expression Profiling in Atopic Eczema Reveals Reciprocal Expression of Inflammatory and Lipid Genes	Plos One
Atopic Eczema	SIAH1	Expression profiling by array	GPL7191	GSE12511	Global Expression Profiling in Atopic Eczema Reveals Reciprocal Expression of Inflammatory and Lipid Genes	Plos One
Atopic Eczema	VPS13A	Expression profiling by array	GPL7191	GSE12511	Global Expression Profiling in Atopic Eczema Reveals Reciprocal Expression of Inflammatory and Lipid Genes	Plos One
Atopic Eczema	KPNA3	Expression profiling by array	GPL7191	GSE12511	Global Expression Profiling in Atopic Eczema Reveals Reciprocal Expression of Inflammatory and Lipid Genes	Plos One
Atopic Eczema	PAPSS2	Expression profiling by array	GPL7191	GSE12511	Global Expression Profiling in Atopic Eczema Reveals Reciprocal Expression of Inflammatory and Lipid Genes	Plos One
Atopic Eczema	CCNH	Expression profiling by array	GPL7191	GSE12511	Global Expression Profiling in Atopic Eczema Reveals Reciprocal Expression of Inflammatory and Lipid Genes	Plos One
Atopic Eczema	RNF7	Expression profiling by array	GPL7191	GSE12511	Global Expression Profiling in Atopic Eczema Reveals Reciprocal Expression of Inflammatory and Lipid Genes	Plos One
Atopic Eczema	PLXNB3	Expression profiling by array	GPL7191	GSE12511	Global Expression Profiling in Atopic Eczema Reveals Reciprocal Expression of Inflammatory and Lipid Genes	Plos One
Atopic Eczema	ALKBH7	Expression profiling by array	GPL7191	GSE12511	Global Expression Profiling in Atopic Eczema Reveals Reciprocal Expression of Inflammatory and Lipid Genes	Plos One

Atopic Eczema	AEBP2	Expression profiling by array	GPL7191	GSE12511	Global Expression Profiling in Atopic Eczema Reveals Reciprocal Expression of Inflammatory and Lipid Genes	Plos One
Atopic Eczema	RAP1A	Expression profiling by array	GPL7191	GSE12511	Global Expression Profiling in Atopic Eczema Reveals Reciprocal Expression of Inflammatory and Lipid Genes	Plos One
Atopic Eczema	S100A13	Expression profiling by array	GPL7191	GSE12511	Global Expression Profiling in Atopic Eczema Reveals Reciprocal Expression of Inflammatory and Lipid Genes	Plos One
Atopic Eczema	EVI1	Expression profiling by array	GPL7191	GSE12511	Global Expression Profiling in Atopic Eczema Reveals Reciprocal Expression of Inflammatory and Lipid Genes	Plos One
Atopic Eczema	YWHAB	Expression profiling by array	GPL7191	GSE12511	Global Expression Profiling in Atopic Eczema Reveals Reciprocal Expression of Inflammatory and Lipid Genes	Plos One
Atopic Eczema	LOC150223	Expression profiling by array	GPL7191	GSE12511	Global Expression Profiling in Atopic Eczema Reveals Reciprocal Expression of Inflammatory and Lipid Genes	Plos One
Atopic Eczema	RPP25	Expression profiling by array	GPL7191	GSE12511	Global Expression Profiling in Atopic Eczema Reveals Reciprocal Expression of Inflammatory and Lipid Genes	Plos One
Atopic Eczema	CTNNA1	Expression profiling by array	GPL7191	GSE12511	Global Expression Profiling in Atopic Eczema Reveals Reciprocal Expression of Inflammatory and Lipid Genes	Plos One
Atopic Eczema	ATP6V0A2	Expression profiling by array	GPL7191	GSE12511	Global Expression Profiling in Atopic Eczema Reveals Reciprocal Expression of Inflammatory and Lipid Genes	Plos One
Atopic Eczema	COL4A3BP	Expression profiling by array	GPL7191	GSE12511	Global Expression Profiling in Atopic Eczema Reveals Reciprocal Expression of Inflammatory and Lipid Genes	Plos One
Atopic Eczema	DLD	Expression profiling by array	GPL7191	GSE12511	Global Expression Profiling in Atopic Eczema Reveals Reciprocal Expression of Inflammatory and Lipid Genes	Plos One
Atopic Eczema	EXOC4	Expression profiling by array	GPL7191	GSE12511	Global Expression Profiling in Atopic Eczema Reveals Reciprocal Expression of Inflammatory and Lipid Genes	Plos One
Atopic Eczema	RTKN	Expression profiling by array	GPL7191	GSE12511	Global Expression Profiling in Atopic Eczema Reveals Reciprocal Expression of Inflammatory and Lipid Genes	Plos One
Atopic Eczema	RPP38	Expression profiling by array	GPL7191	GSE12511	Global Expression Profiling in Atopic Eczema Reveals Reciprocal Expression of Inflammatory and Lipid Genes	Plos One
Atopic Eczema	SURF2	Expression profiling by array	GPL7191	GSE12511	Global Expression Profiling in Atopic Eczema Reveals Reciprocal Expression of Inflammatory and Lipid Genes	Plos One
Atopic Eczema	KPNA2	Expression profiling by array	GPL7191	GSE12511	Global Expression Profiling in Atopic Eczema Reveals Reciprocal Expression of Inflammatory and Lipid Genes	Plos One
Atopic Eczema	CERK	Expression profiling by array	GPL7191	GSE12511	Global Expression Profiling in Atopic Eczema Reveals Reciprocal Expression of Inflammatory and Lipid Genes	Plos One
Atopic Eczema	TPD52L2	Expression profiling by array	GPL7191	GSE12511	Global Expression Profiling in Atopic Eczema Reveals Reciprocal Expression of Inflammatory and Lipid Genes	Plos One
Atopic Eczema	CH25H	Expression profiling by array	GPL7191	GSE12511	Global Expression Profiling in Atopic Eczema Reveals Reciprocal Expression of Inflammatory and Lipid Genes	Plos One
Atopic Eczema	PI3	Expression profiling by array	GPL7191	GSE12511	Global Expression Profiling in Atopic Eczema Reveals Reciprocal Expression of Inflammatory and Lipid Genes	Plos One
Atopic Eczema	RBM6	Expression profiling by array	GPL7191	GSE12511	Global Expression Profiling in Atopic Eczema Reveals Reciprocal Expression of Inflammatory and Lipid Genes	Plos One
Atopic Eczema	WBP5	Expression profiling by array	GPL7191	GSE12511	Global Expression Profiling in Atopic Eczema Reveals Reciprocal Expression of Inflammatory and Lipid Genes	Plos One
Atopic Eczema	DCUN1D1	Expression profiling by array	GPL7191	GSE12511	Global Expression Profiling in Atopic Eczema Reveals Reciprocal Expression of Inflammatory and Lipid Genes	Plos One
Atopic Eczema	PHYHIP	Expression profiling by array	GPL7191	GSE12511	Global Expression Profiling in Atopic Eczema Reveals Reciprocal Expression of Inflammatory and Lipid Genes	Plos One
Atopic Eczema	MCF2L	Expression profiling by array	GPL7191	GSE12511	Global Expression Profiling in Atopic Eczema Reveals Reciprocal Expression of Inflammatory and Lipid Genes	Plos One
Atopic Eczema	GZMK	Expression profiling by array	GPL7191	GSE12511	Global Expression Profiling in Atopic Eczema Reveals Reciprocal Expression of Inflammatory and Lipid Genes	Plos One
Atopic Eczema	TMEM64	Expression profiling by array	GPL7191	GSE12511	Global Expression Profiling in Atopic Eczema Reveals Reciprocal Expression of Inflammatory and Lipid Genes	Plos One
Atopic Eczema	CENPA	Expression profiling by array	GPL7191	GSE12511	Global Expression Profiling in Atopic Eczema Reveals Reciprocal Expression of Inflammatory and Lipid Genes	Plos One
Atopic Eczema	DIRC2	Expression profiling by array	GPL7191	GSE12511	Global Expression Profiling in Atopic Eczema Reveals Reciprocal Expression of Inflammatory and Lipid Genes	Plos One
Atopic Eczema	GAB2	Expression profiling by array	GPL7191	GSE12511	Global Expression Profiling in Atopic Eczema Reveals Reciprocal Expression of Inflammatory and Lipid Genes	Plos One
Atopic Eczema	TNCRNA	Expression profiling by array	GPL7191	GSE12511	Global Expression Profiling in Atopic Eczema Reveals Reciprocal Expression of Inflammatory and Lipid Genes	Plos One
Atopic Eczema	B2M	Expression profiling by array	GPL7191	GSE12511	Global Expression Profiling in Atopic Eczema Reveals Reciprocal Expression of Inflammatory and Lipid Genes	Plos One
Atopic Eczema	ATG7	Expression profiling by array	GPL7191	GSE12511	Global Expression Profiling in Atopic Eczema Reveals Reciprocal Expression of Inflammatory and Lipid Genes	Plos One
Atopic Eczema	C4ORF21	Expression profiling by array	GPL7191	GSE12511	Global Expression Profiling in Atopic Eczema Reveals Reciprocal Expression of Inflammatory and Lipid Genes	Plos One
Atopic Eczema	PIGO	Expression profiling by array	GPL7191	GSE12511	Global Expression Profiling in Atopic Eczema Reveals Reciprocal Expression of Inflammatory and Lipid Genes	Plos One
Atopic Eczema	KIAA0376	Expression profiling by array	GPL7191	GSE12511	Global Expression Profiling in Atopic Eczema Reveals Reciprocal Expression of Inflammatory and Lipid Genes	Plos One
Atopic Eczema	FXYD3	Expression profiling by array	GPL7191	GSE12511	Global Expression Profiling in Atopic Eczema Reveals Reciprocal Expression of Inflammatory and Lipid Genes	Plos One
Atopic Eczema	PGK1	Expression profiling by array	GPL7191	GSE12511	Global Expression Profiling in Atopic Eczema Reveals Reciprocal Expression of Inflammatory and Lipid Genes	Plos One
Atopic Eczema	SFXN1	Expression profiling by array	GPL7191	GSE12511	Global Expression Profiling in Atopic Eczema Reveals Reciprocal Expression of Inflammatory and Lipid Genes	Plos One
Atopic Eczema	RBM4B	Expression profiling by array	GPL7191	GSE12511	Global Expression Profiling in Atopic Eczema Reveals Reciprocal Expression of Inflammatory and Lipid Genes	Plos One
Atopic Eczema	ARL6	Expression profiling by array	GPL7191	GSE12511	Global Expression Profiling in Atopic Eczema Reveals Reciprocal Expression of Inflammatory and Lipid Genes	Plos One
Atopic Eczema	C1ORF117	Expression profiling by array	GPL7191	GSE12511	Global Expression Profiling in Atopic Eczema Reveals Reciprocal Expression of Inflammatory and Lipid Genes	Plos One
Atopic Eczema	TPCN2	Expression profiling by array	GPL7191	GSE12511	Global Expression Profiling in Atopic Eczema Reveals Reciprocal Expression of Inflammatory and Lipid Genes	Plos One
Atopic Eczema	INPP5E	Expression profiling by array	GPL7191	GSE12511	Global Expression Profiling in Atopic Eczema Reveals Reciprocal Expression of Inflammatory and Lipid Genes	Plos One
Atopic Eczema	SYNCRIP	Expression profiling by array	GPL7191	GSE12511	Global Expression Profiling in Atopic Eczema Reveals Reciprocal Expression of Inflammatory and Lipid Genes	Plos One
Atopic Eczema	C6	Expression profiling by array	GPL7191	GSE12511	Global Expression Profiling in Atopic Eczema Reveals Reciprocal Expression of Inflammatory and Lipid Genes	Plos One
Atopic Eczema	EMP2	Expression profiling by array	GPL7191	GSE12511	Global Expression Profiling in Atopic Eczema Reveals Reciprocal Expression of Inflammatory and Lipid Genes	Plos One
Atopic Eczema	DENND1A	Expression profiling by array	GPL7191	GSE12511	Global Expression Profiling in Atopic Eczema Reveals Reciprocal Expression of Inflammatory and Lipid Genes	Plos One
Atopic Eczema	ZCCHC11	Expression profiling by array	GPL7191	GSE12511	Global Expression Profiling in Atopic Eczema Reveals Reciprocal Expression of Inflammatory and Lipid Genes	Plos One
Atopic Eczema	NAT1	Expression profiling by array	GPL7191	GSE12511	Global Expression Profiling in Atopic Eczema Reveals Reciprocal Expression of Inflammatory and Lipid Genes	Plos One
Atopic Eczema	C14ORF102	Expression profiling by array	GPL7191	GSE12511	Global Expression Profiling in Atopic Eczema Reveals Reciprocal Expression of Inflammatory and Lipid Genes	Plos One
Atopic Eczema	KRTCAP2	Expression profiling by array	GPL7191	GSE12511	Global Expression Profiling in Atopic Eczema Reveals Reciprocal Expression of Inflammatory and Lipid Genes	Plos One
Atopic Eczema	C9ORF114	Expression profiling by array	GPL7191	GSE12511	Global Expression Profiling in Atopic Eczema Reveals Reciprocal Expression of Inflammatory and Lipid Genes	Plos One
Atopic Eczema	EHBP1	Expression profiling by array	GPL7191	GSE12511	Global Expression Profiling in Atopic Eczema Reveals Reciprocal Expression of Inflammatory and Lipid Genes	Plos One
Atopic Eczema	XIST	Expression profiling by array	GPL7191	GSE12511	Global Expression Profiling in Atopic Eczema Reveals Reciprocal Expression of Inflammatory and Lipid Genes	Plos One
Atopic Eczema	ZNF284	Expression profiling by array	GPL7191	GSE12511	Global Expression Profiling in Atopic Eczema Reveals Reciprocal Expression of Inflammatory and Lipid Genes	Plos One
Atopic Eczema	HMGCR	Expression profiling by array	GPL7191	GSE12511	Global Expression Profiling in Atopic Eczema Reveals Reciprocal Expression of Inflammatory and Lipid Genes	Plos One
Atopic Eczema	ESPL1	Expression profiling by array	GPL7191	GSE12511	Global Expression Profiling in Atopic Eczema Reveals Reciprocal Expression of Inflammatory and Lipid Genes	Plos One
Atopic Eczema	TIMM44	Expression profiling by array	GPL7191	GSE12511	Global Expression Profiling in Atopic Eczema Reveals Reciprocal Expression of Inflammatory and Lipid Genes	Plos One
Atopic Eczema	ITGA2	Expression profiling by array	GPL7191	GSE12511	Global Expression Profiling in Atopic Eczema Reveals Reciprocal Expression of Inflammatory and Lipid Genes	Plos One
Atopic Eczema	PCQAP	Expression profiling by array	GPL7191	GSE12511	Global Expression Profiling in Atopic Eczema Reveals Reciprocal Expression of Inflammatory and Lipid Genes	Plos One
Atopic Eczema	FAM120B	Expression profiling by array	GPL7191	GSE12511	Global Expression Profiling in Atopic Eczema Reveals Reciprocal Expression of Inflammatory and Lipid Genes	Plos One

Atopic Eczema	PHF13	Expression profiling by array	GPL7191	GSE12511	Global Expression Profiling in Atopic Eczema Reveals Reciprocal Expression of Inflammatory and Lipid Genes	Plos One
Atopic Eczema	CEP152	Expression profiling by array	GPL7191	GSE12511	Global Expression Profiling in Atopic Eczema Reveals Reciprocal Expression of Inflammatory and Lipid Genes	Plos One
Atopic Eczema	LOC150159	Expression profiling by array	GPL7191	GSE12511	Global Expression Profiling in Atopic Eczema Reveals Reciprocal Expression of Inflammatory and Lipid Genes	Plos One
Atopic Eczema	RNF40	Expression profiling by array	GPL7191	GSE12511	Global Expression Profiling in Atopic Eczema Reveals Reciprocal Expression of Inflammatory and Lipid Genes	Plos One
Atopic Eczema	RBM7	Expression profiling by array	GPL7191	GSE12511	Global Expression Profiling in Atopic Eczema Reveals Reciprocal Expression of Inflammatory and Lipid Genes	Plos One
Atopic Eczema	SAMD1	Expression profiling by array	GPL7191	GSE12511	Global Expression Profiling in Atopic Eczema Reveals Reciprocal Expression of Inflammatory and Lipid Genes	Plos One
Atopic Eczema	YPEL2	Expression profiling by array	GPL7191	GSE12511	Global Expression Profiling in Atopic Eczema Reveals Reciprocal Expression of Inflammatory and Lipid Genes	Plos One
Eczema [allergic contact dermatitis	S100A7L2	Expression profiling by array	GPL14550	GSE57225	Intra-individual genome expression analysis reveals a specific molecular signature of psoriasis and eczema / Science Translational Medicine / 2014	Science Translational Medicine
Eczema [allergic contact dermatitis	LCN2	Expression profiling by array	GPL14550	GSE57225	Intra-individual genome expression analysis reveals a specific molecular signature of psoriasis and eczema / Science Translational Medicine / 2014	Science Translational Medicine
Eczema [allergic contact dermatitis	HRNR	Expression profiling by array	GPL14550	GSE57225	Intra-individual genome expression analysis reveals a specific molecular signature of psoriasis and eczema / Science Translational Medicine / 2014	Science Translational Medicine
Eczema [allergic contact dermatitis	LCE3C	Expression profiling by array	GPL14550	GSE57225	Intra-individual genome expression analysis reveals a specific molecular signature of psoriasis and eczema / Science Translational Medicine / 2014	Science Translational Medicine
Eczema [allergic contact dermatitis	SPRR1A	Expression profiling by array	GPL14550	GSE57225	Intra-individual genome expression analysis reveals a specific molecular signature of psoriasis and eczema / Science Translational Medicine / 2014	Science Translational Medicine
Eczema [allergic contact dermatitis	SPRR1B	Expression profiling by array	GPL14550	GSE57225	Intra-individual genome expression analysis reveals a specific molecular signature of psoriasis and eczema / Science Translational Medicine / 2014	Science Translational Medicine
Eczema [allergic contact dermatitis	SPRR2A	Expression profiling by array	GPL14550	GSE57225	Intra-individual genome expression analysis reveals a specific molecular signature of psoriasis and eczema / Science Translational Medicine / 2014	Science Translational Medicine
Eczema [allergic contact dermatitis	SPRR2C	Expression profiling by array	GPL14550	GSE57225	Intra-individual genome expression analysis reveals a specific molecular signature of psoriasis and eczema / Science Translational Medicine / 2014	Science Translational Medicine
Eczema [allergic contact dermatitis	SPRR2D	Expression profiling by array	GPL14550	GSE57225	Intra-individual genome expression analysis reveals a specific molecular signature of psoriasis and eczema / Science Translational Medicine / 2014	Science Translational Medicine
Eczema [allergic contact dermatitis	SPRR3	Expression profiling by array	GPL14550	GSE57225	Intra-individual genome expression analysis reveals a specific molecular signature of psoriasis and eczema / Science Translational Medicine / 2014	Science Translational Medicine
Eczema [allergic contact dermatitis	KRT6B	Expression profiling by array	GPL14550	GSE57225	Intra-individual genome expression analysis reveals a specific molecular signature of psoriasis and eczema / Science Translational Medicine / 2014	Science Translational Medicine
Eczema [allergic contact dermatitis	KRT6C	Expression profiling by array	GPL14550	GSE57225	Intra-individual genome expression analysis reveals a specific molecular signature of psoriasis and eczema / Science Translational Medicine / 2014	Science Translational Medicine
Eczema [allergic contact dermatitis	KRT75	Expression profiling by array	GPL14550	GSE57225	Intra-individual genome expression analysis reveals a specific molecular signature of psoriasis and eczema / Science Translational Medicine / 2014	Science Translational Medicine
Eczema [allergic contact dermatitis	GJB2	Expression profiling by array	GPL14550	GSE57225	Intra-individual genome expression analysis reveals a specific molecular signature of psoriasis and eczema / Science Translational Medicine / 2014	Science Translational Medicine
Eczema [allergic contact dermatitis	GJB6	Expression profiling by array	GPL14550	GSE57225	Intra-individual genome expression analysis reveals a specific molecular signature of psoriasis and eczema / Science Translational Medicine / 2014	Science Translational Medicine
Eczema [allergic contact dermatitis	EPGN	Expression profiling by array	GPL14550	GSE57225	Intra-individual genome expression analysis reveals a specific molecular signature of psoriasis and eczema / Science Translational Medicine / 2014	Science Translational Medicine
Eczema [allergic contact dermatitis	CNFN	Expression profiling by array	GPL14550	GSE57225	Intra-individual genome expression analysis reveals a specific molecular signature of psoriasis and eczema / Science Translational Medicine / 2014	Science Translational Medicine
Eczema [allergic contact dermatitis	FOXE1	Expression profiling by array	GPL14550	GSE57225	Intra-individual genome expression analysis reveals a specific molecular signature of psoriasis and eczema / Science Translational Medicine / 2014	Science Translational Medicine
Eczema [allergic contact dermatitis	GJB4	Expression profiling by array	GPL14550	GSE57225	Intra-individual genome expression analysis reveals a specific molecular signature of psoriasis and eczema / Science Translational Medicine / 2014	Science Translational Medicine
Eczema [allergic contact dermatitis	KRT2	Expression profiling by array	GPL14550	GSE57225	Intra-individual genome expression analysis reveals a specific molecular signature of psoriasis and eczema / Science Translational Medicine / 2014	Science Translational Medicine
Eczema [allergic contact dermatitis	KRT19	Expression profiling by array	GPL14550	GSE57225	Intra-individual genome expression analysis reveals a specific molecular signature of psoriasis and eczema / Science Translational Medicine / 2014	Science Translational Medicine
Eczema [allergic contact dermatitis	KRTAP19-1	Expression profiling by array	GPL14550	GSE57225	Intra-individual genome expression analysis reveals a specific molecular signature of psoriasis and eczema / Science Translational Medicine / 2014	Science Translational Medicine
Eczema [allergic contact dermatitis	PLIN1	Expression profiling by array	GPL14550	GSE57225	Intra-individual genome expression analysis reveals a specific molecular signature of psoriasis and eczema / Science Translational Medicine / 2014	Science Translational Medicine
Eczema [allergic contact dermatitis	S100A7	Expression profiling by array	GPL14550	GSE57225	Intra-individual genome expression analysis reveals a specific molecular signature of psoriasis and eczema / Science Translational Medicine / 2014	Science Translational Medicine
Eczema [allergic contact dermatitis	S100A7A	Expression profiling by array	GPL14550	GSE57225	Intra-individual genome expression analysis reveals a specific molecular signature of psoriasis and eczema / Science Translational Medicine / 2014	Science Translational Medicine
Eczema [allergic contact dermatitis	S100A8	Expression profiling by array	GPL14550	GSE57225	Intra-individual genome expression analysis reveals a specific molecular signature of psoriasis and eczema / Science Translational Medicine / 2014	Science Translational Medicine
Eczema [allergic contact dermatitis	S100A9	Expression profiling by array	GPL14550	GSE57225	Intra-individual genome expression analysis reveals a specific molecular signature of psoriasis and eczema / Science Translational Medicine / 2014	Science Translational Medicine

Eczema [allergic contact dermatitis	S100A12	Expression profiling by array	GPL14550	GSE57225	Intra-individual genome expression analysis reveals a specific molecular signature of psoriasis and eczema / Science Translational Medicine / 2014	Science Translational Medicine
Eczema [allergic contact dermatitis	DEFB4A	Expression profiling by array	GPL14550	GSE57225	Intra-individual genome expression analysis reveals a specific molecular signature of psoriasis and eczema / Science Translational Medicine / 2014	Science Translational Medicine
Eczema [allergic contact dermatitis	DEFB103B	Expression profiling by array	GPL14550	GSE57225	Intra-individual genome expression analysis reveals a specific molecular signature of psoriasis and eczema / Science Translational Medicine / 2014	Science Translational Medicine
Eczema [allergic contact dermatitis	LCE3A	Expression profiling by array	GPL14550	GSE57225	Intra-individual genome expression analysis reveals a specific molecular signature of psoriasis and eczema / Science Translational Medicine / 2014	Science Translational Medicine
Eczema [allergic contact dermatitis	LCE3D	Expression profiling by array	GPL14550	GSE57225	Intra-individual genome expression analysis reveals a specific molecular signature of psoriasis and eczema / Science Translational Medicine / 2014	Science Translational Medicine
Eczema [allergic contact dermatitis	LCE3E	Expression profiling by array	GPL14550	GSE57225	Intra-individual genome expression analysis reveals a specific molecular signature of psoriasis and eczema / Science Translational Medicine / 2014	Science Translational Medicine
Eczema [allergic contact dermatitis	SPRR2B	Expression profiling by array	GPL14550	GSE57225	Intra-individual genome expression analysis reveals a specific molecular signature of psoriasis and eczema / Science Translational Medicine / 2014	Science Translational Medicine
Eczema [allergic contact dermatitis	SPRR2F	Expression profiling by array	GPL14550	GSE57225	Intra-individual genome expression analysis reveals a specific molecular signature of psoriasis and eczema / Science Translational Medicine / 2014	Science Translational Medicine
Eczema [allergic contact dermatitis	KRT6A	Expression profiling by array	GPL14550	GSE57225	Intra-individual genome expression analysis reveals a specific molecular signature of psoriasis and eczema / Science Translational Medicine / 2014	Science Translational Medicine
Eczema [allergic contact dermatitis	KRT16	Expression profiling by array	GPL14550	GSE57225	Intra-individual genome expression analysis reveals a specific molecular signature of psoriasis and eczema / Science Translational Medicine / 2014	Science Translational Medicine
Eczema [allergic contact dermatitis	LCE5A	Expression profiling by array	GPL14550	GSE57225	Intra-individual genome expression analysis reveals a specific molecular signature of psoriasis and eczema / Science Translational Medicine / 2014	Science Translational Medicine
Eczema [allergic contact dermatitis	KRT77	Expression profiling by array	GPL14550	GSE57225	Intra-individual genome expression analysis reveals a specific molecular signature of psoriasis and eczema / Science Translational Medicine / 2014	Science Translational Medicine
Eczema [allergic contact dermatitis	FLG2	Expression profiling by array	GPL14550	GSE57225	Intra-individual genome expression analysis reveals a specific molecular signature of psoriasis and eczema / Science Translational Medicine / 2014	Science Translational Medicine
Eczema [allergic contact dermatitis	BTC	Expression profiling by array	GPL14550	GSE57225	Intra-individual genome expression analysis reveals a specific molecular signature of psoriasis and eczema / Science Translational Medicine / 2014	Science Translational Medicine
Eczema [allergic contact dermatitis	COL6A5	Expression profiling by array	GPL14550	GSE57225	Intra-individual genome expression analysis reveals a specific molecular signature of psoriasis and eczema / Science Translational Medicine / 2014	Science Translational Medicine
Eczema [allergic contact dermatitis	COL6A6	Expression profiling by array	GPL14550	GSE57225	Intra-individual genome expression analysis reveals a specific molecular signature of psoriasis and eczema / Science Translational Medicine / 2014	Science Translational Medicine
Eczema [allergic contact dermatitis	TNC	Expression profiling by array	GPL14550	GSE57225	Intra-individual genome expression analysis reveals a specific molecular signature of psoriasis and eczema / Science Translational Medicine / 2014	Science Translational Medicine
Eczema [allergic contact dermatitis	SAA2	Expression profiling by array	GPL14550	GSE57225	Intra-individual genome expression analysis reveals a specific molecular signature of psoriasis and eczema / Science Translational Medicine / 2014	Science Translational Medicine
Eczema [allergic contact dermatitis	LOR	Expression profiling by array	GPL14550	GSE57225	Intra-individual genome expression analysis reveals a specific molecular signature of psoriasis and eczema / Science Translational Medicine / 2014	Science Translational Medicine
Eczema [allergic contact dermatitis	LCE1B	Expression profiling by array	GPL14550	GSE57225	Intra-individual genome expression analysis reveals a specific molecular signature of psoriasis and eczema / Science Translational Medicine / 2014	Science Translational Medicine
Eczema [allergic contact dermatitis	TSPAN8	Expression profiling by array	GPL14550	GSE57225	Intra-individual genome expression analysis reveals a specific molecular signature of psoriasis and eczema / Science Translational Medicine / 2014	Science Translational Medicine
Eczema [allergic contact dermatitis	KLK6	Expression profiling by array	GPL14550	GSE57225	Intra-individual genome expression analysis reveals a specific molecular signature of psoriasis and eczema / Science Translational Medicine / 2014	Science Translational Medicine
Eczema [allergic contact dermatitis	KLK9	Expression profiling by array	GPL14550	GSE57225	Intra-individual genome expression analysis reveals a specific molecular signature of psoriasis and eczema / Science Translational Medicine / 2014	Science Translational Medicine
Eczema [allergic contact dermatitis	KLK13	Expression profiling by array	GPL14550	GSE57225	Intra-individual genome expression analysis reveals a specific molecular signature of psoriasis and eczema / Science Translational Medicine / 2014	Science Translational Medicine
Eczema [allergic contact dermatitis	CXCL1	Expression profiling by array	GPL14550	GSE57225	Intra-individual genome expression analysis reveals a specific molecular signature of psoriasis and eczema / Science Translational Medicine / 2014	Science Translational Medicine
Eczema [allergic contact dermatitis	GZMB	Expression profiling by array	GPL14550	GSE57225	Intra-individual genome expression analysis reveals a specific molecular signature of psoriasis and eczema / Science Translational Medicine / 2014	Science Translational Medicine
Eczema [allergic contact dermatitis	IL37	Expression profiling by array	GPL14550	GSE57225	Intra-individual genome expression analysis reveals a specific molecular signature of psoriasis and eczema / Science Translational Medicine / 2014	Science Translational Medicine
Eczema [allergic contact dermatitis	CHP2	Expression profiling by array	GPL14550	GSE57225	Intra-individual genome expression analysis reveals a specific molecular signature of psoriasis and eczema / Science Translational Medicine / 2014	Science Translational Medicine
Eczema [allergic contact dermatitis	IL4I1	Expression profiling by array	GPL14550	GSE57225	Intra-individual genome expression analysis reveals a specific molecular signature of psoriasis and eczema / Science Translational Medicine / 2014	Science Translational Medicine
Eczema [allergic contact dermatitis	IL6	Expression profiling by array	GPL14550	GSE57225	Intra-individual genome expression analysis reveals a specific molecular signature of psoriasis and eczema / Science Translational Medicine / 2014	Science Translational Medicine

Eczema [allergic contact dermatitis	IL13	Expression profiling by array	GPL14550	GSE57225	Intra-individual genome expression analysis reveals a specific molecular signature of psoriasis and eczema / Science Translational Medicine / 2014	Science Translational Medicine
Eczema [allergic contact dermatitis	SOCS3	Expression profiling by array	GPL14550	GSE57225	Intra-individual genome expression analysis reveals a specific molecular signature of psoriasis and eczema / Science Translational Medicine / 2014	Science Translational Medicine
Eczema [allergic contact dermatitis	CCR7	Expression profiling by array	GPL14550	GSE57225	Intra-individual genome expression analysis reveals a specific molecular signature of psoriasis and eczema / Science Translational Medicine / 2014	Science Translational Medicine
Eczema [allergic contact dermatitis	CCL17	Expression profiling by array	GPL14550	GSE57225	Intra-individual genome expression analysis reveals a specific molecular signature of psoriasis and eczema / Science Translational Medicine / 2014	Science Translational Medicine
Eczema [allergic contact dermatitis	CCL18	Expression profiling by array	GPL14550	GSE57225	Intra-individual genome expression analysis reveals a specific molecular signature of psoriasis and eczema / Science Translational Medicine / 2014	Science Translational Medicine
Eczema [allergic contact dermatitis	SELE	Expression profiling by array	GPL14550	GSE57225	Intra-individual genome expression analysis reveals a specific molecular signature of psoriasis and eczema / Science Translational Medicine / 2014	Science Translational Medicine
Eczema [allergic contact dermatitis	CLEC4G	Expression profiling by array	GPL14550	GSE57225	Intra-individual genome expression analysis reveals a specific molecular signature of psoriasis and eczema / Science Translational Medicine / 2014	Science Translational Medicine
Eczema [allergic contact dermatitis	SP5	Expression profiling by array	GPL14550	GSE57225	Intra-individual genome expression analysis reveals a specific molecular signature of psoriasis and eczema / Science Translational Medicine / 2014	Science Translational Medicine
Eczema [allergic contact dermatitis	SERPINB13	Expression profiling by array	GPL14550	GSE57225	Intra-individual genome expression analysis reveals a specific molecular signature of psoriasis and eczema / Science Translational Medicine / 2014	Science Translational Medicine
Eczema [allergic contact dermatitis	ATP12A	Expression profiling by array	GPL14550	GSE57225	Intra-individual genome expression analysis reveals a specific molecular signature of psoriasis and eczema / Science Translational Medicine / 2014	Science Translational Medicine
Eczema [allergic contact dermatitis	SOST	Expression profiling by array	GPL14550	GSE57225	Intra-individual genome expression analysis reveals a specific molecular signature of psoriasis and eczema / Science Translational Medicine / 2014	Science Translational Medicine
Eczema [allergic contact dermatitis	PLA2G4D	Expression profiling by array	GPL14550	GSE57225	Intra-individual genome expression analysis reveals a specific molecular signature of psoriasis and eczema / Science Translational Medicine / 2014	Science Translational Medicine
Eczema [allergic contact dermatitis	NOS2	Expression profiling by array	GPL14550	GSE57225	Intra-individual genome expression analysis reveals a specific molecular signature of psoriasis and eczema / Science Translational Medicine / 2014	Science Translational Medicine
Eczema [allergic contact dermatitis	PRSS22	Expression profiling by array	GPL14550	GSE57225	Intra-individual genome expression analysis reveals a specific molecular signature of psoriasis and eczema / Science Translational Medicine / 2014	Science Translational Medicine
Eczema [allergic contact dermatitis	PRSS27	Expression profiling by array	GPL14550	GSE57225	Intra-individual genome expression analysis reveals a specific molecular signature of psoriasis and eczema / Science Translational Medicine / 2014	Science Translational Medicine
Eczema [allergic contact dermatitis	PRSS53	Expression profiling by array	GPL14550	GSE57225	Intra-individual genome expression analysis reveals a specific molecular signature of psoriasis and eczema / Science Translational Medicine / 2014	Science Translational Medicine
Eczema [allergic contact dermatitis	HPSE	Expression profiling by array	GPL14550	GSE57225	Intra-individual genome expression analysis reveals a specific molecular signature of psoriasis and eczema / Science Translational Medicine / 2014	Science Translational Medicine
Eczema [allergic contact dermatitis	KYNU	Expression profiling by array	GPL14550	GSE57225	Intra-individual genome expression analysis reveals a specific molecular signature of psoriasis and eczema / Science Translational Medicine / 2014	Science Translational Medicine
Eczema [allergic contact dermatitis	TGM1	Expression profiling by array	GPL14550	GSE57225	Intra-individual genome expression analysis reveals a specific molecular signature of psoriasis and eczema / Science Translational Medicine / 2014	Science Translational Medicine
Eczema [allergic contact dermatitis	TREX2	Expression profiling by array	GPL14550	GSE57225	Intra-individual genome expression analysis reveals a specific molecular signature of psoriasis and eczema / Science Translational Medicine / 2014	Science Translational Medicine
Eczema [allergic contact dermatitis	GDA	Expression profiling by array	GPL14550	GSE57225	Intra-individual genome expression analysis reveals a specific molecular signature of psoriasis and eczema / Science Translational Medicine / 2014	Science Translational Medicine
Eczema [allergic contact dermatitis	UPP1	Expression profiling by array	GPL14550	GSE57225	Intra-individual genome expression analysis reveals a specific molecular signature of psoriasis and eczema / Science Translational Medicine / 2014	Science Translational Medicine
Eczema [allergic contact dermatitis	GK	Expression profiling by array	GPL14550	GSE57225	Intra-individual genome expression analysis reveals a specific molecular signature of psoriasis and eczema / Science Translational Medicine / 2014	Science Translational Medicine
Eczema [allergic contact dermatitis	FUT3	Expression profiling by array	GPL14550	GSE57225	Intra-individual genome expression analysis reveals a specific molecular signature of psoriasis and eczema / Science Translational Medicine / 2014	Science Translational Medicine
Eczema [allergic contact dermatitis	SLC6A14	Expression profiling by array	GPL14550	GSE57225	Intra-individual genome expression analysis reveals a specific molecular signature of psoriasis and eczema / Science Translational Medicine / 2014	Science Translational Medicine
Eczema [allergic contact dermatitis	SLC26A9	Expression profiling by array	GPL14550	GSE57225	Intra-individual genome expression analysis reveals a specific molecular signature of psoriasis and eczema / Science Translational Medicine / 2014	Science Translational Medicine
Eczema [allergic contact dermatitis	TCN1	Expression profiling by array	GPL14550	GSE57225	Intra-individual genome expression analysis reveals a specific molecular signature of psoriasis and eczema / Science Translational Medicine / 2014	Science Translational Medicine
Eczema [allergic contact dermatitis	ABCG4	Expression profiling by array	GPL14550	GSE57225	Intra-individual genome expression analysis reveals a specific molecular signature of psoriasis and eczema / Science Translational Medicine / 2014	Science Translational Medicine
Eczema [allergic contact dermatitis	CHAC1	Expression profiling by array	GPL14550	GSE57225	Intra-individual genome expression analysis reveals a specific molecular signature of psoriasis and eczema / Science Translational Medicine / 2014	Science Translational Medicine
Eczema [allergic contact dermatitis	FABP5	Expression profiling by array	GPL14550	GSE57225	Intra-individual genome expression analysis reveals a specific molecular signature of psoriasis and eczema / Science Translational Medicine / 2014	Science Translational Medicine

Eczema [allergic contact dermatitis	LRG1	Expression profiling by array	GPL14550	GSE57225	Intra-individual genome expression analysis reveals a specific molecular signature of psoriasis and eczema / Science Translational Medicine / 2014	Science Translational Medicine
Eczema [allergic contact dermatitis	PLAT	Expression profiling by array	GPL14550	GSE57225	Intra-individual genome expression analysis reveals a specific molecular signature of psoriasis and eczema / Science Translational Medicine / 2014	Science Translational Medicine
Eczema [allergic contact dermatitis	KCNK10	Expression profiling by array	GPL14550	GSE57225	Intra-individual genome expression analysis reveals a specific molecular signature of psoriasis and eczema / Science Translational Medicine / 2014	Science Translational Medicine
Eczema [allergic contact dermatitis	RHCG	Expression profiling by array	GPL14550	GSE57225	Intra-individual genome expression analysis reveals a specific molecular signature of psoriasis and eczema / Science Translational Medicine / 2014	Science Translational Medicine
Eczema [allergic contact dermatitis	KLHDC7B	Expression profiling by array	GPL14550	GSE57225	Intra-individual genome expression analysis reveals a specific molecular signature of psoriasis and eczema / Science Translational Medicine / 2014	Science Translational Medicine
Eczema [allergic contact dermatitis	CYP4Z2P	Expression profiling by array	GPL14550	GSE57225	Intra-individual genome expression analysis reveals a specific molecular signature of psoriasis and eczema / Science Translational Medicine / 2014	Science Translational Medicine
Eczema [allergic contact dermatitis	CPLX3	Expression profiling by array	GPL14550	GSE57225	Intra-individual genome expression analysis reveals a specific molecular signature of psoriasis and eczema / Science Translational Medicine / 2014	Science Translational Medicine
Eczema [allergic contact dermatitis	XDH	Expression profiling by array	GPL14550	GSE57225	Intra-individual genome expression analysis reveals a specific molecular signature of psoriasis and eczema / Science Translational Medicine / 2014	Science Translational Medicine
Eczema [allergic contact dermatitis	ARSF	Expression profiling by array	GPL14550	GSE57225	Intra-individual genome expression analysis reveals a specific molecular signature of psoriasis and eczema / Science Translational Medicine / 2014	Science Translational Medicine
Eczema [allergic contact dermatitis	GPR110	Expression profiling by array	GPL14550	GSE57225	Intra-individual genome expression analysis reveals a specific molecular signature of psoriasis and eczema / Science Translational Medicine / 2014	Science Translational Medicine
Eczema [allergic contact dermatitis	VNN1	Expression profiling by array	GPL14550	GSE57225	Intra-individual genome expression analysis reveals a specific molecular signature of psoriasis and eczema / Science Translational Medicine / 2014	Science Translational Medicine
Eczema [allergic contact dermatitis	HAL	Expression profiling by array	GPL14550	GSE57225	Intra-individual genome expression analysis reveals a specific molecular signature of psoriasis and eczema / Science Translational Medicine / 2014	Science Translational Medicine
Eczema [allergic contact dermatitis	ALDH1A3	Expression profiling by array	GPL14550	GSE57225	Intra-individual genome expression analysis reveals a specific molecular signature of psoriasis and eczema / Science Translational Medicine / 2014	Science Translational Medicine
Eczema [allergic contact dermatitis	GALNT15	Expression profiling by array	GPL14550	GSE57225	Intra-individual genome expression analysis reveals a specific molecular signature of psoriasis and eczema / Science Translational Medicine / 2014	Science Translational Medicine
Eczema [allergic contact dermatitis	UGT3A2	Expression profiling by array	GPL14550	GSE57225	Intra-individual genome expression analysis reveals a specific molecular signature of psoriasis and eczema / Science Translational Medicine / 2014	Science Translational Medicine
Eczema [allergic contact dermatitis	AWAT2	Expression profiling by array	GPL14550	GSE57225	Intra-individual genome expression analysis reveals a specific molecular signature of psoriasis and eczema / Science Translational Medicine / 2014	Science Translational Medicine
Eczema [allergic contact dermatitis	RBP4	Expression profiling by array	GPL14550	GSE57225	Intra-individual genome expression analysis reveals a specific molecular signature of psoriasis and eczema / Science Translational Medicine / 2014	Science Translational Medicine
Eczema [allergic contact dermatitis	HS3ST6	Expression profiling by array	GPL14550	GSE57225	Intra-individual genome expression analysis reveals a specific molecular signature of psoriasis and eczema / Science Translational Medicine / 2014	Science Translational Medicine
Eczema [allergic contact dermatitis	NPTX1	Expression profiling by array	GPL14550	GSE57225	Intra-individual genome expression analysis reveals a specific molecular signature of psoriasis and eczema / Science Translational Medicine / 2014	Science Translational Medicine
Eczema [allergic contact dermatitis	SYT8	Expression profiling by array	GPL14550	GSE57225	Intra-individual genome expression analysis reveals a specific molecular signature of psoriasis and eczema / Science Translational Medicine / 2014	Science Translational Medicine
Eczema [allergic contact dermatitis	TNNI2	Expression profiling by array	GPL14550	GSE57225	Intra-individual genome expression analysis reveals a specific molecular signature of psoriasis and eczema / Science Translational Medicine / 2014	Science Translational Medicine
Eczema [allergic contact dermatitis	SOHLH1	Expression profiling by array	GPL14550	GSE57225	Intra-individual genome expression analysis reveals a specific molecular signature of psoriasis and eczema / Science Translational Medicine / 2014	Science Translational Medicine
Eczema [allergic contact dermatitis	BTBD16	Expression profiling by array	GPL14550	GSE57225	Intra-individual genome expression analysis reveals a specific molecular signature of psoriasis and eczema / Science Translational Medicine / 2014	Science Translational Medicine
Eczema [allergic contact dermatitis	AQP9	Expression profiling by array	GPL14550	GSE57225	Intra-individual genome expression analysis reveals a specific molecular signature of psoriasis and eczema / Science Translational Medicine / 2014	Science Translational Medicine
Eczema [allergic contact dermatitis	LPL	Expression profiling by array	GPL14550	GSE57225	Intra-individual genome expression analysis reveals a specific molecular signature of psoriasis and eczema / Science Translational Medicine / 2014	Science Translational Medicine
Eczema [allergic contact dermatitis	HSD11B1	Expression profiling by array	GPL14550	GSE57225	Intra-individual genome expression analysis reveals a specific molecular signature of psoriasis and eczema / Science Translational Medicine / 2014	Science Translational Medicine
Eczema [allergic contact dermatitis	SCGB2A2	Expression profiling by array	GPL14550	GSE57225	Intra-individual genome expression analysis reveals a specific molecular signature of psoriasis and eczema / Science Translational Medicine / 2014	Science Translational Medicine
Eczema [allergic contact dermatitis	HBA2	Expression profiling by array	GPL14550	GSE57225	Intra-individual genome expression analysis reveals a specific molecular signature of psoriasis and eczema / Science Translational Medicine / 2014	Science Translational Medicine
Eczema [allergic contact dermatitis	HBD	Expression profiling by array	GPL14550	GSE57225	Intra-individual genome expression analysis reveals a specific molecular signature of psoriasis and eczema / Science Translational Medicine / 2014	Science Translational Medicine
Eczema [allergic contact dermatitis	SERPINB3	Expression profiling by array	GPL14550	GSE57225	Intra-individual genome expression analysis reveals a specific molecular signature of psoriasis and eczema / Science Translational Medicine / 2014	Science Translational Medicine

Eczema [allergic contact dermatitis	SERPINB4	Expression profiling by array	GPL14550	GSE57225	Intra-individual genome expression analysis reveals a specific molecular signature of psoriasis and eczema / Science Translational Medicine / 2014	Science Translational Medicine
Eczema [allergic contact dermatitis	AKR1B10	Expression profiling by array	GPL14550	GSE57225	Intra-individual genome expression analysis reveals a specific molecular signature of psoriasis and eczema / Science Translational Medicine / 2014	Science Translational Medicine
Eczema [allergic contact dermatitis	AKR1B15	Expression profiling by array	GPL14550	GSE57225	Intra-individual genome expression analysis reveals a specific molecular signature of psoriasis and eczema / Science Translational Medicine / 2014	Science Translational Medicine
Eczema [allergic contact dermatitis	ADAMTS4	Expression profiling by array	GPL14550	GSE57225	Intra-individual genome expression analysis reveals a specific molecular signature of psoriasis and eczema / Science Translational Medicine / 2014	Science Translational Medicine
Eczema [allergic contact dermatitis	PI3	Expression profiling by array	GPL14550	GSE57225	Intra-individual genome expression analysis reveals a specific molecular signature of psoriasis and eczema / Science Translational Medicine / 2014	Science Translational Medicine
Eczema [allergic contact dermatitis	MMP1	Expression profiling by array	GPL14550	GSE57225	Intra-individual genome expression analysis reveals a specific molecular signature of psoriasis and eczema / Science Translational Medicine / 2014	Science Translational Medicine
Eczema [allergic contact dermatitis	CHI3L2	Expression profiling by array	GPL14550	GSE57225	Intra-individual genome expression analysis reveals a specific molecular signature of psoriasis and eczema / Science Translational Medicine / 2014	Science Translational Medicine
Eczema [allergic contact dermatitis	RGS1	Expression profiling by array	GPL14550	GSE57225	Intra-individual genome expression analysis reveals a specific molecular signature of psoriasis and eczema / Science Translational Medicine / 2014	Science Translational Medicine
Eczema [allergic contact dermatitis	LTF	Expression profiling by array	GPL14550	GSE57225	Intra-individual genome expression analysis reveals a specific molecular signature of psoriasis and eczema / Science Translational Medicine / 2014	Science Translational Medicine
Eczema [allergic contact dermatitis	OASL	Expression profiling by array	GPL14550	GSE57225	Intra-individual genome expression analysis reveals a specific molecular signature of psoriasis and eczema / Science Translational Medicine / 2014	Science Translational Medicine
Eczema [allergic contact dermatitis	SCGB1D1	Expression profiling by array	GPL14550	GSE57225	Intra-individual genome expression analysis reveals a specific molecular signature of psoriasis and eczema / Science Translational Medicine / 2014	Science Translational Medicine
Eczema [allergic contact dermatitis	SCGB1D2	Expression profiling by array	GPL14550	GSE57225	Intra-individual genome expression analysis reveals a specific molecular signature of psoriasis and eczema / Science Translational Medicine / 2014	Science Translational Medicine
Eczema [allergic contact dermatitis	PIP	Expression profiling by array	GPL14550	GSE57225	Intra-individual genome expression analysis reveals a specific molecular signature of psoriasis and eczema / Science Translational Medicine / 2014	Science Translational Medicine
Eczema [allergic contact dermatitis	DGAT2L6	Expression profiling by array	GPL14550	GSE57225	Intra-individual genome expression analysis reveals a specific molecular signature of psoriasis and eczema / Science Translational Medicine / 2014	Science Translational Medicine
Eczema [allergic contact dermatitis	MSMB	Expression profiling by array	GPL14550	GSE57225	Intra-individual genome expression analysis reveals a specific molecular signature of psoriasis and eczema / Science Translational Medicine / 2014	Science Translational Medicine
Eczema [allergic contact dermatitis	MT4	Expression profiling by array	GPL14550	GSE57225	Intra-individual genome expression analysis reveals a specific molecular signature of psoriasis and eczema / Science Translational Medicine / 2014	Science Translational Medicine
Eczema [allergic contact dermatitis	WIF1	Expression profiling by array	GPL14550	GSE57225	Intra-individual genome expression analysis reveals a specific molecular signature of psoriasis and eczema / Science Translational Medicine / 2014	Science Translational Medicine
Eczema [allergic contact dermatitis	PSAPL1	Expression profiling by array	GPL14550	GSE57225	Intra-individual genome expression analysis reveals a specific molecular signature of psoriasis and eczema / Science Translational Medicine / 2014	Science Translational Medicine
Eczema [allergic contact dermatitis	LEP	Expression profiling by array	GPL14550	GSE57225	Intra-individual genome expression analysis reveals a specific molecular signature of psoriasis and eczema / Science Translational Medicine / 2014	Science Translational Medicine
Eczema [allergic contact dermatitis	DCD	Expression profiling by array	GPL14550	GSE57225	Intra-individual genome expression analysis reveals a specific molecular signature of psoriasis and eczema / Science Translational Medicine / 2014	Science Translational Medicine
Eczema [allergic contact dermatitis	SERPINA12	Expression profiling by array	GPL14550	GSE57225	Intra-individual genome expression analysis reveals a specific molecular signature of psoriasis and eczema / Science Translational Medicine / 2014	Science Translational Medicine
Eczema [allergic contact dermatitis	PCSK1	Expression profiling by array	GPL14550	GSE57225	Intra-individual genome expression analysis reveals a specific molecular signature of psoriasis and eczema / Science Translational Medicine / 2014	Science Translational Medicine
Eczema [allergic contact dermatitis	MMP3	Expression profiling by array	GPL14550	GSE57225	Intra-individual genome expression analysis reveals a specific molecular signature of psoriasis and eczema / Science Translational Medicine / 2014	Science Translational Medicine
Eczema [allergic contact dermatitis	MMP12	Expression profiling by array	GPL14550	GSE57225	Intra-individual genome expression analysis reveals a specific molecular signature of psoriasis and eczema / Science Translational Medicine / 2014	Science Translational Medicine
Eczema [allergic contact dermatitis	APOBEC3A	Expression profiling by array	GPL14550	GSE57225	Intra-individual genome expression analysis reveals a specific molecular signature of psoriasis and eczema / Science Translational Medicine / 2014	Science Translational Medicine
Eczema [allergic contact dermatitis	MYOC	Expression profiling by array	GPL14550	GSE57225	Intra-individual genome expression analysis reveals a specific molecular signature of psoriasis and eczema / Science Translational Medicine / 2014	Science Translational Medicine
Eczema [allergic contact dermatitis	FABP7	Expression profiling by array	GPL14550	GSE57225	Intra-individual genome expression analysis reveals a specific molecular signature of psoriasis and eczema / Science Translational Medicine / 2014	Science Translational Medicine
Eczema [allergic contact dermatitis	CXCL8	Expression profiling by array	GPL14550	GSE57225	Intra-individual genome expression analysis reveals a specific molecular signature of psoriasis and eczema / Science Translational Medicine / 2014	Science Translational Medicine
Eczema [allergic contact dermatitis	IL19	Expression profiling by array	GPL14550	GSE57225	Intra-individual genome expression analysis reveals a specific molecular signature of psoriasis and eczema / Science Translational Medicine / 2014	Science Translational Medicine
Eczema [allergic contact dermatitis	IL20	Expression profiling by array	GPL14550	GSE57225	Intra-individual genome expression analysis reveals a specific molecular signature of psoriasis and eczema / Science Translational Medicine / 2014	Science Translational Medicine

Eczema [allergic contact dermatitis	IL36G	Expression profiling by array	GPL14550	GSE57225	Intra-individual genome expression analysis reveals a specific molecular signature of psoriasis and eczema / Science Translational Medicine / 2014	Science Translational Medicine
Eczema [allergic contact dermatitis	IL36A	Expression profiling by array	GPL14550	GSE57225	Intra-individual genome expression analysis reveals a specific molecular signature of psoriasis and eczema / Science Translational Medicine / 2014	Science Translational Medicine
Eczema [allergic contact dermatitis	CLEC3A	Expression profiling by array	GPL14550	GSE57225	Intra-individual genome expression analysis reveals a specific molecular signature of psoriasis and eczema / Science Translational Medicine / 2014	Science Translational Medicine
Eczema [allergic contact dermatitis	TMPRSS11D	Expression profiling by array	GPL14550	GSE57225	Intra-individual genome expression analysis reveals a specific molecular signature of psoriasis and eczema / Science Translational Medicine / 2014	Science Translational Medicine
Eczema [allergic contact dermatitis	TNIP3	Expression profiling by array	GPL14550	GSE57225	Intra-individual genome expression analysis reveals a specific molecular signature of psoriasis and eczema / Science Translational Medicine / 2014	Science Translational Medicine
Eczema [allergic contact dermatitis	CCL27	Expression profiling by array	GPL14550	GSE57225	Intra-individual genome expression analysis reveals a specific molecular signature of psoriasis and eczema / Science Translational Medicine / 2014	Science Translational Medicine
Eczema [allergic contact dermatitis	KLK6	Expression profiling by array	GPL14550	GSE57225	Intra-individual genome expression analysis reveals a specific molecular signature of psoriasis and eczema / Science Translational Medicine / 2014	Science Translational Medicine
Eczema [allergic contact dermatitis	KLK9	Expression profiling by array	GPL14550	GSE57225	Intra-individual genome expression analysis reveals a specific molecular signature of psoriasis and eczema / Science Translational Medicine / 2014	Science Translational Medicine
Eczema [allergic contact dermatitis	KLK13	Expression profiling by array	GPL14550	GSE57225	Intra-individual genome expression analysis reveals a specific molecular signature of psoriasis and eczema / Science Translational Medicine / 2014	Science Translational Medicine
Eczema [allergic contact dermatitis	PRSS22	Expression profiling by array	GPL14550	GSE57225	Intra-individual genome expression analysis reveals a specific molecular signature of psoriasis and eczema / Science Translational Medicine / 2014	Science Translational Medicine
Eczema [allergic contact dermatitis	PRSS27	Expression profiling by array	GPL14550	GSE57225	Intra-individual genome expression analysis reveals a specific molecular signature of psoriasis and eczema / Science Translational Medicine / 2014	Science Translational Medicine
Eczema [allergic contact dermatitis	PRSS53	Expression profiling by array	GPL14550	GSE57225	Intra-individual genome expression analysis reveals a specific molecular signature of psoriasis and eczema / Science Translational Medicine / 2014	Science Translational Medicine
Eczema [allergic contact dermatitis	CXCL1	Expression profiling by array	GPL14550	GSE57225	Intra-individual genome expression analysis reveals a specific molecular signature of psoriasis and eczema / Science Translational Medicine / 2014	Science Translational Medicine
Eczema [allergic contact dermatitis	GZMB	Expression profiling by array	GPL14550	GSE57225	Intra-individual genome expression analysis reveals a specific molecular signature of psoriasis and eczema / Science Translational Medicine / 2014	Science Translational Medicine
Eczema [allergic contact dermatitis	IL37	Expression profiling by array	GPL14550	GSE57225	Intra-individual genome expression analysis reveals a specific molecular signature of psoriasis and eczema / Science Translational Medicine / 2014	Science Translational Medicine
Eczema [allergic contact dermatitis	CHP2	Expression profiling by array	GPL14550	GSE57225	Intra-individual genome expression analysis reveals a specific molecular signature of psoriasis and eczema / Science Translational Medicine / 2014	Science Translational Medicine
Eczema [allergic contact dermatitis	IL4I1	Expression profiling by array	GPL14550	GSE57225	Intra-individual genome expression analysis reveals a specific molecular signature of psoriasis and eczema / Science Translational Medicine / 2014	Science Translational Medicine
Eczema [allergic contact dermatitis	IL6	Expression profiling by array	GPL14550	GSE57225	Intra-individual genome expression analysis reveals a specific molecular signature of psoriasis and eczema / Science Translational Medicine / 2014	Science Translational Medicine
Eczema [allergic contact dermatitis	IL13	Expression profiling by array	GPL14550	GSE57225	Intra-individual genome expression analysis reveals a specific molecular signature of psoriasis and eczema / Science Translational Medicine / 2014	Science Translational Medicine
Eczema [allergic contact dermatitis	SOCS3	Expression profiling by array	GPL14550	GSE57225	Intra-individual genome expression analysis reveals a specific molecular signature of psoriasis and eczema / Science Translational Medicine / 2014	Science Translational Medicine
Eczema [allergic contact dermatitis	CCR7	Expression profiling by array	GPL14550	GSE57225	Intra-individual genome expression analysis reveals a specific molecular signature of psoriasis and eczema / Science Translational Medicine / 2014	Science Translational Medicine
Eczema [allergic contact dermatitis	CCL17	Expression profiling by array	GPL14550	GSE57225	Intra-individual genome expression analysis reveals a specific molecular signature of psoriasis and eczema / Science Translational Medicine / 2014	Science Translational Medicine
Eczema [allergic contact dermatitis	CCL18	Expression profiling by array	GPL14550	GSE57225	Intra-individual genome expression analysis reveals a specific molecular signature of psoriasis and eczema / Science Translational Medicine / 2014	Science Translational Medicine
Eczema [allergic contact dermatitis	SELE	Expression profiling by array	GPL14550	GSE57225	Intra-individual genome expression analysis reveals a specific molecular signature of psoriasis and eczema / Science Translational Medicine / 2014	Science Translational Medicine
Eczema [allergic contact dermatitis	CLEC4G	Expression profiling by array	GPL14550	GSE57225	Intra-individual genome expression analysis reveals a specific molecular signature of psoriasis and eczema / Science Translational Medicine / 2014	Science Translational Medicine
Eczema [allergic contact dermatitis	SP5	Expression profiling by array	GPL14550	GSE57225	Intra-individual genome expression analysis reveals a specific molecular signature of psoriasis and eczema / Science Translational Medicine / 2014	Science Translational Medicine
Eczema [allergic contact dermatitis	SERPINB13	Expression profiling by array	GPL14550	GSE57225	Intra-individual genome expression analysis reveals a specific molecular signature of psoriasis and eczema / Science Translational Medicine / 2014	Science Translational Medicine
Eczema [allergic contact dermatitis	ATP12A	Expression profiling by array	GPL14550	GSE57225	Intra-individual genome expression analysis reveals a specific molecular signature of psoriasis and eczema / Science Translational Medicine / 2014	Science Translational Medicine
Eczema [allergic contact dermatitis	SOST	Expression profiling by array	GPL14550	GSE57225	Intra-individual genome expression analysis reveals a specific molecular signature of psoriasis and eczema / Science Translational Medicine / 2014	Science Translational Medicine
Eczema [allergic contact dermatitis	PLA2G4D	Expression profiling by array	GPL14550	GSE57225	Intra-individual genome expression analysis reveals a specific molecular signature of psoriasis and eczema / Science Translational Medicine / 2014	Science Translational Medicine

Eczema [allergic contact dermatitis	NOS2	Expression profiling by array	GPL14550	GSE57225	Intra-individual genome expression analysis reveals a specific molecular signature of psoriasis and eczema / Science Translational Medicine / 2014	Science Translational Medicine
Eczema [allergic contact dermatitis	HPSE	Expression profiling by array	GPL14550	GSE57225	Intra-individual genome expression analysis reveals a specific molecular signature of psoriasis and eczema / Science Translational Medicine / 2014	Science Translational Medicine
Eczema [allergic contact dermatitis	KYNU	Expression profiling by array	GPL14550	GSE57225	Intra-individual genome expression analysis reveals a specific molecular signature of psoriasis and eczema / Science Translational Medicine / 2014	Science Translational Medicine
Eczema [allergic contact dermatitis	TGM1	Expression profiling by array	GPL14550	GSE57225	Intra-individual genome expression analysis reveals a specific molecular signature of psoriasis and eczema / Science Translational Medicine / 2014	Science Translational Medicine
Eczema [allergic contact dermatitis	TREX2	Expression profiling by array	GPL14550	GSE57225	Intra-individual genome expression analysis reveals a specific molecular signature of psoriasis and eczema / Science Translational Medicine / 2014	Science Translational Medicine
Eczema [allergic contact dermatitis	GDA	Expression profiling by array	GPL14550	GSE57225	Intra-individual genome expression analysis reveals a specific molecular signature of psoriasis and eczema / Science Translational Medicine / 2014	Science Translational Medicine
Eczema [allergic contact dermatitis	UPP1	Expression profiling by array	GPL14550	GSE57225	Intra-individual genome expression analysis reveals a specific molecular signature of psoriasis and eczema / Science Translational Medicine / 2014	Science Translational Medicine
Eczema [allergic contact dermatitis	GK	Expression profiling by array	GPL14550	GSE57225	Intra-individual genome expression analysis reveals a specific molecular signature of psoriasis and eczema / Science Translational Medicine / 2014	Science Translational Medicine
Eczema [allergic contact dermatitis	FUT3	Expression profiling by array	GPL14550	GSE57225	Intra-individual genome expression analysis reveals a specific molecular signature of psoriasis and eczema / Science Translational Medicine / 2014	Science Translational Medicine
Eczema [allergic contact dermatitis	SLC6A14	Expression profiling by array	GPL14550	GSE57225	Intra-individual genome expression analysis reveals a specific molecular signature of psoriasis and eczema / Science Translational Medicine / 2014	Science Translational Medicine
Eczema [allergic contact dermatitis	SLC26A9	Expression profiling by array	GPL14550	GSE57225	Intra-individual genome expression analysis reveals a specific molecular signature of psoriasis and eczema / Science Translational Medicine / 2014	Science Translational Medicine
Eczema [allergic contact dermatitis	TCN1	Expression profiling by array	GPL14550	GSE57225	Intra-individual genome expression analysis reveals a specific molecular signature of psoriasis and eczema / Science Translational Medicine / 2014	Science Translational Medicine
Eczema [allergic contact dermatitis	ABCG4	Expression profiling by array	GPL14550	GSE57225	Intra-individual genome expression analysis reveals a specific molecular signature of psoriasis and eczema / Science Translational Medicine / 2014	Science Translational Medicine
Eczema [allergic contact dermatitis	CHAC1	Expression profiling by array	GPL14550	GSE57225	Intra-individual genome expression analysis reveals a specific molecular signature of psoriasis and eczema / Science Translational Medicine / 2014	Science Translational Medicine
Eczema [allergic contact dermatitis	FABP5	Expression profiling by array	GPL14550	GSE57225	Intra-individual genome expression analysis reveals a specific molecular signature of psoriasis and eczema / Science Translational Medicine / 2014	Science Translational Medicine
Eczema [allergic contact dermatitis	LRG1	Expression profiling by array	GPL14550	GSE57225	Intra-individual genome expression analysis reveals a specific molecular signature of psoriasis and eczema / Science Translational Medicine / 2014	Science Translational Medicine
Eczema [allergic contact dermatitis	PLAT	Expression profiling by array	GPL14550	GSE57225	Intra-individual genome expression analysis reveals a specific molecular signature of psoriasis and eczema / Science Translational Medicine / 2014	Science Translational Medicine
Eczema [allergic contact dermatitis	KCNK10	Expression profiling by array	GPL14550	GSE57225	Intra-individual genome expression analysis reveals a specific molecular signature of psoriasis and eczema / Science Translational Medicine / 2014	Science Translational Medicine
Eczema [allergic contact dermatitis	RHCG	Expression profiling by array	GPL14550	GSE57225	Intra-individual genome expression analysis reveals a specific molecular signature of psoriasis and eczema / Science Translational Medicine / 2014	Science Translational Medicine
Eczema [allergic contact dermatitis	KLHDC7B	Expression profiling by array	GPL14550	GSE57225	Intra-individual genome expression analysis reveals a specific molecular signature of psoriasis and eczema / Science Translational Medicine / 2014	Science Translational Medicine
Eczema [allergic contact dermatitis	CYP4Z2P	Expression profiling by array	GPL14550	GSE57225	Intra-individual genome expression analysis reveals a specific molecular signature of psoriasis and eczema / Science Translational Medicine / 2014	Science Translational Medicine
Eczema [allergic contact dermatitis	CPLX3	Expression profiling by array	GPL14550	GSE57225	Intra-individual genome expression analysis reveals a specific molecular signature of psoriasis and eczema / Science Translational Medicine / 2014	Science Translational Medicine
Eczema [allergic contact dermatitis	XDH	Expression profiling by array	GPL14550	GSE57225	Intra-individual genome expression analysis reveals a specific molecular signature of psoriasis and eczema / Science Translational Medicine / 2014	Science Translational Medicine
Eczema [allergic contact dermatitis	ARSF	Expression profiling by array	GPL14550	GSE57225	Intra-individual genome expression analysis reveals a specific molecular signature of psoriasis and eczema / Science Translational Medicine / 2014	Science Translational Medicine
Eczema [allergic contact dermatitis	GPR110	Expression profiling by array	GPL14550	GSE57225	Intra-individual genome expression analysis reveals a specific molecular signature of psoriasis and eczema / Science Translational Medicine / 2014	Science Translational Medicine
Eczema [allergic contact dermatitis	VNN1	Expression profiling by array	GPL14550	GSE57225	Intra-individual genome expression analysis reveals a specific molecular signature of psoriasis and eczema / Science Translational Medicine / 2014	Science Translational Medicine
Eczema [allergic contact dermatitis	HAL	Expression profiling by array	GPL14550	GSE57225	Intra-individual genome expression analysis reveals a specific molecular signature of psoriasis and eczema / Science Translational Medicine / 2014	Science Translational Medicine
Eczema [allergic contact dermatitis	ALDH1A3	Expression profiling by array	GPL14550	GSE57225	Intra-individual genome expression analysis reveals a specific molecular signature of psoriasis and eczema / Science Translational Medicine / 2014	Science Translational Medicine
Eczema [allergic contact dermatitis	GALNT15	Expression profiling by array	GPL14550	GSE57225	Intra-individual genome expression analysis reveals a specific molecular signature of psoriasis and eczema / Science Translational Medicine / 2014	Science Translational Medicine
Eczema [allergic contact dermatitis	UGT3A2	Expression profiling by array	GPL14550	GSE57225	Intra-individual genome expression analysis reveals a specific molecular signature of psoriasis and eczema / Science Translational Medicine / 2014	Science Translational Medicine

Eczema [allergic contact dermatitis	AWAT2	Expression profiling by array	GPL14550	GSE57225	Intra-individual genome expression analysis reveals a specific molecular signature of psoriasis and eczema / Science Translational Medicine / 2014	Science Translational Medicine
Eczema [allergic contact dermatitis	RBP4	Expression profiling by array	GPL14550	GSE57225	Intra-individual genome expression analysis reveals a specific molecular signature of psoriasis and eczema / Science Translational Medicine / 2014	Science Translational Medicine
Eczema [allergic contact dermatitis	HS3ST6	Expression profiling by array	GPL14550	GSE57225	Intra-individual genome expression analysis reveals a specific molecular signature of psoriasis and eczema / Science Translational Medicine / 2014	Science Translational Medicine
Eczema [allergic contact dermatitis	NPTX1	Expression profiling by array	GPL14550	GSE57225	Intra-individual genome expression analysis reveals a specific molecular signature of psoriasis and eczema / Science Translational Medicine / 2014	Science Translational Medicine
Eczema [allergic contact dermatitis	SYT8	Expression profiling by array	GPL14550	GSE57225	Intra-individual genome expression analysis reveals a specific molecular signature of psoriasis and eczema / Science Translational Medicine / 2014	Science Translational Medicine
Eczema [allergic contact dermatitis	TNNI2	Expression profiling by array	GPL14550	GSE57225	Intra-individual genome expression analysis reveals a specific molecular signature of psoriasis and eczema / Science Translational Medicine / 2014	Science Translational Medicine
Eczema [allergic contact dermatitis	SOHLH1	Expression profiling by array	GPL14550	GSE57225	Intra-individual genome expression analysis reveals a specific molecular signature of psoriasis and eczema / Science Translational Medicine / 2014	Science Translational Medicine
Eczema [allergic contact dermatitis	BTBD16	Expression profiling by array	GPL14550	GSE57225	Intra-individual genome expression analysis reveals a specific molecular signature of psoriasis and eczema / Science Translational Medicine / 2014	Science Translational Medicine
Eczema [allergic contact dermatitis	AQP9	Expression profiling by array	GPL14550	GSE57225	Intra-individual genome expression analysis reveals a specific molecular signature of psoriasis and eczema / Science Translational Medicine / 2014	Science Translational Medicine
Eczema [allergic contact dermatitis	LPL	Expression profiling by array	GPL14550	GSE57225	Intra-individual genome expression analysis reveals a specific molecular signature of psoriasis and eczema / Science Translational Medicine / 2014	Science Translational Medicine
Eczema [allergic contact dermatitis	HSD11B1	Expression profiling by array	GPL14550	GSE57225	Intra-individual genome expression analysis reveals a specific molecular signature of psoriasis and eczema / Science Translational Medicine / 2014	Science Translational Medicine
Eczema [allergic contact dermatitis	SCGB2A2	Expression profiling by array	GPL14550	GSE57225	Intra-individual genome expression analysis reveals a specific molecular signature of psoriasis and eczema / Science Translational Medicine / 2014	Science Translational Medicine
Eczema [allergic contact dermatitis	HBA2	Expression profiling by array	GPL14550	GSE57225	Intra-individual genome expression analysis reveals a specific molecular signature of psoriasis and eczema / Science Translational Medicine / 2014	Science Translational Medicine
Eczema [allergic contact dermatitis	HBD	Expression profiling by array	GPL14550	GSE57225	Intra-individual genome expression analysis reveals a specific molecular signature of psoriasis and eczema / Science Translational Medicine / 2014	Science Translational Medicine
Eczema [allergic contact dermatitis	SERPINB3	Expression profiling by array	GPL14550	GSE57225	Intra-individual genome expression analysis reveals a specific molecular signature of psoriasis and eczema / Science Translational Medicine / 2014	Science Translational Medicine
Eczema [allergic contact dermatitis	SERPINB4	Expression profiling by array	GPL14550	GSE57225	Intra-individual genome expression analysis reveals a specific molecular signature of psoriasis and eczema / Science Translational Medicine / 2014	Science Translational Medicine
Eczema [allergic contact dermatitis	AKR1B10	Expression profiling by array	GPL14550	GSE57225	Intra-individual genome expression analysis reveals a specific molecular signature of psoriasis and eczema / Science Translational Medicine / 2014	Science Translational Medicine
Eczema [allergic contact dermatitis	AKR1B15	Expression profiling by array	GPL14550	GSE57225	Intra-individual genome expression analysis reveals a specific molecular signature of psoriasis and eczema / Science Translational Medicine / 2014	Science Translational Medicine
Eczema [allergic contact dermatitis	ADAMTS4	Expression profiling by array	GPL14550	GSE57225	Intra-individual genome expression analysis reveals a specific molecular signature of psoriasis and eczema / Science Translational Medicine / 2014	Science Translational Medicine
Eczema [allergic contact dermatitis	PI3	Expression profiling by array	GPL14550	GSE57225	Intra-individual genome expression analysis reveals a specific molecular signature of psoriasis and eczema / Science Translational Medicine / 2014	Science Translational Medicine
Eczema [allergic contact dermatitis	MMP1	Expression profiling by array	GPL14550	GSE57225	Intra-individual genome expression analysis reveals a specific molecular signature of psoriasis and eczema / Science Translational Medicine / 2014	Science Translational Medicine
Eczema [allergic contact dermatitis	CHI3L2	Expression profiling by array	GPL14550	GSE57225	Intra-individual genome expression analysis reveals a specific molecular signature of psoriasis and eczema / Science Translational Medicine / 2014	Science Translational Medicine
Eczema [allergic contact dermatitis	RGS1	Expression profiling by array	GPL14550	GSE57225	Intra-individual genome expression analysis reveals a specific molecular signature of psoriasis and eczema / Science Translational Medicine / 2014	Science Translational Medicine
Eczema [allergic contact dermatitis	LTF	Expression profiling by array	GPL14550	GSE57225	Intra-individual genome expression analysis reveals a specific molecular signature of psoriasis and eczema / Science Translational Medicine / 2014	Science Translational Medicine
Eczema [allergic contact dermatitis	OASL	Expression profiling by array	GPL14550	GSE57225	Intra-individual genome expression analysis reveals a specific molecular signature of psoriasis and eczema / Science Translational Medicine / 2014	Science Translational Medicine
Eczema [allergic contact dermatitis	SCGB1D1	Expression profiling by array	GPL14550	GSE57225	Intra-individual genome expression analysis reveals a specific molecular signature of psoriasis and eczema / Science Translational Medicine / 2014	Science Translational Medicine
Eczema [allergic contact dermatitis	SCGB1D2	Expression profiling by array	GPL14550	GSE57225	Intra-individual genome expression analysis reveals a specific molecular signature of psoriasis and eczema / Science Translational Medicine / 2014	Science Translational Medicine
Eczema [allergic contact dermatitis	PIP	Expression profiling by array	GPL14550	GSE57225	Intra-individual genome expression analysis reveals a specific molecular signature of psoriasis and eczema / Science Translational Medicine / 2014	Science Translational Medicine
Eczema [allergic contact dermatitis	DGAT2L6	Expression profiling by array	GPL14550	GSE57225	Intra-individual genome expression analysis reveals a specific molecular signature of psoriasis and eczema / Science Translational Medicine / 2014	Science Translational Medicine
Eczema [allergic contact dermatitis	MSMB	Expression profiling by array	GPL14550	GSE57225	Intra-individual genome expression analysis reveals a specific molecular signature of psoriasis and eczema / Science Translational Medicine / 2014	Science Translational Medicine

Eczema [allergic contact dermatitis	MT4	Expression profiling by array	GPL14550	GSE57225	Intra-individual genome expression analysis reveals a specific molecular signature of psoriasis and eczema / Science Translational Medicine / 2014	Science Translational Medicine
Eczema [allergic contact dermatitis	WIF1	Expression profiling by array	GPL14550	GSE57225	Intra-individual genome expression analysis reveals a specific molecular signature of psoriasis and eczema / Science Translational Medicine / 2014	Science Translational Medicine
Eczema [allergic contact dermatitis	PSAPL1	Expression profiling by array	GPL14550	GSE57225	Intra-individual genome expression analysis reveals a specific molecular signature of psoriasis and eczema / Science Translational Medicine / 2014	Science Translational Medicine
Eczema [allergic contact dermatitis	LEP	Expression profiling by array	GPL14550	GSE57225	Intra-individual genome expression analysis reveals a specific molecular signature of psoriasis and eczema / Science Translational Medicine / 2014	Science Translational Medicine
Eczema [allergic contact dermatitis	DCD	Expression profiling by array	GPL14550	GSE57225	Intra-individual genome expression analysis reveals a specific molecular signature of psoriasis and eczema / Science Translational Medicine / 2014	Science Translational Medicine
Eczema [allergic contact dermatitis	SERPINA12	Expression profiling by array	GPL14550	GSE57225	Intra-individual genome expression analysis reveals a specific molecular signature of psoriasis and eczema / Science Translational Medicine / 2014	Science Translational Medicine
Eczema [allergic contact dermatitis	PCSK1	Expression profiling by array	GPL14550	GSE57225	Intra-individual genome expression analysis reveals a specific molecular signature of psoriasis and eczema / Science Translational Medicine / 2014	Science Translational Medicine
Eczema [allergic contact dermatitis	MMP3	Expression profiling by array	GPL14550	GSE57225	Intra-individual genome expression analysis reveals a specific molecular signature of psoriasis and eczema / Science Translational Medicine / 2014	Science Translational Medicine
Eczema [allergic contact dermatitis	MMP12	Expression profiling by array	GPL14550	GSE57225	Intra-individual genome expression analysis reveals a specific molecular signature of psoriasis and eczema / Science Translational Medicine / 2014	Science Translational Medicine
Eczema [allergic contact dermatitis	APOBEC3A	Expression profiling by array	GPL14550	GSE57225	Intra-individual genome expression analysis reveals a specific molecular signature of psoriasis and eczema / Science Translational Medicine / 2014	Science Translational Medicine
Eczema [allergic contact dermatitis	MYOC	Expression profiling by array	GPL14550	GSE57225	Intra-individual genome expression analysis reveals a specific molecular signature of psoriasis and eczema / Science Translational Medicine / 2014	Science Translational Medicine
Eczema [allergic contact dermatitis	FABP7	Expression profiling by array	GPL14550	GSE57225	Intra-individual genome expression analysis reveals a specific molecular signature of psoriasis and eczema / Science Translational Medicine / 2014	Science Translational Medicine
