# Supplementary material for: Integrative analysis to explore the biological association between environmental skin diseases and ambient particulate matter
Source: Sci Rep. 2022 Jun 13;12:9750. doi: 10.1038/s41598-022-13001-x (PMC9192598; doi:10.1038/s41598-022-13001-x)
Supplement: Supplementary file 8 — Supplementary Figures. [file 41598_2022_13001_MOESM8_ESM.docx]

**
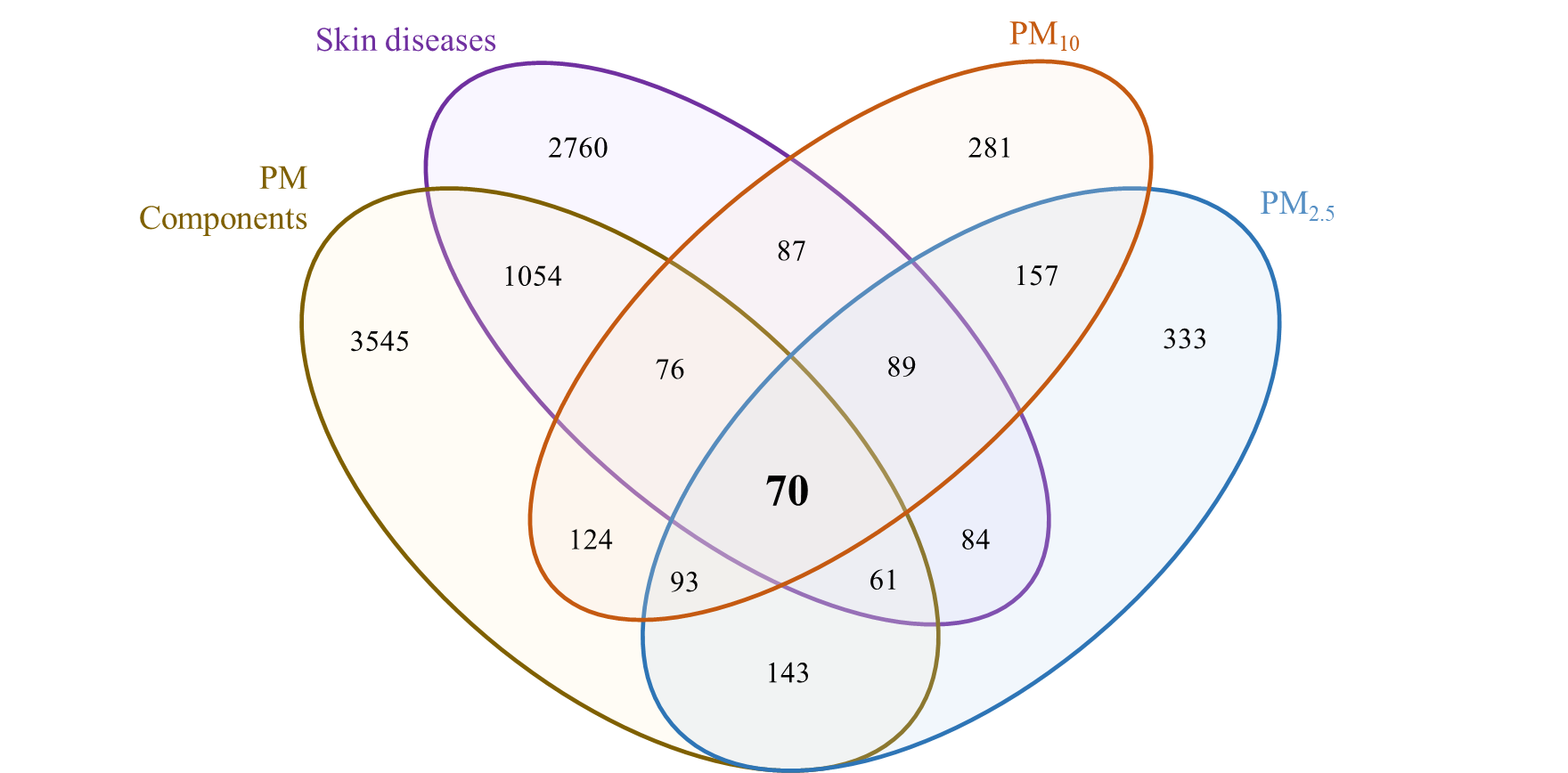
**

**Supplementary Figure S1. Venn diagram for the comparison of gene groups.** Description of the groups was elucidated in “Crawling of global gene expression profiles from public databases.”


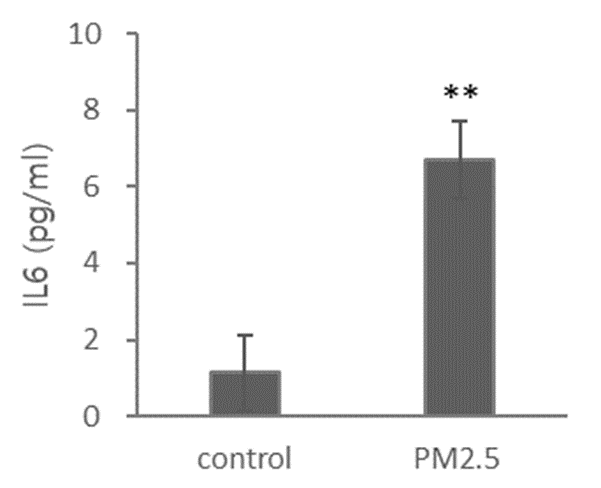


**Supplementary Figure 2. ELISA-based validation of IL-6.** expression alteration of IL-6 at the protein level was validated by enzyme-linked immunosorbent assays (ELISA)


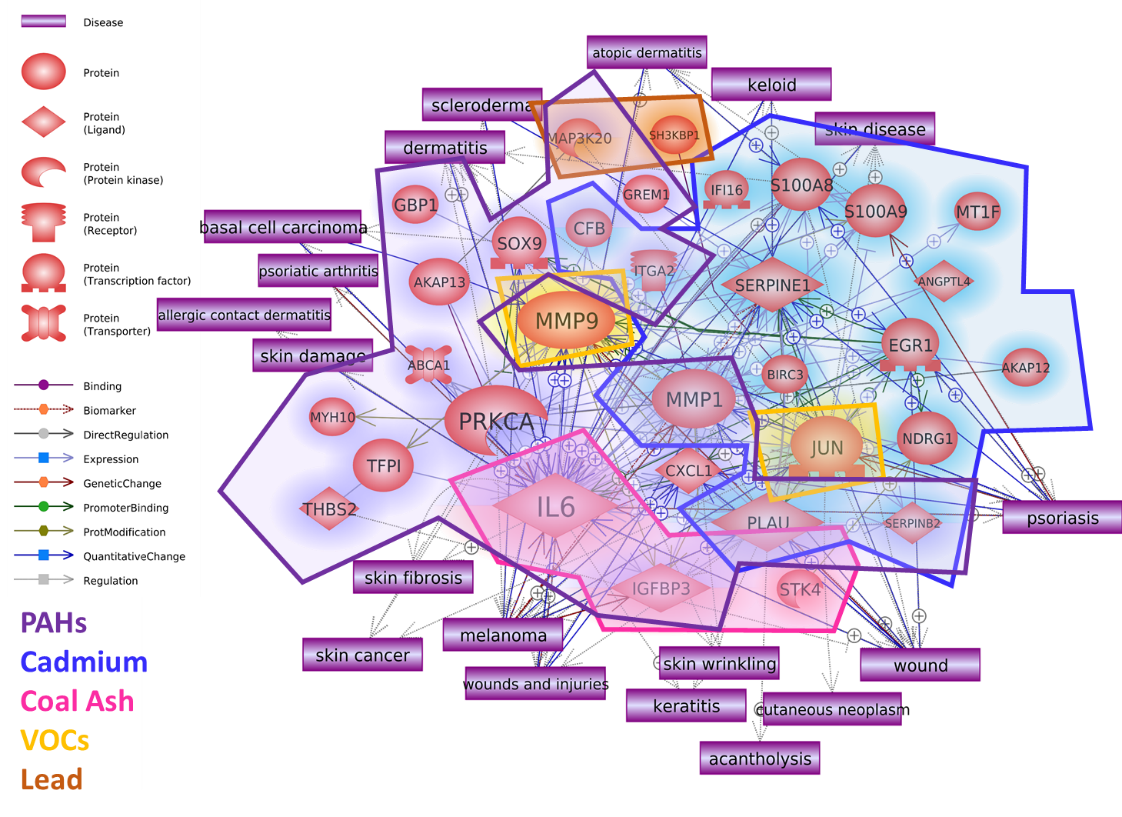


**Supplementary Figure S3. Predicted pathways relevant to skin disorders induced by chemical components of PM.** Signaling networks involved with associations between chemical components of PM and diverse skin disorders, the result of analysis by Pathway Studio software. To improve readability, the genes are highlighted based on associated chemical information from Table 1. A schematic description is located to the left of the pathway.
