# Supplementary material for: Integrative analysis to explore the biological association between environmental skin diseases and ambient particulate matter
Source: Sci Rep. 2022 Jun 13;12:9750. doi: 10.1038/s41598-022-13001-x (PMC9192598; doi:10.1038/s41598-022-13001-x)
Supplement: Supplementary file 9 — Supplementary Tables. [file 41598_2022_13001_MOESM9_ESM.docx]

**Supplementary Table S1.** Profiles of the functional classes and cell processes in the pathway analyzed from literature-based results

|  | **Name** | **Centrality** | |
| --- | --- | --- | --- |
|  |  | **Betweenness** | **Degree** |
| **Functional Classes** | PI3K | 0.03448 | 12 |
|  | Ras GTPase | 0.03222557 | 9 |
|  | Inflammatory cytokine | 0.02184306 | 12 |
|  | NF-kb family | 0.01177785 | 12 |
|  | Cytokine | 0.01112058 | 13 |
|  | Collagen | 0.0085212 | 9 |
|  | ITG | 0.0059893 | 10 |
|  | Mitogen-activated protein kinase | 0.00541319 | 13 |
|  | Matrix metalloproteinase | 0.0032716 | 7 |
|  | IL1 family | 0.00298662 | 9 |
|  | PKC | 0.00236715 | 10 |
|  | NAD(P)H oxidase | 0.00138716 | 7 |
|  | Extracellular matrix protein | 7.97E-04 | 7 |
|  | Growth factor | 3.42E-04 | 10 |
|  | PDGF | 0 | 7 |
| **Cell Processes** | Cell proliferation | 0.06124781 | 23 |
|  | Apoptosis | 0.05430946 | 23 |
|  | Cell migration | 0.04250603 | 20 |
|  | Cell differentiation | 0.02169318 | 18 |
|  | Angiogenesis | 0.02049626 | 16 |
|  | Cell death | 0.007727 | 10 |
|  | Cell growth | 0.00657729 | 15 |
|  | Cell invasion | 0.00488418 | 12 |
|  | Tumor growth | 0.0029174 | 10 |
|  | Inflammatory response | 0.00252503 | 11 |
|  | Epithelial to mesenchymal transition | 9.51E-04 | 5 |
|  | Wound healing | 5.87E-04 | 7 |
|  | Innate immune response | 3.40E-04 | 4 |
|  | Chemotaxis | 2.05E-04 | 6 |
|  | ECM degradation | 1.21E-04 | 6 |
|  | Tissue remodeling | 1.21E-04 | 5 |
|  | Neutrophil migration | 7.73E-05 | 5 |
|  | Cell motility | 0 | 5 |

**Supplementary Table S2.** Primers for qRT-PCR

| **Name** | **Sequence (5'-3')** |
| --- | --- |
| IL6_F | AGGGCTCTTCGGCAAATGT |
| IL6_R | GAAGAAGGAATGCCCATTAACAAC |
| MMP1_F | GCTAACCTTTGATGCTATAACTACGA |
| MMP1_R | TTTGTGCGCATGTAGAATCTG |
| MMP9_F | ATTTCTGCCAGGACCGCTTCTACT |
| MMP9_R | CAGTTTGTATCCGGCAAACTGGCT |
| S100A8_F | GAATTTCCATGCCGTCTACAGG |
| S100A8_R | CCACGCCCATCTTTATCACCAG |
| S100A9_F | GTGGCTCCTCGGCTTTGACA |
| S100A9_R | CCCGAGGCCTGGCTTATGGT |
| PLAU_F | TCACCACCAAAATGCTGTGT |
| PLAU_R | AGGCCATTCTCTCTTCCTTGGT |
| Gapdh_F | GAAACCTGCCAAGTATGATGAC |
| Gapdh_R | ACCTGGTCCTCAGTGTAGC |

**Supplementary Table S3.** Profiles of the functional classes and cell processes in the pathway analyzed from NGS-based results

|  | **Name** | **Betweenness Centrality** | **Degree** |
| --- | --- | --- | --- |
| **Functional classes** | NF-kB family | 0.02441285 | 42 |
|  | Cytokine | 0.01455278 | 41 |
|  | Mitogen-activated protein kinase | 0.00794395 | 29 |
|  | Inflammatory cytokine | 0.01311644 | 28 |
|  | Matrix metalloproteinase | 0.00147299 | 26 |
|  | Growth factor | 0.00643332 | 25 |
|  | Jun/Fos | 0.00430139 | 25 |
|  | Collagen | 8.58E-04 | 22 |
|  | ITG | 6.42E-04 | 21 |
|  | PI3K | 0.0073601 | 21 |
|  | ERK1/2 | 0.0033933 | 20 |
|  | Chemokine | 0.00135061 | 18 |
|  | IL1 family | 7.25E-04 | 17 |
|  | PKC | 0.00624686 | 16 |
|  | JNK | 0.00171443 | 16 |
| **Cell processes** | Cell proliferation | 0.05618451 | 46 |
|  | Cell differentiation | 0.02828798 | 44 |
|  | Apoptosis | 0.03417014 | 43 |
|  | Angiogenesis | 0.00624909 | 29 |
|  | Inflammatory response | 0.00541955 | 27 |
|  | Wound healing | 0.00259151 | 23 |
|  | Tumor growth | 0.00544957 | 22 |
|  | Cell invasion | 0.00628235 | 21 |
|  | Immune response | 0.00165845 | 20 |
|  | ROS generation | 0.00401372 | 18 |
|  | Chemotaxis | 0.00192701 | 15 |
|  | Oxidative stress | 0.00386968 | 14 |
|  | Neutrophil migration | 5.47E-04 | 13 |
|  | Cell motility | 8.86E-04 | 11 |
|  | Neutrophil recruitment | 0 | 11 |
|  | Leukocyte recruitment | 0 | 6 |
|  | T-cell activation | 0 | 5 |
|  | Homeostasis | 9.25E-05 | 5 |
