# Supplementary material for: Integrative analysis to explore the biological association between environmental skin diseases and ambient particulate matter
Source: Sci Rep. 2022 Jun 13;12:9750. doi: 10.1038/s41598-022-13001-x (PMC9192598; doi:10.1038/s41598-022-13001-x)
Supplement: Supplementary file 10 — Supplementary Information 8. [file 41598_2022_13001_MOESM10_ESM.pdf]

		Reference Info			
Keywords	Gene symbol	Author	Title	Journal	Year
Cadmium	ENO1	Guang-Yong Li et al	Gene expression profiling in human lung	Food and Chemical Toxicology	2008
Cadmium	TUBB	Guang-Yong Li et al	Gene expression profiling in human lung	Food and Chemical Toxicology	2008
Cadmium	ACTG2	Guang-Yong Li et al	Gene expression profiling in human lung	Food and Chemical Toxicology	2008
Cadmium	MAD2L1	Guang-Yong Li et al	Gene expression profiling in human lung	Food and Chemical Toxicology	2008
Cadmium	E2F1	Guang-Yong Li et al	Gene expression profiling in human lung	Food and Chemical Toxicology	2008
Cadmium	FOSB	Guang-Yong Li et al	Gene expression profiling in human lung	Food and Chemical Toxicology	2008
Cadmium	TP53	Guang-Yong Li et al	Gene expression profiling in human lung	Food and Chemical Toxicology	2008
Cadmium	CD46	Guang-Yong Li et al	Gene expression profiling in human lung	Food and Chemical Toxicology	2008
Cadmium	IFI16	Guang-Yong Li et al	Gene expression profiling in human lung	Food and Chemical Toxicology	2008
Cadmium	HSPA6	Guang-Yong Li et al	Gene expression profiling in human lung	Food and Chemical Toxicology	2008
Cadmium	IFNGR2	Guang-Yong Li et al	Gene expression profiling in human lung	Food and Chemical Toxicology	2008
Cadmium	HLA-F	Guang-Yong Li et al	Gene expression profiling in human lung	Food and Chemical Toxicology	2008
Cadmium	CCL18	Guang-Yong Li et al	Gene expression profiling in human lung	Food and Chemical Toxicology	2008
Cadmium	CCT3	Guang-Yong Li et al	Gene expression profiling in human lung	Food and Chemical Toxicology	2008
Cadmium	HSP40	Guang-Yong Li et al	Gene expression profiling in human lung	Food and Chemical Toxicology	2008
Cadmium	NMT1	Guang-Yong Li et al	Gene expression profiling in human lung	Food and Chemical Toxicology	2008
Cadmium	PRSS23	Guang-Yong Li et al	Gene expression profiling in human lung	Food and Chemical Toxicology	2008
Cadmium	NPM1	Guang-Yong Li et al	Gene expression profiling in human lung	Food and Chemical Toxicology	2008
Cadmium	RAD23B	Guang-Yong Li et al	Gene expression profiling in human lung	Food and Chemical Toxicology	2008
Cadmium	BCL6	Guang-Yong Li et al	Gene expression profiling in human lung	Food and Chemical Toxicology	2008
Cadmium	CREG1	Guang-Yong Li et al	Gene expression profiling in human lung	Food and Chemical Toxicology	2008
Cadmium	HOX1	Guang-Yong Li et al	Gene expression profiling in human lung	Food and Chemical Toxicology	2008
Cadmium	MTA1	Guang-Yong Li et al	Gene expression profiling in human lung	Food and Chemical Toxicology	2008
Cadmium	NCL	Guang-Yong Li et al	Gene expression profiling in human lung	Food and Chemical Toxicology	2008
Cadmium	BMPR1A	Guang-Yong Li et al	Gene expression profiling in human lung	Food and Chemical Toxicology	2008
Cadmium	FBLN1	Guang-Yong Li et al	Gene expression profiling in human lung	Food and Chemical Toxicology	2008
Cadmium	INHBB	Guang-Yong Li et al	Gene expression profiling in human lung	Food and Chemical Toxicology	2008
Cadmium	PLP2	Guang-Yong Li et al	Gene expression profiling in human lung	Food and Chemical Toxicology	2008
Cadmium	RGS2	Guang-Yong Li et al	Gene expression profiling in human lung	Food and Chemical Toxicology	2008
Cadmium	STAC	Guang-Yong Li et al	Gene expression profiling in human lung	Food and Chemical Toxicology	2008
Cadmium	NR3C1	Guang-Yong Li et al	Gene expression profiling in human lung	Food and Chemical Toxicology	2008
Cadmium	PABPC4	Guang-Yong Li et al	Gene expression profiling in human lung	Food and Chemical Toxicology	2008
Cadmium	SEPTIN7	Guang-Yong Li et al	Gene expression profiling in human lung	Food and Chemical Toxicology	2008
Cadmium	B4GALA2	Guang-Yong Li et al	Gene expression profiling in human lung	Food and Chemical Toxicology	2008
Cadmium	MT1L	Guang-Yong Li et al	Gene expression profiling in human lung	Food and Chemical Toxicology	2008
Cadmium	HSPA1A	Jin-Yong Lee et al	Gene expression analysis using DNA	The Journal of Toxicological Sciences	2013
Cadmium	BAG3	Jin-Yong Lee et al	Gene expression analysis using DNA	The Journal of Toxicological Sciences	2013
Cadmium	HSPA1B	Jin-Yong Lee et al	Gene expression analysis using DNA	The Journal of Toxicological Sciences	2013
Cadmium	HMOX1	Jin-Yong Lee et al	Gene expression analysis using DNA	The Journal of Toxicological Sciences	2013
Cadmium	DNAJB1	Jin-Yong Lee et al	Gene expression analysis using DNA	The Journal of Toxicological Sciences	2013
Cadmium	MT1E	Jin-Yong Lee et al	Gene expression analysis using DNA	The Journal of Toxicological Sciences	2013
Cadmium	HSPH1	Jin-Yong Lee et al	Gene expression analysis using DNA	The Journal of Toxicological Sciences	2013
Cadmium	TRIB3	Jin-Yong Lee et al	Gene expression analysis using DNA	The Journal of Toxicological Sciences	2013
Cadmium	DUSP5	Jin-Yong Lee et al	Gene expression analysis using DNA	The Journal of Toxicological Sciences	2013
Cadmium	CLK1	Jin-Yong Lee et al	Gene expression analysis using DNA	The Journal of Toxicological Sciences	2013
Cadmium	SNAP23	Jin-Yong Lee et al	Gene expression analysis using DNA	The Journal of Toxicological Sciences	2013
Cadmium	GADD45A	Jin-Yong Lee et al	Gene expression analysis using DNA	The Journal of Toxicological Sciences	2013
Cadmium	TXNRD1	Jin-Yong Lee et al	Gene expression analysis using DNA	The Journal of Toxicological Sciences	2013
Cadmium	MT1H	Jin-Yong Lee et al	Gene expression analysis using DNA	The Journal of Toxicological Sciences	2013
Cadmium	RGS2	Jin-Yong Lee et al	Gene expression analysis using DNA	The Journal of Toxicological Sciences	2013
Cadmium	IL8	Jin-Yong Lee et al	Gene expression analysis using DNA	The Journal of Toxicological Sciences	2013
Cadmium	LMCD1	Jin-Yong Lee et al	Gene expression analysis using DNA	The Journal of Toxicological Sciences	2013
Cadmium	FHL2	Jin-Yong Lee et al	Gene expression analysis using DNA	The Journal of Toxicological Sciences	2013
Cadmium	HSP90AB1	Jin-Yong Lee et al	Gene expression analysis using DNA	The Journal of Toxicological Sciences	2013
Cadmium	MT1B	Jin-Yong Lee et al	Gene expression analysis using DNA	The Journal of Toxicological Sciences	2013
Cadmium	RND3	Jin-Yong Lee et al	Gene expression analysis using DNA	The Journal of Toxicological Sciences	2013
Cadmium	SESN2	Jin-Yong Lee et al	Gene expression analysis using DNA	The Journal of Toxicological Sciences	2013
Cadmium	HSP90AA1	Jin-Yong Lee et al	Gene expression analysis using DNA	The Journal of Toxicological Sciences	2013
Cadmium	MT2A	Jin-Yong Lee et al	Gene expression analysis using DNA	The Journal of Toxicological Sciences	2013
Cadmium	CNN3	Jin-Yong Lee et al	Gene expression analysis using DNA	The Journal of Toxicological Sciences	2013
Cadmium	HSPD1	Jin-Yong Lee et al	Gene expression analysis using DNA	The Journal of Toxicological Sciences	2013
Cadmium	WBP5	Jin-Yong Lee et al	Gene expression analysis using DNA	The Journal of Toxicological Sciences	2013
Cadmium	KLF6	Jin-Yong Lee et al	Gene expression analysis using DNA	The Journal of Toxicological Sciences	2013
Cadmium	AMOTL2	Jin-Yong Lee et al	Gene expression analysis using DNA	The Journal of Toxicological Sciences	2013
Cadmium	HSPA8	Jin-Yong Lee et al	Gene expression analysis using DNA	The Journal of Toxicological Sciences	2013
Cadmium	CRYAB	Garrett SH et al	Microarray analysis of gene expression	Journal of Toxicology and Environmental	2010
Cadmium	DNAJA1	Garrett SH et al	Microarray analysis of gene expression	Journal of Toxicology and Environmental	2010
Cadmium	DNAJB1	Garrett SH et al	Microarray analysis of gene expression	Journal of Toxicology and Environmental	2010
Cadmium	DNAJB4	Garrett SH et al	Microarray analysis of gene expression	Journal of Toxicology and Environmental	2010
Cadmium	DNAJB6	Garrett SH et al	Microarray analysis of gene expression	Journal of Toxicology and Environmental	2010
Cadmium	DNAJB9	Garrett SH et al	Microarray analysis of gene expression	Journal of Toxicology and Environmental	2010
Cadmium	EIF2B5	Garrett SH et al	Microarray analysis of gene expression	Journal of Toxicology and Environmental	2010
Cadmium	ERN1	Garrett SH et al	Microarray analysis of gene expression	Journal of Toxicology and Environmental	2010
Cadmium	FKBP4	Garrett SH et al	Microarray analysis of gene expression	Journal of Toxicology and Environmental	2010
Cadmium	GCLC	Garrett SH et al	Microarray analysis of gene expression	Journal of Toxicology and Environmental	2010
Cadmium	HSPA1L	Garrett SH et al	Microarray analysis of gene expression	Journal of Toxicology and Environmental	2010
Cadmium	HSPA4L	Garrett SH et al	Microarray analysis of gene expression	Journal of Toxicology and Environmental	2010
Cadmium	HSPB8	Garrett SH et al	Microarray analysis of gene expression	Journal of Toxicology and Environmental	2010
Cadmium	HSPD1	Garrett SH et al	Microarray analysis of gene expression	Journal of Toxicology and Environmental	2010
Cadmium	HSPE1	Garrett SH et al	Microarray analysis of gene expression	Journal of Toxicology and Environmental	2010
Cadmium	HSPH1	Garrett SH et al	Microarray analysis of gene expression	Journal of Toxicology and Environmental	2010
Cadmium	PPIF	Garrett SH et al	Microarray analysis of gene expression	Journal of Toxicology and Environmental	2010
Cadmium	SERPINH1	Garrett SH et al	Microarray analysis of gene expression	Journal of Toxicology and Environmental	2010
Cadmium	CRYAB	Garrett SH et al	Microarray analysis of gene expression	Journal of Toxicology and Environmental	2010
Cadmium	AHSA2	Garrett SH et al	Microarray analysis of gene expression	Journal of Toxicology and Environmental	2010
Cadmium	DUSP1	Garrett SH et al	Microarray analysis of gene expression	Journal of Toxicology and Environmental	2010
Cadmium	DUSP10	Garrett SH et al	Microarray analysis of gene expression	Journal of Toxicology and Environmental	2010

Cadmium	ERRFI1	Garrett SH et al	Microarray analysis of gene expression	Journal of Toxicology and Environmental	2010
Cadmium	HIG2	Garrett SH et al	Microarray analysis of gene expression	Journal of Toxicology and Environmental	2010
Cadmium	MAFF	Garrett SH et al	Microarray analysis of gene expression	Journal of Toxicology and Environmental	2010
Cadmium	BRE	Garrett SH et al	Microarray analysis of gene expression	Journal of Toxicology and Environmental	2010
Cadmium	DEDD2	Garrett SH et al	Microarray analysis of gene expression	Journal of Toxicology and Environmental	2010
Cadmium	DLC1	Garrett SH et al	Microarray analysis of gene expression	Journal of Toxicology and Environmental	2010
Cadmium	JMJD6	Garrett SH et al	Microarray analysis of gene expression	Journal of Toxicology and Environmental	2010
Cadmium	PMAIP1	Garrett SH et al	Microarray analysis of gene expression	Journal of Toxicology and Environmental	2010
Cadmium	PRLR	Garrett SH et al	Microarray analysis of gene expression	Journal of Toxicology and Environmental	2010
Cadmium	RYBP	Garrett SH et al	Microarray analysis of gene expression	Journal of Toxicology and Environmental	2010
Cadmium	AKR1D1	Garrett SH et al	Microarray analysis of gene expression	Journal of Toxicology and Environmental	2010
Cadmium	MALL	Garrett SH et al	Microarray analysis of gene expression	Journal of Toxicology and Environmental	2010
Cadmium	BCHE	Garrett SH et al	Microarray analysis of gene expression	Journal of Toxicology and Environmental	2010
Cadmium	CA12	Garrett SH et al	Microarray analysis of gene expression	Journal of Toxicology and Environmental	2010
Cadmium	FUT3	Garrett SH et al	Microarray analysis of gene expression	Journal of Toxicology and Environmental	2010
Cadmium	NCOA7	Garrett SH et al	Microarray analysis of gene expression	Journal of Toxicology and Environmental	2010
Cadmium	SULT2B1	Garrett SH et al	Microarray analysis of gene expression	Journal of Toxicology and Environmental	2010
Cadmium	OGDHL	Garrett SH et al	Microarray analysis of gene expression	Journal of Toxicology and Environmental	2010
Cadmium	TXNDC4	Garrett SH et al	Microarray analysis of gene expression	Journal of Toxicology and Environmental	2010
Cadmium	S100A9	Garrett SH et al	Microarray analysis of gene expression	Journal of Toxicology and Environmental	2010
Cadmium	EMR2	Garrett SH et al	Microarray analysis of gene expression	Journal of Toxicology and Environmental	2010
Cadmium	RHOV	Garrett SH et al	Microarray analysis of gene expression	Journal of Toxicology and Environmental	2010
Cadmium	GJA1	Garrett SH et al	Microarray analysis of gene expression	Journal of Toxicology and Environmental	2010
Cadmium	LRRK	Garrett SH et al	Microarray analysis of gene expression	Journal of Toxicology and Environmental	2010
Cadmium	GPR37	Garrett SH et al	Microarray analysis of gene expression	Journal of Toxicology and Environmental	2010
Cadmium	LRRK2	Garrett SH et al	Microarray analysis of gene expression	Journal of Toxicology and Environmental	2010
Cadmium	GPR161	Garrett SH et al	Microarray analysis of gene expression	Journal of Toxicology and Environmental	2010
Cadmium	PITPNC1	Garrett SH et al	Microarray analysis of gene expression	Journal of Toxicology and Environmental	2010
Cadmium	DLGAP1	Garrett SH et al	Microarray analysis of gene expression	Journal of Toxicology and Environmental	2010
Cadmium	GRM8	Garrett SH et al	Microarray analysis of gene expression	Journal of Toxicology and Environmental	2010
Cadmium	SCTR	Garrett SH et al	Microarray analysis of gene expression	Journal of Toxicology and Environmental	2010
Cadmium	CLCA2	Garrett SH et al	Microarray analysis of gene expression	Journal of Toxicology and Environmental	2010
Cadmium	SLC17A1	Garrett SH et al	Microarray analysis of gene expression	Journal of Toxicology and Environmental	2010
Cadmium	SLC17A3	Garrett SH et al	Microarray analysis of gene expression	Journal of Toxicology and Environmental	2010
Cadmium	SLC19A3	Garrett SH et al	Microarray analysis of gene expression	Journal of Toxicology and Environmental	2010
Cadmium	SLC2A3	Garrett SH et al	Microarray analysis of gene expression	Journal of Toxicology and Environmental	2010
Cadmium	SCN3A	Garrett SH et al	Microarray analysis of gene expression	Journal of Toxicology and Environmental	2010
Cadmium	C3	Garrett SH et al	Microarray analysis of gene expression	Journal of Toxicology and Environmental	2010
Cadmium	CLEC7A	Garrett SH et al	Microarray analysis of gene expression	Journal of Toxicology and Environmental	2010
Cadmium	CXCL14	Garrett SH et al	Microarray analysis of gene expression	Journal of Toxicology and Environmental	2010
Cadmium	CXCL5	Garrett SH et al	Microarray analysis of gene expression	Journal of Toxicology and Environmental	2010
Cadmium	ENPP2	Garrett SH et al	Microarray analysis of gene expression	Journal of Toxicology and Environmental	2010
Cadmium	FTH1	Garrett SH et al	Microarray analysis of gene expression	Journal of Toxicology and Environmental	2010
Cadmium	IL15	Garrett SH et al	Microarray analysis of gene expression	Journal of Toxicology and Environmental	2010
Cadmium	IL1RL1	Garrett SH et al	Microarray analysis of gene expression	Journal of Toxicology and Environmental	2010
Cadmium	IL7R	Garrett SH et al	Microarray analysis of gene expression	Journal of Toxicology and Environmental	2010
Cadmium	IL8	Garrett SH et al	Microarray analysis of gene expression	Journal of Toxicology and Environmental	2010
Cadmium	LY96	Garrett SH et al	Microarray analysis of gene expression	Journal of Toxicology and Environmental	2010
Cadmium	OAS1	Garrett SH et al	Microarray analysis of gene expression	Journal of Toxicology and Environmental	2010
Cadmium	PAG1	Garrett SH et al	Microarray analysis of gene expression	Journal of Toxicology and Environmental	2010
Cadmium	PTGER4	Garrett SH et al	Microarray analysis of gene expression	Journal of Toxicology and Environmental	2010
Cadmium	TNFSF10	Garrett SH et al	Microarray analysis of gene expression	Journal of Toxicology and Environmental	2010
Cadmium	TNFSF15	Garrett SH et al	Microarray analysis of gene expression	Journal of Toxicology and Environmental	2010
Cadmium	CYP4F11	Garrett SH et al	Microarray analysis of gene expression	Journal of Toxicology and Environmental	2010
Cadmium	CALCA	Garrett SH et al	Microarray analysis of gene expression	Journal of Toxicology and Environmental	2010
Cadmium	IRAK2	Garrett SH et al	Microarray analysis of gene expression	Journal of Toxicology and Environmental	2010
Cadmium	S100A8	Garrett SH et al	Microarray analysis of gene expression	Journal of Toxicology and Environmental	2010
Cadmium	ANGPTL4	Garrett SH et al	Microarray analysis of gene expression	Journal of Toxicology and Environmental	2010
Cadmium	BAG3	Garrett SH et al	Microarray analysis of gene expression	Journal of Toxicology and Environmental	2010
Cadmium	DNAJA4	Garrett SH et al	Microarray analysis of gene expression	Journal of Toxicology and Environmental	2010
Cadmium	DUSP1	Garrett SH et al	Microarray analysis of gene expression	Journal of Toxicology and Environmental	2010
Cadmium	DUSP10	Garrett SH et al	Microarray analysis of gene expression	Journal of Toxicology and Environmental	2010
Cadmium	EDN1	Garrett SH et al	Microarray analysis of gene expression	Journal of Toxicology and Environmental	2010
Cadmium	GCLM	Garrett SH et al	Microarray analysis of gene expression	Journal of Toxicology and Environmental	2010
Cadmium	HMOX1	Garrett SH et al	Microarray analysis of gene expression	Journal of Toxicology and Environmental	2010
Cadmium	PPP1R15A	Garrett SH et al	Microarray analysis of gene expression	Journal of Toxicology and Environmental	2010
Cadmium	SRXN1	Garrett SH et al	Microarray analysis of gene expression	Journal of Toxicology and Environmental	2010
Cadmium	TTC9	Garrett SH et al	Microarray analysis of gene expression	Journal of Toxicology and Environmental	2010
Cadmium	TXNRD1	Garrett SH et al	Microarray analysis of gene expression	Journal of Toxicology and Environmental	2010
Cadmium	AKR1B10	Garrett SH et al	Microarray analysis of gene expression	Journal of Toxicology and Environmental	2010
Cadmium	AKR1C2	Garrett SH et al	Microarray analysis of gene expression	Journal of Toxicology and Environmental	2010
Cadmium	AKR1C3	Garrett SH et al	Microarray analysis of gene expression	Journal of Toxicology and Environmental	2010
Cadmium	ALDH1L2	Garrett SH et al	Microarray analysis of gene expression	Journal of Toxicology and Environmental	2010
Cadmium	BBOX1	Garrett SH et al	Microarray analysis of gene expression	Journal of Toxicology and Environmental	2010
Cadmium	CYP2E1	Garrett SH et al	Microarray analysis of gene expression	Journal of Toxicology and Environmental	2010
Cadmium	CYP4F3	Garrett SH et al	Microarray analysis of gene expression	Journal of Toxicology and Environmental	2010
Cadmium	LOXL1	Garrett SH et al	Microarray analysis of gene expression	Journal of Toxicology and Environmental	2010
Cadmium	MTHFD2L	Garrett SH et al	Microarray analysis of gene expression	Journal of Toxicology and Environmental	2010
Cadmium	CCNA2	Garrett SH et al	Microarray analysis of gene expression	Journal of Toxicology and Environmental	2010
Cadmium	CCNB1	Garrett SH et al	Microarray analysis of gene expression	Journal of Toxicology and Environmental	2010
Cadmium	CDC20	Garrett SH et al	Microarray analysis of gene expression	Journal of Toxicology and Environmental	2010
Cadmium	CDCA3	Garrett SH et al	Microarray analysis of gene expression	Journal of Toxicology and Environmental	2010
Cadmium	CDKN3	Garrett SH et al	Microarray analysis of gene expression	Journal of Toxicology and Environmental	2010
Cadmium	CENPF	Garrett SH et al	Microarray analysis of gene expression	Journal of Toxicology and Environmental	2010
Cadmium	CEP55	Garrett SH et al	Microarray analysis of gene expression	Journal of Toxicology and Environmental	2010
Cadmium	DLGAP5	Garrett SH et al	Microarray analysis of gene expression	Journal of Toxicology and Environmental	2010
Cadmium	G0S2	Garrett SH et al	Microarray analysis of gene expression	Journal of Toxicology and Environmental	2010
Cadmium	KIF23	Garrett SH et al	Microarray analysis of gene expression	Journal of Toxicology and Environmental	2010
Cadmium	KLK10	Garrett SH et al	Microarray analysis of gene expression	Journal of Toxicology and Environmental	2010

Cadmium	NEK2	Garrett SH et al	Microarray analysis of gene expression	Journal of Toxicology and Environmental	2010
Cadmium	NEK3	Garrett SH et al	Microarray analysis of gene expression	Journal of Toxicology and Environmental	2010
Cadmium	NUF2	Garrett SH et al	Microarray analysis of gene expression	Journal of Toxicology and Environmental	2010
Cadmium	NUSAP1	Garrett SH et al	Microarray analysis of gene expression	Journal of Toxicology and Environmental	2010
Cadmium	PLK1	Garrett SH et al	Microarray analysis of gene expression	Journal of Toxicology and Environmental	2010
Cadmium	PRC1	Garrett SH et al	Microarray analysis of gene expression	Journal of Toxicology and Environmental	2010
Cadmium	RACGAP1	Garrett SH et al	Microarray analysis of gene expression	Journal of Toxicology and Environmental	2010
Cadmium	KRT16	Garrett SH et al	Microarray analysis of gene expression	Journal of Toxicology and Environmental	2010
Cadmium	BCAT1	Garrett SH et al	Microarray analysis of gene expression	Journal of Toxicology and Environmental	2010
Cadmium	TPX2	Garrett SH et al	Microarray analysis of gene expression	Journal of Toxicology and Environmental	2010
Cadmium	TMEM37	Garrett SH et al	Microarray analysis of gene expression	Journal of Toxicology and Environmental	2010
Cadmium	CFTR	Garrett SH et al	Microarray analysis of gene expression	Journal of Toxicology and Environmental	2010
Cadmium	SLC30A1	Garrett SH et al	Microarray analysis of gene expression	Journal of Toxicology and Environmental	2010
Cadmium	TFAP2B	Garrett SH et al	Microarray analysis of gene expression	Journal of Toxicology and Environmental	2010
Cadmium	SLC30A2	Garrett SH et al	Microarray analysis of gene expression	Journal of Toxicology and Environmental	2010
Cadmium	ANXA3	Zelmina Lubovac-Pilav, et al	Using Expression Profiling to Understand the	PLoS One	2013
Cadmium	DAB2	Zelmina Lubovac-Pilav, et al	Using Expression Profiling to Understand the	PLoS One	2013
Cadmium	SLC26A2	Zelmina Lubovac-Pilav, et al	Using Expression Profiling to Understand the	PLoS One	2013
Cadmium	ULBP2	Zelmina Lubovac-Pilav, et al	Using Expression Profiling to Understand the	PLoS One	2013
Cadmium	BST2	Zelmina Lubovac-Pilav, et al	Using Expression Profiling to Understand the	PLoS One	2013
Cadmium	MT1F	Zelmina Lubovac-Pilav, et al	Using Expression Profiling to Understand the	PLoS One	2013
Cadmium	SYTL5	Zelmina Lubovac-Pilav, et al	Using Expression Profiling to Understand the	PLoS One	2013
Cadmium	MIR492	Zelmina Lubovac-Pilav, et al	Using Expression Profiling to Understand the	PLoS One	2013
Cadmium	SCARNA9	Zelmina Lubovac-Pilav, et al	Using Expression Profiling to Understand the	PLoS One	2013
Cadmium	MT1L	Zelmina Lubovac-Pilav, et al	Using Expression Profiling to Understand the	PLoS One	2013
Cadmium	DKK1	Zelmina Lubovac-Pilav, et al	Using Expression Profiling to Understand the	PLoS One	2013
Cadmium	ACTG2	Zelmina Lubovac-Pilav, et al	Using Expression Profiling to Understand the	PLoS One	2013
Cadmium	PIGH	Zelmina Lubovac-Pilav, et al	Using Expression Profiling to Understand the	PLoS One	2013
Cadmium	ZXDA	Zelmina Lubovac-Pilav, et al	Using Expression Profiling to Understand the	PLoS One	2013
Cadmium	PDLIM1	Zelmina Lubovac-Pilav, et al	Using Expression Profiling to Understand the	PLoS One	2013
Cadmium	TMEM229B	Zelmina Lubovac-Pilav, et al	Using Expression Profiling to Understand the	PLoS One	2013
Cadmium	GP1BB	Zelmina Lubovac-Pilav, et al	Using Expression Profiling to Understand the	PLoS One	2013
Cadmium	PTP4A3	Zelmina Lubovac-Pilav, et al	Using Expression Profiling to Understand the	PLoS One	2013
Cadmium	ANXA2P2	Zelmina Lubovac-Pilav, et al	Using Expression Profiling to Understand the	PLoS One	2013
Cadmium	MT1X	Zelmina Lubovac-Pilav, et al	Using Expression Profiling to Understand the	PLoS One	2013
Cadmium	GSTM3	Zelmina Lubovac-Pilav, et al	Using Expression Profiling to Understand the	PLoS One	2013
Cadmium	PRSS8	Zelmina Lubovac-Pilav, et al	Using Expression Profiling to Understand the	PLoS One	2013
Cadmium	UPK2	Zelmina Lubovac-Pilav, et al	Using Expression Profiling to Understand the	PLoS One	2013
Cadmium	MT1H	Zelmina Lubovac-Pilav, et al	Using Expression Profiling to Understand the	PLoS One	2013
Cadmium	CERCAM	Zelmina Lubovac-Pilav, et al	Using Expression Profiling to Understand the	PLoS One	2013
Cadmium	MGLL	Zelmina Lubovac-Pilav, et al	Using Expression Profiling to Understand the	PLoS One	2013
Cadmium	CCDC55	Zelmina Lubovac-Pilav, et al	Using Expression Profiling to Understand the	PLoS One	2013
Cadmium	CD209	Zelmina Lubovac-Pilav, et al	Using Expression Profiling to Understand the	PLoS One	2013
Cadmium	ZNF296	Zelmina Lubovac-Pilav, et al	Using Expression Profiling to Understand the	PLoS One	2013
Cadmium	CRABP1	Zelmina Lubovac-Pilav, et al	Using Expression Profiling to Understand the	PLoS One	2013
Cadmium	MESP1	Zelmina Lubovac-Pilav, et al	Using Expression Profiling to Understand the	PLoS One	2013
Cadmium	HIST1H2BK	Zelmina Lubovac-Pilav, et al	Using Expression Profiling to Understand the	PLoS One	2013
Cadmium	OVOS2	Zelmina Lubovac-Pilav, et al	Using Expression Profiling to Understand the	PLoS One	2013
Cadmium	CBFA2T3	Zelmina Lubovac-Pilav, et al	Using Expression Profiling to Understand the	PLoS One	2013
Cadmium	RPL13AP20	Zelmina Lubovac-Pilav, et al	Using Expression Profiling to Understand the	PLoS One	2013
Cadmium	NTN1	Zelmina Lubovac-Pilav, et al	Using Expression Profiling to Understand the	PLoS One	2013
Cadmium	RASA3	Zelmina Lubovac-Pilav, et al	Using Expression Profiling to Understand the	PLoS One	2013
Cadmium	YBX1P2	Zelmina Lubovac-Pilav, et al	Using Expression Profiling to Understand the	PLoS One	2013
Cadmium	DECR1	Zelmina Lubovac-Pilav, et al	Using Expression Profiling to Understand the	PLoS One	2013
Cadmium	MGAT3	Zelmina Lubovac-Pilav, et al	Using Expression Profiling to Understand the	PLoS One	2013
Cadmium	HOXC9	Zelmina Lubovac-Pilav, et al	Using Expression Profiling to Understand the	PLoS One	2013
Cadmium	FSTL3	Zelmina Lubovac-Pilav, et al	Using Expression Profiling to Understand the	PLoS One	2013
Cadmium	SRR	Zelmina Lubovac-Pilav, et al	Using Expression Profiling to Understand the	PLoS One	2013
Cadmium	RPL18A	Zelmina Lubovac-Pilav, et al	Using Expression Profiling to Understand the	PLoS One	2013
Cadmium	MT1G	Zelmina Lubovac-Pilav, et al	Using Expression Profiling to Understand the	PLoS One	2013
Cadmium	FKBP9	Zelmina Lubovac-Pilav, et al	Using Expression Profiling to Understand the	PLoS One	2013
Cadmium	SLFN12L	Zelmina Lubovac-Pilav, et al	Using Expression Profiling to Understand the	PLoS One	2013
Cadmium	UCP2	Zelmina Lubovac-Pilav, et al	Using Expression Profiling to Understand the	PLoS One	2013
Cadmium	PGLS	Zelmina Lubovac-Pilav, et al	Using Expression Profiling to Understand the	PLoS One	2013
Cadmium	ERCC4	Zelmina Lubovac-Pilav, et al	Using Expression Profiling to Understand the	PLoS One	2013
Cadmium	LOC100509022	Zelmina Lubovac-Pilav, et al	Using Expression Profiling to Understand the	PLoS One	2013
Cadmium	LASP1	Zelmina Lubovac-Pilav, et al	Using Expression Profiling to Understand the	PLoS One	2013
Cadmium	ZNF286B	Zelmina Lubovac-Pilav, et al	Using Expression Profiling to Understand the	PLoS One	2013
Cadmium	SGMS1	Zelmina Lubovac-Pilav, et al	Using Expression Profiling to Understand the	PLoS One	2013
Cadmium	TCF24	Zelmina Lubovac-Pilav, et al	Using Expression Profiling to Understand the	PLoS One	2013
Cadmium	SH2B2	Zelmina Lubovac-Pilav, et al	Using Expression Profiling to Understand the	PLoS One	2013
Cadmium	RPL22	Zelmina Lubovac-Pilav, et al	Using Expression Profiling to Understand the	PLoS One	2013
Cadmium	IRX2	Zelmina Lubovac-Pilav, et al	Using Expression Profiling to Understand the	PLoS One	2013
Cadmium	FN3K	Zelmina Lubovac-Pilav, et al	Using Expression Profiling to Understand the	PLoS One	2013
Cadmium	KRT80	Zelmina Lubovac-Pilav, et al	Using Expression Profiling to Understand the	PLoS One	2013
Cadmium	NKX2-4	Zelmina Lubovac-Pilav, et al	Using Expression Profiling to Understand the	PLoS One	2013
Cadmium	ZNF552	Zelmina Lubovac-Pilav, et al	Using Expression Profiling to Understand the	PLoS One	2013
Cadmium	LCN1	Zelmina Lubovac-Pilav, et al	Using Expression Profiling to Understand the	PLoS One	2013
Cadmium	KRT17	Zelmina Lubovac-Pilav, et al	Using Expression Profiling to Understand the	PLoS One	2013
Cadmium	C1QL3	Zelmina Lubovac-Pilav, et al	Using Expression Profiling to Understand the	PLoS One	2013
Cadmium	LZTR1	Zelmina Lubovac-Pilav, et al	Using Expression Profiling to Understand the	PLoS One	2013
Cadmium	TPM4	Zelmina Lubovac-Pilav, et al	Using Expression Profiling to Understand the	PLoS One	2013
Cadmium	LOC138412	Zelmina Lubovac-Pilav, et al	Using Expression Profiling to Understand the	PLoS One	2013
Cadmium	MT1P2	Zelmina Lubovac-Pilav, et al	Using Expression Profiling to Understand the	PLoS One	2013
Cadmium	DEXI	Zelmina Lubovac-Pilav, et al	Using Expression Profiling to Understand the	PLoS One	2013
Cadmium	MT2A	Zelmina Lubovac-Pilav, et al	Using Expression Profiling to Understand the	PLoS One	2013
Cadmium	GTF3C6	Zelmina Lubovac-Pilav, et al	Using Expression Profiling to Understand the	PLoS One	2013
Cadmium	NFIL3	Zelmina Lubovac-Pilav, et al	Using Expression Profiling to Understand the	PLoS One	2013
Cadmium	DKFZP434I0714	Zelmina Lubovac-Pilav, et al	Using Expression Profiling to Understand the	PLoS One	2013

Cadmium	GSC2	Zelmina Lubovac-Pilav, et al	Using Expression Profiling to Understand the	PLoS One	2013
Cadmium	SLC45A1	Zelmina Lubovac-Pilav, et al	Using Expression Profiling to Understand the	PLoS One	2013
Cadmium	BIN1	Zelmina Lubovac-Pilav, et al	Using Expression Profiling to Understand the	PLoS One	2013
Cadmium	B4GALNT4	Zelmina Lubovac-Pilav, et al	Using Expression Profiling to Understand the	PLoS One	2013
Cadmium	NPDC1	Zelmina Lubovac-Pilav, et al	Using Expression Profiling to Understand the	PLoS One	2013
Cadmium	MYL12A	Zelmina Lubovac-Pilav, et al	Using Expression Profiling to Understand the	PLoS One	2013
Cadmium	SPHK1	Zelmina Lubovac-Pilav, et al	Using Expression Profiling to Understand the	PLoS One	2013
Cadmium	LMCD1	Zelmina Lubovac-Pilav, et al	Using Expression Profiling to Understand the	PLoS One	2013
Cadmium	TRADD	Zelmina Lubovac-Pilav, et al	Using Expression Profiling to Understand the	PLoS One	2013
Cadmium	IGF1R	Zelmina Lubovac-Pilav, et al	Using Expression Profiling to Understand the	PLoS One	2013
Cadmium	CRYL1	Zelmina Lubovac-Pilav, et al	Using Expression Profiling to Understand the	PLoS One	2013
Cadmium	QDPR	Zelmina Lubovac-Pilav, et al	Using Expression Profiling to Understand the	PLoS One	2013
Cadmium	SOX18	Zelmina Lubovac-Pilav, et al	Using Expression Profiling to Understand the	PLoS One	2013
Cadmium	IMPACT	Zelmina Lubovac-Pilav, et al	Using Expression Profiling to Understand the	PLoS One	2013
Cadmium	TELO2	Zelmina Lubovac-Pilav, et al	Using Expression Profiling to Understand the	PLoS One	2013
Cadmium	IFITM3	Zelmina Lubovac-Pilav, et al	Using Expression Profiling to Understand the	PLoS One	2013
Cadmium	RPL23AP82	Zelmina Lubovac-Pilav, et al	Using Expression Profiling to Understand the	PLoS One	2013
Cadmium	MAPK6	Zelmina Lubovac-Pilav, et al	Using Expression Profiling to Understand the	PLoS One	2013
Cadmium	TMSB4X	Zelmina Lubovac-Pilav, et al	Using Expression Profiling to Understand the	PLoS One	2013
Cadmium	FOSL2	Zelmina Lubovac-Pilav, et al	Using Expression Profiling to Understand the	PLoS One	2013
Cadmium	PLS3	Zelmina Lubovac-Pilav, et al	Using Expression Profiling to Understand the	PLoS One	2013
Cadmium	FOXF2	Zelmina Lubovac-Pilav, et al	Using Expression Profiling to Understand the	PLoS One	2013
Cadmium	ZNHIT2	Zelmina Lubovac-Pilav, et al	Using Expression Profiling to Understand the	PLoS One	2013
Cadmium	CLSPN	Zelmina Lubovac-Pilav, et al	Using Expression Profiling to Understand the	PLoS One	2013
Cadmium	C8ORF30A	Zelmina Lubovac-Pilav, et al	Using Expression Profiling to Understand the	PLoS One	2013
Cadmium	WWP1	Zelmina Lubovac-Pilav, et al	Using Expression Profiling to Understand the	PLoS One	2013
Cadmium	KRTAP5-2	Zelmina Lubovac-Pilav, et al	Using Expression Profiling to Understand the	PLoS One	2013
Cadmium	LOC400590	Zelmina Lubovac-Pilav, et al	Using Expression Profiling to Understand the	PLoS One	2013
Cadmium	TTC19	Zelmina Lubovac-Pilav, et al	Using Expression Profiling to Understand the	PLoS One	2013
Cadmium	BEX4	Zelmina Lubovac-Pilav, et al	Using Expression Profiling to Understand the	PLoS One	2013
Cadmium	HNRNPA1L2	Zelmina Lubovac-Pilav, et al	Using Expression Profiling to Understand the	PLoS One	2013
Cadmium	PKMYT1	Zelmina Lubovac-Pilav, et al	Using Expression Profiling to Understand the	PLoS One	2013
Cadmium	TCEAL6	Zelmina Lubovac-Pilav, et al	Using Expression Profiling to Understand the	PLoS One	2013
Cadmium	LHPP	Zelmina Lubovac-Pilav, et al	Using Expression Profiling to Understand the	PLoS One	2013
Cadmium	FLNB	Zelmina Lubovac-Pilav, et al	Using Expression Profiling to Understand the	PLoS One	2013
Cadmium	TSSK6	Zelmina Lubovac-Pilav, et al	Using Expression Profiling to Understand the	PLoS One	2013
Cadmium	SNAI3	Zelmina Lubovac-Pilav, et al	Using Expression Profiling to Understand the	PLoS One	2013
Cadmium	TUBB2A	Zelmina Lubovac-Pilav, et al	Using Expression Profiling to Understand the	PLoS One	2013
Cadmium	HSP90AB2P	Zelmina Lubovac-Pilav, et al	Using Expression Profiling to Understand the	PLoS One	2013
Cadmium	IFNA13	Zelmina Lubovac-Pilav, et al	Using Expression Profiling to Understand the	PLoS One	2013
Cadmium	SGPP1	Zelmina Lubovac-Pilav, et al	Using Expression Profiling to Understand the	PLoS One	2013
Cadmium	RPL13	Zelmina Lubovac-Pilav, et al	Using Expression Profiling to Understand the	PLoS One	2013
Cadmium	CD302	Zelmina Lubovac-Pilav, et al	Using Expression Profiling to Understand the	PLoS One	2013
Cadmium	EID2	Zelmina Lubovac-Pilav, et al	Using Expression Profiling to Understand the	PLoS One	2013
Cadmium	GPC1	Zelmina Lubovac-Pilav, et al	Using Expression Profiling to Understand the	PLoS One	2013
Cadmium	ATP2A3	Zelmina Lubovac-Pilav, et al	Using Expression Profiling to Understand the	PLoS One	2013
Cadmium	MSI1	Zelmina Lubovac-Pilav, et al	Using Expression Profiling to Understand the	PLoS One	2013
Cadmium	REEP1	Zelmina Lubovac-Pilav, et al	Using Expression Profiling to Understand the	PLoS One	2013
Cadmium	CCNE2	Zelmina Lubovac-Pilav, et al	Using Expression Profiling to Understand the	PLoS One	2013
Cadmium	TIGD5	Zelmina Lubovac-Pilav, et al	Using Expression Profiling to Understand the	PLoS One	2013
Cadmium	BAG1	Zelmina Lubovac-Pilav, et al	Using Expression Profiling to Understand the	PLoS One	2013
Cadmium	VGF	Zelmina Lubovac-Pilav, et al	Using Expression Profiling to Understand the	PLoS One	2013
Cadmium	MAFA	Zelmina Lubovac-Pilav, et al	Using Expression Profiling to Understand the	PLoS One	2013
Cadmium	RPS18P9	Zelmina Lubovac-Pilav, et al	Using Expression Profiling to Understand the	PLoS One	2013
Cadmium	RAB25	Zelmina Lubovac-Pilav, et al	Using Expression Profiling to Understand the	PLoS One	2013
Cadmium	SRD5A1	Zelmina Lubovac-Pilav, et al	Using Expression Profiling to Understand the	PLoS One	2013
Cadmium	GABRD	Zelmina Lubovac-Pilav, et al	Using Expression Profiling to Understand the	PLoS One	2013
Cadmium	FAM89B	Zelmina Lubovac-Pilav, et al	Using Expression Profiling to Understand the	PLoS One	2013
Cadmium	FAM82B	Zelmina Lubovac-Pilav, et al	Using Expression Profiling to Understand the	PLoS One	2013
Cadmium	FBXO43	Zelmina Lubovac-Pilav, et al	Using Expression Profiling to Understand the	PLoS One	2013
Cadmium	WDSUB1	Zelmina Lubovac-Pilav, et al	Using Expression Profiling to Understand the	PLoS One	2013
Cadmium	RAB32	Zelmina Lubovac-Pilav, et al	Using Expression Profiling to Understand the	PLoS One	2013
Cadmium	CCDC97	Zelmina Lubovac-Pilav, et al	Using Expression Profiling to Understand the	PLoS One	2013
Cadmium	SMCR8	Zelmina Lubovac-Pilav, et al	Using Expression Profiling to Understand the	PLoS One	2013
Cadmium	FLJ43681	Zelmina Lubovac-Pilav, et al	Using Expression Profiling to Understand the	PLoS One	2013
Cadmium	SPATA20	Zelmina Lubovac-Pilav, et al	Using Expression Profiling to Understand the	PLoS One	2013
Cadmium	RPS2P32	Zelmina Lubovac-Pilav, et al	Using Expression Profiling to Understand the	PLoS One	2013
Cadmium	YTHDF3	Zelmina Lubovac-Pilav, et al	Using Expression Profiling to Understand the	PLoS One	2013
Cadmium	GLUL	Zelmina Lubovac-Pilav, et al	Using Expression Profiling to Understand the	PLoS One	2013
Cadmium	UCN	Zelmina Lubovac-Pilav, et al	Using Expression Profiling to Understand the	PLoS One	2013
Cadmium	GABARAP	Zelmina Lubovac-Pilav, et al	Using Expression Profiling to Understand the	PLoS One	2013
Cadmium	C11ORF80	Zelmina Lubovac-Pilav, et al	Using Expression Profiling to Understand the	PLoS One	2013
Cadmium	HES4	Zelmina Lubovac-Pilav, et al	Using Expression Profiling to Understand the	PLoS One	2013
Cadmium	ANXA2	Zelmina Lubovac-Pilav, et al	Using Expression Profiling to Understand the	PLoS One	2013
Cadmium	GM2A	Zelmina Lubovac-Pilav, et al	Using Expression Profiling to Understand the	PLoS One	2013
Cadmium	MINK1	Zelmina Lubovac-Pilav, et al	Using Expression Profiling to Understand the	PLoS One	2013
Cadmium	PCSK1N	Zelmina Lubovac-Pilav, et al	Using Expression Profiling to Understand the	PLoS One	2013
Cadmium	ZNF90	Zelmina Lubovac-Pilav, et al	Using Expression Profiling to Understand the	PLoS One	2013
Cadmium	RPL13AP6	Zelmina Lubovac-Pilav, et al	Using Expression Profiling to Understand the	PLoS One	2013
Cadmium	EPB41L1	Zelmina Lubovac-Pilav, et al	Using Expression Profiling to Understand the	PLoS One	2013
Cadmium	TSPAN33	Zelmina Lubovac-Pilav, et al	Using Expression Profiling to Understand the	PLoS One	2013
Cadmium	FGF4	Zelmina Lubovac-Pilav, et al	Using Expression Profiling to Understand the	PLoS One	2013
Cadmium	C20ORF144	Zelmina Lubovac-Pilav, et al	Using Expression Profiling to Understand the	PLoS One	2013
Cadmium	RPL7A	Zelmina Lubovac-Pilav, et al	Using Expression Profiling to Understand the	PLoS One	2013
Cadmium	CASP6	Zelmina Lubovac-Pilav, et al	Using Expression Profiling to Understand the	PLoS One	2013
Cadmium	UNC93B1	Zelmina Lubovac-Pilav, et al	Using Expression Profiling to Understand the	PLoS One	2013
Cadmium	CREG1	Zelmina Lubovac-Pilav, et al	Using Expression Profiling to Understand the	PLoS One	2013
Cadmium	ZP1	Zelmina Lubovac-Pilav, et al	Using Expression Profiling to Understand the	PLoS One	2013
Cadmium	TMEM8A	Zelmina Lubovac-Pilav, et al	Using Expression Profiling to Understand the	PLoS One	2013

Cadmium	SLC25A4	Zelmina Lubovac-Pilav, et al	Using Expression Profiling to Understand the	PLoS One	2013
Cadmium	CYHR1	Zelmina Lubovac-Pilav, et al	Using Expression Profiling to Understand the	PLoS One	2013
Cadmium	HES3	Zelmina Lubovac-Pilav, et al	Using Expression Profiling to Understand the	PLoS One	2013
Cadmium	RNMT	Zelmina Lubovac-Pilav, et al	Using Expression Profiling to Understand the	PLoS One	2013
Cadmium	SLC25A24	Zelmina Lubovac-Pilav, et al	Using Expression Profiling to Understand the	PLoS One	2013
Cadmium	TIMM23	Zelmina Lubovac-Pilav, et al	Using Expression Profiling to Understand the	PLoS One	2013
Cadmium	SBK2	Zelmina Lubovac-Pilav, et al	Using Expression Profiling to Understand the	PLoS One	2013
Cadmium	RBMXL2	Zelmina Lubovac-Pilav, et al	Using Expression Profiling to Understand the	PLoS One	2013
Cadmium	USP25	Zelmina Lubovac-Pilav, et al	Using Expression Profiling to Understand the	PLoS One	2013
Cadmium	ST6GALNAC2	Zelmina Lubovac-Pilav, et al	Using Expression Profiling to Understand the	PLoS One	2013
Cadmium	EIF1B	Zelmina Lubovac-Pilav, et al	Using Expression Profiling to Understand the	PLoS One	2013
Cadmium	SLC3A2	Zelmina Lubovac-Pilav, et al	Using Expression Profiling to Understand the	PLoS One	2013
Cadmium	RRAGA	Zelmina Lubovac-Pilav, et al	Using Expression Profiling to Understand the	PLoS One	2013
Cadmium	DSTN	Zelmina Lubovac-Pilav, et al	Using Expression Profiling to Understand the	PLoS One	2013
Cadmium	CASKIN1	Zelmina Lubovac-Pilav, et al	Using Expression Profiling to Understand the	PLoS One	2013
Cadmium	GXYLT1	Zelmina Lubovac-Pilav, et al	Using Expression Profiling to Understand the	PLoS One	2013
Cadmium	MMP17	Zelmina Lubovac-Pilav, et al	Using Expression Profiling to Understand the	PLoS One	2013
Cadmium	EEF1A2	Zelmina Lubovac-Pilav, et al	Using Expression Profiling to Understand the	PLoS One	2013
Cadmium	HELT	Zelmina Lubovac-Pilav, et al	Using Expression Profiling to Understand the	PLoS One	2013
Cadmium	C8ORF55	Zelmina Lubovac-Pilav, et al	Using Expression Profiling to Understand the	PLoS One	2013
Cadmium	MORF4	Zelmina Lubovac-Pilav, et al	Using Expression Profiling to Understand the	PLoS One	2013
Cadmium	CPNE3	Zelmina Lubovac-Pilav, et al	Using Expression Profiling to Understand the	PLoS One	2013
Cadmium	MZT2A	Zelmina Lubovac-Pilav, et al	Using Expression Profiling to Understand the	PLoS One	2013
Cadmium	GNPTG	Zelmina Lubovac-Pilav, et al	Using Expression Profiling to Understand the	PLoS One	2013
Cadmium	C6ORF150	Zelmina Lubovac-Pilav, et al	Using Expression Profiling to Understand the	PLoS One	2013
Cadmium	OSTF1	Zelmina Lubovac-Pilav, et al	Using Expression Profiling to Understand the	PLoS One	2013
Cadmium	ASGR1	Zelmina Lubovac-Pilav, et al	Using Expression Profiling to Understand the	PLoS One	2013
Cadmium	NME4	Zelmina Lubovac-Pilav, et al	Using Expression Profiling to Understand the	PLoS One	2013
Cadmium	OGFOD1	Zelmina Lubovac-Pilav, et al	Using Expression Profiling to Understand the	PLoS One	2013
Cadmium	CLEC11A	Zelmina Lubovac-Pilav, et al	Using Expression Profiling to Understand the	PLoS One	2013
Cadmium	TAX1BP3	Zelmina Lubovac-Pilav, et al	Using Expression Profiling to Understand the	PLoS One	2013
Cadmium	SAC3D1	Zelmina Lubovac-Pilav, et al	Using Expression Profiling to Understand the	PLoS One	2013
Cadmium	HIPK2	Zelmina Lubovac-Pilav, et al	Using Expression Profiling to Understand the	PLoS One	2013
Cadmium	HLA-F	Zelmina Lubovac-Pilav, et al	Using Expression Profiling to Understand the	PLoS One	2013
Cadmium	ANXA2P1	Zelmina Lubovac-Pilav, et al	Using Expression Profiling to Understand the	PLoS One	2013
Cadmium	P2RX4	Zelmina Lubovac-Pilav, et al	Using Expression Profiling to Understand the	PLoS One	2013
Cadmium	CST3	Zelmina Lubovac-Pilav, et al	Using Expression Profiling to Understand the	PLoS One	2013
Cadmium	CCNE1	Zelmina Lubovac-Pilav, et al	Using Expression Profiling to Understand the	PLoS One	2013
Cadmium	SLC15A4	Zelmina Lubovac-Pilav, et al	Using Expression Profiling to Understand the	PLoS One	2013
Cadmium	EXOC3	Zelmina Lubovac-Pilav, et al	Using Expression Profiling to Understand the	PLoS One	2013
Cadmium	TMEM99	Zelmina Lubovac-Pilav, et al	Using Expression Profiling to Understand the	PLoS One	2013
Cadmium	MIR375	Zelmina Lubovac-Pilav, et al	Using Expression Profiling to Understand the	PLoS One	2013
Cadmium	HES7	Zelmina Lubovac-Pilav, et al	Using Expression Profiling to Understand the	PLoS One	2013
Cadmium	ANP32C	Zelmina Lubovac-Pilav, et al	Using Expression Profiling to Understand the	PLoS One	2013
Cadmium	ZNF747	Zelmina Lubovac-Pilav, et al	Using Expression Profiling to Understand the	PLoS One	2013
Cadmium	FRMD8	Zelmina Lubovac-Pilav, et al	Using Expression Profiling to Understand the	PLoS One	2013
Cadmium	HSDL2	Zelmina Lubovac-Pilav, et al	Using Expression Profiling to Understand the	PLoS One	2013
Cadmium	LASS4	Zelmina Lubovac-Pilav, et al	Using Expression Profiling to Understand the	PLoS One	2013
Cadmium	SPTBN2	Zelmina Lubovac-Pilav, et al	Using Expression Profiling to Understand the	PLoS One	2013
Cadmium	C1ORF85	Zelmina Lubovac-Pilav, et al	Using Expression Profiling to Understand the	PLoS One	2013
Cadmium	ZFP36L2	Zelmina Lubovac-Pilav, et al	Using Expression Profiling to Understand the	PLoS One	2013
Cadmium	BPHL	Zelmina Lubovac-Pilav, et al	Using Expression Profiling to Understand the	PLoS One	2013
Cadmium	AMDHD2	Zelmina Lubovac-Pilav, et al	Using Expression Profiling to Understand the	PLoS One	2013
Cadmium	KBTBD13	Zelmina Lubovac-Pilav, et al	Using Expression Profiling to Understand the	PLoS One	2013
Cadmium	SYT7	Zelmina Lubovac-Pilav, et al	Using Expression Profiling to Understand the	PLoS One	2013
Cadmium	BOK	Zelmina Lubovac-Pilav, et al	Using Expression Profiling to Understand the	PLoS One	2013
Cadmium	KCNK5	Zelmina Lubovac-Pilav, et al	Using Expression Profiling to Understand the	PLoS One	2013
Cadmium	SSBP4	Zelmina Lubovac-Pilav, et al	Using Expression Profiling to Understand the	PLoS One	2013
Cadmium	JUB	Zelmina Lubovac-Pilav, et al	Using Expression Profiling to Understand the	PLoS One	2013
Cadmium	EIF4B	Zelmina Lubovac-Pilav, et al	Using Expression Profiling to Understand the	PLoS One	2013
Cadmium	RAMP1	Zelmina Lubovac-Pilav, et al	Using Expression Profiling to Understand the	PLoS One	2013
Cadmium	D2HGDH	Zelmina Lubovac-Pilav, et al	Using Expression Profiling to Understand the	PLoS One	2013
Cadmium	DTL	Zelmina Lubovac-Pilav, et al	Using Expression Profiling to Understand the	PLoS One	2013
Cadmium	PLD6	Zelmina Lubovac-Pilav, et al	Using Expression Profiling to Understand the	PLoS One	2013
Cadmium	HNRNPA0	Zelmina Lubovac-Pilav, et al	Using Expression Profiling to Understand the	PLoS One	2013
Cadmium	B4GALT7	Zelmina Lubovac-Pilav, et al	Using Expression Profiling to Understand the	PLoS One	2013
Cadmium	TIMP3	Zelmina Lubovac-Pilav, et al	Using Expression Profiling to Understand the	PLoS One	2013
Cadmium	MYH14	Zelmina Lubovac-Pilav, et al	Using Expression Profiling to Understand the	PLoS One	2013
Cadmium	KANK2	Zelmina Lubovac-Pilav, et al	Using Expression Profiling to Understand the	PLoS One	2013
Cadmium	SGSH	Zelmina Lubovac-Pilav, et al	Using Expression Profiling to Understand the	PLoS One	2013
Cadmium	ZNF286A	Zelmina Lubovac-Pilav, et al	Using Expression Profiling to Understand the	PLoS One	2013
Cadmium	NT5M	Zelmina Lubovac-Pilav, et al	Using Expression Profiling to Understand the	PLoS One	2013
Cadmium	WHSC2	Zelmina Lubovac-Pilav, et al	Using Expression Profiling to Understand the	PLoS One	2013
Cadmium	LRRC8C	Zelmina Lubovac-Pilav, et al	Using Expression Profiling to Understand the	PLoS One	2013
Cadmium	RASGRP1	Zelmina Lubovac-Pilav, et al	Using Expression Profiling to Understand the	PLoS One	2013
Cadmium	PPIL4	Zelmina Lubovac-Pilav, et al	Using Expression Profiling to Understand the	PLoS One	2013
Cadmium	IFI30	Zelmina Lubovac-Pilav, et al	Using Expression Profiling to Understand the	PLoS One	2013
Cadmium	EZR	Zelmina Lubovac-Pilav, et al	Using Expression Profiling to Understand the	PLoS One	2013
Cadmium	SURF6	Zelmina Lubovac-Pilav, et al	Using Expression Profiling to Understand the	PLoS One	2013
Cadmium	LOC728819	Zelmina Lubovac-Pilav, et al	Using Expression Profiling to Understand the	PLoS One	2013
Cadmium	SLC9A8	Zelmina Lubovac-Pilav, et al	Using Expression Profiling to Understand the	PLoS One	2013
Cadmium	TUBB3	Zelmina Lubovac-Pilav, et al	Using Expression Profiling to Understand the	PLoS One	2013
Cadmium	GAA	Zelmina Lubovac-Pilav, et al	Using Expression Profiling to Understand the	PLoS One	2013
Cadmium	SOLH	Zelmina Lubovac-Pilav, et al	Using Expression Profiling to Understand the	PLoS One	2013
Cadmium	RREB1	Zelmina Lubovac-Pilav, et al	Using Expression Profiling to Understand the	PLoS One	2013
Cadmium	NAA20	Zelmina Lubovac-Pilav, et al	Using Expression Profiling to Understand the	PLoS One	2013
Cadmium	FLJ45248	Zelmina Lubovac-Pilav, et al	Using Expression Profiling to Understand the	PLoS One	2013
Cadmium	ZNF816	Zelmina Lubovac-Pilav, et al	Using Expression Profiling to Understand the	PLoS One	2013
Cadmium	MTSS1L	Zelmina Lubovac-Pilav, et al	Using Expression Profiling to Understand the	PLoS One	2013

Cadmium	SLC25A15	Zelmina Lubovac-Pilav, et al	Using Expression Profiling to Understand the	PLoS One	2013
Cadmium	STX2	Zelmina Lubovac-Pilav, et al	Using Expression Profiling to Understand the	PLoS One	2013
Cadmium	PRKCZ	Zelmina Lubovac-Pilav, et al	Using Expression Profiling to Understand the	PLoS One	2013
Cadmium	PPP1R13L	Zelmina Lubovac-Pilav, et al	Using Expression Profiling to Understand the	PLoS One	2013
Cadmium	HES6	Zelmina Lubovac-Pilav, et al	Using Expression Profiling to Understand the	PLoS One	2013
Cadmium	C11ORF96	Zelmina Lubovac-Pilav, et al	Using Expression Profiling to Understand the	PLoS One	2013
Cadmium	CHPF2	Zelmina Lubovac-Pilav, et al	Using Expression Profiling to Understand the	PLoS One	2013
Cadmium	PCNP	Zelmina Lubovac-Pilav, et al	Using Expression Profiling to Understand the	PLoS One	2013
Cadmium	CCDC96	Zelmina Lubovac-Pilav, et al	Using Expression Profiling to Understand the	PLoS One	2013
Cadmium	VPS37B	Zelmina Lubovac-Pilav, et al	Using Expression Profiling to Understand the	PLoS One	2013
Cadmium	TPM1	Zelmina Lubovac-Pilav, et al	Using Expression Profiling to Understand the	PLoS One	2013
Cadmium	FAM155B	Zelmina Lubovac-Pilav, et al	Using Expression Profiling to Understand the	PLoS One	2013
Cadmium	PDCD2L	Zelmina Lubovac-Pilav, et al	Using Expression Profiling to Understand the	PLoS One	2013
Cadmium	CHST8	Zelmina Lubovac-Pilav, et al	Using Expression Profiling to Understand the	PLoS One	2013
Cadmium	RUFY1	Zelmina Lubovac-Pilav, et al	Using Expression Profiling to Understand the	PLoS One	2013
Cadmium	GPR107	Zelmina Lubovac-Pilav, et al	Using Expression Profiling to Understand the	PLoS One	2013
Cadmium	ARHGDIA	Zelmina Lubovac-Pilav, et al	Using Expression Profiling to Understand the	PLoS One	2013
Cadmium	IPO8	Zelmina Lubovac-Pilav, et al	Using Expression Profiling to Understand the	PLoS One	2013
Cadmium	CKB	Zelmina Lubovac-Pilav, et al	Using Expression Profiling to Understand the	PLoS One	2013
Cadmium	HLA-B	Zelmina Lubovac-Pilav, et al	Using Expression Profiling to Understand the	PLoS One	2013
Cadmium	SPON2	Zelmina Lubovac-Pilav, et al	Using Expression Profiling to Understand the	PLoS One	2013
Cadmium	ATP6V0B	Zelmina Lubovac-Pilav, et al	Using Expression Profiling to Understand the	PLoS One	2013
Cadmium	PQLC2	Zelmina Lubovac-Pilav, et al	Using Expression Profiling to Understand the	PLoS One	2013
Cadmium	DIS3	Zelmina Lubovac-Pilav, et al	Using Expression Profiling to Understand the	PLoS One	2013
Cadmium	PPP1R14C	Zelmina Lubovac-Pilav, et al	Using Expression Profiling to Understand the	PLoS One	2013
Cadmium	NCRNA00188	Zelmina Lubovac-Pilav, et al	Using Expression Profiling to Understand the	PLoS One	2013
Cadmium	ALDH4A1	Zelmina Lubovac-Pilav, et al	Using Expression Profiling to Understand the	PLoS One	2013
Cadmium	TSPYL4	Zelmina Lubovac-Pilav, et al	Using Expression Profiling to Understand the	PLoS One	2013
Cadmium	FADS3	Zelmina Lubovac-Pilav, et al	Using Expression Profiling to Understand the	PLoS One	2013
Cadmium	FOXL2	Zelmina Lubovac-Pilav, et al	Using Expression Profiling to Understand the	PLoS One	2013
Cadmium	RING1	Zelmina Lubovac-Pilav, et al	Using Expression Profiling to Understand the	PLoS One	2013
Cadmium	RNF135	Zelmina Lubovac-Pilav, et al	Using Expression Profiling to Understand the	PLoS One	2013
Cadmium	SAMD1	Zelmina Lubovac-Pilav, et al	Using Expression Profiling to Understand the	PLoS One	2013
Cadmium	NUDT16L1	Zelmina Lubovac-Pilav, et al	Using Expression Profiling to Understand the	PLoS One	2013
Cadmium	MRPS14	Zelmina Lubovac-Pilav, et al	Using Expression Profiling to Understand the	PLoS One	2013
Cadmium	PALMD	Zelmina Lubovac-Pilav, et al	Using Expression Profiling to Understand the	PLoS One	2013
Cadmium	RPUSD1	Zelmina Lubovac-Pilav, et al	Using Expression Profiling to Understand the	PLoS One	2013
Cadmium	STAC2	Zelmina Lubovac-Pilav, et al	Using Expression Profiling to Understand the	PLoS One	2013
Cadmium	KIF1C	Zelmina Lubovac-Pilav, et al	Using Expression Profiling to Understand the	PLoS One	2013
Cadmium	PPCS	Zelmina Lubovac-Pilav, et al	Using Expression Profiling to Understand the	PLoS One	2013
Cadmium	MEX3D	Zelmina Lubovac-Pilav, et al	Using Expression Profiling to Understand the	PLoS One	2013
Cadmium	TMEM132A	Zelmina Lubovac-Pilav, et al	Using Expression Profiling to Understand the	PLoS One	2013
Cadmium	PIGQ	Zelmina Lubovac-Pilav, et al	Using Expression Profiling to Understand the	PLoS One	2013
Cadmium	FBRS	Zelmina Lubovac-Pilav, et al	Using Expression Profiling to Understand the	PLoS One	2013
Cadmium	PAQR4	Zelmina Lubovac-Pilav, et al	Using Expression Profiling to Understand the	PLoS One	2013
Cadmium	EPHB2	Zelmina Lubovac-Pilav, et al	Using Expression Profiling to Understand the	PLoS One	2013
Cadmium	SEPW1	Zelmina Lubovac-Pilav, et al	Using Expression Profiling to Understand the	PLoS One	2013
Cadmium	MAP4	Zelmina Lubovac-Pilav, et al	Using Expression Profiling to Understand the	PLoS One	2013
Cadmium	FAHD1	Zelmina Lubovac-Pilav, et al	Using Expression Profiling to Understand the	PLoS One	2013
Cadmium	RNF8	Zelmina Lubovac-Pilav, et al	Using Expression Profiling to Understand the	PLoS One	2013
Cadmium	ADCY7	Zelmina Lubovac-Pilav, et al	Using Expression Profiling to Understand the	PLoS One	2013
Cadmium	MT1M	Zelmina Lubovac-Pilav, et al	Using Expression Profiling to Understand the	PLoS One	2013
Cadmium	RHEB	Zelmina Lubovac-Pilav, et al	Using Expression Profiling to Understand the	PLoS One	2013
Cadmium	PCYOX1L	Zelmina Lubovac-Pilav, et al	Using Expression Profiling to Understand the	PLoS One	2013
Cadmium	ZNF282	Zelmina Lubovac-Pilav, et al	Using Expression Profiling to Understand the	PLoS One	2013
Cadmium	LZTS2	Zelmina Lubovac-Pilav, et al	Using Expression Profiling to Understand the	PLoS One	2013
Cadmium	ZMIZ1	Zelmina Lubovac-Pilav, et al	Using Expression Profiling to Understand the	PLoS One	2013
Cadmium	RFFL	Zelmina Lubovac-Pilav, et al	Using Expression Profiling to Understand the	PLoS One	2013
Cadmium	ORMDL2	Zelmina Lubovac-Pilav, et al	Using Expression Profiling to Understand the	PLoS One	2013
Cadmium	FEM1B	Zelmina Lubovac-Pilav, et al	Using Expression Profiling to Understand the	PLoS One	2013
Cadmium	TMEM79	Zelmina Lubovac-Pilav, et al	Using Expression Profiling to Understand the	PLoS One	2013
Cadmium	PRR7	Zelmina Lubovac-Pilav, et al	Using Expression Profiling to Understand the	PLoS One	2013
Cadmium	PIGC	Zelmina Lubovac-Pilav, et al	Using Expression Profiling to Understand the	PLoS One	2013
Cadmium	FECH	Zelmina Lubovac-Pilav, et al	Using Expression Profiling to Understand the	PLoS One	2013
Cadmium	RAB40B	Zelmina Lubovac-Pilav, et al	Using Expression Profiling to Understand the	PLoS One	2013
Cadmium	DAG1	Zelmina Lubovac-Pilav, et al	Using Expression Profiling to Understand the	PLoS One	2013
Cadmium	HEXB	Zelmina Lubovac-Pilav, et al	Using Expression Profiling to Understand the	PLoS One	2013
Cadmium	ZNF709	Zelmina Lubovac-Pilav, et al	Using Expression Profiling to Understand the	PLoS One	2013
Cadmium	KRT19	Zelmina Lubovac-Pilav, et al	Using Expression Profiling to Understand the	PLoS One	2013
Cadmium	PAX2	Zelmina Lubovac-Pilav, et al	Using Expression Profiling to Understand the	PLoS One	2013
Cadmium	PPIL5	Zelmina Lubovac-Pilav, et al	Using Expression Profiling to Understand the	PLoS One	2013
Cadmium	NPRL3	Zelmina Lubovac-Pilav, et al	Using Expression Profiling to Understand the	PLoS One	2013
Cadmium	ERF	Zelmina Lubovac-Pilav, et al	Using Expression Profiling to Understand the	PLoS One	2013
Cadmium	RNF187	Zelmina Lubovac-Pilav, et al	Using Expression Profiling to Understand the	PLoS One	2013
Cadmium	ASPHD2	Zelmina Lubovac-Pilav, et al	Using Expression Profiling to Understand the	PLoS One	2013
Cadmium	EXOSC4	Zelmina Lubovac-Pilav, et al	Using Expression Profiling to Understand the	PLoS One	2013
Cadmium	NEDD8	Zelmina Lubovac-Pilav, et al	Using Expression Profiling to Understand the	PLoS One	2013
Cadmium	SLC29A2	Zelmina Lubovac-Pilav, et al	Using Expression Profiling to Understand the	PLoS One	2013
Cadmium	S1PR2	Zelmina Lubovac-Pilav, et al	Using Expression Profiling to Understand the	PLoS One	2013
Cadmium	RPL28	Zelmina Lubovac-Pilav, et al	Using Expression Profiling to Understand the	PLoS One	2013
Cadmium	MCF2L	Zelmina Lubovac-Pilav, et al	Using Expression Profiling to Understand the	PLoS One	2013
Cadmium	ADAM15	Zelmina Lubovac-Pilav, et al	Using Expression Profiling to Understand the	PLoS One	2013
Cadmium	ZNF187	Zelmina Lubovac-Pilav, et al	Using Expression Profiling to Understand the	PLoS One	2013
Cadmium	RABGGTA	Zelmina Lubovac-Pilav, et al	Using Expression Profiling to Understand the	PLoS One	2013
Cadmium	ADCK3	Zelmina Lubovac-Pilav, et al	Using Expression Profiling to Understand the	PLoS One	2013
Cadmium	PGRMC2	Zelmina Lubovac-Pilav, et al	Using Expression Profiling to Understand the	PLoS One	2013
Cadmium	ARHGAP17	Zelmina Lubovac-Pilav, et al	Using Expression Profiling to Understand the	PLoS One	2013
Cadmium	ATAT1	Zelmina Lubovac-Pilav, et al	Using Expression Profiling to Understand the	PLoS One	2013
Cadmium	ZNF554	Zelmina Lubovac-Pilav, et al	Using Expression Profiling to Understand the	PLoS One	2013

Cadmium	HOXA7	Zelmina Lubovac-Pilav, et al	Using Expression Profiling to Understand the	PLoS One	2013
Cadmium	GRTP1	Zelmina Lubovac-Pilav, et al	Using Expression Profiling to Understand the	PLoS One	2013
Cadmium	HILS1	Zelmina Lubovac-Pilav, et al	Using Expression Profiling to Understand the	PLoS One	2013
Cadmium	INTS5	Zelmina Lubovac-Pilav, et al	Using Expression Profiling to Understand the	PLoS One	2013
Cadmium	MARS2	Zelmina Lubovac-Pilav, et al	Using Expression Profiling to Understand the	PLoS One	2013
Cadmium	ZNF467	Zelmina Lubovac-Pilav, et al	Using Expression Profiling to Understand the	PLoS One	2013
Cadmium	MS4A12	Zelmina Lubovac-Pilav, et al	Using Expression Profiling to Understand the	PLoS One	2013
Cadmium	RNF4	Zelmina Lubovac-Pilav, et al	Using Expression Profiling to Understand the	PLoS One	2013
Cadmium	KISS1R	Zelmina Lubovac-Pilav, et al	Using Expression Profiling to Understand the	PLoS One	2013
Cadmium	KCNK4	Zelmina Lubovac-Pilav, et al	Using Expression Profiling to Understand the	PLoS One	2013
Cadmium	RNF208	Zelmina Lubovac-Pilav, et al	Using Expression Profiling to Understand the	PLoS One	2013
Cadmium	IMP3	Zelmina Lubovac-Pilav, et al	Using Expression Profiling to Understand the	PLoS One	2013
Cadmium	JDP2	Zelmina Lubovac-Pilav, et al	Using Expression Profiling to Understand the	PLoS One	2013
Cadmium	DOT1L	Zelmina Lubovac-Pilav, et al	Using Expression Profiling to Understand the	PLoS One	2013
Cadmium	COL22A1	Zelmina Lubovac-Pilav, et al	Using Expression Profiling to Understand the	PLoS One	2013
Cadmium	CIB3	Zelmina Lubovac-Pilav, et al	Using Expression Profiling to Understand the	PLoS One	2013
Cadmium	NUP210L	Zelmina Lubovac-Pilav, et al	Using Expression Profiling to Understand the	PLoS One	2013
Cadmium	LRRC34	Zelmina Lubovac-Pilav, et al	Using Expression Profiling to Understand the	PLoS One	2013
Cadmium	GNAO1	Zelmina Lubovac-Pilav, et al	Using Expression Profiling to Understand the	PLoS One	2013
Cadmium	PKDCC	Zelmina Lubovac-Pilav, et al	Using Expression Profiling to Understand the	PLoS One	2013
Cadmium	SERTAD4	Zelmina Lubovac-Pilav, et al	Using Expression Profiling to Understand the	PLoS One	2013
Cadmium	OR10J1	Zelmina Lubovac-Pilav, et al	Using Expression Profiling to Understand the	PLoS One	2013
Cadmium	FOLR2	Zelmina Lubovac-Pilav, et al	Using Expression Profiling to Understand the	PLoS One	2013
Cadmium	SLC44A4	Zelmina Lubovac-Pilav, et al	Using Expression Profiling to Understand the	PLoS One	2013
Cadmium	SERPINB2	Zelmina Lubovac-Pilav, et al	Using Expression Profiling to Understand the	PLoS One	2013
Cadmium	OR2Y1	Zelmina Lubovac-Pilav, et al	Using Expression Profiling to Understand the	PLoS One	2013
Cadmium	PTPRC	Zelmina Lubovac-Pilav, et al	Using Expression Profiling to Understand the	PLoS One	2013
Cadmium	USP50	Zelmina Lubovac-Pilav, et al	Using Expression Profiling to Understand the	PLoS One	2013
Cadmium	F8	Zelmina Lubovac-Pilav, et al	Using Expression Profiling to Understand the	PLoS One	2013
Cadmium	RUNDC3B	Zelmina Lubovac-Pilav, et al	Using Expression Profiling to Understand the	PLoS One	2013
Cadmium	ZNF831	Zelmina Lubovac-Pilav, et al	Using Expression Profiling to Understand the	PLoS One	2013
Cadmium	KIF18B	Zelmina Lubovac-Pilav, et al	Using Expression Profiling to Understand the	PLoS One	2013
Cadmium	SEPT6	Zelmina Lubovac-Pilav, et al	Using Expression Profiling to Understand the	PLoS One	2013
Cadmium	ANKRD20A3	Zelmina Lubovac-Pilav, et al	Using Expression Profiling to Understand the	PLoS One	2013
Cadmium	CX3CR1	Zelmina Lubovac-Pilav, et al	Using Expression Profiling to Understand the	PLoS One	2013
Cadmium	ALS2CR12	Zelmina Lubovac-Pilav, et al	Using Expression Profiling to Understand the	PLoS One	2013
Cadmium	CD3G	Zelmina Lubovac-Pilav, et al	Using Expression Profiling to Understand the	PLoS One	2013
Cadmium	UBE2D4	Zelmina Lubovac-Pilav, et al	Using Expression Profiling to Understand the	PLoS One	2013
Cadmium	ABCC11	Zelmina Lubovac-Pilav, et al	Using Expression Profiling to Understand the	PLoS One	2013
Cadmium	TRIM17	Zelmina Lubovac-Pilav, et al	Using Expression Profiling to Understand the	PLoS One	2013
Cadmium	THPO	Zelmina Lubovac-Pilav, et al	Using Expression Profiling to Understand the	PLoS One	2013
Cadmium	GPR26	Zelmina Lubovac-Pilav, et al	Using Expression Profiling to Understand the	PLoS One	2013
Cadmium	FBXO3	Zelmina Lubovac-Pilav, et al	Using Expression Profiling to Understand the	PLoS One	2013
Cadmium	SLC16A12	Zelmina Lubovac-Pilav, et al	Using Expression Profiling to Understand the	PLoS One	2013
Cadmium	FAM74A3	Zelmina Lubovac-Pilav, et al	Using Expression Profiling to Understand the	PLoS One	2013
Cadmium	OR51B4	Zelmina Lubovac-Pilav, et al	Using Expression Profiling to Understand the	PLoS One	2013
Cadmium	BTN2A1	Zelmina Lubovac-Pilav, et al	Using Expression Profiling to Understand the	PLoS One	2013
Cadmium	GOLGA6L9	Zelmina Lubovac-Pilav, et al	Using Expression Profiling to Understand the	PLoS One	2013
Cadmium	NUP35	Zelmina Lubovac-Pilav, et al	Using Expression Profiling to Understand the	PLoS One	2013
Cadmium	LGALS4	Zelmina Lubovac-Pilav, et al	Using Expression Profiling to Understand the	PLoS One	2013
Cadmium	MST1P2	Zelmina Lubovac-Pilav, et al	Using Expression Profiling to Understand the	PLoS One	2013
Cadmium	DSCAML1	Zelmina Lubovac-Pilav, et al	Using Expression Profiling to Understand the	PLoS One	2013
Cadmium	GSTM2P1	Zelmina Lubovac-Pilav, et al	Using Expression Profiling to Understand the	PLoS One	2013
Cadmium	VSNL1	Zelmina Lubovac-Pilav, et al	Using Expression Profiling to Understand the	PLoS One	2013
Cadmium	ARHGAP19	Zelmina Lubovac-Pilav, et al	Using Expression Profiling to Understand the	PLoS One	2013
Cadmium	MIR206	Zelmina Lubovac-Pilav, et al	Using Expression Profiling to Understand the	PLoS One	2013
Cadmium	WFDC13	Zelmina Lubovac-Pilav, et al	Using Expression Profiling to Understand the	PLoS One	2013
Cadmium	MIR19B2	Zelmina Lubovac-Pilav, et al	Using Expression Profiling to Understand the	PLoS One	2013
Cadmium	TLR4	Zelmina Lubovac-Pilav, et al	Using Expression Profiling to Understand the	PLoS One	2013
Cadmium	SPANXE	Zelmina Lubovac-Pilav, et al	Using Expression Profiling to Understand the	PLoS One	2013
Cadmium	LRP2	Zelmina Lubovac-Pilav, et al	Using Expression Profiling to Understand the	PLoS One	2013
Cadmium	KIF22	Zelmina Lubovac-Pilav, et al	Using Expression Profiling to Understand the	PLoS One	2013
Cadmium	TAP2	Zelmina Lubovac-Pilav, et al	Using Expression Profiling to Understand the	PLoS One	2013
Cadmium	PDE1C	Zelmina Lubovac-Pilav, et al	Using Expression Profiling to Understand the	PLoS One	2013
Cadmium	FLJ40448	Zelmina Lubovac-Pilav, et al	Using Expression Profiling to Understand the	PLoS One	2013
Cadmium	C6ORF227	Zelmina Lubovac-Pilav, et al	Using Expression Profiling to Understand the	PLoS One	2013
Cadmium	F11	Zelmina Lubovac-Pilav, et al	Using Expression Profiling to Understand the	PLoS One	2013
Cadmium	PAGE2	Zelmina Lubovac-Pilav, et al	Using Expression Profiling to Understand the	PLoS One	2013
Cadmium	TRIB1	Zelmina Lubovac-Pilav, et al	Using Expression Profiling to Understand the	PLoS One	2013
Cadmium	FER1L6	Zelmina Lubovac-Pilav, et al	Using Expression Profiling to Understand the	PLoS One	2013
Cadmium	HCK	Zelmina Lubovac-Pilav, et al	Using Expression Profiling to Understand the	PLoS One	2013
Cadmium	FCAMR	Zelmina Lubovac-Pilav, et al	Using Expression Profiling to Understand the	PLoS One	2013
Cadmium	ARHGAP30	Zelmina Lubovac-Pilav, et al	Using Expression Profiling to Understand the	PLoS One	2013
Cadmium	MYBPC2	Zelmina Lubovac-Pilav, et al	Using Expression Profiling to Understand the	PLoS One	2013
Cadmium	ANGPT2	Zelmina Lubovac-Pilav, et al	Using Expression Profiling to Understand the	PLoS One	2013
Cadmium	TREML1	Zelmina Lubovac-Pilav, et al	Using Expression Profiling to Understand the	PLoS One	2013
Cadmium	ADORA3	Zelmina Lubovac-Pilav, et al	Using Expression Profiling to Understand the	PLoS One	2013
Cadmium	SNORD115-40	Zelmina Lubovac-Pilav, et al	Using Expression Profiling to Understand the	PLoS One	2013
Cadmium	PPP1R16B	Zelmina Lubovac-Pilav, et al	Using Expression Profiling to Understand the	PLoS One	2013
Cadmium	DCTN6	Zelmina Lubovac-Pilav, et al	Using Expression Profiling to Understand the	PLoS One	2013
Cadmium	GPR119	Zelmina Lubovac-Pilav, et al	Using Expression Profiling to Understand the	PLoS One	2013
Cadmium	NPIP	Zelmina Lubovac-Pilav, et al	Using Expression Profiling to Understand the	PLoS One	2013
Cadmium	NUDT1	Zelmina Lubovac-Pilav, et al	Using Expression Profiling to Understand the	PLoS One	2013
Cadmium	PRSS38	Zelmina Lubovac-Pilav, et al	Using Expression Profiling to Understand the	PLoS One	2013
Cadmium	BTC	Zelmina Lubovac-Pilav, et al	Using Expression Profiling to Understand the	PLoS One	2013
Cadmium	MPL	Zelmina Lubovac-Pilav, et al	Using Expression Profiling to Understand the	PLoS One	2013
Cadmium	DPF3	Zelmina Lubovac-Pilav, et al	Using Expression Profiling to Understand the	PLoS One	2013
Cadmium	OR5AK3P	Zelmina Lubovac-Pilav, et al	Using Expression Profiling to Understand the	PLoS One	2013
Cadmium	AAA1	Zelmina Lubovac-Pilav, et al	Using Expression Profiling to Understand the	PLoS One	2013

Cadmium	PIK3CG	Zelmina Lubovac-Pilav, et al	Using Expression Profiling to Understand the	PLoS One	2013
Cadmium	MIR129-2	Zelmina Lubovac-Pilav, et al	Using Expression Profiling to Understand the	PLoS One	2013
Cadmium	MAGEB18	Zelmina Lubovac-Pilav, et al	Using Expression Profiling to Understand the	PLoS One	2013
Cadmium	RNF39	Zelmina Lubovac-Pilav, et al	Using Expression Profiling to Understand the	PLoS One	2013
Cadmium	PPIAL4A	Zelmina Lubovac-Pilav, et al	Using Expression Profiling to Understand the	PLoS One	2013
Cadmium	C1D	Zelmina Lubovac-Pilav, et al	Using Expression Profiling to Understand the	PLoS One	2013
Cadmium	CXORF40B	Zelmina Lubovac-Pilav, et al	Using Expression Profiling to Understand the	PLoS One	2013
Cadmium	PLGLA	Zelmina Lubovac-Pilav, et al	Using Expression Profiling to Understand the	PLoS One	2013
Cadmium	ADAM5P	Zelmina Lubovac-Pilav, et al	Using Expression Profiling to Understand the	PLoS One	2013
Cadmium	TPTE	Zelmina Lubovac-Pilav, et al	Using Expression Profiling to Understand the	PLoS One	2013
Cadmium	FAM90A25P	Zelmina Lubovac-Pilav, et al	Using Expression Profiling to Understand the	PLoS One	2013
Cadmium	EDDM3B	Zelmina Lubovac-Pilav, et al	Using Expression Profiling to Understand the	PLoS One	2013
Cadmium	KIR2DS4	Zelmina Lubovac-Pilav, et al	Using Expression Profiling to Understand the	PLoS One	2013
Cadmium	GP6	Zelmina Lubovac-Pilav, et al	Using Expression Profiling to Understand the	PLoS One	2013
Cadmium	PLA2G2A	Zelmina Lubovac-Pilav, et al	Using Expression Profiling to Understand the	PLoS One	2013
Cadmium	HIST2H2BF	Zelmina Lubovac-Pilav, et al	Using Expression Profiling to Understand the	PLoS One	2013
Cadmium	FAM90A7	Zelmina Lubovac-Pilav, et al	Using Expression Profiling to Understand the	PLoS One	2013
Cadmium	LBR	Zelmina Lubovac-Pilav, et al	Using Expression Profiling to Understand the	PLoS One	2013
Cadmium	BCAS1	Zelmina Lubovac-Pilav, et al	Using Expression Profiling to Understand the	PLoS One	2013
Cadmium	C16ORF73	Zelmina Lubovac-Pilav, et al	Using Expression Profiling to Understand the	PLoS One	2013
Cadmium	ABCA9	Zelmina Lubovac-Pilav, et al	Using Expression Profiling to Understand the	PLoS One	2013
Cadmium	GPR4	Zelmina Lubovac-Pilav, et al	Using Expression Profiling to Understand the	PLoS One	2013
Cadmium	AGAP5	Zelmina Lubovac-Pilav, et al	Using Expression Profiling to Understand the	PLoS One	2013
Cadmium	GABRA6	Zelmina Lubovac-Pilav, et al	Using Expression Profiling to Understand the	PLoS One	2013
Cadmium	CIT	Zelmina Lubovac-Pilav, et al	Using Expression Profiling to Understand the	PLoS One	2013
Cadmium	RNLS	Zelmina Lubovac-Pilav, et al	Using Expression Profiling to Understand the	PLoS One	2013
Cadmium	CHAT	Zelmina Lubovac-Pilav, et al	Using Expression Profiling to Understand the	PLoS One	2013
Cadmium	MYF5	Zelmina Lubovac-Pilav, et al	Using Expression Profiling to Understand the	PLoS One	2013
Cadmium	CRP	Zelmina Lubovac-Pilav, et al	Using Expression Profiling to Understand the	PLoS One	2013
Cadmium	ATP7A	Zelmina Lubovac-Pilav, et al	Using Expression Profiling to Understand the	PLoS One	2013
Cadmium	ATF3	Zelmina Lubovac-Pilav, et al	Using Expression Profiling to Understand the	PLoS One	2013
Cadmium	ZNF404	Zelmina Lubovac-Pilav, et al	Using Expression Profiling to Understand the	PLoS One	2013
Cadmium	FCGR2B	Zelmina Lubovac-Pilav, et al	Using Expression Profiling to Understand the	PLoS One	2013
Cadmium	NANOG	Zelmina Lubovac-Pilav, et al	Using Expression Profiling to Understand the	PLoS One	2013
Cadmium	SLAMF8	Zelmina Lubovac-Pilav, et al	Using Expression Profiling to Understand the	PLoS One	2013
Cadmium	C1ORF129	Zelmina Lubovac-Pilav, et al	Using Expression Profiling to Understand the	PLoS One	2013
Cadmium	ZDHHC9	Zelmina Lubovac-Pilav, et al	Using Expression Profiling to Understand the	PLoS One	2013
Cadmium	PLGLB2	Zelmina Lubovac-Pilav, et al	Using Expression Profiling to Understand the	PLoS One	2013
Cadmium	CPN1	Zelmina Lubovac-Pilav, et al	Using Expression Profiling to Understand the	PLoS One	2013
Cadmium	NBPF4	Zelmina Lubovac-Pilav, et al	Using Expression Profiling to Understand the	PLoS One	2013
Cadmium	MIR1247	Zelmina Lubovac-Pilav, et al	Using Expression Profiling to Understand the	PLoS One	2013
Cadmium	EMR1	Zelmina Lubovac-Pilav, et al	Using Expression Profiling to Understand the	PLoS One	2013
Cadmium	AGAP4	Zelmina Lubovac-Pilav, et al	Using Expression Profiling to Understand the	PLoS One	2013
Cadmium	BTF3P9	Zelmina Lubovac-Pilav, et al	Using Expression Profiling to Understand the	PLoS One	2013
Cadmium	CKS1B	Zelmina Lubovac-Pilav, et al	Using Expression Profiling to Understand the	PLoS One	2013
Cadmium	SDCCAG1	Zelmina Lubovac-Pilav, et al	Using Expression Profiling to Understand the	PLoS One	2013
Cadmium	C15ORF21	Zelmina Lubovac-Pilav, et al	Using Expression Profiling to Understand the	PLoS One	2013
Cadmium	FBLIM1	Zelmina Lubovac-Pilav, et al	Using Expression Profiling to Understand the	PLoS One	2013
Cadmium	GABPB1	Zelmina Lubovac-Pilav, et al	Using Expression Profiling to Understand the	PLoS One	2013
Cadmium	ZFAND2A	Zelmina Lubovac-Pilav, et al	Using Expression Profiling to Understand the	PLoS One	2013
Cadmium	ATP8B4	Zelmina Lubovac-Pilav, et al	Using Expression Profiling to Understand the	PLoS One	2013
Cadmium	OR4D5	Zelmina Lubovac-Pilav, et al	Using Expression Profiling to Understand the	PLoS One	2013
Cadmium	NBPF6	Zelmina Lubovac-Pilav, et al	Using Expression Profiling to Understand the	PLoS One	2013
Cadmium	WEE1	Zelmina Lubovac-Pilav, et al	Using Expression Profiling to Understand the	PLoS One	2013
Cadmium	LRRTM4	Zelmina Lubovac-Pilav, et al	Using Expression Profiling to Understand the	PLoS One	2013
Cadmium	CENPA	Zelmina Lubovac-Pilav, et al	Using Expression Profiling to Understand the	PLoS One	2013
Cadmium	CADPS	Zelmina Lubovac-Pilav, et al	Using Expression Profiling to Understand the	PLoS One	2013
Cadmium	SELE	Zelmina Lubovac-Pilav, et al	Using Expression Profiling to Understand the	PLoS One	2013
Cadmium	DFNA5	Zelmina Lubovac-Pilav, et al	Using Expression Profiling to Understand the	PLoS One	2013
Cadmium	C7ORF66	Zelmina Lubovac-Pilav, et al	Using Expression Profiling to Understand the	PLoS One	2013
Cadmium	PRAMEF5	Zelmina Lubovac-Pilav, et al	Using Expression Profiling to Understand the	PLoS One	2013
Cadmium	FAM55B	Zelmina Lubovac-Pilav, et al	Using Expression Profiling to Understand the	PLoS One	2013
Cadmium	LGSN	Zelmina Lubovac-Pilav, et al	Using Expression Profiling to Understand the	PLoS One	2013
Cadmium	LOC440993	Zelmina Lubovac-Pilav, et al	Using Expression Profiling to Understand the	PLoS One	2013
Cadmium	KIR2DL2	Zelmina Lubovac-Pilav, et al	Using Expression Profiling to Understand the	PLoS One	2013
Cadmium	C8ORF59	Zelmina Lubovac-Pilav, et al	Using Expression Profiling to Understand the	PLoS One	2013
Cadmium	C20ORF12	Zelmina Lubovac-Pilav, et al	Using Expression Profiling to Understand the	PLoS One	2013
Cadmium	DRD5	Zelmina Lubovac-Pilav, et al	Using Expression Profiling to Understand the	PLoS One	2013
Cadmium	GPCPD1	Zelmina Lubovac-Pilav, et al	Using Expression Profiling to Understand the	PLoS One	2013
Cadmium	CPO	Zelmina Lubovac-Pilav, et al	Using Expression Profiling to Understand the	PLoS One	2013
Cadmium	PRG4	Zelmina Lubovac-Pilav, et al	Using Expression Profiling to Understand the	PLoS One	2013
Cadmium	MAGEB16	Zelmina Lubovac-Pilav, et al	Using Expression Profiling to Understand the	PLoS One	2013
Cadmium	MTMR11	Zelmina Lubovac-Pilav, et al	Using Expression Profiling to Understand the	PLoS One	2013
Cadmium	FRG2C	Zelmina Lubovac-Pilav, et al	Using Expression Profiling to Understand the	PLoS One	2013
Cadmium	GIF	Zelmina Lubovac-Pilav, et al	Using Expression Profiling to Understand the	PLoS One	2013
Cadmium	OIP5	Zelmina Lubovac-Pilav, et al	Using Expression Profiling to Understand the	PLoS One	2013
Cadmium	AGAP7	Zelmina Lubovac-Pilav, et al	Using Expression Profiling to Understand the	PLoS One	2013
Cadmium	CENPM	Zelmina Lubovac-Pilav, et al	Using Expression Profiling to Understand the	PLoS One	2013
Cadmium	GOLGA6L10	Zelmina Lubovac-Pilav, et al	Using Expression Profiling to Understand the	PLoS One	2013
Cadmium	FAM122B	Zelmina Lubovac-Pilav, et al	Using Expression Profiling to Understand the	PLoS One	2013
Cadmium	C11ORF40	Zelmina Lubovac-Pilav, et al	Using Expression Profiling to Understand the	PLoS One	2013
Cadmium	HIST2H3A	Zelmina Lubovac-Pilav, et al	Using Expression Profiling to Understand the	PLoS One	2013
Cadmium	ANKRD30BP2	Zelmina Lubovac-Pilav, et al	Using Expression Profiling to Understand the	PLoS One	2013
Cadmium	VAMP7	Zelmina Lubovac-Pilav, et al	Using Expression Profiling to Understand the	PLoS One	2013
Cadmium	RGSL1	Zelmina Lubovac-Pilav, et al	Using Expression Profiling to Understand the	PLoS One	2013
Cadmium	NMU	Zelmina Lubovac-Pilav, et al	Using Expression Profiling to Understand the	PLoS One	2013
Cadmium	CCNF	Zelmina Lubovac-Pilav, et al	Using Expression Profiling to Understand the	PLoS One	2013
Cadmium	EPGN	Zelmina Lubovac-Pilav, et al	Using Expression Profiling to Understand the	PLoS One	2013
Cadmium	RSPH10B	Zelmina Lubovac-Pilav, et al	Using Expression Profiling to Understand the	PLoS One	2013

Cadmium	DUX4L4	Zelmina Lubovac-Pilav, et al	Using Expression Profiling to Understand the	PLoS One	2013
Cadmium	TAC3	Zelmina Lubovac-Pilav, et al	Using Expression Profiling to Understand the	PLoS One	2013
Cadmium	C14ORF38	Zelmina Lubovac-Pilav, et al	Using Expression Profiling to Understand the	PLoS One	2013
Cadmium	KAT2B	Zelmina Lubovac-Pilav, et al	Using Expression Profiling to Understand the	PLoS One	2013
Cadmium	C15ORF57	Zelmina Lubovac-Pilav, et al	Using Expression Profiling to Understand the	PLoS One	2013
Cadmium	HSH2D	Zelmina Lubovac-Pilav, et al	Using Expression Profiling to Understand the	PLoS One	2013
Cadmium	ARID3B	Zelmina Lubovac-Pilav, et al	Using Expression Profiling to Understand the	PLoS One	2013
Cadmium	SLC5A12	Zelmina Lubovac-Pilav, et al	Using Expression Profiling to Understand the	PLoS One	2013
Cadmium	FLJ45340	Zelmina Lubovac-Pilav, et al	Using Expression Profiling to Understand the	PLoS One	2013
Cadmium	TBC1D3F	Zelmina Lubovac-Pilav, et al	Using Expression Profiling to Understand the	PLoS One	2013
Cadmium	ATXN7	Zelmina Lubovac-Pilav, et al	Using Expression Profiling to Understand the	PLoS One	2013
Cadmium	MLLT11	Zelmina Lubovac-Pilav, et al	Using Expression Profiling to Understand the	PLoS One	2013
Cadmium	EMR3	Zelmina Lubovac-Pilav, et al	Using Expression Profiling to Understand the	PLoS One	2013
Cadmium	OR10H3	Zelmina Lubovac-Pilav, et al	Using Expression Profiling to Understand the	PLoS One	2013
Cadmium	TFCP2	Zelmina Lubovac-Pilav, et al	Using Expression Profiling to Understand the	PLoS One	2013
Cadmium	OR5AN1	Zelmina Lubovac-Pilav, et al	Using Expression Profiling to Understand the	PLoS One	2013
Cadmium	SLC26A10	Zelmina Lubovac-Pilav, et al	Using Expression Profiling to Understand the	PLoS One	2013
Cadmium	EDA2R	Zelmina Lubovac-Pilav, et al	Using Expression Profiling to Understand the	PLoS One	2013
Cadmium	CA14	Zelmina Lubovac-Pilav, et al	Using Expression Profiling to Understand the	PLoS One	2013
Cadmium	MYBPH	Zelmina Lubovac-Pilav, et al	Using Expression Profiling to Understand the	PLoS One	2013
Cadmium	TBC1D3B	Zelmina Lubovac-Pilav, et al	Using Expression Profiling to Understand the	PLoS One	2013
Cadmium	POM121L2	Zelmina Lubovac-Pilav, et al	Using Expression Profiling to Understand the	PLoS One	2013
Cadmium	SH3GL3	Zelmina Lubovac-Pilav, et al	Using Expression Profiling to Understand the	PLoS One	2013
Cadmium	NXF5	Zelmina Lubovac-Pilav, et al	Using Expression Profiling to Understand the	PLoS One	2013
Cadmium	GSTM5	Zelmina Lubovac-Pilav, et al	Using Expression Profiling to Understand the	PLoS One	2013
Cadmium	APOL5	Zelmina Lubovac-Pilav, et al	Using Expression Profiling to Understand the	PLoS One	2013
Cadmium	FSTL5	Zelmina Lubovac-Pilav, et al	Using Expression Profiling to Understand the	PLoS One	2013
Cadmium	RBP2	Zelmina Lubovac-Pilav, et al	Using Expression Profiling to Understand the	PLoS One	2013
Cadmium	HIST2H3D	Zelmina Lubovac-Pilav, et al	Using Expression Profiling to Understand the	PLoS One	2013
Cadmium	GAP43	Zelmina Lubovac-Pilav, et al	Using Expression Profiling to Understand the	PLoS One	2013
Cadmium	ARHGEF6	Zelmina Lubovac-Pilav, et al	Using Expression Profiling to Understand the	PLoS One	2013
Cadmium	SETD8	Zelmina Lubovac-Pilav, et al	Using Expression Profiling to Understand the	PLoS One	2013
Cadmium	NLRP7	Zelmina Lubovac-Pilav, et al	Using Expression Profiling to Understand the	PLoS One	2013
Cadmium	PLEKHH2	Zelmina Lubovac-Pilav, et al	Using Expression Profiling to Understand the	PLoS One	2013
Cadmium	ZNF256	Zelmina Lubovac-Pilav, et al	Using Expression Profiling to Understand the	PLoS One	2013
Cadmium	OR5B3	Zelmina Lubovac-Pilav, et al	Using Expression Profiling to Understand the	PLoS One	2013
Cadmium	GUSBP3	Zelmina Lubovac-Pilav, et al	Using Expression Profiling to Understand the	PLoS One	2013
Cadmium	XKR9	Zelmina Lubovac-Pilav, et al	Using Expression Profiling to Understand the	PLoS One	2013
Cadmium	SLC25A5	Zelmina Lubovac-Pilav, et al	Using Expression Profiling to Understand the	PLoS One	2013
Cadmium	AMICA1	Zelmina Lubovac-Pilav, et al	Using Expression Profiling to Understand the	PLoS One	2013
Cadmium	C1ORF137	Zelmina Lubovac-Pilav, et al	Using Expression Profiling to Understand the	PLoS One	2013
Cadmium	PSRC1	Zelmina Lubovac-Pilav, et al	Using Expression Profiling to Understand the	PLoS One	2013
Cadmium	NUP62CL	Zelmina Lubovac-Pilav, et al	Using Expression Profiling to Understand the	PLoS One	2013
Cadmium	OR1L8	Zelmina Lubovac-Pilav, et al	Using Expression Profiling to Understand the	PLoS One	2013
Cadmium	IGHM	Zelmina Lubovac-Pilav, et al	Using Expression Profiling to Understand the	PLoS One	2013
Cadmium	NLGN4X	Zelmina Lubovac-Pilav, et al	Using Expression Profiling to Understand the	PLoS One	2013
Cadmium	CSTL1	Zelmina Lubovac-Pilav, et al	Using Expression Profiling to Understand the	PLoS One	2013
Cadmium	GUSBP1	Zelmina Lubovac-Pilav, et al	Using Expression Profiling to Understand the	PLoS One	2013
Cadmium	SMA5	Zelmina Lubovac-Pilav, et al	Using Expression Profiling to Understand the	PLoS One	2013
Cadmium	ITIH4	Zelmina Lubovac-Pilav, et al	Using Expression Profiling to Understand the	PLoS One	2013
Cadmium	AMELX	Zelmina Lubovac-Pilav, et al	Using Expression Profiling to Understand the	PLoS One	2013
Cadmium	DUX4	Zelmina Lubovac-Pilav, et al	Using Expression Profiling to Understand the	PLoS One	2013
Cadmium	TBC1D3	Zelmina Lubovac-Pilav, et al	Using Expression Profiling to Understand the	PLoS One	2013
Cadmium	OR1L3	Zelmina Lubovac-Pilav, et al	Using Expression Profiling to Understand the	PLoS One	2013
Cadmium	OR4C46	Zelmina Lubovac-Pilav, et al	Using Expression Profiling to Understand the	PLoS One	2013
Cadmium	C12ORF28	Zelmina Lubovac-Pilav, et al	Using Expression Profiling to Understand the	PLoS One	2013
Cadmium	TXNDC2	Zelmina Lubovac-Pilav, et al	Using Expression Profiling to Understand the	PLoS One	2013
Cadmium	LHFP	Zelmina Lubovac-Pilav, et al	Using Expression Profiling to Understand the	PLoS One	2013
Cadmium	LY6G6C	Zelmina Lubovac-Pilav, et al	Using Expression Profiling to Understand the	PLoS One	2013
Cadmium	C1ORF63	Zelmina Lubovac-Pilav, et al	Using Expression Profiling to Understand the	PLoS One	2013
Cadmium	OR4X1	Zelmina Lubovac-Pilav, et al	Using Expression Profiling to Understand the	PLoS One	2013
Cadmium	OR4K1	Zelmina Lubovac-Pilav, et al	Using Expression Profiling to Understand the	PLoS One	2013
Cadmium	C6ORF97	Zelmina Lubovac-Pilav, et al	Using Expression Profiling to Understand the	PLoS One	2013
Cadmium	OR3A1	Zelmina Lubovac-Pilav, et al	Using Expression Profiling to Understand the	PLoS One	2013
Cadmium	VIT	Zelmina Lubovac-Pilav, et al	Using Expression Profiling to Understand the	PLoS One	2013
Cadmium	FLJ27243	Zelmina Lubovac-Pilav, et al	Using Expression Profiling to Understand the	PLoS One	2013
Cadmium	MIR181B1	Zelmina Lubovac-Pilav, et al	Using Expression Profiling to Understand the	PLoS One	2013
Cadmium	TBC1D3G	Zelmina Lubovac-Pilav, et al	Using Expression Profiling to Understand the	PLoS One	2013
Cadmium	PRAMEF1	Zelmina Lubovac-Pilav, et al	Using Expression Profiling to Understand the	PLoS One	2013
Cadmium	DCD	Zelmina Lubovac-Pilav, et al	Using Expression Profiling to Understand the	PLoS One	2013
Cadmium	RAB3C	Zelmina Lubovac-Pilav, et al	Using Expression Profiling to Understand the	PLoS One	2013
Cadmium	C15ORF23	Zelmina Lubovac-Pilav, et al	Using Expression Profiling to Understand the	PLoS One	2013
Cadmium	NPIPL3	Zelmina Lubovac-Pilav, et al	Using Expression Profiling to Understand the	PLoS One	2013
Cadmium	IFI44	Zelmina Lubovac-Pilav, et al	Using Expression Profiling to Understand the	PLoS One	2013
Cadmium	MIRLET7B	Zelmina Lubovac-Pilav, et al	Using Expression Profiling to Understand the	PLoS One	2013
Cadmium	COX17	Zelmina Lubovac-Pilav, et al	Using Expression Profiling to Understand the	PLoS One	2013
Cadmium	FAM27E3	Zelmina Lubovac-Pilav, et al	Using Expression Profiling to Understand the	PLoS One	2013
Cadmium	TMEM27	Zelmina Lubovac-Pilav, et al	Using Expression Profiling to Understand the	PLoS One	2013
Cadmium	C8ORF4	Zelmina Lubovac-Pilav, et al	Using Expression Profiling to Understand the	PLoS One	2013
Cadmium	DDX26B	Zelmina Lubovac-Pilav, et al	Using Expression Profiling to Understand the	PLoS One	2013
Cadmium	CCNB1	Zelmina Lubovac-Pilav, et al	Using Expression Profiling to Understand the	PLoS One	2013
Cadmium	OR6C75	Zelmina Lubovac-Pilav, et al	Using Expression Profiling to Understand the	PLoS One	2013
Cadmium	TBC1D3P2	Zelmina Lubovac-Pilav, et al	Using Expression Profiling to Understand the	PLoS One	2013
Cadmium	TDGF3	Zelmina Lubovac-Pilav, et al	Using Expression Profiling to Understand the	PLoS One	2013
Cadmium	GPR21	Zelmina Lubovac-Pilav, et al	Using Expression Profiling to Understand the	PLoS One	2013
Cadmium	KIR2DS2	Zelmina Lubovac-Pilav, et al	Using Expression Profiling to Understand the	PLoS One	2013
Cadmium	SNORA58	Zelmina Lubovac-Pilav, et al	Using Expression Profiling to Understand the	PLoS One	2013
Cadmium	HEMGN	Zelmina Lubovac-Pilav, et al	Using Expression Profiling to Understand the	PLoS One	2013
Cadmium	GPX5	Zelmina Lubovac-Pilav, et al	Using Expression Profiling to Understand the	PLoS One	2013

Cadmium	LOC100131860	Zelmina Lubovac-Pilav, et al	Using Expression Profiling to Understand the	PLoS One	2013
Cadmium	GAS2L3	Zelmina Lubovac-Pilav, et al	Using Expression Profiling to Understand the	PLoS One	2013
Cadmium	GPR64	Zelmina Lubovac-Pilav, et al	Using Expression Profiling to Understand the	PLoS One	2013
Cadmium	SPRYD5	Zelmina Lubovac-Pilav, et al	Using Expression Profiling to Understand the	PLoS One	2013
Cadmium	MIR32	Zelmina Lubovac-Pilav, et al	Using Expression Profiling to Understand the	PLoS One	2013
Cadmium	DEFB108B	Zelmina Lubovac-Pilav, et al	Using Expression Profiling to Understand the	PLoS One	2013
Cadmium	FAM177B	Zelmina Lubovac-Pilav, et al	Using Expression Profiling to Understand the	PLoS One	2013
Cadmium	TBC1D29	Zelmina Lubovac-Pilav, et al	Using Expression Profiling to Understand the	PLoS One	2013
Cadmium	NPSR1	Zelmina Lubovac-Pilav, et al	Using Expression Profiling to Understand the	PLoS One	2013
Cadmium	TK1	Zelmina Lubovac-Pilav, et al	Using Expression Profiling to Understand the	PLoS One	2013
Cadmium	ECRP	Zelmina Lubovac-Pilav, et al	Using Expression Profiling to Understand the	PLoS One	2013
Cadmium	ANP32E	Zelmina Lubovac-Pilav, et al	Using Expression Profiling to Understand the	PLoS One	2013
Cadmium	NXF4	Zelmina Lubovac-Pilav, et al	Using Expression Profiling to Understand the	PLoS One	2013
Cadmium	PRAMEF13	Zelmina Lubovac-Pilav, et al	Using Expression Profiling to Understand the	PLoS One	2013
Cadmium	TCEB3B	Zelmina Lubovac-Pilav, et al	Using Expression Profiling to Understand the	PLoS One	2013
Cadmium	HMGN5	Zelmina Lubovac-Pilav, et al	Using Expression Profiling to Understand the	PLoS One	2013
Cadmium	SNORD115-1	Zelmina Lubovac-Pilav, et al	Using Expression Profiling to Understand the	PLoS One	2013
Cadmium	MUC7	Zelmina Lubovac-Pilav, et al	Using Expression Profiling to Understand the	PLoS One	2013
Cadmium	PECAM1	Zelmina Lubovac-Pilav, et al	Using Expression Profiling to Understand the	PLoS One	2013
Cadmium	SNORD115-6	Zelmina Lubovac-Pilav, et al	Using Expression Profiling to Understand the	PLoS One	2013
Cadmium	MUC3A	Zelmina Lubovac-Pilav, et al	Using Expression Profiling to Understand the	PLoS One	2013
Cadmium	NCRNA00277	Zelmina Lubovac-Pilav, et al	Using Expression Profiling to Understand the	PLoS One	2013
Cadmium	RACGAP1	Zelmina Lubovac-Pilav, et al	Using Expression Profiling to Understand the	PLoS One	2013
Cadmium	ETV5	Zelmina Lubovac-Pilav, et al	Using Expression Profiling to Understand the	PLoS One	2013
Cadmium	NCAPD2	Zelmina Lubovac-Pilav, et al	Using Expression Profiling to Understand the	PLoS One	2013
Cadmium	CELA2B	Zelmina Lubovac-Pilav, et al	Using Expression Profiling to Understand the	PLoS One	2013
Cadmium	LOC100127951	Zelmina Lubovac-Pilav, et al	Using Expression Profiling to Understand the	PLoS One	2013
Cadmium	OSTN	Zelmina Lubovac-Pilav, et al	Using Expression Profiling to Understand the	PLoS One	2013
Cadmium	RPL21P44	Zelmina Lubovac-Pilav, et al	Using Expression Profiling to Understand the	PLoS One	2013
Cadmium	RHEBL1	Zelmina Lubovac-Pilav, et al	Using Expression Profiling to Understand the	PLoS One	2013
Cadmium	RNF183	Zelmina Lubovac-Pilav, et al	Using Expression Profiling to Understand the	PLoS One	2013
Cadmium	CELA1	Zelmina Lubovac-Pilav, et al	Using Expression Profiling to Understand the	PLoS One	2013
Cadmium	CYP19A1	Zelmina Lubovac-Pilav, et al	Using Expression Profiling to Understand the	PLoS One	2013
Cadmium	DDX4	Zelmina Lubovac-Pilav, et al	Using Expression Profiling to Understand the	PLoS One	2013
Cadmium	ARGFX	Zelmina Lubovac-Pilav, et al	Using Expression Profiling to Understand the	PLoS One	2013
Cadmium	ARHGAP23	Zelmina Lubovac-Pilav, et al	Using Expression Profiling to Understand the	PLoS One	2013
Cadmium	SNORD115-42	Zelmina Lubovac-Pilav, et al	Using Expression Profiling to Understand the	PLoS One	2013
Cadmium	CA4	Zelmina Lubovac-Pilav, et al	Using Expression Profiling to Understand the	PLoS One	2013
Cadmium	IL1RAPL1	Zelmina Lubovac-Pilav, et al	Using Expression Profiling to Understand the	PLoS One	2013
Cadmium	SPDYE2	Zelmina Lubovac-Pilav, et al	Using Expression Profiling to Understand the	PLoS One	2013
Cadmium	C17ORF64	Zelmina Lubovac-Pilav, et al	Using Expression Profiling to Understand the	PLoS One	2013
Cadmium	MIR509-1	Zelmina Lubovac-Pilav, et al	Using Expression Profiling to Understand the	PLoS One	2013
Cadmium	SLC7A11	Zelmina Lubovac-Pilav, et al	Using Expression Profiling to Understand the	PLoS One	2013
Cadmium	GK	Zelmina Lubovac-Pilav, et al	Using Expression Profiling to Understand the	PLoS One	2013
Cadmium	AMY1A	Zelmina Lubovac-Pilav, et al	Using Expression Profiling to Understand the	PLoS One	2013
Cadmium	C1ORF161	Zelmina Lubovac-Pilav, et al	Using Expression Profiling to Understand the	PLoS One	2013
Cadmium	PRR20A	Zelmina Lubovac-Pilav, et al	Using Expression Profiling to Understand the	PLoS One	2013
Cadmium	C11ORF34	Zelmina Lubovac-Pilav, et al	Using Expression Profiling to Understand the	PLoS One	2013
Cadmium	LOC440896	Zelmina Lubovac-Pilav, et al	Using Expression Profiling to Understand the	PLoS One	2013
Cadmium	SH3BGRL	Zelmina Lubovac-Pilav, et al	Using Expression Profiling to Understand the	PLoS One	2013
Cadmium	TMEM31	Zelmina Lubovac-Pilav, et al	Using Expression Profiling to Understand the	PLoS One	2013
Cadmium	GPR128	Zelmina Lubovac-Pilav, et al	Using Expression Profiling to Understand the	PLoS One	2013
Cadmium	OR2T3	Zelmina Lubovac-Pilav, et al	Using Expression Profiling to Understand the	PLoS One	2013
Cadmium	OR4Q3	Zelmina Lubovac-Pilav, et al	Using Expression Profiling to Understand the	PLoS One	2013
Cadmium	FRG2B	Zelmina Lubovac-Pilav, et al	Using Expression Profiling to Understand the	PLoS One	2013
Cadmium	MST4	Zelmina Lubovac-Pilav, et al	Using Expression Profiling to Understand the	PLoS One	2013
Cadmium	DEPDC1B	Zelmina Lubovac-Pilav, et al	Using Expression Profiling to Understand the	PLoS One	2013
Cadmium	SLC7A7	Zelmina Lubovac-Pilav, et al	Using Expression Profiling to Understand the	PLoS One	2013
Cadmium	SPDYE5	Zelmina Lubovac-Pilav, et al	Using Expression Profiling to Understand the	PLoS One	2013
Cadmium	OR52D1	Zelmina Lubovac-Pilav, et al	Using Expression Profiling to Understand the	PLoS One	2013
Cadmium	CHI3L1	Zelmina Lubovac-Pilav, et al	Using Expression Profiling to Understand the	PLoS One	2013
Cadmium	OR7E125P	Zelmina Lubovac-Pilav, et al	Using Expression Profiling to Understand the	PLoS One	2013
Cadmium	RNU2-1	Zelmina Lubovac-Pilav, et al	Using Expression Profiling to Understand the	PLoS One	2013
Cadmium	GOLGA6L5	Zelmina Lubovac-Pilav, et al	Using Expression Profiling to Understand the	PLoS One	2013
Cadmium	LRRC37A2	Zelmina Lubovac-Pilav, et al	Using Expression Profiling to Understand the	PLoS One	2013
Cadmium	SPZ1	Zelmina Lubovac-Pilav, et al	Using Expression Profiling to Understand the	PLoS One	2013
Cadmium	MIR138-1	Zelmina Lubovac-Pilav, et al	Using Expression Profiling to Understand the	PLoS One	2013
Cadmium	PKD1P1	Zelmina Lubovac-Pilav, et al	Using Expression Profiling to Understand the	PLoS One	2013
Cadmium	BUB1	Zelmina Lubovac-Pilav, et al	Using Expression Profiling to Understand the	PLoS One	2013
Cadmium	LPAR6	Zelmina Lubovac-Pilav, et al	Using Expression Profiling to Understand the	PLoS One	2013
Cadmium	KIF14	Zelmina Lubovac-Pilav, et al	Using Expression Profiling to Understand the	PLoS One	2013
Cadmium	OR4F16	Zelmina Lubovac-Pilav, et al	Using Expression Profiling to Understand the	PLoS One	2013
Cadmium	C1ORF51	Zelmina Lubovac-Pilav, et al	Using Expression Profiling to Understand the	PLoS One	2013
Cadmium	VHL	Zelmina Lubovac-Pilav, et al	Using Expression Profiling to Understand the	PLoS One	2013
Cadmium	CENPK	Zelmina Lubovac-Pilav, et al	Using Expression Profiling to Understand the	PLoS One	2013
Cadmium	FAM138A	Zelmina Lubovac-Pilav, et al	Using Expression Profiling to Understand the	PLoS One	2013
Cadmium	OR4F21	Zelmina Lubovac-Pilav, et al	Using Expression Profiling to Understand the	PLoS One	2013
Cadmium	SPDYE7P	Zelmina Lubovac-Pilav, et al	Using Expression Profiling to Understand the	PLoS One	2013
Cadmium	CT45A3	Zelmina Lubovac-Pilav, et al	Using Expression Profiling to Understand the	PLoS One	2013
Cadmium	DOK5	Zelmina Lubovac-Pilav, et al	Using Expression Profiling to Understand the	PLoS One	2013
Cadmium	ABCA6	Zelmina Lubovac-Pilav, et al	Using Expression Profiling to Understand the	PLoS One	2013
Cadmium	ZIM3	Zelmina Lubovac-Pilav, et al	Using Expression Profiling to Understand the	PLoS One	2013
Cadmium	FAM106C	Zelmina Lubovac-Pilav, et al	Using Expression Profiling to Understand the	PLoS One	2013
Cadmium	C20ORF62	Zelmina Lubovac-Pilav, et al	Using Expression Profiling to Understand the	PLoS One	2013
Cadmium	SLITRK6	Zelmina Lubovac-Pilav, et al	Using Expression Profiling to Understand the	PLoS One	2013
Cadmium	LOC93622	Zelmina Lubovac-Pilav, et al	Using Expression Profiling to Understand the	PLoS One	2013
Cadmium	SNCG	Zelmina Lubovac-Pilav, et al	Using Expression Profiling to Understand the	PLoS One	2013
Cadmium	OR6S1	Zelmina Lubovac-Pilav, et al	Using Expression Profiling to Understand the	PLoS One	2013
Cadmium	FAM138B	Zelmina Lubovac-Pilav, et al	Using Expression Profiling to Understand the	PLoS One	2013

Cadmium	SNORD115-30	Zelmina Lubovac-Pilav, et al	Using Expression Profiling to Understand the	PLoS One	2013
Cadmium	AMPD1	Zelmina Lubovac-Pilav, et al	Using Expression Profiling to Understand the	PLoS One	2013
Cadmium	SPDYE1	Zelmina Lubovac-Pilav, et al	Using Expression Profiling to Understand the	PLoS One	2013
Cadmium	TRIM49	Zelmina Lubovac-Pilav, et al	Using Expression Profiling to Understand the	PLoS One	2013
Cadmium	LOC349196	Zelmina Lubovac-Pilav, et al	Using Expression Profiling to Understand the	PLoS One	2013
Cadmium	SIAE	Zelmina Lubovac-Pilav, et al	Using Expression Profiling to Understand the	PLoS One	2013
Cadmium	FAM72D	Zelmina Lubovac-Pilav, et al	Using Expression Profiling to Understand the	PLoS One	2013
Cadmium	KIR3DL3	Zelmina Lubovac-Pilav, et al	Using Expression Profiling to Understand the	PLoS One	2013
Cadmium	CT45A5	Zelmina Lubovac-Pilav, et al	Using Expression Profiling to Understand the	PLoS One	2013
Cadmium	NUSAP1	Zelmina Lubovac-Pilav, et al	Using Expression Profiling to Understand the	PLoS One	2013
Cadmium	LOC100128364	Zelmina Lubovac-Pilav, et al	Using Expression Profiling to Understand the	PLoS One	2013
Cadmium	C20ORF107	Zelmina Lubovac-Pilav, et al	Using Expression Profiling to Understand the	PLoS One	2013
Cadmium	LOC729461	Zelmina Lubovac-Pilav, et al	Using Expression Profiling to Understand the	PLoS One	2013
Cadmium	PGK1	Zelmina Lubovac-Pilav, et al	Using Expression Profiling to Understand the	PLoS One	2013
Cadmium	SNORD104	Zelmina Lubovac-Pilav, et al	Using Expression Profiling to Understand the	PLoS One	2013
Cadmium	KIF4A	Zelmina Lubovac-Pilav, et al	Using Expression Profiling to Understand the	PLoS One	2013
Cadmium	ANKRD20B	Zelmina Lubovac-Pilav, et al	Using Expression Profiling to Understand the	PLoS One	2013
Cadmium	LOC400968	Zelmina Lubovac-Pilav, et al	Using Expression Profiling to Understand the	PLoS One	2013
Cadmium	CSTF2T	Zelmina Lubovac-Pilav, et al	Using Expression Profiling to Understand the	PLoS One	2013
Cadmium	OR2T34	Zelmina Lubovac-Pilav, et al	Using Expression Profiling to Understand the	PLoS One	2013
Cadmium	CT45A4	Zelmina Lubovac-Pilav, et al	Using Expression Profiling to Understand the	PLoS One	2013
Cadmium	REXO1L2P	Zelmina Lubovac-Pilav, et al	Using Expression Profiling to Understand the	PLoS One	2013
Cadmium	TAF9B	Zelmina Lubovac-Pilav, et al	Using Expression Profiling to Understand the	PLoS One	2013
Cadmium	SMR3A	Zelmina Lubovac-Pilav, et al	Using Expression Profiling to Understand the	PLoS One	2013
Cadmium	CASP14	Zelmina Lubovac-Pilav, et al	Using Expression Profiling to Understand the	PLoS One	2013
Cadmium	CD1A	Zelmina Lubovac-Pilav, et al	Using Expression Profiling to Understand the	PLoS One	2013
Cadmium	MIR21	Zelmina Lubovac-Pilav, et al	Using Expression Profiling to Understand the	PLoS One	2013
Cadmium	LOC51152	Zelmina Lubovac-Pilav, et al	Using Expression Profiling to Understand the	PLoS One	2013
Cadmium	CT45A2	Zelmina Lubovac-Pilav, et al	Using Expression Profiling to Understand the	PLoS One	2013
Cadmium	CT45A6	Zelmina Lubovac-Pilav, et al	Using Expression Profiling to Understand the	PLoS One	2013
Cadmium	FST	Zelmina Lubovac-Pilav, et al	Using Expression Profiling to Understand the	PLoS One	2013
Cadmium	SPDYE8P	Zelmina Lubovac-Pilav, et al	Using Expression Profiling to Understand the	PLoS One	2013
Cadmium	SNORD115-20	Zelmina Lubovac-Pilav, et al	Using Expression Profiling to Understand the	PLoS One	2013
Cadmium	EGR1	Zelmina Lubovac-Pilav, et al	Using Expression Profiling to Understand the	PLoS One	2013
Cadmium	FAM182A	Zelmina Lubovac-Pilav, et al	Using Expression Profiling to Understand the	PLoS One	2013
Cadmium	SSX2	Zelmina Lubovac-Pilav, et al	Using Expression Profiling to Understand the	PLoS One	2013
Cadmium	OR8B2	Zelmina Lubovac-Pilav, et al	Using Expression Profiling to Understand the	PLoS One	2013
Cadmium	CENPF	Zelmina Lubovac-Pilav, et al	Using Expression Profiling to Understand the	PLoS One	2013
Cadmium	OR1J1	Zelmina Lubovac-Pilav, et al	Using Expression Profiling to Understand the	PLoS One	2013
Cadmium	REXO1L1	Zelmina Lubovac-Pilav, et al	Using Expression Profiling to Understand the	PLoS One	2013
Cadmium	OR8B3	Zelmina Lubovac-Pilav, et al	Using Expression Profiling to Understand the	PLoS One	2013
Cadmium	TSPAN16	Zelmina Lubovac-Pilav, et al	Using Expression Profiling to Understand the	PLoS One	2013
Cadmium	HCG8	Zelmina Lubovac-Pilav, et al	Using Expression Profiling to Understand the	PLoS One	2013
Cadmium	LOC284757	Zelmina Lubovac-Pilav, et al	Using Expression Profiling to Understand the	PLoS One	2013
Cadmium	GDPD1	Zelmina Lubovac-Pilav, et al	Using Expression Profiling to Understand the	PLoS One	2013
Cadmium	OR1D5	Zelmina Lubovac-Pilav, et al	Using Expression Profiling to Understand the	PLoS One	2013
Cadmium	GAGE12J	Zelmina Lubovac-Pilav, et al	Using Expression Profiling to Understand the	PLoS One	2013
Cadmium	GOLGA6L1	Zelmina Lubovac-Pilav, et al	Using Expression Profiling to Understand the	PLoS One	2013
Cadmium	LOC645188	Zelmina Lubovac-Pilav, et al	Using Expression Profiling to Understand the	PLoS One	2013
Cadmium	GAGE12I	Zelmina Lubovac-Pilav, et al	Using Expression Profiling to Understand the	PLoS One	2013
Cadmium	IGKC	Zelmina Lubovac-Pilav, et al	Using Expression Profiling to Understand the	PLoS One	2013
Cadmium	OR2AT4	Zelmina Lubovac-Pilav, et al	Using Expression Profiling to Understand the	PLoS One	2013
Cadmium	GAGE12G	Zelmina Lubovac-Pilav, et al	Using Expression Profiling to Understand the	PLoS One	2013
Cadmium	AMY2A	Zelmina Lubovac-Pilav, et al	Using Expression Profiling to Understand the	PLoS One	2013
Cadmium	NDUFS7	Zelmina Lubovac-Pilav, et al	Using Expression Profiling to Understand the	PLoS One	2013
Cadmium	SSX4	Zelmina Lubovac-Pilav, et al	Using Expression Profiling to Understand the	PLoS One	2013
Cadmium	CD3D	Zelmina Lubovac-Pilav, et al	Using Expression Profiling to Understand the	PLoS One	2013
Cadmium	AQP7P1	Zelmina Lubovac-Pilav, et al	Using Expression Profiling to Understand the	PLoS One	2013
Cadmium	LOC151009	Zelmina Lubovac-Pilav, et al	Using Expression Profiling to Understand the	PLoS One	2013
Cadmium	FLJ11710	Zelmina Lubovac-Pilav, et al	Using Expression Profiling to Understand the	PLoS One	2013
Cadmium	OCM2	Zelmina Lubovac-Pilav, et al	Using Expression Profiling to Understand the	PLoS One	2013
Cadmium	CADPS2	Zelmina Lubovac-Pilav, et al	Using Expression Profiling to Understand the	PLoS One	2013
Cadmium	TBX4	Zelmina Lubovac-Pilav, et al	Using Expression Profiling to Understand the	PLoS One	2013
Cadmium	C17ORF47	Zelmina Lubovac-Pilav, et al	Using Expression Profiling to Understand the	PLoS One	2013
Cadmium	OR7E87P	Zelmina Lubovac-Pilav, et al	Using Expression Profiling to Understand the	PLoS One	2013
Cadmium	GAGE12B	Zelmina Lubovac-Pilav, et al	Using Expression Profiling to Understand the	PLoS One	2013
Cadmium	DENND2C	Zelmina Lubovac-Pilav, et al	Using Expression Profiling to Understand the	PLoS One	2013
Cadmium	IGKC	Zelmina Lubovac-Pilav, et al	Using Expression Profiling to Understand the	PLoS One	2013
Cadmium	PSITPTE22	Zelmina Lubovac-Pilav, et al	Using Expression Profiling to Understand the	PLoS One	2013
Cadmium	LOC652493	Zelmina Lubovac-Pilav, et al	Using Expression Profiling to Understand the	PLoS One	2013
Cadmium	OCM	Zelmina Lubovac-Pilav, et al	Using Expression Profiling to Understand the	PLoS One	2013
Cadmium	CYP24A1	Zelmina Lubovac-Pilav, et al	Using Expression Profiling to Understand the	PLoS One	2013
Cadmium	C1ORF54	Zelmina Lubovac-Pilav, et al	Using Expression Profiling to Understand the	PLoS One	2013
Cadmium	SNORD115-12	Zelmina Lubovac-Pilav, et al	Using Expression Profiling to Understand the	PLoS One	2013
Cadmium	PRR11	Zelmina Lubovac-Pilav, et al	Using Expression Profiling to Understand the	PLoS One	2013
Cadmium	ANTXR2	Zelmina Lubovac-Pilav, et al	Using Expression Profiling to Understand the	PLoS One	2013
Cadmium	SNORD115-26	Zelmina Lubovac-Pilav, et al	Using Expression Profiling to Understand the	PLoS One	2013
Cadmium	LOC100130713	Zelmina Lubovac-Pilav, et al	Using Expression Profiling to Understand the	PLoS One	2013
Cadmium	SNORD59B	Zelmina Lubovac-Pilav, et al	Using Expression Profiling to Understand the	PLoS One	2013
Cadmium	CDR1	Zelmina Lubovac-Pilav, et al	Using Expression Profiling to Understand the	PLoS One	2013
Cadmium	SNORD115-11	Zelmina Lubovac-Pilav, et al	Using Expression Profiling to Understand the	PLoS One	2013
Cadmium	IGLJ3	Zelmina Lubovac-Pilav, et al	Using Expression Profiling to Understand the	PLoS One	2013
Cadmium	AVIL	Zelmina Lubovac-Pilav, et al	Using Expression Profiling to Understand the	PLoS One	2013
Cadmium	IGHA1	Zelmina Lubovac-Pilav, et al	Using Expression Profiling to Understand the	PLoS One	2013
Cadmium	NUPR1	Zelmina Lubovac-Pilav, et al	Using Expression Profiling to Understand the	PLoS One	2013
Cadmium	KCNK2	Zelmina Lubovac-Pilav, et al	Using Expression Profiling to Understand the	PLoS One	2013
Cadmium	CLEC3A	Zelmina Lubovac-Pilav, et al	Using Expression Profiling to Understand the	PLoS One	2013
Cadmium	LOC100132147	Zelmina Lubovac-Pilav, et al	Using Expression Profiling to Understand the	PLoS One	2013
Cadmium	MIR222	Zelmina Lubovac-Pilav, et al	Using Expression Profiling to Understand the	PLoS One	2013

Cadmium	XCL1	Zelmina Lubovac-Pilav, et al	Using Expression Profiling to Understand the	PLoS One	2013
Cadmium	DUB3	Zelmina Lubovac-Pilav, et al	Using Expression Profiling to Understand the	PLoS One	2013
Cadmium	DUB4	Zelmina Lubovac-Pilav, et al	Using Expression Profiling to Understand the	PLoS One	2013
Cadmium	MUC12	Zelmina Lubovac-Pilav, et al	Using Expression Profiling to Understand the	PLoS One	2013
Cadmium	USP17	Zelmina Lubovac-Pilav, et al	Using Expression Profiling to Understand the	PLoS One	2013
Cadmium	IGHD	Zelmina Lubovac-Pilav, et al	Using Expression Profiling to Understand the	PLoS One	2013
Cadmium	USP17L6P	Zelmina Lubovac-Pilav, et al	Using Expression Profiling to Understand the	PLoS One	2013
Cadmium	KIR2DL3	Zelmina Lubovac-Pilav, et al	Using Expression Profiling to Understand the	PLoS One	2013
Cadmium	LOC440570	Zelmina Lubovac-Pilav, et al	Using Expression Profiling to Understand the	PLoS One	2013
Cadmium	MT1JP	Marco Fabbri et al	Whole genome analysis and microRNAs	Alternatives to Animal Experimentation	2012
Cadmium	HSPA6	Marco Fabbri et al	Whole genome analysis and microRNAs	Alternatives to Animal Experimentation	2012
Cadmium	GADD45G	Marco Fabbri et al	Whole genome analysis and microRNAs	Alternatives to Animal Experimentation	2012
Cadmium	RRAD	Marco Fabbri et al	Whole genome analysis and microRNAs	Alternatives to Animal Experimentation	2012
Cadmium	MT1G	Marco Fabbri et al	Whole genome analysis and microRNAs	Alternatives to Animal Experimentation	2012
Cadmium	KRT37	Marco Fabbri et al	Whole genome analysis and microRNAs	Alternatives to Animal Experimentation	2012
Cadmium	HTRA3	Marco Fabbri et al	Whole genome analysis and microRNAs	Alternatives to Animal Experimentation	2012
Cadmium	MT1E	Marco Fabbri et al	Whole genome analysis and microRNAs	Alternatives to Animal Experimentation	2012
Cadmium	CRYAB	Marco Fabbri et al	Whole genome analysis and microRNAs	Alternatives to Animal Experimentation	2012
Cadmium	MT1X	Marco Fabbri et al	Whole genome analysis and microRNAs	Alternatives to Animal Experimentation	2012
Cadmium	GADD45B	Marco Fabbri et al	Whole genome analysis and microRNAs	Alternatives to Animal Experimentation	2012
Cadmium	MT1M	Marco Fabbri et al	Whole genome analysis and microRNAs	Alternatives to Animal Experimentation	2012
Cadmium	MT1B	Marco Fabbri et al	Whole genome analysis and microRNAs	Alternatives to Animal Experimentation	2012
Cadmium	LOC730755	Marco Fabbri et al	Whole genome analysis and microRNAs	Alternatives to Animal Experimentation	2012
Cadmium	SNAI1	Marco Fabbri et al	Whole genome analysis and microRNAs	Alternatives to Animal Experimentation	2012
Cadmium	MT1H	Marco Fabbri et al	Whole genome analysis and microRNAs	Alternatives to Animal Experimentation	2012
Cadmium	CCL26	Marco Fabbri et al	Whole genome analysis and microRNAs	Alternatives to Animal Experimentation	2012
Cadmium	LAMB3	Marco Fabbri et al	Whole genome analysis and microRNAs	Alternatives to Animal Experimentation	2012
Cadmium	MLC1	Marco Fabbri et al	Whole genome analysis and microRNAs	Alternatives to Animal Experimentation	2012
Cadmium	ARC	Marco Fabbri et al	Whole genome analysis and microRNAs	Alternatives to Animal Experimentation	2012
Cadmium	MT1L	Marco Fabbri et al	Whole genome analysis and microRNAs	Alternatives to Animal Experimentation	2012
Cadmium	NPPB	Marco Fabbri et al	Whole genome analysis and microRNAs	Alternatives to Animal Experimentation	2012
Cadmium	MT1F	Marco Fabbri et al	Whole genome analysis and microRNAs	Alternatives to Animal Experimentation	2012
Cadmium	IL11	Marco Fabbri et al	Whole genome analysis and microRNAs	Alternatives to Animal Experimentation	2012
Cadmium	EGR1	Marco Fabbri et al	Whole genome analysis and microRNAs	Alternatives to Animal Experimentation	2012
Cadmium	ASB2	Marco Fabbri et al	Whole genome analysis and microRNAs	Alternatives to Animal Experimentation	2012
Cadmium	MT1A	Marco Fabbri et al	Whole genome analysis and microRNAs	Alternatives to Animal Experimentation	2012
Cadmium	HSPA1A	Marco Fabbri et al	Whole genome analysis and microRNAs	Alternatives to Animal Experimentation	2012
Cadmium	UCHL1	Marco Fabbri et al	Whole genome analysis and microRNAs	Alternatives to Animal Experimentation	2012
Cadmium	KRTAP3-1	Marco Fabbri et al	Whole genome analysis and microRNAs	Alternatives to Animal Experimentation	2012
Cadmium	EDN1	Marco Fabbri et al	Whole genome analysis and microRNAs	Alternatives to Animal Experimentation	2012
Cadmium	TNS4	Marco Fabbri et al	Whole genome analysis and microRNAs	Alternatives to Animal Experimentation	2012
Cadmium	RGS2	Marco Fabbri et al	Whole genome analysis and microRNAs	Alternatives to Animal Experimentation	2012
Cadmium	AKAP12	Marco Fabbri et al	Whole genome analysis and microRNAs	Alternatives to Animal Experimentation	2012
Cadmium	VCX3A	Marco Fabbri et al	Whole genome analysis and microRNAs	Alternatives to Animal Experimentation	2012
Cadmium	PRSS23	Marco Fabbri et al	Whole genome analysis and microRNAs	Alternatives to Animal Experimentation	2012
Cadmium	NCF2	Marco Fabbri et al	Whole genome analysis and microRNAs	Alternatives to Animal Experimentation	2012
Cadmium	FOS	Marco Fabbri et al	Whole genome analysis and microRNAs	Alternatives to Animal Experimentation	2012
Cadmium	PPP1R15A	Marco Fabbri et al	Whole genome analysis and microRNAs	Alternatives to Animal Experimentation	2012
Cadmium	MYOF	Marco Fabbri et al	Whole genome analysis and microRNAs	Alternatives to Animal Experimentation	2012
Cadmium	SYMBOL	Marco Fabbri et al	Whole genome analysis and microRNAs	Alternatives to Animal Experimentation	2012
Cadmium	MMP3	Marco Fabbri et al	Whole genome analysis and microRNAs	Alternatives to Animal Experimentation	2012
Cadmium	EGR4	Marco Fabbri et al	Whole genome analysis and microRNAs	Alternatives to Animal Experimentation	2012
Cadmium	IGFBP1	Marco Fabbri et al	Whole genome analysis and microRNAs	Alternatives to Animal Experimentation	2012
Cadmium	AMPD3	Marco Fabbri et al	Whole genome analysis and microRNAs	Alternatives to Animal Experimentation	2012
Cadmium	LOC100129113	Marco Fabbri et al	Whole genome analysis and microRNAs	Alternatives to Animal Experimentation	2012
Cadmium	AQP3	Marco Fabbri et al	Whole genome analysis and microRNAs	Alternatives to Animal Experimentation	2012
Cadmium	S100A2	Marco Fabbri et al	Whole genome analysis and microRNAs	Alternatives to Animal Experimentation	2012
Cadmium	SPOCD1	Marco Fabbri et al	Whole genome analysis and microRNAs	Alternatives to Animal Experimentation	2012
Cadmium	MYLIP	Marco Fabbri et al	Whole genome analysis and microRNAs	Alternatives to Animal Experimentation	2012
Cadmium	BATF	Marco Fabbri et al	Whole genome analysis and microRNAs	Alternatives to Animal Experimentation	2012
Cadmium	VCX	Marco Fabbri et al	Whole genome analysis and microRNAs	Alternatives to Animal Experimentation	2012
Cadmium	LOXL2	Marco Fabbri et al	Whole genome analysis and microRNAs	Alternatives to Animal Experimentation	2012
Cadmium	ESAM	Marco Fabbri et al	Whole genome analysis and microRNAs	Alternatives to Animal Experimentation	2012
Cadmium	GLIPR1	Marco Fabbri et al	Whole genome analysis and microRNAs	Alternatives to Animal Experimentation	2012
Cadmium	VCX2	Marco Fabbri et al	Whole genome analysis and microRNAs	Alternatives to Animal Experimentation	2012
Cadmium	TMSL3	Marco Fabbri et al	Whole genome analysis and microRNAs	Alternatives to Animal Experimentation	2012
Cadmium	AREG	Marco Fabbri et al	Whole genome analysis and microRNAs	Alternatives to Animal Experimentation	2012
Cadmium	F2RL1	Marco Fabbri et al	Whole genome analysis and microRNAs	Alternatives to Animal Experimentation	2012
Cadmium	ASPH	Marco Fabbri et al	Whole genome analysis and microRNAs	Alternatives to Animal Experimentation	2012
Cadmium	PLAU	Marco Fabbri et al	Whole genome analysis and microRNAs	Alternatives to Animal Experimentation	2012
Cadmium	RASD1	Marco Fabbri et al	Whole genome analysis and microRNAs	Alternatives to Animal Experimentation	2012
Cadmium	METRNL	Marco Fabbri et al	Whole genome analysis and microRNAs	Alternatives to Animal Experimentation	2012
Cadmium	FLJ38109	Marco Fabbri et al	Whole genome analysis and microRNAs	Alternatives to Animal Experimentation	2012
Cadmium	PLCH2	Marco Fabbri et al	Whole genome analysis and microRNAs	Alternatives to Animal Experimentation	2012
Cadmium	TFPI2	Marco Fabbri et al	Whole genome analysis and microRNAs	Alternatives to Animal Experimentation	2012
Cadmium	GRB10	Marco Fabbri et al	Whole genome analysis and microRNAs	Alternatives to Animal Experimentation	2012
Cadmium	MMP1	Marco Fabbri et al	Whole genome analysis and microRNAs	Alternatives to Animal Experimentation	2012
Cadmium	ADAM19	Marco Fabbri et al	Whole genome analysis and microRNAs	Alternatives to Animal Experimentation	2012
Cadmium	MLLT11	Marco Fabbri et al	Whole genome analysis and microRNAs	Alternatives to Animal Experimentation	2012
Cadmium	CYP1A1	Marco Fabbri et al	Whole genome analysis and microRNAs	Alternatives to Animal Experimentation	2012
Cadmium	DOK7	Marco Fabbri et al	Whole genome analysis and microRNAs	Alternatives to Animal Experimentation	2012
Cadmium	MT2A	Marco Fabbri et al	Whole genome analysis and microRNAs	Alternatives to Animal Experimentation	2012
Cadmium	GFPT2	Marco Fabbri et al	Whole genome analysis and microRNAs	Alternatives to Animal Experimentation	2012
Cadmium	CCK	Marco Fabbri et al	Whole genome analysis and microRNAs	Alternatives to Animal Experimentation	2012
Cadmium	CXCR4	Marco Fabbri et al	Whole genome analysis and microRNAs	Alternatives to Animal Experimentation	2012
Cadmium	BCL2A1	Marco Fabbri et al	Whole genome analysis and microRNAs	Alternatives to Animal Experimentation	2012
Cadmium	SERPINE1	Marco Fabbri et al	Whole genome analysis and microRNAs	Alternatives to Animal Experimentation	2012
Cadmium	TSPAN5	Marco Fabbri et al	Whole genome analysis and microRNAs	Alternatives to Animal Experimentation	2012
Cadmium	PHLDA2	Marco Fabbri et al	Whole genome analysis and microRNAs	Alternatives to Animal Experimentation	2012

Cadmium	AXL	Marco Fabbri et al	Whole genome analysis and microRNAs	Alternatives to Animal Experimentation	2012
Cadmium	ELK3	Marco Fabbri et al	Whole genome analysis and microRNAs	Alternatives to Animal Experimentation	2012
Cadmium	C15ORF52	Marco Fabbri et al	Whole genome analysis and microRNAs	Alternatives to Animal Experimentation	2012
Cadmium	FHL2	Marco Fabbri et al	Whole genome analysis and microRNAs	Alternatives to Animal Experimentation	2012
Cadmium	JUN	Marco Fabbri et al	Whole genome analysis and microRNAs	Alternatives to Animal Experimentation	2012
Cadmium	DCBLD2	Marco Fabbri et al	Whole genome analysis and microRNAs	Alternatives to Animal Experimentation	2012
Cadmium	S100A3	Marco Fabbri et al	Whole genome analysis and microRNAs	Alternatives to Animal Experimentation	2012
Cadmium	NR4A1	Marco Fabbri et al	Whole genome analysis and microRNAs	Alternatives to Animal Experimentation	2012
Cadmium	KRT80	Marco Fabbri et al	Whole genome analysis and microRNAs	Alternatives to Animal Experimentation	2012
Cadmium	TMSB4X	Marco Fabbri et al	Whole genome analysis and microRNAs	Alternatives to Animal Experimentation	2012
Cadmium	LIF	Marco Fabbri et al	Whole genome analysis and microRNAs	Alternatives to Animal Experimentation	2012
Cadmium	MICAL2	Marco Fabbri et al	Whole genome analysis and microRNAs	Alternatives to Animal Experimentation	2012
Cadmium	CCR7	Marco Fabbri et al	Whole genome analysis and microRNAs	Alternatives to Animal Experimentation	2012
Cadmium	SERPINE2	Marco Fabbri et al	Whole genome analysis and microRNAs	Alternatives to Animal Experimentation	2012
Cadmium	MYEOV	Marco Fabbri et al	Whole genome analysis and microRNAs	Alternatives to Animal Experimentation	2012
Cadmium	RAB3B	Marco Fabbri et al	Whole genome analysis and microRNAs	Alternatives to Animal Experimentation	2012
Cadmium	TUBB2B	Marco Fabbri et al	Whole genome analysis and microRNAs	Alternatives to Animal Experimentation	2012
Cadmium	SH2D5	Marco Fabbri et al	Whole genome analysis and microRNAs	Alternatives to Animal Experimentation	2012
Cadmium	LAIR2	Marco Fabbri et al	Whole genome analysis and microRNAs	Alternatives to Animal Experimentation	2012
Cadmium	EREG	Marco Fabbri et al	Whole genome analysis and microRNAs	Alternatives to Animal Experimentation	2012
Cadmium	LY6K	Marco Fabbri et al	Whole genome analysis and microRNAs	Alternatives to Animal Experimentation	2012
Cadmium	C19ORF33	Marco Fabbri et al	Whole genome analysis and microRNAs	Alternatives to Animal Experimentation	2012
Cadmium	ADAMTS4	Marco Fabbri et al	Whole genome analysis and microRNAs	Alternatives to Animal Experimentation	2012
Cadmium	MPP3	Marco Fabbri et al	Whole genome analysis and microRNAs	Alternatives to Animal Experimentation	2012
Cadmium	TUBA4A	Marco Fabbri et al	Whole genome analysis and microRNAs	Alternatives to Animal Experimentation	2012
Cadmium	GLCCI1	Marco Fabbri et al	Whole genome analysis and microRNAs	Alternatives to Animal Experimentation	2012
Cadmium	JUNB	Marco Fabbri et al	Whole genome analysis and microRNAs	Alternatives to Animal Experimentation	2012
Cadmium	LBH	Marco Fabbri et al	Whole genome analysis and microRNAs	Alternatives to Animal Experimentation	2012
Cadmium	RGS10	Marco Fabbri et al	Whole genome analysis and microRNAs	Alternatives to Animal Experimentation	2012
Cadmium	CAPN2	Marco Fabbri et al	Whole genome analysis and microRNAs	Alternatives to Animal Experimentation	2012
Cadmium	HRK	Marco Fabbri et al	Whole genome analysis and microRNAs	Alternatives to Animal Experimentation	2012
Cadmium	CYR61	Marco Fabbri et al	Whole genome analysis and microRNAs	Alternatives to Animal Experimentation	2012
Cadmium	CD109	Marco Fabbri et al	Whole genome analysis and microRNAs	Alternatives to Animal Experimentation	2012
Cadmium	FRMD5	Marco Fabbri et al	Whole genome analysis and microRNAs	Alternatives to Animal Experimentation	2012
Cadmium	SERPINB8	Marco Fabbri et al	Whole genome analysis and microRNAs	Alternatives to Animal Experimentation	2012
Cadmium	NRG1	Marco Fabbri et al	Whole genome analysis and microRNAs	Alternatives to Animal Experimentation	2012
Cadmium	IER5	Marco Fabbri et al	Whole genome analysis and microRNAs	Alternatives to Animal Experimentation	2012
Cadmium	PFKP	Marco Fabbri et al	Whole genome analysis and microRNAs	Alternatives to Animal Experimentation	2012
Cadmium	LARP6	Marco Fabbri et al	Whole genome analysis and microRNAs	Alternatives to Animal Experimentation	2012
Cadmium	WNT6	Marco Fabbri et al	Whole genome analysis and microRNAs	Alternatives to Animal Experimentation	2012
Cadmium	NUAK1	Marco Fabbri et al	Whole genome analysis and microRNAs	Alternatives to Animal Experimentation	2012
Cadmium	DST	Marco Fabbri et al	Whole genome analysis and microRNAs	Alternatives to Animal Experimentation	2012
Cadmium	AGPAT4	Marco Fabbri et al	Whole genome analysis and microRNAs	Alternatives to Animal Experimentation	2012
Cadmium	SOX4	Marco Fabbri et al	Whole genome analysis and microRNAs	Alternatives to Animal Experimentation	2012
Cadmium	STK17B	Marco Fabbri et al	Whole genome analysis and microRNAs	Alternatives to Animal Experimentation	2012
Cadmium	SYMBOL	Marco Fabbri et al	Whole genome analysis and microRNAs	Alternatives to Animal Experimentation	2012
Cadmium	CYP24A1	Marco Fabbri et al	Whole genome analysis and microRNAs	Alternatives to Animal Experimentation	2012
Cadmium	LOC79999	Marco Fabbri et al	Whole genome analysis and microRNAs	Alternatives to Animal Experimentation	2012
Cadmium	SPANXB2	Marco Fabbri et al	Whole genome analysis and microRNAs	Alternatives to Animal Experimentation	2012
Cadmium	QSOX1	Marco Fabbri et al	Whole genome analysis and microRNAs	Alternatives to Animal Experimentation	2012
Cadmium	EMP3	Marco Fabbri et al	Whole genome analysis and microRNAs	Alternatives to Animal Experimentation	2012
Cadmium	S100A6	Marco Fabbri et al	Whole genome analysis and microRNAs	Alternatives to Animal Experimentation	2012
Cadmium	MAP1B	Marco Fabbri et al	Whole genome analysis and microRNAs	Alternatives to Animal Experimentation	2012
Cadmium	TMEM49	Marco Fabbri et al	Whole genome analysis and microRNAs	Alternatives to Animal Experimentation	2012
Cadmium	NKAIN1	Marco Fabbri et al	Whole genome analysis and microRNAs	Alternatives to Animal Experimentation	2012
Cadmium	PLAUR	Marco Fabbri et al	Whole genome analysis and microRNAs	Alternatives to Animal Experimentation	2012
Cadmium	ACTA1	Marco Fabbri et al	Whole genome analysis and microRNAs	Alternatives to Animal Experimentation	2012
Cadmium	TGM2	Marco Fabbri et al	Whole genome analysis and microRNAs	Alternatives to Animal Experimentation	2012
Cadmium	UCA1	Marco Fabbri et al	Whole genome analysis and microRNAs	Alternatives to Animal Experimentation	2012
Cadmium	PROCR	Marco Fabbri et al	Whole genome analysis and microRNAs	Alternatives to Animal Experimentation	2012
Cadmium	ADAP2	Marco Fabbri et al	Whole genome analysis and microRNAs	Alternatives to Animal Experimentation	2012
Cadmium	TIMP2	Marco Fabbri et al	Whole genome analysis and microRNAs	Alternatives to Animal Experimentation	2012
Cadmium	COTL1	Marco Fabbri et al	Whole genome analysis and microRNAs	Alternatives to Animal Experimentation	2012
Cadmium	GPSM1	Marco Fabbri et al	Whole genome analysis and microRNAs	Alternatives to Animal Experimentation	2012
Cadmium	SCHIP1	Marco Fabbri et al	Whole genome analysis and microRNAs	Alternatives to Animal Experimentation	2012
Cadmium	ZG16B	Marco Fabbri et al	Whole genome analysis and microRNAs	Alternatives to Animal Experimentation	2012
Cadmium	SOCS2	Marco Fabbri et al	Whole genome analysis and microRNAs	Alternatives to Animal Experimentation	2012
Cadmium	PDLIM4	Marco Fabbri et al	Whole genome analysis and microRNAs	Alternatives to Animal Experimentation	2012
Cadmium	FJX1	Marco Fabbri et al	Whole genome analysis and microRNAs	Alternatives to Animal Experimentation	2012
Cadmium	GGT5	Marco Fabbri et al	Whole genome analysis and microRNAs	Alternatives to Animal Experimentation	2012
Cadmium	ANXA3	Marco Fabbri et al	Whole genome analysis and microRNAs	Alternatives to Animal Experimentation	2012
Cadmium	IL8	Marco Fabbri et al	Whole genome analysis and microRNAs	Alternatives to Animal Experimentation	2012
Cadmium	DUSP6	Marco Fabbri et al	Whole genome analysis and microRNAs	Alternatives to Animal Experimentation	2012
Cadmium	ITGA2	Marco Fabbri et al	Whole genome analysis and microRNAs	Alternatives to Animal Experimentation	2012
Cadmium	TUBB6	Marco Fabbri et al	Whole genome analysis and microRNAs	Alternatives to Animal Experimentation	2012
Cadmium	RND3	Marco Fabbri et al	Whole genome analysis and microRNAs	Alternatives to Animal Experimentation	2012
Cadmium	TEP1	Marco Fabbri et al	Whole genome analysis and microRNAs	Alternatives to Animal Experimentation	2012
Cadmium	BEND7	Marco Fabbri et al	Whole genome analysis and microRNAs	Alternatives to Animal Experimentation	2012
Cadmium	COL1A1	Marco Fabbri et al	Whole genome analysis and microRNAs	Alternatives to Animal Experimentation	2012
Cadmium	AGPAT9	Marco Fabbri et al	Whole genome analysis and microRNAs	Alternatives to Animal Experimentation	2012
Cadmium	BIRC3	Marco Fabbri et al	Whole genome analysis and microRNAs	Alternatives to Animal Experimentation	2012
Cadmium	SEL1L3	Marco Fabbri et al	Whole genome analysis and microRNAs	Alternatives to Animal Experimentation	2012
Cadmium	B3GNT7	Marco Fabbri et al	Whole genome analysis and microRNAs	Alternatives to Animal Experimentation	2012
Cadmium	VCAN	Marco Fabbri et al	Whole genome analysis and microRNAs	Alternatives to Animal Experimentation	2012
Cadmium	C19ORF28	Marco Fabbri et al	Whole genome analysis and microRNAs	Alternatives to Animal Experimentation	2012
Cadmium	MOSPD1	Marco Fabbri et al	Whole genome analysis and microRNAs	Alternatives to Animal Experimentation	2012
Cadmium	GPRC5A	Marco Fabbri et al	Whole genome analysis and microRNAs	Alternatives to Animal Experimentation	2012
Cadmium	PMAIP1	Marco Fabbri et al	Whole genome analysis and microRNAs	Alternatives to Animal Experimentation	2012
Cadmium	CNN1	Marco Fabbri et al	Whole genome analysis and microRNAs	Alternatives to Animal Experimentation	2012

Cadmium	GCNT3	Marco Fabbri et al	Whole genome analysis and microRNAs	Alternatives to Animal Experimentation	2012
Cadmium	SYMBOL	Marco Fabbri et al	Whole genome analysis and microRNAs	Alternatives to Animal Experimentation	2012
Cadmium	TNFRSF25	Marco Fabbri et al	Whole genome analysis and microRNAs	Alternatives to Animal Experimentation	2012
Cadmium	C19ORF21	Marco Fabbri et al	Whole genome analysis and microRNAs	Alternatives to Animal Experimentation	2012
Cadmium	WDR66	Marco Fabbri et al	Whole genome analysis and microRNAs	Alternatives to Animal Experimentation	2012
Cadmium	PLEKHG5	Marco Fabbri et al	Whole genome analysis and microRNAs	Alternatives to Animal Experimentation	2012
Cadmium	ZNF280A	Marco Fabbri et al	Whole genome analysis and microRNAs	Alternatives to Animal Experimentation	2012
Cadmium	RGS20	Marco Fabbri et al	Whole genome analysis and microRNAs	Alternatives to Animal Experimentation	2012
Cadmium	NPAS1	Marco Fabbri et al	Whole genome analysis and microRNAs	Alternatives to Animal Experimentation	2012
Cadmium	KRT15	Marco Fabbri et al	Whole genome analysis and microRNAs	Alternatives to Animal Experimentation	2012
Cadmium	SH3RF1	Marco Fabbri et al	Whole genome analysis and microRNAs	Alternatives to Animal Experimentation	2012
Cadmium	ANKRD1	Marco Fabbri et al	Whole genome analysis and microRNAs	Alternatives to Animal Experimentation	2012
Cadmium	IER3	Marco Fabbri et al	Whole genome analysis and microRNAs	Alternatives to Animal Experimentation	2012
Cadmium	KLF6	Marco Fabbri et al	Whole genome analysis and microRNAs	Alternatives to Animal Experimentation	2012
Cadmium	FBLIM1	Marco Fabbri et al	Whole genome analysis and microRNAs	Alternatives to Animal Experimentation	2012
Cadmium	SOHLH1	Marco Fabbri et al	Whole genome analysis and microRNAs	Alternatives to Animal Experimentation	2012
Cadmium	NDRG1	Marco Fabbri et al	Whole genome analysis and microRNAs	Alternatives to Animal Experimentation	2012
Cadmium	ZFP36	Marco Fabbri et al	Whole genome analysis and microRNAs	Alternatives to Animal Experimentation	2012
Cadmium	SYNE1	Marco Fabbri et al	Whole genome analysis and microRNAs	Alternatives to Animal Experimentation	2012
Cadmium	SAT1	Marco Fabbri et al	Whole genome analysis and microRNAs	Alternatives to Animal Experimentation	2012
Cadmium	MAFF	Marco Fabbri et al	Whole genome analysis and microRNAs	Alternatives to Animal Experimentation	2012
Cadmium	RAB7B	Marco Fabbri et al	Whole genome analysis and microRNAs	Alternatives to Animal Experimentation	2012
Cadmium	S100A11	Marco Fabbri et al	Whole genome analysis and microRNAs	Alternatives to Animal Experimentation	2012
Cadmium	DUSP13	Marco Fabbri et al	Whole genome analysis and microRNAs	Alternatives to Animal Experimentation	2012
Cadmium	MICAL1	Marco Fabbri et al	Whole genome analysis and microRNAs	Alternatives to Animal Experimentation	2012
Cadmium	OBFC2A	Marco Fabbri et al	Whole genome analysis and microRNAs	Alternatives to Animal Experimentation	2012
Cadmium	ASAP2	Marco Fabbri et al	Whole genome analysis and microRNAs	Alternatives to Animal Experimentation	2012
Cadmium	B3GNT3	Marco Fabbri et al	Whole genome analysis and microRNAs	Alternatives to Animal Experimentation	2012
Cadmium	SVIL	Marco Fabbri et al	Whole genome analysis and microRNAs	Alternatives to Animal Experimentation	2012
Cadmium	NES	Marco Fabbri et al	Whole genome analysis and microRNAs	Alternatives to Animal Experimentation	2012
Cadmium	BIK	Marco Fabbri et al	Whole genome analysis and microRNAs	Alternatives to Animal Experimentation	2012
Cadmium	STK10	Marco Fabbri et al	Whole genome analysis and microRNAs	Alternatives to Animal Experimentation	2012
Cadmium	ARHGEF4	Marco Fabbri et al	Whole genome analysis and microRNAs	Alternatives to Animal Experimentation	2012
Cadmium	ADM	Marco Fabbri et al	Whole genome analysis and microRNAs	Alternatives to Animal Experimentation	2012
Cadmium	POMC	Marco Fabbri et al	Whole genome analysis and microRNAs	Alternatives to Animal Experimentation	2012
Cadmium	CSGALNACT2	Marco Fabbri et al	Whole genome analysis and microRNAs	Alternatives to Animal Experimentation	2012
Cadmium	OTUB2	Marco Fabbri et al	Whole genome analysis and microRNAs	Alternatives to Animal Experimentation	2012
Cadmium	TNFRSF12A	Marco Fabbri et al	Whole genome analysis and microRNAs	Alternatives to Animal Experimentation	2012
Cadmium	SPEG	Marco Fabbri et al	Whole genome analysis and microRNAs	Alternatives to Animal Experimentation	2012
Cadmium	LOC284454	Marco Fabbri et al	Whole genome analysis and microRNAs	Alternatives to Animal Experimentation	2012
Cadmium	LGALS1	Marco Fabbri et al	Whole genome analysis and microRNAs	Alternatives to Animal Experimentation	2012
Cadmium	NDRG4	Marco Fabbri et al	Whole genome analysis and microRNAs	Alternatives to Animal Experimentation	2012
Cadmium	SPINT2	Marco Fabbri et al	Whole genome analysis and microRNAs	Alternatives to Animal Experimentation	2012
Cadmium	INHBB	Marco Fabbri et al	Whole genome analysis and microRNAs	Alternatives to Animal Experimentation	2012
Cadmium	TEX19	Marco Fabbri et al	Whole genome analysis and microRNAs	Alternatives to Animal Experimentation	2012
Cadmium	TIMP1	Marco Fabbri et al	Whole genome analysis and microRNAs	Alternatives to Animal Experimentation	2012
Cadmium	SPANXA1	Marco Fabbri et al	Whole genome analysis and microRNAs	Alternatives to Animal Experimentation	2012
Cadmium	IL23A	Marco Fabbri et al	Whole genome analysis and microRNAs	Alternatives to Animal Experimentation	2012
Cadmium	TMCC1	Marco Fabbri et al	Whole genome analysis and microRNAs	Alternatives to Animal Experimentation	2012
Cadmium	DEFB103B	Marco Fabbri et al	Whole genome analysis and microRNAs	Alternatives to Animal Experimentation	2012
Cadmium	FAM110C	Marco Fabbri et al	Whole genome analysis and microRNAs	Alternatives to Animal Experimentation	2012
Cadmium	UBE2H	Marco Fabbri et al	Whole genome analysis and microRNAs	Alternatives to Animal Experimentation	2012
Cadmium	AP1S3	Marco Fabbri et al	Whole genome analysis and microRNAs	Alternatives to Animal Experimentation	2012
Cadmium	HIST2H2AA4	Marco Fabbri et al	Whole genome analysis and microRNAs	Alternatives to Animal Experimentation	2012
Cadmium	TRPV2	Marco Fabbri et al	Whole genome analysis and microRNAs	Alternatives to Animal Experimentation	2012
Cadmium	KIF3C	Marco Fabbri et al	Whole genome analysis and microRNAs	Alternatives to Animal Experimentation	2012
Cadmium	SPSB1	Marco Fabbri et al	Whole genome analysis and microRNAs	Alternatives to Animal Experimentation	2012
Cadmium	ITPRIP	Marco Fabbri et al	Whole genome analysis and microRNAs	Alternatives to Animal Experimentation	2012
Cadmium	C13ORF15	Marco Fabbri et al	Whole genome analysis and microRNAs	Alternatives to Animal Experimentation	2012
Cadmium	C16ORF45	Marco Fabbri et al	Whole genome analysis and microRNAs	Alternatives to Animal Experimentation	2012
Cadmium	RAC2	Marco Fabbri et al	Whole genome analysis and microRNAs	Alternatives to Animal Experimentation	2012
Cadmium	SLC6A6	Marco Fabbri et al	Whole genome analysis and microRNAs	Alternatives to Animal Experimentation	2012
Cadmium	AHNAK	Marco Fabbri et al	Whole genome analysis and microRNAs	Alternatives to Animal Experimentation	2012
Cadmium	KLF5	Marco Fabbri et al	Whole genome analysis and microRNAs	Alternatives to Animal Experimentation	2012
Cadmium	ZBTB20	Marco Fabbri et al	Whole genome analysis and microRNAs	Alternatives to Animal Experimentation	2012
Cadmium	IER5L	Marco Fabbri et al	Whole genome analysis and microRNAs	Alternatives to Animal Experimentation	2012
Cadmium	ADAMTS14	Marco Fabbri et al	Whole genome analysis and microRNAs	Alternatives to Animal Experimentation	2012
Cadmium	IDS	Marco Fabbri et al	Whole genome analysis and microRNAs	Alternatives to Animal Experimentation	2012
Cadmium	CATSPER1	Marco Fabbri et al	Whole genome analysis and microRNAs	Alternatives to Animal Experimentation	2012
Cadmium	S1PR4	Marco Fabbri et al	Whole genome analysis and microRNAs	Alternatives to Animal Experimentation	2012
Cadmium	GRB14	Marco Fabbri et al	Whole genome analysis and microRNAs	Alternatives to Animal Experimentation	2012
Cadmium	ICAM2	Marco Fabbri et al	Whole genome analysis and microRNAs	Alternatives to Animal Experimentation	2012
Cadmium	CILP	Marco Fabbri et al	Whole genome analysis and microRNAs	Alternatives to Animal Experimentation	2012
Cadmium	PLK3	Marco Fabbri et al	Whole genome analysis and microRNAs	Alternatives to Animal Experimentation	2012
Cadmium	OAS1	Marco Fabbri et al	Whole genome analysis and microRNAs	Alternatives to Animal Experimentation	2012
Cadmium	CLCF1	Marco Fabbri et al	Whole genome analysis and microRNAs	Alternatives to Animal Experimentation	2012
Cadmium	SLC16A6	Marco Fabbri et al	Whole genome analysis and microRNAs	Alternatives to Animal Experimentation	2012
Cadmium	PLEKHA6	Marco Fabbri et al	Whole genome analysis and microRNAs	Alternatives to Animal Experimentation	2012
Cadmium	CELA3B	Marco Fabbri et al	Whole genome analysis and microRNAs	Alternatives to Animal Experimentation	2012
Cadmium	RIN1	Marco Fabbri et al	Whole genome analysis and microRNAs	Alternatives to Animal Experimentation	2012
Cadmium	HES1	Marco Fabbri et al	Whole genome analysis and microRNAs	Alternatives to Animal Experimentation	2012
Cadmium	UPP1	Marco Fabbri et al	Whole genome analysis and microRNAs	Alternatives to Animal Experimentation	2012
Cadmium	C17ORF91	Marco Fabbri et al	Whole genome analysis and microRNAs	Alternatives to Animal Experimentation	2012
Cadmium	NFE2L3	Marco Fabbri et al	Whole genome analysis and microRNAs	Alternatives to Animal Experimentation	2012
Cadmium	PTRF	Marco Fabbri et al	Whole genome analysis and microRNAs	Alternatives to Animal Experimentation	2012
Cadmium	HSPB8	Marco Fabbri et al	Whole genome analysis and microRNAs	Alternatives to Animal Experimentation	2012
Cadmium	CD59	Marco Fabbri et al	Whole genome analysis and microRNAs	Alternatives to Animal Experimentation	2012
Cadmium	PSG6	Marco Fabbri et al	Whole genome analysis and microRNAs	Alternatives to Animal Experimentation	2012
Cadmium	LRRC8A	Marco Fabbri et al	Whole genome analysis and microRNAs	Alternatives to Animal Experimentation	2012

Cadmium	MLF1	Marco Fabbri et al	Whole genome analysis and microRNAs	Alternatives to Animal Experimentation	2012
Cadmium	LOC440900	Marco Fabbri et al	Whole genome analysis and microRNAs	Alternatives to Animal Experimentation	2012
Cadmium	RBP1	Marco Fabbri et al	Whole genome analysis and microRNAs	Alternatives to Animal Experimentation	2012
Cadmium	PHLDA1	Marco Fabbri et al	Whole genome analysis and microRNAs	Alternatives to Animal Experimentation	2012
Cadmium	CDA	Marco Fabbri et al	Whole genome analysis and microRNAs	Alternatives to Animal Experimentation	2012
Cadmium	MET	Marco Fabbri et al	Whole genome analysis and microRNAs	Alternatives to Animal Experimentation	2012
Cadmium	RHBDF2	Marco Fabbri et al	Whole genome analysis and microRNAs	Alternatives to Animal Experimentation	2012
Cadmium	RNF183	Marco Fabbri et al	Whole genome analysis and microRNAs	Alternatives to Animal Experimentation	2012
Cadmium	SERTAD1	Marco Fabbri et al	Whole genome analysis and microRNAs	Alternatives to Animal Experimentation	2012
Cadmium	FBXL16	Marco Fabbri et al	Whole genome analysis and microRNAs	Alternatives to Animal Experimentation	2012
Cadmium	SOX8	Marco Fabbri et al	Whole genome analysis and microRNAs	Alternatives to Animal Experimentation	2012
Cadmium	FAT1	Marco Fabbri et al	Whole genome analysis and microRNAs	Alternatives to Animal Experimentation	2012
Cadmium	C3ORF52	Marco Fabbri et al	Whole genome analysis and microRNAs	Alternatives to Animal Experimentation	2012
Cadmium	TGFB1I1	Marco Fabbri et al	Whole genome analysis and microRNAs	Alternatives to Animal Experimentation	2012
Cadmium	FOSL1	Marco Fabbri et al	Whole genome analysis and microRNAs	Alternatives to Animal Experimentation	2012
Cadmium	EIF5A2	Marco Fabbri et al	Whole genome analysis and microRNAs	Alternatives to Animal Experimentation	2012
Cadmium	ITPR3	Marco Fabbri et al	Whole genome analysis and microRNAs	Alternatives to Animal Experimentation	2012
Cadmium	TUBB2A	Marco Fabbri et al	Whole genome analysis and microRNAs	Alternatives to Animal Experimentation	2012
Cadmium	SPRY4	Marco Fabbri et al	Whole genome analysis and microRNAs	Alternatives to Animal Experimentation	2012
Cadmium	ATF3	Marco Fabbri et al	Whole genome analysis and microRNAs	Alternatives to Animal Experimentation	2012
Cadmium	GDF15	Marco Fabbri et al	Whole genome analysis and microRNAs	Alternatives to Animal Experimentation	2012
Cadmium	LHX6	Marco Fabbri et al	Whole genome analysis and microRNAs	Alternatives to Animal Experimentation	2012
Cadmium	LETM2	Marco Fabbri et al	Whole genome analysis and microRNAs	Alternatives to Animal Experimentation	2012
Cadmium	OSMR	Marco Fabbri et al	Whole genome analysis and microRNAs	Alternatives to Animal Experimentation	2012
Cadmium	STEAP1	Marco Fabbri et al	Whole genome analysis and microRNAs	Alternatives to Animal Experimentation	2012
Cadmium	HIVEP2	Marco Fabbri et al	Whole genome analysis and microRNAs	Alternatives to Animal Experimentation	2012
Cadmium	RHOC	Marco Fabbri et al	Whole genome analysis and microRNAs	Alternatives to Animal Experimentation	2012
Cadmium	PLK2	Marco Fabbri et al	Whole genome analysis and microRNAs	Alternatives to Animal Experimentation	2012
Cadmium	C6ORF115	Marco Fabbri et al	Whole genome analysis and microRNAs	Alternatives to Animal Experimentation	2012
Cadmium	EPHA2	Marco Fabbri et al	Whole genome analysis and microRNAs	Alternatives to Animal Experimentation	2012
Cadmium	TMEM158	Marco Fabbri et al	Whole genome analysis and microRNAs	Alternatives to Animal Experimentation	2012
Cadmium	LFNG	Marco Fabbri et al	Whole genome analysis and microRNAs	Alternatives to Animal Experimentation	2012
Cadmium	SLC25A12	Marco Fabbri et al	Whole genome analysis and microRNAs	Alternatives to Animal Experimentation	2012
Cadmium	SLC2A1	Marco Fabbri et al	Whole genome analysis and microRNAs	Alternatives to Animal Experimentation	2012
Cadmium	ABR	Marco Fabbri et al	Whole genome analysis and microRNAs	Alternatives to Animal Experimentation	2012
Cadmium	EMP1	Marco Fabbri et al	Whole genome analysis and microRNAs	Alternatives to Animal Experimentation	2012
Cadmium	DUSP1	Marco Fabbri et al	Whole genome analysis and microRNAs	Alternatives to Animal Experimentation	2012
Cadmium	CARD10	Marco Fabbri et al	Whole genome analysis and microRNAs	Alternatives to Animal Experimentation	2012
Cadmium	LCK	Marco Fabbri et al	Whole genome analysis and microRNAs	Alternatives to Animal Experimentation	2012
Cadmium	RELB	Marco Fabbri et al	Whole genome analysis and microRNAs	Alternatives to Animal Experimentation	2012
Cadmium	PXN	Marco Fabbri et al	Whole genome analysis and microRNAs	Alternatives to Animal Experimentation	2012
Cadmium	CREB5	Marco Fabbri et al	Whole genome analysis and microRNAs	Alternatives to Animal Experimentation	2012
Cadmium	HIST1H2AD	Marco Fabbri et al	Whole genome analysis and microRNAs	Alternatives to Animal Experimentation	2012
Cadmium	FOXC1	Marco Fabbri et al	Whole genome analysis and microRNAs	Alternatives to Animal Experimentation	2012
Cadmium	SPANXD	Marco Fabbri et al	Whole genome analysis and microRNAs	Alternatives to Animal Experimentation	2012
Cadmium	GPC1	Marco Fabbri et al	Whole genome analysis and microRNAs	Alternatives to Animal Experimentation	2012
Cadmium	ROBO4	Marco Fabbri et al	Whole genome analysis and microRNAs	Alternatives to Animal Experimentation	2012
Cadmium	RAB3A	Marco Fabbri et al	Whole genome analysis and microRNAs	Alternatives to Animal Experimentation	2012
Cadmium	KIAA0802	Marco Fabbri et al	Whole genome analysis and microRNAs	Alternatives to Animal Experimentation	2012
Cadmium	COL4A2	Marco Fabbri et al	Whole genome analysis and microRNAs	Alternatives to Animal Experimentation	2012
Cadmium	CLTB	Marco Fabbri et al	Whole genome analysis and microRNAs	Alternatives to Animal Experimentation	2012
Cadmium	ARRDC4	Marco Fabbri et al	Whole genome analysis and microRNAs	Alternatives to Animal Experimentation	2012
Cadmium	TMEM54	Marco Fabbri et al	Whole genome analysis and microRNAs	Alternatives to Animal Experimentation	2012
Cadmium	COL7A1	Marco Fabbri et al	Whole genome analysis and microRNAs	Alternatives to Animal Experimentation	2012
Cadmium	ITPRIPL2	Marco Fabbri et al	Whole genome analysis and microRNAs	Alternatives to Animal Experimentation	2012
Cadmium	ACTG2	Marco Fabbri et al	Whole genome analysis and microRNAs	Alternatives to Animal Experimentation	2012
Cadmium	LTBP1	Marco Fabbri et al	Whole genome analysis and microRNAs	Alternatives to Animal Experimentation	2012
Cadmium	PAQR3	Marco Fabbri et al	Whole genome analysis and microRNAs	Alternatives to Animal Experimentation	2012
Cadmium	HK1	Marco Fabbri et al	Whole genome analysis and microRNAs	Alternatives to Animal Experimentation	2012
Cadmium	ACRC	Marco Fabbri et al	Whole genome analysis and microRNAs	Alternatives to Animal Experimentation	2012
Cadmium	USP35	Marco Fabbri et al	Whole genome analysis and microRNAs	Alternatives to Animal Experimentation	2012
Cadmium	IQCD	Marco Fabbri et al	Whole genome analysis and microRNAs	Alternatives to Animal Experimentation	2012
Cadmium	SDC4	Marco Fabbri et al	Whole genome analysis and microRNAs	Alternatives to Animal Experimentation	2012
Cadmium	DSCR6	Marco Fabbri et al	Whole genome analysis and microRNAs	Alternatives to Animal Experimentation	2012
Cadmium	PALLD	Marco Fabbri et al	Whole genome analysis and microRNAs	Alternatives to Animal Experimentation	2012
Cadmium	LCE2C	Marco Fabbri et al	Whole genome analysis and microRNAs	Alternatives to Animal Experimentation	2012
Cadmium	PLEKHO1	Marco Fabbri et al	Whole genome analysis and microRNAs	Alternatives to Animal Experimentation	2012
Cadmium	GABARAPL1	Marco Fabbri et al	Whole genome analysis and microRNAs	Alternatives to Animal Experimentation	2012
Cadmium	GAL	Marco Fabbri et al	Whole genome analysis and microRNAs	Alternatives to Animal Experimentation	2012
Cadmium	FAM110B	Marco Fabbri et al	Whole genome analysis and microRNAs	Alternatives to Animal Experimentation	2012
Cadmium	NAV3	Marco Fabbri et al	Whole genome analysis and microRNAs	Alternatives to Animal Experimentation	2012
Cadmium	NOSTRIN	Marco Fabbri et al	Whole genome analysis and microRNAs	Alternatives to Animal Experimentation	2012
Cadmium	OSCAR	Marco Fabbri et al	Whole genome analysis and microRNAs	Alternatives to Animal Experimentation	2012
Cadmium	CT45A5	Marco Fabbri et al	Whole genome analysis and microRNAs	Alternatives to Animal Experimentation	2012
Cadmium	WSB2	Marco Fabbri et al	Whole genome analysis and microRNAs	Alternatives to Animal Experimentation	2012
Cadmium	KRT33A	Marco Fabbri et al	Whole genome analysis and microRNAs	Alternatives to Animal Experimentation	2012
Cadmium	C6ORF145	Marco Fabbri et al	Whole genome analysis and microRNAs	Alternatives to Animal Experimentation	2012
Cadmium	NPPC	Marco Fabbri et al	Whole genome analysis and microRNAs	Alternatives to Animal Experimentation	2012
Cadmium	TAGLN	Marco Fabbri et al	Whole genome analysis and microRNAs	Alternatives to Animal Experimentation	2012
Cadmium	TRIB1	Marco Fabbri et al	Whole genome analysis and microRNAs	Alternatives to Animal Experimentation	2012
Cadmium	SERPINB9	Marco Fabbri et al	Whole genome analysis and microRNAs	Alternatives to Animal Experimentation	2012
Cadmium	FRG2	Marco Fabbri et al	Whole genome analysis and microRNAs	Alternatives to Animal Experimentation	2012
Cadmium	S100A16	Marco Fabbri et al	Whole genome analysis and microRNAs	Alternatives to Animal Experimentation	2012
Cadmium	LOC645954	Marco Fabbri et al	Whole genome analysis and microRNAs	Alternatives to Animal Experimentation	2012
Cadmium	TMEM217	Marco Fabbri et al	Whole genome analysis and microRNAs	Alternatives to Animal Experimentation	2012
Cadmium	NAGS	Marco Fabbri et al	Whole genome analysis and microRNAs	Alternatives to Animal Experimentation	2012
Cadmium	LOC285986	Marco Fabbri et al	Whole genome analysis and microRNAs	Alternatives to Animal Experimentation	2012
Cadmium	NRP1	Marco Fabbri et al	Whole genome analysis and microRNAs	Alternatives to Animal Experimentation	2012
Cadmium	AK5	Marco Fabbri et al	Whole genome analysis and microRNAs	Alternatives to Animal Experimentation	2012

Cadmium	NEXN	Marco Fabbri et al	Whole genome analysis and microRNAs	Alternatives to Animal Experimentation	2012
Cadmium	ATP8A2	Marco Fabbri et al	Whole genome analysis and microRNAs	Alternatives to Animal Experimentation	2012
Cadmium	GLRX	Marco Fabbri et al	Whole genome analysis and microRNAs	Alternatives to Animal Experimentation	2012
Cadmium	EPPK1	Marco Fabbri et al	Whole genome analysis and microRNAs	Alternatives to Animal Experimentation	2012
Cadmium	KYNU	Marco Fabbri et al	Whole genome analysis and microRNAs	Alternatives to Animal Experimentation	2012
Cadmium	MCL1	Marco Fabbri et al	Whole genome analysis and microRNAs	Alternatives to Animal Experimentation	2012
Cadmium	VGF	Marco Fabbri et al	Whole genome analysis and microRNAs	Alternatives to Animal Experimentation	2012
Cadmium	BAAT	Marco Fabbri et al	Whole genome analysis and microRNAs	Alternatives to Animal Experimentation	2012
Cadmium	ZFP36L1	Marco Fabbri et al	Whole genome analysis and microRNAs	Alternatives to Animal Experimentation	2012
Cadmium	DUSP4	Marco Fabbri et al	Whole genome analysis and microRNAs	Alternatives to Animal Experimentation	2012
Cadmium	IER2	Marco Fabbri et al	Whole genome analysis and microRNAs	Alternatives to Animal Experimentation	2012
Cadmium	RIT1	Marco Fabbri et al	Whole genome analysis and microRNAs	Alternatives to Animal Experimentation	2012
Cadmium	ITGA3	Marco Fabbri et al	Whole genome analysis and microRNAs	Alternatives to Animal Experimentation	2012
Cadmium	STX1A	Marco Fabbri et al	Whole genome analysis and microRNAs	Alternatives to Animal Experimentation	2012
Cadmium	ITGB1	Marco Fabbri et al	Whole genome analysis and microRNAs	Alternatives to Animal Experimentation	2012
Cadmium	RELT	Marco Fabbri et al	Whole genome analysis and microRNAs	Alternatives to Animal Experimentation	2012
Cadmium	GALNT10	Marco Fabbri et al	Whole genome analysis and microRNAs	Alternatives to Animal Experimentation	2012
Cadmium	FAM46C	Marco Fabbri et al	Whole genome analysis and microRNAs	Alternatives to Animal Experimentation	2012
Cadmium	CHIC2	Marco Fabbri et al	Whole genome analysis and microRNAs	Alternatives to Animal Experimentation	2012
Cadmium	FOLR1	Marco Fabbri et al	Whole genome analysis and microRNAs	Alternatives to Animal Experimentation	2012
Cadmium	DISP2	Marco Fabbri et al	Whole genome analysis and microRNAs	Alternatives to Animal Experimentation	2012
Cadmium	KREMEN2	Marco Fabbri et al	Whole genome analysis and microRNAs	Alternatives to Animal Experimentation	2012
Cadmium	KRTAP1-5	Marco Fabbri et al	Whole genome analysis and microRNAs	Alternatives to Animal Experimentation	2012
Cadmium	SH3RF2	Marco Fabbri et al	Whole genome analysis and microRNAs	Alternatives to Animal Experimentation	2012
Cadmium	TAGLN3	Marco Fabbri et al	Whole genome analysis and microRNAs	Alternatives to Animal Experimentation	2012
Cadmium	GRAP	Marco Fabbri et al	Whole genome analysis and microRNAs	Alternatives to Animal Experimentation	2012
Cadmium	FOSB	Marco Fabbri et al	Whole genome analysis and microRNAs	Alternatives to Animal Experimentation	2012
Cadmium	CPEB4	Marco Fabbri et al	Whole genome analysis and microRNAs	Alternatives to Animal Experimentation	2012
Cadmium	MLPH	Marco Fabbri et al	Whole genome analysis and microRNAs	Alternatives to Animal Experimentation	2012
Cadmium	NTSR1	Marco Fabbri et al	Whole genome analysis and microRNAs	Alternatives to Animal Experimentation	2012
Cadmium	KRT19P2	Marco Fabbri et al	Whole genome analysis and microRNAs	Alternatives to Animal Experimentation	2012
Cadmium	FILIP1L	Marco Fabbri et al	Whole genome analysis and microRNAs	Alternatives to Animal Experimentation	2012
Cadmium	CT45A1	Marco Fabbri et al	Whole genome analysis and microRNAs	Alternatives to Animal Experimentation	2012
Cadmium	WNK4	Marco Fabbri et al	Whole genome analysis and microRNAs	Alternatives to Animal Experimentation	2012
Cadmium	KIAA1949	Marco Fabbri et al	Whole genome analysis and microRNAs	Alternatives to Animal Experimentation	2012
Cadmium	KRT23	Marco Fabbri et al	Whole genome analysis and microRNAs	Alternatives to Animal Experimentation	2012
Cadmium	FAM46B	Marco Fabbri et al	Whole genome analysis and microRNAs	Alternatives to Animal Experimentation	2012
Cadmium	CYTH3	Marco Fabbri et al	Whole genome analysis and microRNAs	Alternatives to Animal Experimentation	2012
Cadmium	PPM1N	Marco Fabbri et al	Whole genome analysis and microRNAs	Alternatives to Animal Experimentation	2012
Cadmium	CTSC	Marco Fabbri et al	Whole genome analysis and microRNAs	Alternatives to Animal Experimentation	2012
Cadmium	GCLM	Marco Fabbri et al	Whole genome analysis and microRNAs	Alternatives to Animal Experimentation	2012
Cadmium	ACSL5	Marco Fabbri et al	Whole genome analysis and microRNAs	Alternatives to Animal Experimentation	2012
Cadmium	ARHGEF18	Marco Fabbri et al	Whole genome analysis and microRNAs	Alternatives to Animal Experimentation	2012
Cadmium	PLXND1	Marco Fabbri et al	Whole genome analysis and microRNAs	Alternatives to Animal Experimentation	2012
Cadmium	ACTC1	Marco Fabbri et al	Whole genome analysis and microRNAs	Alternatives to Animal Experimentation	2012
Cadmium	PPFIBP1	Marco Fabbri et al	Whole genome analysis and microRNAs	Alternatives to Animal Experimentation	2012
Cadmium	NFATC1	Marco Fabbri et al	Whole genome analysis and microRNAs	Alternatives to Animal Experimentation	2012
Cadmium	SH3BGRL3	Marco Fabbri et al	Whole genome analysis and microRNAs	Alternatives to Animal Experimentation	2012
Cadmium	EDEM1	Marco Fabbri et al	Whole genome analysis and microRNAs	Alternatives to Animal Experimentation	2012
Cadmium	AMICA1	Marco Fabbri et al	Whole genome analysis and microRNAs	Alternatives to Animal Experimentation	2012
Cadmium	KIAA0284	Marco Fabbri et al	Whole genome analysis and microRNAs	Alternatives to Animal Experimentation	2012
Cadmium	DYNC2H1	Marco Fabbri et al	Whole genome analysis and microRNAs	Alternatives to Animal Experimentation	2012
Cadmium	PPP1R13L	Marco Fabbri et al	Whole genome analysis and microRNAs	Alternatives to Animal Experimentation	2012
Cadmium	DSP	Marco Fabbri et al	Whole genome analysis and microRNAs	Alternatives to Animal Experimentation	2012
Cadmium	RRP12	Marco Fabbri et al	Whole genome analysis and microRNAs	Alternatives to Animal Experimentation	2012
Cadmium	SLC38A2	Marco Fabbri et al	Whole genome analysis and microRNAs	Alternatives to Animal Experimentation	2012
Cadmium	CCNO	Marco Fabbri et al	Whole genome analysis and microRNAs	Alternatives to Animal Experimentation	2012
Cadmium	GULP1	Marco Fabbri et al	Whole genome analysis and microRNAs	Alternatives to Animal Experimentation	2012
Cadmium	GSTP1	Marco Fabbri et al	Whole genome analysis and microRNAs	Alternatives to Animal Experimentation	2012
Cadmium	CHI3L2	Marco Fabbri et al	Whole genome analysis and microRNAs	Alternatives to Animal Experimentation	2012
Cadmium	SUSD2	Marco Fabbri et al	Whole genome analysis and microRNAs	Alternatives to Animal Experimentation	2012
Cadmium	HAS3	Marco Fabbri et al	Whole genome analysis and microRNAs	Alternatives to Animal Experimentation	2012
Cadmium	SLC16A5	Marco Fabbri et al	Whole genome analysis and microRNAs	Alternatives to Animal Experimentation	2012
Cadmium	BHLHE40	Marco Fabbri et al	Whole genome analysis and microRNAs	Alternatives to Animal Experimentation	2012
Cadmium	SLC39A10	Marco Fabbri et al	Whole genome analysis and microRNAs	Alternatives to Animal Experimentation	2012
Cadmium	PREX1	Marco Fabbri et al	Whole genome analysis and microRNAs	Alternatives to Animal Experimentation	2012
Cadmium	C2CD2L	Marco Fabbri et al	Whole genome analysis and microRNAs	Alternatives to Animal Experimentation	2012
Cadmium	LOC100289494	Marco Fabbri et al	Whole genome analysis and microRNAs	Alternatives to Animal Experimentation	2012
Cadmium	ORAI2	Marco Fabbri et al	Whole genome analysis and microRNAs	Alternatives to Animal Experimentation	2012
Cadmium	GPR157	Marco Fabbri et al	Whole genome analysis and microRNAs	Alternatives to Animal Experimentation	2012
Cadmium	INPP1	Marco Fabbri et al	Whole genome analysis and microRNAs	Alternatives to Animal Experimentation	2012
Cadmium	TSPAN1	Marco Fabbri et al	Whole genome analysis and microRNAs	Alternatives to Animal Experimentation	2012
Cadmium	SAMD4A	Marco Fabbri et al	Whole genome analysis and microRNAs	Alternatives to Animal Experimentation	2012
Cadmium	GOLT1A	Marco Fabbri et al	Whole genome analysis and microRNAs	Alternatives to Animal Experimentation	2012
Cadmium	IFRD1	Marco Fabbri et al	Whole genome analysis and microRNAs	Alternatives to Animal Experimentation	2012
Cadmium	ECSCR	Marco Fabbri et al	Whole genome analysis and microRNAs	Alternatives to Animal Experimentation	2012
Cadmium	SNAPC4	Marco Fabbri et al	Whole genome analysis and microRNAs	Alternatives to Animal Experimentation	2012
Cadmium	CYB5R2	Marco Fabbri et al	Whole genome analysis and microRNAs	Alternatives to Animal Experimentation	2012
Cadmium	TNFRSF21	Marco Fabbri et al	Whole genome analysis and microRNAs	Alternatives to Animal Experimentation	2012
Cadmium	FOSL2	Marco Fabbri et al	Whole genome analysis and microRNAs	Alternatives to Animal Experimentation	2012
Cadmium	MALT1	Marco Fabbri et al	Whole genome analysis and microRNAs	Alternatives to Animal Experimentation	2012
Cadmium	FADS3	Marco Fabbri et al	Whole genome analysis and microRNAs	Alternatives to Animal Experimentation	2012
Cadmium	MTMR11	Marco Fabbri et al	Whole genome analysis and microRNAs	Alternatives to Animal Experimentation	2012
Cadmium	SLC43A2	Marco Fabbri et al	Whole genome analysis and microRNAs	Alternatives to Animal Experimentation	2012
Cadmium	SMOX	Marco Fabbri et al	Whole genome analysis and microRNAs	Alternatives to Animal Experimentation	2012
Cadmium	GPR126	Marco Fabbri et al	Whole genome analysis and microRNAs	Alternatives to Animal Experimentation	2012
Cadmium	DUSP5P	Marco Fabbri et al	Whole genome analysis and microRNAs	Alternatives to Animal Experimentation	2012
Cadmium	UBR4	Marco Fabbri et al	Whole genome analysis and microRNAs	Alternatives to Animal Experimentation	2012
Cadmium	SYMBOL	Marco Fabbri et al	Whole genome analysis and microRNAs	Alternatives to Animal Experimentation	2012

Cadmium	OXTR	Marco Fabbri et al	Whole genome analysis and microRNAs	Alternatives to Animal Experimentation	2012
Cadmium	PAK1	Marco Fabbri et al	Whole genome analysis and microRNAs	Alternatives to Animal Experimentation	2012
Cadmium	KCNN4	Marco Fabbri et al	Whole genome analysis and microRNAs	Alternatives to Animal Experimentation	2012
Cadmium	POLD4	Marco Fabbri et al	Whole genome analysis and microRNAs	Alternatives to Animal Experimentation	2012
Cadmium	NCEH1	Marco Fabbri et al	Whole genome analysis and microRNAs	Alternatives to Animal Experimentation	2012
Cadmium	LPCAT1	Marco Fabbri et al	Whole genome analysis and microRNAs	Alternatives to Animal Experimentation	2012
Cadmium	AHNAK2	Marco Fabbri et al	Whole genome analysis and microRNAs	Alternatives to Animal Experimentation	2012
Cadmium	DUSP18	Marco Fabbri et al	Whole genome analysis and microRNAs	Alternatives to Animal Experimentation	2012
Cadmium	HOMER2	Marco Fabbri et al	Whole genome analysis and microRNAs	Alternatives to Animal Experimentation	2012
Cadmium	VCY	Marco Fabbri et al	Whole genome analysis and microRNAs	Alternatives to Animal Experimentation	2012
Cadmium	RFTN1	Marco Fabbri et al	Whole genome analysis and microRNAs	Alternatives to Animal Experimentation	2012
Cadmium	EGLN1	Marco Fabbri et al	Whole genome analysis and microRNAs	Alternatives to Animal Experimentation	2012
Cadmium	PPP2R5C	Marco Fabbri et al	Whole genome analysis and microRNAs	Alternatives to Animal Experimentation	2012
Cadmium	VASP	Marco Fabbri et al	Whole genome analysis and microRNAs	Alternatives to Animal Experimentation	2012
Cadmium	ZBED2	Marco Fabbri et al	Whole genome analysis and microRNAs	Alternatives to Animal Experimentation	2012
Cadmium	CDC42EP2	Marco Fabbri et al	Whole genome analysis and microRNAs	Alternatives to Animal Experimentation	2012
Cadmium	SDCBP2	Marco Fabbri et al	Whole genome analysis and microRNAs	Alternatives to Animal Experimentation	2012
Cadmium	ABL2	Marco Fabbri et al	Whole genome analysis and microRNAs	Alternatives to Animal Experimentation	2012
Cadmium	TMEM173	Marco Fabbri et al	Whole genome analysis and microRNAs	Alternatives to Animal Experimentation	2012
Cadmium	TIE1	Marco Fabbri et al	Whole genome analysis and microRNAs	Alternatives to Animal Experimentation	2012
Cadmium	PPP1R2	Marco Fabbri et al	Whole genome analysis and microRNAs	Alternatives to Animal Experimentation	2012
Cadmium	BCAR3	Marco Fabbri et al	Whole genome analysis and microRNAs	Alternatives to Animal Experimentation	2012
Cadmium	CCDC84	Marco Fabbri et al	Whole genome analysis and microRNAs	Alternatives to Animal Experimentation	2012
Cadmium	FAM135A	Marco Fabbri et al	Whole genome analysis and microRNAs	Alternatives to Animal Experimentation	2012
Cadmium	ANTXR2	Marco Fabbri et al	Whole genome analysis and microRNAs	Alternatives to Animal Experimentation	2012
Cadmium	MTHFD1L	Marco Fabbri et al	Whole genome analysis and microRNAs	Alternatives to Animal Experimentation	2012
Cadmium	IQCG	Marco Fabbri et al	Whole genome analysis and microRNAs	Alternatives to Animal Experimentation	2012
Cadmium	HKDC1	Marco Fabbri et al	Whole genome analysis and microRNAs	Alternatives to Animal Experimentation	2012
Cadmium	GLIPR2	Marco Fabbri et al	Whole genome analysis and microRNAs	Alternatives to Animal Experimentation	2012
Cadmium	DGKG	Marco Fabbri et al	Whole genome analysis and microRNAs	Alternatives to Animal Experimentation	2012
Cadmium	RAB31	Marco Fabbri et al	Whole genome analysis and microRNAs	Alternatives to Animal Experimentation	2012
Cadmium	LIMS3	Marco Fabbri et al	Whole genome analysis and microRNAs	Alternatives to Animal Experimentation	2012
Cadmium	SYT11	Marco Fabbri et al	Whole genome analysis and microRNAs	Alternatives to Animal Experimentation	2012
Cadmium	SMYD3	Marco Fabbri et al	Whole genome analysis and microRNAs	Alternatives to Animal Experimentation	2012
Cadmium	TM4SF19	Marco Fabbri et al	Whole genome analysis and microRNAs	Alternatives to Animal Experimentation	2012
Cadmium	KRT12	Marco Fabbri et al	Whole genome analysis and microRNAs	Alternatives to Animal Experimentation	2012
Cadmium	TRMT1	Marco Fabbri et al	Whole genome analysis and microRNAs	Alternatives to Animal Experimentation	2012
Cadmium	ITGA6	Marco Fabbri et al	Whole genome analysis and microRNAs	Alternatives to Animal Experimentation	2012
Cadmium	MEI1	Marco Fabbri et al	Whole genome analysis and microRNAs	Alternatives to Animal Experimentation	2012
Cadmium	SLC16A4	Marco Fabbri et al	Whole genome analysis and microRNAs	Alternatives to Animal Experimentation	2012
Cadmium	RBM24	Marco Fabbri et al	Whole genome analysis and microRNAs	Alternatives to Animal Experimentation	2012
Cadmium	PDLIM7	Marco Fabbri et al	Whole genome analysis and microRNAs	Alternatives to Animal Experimentation	2012
Cadmium	CORO2B	Marco Fabbri et al	Whole genome analysis and microRNAs	Alternatives to Animal Experimentation	2012
Cadmium	HES2	Marco Fabbri et al	Whole genome analysis and microRNAs	Alternatives to Animal Experimentation	2012
Cadmium	SYMBOL	Marco Fabbri et al	Whole genome analysis and microRNAs	Alternatives to Animal Experimentation	2012
Cadmium	FAM59A	Marco Fabbri et al	Whole genome analysis and microRNAs	Alternatives to Animal Experimentation	2012
Cadmium	ANKRD30BP2	Marco Fabbri et al	Whole genome analysis and microRNAs	Alternatives to Animal Experimentation	2012
Cadmium	CTAG1A	Marco Fabbri et al	Whole genome analysis and microRNAs	Alternatives to Animal Experimentation	2012
Cadmium	NCRNA00162	Marco Fabbri et al	Whole genome analysis and microRNAs	Alternatives to Animal Experimentation	2012
Cadmium	TRIO	Marco Fabbri et al	Whole genome analysis and microRNAs	Alternatives to Animal Experimentation	2012
Cadmium	SRGAP1	Marco Fabbri et al	Whole genome analysis and microRNAs	Alternatives to Animal Experimentation	2012
Cadmium	TICAM1	Marco Fabbri et al	Whole genome analysis and microRNAs	Alternatives to Animal Experimentation	2012
Cadmium	SRSF12	Marco Fabbri et al	Whole genome analysis and microRNAs	Alternatives to Animal Experimentation	2012
Cadmium	BDH1	Marco Fabbri et al	Whole genome analysis and microRNAs	Alternatives to Animal Experimentation	2012
Cadmium	AGR2	Marco Fabbri et al	Whole genome analysis and microRNAs	Alternatives to Animal Experimentation	2012
Cadmium	YKT6	Marco Fabbri et al	Whole genome analysis and microRNAs	Alternatives to Animal Experimentation	2012
Cadmium	MICALL2	Marco Fabbri et al	Whole genome analysis and microRNAs	Alternatives to Animal Experimentation	2012
Cadmium	SLCO4A1	Marco Fabbri et al	Whole genome analysis and microRNAs	Alternatives to Animal Experimentation	2012
Cadmium	SMURF2	Marco Fabbri et al	Whole genome analysis and microRNAs	Alternatives to Animal Experimentation	2012
Cadmium	UBASH3B	Marco Fabbri et al	Whole genome analysis and microRNAs	Alternatives to Animal Experimentation	2012
Cadmium	LDHB	Marco Fabbri et al	Whole genome analysis and microRNAs	Alternatives to Animal Experimentation	2012
Cadmium	FLJ32255	Marco Fabbri et al	Whole genome analysis and microRNAs	Alternatives to Animal Experimentation	2012
Cadmium	TES	Marco Fabbri et al	Whole genome analysis and microRNAs	Alternatives to Animal Experimentation	2012
Cadmium	LRP10	Marco Fabbri et al	Whole genome analysis and microRNAs	Alternatives to Animal Experimentation	2012
Cadmium	TM4SF1	Marco Fabbri et al	Whole genome analysis and microRNAs	Alternatives to Animal Experimentation	2012
Cadmium	ACOT9	Marco Fabbri et al	Whole genome analysis and microRNAs	Alternatives to Animal Experimentation	2012
Cadmium	FSTL3	Marco Fabbri et al	Whole genome analysis and microRNAs	Alternatives to Animal Experimentation	2012
Cadmium	TRIM7	Marco Fabbri et al	Whole genome analysis and microRNAs	Alternatives to Animal Experimentation	2012
Cadmium	ARID3B	Marco Fabbri et al	Whole genome analysis and microRNAs	Alternatives to Animal Experimentation	2012
Cadmium	PAM	Marco Fabbri et al	Whole genome analysis and microRNAs	Alternatives to Animal Experimentation	2012
Cadmium	CDKN1A	Marco Fabbri et al	Whole genome analysis and microRNAs	Alternatives to Animal Experimentation	2012
Cadmium	STK17A	Marco Fabbri et al	Whole genome analysis and microRNAs	Alternatives to Animal Experimentation	2012
Cadmium	BAIAP2L2	Marco Fabbri et al	Whole genome analysis and microRNAs	Alternatives to Animal Experimentation	2012
Cadmium	ELF4	Marco Fabbri et al	Whole genome analysis and microRNAs	Alternatives to Animal Experimentation	2012
Cadmium	LOC151146	Marco Fabbri et al	Whole genome analysis and microRNAs	Alternatives to Animal Experimentation	2012
Cadmium	CLCN5	Marco Fabbri et al	Whole genome analysis and microRNAs	Alternatives to Animal Experimentation	2012
Cadmium	PLIN5	Marco Fabbri et al	Whole genome analysis and microRNAs	Alternatives to Animal Experimentation	2012
Cadmium	GEM	Marco Fabbri et al	Whole genome analysis and microRNAs	Alternatives to Animal Experimentation	2012
Cadmium	SGCB	Marco Fabbri et al	Whole genome analysis and microRNAs	Alternatives to Animal Experimentation	2012
Cadmium	UTP11L	Marco Fabbri et al	Whole genome analysis and microRNAs	Alternatives to Animal Experimentation	2012
Cadmium	EPS8L1	Marco Fabbri et al	Whole genome analysis and microRNAs	Alternatives to Animal Experimentation	2012
Cadmium	TGFBR2	Marco Fabbri et al	Whole genome analysis and microRNAs	Alternatives to Animal Experimentation	2012
Cadmium	C1ORF161	Marco Fabbri et al	Whole genome analysis and microRNAs	Alternatives to Animal Experimentation	2012
Cadmium	RHOQ	Marco Fabbri et al	Whole genome analysis and microRNAs	Alternatives to Animal Experimentation	2012
Cadmium	PFKFB3	Marco Fabbri et al	Whole genome analysis and microRNAs	Alternatives to Animal Experimentation	2012
Cadmium	UBL3	Marco Fabbri et al	Whole genome analysis and microRNAs	Alternatives to Animal Experimentation	2012
Cadmium	TLE3	Marco Fabbri et al	Whole genome analysis and microRNAs	Alternatives to Animal Experimentation	2012
Cadmium	MAP3K6	Marco Fabbri et al	Whole genome analysis and microRNAs	Alternatives to Animal Experimentation	2012
Cadmium	MYH9	Marco Fabbri et al	Whole genome analysis and microRNAs	Alternatives to Animal Experimentation	2012

Cadmium	MAP3K14	Marco Fabbri et al	Whole genome analysis and microRNAs	Alternatives to Animal Experimentation	2012
Cadmium	RRN3P3	Marco Fabbri et al	Whole genome analysis and microRNAs	Alternatives to Animal Experimentation	2012
Cadmium	PDLIM5	Marco Fabbri et al	Whole genome analysis and microRNAs	Alternatives to Animal Experimentation	2012
Cadmium	DOCK4	Marco Fabbri et al	Whole genome analysis and microRNAs	Alternatives to Animal Experimentation	2012
Cadmium	MACF1	Marco Fabbri et al	Whole genome analysis and microRNAs	Alternatives to Animal Experimentation	2012
Cadmium	RNF128	Marco Fabbri et al	Whole genome analysis and microRNAs	Alternatives to Animal Experimentation	2012
Cadmium	TMC7	Marco Fabbri et al	Whole genome analysis and microRNAs	Alternatives to Animal Experimentation	2012
Cadmium	BICD1	Marco Fabbri et al	Whole genome analysis and microRNAs	Alternatives to Animal Experimentation	2012
Cadmium	PLEC	Marco Fabbri et al	Whole genome analysis and microRNAs	Alternatives to Animal Experimentation	2012
Cadmium	ZNF556	Marco Fabbri et al	Whole genome analysis and microRNAs	Alternatives to Animal Experimentation	2012
Cadmium	CSNK1A1	Marco Fabbri et al	Whole genome analysis and microRNAs	Alternatives to Animal Experimentation	2012
Cadmium	LOC338620	Marco Fabbri et al	Whole genome analysis and microRNAs	Alternatives to Animal Experimentation	2012
Cadmium	SPOCK2	Marco Fabbri et al	Whole genome analysis and microRNAs	Alternatives to Animal Experimentation	2012
Cadmium	CDC42SE1	Marco Fabbri et al	Whole genome analysis and microRNAs	Alternatives to Animal Experimentation	2012
Cadmium	HSPBAP1	Marco Fabbri et al	Whole genome analysis and microRNAs	Alternatives to Animal Experimentation	2012
Cadmium	DBN1	Marco Fabbri et al	Whole genome analysis and microRNAs	Alternatives to Animal Experimentation	2012
Cadmium	EPHX1	Marco Fabbri et al	Whole genome analysis and microRNAs	Alternatives to Animal Experimentation	2012
Cadmium	HSD17B7	Marco Fabbri et al	Whole genome analysis and microRNAs	Alternatives to Animal Experimentation	2012
Cadmium	LOC729082	Marco Fabbri et al	Whole genome analysis and microRNAs	Alternatives to Animal Experimentation	2012
Cadmium	C5ORF13	Marco Fabbri et al	Whole genome analysis and microRNAs	Alternatives to Animal Experimentation	2012
Cadmium	POLR2J2	Marco Fabbri et al	Whole genome analysis and microRNAs	Alternatives to Animal Experimentation	2012
Cadmium	CYP8B1	Marco Fabbri et al	Whole genome analysis and microRNAs	Alternatives to Animal Experimentation	2012
Cadmium	ALDH1A1	Marco Fabbri et al	Whole genome analysis and microRNAs	Alternatives to Animal Experimentation	2012
Cadmium	GSTM4	Marco Fabbri et al	Whole genome analysis and microRNAs	Alternatives to Animal Experimentation	2012
Cadmium	LSS	Marco Fabbri et al	Whole genome analysis and microRNAs	Alternatives to Animal Experimentation	2012
Cadmium	TCP10L	Marco Fabbri et al	Whole genome analysis and microRNAs	Alternatives to Animal Experimentation	2012
Cadmium	CYP2B6	Marco Fabbri et al	Whole genome analysis and microRNAs	Alternatives to Animal Experimentation	2012
Cadmium	SLC25A1	Marco Fabbri et al	Whole genome analysis and microRNAs	Alternatives to Animal Experimentation	2012
Cadmium	CENPM	Marco Fabbri et al	Whole genome analysis and microRNAs	Alternatives to Animal Experimentation	2012
Cadmium	H1FX	Marco Fabbri et al	Whole genome analysis and microRNAs	Alternatives to Animal Experimentation	2012
Cadmium	PTPN18	Marco Fabbri et al	Whole genome analysis and microRNAs	Alternatives to Animal Experimentation	2012
Cadmium	CTDSP2	Marco Fabbri et al	Whole genome analysis and microRNAs	Alternatives to Animal Experimentation	2012
Cadmium	GM2A	Marco Fabbri et al	Whole genome analysis and microRNAs	Alternatives to Animal Experimentation	2012
Cadmium	ACSM5	Marco Fabbri et al	Whole genome analysis and microRNAs	Alternatives to Animal Experimentation	2012
Cadmium	DMGDH	Marco Fabbri et al	Whole genome analysis and microRNAs	Alternatives to Animal Experimentation	2012
Cadmium	C3	Marco Fabbri et al	Whole genome analysis and microRNAs	Alternatives to Animal Experimentation	2012
Cadmium	PBK	Marco Fabbri et al	Whole genome analysis and microRNAs	Alternatives to Animal Experimentation	2012
Cadmium	AATK	Marco Fabbri et al	Whole genome analysis and microRNAs	Alternatives to Animal Experimentation	2012
Cadmium	PANK1	Marco Fabbri et al	Whole genome analysis and microRNAs	Alternatives to Animal Experimentation	2012
Cadmium	CGREF1	Marco Fabbri et al	Whole genome analysis and microRNAs	Alternatives to Animal Experimentation	2012
Cadmium	DBP	Marco Fabbri et al	Whole genome analysis and microRNAs	Alternatives to Animal Experimentation	2012
Cadmium	STOX1	Marco Fabbri et al	Whole genome analysis and microRNAs	Alternatives to Animal Experimentation	2012
Cadmium	LPPR1	Marco Fabbri et al	Whole genome analysis and microRNAs	Alternatives to Animal Experimentation	2012
Cadmium	TLN2	Marco Fabbri et al	Whole genome analysis and microRNAs	Alternatives to Animal Experimentation	2012
Cadmium	HMGCS1	Marco Fabbri et al	Whole genome analysis and microRNAs	Alternatives to Animal Experimentation	2012
Cadmium	GPER	Marco Fabbri et al	Whole genome analysis and microRNAs	Alternatives to Animal Experimentation	2012
Cadmium	CDCA3	Marco Fabbri et al	Whole genome analysis and microRNAs	Alternatives to Animal Experimentation	2012
Cadmium	NCRNA00262	Marco Fabbri et al	Whole genome analysis and microRNAs	Alternatives to Animal Experimentation	2012
Cadmium	TMEM143	Marco Fabbri et al	Whole genome analysis and microRNAs	Alternatives to Animal Experimentation	2012
Cadmium	ALDH7A1	Marco Fabbri et al	Whole genome analysis and microRNAs	Alternatives to Animal Experimentation	2012
Cadmium	MOGAT2	Marco Fabbri et al	Whole genome analysis and microRNAs	Alternatives to Animal Experimentation	2012
Cadmium	ADH5	Marco Fabbri et al	Whole genome analysis and microRNAs	Alternatives to Animal Experimentation	2012
Cadmium	NCRNA00261	Marco Fabbri et al	Whole genome analysis and microRNAs	Alternatives to Animal Experimentation	2012
Cadmium	TNFAIP8L3	Marco Fabbri et al	Whole genome analysis and microRNAs	Alternatives to Animal Experimentation	2012
Cadmium	ALDH5A1	Marco Fabbri et al	Whole genome analysis and microRNAs	Alternatives to Animal Experimentation	2012
Cadmium	MTSS1L	Marco Fabbri et al	Whole genome analysis and microRNAs	Alternatives to Animal Experimentation	2012
Cadmium	CHEK2	Marco Fabbri et al	Whole genome analysis and microRNAs	Alternatives to Animal Experimentation	2012
Cadmium	HSDL2	Marco Fabbri et al	Whole genome analysis and microRNAs	Alternatives to Animal Experimentation	2012
Cadmium	CYP4F2	Marco Fabbri et al	Whole genome analysis and microRNAs	Alternatives to Animal Experimentation	2012
Cadmium	ABCB6	Marco Fabbri et al	Whole genome analysis and microRNAs	Alternatives to Animal Experimentation	2012
Cadmium	AHSG	Marco Fabbri et al	Whole genome analysis and microRNAs	Alternatives to Animal Experimentation	2012
Cadmium	FETUB	Marco Fabbri et al	Whole genome analysis and microRNAs	Alternatives to Animal Experimentation	2012
Cadmium	RAB26	Marco Fabbri et al	Whole genome analysis and microRNAs	Alternatives to Animal Experimentation	2012
Cadmium	HNF4A	Marco Fabbri et al	Whole genome analysis and microRNAs	Alternatives to Animal Experimentation	2012
Cadmium	ORM1	Marco Fabbri et al	Whole genome analysis and microRNAs	Alternatives to Animal Experimentation	2012
Cadmium	SLCO2B1	Marco Fabbri et al	Whole genome analysis and microRNAs	Alternatives to Animal Experimentation	2012
Cadmium	LOC80054	Marco Fabbri et al	Whole genome analysis and microRNAs	Alternatives to Animal Experimentation	2012
Cadmium	FNTB	Marco Fabbri et al	Whole genome analysis and microRNAs	Alternatives to Animal Experimentation	2012
Cadmium	PRPSAP1	Marco Fabbri et al	Whole genome analysis and microRNAs	Alternatives to Animal Experimentation	2012
Cadmium	SLC25A42	Marco Fabbri et al	Whole genome analysis and microRNAs	Alternatives to Animal Experimentation	2012
Cadmium	FBXO31	Marco Fabbri et al	Whole genome analysis and microRNAs	Alternatives to Animal Experimentation	2012
Cadmium	ARSE	Marco Fabbri et al	Whole genome analysis and microRNAs	Alternatives to Animal Experimentation	2012
Cadmium	FDFT1	Marco Fabbri et al	Whole genome analysis and microRNAs	Alternatives to Animal Experimentation	2012
Cadmium	SERPINA1	Marco Fabbri et al	Whole genome analysis and microRNAs	Alternatives to Animal Experimentation	2012
Cadmium	GLTPD2	Marco Fabbri et al	Whole genome analysis and microRNAs	Alternatives to Animal Experimentation	2012
Cadmium	GTSE1	Marco Fabbri et al	Whole genome analysis and microRNAs	Alternatives to Animal Experimentation	2012
Cadmium	CDT1	Marco Fabbri et al	Whole genome analysis and microRNAs	Alternatives to Animal Experimentation	2012
Cadmium	SORD	Marco Fabbri et al	Whole genome analysis and microRNAs	Alternatives to Animal Experimentation	2012
Cadmium	CLGN	Marco Fabbri et al	Whole genome analysis and microRNAs	Alternatives to Animal Experimentation	2012
Cadmium	AUH	Marco Fabbri et al	Whole genome analysis and microRNAs	Alternatives to Animal Experimentation	2012
Cadmium	SLC29A4	Marco Fabbri et al	Whole genome analysis and microRNAs	Alternatives to Animal Experimentation	2012
Cadmium	ANTXR1	Marco Fabbri et al	Whole genome analysis and microRNAs	Alternatives to Animal Experimentation	2012
Cadmium	AFMID	Marco Fabbri et al	Whole genome analysis and microRNAs	Alternatives to Animal Experimentation	2012
Cadmium	SORBS3	Marco Fabbri et al	Whole genome analysis and microRNAs	Alternatives to Animal Experimentation	2012
Cadmium	RARRES2	Marco Fabbri et al	Whole genome analysis and microRNAs	Alternatives to Animal Experimentation	2012
Cadmium	PSPH	Marco Fabbri et al	Whole genome analysis and microRNAs	Alternatives to Animal Experimentation	2012
Cadmium	CIDEB	Marco Fabbri et al	Whole genome analysis and microRNAs	Alternatives to Animal Experimentation	2012
Cadmium	SEMA6B	Marco Fabbri et al	Whole genome analysis and microRNAs	Alternatives to Animal Experimentation	2012
Cadmium	TJP3	Marco Fabbri et al	Whole genome analysis and microRNAs	Alternatives to Animal Experimentation	2012

Cadmium	SORBS2	Marco Fabbri et al	Whole genome analysis and microRNAs	Alternatives to Animal Experimentation	2012
Cadmium	GALM	Marco Fabbri et al	Whole genome analysis and microRNAs	Alternatives to Animal Experimentation	2012
Cadmium	SQLE	Marco Fabbri et al	Whole genome analysis and microRNAs	Alternatives to Animal Experimentation	2012
Cadmium	PRUNE	Marco Fabbri et al	Whole genome analysis and microRNAs	Alternatives to Animal Experimentation	2012
Cadmium	CAT	Marco Fabbri et al	Whole genome analysis and microRNAs	Alternatives to Animal Experimentation	2012
Cadmium	NEDD4L	Marco Fabbri et al	Whole genome analysis and microRNAs	Alternatives to Animal Experimentation	2012
Cadmium	ADORA2A	Marco Fabbri et al	Whole genome analysis and microRNAs	Alternatives to Animal Experimentation	2012
Cadmium	EHHADH	Marco Fabbri et al	Whole genome analysis and microRNAs	Alternatives to Animal Experimentation	2012
Cadmium	KHK	Marco Fabbri et al	Whole genome analysis and microRNAs	Alternatives to Animal Experimentation	2012
Cadmium	SGK3	Marco Fabbri et al	Whole genome analysis and microRNAs	Alternatives to Animal Experimentation	2012
Cadmium	ANGPTL1	Marco Fabbri et al	Whole genome analysis and microRNAs	Alternatives to Animal Experimentation	2012
Cadmium	SLA2	Marco Fabbri et al	Whole genome analysis and microRNAs	Alternatives to Animal Experimentation	2012
Cadmium	CCDC34	Marco Fabbri et al	Whole genome analysis and microRNAs	Alternatives to Animal Experimentation	2012
Cadmium	C12ORF34	Marco Fabbri et al	Whole genome analysis and microRNAs	Alternatives to Animal Experimentation	2012
Cadmium	KIF11	Marco Fabbri et al	Whole genome analysis and microRNAs	Alternatives to Animal Experimentation	2012
Cadmium	TMEM121	Marco Fabbri et al	Whole genome analysis and microRNAs	Alternatives to Animal Experimentation	2012
Cadmium	CYP19A1	Marco Fabbri et al	Whole genome analysis and microRNAs	Alternatives to Animal Experimentation	2012
Cadmium	CCDC142	Marco Fabbri et al	Whole genome analysis and microRNAs	Alternatives to Animal Experimentation	2012
Cadmium	DAPK1	Marco Fabbri et al	Whole genome analysis and microRNAs	Alternatives to Animal Experimentation	2012
Cadmium	SPC24	Marco Fabbri et al	Whole genome analysis and microRNAs	Alternatives to Animal Experimentation	2012
Cadmium	SHPK	Marco Fabbri et al	Whole genome analysis and microRNAs	Alternatives to Animal Experimentation	2012
Cadmium	DAO	Marco Fabbri et al	Whole genome analysis and microRNAs	Alternatives to Animal Experimentation	2012
Cadmium	HLF	Marco Fabbri et al	Whole genome analysis and microRNAs	Alternatives to Animal Experimentation	2012
Cadmium	MPV17L	Marco Fabbri et al	Whole genome analysis and microRNAs	Alternatives to Animal Experimentation	2012
Cadmium	LASS4	Marco Fabbri et al	Whole genome analysis and microRNAs	Alternatives to Animal Experimentation	2012
Cadmium	TMEM141	Marco Fabbri et al	Whole genome analysis and microRNAs	Alternatives to Animal Experimentation	2012
Cadmium	LOC401022	Marco Fabbri et al	Whole genome analysis and microRNAs	Alternatives to Animal Experimentation	2012
Cadmium	CDC25C	Marco Fabbri et al	Whole genome analysis and microRNAs	Alternatives to Animal Experimentation	2012
Cadmium	HSD17B4	Marco Fabbri et al	Whole genome analysis and microRNAs	Alternatives to Animal Experimentation	2012
Cadmium	SERPINA7	Marco Fabbri et al	Whole genome analysis and microRNAs	Alternatives to Animal Experimentation	2012
Cadmium	SOAT2	Marco Fabbri et al	Whole genome analysis and microRNAs	Alternatives to Animal Experimentation	2012
Cadmium	SULT1C2	Marco Fabbri et al	Whole genome analysis and microRNAs	Alternatives to Animal Experimentation	2012
Cadmium	PHLPP1	Marco Fabbri et al	Whole genome analysis and microRNAs	Alternatives to Animal Experimentation	2012
Cadmium	IGDCC3	Marco Fabbri et al	Whole genome analysis and microRNAs	Alternatives to Animal Experimentation	2012
Cadmium	DQX1	Marco Fabbri et al	Whole genome analysis and microRNAs	Alternatives to Animal Experimentation	2012
Cadmium	CYP4F8	Marco Fabbri et al	Whole genome analysis and microRNAs	Alternatives to Animal Experimentation	2012
Cadmium	TMEM149	Marco Fabbri et al	Whole genome analysis and microRNAs	Alternatives to Animal Experimentation	2012
Cadmium	XBP1	Marco Fabbri et al	Whole genome analysis and microRNAs	Alternatives to Animal Experimentation	2012
Cadmium	C10ORF114	Marco Fabbri et al	Whole genome analysis and microRNAs	Alternatives to Animal Experimentation	2012
Cadmium	GPAM	Marco Fabbri et al	Whole genome analysis and microRNAs	Alternatives to Animal Experimentation	2012
Cadmium	ACAA1	Marco Fabbri et al	Whole genome analysis and microRNAs	Alternatives to Animal Experimentation	2012
Cadmium	KIF20B	Marco Fabbri et al	Whole genome analysis and microRNAs	Alternatives to Animal Experimentation	2012
Cadmium	SHMT1	Marco Fabbri et al	Whole genome analysis and microRNAs	Alternatives to Animal Experimentation	2012
Cadmium	SYMBOL	Marco Fabbri et al	Whole genome analysis and microRNAs	Alternatives to Animal Experimentation	2012
Cadmium	C1ORF186	Marco Fabbri et al	Whole genome analysis and microRNAs	Alternatives to Animal Experimentation	2012
Cadmium	FMO5	Marco Fabbri et al	Whole genome analysis and microRNAs	Alternatives to Animal Experimentation	2012
Cadmium	GRTP1	Marco Fabbri et al	Whole genome analysis and microRNAs	Alternatives to Animal Experimentation	2012
Cadmium	SKP2	Marco Fabbri et al	Whole genome analysis and microRNAs	Alternatives to Animal Experimentation	2012
Cadmium	APOH	Marco Fabbri et al	Whole genome analysis and microRNAs	Alternatives to Animal Experimentation	2012
Cadmium	C16ORF79	Marco Fabbri et al	Whole genome analysis and microRNAs	Alternatives to Animal Experimentation	2012
Cadmium	MVD	Marco Fabbri et al	Whole genome analysis and microRNAs	Alternatives to Animal Experimentation	2012
Cadmium	NIPSNAP1	Marco Fabbri et al	Whole genome analysis and microRNAs	Alternatives to Animal Experimentation	2012
Cadmium	DHFR	Marco Fabbri et al	Whole genome analysis and microRNAs	Alternatives to Animal Experimentation	2012
Cadmium	RHBG	Marco Fabbri et al	Whole genome analysis and microRNAs	Alternatives to Animal Experimentation	2012
Cadmium	OBSL1	Marco Fabbri et al	Whole genome analysis and microRNAs	Alternatives to Animal Experimentation	2012
Cadmium	PCK2	Marco Fabbri et al	Whole genome analysis and microRNAs	Alternatives to Animal Experimentation	2012
Cadmium	FBXW2	Marco Fabbri et al	Whole genome analysis and microRNAs	Alternatives to Animal Experimentation	2012
Cadmium	C9ORF100	Marco Fabbri et al	Whole genome analysis and microRNAs	Alternatives to Animal Experimentation	2012
Cadmium	KAZALD1	Marco Fabbri et al	Whole genome analysis and microRNAs	Alternatives to Animal Experimentation	2012
Cadmium	ECH1	Marco Fabbri et al	Whole genome analysis and microRNAs	Alternatives to Animal Experimentation	2012
Cadmium	TMEM97	Marco Fabbri et al	Whole genome analysis and microRNAs	Alternatives to Animal Experimentation	2012
Cadmium	FRAT2	Marco Fabbri et al	Whole genome analysis and microRNAs	Alternatives to Animal Experimentation	2012
Cadmium	CRYL1	Marco Fabbri et al	Whole genome analysis and microRNAs	Alternatives to Animal Experimentation	2012
Cadmium	IL11RA	Marco Fabbri et al	Whole genome analysis and microRNAs	Alternatives to Animal Experimentation	2012
Cadmium	PPARGC1A	Marco Fabbri et al	Whole genome analysis and microRNAs	Alternatives to Animal Experimentation	2012
Cadmium	HMGB2	Marco Fabbri et al	Whole genome analysis and microRNAs	Alternatives to Animal Experimentation	2012
Cadmium	AGT	Marco Fabbri et al	Whole genome analysis and microRNAs	Alternatives to Animal Experimentation	2012
Cadmium	WFIKKN1	Marco Fabbri et al	Whole genome analysis and microRNAs	Alternatives to Animal Experimentation	2012
Cadmium	RPS15A	Marco Fabbri et al	Whole genome analysis and microRNAs	Alternatives to Animal Experimentation	2012
Cadmium	MASP2	Marco Fabbri et al	Whole genome analysis and microRNAs	Alternatives to Animal Experimentation	2012
Cadmium	KIF22	Marco Fabbri et al	Whole genome analysis and microRNAs	Alternatives to Animal Experimentation	2012
Cadmium	DLK1	Marco Fabbri et al	Whole genome analysis and microRNAs	Alternatives to Animal Experimentation	2012
Cadmium	CEBPE	Marco Fabbri et al	Whole genome analysis and microRNAs	Alternatives to Animal Experimentation	2012
Cadmium	IFIH1	Marco Fabbri et al	Whole genome analysis and microRNAs	Alternatives to Animal Experimentation	2012
Cadmium	CYP4F12	Marco Fabbri et al	Whole genome analysis and microRNAs	Alternatives to Animal Experimentation	2012
Cadmium	FADS1	Marco Fabbri et al	Whole genome analysis and microRNAs	Alternatives to Animal Experimentation	2012
Cadmium	USP18	Marco Fabbri et al	Whole genome analysis and microRNAs	Alternatives to Animal Experimentation	2012
Cadmium	ACAD11	Marco Fabbri et al	Whole genome analysis and microRNAs	Alternatives to Animal Experimentation	2012
Cadmium	RPS6KA3	Marco Fabbri et al	Whole genome analysis and microRNAs	Alternatives to Animal Experimentation	2012
Cadmium	TNFAIP8L1	Marco Fabbri et al	Whole genome analysis and microRNAs	Alternatives to Animal Experimentation	2012
Cadmium	ACSL4	Marco Fabbri et al	Whole genome analysis and microRNAs	Alternatives to Animal Experimentation	2012
Cadmium	GALK1	Marco Fabbri et al	Whole genome analysis and microRNAs	Alternatives to Animal Experimentation	2012
Cadmium	SEMA4G	Marco Fabbri et al	Whole genome analysis and microRNAs	Alternatives to Animal Experimentation	2012
Cadmium	APOA1	Marco Fabbri et al	Whole genome analysis and microRNAs	Alternatives to Animal Experimentation	2012
Cadmium	GLUL	Marco Fabbri et al	Whole genome analysis and microRNAs	Alternatives to Animal Experimentation	2012
Cadmium	DHCR24	Marco Fabbri et al	Whole genome analysis and microRNAs	Alternatives to Animal Experimentation	2012
Cadmium	HIST3H2BB	Marco Fabbri et al	Whole genome analysis and microRNAs	Alternatives to Animal Experimentation	2012
Cadmium	SERPINF1	Marco Fabbri et al	Whole genome analysis and microRNAs	Alternatives to Animal Experimentation	2012
Cadmium	SELENBP1	Marco Fabbri et al	Whole genome analysis and microRNAs	Alternatives to Animal Experimentation	2012

Cadmium	TMCO6	Marco Fabbri et al	Whole genome analysis and microRNAs	Alternatives to Animal Experimentation	2012
Cadmium	SCRN2	Marco Fabbri et al	Whole genome analysis and microRNAs	Alternatives to Animal Experimentation	2012
Cadmium	SECTM1	Marco Fabbri et al	Whole genome analysis and microRNAs	Alternatives to Animal Experimentation	2012
Cadmium	SOBP	Marco Fabbri et al	Whole genome analysis and microRNAs	Alternatives to Animal Experimentation	2012
Cadmium	KIF15	Marco Fabbri et al	Whole genome analysis and microRNAs	Alternatives to Animal Experimentation	2012
Cadmium	AMT	Marco Fabbri et al	Whole genome analysis and microRNAs	Alternatives to Animal Experimentation	2012
Cadmium	PHF17	Marco Fabbri et al	Whole genome analysis and microRNAs	Alternatives to Animal Experimentation	2012
Cadmium	C18ORF56	Marco Fabbri et al	Whole genome analysis and microRNAs	Alternatives to Animal Experimentation	2012
Cadmium	LOC645722	Marco Fabbri et al	Whole genome analysis and microRNAs	Alternatives to Animal Experimentation	2012
Cadmium	HAAO	Marco Fabbri et al	Whole genome analysis and microRNAs	Alternatives to Animal Experimentation	2012
Cadmium	PRIMA1	Marco Fabbri et al	Whole genome analysis and microRNAs	Alternatives to Animal Experimentation	2012
Cadmium	A2M	Marco Fabbri et al	Whole genome analysis and microRNAs	Alternatives to Animal Experimentation	2012
Cadmium	MYL5	Marco Fabbri et al	Whole genome analysis and microRNAs	Alternatives to Animal Experimentation	2012
Cadmium	ITIH2	Marco Fabbri et al	Whole genome analysis and microRNAs	Alternatives to Animal Experimentation	2012
Cadmium	CABLES2	Marco Fabbri et al	Whole genome analysis and microRNAs	Alternatives to Animal Experimentation	2012
Cadmium	PLA2G12B	Marco Fabbri et al	Whole genome analysis and microRNAs	Alternatives to Animal Experimentation	2012
Cadmium	ESPN	Marco Fabbri et al	Whole genome analysis and microRNAs	Alternatives to Animal Experimentation	2012
Cadmium	KCNK7	Marco Fabbri et al	Whole genome analysis and microRNAs	Alternatives to Animal Experimentation	2012
Cadmium	GPX2	Marco Fabbri et al	Whole genome analysis and microRNAs	Alternatives to Animal Experimentation	2012
Cadmium	PGM1	Marco Fabbri et al	Whole genome analysis and microRNAs	Alternatives to Animal Experimentation	2012
Cadmium	MCM2	Marco Fabbri et al	Whole genome analysis and microRNAs	Alternatives to Animal Experimentation	2012
Cadmium	TCEA2	Marco Fabbri et al	Whole genome analysis and microRNAs	Alternatives to Animal Experimentation	2012
Cadmium	AGMAT	Marco Fabbri et al	Whole genome analysis and microRNAs	Alternatives to Animal Experimentation	2012
Cadmium	C21ORF45	Marco Fabbri et al	Whole genome analysis and microRNAs	Alternatives to Animal Experimentation	2012
Cadmium	FAM20C	Marco Fabbri et al	Whole genome analysis and microRNAs	Alternatives to Animal Experimentation	2012
Cadmium	F11	Marco Fabbri et al	Whole genome analysis and microRNAs	Alternatives to Animal Experimentation	2012
Cadmium	FOXD2	Marco Fabbri et al	Whole genome analysis and microRNAs	Alternatives to Animal Experimentation	2012
Cadmium	DHDPSL	Marco Fabbri et al	Whole genome analysis and microRNAs	Alternatives to Animal Experimentation	2012
Cadmium	NFE2	Marco Fabbri et al	Whole genome analysis and microRNAs	Alternatives to Animal Experimentation	2012
Cadmium	UPB1	Marco Fabbri et al	Whole genome analysis and microRNAs	Alternatives to Animal Experimentation	2012
Cadmium	C5ORF33	Marco Fabbri et al	Whole genome analysis and microRNAs	Alternatives to Animal Experimentation	2012
Cadmium	NRTN	Marco Fabbri et al	Whole genome analysis and microRNAs	Alternatives to Animal Experimentation	2012
Cadmium	C16ORF3	Marco Fabbri et al	Whole genome analysis and microRNAs	Alternatives to Animal Experimentation	2012
Cadmium	AVPI1	Marco Fabbri et al	Whole genome analysis and microRNAs	Alternatives to Animal Experimentation	2012
Cadmium	SHROOM1	Marco Fabbri et al	Whole genome analysis and microRNAs	Alternatives to Animal Experimentation	2012
Cadmium	AKR1D1	Marco Fabbri et al	Whole genome analysis and microRNAs	Alternatives to Animal Experimentation	2012
Cadmium	C5ORF4	Marco Fabbri et al	Whole genome analysis and microRNAs	Alternatives to Animal Experimentation	2012
Cadmium	AMDHD1	Marco Fabbri et al	Whole genome analysis and microRNAs	Alternatives to Animal Experimentation	2012
Cadmium	APOA4	Marco Fabbri et al	Whole genome analysis and microRNAs	Alternatives to Animal Experimentation	2012
Cadmium	GAMT	Marco Fabbri et al	Whole genome analysis and microRNAs	Alternatives to Animal Experimentation	2012
Cadmium	IDI1	Marco Fabbri et al	Whole genome analysis and microRNAs	Alternatives to Animal Experimentation	2012
Cadmium	ITIH3	Marco Fabbri et al	Whole genome analysis and microRNAs	Alternatives to Animal Experimentation	2012
Cadmium	ANXA9	Marco Fabbri et al	Whole genome analysis and microRNAs	Alternatives to Animal Experimentation	2012
Cadmium	SMAD9	Marco Fabbri et al	Whole genome analysis and microRNAs	Alternatives to Animal Experimentation	2012
Cadmium	GLYCTK	Marco Fabbri et al	Whole genome analysis and microRNAs	Alternatives to Animal Experimentation	2012
Cadmium	HIST1H2BD	Marco Fabbri et al	Whole genome analysis and microRNAs	Alternatives to Animal Experimentation	2012
Cadmium	POC1A	Marco Fabbri et al	Whole genome analysis and microRNAs	Alternatives to Animal Experimentation	2012
Cadmium	SLC22A3	Marco Fabbri et al	Whole genome analysis and microRNAs	Alternatives to Animal Experimentation	2012
Cadmium	SLC25A33	Marco Fabbri et al	Whole genome analysis and microRNAs	Alternatives to Animal Experimentation	2012
Cadmium	GYG2	Marco Fabbri et al	Whole genome analysis and microRNAs	Alternatives to Animal Experimentation	2012
Cadmium	PKLR	Marco Fabbri et al	Whole genome analysis and microRNAs	Alternatives to Animal Experimentation	2012
Cadmium	IGF2	Marco Fabbri et al	Whole genome analysis and microRNAs	Alternatives to Animal Experimentation	2012
Cadmium	C16ORF75	Marco Fabbri et al	Whole genome analysis and microRNAs	Alternatives to Animal Experimentation	2012
Cadmium	NUPR1	Marco Fabbri et al	Whole genome analysis and microRNAs	Alternatives to Animal Experimentation	2012
Cadmium	ASF1B	Marco Fabbri et al	Whole genome analysis and microRNAs	Alternatives to Animal Experimentation	2012
Cadmium	FAM117A	Marco Fabbri et al	Whole genome analysis and microRNAs	Alternatives to Animal Experimentation	2012
Cadmium	CA5A	Marco Fabbri et al	Whole genome analysis and microRNAs	Alternatives to Animal Experimentation	2012
Cadmium	E2F8	Marco Fabbri et al	Whole genome analysis and microRNAs	Alternatives to Animal Experimentation	2012
Cadmium	KIRREL2	Marco Fabbri et al	Whole genome analysis and microRNAs	Alternatives to Animal Experimentation	2012
Cadmium	ABCC6	Marco Fabbri et al	Whole genome analysis and microRNAs	Alternatives to Animal Experimentation	2012
Cadmium	BRP44L	Marco Fabbri et al	Whole genome analysis and microRNAs	Alternatives to Animal Experimentation	2012
Cadmium	C1S	Marco Fabbri et al	Whole genome analysis and microRNAs	Alternatives to Animal Experimentation	2012
Cadmium	LDLRAD1	Marco Fabbri et al	Whole genome analysis and microRNAs	Alternatives to Animal Experimentation	2012
Cadmium	CYP2W1	Marco Fabbri et al	Whole genome analysis and microRNAs	Alternatives to Animal Experimentation	2012
Cadmium	SLC23A1	Marco Fabbri et al	Whole genome analysis and microRNAs	Alternatives to Animal Experimentation	2012
Cadmium	KLC4	Marco Fabbri et al	Whole genome analysis and microRNAs	Alternatives to Animal Experimentation	2012
Cadmium	HNMT	Marco Fabbri et al	Whole genome analysis and microRNAs	Alternatives to Animal Experimentation	2012
Cadmium	SERPINA3	Marco Fabbri et al	Whole genome analysis and microRNAs	Alternatives to Animal Experimentation	2012
Cadmium	PCOLCE	Marco Fabbri et al	Whole genome analysis and microRNAs	Alternatives to Animal Experimentation	2012
Cadmium	C2	Marco Fabbri et al	Whole genome analysis and microRNAs	Alternatives to Animal Experimentation	2012
Cadmium	KLHDC2	Marco Fabbri et al	Whole genome analysis and microRNAs	Alternatives to Animal Experimentation	2012
Cadmium	HMGCR	Marco Fabbri et al	Whole genome analysis and microRNAs	Alternatives to Animal Experimentation	2012
Cadmium	FGFR2	Marco Fabbri et al	Whole genome analysis and microRNAs	Alternatives to Animal Experimentation	2012
Cadmium	TIGD3	Marco Fabbri et al	Whole genome analysis and microRNAs	Alternatives to Animal Experimentation	2012
Cadmium	CHST13	Marco Fabbri et al	Whole genome analysis and microRNAs	Alternatives to Animal Experimentation	2012
Cadmium	VTN	Marco Fabbri et al	Whole genome analysis and microRNAs	Alternatives to Animal Experimentation	2012
Cadmium	FNDC5	Marco Fabbri et al	Whole genome analysis and microRNAs	Alternatives to Animal Experimentation	2012
Cadmium	PAQR8	Marco Fabbri et al	Whole genome analysis and microRNAs	Alternatives to Animal Experimentation	2012
Cadmium	MVK	Marco Fabbri et al	Whole genome analysis and microRNAs	Alternatives to Animal Experimentation	2012
Cadmium	SFXN2	Marco Fabbri et al	Whole genome analysis and microRNAs	Alternatives to Animal Experimentation	2012
Cadmium	IGFALS	Marco Fabbri et al	Whole genome analysis and microRNAs	Alternatives to Animal Experimentation	2012
Cadmium	PLIN1	Marco Fabbri et al	Whole genome analysis and microRNAs	Alternatives to Animal Experimentation	2012
Cadmium	RBP4	Marco Fabbri et al	Whole genome analysis and microRNAs	Alternatives to Animal Experimentation	2012
Cadmium	ACOT2	Marco Fabbri et al	Whole genome analysis and microRNAs	Alternatives to Animal Experimentation	2012
Cadmium	IMPA2	Marco Fabbri et al	Whole genome analysis and microRNAs	Alternatives to Animal Experimentation	2012
Cadmium	ASB9	Marco Fabbri et al	Whole genome analysis and microRNAs	Alternatives to Animal Experimentation	2012
Cadmium	TM7SF2	Marco Fabbri et al	Whole genome analysis and microRNAs	Alternatives to Animal Experimentation	2012
Cadmium	TMEM37	Marco Fabbri et al	Whole genome analysis and microRNAs	Alternatives to Animal Experimentation	2012
Cadmium	PCSK6	Marco Fabbri et al	Whole genome analysis and microRNAs	Alternatives to Animal Experimentation	2012

Cadmium	ATP6V0E2	Marco Fabbri et al	Whole genome analysis and microRNAs	Alternatives to Animal Experimentation	2012
Cadmium	MMAB	Marco Fabbri et al	Whole genome analysis and microRNAs	Alternatives to Animal Experimentation	2012
Cadmium	TTC39C	Marco Fabbri et al	Whole genome analysis and microRNAs	Alternatives to Animal Experimentation	2012
Cadmium	IL1RAP	Marco Fabbri et al	Whole genome analysis and microRNAs	Alternatives to Animal Experimentation	2012
Cadmium	FAM151A	Marco Fabbri et al	Whole genome analysis and microRNAs	Alternatives to Animal Experimentation	2012
Cadmium	LRP11	Marco Fabbri et al	Whole genome analysis and microRNAs	Alternatives to Animal Experimentation	2012
Cadmium	GRB7	Marco Fabbri et al	Whole genome analysis and microRNAs	Alternatives to Animal Experimentation	2012
Cadmium	EPHA1	Marco Fabbri et al	Whole genome analysis and microRNAs	Alternatives to Animal Experimentation	2012
Cadmium	CPN2	Marco Fabbri et al	Whole genome analysis and microRNAs	Alternatives to Animal Experimentation	2012
Cadmium	SCARA3	Marco Fabbri et al	Whole genome analysis and microRNAs	Alternatives to Animal Experimentation	2012
Cadmium	SERPINA5	Marco Fabbri et al	Whole genome analysis and microRNAs	Alternatives to Animal Experimentation	2012
Cadmium	MID1IP1	Marco Fabbri et al	Whole genome analysis and microRNAs	Alternatives to Animal Experimentation	2012
Cadmium	ST6GAL1	Marco Fabbri et al	Whole genome analysis and microRNAs	Alternatives to Animal Experimentation	2012
Cadmium	ACAT2	Marco Fabbri et al	Whole genome analysis and microRNAs	Alternatives to Animal Experimentation	2012
Cadmium	SLC37A4	Marco Fabbri et al	Whole genome analysis and microRNAs	Alternatives to Animal Experimentation	2012
Cadmium	SLC26A1	Marco Fabbri et al	Whole genome analysis and microRNAs	Alternatives to Animal Experimentation	2012
Cadmium	OSTALPHA	Marco Fabbri et al	Whole genome analysis and microRNAs	Alternatives to Animal Experimentation	2012
Cadmium	PZP	Marco Fabbri et al	Whole genome analysis and microRNAs	Alternatives to Animal Experimentation	2012
Cadmium	TMEM135	Marco Fabbri et al	Whole genome analysis and microRNAs	Alternatives to Animal Experimentation	2012
Cadmium	ACADSB	Marco Fabbri et al	Whole genome analysis and microRNAs	Alternatives to Animal Experimentation	2012
Cadmium	LDHD	Marco Fabbri et al	Whole genome analysis and microRNAs	Alternatives to Animal Experimentation	2012
Cadmium	A1BG	Marco Fabbri et al	Whole genome analysis and microRNAs	Alternatives to Animal Experimentation	2012
Cadmium	CFI	Marco Fabbri et al	Whole genome analysis and microRNAs	Alternatives to Animal Experimentation	2012
Cadmium	CYP27A1	Marco Fabbri et al	Whole genome analysis and microRNAs	Alternatives to Animal Experimentation	2012
Cadmium	PECR	Marco Fabbri et al	Whole genome analysis and microRNAs	Alternatives to Animal Experimentation	2012
Cadmium	TF	Marco Fabbri et al	Whole genome analysis and microRNAs	Alternatives to Animal Experimentation	2012
Cadmium	LIPC	Marco Fabbri et al	Whole genome analysis and microRNAs	Alternatives to Animal Experimentation	2012
Cadmium	LOC151534	Marco Fabbri et al	Whole genome analysis and microRNAs	Alternatives to Animal Experimentation	2012
Cadmium	C8B	Marco Fabbri et al	Whole genome analysis and microRNAs	Alternatives to Animal Experimentation	2012
Cadmium	PCYT2	Marco Fabbri et al	Whole genome analysis and microRNAs	Alternatives to Animal Experimentation	2012
Cadmium	FOXA1	Marco Fabbri et al	Whole genome analysis and microRNAs	Alternatives to Animal Experimentation	2012
Cadmium	AIM1	Marco Fabbri et al	Whole genome analysis and microRNAs	Alternatives to Animal Experimentation	2012
Cadmium	ACSM2A	Marco Fabbri et al	Whole genome analysis and microRNAs	Alternatives to Animal Experimentation	2012
Cadmium	PRR15L	Marco Fabbri et al	Whole genome analysis and microRNAs	Alternatives to Animal Experimentation	2012
Cadmium	PHGDH	Marco Fabbri et al	Whole genome analysis and microRNAs	Alternatives to Animal Experimentation	2012
Cadmium	C18ORF1	Marco Fabbri et al	Whole genome analysis and microRNAs	Alternatives to Animal Experimentation	2012
Cadmium	EFNA1	Marco Fabbri et al	Whole genome analysis and microRNAs	Alternatives to Animal Experimentation	2012
Cadmium	C4BPA	Marco Fabbri et al	Whole genome analysis and microRNAs	Alternatives to Animal Experimentation	2012
Cadmium	INHBE	Marco Fabbri et al	Whole genome analysis and microRNAs	Alternatives to Animal Experimentation	2012
Cadmium	MAT1A	Marco Fabbri et al	Whole genome analysis and microRNAs	Alternatives to Animal Experimentation	2012
Cadmium	C14ORF73	Marco Fabbri et al	Whole genome analysis and microRNAs	Alternatives to Animal Experimentation	2012
Cadmium	PIPOX	Marco Fabbri et al	Whole genome analysis and microRNAs	Alternatives to Animal Experimentation	2012
Cadmium	ADH4	Marco Fabbri et al	Whole genome analysis and microRNAs	Alternatives to Animal Experimentation	2012
Cadmium	SLC2A4RG	Marco Fabbri et al	Whole genome analysis and microRNAs	Alternatives to Animal Experimentation	2012
Cadmium	CCDC69	Marco Fabbri et al	Whole genome analysis and microRNAs	Alternatives to Animal Experimentation	2012
Cadmium	SLC39A5	Marco Fabbri et al	Whole genome analysis and microRNAs	Alternatives to Animal Experimentation	2012
Cadmium	PGLYRP2	Marco Fabbri et al	Whole genome analysis and microRNAs	Alternatives to Animal Experimentation	2012
Cadmium	DAK	Marco Fabbri et al	Whole genome analysis and microRNAs	Alternatives to Animal Experimentation	2012
Cadmium	CDKN2C	Marco Fabbri et al	Whole genome analysis and microRNAs	Alternatives to Animal Experimentation	2012
Cadmium	HHEX	Marco Fabbri et al	Whole genome analysis and microRNAs	Alternatives to Animal Experimentation	2012
Cadmium	ERBB3	Marco Fabbri et al	Whole genome analysis and microRNAs	Alternatives to Animal Experimentation	2012
Cadmium	NDRG2	Marco Fabbri et al	Whole genome analysis and microRNAs	Alternatives to Animal Experimentation	2012
Cadmium	ACSS1	Marco Fabbri et al	Whole genome analysis and microRNAs	Alternatives to Animal Experimentation	2012
Cadmium	SERPINA11	Marco Fabbri et al	Whole genome analysis and microRNAs	Alternatives to Animal Experimentation	2012
Cadmium	TNNI2	Marco Fabbri et al	Whole genome analysis and microRNAs	Alternatives to Animal Experimentation	2012
Cadmium	ONECUT1	Marco Fabbri et al	Whole genome analysis and microRNAs	Alternatives to Animal Experimentation	2012
Cadmium	ZG16	Marco Fabbri et al	Whole genome analysis and microRNAs	Alternatives to Animal Experimentation	2012
Cadmium	HOXD1	Marco Fabbri et al	Whole genome analysis and microRNAs	Alternatives to Animal Experimentation	2012
Cadmium	SERPINA4	Marco Fabbri et al	Whole genome analysis and microRNAs	Alternatives to Animal Experimentation	2012
Cadmium	IFIT1	Marco Fabbri et al	Whole genome analysis and microRNAs	Alternatives to Animal Experimentation	2012
Cadmium	MOSC1	Marco Fabbri et al	Whole genome analysis and microRNAs	Alternatives to Animal Experimentation	2012
Cadmium	SLC47A1	Marco Fabbri et al	Whole genome analysis and microRNAs	Alternatives to Animal Experimentation	2012
Cadmium	LRRC47	Marco Fabbri et al	Whole genome analysis and microRNAs	Alternatives to Animal Experimentation	2012
Cadmium	SRCRB4D	Marco Fabbri et al	Whole genome analysis and microRNAs	Alternatives to Animal Experimentation	2012
Cadmium	METTL7A	Marco Fabbri et al	Whole genome analysis and microRNAs	Alternatives to Animal Experimentation	2012
Cadmium	CDO1	Marco Fabbri et al	Whole genome analysis and microRNAs	Alternatives to Animal Experimentation	2012
Cadmium	PEG10	Marco Fabbri et al	Whole genome analysis and microRNAs	Alternatives to Animal Experimentation	2012
Cadmium	A1CF	Marco Fabbri et al	Whole genome analysis and microRNAs	Alternatives to Animal Experimentation	2012
Cadmium	HADH	Marco Fabbri et al	Whole genome analysis and microRNAs	Alternatives to Animal Experimentation	2012
Cadmium	ACSF2	Marco Fabbri et al	Whole genome analysis and microRNAs	Alternatives to Animal Experimentation	2012
Cadmium	CFB	Marco Fabbri et al	Whole genome analysis and microRNAs	Alternatives to Animal Experimentation	2012
Cadmium	EPN3	Marco Fabbri et al	Whole genome analysis and microRNAs	Alternatives to Animal Experimentation	2012
Cadmium	SLC38A3	Marco Fabbri et al	Whole genome analysis and microRNAs	Alternatives to Animal Experimentation	2012
Cadmium	PNPLA3	Marco Fabbri et al	Whole genome analysis and microRNAs	Alternatives to Animal Experimentation	2012
Cadmium	SERPIND1	Marco Fabbri et al	Whole genome analysis and microRNAs	Alternatives to Animal Experimentation	2012
Cadmium	ERP27	Marco Fabbri et al	Whole genome analysis and microRNAs	Alternatives to Animal Experimentation	2012
Cadmium	STAR	Marco Fabbri et al	Whole genome analysis and microRNAs	Alternatives to Animal Experimentation	2012
Cadmium	GLUD1	Marco Fabbri et al	Whole genome analysis and microRNAs	Alternatives to Animal Experimentation	2012
Cadmium	IL22RA1	Marco Fabbri et al	Whole genome analysis and microRNAs	Alternatives to Animal Experimentation	2012
Cadmium	FGL1	Marco Fabbri et al	Whole genome analysis and microRNAs	Alternatives to Animal Experimentation	2012
Cadmium	C5	Marco Fabbri et al	Whole genome analysis and microRNAs	Alternatives to Animal Experimentation	2012
Cadmium	PGC	Marco Fabbri et al	Whole genome analysis and microRNAs	Alternatives to Animal Experimentation	2012
Cadmium	PPP1R3C	Marco Fabbri et al	Whole genome analysis and microRNAs	Alternatives to Animal Experimentation	2012
Cadmium	GPR44	Marco Fabbri et al	Whole genome analysis and microRNAs	Alternatives to Animal Experimentation	2012
Cadmium	MAP2K6	Marco Fabbri et al	Whole genome analysis and microRNAs	Alternatives to Animal Experimentation	2012
Cadmium	PIP5K1B	Marco Fabbri et al	Whole genome analysis and microRNAs	Alternatives to Animal Experimentation	2012
Cadmium	TMPRSS6	Marco Fabbri et al	Whole genome analysis and microRNAs	Alternatives to Animal Experimentation	2012
Cadmium	TRIM74	Marco Fabbri et al	Whole genome analysis and microRNAs	Alternatives to Animal Experimentation	2012
Cadmium	GLUD2	Marco Fabbri et al	Whole genome analysis and microRNAs	Alternatives to Animal Experimentation	2012

Cadmium	IDH1	Marco Fabbri et al	Whole genome analysis and microRNAs	Alternatives to Animal Experimentation	2012
Cadmium	SEPP1	Marco Fabbri et al	Whole genome analysis and microRNAs	Alternatives to Animal Experimentation	2012
Cadmium	TCEA3	Marco Fabbri et al	Whole genome analysis and microRNAs	Alternatives to Animal Experimentation	2012
Cadmium	ALDH3A2	Marco Fabbri et al	Whole genome analysis and microRNAs	Alternatives to Animal Experimentation	2012
Cadmium	HPN	Marco Fabbri et al	Whole genome analysis and microRNAs	Alternatives to Animal Experimentation	2012
Cadmium	C1ORF115	Marco Fabbri et al	Whole genome analysis and microRNAs	Alternatives to Animal Experimentation	2012
Cadmium	ASS1	Marco Fabbri et al	Whole genome analysis and microRNAs	Alternatives to Animal Experimentation	2012
Cadmium	CASP1	Marco Fabbri et al	Whole genome analysis and microRNAs	Alternatives to Animal Experimentation	2012
Cadmium	GDPD5	Marco Fabbri et al	Whole genome analysis and microRNAs	Alternatives to Animal Experimentation	2012
Cadmium	RAB11FIP4	Marco Fabbri et al	Whole genome analysis and microRNAs	Alternatives to Animal Experimentation	2012
Cadmium	ADH6	Marco Fabbri et al	Whole genome analysis and microRNAs	Alternatives to Animal Experimentation	2012
Cadmium	GSTA1	Marco Fabbri et al	Whole genome analysis and microRNAs	Alternatives to Animal Experimentation	2012
Cadmium	SLC6A14	Marco Fabbri et al	Whole genome analysis and microRNAs	Alternatives to Animal Experimentation	2012
Cadmium	FAM3B	Marco Fabbri et al	Whole genome analysis and microRNAs	Alternatives to Animal Experimentation	2012
Cadmium	GSTA3	Marco Fabbri et al	Whole genome analysis and microRNAs	Alternatives to Animal Experimentation	2012
Cadmium	RGN	Marco Fabbri et al	Whole genome analysis and microRNAs	Alternatives to Animal Experimentation	2012
Cadmium	BHMT	Marco Fabbri et al	Whole genome analysis and microRNAs	Alternatives to Animal Experimentation	2012
Cadmium	HPX	Marco Fabbri et al	Whole genome analysis and microRNAs	Alternatives to Animal Experimentation	2012
Cadmium	MRAP	Marco Fabbri et al	Whole genome analysis and microRNAs	Alternatives to Animal Experimentation	2012
Cadmium	SLCO4C1	Marco Fabbri et al	Whole genome analysis and microRNAs	Alternatives to Animal Experimentation	2012
Cadmium	TRIM50	Marco Fabbri et al	Whole genome analysis and microRNAs	Alternatives to Animal Experimentation	2012
Cadmium	TMEM86B	Marco Fabbri et al	Whole genome analysis and microRNAs	Alternatives to Animal Experimentation	2012
Cadmium	CEBPA	Marco Fabbri et al	Whole genome analysis and microRNAs	Alternatives to Animal Experimentation	2012
Cadmium	KNG1	Marco Fabbri et al	Whole genome analysis and microRNAs	Alternatives to Animal Experimentation	2012
Cadmium	LGALS2	Marco Fabbri et al	Whole genome analysis and microRNAs	Alternatives to Animal Experimentation	2012
Cadmium	MMP11	Marco Fabbri et al	Whole genome analysis and microRNAs	Alternatives to Animal Experimentation	2012
Cadmium	DUSP9	Marco Fabbri et al	Whole genome analysis and microRNAs	Alternatives to Animal Experimentation	2012
Cadmium	ALDH6A1	Marco Fabbri et al	Whole genome analysis and microRNAs	Alternatives to Animal Experimentation	2012
Cadmium	ANKRD43	Marco Fabbri et al	Whole genome analysis and microRNAs	Alternatives to Animal Experimentation	2012
Cadmium	PRODH2	Marco Fabbri et al	Whole genome analysis and microRNAs	Alternatives to Animal Experimentation	2012
Cadmium	C2ORF72	Marco Fabbri et al	Whole genome analysis and microRNAs	Alternatives to Animal Experimentation	2012
Cadmium	MLXIPL	Marco Fabbri et al	Whole genome analysis and microRNAs	Alternatives to Animal Experimentation	2012
Cadmium	ZCCHC2	Marco Fabbri et al	Whole genome analysis and microRNAs	Alternatives to Animal Experimentation	2012
Cadmium	GSTA5	Marco Fabbri et al	Whole genome analysis and microRNAs	Alternatives to Animal Experimentation	2012
Cadmium	SAMD11	Marco Fabbri et al	Whole genome analysis and microRNAs	Alternatives to Animal Experimentation	2012
Cadmium	FZD4	Marco Fabbri et al	Whole genome analysis and microRNAs	Alternatives to Animal Experimentation	2012
Cadmium	ENPP3	Marco Fabbri et al	Whole genome analysis and microRNAs	Alternatives to Animal Experimentation	2012
Cadmium	F7	Marco Fabbri et al	Whole genome analysis and microRNAs	Alternatives to Animal Experimentation	2012
Cadmium	EPHX2	Marco Fabbri et al	Whole genome analysis and microRNAs	Alternatives to Animal Experimentation	2012
Cadmium	ANG	Marco Fabbri et al	Whole genome analysis and microRNAs	Alternatives to Animal Experimentation	2012
Cadmium	LIME1	Marco Fabbri et al	Whole genome analysis and microRNAs	Alternatives to Animal Experimentation	2012
Cadmium	MIXL1	Marco Fabbri et al	Whole genome analysis and microRNAs	Alternatives to Animal Experimentation	2012
Cadmium	LOC149703	Marco Fabbri et al	Whole genome analysis and microRNAs	Alternatives to Animal Experimentation	2012
Cadmium	ENPP2	Marco Fabbri et al	Whole genome analysis and microRNAs	Alternatives to Animal Experimentation	2012
Cadmium	IGSF1	Marco Fabbri et al	Whole genome analysis and microRNAs	Alternatives to Animal Experimentation	2012
Cadmium	APOC3	Marco Fabbri et al	Whole genome analysis and microRNAs	Alternatives to Animal Experimentation	2012
Cadmium	SLC13A3	Marco Fabbri et al	Whole genome analysis and microRNAs	Alternatives to Animal Experimentation	2012
Cadmium	LOC100289632	Marco Fabbri et al	Whole genome analysis and microRNAs	Alternatives to Animal Experimentation	2012
Cadmium	GJB1	Marco Fabbri et al	Whole genome analysis and microRNAs	Alternatives to Animal Experimentation	2012
Cadmium	SERPINC1	Marco Fabbri et al	Whole genome analysis and microRNAs	Alternatives to Animal Experimentation	2012
Cadmium	SLC13A5	Marco Fabbri et al	Whole genome analysis and microRNAs	Alternatives to Animal Experimentation	2012
Cadmium	LOC55908	Marco Fabbri et al	Whole genome analysis and microRNAs	Alternatives to Animal Experimentation	2012
Cadmium	PCSK9	Marco Fabbri et al	Whole genome analysis and microRNAs	Alternatives to Animal Experimentation	2012
Cadmium	ACSM2B	Marco Fabbri et al	Whole genome analysis and microRNAs	Alternatives to Animal Experimentation	2012
Cadmium	C8A	Marco Fabbri et al	Whole genome analysis and microRNAs	Alternatives to Animal Experimentation	2012
Cadmium	SULT2A1	Marco Fabbri et al	Whole genome analysis and microRNAs	Alternatives to Animal Experimentation	2012
Cadmium	SLC5A9	Marco Fabbri et al	Whole genome analysis and microRNAs	Alternatives to Animal Experimentation	2012
Cadmium	VAV3	Marco Fabbri et al	Whole genome analysis and microRNAs	Alternatives to Animal Experimentation	2012
Cadmium	SPTLC3	Marco Fabbri et al	Whole genome analysis and microRNAs	Alternatives to Animal Experimentation	2012
Cadmium	GNMT	Marco Fabbri et al	Whole genome analysis and microRNAs	Alternatives to Animal Experimentation	2012
Cadmium	DDC	Marco Fabbri et al	Whole genome analysis and microRNAs	Alternatives to Animal Experimentation	2012
Cadmium	ITIH1	Marco Fabbri et al	Whole genome analysis and microRNAs	Alternatives to Animal Experimentation	2012
Cadmium	EPO	Marco Fabbri et al	Whole genome analysis and microRNAs	Alternatives to Animal Experimentation	2012
Cadmium	HAMP	Marco Fabbri et al	Whole genome analysis and microRNAs	Alternatives to Animal Experimentation	2012
Cadmium	DIO1	Marco Fabbri et al	Whole genome analysis and microRNAs	Alternatives to Animal Experimentation	2012
Cadmium	MTTP	Marco Fabbri et al	Whole genome analysis and microRNAs	Alternatives to Animal Experimentation	2012
Cadmium	PAH	Marco Fabbri et al	Whole genome analysis and microRNAs	Alternatives to Animal Experimentation	2012
Cadmium	GSTA2	Marco Fabbri et al	Whole genome analysis and microRNAs	Alternatives to Animal Experimentation	2012
Cadmium	PPP1R1A	Marco Fabbri et al	Whole genome analysis and microRNAs	Alternatives to Animal Experimentation	2012
Cadmium	APOA5	Marco Fabbri et al	Whole genome analysis and microRNAs	Alternatives to Animal Experimentation	2012
Cadmium	CDC42EP4	Marco Fabbri et al	Whole genome analysis and microRNAs	Alternatives to Animal Experimentation	2012
Cadmium	FGB	Marco Fabbri et al	Whole genome analysis and microRNAs	Alternatives to Animal Experimentation	2012
Cadmium	KLB	Marco Fabbri et al	Whole genome analysis and microRNAs	Alternatives to Animal Experimentation	2012
Cadmium	SERPINF2	Marco Fabbri et al	Whole genome analysis and microRNAs	Alternatives to Animal Experimentation	2012
Cadmium	SLC22A7	Marco Fabbri et al	Whole genome analysis and microRNAs	Alternatives to Animal Experimentation	2012
Cadmium	FGA	Marco Fabbri et al	Whole genome analysis and microRNAs	Alternatives to Animal Experimentation	2012
Cadmium	KANK4	Marco Fabbri et al	Whole genome analysis and microRNAs	Alternatives to Animal Experimentation	2012
Cadmium	SLC30A10	Marco Fabbri et al	Whole genome analysis and microRNAs	Alternatives to Animal Experimentation	2012
Cadmium	SERPINA6	Marco Fabbri et al	Whole genome analysis and microRNAs	Alternatives to Animal Experimentation	2012
Cadmium	FGG	Marco Fabbri et al	Whole genome analysis and microRNAs	Alternatives to Animal Experimentation	2012
Cadmium	LEAP2	Marco Fabbri et al	Whole genome analysis and microRNAs	Alternatives to Animal Experimentation	2012
Cadmium	HP	Marco Fabbri et al	Whole genome analysis and microRNAs	Alternatives to Animal Experimentation	2012
Cadmium	HPR	Marco Fabbri et al	Whole genome analysis and microRNAs	Alternatives to Animal Experimentation	2012
Cadmium	NR1H4	Marco Fabbri et al	Whole genome analysis and microRNAs	Alternatives to Animal Experimentation	2012
Cadmium	ALDH8A1	Marco Fabbri et al	Whole genome analysis and microRNAs	Alternatives to Animal Experimentation	2012
Lead	ZNF182	Tian Y, et al.	Correlations of gene expression with blood	Neurotox Res.	2011
Lead	RASEF	Tian Y, et al.	Correlations of gene expression with blood	Neurotox Res.	2011
Lead	CDKN2AIPNL	Tian Y, et al.	Correlations of gene expression with blood	Neurotox Res.	2011
Lead	NLK	Tian Y, et al.	Correlations of gene expression with blood	Neurotox Res.	2011

Lead	DNAJB9	Tian Y, et al.	Correlations of gene expression with blood	Neurotox Res.	2011
Lead	ZNF280D	Tian Y, et al.	Correlations of gene expression with blood	Neurotox Res.	2011
Lead	MGC34774	Tian Y, et al.	Correlations of gene expression with blood	Neurotox Res.	2011
Lead	TMEFF2	Tian Y, et al.	Correlations of gene expression with blood	Neurotox Res.	2011
Lead	FLJ40142	Tian Y, et al.	Correlations of gene expression with blood	Neurotox Res.	2011
Lead	SSPN	Tian Y, et al.	Correlations of gene expression with blood	Neurotox Res.	2011
Lead	RHEB	Tian Y, et al.	Correlations of gene expression with blood	Neurotox Res.	2011
Lead	WRB	Tian Y, et al.	Correlations of gene expression with blood	Neurotox Res.	2011
Lead	CEP350	Tian Y, et al.	Correlations of gene expression with blood	Neurotox Res.	2011
Lead	LOC100133109	Tian Y, et al.	Correlations of gene expression with blood	Neurotox Res.	2011
Lead	AKTIP	Tian Y, et al.	Correlations of gene expression with blood	Neurotox Res.	2011
Lead	ULK4	Tian Y, et al.	Correlations of gene expression with blood	Neurotox Res.	2011
Lead	UBIAD1	Tian Y, et al.	Correlations of gene expression with blood	Neurotox Res.	2011
Lead	PRMT6	Tian Y, et al.	Correlations of gene expression with blood	Neurotox Res.	2011
Lead	RHOXF1	Tian Y, et al.	Correlations of gene expression with blood	Neurotox Res.	2011
Lead	LOC730236	Tian Y, et al.	Correlations of gene expression with blood	Neurotox Res.	2011
Lead	GNB2L1	Tian Y, et al.	Correlations of gene expression with blood	Neurotox Res.	2011
Lead	POLR3B	Tian Y, et al.	Correlations of gene expression with blood	Neurotox Res.	2011
Lead	RAX2	Tian Y, et al.	Correlations of gene expression with blood	Neurotox Res.	2011
Lead	CEP27	Tian Y, et al.	Correlations of gene expression with blood	Neurotox Res.	2011
Lead	RPL35A	Tian Y, et al.	Correlations of gene expression with blood	Neurotox Res.	2011
Lead	MSL3	Tian Y, et al.	Correlations of gene expression with blood	Neurotox Res.	2011
Lead	TBRG1	Tian Y, et al.	Correlations of gene expression with blood	Neurotox Res.	2011
Lead	FDX1	Tian Y, et al.	Correlations of gene expression with blood	Neurotox Res.	2011
Lead	CD58	Tian Y, et al.	Correlations of gene expression with blood	Neurotox Res.	2011
Lead	TCTN2	Tian Y, et al.	Correlations of gene expression with blood	Neurotox Res.	2011
Lead	SMUG1	Tian Y, et al.	Correlations of gene expression with blood	Neurotox Res.	2011
Lead	FLJ10038	Tian Y, et al.	Correlations of gene expression with blood	Neurotox Res.	2011
Lead	SCML4	Tian Y, et al.	Correlations of gene expression with blood	Neurotox Res.	2011
Lead	CALD1	Tian Y, et al.	Correlations of gene expression with blood	Neurotox Res.	2011
Lead	YRDC	Tian Y, et al.	Correlations of gene expression with blood	Neurotox Res.	2011
Lead	ZNF397OS	Tian Y, et al.	Correlations of gene expression with blood	Neurotox Res.	2011
Lead	TIMM8B	Tian Y, et al.	Correlations of gene expression with blood	Neurotox Res.	2011
Lead	LMTK3	Tian Y, et al.	Correlations of gene expression with blood	Neurotox Res.	2011
Lead	ESRRA	Tian Y, et al.	Correlations of gene expression with blood	Neurotox Res.	2011
Lead	PPIL5	Tian Y, et al.	Correlations of gene expression with blood	Neurotox Res.	2011
Lead	SLC35D2	Tian Y, et al.	Correlations of gene expression with blood	Neurotox Res.	2011
Lead	F10	Tian Y, et al.	Correlations of gene expression with blood	Neurotox Res.	2011
Lead	LOC133874	Tian Y, et al.	Correlations of gene expression with blood	Neurotox Res.	2011
Lead	HR	Tian Y, et al.	Correlations of gene expression with blood	Neurotox Res.	2011
Lead	PACS2	Tian Y, et al.	Correlations of gene expression with blood	Neurotox Res.	2011
Lead	DPY19L4	Tian Y, et al.	Correlations of gene expression with blood	Neurotox Res.	2011
Lead	CMPK1	Tian Y, et al.	Correlations of gene expression with blood	Neurotox Res.	2011
Lead	SLC22A11	Tian Y, et al.	Correlations of gene expression with blood	Neurotox Res.	2011
Lead	LOC100134365	Tian Y, et al.	Correlations of gene expression with blood	Neurotox Res.	2011
Lead	SLC4A1AP	Tian Y, et al.	Correlations of gene expression with blood	Neurotox Res.	2011
Lead	LLGL1	Tian Y, et al.	Correlations of gene expression with blood	Neurotox Res.	2011
Lead	FASN	Tian Y, et al.	Correlations of gene expression with blood	Neurotox Res.	2011
Lead	C13orf28	Tian Y, et al.	Correlations of gene expression with blood	Neurotox Res.	2011
Lead	VAPB	Tian Y, et al.	Correlations of gene expression with blood	Neurotox Res.	2011
Lead	MAX	Tian Y, et al.	Correlations of gene expression with blood	Neurotox Res.	2011
Lead	USP32	Tian Y, et al.	Correlations of gene expression with blood	Neurotox Res.	2011
Lead	LOC728190	Tian Y, et al.	Correlations of gene expression with blood	Neurotox Res.	2011
Lead	SELT	Tian Y, et al.	Correlations of gene expression with blood	Neurotox Res.	2011
Lead	GDPD1	Tian Y, et al.	Correlations of gene expression with blood	Neurotox Res.	2011
Lead	AKT3	Tian Y, et al.	Correlations of gene expression with blood	Neurotox Res.	2011
Lead	SHC2	Tian Y, et al.	Correlations of gene expression with blood	Neurotox Res.	2011
Lead	PIGX	Tian Y, et al.	Correlations of gene expression with blood	Neurotox Res.	2011
Lead	CAND1	Tian Y, et al.	Correlations of gene expression with blood	Neurotox Res.	2011
Lead	ZNF576	Tian Y, et al.	Correlations of gene expression with blood	Neurotox Res.	2011
Lead	SOX13	Tian Y, et al.	Correlations of gene expression with blood	Neurotox Res.	2011
Lead	DLGAP4	Tian Y, et al.	Correlations of gene expression with blood	Neurotox Res.	2011
Lead	LOC285178	Tian Y, et al.	Correlations of gene expression with blood	Neurotox Res.	2011
Lead	FAM115A	Tian Y, et al.	Correlations of gene expression with blood	Neurotox Res.	2011
Lead	ADH4	Tian Y, et al.	Correlations of gene expression with blood	Neurotox Res.	2011
Lead	WDR61	Tian Y, et al.	Correlations of gene expression with blood	Neurotox Res.	2011
Lead	GYPB	Tian Y, et al.	Correlations of gene expression with blood	Neurotox Res.	2011
Lead	OTOS	Tian Y, et al.	Correlations of gene expression with blood	Neurotox Res.	2011
Lead	METTL5	Tian Y, et al.	Correlations of gene expression with blood	Neurotox Res.	2011
Lead	PDE6B	Tian Y, et al.	Correlations of gene expression with blood	Neurotox Res.	2011
Lead	LOC100133503 ///	Tian Y, et al.	Correlations of gene expression with blood	Neurotox Res.	2011
Lead	ALKBH7	Tian Y, et al.	Correlations of gene expression with blood	Neurotox Res.	2011
Lead	TMTC2	Tian Y, et al.	Correlations of gene expression with blood	Neurotox Res.	2011
Lead	MASP1	Tian Y, et al.	Correlations of gene expression with blood	Neurotox Res.	2011
Lead	CA5BL	Tian Y, et al.	Correlations of gene expression with blood	Neurotox Res.	2011
Lead	PDCL	Tian Y, et al.	Correlations of gene expression with blood	Neurotox Res.	2011
Lead	CKMT1A ///	Tian Y, et al.	Correlations of gene expression with blood	Neurotox Res.	2011
Lead	LAMC3	Tian Y, et al.	Correlations of gene expression with blood	Neurotox Res.	2011
Lead	WDR42A	Tian Y, et al.	Correlations of gene expression with blood	Neurotox Res.	2011
Lead	KCNK1	Tian Y, et al.	Correlations of gene expression with blood	Neurotox Res.	2011
Lead	TSHZ2	Tian Y, et al.	Correlations of gene expression with blood	Neurotox Res.	2011
Lead	NARG1	Tian Y, et al.	Correlations of gene expression with blood	Neurotox Res.	2011
Lead	GIT2	Tian Y, et al.	Correlations of gene expression with blood	Neurotox Res.	2011
Lead	STK36	Tian Y, et al.	Correlations of gene expression with blood	Neurotox Res.	2011
Lead	C4orf3	Tian Y, et al.	Correlations of gene expression with blood	Neurotox Res.	2011
Lead	C14orf167	Tian Y, et al.	Correlations of gene expression with blood	Neurotox Res.	2011
Lead	FN1	Tian Y, et al.	Correlations of gene expression with blood	Neurotox Res.	2011
Lead	CAPN14	Tian Y, et al.	Correlations of gene expression with blood	Neurotox Res.	2011
Lead	ENTPD1	Tian Y, et al.	Correlations of gene expression with blood	Neurotox Res.	2011

Lead	HAR1A	Tian Y, et al.	Correlations of gene expression with blood	Neurotox Res.	2011
Lead	ZNF786	Tian Y, et al.	Correlations of gene expression with blood	Neurotox Res.	2011
Lead	DIP2A	Tian Y, et al.	Correlations of gene expression with blood	Neurotox Res.	2011
Lead	POLR1B	Tian Y, et al.	Correlations of gene expression with blood	Neurotox Res.	2011
Lead	C20orf106 ///	Tian Y, et al.	Correlations of gene expression with blood	Neurotox Res.	2011
Lead	LOC284801	Tian Y, et al.	Correlations of gene expression with blood	Neurotox Res.	2011
Lead	C20orf107	Tian Y, et al.	Correlations of gene expression with blood	Neurotox Res.	2011
Lead	CYP3A4	Tian Y, et al.	Correlations of gene expression with blood	Neurotox Res.	2011
Lead	D2HGDH ///	Tian Y, et al.	Correlations of gene expression with blood	Neurotox Res.	2011
Lead	MTHFD1	Tian Y, et al.	Correlations of gene expression with blood	Neurotox Res.	2011
Lead	CRHR1	Tian Y, et al.	Correlations of gene expression with blood	Neurotox Res.	2011
Lead	SCARNA2	Tian Y, et al.	Correlations of gene expression with blood	Neurotox Res.	2011
Lead	CSAD	Tian Y, et al.	Correlations of gene expression with blood	Neurotox Res.	2011
Lead	GUCA1C	Tian Y, et al.	Correlations of gene expression with blood	Neurotox Res.	2011
Lead	DCHS1	Tian Y, et al.	Correlations of gene expression with blood	Neurotox Res.	2011
Lead	PXMP3	Tian Y, et al.	Correlations of gene expression with blood	Neurotox Res.	2011
Lead	PRMT2	Tian Y, et al.	Correlations of gene expression with blood	Neurotox Res.	2011
Lead	KCNJ6	Tian Y, et al.	Correlations of gene expression with blood	Neurotox Res.	2011
Lead	RPL31	Tian Y, et al.	Correlations of gene expression with blood	Neurotox Res.	2011
Lead	C13orf15	Tian Y, et al.	Correlations of gene expression with blood	Neurotox Res.	2011
Lead	WDR27	Tian Y, et al.	Correlations of gene expression with blood	Neurotox Res.	2011
Lead	CRB3	Tian Y, et al.	Correlations of gene expression with blood	Neurotox Res.	2011
Lead	C5	Tian Y, et al.	Correlations of gene expression with blood	Neurotox Res.	2011
Lead	TNXA /// TNXB	Tian Y, et al.	Correlations of gene expression with blood	Neurotox Res.	2011
Lead	PTPN7	Tian Y, et al.	Correlations of gene expression with blood	Neurotox Res.	2011
Lead	C4orf19	Tian Y, et al.	Correlations of gene expression with blood	Neurotox Res.	2011
Lead	FBN1	Tian Y, et al.	Correlations of gene expression with blood	Neurotox Res.	2011
Lead	LOC284067	Tian Y, et al.	Correlations of gene expression with blood	Neurotox Res.	2011
Lead	GOLT1B	Tian Y, et al.	Correlations of gene expression with blood	Neurotox Res.	2011
Lead	LOC100133696 ///	Tian Y, et al.	Correlations of gene expression with blood	Neurotox Res.	2011
Lead	PDE7B	Tian Y, et al.	Correlations of gene expression with blood	Neurotox Res.	2011
Lead	GRIK5	Tian Y, et al.	Correlations of gene expression with blood	Neurotox Res.	2011
Lead	PRSS3	Tian Y, et al.	Correlations of gene expression with blood	Neurotox Res.	2011
Lead	MAP6	Tian Y, et al.	Correlations of gene expression with blood	Neurotox Res.	2011
Lead	HTR6	Tian Y, et al.	Correlations of gene expression with blood	Neurotox Res.	2011
Lead	SETD5	Tian Y, et al.	Correlations of gene expression with blood	Neurotox Res.	2011
Lead	CROCCL2	Tian Y, et al.	Correlations of gene expression with blood	Neurotox Res.	2011
Lead	KRT6A /// KRT6B ///	Tian Y, et al.	Correlations of gene expression with blood	Neurotox Res.	2011
Lead	CD209	Tian Y, et al.	Correlations of gene expression with blood	Neurotox Res.	2011
Lead	GABRA1	Tian Y, et al.	Correlations of gene expression with blood	Neurotox Res.	2011
Lead	TSPAN2	Tian Y, et al.	Correlations of gene expression with blood	Neurotox Res.	2011
Lead	LRRC17	Tian Y, et al.	Correlations of gene expression with blood	Neurotox Res.	2011
Lead	ISLR	Tian Y, et al.	Correlations of gene expression with blood	Neurotox Res.	2011
Lead	CHRM3	Tian Y, et al.	Correlations of gene expression with blood	Neurotox Res.	2011
Lead	DAOA	Tian Y, et al.	Correlations of gene expression with blood	Neurotox Res.	2011
Lead	ABCC3	Tian Y, et al.	Correlations of gene expression with blood	Neurotox Res.	2011
Lead	CDGAP	Tian Y, et al.	Correlations of gene expression with blood	Neurotox Res.	2011
Lead	IFNA21	Tian Y, et al.	Correlations of gene expression with blood	Neurotox Res.	2011
Lead	UHRF1BP1L	Tian Y, et al.	Correlations of gene expression with blood	Neurotox Res.	2011
Lead	TRIM11	Tian Y, et al.	Correlations of gene expression with blood	Neurotox Res.	2011
Lead	C21orf77	Tian Y, et al.	Correlations of gene expression with blood	Neurotox Res.	2011
Lead	OR6A2	Tian Y, et al.	Correlations of gene expression with blood	Neurotox Res.	2011
Lead	C5orf44	Tian Y, et al.	Correlations of gene expression with blood	Neurotox Res.	2011
Lead	LOC92659	Tian Y, et al.	Correlations of gene expression with blood	Neurotox Res.	2011
Lead	APP	Tian Y, et al.	Correlations of gene expression with blood	Neurotox Res.	2011
Lead	PPM1H	Tian Y, et al.	Correlations of gene expression with blood	Neurotox Res.	2011
Lead	CCBP2	Tian Y, et al.	Correlations of gene expression with blood	Neurotox Res.	2011
Lead	DKFZp686O1327	Tian Y, et al.	Correlations of gene expression with blood	Neurotox Res.	2011
Lead	PNMAL1	Tian Y, et al.	Correlations of gene expression with blood	Neurotox Res.	2011
Lead	SATB2	Tian Y, et al.	Correlations of gene expression with blood	Neurotox Res.	2011
Lead	FAM82A1	Tian Y, et al.	Correlations of gene expression with blood	Neurotox Res.	2011
Lead	KIAA1648	Tian Y, et al.	Correlations of gene expression with blood	Neurotox Res.	2011
Lead	FAM128B	Tian Y, et al.	Correlations of gene expression with blood	Neurotox Res.	2011
Lead	ZNF233	Tian Y, et al.	Correlations of gene expression with blood	Neurotox Res.	2011
Lead	hCG_1730474	Tian Y, et al.	Correlations of gene expression with blood	Neurotox Res.	2011
Lead	FLJ21369	Tian Y, et al.	Correlations of gene expression with blood	Neurotox Res.	2011
Lead	ARHGAP22	Tian Y, et al.	Correlations of gene expression with blood	Neurotox Res.	2011
Lead	TAC4	Tian Y, et al.	Correlations of gene expression with blood	Neurotox Res.	2011
Lead	CLMN	Tian Y, et al.	Correlations of gene expression with blood	Neurotox Res.	2011
Lead	MAGIX	Tian Y, et al.	Correlations of gene expression with blood	Neurotox Res.	2011
Lead	PSG6	Tian Y, et al.	Correlations of gene expression with blood	Neurotox Res.	2011
Lead	WHSC1	Tian Y, et al.	Correlations of gene expression with blood	Neurotox Res.	2011
Lead	PKD2L2	Tian Y, et al.	Correlations of gene expression with blood	Neurotox Res.	2011
Lead	LOC51336	Tian Y, et al.	Correlations of gene expression with blood	Neurotox Res.	2011
Lead	RAPGEF1	Tian Y, et al.	Correlations of gene expression with blood	Neurotox Res.	2011
Lead	LONRF2	Tian Y, et al.	Correlations of gene expression with blood	Neurotox Res.	2011
Lead	ST6GALNAC1	Tian Y, et al.	Correlations of gene expression with blood	Neurotox Res.	2011
Lead	PRUNE	Tian Y, et al.	Correlations of gene expression with blood	Neurotox Res.	2011
Lead	PARP2	Tian Y, et al.	Correlations of gene expression with blood	Neurotox Res.	2011
Lead	ADD1	Tian Y, et al.	Correlations of gene expression with blood	Neurotox Res.	2011
Lead	CDCA7L	Tian Y, et al.	Correlations of gene expression with blood	Neurotox Res.	2011
Lead	TSR2	Tian Y, et al.	Correlations of gene expression with blood	Neurotox Res.	2011
Lead	ALDH9A1	Tian Y, et al.	Correlations of gene expression with blood	Neurotox Res.	2011
Lead	GATAD2B	Tian Y, et al.	Correlations of gene expression with blood	Neurotox Res.	2011
Lead	PRPF19	Tian Y, et al.	Correlations of gene expression with blood	Neurotox Res.	2011
Lead	LSM14A	Tian Y, et al.	Correlations of gene expression with blood	Neurotox Res.	2011
Lead	ANAPC5	Tian Y, et al.	Correlations of gene expression with blood	Neurotox Res.	2011
Lead	SLAIN2	Tian Y, et al.	Correlations of gene expression with blood	Neurotox Res.	2011
Lead	PHF20	Tian Y, et al.	Correlations of gene expression with blood	Neurotox Res.	2011

Lead	TAS2R41	Tian Y, et al.	Correlations of gene expression with blood	Neurotox Res.	2011
Lead	ZNF609	Tian Y, et al.	Correlations of gene expression with blood	Neurotox Res.	2011
Lead	ZNF214	Tian Y, et al.	Correlations of gene expression with blood	Neurotox Res.	2011
Lead	HYI	Tian Y, et al.	Correlations of gene expression with blood	Neurotox Res.	2011
Lead	DNALI1	Tian Y, et al.	Correlations of gene expression with blood	Neurotox Res.	2011
Lead	ZNF331	Tian Y, et al.	Correlations of gene expression with blood	Neurotox Res.	2011
Lead	PDE4DIP	Tian Y, et al.	Correlations of gene expression with blood	Neurotox Res.	2011
Lead	NHP2L1	Tian Y, et al.	Correlations of gene expression with blood	Neurotox Res.	2011
Lead	NCRNA00032	Tian Y, et al.	Correlations of gene expression with blood	Neurotox Res.	2011
Lead	CFL1	Tian Y, et al.	Correlations of gene expression with blood	Neurotox Res.	2011
Lead	CSNK2A2	Tian Y, et al.	Correlations of gene expression with blood	Neurotox Res.	2011
Lead	TAF1B	Tian Y, et al.	Correlations of gene expression with blood	Neurotox Res.	2011
Lead	BTRC	Tian Y, et al.	Correlations of gene expression with blood	Neurotox Res.	2011
Lead	SPG7	Tian Y, et al.	Correlations of gene expression with blood	Neurotox Res.	2011
Lead	RABGAP1	Tian Y, et al.	Correlations of gene expression with blood	Neurotox Res.	2011
Lead	NFRKB	Tian Y, et al.	Correlations of gene expression with blood	Neurotox Res.	2011
Lead	SERPINA10	Tian Y, et al.	Correlations of gene expression with blood	Neurotox Res.	2011
Lead	CXCR5	Tian Y, et al.	Correlations of gene expression with blood	Neurotox Res.	2011
Lead	BRI3BP	Tian Y, et al.	Correlations of gene expression with blood	Neurotox Res.	2011
Lead	RBMS1	Tian Y, et al.	Correlations of gene expression with blood	Neurotox Res.	2011
Lead	PPM1G	Tian Y, et al.	Correlations of gene expression with blood	Neurotox Res.	2011
Lead	SMC1B	Tian Y, et al.	Correlations of gene expression with blood	Neurotox Res.	2011
Lead	KIAA1429	Tian Y, et al.	Correlations of gene expression with blood	Neurotox Res.	2011
Lead	ALAD	Tian Y, et al.	Correlations of gene expression with blood	Neurotox Res.	2011
Lead	ELP3	Tian Y, et al.	Correlations of gene expression with blood	Neurotox Res.	2011
Lead	FXC1	Tian Y, et al.	Correlations of gene expression with blood	Neurotox Res.	2011
Lead	D4S234E /// GPS2	Tian Y, et al.	Correlations of gene expression with blood	Neurotox Res.	2011
Lead	PDDC1	Tian Y, et al.	Correlations of gene expression with blood	Neurotox Res.	2011
Lead	HNRNPA3 ///	Tian Y, et al.	Correlations of gene expression with blood	Neurotox Res.	2011
Lead	TDRD12	Tian Y, et al.	Correlations of gene expression with blood	Neurotox Res.	2011
Lead	MPEG1	Tian Y, et al.	Correlations of gene expression with blood	Neurotox Res.	2011
Lead	RPN2	Tian Y, et al.	Correlations of gene expression with blood	Neurotox Res.	2011
Lead	TSPAN18	Tian Y, et al.	Correlations of gene expression with blood	Neurotox Res.	2011
Lead	KHSRP	Tian Y, et al.	Correlations of gene expression with blood	Neurotox Res.	2011
Lead	CLOCK	Tian Y, et al.	Correlations of gene expression with blood	Neurotox Res.	2011
Lead	CTNS	Tian Y, et al.	Correlations of gene expression with blood	Neurotox Res.	2011
Lead	TMEM151B	Tian Y, et al.	Correlations of gene expression with blood	Neurotox Res.	2011
Lead	FTSJ2	Tian Y, et al.	Correlations of gene expression with blood	Neurotox Res.	2011
Lead	USP39	Tian Y, et al.	Correlations of gene expression with blood	Neurotox Res.	2011
Lead	UNQ9391	Tian Y, et al.	Correlations of gene expression with blood	Neurotox Res.	2011
Lead	XPNPEP3	Tian Y, et al.	Correlations of gene expression with blood	Neurotox Res.	2011
Lead	EIF4A1	Tian Y, et al.	Correlations of gene expression with blood	Neurotox Res.	2011
Lead	JMJD1C	Tian Y, et al.	Correlations of gene expression with blood	Neurotox Res.	2011
Lead	PRPF40A	Tian Y, et al.	Correlations of gene expression with blood	Neurotox Res.	2011
Lead	CASK	Tian Y, et al.	Correlations of gene expression with blood	Neurotox Res.	2011
Lead	SAMM50	Tian Y, et al.	Correlations of gene expression with blood	Neurotox Res.	2011
Lead	VPS45	Tian Y, et al.	Correlations of gene expression with blood	Neurotox Res.	2011
Lead	ANAPC2	Tian Y, et al.	Correlations of gene expression with blood	Neurotox Res.	2011
Lead	MAP4K1	Tian Y, et al.	Correlations of gene expression with blood	Neurotox Res.	2011
Lead	MICAL3	Tian Y, et al.	Correlations of gene expression with blood	Neurotox Res.	2011
Lead	SFRS15	Tian Y, et al.	Correlations of gene expression with blood	Neurotox Res.	2011
Lead	EML2	Tian Y, et al.	Correlations of gene expression with blood	Neurotox Res.	2011
Lead	IFT80	Tian Y, et al.	Correlations of gene expression with blood	Neurotox Res.	2011
Lead	PLXNB2	Tian Y, et al.	Correlations of gene expression with blood	Neurotox Res.	2011
Lead	CACNA2D1	Tian Y, et al.	Correlations of gene expression with blood	Neurotox Res.	2011
Lead	DNAL1	Tian Y, et al.	Correlations of gene expression with blood	Neurotox Res.	2011
Lead	COLEC12	Tian Y, et al.	Correlations of gene expression with blood	Neurotox Res.	2011
Lead	FAM159A	Tian Y, et al.	Correlations of gene expression with blood	Neurotox Res.	2011
Lead	FDFT1	Tian Y, et al.	Correlations of gene expression with blood	Neurotox Res.	2011
Lead	SET	Tian Y, et al.	Correlations of gene expression with blood	Neurotox Res.	2011
Lead	ADH1B	Tian Y, et al.	Correlations of gene expression with blood	Neurotox Res.	2011
Lead	NUCB1	Tian Y, et al.	Correlations of gene expression with blood	Neurotox Res.	2011
Lead	ALG8	Tian Y, et al.	Correlations of gene expression with blood	Neurotox Res.	2011
Lead	PREP	Tian Y, et al.	Correlations of gene expression with blood	Neurotox Res.	2011
Lead	RUFY1	Tian Y, et al.	Correlations of gene expression with blood	Neurotox Res.	2011
Lead	ATP5B	Tian Y, et al.	Correlations of gene expression with blood	Neurotox Res.	2011
Lead	GARNL3	Tian Y, et al.	Correlations of gene expression with blood	Neurotox Res.	2011
Lead	OBFC1	Tian Y, et al.	Correlations of gene expression with blood	Neurotox Res.	2011
Lead	FNDC7	Tian Y, et al.	Correlations of gene expression with blood	Neurotox Res.	2011
Lead	EXDL2	Tian Y, et al.	Correlations of gene expression with blood	Neurotox Res.	2011
Lead	PGLYRP3	Tian Y, et al.	Correlations of gene expression with blood	Neurotox Res.	2011
Lead	SF4	Tian Y, et al.	Correlations of gene expression with blood	Neurotox Res.	2011
Lead	AIFM1	Tian Y, et al.	Correlations of gene expression with blood	Neurotox Res.	2011
Lead	OGFOD1	Tian Y, et al.	Correlations of gene expression with blood	Neurotox Res.	2011
Lead	FOXRED2	Tian Y, et al.	Correlations of gene expression with blood	Neurotox Res.	2011
Lead	LOC100130987	Tian Y, et al.	Correlations of gene expression with blood	Neurotox Res.	2011
Lead	ARRDC1	Tian Y, et al.	Correlations of gene expression with blood	Neurotox Res.	2011
Lead	EIF3B	Tian Y, et al.	Correlations of gene expression with blood	Neurotox Res.	2011
Lead	ITGB3	Tian Y, et al.	Correlations of gene expression with blood	Neurotox Res.	2011
Lead	TP53	Tian Y, et al.	Correlations of gene expression with blood	Neurotox Res.	2011
Lead	DHPS	Tian Y, et al.	Correlations of gene expression with blood	Neurotox Res.	2011
Lead	CSNK2B /// LY6G5B	Tian Y, et al.	Correlations of gene expression with blood	Neurotox Res.	2011
Lead	LOC254028	Tian Y, et al.	Correlations of gene expression with blood	Neurotox Res.	2011
Lead	LAYN	Tian Y, et al.	Correlations of gene expression with blood	Neurotox Res.	2011
Lead	MYST2	Tian Y, et al.	Correlations of gene expression with blood	Neurotox Res.	2011
Lead	RDBP	Tian Y, et al.	Correlations of gene expression with blood	Neurotox Res.	2011
Lead	OR2L1P	Tian Y, et al.	Correlations of gene expression with blood	Neurotox Res.	2011
Lead	ADCK4	Tian Y, et al.	Correlations of gene expression with blood	Neurotox Res.	2011
Lead	HCLS1	Tian Y, et al.	Correlations of gene expression with blood	Neurotox Res.	2011

Lead	BTBD9	Tian Y, et al.	Correlations of gene expression with blood	Neurotox Res.	2011
Lead	TRIB3	Tian Y, et al.	Correlations of gene expression with blood	Neurotox Res.	2011
Lead	DDX24	Tian Y, et al.	Correlations of gene expression with blood	Neurotox Res.	2011
Lead	FAM21A /// FAM21B	Tian Y, et al.	Correlations of gene expression with blood	Neurotox Res.	2011
Lead	KIAA1211	Tian Y, et al.	Correlations of gene expression with blood	Neurotox Res.	2011
Lead	MYO1E	Tian Y, et al.	Correlations of gene expression with blood	Neurotox Res.	2011
Lead	TGFBI	Tian Y, et al.	Correlations of gene expression with blood	Neurotox Res.	2011
Lead	SHROOM1	Tian Y, et al.	Correlations of gene expression with blood	Neurotox Res.	2011
Lead	UBAP2	Tian Y, et al.	Correlations of gene expression with blood	Neurotox Res.	2011
Lead	PEF1	Tian Y, et al.	Correlations of gene expression with blood	Neurotox Res.	2011
Lead	SND1	Tian Y, et al.	Correlations of gene expression with blood	Neurotox Res.	2011
Lead	CTSH	Tian Y, et al.	Correlations of gene expression with blood	Neurotox Res.	2011
Lead	CAMSAP1	Tian Y, et al.	Correlations of gene expression with blood	Neurotox Res.	2011
Lead	EML3	Tian Y, et al.	Correlations of gene expression with blood	Neurotox Res.	2011
Lead	WHSC1L1	Tian Y, et al.	Correlations of gene expression with blood	Neurotox Res.	2011
Lead	RNF220	Tian Y, et al.	Correlations of gene expression with blood	Neurotox Res.	2011
Lead	EIF4A3	Tian Y, et al.	Correlations of gene expression with blood	Neurotox Res.	2011
Lead	LOC646482	Tian Y, et al.	Correlations of gene expression with blood	Neurotox Res.	2011
Lead	SNRPB	Tian Y, et al.	Correlations of gene expression with blood	Neurotox Res.	2011
Lead	IDH3B	Tian Y, et al.	Correlations of gene expression with blood	Neurotox Res.	2011
Lead	LOC728411	Tian Y, et al.	Correlations of gene expression with blood	Neurotox Res.	2011
Lead	MCM3	Tian Y, et al.	Correlations of gene expression with blood	Neurotox Res.	2011
Lead	UNKL	Tian Y, et al.	Correlations of gene expression with blood	Neurotox Res.	2011
Lead	GTF3C2	Tian Y, et al.	Correlations of gene expression with blood	Neurotox Res.	2011
Lead	CDC42	Tian Y, et al.	Correlations of gene expression with blood	Neurotox Res.	2011
Lead	HSD17B4	Tian Y, et al.	Correlations of gene expression with blood	Neurotox Res.	2011
Lead	DOM3Z	Tian Y, et al.	Correlations of gene expression with blood	Neurotox Res.	2011
Lead	RAB7A	Tian Y, et al.	Correlations of gene expression with blood	Neurotox Res.	2011
Lead	LOC100130648 ///	Tian Y, et al.	Correlations of gene expression with blood	Neurotox Res.	2011
Lead	ODF3L1	Tian Y, et al.	Correlations of gene expression with blood	Neurotox Res.	2011
Lead	SH3PXD2B	Tian Y, et al.	Correlations of gene expression with blood	Neurotox Res.	2011
Lead	MCF2L	Tian Y, et al.	Correlations of gene expression with blood	Neurotox Res.	2011
Lead	WFDC1	Tian Y, et al.	Correlations of gene expression with blood	Neurotox Res.	2011
Lead	GRK6	Tian Y, et al.	Correlations of gene expression with blood	Neurotox Res.	2011
Lead	AP2M1	Tian Y, et al.	Correlations of gene expression with blood	Neurotox Res.	2011
Lead	PPFIA1	Tian Y, et al.	Correlations of gene expression with blood	Neurotox Res.	2011
Lead	PSMD1	Tian Y, et al.	Correlations of gene expression with blood	Neurotox Res.	2011
Lead	TDP1	Tian Y, et al.	Correlations of gene expression with blood	Neurotox Res.	2011
Lead	AGBL4	Tian Y, et al.	Correlations of gene expression with blood	Neurotox Res.	2011
Lead	DLST /// DLSTP	Tian Y, et al.	Correlations of gene expression with blood	Neurotox Res.	2011
Lead	LOC142937	Tian Y, et al.	Correlations of gene expression with blood	Neurotox Res.	2011
Lead	COPA	Tian Y, et al.	Correlations of gene expression with blood	Neurotox Res.	2011
Lead	ANP32C	Tian Y, et al.	Correlations of gene expression with blood	Neurotox Res.	2011
Lead	FNDC1	Tian Y, et al.	Correlations of gene expression with blood	Neurotox Res.	2011
Lead	WDR4	Tian Y, et al.	Correlations of gene expression with blood	Neurotox Res.	2011
Lead	ACD	Tian Y, et al.	Correlations of gene expression with blood	Neurotox Res.	2011
Lead	C1orf162	Tian Y, et al.	Correlations of gene expression with blood	Neurotox Res.	2011
Lead	NAG	Tian Y, et al.	Correlations of gene expression with blood	Neurotox Res.	2011
Lead	SGCB	Tian Y, et al.	Correlations of gene expression with blood	Neurotox Res.	2011
Lead	RPH3AL	Tian Y, et al.	Correlations of gene expression with blood	Neurotox Res.	2011
Lead	RBM42	Tian Y, et al.	Correlations of gene expression with blood	Neurotox Res.	2011
Lead	APOL4	Tian Y, et al.	Correlations of gene expression with blood	Neurotox Res.	2011
Lead	LOC644165 ///	Tian Y, et al.	Correlations of gene expression with blood	Neurotox Res.	2011
Lead	KAL1	Tian Y, et al.	Correlations of gene expression with blood	Neurotox Res.	2011
Lead	GAL	Tian Y, et al.	Correlations of gene expression with blood	Neurotox Res.	2011
Lead	KCNH8	Tian Y, et al.	Correlations of gene expression with blood	Neurotox Res.	2011
Lead	RPL3L	Tian Y, et al.	Correlations of gene expression with blood	Neurotox Res.	2011
Lead	C18orf25	Tian Y, et al.	Correlations of gene expression with blood	Neurotox Res.	2011
Lead	SOBP	Tian Y, et al.	Correlations of gene expression with blood	Neurotox Res.	2011
Lead	POU6F1	Tian Y, et al.	Correlations of gene expression with blood	Neurotox Res.	2011
Lead	ADORA1	Tian Y, et al.	Correlations of gene expression with blood	Neurotox Res.	2011
Lead	RNF113A	Tian Y, et al.	Correlations of gene expression with blood	Neurotox Res.	2011
Lead	PHLPPL	Tian Y, et al.	Correlations of gene expression with blood	Neurotox Res.	2011
Lead	SDC4	Tian Y, et al.	Correlations of gene expression with blood	Neurotox Res.	2011
Lead	NOVA1	Tian Y, et al.	Correlations of gene expression with blood	Neurotox Res.	2011
Lead	PROSC	Tian Y, et al.	Correlations of gene expression with blood	Neurotox Res.	2011
Lead	COL4A2	Tian Y, et al.	Correlations of gene expression with blood	Neurotox Res.	2011
Lead	NFKBIB	Tian Y, et al.	Correlations of gene expression with blood	Neurotox Res.	2011
Lead	YWHAB	Tian Y, et al.	Correlations of gene expression with blood	Neurotox Res.	2011
Lead	SAPS1	Tian Y, et al.	Correlations of gene expression with blood	Neurotox Res.	2011
Lead	LZTS2	Tian Y, et al.	Correlations of gene expression with blood	Neurotox Res.	2011
Lead	SLC6A2	Tian Y, et al.	Correlations of gene expression with blood	Neurotox Res.	2011
Lead	XPO5	Tian Y, et al.	Correlations of gene expression with blood	Neurotox Res.	2011
Lead	IL1RAPL2	Tian Y, et al.	Correlations of gene expression with blood	Neurotox Res.	2011
Lead	FLJ44606	Tian Y, et al.	Correlations of gene expression with blood	Neurotox Res.	2011
Lead	ACADL	Tian Y, et al.	Correlations of gene expression with blood	Neurotox Res.	2011
Lead	LRRC41	Tian Y, et al.	Correlations of gene expression with blood	Neurotox Res.	2011
Lead	MTMR14	Tian Y, et al.	Correlations of gene expression with blood	Neurotox Res.	2011
Lead	RNF7	Tian Y, et al.	Correlations of gene expression with blood	Neurotox Res.	2011
Lead	ACRV1	Tian Y, et al.	Correlations of gene expression with blood	Neurotox Res.	2011
Lead	PSMC4	Tian Y, et al.	Correlations of gene expression with blood	Neurotox Res.	2011
Lead	LOC147343	Tian Y, et al.	Correlations of gene expression with blood	Neurotox Res.	2011
Lead	GALT	Tian Y, et al.	Correlations of gene expression with blood	Neurotox Res.	2011
Lead	C21orf119	Tian Y, et al.	Correlations of gene expression with blood	Neurotox Res.	2011
Lead	C1orf51	Tian Y, et al.	Correlations of gene expression with blood	Neurotox Res.	2011
Lead	CA12	Tian Y, et al.	Correlations of gene expression with blood	Neurotox Res.	2011
Lead	TM4SF5	Tian Y, et al.	Correlations of gene expression with blood	Neurotox Res.	2011
Lead	IQCG	Tian Y, et al.	Correlations of gene expression with blood	Neurotox Res.	2011
Lead	FLJ14327	Tian Y, et al.	Correlations of gene expression with blood	Neurotox Res.	2011

Lead	HADHB	Tian Y, et al.	Correlations of gene expression with blood	Neurotox Res.	2011
Lead	MAP1A	Tian Y, et al.	Correlations of gene expression with blood	Neurotox Res.	2011
Lead	PABPN1	Tian Y, et al.	Correlations of gene expression with blood	Neurotox Res.	2011
Lead	AKT2	Tian Y, et al.	Correlations of gene expression with blood	Neurotox Res.	2011
Lead	PARS2	Tian Y, et al.	Correlations of gene expression with blood	Neurotox Res.	2011
Lead	CYBASC3	Tian Y, et al.	Correlations of gene expression with blood	Neurotox Res.	2011
Lead	SOX10	Tian Y, et al.	Correlations of gene expression with blood	Neurotox Res.	2011
Lead	MTDH	Tian Y, et al.	Correlations of gene expression with blood	Neurotox Res.	2011
Lead	ERCC2	Tian Y, et al.	Correlations of gene expression with blood	Neurotox Res.	2011
Lead	ACSS1	Tian Y, et al.	Correlations of gene expression with blood	Neurotox Res.	2011
Lead	CXorf30	Tian Y, et al.	Correlations of gene expression with blood	Neurotox Res.	2011
Lead	KIAA1328	Tian Y, et al.	Correlations of gene expression with blood	Neurotox Res.	2011
Lead	PLXND1	Tian Y, et al.	Correlations of gene expression with blood	Neurotox Res.	2011
Lead	LAS1L	Tian Y, et al.	Correlations of gene expression with blood	Neurotox Res.	2011
Lead	ANKRD16	Tian Y, et al.	Correlations of gene expression with blood	Neurotox Res.	2011
Lead	LOC157562	Tian Y, et al.	Correlations of gene expression with blood	Neurotox Res.	2011
Lead	UQCRC1	Tian Y, et al.	Correlations of gene expression with blood	Neurotox Res.	2011
Lead	OCRL	Tian Y, et al.	Correlations of gene expression with blood	Neurotox Res.	2011
Lead	EVPL	Tian Y, et al.	Correlations of gene expression with blood	Neurotox Res.	2011
Lead	NAT10	Tian Y, et al.	Correlations of gene expression with blood	Neurotox Res.	2011
Lead	CES2	Tian Y, et al.	Correlations of gene expression with blood	Neurotox Res.	2011
Lead	CIDEB	Tian Y, et al.	Correlations of gene expression with blood	Neurotox Res.	2011
Lead	VPS35	Tian Y, et al.	Correlations of gene expression with blood	Neurotox Res.	2011
Lead	ZNF793	Tian Y, et al.	Correlations of gene expression with blood	Neurotox Res.	2011
Lead	ZBTB46	Tian Y, et al.	Correlations of gene expression with blood	Neurotox Res.	2011
Lead	ZAK	Tian Y, et al.	Correlations of gene expression with blood	Neurotox Res.	2011
Lead	C7orf20	Tian Y, et al.	Correlations of gene expression with blood	Neurotox Res.	2011
Lead	PDK1	Tian Y, et al.	Correlations of gene expression with blood	Neurotox Res.	2011
Lead	VPS11	Tian Y, et al.	Correlations of gene expression with blood	Neurotox Res.	2011
Lead	C10orf122	Tian Y, et al.	Correlations of gene expression with blood	Neurotox Res.	2011
Lead	TNFSF13	Tian Y, et al.	Correlations of gene expression with blood	Neurotox Res.	2011
Lead	TRAF3IP2	Tian Y, et al.	Correlations of gene expression with blood	Neurotox Res.	2011
Lead	MCCC2	Tian Y, et al.	Correlations of gene expression with blood	Neurotox Res.	2011
Lead	HOXD9	Tian Y, et al.	Correlations of gene expression with blood	Neurotox Res.	2011
Lead	SH3KBP1	Tian Y, et al.	Correlations of gene expression with blood	Neurotox Res.	2011
Lead	TINF2	Tian Y, et al.	Correlations of gene expression with blood	Neurotox Res.	2011
Lead	CEP68	Tian Y, et al.	Correlations of gene expression with blood	Neurotox Res.	2011
Lead	SDF4	Tian Y, et al.	Correlations of gene expression with blood	Neurotox Res.	2011
Lead	GABRR2	Tian Y, et al.	Correlations of gene expression with blood	Neurotox Res.	2011
Lead	PADI4	Tian Y, et al.	Correlations of gene expression with blood	Neurotox Res.	2011
Lead	XRCC1	Tian Y, et al.	Correlations of gene expression with blood	Neurotox Res.	2011
Lead	FAM21C /// FAM21D	Tian Y, et al.	Correlations of gene expression with blood	Neurotox Res.	2011
Lead	REG3A /// REG3G	Tian Y, et al.	Correlations of gene expression with blood	Neurotox Res.	2011
Lead	C1orf2	Tian Y, et al.	Correlations of gene expression with blood	Neurotox Res.	2011
Lead	HDAC3	Tian Y, et al.	Correlations of gene expression with blood	Neurotox Res.	2011
Lead	EGFL7	Tian Y, et al.	Correlations of gene expression with blood	Neurotox Res.	2011
Lead	TMED6	Tian Y, et al.	Correlations of gene expression with blood	Neurotox Res.	2011
Lead	HEBP1	Tian Y, et al.	Correlations of gene expression with blood	Neurotox Res.	2011
Lead	METT10D	Tian Y, et al.	Correlations of gene expression with blood	Neurotox Res.	2011
Lead	MICAL2	Tian Y, et al.	Correlations of gene expression with blood	Neurotox Res.	2011
Lead	TNR	Tian Y, et al.	Correlations of gene expression with blood	Neurotox Res.	2011
Lead	RANGAP1	Tian Y, et al.	Correlations of gene expression with blood	Neurotox Res.	2011
Lead	GALNT6	Tian Y, et al.	Correlations of gene expression with blood	Neurotox Res.	2011
Lead	ABHD12	Tian Y, et al.	Correlations of gene expression with blood	Neurotox Res.	2011
Lead	ICA1L	Tian Y, et al.	Correlations of gene expression with blood	Neurotox Res.	2011
Lead	KRTAP4-3	Tian Y, et al.	Correlations of gene expression with blood	Neurotox Res.	2011
Lead	CUEDC1	Tian Y, et al.	Correlations of gene expression with blood	Neurotox Res.	2011
Lead	TRIO	Tian Y, et al.	Correlations of gene expression with blood	Neurotox Res.	2011
Lead	FAM181B	Tian Y, et al.	Correlations of gene expression with blood	Neurotox Res.	2011
Lead	ATP8B1	Tian Y, et al.	Correlations of gene expression with blood	Neurotox Res.	2011
Lead	XDH	Tian Y, et al.	Correlations of gene expression with blood	Neurotox Res.	2011
Lead	GSTT1	Tian Y, et al.	Correlations of gene expression with blood	Neurotox Res.	2011
Lead	ALDH1B1	Tian Y, et al.	Correlations of gene expression with blood	Neurotox Res.	2011
Lead	SEMA6A	Tian Y, et al.	Correlations of gene expression with blood	Neurotox Res.	2011
Lead	ACSBG1	Tian Y, et al.	Correlations of gene expression with blood	Neurotox Res.	2011
Lead	PAX8	Tian Y, et al.	Correlations of gene expression with blood	Neurotox Res.	2011
Lead	CD2BP2	Tian Y, et al.	Correlations of gene expression with blood	Neurotox Res.	2011
Lead	LOC285431	Tian Y, et al.	Correlations of gene expression with blood	Neurotox Res.	2011
Lead	HLA-DOA	Tian Y, et al.	Correlations of gene expression with blood	Neurotox Res.	2011
Lead	USP40	Tian Y, et al.	Correlations of gene expression with blood	Neurotox Res.	2011
Lead	KARS	Tian Y, et al.	Correlations of gene expression with blood	Neurotox Res.	2011
Lead	PSMB2	Tian Y, et al.	Correlations of gene expression with blood	Neurotox Res.	2011
Lead	ASB2	Tian Y, et al.	Correlations of gene expression with blood	Neurotox Res.	2011
Lead	PDLIM7	Tian Y, et al.	Correlations of gene expression with blood	Neurotox Res.	2011
Lead	DDX54	Tian Y, et al.	Correlations of gene expression with blood	Neurotox Res.	2011
Lead	C19orf63	Tian Y, et al.	Correlations of gene expression with blood	Neurotox Res.	2011
Lead	LOC100130476	Tian Y, et al.	Correlations of gene expression with blood	Neurotox Res.	2011
Lead	WDR38	Tian Y, et al.	Correlations of gene expression with blood	Neurotox Res.	2011
Lead	C7orf10	Tian Y, et al.	Correlations of gene expression with blood	Neurotox Res.	2011
Lead	C3orf37	Tian Y, et al.	Correlations of gene expression with blood	Neurotox Res.	2011
Lead	LOC401022	Tian Y, et al.	Correlations of gene expression with blood	Neurotox Res.	2011
Lead	PSMD2	Tian Y, et al.	Correlations of gene expression with blood	Neurotox Res.	2011
Lead	ECHS1	Tian Y, et al.	Correlations of gene expression with blood	Neurotox Res.	2011
Lead	RALY	Tian Y, et al.	Correlations of gene expression with blood	Neurotox Res.	2011
Lead	EEF2K	Tian Y, et al.	Correlations of gene expression with blood	Neurotox Res.	2011
Lead	C16orf53	Tian Y, et al.	Correlations of gene expression with blood	Neurotox Res.	2011
Lead	CLDN20	Tian Y, et al.	Correlations of gene expression with blood	Neurotox Res.	2011
Lead	LOC100144602	Tian Y, et al.	Correlations of gene expression with blood	Neurotox Res.	2011
Lead	AGAP5 /// AGAP9 ///	Tian Y, et al.	Correlations of gene expression with blood	Neurotox Res.	2011

Lead	INO80	Tian Y, et al.	Correlations of gene expression with blood	Neurotox Res.	2011
Lead	GPR173	Tian Y, et al.	Correlations of gene expression with blood	Neurotox Res.	2011
Lead	GALR3	Tian Y, et al.	Correlations of gene expression with blood	Neurotox Res.	2011
Lead	FCER2	Tian Y, et al.	Correlations of gene expression with blood	Neurotox Res.	2011
Lead	GPKOW	Tian Y, et al.	Correlations of gene expression with blood	Neurotox Res.	2011
Lead	NLRP14	Tian Y, et al.	Correlations of gene expression with blood	Neurotox Res.	2011
Lead	C11orf49	Tian Y, et al.	Correlations of gene expression with blood	Neurotox Res.	2011
Lead	LOC730092	Tian Y, et al.	Correlations of gene expression with blood	Neurotox Res.	2011
Lead	ANXA2	Tian Y, et al.	Correlations of gene expression with blood	Neurotox Res.	2011
Lead	TXNL4A	Tian Y, et al.	Correlations of gene expression with blood	Neurotox Res.	2011
Lead	SEC14L4	Tian Y, et al.	Correlations of gene expression with blood	Neurotox Res.	2011
Lead	TBXA2R	Tian Y, et al.	Correlations of gene expression with blood	Neurotox Res.	2011
Lead	RNH1	Tian Y, et al.	Correlations of gene expression with blood	Neurotox Res.	2011
Lead	RASL11A	Tian Y, et al.	Correlations of gene expression with blood	Neurotox Res.	2011
Lead	POGK	Tian Y, et al.	Correlations of gene expression with blood	Neurotox Res.	2011
Lead	GCS1	Tian Y, et al.	Correlations of gene expression with blood	Neurotox Res.	2011
Lead	CLTA	Tian Y, et al.	Correlations of gene expression with blood	Neurotox Res.	2011
Lead	UBE2L3	Tian Y, et al.	Correlations of gene expression with blood	Neurotox Res.	2011
Lead	HLA-DMA	Tian Y, et al.	Correlations of gene expression with blood	Neurotox Res.	2011
Lead	KCNK5	Tian Y, et al.	Correlations of gene expression with blood	Neurotox Res.	2011
Lead	LEPREL1	Tian Y, et al.	Correlations of gene expression with blood	Neurotox Res.	2011
Lead	FH	Tian Y, et al.	Correlations of gene expression with blood	Neurotox Res.	2011
Lead	CYSLTR2	Tian Y, et al.	Correlations of gene expression with blood	Neurotox Res.	2011
Lead	LPAR1	Tian Y, et al.	Correlations of gene expression with blood	Neurotox Res.	2011
Lead	LPXN	Tian Y, et al.	Correlations of gene expression with blood	Neurotox Res.	2011
Lead	MYT1	Tian Y, et al.	Correlations of gene expression with blood	Neurotox Res.	2011
Lead	MTHFSD	Tian Y, et al.	Correlations of gene expression with blood	Neurotox Res.	2011
Lead	IGHD	Tian Y, et al.	Correlations of gene expression with blood	Neurotox Res.	2011
Lead	C1orf20	Tian Y, et al.	Correlations of gene expression with blood	Neurotox Res.	2011
Lead	PFN4	Tian Y, et al.	Correlations of gene expression with blood	Neurotox Res.	2011
Lead	SERPINF2	Tian Y, et al.	Correlations of gene expression with blood	Neurotox Res.	2011
Lead	SYNE1	Tian Y, et al.	Correlations of gene expression with blood	Neurotox Res.	2011
Lead	TERF2	Tian Y, et al.	Correlations of gene expression with blood	Neurotox Res.	2011
Lead	MCOLN3	Tian Y, et al.	Correlations of gene expression with blood	Neurotox Res.	2011
Lead	CSPP1	Tian Y, et al.	Correlations of gene expression with blood	Neurotox Res.	2011
Lead	SLC48A1	Tian Y, et al.	Correlations of gene expression with blood	Neurotox Res.	2011
Lead	GEM	Tian Y, et al.	Correlations of gene expression with blood	Neurotox Res.	2011
Lead	CCDC61	Tian Y, et al.	Correlations of gene expression with blood	Neurotox Res.	2011
Lead	KLK13	Tian Y, et al.	Correlations of gene expression with blood	Neurotox Res.	2011
Lead	MAP6D1	Tian Y, et al.	Correlations of gene expression with blood	Neurotox Res.	2011
Lead	LMAN1L	Tian Y, et al.	Correlations of gene expression with blood	Neurotox Res.	2011
Lead	RDH13	Tian Y, et al.	Correlations of gene expression with blood	Neurotox Res.	2011
Lead	SLC38A10	Tian Y, et al.	Correlations of gene expression with blood	Neurotox Res.	2011
Lead	CYP2C9	Tian Y, et al.	Correlations of gene expression with blood	Neurotox Res.	2011
Lead	DNAJA3	Tian Y, et al.	Correlations of gene expression with blood	Neurotox Res.	2011
Lead	PGAM1	Tian Y, et al.	Correlations of gene expression with blood	Neurotox Res.	2011
Lead	KLKB1	Tian Y, et al.	Correlations of gene expression with blood	Neurotox Res.	2011
Lead	C22orf28	Tian Y, et al.	Correlations of gene expression with blood	Neurotox Res.	2011
Lead	APLP2	Tian Y, et al.	Correlations of gene expression with blood	Neurotox Res.	2011
Lead	DCDC2	Tian Y, et al.	Correlations of gene expression with blood	Neurotox Res.	2011
Lead	HRK	Tian Y, et al.	Correlations of gene expression with blood	Neurotox Res.	2011
Lead	CBLN3	Tian Y, et al.	Correlations of gene expression with blood	Neurotox Res.	2011
Lead	DCTN2	Tian Y, et al.	Correlations of gene expression with blood	Neurotox Res.	2011
Lead	POU3F1	Tian Y, et al.	Correlations of gene expression with blood	Neurotox Res.	2011
Lead	SLC27A1	Tian Y, et al.	Correlations of gene expression with blood	Neurotox Res.	2011
Lead	C1orf134	Tian Y, et al.	Correlations of gene expression with blood	Neurotox Res.	2011
Lead	ARHGDIA	Tian Y, et al.	Correlations of gene expression with blood	Neurotox Res.	2011
Lead	FRZB	Tian Y, et al.	Correlations of gene expression with blood	Neurotox Res.	2011
Lead	USP31	Tian Y, et al.	Correlations of gene expression with blood	Neurotox Res.	2011
Lead	HLA-DRB6	Tian Y, et al.	Correlations of gene expression with blood	Neurotox Res.	2011
Lead	ASNA1	Tian Y, et al.	Correlations of gene expression with blood	Neurotox Res.	2011
Lead	TCTN1	Tian Y, et al.	Correlations of gene expression with blood	Neurotox Res.	2011
Lead	MSL1	Tian Y, et al.	Correlations of gene expression with blood	Neurotox Res.	2011
Lead	ZNF777	Tian Y, et al.	Correlations of gene expression with blood	Neurotox Res.	2011
Lead	HDAC10	Tian Y, et al.	Correlations of gene expression with blood	Neurotox Res.	2011
Lead	HLA-DMB	Tian Y, et al.	Correlations of gene expression with blood	Neurotox Res.	2011
Lead	CDC20B	Tian Y, et al.	Correlations of gene expression with blood	Neurotox Res.	2011
Lead	MAFG	Tian Y, et al.	Correlations of gene expression with blood	Neurotox Res.	2011
Lead	ABCF1	Tian Y, et al.	Correlations of gene expression with blood	Neurotox Res.	2011
Lead	DDX25	Tian Y, et al.	Correlations of gene expression with blood	Neurotox Res.	2011
Lead	NKIRAS2	Tian Y, et al.	Correlations of gene expression with blood	Neurotox Res.	2011
Lead	KIAA1659	Tian Y, et al.	Correlations of gene expression with blood	Neurotox Res.	2011
Lead	HMGCL	Tian Y, et al.	Correlations of gene expression with blood	Neurotox Res.	2011
Lead	BAT3	Tian Y, et al.	Correlations of gene expression with blood	Neurotox Res.	2011
Lead	NDUFV3	Tian Y, et al.	Correlations of gene expression with blood	Neurotox Res.	2011
Lead	CEACAM5	Tian Y, et al.	Correlations of gene expression with blood	Neurotox Res.	2011
Lead	BOP1	Tian Y, et al.	Correlations of gene expression with blood	Neurotox Res.	2011
Lead	EGR4	Tian Y, et al.	Correlations of gene expression with blood	Neurotox Res.	2011
Lead	MCM4	Tian Y, et al.	Correlations of gene expression with blood	Neurotox Res.	2011
Lead	ARF3	Tian Y, et al.	Correlations of gene expression with blood	Neurotox Res.	2011
Lead	WNK2	Tian Y, et al.	Correlations of gene expression with blood	Neurotox Res.	2011
Lead	LARP5	Tian Y, et al.	Correlations of gene expression with blood	Neurotox Res.	2011
Lead	CLN8	Tian Y, et al.	Correlations of gene expression with blood	Neurotox Res.	2011
Lead	HHLA3	Tian Y, et al.	Correlations of gene expression with blood	Neurotox Res.	2011
Lead	ANXA2P2	Tian Y, et al.	Correlations of gene expression with blood	Neurotox Res.	2011
Lead	ABL2	Tian Y, et al.	Correlations of gene expression with blood	Neurotox Res.	2011
Lead	INTS9	Tian Y, et al.	Correlations of gene expression with blood	Neurotox Res.	2011
Lead	PCIF1	Tian Y, et al.	Correlations of gene expression with blood	Neurotox Res.	2011
Lead	STON2	Tian Y, et al.	Correlations of gene expression with blood	Neurotox Res.	2011

Lead	RUVBL2	Tian Y, et al.	Correlations of gene expression with blood	Neurotox Res.	2011
Lead	SAP18	Tian Y, et al.	Correlations of gene expression with blood	Neurotox Res.	2011
Lead	CAP1	Tian Y, et al.	Correlations of gene expression with blood	Neurotox Res.	2011
Lead	FAF1	Tian Y, et al.	Correlations of gene expression with blood	Neurotox Res.	2011
Lead	ZNF687	Tian Y, et al.	Correlations of gene expression with blood	Neurotox Res.	2011
Lead	NR0B1	Tian Y, et al.	Correlations of gene expression with blood	Neurotox Res.	2011
Lead	PPP2R1A	Tian Y, et al.	Correlations of gene expression with blood	Neurotox Res.	2011
Lead	PPP1R11	Tian Y, et al.	Correlations of gene expression with blood	Neurotox Res.	2011
Lead	HEMK1	Tian Y, et al.	Correlations of gene expression with blood	Neurotox Res.	2011
Lead	SECISBP2	Tian Y, et al.	Correlations of gene expression with blood	Neurotox Res.	2011
Lead	CENPB	Tian Y, et al.	Correlations of gene expression with blood	Neurotox Res.	2011
Lead	SF3B3	Tian Y, et al.	Correlations of gene expression with blood	Neurotox Res.	2011
Lead	WBSCR16	Tian Y, et al.	Correlations of gene expression with blood	Neurotox Res.	2011
Lead	NOP2	Tian Y, et al.	Correlations of gene expression with blood	Neurotox Res.	2011
Lead	CRTC2	Tian Y, et al.	Correlations of gene expression with blood	Neurotox Res.	2011
Lead	ZNF275	Tian Y, et al.	Correlations of gene expression with blood	Neurotox Res.	2011
Lead	TTC7A	Tian Y, et al.	Correlations of gene expression with blood	Neurotox Res.	2011
Lead	ORAI2	Tian Y, et al.	Correlations of gene expression with blood	Neurotox Res.	2011
Lead	AFTPH	Tian Y, et al.	Correlations of gene expression with blood	Neurotox Res.	2011
Lead	FKBP4	Tian Y, et al.	Correlations of gene expression with blood	Neurotox Res.	2011
Lead	FOXP3	Tian Y, et al.	Correlations of gene expression with blood	Neurotox Res.	2011
Lead	PFKM	Tian Y, et al.	Correlations of gene expression with blood	Neurotox Res.	2011
Lead	GPC1	Tian Y, et al.	Correlations of gene expression with blood	Neurotox Res.	2011
Lead	ARAF	Tian Y, et al.	Correlations of gene expression with blood	Neurotox Res.	2011
Lead	GTF2F1	Tian Y, et al.	Correlations of gene expression with blood	Neurotox Res.	2011
Lead	TCF15	Tian Y, et al.	Correlations of gene expression with blood	Neurotox Res.	2011
Lead	DCP1B	Tian Y, et al.	Correlations of gene expression with blood	Neurotox Res.	2011
Lead	PTK2B	Tian Y, et al.	Correlations of gene expression with blood	Neurotox Res.	2011
Lead	EIF2B4	Tian Y, et al.	Correlations of gene expression with blood	Neurotox Res.	2011
Lead	DNPEP	Tian Y, et al.	Correlations of gene expression with blood	Neurotox Res.	2011
Lead	GPS1	Tian Y, et al.	Correlations of gene expression with blood	Neurotox Res.	2011
Lead	ASPSCR1	Tian Y, et al.	Correlations of gene expression with blood	Neurotox Res.	2011
Lead	COG2	Tian Y, et al.	Correlations of gene expression with blood	Neurotox Res.	2011
Lead	DET1	Tian Y, et al.	Correlations of gene expression with blood	Neurotox Res.	2011
Lead	ARS2	Tian Y, et al.	Correlations of gene expression with blood	Neurotox Res.	2011
Lead	FAM82A2	Tian Y, et al.	Correlations of gene expression with blood	Neurotox Res.	2011
Lead	EIF2B1	Tian Y, et al.	Correlations of gene expression with blood	Neurotox Res.	2011
Lead	FARSB	Tian Y, et al.	Correlations of gene expression with blood	Neurotox Res.	2011
Lead	C1orf107	Tian Y, et al.	Correlations of gene expression with blood	Neurotox Res.	2011
Lead	NADSYN1	Tian Y, et al.	Correlations of gene expression with blood	Neurotox Res.	2011
Lead	LRSAM1	Tian Y, et al.	Correlations of gene expression with blood	Neurotox Res.	2011
Lead	COX10	Tian Y, et al.	Correlations of gene expression with blood	Neurotox Res.	2011
Lead	PPFIBP2	Tian Y, et al.	Correlations of gene expression with blood	Neurotox Res.	2011
Lead	EFNA3	Tian Y, et al.	Correlations of gene expression with blood	Neurotox Res.	2011
Lead	PLEKHB1	Tian Y, et al.	Correlations of gene expression with blood	Neurotox Res.	2011
Lead	DTX4	Tian Y, et al.	Correlations of gene expression with blood	Neurotox Res.	2011
Lead	ZNF350	Tian Y, et al.	Correlations of gene expression with blood	Neurotox Res.	2011
Lead	ALDH3A2	Tian Y, et al.	Correlations of gene expression with blood	Neurotox Res.	2011
Lead	GRHPR	Tian Y, et al.	Correlations of gene expression with blood	Neurotox Res.	2011
Lead	CDC37	Tian Y, et al.	Correlations of gene expression with blood	Neurotox Res.	2011
Lead	SMARCC2	Tian Y, et al.	Correlations of gene expression with blood	Neurotox Res.	2011
Lead	FOXRED1	Tian Y, et al.	Correlations of gene expression with blood	Neurotox Res.	2011
Lead	YIPF6	Tian Y, et al.	Correlations of gene expression with blood	Neurotox Res.	2011
Lead	GAR1	Tian Y, et al.	Correlations of gene expression with blood	Neurotox Res.	2011
Lead	TP53I11	Tian Y, et al.	Correlations of gene expression with blood	Neurotox Res.	2011
Lead	DGCR14	Tian Y, et al.	Correlations of gene expression with blood	Neurotox Res.	2011
Lead	SNF8	Tian Y, et al.	Correlations of gene expression with blood	Neurotox Res.	2011
Lead	RIC8A	Tian Y, et al.	Correlations of gene expression with blood	Neurotox Res.	2011
Volatile Organic	SSU72	Jin Hee KIM et al	Changes in oxidative stress biomarker and	Industrial Health	2011
Volatile Organic	KIN	Jin Hee KIM et al	Changes in oxidative stress biomarker and	Industrial Health	2011
Volatile Organic	PAMCI	Jin Hee KIM et al	Changes in oxidative stress biomarker and	Industrial Health	2011
Volatile Organic	KIAA1713	Jin Hee KIM et al	Changes in oxidative stress biomarker and	Industrial Health	2011
Volatile Organic	UBE2L3	Jin Hee KIM et al	Changes in oxidative stress biomarker and	Industrial Health	2011
Volatile Organic	LOC440602	Jin Hee KIM et al	Changes in oxidative stress biomarker and	Industrial Health	2011
Volatile Organic	BRP44L	Jin Hee KIM et al	Changes in oxidative stress biomarker and	Industrial Health	2011
Volatile Organic	ENO3	Jin Hee KIM et al	Changes in oxidative stress biomarker and	Industrial Health	2011
Volatile Organic	THOP1	Jin Hee KIM et al	Changes in oxidative stress biomarker and	Industrial Health	2011
Volatile Organic	SKP1A	Jin Hee KIM et al	Changes in oxidative stress biomarker and	Industrial Health	2011
Volatile Organic	NBL1	Jin Hee KIM et al	Changes in oxidative stress biomarker and	Industrial Health	2011
Volatile Organic	CPSF1	Jin Hee KIM et al	Changes in oxidative stress biomarker and	Industrial Health	2011
Volatile Organic	MGC61571	Jin Hee KIM et al	Changes in oxidative stress biomarker and	Industrial Health	2011
Volatile Organic	KLF16	Jin Hee KIM et al	Changes in oxidative stress biomarker and	Industrial Health	2011
Volatile Organic	ZNF136	Jin Hee KIM et al	Changes in oxidative stress biomarker and	Industrial Health	2011
Volatile Organic	DAZ4	Jin Hee KIM et al	Changes in oxidative stress biomarker and	Industrial Health	2011
Volatile Organic	SEC24C	Jin Hee KIM et al	Changes in oxidative stress biomarker and	Industrial Health	2011
Volatile Organic	ZNF524	Jin Hee KIM et al	Changes in oxidative stress biomarker and	Industrial Health	2011
Volatile Organic	LOC389077	Jin Hee KIM et al	Changes in oxidative stress biomarker and	Industrial Health	2011
Volatile Organic	C19orf28	Jin Hee KIM et al	Changes in oxidative stress biomarker and	Industrial Health	2011
Volatile Organic	LOC441444	Jin Hee KIM et al	Changes in oxidative stress biomarker and	Industrial Health	2011
Volatile Organic	HSPA1B	CLIONA M. MCHALE et al	Changes in the Peripheral Blood	Genomics	2009
Volatile Organic	IFI44L	CLIONA M. MCHALE et al	Changes in the Peripheral Blood	Genomics	2009
Volatile Organic	HSPA1A	CLIONA M. MCHALE et al	Changes in the Peripheral Blood	Genomics	2009
Volatile Organic	JUN	CLIONA M. MCHALE et al	Changes in the Peripheral Blood	Genomics	2009
Volatile Organic	IFNG	CLIONA M. MCHALE et al	Changes in the Peripheral Blood	Genomics	2009
Volatile Organic	PMAIP1	CLIONA M. MCHALE et al	Changes in the Peripheral Blood	Genomics	2009
Volatile Organic	PDZK1IP1	CLIONA M. MCHALE et al	Changes in the Peripheral Blood	Genomics	2009
Volatile Organic	BCL2	CLIONA M. MCHALE et al	Changes in the Peripheral Blood	Genomics	2009
Volatile Organic	G1P2	CLIONA M. MCHALE et al	Changes in the Peripheral Blood	Genomics	2009
Volatile Organic	KLF6	CLIONA M. MCHALE et al	Changes in the Peripheral Blood	Genomics	2009

Volatile Organic	MX1	CLIONA M. MCHALE et al	Changes in the Peripheral Blood	Genomics	2009
Volatile Organic	PTGS1	CLIONA M. MCHALE et al	Changes in the Peripheral Blood	Genomics	2009
Volatile Organic	FLJ39779	CLIONA M. MCHALE et al	Changes in the Peripheral Blood	Genomics	2009
Volatile Organic	TAGAP	CLIONA M. MCHALE et al	Changes in the Peripheral Blood	Genomics	2009
Volatile Organic	STK17B	CLIONA M. MCHALE et al	Changes in the Peripheral Blood	Genomics	2009
Volatile Organic	G1P3	CLIONA M. MCHALE et al	Changes in the Peripheral Blood	Genomics	2009
Volatile Organic	FOSB	CLIONA M. MCHALE et al	Changes in the Peripheral Blood	Genomics	2009
Volatile Organic	PPP1R15A	CLIONA M. MCHALE et al	Changes in the Peripheral Blood	Genomics	2009
Volatile Organic	CD69	CLIONA M. MCHALE et al	Changes in the Peripheral Blood	Genomics	2009
Volatile Organic	HIST1H2BB	CLIONA M. MCHALE et al	Changes in the Peripheral Blood	Genomics	2009
Volatile Organic	HIST2H2BE	CLIONA M. MCHALE et al	Changes in the Peripheral Blood	Genomics	2009
Volatile Organic	SH3BGRL2	CLIONA M. MCHALE et al	Changes in the Peripheral Blood	Genomics	2009
Volatile Organic	HIST1H2AC	CLIONA M. MCHALE et al	Changes in the Peripheral Blood	Genomics	2009
Volatile Organic	RGS1	CLIONA M. MCHALE et al	Changes in the Peripheral Blood	Genomics	2009
Volatile Organic	F13A1	CLIONA M. MCHALE et al	Changes in the Peripheral Blood	Genomics	2009
Volatile Organic	LTBP1	CLIONA M. MCHALE et al	Changes in the Peripheral Blood	Genomics	2009
Volatile Organic	PCSK5	CLIONA M. MCHALE et al	Changes in the Peripheral Blood	Genomics	2009
Volatile Organic	HIST2H2AA	CLIONA M. MCHALE et al	Changes in the Peripheral Blood	Genomics	2009
Volatile Organic	MEIS1	CLIONA M. MCHALE et al	Changes in the Peripheral Blood	Genomics	2009
Volatile Organic	INPP4B	CLIONA M. MCHALE et al	Changes in the Peripheral Blood	Genomics	2009
Volatile Organic	GNG11	CLIONA M. MCHALE et al	Changes in the Peripheral Blood	Genomics	2009
Volatile Organic	PF4	CLIONA M. MCHALE et al	Changes in the Peripheral Blood	Genomics	2009
Volatile Organic	MGC16169	CLIONA M. MCHALE et al	Changes in the Peripheral Blood	Genomics	2009
Volatile Organic	PTGS1	CLIONA M. MCHALE et al	Changes in the Peripheral Blood	Genomics	2009
Volatile Organic	GNAZ	CLIONA M. MCHALE et al	Changes in the Peripheral Blood	Genomics	2009
Volatile Organic	CTDSPL	CLIONA M. MCHALE et al	Changes in the Peripheral Blood	Genomics	2009
Volatile Organic	HIST2H2AA	CLIONA M. MCHALE et al	Changes in the Peripheral Blood	Genomics	2009
Volatile Organic	HIST1H2BK	CLIONA M. MCHALE et al	Changes in the Peripheral Blood	Genomics	2009
Volatile Organic	SSBP2	CLIONA M. MCHALE et al	Changes in the Peripheral Blood	Genomics	2009
Volatile Organic	C9orf91	CLIONA M. MCHALE et al	Changes in the Peripheral Blood	Genomics	2009
Volatile Organic	FHL1	CLIONA M. MCHALE et al	Changes in the Peripheral Blood	Genomics	2009
Volatile Organic	RSU1	CLIONA M. MCHALE et al	Changes in the Peripheral Blood	Genomics	2009
Volatile Organic	ITGB5	CLIONA M. MCHALE et al	Changes in the Peripheral Blood	Genomics	2009
Volatile Organic	ALOX12	CLIONA M. MCHALE et al	Changes in the Peripheral Blood	Genomics	2009
Volatile Organic	ITGA2B	CLIONA M. MCHALE et al	Changes in the Peripheral Blood	Genomics	2009
Volatile Organic	ACSBG1	CLIONA M. MCHALE et al	Changes in the Peripheral Blood	Genomics	2009
Volatile Organic	RUFY1	CLIONA M. MCHALE et al	Changes in the Peripheral Blood	Genomics	2009
Volatile Organic	MPL	CLIONA M. MCHALE et al	Changes in the Peripheral Blood	Genomics	2009
Volatile Organic	ZFP36	CLIONA M. MCHALE et al	Changes in the Peripheral Blood	Genomics	2009
Volatile Organic	WASF3	CLIONA M. MCHALE et al	Changes in the Peripheral Blood	Genomics	2009
Volatile Organic	FCHSD2	CLIONA M. MCHALE et al	Changes in the Peripheral Blood	Genomics	2009
Volatile Organic	CLU	CLIONA M. MCHALE et al	Changes in the Peripheral Blood	Genomics	2009
Volatile Organic	FARS2	CLIONA M. MCHALE et al	Changes in the Peripheral Blood	Genomics	2009
Volatile Organic	GP9	CLIONA M. MCHALE et al	Changes in the Peripheral Blood	Genomics	2009
Volatile Organic	SPARC	CLIONA M. MCHALE et al	Changes in the Peripheral Blood	Genomics	2009
Volatile Organic	HGD	CLIONA M. MCHALE et al	Changes in the Peripheral Blood	Genomics	2009
Volatile Organic	KLF2	CLIONA M. MCHALE et al	Changes in the Peripheral Blood	Genomics	2009
Volatile Organic	DUSP1	CLIONA M. MCHALE et al	Changes in the Peripheral Blood	Genomics	2009
Volatile Organic	ZDHHC14	CLIONA M. MCHALE et al	Changes in the Peripheral Blood	Genomics	2009
Volatile Organic	FCRLM1	CLIONA M. MCHALE et al	Changes in the Peripheral Blood	Genomics	2009
Volatile Organic	FDFT1	CLIONA M. MCHALE et al	Changes in the Peripheral Blood	Genomics	2009
Volatile Organic	TSPAN33	CLIONA M. MCHALE et al	Changes in the Peripheral Blood	Genomics	2009
Volatile Organic	MGC13057	CLIONA M. MCHALE et al	Changes in the Peripheral Blood	Genomics	2009
Volatile Organic	DNAJB1	CLIONA M. MCHALE et al	Changes in the Peripheral Blood	Genomics	2009
Volatile Organic	RSU1	CLIONA M. MCHALE et al	Changes in the Peripheral Blood	Genomics	2009
Volatile Organic	GP1BA	CLIONA M. MCHALE et al	Changes in the Peripheral Blood	Genomics	2009
Volatile Organic	RNF144	CLIONA M. MCHALE et al	Changes in the Peripheral Blood	Genomics	2009
Volatile Organic	TRIP11	CLIONA M. MCHALE et al	Changes in the Peripheral Blood	Genomics	2009
Volatile Organic	SNTB1	CLIONA M. MCHALE et al	Changes in the Peripheral Blood	Genomics	2009
Volatile Organic	SPOCD1	CLIONA M. MCHALE et al	Changes in the Peripheral Blood	Genomics	2009
Volatile Organic	RP5-1077B9.4	CLIONA M. MCHALE et al	Changes in the Peripheral Blood	Genomics	2009
Volatile Organic	HIST1H3A	CLIONA M. MCHALE et al	Changes in the Peripheral Blood	Genomics	2009
Volatile Organic	RPS10	CLIONA M. MCHALE et al	Changes in the Peripheral Blood	Genomics	2009
Volatile Organic	DGCR6L	CLIONA M. MCHALE et al	Changes in the Peripheral Blood	Genomics	2009
Volatile Organic	RPL35	CLIONA M. MCHALE et al	Changes in the Peripheral Blood	Genomics	2009
Volatile Organic	HIST1H3D	CLIONA M. MCHALE et al	Changes in the Peripheral Blood	Genomics	2009
Volatile Organic	CTTN	CLIONA M. MCHALE et al	Changes in the Peripheral Blood	Genomics	2009
Volatile Organic	RPS21	CLIONA M. MCHALE et al	Changes in the Peripheral Blood	Genomics	2009
Volatile Organic	TNFAIP3	CLIONA M. MCHALE et al	Changes in the Peripheral Blood	Genomics	2009
Volatile Organic	STRBP	CLIONA M. MCHALE et al	Changes in the Peripheral Blood	Genomics	2009
Volatile Organic	LARGE	CLIONA M. MCHALE et al	Changes in the Peripheral Blood	Genomics	2009
Volatile Organic	CSNK1E	CLIONA M. MCHALE et al	Changes in the Peripheral Blood	Genomics	2009
Volatile Organic	RPL30	CLIONA M. MCHALE et al	Changes in the Peripheral Blood	Genomics	2009
Volatile Organic	HBG1	CLIONA M. MCHALE et al	Changes in the Peripheral Blood	Genomics	2009
Volatile Organic	HMOX1	CLIONA M. MCHALE et al	Changes in the Peripheral Blood	Genomics	2009
Volatile Organic	PAPSS2	CLIONA M. MCHALE et al	Changes in the Peripheral Blood	Genomics	2009
Volatile Organic	HAS1	CLIONA M. MCHALE et al	Changes in the Peripheral Blood	Genomics	2009
Volatile Organic	TIMP1	CLIONA M. MCHALE et al	Changes in the Peripheral Blood	Genomics	2009
Volatile Organic	IL1R2	CLIONA M. MCHALE et al	Changes in the Peripheral Blood	Genomics	2009
Volatile Organic	CXCL16	CLIONA M. MCHALE et al	Changes in the Peripheral Blood	Genomics	2009
Volatile Organic	KIAA1189	CLIONA M. MCHALE et al	Changes in the Peripheral Blood	Genomics	2009
Volatile Organic	EGR3	CLIONA M. MCHALE et al	Changes in the Peripheral Blood	Genomics	2009
Volatile Organic	CLEC5A	CLIONA M. MCHALE et al	Changes in the Peripheral Blood	Genomics	2009
Volatile Organic	EGR2	CLIONA M. MCHALE et al	Changes in the Peripheral Blood	Genomics	2009
Volatile Organic	PLK2	CLIONA M. MCHALE et al	Changes in the Peripheral Blood	Genomics	2009
Volatile Organic	ZNF331	CLIONA M. MCHALE et al	Changes in the Peripheral Blood	Genomics	2009
Volatile Organic	TLR2	CLIONA M. MCHALE et al	Changes in the Peripheral Blood	Genomics	2009
Volatile Organic	MCEMP1	CLIONA M. MCHALE et al	Changes in the Peripheral Blood	Genomics	2009
Volatile Organic	PLA2G7	CLIONA M. MCHALE et al	Changes in the Peripheral Blood	Genomics	2009

Volatile Organic	AUTS2	CLIONA M. MCHALE et al	Changes in the Peripheral Blood	Genomics	2009
Volatile Organic	NRIP3	CLIONA M. MCHALE et al	Changes in the Peripheral Blood	Genomics	2009
Volatile Organic	ZFYVE16	CLIONA M. MCHALE et al	Changes in the Peripheral Blood	Genomics	2009
Volatile Organic	ANPEP	CLIONA M. MCHALE et al	Changes in the Peripheral Blood	Genomics	2009
Volatile Organic	STAT4	CLIONA M. MCHALE et al	Changes in the Peripheral Blood	Genomics	2009
Volatile Organic	SLC16A3	CLIONA M. MCHALE et al	Changes in the Peripheral Blood	Genomics	2009
Volatile Organic	C6orf62	CLIONA M. MCHALE et al	Changes in the Peripheral Blood	Genomics	2009
Volatile Organic	JOSD1	CLIONA M. MCHALE et al	Changes in the Peripheral Blood	Genomics	2009
Volatile Organic	ATP2B1	CLIONA M. MCHALE et al	Changes in the Peripheral Blood	Genomics	2009
Volatile Organic	VMO1	CLIONA M. MCHALE et al	Changes in the Peripheral Blood	Genomics	2009
Volatile Organic	NR4A2	CLIONA M. MCHALE et al	Changes in the Peripheral Blood	Genomics	2009
Volatile Organic	C7orf16	CLIONA M. MCHALE et al	Changes in the Peripheral Blood	Genomics	2009
Volatile Organic	PPARG	CLIONA M. MCHALE et al	Changes in the Peripheral Blood	Genomics	2009
Volatile Organic	NPC1	CLIONA M. MCHALE et al	Changes in the Peripheral Blood	Genomics	2009
Volatile Organic	ATP6V1B2	CLIONA M. MCHALE et al	Changes in the Peripheral Blood	Genomics	2009
Volatile Organic	TNFSF14	CLIONA M. MCHALE et al	Changes in the Peripheral Blood	Genomics	2009
Volatile Organic	MGC14376	CLIONA M. MCHALE et al	Changes in the Peripheral Blood	Genomics	2009
Volatile Organic	DUSP4	CLIONA M. MCHALE et al	Changes in the Peripheral Blood	Genomics	2009
Volatile Organic	MAEA	CLIONA M. MCHALE et al	Changes in the Peripheral Blood	Genomics	2009
Volatile Organic	MITF	CLIONA M. MCHALE et al	Changes in the Peripheral Blood	Genomics	2009
Volatile Organic	PHF13	CLIONA M. MCHALE et al	Changes in the Peripheral Blood	Genomics	2009
Volatile Organic	MAT2A	CLIONA M. MCHALE et al	Changes in the Peripheral Blood	Genomics	2009
Volatile Organic	FLJ20273	CLIONA M. MCHALE et al	Changes in the Peripheral Blood	Genomics	2009
Volatile Organic	KLF13	CLIONA M. MCHALE et al	Changes in the Peripheral Blood	Genomics	2009
Volatile Organic	ENC1	CLIONA M. MCHALE et al	Changes in the Peripheral Blood	Genomics	2009
Volatile Organic	FAM20C	CLIONA M. MCHALE et al	Changes in the Peripheral Blood	Genomics	2009
Volatile Organic	RASSF2	CLIONA M. MCHALE et al	Changes in the Peripheral Blood	Genomics	2009
Volatile Organic	ETF1	CLIONA M. MCHALE et al	Changes in the Peripheral Blood	Genomics	2009
Volatile Organic	AVPI1	CLIONA M. MCHALE et al	Changes in the Peripheral Blood	Genomics	2009
Volatile Organic	NAB2	CLIONA M. MCHALE et al	Changes in the Peripheral Blood	Genomics	2009
Volatile Organic	CHST7	CLIONA M. MCHALE et al	Changes in the Peripheral Blood	Genomics	2009
Volatile Organic	TBC1D7	CLIONA M. MCHALE et al	Changes in the Peripheral Blood	Genomics	2009
Volatile Organic	UAP1	CLIONA M. MCHALE et al	Changes in the Peripheral Blood	Genomics	2009
Volatile Organic	ZCCHC14	CLIONA M. MCHALE et al	Changes in the Peripheral Blood	Genomics	2009
Volatile Organic	PTPNS1	CLIONA M. MCHALE et al	Changes in the Peripheral Blood	Genomics	2009
Volatile Organic	SRXN1	CLIONA M. MCHALE et al	Changes in the Peripheral Blood	Genomics	2009
Volatile Organic	SPAG9	CLIONA M. MCHALE et al	Changes in the Peripheral Blood	Genomics	2009
Volatile Organic	CTSB	CLIONA M. MCHALE et al	Changes in the Peripheral Blood	Genomics	2009
Volatile Organic	SLC39A8	CLIONA M. MCHALE et al	Changes in the Peripheral Blood	Genomics	2009
Volatile Organic	MFI2	CLIONA M. MCHALE et al	Changes in the Peripheral Blood	Genomics	2009
Volatile Organic	IL21R	CLIONA M. MCHALE et al	Changes in the Peripheral Blood	Genomics	2009
Volatile Organic	LHFPL2	CLIONA M. MCHALE et al	Changes in the Peripheral Blood	Genomics	2009
Volatile Organic	CLTC	CLIONA M. MCHALE et al	Changes in the Peripheral Blood	Genomics	2009
Volatile Organic	FOSL2	CLIONA M. MCHALE et al	Changes in the Peripheral Blood	Genomics	2009
Volatile Organic	SMAD3	CLIONA M. MCHALE et al	Changes in the Peripheral Blood	Genomics	2009
Volatile Organic	P2RY8	CLIONA M. MCHALE et al	Changes in the Peripheral Blood	Genomics	2009
Volatile Organic	ADNP	CLIONA M. MCHALE et al	Changes in the Peripheral Blood	Genomics	2009
Volatile Organic	ZC3HAV1	CLIONA M. MCHALE et al	Changes in the Peripheral Blood	Genomics	2009
Volatile Organic	MMP9	CLIONA M. MCHALE et al	Changes in the Peripheral Blood	Genomics	2009
Volatile Organic	P2RX7	CLIONA M. MCHALE et al	Changes in the Peripheral Blood	Genomics	2009
Volatile Organic	PPP1R16B	CLIONA M. MCHALE et al	Changes in the Peripheral Blood	Genomics	2009
Volatile Organic	CTLA4	CLIONA M. MCHALE et al	Changes in the Peripheral Blood	Genomics	2009
Volatile Organic	CERK	CLIONA M. MCHALE et al	Changes in the Peripheral Blood	Genomics	2009
Volatile Organic	TMEPAI	CLIONA M. MCHALE et al	Changes in the Peripheral Blood	Genomics	2009
Volatile Organic	PSMD11	CLIONA M. MCHALE et al	Changes in the Peripheral Blood	Genomics	2009
Volatile Organic	AGPAT4	CLIONA M. MCHALE et al	Changes in the Peripheral Blood	Genomics	2009
Volatile Organic	CCR5	CLIONA M. MCHALE et al	Changes in the Peripheral Blood	Genomics	2009
Volatile Organic	MECP2	CLIONA M. MCHALE et al	Changes in the Peripheral Blood	Genomics	2009
Volatile Organic	NR4A3	CLIONA M. MCHALE et al	Changes in the Peripheral Blood	Genomics	2009
Volatile Organic	PSMD12	CLIONA M. MCHALE et al	Changes in the Peripheral Blood	Genomics	2009
Volatile Organic	FLJ10803	CLIONA M. MCHALE et al	Changes in the Peripheral Blood	Genomics	2009
Volatile Organic	SLC7A5	CLIONA M. MCHALE et al	Changes in the Peripheral Blood	Genomics	2009
Volatile Organic	NSMAF	CLIONA M. MCHALE et al	Changes in the Peripheral Blood	Genomics	2009
Volatile Organic	PPAP2B	CLIONA M. MCHALE et al	Changes in the Peripheral Blood	Genomics	2009
Volatile Organic	SLC38A2	CLIONA M. MCHALE et al	Changes in the Peripheral Blood	Genomics	2009
Volatile Organic	EML4	CLIONA M. MCHALE et al	Changes in the Peripheral Blood	Genomics	2009
Volatile Organic	C14orf130	CLIONA M. MCHALE et al	Changes in the Peripheral Blood	Genomics	2009
Volatile Organic	RAB7B	CLIONA M. MCHALE et al	Changes in the Peripheral Blood	Genomics	2009
Volatile Organic	C3orf40	CLIONA M. MCHALE et al	Changes in the Peripheral Blood	Genomics	2009
Volatile Organic	ARPC1A	CLIONA M. MCHALE et al	Changes in the Peripheral Blood	Genomics	2009
Volatile Organic	EED	CLIONA M. MCHALE et al	Changes in the Peripheral Blood	Genomics	2009
Volatile Organic	KIAA0232	CLIONA M. MCHALE et al	Changes in the Peripheral Blood	Genomics	2009
Volatile Organic	AIM1	CLIONA M. MCHALE et al	Changes in the Peripheral Blood	Genomics	2009
Volatile Organic	PAFAH1B1	CLIONA M. MCHALE et al	Changes in the Peripheral Blood	Genomics	2009
Volatile Organic	ZNF302	CLIONA M. MCHALE et al	Changes in the Peripheral Blood	Genomics	2009
Volatile Organic	SURF4	CLIONA M. MCHALE et al	Changes in the Peripheral Blood	Genomics	2009
Volatile Organic	PPP1CB	CLIONA M. MCHALE et al	Changes in the Peripheral Blood	Genomics	2009
Volatile Organic	RELB	CLIONA M. MCHALE et al	Changes in the Peripheral Blood	Genomics	2009
Volatile Organic	KIAA0323	CLIONA M. MCHALE et al	Changes in the Peripheral Blood	Genomics	2009
Volatile Organic	CBLB	CLIONA M. MCHALE et al	Changes in the Peripheral Blood	Genomics	2009
Volatile Organic	NAB1	CLIONA M. MCHALE et al	Changes in the Peripheral Blood	Genomics	2009
Volatile Organic	DIRC2	CLIONA M. MCHALE et al	Changes in the Peripheral Blood	Genomics	2009
Volatile Organic	USP36	CLIONA M. MCHALE et al	Changes in the Peripheral Blood	Genomics	2009
Volatile Organic	CAMKK2	CLIONA M. MCHALE et al	Changes in the Peripheral Blood	Genomics	2009
Volatile Organic	SLC25A3	CLIONA M. MCHALE et al	Changes in the Peripheral Blood	Genomics	2009
Volatile Organic	SLTM	CLIONA M. MCHALE et al	Changes in the Peripheral Blood	Genomics	2009
Volatile Organic	MFSD5	CLIONA M. MCHALE et al	Changes in the Peripheral Blood	Genomics	2009
Volatile Organic	MSL2L1	CLIONA M. MCHALE et al	Changes in the Peripheral Blood	Genomics	2009
Volatile Organic	TP53INP2	CLIONA M. MCHALE et al	Changes in the Peripheral Blood	Genomics	2009

Volatile Organic	TPCN2	CLIONA M. MCHALE et al	Changes in the Peripheral Blood	Genomics	2009
Volatile Organic	TM2D3	CLIONA M. MCHALE et al	Changes in the Peripheral Blood	Genomics	2009
Volatile Organic	KIAA1324L	CLIONA M. MCHALE et al	Changes in the Peripheral Blood	Genomics	2009
Volatile Organic	LITAF	CLIONA M. MCHALE et al	Changes in the Peripheral Blood	Genomics	2009
Volatile Organic	PI4KII	CLIONA M. MCHALE et al	Changes in the Peripheral Blood	Genomics	2009
Volatile Organic	SLC1A4	CLIONA M. MCHALE et al	Changes in the Peripheral Blood	Genomics	2009
Volatile Organic	CD59	CLIONA M. MCHALE et al	Changes in the Peripheral Blood	Genomics	2009
Volatile Organic	FBXW2	CLIONA M. MCHALE et al	Changes in the Peripheral Blood	Genomics	2009
Volatile Organic	TM2D2	CLIONA M. MCHALE et al	Changes in the Peripheral Blood	Genomics	2009
Volatile Organic	ST3GAL1	CLIONA M. MCHALE et al	Changes in the Peripheral Blood	Genomics	2009
Volatile Organic	SMC4L1	CLIONA M. MCHALE et al	Changes in the Peripheral Blood	Genomics	2009
Volatile Organic	FRAT2	CLIONA M. MCHALE et al	Changes in the Peripheral Blood	Genomics	2009
Volatile Organic	HIVEP2	CLIONA M. MCHALE et al	Changes in the Peripheral Blood	Genomics	2009
Volatile Organic	CDCP1	CLIONA M. MCHALE et al	Changes in the Peripheral Blood	Genomics	2009
Volatile Organic	C3orf29	CLIONA M. MCHALE et al	Changes in the Peripheral Blood	Genomics	2009
Volatile Organic	HBS1L	CLIONA M. MCHALE et al	Changes in the Peripheral Blood	Genomics	2009
Volatile Organic	SETD5	CLIONA M. MCHALE et al	Changes in the Peripheral Blood	Genomics	2009
Volatile Organic	TPD52L2	CLIONA M. MCHALE et al	Changes in the Peripheral Blood	Genomics	2009
Volatile Organic	LGALS8	CLIONA M. MCHALE et al	Changes in the Peripheral Blood	Genomics	2009
Volatile Organic	CXCR6	CLIONA M. MCHALE et al	Changes in the Peripheral Blood	Genomics	2009
Volatile Organic	TMEM1	CLIONA M. MCHALE et al	Changes in the Peripheral Blood	Genomics	2009
Volatile Organic	CXorf26	CLIONA M. MCHALE et al	Changes in the Peripheral Blood	Genomics	2009
Volatile Organic	ABCB1	CLIONA M. MCHALE et al	Changes in the Peripheral Blood	Genomics	2009
Volatile Organic	CSNK1D	CLIONA M. MCHALE et al	Changes in the Peripheral Blood	Genomics	2009
Volatile Organic	PSME4	CLIONA M. MCHALE et al	Changes in the Peripheral Blood	Genomics	2009
Volatile Organic	ENPP2	CLIONA M. MCHALE et al	Changes in the Peripheral Blood	Genomics	2009
Volatile Organic	RBM23	CLIONA M. MCHALE et al	Changes in the Peripheral Blood	Genomics	2009
Volatile Organic	CD86	CLIONA M. MCHALE et al	Changes in the Peripheral Blood	Genomics	2009
Volatile Organic	KIAA0310	CLIONA M. MCHALE et al	Changes in the Peripheral Blood	Genomics	2009
Volatile Organic	KRT8	CLIONA M. MCHALE et al	Changes in the Peripheral Blood	Genomics	2009
Volatile Organic	RBPSUH	CLIONA M. MCHALE et al	Changes in the Peripheral Blood	Genomics	2009
Volatile Organic	ATP1B3	CLIONA M. MCHALE et al	Changes in the Peripheral Blood	Genomics	2009
Volatile Organic	SLC17A5	CLIONA M. MCHALE et al	Changes in the Peripheral Blood	Genomics	2009
Volatile Organic	CSPG2	CLIONA M. MCHALE et al	Changes in the Peripheral Blood	Genomics	2009
Volatile Organic	VPS37C	CLIONA M. MCHALE et al	Changes in the Peripheral Blood	Genomics	2009
Volatile Organic	MCM6	CLIONA M. MCHALE et al	Changes in the Peripheral Blood	Genomics	2009
Volatile Organic	ADPRHL2	CLIONA M. MCHALE et al	Changes in the Peripheral Blood	Genomics	2009
Volatile Organic	NARF	CLIONA M. MCHALE et al	Changes in the Peripheral Blood	Genomics	2009
Volatile Organic	BTBD11	CLIONA M. MCHALE et al	Changes in the Peripheral Blood	Genomics	2009
Volatile Organic	CD93	CLIONA M. MCHALE et al	Changes in the Peripheral Blood	Genomics	2009
Volatile Organic	PDXK	CLIONA M. MCHALE et al	Changes in the Peripheral Blood	Genomics	2009
Volatile Organic	PTPRC	CLIONA M. MCHALE et al	Changes in the Peripheral Blood	Genomics	2009
Volatile Organic	IL4R	CLIONA M. MCHALE et al	Changes in the Peripheral Blood	Genomics	2009
Volatile Organic	SDS	CLIONA M. MCHALE et al	Changes in the Peripheral Blood	Genomics	2009
Volatile Organic	DYNC1H1	CLIONA M. MCHALE et al	Changes in the Peripheral Blood	Genomics	2009
Volatile Organic	PSCD1	CLIONA M. MCHALE et al	Changes in the Peripheral Blood	Genomics	2009
Volatile Organic	PDE4A	CLIONA M. MCHALE et al	Changes in the Peripheral Blood	Genomics	2009
Volatile Organic	COPB	CLIONA M. MCHALE et al	Changes in the Peripheral Blood	Genomics	2009
Volatile Organic	BPY2IP1	CLIONA M. MCHALE et al	Changes in the Peripheral Blood	Genomics	2009
Volatile Organic	ITGA5	CLIONA M. MCHALE et al	Changes in the Peripheral Blood	Genomics	2009
Volatile Organic	MGC17791	CLIONA M. MCHALE et al	Changes in the Peripheral Blood	Genomics	2009
Volatile Organic	PTPN22	CLIONA M. MCHALE et al	Changes in the Peripheral Blood	Genomics	2009
Volatile Organic	PTPN7	CLIONA M. MCHALE et al	Changes in the Peripheral Blood	Genomics	2009
Volatile Organic	MMP24	CLIONA M. MCHALE et al	Changes in the Peripheral Blood	Genomics	2009
Volatile Organic	PRKX	CLIONA M. MCHALE et al	Changes in the Peripheral Blood	Genomics	2009
Volatile Organic	HMGCR	CLIONA M. MCHALE et al	Changes in the Peripheral Blood	Genomics	2009
Volatile Organic	LANCL2	CLIONA M. MCHALE et al	Changes in the Peripheral Blood	Genomics	2009
Volatile Organic	KIAA0143	CLIONA M. MCHALE et al	Changes in the Peripheral Blood	Genomics	2009
Volatile Organic	CD5	CLIONA M. MCHALE et al	Changes in the Peripheral Blood	Genomics	2009
Volatile Organic	AP3M2	CLIONA M. MCHALE et al	Changes in the Peripheral Blood	Genomics	2009
Volatile Organic	EIF3S9	CLIONA M. MCHALE et al	Changes in the Peripheral Blood	Genomics	2009
Volatile Organic	PELO	CLIONA M. MCHALE et al	Changes in the Peripheral Blood	Genomics	2009
Volatile Organic	P2RX4	CLIONA M. MCHALE et al	Changes in the Peripheral Blood	Genomics	2009
Volatile Organic	PRPF4	CLIONA M. MCHALE et al	Changes in the Peripheral Blood	Genomics	2009
Volatile Organic	ST3GAL5	CLIONA M. MCHALE et al	Changes in the Peripheral Blood	Genomics	2009
Volatile Organic	ANKDD1A	CLIONA M. MCHALE et al	Changes in the Peripheral Blood	Genomics	2009
Volatile Organic	RHBDD2	CLIONA M. MCHALE et al	Changes in the Peripheral Blood	Genomics	2009
Volatile Organic	FMNL1	CLIONA M. MCHALE et al	Changes in the Peripheral Blood	Genomics	2009
Volatile Organic	DHRS9	CLIONA M. MCHALE et al	Changes in the Peripheral Blood	Genomics	2009
Volatile Organic	PLEKHB2	CLIONA M. MCHALE et al	Changes in the Peripheral Blood	Genomics	2009
Volatile Organic	KIAA1600	CLIONA M. MCHALE et al	Changes in the Peripheral Blood	Genomics	2009
Volatile Organic	CRNKL1	CLIONA M. MCHALE et al	Changes in the Peripheral Blood	Genomics	2009
Volatile Organic	MYST3	CLIONA M. MCHALE et al	Changes in the Peripheral Blood	Genomics	2009
Volatile Organic	ATP2A2	CLIONA M. MCHALE et al	Changes in the Peripheral Blood	Genomics	2009
Volatile Organic	PLEKHQ1	CLIONA M. MCHALE et al	Changes in the Peripheral Blood	Genomics	2009
Volatile Organic	EDD1	CLIONA M. MCHALE et al	Changes in the Peripheral Blood	Genomics	2009
Volatile Organic	ZNF289	CLIONA M. MCHALE et al	Changes in the Peripheral Blood	Genomics	2009
Volatile Organic	C12orf22	CLIONA M. MCHALE et al	Changes in the Peripheral Blood	Genomics	2009
Volatile Organic	SC5DL	CLIONA M. MCHALE et al	Changes in the Peripheral Blood	Genomics	2009
Volatile Organic	SRP54	CLIONA M. MCHALE et al	Changes in the Peripheral Blood	Genomics	2009
Volatile Organic	TAF5	CLIONA M. MCHALE et al	Changes in the Peripheral Blood	Genomics	2009
Volatile Organic	VAPA	CLIONA M. MCHALE et al	Changes in the Peripheral Blood	Genomics	2009
Volatile Organic	AGPAT6	CLIONA M. MCHALE et al	Changes in the Peripheral Blood	Genomics	2009
Volatile Organic	EXOC1	CLIONA M. MCHALE et al	Changes in the Peripheral Blood	Genomics	2009
Volatile Organic	GFI1	CLIONA M. MCHALE et al	Changes in the Peripheral Blood	Genomics	2009
Volatile Organic	KLHL12	CLIONA M. MCHALE et al	Changes in the Peripheral Blood	Genomics	2009
Volatile Organic	C6orf113	CLIONA M. MCHALE et al	Changes in the Peripheral Blood	Genomics	2009
Volatile Organic	BCOR	CLIONA M. MCHALE et al	Changes in the Peripheral Blood	Genomics	2009
Volatile Organic	C6orf75	CLIONA M. MCHALE et al	Changes in the Peripheral Blood	Genomics	2009

Volatile Organic	MAFG	CLIONA M. MCHALE et al	Changes in the Peripheral Blood	Genomics	2009
Volatile Organic	CD7	CLIONA M. MCHALE et al	Changes in the Peripheral Blood	Genomics	2009
Volatile Organic	SREBF1	CLIONA M. MCHALE et al	Changes in the Peripheral Blood	Genomics	2009
Volatile Organic	HARSL	CLIONA M. MCHALE et al	Changes in the Peripheral Blood	Genomics	2009
Volatile Organic	MLL4	CLIONA M. MCHALE et al	Changes in the Peripheral Blood	Genomics	2009
Volatile Organic	INPP1	CLIONA M. MCHALE et al	Changes in the Peripheral Blood	Genomics	2009
Volatile Organic	GPR157	CLIONA M. MCHALE et al	Changes in the Peripheral Blood	Genomics	2009
Volatile Organic	TBC1D2B	CLIONA M. MCHALE et al	Changes in the Peripheral Blood	Genomics	2009
Volatile Organic	KPNA1	CLIONA M. MCHALE et al	Changes in the Peripheral Blood	Genomics	2009
Volatile Organic	GMEB2	CLIONA M. MCHALE et al	Changes in the Peripheral Blood	Genomics	2009
Volatile Organic	MFAP1	CLIONA M. MCHALE et al	Changes in the Peripheral Blood	Genomics	2009
Volatile Organic	DLL1	CLIONA M. MCHALE et al	Changes in the Peripheral Blood	Genomics	2009
Volatile Organic	MANBAL	CLIONA M. MCHALE et al	Changes in the Peripheral Blood	Genomics	2009
Volatile Organic	TTC19	CLIONA M. MCHALE et al	Changes in the Peripheral Blood	Genomics	2009
Volatile Organic	GLUL	CLIONA M. MCHALE et al	Changes in the Peripheral Blood	Genomics	2009
Volatile Organic	TOR1B	CLIONA M. MCHALE et al	Changes in the Peripheral Blood	Genomics	2009
Volatile Organic	SORL1	CLIONA M. MCHALE et al	Changes in the Peripheral Blood	Genomics	2009
Volatile Organic	ZNF700	CLIONA M. MCHALE et al	Changes in the Peripheral Blood	Genomics	2009
Volatile Organic	KIAA2002	CLIONA M. MCHALE et al	Changes in the Peripheral Blood	Genomics	2009
Volatile Organic	VPS26A	CLIONA M. MCHALE et al	Changes in the Peripheral Blood	Genomics	2009
Volatile Organic	PRPF8	CLIONA M. MCHALE et al	Changes in the Peripheral Blood	Genomics	2009
Volatile Organic	ST6GALNAC2	CLIONA M. MCHALE et al	Changes in the Peripheral Blood	Genomics	2009
Volatile Organic	ZNF655	CLIONA M. MCHALE et al	Changes in the Peripheral Blood	Genomics	2009
Volatile Organic	TESK1	CLIONA M. MCHALE et al	Changes in the Peripheral Blood	Genomics	2009
Volatile Organic	LOC93622	CLIONA M. MCHALE et al	Changes in the Peripheral Blood	Genomics	2009
Volatile Organic	ZNF200	CLIONA M. MCHALE et al	Changes in the Peripheral Blood	Genomics	2009
Volatile Organic	KIAA0467	CLIONA M. MCHALE et al	Changes in the Peripheral Blood	Genomics	2009
Volatile Organic	RAB2B	CLIONA M. MCHALE et al	Changes in the Peripheral Blood	Genomics	2009
Volatile Organic	DHX40	CLIONA M. MCHALE et al	Changes in the Peripheral Blood	Genomics	2009
Volatile Organic	UBE2Q1	CLIONA M. MCHALE et al	Changes in the Peripheral Blood	Genomics	2009
Volatile Organic	POLR2B	CLIONA M. MCHALE et al	Changes in the Peripheral Blood	Genomics	2009
Volatile Organic	POR	CLIONA M. MCHALE et al	Changes in the Peripheral Blood	Genomics	2009
Volatile Organic	TMEM70	CLIONA M. MCHALE et al	Changes in the Peripheral Blood	Genomics	2009
Volatile Organic	AKNA	CLIONA M. MCHALE et al	Changes in the Peripheral Blood	Genomics	2009
Volatile Organic	XPC	CLIONA M. MCHALE et al	Changes in the Peripheral Blood	Genomics	2009
Volatile Organic	GGA2	CLIONA M. MCHALE et al	Changes in the Peripheral Blood	Genomics	2009
Volatile Organic	PPM1F	CLIONA M. MCHALE et al	Changes in the Peripheral Blood	Genomics	2009
Volatile Organic	ARRDC2	CLIONA M. MCHALE et al	Changes in the Peripheral Blood	Genomics	2009
Volatile Organic	BTN2A1	CLIONA M. MCHALE et al	Changes in the Peripheral Blood	Genomics	2009
Volatile Organic	RP13-297E16.1	CLIONA M. MCHALE et al	Changes in the Peripheral Blood	Genomics	2009
Volatile Organic	SFRS2	CLIONA M. MCHALE et al	Changes in the Peripheral Blood	Genomics	2009
Volatile Organic	AMPD2	CLIONA M. MCHALE et al	Changes in the Peripheral Blood	Genomics	2009
Volatile Organic	FASN	CLIONA M. MCHALE et al	Changes in the Peripheral Blood	Genomics	2009
Volatile Organic	SPOCK2	CLIONA M. MCHALE et al	Changes in the Peripheral Blood	Genomics	2009
Volatile Organic	LPXN	CLIONA M. MCHALE et al	Changes in the Peripheral Blood	Genomics	2009
Volatile Organic	BAP1	CLIONA M. MCHALE et al	Changes in the Peripheral Blood	Genomics	2009
Volatile Organic	RAB11FIP4	CLIONA M. MCHALE et al	Changes in the Peripheral Blood	Genomics	2009
Volatile Organic	PPP1R10	CLIONA M. MCHALE et al	Changes in the Peripheral Blood	Genomics	2009
Volatile Organic	STK40	CLIONA M. MCHALE et al	Changes in the Peripheral Blood	Genomics	2009
Volatile Organic	HSPC049	CLIONA M. MCHALE et al	Changes in the Peripheral Blood	Genomics	2009
Volatile Organic	STRAP	CLIONA M. MCHALE et al	Changes in the Peripheral Blood	Genomics	2009
Volatile Organic	KLHDC4	CLIONA M. MCHALE et al	Changes in the Peripheral Blood	Genomics	2009
Volatile Organic	ING5	CLIONA M. MCHALE et al	Changes in the Peripheral Blood	Genomics	2009
Volatile Organic	LEPROTL1	CLIONA M. MCHALE et al	Changes in the Peripheral Blood	Genomics	2009
Volatile Organic	CRADD	CLIONA M. MCHALE et al	Changes in the Peripheral Blood	Genomics	2009
Volatile Organic	RRAGC	CLIONA M. MCHALE et al	Changes in the Peripheral Blood	Genomics	2009
Volatile Organic	SLC15A3	CLIONA M. MCHALE et al	Changes in the Peripheral Blood	Genomics	2009
Volatile Organic	CASC3	CLIONA M. MCHALE et al	Changes in the Peripheral Blood	Genomics	2009
Volatile Organic	CLN8	CLIONA M. MCHALE et al	Changes in the Peripheral Blood	Genomics	2009
Volatile Organic	MGC11257	CLIONA M. MCHALE et al	Changes in the Peripheral Blood	Genomics	2009
Volatile Organic	E2F6	CLIONA M. MCHALE et al	Changes in the Peripheral Blood	Genomics	2009
Volatile Organic	RIC8A	CLIONA M. MCHALE et al	Changes in the Peripheral Blood	Genomics	2009
Volatile Organic	ANKH	CLIONA M. MCHALE et al	Changes in the Peripheral Blood	Genomics	2009
Volatile Organic	GPR137B	CLIONA M. MCHALE et al	Changes in the Peripheral Blood	Genomics	2009
Volatile Organic	MAN1B1	CLIONA M. MCHALE et al	Changes in the Peripheral Blood	Genomics	2009
Volatile Organic	IDS	CLIONA M. MCHALE et al	Changes in the Peripheral Blood	Genomics	2009
Volatile Organic	ABL1	CLIONA M. MCHALE et al	Changes in the Peripheral Blood	Genomics	2009
Volatile Organic	SLC35E1	CLIONA M. MCHALE et al	Changes in the Peripheral Blood	Genomics	2009
Volatile Organic	MAPKAPK2	CLIONA M. MCHALE et al	Changes in the Peripheral Blood	Genomics	2009
Volatile Organic	DOCK5	CLIONA M. MCHALE et al	Changes in the Peripheral Blood	Genomics	2009
Volatile Organic	FAM98A	CLIONA M. MCHALE et al	Changes in the Peripheral Blood	Genomics	2009
Volatile Organic	HEG1	CLIONA M. MCHALE et al	Changes in the Peripheral Blood	Genomics	2009
Volatile Organic	ASMTL	CLIONA M. MCHALE et al	Changes in the Peripheral Blood	Genomics	2009
Volatile Organic	DDX52	CLIONA M. MCHALE et al	Changes in the Peripheral Blood	Genomics	2009
Volatile Organic	ECOP	CLIONA M. MCHALE et al	Changes in the Peripheral Blood	Genomics	2009
Volatile Organic	KLHL21	CLIONA M. MCHALE et al	Changes in the Peripheral Blood	Genomics	2009
Volatile Organic	YKT6	CLIONA M. MCHALE et al	Changes in the Peripheral Blood	Genomics	2009
Volatile Organic	AHCTF1	CLIONA M. MCHALE et al	Changes in the Peripheral Blood	Genomics	2009
Volatile Organic	TNRC6A	CLIONA M. MCHALE et al	Changes in the Peripheral Blood	Genomics	2009
Volatile Organic	CTPS	CLIONA M. MCHALE et al	Changes in the Peripheral Blood	Genomics	2009
Volatile Organic	C6orf59	CLIONA M. MCHALE et al	Changes in the Peripheral Blood	Genomics	2009
Volatile Organic	ALG2	CLIONA M. MCHALE et al	Changes in the Peripheral Blood	Genomics	2009
Volatile Organic	TMEM127	CLIONA M. MCHALE et al	Changes in the Peripheral Blood	Genomics	2009
Volatile Organic	HKR2	CLIONA M. MCHALE et al	Changes in the Peripheral Blood	Genomics	2009
Volatile Organic	PRO0149	CLIONA M. MCHALE et al	Changes in the Peripheral Blood	Genomics	2009
Volatile Organic	TUBB3	CLIONA M. MCHALE et al	Changes in the Peripheral Blood	Genomics	2009
Volatile Organic	GK	CLIONA M. MCHALE et al	Changes in the Peripheral Blood	Genomics	2009
Volatile Organic	GSPT1	CLIONA M. MCHALE et al	Changes in the Peripheral Blood	Genomics	2009
Volatile Organic	LOC89944	CLIONA M. MCHALE et al	Changes in the Peripheral Blood	Genomics	2009

Volatile Organic	STATIP1	CLIONA M. MCHALE et al	Changes in the Peripheral Blood	Genomics	2009
Volatile Organic	TMPIT	CLIONA M. MCHALE et al	Changes in the Peripheral Blood	Genomics	2009
Volatile Organic	FLJ20209	CLIONA M. MCHALE et al	Changes in the Peripheral Blood	Genomics	2009
Volatile Organic	ZNF230	CLIONA M. MCHALE et al	Changes in the Peripheral Blood	Genomics	2009
Volatile Organic	SIPA1	CLIONA M. MCHALE et al	Changes in the Peripheral Blood	Genomics	2009
Volatile Organic	JARID1B	CLIONA M. MCHALE et al	Changes in the Peripheral Blood	Genomics	2009
Volatile Organic	PLEKHM1	CLIONA M. MCHALE et al	Changes in the Peripheral Blood	Genomics	2009
Volatile Organic	NFATC1	CLIONA M. MCHALE et al	Changes in the Peripheral Blood	Genomics	2009
Volatile Organic	DOPEY1	CLIONA M. MCHALE et al	Changes in the Peripheral Blood	Genomics	2009
Volatile Organic	SH2D2A	CLIONA M. MCHALE et al	Changes in the Peripheral Blood	Genomics	2009
Volatile Organic	CCT6B	CLIONA M. MCHALE et al	Changes in the Peripheral Blood	Genomics	2009
Volatile Organic	ATRN	CLIONA M. MCHALE et al	Changes in the Peripheral Blood	Genomics	2009
Volatile Organic	ARID2	CLIONA M. MCHALE et al	Changes in the Peripheral Blood	Genomics	2009
Volatile Organic	ILVBL	CLIONA M. MCHALE et al	Changes in the Peripheral Blood	Genomics	2009
Volatile Organic	HPS6	CLIONA M. MCHALE et al	Changes in the Peripheral Blood	Genomics	2009
Volatile Organic	ZNF434	CLIONA M. MCHALE et al	Changes in the Peripheral Blood	Genomics	2009
Volatile Organic	RAB22A	CLIONA M. MCHALE et al	Changes in the Peripheral Blood	Genomics	2009
Volatile Organic	PHF20L1	CLIONA M. MCHALE et al	Changes in the Peripheral Blood	Genomics	2009
Volatile Organic	GPSN2	CLIONA M. MCHALE et al	Changes in the Peripheral Blood	Genomics	2009
Volatile Organic	TNFAIP8L1	CLIONA M. MCHALE et al	Changes in the Peripheral Blood	Genomics	2009
Volatile Organic	CUL4A	CLIONA M. MCHALE et al	Changes in the Peripheral Blood	Genomics	2009
Volatile Organic	TSEN54	CLIONA M. MCHALE et al	Changes in the Peripheral Blood	Genomics	2009
Volatile Organic	CPT2	CLIONA M. MCHALE et al	Changes in the Peripheral Blood	Genomics	2009
Volatile Organic	ZNF268	CLIONA M. MCHALE et al	Changes in the Peripheral Blood	Genomics	2009
Volatile Organic	OXSM	CLIONA M. MCHALE et al	Changes in the Peripheral Blood	Genomics	2009
Volatile Organic	SPAG4	CLIONA M. MCHALE et al	Changes in the Peripheral Blood	Genomics	2009
Volatile Organic	NUP205	CLIONA M. MCHALE et al	Changes in the Peripheral Blood	Genomics	2009
Volatile Organic	C20orf4	CLIONA M. MCHALE et al	Changes in the Peripheral Blood	Genomics	2009
Volatile Organic	KIAA1539	CLIONA M. MCHALE et al	Changes in the Peripheral Blood	Genomics	2009
Volatile Organic	RNF24	CLIONA M. MCHALE et al	Changes in the Peripheral Blood	Genomics	2009
Volatile Organic	TRIM39	CLIONA M. MCHALE et al	Changes in the Peripheral Blood	Genomics	2009
Volatile Organic	WIPI1	CLIONA M. MCHALE et al	Changes in the Peripheral Blood	Genomics	2009
Volatile Organic	PGK1	CLIONA M. MCHALE et al	Changes in the Peripheral Blood	Genomics	2009
Volatile Organic	RIPK5	CLIONA M. MCHALE et al	Changes in the Peripheral Blood	Genomics	2009
Volatile Organic	EIF4ENIF1	CLIONA M. MCHALE et al	Changes in the Peripheral Blood	Genomics	2009
Volatile Organic	DIDO1	CLIONA M. MCHALE et al	Changes in the Peripheral Blood	Genomics	2009
Volatile Organic	GGA1	CLIONA M. MCHALE et al	Changes in the Peripheral Blood	Genomics	2009
Volatile Organic	RARA	CLIONA M. MCHALE et al	Changes in the Peripheral Blood	Genomics	2009
Volatile Organic	KIAA0329	CLIONA M. MCHALE et al	Changes in the Peripheral Blood	Genomics	2009
Volatile Organic	C7orf19	CLIONA M. MCHALE et al	Changes in the Peripheral Blood	Genomics	2009
Volatile Organic	SPAG5	CLIONA M. MCHALE et al	Changes in the Peripheral Blood	Genomics	2009
Volatile Organic	ZC3H8	CLIONA M. MCHALE et al	Changes in the Peripheral Blood	Genomics	2009
Volatile Organic	TAOK2	CLIONA M. MCHALE et al	Changes in the Peripheral Blood	Genomics	2009
Volatile Organic	SLCO3A1	CLIONA M. MCHALE et al	Changes in the Peripheral Blood	Genomics	2009
Volatile Organic	GAS2L3	CLIONA M. MCHALE et al	Changes in the Peripheral Blood	Genomics	2009
Volatile Organic	MGC5139	CLIONA M. MCHALE et al	Changes in the Peripheral Blood	Genomics	2009
Volatile Organic	CASP3	CLIONA M. MCHALE et al	Changes in the Peripheral Blood	Genomics	2009
Volatile Organic	MYL5	CLIONA M. MCHALE et al	Changes in the Peripheral Blood	Genomics	2009
Volatile Organic	VENTX	CLIONA M. MCHALE et al	Changes in the Peripheral Blood	Genomics	2009
Volatile Organic	CASP9	CLIONA M. MCHALE et al	Changes in the Peripheral Blood	Genomics	2009
Volatile Organic	PHLPPL	CLIONA M. MCHALE et al	Changes in the Peripheral Blood	Genomics	2009
Volatile Organic	TNIP2	CLIONA M. MCHALE et al	Changes in the Peripheral Blood	Genomics	2009
Volatile Organic	ZNF615	CLIONA M. MCHALE et al	Changes in the Peripheral Blood	Genomics	2009
Volatile Organic	NFE2L3	CLIONA M. MCHALE et al	Changes in the Peripheral Blood	Genomics	2009
Volatile Organic	TRAF3	CLIONA M. MCHALE et al	Changes in the Peripheral Blood	Genomics	2009
Volatile Organic	DUSP18	CLIONA M. MCHALE et al	Changes in the Peripheral Blood	Genomics	2009
Volatile Organic	PCOLN3	CLIONA M. MCHALE et al	Changes in the Peripheral Blood	Genomics	2009
Volatile Organic	SNF1LK2	CLIONA M. MCHALE et al	Changes in the Peripheral Blood	Genomics	2009
Volatile Organic	MYD88	CLIONA M. MCHALE et al	Changes in the Peripheral Blood	Genomics	2009
Volatile Organic	UBL3	CLIONA M. MCHALE et al	Changes in the Peripheral Blood	Genomics	2009
Volatile Organic	OSBP	CLIONA M. MCHALE et al	Changes in the Peripheral Blood	Genomics	2009
Volatile Organic	TMEM39A	CLIONA M. MCHALE et al	Changes in the Peripheral Blood	Genomics	2009
Volatile Organic	SIRT3	CLIONA M. MCHALE et al	Changes in the Peripheral Blood	Genomics	2009
Volatile Organic	DFNA5	CLIONA M. MCHALE et al	Changes in the Peripheral Blood	Genomics	2009
Volatile Organic	GTF3C2	CLIONA M. MCHALE et al	Changes in the Peripheral Blood	Genomics	2009
Volatile Organic	RBKS	CLIONA M. MCHALE et al	Changes in the Peripheral Blood	Genomics	2009
Volatile Organic	OPTN	CLIONA M. MCHALE et al	Changes in the Peripheral Blood	Genomics	2009
Volatile Organic	PDCD4	CLIONA M. MCHALE et al	Changes in the Peripheral Blood	Genomics	2009
Volatile Organic	NFKBIB	CLIONA M. MCHALE et al	Changes in the Peripheral Blood	Genomics	2009
Volatile Organic	PPME1	CLIONA M. MCHALE et al	Changes in the Peripheral Blood	Genomics	2009
Volatile Organic	EGLN2	CLIONA M. MCHALE et al	Changes in the Peripheral Blood	Genomics	2009
Volatile Organic	MAP3K7	CLIONA M. MCHALE et al	Changes in the Peripheral Blood	Genomics	2009
Volatile Organic	TRIM11	CLIONA M. MCHALE et al	Changes in the Peripheral Blood	Genomics	2009
Volatile Organic	CNOT1	CLIONA M. MCHALE et al	Changes in the Peripheral Blood	Genomics	2009
Volatile Organic	ZBTB3	CLIONA M. MCHALE et al	Changes in the Peripheral Blood	Genomics	2009
Volatile Organic	C21orf45	CLIONA M. MCHALE et al	Changes in the Peripheral Blood	Genomics	2009
Volatile Organic	ZNF235	CLIONA M. MCHALE et al	Changes in the Peripheral Blood	Genomics	2009
Volatile Organic	TMEM15	CLIONA M. MCHALE et al	Changes in the Peripheral Blood	Genomics	2009
Volatile Organic	EIF2C1	CLIONA M. MCHALE et al	Changes in the Peripheral Blood	Genomics	2009
Volatile Organic	RUNX1	CLIONA M. MCHALE et al	Changes in the Peripheral Blood	Genomics	2009
Volatile Organic	KIF1B	CLIONA M. MCHALE et al	Changes in the Peripheral Blood	Genomics	2009
Volatile Organic	RP11-484I6.3	CLIONA M. MCHALE et al	Changes in the Peripheral Blood	Genomics	2009
Volatile Organic	RHOF	CLIONA M. MCHALE et al	Changes in the Peripheral Blood	Genomics	2009
Volatile Organic	KIAA0368	CLIONA M. MCHALE et al	Changes in the Peripheral Blood	Genomics	2009
Volatile Organic	BCDIN3	CLIONA M. MCHALE et al	Changes in the Peripheral Blood	Genomics	2009
Volatile Organic	SPON1	CLIONA M. MCHALE et al	Changes in the Peripheral Blood	Genomics	2009
Volatile Organic	AMDHD2	CLIONA M. MCHALE et al	Changes in the Peripheral Blood	Genomics	2009
Volatile Organic	DGAT2	CLIONA M. MCHALE et al	Changes in the Peripheral Blood	Genomics	2009
Volatile Organic	RSPRY1	CLIONA M. MCHALE et al	Changes in the Peripheral Blood	Genomics	2009

Volatile Organic	ZNF198	CLIONA M. MCHALE et al	Changes in the Peripheral Blood	Genomics	2009
Volatile Organic	RANGAP1	CLIONA M. MCHALE et al	Changes in the Peripheral Blood	Genomics	2009
Volatile Organic	POLR3F	CLIONA M. MCHALE et al	Changes in the Peripheral Blood	Genomics	2009
Volatile Organic	FAM40A	CLIONA M. MCHALE et al	Changes in the Peripheral Blood	Genomics	2009
Volatile Organic	C8orf76	CLIONA M. MCHALE et al	Changes in the Peripheral Blood	Genomics	2009
Volatile Organic	ZNF250	CLIONA M. MCHALE et al	Changes in the Peripheral Blood	Genomics	2009
Volatile Organic	HYAL2	CLIONA M. MCHALE et al	Changes in the Peripheral Blood	Genomics	2009
Volatile Organic	TMEM87A	CLIONA M. MCHALE et al	Changes in the Peripheral Blood	Genomics	2009
Volatile Organic	KBTBD7	CLIONA M. MCHALE et al	Changes in the Peripheral Blood	Genomics	2009
Volatile Organic	CYP2E1	CLIONA M. MCHALE et al	Changes in the Peripheral Blood	Genomics	2009
Volatile Organic	NF1	CLIONA M. MCHALE et al	Changes in the Peripheral Blood	Genomics	2009
Volatile Organic	ASXL1	CLIONA M. MCHALE et al	Changes in the Peripheral Blood	Genomics	2009
Volatile Organic	ACOT9	CLIONA M. MCHALE et al	Changes in the Peripheral Blood	Genomics	2009
Volatile Organic	ABCG1	CLIONA M. MCHALE et al	Changes in the Peripheral Blood	Genomics	2009
Volatile Organic	COPA	CLIONA M. MCHALE et al	Changes in the Peripheral Blood	Genomics	2009
Volatile Organic	TBC1D13	CLIONA M. MCHALE et al	Changes in the Peripheral Blood	Genomics	2009
Volatile Organic	RRAGB	CLIONA M. MCHALE et al	Changes in the Peripheral Blood	Genomics	2009
Volatile Organic	RAB5B	CLIONA M. MCHALE et al	Changes in the Peripheral Blood	Genomics	2009
Volatile Organic	ZNF337	CLIONA M. MCHALE et al	Changes in the Peripheral Blood	Genomics	2009
Volatile Organic	ZFAND2B	CLIONA M. MCHALE et al	Changes in the Peripheral Blood	Genomics	2009
Volatile Organic	KIAA0652	CLIONA M. MCHALE et al	Changes in the Peripheral Blood	Genomics	2009
Volatile Organic	SESN2	CLIONA M. MCHALE et al	Changes in the Peripheral Blood	Genomics	2009
Volatile Organic	C10orf39	CLIONA M. MCHALE et al	Changes in the Peripheral Blood	Genomics	2009
Volatile Organic	STAU1	CLIONA M. MCHALE et al	Changes in the Peripheral Blood	Genomics	2009
Volatile Organic	SEC14L2	CLIONA M. MCHALE et al	Changes in the Peripheral Blood	Genomics	2009
Volatile Organic	XPO5	CLIONA M. MCHALE et al	Changes in the Peripheral Blood	Genomics	2009
Volatile Organic	CHRNA10	CLIONA M. MCHALE et al	Changes in the Peripheral Blood	Genomics	2009
Volatile Organic	CNOT3	CLIONA M. MCHALE et al	Changes in the Peripheral Blood	Genomics	2009
Volatile Organic	TINF2	CLIONA M. MCHALE et al	Changes in the Peripheral Blood	Genomics	2009
Volatile Organic	ZNF416	CLIONA M. MCHALE et al	Changes in the Peripheral Blood	Genomics	2009
Volatile Organic	RNF121	CLIONA M. MCHALE et al	Changes in the Peripheral Blood	Genomics	2009
Volatile Organic	C1ORF52	CLIONA M. MCHALE et al	Changes in the Peripheral Blood	Genomics	2009
Volatile Organic	SUV420H1	CLIONA M. MCHALE et al	Changes in the Peripheral Blood	Genomics	2009
Volatile Organic	RABEP1	CLIONA M. MCHALE et al	Changes in the Peripheral Blood	Genomics	2009
PAHs	ILK	Mi-Kyung Song, Youn-Jung	Gene Expression Analysis Identifies Potential	Toxicology and Environmental Health	2011
PAHs	VEGF	Mi-Kyung Song, Youn-Jung	Gene Expression Analysis Identifies Potential	Toxicology and Environmental Health	2011
PAHs	JAG1	Mi-Kyung Song, Youn-Jung	Gene Expression Analysis Identifies Potential	Toxicology and Environmental Health	2011
PAHs	UNC5C	Mi-Kyung Song, Youn-Jung	Gene Expression Analysis Identifies Potential	Toxicology and Environmental Health	2011
PAHs	CXCL16	Mi-Kyung Song, Youn-Jung	Gene Expression Analysis Identifies Potential	Toxicology and Environmental Health	2011
PAHs	ROBO4	Mi-Kyung Song, Youn-Jung	Gene Expression Analysis Identifies Potential	Toxicology and Environmental Health	2011
PAHs	CXCL12	Mi-Kyung Song, Youn-Jung	Gene Expression Analysis Identifies Potential	Toxicology and Environmental Health	2011
PAHs	FURIN	Mi-Kyung Song, Youn-Jung	Gene Expression Analysis Identifies Potential	Toxicology and Environmental Health	2011
PAHs	F2RL1	Mi-Kyung Song, Youn-Jung	Gene Expression Analysis Identifies Potential	Toxicology and Environmental Health	2011
PAHs	RRAS	Mi-Kyung Song, Youn-Jung	Gene Expression Analysis Identifies Potential	Toxicology and Environmental Health	2011
PAHs	GNA13	Mi-Kyung Song, Youn-Jung	Gene Expression Analysis Identifies Potential	Toxicology and Environmental Health	2011
PAHs	TRIB1	Mi-Kyung Song, Youn-Jung	Gene Expression Analysis Identifies Potential	Toxicology and Environmental Health	2011
PAHs	CSF1	Mi-Kyung Song, Youn-Jung	Gene Expression Analysis Identifies Potential	Toxicology and Environmental Health	2011
PAHs	TGFB1	Mi-Kyung Song, Youn-Jung	Gene Expression Analysis Identifies Potential	Toxicology and Environmental Health	2011
PAHs	ITGA2	Mi-Kyung Song, Youn-Jung	Gene Expression Analysis Identifies Potential	Toxicology and Environmental Health	2011
PAHs	PPARD	Mi-Kyung Song, Youn-Jung	Gene Expression Analysis Identifies Potential	Toxicology and Environmental Health	2011
PAHs	P2RX1	Mi-Kyung Song, Youn-Jung	Gene Expression Analysis Identifies Potential	Toxicology and Environmental Health	2011
PAHs	PLAUR	Mi-Kyung Song, Youn-Jung	Gene Expression Analysis Identifies Potential	Toxicology and Environmental Health	2011
PAHs	IGFBP1	Mi-Kyung Song, Youn-Jung	Gene Expression Analysis Identifies Potential	Toxicology and Environmental Health	2011
PAHs	IL11	Mi-Kyung Song, Youn-Jung	Gene Expression Analysis Identifies Potential	Toxicology and Environmental Health	2011
PAHs	KLF6	Mi-Kyung Song, Youn-Jung	Gene Expression Analysis Identifies Potential	Toxicology and Environmental Health	2011
PAHs	CD9	Mi-Kyung Song, Youn-Jung	Gene Expression Analysis Identifies Potential	Toxicology and Environmental Health	2011
PAHs	PLAU	Mi-Kyung Song, Youn-Jung	Gene Expression Analysis Identifies Potential	Toxicology and Environmental Health	2011
PAHs	STXBP1	Mi-Kyung Song, Youn-Jung	Gene Expression Analysis Identifies Potential	Toxicology and Environmental Health	2011
PAHs	NINJ1	Mi-Kyung Song, Youn-Jung	Gene Expression Analysis Identifies Potential	Toxicology and Environmental Health	2011
PAHs	ERBB3	Mi-Kyung Song, Youn-Jung	Gene Expression Analysis Identifies Potential	Toxicology and Environmental Health	2011
PAHs	PLXNA2	Mi-Kyung Song, Youn-Jung	Gene Expression Analysis Identifies Potential	Toxicology and Environmental Health	2011
PAHs	NRXN1	Mi-Kyung Song, Youn-Jung	Gene Expression Analysis Identifies Potential	Toxicology and Environmental Health	2011
PAHs	UNC5B	Mi-Kyung Song, Youn-Jung	Gene Expression Analysis Identifies Potential	Toxicology and Environmental Health	2011
PAHs	SEMA3B	Mi-Kyung Song, Youn-Jung	Gene Expression Analysis Identifies Potential	Toxicology and Environmental Health	2011
PAHs	DCAMKL1	Mi-Kyung Song, Youn-Jung	Gene Expression Analysis Identifies Potential	Toxicology and Environmental Health	2011
PAHs	GDNF	Mi-Kyung Song, Youn-Jung	Gene Expression Analysis Identifies Potential	Toxicology and Environmental Health	2011
PAHs	VNN1	Mi-Kyung Song, Youn-Jung	Gene Expression Analysis Identifies Potential	Toxicology and Environmental Health	2011
PAHs	DNAH17	Mi-Kyung Song, Youn-Jung	Gene Expression Analysis Identifies Potential	Toxicology and Environmental Health	2011
PAHs	THBS4	Mi-Kyung Song, Youn-Jung	Gene Expression Analysis Identifies Potential	Toxicology and Environmental Health	2011
PAHs	ACTG1	Mi-Kyung Song, Youn-Jung	Gene Expression Analysis Identifies Potential	Toxicology and Environmental Health	2011
PAHs	TWIST1	Mi-Kyung Song, Youn-Jung	Gene Expression Analysis Identifies Potential	Toxicology and Environmental Health	2011
PAHs	DSTN	Mi-Kyung Song, Youn-Jung	Gene Expression Analysis Identifies Potential	Toxicology and Environmental Health	2011
PAHs	ITGB1	Mi-Kyung Song, Youn-Jung	Gene Expression Analysis Identifies Potential	Toxicology and Environmental Health	2011
PAHs	PRKCA	Mi-Kyung Song, Youn-Jung	Gene Expression Analysis Identifies Potential	Toxicology and Environmental Health	2011
PAHs	VASP	Mi-Kyung Song, Youn-Jung	Gene Expression Analysis Identifies Potential	Toxicology and Environmental Health	2011
PAHs	NEURL	Mi-Kyung Song, Youn-Jung	Gene Expression Analysis Identifies Potential	Toxicology and Environmental Health	2011
PAHs	PVR	Mi-Kyung Song, Youn-Jung	Gene Expression Analysis Identifies Potential	Toxicology and Environmental Health	2011
PAHs	GAB2	Mi-Kyung Song, Youn-Jung	Gene Expression Analysis Identifies Potential	Toxicology and Environmental Health	2011
PAHs	TNS	Mi-Kyung Song, Youn-Jung	Gene Expression Analysis Identifies Potential	Toxicology and Environmental Health	2011
PAHs	ETV4	Mi-Kyung Song, Youn-Jung	Gene Expression Analysis Identifies Potential	Toxicology and Environmental Health	2011
PAHs	IGFBP3	Mi-Kyung Song, Youn-Jung	Gene Expression Analysis Identifies Potential	Toxicology and Environmental Health	2011
PAHs	PDGFA	Mi-Kyung Song, Youn-Jung	Gene Expression Analysis Identifies Potential	Toxicology and Environmental Health	2011
PAHs	ABCB7	Mi-Kyung Song, Mee Song	Identification of molecular signatures	Toxicology Letters	2012
PAHs	ABCC2	Mi-Kyung Song, Mee Song	Identification of molecular signatures	Toxicology Letters	2012
PAHs	ACOT2	Mi-Kyung Song, Mee Song	Identification of molecular signatures	Toxicology Letters	2012
PAHs	ACOT9	Mi-Kyung Song, Mee Song	Identification of molecular signatures	Toxicology Letters	2012
PAHs	ACTB	Mi-Kyung Song, Mee Song	Identification of molecular signatures	Toxicology Letters	2012
PAHs	ACTR1B	Mi-Kyung Song, Mee Song	Identification of molecular signatures	Toxicology Letters	2012
PAHs	ADAM17	Mi-Kyung Song, Mee Song	Identification of molecular signatures	Toxicology Letters	2012

PAHs	ADH5	Mi-Kyung Song, Mee Song	Identification of molecular signatures	Toxicology Letters	2012
PAHs	AGPAT3	Mi-Kyung Song, Mee Song	Identification of molecular signatures	Toxicology Letters	2012
PAHs	AHCYL1	Mi-Kyung Song, Mee Song	Identification of molecular signatures	Toxicology Letters	2012
PAHs	AKR1B10	Mi-Kyung Song, Mee Song	Identification of molecular signatures	Toxicology Letters	2012
PAHs	AKR1C1	Mi-Kyung Song, Mee Song	Identification of molecular signatures	Toxicology Letters	2012
PAHs	ALB	Mi-Kyung Song, Mee Song	Identification of molecular signatures	Toxicology Letters	2012
PAHs	ANKRD15	Mi-Kyung Song, Mee Song	Identification of molecular signatures	Toxicology Letters	2012
PAHs	ANKRD57	Mi-Kyung Song, Mee Song	Identification of molecular signatures	Toxicology Letters	2012
PAHs	ANP32B	Mi-Kyung Song, Mee Song	Identification of molecular signatures	Toxicology Letters	2012
PAHs	ANXA7	Mi-Kyung Song, Mee Song	Identification of molecular signatures	Toxicology Letters	2012
PAHs	AP1G1	Mi-Kyung Song, Mee Song	Identification of molecular signatures	Toxicology Letters	2012
PAHs	ARD1A	Mi-Kyung Song, Mee Song	Identification of molecular signatures	Toxicology Letters	2012
PAHs	ARIH2	Mi-Kyung Song, Mee Song	Identification of molecular signatures	Toxicology Letters	2012
PAHs	ASAHL	Mi-Kyung Song, Mee Song	Identification of molecular signatures	Toxicology Letters	2012
PAHs	ASCC1	Mi-Kyung Song, Mee Song	Identification of molecular signatures	Toxicology Letters	2012
PAHs	ASPM	Mi-Kyung Song, Mee Song	Identification of molecular signatures	Toxicology Letters	2012
PAHs	B4GALT5	Mi-Kyung Song, Mee Song	Identification of molecular signatures	Toxicology Letters	2012
PAHs	BACH1	Mi-Kyung Song, Mee Song	Identification of molecular signatures	Toxicology Letters	2012
PAHs	BRAP	Mi-Kyung Song, Mee Song	Identification of molecular signatures	Toxicology Letters	2012
PAHs	C10orf56	Mi-Kyung Song, Mee Song	Identification of molecular signatures	Toxicology Letters	2012
PAHs	C10orf6	Mi-Kyung Song, Mee Song	Identification of molecular signatures	Toxicology Letters	2012
PAHs	C11orf79	Mi-Kyung Song, Mee Song	Identification of molecular signatures	Toxicology Letters	2012
PAHs	C12orf47	Mi-Kyung Song, Mee Song	Identification of molecular signatures	Toxicology Letters	2012
PAHs	C12orf49	Mi-Kyung Song, Mee Song	Identification of molecular signatures	Toxicology Letters	2012
PAHs	C13orf15	Mi-Kyung Song, Mee Song	Identification of molecular signatures	Toxicology Letters	2012
PAHs	C14orf159	Mi-Kyung Song, Mee Song	Identification of molecular signatures	Toxicology Letters	2012
PAHs	C14orf24	Mi-Kyung Song, Mee Song	Identification of molecular signatures	Toxicology Letters	2012
PAHs	C16orf63	Mi-Kyung Song, Mee Song	Identification of molecular signatures	Toxicology Letters	2012
PAHs	C17orf44	Mi-Kyung Song, Mee Song	Identification of molecular signatures	Toxicology Letters	2012
PAHs	C17orf63	Mi-Kyung Song, Mee Song	Identification of molecular signatures	Toxicology Letters	2012
PAHs	C17orf85	Mi-Kyung Song, Mee Song	Identification of molecular signatures	Toxicology Letters	2012
PAHs	C19orf52	Mi-Kyung Song, Mee Song	Identification of molecular signatures	Toxicology Letters	2012
PAHs	C1orf112	Mi-Kyung Song, Mee Song	Identification of molecular signatures	Toxicology Letters	2012
PAHs	C1orf128	Mi-Kyung Song, Mee Song	Identification of molecular signatures	Toxicology Letters	2012
PAHs	C1orf85	Mi-Kyung Song, Mee Song	Identification of molecular signatures	Toxicology Letters	2012
PAHs	C22orf16	Mi-Kyung Song, Mee Song	Identification of molecular signatures	Toxicology Letters	2012
PAHs	C6orf145	Mi-Kyung Song, Mee Song	Identification of molecular signatures	Toxicology Letters	2012
PAHs	C9orf150	Mi-Kyung Song, Mee Song	Identification of molecular signatures	Toxicology Letters	2012
PAHs	C9orf89	Mi-Kyung Song, Mee Song	Identification of molecular signatures	Toxicology Letters	2012
PAHs	CALCOCO2	Mi-Kyung Song, Mee Song	Identification of molecular signatures	Toxicology Letters	2012
PAHs	CAPNS1	Mi-Kyung Song, Mee Song	Identification of molecular signatures	Toxicology Letters	2012
PAHs	CCDC95	Mi-Kyung Song, Mee Song	Identification of molecular signatures	Toxicology Letters	2012
PAHs	CCDC99	Mi-Kyung Song, Mee Song	Identification of molecular signatures	Toxicology Letters	2012
PAHs	CCNY	Mi-Kyung Song, Mee Song	Identification of molecular signatures	Toxicology Letters	2012
PAHs	CD46	Mi-Kyung Song, Mee Song	Identification of molecular signatures	Toxicology Letters	2012
PAHs	CD97	Mi-Kyung Song, Mee Song	Identification of molecular signatures	Toxicology Letters	2012
PAHs	CDC16	Mi-Kyung Song, Mee Song	Identification of molecular signatures	Toxicology Letters	2012
PAHs	CDC40	Mi-Kyung Song, Mee Song	Identification of molecular signatures	Toxicology Letters	2012
PAHs	CDC42EP4	Mi-Kyung Song, Mee Song	Identification of molecular signatures	Toxicology Letters	2012
PAHs	CDC42EP5	Mi-Kyung Song, Mee Song	Identification of molecular signatures	Toxicology Letters	2012
PAHs	CDC45L	Mi-Kyung Song, Mee Song	Identification of molecular signatures	Toxicology Letters	2012
PAHs	CDK5RAP2	Mi-Kyung Song, Mee Song	Identification of molecular signatures	Toxicology Letters	2012
PAHs	CEP55	Mi-Kyung Song, Mee Song	Identification of molecular signatures	Toxicology Letters	2012
PAHs	CFDP1	Mi-Kyung Song, Mee Song	Identification of molecular signatures	Toxicology Letters	2012
PAHs	CHRNB1	Mi-Kyung Song, Mee Song	Identification of molecular signatures	Toxicology Letters	2012
PAHs	CKS1B	Mi-Kyung Song, Mee Song	Identification of molecular signatures	Toxicology Letters	2012
PAHs	CLASP2	Mi-Kyung Song, Mee Song	Identification of molecular signatures	Toxicology Letters	2012
PAHs	CLP1	Mi-Kyung Song, Mee Song	Identification of molecular signatures	Toxicology Letters	2012
PAHs	CLPX	Mi-Kyung Song, Mee Song	Identification of molecular signatures	Toxicology Letters	2012
PAHs	CLTA	Mi-Kyung Song, Mee Song	Identification of molecular signatures	Toxicology Letters	2012
PAHs	COX17	Mi-Kyung Song, Mee Song	Identification of molecular signatures	Toxicology Letters	2012
PAHs	CPVL	Mi-Kyung Song, Mee Song	Identification of molecular signatures	Toxicology Letters	2012
PAHs	CRAMP1L	Mi-Kyung Song, Mee Song	Identification of molecular signatures	Toxicology Letters	2012
PAHs	CSDA	Mi-Kyung Song, Mee Song	Identification of molecular signatures	Toxicology Letters	2012
PAHs	CSNK2B	Mi-Kyung Song, Mee Song	Identification of molecular signatures	Toxicology Letters	2012
PAHs	CSTF1	Mi-Kyung Song, Mee Song	Identification of molecular signatures	Toxicology Letters	2012
PAHs	CTSH	Mi-Kyung Song, Mee Song	Identification of molecular signatures	Toxicology Letters	2012
PAHs	CYP2W1	Mi-Kyung Song, Mee Song	Identification of molecular signatures	Toxicology Letters	2012
PAHs	CYP4F8	Mi-Kyung Song, Mee Song	Identification of molecular signatures	Toxicology Letters	2012
PAHs	DAD1	Mi-Kyung Song, Mee Song	Identification of molecular signatures	Toxicology Letters	2012
PAHs	DARS	Mi-Kyung Song, Mee Song	Identification of molecular signatures	Toxicology Letters	2012
PAHs	DBF4	Mi-Kyung Song, Mee Song	Identification of molecular signatures	Toxicology Letters	2012
PAHs	DDX27	Mi-Kyung Song, Mee Song	Identification of molecular signatures	Toxicology Letters	2012
PAHs	DEPDC1	Mi-Kyung Song, Mee Song	Identification of molecular signatures	Toxicology Letters	2012
PAHs	DEPDC1B	Mi-Kyung Song, Mee Song	Identification of molecular signatures	Toxicology Letters	2012
PAHs	DHCR24	Mi-Kyung Song, Mee Song	Identification of molecular signatures	Toxicology Letters	2012
PAHs	DHCR7	Mi-Kyung Song, Mee Song	Identification of molecular signatures	Toxicology Letters	2012
PAHs	DHRS13	Mi-Kyung Song, Mee Song	Identification of molecular signatures	Toxicology Letters	2012
PAHs	DHRSX	Mi-Kyung Song, Mee Song	Identification of molecular signatures	Toxicology Letters	2012
PAHs	DNAJB9	Mi-Kyung Song, Mee Song	Identification of molecular signatures	Toxicology Letters	2012
PAHs	DNAJC16	Mi-Kyung Song, Mee Song	Identification of molecular signatures	Toxicology Letters	2012
PAHs	DNAJC4	Mi-Kyung Song, Mee Song	Identification of molecular signatures	Toxicology Letters	2012
PAHs	DPYSL2	Mi-Kyung Song, Mee Song	Identification of molecular signatures	Toxicology Letters	2012
PAHs	DYM	Mi-Kyung Song, Mee Song	Identification of molecular signatures	Toxicology Letters	2012
PAHs	EDC3	Mi-Kyung Song, Mee Song	Identification of molecular signatures	Toxicology Letters	2012
PAHs	EIF3S2	Mi-Kyung Song, Mee Song	Identification of molecular signatures	Toxicology Letters	2012
PAHs	EIF3S3	Mi-Kyung Song, Mee Song	Identification of molecular signatures	Toxicology Letters	2012
PAHs	EIF3S5	Mi-Kyung Song, Mee Song	Identification of molecular signatures	Toxicology Letters	2012
PAHs	EIF3S6	Mi-Kyung Song, Mee Song	Identification of molecular signatures	Toxicology Letters	2012

PAHs	ERRFI1	Mi-Kyung Song, Mee Song	Identification of molecular signatures	Toxicology Letters	2012
PAHs	FAM126A	Mi-Kyung Song, Mee Song	Identification of molecular signatures	Toxicology Letters	2012
PAHs	FAM134A	Mi-Kyung Song, Mee Song	Identification of molecular signatures	Toxicology Letters	2012
PAHs	FAM29A	Mi-Kyung Song, Mee Song	Identification of molecular signatures	Toxicology Letters	2012
PAHs	FAM44B	Mi-Kyung Song, Mee Song	Identification of molecular signatures	Toxicology Letters	2012
PAHs	FAM8A1	Mi-Kyung Song, Mee Song	Identification of molecular signatures	Toxicology Letters	2012
PAHs	FAM91A2	Mi-Kyung Song, Mee Song	Identification of molecular signatures	Toxicology Letters	2012
PAHs	FBXO17	Mi-Kyung Song, Mee Song	Identification of molecular signatures	Toxicology Letters	2012
PAHs	FBXO22	Mi-Kyung Song, Mee Song	Identification of molecular signatures	Toxicology Letters	2012
PAHs	FBXO31	Mi-Kyung Song, Mee Song	Identification of molecular signatures	Toxicology Letters	2012
PAHs	FBXW4	Mi-Kyung Song, Mee Song	Identification of molecular signatures	Toxicology Letters	2012
PAHs	FDPS	Mi-Kyung Song, Mee Song	Identification of molecular signatures	Toxicology Letters	2012
PAHs	FIS1	Mi-Kyung Song, Mee Song	Identification of molecular signatures	Toxicology Letters	2012
PAHs	FKBP1A	Mi-Kyung Song, Mee Song	Identification of molecular signatures	Toxicology Letters	2012
PAHs	GAPVD1	Mi-Kyung Song, Mee Song	Identification of molecular signatures	Toxicology Letters	2012
PAHs	GCLC	Mi-Kyung Song, Mee Song	Identification of molecular signatures	Toxicology Letters	2012
PAHs	GCLM	Mi-Kyung Song, Mee Song	Identification of molecular signatures	Toxicology Letters	2012
PAHs	GDI2	Mi-Kyung Song, Mee Song	Identification of molecular signatures	Toxicology Letters	2012
PAHs	GNG4	Mi-Kyung Song, Mee Song	Identification of molecular signatures	Toxicology Letters	2012
PAHs	GOLT1A	Mi-Kyung Song, Mee Song	Identification of molecular signatures	Toxicology Letters	2012
PAHs	GPAM	Mi-Kyung Song, Mee Song	Identification of molecular signatures	Toxicology Letters	2012
PAHs	GSN	Mi-Kyung Song, Mee Song	Identification of molecular signatures	Toxicology Letters	2012
PAHs	GSR	Mi-Kyung Song, Mee Song	Identification of molecular signatures	Toxicology Letters	2012
PAHs	GSTA2	Mi-Kyung Song, Mee Song	Identification of molecular signatures	Toxicology Letters	2012
PAHs	GSTA3	Mi-Kyung Song, Mee Song	Identification of molecular signatures	Toxicology Letters	2012
PAHs	GSTA4	Mi-Kyung Song, Mee Song	Identification of molecular signatures	Toxicology Letters	2012
PAHs	GTPBP1	Mi-Kyung Song, Mee Song	Identification of molecular signatures	Toxicology Letters	2012
PAHs	H2AFJ	Mi-Kyung Song, Mee Song	Identification of molecular signatures	Toxicology Letters	2012
PAHs	H2AFX	Mi-Kyung Song, Mee Song	Identification of molecular signatures	Toxicology Letters	2012
PAHs	H2AFZ	Mi-Kyung Song, Mee Song	Identification of molecular signatures	Toxicology Letters	2012
PAHs	HCN4	Mi-Kyung Song, Mee Song	Identification of molecular signatures	Toxicology Letters	2012
PAHs	HD	Mi-Kyung Song, Mee Song	Identification of molecular signatures	Toxicology Letters	2012
PAHs	HECTD1	Mi-Kyung Song, Mee Song	Identification of molecular signatures	Toxicology Letters	2012
PAHs	HIGD2A	Mi-Kyung Song, Mee Song	Identification of molecular signatures	Toxicology Letters	2012
PAHs	HIST1H2AG	Mi-Kyung Song, Mee Song	Identification of molecular signatures	Toxicology Letters	2012
PAHs	HIST2H4A	Mi-Kyung Song, Mee Song	Identification of molecular signatures	Toxicology Letters	2012
PAHs	HK2	Mi-Kyung Song, Mee Song	Identification of molecular signatures	Toxicology Letters	2012
PAHs	HLA-C	Mi-Kyung Song, Mee Song	Identification of molecular signatures	Toxicology Letters	2012
PAHs	HMGN1	Mi-Kyung Song, Mee Song	Identification of molecular signatures	Toxicology Letters	2012
PAHs	HNRPL	Mi-Kyung Song, Mee Song	Identification of molecular signatures	Toxicology Letters	2012
PAHs	HPN	Mi-Kyung Song, Mee Song	Identification of molecular signatures	Toxicology Letters	2012
PAHs	IER3	Mi-Kyung Song, Mee Song	Identification of molecular signatures	Toxicology Letters	2012
PAHs	IGF2BP2	Mi-Kyung Song, Mee Song	Identification of molecular signatures	Toxicology Letters	2012
PAHs	IKBKAP	Mi-Kyung Song, Mee Song	Identification of molecular signatures	Toxicology Letters	2012
PAHs	INTS12	Mi-Kyung Song, Mee Song	Identification of molecular signatures	Toxicology Letters	2012
PAHs	IRF2BP2	Mi-Kyung Song, Mee Song	Identification of molecular signatures	Toxicology Letters	2012
PAHs	ITCH	Mi-Kyung Song, Mee Song	Identification of molecular signatures	Toxicology Letters	2012
PAHs	ITFG3	Mi-Kyung Song, Mee Song	Identification of molecular signatures	Toxicology Letters	2012
PAHs	JAG1	Mi-Kyung Song, Mee Song	Identification of molecular signatures	Toxicology Letters	2012
PAHs	KHDRBS1	Mi-Kyung Song, Mee Song	Identification of molecular signatures	Toxicology Letters	2012
PAHs	KIAA1609	Mi-Kyung Song, Mee Song	Identification of molecular signatures	Toxicology Letters	2012
PAHs	KLB	Mi-Kyung Song, Mee Song	Identification of molecular signatures	Toxicology Letters	2012
PAHs	KLHDC2	Mi-Kyung Song, Mee Song	Identification of molecular signatures	Toxicology Letters	2012
PAHs	KYNU	Mi-Kyung Song, Mee Song	Identification of molecular signatures	Toxicology Letters	2012
PAHs	LASP1	Mi-Kyung Song, Mee Song	Identification of molecular signatures	Toxicology Letters	2012
PAHs	LASS6	Mi-Kyung Song, Mee Song	Identification of molecular signatures	Toxicology Letters	2012
PAHs	LEPRE1	Mi-Kyung Song, Mee Song	Identification of molecular signatures	Toxicology Letters	2012
PAHs	LGALS1	Mi-Kyung Song, Mee Song	Identification of molecular signatures	Toxicology Letters	2012
PAHs	LGALS2	Mi-Kyung Song, Mee Song	Identification of molecular signatures	Toxicology Letters	2012
PAHs	LHB	Mi-Kyung Song, Mee Song	Identification of molecular signatures	Toxicology Letters	2012
PAHs	LRP10	Mi-Kyung Song, Mee Song	Identification of molecular signatures	Toxicology Letters	2012
PAHs	LRRC51	Mi-Kyung Song, Mee Song	Identification of molecular signatures	Toxicology Letters	2012
PAHs	LRRC8D	Mi-Kyung Song, Mee Song	Identification of molecular signatures	Toxicology Letters	2012
PAHs	LSG1	Mi-Kyung Song, Mee Song	Identification of molecular signatures	Toxicology Letters	2012
PAHs	LSS	Mi-Kyung Song, Mee Song	Identification of molecular signatures	Toxicology Letters	2012
PAHs	LZIC	Mi-Kyung Song, Mee Song	Identification of molecular signatures	Toxicology Letters	2012
PAHs	MAFA	Mi-Kyung Song, Mee Song	Identification of molecular signatures	Toxicology Letters	2012
PAHs	MAOA	Mi-Kyung Song, Mee Song	Identification of molecular signatures	Toxicology Letters	2012
PAHs	MAOB	Mi-Kyung Song, Mee Song	Identification of molecular signatures	Toxicology Letters	2012
PAHs	MBD6	Mi-Kyung Song, Mee Song	Identification of molecular signatures	Toxicology Letters	2012
PAHs	METTL9	Mi-Kyung Song, Mee Song	Identification of molecular signatures	Toxicology Letters	2012
PAHs	MGEA5	Mi-Kyung Song, Mee Song	Identification of molecular signatures	Toxicology Letters	2012
PAHs	MIS12	Mi-Kyung Song, Mee Song	Identification of molecular signatures	Toxicology Letters	2012
PAHs	MLL2	Mi-Kyung Song, Mee Song	Identification of molecular signatures	Toxicology Letters	2012
PAHs	MLLT1	Mi-Kyung Song, Mee Song	Identification of molecular signatures	Toxicology Letters	2012
PAHs	MLYCD	Mi-Kyung Song, Mee Song	Identification of molecular signatures	Toxicology Letters	2012
PAHs	MMP15	Mi-Kyung Song, Mee Song	Identification of molecular signatures	Toxicology Letters	2012
PAHs	MRPL41	Mi-Kyung Song, Mee Song	Identification of molecular signatures	Toxicology Letters	2012
PAHs	MTRF1L	Mi-Kyung Song, Mee Song	Identification of molecular signatures	Toxicology Letters	2012
PAHs	MYH14	Mi-Kyung Song, Mee Song	Identification of molecular signatures	Toxicology Letters	2012
PAHs	MYL6B	Mi-Kyung Song, Mee Song	Identification of molecular signatures	Toxicology Letters	2012
PAHs	MYO1A	Mi-Kyung Song, Mee Song	Identification of molecular signatures	Toxicology Letters	2012
PAHs	MYOHD1	Mi-Kyung Song, Mee Song	Identification of molecular signatures	Toxicology Letters	2012
PAHs	NDC80	Mi-Kyung Song, Mee Song	Identification of molecular signatures	Toxicology Letters	2012
PAHs	NDST2	Mi-Kyung Song, Mee Song	Identification of molecular signatures	Toxicology Letters	2012
PAHs	NDUFA9	Mi-Kyung Song, Mee Song	Identification of molecular signatures	Toxicology Letters	2012
PAHs	NFE2L3	Mi-Kyung Song, Mee Song	Identification of molecular signatures	Toxicology Letters	2012
PAHs	NIF3L1	Mi-Kyung Song, Mee Song	Identification of molecular signatures	Toxicology Letters	2012
PAHs	NINJ1	Mi-Kyung Song, Mee Song	Identification of molecular signatures	Toxicology Letters	2012

PAHs	NIPSNAP3B	Mi-Kyung Song, Mee Song	Identification of molecular signatures	Toxicology Letters	2012
PAHs	NP	Mi-Kyung Song, Mee Song	Identification of molecular signatures	Toxicology Letters	2012
PAHs	NPEPPS	Mi-Kyung Song, Mee Song	Identification of molecular signatures	Toxicology Letters	2012
PAHs	NPM1	Mi-Kyung Song, Mee Song	Identification of molecular signatures	Toxicology Letters	2012
PAHs	NPTXR	Mi-Kyung Song, Mee Song	Identification of molecular signatures	Toxicology Letters	2012
PAHs	NSL1	Mi-Kyung Song, Mee Song	Identification of molecular signatures	Toxicology Letters	2012
PAHs	NSUN5	Mi-Kyung Song, Mee Song	Identification of molecular signatures	Toxicology Letters	2012
PAHs	NUP37	Mi-Kyung Song, Mee Song	Identification of molecular signatures	Toxicology Letters	2012
PAHs	NUPL2	Mi-Kyung Song, Mee Song	Identification of molecular signatures	Toxicology Letters	2012
PAHs	OTX1	Mi-Kyung Song, Mee Song	Identification of molecular signatures	Toxicology Letters	2012
PAHs	PAH	Mi-Kyung Song, Mee Song	Identification of molecular signatures	Toxicology Letters	2012
PAHs	PDCD6IP	Mi-Kyung Song, Mee Song	Identification of molecular signatures	Toxicology Letters	2012
PAHs	PDCL3	Mi-Kyung Song, Mee Song	Identification of molecular signatures	Toxicology Letters	2012
PAHs	PEG10	Mi-Kyung Song, Mee Song	Identification of molecular signatures	Toxicology Letters	2012
PAHs	PGLS	Mi-Kyung Song, Mee Song	Identification of molecular signatures	Toxicology Letters	2012
PAHs	PGRMC2	Mi-Kyung Song, Mee Song	Identification of molecular signatures	Toxicology Letters	2012
PAHs	PHF5A	Mi-Kyung Song, Mee Song	Identification of molecular signatures	Toxicology Letters	2012
PAHs	PHLDA1	Mi-Kyung Song, Mee Song	Identification of molecular signatures	Toxicology Letters	2012
PAHs	PIGW	Mi-Kyung Song, Mee Song	Identification of molecular signatures	Toxicology Letters	2012
PAHs	PIR	Mi-Kyung Song, Mee Song	Identification of molecular signatures	Toxicology Letters	2012
PAHs	PLAA	Mi-Kyung Song, Mee Song	Identification of molecular signatures	Toxicology Letters	2012
PAHs	PLK2	Mi-Kyung Song, Mee Song	Identification of molecular signatures	Toxicology Letters	2012
PAHs	POLR2L	Mi-Kyung Song, Mee Song	Identification of molecular signatures	Toxicology Letters	2012
PAHs	POP1	Mi-Kyung Song, Mee Song	Identification of molecular signatures	Toxicology Letters	2012
PAHs	PPID	Mi-Kyung Song, Mee Song	Identification of molecular signatures	Toxicology Letters	2012
PAHs	PPP1R11	Mi-Kyung Song, Mee Song	Identification of molecular signatures	Toxicology Letters	2012
PAHs	PRIM2A	Mi-Kyung Song, Mee Song	Identification of molecular signatures	Toxicology Letters	2012
PAHs	PRKRA	Mi-Kyung Song, Mee Song	Identification of molecular signatures	Toxicology Letters	2012
PAHs	PROC	Mi-Kyung Song, Mee Song	Identification of molecular signatures	Toxicology Letters	2012
PAHs	PRPF38A	Mi-Kyung Song, Mee Song	Identification of molecular signatures	Toxicology Letters	2012
PAHs	PRRG1	Mi-Kyung Song, Mee Song	Identification of molecular signatures	Toxicology Letters	2012
PAHs	PSMA1	Mi-Kyung Song, Mee Song	Identification of molecular signatures	Toxicology Letters	2012
PAHs	PSMA5	Mi-Kyung Song, Mee Song	Identification of molecular signatures	Toxicology Letters	2012
PAHs	PSMB1	Mi-Kyung Song, Mee Song	Identification of molecular signatures	Toxicology Letters	2012
PAHs	PSMB3	Mi-Kyung Song, Mee Song	Identification of molecular signatures	Toxicology Letters	2012
PAHs	PSMB5	Mi-Kyung Song, Mee Song	Identification of molecular signatures	Toxicology Letters	2012
PAHs	PSMC1	Mi-Kyung Song, Mee Song	Identification of molecular signatures	Toxicology Letters	2012
PAHs	PSMC2	Mi-Kyung Song, Mee Song	Identification of molecular signatures	Toxicology Letters	2012
PAHs	PSMD1	Mi-Kyung Song, Mee Song	Identification of molecular signatures	Toxicology Letters	2012
PAHs	PSMD4	Mi-Kyung Song, Mee Song	Identification of molecular signatures	Toxicology Letters	2012
PAHs	PSRC1	Mi-Kyung Song, Mee Song	Identification of molecular signatures	Toxicology Letters	2012
PAHs	PTOV1	Mi-Kyung Song, Mee Song	Identification of molecular signatures	Toxicology Letters	2012
PAHs	PTPRH	Mi-Kyung Song, Mee Song	Identification of molecular signatures	Toxicology Letters	2012
PAHs	PYCR2	Mi-Kyung Song, Mee Song	Identification of molecular signatures	Toxicology Letters	2012
PAHs	RAB10	Mi-Kyung Song, Mee Song	Identification of molecular signatures	Toxicology Letters	2012
PAHs	RAB2B	Mi-Kyung Song, Mee Song	Identification of molecular signatures	Toxicology Letters	2012
PAHs	RAB6IP1	Mi-Kyung Song, Mee Song	Identification of molecular signatures	Toxicology Letters	2012
PAHs	RACGAP1	Mi-Kyung Song, Mee Song	Identification of molecular signatures	Toxicology Letters	2012
PAHs	RAI14	Mi-Kyung Song, Mee Song	Identification of molecular signatures	Toxicology Letters	2012
PAHs	RARRES2	Mi-Kyung Song, Mee Song	Identification of molecular signatures	Toxicology Letters	2012
PAHs	RARS2	Mi-Kyung Song, Mee Song	Identification of molecular signatures	Toxicology Letters	2012
PAHs	RASSF4	Mi-Kyung Song, Mee Song	Identification of molecular signatures	Toxicology Letters	2012
PAHs	RBM24	Mi-Kyung Song, Mee Song	Identification of molecular signatures	Toxicology Letters	2012
PAHs	RHOC	Mi-Kyung Song, Mee Song	Identification of molecular signatures	Toxicology Letters	2012
PAHs	RKHD1	Mi-Kyung Song, Mee Song	Identification of molecular signatures	Toxicology Letters	2012
PAHs	RNASE4	Mi-Kyung Song, Mee Song	Identification of molecular signatures	Toxicology Letters	2012
PAHs	RNF24	Mi-Kyung Song, Mee Song	Identification of molecular signatures	Toxicology Letters	2012
PAHs	RPL28	Mi-Kyung Song, Mee Song	Identification of molecular signatures	Toxicology Letters	2012
PAHs	RPL39	Mi-Kyung Song, Mee Song	Identification of molecular signatures	Toxicology Letters	2012
PAHs	RPS4X	Mi-Kyung Song, Mee Song	Identification of molecular signatures	Toxicology Letters	2012
PAHs	RPSAP15	Mi-Kyung Song, Mee Song	Identification of molecular signatures	Toxicology Letters	2012
PAHs	RRAS2	Mi-Kyung Song, Mee Song	Identification of molecular signatures	Toxicology Letters	2012
PAHs	RRM2	Mi-Kyung Song, Mee Song	Identification of molecular signatures	Toxicology Letters	2012
PAHs	RSL1D1	Mi-Kyung Song, Mee Song	Identification of molecular signatures	Toxicology Letters	2012
PAHs	RTF1	Mi-Kyung Song, Mee Song	Identification of molecular signatures	Toxicology Letters	2012
PAHs	RYBP	Mi-Kyung Song, Mee Song	Identification of molecular signatures	Toxicology Letters	2012
PAHs	S100A13	Mi-Kyung Song, Mee Song	Identification of molecular signatures	Toxicology Letters	2012
PAHs	SAAL1	Mi-Kyung Song, Mee Song	Identification of molecular signatures	Toxicology Letters	2012
PAHs	SDF2	Mi-Kyung Song, Mee Song	Identification of molecular signatures	Toxicology Letters	2012
PAHs	SDHB	Mi-Kyung Song, Mee Song	Identification of molecular signatures	Toxicology Letters	2012
PAHs	SEC61A2	Mi-Kyung Song, Mee Song	Identification of molecular signatures	Toxicology Letters	2012
PAHs	SELENBP1	Mi-Kyung Song, Mee Song	Identification of molecular signatures	Toxicology Letters	2012
PAHs	SF3B3	Mi-Kyung Song, Mee Song	Identification of molecular signatures	Toxicology Letters	2012
PAHs	SFRS1	Mi-Kyung Song, Mee Song	Identification of molecular signatures	Toxicology Letters	2012
PAHs	SFRS3	Mi-Kyung Song, Mee Song	Identification of molecular signatures	Toxicology Letters	2012
PAHs	SFRS7	Mi-Kyung Song, Mee Song	Identification of molecular signatures	Toxicology Letters	2012
PAHs	SFT2D1	Mi-Kyung Song, Mee Song	Identification of molecular signatures	Toxicology Letters	2012
PAHs	SLAIN2	Mi-Kyung Song, Mee Song	Identification of molecular signatures	Toxicology Letters	2012
PAHs	SLC38A1	Mi-Kyung Song, Mee Song	Identification of molecular signatures	Toxicology Letters	2012
PAHs	SLC39A11	Mi-Kyung Song, Mee Song	Identification of molecular signatures	Toxicology Letters	2012
PAHs	SLC46A1	Mi-Kyung Song, Mee Song	Identification of molecular signatures	Toxicology Letters	2012
PAHs	SMARCA1	Mi-Kyung Song, Mee Song	Identification of molecular signatures	Toxicology Letters	2012
PAHs	SNF1LK2	Mi-Kyung Song, Mee Song	Identification of molecular signatures	Toxicology Letters	2012
PAHs	SNF8	Mi-Kyung Song, Mee Song	Identification of molecular signatures	Toxicology Letters	2012
PAHs	SNX10	Mi-Kyung Song, Mee Song	Identification of molecular signatures	Toxicology Letters	2012
PAHs	SNX5	Mi-Kyung Song, Mee Song	Identification of molecular signatures	Toxicology Letters	2012
PAHs	SNX8	Mi-Kyung Song, Mee Song	Identification of molecular signatures	Toxicology Letters	2012
PAHs	SOX9	Mi-Kyung Song, Mee Song	Identification of molecular signatures	Toxicology Letters	2012
PAHs	SP5	Mi-Kyung Song, Mee Song	Identification of molecular signatures	Toxicology Letters	2012

PAHs	SPCS3	Mi-Kyung Song, Mee Song	Identification of molecular signatures	Toxicology Letters	2012
PAHs	SPON2	Mi-Kyung Song, Mee Song	Identification of molecular signatures	Toxicology Letters	2012
PAHs	SRGAP2	Mi-Kyung Song, Mee Song	Identification of molecular signatures	Toxicology Letters	2012
PAHs	STEAP1	Mi-Kyung Song, Mee Song	Identification of molecular signatures	Toxicology Letters	2012
PAHs	STMN1	Mi-Kyung Song, Mee Song	Identification of molecular signatures	Toxicology Letters	2012
PAHs	STRA13	Mi-Kyung Song, Mee Song	Identification of molecular signatures	Toxicology Letters	2012
PAHs	STRBP	Mi-Kyung Song, Mee Song	Identification of molecular signatures	Toxicology Letters	2012
PAHs	STX5	Mi-Kyung Song, Mee Song	Identification of molecular signatures	Toxicology Letters	2012
PAHs	SUCLA2	Mi-Kyung Song, Mee Song	Identification of molecular signatures	Toxicology Letters	2012
PAHs	TAF10	Mi-Kyung Song, Mee Song	Identification of molecular signatures	Toxicology Letters	2012
PAHs	TAF9B	Mi-Kyung Song, Mee Song	Identification of molecular signatures	Toxicology Letters	2012
PAHs	TATDN1	Mi-Kyung Song, Mee Song	Identification of molecular signatures	Toxicology Letters	2012
PAHs	TBC1D13	Mi-Kyung Song, Mee Song	Identification of molecular signatures	Toxicology Letters	2012
PAHs	TBC1D3	Mi-Kyung Song, Mee Song	Identification of molecular signatures	Toxicology Letters	2012
PAHs	TCEB2	Mi-Kyung Song, Mee Song	Identification of molecular signatures	Toxicology Letters	2012
PAHs	TCFL5	Mi-Kyung Song, Mee Song	Identification of molecular signatures	Toxicology Letters	2012
PAHs	TEGT	Mi-Kyung Song, Mee Song	Identification of molecular signatures	Toxicology Letters	2012
PAHs	THRAP4	Mi-Kyung Song, Mee Song	Identification of molecular signatures	Toxicology Letters	2012
PAHs	TICAM1	Mi-Kyung Song, Mee Song	Identification of molecular signatures	Toxicology Letters	2012
PAHs	TIMP1	Mi-Kyung Song, Mee Song	Identification of molecular signatures	Toxicology Letters	2012
PAHs	TMEM126B	Mi-Kyung Song, Mee Song	Identification of molecular signatures	Toxicology Letters	2012
PAHs	TMEM136	Mi-Kyung Song, Mee Song	Identification of molecular signatures	Toxicology Letters	2012
PAHs	TMEM2	Mi-Kyung Song, Mee Song	Identification of molecular signatures	Toxicology Letters	2012
PAHs	TMEM32	Mi-Kyung Song, Mee Song	Identification of molecular signatures	Toxicology Letters	2012
PAHs	TMEM48	Mi-Kyung Song, Mee Song	Identification of molecular signatures	Toxicology Letters	2012
PAHs	TMEM5	Mi-Kyung Song, Mee Song	Identification of molecular signatures	Toxicology Letters	2012
PAHs	TMEM9B	Mi-Kyung Song, Mee Song	Identification of molecular signatures	Toxicology Letters	2012
PAHs	TNFAIP3	Mi-Kyung Song, Mee Song	Identification of molecular signatures	Toxicology Letters	2012
PAHs	TNNC1	Mi-Kyung Song, Mee Song	Identification of molecular signatures	Toxicology Letters	2012
PAHs	TOLLIP	Mi-Kyung Song, Mee Song	Identification of molecular signatures	Toxicology Letters	2012
PAHs	TPMT	Mi-Kyung Song, Mee Song	Identification of molecular signatures	Toxicology Letters	2012
PAHs	TRAM2	Mi-Kyung Song, Mee Song	Identification of molecular signatures	Toxicology Letters	2012
PAHs	TRAPPC3	Mi-Kyung Song, Mee Song	Identification of molecular signatures	Toxicology Letters	2012
PAHs	TTC3	Mi-Kyung Song, Mee Song	Identification of molecular signatures	Toxicology Letters	2012
PAHs	TUBA3C	Mi-Kyung Song, Mee Song	Identification of molecular signatures	Toxicology Letters	2012
PAHs	TUFM	Mi-Kyung Song, Mee Song	Identification of molecular signatures	Toxicology Letters	2012
PAHs	TUSC2	Mi-Kyung Song, Mee Song	Identification of molecular signatures	Toxicology Letters	2012
PAHs	TXNDC14	Mi-Kyung Song, Mee Song	Identification of molecular signatures	Toxicology Letters	2012
PAHs	UBE2G1	Mi-Kyung Song, Mee Song	Identification of molecular signatures	Toxicology Letters	2012
PAHs	UPB1	Mi-Kyung Song, Mee Song	Identification of molecular signatures	Toxicology Letters	2012
PAHs	USP1	Mi-Kyung Song, Mee Song	Identification of molecular signatures	Toxicology Letters	2012
PAHs	UTP6	Mi-Kyung Song, Mee Song	Identification of molecular signatures	Toxicology Letters	2012
PAHs	VASP	Mi-Kyung Song, Mee Song	Identification of molecular signatures	Toxicology Letters	2012
PAHs	VIL1	Mi-Kyung Song, Mee Song	Identification of molecular signatures	Toxicology Letters	2012
PAHs	VSIG2	Mi-Kyung Song, Mee Song	Identification of molecular signatures	Toxicology Letters	2012
PAHs	VTI1B	Mi-Kyung Song, Mee Song	Identification of molecular signatures	Toxicology Letters	2012
PAHs	WDR45	Mi-Kyung Song, Mee Song	Identification of molecular signatures	Toxicology Letters	2012
PAHs	WDR81	Mi-Kyung Song, Mee Song	Identification of molecular signatures	Toxicology Letters	2012
PAHs	YIPF3	Mi-Kyung Song, Mee Song	Identification of molecular signatures	Toxicology Letters	2012
PAHs	YWHAH	Mi-Kyung Song, Mee Song	Identification of molecular signatures	Toxicology Letters	2012
PAHs	ZCCHC6	Mi-Kyung Song, Mee Song	Identification of molecular signatures	Toxicology Letters	2012
PAHs	ZFAND2A	Mi-Kyung Song, Mee Song	Identification of molecular signatures	Toxicology Letters	2012
PAHs	ZNF709	Mi-Kyung Song, Mee Song	Identification of molecular signatures	Toxicology Letters	2012
PAHs	ZW10	Mi-Kyung Song, Mee Song	Identification of molecular signatures	Toxicology Letters	2012
PAHs	NPTX1	Helena Líbalová, Kateřina	Global gene expression changes in human	Particle and Fibre Toxicology	2012
PAHs	BMF	Helena Líbalová, Kateřina	Global gene expression changes in human	Particle and Fibre Toxicology	2012
PAHs	GPR56	Helena Líbalová, Kateřina	Global gene expression changes in human	Particle and Fibre Toxicology	2012
PAHs	ANTXR1	Helena Líbalová, Kateřina	Global gene expression changes in human	Particle and Fibre Toxicology	2012
PAHs	PHYH	Helena Líbalová, Kateřina	Global gene expression changes in human	Particle and Fibre Toxicology	2012
PAHs	CTSL1	Helena Líbalová, Kateřina	Global gene expression changes in human	Particle and Fibre Toxicology	2012
PAHs	DNAJC12	Helena Líbalová, Kateřina	Global gene expression changes in human	Particle and Fibre Toxicology	2012
PAHs	HAGHL	Helena Líbalová, Kateřina	Global gene expression changes in human	Particle and Fibre Toxicology	2012
PAHs	PTGS2	Helena Líbalová, Kateřina	Global gene expression changes in human	Particle and Fibre Toxicology	2012
PAHs	ABCC4	Helena Líbalová, Kateřina	Global gene expression changes in human	Particle and Fibre Toxicology	2012
PAHs	ENPP2	Helena Líbalová, Kateřina	Global gene expression changes in human	Particle and Fibre Toxicology	2012
PAHs	GK	Helena Líbalová, Kateřina	Global gene expression changes in human	Particle and Fibre Toxicology	2012
PAHs	GREM1	Helena Líbalová, Kateřina	Global gene expression changes in human	Particle and Fibre Toxicology	2012
PAHs	HNMT	Helena Líbalová, Kateřina	Global gene expression changes in human	Particle and Fibre Toxicology	2012
PAHs	FBXL5	Helena Líbalová, Kateřina	Global gene expression changes in human	Particle and Fibre Toxicology	2012
PAHs	MGST1	Helena Líbalová, Kateřina	Global gene expression changes in human	Particle and Fibre Toxicology	2012
PAHs	PIR	Helena Líbalová, Kateřina	Global gene expression changes in human	Particle and Fibre Toxicology	2012
PAHs	CRIP1	Helena Líbalová, Kateřina	Global gene expression changes in human	Particle and Fibre Toxicology	2012
PAHs	CYP1B1	Helena Líbalová, Kateřina	Global gene expression changes in human	Particle and Fibre Toxicology	2012
PAHs	KDELR3	Helena Líbalová, Kateřina	Global gene expression changes in human	Particle and Fibre Toxicology	2012
PAHs	KIAA1644	Helena Líbalová, Kateřina	Global gene expression changes in human	Particle and Fibre Toxicology	2012
PAHs	MTSS1	Helena Líbalová, Kateřina	Global gene expression changes in human	Particle and Fibre Toxicology	2012
PAHs	NKD2	Helena Líbalová, Kateřina	Global gene expression changes in human	Particle and Fibre Toxicology	2012
PAHs	NQO1	Helena Líbalová, Kateřina	Global gene expression changes in human	Particle and Fibre Toxicology	2012
PAHs	OSAP	Helena Líbalová, Kateřina	Global gene expression changes in human	Particle and Fibre Toxicology	2012
PAHs	SLC6A15	Helena Líbalová, Kateřina	Global gene expression changes in human	Particle and Fibre Toxicology	2012
PAHs	SNCA	Helena Líbalová, Kateřina	Global gene expression changes in human	Particle and Fibre Toxicology	2012
PAHs	TBXAS1	Helena Líbalová, Kateřina	Global gene expression changes in human	Particle and Fibre Toxicology	2012
PAHs	TIPARP	Helena Líbalová, Kateřina	Global gene expression changes in human	Particle and Fibre Toxicology	2012
PAHs	TUBA1A	Helena Líbalová, Kateřina	Global gene expression changes in human	Particle and Fibre Toxicology	2012
PAHs	BMP2	Helena Líbalová, Kateřina	Global gene expression changes in human	Particle and Fibre Toxicology	2012
PAHs	C17orf68	Helena Líbalová, Kateřina	Global gene expression changes in human	Particle and Fibre Toxicology	2012
PAHs	HSD17B14	Helena Líbalová, Kateřina	Global gene expression changes in human	Particle and Fibre Toxicology	2012
PAHs	MAFG	Helena Líbalová, Kateřina	Global gene expression changes in human	Particle and Fibre Toxicology	2012
PAHs	MAPK13	Helena Líbalová, Kateřina	Global gene expression changes in human	Particle and Fibre Toxicology	2012

PAHs	PTGR1	Helena Líbalová, Kateřina	Global gene expression changes in human	Particle and Fibre Toxicology	2012
PAHs	RENBP	Helena Líbalová, Kateřina	Global gene expression changes in human	Particle and Fibre Toxicology	2012
PAHs	RGNEF	Helena Líbalová, Kateřina	Global gene expression changes in human	Particle and Fibre Toxicology	2012
PAHs	SOCS2	Helena Líbalová, Kateřina	Global gene expression changes in human	Particle and Fibre Toxicology	2012
PAHs	STK32B	Helena Líbalová, Kateřina	Global gene expression changes in human	Particle and Fibre Toxicology	2012
PAHs	VEGFB	Helena Líbalová, Kateřina	Global gene expression changes in human	Particle and Fibre Toxicology	2012
PAHs	WARS	Helena Líbalová, Kateřina	Global gene expression changes in human	Particle and Fibre Toxicology	2012
PAHs	ZMYND8	Helena Líbalová, Kateřina	Global gene expression changes in human	Particle and Fibre Toxicology	2012
PAHs	ABCC3	Helena Líbalová, Kateřina	Global gene expression changes in human	Particle and Fibre Toxicology	2012
PAHs	AKR1B1	Helena Líbalová, Kateřina	Global gene expression changes in human	Particle and Fibre Toxicology	2012
PAHs	ALAS1	Helena Líbalová, Kateřina	Global gene expression changes in human	Particle and Fibre Toxicology	2012
PAHs	ARRB1	Helena Líbalová, Kateřina	Global gene expression changes in human	Particle and Fibre Toxicology	2012
PAHs	BIN1	Helena Líbalová, Kateřina	Global gene expression changes in human	Particle and Fibre Toxicology	2012
PAHs	CLN8	Helena Líbalová, Kateřina	Global gene expression changes in human	Particle and Fibre Toxicology	2012
PAHs	EEPD1	Helena Líbalová, Kateřina	Global gene expression changes in human	Particle and Fibre Toxicology	2012
PAHs	GSTO1	Helena Líbalová, Kateřina	Global gene expression changes in human	Particle and Fibre Toxicology	2012
PAHs	HVCN1	Helena Líbalová, Kateřina	Global gene expression changes in human	Particle and Fibre Toxicology	2012
PAHs	ITGB5	Helena Líbalová, Kateřina	Global gene expression changes in human	Particle and Fibre Toxicology	2012
PAHs	KEAP1	Helena Líbalová, Kateřina	Global gene expression changes in human	Particle and Fibre Toxicology	2012
PAHs	LAMA1	Helena Líbalová, Kateřina	Global gene expression changes in human	Particle and Fibre Toxicology	2012
PAHs	LIF	Helena Líbalová, Kateřina	Global gene expression changes in human	Particle and Fibre Toxicology	2012
PAHs	METRNL	Helena Líbalová, Kateřina	Global gene expression changes in human	Particle and Fibre Toxicology	2012
PAHs	PGM5	Helena Líbalová, Kateřina	Global gene expression changes in human	Particle and Fibre Toxicology	2012
PAHs	STAT1	Helena Líbalová, Kateřina	Global gene expression changes in human	Particle and Fibre Toxicology	2012
PAHs	TOM1	Helena Líbalová, Kateřina	Global gene expression changes in human	Particle and Fibre Toxicology	2012
PAHs	TXN	Helena Líbalová, Kateřina	Global gene expression changes in human	Particle and Fibre Toxicology	2012
PAHs	TYSND1	Helena Líbalová, Kateřina	Global gene expression changes in human	Particle and Fibre Toxicology	2012
PAHs	ADFP	Helena Líbalová, Kateřina	Global gene expression changes in human	Particle and Fibre Toxicology	2012
PAHs	C14orf93	Helena Líbalová, Kateřina	Global gene expression changes in human	Particle and Fibre Toxicology	2012
PAHs	C6orf1	Helena Líbalová, Kateřina	Global gene expression changes in human	Particle and Fibre Toxicology	2012
PAHs	CHKA	Helena Líbalová, Kateřina	Global gene expression changes in human	Particle and Fibre Toxicology	2012
PAHs	CLN3	Helena Líbalová, Kateřina	Global gene expression changes in human	Particle and Fibre Toxicology	2012
PAHs	COL13A1	Helena Líbalová, Kateřina	Global gene expression changes in human	Particle and Fibre Toxicology	2012
PAHs	CTNS	Helena Líbalová, Kateřina	Global gene expression changes in human	Particle and Fibre Toxicology	2012
PAHs	ELOVL6	Helena Líbalová, Kateřina	Global gene expression changes in human	Particle and Fibre Toxicology	2012
PAHs	FBLN5	Helena Líbalová, Kateřina	Global gene expression changes in human	Particle and Fibre Toxicology	2012
PAHs	HABP4	Helena Líbalová, Kateřina	Global gene expression changes in human	Particle and Fibre Toxicology	2012
PAHs	HK2	Helena Líbalová, Kateřina	Global gene expression changes in human	Particle and Fibre Toxicology	2012
PAHs	HSD11B1	Helena Líbalová, Kateřina	Global gene expression changes in human	Particle and Fibre Toxicology	2012
PAHs	KIF11	Helena Líbalová, Kateřina	Global gene expression changes in human	Particle and Fibre Toxicology	2012
PAHs	LGR6	Helena Líbalová, Kateřina	Global gene expression changes in human	Particle and Fibre Toxicology	2012
PAHs	NAMPT	Helena Líbalová, Kateřina	Global gene expression changes in human	Particle and Fibre Toxicology	2012
PAHs	PAG1	Helena Líbalová, Kateřina	Global gene expression changes in human	Particle and Fibre Toxicology	2012
PAHs	PGK1	Helena Líbalová, Kateřina	Global gene expression changes in human	Particle and Fibre Toxicology	2012
PAHs	PSME3	Helena Líbalová, Kateřina	Global gene expression changes in human	Particle and Fibre Toxicology	2012
PAHs	SLMO1	Helena Líbalová, Kateřina	Global gene expression changes in human	Particle and Fibre Toxicology	2012
PAHs	SP110	Helena Líbalová, Kateřina	Global gene expression changes in human	Particle and Fibre Toxicology	2012
PAHs	SRI	Helena Líbalová, Kateřina	Global gene expression changes in human	Particle and Fibre Toxicology	2012
PAHs	TXNRD1	Helena Líbalová, Kateřina	Global gene expression changes in human	Particle and Fibre Toxicology	2012
PAHs	VWCE	Helena Líbalová, Kateřina	Global gene expression changes in human	Particle and Fibre Toxicology	2012
PAHs	ABCB9	Helena Líbalová, Kateřina	Global gene expression changes in human	Particle and Fibre Toxicology	2012
PAHs	AGT	Helena Líbalová, Kateřina	Global gene expression changes in human	Particle and Fibre Toxicology	2012
PAHs	ALG14	Helena Líbalová, Kateřina	Global gene expression changes in human	Particle and Fibre Toxicology	2012
PAHs	C1orf2	Helena Líbalová, Kateřina	Global gene expression changes in human	Particle and Fibre Toxicology	2012
PAHs	CCND2	Helena Líbalová, Kateřina	Global gene expression changes in human	Particle and Fibre Toxicology	2012
PAHs	CDC14B	Helena Líbalová, Kateřina	Global gene expression changes in human	Particle and Fibre Toxicology	2012
PAHs	CDK5RAP2	Helena Líbalová, Kateřina	Global gene expression changes in human	Particle and Fibre Toxicology	2012
PAHs	CISD1	Helena Líbalová, Kateřina	Global gene expression changes in human	Particle and Fibre Toxicology	2012
PAHs	CPEB1	Helena Líbalová, Kateřina	Global gene expression changes in human	Particle and Fibre Toxicology	2012
PAHs	DHX29	Helena Líbalová, Kateřina	Global gene expression changes in human	Particle and Fibre Toxicology	2012
PAHs	DIAPH3	Helena Líbalová, Kateřina	Global gene expression changes in human	Particle and Fibre Toxicology	2012
PAHs	FBXO32	Helena Líbalová, Kateřina	Global gene expression changes in human	Particle and Fibre Toxicology	2012
PAHs	FMO3	Helena Líbalová, Kateřina	Global gene expression changes in human	Particle and Fibre Toxicology	2012
PAHs	FOXQ1	Helena Líbalová, Kateřina	Global gene expression changes in human	Particle and Fibre Toxicology	2012
PAHs	FTHL11	Helena Líbalová, Kateřina	Global gene expression changes in human	Particle and Fibre Toxicology	2012
PAHs	FVT1	Helena Líbalová, Kateřina	Global gene expression changes in human	Particle and Fibre Toxicology	2012
PAHs	GABARAPL1	Helena Líbalová, Kateřina	Global gene expression changes in human	Particle and Fibre Toxicology	2012
PAHs	GALNTL1	Helena Líbalová, Kateřina	Global gene expression changes in human	Particle and Fibre Toxicology	2012
PAHs	GCLM	Helena Líbalová, Kateřina	Global gene expression changes in human	Particle and Fibre Toxicology	2012
PAHs	GPR68	Helena Líbalová, Kateřina	Global gene expression changes in human	Particle and Fibre Toxicology	2012
PAHs	GPX3	Helena Líbalová, Kateřina	Global gene expression changes in human	Particle and Fibre Toxicology	2012
PAHs	HTR2B	Helena Líbalová, Kateřina	Global gene expression changes in human	Particle and Fibre Toxicology	2012
PAHs	ID2	Helena Líbalová, Kateřina	Global gene expression changes in human	Particle and Fibre Toxicology	2012
PAHs	KCNF1	Helena Líbalová, Kateřina	Global gene expression changes in human	Particle and Fibre Toxicology	2012
PAHs	MAGED1	Helena Líbalová, Kateřina	Global gene expression changes in human	Particle and Fibre Toxicology	2012
PAHs	MGAT2	Helena Líbalová, Kateřina	Global gene expression changes in human	Particle and Fibre Toxicology	2012
PAHs	MMP10	Helena Líbalová, Kateřina	Global gene expression changes in human	Particle and Fibre Toxicology	2012
PAHs	MMP3	Helena Líbalová, Kateřina	Global gene expression changes in human	Particle and Fibre Toxicology	2012
PAHs	MTUS1	Helena Líbalová, Kateřina	Global gene expression changes in human	Particle and Fibre Toxicology	2012
PAHs	NOV	Helena Líbalová, Kateřina	Global gene expression changes in human	Particle and Fibre Toxicology	2012
PAHs	OKL38	Helena Líbalová, Kateřina	Global gene expression changes in human	Particle and Fibre Toxicology	2012
PAHs	OXCT2	Helena Líbalová, Kateřina	Global gene expression changes in human	Particle and Fibre Toxicology	2012
PAHs	POPDC3	Helena Líbalová, Kateřina	Global gene expression changes in human	Particle and Fibre Toxicology	2012
PAHs	SERP2	Helena Líbalová, Kateřina	Global gene expression changes in human	Particle and Fibre Toxicology	2012
PAHs	SLC16A6	Helena Líbalová, Kateřina	Global gene expression changes in human	Particle and Fibre Toxicology	2012
PAHs	SLFN11	Helena Líbalová, Kateřina	Global gene expression changes in human	Particle and Fibre Toxicology	2012
PAHs	SQRDL	Helena Líbalová, Kateřina	Global gene expression changes in human	Particle and Fibre Toxicology	2012
PAHs	SSBP3	Helena Líbalová, Kateřina	Global gene expression changes in human	Particle and Fibre Toxicology	2012
PAHs	STX3	Helena Líbalová, Kateřina	Global gene expression changes in human	Particle and Fibre Toxicology	2012

PAHs	TFPI	Helena Líbalová, Kateřina	Global gene expression changes in human	Particle and Fibre Toxicology	2012
PAHs	TMEM45A	Helena Líbalová, Kateřina	Global gene expression changes in human	Particle and Fibre Toxicology	2012
PAHs	TXNIP	Helena Líbalová, Kateřina	Global gene expression changes in human	Particle and Fibre Toxicology	2012
PAHs	VWF	Helena Líbalová, Kateřina	Global gene expression changes in human	Particle and Fibre Toxicology	2012
PAHs	ZFP91	Helena Líbalová, Kateřina	Global gene expression changes in human	Particle and Fibre Toxicology	2012
PAHs	ZNF207	Helena Líbalová, Kateřina	Global gene expression changes in human	Particle and Fibre Toxicology	2012
PAHs	ACTA2	Helena Líbalová, Kateřina	Global gene expression changes in human	Particle and Fibre Toxicology	2012
PAHs	AKR1B10	Helena Líbalová, Kateřina	Global gene expression changes in human	Particle and Fibre Toxicology	2012
PAHs	ANXA6	Helena Líbalová, Kateřina	Global gene expression changes in human	Particle and Fibre Toxicology	2012
PAHs	C14orf43	Helena Líbalová, Kateřina	Global gene expression changes in human	Particle and Fibre Toxicology	2012
PAHs	CABYR	Helena Líbalová, Kateřina	Global gene expression changes in human	Particle and Fibre Toxicology	2012
PAHs	CD9	Helena Líbalová, Kateřina	Global gene expression changes in human	Particle and Fibre Toxicology	2012
PAHs	CDKL3	Helena Líbalová, Kateřina	Global gene expression changes in human	Particle and Fibre Toxicology	2012
PAHs	CSRP1	Helena Líbalová, Kateřina	Global gene expression changes in human	Particle and Fibre Toxicology	2012
PAHs	CSTF3	Helena Líbalová, Kateřina	Global gene expression changes in human	Particle and Fibre Toxicology	2012
PAHs	DGCR8	Helena Líbalová, Kateřina	Global gene expression changes in human	Particle and Fibre Toxicology	2012
PAHs	DPP7	Helena Líbalová, Kateřina	Global gene expression changes in human	Particle and Fibre Toxicology	2012
PAHs	DUSP3	Helena Líbalová, Kateřina	Global gene expression changes in human	Particle and Fibre Toxicology	2012
PAHs	EWSR1	Helena Líbalová, Kateřina	Global gene expression changes in human	Particle and Fibre Toxicology	2012
PAHs	FLJ46906	Helena Líbalová, Kateřina	Global gene expression changes in human	Particle and Fibre Toxicology	2012
PAHs	GPER	Helena Líbalová, Kateřina	Global gene expression changes in human	Particle and Fibre Toxicology	2012
PAHs	HTATIP2	Helena Líbalová, Kateřina	Global gene expression changes in human	Particle and Fibre Toxicology	2012
PAHs	IL4R	Helena Líbalová, Kateřina	Global gene expression changes in human	Particle and Fibre Toxicology	2012
PAHs	ITPR3	Helena Líbalová, Kateřina	Global gene expression changes in human	Particle and Fibre Toxicology	2012
PAHs	JAG2	Helena Líbalová, Kateřina	Global gene expression changes in human	Particle and Fibre Toxicology	2012
PAHs	KAZALD1	Helena Líbalová, Kateřina	Global gene expression changes in human	Particle and Fibre Toxicology	2012
PAHs	KBTBD11	Helena Líbalová, Kateřina	Global gene expression changes in human	Particle and Fibre Toxicology	2012
PAHs	KYNU	Helena Líbalová, Kateřina	Global gene expression changes in human	Particle and Fibre Toxicology	2012
PAHs	LEF1	Helena Líbalová, Kateřina	Global gene expression changes in human	Particle and Fibre Toxicology	2012
PAHs	LOC346887	Helena Líbalová, Kateřina	Global gene expression changes in human	Particle and Fibre Toxicology	2012
PAHs	LYL1	Helena Líbalová, Kateřina	Global gene expression changes in human	Particle and Fibre Toxicology	2012
PAHs	MMP1	Helena Líbalová, Kateřina	Global gene expression changes in human	Particle and Fibre Toxicology	2012
PAHs	OLR1	Helena Líbalová, Kateřina	Global gene expression changes in human	Particle and Fibre Toxicology	2012
PAHs	PAICS	Helena Líbalová, Kateřina	Global gene expression changes in human	Particle and Fibre Toxicology	2012
PAHs	PSEN2	Helena Líbalová, Kateřina	Global gene expression changes in human	Particle and Fibre Toxicology	2012
PAHs	RBM4	Helena Líbalová, Kateřina	Global gene expression changes in human	Particle and Fibre Toxicology	2012
PAHs	REV3L	Helena Líbalová, Kateřina	Global gene expression changes in human	Particle and Fibre Toxicology	2012
PAHs	RHOJ	Helena Líbalová, Kateřina	Global gene expression changes in human	Particle and Fibre Toxicology	2012
PAHs	SERPINB1	Helena Líbalová, Kateřina	Global gene expression changes in human	Particle and Fibre Toxicology	2012
PAHs	SLC16A4	Helena Líbalová, Kateřina	Global gene expression changes in human	Particle and Fibre Toxicology	2012
PAHs	SLC6A8	Helena Líbalová, Kateřina	Global gene expression changes in human	Particle and Fibre Toxicology	2012
PAHs	SLC7A2	Helena Líbalová, Kateřina	Global gene expression changes in human	Particle and Fibre Toxicology	2012
PAHs	SOD2	Helena Líbalová, Kateřina	Global gene expression changes in human	Particle and Fibre Toxicology	2012
PAHs	SULF1	Helena Líbalová, Kateřina	Global gene expression changes in human	Particle and Fibre Toxicology	2012
PAHs	TAZ	Helena Líbalová, Kateřina	Global gene expression changes in human	Particle and Fibre Toxicology	2012
PAHs	TKT	Helena Líbalová, Kateřina	Global gene expression changes in human	Particle and Fibre Toxicology	2012
PAHs	TNFRSF11B	Helena Líbalová, Kateřina	Global gene expression changes in human	Particle and Fibre Toxicology	2012
PAHs	TTC39C	Helena Líbalová, Kateřina	Global gene expression changes in human	Particle and Fibre Toxicology	2012
PAHs	TUBB6	Helena Líbalová, Kateřina	Global gene expression changes in human	Particle and Fibre Toxicology	2012
PAHs	UCRC	Helena Líbalová, Kateřina	Global gene expression changes in human	Particle and Fibre Toxicology	2012
PAHs	AADACL1	Helena Líbalová, Kateřina	Global gene expression changes in human	Particle and Fibre Toxicology	2012
PAHs	AKR1C4	Helena Líbalová, Kateřina	Global gene expression changes in human	Particle and Fibre Toxicology	2012
PAHs	AMOT	Helena Líbalová, Kateřina	Global gene expression changes in human	Particle and Fibre Toxicology	2012
PAHs	APCDD1	Helena Líbalová, Kateřina	Global gene expression changes in human	Particle and Fibre Toxicology	2012
PAHs	ARL4A	Helena Líbalová, Kateřina	Global gene expression changes in human	Particle and Fibre Toxicology	2012
PAHs	B3GALTL	Helena Líbalová, Kateřina	Global gene expression changes in human	Particle and Fibre Toxicology	2012
PAHs	BCL2	Helena Líbalová, Kateřina	Global gene expression changes in human	Particle and Fibre Toxicology	2012
PAHs	BRI3	Helena Líbalová, Kateřina	Global gene expression changes in human	Particle and Fibre Toxicology	2012
PAHs	C1orf107	Helena Líbalová, Kateřina	Global gene expression changes in human	Particle and Fibre Toxicology	2012
PAHs	C1orf51	Helena Líbalová, Kateřina	Global gene expression changes in human	Particle and Fibre Toxicology	2012
PAHs	CCDC28B	Helena Líbalová, Kateřina	Global gene expression changes in human	Particle and Fibre Toxicology	2012
PAHs	CDC25B	Helena Líbalová, Kateřina	Global gene expression changes in human	Particle and Fibre Toxicology	2012
PAHs	CEBPB	Helena Líbalová, Kateřina	Global gene expression changes in human	Particle and Fibre Toxicology	2012
PAHs	CHEK2	Helena Líbalová, Kateřina	Global gene expression changes in human	Particle and Fibre Toxicology	2012
PAHs	CLCN7	Helena Líbalová, Kateřina	Global gene expression changes in human	Particle and Fibre Toxicology	2012
PAHs	CLDN23	Helena Líbalová, Kateřina	Global gene expression changes in human	Particle and Fibre Toxicology	2012
PAHs	CYB5D1	Helena Líbalová, Kateřina	Global gene expression changes in human	Particle and Fibre Toxicology	2012
PAHs	DGUOK	Helena Líbalová, Kateřina	Global gene expression changes in human	Particle and Fibre Toxicology	2012
PAHs	DHCR7	Helena Líbalová, Kateřina	Global gene expression changes in human	Particle and Fibre Toxicology	2012
PAHs	DLL1	Helena Líbalová, Kateřina	Global gene expression changes in human	Particle and Fibre Toxicology	2012
PAHs	ENO2	Helena Líbalová, Kateřina	Global gene expression changes in human	Particle and Fibre Toxicology	2012
PAHs	ETS2	Helena Líbalová, Kateřina	Global gene expression changes in human	Particle and Fibre Toxicology	2012
PAHs	FAM43A	Helena Líbalová, Kateřina	Global gene expression changes in human	Particle and Fibre Toxicology	2012
PAHs	FAM65B	Helena Líbalová, Kateřina	Global gene expression changes in human	Particle and Fibre Toxicology	2012
PAHs	FAM83D	Helena Líbalová, Kateřina	Global gene expression changes in human	Particle and Fibre Toxicology	2012
PAHs	FLJ14166	Helena Líbalová, Kateřina	Global gene expression changes in human	Particle and Fibre Toxicology	2012
PAHs	GNPDA1	Helena Líbalová, Kateřina	Global gene expression changes in human	Particle and Fibre Toxicology	2012
PAHs	GPNMB	Helena Líbalová, Kateřina	Global gene expression changes in human	Particle and Fibre Toxicology	2012
PAHs	GYPC	Helena Líbalová, Kateřina	Global gene expression changes in human	Particle and Fibre Toxicology	2012
PAHs	HEXB	Helena Líbalová, Kateřina	Global gene expression changes in human	Particle and Fibre Toxicology	2012
PAHs	HPS1	Helena Líbalová, Kateřina	Global gene expression changes in human	Particle and Fibre Toxicology	2012
PAHs	IFI44	Helena Líbalová, Kateřina	Global gene expression changes in human	Particle and Fibre Toxicology	2012
PAHs	IL18BP	Helena Líbalová, Kateřina	Global gene expression changes in human	Particle and Fibre Toxicology	2012
PAHs	IL8	Helena Líbalová, Kateřina	Global gene expression changes in human	Particle and Fibre Toxicology	2012
PAHs	LASS1	Helena Líbalová, Kateřina	Global gene expression changes in human	Particle and Fibre Toxicology	2012
PAHs	LDHA	Helena Líbalová, Kateřina	Global gene expression changes in human	Particle and Fibre Toxicology	2012
PAHs	LOC143666	Helena Líbalová, Kateřina	Global gene expression changes in human	Particle and Fibre Toxicology	2012
PAHs	LOC399942	Helena Líbalová, Kateřina	Global gene expression changes in human	Particle and Fibre Toxicology	2012
PAHs	LRRC17	Helena Líbalová, Kateřina	Global gene expression changes in human	Particle and Fibre Toxicology	2012

PAHs	MLPH	Helena Líbalová, Kateřina	Global gene expression changes in human	Particle and Fibre Toxicology	2012
PAHs	MN1	Helena Líbalová, Kateřina	Global gene expression changes in human	Particle and Fibre Toxicology	2012
PAHs	MT1A	Helena Líbalová, Kateřina	Global gene expression changes in human	Particle and Fibre Toxicology	2012
PAHs	OCIAD2	Helena Líbalová, Kateřina	Global gene expression changes in human	Particle and Fibre Toxicology	2012
PAHs	OSBPL1A	Helena Líbalová, Kateřina	Global gene expression changes in human	Particle and Fibre Toxicology	2012
PAHs	P2RX4	Helena Líbalová, Kateřina	Global gene expression changes in human	Particle and Fibre Toxicology	2012
PAHs	PAPPA	Helena Líbalová, Kateřina	Global gene expression changes in human	Particle and Fibre Toxicology	2012
PAHs	PCDH7	Helena Líbalová, Kateřina	Global gene expression changes in human	Particle and Fibre Toxicology	2012
PAHs	PDCD4	Helena Líbalová, Kateřina	Global gene expression changes in human	Particle and Fibre Toxicology	2012
PAHs	PGBD5	Helena Líbalová, Kateřina	Global gene expression changes in human	Particle and Fibre Toxicology	2012
PAHs	PIGL	Helena Líbalová, Kateřina	Global gene expression changes in human	Particle and Fibre Toxicology	2012
PAHs	PLAU	Helena Líbalová, Kateřina	Global gene expression changes in human	Particle and Fibre Toxicology	2012
PAHs	PLEKHO1	Helena Líbalová, Kateřina	Global gene expression changes in human	Particle and Fibre Toxicology	2012
PAHs	PNCK	Helena Líbalová, Kateřina	Global gene expression changes in human	Particle and Fibre Toxicology	2012
PAHs	PPAP2C	Helena Líbalová, Kateřina	Global gene expression changes in human	Particle and Fibre Toxicology	2012
PAHs	PPARG	Helena Líbalová, Kateřina	Global gene expression changes in human	Particle and Fibre Toxicology	2012
PAHs	RAB38	Helena Líbalová, Kateřina	Global gene expression changes in human	Particle and Fibre Toxicology	2012
PAHs	RAB3IL1	Helena Líbalová, Kateřina	Global gene expression changes in human	Particle and Fibre Toxicology	2012
PAHs	RAPGEF1	Helena Líbalová, Kateřina	Global gene expression changes in human	Particle and Fibre Toxicology	2012
PAHs	RFNG	Helena Líbalová, Kateřina	Global gene expression changes in human	Particle and Fibre Toxicology	2012
PAHs	RGS11	Helena Líbalová, Kateřina	Global gene expression changes in human	Particle and Fibre Toxicology	2012
PAHs	RGS20	Helena Líbalová, Kateřina	Global gene expression changes in human	Particle and Fibre Toxicology	2012
PAHs	RRP12	Helena Líbalová, Kateřina	Global gene expression changes in human	Particle and Fibre Toxicology	2012
PAHs	RTN3	Helena Líbalová, Kateřina	Global gene expression changes in human	Particle and Fibre Toxicology	2012
PAHs	SDC4	Helena Líbalová, Kateřina	Global gene expression changes in human	Particle and Fibre Toxicology	2012
PAHs	SFRS10	Helena Líbalová, Kateřina	Global gene expression changes in human	Particle and Fibre Toxicology	2012
PAHs	SFRS3	Helena Líbalová, Kateřina	Global gene expression changes in human	Particle and Fibre Toxicology	2012
PAHs	SLC16A14	Helena Líbalová, Kateřina	Global gene expression changes in human	Particle and Fibre Toxicology	2012
PAHs	SLC26A6	Helena Líbalová, Kateřina	Global gene expression changes in human	Particle and Fibre Toxicology	2012
PAHs	SNHG3-RCC1	Helena Líbalová, Kateřina	Global gene expression changes in human	Particle and Fibre Toxicology	2012
PAHs	SQSTM1	Helena Líbalová, Kateřina	Global gene expression changes in human	Particle and Fibre Toxicology	2012
PAHs	STAT5A	Helena Líbalová, Kateřina	Global gene expression changes in human	Particle and Fibre Toxicology	2012
PAHs	TALDO1	Helena Líbalová, Kateřina	Global gene expression changes in human	Particle and Fibre Toxicology	2012
PAHs	TBC1D8	Helena Líbalová, Kateřina	Global gene expression changes in human	Particle and Fibre Toxicology	2012
PAHs	TGIF1	Helena Líbalová, Kateřina	Global gene expression changes in human	Particle and Fibre Toxicology	2012
PAHs	THRA	Helena Líbalová, Kateřina	Global gene expression changes in human	Particle and Fibre Toxicology	2012
PAHs	TRAF3IP2	Helena Líbalová, Kateřina	Global gene expression changes in human	Particle and Fibre Toxicology	2012
PAHs	TRIM55	Helena Líbalová, Kateřina	Global gene expression changes in human	Particle and Fibre Toxicology	2012
PAHs	TSHZ1	Helena Líbalová, Kateřina	Global gene expression changes in human	Particle and Fibre Toxicology	2012
PAHs	TSPYL2	Helena Líbalová, Kateřina	Global gene expression changes in human	Particle and Fibre Toxicology	2012
PAHs	TUBA1C	Helena Líbalová, Kateřina	Global gene expression changes in human	Particle and Fibre Toxicology	2012
PAHs	TUBB2A	Helena Líbalová, Kateřina	Global gene expression changes in human	Particle and Fibre Toxicology	2012
PAHs	TUBB2B	Helena Líbalová, Kateřina	Global gene expression changes in human	Particle and Fibre Toxicology	2012
PAHs	UAP1L1	Helena Líbalová, Kateřina	Global gene expression changes in human	Particle and Fibre Toxicology	2012
PAHs	UBE2C	Helena Líbalová, Kateřina	Global gene expression changes in human	Particle and Fibre Toxicology	2012
PAHs	UGP2	Helena Líbalová, Kateřina	Global gene expression changes in human	Particle and Fibre Toxicology	2012
PAHs	USP36	Helena Líbalová, Kateřina	Global gene expression changes in human	Particle and Fibre Toxicology	2012
PAHs	YWHAB	Helena Líbalová, Kateřina	Global gene expression changes in human	Particle and Fibre Toxicology	2012
PAHs	ZNF598	Helena Líbalová, Kateřina	Global gene expression changes in human	Particle and Fibre Toxicology	2012
PAHs	ABHD6	Helena Líbalová, Kateřina	Global gene expression changes in human	Particle and Fibre Toxicology	2012
PAHs	ACOX2	Helena Líbalová, Kateřina	Global gene expression changes in human	Particle and Fibre Toxicology	2012
PAHs	ADAM17	Helena Líbalová, Kateřina	Global gene expression changes in human	Particle and Fibre Toxicology	2012
PAHs	AK3L1	Helena Líbalová, Kateřina	Global gene expression changes in human	Particle and Fibre Toxicology	2012
PAHs	ANXA2	Helena Líbalová, Kateřina	Global gene expression changes in human	Particle and Fibre Toxicology	2012
PAHs	APAF1	Helena Líbalová, Kateřina	Global gene expression changes in human	Particle and Fibre Toxicology	2012
PAHs	AQP11	Helena Líbalová, Kateřina	Global gene expression changes in human	Particle and Fibre Toxicology	2012
PAHs	ARHGDIB	Helena Líbalová, Kateřina	Global gene expression changes in human	Particle and Fibre Toxicology	2012
PAHs	ARL4C	Helena Líbalová, Kateřina	Global gene expression changes in human	Particle and Fibre Toxicology	2012
PAHs	ARMCX3	Helena Líbalová, Kateřina	Global gene expression changes in human	Particle and Fibre Toxicology	2012
PAHs	ARSG	Helena Líbalová, Kateřina	Global gene expression changes in human	Particle and Fibre Toxicology	2012
PAHs	ATP1B3	Helena Líbalová, Kateřina	Global gene expression changes in human	Particle and Fibre Toxicology	2012
PAHs	ATP6V1C1	Helena Líbalová, Kateřina	Global gene expression changes in human	Particle and Fibre Toxicology	2012
PAHs	ATP6V1G2	Helena Líbalová, Kateřina	Global gene expression changes in human	Particle and Fibre Toxicology	2012
PAHs	AURKA	Helena Líbalová, Kateřina	Global gene expression changes in human	Particle and Fibre Toxicology	2012
PAHs	C13orf15	Helena Líbalová, Kateřina	Global gene expression changes in human	Particle and Fibre Toxicology	2012
PAHs	C16orf52	Helena Líbalová, Kateřina	Global gene expression changes in human	Particle and Fibre Toxicology	2012
PAHs	C17orf91	Helena Líbalová, Kateřina	Global gene expression changes in human	Particle and Fibre Toxicology	2012
PAHs	C1orf97	Helena Líbalová, Kateřina	Global gene expression changes in human	Particle and Fibre Toxicology	2012
PAHs	C21orf121	Helena Líbalová, Kateřina	Global gene expression changes in human	Particle and Fibre Toxicology	2012
PAHs	C2orf56	Helena Líbalová, Kateřina	Global gene expression changes in human	Particle and Fibre Toxicology	2012
PAHs	C3orf59	Helena Líbalová, Kateřina	Global gene expression changes in human	Particle and Fibre Toxicology	2012
PAHs	C6orf162	Helena Líbalová, Kateřina	Global gene expression changes in human	Particle and Fibre Toxicology	2012
PAHs	C9orf21	Helena Líbalová, Kateřina	Global gene expression changes in human	Particle and Fibre Toxicology	2012
PAHs	CCDC109B	Helena Líbalová, Kateřina	Global gene expression changes in human	Particle and Fibre Toxicology	2012
PAHs	CCDC123	Helena Líbalová, Kateřina	Global gene expression changes in human	Particle and Fibre Toxicology	2012
PAHs	CCDC68	Helena Líbalová, Kateřina	Global gene expression changes in human	Particle and Fibre Toxicology	2012
PAHs	CCDC80	Helena Líbalová, Kateřina	Global gene expression changes in human	Particle and Fibre Toxicology	2012
PAHs	CD55	Helena Líbalová, Kateřina	Global gene expression changes in human	Particle and Fibre Toxicology	2012
PAHs	CDCP1	Helena Líbalová, Kateřina	Global gene expression changes in human	Particle and Fibre Toxicology	2012
PAHs	CDO1	Helena Líbalová, Kateřina	Global gene expression changes in human	Particle and Fibre Toxicology	2012
PAHs	CEND1	Helena Líbalová, Kateřina	Global gene expression changes in human	Particle and Fibre Toxicology	2012
PAHs	CFH	Helena Líbalová, Kateřina	Global gene expression changes in human	Particle and Fibre Toxicology	2012
PAHs	CHURC1	Helena Líbalová, Kateřina	Global gene expression changes in human	Particle and Fibre Toxicology	2012
PAHs	CNOT1	Helena Líbalová, Kateřina	Global gene expression changes in human	Particle and Fibre Toxicology	2012
PAHs	COL1A1	Helena Líbalová, Kateřina	Global gene expression changes in human	Particle and Fibre Toxicology	2012
PAHs	CPA4	Helena Líbalová, Kateřina	Global gene expression changes in human	Particle and Fibre Toxicology	2012
PAHs	D2HGDH	Helena Líbalová, Kateřina	Global gene expression changes in human	Particle and Fibre Toxicology	2012
PAHs	DBNL	Helena Líbalová, Kateřina	Global gene expression changes in human	Particle and Fibre Toxicology	2012
PAHs	DDIT4L	Helena Líbalová, Kateřina	Global gene expression changes in human	Particle and Fibre Toxicology	2012

PAHs	DIRAS3	Helena Líbalová, Kateřina	Global gene expression changes in human	Particle and Fibre Toxicology	2012
PAHs	DOCK11	Helena Líbalová, Kateřina	Global gene expression changes in human	Particle and Fibre Toxicology	2012
PAHs	DPF3	Helena Líbalová, Kateřina	Global gene expression changes in human	Particle and Fibre Toxicology	2012
PAHs	DPY19L2	Helena Líbalová, Kateřina	Global gene expression changes in human	Particle and Fibre Toxicology	2012
PAHs	ENDOGL1	Helena Líbalová, Kateřina	Global gene expression changes in human	Particle and Fibre Toxicology	2012
PAHs	EPHA4	Helena Líbalová, Kateřina	Global gene expression changes in human	Particle and Fibre Toxicology	2012
PAHs	ERRFI1	Helena Líbalová, Kateřina	Global gene expression changes in human	Particle and Fibre Toxicology	2012
PAHs	FAR1	Helena Líbalová, Kateřina	Global gene expression changes in human	Particle and Fibre Toxicology	2012
PAHs	FBN1	Helena Líbalová, Kateřina	Global gene expression changes in human	Particle and Fibre Toxicology	2012
PAHs	FLCN	Helena Líbalová, Kateřina	Global gene expression changes in human	Particle and Fibre Toxicology	2012
PAHs	FTHL2	Helena Líbalová, Kateřina	Global gene expression changes in human	Particle and Fibre Toxicology	2012
PAHs	FUBP1	Helena Líbalová, Kateřina	Global gene expression changes in human	Particle and Fibre Toxicology	2012
PAHs	GAS1	Helena Líbalová, Kateřina	Global gene expression changes in human	Particle and Fibre Toxicology	2012
PAHs	GBGT1	Helena Líbalová, Kateřina	Global gene expression changes in human	Particle and Fibre Toxicology	2012
PAHs	GBP2	Helena Líbalová, Kateřina	Global gene expression changes in human	Particle and Fibre Toxicology	2012
PAHs	GDF15	Helena Líbalová, Kateřina	Global gene expression changes in human	Particle and Fibre Toxicology	2012
PAHs	GEM	Helena Líbalová, Kateřina	Global gene expression changes in human	Particle and Fibre Toxicology	2012
PAHs	GLA	Helena Líbalová, Kateřina	Global gene expression changes in human	Particle and Fibre Toxicology	2012
PAHs	GLRB	Helena Líbalová, Kateřina	Global gene expression changes in human	Particle and Fibre Toxicology	2012
PAHs	GLT8D2	Helena Líbalová, Kateřina	Global gene expression changes in human	Particle and Fibre Toxicology	2012
PAHs	GSR	Helena Líbalová, Kateřina	Global gene expression changes in human	Particle and Fibre Toxicology	2012
PAHs	GTPBP6	Helena Líbalová, Kateřina	Global gene expression changes in human	Particle and Fibre Toxicology	2012
PAHs	HES4	Helena Líbalová, Kateřina	Global gene expression changes in human	Particle and Fibre Toxicology	2012
PAHs	HIST2H4A	Helena Líbalová, Kateřina	Global gene expression changes in human	Particle and Fibre Toxicology	2012
PAHs	HS1BP3	Helena Líbalová, Kateřina	Global gene expression changes in human	Particle and Fibre Toxicology	2012
PAHs	HSPBAP1	Helena Líbalová, Kateřina	Global gene expression changes in human	Particle and Fibre Toxicology	2012
PAHs	ID1	Helena Líbalová, Kateřina	Global gene expression changes in human	Particle and Fibre Toxicology	2012
PAHs	IER3	Helena Líbalová, Kateřina	Global gene expression changes in human	Particle and Fibre Toxicology	2012
PAHs	IRS2	Helena Líbalová, Kateřina	Global gene expression changes in human	Particle and Fibre Toxicology	2012
PAHs	KAT2A	Helena Líbalová, Kateřina	Global gene expression changes in human	Particle and Fibre Toxicology	2012
PAHs	KCNS3	Helena Líbalová, Kateřina	Global gene expression changes in human	Particle and Fibre Toxicology	2012
PAHs	KIAA1522	Helena Líbalová, Kateřina	Global gene expression changes in human	Particle and Fibre Toxicology	2012
PAHs	KIF20A	Helena Líbalová, Kateřina	Global gene expression changes in human	Particle and Fibre Toxicology	2012
PAHs	LAMP2	Helena Líbalová, Kateřina	Global gene expression changes in human	Particle and Fibre Toxicology	2012
PAHs	LOC338758	Helena Líbalová, Kateřina	Global gene expression changes in human	Particle and Fibre Toxicology	2012
PAHs	LOC644979	Helena Líbalová, Kateřina	Global gene expression changes in human	Particle and Fibre Toxicology	2012
PAHs	LOC650029	Helena Líbalová, Kateřina	Global gene expression changes in human	Particle and Fibre Toxicology	2012
PAHs	LOC650803	Helena Líbalová, Kateřina	Global gene expression changes in human	Particle and Fibre Toxicology	2012
PAHs	LOC651380	Helena Líbalová, Kateřina	Global gene expression changes in human	Particle and Fibre Toxicology	2012
PAHs	LOC730455	Helena Líbalová, Kateřina	Global gene expression changes in human	Particle and Fibre Toxicology	2012
PAHs	LRRFIP2	Helena Líbalová, Kateřina	Global gene expression changes in human	Particle and Fibre Toxicology	2012
PAHs	LXN	Helena Líbalová, Kateřina	Global gene expression changes in human	Particle and Fibre Toxicology	2012
PAHs	MANBA	Helena Líbalová, Kateřina	Global gene expression changes in human	Particle and Fibre Toxicology	2012
PAHs	MEF2A	Helena Líbalová, Kateřina	Global gene expression changes in human	Particle and Fibre Toxicology	2012
PAHs	MMAB	Helena Líbalová, Kateřina	Global gene expression changes in human	Particle and Fibre Toxicology	2012
PAHs	MMP14	Helena Líbalová, Kateřina	Global gene expression changes in human	Particle and Fibre Toxicology	2012
PAHs	MTERFD1	Helena Líbalová, Kateřina	Global gene expression changes in human	Particle and Fibre Toxicology	2012
PAHs	NEU1	Helena Líbalová, Kateřina	Global gene expression changes in human	Particle and Fibre Toxicology	2012
PAHs	NMT2	Helena Líbalová, Kateřina	Global gene expression changes in human	Particle and Fibre Toxicology	2012
PAHs	NTNG1	Helena Líbalová, Kateřina	Global gene expression changes in human	Particle and Fibre Toxicology	2012
PAHs	NUSAP1	Helena Líbalová, Kateřina	Global gene expression changes in human	Particle and Fibre Toxicology	2012
PAHs	OSGIN1	Helena Líbalová, Kateřina	Global gene expression changes in human	Particle and Fibre Toxicology	2012
PAHs	PAQR5	Helena Líbalová, Kateřina	Global gene expression changes in human	Particle and Fibre Toxicology	2012
PAHs	PDGFRL	Helena Líbalová, Kateřina	Global gene expression changes in human	Particle and Fibre Toxicology	2012
PAHs	PER3	Helena Líbalová, Kateřina	Global gene expression changes in human	Particle and Fibre Toxicology	2012
PAHs	PGAM4	Helena Líbalová, Kateřina	Global gene expression changes in human	Particle and Fibre Toxicology	2012
PAHs	PGD	Helena Líbalová, Kateřina	Global gene expression changes in human	Particle and Fibre Toxicology	2012
PAHs	PHF1	Helena Líbalová, Kateřina	Global gene expression changes in human	Particle and Fibre Toxicology	2012
PAHs	PHF11	Helena Líbalová, Kateřina	Global gene expression changes in human	Particle and Fibre Toxicology	2012
PAHs	PJA1	Helena Líbalová, Kateřina	Global gene expression changes in human	Particle and Fibre Toxicology	2012
PAHs	PLEKHM2	Helena Líbalová, Kateřina	Global gene expression changes in human	Particle and Fibre Toxicology	2012
PAHs	PODN	Helena Líbalová, Kateřina	Global gene expression changes in human	Particle and Fibre Toxicology	2012
PAHs	POLE2	Helena Líbalová, Kateřina	Global gene expression changes in human	Particle and Fibre Toxicology	2012
PAHs	PPFIA1	Helena Líbalová, Kateřina	Global gene expression changes in human	Particle and Fibre Toxicology	2012
PAHs	PPIC	Helena Líbalová, Kateřina	Global gene expression changes in human	Particle and Fibre Toxicology	2012
PAHs	PPIL1	Helena Líbalová, Kateřina	Global gene expression changes in human	Particle and Fibre Toxicology	2012
PAHs	PSMD9	Helena Líbalová, Kateřina	Global gene expression changes in human	Particle and Fibre Toxicology	2012
PAHs	PWWP2B	Helena Líbalová, Kateřina	Global gene expression changes in human	Particle and Fibre Toxicology	2012
PAHs	RARRES3	Helena Líbalová, Kateřina	Global gene expression changes in human	Particle and Fibre Toxicology	2012
PAHs	RASL11B	Helena Líbalová, Kateřina	Global gene expression changes in human	Particle and Fibre Toxicology	2012
PAHs	RGS4	Helena Líbalová, Kateřina	Global gene expression changes in human	Particle and Fibre Toxicology	2012
PAHs	RMI1	Helena Líbalová, Kateřina	Global gene expression changes in human	Particle and Fibre Toxicology	2012
PAHs	RPUSD4	Helena Líbalová, Kateřina	Global gene expression changes in human	Particle and Fibre Toxicology	2012
PAHs	RRAGC	Helena Líbalová, Kateřina	Global gene expression changes in human	Particle and Fibre Toxicology	2012
PAHs	RSU1	Helena Líbalová, Kateřina	Global gene expression changes in human	Particle and Fibre Toxicology	2012
PAHs	SAMD4A	Helena Líbalová, Kateřina	Global gene expression changes in human	Particle and Fibre Toxicology	2012
PAHs	SAMD9	Helena Líbalová, Kateřina	Global gene expression changes in human	Particle and Fibre Toxicology	2012
PAHs	SERPINA3	Helena Líbalová, Kateřina	Global gene expression changes in human	Particle and Fibre Toxicology	2012
PAHs	SGCD	Helena Líbalová, Kateřina	Global gene expression changes in human	Particle and Fibre Toxicology	2012
PAHs	SH2D5	Helena Líbalová, Kateřina	Global gene expression changes in human	Particle and Fibre Toxicology	2012
PAHs	SIPA1L2	Helena Líbalová, Kateřina	Global gene expression changes in human	Particle and Fibre Toxicology	2012
PAHs	SKP2	Helena Líbalová, Kateřina	Global gene expression changes in human	Particle and Fibre Toxicology	2012
PAHs	SLC16A2	Helena Líbalová, Kateřina	Global gene expression changes in human	Particle and Fibre Toxicology	2012
PAHs	SLC25A40	Helena Líbalová, Kateřina	Global gene expression changes in human	Particle and Fibre Toxicology	2012
PAHs	SLC7A11	Helena Líbalová, Kateřina	Global gene expression changes in human	Particle and Fibre Toxicology	2012
PAHs	SMARCA2	Helena Líbalová, Kateřina	Global gene expression changes in human	Particle and Fibre Toxicology	2012
PAHs	SNX8	Helena Líbalová, Kateřina	Global gene expression changes in human	Particle and Fibre Toxicology	2012
PAHs	SRXN1	Helena Líbalová, Kateřina	Global gene expression changes in human	Particle and Fibre Toxicology	2012
PAHs	STX6	Helena Líbalová, Kateřina	Global gene expression changes in human	Particle and Fibre Toxicology	2012

PAHs	SVIL	Helena Líbalová, Kateřina	Global gene expression changes in human	Particle and Fibre Toxicology	2012
PAHs	SYT1	Helena Líbalová, Kateřina	Global gene expression changes in human	Particle and Fibre Toxicology	2012
PAHs	TAF6L	Helena Líbalová, Kateřina	Global gene expression changes in human	Particle and Fibre Toxicology	2012
PAHs	TFB1M	Helena Líbalová, Kateřina	Global gene expression changes in human	Particle and Fibre Toxicology	2012
PAHs	TIMP3	Helena Líbalová, Kateřina	Global gene expression changes in human	Particle and Fibre Toxicology	2012
PAHs	TJP1	Helena Líbalová, Kateřina	Global gene expression changes in human	Particle and Fibre Toxicology	2012
PAHs	TMEM147	Helena Líbalová, Kateřina	Global gene expression changes in human	Particle and Fibre Toxicology	2012
PAHs	TMEM156	Helena Líbalová, Kateřina	Global gene expression changes in human	Particle and Fibre Toxicology	2012
PAHs	TMEM38B	Helena Líbalová, Kateřina	Global gene expression changes in human	Particle and Fibre Toxicology	2012
PAHs	TMTC2	Helena Líbalová, Kateřina	Global gene expression changes in human	Particle and Fibre Toxicology	2012
PAHs	TOP1MT	Helena Líbalová, Kateřina	Global gene expression changes in human	Particle and Fibre Toxicology	2012
PAHs	TPM1	Helena Líbalová, Kateřina	Global gene expression changes in human	Particle and Fibre Toxicology	2012
PAHs	TTLL4	Helena Líbalová, Kateřina	Global gene expression changes in human	Particle and Fibre Toxicology	2012
PAHs	UHRF1	Helena Líbalová, Kateřina	Global gene expression changes in human	Particle and Fibre Toxicology	2012
PAHs	VAT1	Helena Líbalová, Kateřina	Global gene expression changes in human	Particle and Fibre Toxicology	2012
PAHs	WDR19	Helena Líbalová, Kateřina	Global gene expression changes in human	Particle and Fibre Toxicology	2012
PAHs	WNT2	Helena Líbalová, Kateřina	Global gene expression changes in human	Particle and Fibre Toxicology	2012
PAHs	ZCCHC14	Helena Líbalová, Kateřina	Global gene expression changes in human	Particle and Fibre Toxicology	2012
PAHs	ZMYM4	Helena Líbalová, Kateřina	Global gene expression changes in human	Particle and Fibre Toxicology	2012
PAHs	ZNF330	Helena Líbalová, Kateřina	Global gene expression changes in human	Particle and Fibre Toxicology	2012
PAHs	ZNF581	Helena Líbalová, Kateřina	Global gene expression changes in human	Particle and Fibre Toxicology	2012
PAHs	ZNF746	Helena Líbalová, Kateřina	Global gene expression changes in human	Particle and Fibre Toxicology	2012
PAHs	ABCA1	Helena Líbalová, Kateřina	Global gene expression changes in human	Particle and Fibre Toxicology	2012
PAHs	ABCC5	Helena Líbalová, Kateřina	Global gene expression changes in human	Particle and Fibre Toxicology	2012
PAHs	ACACB	Helena Líbalová, Kateřina	Global gene expression changes in human	Particle and Fibre Toxicology	2012
PAHs	ACAD11	Helena Líbalová, Kateřina	Global gene expression changes in human	Particle and Fibre Toxicology	2012
PAHs	ACAT2	Helena Líbalová, Kateřina	Global gene expression changes in human	Particle and Fibre Toxicology	2012
PAHs	ACOX3	Helena Líbalová, Kateřina	Global gene expression changes in human	Particle and Fibre Toxicology	2012
PAHs	ACSL3	Helena Líbalová, Kateřina	Global gene expression changes in human	Particle and Fibre Toxicology	2012
PAHs	ADO	Helena Líbalová, Kateřina	Global gene expression changes in human	Particle and Fibre Toxicology	2012
PAHs	AK5	Helena Líbalová, Kateřina	Global gene expression changes in human	Particle and Fibre Toxicology	2012
PAHs	AKR1C2	Helena Líbalová, Kateřina	Global gene expression changes in human	Particle and Fibre Toxicology	2012
PAHs	ALDH18A1	Helena Líbalová, Kateřina	Global gene expression changes in human	Particle and Fibre Toxicology	2012
PAHs	ALOX5AP	Helena Líbalová, Kateřina	Global gene expression changes in human	Particle and Fibre Toxicology	2012
PAHs	ANKRD10	Helena Líbalová, Kateřina	Global gene expression changes in human	Particle and Fibre Toxicology	2012
PAHs	ANXA2P1	Helena Líbalová, Kateřina	Global gene expression changes in human	Particle and Fibre Toxicology	2012
PAHs	APH1B	Helena Líbalová, Kateřina	Global gene expression changes in human	Particle and Fibre Toxicology	2012
PAHs	APOL2	Helena Líbalová, Kateřina	Global gene expression changes in human	Particle and Fibre Toxicology	2012
PAHs	ARHGAP23	Helena Líbalová, Kateřina	Global gene expression changes in human	Particle and Fibre Toxicology	2012
PAHs	ARHGAP24	Helena Líbalová, Kateřina	Global gene expression changes in human	Particle and Fibre Toxicology	2012
PAHs	ARL16	Helena Líbalová, Kateřina	Global gene expression changes in human	Particle and Fibre Toxicology	2012
PAHs	ARL3	Helena Líbalová, Kateřina	Global gene expression changes in human	Particle and Fibre Toxicology	2012
PAHs	ARMCX2	Helena Líbalová, Kateřina	Global gene expression changes in human	Particle and Fibre Toxicology	2012
PAHs	ATP6V0B	Helena Líbalová, Kateřina	Global gene expression changes in human	Particle and Fibre Toxicology	2012
PAHs	ATP6V1A	Helena Líbalová, Kateřina	Global gene expression changes in human	Particle and Fibre Toxicology	2012
PAHs	ATP6V1B2	Helena Líbalová, Kateřina	Global gene expression changes in human	Particle and Fibre Toxicology	2012
PAHs	ATP6V1F	Helena Líbalová, Kateřina	Global gene expression changes in human	Particle and Fibre Toxicology	2012
PAHs	AURKB	Helena Líbalová, Kateřina	Global gene expression changes in human	Particle and Fibre Toxicology	2012
PAHs	BAALC	Helena Líbalová, Kateřina	Global gene expression changes in human	Particle and Fibre Toxicology	2012
PAHs	BBS7	Helena Líbalová, Kateřina	Global gene expression changes in human	Particle and Fibre Toxicology	2012
PAHs	BCAR3	Helena Líbalová, Kateřina	Global gene expression changes in human	Particle and Fibre Toxicology	2012
PAHs	BCCIP	Helena Líbalová, Kateřina	Global gene expression changes in human	Particle and Fibre Toxicology	2012
PAHs	BCL7C	Helena Líbalová, Kateřina	Global gene expression changes in human	Particle and Fibre Toxicology	2012
PAHs	BNC2	Helena Líbalová, Kateřina	Global gene expression changes in human	Particle and Fibre Toxicology	2012
PAHs	BTBD6	Helena Líbalová, Kateřina	Global gene expression changes in human	Particle and Fibre Toxicology	2012
PAHs	BTF3L4	Helena Líbalová, Kateřina	Global gene expression changes in human	Particle and Fibre Toxicology	2012
PAHs	C15orf17	Helena Líbalová, Kateřina	Global gene expression changes in human	Particle and Fibre Toxicology	2012
PAHs	C17orf39	Helena Líbalová, Kateřina	Global gene expression changes in human	Particle and Fibre Toxicology	2012
PAHs	C18orf8	Helena Líbalová, Kateřina	Global gene expression changes in human	Particle and Fibre Toxicology	2012
PAHs	C1orf31	Helena Líbalová, Kateřina	Global gene expression changes in human	Particle and Fibre Toxicology	2012
PAHs	C1orf85	Helena Líbalová, Kateřina	Global gene expression changes in human	Particle and Fibre Toxicology	2012
PAHs	C20orf175	Helena Líbalová, Kateřina	Global gene expression changes in human	Particle and Fibre Toxicology	2012
PAHs	C2orf34	Helena Líbalová, Kateřina	Global gene expression changes in human	Particle and Fibre Toxicology	2012
PAHs	C4orf14	Helena Líbalová, Kateřina	Global gene expression changes in human	Particle and Fibre Toxicology	2012
PAHs	C5orf32	Helena Líbalová, Kateřina	Global gene expression changes in human	Particle and Fibre Toxicology	2012
PAHs	C5orf4	Helena Líbalová, Kateřina	Global gene expression changes in human	Particle and Fibre Toxicology	2012
PAHs	C8orf4	Helena Líbalová, Kateřina	Global gene expression changes in human	Particle and Fibre Toxicology	2012
PAHs	C9orf150	Helena Líbalová, Kateřina	Global gene expression changes in human	Particle and Fibre Toxicology	2012
PAHs	CABLES1	Helena Líbalová, Kateřina	Global gene expression changes in human	Particle and Fibre Toxicology	2012
PAHs	CALB2	Helena Líbalová, Kateřina	Global gene expression changes in human	Particle and Fibre Toxicology	2012
PAHs	CALD1	Helena Líbalová, Kateřina	Global gene expression changes in human	Particle and Fibre Toxicology	2012
PAHs	CALML4	Helena Líbalová, Kateřina	Global gene expression changes in human	Particle and Fibre Toxicology	2012
PAHs	CAV1	Helena Líbalová, Kateřina	Global gene expression changes in human	Particle and Fibre Toxicology	2012
PAHs	CCDC101	Helena Líbalová, Kateřina	Global gene expression changes in human	Particle and Fibre Toxicology	2012
PAHs	CCNB2	Helena Líbalová, Kateřina	Global gene expression changes in human	Particle and Fibre Toxicology	2012
PAHs	CCNE1	Helena Líbalová, Kateřina	Global gene expression changes in human	Particle and Fibre Toxicology	2012
PAHs	CDC2	Helena Líbalová, Kateřina	Global gene expression changes in human	Particle and Fibre Toxicology	2012
PAHs	CDCA3	Helena Líbalová, Kateřina	Global gene expression changes in human	Particle and Fibre Toxicology	2012
PAHs	CDKN1A	Helena Líbalová, Kateřina	Global gene expression changes in human	Particle and Fibre Toxicology	2012
PAHs	CDKN2A	Helena Líbalová, Kateřina	Global gene expression changes in human	Particle and Fibre Toxicology	2012
PAHs	CENPM	Helena Líbalová, Kateřina	Global gene expression changes in human	Particle and Fibre Toxicology	2012
PAHs	CHCHD7	Helena Líbalová, Kateřina	Global gene expression changes in human	Particle and Fibre Toxicology	2012
PAHs	CITED4	Helena Líbalová, Kateřina	Global gene expression changes in human	Particle and Fibre Toxicology	2012
PAHs	CLCN6	Helena Líbalová, Kateřina	Global gene expression changes in human	Particle and Fibre Toxicology	2012
PAHs	CLDN14	Helena Líbalová, Kateřina	Global gene expression changes in human	Particle and Fibre Toxicology	2012
PAHs	CNIH2	Helena Líbalová, Kateřina	Global gene expression changes in human	Particle and Fibre Toxicology	2012
PAHs	COL4A5	Helena Líbalová, Kateřina	Global gene expression changes in human	Particle and Fibre Toxicology	2012
PAHs	CORO7	Helena Líbalová, Kateřina	Global gene expression changes in human	Particle and Fibre Toxicology	2012
PAHs	CPZ	Helena Líbalová, Kateřina	Global gene expression changes in human	Particle and Fibre Toxicology	2012

PAHs	CRABP2	Helena Líbalová, Kateřina	Global gene expression changes in human	Particle and Fibre Toxicology	2012
PAHs	CREG1	Helena Líbalová, Kateřina	Global gene expression changes in human	Particle and Fibre Toxicology	2012
PAHs	CSNK2A2	Helena Líbalová, Kateřina	Global gene expression changes in human	Particle and Fibre Toxicology	2012
PAHs	CSTB	Helena Líbalová, Kateřina	Global gene expression changes in human	Particle and Fibre Toxicology	2012
PAHs	DCTN3	Helena Líbalová, Kateřina	Global gene expression changes in human	Particle and Fibre Toxicology	2012
PAHs	DENND1A	Helena Líbalová, Kateřina	Global gene expression changes in human	Particle and Fibre Toxicology	2012
PAHs	DHRSX	Helena Líbalová, Kateřina	Global gene expression changes in human	Particle and Fibre Toxicology	2012
PAHs	DIMT1L	Helena Líbalová, Kateřina	Global gene expression changes in human	Particle and Fibre Toxicology	2012
PAHs	DKK1	Helena Líbalová, Kateřina	Global gene expression changes in human	Particle and Fibre Toxicology	2012
PAHs	DMAP1	Helena Líbalová, Kateřina	Global gene expression changes in human	Particle and Fibre Toxicology	2012
PAHs	DNAJA3	Helena Líbalová, Kateřina	Global gene expression changes in human	Particle and Fibre Toxicology	2012
PAHs	DNAJA4	Helena Líbalová, Kateřina	Global gene expression changes in human	Particle and Fibre Toxicology	2012
PAHs	DNAJC19	Helena Líbalová, Kateřina	Global gene expression changes in human	Particle and Fibre Toxicology	2012
PAHs	DPYSL2	Helena Líbalová, Kateřina	Global gene expression changes in human	Particle and Fibre Toxicology	2012
PAHs	DUSP5	Helena Líbalová, Kateřina	Global gene expression changes in human	Particle and Fibre Toxicology	2012
PAHs	EGLN2	Helena Líbalová, Kateřina	Global gene expression changes in human	Particle and Fibre Toxicology	2012
PAHs	EHBP1	Helena Líbalová, Kateřina	Global gene expression changes in human	Particle and Fibre Toxicology	2012
PAHs	EHHADH	Helena Líbalová, Kateřina	Global gene expression changes in human	Particle and Fibre Toxicology	2012
PAHs	EIF1	Helena Líbalová, Kateřina	Global gene expression changes in human	Particle and Fibre Toxicology	2012
PAHs	EIF2A	Helena Líbalová, Kateřina	Global gene expression changes in human	Particle and Fibre Toxicology	2012
PAHs	EIF2S2	Helena Líbalová, Kateřina	Global gene expression changes in human	Particle and Fibre Toxicology	2012
PAHs	EIF4A2	Helena Líbalová, Kateřina	Global gene expression changes in human	Particle and Fibre Toxicology	2012
PAHs	ELMOD2	Helena Líbalová, Kateřina	Global gene expression changes in human	Particle and Fibre Toxicology	2012
PAHs	EPB41L2	Helena Líbalová, Kateřina	Global gene expression changes in human	Particle and Fibre Toxicology	2012
PAHs	EPM2AIP1	Helena Líbalová, Kateřina	Global gene expression changes in human	Particle and Fibre Toxicology	2012
PAHs	FAIM2	Helena Líbalová, Kateřina	Global gene expression changes in human	Particle and Fibre Toxicology	2012
PAHs	FAM134A	Helena Líbalová, Kateřina	Global gene expression changes in human	Particle and Fibre Toxicology	2012
PAHs	FAM44B	Helena Líbalová, Kateřina	Global gene expression changes in human	Particle and Fibre Toxicology	2012
PAHs	FBXO3	Helena Líbalová, Kateřina	Global gene expression changes in human	Particle and Fibre Toxicology	2012
PAHs	FBXO6	Helena Líbalová, Kateřina	Global gene expression changes in human	Particle and Fibre Toxicology	2012
PAHs	FKBP11	Helena Líbalová, Kateřina	Global gene expression changes in human	Particle and Fibre Toxicology	2012
PAHs	FLJ38482	Helena Líbalová, Kateřina	Global gene expression changes in human	Particle and Fibre Toxicology	2012
PAHs	FLJ40852	Helena Líbalová, Kateřina	Global gene expression changes in human	Particle and Fibre Toxicology	2012
PAHs	FMO2	Helena Líbalová, Kateřina	Global gene expression changes in human	Particle and Fibre Toxicology	2012
PAHs	FMO4	Helena Líbalová, Kateřina	Global gene expression changes in human	Particle and Fibre Toxicology	2012
PAHs	FTHL12	Helena Líbalová, Kateřina	Global gene expression changes in human	Particle and Fibre Toxicology	2012
PAHs	GBF1	Helena Líbalová, Kateřina	Global gene expression changes in human	Particle and Fibre Toxicology	2012
PAHs	GNA13	Helena Líbalová, Kateřina	Global gene expression changes in human	Particle and Fibre Toxicology	2012
PAHs	GOLPH3L	Helena Líbalová, Kateřina	Global gene expression changes in human	Particle and Fibre Toxicology	2012
PAHs	GPC4	Helena Líbalová, Kateřina	Global gene expression changes in human	Particle and Fibre Toxicology	2012
PAHs	GPR137B	Helena Líbalová, Kateřina	Global gene expression changes in human	Particle and Fibre Toxicology	2012
PAHs	GRAMD3	Helena Líbalová, Kateřina	Global gene expression changes in human	Particle and Fibre Toxicology	2012
PAHs	GTF2IRD1	Helena Líbalová, Kateřina	Global gene expression changes in human	Particle and Fibre Toxicology	2012
PAHs	HEATR1	Helena Líbalová, Kateřina	Global gene expression changes in human	Particle and Fibre Toxicology	2012
PAHs	HEBP1	Helena Líbalová, Kateřina	Global gene expression changes in human	Particle and Fibre Toxicology	2012
PAHs	HERC5	Helena Líbalová, Kateřina	Global gene expression changes in human	Particle and Fibre Toxicology	2012
PAHs	HEY1	Helena Líbalová, Kateřina	Global gene expression changes in human	Particle and Fibre Toxicology	2012
PAHs	HMOX1	Helena Líbalová, Kateřina	Global gene expression changes in human	Particle and Fibre Toxicology	2012
PAHs	HNRNPA1	Helena Líbalová, Kateřina	Global gene expression changes in human	Particle and Fibre Toxicology	2012
PAHs	HOXB3	Helena Líbalová, Kateřina	Global gene expression changes in human	Particle and Fibre Toxicology	2012
PAHs	HSPA1A	Helena Líbalová, Kateřina	Global gene expression changes in human	Particle and Fibre Toxicology	2012
PAHs	HSPH1	Helena Líbalová, Kateřina	Global gene expression changes in human	Particle and Fibre Toxicology	2012
PAHs	IFI35	Helena Líbalová, Kateřina	Global gene expression changes in human	Particle and Fibre Toxicology	2012
PAHs	IFT172	Helena Líbalová, Kateřina	Global gene expression changes in human	Particle and Fibre Toxicology	2012
PAHs	IFT57	Helena Líbalová, Kateřina	Global gene expression changes in human	Particle and Fibre Toxicology	2012
PAHs	IGF2R	Helena Líbalová, Kateřina	Global gene expression changes in human	Particle and Fibre Toxicology	2012
PAHs	IKBKE	Helena Líbalová, Kateřina	Global gene expression changes in human	Particle and Fibre Toxicology	2012
PAHs	IL27RA	Helena Líbalová, Kateřina	Global gene expression changes in human	Particle and Fibre Toxicology	2012
PAHs	IL7R	Helena Líbalová, Kateřina	Global gene expression changes in human	Particle and Fibre Toxicology	2012
PAHs	INO80B	Helena Líbalová, Kateřina	Global gene expression changes in human	Particle and Fibre Toxicology	2012
PAHs	INPP1	Helena Líbalová, Kateřina	Global gene expression changes in human	Particle and Fibre Toxicology	2012
PAHs	INPP4B	Helena Líbalová, Kateřina	Global gene expression changes in human	Particle and Fibre Toxicology	2012
PAHs	ITGA2	Helena Líbalová, Kateřina	Global gene expression changes in human	Particle and Fibre Toxicology	2012
PAHs	ITGB1BP1	Helena Líbalová, Kateřina	Global gene expression changes in human	Particle and Fibre Toxicology	2012
PAHs	KLF11	Helena Líbalová, Kateřina	Global gene expression changes in human	Particle and Fibre Toxicology	2012
PAHs	KLHL21	Helena Líbalová, Kateřina	Global gene expression changes in human	Particle and Fibre Toxicology	2012
PAHs	LOC339344	Helena Líbalová, Kateřina	Global gene expression changes in human	Particle and Fibre Toxicology	2012
PAHs	LOC387763	Helena Líbalová, Kateřina	Global gene expression changes in human	Particle and Fibre Toxicology	2012
PAHs	LOC404266	Helena Líbalová, Kateřina	Global gene expression changes in human	Particle and Fibre Toxicology	2012
PAHs	LOC646723	Helena Líbalová, Kateřina	Global gene expression changes in human	Particle and Fibre Toxicology	2012
PAHs	LOC652595	Helena Líbalová, Kateřina	Global gene expression changes in human	Particle and Fibre Toxicology	2012
PAHs	LOC653377	Helena Líbalová, Kateřina	Global gene expression changes in human	Particle and Fibre Toxicology	2012
PAHs	LOC731314	Helena Líbalová, Kateřina	Global gene expression changes in human	Particle and Fibre Toxicology	2012
PAHs	LONRF1	Helena Líbalová, Kateřina	Global gene expression changes in human	Particle and Fibre Toxicology	2012
PAHs	LPIN2	Helena Líbalová, Kateřina	Global gene expression changes in human	Particle and Fibre Toxicology	2012
PAHs	LPPR4	Helena Líbalová, Kateřina	Global gene expression changes in human	Particle and Fibre Toxicology	2012
PAHs	LRRC49	Helena Líbalová, Kateřina	Global gene expression changes in human	Particle and Fibre Toxicology	2012
PAHs	LRRC58	Helena Líbalová, Kateřina	Global gene expression changes in human	Particle and Fibre Toxicology	2012
PAHs	LZTS1	Helena Líbalová, Kateřina	Global gene expression changes in human	Particle and Fibre Toxicology	2012
PAHs	MARS2	Helena Líbalová, Kateřina	Global gene expression changes in human	Particle and Fibre Toxicology	2012
PAHs	MCEE	Helena Líbalová, Kateřina	Global gene expression changes in human	Particle and Fibre Toxicology	2012
PAHs	MCM10	Helena Líbalová, Kateřina	Global gene expression changes in human	Particle and Fibre Toxicology	2012
PAHs	MED8	Helena Líbalová, Kateřina	Global gene expression changes in human	Particle and Fibre Toxicology	2012
PAHs	MGC10997	Helena Líbalová, Kateřina	Global gene expression changes in human	Particle and Fibre Toxicology	2012
PAHs	MGC4172	Helena Líbalová, Kateřina	Global gene expression changes in human	Particle and Fibre Toxicology	2012
PAHs	MIZF	Helena Líbalová, Kateřina	Global gene expression changes in human	Particle and Fibre Toxicology	2012
PAHs	MRPL34	Helena Líbalová, Kateřina	Global gene expression changes in human	Particle and Fibre Toxicology	2012
PAHs	MSI2	Helena Líbalová, Kateřina	Global gene expression changes in human	Particle and Fibre Toxicology	2012
PAHs	MT1G	Helena Líbalová, Kateřina	Global gene expression changes in human	Particle and Fibre Toxicology	2012

PAHs	MT1X	Helena Líbalová, Kateřina	Global gene expression changes in human	Particle and Fibre Toxicology	2012
PAHs	MT2A	Helena Líbalová, Kateřina	Global gene expression changes in human	Particle and Fibre Toxicology	2012
PAHs	MX2	Helena Líbalová, Kateřina	Global gene expression changes in human	Particle and Fibre Toxicology	2012
PAHs	NAP1L5	Helena Líbalová, Kateřina	Global gene expression changes in human	Particle and Fibre Toxicology	2012
PAHs	NBPF3	Helena Líbalová, Kateřina	Global gene expression changes in human	Particle and Fibre Toxicology	2012
PAHs	NES	Helena Líbalová, Kateřina	Global gene expression changes in human	Particle and Fibre Toxicology	2012
PAHs	NOB1	Helena Líbalová, Kateřina	Global gene expression changes in human	Particle and Fibre Toxicology	2012
PAHs	NPC1	Helena Líbalová, Kateřina	Global gene expression changes in human	Particle and Fibre Toxicology	2012
PAHs	NPR2	Helena Líbalová, Kateřina	Global gene expression changes in human	Particle and Fibre Toxicology	2012
PAHs	NR1D2	Helena Líbalová, Kateřina	Global gene expression changes in human	Particle and Fibre Toxicology	2012
PAHs	OLFML3	Helena Líbalová, Kateřina	Global gene expression changes in human	Particle and Fibre Toxicology	2012
PAHs	OSGIN2	Helena Líbalová, Kateřina	Global gene expression changes in human	Particle and Fibre Toxicology	2012
PAHs	OXA1L	Helena Líbalová, Kateřina	Global gene expression changes in human	Particle and Fibre Toxicology	2012
PAHs	PANX2	Helena Líbalová, Kateřina	Global gene expression changes in human	Particle and Fibre Toxicology	2012
PAHs	PCBP4	Helena Líbalová, Kateřina	Global gene expression changes in human	Particle and Fibre Toxicology	2012
PAHs	PCNX	Helena Líbalová, Kateřina	Global gene expression changes in human	Particle and Fibre Toxicology	2012
PAHs	PDCD6IP	Helena Líbalová, Kateřina	Global gene expression changes in human	Particle and Fibre Toxicology	2012
PAHs	PDE5A	Helena Líbalová, Kateřina	Global gene expression changes in human	Particle and Fibre Toxicology	2012
PAHs	PDIA5	Helena Líbalová, Kateřina	Global gene expression changes in human	Particle and Fibre Toxicology	2012
PAHs	PDLIM1	Helena Líbalová, Kateřina	Global gene expression changes in human	Particle and Fibre Toxicology	2012
PAHs	PELI3	Helena Líbalová, Kateřina	Global gene expression changes in human	Particle and Fibre Toxicology	2012
PAHs	PHF14	Helena Líbalová, Kateřina	Global gene expression changes in human	Particle and Fibre Toxicology	2012
PAHs	PIN4	Helena Líbalová, Kateřina	Global gene expression changes in human	Particle and Fibre Toxicology	2012
PAHs	PINX1	Helena Líbalová, Kateřina	Global gene expression changes in human	Particle and Fibre Toxicology	2012
PAHs	PITPNC1	Helena Líbalová, Kateřina	Global gene expression changes in human	Particle and Fibre Toxicology	2012
PAHs	PLEKHA2	Helena Líbalová, Kateřina	Global gene expression changes in human	Particle and Fibre Toxicology	2012
PAHs	PMP22	Helena Líbalová, Kateřina	Global gene expression changes in human	Particle and Fibre Toxicology	2012
PAHs	PNMA3	Helena Líbalová, Kateřina	Global gene expression changes in human	Particle and Fibre Toxicology	2012
PAHs	POLR1C	Helena Líbalová, Kateřina	Global gene expression changes in human	Particle and Fibre Toxicology	2012
PAHs	POLR2L	Helena Líbalová, Kateřina	Global gene expression changes in human	Particle and Fibre Toxicology	2012
PAHs	PPAP2B	Helena Líbalová, Kateřina	Global gene expression changes in human	Particle and Fibre Toxicology	2012
PAHs	PPP1R15A	Helena Líbalová, Kateřina	Global gene expression changes in human	Particle and Fibre Toxicology	2012
PAHs	PPP2R3B	Helena Líbalová, Kateřina	Global gene expression changes in human	Particle and Fibre Toxicology	2012
PAHs	PQLC3	Helena Líbalová, Kateřina	Global gene expression changes in human	Particle and Fibre Toxicology	2012
PAHs	PRKCD	Helena Líbalová, Kateřina	Global gene expression changes in human	Particle and Fibre Toxicology	2012
PAHs	PSMB9	Helena Líbalová, Kateřina	Global gene expression changes in human	Particle and Fibre Toxicology	2012
PAHs	PWWP2A	Helena Líbalová, Kateřina	Global gene expression changes in human	Particle and Fibre Toxicology	2012
PAHs	RAB23	Helena Líbalová, Kateřina	Global gene expression changes in human	Particle and Fibre Toxicology	2012
PAHs	RAMP1	Helena Líbalová, Kateřina	Global gene expression changes in human	Particle and Fibre Toxicology	2012
PAHs	RARRES1	Helena Líbalová, Kateřina	Global gene expression changes in human	Particle and Fibre Toxicology	2012
PAHs	RBBP8	Helena Líbalová, Kateřina	Global gene expression changes in human	Particle and Fibre Toxicology	2012
PAHs	RBM14	Helena Líbalová, Kateřina	Global gene expression changes in human	Particle and Fibre Toxicology	2012
PAHs	RDH5	Helena Líbalová, Kateřina	Global gene expression changes in human	Particle and Fibre Toxicology	2012
PAHs	RHOT1	Helena Líbalová, Kateřina	Global gene expression changes in human	Particle and Fibre Toxicology	2012
PAHs	RNASEL	Helena Líbalová, Kateřina	Global gene expression changes in human	Particle and Fibre Toxicology	2012
PAHs	RND3	Helena Líbalová, Kateřina	Global gene expression changes in human	Particle and Fibre Toxicology	2012
PAHs	RNF10	Helena Líbalová, Kateřina	Global gene expression changes in human	Particle and Fibre Toxicology	2012
PAHs	RNF149	Helena Líbalová, Kateřina	Global gene expression changes in human	Particle and Fibre Toxicology	2012
PAHs	RUFY1	Helena Líbalová, Kateřina	Global gene expression changes in human	Particle and Fibre Toxicology	2012
PAHs	SBF1	Helena Líbalová, Kateřina	Global gene expression changes in human	Particle and Fibre Toxicology	2012
PAHs	SDPR	Helena Líbalová, Kateřina	Global gene expression changes in human	Particle and Fibre Toxicology	2012
PAHs	SEC24B	Helena Líbalová, Kateřina	Global gene expression changes in human	Particle and Fibre Toxicology	2012
PAHs	SEMA4B	Helena Líbalová, Kateřina	Global gene expression changes in human	Particle and Fibre Toxicology	2012
PAHs	SETMAR	Helena Líbalová, Kateřina	Global gene expression changes in human	Particle and Fibre Toxicology	2012
PAHs	SFRS2	Helena Líbalová, Kateřina	Global gene expression changes in human	Particle and Fibre Toxicology	2012
PAHs	SFRS2B	Helena Líbalová, Kateřina	Global gene expression changes in human	Particle and Fibre Toxicology	2012
PAHs	SH3D19	Helena Líbalová, Kateřina	Global gene expression changes in human	Particle and Fibre Toxicology	2012
PAHs	SHANK3	Helena Líbalová, Kateřina	Global gene expression changes in human	Particle and Fibre Toxicology	2012
PAHs	SHISA3	Helena Líbalová, Kateřina	Global gene expression changes in human	Particle and Fibre Toxicology	2012
PAHs	SHROOM2	Helena Líbalová, Kateřina	Global gene expression changes in human	Particle and Fibre Toxicology	2012
PAHs	SLC10A7	Helena Líbalová, Kateřina	Global gene expression changes in human	Particle and Fibre Toxicology	2012
PAHs	SLC25A12	Helena Líbalová, Kateřina	Global gene expression changes in human	Particle and Fibre Toxicology	2012
PAHs	SLC25A20	Helena Líbalová, Kateřina	Global gene expression changes in human	Particle and Fibre Toxicology	2012
PAHs	SLC25A25	Helena Líbalová, Kateřina	Global gene expression changes in human	Particle and Fibre Toxicology	2012
PAHs	SLC25A28	Helena Líbalová, Kateřina	Global gene expression changes in human	Particle and Fibre Toxicology	2012
PAHs	SLC31A2	Helena Líbalová, Kateřina	Global gene expression changes in human	Particle and Fibre Toxicology	2012
PAHs	SLC35A2	Helena Líbalová, Kateřina	Global gene expression changes in human	Particle and Fibre Toxicology	2012
PAHs	SLC5A6	Helena Líbalová, Kateřina	Global gene expression changes in human	Particle and Fibre Toxicology	2012
PAHs	SLC6A10P	Helena Líbalová, Kateřina	Global gene expression changes in human	Particle and Fibre Toxicology	2012
PAHs	SMTN	Helena Líbalová, Kateřina	Global gene expression changes in human	Particle and Fibre Toxicology	2012
PAHs	SNRPA1	Helena Líbalová, Kateřina	Global gene expression changes in human	Particle and Fibre Toxicology	2012
PAHs	SNX30	Helena Líbalová, Kateřina	Global gene expression changes in human	Particle and Fibre Toxicology	2012
PAHs	SON	Helena Líbalová, Kateřina	Global gene expression changes in human	Particle and Fibre Toxicology	2012
PAHs	SOX15	Helena Líbalová, Kateřina	Global gene expression changes in human	Particle and Fibre Toxicology	2012
PAHs	SPNS1	Helena Líbalová, Kateřina	Global gene expression changes in human	Particle and Fibre Toxicology	2012
PAHs	SPRY2	Helena Líbalová, Kateřina	Global gene expression changes in human	Particle and Fibre Toxicology	2012
PAHs	SQLE	Helena Líbalová, Kateřina	Global gene expression changes in human	Particle and Fibre Toxicology	2012
PAHs	SRA1	Helena Líbalová, Kateřina	Global gene expression changes in human	Particle and Fibre Toxicology	2012
PAHs	STC1	Helena Líbalová, Kateřina	Global gene expression changes in human	Particle and Fibre Toxicology	2012
PAHs	STC2	Helena Líbalová, Kateřina	Global gene expression changes in human	Particle and Fibre Toxicology	2012
PAHs	STX4	Helena Líbalová, Kateřina	Global gene expression changes in human	Particle and Fibre Toxicology	2012
PAHs	SULF2	Helena Líbalová, Kateřina	Global gene expression changes in human	Particle and Fibre Toxicology	2012
PAHs	SYNJ2	Helena Líbalová, Kateřina	Global gene expression changes in human	Particle and Fibre Toxicology	2012
PAHs	TAX1BP3	Helena Líbalová, Kateřina	Global gene expression changes in human	Particle and Fibre Toxicology	2012
PAHs	TBC1D16	Helena Líbalová, Kateřina	Global gene expression changes in human	Particle and Fibre Toxicology	2012
PAHs	TBC1D8B	Helena Líbalová, Kateřina	Global gene expression changes in human	Particle and Fibre Toxicology	2012
PAHs	TCEB2	Helena Líbalová, Kateřina	Global gene expression changes in human	Particle and Fibre Toxicology	2012
PAHs	TCF12	Helena Líbalová, Kateřina	Global gene expression changes in human	Particle and Fibre Toxicology	2012
PAHs	TCTA	Helena Líbalová, Kateřina	Global gene expression changes in human	Particle and Fibre Toxicology	2012

PAHs	TESK1	Helena Líbalová, Kateřina	Global gene expression changes in human	Particle and Fibre Toxicology	2012
PAHs	TESK2	Helena Líbalová, Kateřina	Global gene expression changes in human	Particle and Fibre Toxicology	2012
PAHs	TFPI2	Helena Líbalová, Kateřina	Global gene expression changes in human	Particle and Fibre Toxicology	2012
PAHs	THOC4	Helena Líbalová, Kateřina	Global gene expression changes in human	Particle and Fibre Toxicology	2012
PAHs	TIMM23	Helena Líbalová, Kateřina	Global gene expression changes in human	Particle and Fibre Toxicology	2012
PAHs	TMEM130	Helena Líbalová, Kateřina	Global gene expression changes in human	Particle and Fibre Toxicology	2012
PAHs	TMEM158	Helena Líbalová, Kateřina	Global gene expression changes in human	Particle and Fibre Toxicology	2012
PAHs	TMEM173	Helena Líbalová, Kateřina	Global gene expression changes in human	Particle and Fibre Toxicology	2012
PAHs	TMEM192	Helena Líbalová, Kateřina	Global gene expression changes in human	Particle and Fibre Toxicology	2012
PAHs	TMPO	Helena Líbalová, Kateřina	Global gene expression changes in human	Particle and Fibre Toxicology	2012
PAHs	TNFRSF6B	Helena Líbalová, Kateřina	Global gene expression changes in human	Particle and Fibre Toxicology	2012
PAHs	TNFSF4	Helena Líbalová, Kateřina	Global gene expression changes in human	Particle and Fibre Toxicology	2012
PAHs	TNK2	Helena Líbalová, Kateřina	Global gene expression changes in human	Particle and Fibre Toxicology	2012
PAHs	TPMT	Helena Líbalová, Kateřina	Global gene expression changes in human	Particle and Fibre Toxicology	2012
PAHs	TSPAN9	Helena Líbalová, Kateřina	Global gene expression changes in human	Particle and Fibre Toxicology	2012
PAHs	TUBA1B	Helena Líbalová, Kateřina	Global gene expression changes in human	Particle and Fibre Toxicology	2012
PAHs	TUBB	Helena Líbalová, Kateřina	Global gene expression changes in human	Particle and Fibre Toxicology	2012
PAHs	TUBB3	Helena Líbalová, Kateřina	Global gene expression changes in human	Particle and Fibre Toxicology	2012
PAHs	TUSC1	Helena Líbalová, Kateřina	Global gene expression changes in human	Particle and Fibre Toxicology	2012
PAHs	TXNDC13	Helena Líbalová, Kateřina	Global gene expression changes in human	Particle and Fibre Toxicology	2012
PAHs	UACA	Helena Líbalová, Kateřina	Global gene expression changes in human	Particle and Fibre Toxicology	2012
PAHs	UBE2D2	Helena Líbalová, Kateřina	Global gene expression changes in human	Particle and Fibre Toxicology	2012
PAHs	VPS8	Helena Líbalová, Kateřina	Global gene expression changes in human	Particle and Fibre Toxicology	2012
PAHs	WDR12	Helena Líbalová, Kateřina	Global gene expression changes in human	Particle and Fibre Toxicology	2012
PAHs	WDSUB1	Helena Líbalová, Kateřina	Global gene expression changes in human	Particle and Fibre Toxicology	2012
PAHs	WFDC3	Helena Líbalová, Kateřina	Global gene expression changes in human	Particle and Fibre Toxicology	2012
PAHs	ZFP36	Helena Líbalová, Kateřina	Global gene expression changes in human	Particle and Fibre Toxicology	2012
PAHs	ZMIZ1	Helena Líbalová, Kateřina	Global gene expression changes in human	Particle and Fibre Toxicology	2012
PAHs	ZNF25	Helena Líbalová, Kateřina	Global gene expression changes in human	Particle and Fibre Toxicology	2012
PAHs	ZNF512	Helena Líbalová, Kateřina	Global gene expression changes in human	Particle and Fibre Toxicology	2012
PAHs	ZNF524	Helena Líbalová, Kateřina	Global gene expression changes in human	Particle and Fibre Toxicology	2012
PAHs	ZRANB1	Helena Líbalová, Kateřina	Global gene expression changes in human	Particle and Fibre Toxicology	2012
PAHs	AASS	Helena Líbalová, Kateřina	Global gene expression changes in human	Particle and Fibre Toxicology	2012
PAHs	ABCB6	Helena Líbalová, Kateřina	Global gene expression changes in human	Particle and Fibre Toxicology	2012
PAHs	ABLIM1	Helena Líbalová, Kateřina	Global gene expression changes in human	Particle and Fibre Toxicology	2012
PAHs	ABR	Helena Líbalová, Kateřina	Global gene expression changes in human	Particle and Fibre Toxicology	2012
PAHs	ACSL1	Helena Líbalová, Kateřina	Global gene expression changes in human	Particle and Fibre Toxicology	2012
PAHs	ADORA1	Helena Líbalová, Kateřina	Global gene expression changes in human	Particle and Fibre Toxicology	2012
PAHs	ADORA2B	Helena Líbalová, Kateřina	Global gene expression changes in human	Particle and Fibre Toxicology	2012
PAHs	AFAP1	Helena Líbalová, Kateřina	Global gene expression changes in human	Particle and Fibre Toxicology	2012
PAHs	AFG3L2	Helena Líbalová, Kateřina	Global gene expression changes in human	Particle and Fibre Toxicology	2012
PAHs	AK2	Helena Líbalová, Kateřina	Global gene expression changes in human	Particle and Fibre Toxicology	2012
PAHs	ALDH1A1	Helena Líbalová, Kateřina	Global gene expression changes in human	Particle and Fibre Toxicology	2012
PAHs	ALDOC	Helena Líbalová, Kateřina	Global gene expression changes in human	Particle and Fibre Toxicology	2012
PAHs	AMDHD2	Helena Líbalová, Kateřina	Global gene expression changes in human	Particle and Fibre Toxicology	2012
PAHs	ANXA11	Helena Líbalová, Kateřina	Global gene expression changes in human	Particle and Fibre Toxicology	2012
PAHs	ARL1	Helena Líbalová, Kateřina	Global gene expression changes in human	Particle and Fibre Toxicology	2012
PAHs	ARL2BP	Helena Líbalová, Kateřina	Global gene expression changes in human	Particle and Fibre Toxicology	2012
PAHs	ARPC1A	Helena Líbalová, Kateřina	Global gene expression changes in human	Particle and Fibre Toxicology	2012
PAHs	ATP6V1B1	Helena Líbalová, Kateřina	Global gene expression changes in human	Particle and Fibre Toxicology	2012
PAHs	ATXN10	Helena Líbalová, Kateřina	Global gene expression changes in human	Particle and Fibre Toxicology	2012
PAHs	AXIN1	Helena Líbalová, Kateřina	Global gene expression changes in human	Particle and Fibre Toxicology	2012
PAHs	BATF3	Helena Líbalová, Kateřina	Global gene expression changes in human	Particle and Fibre Toxicology	2012
PAHs	BCL9	Helena Líbalová, Kateřina	Global gene expression changes in human	Particle and Fibre Toxicology	2012
PAHs	BIRC5	Helena Líbalová, Kateřina	Global gene expression changes in human	Particle and Fibre Toxicology	2012
PAHs	BIVM	Helena Líbalová, Kateřina	Global gene expression changes in human	Particle and Fibre Toxicology	2012
PAHs	BRE	Helena Líbalová, Kateřina	Global gene expression changes in human	Particle and Fibre Toxicology	2012
PAHs	BRPF3	Helena Líbalová, Kateřina	Global gene expression changes in human	Particle and Fibre Toxicology	2012
PAHs	C11orf17	Helena Líbalová, Kateřina	Global gene expression changes in human	Particle and Fibre Toxicology	2012
PAHs	C11orf82	Helena Líbalová, Kateřina	Global gene expression changes in human	Particle and Fibre Toxicology	2012
PAHs	C12orf32	Helena Líbalová, Kateřina	Global gene expression changes in human	Particle and Fibre Toxicology	2012
PAHs	C14orf159	Helena Líbalová, Kateřina	Global gene expression changes in human	Particle and Fibre Toxicology	2012
PAHs	C15orf23	Helena Líbalová, Kateřina	Global gene expression changes in human	Particle and Fibre Toxicology	2012
PAHs	C16orf30	Helena Líbalová, Kateřina	Global gene expression changes in human	Particle and Fibre Toxicology	2012
PAHs	C16orf33	Helena Líbalová, Kateřina	Global gene expression changes in human	Particle and Fibre Toxicology	2012
PAHs	C17orf97	Helena Líbalová, Kateřina	Global gene expression changes in human	Particle and Fibre Toxicology	2012
PAHs	C18orf55	Helena Líbalová, Kateřina	Global gene expression changes in human	Particle and Fibre Toxicology	2012
PAHs	C1orf102	Helena Líbalová, Kateřina	Global gene expression changes in human	Particle and Fibre Toxicology	2012
PAHs	C20orf111	Helena Líbalová, Kateřina	Global gene expression changes in human	Particle and Fibre Toxicology	2012
PAHs	C20orf116	Helena Líbalová, Kateřina	Global gene expression changes in human	Particle and Fibre Toxicology	2012
PAHs	C21orf122	Helena Líbalová, Kateřina	Global gene expression changes in human	Particle and Fibre Toxicology	2012
PAHs	C3orf31	Helena Líbalová, Kateřina	Global gene expression changes in human	Particle and Fibre Toxicology	2012
PAHs	C3orf58	Helena Líbalová, Kateřina	Global gene expression changes in human	Particle and Fibre Toxicology	2012
PAHs	C5orf5	Helena Líbalová, Kateřina	Global gene expression changes in human	Particle and Fibre Toxicology	2012
PAHs	C6orf136	Helena Líbalová, Kateřina	Global gene expression changes in human	Particle and Fibre Toxicology	2012
PAHs	C8orf32	Helena Líbalová, Kateřina	Global gene expression changes in human	Particle and Fibre Toxicology	2012
PAHs	C9orf30	Helena Líbalová, Kateřina	Global gene expression changes in human	Particle and Fibre Toxicology	2012
PAHs	CALM1	Helena Líbalová, Kateřina	Global gene expression changes in human	Particle and Fibre Toxicology	2012
PAHs	CCDC58	Helena Líbalová, Kateřina	Global gene expression changes in human	Particle and Fibre Toxicology	2012
PAHs	CCDC90A	Helena Líbalová, Kateřina	Global gene expression changes in human	Particle and Fibre Toxicology	2012
PAHs	CCNA2	Helena Líbalová, Kateřina	Global gene expression changes in human	Particle and Fibre Toxicology	2012
PAHs	CD47	Helena Líbalová, Kateřina	Global gene expression changes in human	Particle and Fibre Toxicology	2012
PAHs	CDCA8	Helena Líbalová, Kateřina	Global gene expression changes in human	Particle and Fibre Toxicology	2012
PAHs	CDH11	Helena Líbalová, Kateřina	Global gene expression changes in human	Particle and Fibre Toxicology	2012
PAHs	CDK5R1	Helena Líbalová, Kateřina	Global gene expression changes in human	Particle and Fibre Toxicology	2012
PAHs	CDK5RAP3	Helena Líbalová, Kateřina	Global gene expression changes in human	Particle and Fibre Toxicology	2012
PAHs	CDKN2C	Helena Líbalová, Kateřina	Global gene expression changes in human	Particle and Fibre Toxicology	2012
PAHs	CDR2L	Helena Líbalová, Kateřina	Global gene expression changes in human	Particle and Fibre Toxicology	2012
PAHs	CEP55	Helena Líbalová, Kateřina	Global gene expression changes in human	Particle and Fibre Toxicology	2012

PAHs	CHN1	Helena Líbalová, Kateřina	Global gene expression changes in human	Particle and Fibre Toxicology	2012
PAHs	CLK3	Helena Líbalová, Kateřina	Global gene expression changes in human	Particle and Fibre Toxicology	2012
PAHs	CMTM8	Helena Líbalová, Kateřina	Global gene expression changes in human	Particle and Fibre Toxicology	2012
PAHs	CNKSR3	Helena Líbalová, Kateřina	Global gene expression changes in human	Particle and Fibre Toxicology	2012
PAHs	COASY	Helena Líbalová, Kateřina	Global gene expression changes in human	Particle and Fibre Toxicology	2012
PAHs	COL11A1	Helena Líbalová, Kateřina	Global gene expression changes in human	Particle and Fibre Toxicology	2012
PAHs	COL14A1	Helena Líbalová, Kateřina	Global gene expression changes in human	Particle and Fibre Toxicology	2012
PAHs	COL1A2	Helena Líbalová, Kateřina	Global gene expression changes in human	Particle and Fibre Toxicology	2012
PAHs	COL4A6	Helena Líbalová, Kateřina	Global gene expression changes in human	Particle and Fibre Toxicology	2012
PAHs	COPB2	Helena Líbalová, Kateřina	Global gene expression changes in human	Particle and Fibre Toxicology	2012
PAHs	CREB3L2	Helena Líbalová, Kateřina	Global gene expression changes in human	Particle and Fibre Toxicology	2012
PAHs	CRMP1	Helena Líbalová, Kateřina	Global gene expression changes in human	Particle and Fibre Toxicology	2012
PAHs	CRNKL1	Helena Líbalová, Kateřina	Global gene expression changes in human	Particle and Fibre Toxicology	2012
PAHs	CTNNBIP1	Helena Líbalová, Kateřina	Global gene expression changes in human	Particle and Fibre Toxicology	2012
PAHs	CXCL16	Helena Líbalová, Kateřina	Global gene expression changes in human	Particle and Fibre Toxicology	2012
PAHs	CXXC5	Helena Líbalová, Kateřina	Global gene expression changes in human	Particle and Fibre Toxicology	2012
PAHs	CYB5B	Helena Líbalová, Kateřina	Global gene expression changes in human	Particle and Fibre Toxicology	2012
PAHs	CYCS	Helena Líbalová, Kateřina	Global gene expression changes in human	Particle and Fibre Toxicology	2012
PAHs	CYFIP2	Helena Líbalová, Kateřina	Global gene expression changes in human	Particle and Fibre Toxicology	2012
PAHs	CYP27A1	Helena Líbalová, Kateřina	Global gene expression changes in human	Particle and Fibre Toxicology	2012
PAHs	DACT1	Helena Líbalová, Kateřina	Global gene expression changes in human	Particle and Fibre Toxicology	2012
PAHs	DENND4A	Helena Líbalová, Kateřina	Global gene expression changes in human	Particle and Fibre Toxicology	2012
PAHs	DEPDC6	Helena Líbalová, Kateřina	Global gene expression changes in human	Particle and Fibre Toxicology	2012
PAHs	DHCR24	Helena Líbalová, Kateřina	Global gene expression changes in human	Particle and Fibre Toxicology	2012
PAHs	DHX30	Helena Líbalová, Kateřina	Global gene expression changes in human	Particle and Fibre Toxicology	2012
PAHs	DHX32	Helena Líbalová, Kateřina	Global gene expression changes in human	Particle and Fibre Toxicology	2012
PAHs	DHX37	Helena Líbalová, Kateřina	Global gene expression changes in human	Particle and Fibre Toxicology	2012
PAHs	DIXDC1	Helena Líbalová, Kateřina	Global gene expression changes in human	Particle and Fibre Toxicology	2012
PAHs	dJ341D10.1	Helena Líbalová, Kateřina	Global gene expression changes in human	Particle and Fibre Toxicology	2012
PAHs	DLG4	Helena Líbalová, Kateřina	Global gene expression changes in human	Particle and Fibre Toxicology	2012
PAHs	DVL2	Helena Líbalová, Kateřina	Global gene expression changes in human	Particle and Fibre Toxicology	2012
PAHs	DYNLL1	Helena Líbalová, Kateřina	Global gene expression changes in human	Particle and Fibre Toxicology	2012
PAHs	E2F5	Helena Líbalová, Kateřina	Global gene expression changes in human	Particle and Fibre Toxicology	2012
PAHs	E2F7	Helena Líbalová, Kateřina	Global gene expression changes in human	Particle and Fibre Toxicology	2012
PAHs	EDC3	Helena Líbalová, Kateřina	Global gene expression changes in human	Particle and Fibre Toxicology	2012
PAHs	EFHD2	Helena Líbalová, Kateřina	Global gene expression changes in human	Particle and Fibre Toxicology	2012
PAHs	EFR3A	Helena Líbalová, Kateřina	Global gene expression changes in human	Particle and Fibre Toxicology	2012
PAHs	ERAP2	Helena Líbalová, Kateřina	Global gene expression changes in human	Particle and Fibre Toxicology	2012
PAHs	EXTL2	Helena Líbalová, Kateřina	Global gene expression changes in human	Particle and Fibre Toxicology	2012
PAHs	F10	Helena Líbalová, Kateřina	Global gene expression changes in human	Particle and Fibre Toxicology	2012
PAHs	FAM173B	Helena Líbalová, Kateřina	Global gene expression changes in human	Particle and Fibre Toxicology	2012
PAHs	FAM46B	Helena Líbalová, Kateřina	Global gene expression changes in human	Particle and Fibre Toxicology	2012
PAHs	FAM50A	Helena Líbalová, Kateřina	Global gene expression changes in human	Particle and Fibre Toxicology	2012
PAHs	FAM64A	Helena Líbalová, Kateřina	Global gene expression changes in human	Particle and Fibre Toxicology	2012
PAHs	FAM89A	Helena Líbalová, Kateřina	Global gene expression changes in human	Particle and Fibre Toxicology	2012
PAHs	FAM91A1	Helena Líbalová, Kateřina	Global gene expression changes in human	Particle and Fibre Toxicology	2012
PAHs	FBXO4	Helena Líbalová, Kateřina	Global gene expression changes in human	Particle and Fibre Toxicology	2012
PAHs	FBXO5	Helena Líbalová, Kateřina	Global gene expression changes in human	Particle and Fibre Toxicology	2012
PAHs	FBXW5	Helena Líbalová, Kateřina	Global gene expression changes in human	Particle and Fibre Toxicology	2012
PAHs	FEN1	Helena Líbalová, Kateřina	Global gene expression changes in human	Particle and Fibre Toxicology	2012
PAHs	FGF2	Helena Líbalová, Kateřina	Global gene expression changes in human	Particle and Fibre Toxicology	2012
PAHs	FHOD3	Helena Líbalová, Kateřina	Global gene expression changes in human	Particle and Fibre Toxicology	2012
PAHs	FILIP1L	Helena Líbalová, Kateřina	Global gene expression changes in human	Particle and Fibre Toxicology	2012
PAHs	FKBP14	Helena Líbalová, Kateřina	Global gene expression changes in human	Particle and Fibre Toxicology	2012
PAHs	FKBP1A	Helena Líbalová, Kateřina	Global gene expression changes in human	Particle and Fibre Toxicology	2012
PAHs	FLJ20323	Helena Líbalová, Kateřina	Global gene expression changes in human	Particle and Fibre Toxicology	2012
PAHs	FLNB	Helena Líbalová, Kateřina	Global gene expression changes in human	Particle and Fibre Toxicology	2012
PAHs	FOXRED2	Helena Líbalová, Kateřina	Global gene expression changes in human	Particle and Fibre Toxicology	2012
PAHs	FTHL3P	Helena Líbalová, Kateřina	Global gene expression changes in human	Particle and Fibre Toxicology	2012
PAHs	GAB2	Helena Líbalová, Kateřina	Global gene expression changes in human	Particle and Fibre Toxicology	2012
PAHs	GAS7	Helena Líbalová, Kateřina	Global gene expression changes in human	Particle and Fibre Toxicology	2012
PAHs	GATAD1	Helena Líbalová, Kateřina	Global gene expression changes in human	Particle and Fibre Toxicology	2012
PAHs	GFOD1	Helena Líbalová, Kateřina	Global gene expression changes in human	Particle and Fibre Toxicology	2012
PAHs	GINS2	Helena Líbalová, Kateřina	Global gene expression changes in human	Particle and Fibre Toxicology	2012
PAHs	GINS3	Helena Líbalová, Kateřina	Global gene expression changes in human	Particle and Fibre Toxicology	2012
PAHs	GJA3	Helena Líbalová, Kateřina	Global gene expression changes in human	Particle and Fibre Toxicology	2012
PAHs	GNG2	Helena Líbalová, Kateřina	Global gene expression changes in human	Particle and Fibre Toxicology	2012
PAHs	GRINA	Helena Líbalová, Kateřina	Global gene expression changes in human	Particle and Fibre Toxicology	2012
PAHs	GSTM4	Helena Líbalová, Kateřina	Global gene expression changes in human	Particle and Fibre Toxicology	2012
PAHs	GTF3C6	Helena Líbalová, Kateřina	Global gene expression changes in human	Particle and Fibre Toxicology	2012
PAHs	GYG2	Helena Líbalová, Kateřina	Global gene expression changes in human	Particle and Fibre Toxicology	2012
PAHs	HBEGF	Helena Líbalová, Kateřina	Global gene expression changes in human	Particle and Fibre Toxicology	2012
PAHs	HGS	Helena Líbalová, Kateřina	Global gene expression changes in human	Particle and Fibre Toxicology	2012
PAHs	HIST2H2BE	Helena Líbalová, Kateřina	Global gene expression changes in human	Particle and Fibre Toxicology	2012
PAHs	HMGCR	Helena Líbalová, Kateřina	Global gene expression changes in human	Particle and Fibre Toxicology	2012
PAHs	HNRPA2B1	Helena Líbalová, Kateřina	Global gene expression changes in human	Particle and Fibre Toxicology	2012
PAHs	HNRPM	Helena Líbalová, Kateřina	Global gene expression changes in human	Particle and Fibre Toxicology	2012
PAHs	HNRPR	Helena Líbalová, Kateřina	Global gene expression changes in human	Particle and Fibre Toxicology	2012
PAHs	HSPA12A	Helena Líbalová, Kateřina	Global gene expression changes in human	Particle and Fibre Toxicology	2012
PAHs	HTRA2	Helena Líbalová, Kateřina	Global gene expression changes in human	Particle and Fibre Toxicology	2012
PAHs	IL32	Helena Líbalová, Kateřina	Global gene expression changes in human	Particle and Fibre Toxicology	2012
PAHs	IRAK1	Helena Líbalová, Kateřina	Global gene expression changes in human	Particle and Fibre Toxicology	2012
PAHs	ITFG2	Helena Líbalová, Kateřina	Global gene expression changes in human	Particle and Fibre Toxicology	2012
PAHs	ITGA10	Helena Líbalová, Kateřina	Global gene expression changes in human	Particle and Fibre Toxicology	2012
PAHs	KATNAL2	Helena Líbalová, Kateřina	Global gene expression changes in human	Particle and Fibre Toxicology	2012
PAHs	KCNJ2	Helena Líbalová, Kateřina	Global gene expression changes in human	Particle and Fibre Toxicology	2012
PAHs	KCTD10	Helena Líbalová, Kateřina	Global gene expression changes in human	Particle and Fibre Toxicology	2012
PAHs	KIAA0194	Helena Líbalová, Kateřina	Global gene expression changes in human	Particle and Fibre Toxicology	2012
PAHs	KIAA0664	Helena Líbalová, Kateřina	Global gene expression changes in human	Particle and Fibre Toxicology	2012

PAHs	KIAA1949	Helena Líbalová, Kateřina	Global gene expression changes in human	Particle and Fibre Toxicology	2012
PAHs	KIFC2	Helena Líbalová, Kateřina	Global gene expression changes in human	Particle and Fibre Toxicology	2012
PAHs	LDLR	Helena Líbalová, Kateřina	Global gene expression changes in human	Particle and Fibre Toxicology	2012
PAHs	LEMD2	Helena Líbalová, Kateřina	Global gene expression changes in human	Particle and Fibre Toxicology	2012
PAHs	LEPR	Helena Líbalová, Kateřina	Global gene expression changes in human	Particle and Fibre Toxicology	2012
PAHs	LOC149134	Helena Líbalová, Kateřina	Global gene expression changes in human	Particle and Fibre Toxicology	2012
PAHs	LOC338799	Helena Líbalová, Kateřina	Global gene expression changes in human	Particle and Fibre Toxicology	2012
PAHs	LOC652968	Helena Líbalová, Kateřina	Global gene expression changes in human	Particle and Fibre Toxicology	2012
PAHs	LOC653226	Helena Líbalová, Kateřina	Global gene expression changes in human	Particle and Fibre Toxicology	2012
PAHs	LOC654244	Helena Líbalová, Kateřina	Global gene expression changes in human	Particle and Fibre Toxicology	2012
PAHs	LOC727866	Helena Líbalová, Kateřina	Global gene expression changes in human	Particle and Fibre Toxicology	2012
PAHs	LOXL1	Helena Líbalová, Kateřina	Global gene expression changes in human	Particle and Fibre Toxicology	2012
PAHs	LOXL4	Helena Líbalová, Kateřina	Global gene expression changes in human	Particle and Fibre Toxicology	2012
PAHs	LPIN1	Helena Líbalová, Kateřina	Global gene expression changes in human	Particle and Fibre Toxicology	2012
PAHs	LRRC2	Helena Líbalová, Kateřina	Global gene expression changes in human	Particle and Fibre Toxicology	2012
PAHs	LRWD1	Helena Líbalová, Kateřina	Global gene expression changes in human	Particle and Fibre Toxicology	2012
PAHs	LSP1	Helena Líbalová, Kateřina	Global gene expression changes in human	Particle and Fibre Toxicology	2012
PAHs	LTBR	Helena Líbalová, Kateřina	Global gene expression changes in human	Particle and Fibre Toxicology	2012
PAHs	LZTS2	Helena Líbalová, Kateřina	Global gene expression changes in human	Particle and Fibre Toxicology	2012
PAHs	MANSC1	Helena Líbalová, Kateřina	Global gene expression changes in human	Particle and Fibre Toxicology	2012
PAHs	MAP3K4	Helena Líbalová, Kateřina	Global gene expression changes in human	Particle and Fibre Toxicology	2012
PAHs	MASTL	Helena Líbalová, Kateřina	Global gene expression changes in human	Particle and Fibre Toxicology	2012
PAHs	MCOLN1	Helena Líbalová, Kateřina	Global gene expression changes in human	Particle and Fibre Toxicology	2012
PAHs	MED20	Helena Líbalová, Kateřina	Global gene expression changes in human	Particle and Fibre Toxicology	2012
PAHs	MGC24103	Helena Líbalová, Kateřina	Global gene expression changes in human	Particle and Fibre Toxicology	2012
PAHs	MGEA5	Helena Líbalová, Kateřina	Global gene expression changes in human	Particle and Fibre Toxicology	2012
PAHs	MKL1	Helena Líbalová, Kateřina	Global gene expression changes in human	Particle and Fibre Toxicology	2012
PAHs	MKNK2	Helena Líbalová, Kateřina	Global gene expression changes in human	Particle and Fibre Toxicology	2012
PAHs	MORF4L2	Helena Líbalová, Kateřina	Global gene expression changes in human	Particle and Fibre Toxicology	2012
PAHs	MPP5	Helena Líbalová, Kateřina	Global gene expression changes in human	Particle and Fibre Toxicology	2012
PAHs	MR1	Helena Líbalová, Kateřina	Global gene expression changes in human	Particle and Fibre Toxicology	2012
PAHs	MSC	Helena Líbalová, Kateřina	Global gene expression changes in human	Particle and Fibre Toxicology	2012
PAHs	MSRB3	Helena Líbalová, Kateřina	Global gene expression changes in human	Particle and Fibre Toxicology	2012
PAHs	MT1E	Helena Líbalová, Kateřina	Global gene expression changes in human	Particle and Fibre Toxicology	2012
PAHs	MXD4	Helena Líbalová, Kateřina	Global gene expression changes in human	Particle and Fibre Toxicology	2012
PAHs	MYLIP	Helena Líbalová, Kateřina	Global gene expression changes in human	Particle and Fibre Toxicology	2012
PAHs	MYO6	Helena Líbalová, Kateřina	Global gene expression changes in human	Particle and Fibre Toxicology	2012
PAHs	MYST1	Helena Líbalová, Kateřina	Global gene expression changes in human	Particle and Fibre Toxicology	2012
PAHs	NAGPA	Helena Líbalová, Kateřina	Global gene expression changes in human	Particle and Fibre Toxicology	2012
PAHs	NAT1	Helena Líbalová, Kateřina	Global gene expression changes in human	Particle and Fibre Toxicology	2012
PAHs	NCDN	Helena Líbalová, Kateřina	Global gene expression changes in human	Particle and Fibre Toxicology	2012
PAHs	NCOA6	Helena Líbalová, Kateřina	Global gene expression changes in human	Particle and Fibre Toxicology	2012
PAHs	NDC80	Helena Líbalová, Kateřina	Global gene expression changes in human	Particle and Fibre Toxicology	2012
PAHs	NEDD9	Helena Líbalová, Kateřina	Global gene expression changes in human	Particle and Fibre Toxicology	2012
PAHs	NFATC4	Helena Líbalová, Kateřina	Global gene expression changes in human	Particle and Fibre Toxicology	2012
PAHs	NIP30	Helena Líbalová, Kateřina	Global gene expression changes in human	Particle and Fibre Toxicology	2012
PAHs	NME1	Helena Líbalová, Kateřina	Global gene expression changes in human	Particle and Fibre Toxicology	2012
PAHs	NNAT	Helena Líbalová, Kateřina	Global gene expression changes in human	Particle and Fibre Toxicology	2012
PAHs	NOL1	Helena Líbalová, Kateřina	Global gene expression changes in human	Particle and Fibre Toxicology	2012
PAHs	NR2C2AP	Helena Líbalová, Kateřina	Global gene expression changes in human	Particle and Fibre Toxicology	2012
PAHs	NT5DC2	Helena Líbalová, Kateřina	Global gene expression changes in human	Particle and Fibre Toxicology	2012
PAHs	NTAN1	Helena Líbalová, Kateřina	Global gene expression changes in human	Particle and Fibre Toxicology	2012
PAHs	NTF3	Helena Líbalová, Kateřina	Global gene expression changes in human	Particle and Fibre Toxicology	2012
PAHs	NTN1	Helena Líbalová, Kateřina	Global gene expression changes in human	Particle and Fibre Toxicology	2012
PAHs	NUDT1	Helena Líbalová, Kateřina	Global gene expression changes in human	Particle and Fibre Toxicology	2012
PAHs	NUDT11	Helena Líbalová, Kateřina	Global gene expression changes in human	Particle and Fibre Toxicology	2012
PAHs	OGG1	Helena Líbalová, Kateřina	Global gene expression changes in human	Particle and Fibre Toxicology	2012
PAHs	PACS2	Helena Líbalová, Kateřina	Global gene expression changes in human	Particle and Fibre Toxicology	2012
PAHs	PACSIN2	Helena Líbalová, Kateřina	Global gene expression changes in human	Particle and Fibre Toxicology	2012
PAHs	PARP6	Helena Líbalová, Kateřina	Global gene expression changes in human	Particle and Fibre Toxicology	2012
PAHs	PARP9	Helena Líbalová, Kateřina	Global gene expression changes in human	Particle and Fibre Toxicology	2012
PAHs	PARVA	Helena Líbalová, Kateřina	Global gene expression changes in human	Particle and Fibre Toxicology	2012
PAHs	PDS5A	Helena Líbalová, Kateřina	Global gene expression changes in human	Particle and Fibre Toxicology	2012
PAHs	PELI2	Helena Líbalová, Kateřina	Global gene expression changes in human	Particle and Fibre Toxicology	2012
PAHs	PEPD	Helena Líbalová, Kateřina	Global gene expression changes in human	Particle and Fibre Toxicology	2012
PAHs	PGM2L1	Helena Líbalová, Kateřina	Global gene expression changes in human	Particle and Fibre Toxicology	2012
PAHs	PHF16	Helena Líbalová, Kateřina	Global gene expression changes in human	Particle and Fibre Toxicology	2012
PAHs	PIAS2	Helena Líbalová, Kateřina	Global gene expression changes in human	Particle and Fibre Toxicology	2012
PAHs	PITPNB	Helena Líbalová, Kateřina	Global gene expression changes in human	Particle and Fibre Toxicology	2012
PAHs	PKNOX2	Helena Líbalová, Kateřina	Global gene expression changes in human	Particle and Fibre Toxicology	2012
PAHs	PLOD2	Helena Líbalová, Kateřina	Global gene expression changes in human	Particle and Fibre Toxicology	2012
PAHs	POLR3H	Helena Líbalová, Kateřina	Global gene expression changes in human	Particle and Fibre Toxicology	2012
PAHs	POMT2	Helena Líbalová, Kateřina	Global gene expression changes in human	Particle and Fibre Toxicology	2012
PAHs	PPP2CA	Helena Líbalová, Kateřina	Global gene expression changes in human	Particle and Fibre Toxicology	2012
PAHs	PPP2R2B	Helena Líbalová, Kateřina	Global gene expression changes in human	Particle and Fibre Toxicology	2012
PAHs	PPP3CC	Helena Líbalová, Kateřina	Global gene expression changes in human	Particle and Fibre Toxicology	2012
PAHs	PPTC7	Helena Líbalová, Kateřina	Global gene expression changes in human	Particle and Fibre Toxicology	2012
PAHs	PRC1	Helena Líbalová, Kateřina	Global gene expression changes in human	Particle and Fibre Toxicology	2012
PAHs	PREPL	Helena Líbalová, Kateřina	Global gene expression changes in human	Particle and Fibre Toxicology	2012
PAHs	PRKCDBP	Helena Líbalová, Kateřina	Global gene expression changes in human	Particle and Fibre Toxicology	2012
PAHs	PSMA2	Helena Líbalová, Kateřina	Global gene expression changes in human	Particle and Fibre Toxicology	2012
PAHs	PSPH	Helena Líbalová, Kateřina	Global gene expression changes in human	Particle and Fibre Toxicology	2012
PAHs	PTPLAD1	Helena Líbalová, Kateřina	Global gene expression changes in human	Particle and Fibre Toxicology	2012
PAHs	PUS7	Helena Líbalová, Kateřina	Global gene expression changes in human	Particle and Fibre Toxicology	2012
PAHs	PXMP2	Helena Líbalová, Kateřina	Global gene expression changes in human	Particle and Fibre Toxicology	2012
PAHs	QSOX2	Helena Líbalová, Kateřina	Global gene expression changes in human	Particle and Fibre Toxicology	2012
PAHs	RAB26	Helena Líbalová, Kateřina	Global gene expression changes in human	Particle and Fibre Toxicology	2012
PAHs	RAB40B	Helena Líbalová, Kateřina	Global gene expression changes in human	Particle and Fibre Toxicology	2012
PAHs	RABGGTB	Helena Líbalová, Kateřina	Global gene expression changes in human	Particle and Fibre Toxicology	2012

PAHs	RAD23A	Helena Líbalová, Kateřina	Global gene expression changes in human	Particle and Fibre Toxicology	2012
PAHs	RAD23B	Helena Líbalová, Kateřina	Global gene expression changes in human	Particle and Fibre Toxicology	2012
PAHs	RAPGEF4	Helena Líbalová, Kateřina	Global gene expression changes in human	Particle and Fibre Toxicology	2012
PAHs	RAPGEF6	Helena Líbalová, Kateřina	Global gene expression changes in human	Particle and Fibre Toxicology	2012
PAHs	RARRES2	Helena Líbalová, Kateřina	Global gene expression changes in human	Particle and Fibre Toxicology	2012
PAHs	RB1CC1	Helena Líbalová, Kateřina	Global gene expression changes in human	Particle and Fibre Toxicology	2012
PAHs	RBP1	Helena Líbalová, Kateřina	Global gene expression changes in human	Particle and Fibre Toxicology	2012
PAHs	RCBTB1	Helena Líbalová, Kateřina	Global gene expression changes in human	Particle and Fibre Toxicology	2012
PAHs	RIT1	Helena Líbalová, Kateřina	Global gene expression changes in human	Particle and Fibre Toxicology	2012
PAHs	RND2	Helena Líbalová, Kateřina	Global gene expression changes in human	Particle and Fibre Toxicology	2012
PAHs	RNF126	Helena Líbalová, Kateřina	Global gene expression changes in human	Particle and Fibre Toxicology	2012
PAHs	RNF31	Helena Líbalová, Kateřina	Global gene expression changes in human	Particle and Fibre Toxicology	2012
PAHs	RNGTT	Helena Líbalová, Kateřina	Global gene expression changes in human	Particle and Fibre Toxicology	2012
PAHs	RNMTL1	Helena Líbalová, Kateřina	Global gene expression changes in human	Particle and Fibre Toxicology	2012
PAHs	SAMD9L	Helena Líbalová, Kateřina	Global gene expression changes in human	Particle and Fibre Toxicology	2012
PAHs	SAP18	Helena Líbalová, Kateřina	Global gene expression changes in human	Particle and Fibre Toxicology	2012
PAHs	SCO1	Helena Líbalová, Kateřina	Global gene expression changes in human	Particle and Fibre Toxicology	2012
PAHs	SEPX1	Helena Líbalová, Kateřina	Global gene expression changes in human	Particle and Fibre Toxicology	2012
PAHs	SERPINB2	Helena Líbalová, Kateřina	Global gene expression changes in human	Particle and Fibre Toxicology	2012
PAHs	SERTAD1	Helena Líbalová, Kateřina	Global gene expression changes in human	Particle and Fibre Toxicology	2012
PAHs	SFRS6	Helena Líbalová, Kateřina	Global gene expression changes in human	Particle and Fibre Toxicology	2012
PAHs	SHC4	Helena Líbalová, Kateřina	Global gene expression changes in human	Particle and Fibre Toxicology	2012
PAHs	SLC22A18	Helena Líbalová, Kateřina	Global gene expression changes in human	Particle and Fibre Toxicology	2012
PAHs	SLC24A1	Helena Líbalová, Kateřina	Global gene expression changes in human	Particle and Fibre Toxicology	2012
PAHs	SLC24A6	Helena Líbalová, Kateřina	Global gene expression changes in human	Particle and Fibre Toxicology	2012
PAHs	SLC25A13	Helena Líbalová, Kateřina	Global gene expression changes in human	Particle and Fibre Toxicology	2012
PAHs	SLC25A38	Helena Líbalová, Kateřina	Global gene expression changes in human	Particle and Fibre Toxicology	2012
PAHs	SLC25A5	Helena Líbalová, Kateřina	Global gene expression changes in human	Particle and Fibre Toxicology	2012
PAHs	SLC26A11	Helena Líbalová, Kateřina	Global gene expression changes in human	Particle and Fibre Toxicology	2012
PAHs	SLC2A1	Helena Líbalová, Kateřina	Global gene expression changes in human	Particle and Fibre Toxicology	2012
PAHs	SLC30A5	Helena Líbalová, Kateřina	Global gene expression changes in human	Particle and Fibre Toxicology	2012
PAHs	SLC3A2	Helena Líbalová, Kateřina	Global gene expression changes in human	Particle and Fibre Toxicology	2012
PAHs	SLCO2A1	Helena Líbalová, Kateřina	Global gene expression changes in human	Particle and Fibre Toxicology	2012
PAHs	SLCO3A1	Helena Líbalová, Kateřina	Global gene expression changes in human	Particle and Fibre Toxicology	2012
PAHs	SPATA7	Helena Líbalová, Kateřina	Global gene expression changes in human	Particle and Fibre Toxicology	2012
PAHs	SPIRE1	Helena Líbalová, Kateřina	Global gene expression changes in human	Particle and Fibre Toxicology	2012
PAHs	SPTAN1	Helena Líbalová, Kateřina	Global gene expression changes in human	Particle and Fibre Toxicology	2012
PAHs	SRGN	Helena Líbalová, Kateřina	Global gene expression changes in human	Particle and Fibre Toxicology	2012
PAHs	SRP54	Helena Líbalová, Kateřina	Global gene expression changes in human	Particle and Fibre Toxicology	2012
PAHs	SRPX	Helena Líbalová, Kateřina	Global gene expression changes in human	Particle and Fibre Toxicology	2012
PAHs	STAU2	Helena Líbalová, Kateřina	Global gene expression changes in human	Particle and Fibre Toxicology	2012
PAHs	SUB1	Helena Líbalová, Kateřina	Global gene expression changes in human	Particle and Fibre Toxicology	2012
PAHs	SULT1A3	Helena Líbalová, Kateřina	Global gene expression changes in human	Particle and Fibre Toxicology	2012
PAHs	TAF9L	Helena Líbalová, Kateřina	Global gene expression changes in human	Particle and Fibre Toxicology	2012
PAHs	TAGLN3	Helena Líbalová, Kateřina	Global gene expression changes in human	Particle and Fibre Toxicology	2012
PAHs	TCF21	Helena Líbalová, Kateřina	Global gene expression changes in human	Particle and Fibre Toxicology	2012
PAHs	TEF	Helena Líbalová, Kateřina	Global gene expression changes in human	Particle and Fibre Toxicology	2012
PAHs	TFDP1	Helena Líbalová, Kateřina	Global gene expression changes in human	Particle and Fibre Toxicology	2012
PAHs	TICAM2	Helena Líbalová, Kateřina	Global gene expression changes in human	Particle and Fibre Toxicology	2012
PAHs	TK1	Helena Líbalová, Kateřina	Global gene expression changes in human	Particle and Fibre Toxicology	2012
PAHs	TM7SF2	Helena Líbalová, Kateřina	Global gene expression changes in human	Particle and Fibre Toxicology	2012
PAHs	TMBIM1	Helena Líbalová, Kateřina	Global gene expression changes in human	Particle and Fibre Toxicology	2012
PAHs	TMEM166	Helena Líbalová, Kateřina	Global gene expression changes in human	Particle and Fibre Toxicology	2012
PAHs	TMEM55B	Helena Líbalová, Kateřina	Global gene expression changes in human	Particle and Fibre Toxicology	2012
PAHs	TMEM97	Helena Líbalová, Kateřina	Global gene expression changes in human	Particle and Fibre Toxicology	2012
PAHs	TNFAIP2	Helena Líbalová, Kateřina	Global gene expression changes in human	Particle and Fibre Toxicology	2012
PAHs	TNFRSF21	Helena Líbalová, Kateřina	Global gene expression changes in human	Particle and Fibre Toxicology	2012
PAHs	TNS1	Helena Líbalová, Kateřina	Global gene expression changes in human	Particle and Fibre Toxicology	2012
PAHs	TP53INP2	Helena Líbalová, Kateřina	Global gene expression changes in human	Particle and Fibre Toxicology	2012
PAHs	TRIM6	Helena Líbalová, Kateřina	Global gene expression changes in human	Particle and Fibre Toxicology	2012
PAHs	TRO	Helena Líbalová, Kateřina	Global gene expression changes in human	Particle and Fibre Toxicology	2012
PAHs	TSEN54	Helena Líbalová, Kateřina	Global gene expression changes in human	Particle and Fibre Toxicology	2012
PAHs	TTC23	Helena Líbalová, Kateřina	Global gene expression changes in human	Particle and Fibre Toxicology	2012
PAHs	TUBA3D	Helena Líbalová, Kateřina	Global gene expression changes in human	Particle and Fibre Toxicology	2012
PAHs	UBE2N	Helena Líbalová, Kateřina	Global gene expression changes in human	Particle and Fibre Toxicology	2012
PAHs	UNC93B1	Helena Líbalová, Kateřina	Global gene expression changes in human	Particle and Fibre Toxicology	2012
PAHs	USF2	Helena Líbalová, Kateřina	Global gene expression changes in human	Particle and Fibre Toxicology	2012
PAHs	UTP18	Helena Líbalová, Kateřina	Global gene expression changes in human	Particle and Fibre Toxicology	2012
PAHs	VAC14	Helena Líbalová, Kateřina	Global gene expression changes in human	Particle and Fibre Toxicology	2012
PAHs	VAMP1	Helena Líbalová, Kateřina	Global gene expression changes in human	Particle and Fibre Toxicology	2012
PAHs	VASN	Helena Líbalová, Kateřina	Global gene expression changes in human	Particle and Fibre Toxicology	2012
PAHs	WBP2	Helena Líbalová, Kateřina	Global gene expression changes in human	Particle and Fibre Toxicology	2012
PAHs	WDR22	Helena Líbalová, Kateřina	Global gene expression changes in human	Particle and Fibre Toxicology	2012
PAHs	WISP1	Helena Líbalová, Kateřina	Global gene expression changes in human	Particle and Fibre Toxicology	2012
PAHs	XKR8	Helena Líbalová, Kateřina	Global gene expression changes in human	Particle and Fibre Toxicology	2012
PAHs	XPO1	Helena Líbalová, Kateřina	Global gene expression changes in human	Particle and Fibre Toxicology	2012
PAHs	ZC3H4	Helena Líbalová, Kateřina	Global gene expression changes in human	Particle and Fibre Toxicology	2012
PAHs	ZCCHC17	Helena Líbalová, Kateřina	Global gene expression changes in human	Particle and Fibre Toxicology	2012
PAHs	ZDHHC14	Helena Líbalová, Kateřina	Global gene expression changes in human	Particle and Fibre Toxicology	2012
PAHs	ZMYM2	Helena Líbalová, Kateřina	Global gene expression changes in human	Particle and Fibre Toxicology	2012
PAHs	ZNF205	Helena Líbalová, Kateřina	Global gene expression changes in human	Particle and Fibre Toxicology	2012
PAHs	ZNF277	Helena Líbalová, Kateřina	Global gene expression changes in human	Particle and Fibre Toxicology	2012
PAHs	ZNF451	Helena Líbalová, Kateřina	Global gene expression changes in human	Particle and Fibre Toxicology	2012
PAHs	ZNF605	Helena Líbalová, Kateřina	Global gene expression changes in human	Particle and Fibre Toxicology	2012
PAHs	ZNF74	Helena Líbalová, Kateřina	Global gene expression changes in human	Particle and Fibre Toxicology	2012
PAHs	ZNF789	Helena Líbalová, Kateřina	Global gene expression changes in human	Particle and Fibre Toxicology	2012
PAHs	ZNFX1	Helena Líbalová, Kateřina	Global gene expression changes in human	Particle and Fibre Toxicology	2012
PAHs	ZP3	Helena Líbalová, Kateřina	Global gene expression changes in human	Particle and Fibre Toxicology	2012
PAHs	ZRANB2	Helena Líbalová, Kateřina	Global gene expression changes in human	Particle and Fibre Toxicology	2012

PAHs	ZWINT	Helena Líbalová, Kateřina	Global gene expression changes in human	Particle and Fibre Toxicology	2012
PAHs	ABHD2	Helena Líbalová, Kateřina	Global gene expression changes in human	Particle and Fibre Toxicology	2012
PAHs	ABL2	Helena Líbalová, Kateřina	Global gene expression changes in human	Particle and Fibre Toxicology	2012
PAHs	ADAM15	Helena Líbalová, Kateřina	Global gene expression changes in human	Particle and Fibre Toxicology	2012
PAHs	ADIPOR2	Helena Líbalová, Kateřina	Global gene expression changes in human	Particle and Fibre Toxicology	2012
PAHs	AGK	Helena Líbalová, Kateřina	Global gene expression changes in human	Particle and Fibre Toxicology	2012
PAHs	AGPAT6	Helena Líbalová, Kateřina	Global gene expression changes in human	Particle and Fibre Toxicology	2012
PAHs	AIFM2	Helena Líbalová, Kateřina	Global gene expression changes in human	Particle and Fibre Toxicology	2012
PAHs	AKAP8L	Helena Líbalová, Kateřina	Global gene expression changes in human	Particle and Fibre Toxicology	2012
PAHs	ALAD	Helena Líbalová, Kateřina	Global gene expression changes in human	Particle and Fibre Toxicology	2012
PAHs	ALDH3B1	Helena Líbalová, Kateřina	Global gene expression changes in human	Particle and Fibre Toxicology	2012
PAHs	ALS2CR13	Helena Líbalová, Kateřina	Global gene expression changes in human	Particle and Fibre Toxicology	2012
PAHs	ANGPT1	Helena Líbalová, Kateřina	Global gene expression changes in human	Particle and Fibre Toxicology	2012
PAHs	ANKRD16	Helena Líbalová, Kateřina	Global gene expression changes in human	Particle and Fibre Toxicology	2012
PAHs	ANKRD9	Helena Líbalová, Kateřina	Global gene expression changes in human	Particle and Fibre Toxicology	2012
PAHs	ANO10	Helena Líbalová, Kateřina	Global gene expression changes in human	Particle and Fibre Toxicology	2012
PAHs	ANP32B	Helena Líbalová, Kateřina	Global gene expression changes in human	Particle and Fibre Toxicology	2012
PAHs	AP1G1	Helena Líbalová, Kateřina	Global gene expression changes in human	Particle and Fibre Toxicology	2012
PAHs	AP3M1	Helena Líbalová, Kateřina	Global gene expression changes in human	Particle and Fibre Toxicology	2012
PAHs	APBA3	Helena Líbalová, Kateřina	Global gene expression changes in human	Particle and Fibre Toxicology	2012
PAHs	ARG99	Helena Líbalová, Kateřina	Global gene expression changes in human	Particle and Fibre Toxicology	2012
PAHs	ARHGAP19	Helena Líbalová, Kateřina	Global gene expression changes in human	Particle and Fibre Toxicology	2012
PAHs	ARHGAP22	Helena Líbalová, Kateřina	Global gene expression changes in human	Particle and Fibre Toxicology	2012
PAHs	ARID5B	Helena Líbalová, Kateřina	Global gene expression changes in human	Particle and Fibre Toxicology	2012
PAHs	ARIH1	Helena Líbalová, Kateřina	Global gene expression changes in human	Particle and Fibre Toxicology	2012
PAHs	ARS2	Helena Líbalová, Kateřina	Global gene expression changes in human	Particle and Fibre Toxicology	2012
PAHs	ASF1A	Helena Líbalová, Kateřina	Global gene expression changes in human	Particle and Fibre Toxicology	2012
PAHs	ATF3	Helena Líbalová, Kateřina	Global gene expression changes in human	Particle and Fibre Toxicology	2012
PAHs	ATG4A	Helena Líbalová, Kateřina	Global gene expression changes in human	Particle and Fibre Toxicology	2012
PAHs	ATG5	Helena Líbalová, Kateřina	Global gene expression changes in human	Particle and Fibre Toxicology	2012
PAHs	ATIC	Helena Líbalová, Kateřina	Global gene expression changes in human	Particle and Fibre Toxicology	2012
PAHs	ATL2	Helena Líbalová, Kateřina	Global gene expression changes in human	Particle and Fibre Toxicology	2012
PAHs	ATP2B4	Helena Líbalová, Kateřina	Global gene expression changes in human	Particle and Fibre Toxicology	2012
PAHs	ATP2C1	Helena Líbalová, Kateřina	Global gene expression changes in human	Particle and Fibre Toxicology	2012
PAHs	AVPI1	Helena Líbalová, Kateřina	Global gene expression changes in human	Particle and Fibre Toxicology	2012
PAHs	B4GALT3	Helena Líbalová, Kateřina	Global gene expression changes in human	Particle and Fibre Toxicology	2012
PAHs	BAIAP2L1	Helena Líbalová, Kateřina	Global gene expression changes in human	Particle and Fibre Toxicology	2012
PAHs	BBS2	Helena Líbalová, Kateřina	Global gene expression changes in human	Particle and Fibre Toxicology	2012
PAHs	BCAT1	Helena Líbalová, Kateřina	Global gene expression changes in human	Particle and Fibre Toxicology	2012
PAHs	BCKDHB	Helena Líbalová, Kateřina	Global gene expression changes in human	Particle and Fibre Toxicology	2012
PAHs	BCL11A	Helena Líbalová, Kateřina	Global gene expression changes in human	Particle and Fibre Toxicology	2012
PAHs	BCLAF1	Helena Líbalová, Kateřina	Global gene expression changes in human	Particle and Fibre Toxicology	2012
PAHs	BID	Helena Líbalová, Kateřina	Global gene expression changes in human	Particle and Fibre Toxicology	2012
PAHs	BLCAP	Helena Líbalová, Kateřina	Global gene expression changes in human	Particle and Fibre Toxicology	2012
PAHs	BLOC1S2	Helena Líbalová, Kateřina	Global gene expression changes in human	Particle and Fibre Toxicology	2012
PAHs	BMP4	Helena Líbalová, Kateřina	Global gene expression changes in human	Particle and Fibre Toxicology	2012
PAHs	BTN2A2	Helena Líbalová, Kateřina	Global gene expression changes in human	Particle and Fibre Toxicology	2012
PAHs	BXDC1	Helena Líbalová, Kateřina	Global gene expression changes in human	Particle and Fibre Toxicology	2012
PAHs	C12orf23	Helena Líbalová, Kateřina	Global gene expression changes in human	Particle and Fibre Toxicology	2012
PAHs	C12orf4	Helena Líbalová, Kateřina	Global gene expression changes in human	Particle and Fibre Toxicology	2012
PAHs	C14orf172	Helena Líbalová, Kateřina	Global gene expression changes in human	Particle and Fibre Toxicology	2012
PAHs	C14orf173	Helena Líbalová, Kateřina	Global gene expression changes in human	Particle and Fibre Toxicology	2012
PAHs	C17orf44	Helena Líbalová, Kateřina	Global gene expression changes in human	Particle and Fibre Toxicology	2012
PAHs	C17orf48	Helena Líbalová, Kateřina	Global gene expression changes in human	Particle and Fibre Toxicology	2012
PAHs	C18orf54	Helena Líbalová, Kateřina	Global gene expression changes in human	Particle and Fibre Toxicology	2012
PAHs	C1orf198	Helena Líbalová, Kateřina	Global gene expression changes in human	Particle and Fibre Toxicology	2012
PAHs	C1orf21	Helena Líbalová, Kateřina	Global gene expression changes in human	Particle and Fibre Toxicology	2012
PAHs	C1QTNF1	Helena Líbalová, Kateřina	Global gene expression changes in human	Particle and Fibre Toxicology	2012
PAHs	C22orf15	Helena Líbalová, Kateřina	Global gene expression changes in human	Particle and Fibre Toxicology	2012
PAHs	C2orf33	Helena Líbalová, Kateřina	Global gene expression changes in human	Particle and Fibre Toxicology	2012
PAHs	C2orf49	Helena Líbalová, Kateřina	Global gene expression changes in human	Particle and Fibre Toxicology	2012
PAHs	C7orf20	Helena Líbalová, Kateřina	Global gene expression changes in human	Particle and Fibre Toxicology	2012
PAHs	C7orf26	Helena Líbalová, Kateřina	Global gene expression changes in human	Particle and Fibre Toxicology	2012
PAHs	C7orf27	Helena Líbalová, Kateřina	Global gene expression changes in human	Particle and Fibre Toxicology	2012
PAHs	C7orf50	Helena Líbalová, Kateřina	Global gene expression changes in human	Particle and Fibre Toxicology	2012
PAHs	C8orf34	Helena Líbalová, Kateřina	Global gene expression changes in human	Particle and Fibre Toxicology	2012
PAHs	C9orf103	Helena Líbalová, Kateřina	Global gene expression changes in human	Particle and Fibre Toxicology	2012
PAHs	CA11	Helena Líbalová, Kateřina	Global gene expression changes in human	Particle and Fibre Toxicology	2012
PAHs	CACHD1	Helena Líbalová, Kateřina	Global gene expression changes in human	Particle and Fibre Toxicology	2012
PAHs	CACNA1C	Helena Líbalová, Kateřina	Global gene expression changes in human	Particle and Fibre Toxicology	2012
PAHs	CACNA1H	Helena Líbalová, Kateřina	Global gene expression changes in human	Particle and Fibre Toxicology	2012
PAHs	CAMK2D	Helena Líbalová, Kateřina	Global gene expression changes in human	Particle and Fibre Toxicology	2012
PAHs	CAPNS1	Helena Líbalová, Kateřina	Global gene expression changes in human	Particle and Fibre Toxicology	2012
PAHs	CASP7	Helena Líbalová, Kateřina	Global gene expression changes in human	Particle and Fibre Toxicology	2012
PAHs	CCDC107	Helena Líbalová, Kateřina	Global gene expression changes in human	Particle and Fibre Toxicology	2012
PAHs	CCDC130	Helena Líbalová, Kateřina	Global gene expression changes in human	Particle and Fibre Toxicology	2012
PAHs	CCDC146	Helena Líbalová, Kateřina	Global gene expression changes in human	Particle and Fibre Toxicology	2012
PAHs	CCDC86	Helena Líbalová, Kateřina	Global gene expression changes in human	Particle and Fibre Toxicology	2012
PAHs	CCNB1IP1	Helena Líbalová, Kateřina	Global gene expression changes in human	Particle and Fibre Toxicology	2012
PAHs	CCND3	Helena Líbalová, Kateřina	Global gene expression changes in human	Particle and Fibre Toxicology	2012
PAHs	CCNG2	Helena Líbalová, Kateřina	Global gene expression changes in human	Particle and Fibre Toxicology	2012
PAHs	CCRK	Helena Líbalová, Kateřina	Global gene expression changes in human	Particle and Fibre Toxicology	2012
PAHs	CD248	Helena Líbalová, Kateřina	Global gene expression changes in human	Particle and Fibre Toxicology	2012
PAHs	CD83	Helena Líbalová, Kateřina	Global gene expression changes in human	Particle and Fibre Toxicology	2012
PAHs	CDC34	Helena Líbalová, Kateřina	Global gene expression changes in human	Particle and Fibre Toxicology	2012
PAHs	CDC42EP4	Helena Líbalová, Kateřina	Global gene expression changes in human	Particle and Fibre Toxicology	2012
PAHs	CDC45L	Helena Líbalová, Kateřina	Global gene expression changes in human	Particle and Fibre Toxicology	2012
PAHs	CDK6	Helena Líbalová, Kateřina	Global gene expression changes in human	Particle and Fibre Toxicology	2012
PAHs	CECR1	Helena Líbalová, Kateřina	Global gene expression changes in human	Particle and Fibre Toxicology	2012

PAHs	CENPA	Helena Líbalová, Kateřina	Global gene expression changes in human	Particle and Fibre Toxicology	2012
PAHs	CENTD3	Helena Líbalová, Kateřina	Global gene expression changes in human	Particle and Fibre Toxicology	2012
PAHs	CHES1	Helena Líbalová, Kateřina	Global gene expression changes in human	Particle and Fibre Toxicology	2012
PAHs	CHKB	Helena Líbalová, Kateřina	Global gene expression changes in human	Particle and Fibre Toxicology	2012
PAHs	CHPT1	Helena Líbalová, Kateřina	Global gene expression changes in human	Particle and Fibre Toxicology	2012
PAHs	CKAP2	Helena Líbalová, Kateřina	Global gene expression changes in human	Particle and Fibre Toxicology	2012
PAHs	CNIH3	Helena Líbalová, Kateřina	Global gene expression changes in human	Particle and Fibre Toxicology	2012
PAHs	CNOT6	Helena Líbalová, Kateřina	Global gene expression changes in human	Particle and Fibre Toxicology	2012
PAHs	COL3A1	Helena Líbalová, Kateřina	Global gene expression changes in human	Particle and Fibre Toxicology	2012
PAHs	COPS6	Helena Líbalová, Kateřina	Global gene expression changes in human	Particle and Fibre Toxicology	2012
PAHs	COPZ2	Helena Líbalová, Kateřina	Global gene expression changes in human	Particle and Fibre Toxicology	2012
PAHs	CPSF4	Helena Líbalová, Kateřina	Global gene expression changes in human	Particle and Fibre Toxicology	2012
PAHs	CRLF3	Helena Líbalová, Kateřina	Global gene expression changes in human	Particle and Fibre Toxicology	2012
PAHs	CSDC2	Helena Líbalová, Kateřina	Global gene expression changes in human	Particle and Fibre Toxicology	2012
PAHs	CSNK1E	Helena Líbalová, Kateřina	Global gene expression changes in human	Particle and Fibre Toxicology	2012
PAHs	CSNK2A1	Helena Líbalová, Kateřina	Global gene expression changes in human	Particle and Fibre Toxicology	2012
PAHs	CSTF2T	Helena Líbalová, Kateřina	Global gene expression changes in human	Particle and Fibre Toxicology	2012
PAHs	CYB5D2	Helena Líbalová, Kateřina	Global gene expression changes in human	Particle and Fibre Toxicology	2012
PAHs	DAAM2	Helena Líbalová, Kateřina	Global gene expression changes in human	Particle and Fibre Toxicology	2012
PAHs	DCUN1D3	Helena Líbalová, Kateřina	Global gene expression changes in human	Particle and Fibre Toxicology	2012
PAHs	DDIT3	Helena Líbalová, Kateřina	Global gene expression changes in human	Particle and Fibre Toxicology	2012
PAHs	DDX10	Helena Líbalová, Kateřina	Global gene expression changes in human	Particle and Fibre Toxicology	2012
PAHs	DDX39	Helena Líbalová, Kateřina	Global gene expression changes in human	Particle and Fibre Toxicology	2012
PAHs	DDX55	Helena Líbalová, Kateřina	Global gene expression changes in human	Particle and Fibre Toxicology	2012
PAHs	DGKQ	Helena Líbalová, Kateřina	Global gene expression changes in human	Particle and Fibre Toxicology	2012
PAHs	DHPS	Helena Líbalová, Kateřina	Global gene expression changes in human	Particle and Fibre Toxicology	2012
PAHs	DHRS7	Helena Líbalová, Kateřina	Global gene expression changes in human	Particle and Fibre Toxicology	2012
PAHs	DLG7	Helena Líbalová, Kateřina	Global gene expression changes in human	Particle and Fibre Toxicology	2012
PAHs	DNAI2	Helena Líbalová, Kateřina	Global gene expression changes in human	Particle and Fibre Toxicology	2012
PAHs	DNAJC15	Helena Líbalová, Kateřina	Global gene expression changes in human	Particle and Fibre Toxicology	2012
PAHs	DNAL4	Helena Líbalová, Kateřina	Global gene expression changes in human	Particle and Fibre Toxicology	2012
PAHs	DNALI1	Helena Líbalová, Kateřina	Global gene expression changes in human	Particle and Fibre Toxicology	2012
PAHs	DNASE2	Helena Líbalová, Kateřina	Global gene expression changes in human	Particle and Fibre Toxicology	2012
PAHs	DOCK6	Helena Líbalová, Kateřina	Global gene expression changes in human	Particle and Fibre Toxicology	2012
PAHs	DPAGT1	Helena Líbalová, Kateřina	Global gene expression changes in human	Particle and Fibre Toxicology	2012
PAHs	DPP4	Helena Líbalová, Kateřina	Global gene expression changes in human	Particle and Fibre Toxicology	2012
PAHs	DPYSL3	Helena Líbalová, Kateřina	Global gene expression changes in human	Particle and Fibre Toxicology	2012
PAHs	DSCR3	Helena Líbalová, Kateřina	Global gene expression changes in human	Particle and Fibre Toxicology	2012
PAHs	DUS3L	Helena Líbalová, Kateřina	Global gene expression changes in human	Particle and Fibre Toxicology	2012
PAHs	DYRK1A	Helena Líbalová, Kateřina	Global gene expression changes in human	Particle and Fibre Toxicology	2012
PAHs	ECHDC2	Helena Líbalová, Kateřina	Global gene expression changes in human	Particle and Fibre Toxicology	2012
PAHs	EDNRB	Helena Líbalová, Kateřina	Global gene expression changes in human	Particle and Fibre Toxicology	2012
PAHs	EGFLAM	Helena Líbalová, Kateřina	Global gene expression changes in human	Particle and Fibre Toxicology	2012
PAHs	EIF2B4	Helena Líbalová, Kateřina	Global gene expression changes in human	Particle and Fibre Toxicology	2012
PAHs	EIF4EBP3	Helena Líbalová, Kateřina	Global gene expression changes in human	Particle and Fibre Toxicology	2012
PAHs	ENSA	Helena Líbalová, Kateřina	Global gene expression changes in human	Particle and Fibre Toxicology	2012
PAHs	EPHB3	Helena Líbalová, Kateřina	Global gene expression changes in human	Particle and Fibre Toxicology	2012
PAHs	EPSTI1	Helena Líbalová, Kateřina	Global gene expression changes in human	Particle and Fibre Toxicology	2012
PAHs	ESPNL	Helena Líbalová, Kateřina	Global gene expression changes in human	Particle and Fibre Toxicology	2012
PAHs	ETV4	Helena Líbalová, Kateřina	Global gene expression changes in human	Particle and Fibre Toxicology	2012
PAHs	EXOC3	Helena Líbalová, Kateřina	Global gene expression changes in human	Particle and Fibre Toxicology	2012
PAHs	EXOSC5	Helena Líbalová, Kateřina	Global gene expression changes in human	Particle and Fibre Toxicology	2012
PAHs	EYA4	Helena Líbalová, Kateřina	Global gene expression changes in human	Particle and Fibre Toxicology	2012
PAHs	FAM131A	Helena Líbalová, Kateřina	Global gene expression changes in human	Particle and Fibre Toxicology	2012
PAHs	FAM156A	Helena Líbalová, Kateřina	Global gene expression changes in human	Particle and Fibre Toxicology	2012
PAHs	FAM18B	Helena Líbalová, Kateřina	Global gene expression changes in human	Particle and Fibre Toxicology	2012
PAHs	FAM40A	Helena Líbalová, Kateřina	Global gene expression changes in human	Particle and Fibre Toxicology	2012
PAHs	FAM45A	Helena Líbalová, Kateřina	Global gene expression changes in human	Particle and Fibre Toxicology	2012
PAHs	FAM50B	Helena Líbalová, Kateřina	Global gene expression changes in human	Particle and Fibre Toxicology	2012
PAHs	FAM98A	Helena Líbalová, Kateřina	Global gene expression changes in human	Particle and Fibre Toxicology	2012
PAHs	FANCE	Helena Líbalová, Kateřina	Global gene expression changes in human	Particle and Fibre Toxicology	2012
PAHs	FBN2	Helena Líbalová, Kateřina	Global gene expression changes in human	Particle and Fibre Toxicology	2012
PAHs	FBXO46	Helena Líbalová, Kateřina	Global gene expression changes in human	Particle and Fibre Toxicology	2012
PAHs	FBXW4	Helena Líbalová, Kateřina	Global gene expression changes in human	Particle and Fibre Toxicology	2012
PAHs	FGFR1OP	Helena Líbalová, Kateřina	Global gene expression changes in human	Particle and Fibre Toxicology	2012
PAHs	FKBP4	Helena Líbalová, Kateřina	Global gene expression changes in human	Particle and Fibre Toxicology	2012
PAHs	FLJ10986	Helena Líbalová, Kateřina	Global gene expression changes in human	Particle and Fibre Toxicology	2012
PAHs	FLJ23584	Helena Líbalová, Kateřina	Global gene expression changes in human	Particle and Fibre Toxicology	2012
PAHs	FLJ33996	Helena Líbalová, Kateřina	Global gene expression changes in human	Particle and Fibre Toxicology	2012
PAHs	FLVCR2	Helena Líbalová, Kateřina	Global gene expression changes in human	Particle and Fibre Toxicology	2012
PAHs	FNTB	Helena Líbalová, Kateřina	Global gene expression changes in human	Particle and Fibre Toxicology	2012
PAHs	FOXN2	Helena Líbalová, Kateřina	Global gene expression changes in human	Particle and Fibre Toxicology	2012
PAHs	FTHL8	Helena Líbalová, Kateřina	Global gene expression changes in human	Particle and Fibre Toxicology	2012
PAHs	FUSIP1	Helena Líbalová, Kateřina	Global gene expression changes in human	Particle and Fibre Toxicology	2012
PAHs	FYTTD1	Helena Líbalová, Kateřina	Global gene expression changes in human	Particle and Fibre Toxicology	2012
PAHs	FZD4	Helena Líbalová, Kateřina	Global gene expression changes in human	Particle and Fibre Toxicology	2012
PAHs	GALT	Helena Líbalová, Kateřina	Global gene expression changes in human	Particle and Fibre Toxicology	2012
PAHs	GATS	Helena Líbalová, Kateřina	Global gene expression changes in human	Particle and Fibre Toxicology	2012
PAHs	GBE1	Helena Líbalová, Kateřina	Global gene expression changes in human	Particle and Fibre Toxicology	2012
PAHs	GBL	Helena Líbalová, Kateřina	Global gene expression changes in human	Particle and Fibre Toxicology	2012
PAHs	GBP1	Helena Líbalová, Kateřina	Global gene expression changes in human	Particle and Fibre Toxicology	2012
PAHs	GFM1	Helena Líbalová, Kateřina	Global gene expression changes in human	Particle and Fibre Toxicology	2012
PAHs	GGCX	Helena Líbalová, Kateřina	Global gene expression changes in human	Particle and Fibre Toxicology	2012
PAHs	GLIPR2	Helena Líbalová, Kateřina	Global gene expression changes in human	Particle and Fibre Toxicology	2012
PAHs	GLT25D1	Helena Líbalová, Kateřina	Global gene expression changes in human	Particle and Fibre Toxicology	2012
PAHs	GOLM1	Helena Líbalová, Kateřina	Global gene expression changes in human	Particle and Fibre Toxicology	2012
PAHs	GPATCH3	Helena Líbalová, Kateřina	Global gene expression changes in human	Particle and Fibre Toxicology	2012
PAHs	GPRC5A	Helena Líbalová, Kateřina	Global gene expression changes in human	Particle and Fibre Toxicology	2012
PAHs	GRAMD1A	Helena Líbalová, Kateřina	Global gene expression changes in human	Particle and Fibre Toxicology	2012

PAHs	GSPT2	Helena Líbalová, Kateřina	Global gene expression changes in human	Particle and Fibre Toxicology	2012
PAHs	GTSE1	Helena Líbalová, Kateřina	Global gene expression changes in human	Particle and Fibre Toxicology	2012
PAHs	H1FX	Helena Líbalová, Kateřina	Global gene expression changes in human	Particle and Fibre Toxicology	2012
PAHs	HCCS	Helena Líbalová, Kateřina	Global gene expression changes in human	Particle and Fibre Toxicology	2012
PAHs	HERC6	Helena Líbalová, Kateřina	Global gene expression changes in human	Particle and Fibre Toxicology	2012
PAHs	HIBCH	Helena Líbalová, Kateřina	Global gene expression changes in human	Particle and Fibre Toxicology	2012
PAHs	HLX	Helena Líbalová, Kateřina	Global gene expression changes in human	Particle and Fibre Toxicology	2012
PAHs	HMG20A	Helena Líbalová, Kateřina	Global gene expression changes in human	Particle and Fibre Toxicology	2012
PAHs	HNRNPU	Helena Líbalová, Kateřina	Global gene expression changes in human	Particle and Fibre Toxicology	2012
PAHs	HNRPK	Helena Líbalová, Kateřina	Global gene expression changes in human	Particle and Fibre Toxicology	2012
PAHs	HOMER3	Helena Líbalová, Kateřina	Global gene expression changes in human	Particle and Fibre Toxicology	2012
PAHs	HPCAL1	Helena Líbalová, Kateřina	Global gene expression changes in human	Particle and Fibre Toxicology	2012
PAHs	HRSP12	Helena Líbalová, Kateřina	Global gene expression changes in human	Particle and Fibre Toxicology	2012
PAHs	HSPA5	Helena Líbalová, Kateřina	Global gene expression changes in human	Particle and Fibre Toxicology	2012
PAHs	ID3	Helena Líbalová, Kateřina	Global gene expression changes in human	Particle and Fibre Toxicology	2012
PAHs	IFFO1	Helena Líbalová, Kateřina	Global gene expression changes in human	Particle and Fibre Toxicology	2012
PAHs	IFIT2	Helena Líbalová, Kateřina	Global gene expression changes in human	Particle and Fibre Toxicology	2012
PAHs	IFRD2	Helena Líbalová, Kateřina	Global gene expression changes in human	Particle and Fibre Toxicology	2012
PAHs	IGF2BP3	Helena Líbalová, Kateřina	Global gene expression changes in human	Particle and Fibre Toxicology	2012
PAHs	IKIP	Helena Líbalová, Kateřina	Global gene expression changes in human	Particle and Fibre Toxicology	2012
PAHs	IRAK2	Helena Líbalová, Kateřina	Global gene expression changes in human	Particle and Fibre Toxicology	2012
PAHs	ISG20L1	Helena Líbalová, Kateřina	Global gene expression changes in human	Particle and Fibre Toxicology	2012
PAHs	KCNE1L	Helena Líbalová, Kateřina	Global gene expression changes in human	Particle and Fibre Toxicology	2012
PAHs	KIAA0368	Helena Líbalová, Kateřina	Global gene expression changes in human	Particle and Fibre Toxicology	2012
PAHs	KIAA0562	Helena Líbalová, Kateřina	Global gene expression changes in human	Particle and Fibre Toxicology	2012
PAHs	KIAA0913	Helena Líbalová, Kateřina	Global gene expression changes in human	Particle and Fibre Toxicology	2012
PAHs	KIAA1737	Helena Líbalová, Kateřina	Global gene expression changes in human	Particle and Fibre Toxicology	2012
PAHs	KIF2C	Helena Líbalová, Kateřina	Global gene expression changes in human	Particle and Fibre Toxicology	2012
PAHs	KIF4A	Helena Líbalová, Kateřina	Global gene expression changes in human	Particle and Fibre Toxicology	2012
PAHs	KIFC1	Helena Líbalová, Kateřina	Global gene expression changes in human	Particle and Fibre Toxicology	2012
PAHs	KLC1	Helena Líbalová, Kateřina	Global gene expression changes in human	Particle and Fibre Toxicology	2012
PAHs	KLF12	Helena Líbalová, Kateřina	Global gene expression changes in human	Particle and Fibre Toxicology	2012
PAHs	KLF9	Helena Líbalová, Kateřina	Global gene expression changes in human	Particle and Fibre Toxicology	2012
PAHs	KLHL17	Helena Líbalová, Kateřina	Global gene expression changes in human	Particle and Fibre Toxicology	2012
PAHs	KLHL3	Helena Líbalová, Kateřina	Global gene expression changes in human	Particle and Fibre Toxicology	2012
PAHs	LAMA4	Helena Líbalová, Kateřina	Global gene expression changes in human	Particle and Fibre Toxicology	2012
PAHs	LANCL1	Helena Líbalová, Kateřina	Global gene expression changes in human	Particle and Fibre Toxicology	2012
PAHs	LARP1	Helena Líbalová, Kateřina	Global gene expression changes in human	Particle and Fibre Toxicology	2012
PAHs	LARP2	Helena Líbalová, Kateřina	Global gene expression changes in human	Particle and Fibre Toxicology	2012
PAHs	LASS2	Helena Líbalová, Kateřina	Global gene expression changes in human	Particle and Fibre Toxicology	2012
PAHs	LHFPL2	Helena Líbalová, Kateřina	Global gene expression changes in human	Particle and Fibre Toxicology	2012
PAHs	LIMS1	Helena Líbalová, Kateřina	Global gene expression changes in human	Particle and Fibre Toxicology	2012
PAHs	LIN7A	Helena Líbalová, Kateřina	Global gene expression changes in human	Particle and Fibre Toxicology	2012
PAHs	LMAN2L	Helena Líbalová, Kateřina	Global gene expression changes in human	Particle and Fibre Toxicology	2012
PAHs	LMCD1	Helena Líbalová, Kateřina	Global gene expression changes in human	Particle and Fibre Toxicology	2012
PAHs	LMNB1	Helena Líbalová, Kateřina	Global gene expression changes in human	Particle and Fibre Toxicology	2012
PAHs	LMOD1	Helena Líbalová, Kateřina	Global gene expression changes in human	Particle and Fibre Toxicology	2012
PAHs	LOC401052	Helena Líbalová, Kateřina	Global gene expression changes in human	Particle and Fibre Toxicology	2012
PAHs	LOC401074	Helena Líbalová, Kateřina	Global gene expression changes in human	Particle and Fibre Toxicology	2012
PAHs	LOC442597	Helena Líbalová, Kateřina	Global gene expression changes in human	Particle and Fibre Toxicology	2012
PAHs	LOC641765	Helena Líbalová, Kateřina	Global gene expression changes in human	Particle and Fibre Toxicology	2012
PAHs	LOC641804	Helena Líbalová, Kateřina	Global gene expression changes in human	Particle and Fibre Toxicology	2012
PAHs	LOC642897	Helena Líbalová, Kateřina	Global gene expression changes in human	Particle and Fibre Toxicology	2012
PAHs	LOC644033	Helena Líbalová, Kateřina	Global gene expression changes in human	Particle and Fibre Toxicology	2012
PAHs	LOC645236	Helena Líbalová, Kateřina	Global gene expression changes in human	Particle and Fibre Toxicology	2012
PAHs	LOC645362	Helena Líbalová, Kateřina	Global gene expression changes in human	Particle and Fibre Toxicology	2012
PAHs	LOC645937	Helena Líbalová, Kateřina	Global gene expression changes in human	Particle and Fibre Toxicology	2012
PAHs	LOC647346	Helena Líbalová, Kateřina	Global gene expression changes in human	Particle and Fibre Toxicology	2012
PAHs	LOC653210	Helena Líbalová, Kateřina	Global gene expression changes in human	Particle and Fibre Toxicology	2012
PAHs	LOC728014	Helena Líbalová, Kateřina	Global gene expression changes in human	Particle and Fibre Toxicology	2012
PAHs	LOC729101	Helena Líbalová, Kateřina	Global gene expression changes in human	Particle and Fibre Toxicology	2012
PAHs	LOC93622	Helena Líbalová, Kateřina	Global gene expression changes in human	Particle and Fibre Toxicology	2012
PAHs	LRRN3	Helena Líbalová, Kateřina	Global gene expression changes in human	Particle and Fibre Toxicology	2012
PAHs	LSM3	Helena Líbalová, Kateřina	Global gene expression changes in human	Particle and Fibre Toxicology	2012
PAHs	LUZP1	Helena Líbalová, Kateřina	Global gene expression changes in human	Particle and Fibre Toxicology	2012
PAHs	LYST	Helena Líbalová, Kateřina	Global gene expression changes in human	Particle and Fibre Toxicology	2012
PAHs	MAD2L1	Helena Líbalová, Kateřina	Global gene expression changes in human	Particle and Fibre Toxicology	2012
PAHs	MAP3K2	Helena Líbalová, Kateřina	Global gene expression changes in human	Particle and Fibre Toxicology	2012
PAHs	MAP4K2	Helena Líbalová, Kateřina	Global gene expression changes in human	Particle and Fibre Toxicology	2012
PAHs	MAPK1	Helena Líbalová, Kateřina	Global gene expression changes in human	Particle and Fibre Toxicology	2012
PAHs	MCM3	Helena Líbalová, Kateřina	Global gene expression changes in human	Particle and Fibre Toxicology	2012
PAHs	MCM6	Helena Líbalová, Kateřina	Global gene expression changes in human	Particle and Fibre Toxicology	2012
PAHs	MFHAS1	Helena Líbalová, Kateřina	Global gene expression changes in human	Particle and Fibre Toxicology	2012
PAHs	MFSD3	Helena Líbalová, Kateřina	Global gene expression changes in human	Particle and Fibre Toxicology	2012
PAHs	MGC40489	Helena Líbalová, Kateřina	Global gene expression changes in human	Particle and Fibre Toxicology	2012
PAHs	MGC70857	Helena Líbalová, Kateřina	Global gene expression changes in human	Particle and Fibre Toxicology	2012
PAHs	MGLL	Helena Líbalová, Kateřina	Global gene expression changes in human	Particle and Fibre Toxicology	2012
PAHs	MLX	Helena Líbalová, Kateřina	Global gene expression changes in human	Particle and Fibre Toxicology	2012
PAHs	MOV10	Helena Líbalová, Kateřina	Global gene expression changes in human	Particle and Fibre Toxicology	2012
PAHs	MRPL35	Helena Líbalová, Kateřina	Global gene expression changes in human	Particle and Fibre Toxicology	2012
PAHs	MRPL36	Helena Líbalová, Kateřina	Global gene expression changes in human	Particle and Fibre Toxicology	2012
PAHs	MRPL47	Helena Líbalová, Kateřina	Global gene expression changes in human	Particle and Fibre Toxicology	2012
PAHs	MRPS2	Helena Líbalová, Kateřina	Global gene expression changes in human	Particle and Fibre Toxicology	2012
PAHs	MRPS30	Helena Líbalová, Kateřina	Global gene expression changes in human	Particle and Fibre Toxicology	2012
PAHs	MRPS5	Helena Líbalová, Kateřina	Global gene expression changes in human	Particle and Fibre Toxicology	2012
PAHs	MSN	Helena Líbalová, Kateřina	Global gene expression changes in human	Particle and Fibre Toxicology	2012
PAHs	MTE	Helena Líbalová, Kateřina	Global gene expression changes in human	Particle and Fibre Toxicology	2012
PAHs	MYH10	Helena Líbalová, Kateřina	Global gene expression changes in human	Particle and Fibre Toxicology	2012
PAHs	N6AMT1	Helena Líbalová, Kateřina	Global gene expression changes in human	Particle and Fibre Toxicology	2012

PAHs	NAV2	Helena Líbalová, Kateřina	Global gene expression changes in human	Particle and Fibre Toxicology	2012
PAHs	NBPF10	Helena Líbalová, Kateřina	Global gene expression changes in human	Particle and Fibre Toxicology	2012
PAHs	NCAM2	Helena Líbalová, Kateřina	Global gene expression changes in human	Particle and Fibre Toxicology	2012
PAHs	NDE1	Helena Líbalová, Kateřina	Global gene expression changes in human	Particle and Fibre Toxicology	2012
PAHs	NDN	Helena Líbalová, Kateřina	Global gene expression changes in human	Particle and Fibre Toxicology	2012
PAHs	NET1	Helena Líbalová, Kateřina	Global gene expression changes in human	Particle and Fibre Toxicology	2012
PAHs	NFE2L3	Helena Líbalová, Kateřina	Global gene expression changes in human	Particle and Fibre Toxicology	2012
PAHs	NHLRC2	Helena Líbalová, Kateřina	Global gene expression changes in human	Particle and Fibre Toxicology	2012
PAHs	NOL3	Helena Líbalová, Kateřina	Global gene expression changes in human	Particle and Fibre Toxicology	2012
PAHs	NOL8	Helena Líbalová, Kateřina	Global gene expression changes in human	Particle and Fibre Toxicology	2012
PAHs	NR1H2	Helena Líbalová, Kateřina	Global gene expression changes in human	Particle and Fibre Toxicology	2012
PAHs	NR4A2	Helena Líbalová, Kateřina	Global gene expression changes in human	Particle and Fibre Toxicology	2012
PAHs	NSFL1C	Helena Líbalová, Kateřina	Global gene expression changes in human	Particle and Fibre Toxicology	2012
PAHs	NSMAF	Helena Líbalová, Kateřina	Global gene expression changes in human	Particle and Fibre Toxicology	2012
PAHs	OGFOD1	Helena Líbalová, Kateřina	Global gene expression changes in human	Particle and Fibre Toxicology	2012
PAHs	OPN3	Helena Líbalová, Kateřina	Global gene expression changes in human	Particle and Fibre Toxicology	2012
PAHs	OPTN	Helena Líbalová, Kateřina	Global gene expression changes in human	Particle and Fibre Toxicology	2012
PAHs	OSTF1	Helena Líbalová, Kateřina	Global gene expression changes in human	Particle and Fibre Toxicology	2012
PAHs	OSTM1	Helena Líbalová, Kateřina	Global gene expression changes in human	Particle and Fibre Toxicology	2012
PAHs	OXTR	Helena Líbalová, Kateřina	Global gene expression changes in human	Particle and Fibre Toxicology	2012
PAHs	PACSIN3	Helena Líbalová, Kateřina	Global gene expression changes in human	Particle and Fibre Toxicology	2012
PAHs	PAFAH1B1	Helena Líbalová, Kateřina	Global gene expression changes in human	Particle and Fibre Toxicology	2012
PAHs	PAIP2	Helena Líbalová, Kateřina	Global gene expression changes in human	Particle and Fibre Toxicology	2012
PAHs	PANK1	Helena Líbalová, Kateřina	Global gene expression changes in human	Particle and Fibre Toxicology	2012
PAHs	PAPSS2	Helena Líbalová, Kateřina	Global gene expression changes in human	Particle and Fibre Toxicology	2012
PAHs	PARP1	Helena Líbalová, Kateřina	Global gene expression changes in human	Particle and Fibre Toxicology	2012
PAHs	PATL1	Helena Líbalová, Kateřina	Global gene expression changes in human	Particle and Fibre Toxicology	2012
PAHs	PBRM1	Helena Líbalová, Kateřina	Global gene expression changes in human	Particle and Fibre Toxicology	2012
PAHs	PCBP1	Helena Líbalová, Kateřina	Global gene expression changes in human	Particle and Fibre Toxicology	2012
PAHs	PCNXL2	Helena Líbalová, Kateřina	Global gene expression changes in human	Particle and Fibre Toxicology	2012
PAHs	PDGFD	Helena Líbalová, Kateřina	Global gene expression changes in human	Particle and Fibre Toxicology	2012
PAHs	PDPN	Helena Líbalová, Kateřina	Global gene expression changes in human	Particle and Fibre Toxicology	2012
PAHs	PEX5	Helena Líbalová, Kateřina	Global gene expression changes in human	Particle and Fibre Toxicology	2012
PAHs	PFAAP5	Helena Líbalová, Kateřina	Global gene expression changes in human	Particle and Fibre Toxicology	2012
PAHs	PHKA1	Helena Líbalová, Kateřina	Global gene expression changes in human	Particle and Fibre Toxicology	2012
PAHs	PHKB	Helena Líbalová, Kateřina	Global gene expression changes in human	Particle and Fibre Toxicology	2012
PAHs	PHLDA1	Helena Líbalová, Kateřina	Global gene expression changes in human	Particle and Fibre Toxicology	2012
PAHs	PI4KB	Helena Líbalová, Kateřina	Global gene expression changes in human	Particle and Fibre Toxicology	2012
PAHs	PIGC	Helena Líbalová, Kateřina	Global gene expression changes in human	Particle and Fibre Toxicology	2012
PAHs	PIM3	Helena Líbalová, Kateřina	Global gene expression changes in human	Particle and Fibre Toxicology	2012
PAHs	PITPNM1	Helena Líbalová, Kateřina	Global gene expression changes in human	Particle and Fibre Toxicology	2012
PAHs	PITX1	Helena Líbalová, Kateřina	Global gene expression changes in human	Particle and Fibre Toxicology	2012
PAHs	PKP4	Helena Líbalová, Kateřina	Global gene expression changes in human	Particle and Fibre Toxicology	2012
PAHs	PLD1	Helena Líbalová, Kateřina	Global gene expression changes in human	Particle and Fibre Toxicology	2012
PAHs	PLS3	Helena Líbalová, Kateřina	Global gene expression changes in human	Particle and Fibre Toxicology	2012
PAHs	POMZP3	Helena Líbalová, Kateřina	Global gene expression changes in human	Particle and Fibre Toxicology	2012
PAHs	POP5	Helena Líbalová, Kateřina	Global gene expression changes in human	Particle and Fibre Toxicology	2012
PAHs	PORCN	Helena Líbalová, Kateřina	Global gene expression changes in human	Particle and Fibre Toxicology	2012
PAHs	POSTN	Helena Líbalová, Kateřina	Global gene expression changes in human	Particle and Fibre Toxicology	2012
PAHs	PPHLN1	Helena Líbalová, Kateřina	Global gene expression changes in human	Particle and Fibre Toxicology	2012
PAHs	PPP1CB	Helena Líbalová, Kateřina	Global gene expression changes in human	Particle and Fibre Toxicology	2012
PAHs	PPP2R3A	Helena Líbalová, Kateřina	Global gene expression changes in human	Particle and Fibre Toxicology	2012
PAHs	PRCC	Helena Líbalová, Kateřina	Global gene expression changes in human	Particle and Fibre Toxicology	2012
PAHs	PRDM4	Helena Líbalová, Kateřina	Global gene expression changes in human	Particle and Fibre Toxicology	2012
PAHs	PRMT6	Helena Líbalová, Kateřina	Global gene expression changes in human	Particle and Fibre Toxicology	2012
PAHs	PRPS2	Helena Líbalová, Kateřina	Global gene expression changes in human	Particle and Fibre Toxicology	2012
PAHs	PTPRE	Helena Líbalová, Kateřina	Global gene expression changes in human	Particle and Fibre Toxicology	2012
PAHs	PUS1	Helena Líbalová, Kateřina	Global gene expression changes in human	Particle and Fibre Toxicology	2012
PAHs	PXMP3	Helena Líbalová, Kateřina	Global gene expression changes in human	Particle and Fibre Toxicology	2012
PAHs	RAB11FIP5	Helena Líbalová, Kateřina	Global gene expression changes in human	Particle and Fibre Toxicology	2012
PAHs	RAB1A	Helena Líbalová, Kateřina	Global gene expression changes in human	Particle and Fibre Toxicology	2012
PAHs	RAB8A	Helena Líbalová, Kateřina	Global gene expression changes in human	Particle and Fibre Toxicology	2012
PAHs	RAB9A	Helena Líbalová, Kateřina	Global gene expression changes in human	Particle and Fibre Toxicology	2012
PAHs	RAD51AP1	Helena Líbalová, Kateřina	Global gene expression changes in human	Particle and Fibre Toxicology	2012
PAHs	RBM9	Helena Líbalová, Kateřina	Global gene expression changes in human	Particle and Fibre Toxicology	2012
PAHs	RDH14	Helena Líbalová, Kateřina	Global gene expression changes in human	Particle and Fibre Toxicology	2012
PAHs	REV1	Helena Líbalová, Kateřina	Global gene expression changes in human	Particle and Fibre Toxicology	2012
PAHs	RFX5	Helena Líbalová, Kateřina	Global gene expression changes in human	Particle and Fibre Toxicology	2012
PAHs	RGMA	Helena Líbalová, Kateřina	Global gene expression changes in human	Particle and Fibre Toxicology	2012
PAHs	RHBDF1	Helena Líbalová, Kateřina	Global gene expression changes in human	Particle and Fibre Toxicology	2012
PAHs	RHBDF2	Helena Líbalová, Kateřina	Global gene expression changes in human	Particle and Fibre Toxicology	2012
PAHs	RIPK2	Helena Líbalová, Kateřina	Global gene expression changes in human	Particle and Fibre Toxicology	2012
PAHs	RNF121	Helena Líbalová, Kateřina	Global gene expression changes in human	Particle and Fibre Toxicology	2012
PAHs	RNF214	Helena Líbalová, Kateřina	Global gene expression changes in human	Particle and Fibre Toxicology	2012
PAHs	RP11-529I10.4	Helena Líbalová, Kateřina	Global gene expression changes in human	Particle and Fibre Toxicology	2012
PAHs	RPIA	Helena Líbalová, Kateřina	Global gene expression changes in human	Particle and Fibre Toxicology	2012
PAHs	RPL28	Helena Líbalová, Kateřina	Global gene expression changes in human	Particle and Fibre Toxicology	2012
PAHs	RPP40	Helena Líbalová, Kateřina	Global gene expression changes in human	Particle and Fibre Toxicology	2012
PAHs	S100A10	Helena Líbalová, Kateřina	Global gene expression changes in human	Particle and Fibre Toxicology	2012
PAHs	S1PR3	Helena Líbalová, Kateřina	Global gene expression changes in human	Particle and Fibre Toxicology	2012
PAHs	SCARA3	Helena Líbalová, Kateřina	Global gene expression changes in human	Particle and Fibre Toxicology	2012
PAHs	SCRIB	Helena Líbalová, Kateřina	Global gene expression changes in human	Particle and Fibre Toxicology	2012
PAHs	SDCBP	Helena Líbalová, Kateřina	Global gene expression changes in human	Particle and Fibre Toxicology	2012
PAHs	SDHA	Helena Líbalová, Kateřina	Global gene expression changes in human	Particle and Fibre Toxicology	2012
PAHs	SEH1L	Helena Líbalová, Kateřina	Global gene expression changes in human	Particle and Fibre Toxicology	2012
PAHs	SERTAD4	Helena Líbalová, Kateřina	Global gene expression changes in human	Particle and Fibre Toxicology	2012
PAHs	SFRS7	Helena Líbalová, Kateřina	Global gene expression changes in human	Particle and Fibre Toxicology	2012
PAHs	SFXN5	Helena Líbalová, Kateřina	Global gene expression changes in human	Particle and Fibre Toxicology	2012
PAHs	SH3BGRL	Helena Líbalová, Kateřina	Global gene expression changes in human	Particle and Fibre Toxicology	2012

PAHs	SH3BP5L	Helena Líbalová, Kateřina	Global gene expression changes in human	Particle and Fibre Toxicology	2012
PAHs	SHC3	Helena Líbalová, Kateřina	Global gene expression changes in human	Particle and Fibre Toxicology	2012
PAHs	SKP1A	Helena Líbalová, Kateřina	Global gene expression changes in human	Particle and Fibre Toxicology	2012
PAHs	SLC25A4	Helena Líbalová, Kateřina	Global gene expression changes in human	Particle and Fibre Toxicology	2012
PAHs	SLC35A4	Helena Líbalová, Kateřina	Global gene expression changes in human	Particle and Fibre Toxicology	2012
PAHs	SLC38A10	Helena Líbalová, Kateřina	Global gene expression changes in human	Particle and Fibre Toxicology	2012
PAHs	SLC4A11	Helena Líbalová, Kateřina	Global gene expression changes in human	Particle and Fibre Toxicology	2012
PAHs	SLC4A7	Helena Líbalová, Kateřina	Global gene expression changes in human	Particle and Fibre Toxicology	2012
PAHs	SMA5	Helena Líbalová, Kateřina	Global gene expression changes in human	Particle and Fibre Toxicology	2012
PAHs	SMAD3	Helena Líbalová, Kateřina	Global gene expression changes in human	Particle and Fibre Toxicology	2012
PAHs	SMAD6	Helena Líbalová, Kateřina	Global gene expression changes in human	Particle and Fibre Toxicology	2012
PAHs	SMARCAD1	Helena Líbalová, Kateřina	Global gene expression changes in human	Particle and Fibre Toxicology	2012
PAHs	SMO	Helena Líbalová, Kateřina	Global gene expression changes in human	Particle and Fibre Toxicology	2012
PAHs	SNAPC3	Helena Líbalová, Kateřina	Global gene expression changes in human	Particle and Fibre Toxicology	2012
PAHs	SNX21	Helena Líbalová, Kateřina	Global gene expression changes in human	Particle and Fibre Toxicology	2012
PAHs	SPAG9	Helena Líbalová, Kateřina	Global gene expression changes in human	Particle and Fibre Toxicology	2012
PAHs	SPATA6	Helena Líbalová, Kateřina	Global gene expression changes in human	Particle and Fibre Toxicology	2012
PAHs	SPG3A	Helena Líbalová, Kateřina	Global gene expression changes in human	Particle and Fibre Toxicology	2012
PAHs	SPSB2	Helena Líbalová, Kateřina	Global gene expression changes in human	Particle and Fibre Toxicology	2012
PAHs	ST3GAL3	Helena Líbalová, Kateřina	Global gene expression changes in human	Particle and Fibre Toxicology	2012
PAHs	ST6GAL1	Helena Líbalová, Kateřina	Global gene expression changes in human	Particle and Fibre Toxicology	2012
PAHs	STBD1	Helena Líbalová, Kateřina	Global gene expression changes in human	Particle and Fibre Toxicology	2012
PAHs	STK19	Helena Líbalová, Kateřina	Global gene expression changes in human	Particle and Fibre Toxicology	2012
PAHs	STK24	Helena Líbalová, Kateřina	Global gene expression changes in human	Particle and Fibre Toxicology	2012
PAHs	STOML2	Helena Líbalová, Kateřina	Global gene expression changes in human	Particle and Fibre Toxicology	2012
PAHs	SULT1A1	Helena Líbalová, Kateřina	Global gene expression changes in human	Particle and Fibre Toxicology	2012
PAHs	SUOX	Helena Líbalová, Kateřina	Global gene expression changes in human	Particle and Fibre Toxicology	2012
PAHs	SWAP70	Helena Líbalová, Kateřina	Global gene expression changes in human	Particle and Fibre Toxicology	2012
PAHs	SYF2	Helena Líbalová, Kateřina	Global gene expression changes in human	Particle and Fibre Toxicology	2012
PAHs	SYNGR1	Helena Líbalová, Kateřina	Global gene expression changes in human	Particle and Fibre Toxicology	2012
PAHs	SYTL2	Helena Líbalová, Kateřina	Global gene expression changes in human	Particle and Fibre Toxicology	2012
PAHs	TAC3	Helena Líbalová, Kateřina	Global gene expression changes in human	Particle and Fibre Toxicology	2012
PAHs	TAF1A	Helena Líbalová, Kateřina	Global gene expression changes in human	Particle and Fibre Toxicology	2012
PAHs	TBC1D15	Helena Líbalová, Kateřina	Global gene expression changes in human	Particle and Fibre Toxicology	2012
PAHs	TBC1D3G	Helena Líbalová, Kateřina	Global gene expression changes in human	Particle and Fibre Toxicology	2012
PAHs	TBRG4	Helena Líbalová, Kateřina	Global gene expression changes in human	Particle and Fibre Toxicology	2012
PAHs	TBX2	Helena Líbalová, Kateřina	Global gene expression changes in human	Particle and Fibre Toxicology	2012
PAHs	TCIRG1	Helena Líbalová, Kateřina	Global gene expression changes in human	Particle and Fibre Toxicology	2012
PAHs	TGFB3	Helena Líbalová, Kateřina	Global gene expression changes in human	Particle and Fibre Toxicology	2012
PAHs	THEM4	Helena Líbalová, Kateřina	Global gene expression changes in human	Particle and Fibre Toxicology	2012
PAHs	THG1L	Helena Líbalová, Kateřina	Global gene expression changes in human	Particle and Fibre Toxicology	2012
PAHs	THOC2	Helena Líbalová, Kateřina	Global gene expression changes in human	Particle and Fibre Toxicology	2012
PAHs	THOP1	Helena Líbalová, Kateřina	Global gene expression changes in human	Particle and Fibre Toxicology	2012
PAHs	TIGA1	Helena Líbalová, Kateřina	Global gene expression changes in human	Particle and Fibre Toxicology	2012
PAHs	TIGD5	Helena Líbalová, Kateřina	Global gene expression changes in human	Particle and Fibre Toxicology	2012
PAHs	TIMELESS	Helena Líbalová, Kateřina	Global gene expression changes in human	Particle and Fibre Toxicology	2012
PAHs	TIMP4	Helena Líbalová, Kateřina	Global gene expression changes in human	Particle and Fibre Toxicology	2012
PAHs	TM2D2	Helena Líbalová, Kateřina	Global gene expression changes in human	Particle and Fibre Toxicology	2012
PAHs	TMEM103	Helena Líbalová, Kateřina	Global gene expression changes in human	Particle and Fibre Toxicology	2012
PAHs	TMEM116	Helena Líbalová, Kateřina	Global gene expression changes in human	Particle and Fibre Toxicology	2012
PAHs	TMEM119	Helena Líbalová, Kateřina	Global gene expression changes in human	Particle and Fibre Toxicology	2012
PAHs	TMEM134	Helena Líbalová, Kateřina	Global gene expression changes in human	Particle and Fibre Toxicology	2012
PAHs	TMEM14B	Helena Líbalová, Kateřina	Global gene expression changes in human	Particle and Fibre Toxicology	2012
PAHs	TMEM150	Helena Líbalová, Kateřina	Global gene expression changes in human	Particle and Fibre Toxicology	2012
PAHs	TMEM177	Helena Líbalová, Kateřina	Global gene expression changes in human	Particle and Fibre Toxicology	2012
PAHs	TMEM30A	Helena Líbalová, Kateřina	Global gene expression changes in human	Particle and Fibre Toxicology	2012
PAHs	TMEM48	Helena Líbalová, Kateřina	Global gene expression changes in human	Particle and Fibre Toxicology	2012
PAHs	TMEM50B	Helena Líbalová, Kateřina	Global gene expression changes in human	Particle and Fibre Toxicology	2012
PAHs	TMEM62	Helena Líbalová, Kateřina	Global gene expression changes in human	Particle and Fibre Toxicology	2012
PAHs	TMOD1	Helena Líbalová, Kateřina	Global gene expression changes in human	Particle and Fibre Toxicology	2012
PAHs	TOLLIP	Helena Líbalová, Kateřina	Global gene expression changes in human	Particle and Fibre Toxicology	2012
PAHs	TOP2A	Helena Líbalová, Kateřina	Global gene expression changes in human	Particle and Fibre Toxicology	2012
PAHs	TOP2B	Helena Líbalová, Kateřina	Global gene expression changes in human	Particle and Fibre Toxicology	2012
PAHs	TPP1	Helena Líbalová, Kateřina	Global gene expression changes in human	Particle and Fibre Toxicology	2012
PAHs	TPRG1L	Helena Líbalová, Kateřina	Global gene expression changes in human	Particle and Fibre Toxicology	2012
PAHs	TPST2	Helena Líbalová, Kateřina	Global gene expression changes in human	Particle and Fibre Toxicology	2012
PAHs	TRAK2	Helena Líbalová, Kateřina	Global gene expression changes in human	Particle and Fibre Toxicology	2012
PAHs	TRIB1	Helena Líbalová, Kateřina	Global gene expression changes in human	Particle and Fibre Toxicology	2012
PAHs	TRIB3	Helena Líbalová, Kateřina	Global gene expression changes in human	Particle and Fibre Toxicology	2012
PAHs	TRIM2	Helena Líbalová, Kateřina	Global gene expression changes in human	Particle and Fibre Toxicology	2012
PAHs	TRIM37	Helena Líbalová, Kateřina	Global gene expression changes in human	Particle and Fibre Toxicology	2012
PAHs	TRIM4	Helena Líbalová, Kateřina	Global gene expression changes in human	Particle and Fibre Toxicology	2012
PAHs	TRIP13	Helena Líbalová, Kateřina	Global gene expression changes in human	Particle and Fibre Toxicology	2012
PAHs	TRPT1	Helena Líbalová, Kateřina	Global gene expression changes in human	Particle and Fibre Toxicology	2012
PAHs	TSPAN31	Helena Líbalová, Kateřina	Global gene expression changes in human	Particle and Fibre Toxicology	2012
PAHs	TSSC1	Helena Líbalová, Kateřina	Global gene expression changes in human	Particle and Fibre Toxicology	2012
PAHs	TTC4	Helena Líbalová, Kateřina	Global gene expression changes in human	Particle and Fibre Toxicology	2012
PAHs	TTL	Helena Líbalová, Kateřina	Global gene expression changes in human	Particle and Fibre Toxicology	2012
PAHs	TTLL12	Helena Líbalová, Kateřina	Global gene expression changes in human	Particle and Fibre Toxicology	2012
PAHs	TUBB2C	Helena Líbalová, Kateřina	Global gene expression changes in human	Particle and Fibre Toxicology	2012
PAHs	UBE2T	Helena Líbalová, Kateřina	Global gene expression changes in human	Particle and Fibre Toxicology	2012
PAHs	UBIAD1	Helena Líbalová, Kateřina	Global gene expression changes in human	Particle and Fibre Toxicology	2012
PAHs	UBL5	Helena Líbalová, Kateřina	Global gene expression changes in human	Particle and Fibre Toxicology	2012
PAHs	UBQLN1	Helena Líbalová, Kateřina	Global gene expression changes in human	Particle and Fibre Toxicology	2012
PAHs	UBR2	Helena Líbalová, Kateřina	Global gene expression changes in human	Particle and Fibre Toxicology	2012
PAHs	UBR5	Helena Líbalová, Kateřina	Global gene expression changes in human	Particle and Fibre Toxicology	2012
PAHs	UPP1	Helena Líbalová, Kateřina	Global gene expression changes in human	Particle and Fibre Toxicology	2012
PAHs	USP3	Helena Líbalová, Kateřina	Global gene expression changes in human	Particle and Fibre Toxicology	2012
PAHs	USP30	Helena Líbalová, Kateřina	Global gene expression changes in human	Particle and Fibre Toxicology	2012

PAHs	VPS11	Helena Líbalová, Kateřina	Global gene expression changes in human	Particle and Fibre Toxicology	2012
PAHs	VPS45	Helena Líbalová, Kateřina	Global gene expression changes in human	Particle and Fibre Toxicology	2012
PAHs	VRK2	Helena Líbalová, Kateřina	Global gene expression changes in human	Particle and Fibre Toxicology	2012
PAHs	WAC	Helena Líbalová, Kateřina	Global gene expression changes in human	Particle and Fibre Toxicology	2012
PAHs	WDR61	Helena Líbalová, Kateřina	Global gene expression changes in human	Particle and Fibre Toxicology	2012
PAHs	WDR75	Helena Líbalová, Kateřina	Global gene expression changes in human	Particle and Fibre Toxicology	2012
PAHs	WDR92	Helena Líbalová, Kateřina	Global gene expression changes in human	Particle and Fibre Toxicology	2012
PAHs	WEE1	Helena Líbalová, Kateřina	Global gene expression changes in human	Particle and Fibre Toxicology	2012
PAHs	WIPI1	Helena Líbalová, Kateřina	Global gene expression changes in human	Particle and Fibre Toxicology	2012
PAHs	WWC1	Helena Líbalová, Kateřina	Global gene expression changes in human	Particle and Fibre Toxicology	2012
PAHs	XTP3TPA	Helena Líbalová, Kateřina	Global gene expression changes in human	Particle and Fibre Toxicology	2012
PAHs	ZBED1	Helena Líbalová, Kateřina	Global gene expression changes in human	Particle and Fibre Toxicology	2012
PAHs	ZBTB40	Helena Líbalová, Kateřina	Global gene expression changes in human	Particle and Fibre Toxicology	2012
PAHs	ZC3H14	Helena Líbalová, Kateřina	Global gene expression changes in human	Particle and Fibre Toxicology	2012
PAHs	ZDHHC7	Helena Líbalová, Kateřina	Global gene expression changes in human	Particle and Fibre Toxicology	2012
PAHs	ZFAND2B	Helena Líbalová, Kateřina	Global gene expression changes in human	Particle and Fibre Toxicology	2012
PAHs	ZNF142	Helena Líbalová, Kateřina	Global gene expression changes in human	Particle and Fibre Toxicology	2012
PAHs	ZNF143	Helena Líbalová, Kateřina	Global gene expression changes in human	Particle and Fibre Toxicology	2012
PAHs	ZNF232	Helena Líbalová, Kateřina	Global gene expression changes in human	Particle and Fibre Toxicology	2012
PAHs	ZNF250	Helena Líbalová, Kateřina	Global gene expression changes in human	Particle and Fibre Toxicology	2012
PAHs	ZNF274	Helena Líbalová, Kateřina	Global gene expression changes in human	Particle and Fibre Toxicology	2012
PAHs	ZNF319	Helena Líbalová, Kateřina	Global gene expression changes in human	Particle and Fibre Toxicology	2012
PAHs	ZNF334	Helena Líbalová, Kateřina	Global gene expression changes in human	Particle and Fibre Toxicology	2012
PAHs	ZNF407	Helena Líbalová, Kateřina	Global gene expression changes in human	Particle and Fibre Toxicology	2012
PAHs	ZNF438	Helena Líbalová, Kateřina	Global gene expression changes in human	Particle and Fibre Toxicology	2012
PAHs	ZNF462	Helena Líbalová, Kateřina	Global gene expression changes in human	Particle and Fibre Toxicology	2012
PAHs	ZNF593	Helena Líbalová, Kateřina	Global gene expression changes in human	Particle and Fibre Toxicology	2012
PAHs	ZNF616	Helena Líbalová, Kateřina	Global gene expression changes in human	Particle and Fibre Toxicology	2012
PAHs	ZNF706	Helena Líbalová, Kateřina	Global gene expression changes in human	Particle and Fibre Toxicology	2012
PAHs	ZSCAN18	Helena Líbalová, Kateřina	Global gene expression changes in human	Particle and Fibre Toxicology	2012
PAHs	ZSWIM3	Helena Líbalová, Kateřina	Global gene expression changes in human	Particle and Fibre Toxicology	2012
PAHs	ZWILCH	Helena Líbalová, Kateřina	Global gene expression changes in human	Particle and Fibre Toxicology	2012
PAHs	ZXDC	Helena Líbalová, Kateřina	Global gene expression changes in human	Particle and Fibre Toxicology	2012
PAHs	ABCA8	Helena Líbalová, Kateřina	Global gene expression changes in human	Particle and Fibre Toxicology	2012
PAHs	ABCF2	Helena Líbalová, Kateřina	Global gene expression changes in human	Particle and Fibre Toxicology	2012
PAHs	ACACA	Helena Líbalová, Kateřina	Global gene expression changes in human	Particle and Fibre Toxicology	2012
PAHs	ACAT1	Helena Líbalová, Kateřina	Global gene expression changes in human	Particle and Fibre Toxicology	2012
PAHs	ACN9	Helena Líbalová, Kateřina	Global gene expression changes in human	Particle and Fibre Toxicology	2012
PAHs	ACOT7	Helena Líbalová, Kateřina	Global gene expression changes in human	Particle and Fibre Toxicology	2012
PAHs	ACP1	Helena Líbalová, Kateřina	Global gene expression changes in human	Particle and Fibre Toxicology	2012
PAHs	ACTG2	Helena Líbalová, Kateřina	Global gene expression changes in human	Particle and Fibre Toxicology	2012
PAHs	ACTN4	Helena Líbalová, Kateřina	Global gene expression changes in human	Particle and Fibre Toxicology	2012
PAHs	ACVR1B	Helena Líbalová, Kateřina	Global gene expression changes in human	Particle and Fibre Toxicology	2012
PAHs	ACVRL1	Helena Líbalová, Kateřina	Global gene expression changes in human	Particle and Fibre Toxicology	2012
PAHs	ADAMTS1	Helena Líbalová, Kateřina	Global gene expression changes in human	Particle and Fibre Toxicology	2012
PAHs	ADAMTS19	Helena Líbalová, Kateřina	Global gene expression changes in human	Particle and Fibre Toxicology	2012
PAHs	ADAMTS9	Helena Líbalová, Kateřina	Global gene expression changes in human	Particle and Fibre Toxicology	2012
PAHs	ADAT3	Helena Líbalová, Kateřina	Global gene expression changes in human	Particle and Fibre Toxicology	2012
PAHs	ADD3	Helena Líbalová, Kateřina	Global gene expression changes in human	Particle and Fibre Toxicology	2012
PAHs	ADRB2	Helena Líbalová, Kateřina	Global gene expression changes in human	Particle and Fibre Toxicology	2012
PAHs	AGTRAP	Helena Líbalová, Kateřina	Global gene expression changes in human	Particle and Fibre Toxicology	2012
PAHs	AHCTF1	Helena Líbalová, Kateřina	Global gene expression changes in human	Particle and Fibre Toxicology	2012
PAHs	AHCYL2	Helena Líbalová, Kateřina	Global gene expression changes in human	Particle and Fibre Toxicology	2012
PAHs	AHDC1	Helena Líbalová, Kateřina	Global gene expression changes in human	Particle and Fibre Toxicology	2012
PAHs	AKAP13	Helena Líbalová, Kateřina	Global gene expression changes in human	Particle and Fibre Toxicology	2012
PAHs	ALPK2	Helena Líbalová, Kateřina	Global gene expression changes in human	Particle and Fibre Toxicology	2012
PAHs	AMFR	Helena Líbalová, Kateřina	Global gene expression changes in human	Particle and Fibre Toxicology	2012
PAHs	AMY1A	Helena Líbalová, Kateřina	Global gene expression changes in human	Particle and Fibre Toxicology	2012
PAHs	ANAPC11	Helena Líbalová, Kateřina	Global gene expression changes in human	Particle and Fibre Toxicology	2012
PAHs	ANKRD12	Helena Líbalová, Kateřina	Global gene expression changes in human	Particle and Fibre Toxicology	2012
PAHs	ANKRD13A	Helena Líbalová, Kateřina	Global gene expression changes in human	Particle and Fibre Toxicology	2012
PAHs	ANKRD28	Helena Líbalová, Kateřina	Global gene expression changes in human	Particle and Fibre Toxicology	2012
PAHs	ANKRD35	Helena Líbalová, Kateřina	Global gene expression changes in human	Particle and Fibre Toxicology	2012
PAHs	ANKRD37	Helena Líbalová, Kateřina	Global gene expression changes in human	Particle and Fibre Toxicology	2012
PAHs	ANKS1A	Helena Líbalová, Kateřina	Global gene expression changes in human	Particle and Fibre Toxicology	2012
PAHs	ANLN	Helena Líbalová, Kateřina	Global gene expression changes in human	Particle and Fibre Toxicology	2012
PAHs	ANPEP	Helena Líbalová, Kateřina	Global gene expression changes in human	Particle and Fibre Toxicology	2012
PAHs	ANXA1	Helena Líbalová, Kateřina	Global gene expression changes in human	Particle and Fibre Toxicology	2012
PAHs	AOX1	Helena Líbalová, Kateřina	Global gene expression changes in human	Particle and Fibre Toxicology	2012
PAHs	AP2A2	Helena Líbalová, Kateřina	Global gene expression changes in human	Particle and Fibre Toxicology	2012
PAHs	AP2M1	Helena Líbalová, Kateřina	Global gene expression changes in human	Particle and Fibre Toxicology	2012
PAHs	AP3M2	Helena Líbalová, Kateřina	Global gene expression changes in human	Particle and Fibre Toxicology	2012
PAHs	APCDD1L	Helena Líbalová, Kateřina	Global gene expression changes in human	Particle and Fibre Toxicology	2012
PAHs	APOBEC3F	Helena Líbalová, Kateřina	Global gene expression changes in human	Particle and Fibre Toxicology	2012
PAHs	APOM	Helena Líbalová, Kateřina	Global gene expression changes in human	Particle and Fibre Toxicology	2012
PAHs	ARF3	Helena Líbalová, Kateřina	Global gene expression changes in human	Particle and Fibre Toxicology	2012
PAHs	ARF4	Helena Líbalová, Kateřina	Global gene expression changes in human	Particle and Fibre Toxicology	2012
PAHs	ARHGAP1	Helena Líbalová, Kateřina	Global gene expression changes in human	Particle and Fibre Toxicology	2012
PAHs	ARHGAP28	Helena Líbalová, Kateřina	Global gene expression changes in human	Particle and Fibre Toxicology	2012
PAHs	ARMCX5	Helena Líbalová, Kateřina	Global gene expression changes in human	Particle and Fibre Toxicology	2012
PAHs	ARPP-19	Helena Líbalová, Kateřina	Global gene expression changes in human	Particle and Fibre Toxicology	2012
PAHs	ASPM	Helena Líbalová, Kateřina	Global gene expression changes in human	Particle and Fibre Toxicology	2012
PAHs	ATG2B	Helena Líbalová, Kateřina	Global gene expression changes in human	Particle and Fibre Toxicology	2012
PAHs	ATG9A	Helena Líbalová, Kateřina	Global gene expression changes in human	Particle and Fibre Toxicology	2012
PAHs	ATP11C	Helena Líbalová, Kateřina	Global gene expression changes in human	Particle and Fibre Toxicology	2012
PAHs	ATP2A2	Helena Líbalová, Kateřina	Global gene expression changes in human	Particle and Fibre Toxicology	2012
PAHs	ATP5G3	Helena Líbalová, Kateřina	Global gene expression changes in human	Particle and Fibre Toxicology	2012
PAHs	ATP5J	Helena Líbalová, Kateřina	Global gene expression changes in human	Particle and Fibre Toxicology	2012
PAHs	ATP6V0A1	Helena Líbalová, Kateřina	Global gene expression changes in human	Particle and Fibre Toxicology	2012

PAHs	ATP6V0E2	Helena Líbalová, Kateřina	Global gene expression changes in human	Particle and Fibre Toxicology	2012
PAHs	ATP6V1G1	Helena Líbalová, Kateřina	Global gene expression changes in human	Particle and Fibre Toxicology	2012
PAHs	ATXN2	Helena Líbalová, Kateřina	Global gene expression changes in human	Particle and Fibre Toxicology	2012
PAHs	ATXN2L	Helena Líbalová, Kateřina	Global gene expression changes in human	Particle and Fibre Toxicology	2012
PAHs	ATXN3	Helena Líbalová, Kateřina	Global gene expression changes in human	Particle and Fibre Toxicology	2012
PAHs	B3GALT4	Helena Líbalová, Kateřina	Global gene expression changes in human	Particle and Fibre Toxicology	2012
PAHs	BACH2	Helena Líbalová, Kateřina	Global gene expression changes in human	Particle and Fibre Toxicology	2012
PAHs	BAG3	Helena Líbalová, Kateřina	Global gene expression changes in human	Particle and Fibre Toxicology	2012
PAHs	BAMBI	Helena Líbalová, Kateřina	Global gene expression changes in human	Particle and Fibre Toxicology	2012
PAHs	BAX	Helena Líbalová, Kateřina	Global gene expression changes in human	Particle and Fibre Toxicology	2012
PAHs	BBS4	Helena Líbalová, Kateřina	Global gene expression changes in human	Particle and Fibre Toxicology	2012
PAHs	BCKDHA	Helena Líbalová, Kateřina	Global gene expression changes in human	Particle and Fibre Toxicology	2012
PAHs	BCL7B	Helena Líbalová, Kateřina	Global gene expression changes in human	Particle and Fibre Toxicology	2012
PAHs	BCR	Helena Líbalová, Kateřina	Global gene expression changes in human	Particle and Fibre Toxicology	2012
PAHs	BEX2	Helena Líbalová, Kateřina	Global gene expression changes in human	Particle and Fibre Toxicology	2012
PAHs	BEXL1	Helena Líbalová, Kateřina	Global gene expression changes in human	Particle and Fibre Toxicology	2012
PAHs	BFAR	Helena Líbalová, Kateřina	Global gene expression changes in human	Particle and Fibre Toxicology	2012
PAHs	BHLHB2	Helena Líbalová, Kateřina	Global gene expression changes in human	Particle and Fibre Toxicology	2012
PAHs	BMP2K	Helena Líbalová, Kateřina	Global gene expression changes in human	Particle and Fibre Toxicology	2012
PAHs	BNIP2	Helena Líbalová, Kateřina	Global gene expression changes in human	Particle and Fibre Toxicology	2012
PAHs	BOK	Helena Líbalová, Kateřina	Global gene expression changes in human	Particle and Fibre Toxicology	2012
PAHs	BRP44	Helena Líbalová, Kateřina	Global gene expression changes in human	Particle and Fibre Toxicology	2012
PAHs	BTG1	Helena Líbalová, Kateřina	Global gene expression changes in human	Particle and Fibre Toxicology	2012
PAHs	BTN3A1	Helena Líbalová, Kateřina	Global gene expression changes in human	Particle and Fibre Toxicology	2012
PAHs	BUB1	Helena Líbalová, Kateřina	Global gene expression changes in human	Particle and Fibre Toxicology	2012
PAHs	BUD13	Helena Líbalová, Kateřina	Global gene expression changes in human	Particle and Fibre Toxicology	2012
PAHs	BVES	Helena Líbalová, Kateřina	Global gene expression changes in human	Particle and Fibre Toxicology	2012
PAHs	C10orf2	Helena Líbalová, Kateřina	Global gene expression changes in human	Particle and Fibre Toxicology	2012
PAHs	C10orf33	Helena Líbalová, Kateřina	Global gene expression changes in human	Particle and Fibre Toxicology	2012
PAHs	C10orf76	Helena Líbalová, Kateřina	Global gene expression changes in human	Particle and Fibre Toxicology	2012
PAHs	C13orf23	Helena Líbalová, Kateřina	Global gene expression changes in human	Particle and Fibre Toxicology	2012
PAHs	C14orf132	Helena Líbalová, Kateřina	Global gene expression changes in human	Particle and Fibre Toxicology	2012
PAHs	C14orf149	Helena Líbalová, Kateřina	Global gene expression changes in human	Particle and Fibre Toxicology	2012
PAHs	C15orf15	Helena Líbalová, Kateřina	Global gene expression changes in human	Particle and Fibre Toxicology	2012
PAHs	C16orf14	Helena Líbalová, Kateřina	Global gene expression changes in human	Particle and Fibre Toxicology	2012
PAHs	C16orf45	Helena Líbalová, Kateřina	Global gene expression changes in human	Particle and Fibre Toxicology	2012
PAHs	C16orf56	Helena Líbalová, Kateřina	Global gene expression changes in human	Particle and Fibre Toxicology	2012
PAHs	C16orf63	Helena Líbalová, Kateřina	Global gene expression changes in human	Particle and Fibre Toxicology	2012
PAHs	C16orf75	Helena Líbalová, Kateřina	Global gene expression changes in human	Particle and Fibre Toxicology	2012
PAHs	C16orf80	Helena Líbalová, Kateřina	Global gene expression changes in human	Particle and Fibre Toxicology	2012
PAHs	C17orf79	Helena Líbalová, Kateřina	Global gene expression changes in human	Particle and Fibre Toxicology	2012
PAHs	C17orf82	Helena Líbalová, Kateřina	Global gene expression changes in human	Particle and Fibre Toxicology	2012
PAHs	C17orf95	Helena Líbalová, Kateřina	Global gene expression changes in human	Particle and Fibre Toxicology	2012
PAHs	C18orf10	Helena Líbalová, Kateřina	Global gene expression changes in human	Particle and Fibre Toxicology	2012
PAHs	C18orf19	Helena Líbalová, Kateřina	Global gene expression changes in human	Particle and Fibre Toxicology	2012
PAHs	C18orf21	Helena Líbalová, Kateřina	Global gene expression changes in human	Particle and Fibre Toxicology	2012
PAHs	C19orf24	Helena Líbalová, Kateřina	Global gene expression changes in human	Particle and Fibre Toxicology	2012
PAHs	C19orf48	Helena Líbalová, Kateřina	Global gene expression changes in human	Particle and Fibre Toxicology	2012
PAHs	C1orf131	Helena Líbalová, Kateřina	Global gene expression changes in human	Particle and Fibre Toxicology	2012
PAHs	C1orf144	Helena Líbalová, Kateřina	Global gene expression changes in human	Particle and Fibre Toxicology	2012
PAHs	C1orf225	Helena Líbalová, Kateřina	Global gene expression changes in human	Particle and Fibre Toxicology	2012
PAHs	C1orf83	Helena Líbalová, Kateřina	Global gene expression changes in human	Particle and Fibre Toxicology	2012
PAHs	C1QL1	Helena Líbalová, Kateřina	Global gene expression changes in human	Particle and Fibre Toxicology	2012
PAHs	C20orf11	Helena Líbalová, Kateřina	Global gene expression changes in human	Particle and Fibre Toxicology	2012
PAHs	C20orf127	Helena Líbalová, Kateřina	Global gene expression changes in human	Particle and Fibre Toxicology	2012
PAHs	C21orf58	Helena Líbalová, Kateřina	Global gene expression changes in human	Particle and Fibre Toxicology	2012
PAHs	C21orf7	Helena Líbalová, Kateřina	Global gene expression changes in human	Particle and Fibre Toxicology	2012
PAHs	C21orf91	Helena Líbalová, Kateřina	Global gene expression changes in human	Particle and Fibre Toxicology	2012
PAHs	C22orf36	Helena Líbalová, Kateřina	Global gene expression changes in human	Particle and Fibre Toxicology	2012
PAHs	C22orf39	Helena Líbalová, Kateřina	Global gene expression changes in human	Particle and Fibre Toxicology	2012
PAHs	C3orf1	Helena Líbalová, Kateřina	Global gene expression changes in human	Particle and Fibre Toxicology	2012
PAHs	C3orf26	Helena Líbalová, Kateřina	Global gene expression changes in human	Particle and Fibre Toxicology	2012
PAHs	C4orf27	Helena Líbalová, Kateřina	Global gene expression changes in human	Particle and Fibre Toxicology	2012
PAHs	C4orf30	Helena Líbalová, Kateřina	Global gene expression changes in human	Particle and Fibre Toxicology	2012
PAHs	C5orf21	Helena Líbalová, Kateřina	Global gene expression changes in human	Particle and Fibre Toxicology	2012
PAHs	C5orf35	Helena Líbalová, Kateřina	Global gene expression changes in human	Particle and Fibre Toxicology	2012
PAHs	C5orf37	Helena Líbalová, Kateřina	Global gene expression changes in human	Particle and Fibre Toxicology	2012
PAHs	C5orf41	Helena Líbalová, Kateřina	Global gene expression changes in human	Particle and Fibre Toxicology	2012
PAHs	C6orf106	Helena Líbalová, Kateřina	Global gene expression changes in human	Particle and Fibre Toxicology	2012
PAHs	C6orf125	Helena Líbalová, Kateřina	Global gene expression changes in human	Particle and Fibre Toxicology	2012
PAHs	C6orf173	Helena Líbalová, Kateřina	Global gene expression changes in human	Particle and Fibre Toxicology	2012
PAHs	C6orf66	Helena Líbalová, Kateřina	Global gene expression changes in human	Particle and Fibre Toxicology	2012
PAHs	C6orf89	Helena Líbalová, Kateřina	Global gene expression changes in human	Particle and Fibre Toxicology	2012
PAHs	C9orf125	Helena Líbalová, Kateřina	Global gene expression changes in human	Particle and Fibre Toxicology	2012
PAHs	C9orf142	Helena Líbalová, Kateřina	Global gene expression changes in human	Particle and Fibre Toxicology	2012
PAHs	C9orf80	Helena Líbalová, Kateřina	Global gene expression changes in human	Particle and Fibre Toxicology	2012
PAHs	C9orf82	Helena Líbalová, Kateřina	Global gene expression changes in human	Particle and Fibre Toxicology	2012
PAHs	CAD	Helena Líbalová, Kateřina	Global gene expression changes in human	Particle and Fibre Toxicology	2012
PAHs	CADM1	Helena Líbalová, Kateřina	Global gene expression changes in human	Particle and Fibre Toxicology	2012
PAHs	CALCOCO1	Helena Líbalová, Kateřina	Global gene expression changes in human	Particle and Fibre Toxicology	2012
PAHs	CALCOCO2	Helena Líbalová, Kateřina	Global gene expression changes in human	Particle and Fibre Toxicology	2012
PAHs	CAPRIN2	Helena Líbalová, Kateřina	Global gene expression changes in human	Particle and Fibre Toxicology	2012
PAHs	CATSPER2	Helena Líbalová, Kateřina	Global gene expression changes in human	Particle and Fibre Toxicology	2012
PAHs	CBFA2T2	Helena Líbalová, Kateřina	Global gene expression changes in human	Particle and Fibre Toxicology	2012
PAHs	CBLN3	Helena Líbalová, Kateřina	Global gene expression changes in human	Particle and Fibre Toxicology	2012
PAHs	CBR4	Helena Líbalová, Kateřina	Global gene expression changes in human	Particle and Fibre Toxicology	2012
PAHs	CBX1	Helena Líbalová, Kateřina	Global gene expression changes in human	Particle and Fibre Toxicology	2012
PAHs	CCBE1	Helena Líbalová, Kateřina	Global gene expression changes in human	Particle and Fibre Toxicology	2012
PAHs	CCDC109A	Helena Líbalová, Kateřina	Global gene expression changes in human	Particle and Fibre Toxicology	2012

PAHs	CCDC111	Helena Líbalová, Kateřina	Global gene expression changes in human	Particle and Fibre Toxicology	2012
PAHs	CCDC112	Helena Líbalová, Kateřina	Global gene expression changes in human	Particle and Fibre Toxicology	2012
PAHs	CCDC117	Helena Líbalová, Kateřina	Global gene expression changes in human	Particle and Fibre Toxicology	2012
PAHs	CCDC18	Helena Líbalová, Kateřina	Global gene expression changes in human	Particle and Fibre Toxicology	2012
PAHs	CCDC41	Helena Líbalová, Kateřina	Global gene expression changes in human	Particle and Fibre Toxicology	2012
PAHs	CCDC45	Helena Líbalová, Kateřina	Global gene expression changes in human	Particle and Fibre Toxicology	2012
PAHs	CCDC50	Helena Líbalová, Kateřina	Global gene expression changes in human	Particle and Fibre Toxicology	2012
PAHs	CCNF	Helena Líbalová, Kateřina	Global gene expression changes in human	Particle and Fibre Toxicology	2012
PAHs	CCNG1	Helena Líbalová, Kateřina	Global gene expression changes in human	Particle and Fibre Toxicology	2012
PAHs	CD68	Helena Líbalová, Kateřina	Global gene expression changes in human	Particle and Fibre Toxicology	2012
PAHs	CD97	Helena Líbalová, Kateřina	Global gene expression changes in human	Particle and Fibre Toxicology	2012
PAHs	CDC123	Helena Líbalová, Kateřina	Global gene expression changes in human	Particle and Fibre Toxicology	2012
PAHs	CDC42EP2	Helena Líbalová, Kateřina	Global gene expression changes in human	Particle and Fibre Toxicology	2012
PAHs	CDC42EP3	Helena Líbalová, Kateřina	Global gene expression changes in human	Particle and Fibre Toxicology	2012
PAHs	CDH4	Helena Líbalová, Kateřina	Global gene expression changes in human	Particle and Fibre Toxicology	2012
PAHs	CDKN1B	Helena Líbalová, Kateřina	Global gene expression changes in human	Particle and Fibre Toxicology	2012
PAHs	CECR5	Helena Líbalová, Kateřina	Global gene expression changes in human	Particle and Fibre Toxicology	2012
PAHs	CECR7	Helena Líbalová, Kateřina	Global gene expression changes in human	Particle and Fibre Toxicology	2012
PAHs	CELSR3	Helena Líbalová, Kateřina	Global gene expression changes in human	Particle and Fibre Toxicology	2012
PAHs	CENPE	Helena Líbalová, Kateřina	Global gene expression changes in human	Particle and Fibre Toxicology	2012
PAHs	CENPF	Helena Líbalová, Kateřina	Global gene expression changes in human	Particle and Fibre Toxicology	2012
PAHs	CFB	Helena Líbalová, Kateřina	Global gene expression changes in human	Particle and Fibre Toxicology	2012
PAHs	CGRRF1	Helena Líbalová, Kateřina	Global gene expression changes in human	Particle and Fibre Toxicology	2012
PAHs	CHCHD3	Helena Líbalová, Kateřina	Global gene expression changes in human	Particle and Fibre Toxicology	2012
PAHs	CHCHD4	Helena Líbalová, Kateřina	Global gene expression changes in human	Particle and Fibre Toxicology	2012
PAHs	CHCHD6	Helena Líbalová, Kateřina	Global gene expression changes in human	Particle and Fibre Toxicology	2012
PAHs	CHMP1B	Helena Líbalová, Kateřina	Global gene expression changes in human	Particle and Fibre Toxicology	2012
PAHs	CHMP4B	Helena Líbalová, Kateřina	Global gene expression changes in human	Particle and Fibre Toxicology	2012
PAHs	CHRNB1	Helena Líbalová, Kateřina	Global gene expression changes in human	Particle and Fibre Toxicology	2012
PAHs	CLDN1	Helena Líbalová, Kateřina	Global gene expression changes in human	Particle and Fibre Toxicology	2012
PAHs	CLDN12	Helena Líbalová, Kateřina	Global gene expression changes in human	Particle and Fibre Toxicology	2012
PAHs	CLIP2	Helena Líbalová, Kateřina	Global gene expression changes in human	Particle and Fibre Toxicology	2012
PAHs	CLIP4	Helena Líbalová, Kateřina	Global gene expression changes in human	Particle and Fibre Toxicology	2012
PAHs	CLK1	Helena Líbalová, Kateřina	Global gene expression changes in human	Particle and Fibre Toxicology	2012
PAHs	CLTB	Helena Líbalová, Kateřina	Global gene expression changes in human	Particle and Fibre Toxicology	2012
PAHs	CMAS	Helena Líbalová, Kateřina	Global gene expression changes in human	Particle and Fibre Toxicology	2012
PAHs	CNBP	Helena Líbalová, Kateřina	Global gene expression changes in human	Particle and Fibre Toxicology	2012
PAHs	CNN1	Helena Líbalová, Kateřina	Global gene expression changes in human	Particle and Fibre Toxicology	2012
PAHs	CNNM3	Helena Líbalová, Kateřina	Global gene expression changes in human	Particle and Fibre Toxicology	2012
PAHs	COBRA1	Helena Líbalová, Kateřina	Global gene expression changes in human	Particle and Fibre Toxicology	2012
PAHs	COG5	Helena Líbalová, Kateřina	Global gene expression changes in human	Particle and Fibre Toxicology	2012
PAHs	COL5A2	Helena Líbalová, Kateřina	Global gene expression changes in human	Particle and Fibre Toxicology	2012
PAHs	COMMD5	Helena Líbalová, Kateřina	Global gene expression changes in human	Particle and Fibre Toxicology	2012
PAHs	COMMD7	Helena Líbalová, Kateřina	Global gene expression changes in human	Particle and Fibre Toxicology	2012
PAHs	COPS7B	Helena Líbalová, Kateřina	Global gene expression changes in human	Particle and Fibre Toxicology	2012
PAHs	CORO1B	Helena Líbalová, Kateřina	Global gene expression changes in human	Particle and Fibre Toxicology	2012
PAHs	CORO6	Helena Líbalová, Kateřina	Global gene expression changes in human	Particle and Fibre Toxicology	2012
PAHs	COX10	Helena Líbalová, Kateřina	Global gene expression changes in human	Particle and Fibre Toxicology	2012
PAHs	CPNE1	Helena Líbalová, Kateřina	Global gene expression changes in human	Particle and Fibre Toxicology	2012
PAHs	CPSF3L	Helena Líbalová, Kateřina	Global gene expression changes in human	Particle and Fibre Toxicology	2012
PAHs	CRBN	Helena Líbalová, Kateřina	Global gene expression changes in human	Particle and Fibre Toxicology	2012
PAHs	CRELD2	Helena Líbalová, Kateřina	Global gene expression changes in human	Particle and Fibre Toxicology	2012
PAHs	CRYAB	Helena Líbalová, Kateřina	Global gene expression changes in human	Particle and Fibre Toxicology	2012
PAHs	CRYBB2	Helena Líbalová, Kateřina	Global gene expression changes in human	Particle and Fibre Toxicology	2012
PAHs	CSAD	Helena Líbalová, Kateřina	Global gene expression changes in human	Particle and Fibre Toxicology	2012
PAHs	CSRP2BP	Helena Líbalová, Kateřina	Global gene expression changes in human	Particle and Fibre Toxicology	2012
PAHs	CTNNAL1	Helena Líbalová, Kateřina	Global gene expression changes in human	Particle and Fibre Toxicology	2012
PAHs	CTXN1	Helena Líbalová, Kateřina	Global gene expression changes in human	Particle and Fibre Toxicology	2012
PAHs	CWF19L2	Helena Líbalová, Kateřina	Global gene expression changes in human	Particle and Fibre Toxicology	2012
PAHs	CYB5R1	Helena Líbalová, Kateřina	Global gene expression changes in human	Particle and Fibre Toxicology	2012
PAHs	CYBASC3	Helena Líbalová, Kateřina	Global gene expression changes in human	Particle and Fibre Toxicology	2012
PAHs	CYGB	Helena Líbalová, Kateřina	Global gene expression changes in human	Particle and Fibre Toxicology	2012
PAHs	CYLN2	Helena Líbalová, Kateřina	Global gene expression changes in human	Particle and Fibre Toxicology	2012
PAHs	DACT3	Helena Líbalová, Kateřina	Global gene expression changes in human	Particle and Fibre Toxicology	2012
PAHs	DAGLB	Helena Líbalová, Kateřina	Global gene expression changes in human	Particle and Fibre Toxicology	2012
PAHs	DBI	Helena Líbalová, Kateřina	Global gene expression changes in human	Particle and Fibre Toxicology	2012
PAHs	DCBLD2	Helena Líbalová, Kateřina	Global gene expression changes in human	Particle and Fibre Toxicology	2012
PAHs	DCP1B	Helena Líbalová, Kateřina	Global gene expression changes in human	Particle and Fibre Toxicology	2012
PAHs	DDR1	Helena Líbalová, Kateřina	Global gene expression changes in human	Particle and Fibre Toxicology	2012
PAHs	DDX23	Helena Líbalová, Kateřina	Global gene expression changes in human	Particle and Fibre Toxicology	2012
PAHs	DDX27	Helena Líbalová, Kateřina	Global gene expression changes in human	Particle and Fibre Toxicology	2012
PAHs	DDX46	Helena Líbalová, Kateřina	Global gene expression changes in human	Particle and Fibre Toxicology	2012
PAHs	DDX58	Helena Líbalová, Kateřina	Global gene expression changes in human	Particle and Fibre Toxicology	2012
PAHs	DEPDC1	Helena Líbalová, Kateřina	Global gene expression changes in human	Particle and Fibre Toxicology	2012
PAHs	DGAT1	Helena Líbalová, Kateřina	Global gene expression changes in human	Particle and Fibre Toxicology	2012
PAHs	DHX36	Helena Líbalová, Kateřina	Global gene expression changes in human	Particle and Fibre Toxicology	2012
PAHs	DHX9	Helena Líbalová, Kateřina	Global gene expression changes in human	Particle and Fibre Toxicology	2012
PAHs	DNAJA1	Helena Líbalová, Kateřina	Global gene expression changes in human	Particle and Fibre Toxicology	2012
PAHs	DNAJB9	Helena Líbalová, Kateřina	Global gene expression changes in human	Particle and Fibre Toxicology	2012
PAHs	DNAJC22	Helena Líbalová, Kateřina	Global gene expression changes in human	Particle and Fibre Toxicology	2012
PAHs	DNCL1	Helena Líbalová, Kateřina	Global gene expression changes in human	Particle and Fibre Toxicology	2012
PAHs	DOCK1	Helena Líbalová, Kateřina	Global gene expression changes in human	Particle and Fibre Toxicology	2012
PAHs	DOCK5	Helena Líbalová, Kateřina	Global gene expression changes in human	Particle and Fibre Toxicology	2012
PAHs	DPH2	Helena Líbalová, Kateřina	Global gene expression changes in human	Particle and Fibre Toxicology	2012
PAHs	DPH5	Helena Líbalová, Kateřina	Global gene expression changes in human	Particle and Fibre Toxicology	2012
PAHs	DTNA	Helena Líbalová, Kateřina	Global gene expression changes in human	Particle and Fibre Toxicology	2012
PAHs	DTNBP1	Helena Líbalová, Kateřina	Global gene expression changes in human	Particle and Fibre Toxicology	2012
PAHs	DTWD1	Helena Líbalová, Kateřina	Global gene expression changes in human	Particle and Fibre Toxicology	2012
PAHs	DUSP1	Helena Líbalová, Kateřina	Global gene expression changes in human	Particle and Fibre Toxicology	2012

PAHs	DUSP14	Helena Líbalová, Kateřina	Global gene expression changes in human	Particle and Fibre Toxicology	2012
PAHs	DUT	Helena Líbalová, Kateřina	Global gene expression changes in human	Particle and Fibre Toxicology	2012
PAHs	DYNC1LI2	Helena Líbalová, Kateřina	Global gene expression changes in human	Particle and Fibre Toxicology	2012
PAHs	DYRK2	Helena Líbalová, Kateřina	Global gene expression changes in human	Particle and Fibre Toxicology	2012
PAHs	DYRK3	Helena Líbalová, Kateřina	Global gene expression changes in human	Particle and Fibre Toxicology	2012
PAHs	ECHDC3	Helena Líbalová, Kateřina	Global gene expression changes in human	Particle and Fibre Toxicology	2012
PAHs	EDG1	Helena Líbalová, Kateřina	Global gene expression changes in human	Particle and Fibre Toxicology	2012
PAHs	EFNB1	Helena Líbalová, Kateřina	Global gene expression changes in human	Particle and Fibre Toxicology	2012
PAHs	EGFR	Helena Líbalová, Kateřina	Global gene expression changes in human	Particle and Fibre Toxicology	2012
PAHs	EHD1	Helena Líbalová, Kateřina	Global gene expression changes in human	Particle and Fibre Toxicology	2012
PAHs	EID1	Helena Líbalová, Kateřina	Global gene expression changes in human	Particle and Fibre Toxicology	2012
PAHs	EIF2C2	Helena Líbalová, Kateřina	Global gene expression changes in human	Particle and Fibre Toxicology	2012
PAHs	ELAC1	Helena Líbalová, Kateřina	Global gene expression changes in human	Particle and Fibre Toxicology	2012
PAHs	ELF2	Helena Líbalová, Kateřina	Global gene expression changes in human	Particle and Fibre Toxicology	2012
PAHs	ELL2	Helena Líbalová, Kateřina	Global gene expression changes in human	Particle and Fibre Toxicology	2012
PAHs	EMILIN1	Helena Líbalová, Kateřina	Global gene expression changes in human	Particle and Fibre Toxicology	2012
PAHs	EML4	Helena Líbalová, Kateřina	Global gene expression changes in human	Particle and Fibre Toxicology	2012
PAHs	EMP1	Helena Líbalová, Kateřina	Global gene expression changes in human	Particle and Fibre Toxicology	2012
PAHs	ENAH	Helena Líbalová, Kateřina	Global gene expression changes in human	Particle and Fibre Toxicology	2012
PAHs	ENOPH1	Helena Líbalová, Kateřina	Global gene expression changes in human	Particle and Fibre Toxicology	2012
PAHs	EPB41L5	Helena Líbalová, Kateřina	Global gene expression changes in human	Particle and Fibre Toxicology	2012
PAHs	EPS15	Helena Líbalová, Kateřina	Global gene expression changes in human	Particle and Fibre Toxicology	2012
PAHs	ERBB2	Helena Líbalová, Kateřina	Global gene expression changes in human	Particle and Fibre Toxicology	2012
PAHs	ERCC5	Helena Líbalová, Kateřina	Global gene expression changes in human	Particle and Fibre Toxicology	2012
PAHs	ERO1L	Helena Líbalová, Kateřina	Global gene expression changes in human	Particle and Fibre Toxicology	2012
PAHs	ESRRA	Helena Líbalová, Kateřina	Global gene expression changes in human	Particle and Fibre Toxicology	2012
PAHs	ESRRAP2	Helena Líbalová, Kateřina	Global gene expression changes in human	Particle and Fibre Toxicology	2012
PAHs	ETFB	Helena Líbalová, Kateřina	Global gene expression changes in human	Particle and Fibre Toxicology	2012
PAHs	EXOSC10	Helena Líbalová, Kateřina	Global gene expression changes in human	Particle and Fibre Toxicology	2012
PAHs	EXOSC4	Helena Líbalová, Kateřina	Global gene expression changes in human	Particle and Fibre Toxicology	2012
PAHs	EXTL3	Helena Líbalová, Kateřina	Global gene expression changes in human	Particle and Fibre Toxicology	2012
PAHs	FADS2	Helena Líbalová, Kateřina	Global gene expression changes in human	Particle and Fibre Toxicology	2012
PAHs	FAHD2A	Helena Líbalová, Kateřina	Global gene expression changes in human	Particle and Fibre Toxicology	2012
PAHs	FAM108C1	Helena Líbalová, Kateřina	Global gene expression changes in human	Particle and Fibre Toxicology	2012
PAHs	FAM114A2	Helena Líbalová, Kateřina	Global gene expression changes in human	Particle and Fibre Toxicology	2012
PAHs	FAM122B	Helena Líbalová, Kateřina	Global gene expression changes in human	Particle and Fibre Toxicology	2012
PAHs	FAM127A	Helena Líbalová, Kateřina	Global gene expression changes in human	Particle and Fibre Toxicology	2012
PAHs	FAM171A1	Helena Líbalová, Kateřina	Global gene expression changes in human	Particle and Fibre Toxicology	2012
PAHs	FAM176B	Helena Líbalová, Kateřina	Global gene expression changes in human	Particle and Fibre Toxicology	2012
PAHs	FAM20C	Helena Líbalová, Kateřina	Global gene expression changes in human	Particle and Fibre Toxicology	2012
PAHs	FAM39DP	Helena Líbalová, Kateřina	Global gene expression changes in human	Particle and Fibre Toxicology	2012
PAHs	FAM39E	Helena Líbalová, Kateřina	Global gene expression changes in human	Particle and Fibre Toxicology	2012
PAHs	FAM65A	Helena Líbalová, Kateřina	Global gene expression changes in human	Particle and Fibre Toxicology	2012
PAHs	FAM86A	Helena Líbalová, Kateřina	Global gene expression changes in human	Particle and Fibre Toxicology	2012
PAHs	FARP1	Helena Líbalová, Kateřina	Global gene expression changes in human	Particle and Fibre Toxicology	2012
PAHs	FASTK	Helena Líbalová, Kateřina	Global gene expression changes in human	Particle and Fibre Toxicology	2012
PAHs	FASTKD2	Helena Líbalová, Kateřina	Global gene expression changes in human	Particle and Fibre Toxicology	2012
PAHs	FBXO17	Helena Líbalová, Kateřina	Global gene expression changes in human	Particle and Fibre Toxicology	2012
PAHs	FBXO7	Helena Líbalová, Kateřina	Global gene expression changes in human	Particle and Fibre Toxicology	2012
PAHs	FEM1B	Helena Líbalová, Kateřina	Global gene expression changes in human	Particle and Fibre Toxicology	2012
PAHs	FEZ1	Helena Líbalová, Kateřina	Global gene expression changes in human	Particle and Fibre Toxicology	2012
PAHs	FGF5	Helena Líbalová, Kateřina	Global gene expression changes in human	Particle and Fibre Toxicology	2012
PAHs	FGL2	Helena Líbalová, Kateřina	Global gene expression changes in human	Particle and Fibre Toxicology	2012
PAHs	FHL2	Helena Líbalová, Kateřina	Global gene expression changes in human	Particle and Fibre Toxicology	2012
PAHs	FIGNL1	Helena Líbalová, Kateřina	Global gene expression changes in human	Particle and Fibre Toxicology	2012
PAHs	FKBP7	Helena Líbalová, Kateřina	Global gene expression changes in human	Particle and Fibre Toxicology	2012
PAHs	FKTN	Helena Líbalová, Kateřina	Global gene expression changes in human	Particle and Fibre Toxicology	2012
PAHs	FLAD1	Helena Líbalová, Kateřina	Global gene expression changes in human	Particle and Fibre Toxicology	2012
PAHs	FLII	Helena Líbalová, Kateřina	Global gene expression changes in human	Particle and Fibre Toxicology	2012
PAHs	FLJ10374	Helena Líbalová, Kateřina	Global gene expression changes in human	Particle and Fibre Toxicology	2012
PAHs	FLJ35801	Helena Líbalová, Kateřina	Global gene expression changes in human	Particle and Fibre Toxicology	2012
PAHs	FLJ40142	Helena Líbalová, Kateřina	Global gene expression changes in human	Particle and Fibre Toxicology	2012
PAHs	FLJ42986	Helena Líbalová, Kateřina	Global gene expression changes in human	Particle and Fibre Toxicology	2012
PAHs	FLYWCH1	Helena Líbalová, Kateřina	Global gene expression changes in human	Particle and Fibre Toxicology	2012
PAHs	FNIP1	Helena Líbalová, Kateřina	Global gene expression changes in human	Particle and Fibre Toxicology	2012
PAHs	FOXF2	Helena Líbalová, Kateřina	Global gene expression changes in human	Particle and Fibre Toxicology	2012
PAHs	FRS3	Helena Líbalová, Kateřina	Global gene expression changes in human	Particle and Fibre Toxicology	2012
PAHs	FZD7	Helena Líbalová, Kateřina	Global gene expression changes in human	Particle and Fibre Toxicology	2012
PAHs	FZD9	Helena Líbalová, Kateřina	Global gene expression changes in human	Particle and Fibre Toxicology	2012
PAHs	G6PD	Helena Líbalová, Kateřina	Global gene expression changes in human	Particle and Fibre Toxicology	2012
PAHs	GAL	Helena Líbalová, Kateřina	Global gene expression changes in human	Particle and Fibre Toxicology	2012
PAHs	GALNT4	Helena Líbalová, Kateřina	Global gene expression changes in human	Particle and Fibre Toxicology	2012
PAHs	GALNTL4	Helena Líbalová, Kateřina	Global gene expression changes in human	Particle and Fibre Toxicology	2012
PAHs	GAS6	Helena Líbalová, Kateřina	Global gene expression changes in human	Particle and Fibre Toxicology	2012
PAHs	GBA	Helena Líbalová, Kateřina	Global gene expression changes in human	Particle and Fibre Toxicology	2012
PAHs	GBA2	Helena Líbalová, Kateřina	Global gene expression changes in human	Particle and Fibre Toxicology	2012
PAHs	GIYD1	Helena Líbalová, Kateřina	Global gene expression changes in human	Particle and Fibre Toxicology	2012
PAHs	GLB1	Helena Líbalová, Kateřina	Global gene expression changes in human	Particle and Fibre Toxicology	2012
PAHs	GLI2	Helena Líbalová, Kateřina	Global gene expression changes in human	Particle and Fibre Toxicology	2012
PAHs	GMEB1	Helena Líbalová, Kateřina	Global gene expression changes in human	Particle and Fibre Toxicology	2012
PAHs	GMFB	Helena Líbalová, Kateřina	Global gene expression changes in human	Particle and Fibre Toxicology	2012
PAHs	GNAI1	Helena Líbalová, Kateřina	Global gene expression changes in human	Particle and Fibre Toxicology	2012
PAHs	GNB4	Helena Líbalová, Kateřina	Global gene expression changes in human	Particle and Fibre Toxicology	2012
PAHs	GORASP2	Helena Líbalová, Kateřina	Global gene expression changes in human	Particle and Fibre Toxicology	2012
PAHs	GRK6	Helena Líbalová, Kateřina	Global gene expression changes in human	Particle and Fibre Toxicology	2012
PAHs	GSDMB	Helena Líbalová, Kateřina	Global gene expression changes in human	Particle and Fibre Toxicology	2012
PAHs	GSTM2	Helena Líbalová, Kateřina	Global gene expression changes in human	Particle and Fibre Toxicology	2012
PAHs	GSTT1	Helena Líbalová, Kateřina	Global gene expression changes in human	Particle and Fibre Toxicology	2012
PAHs	GTF3C3	Helena Líbalová, Kateřina	Global gene expression changes in human	Particle and Fibre Toxicology	2012

PAHs	GYS1	Helena Líbalová, Kateřina	Global gene expression changes in human	Particle and Fibre Toxicology	2012
PAHs	H1F0	Helena Líbalová, Kateřina	Global gene expression changes in human	Particle and Fibre Toxicology	2012
PAHs	H2AFX	Helena Líbalová, Kateřina	Global gene expression changes in human	Particle and Fibre Toxicology	2012
PAHs	H2AFY2	Helena Líbalová, Kateřina	Global gene expression changes in human	Particle and Fibre Toxicology	2012
PAHs	H3F3B	Helena Líbalová, Kateřina	Global gene expression changes in human	Particle and Fibre Toxicology	2012
PAHs	HAPLN3	Helena Líbalová, Kateřina	Global gene expression changes in human	Particle and Fibre Toxicology	2012
PAHs	HDAC3	Helena Líbalová, Kateřina	Global gene expression changes in human	Particle and Fibre Toxicology	2012
PAHs	HDAC6	Helena Líbalová, Kateřina	Global gene expression changes in human	Particle and Fibre Toxicology	2012
PAHs	HELZ	Helena Líbalová, Kateřina	Global gene expression changes in human	Particle and Fibre Toxicology	2012
PAHs	HERC3	Helena Líbalová, Kateřina	Global gene expression changes in human	Particle and Fibre Toxicology	2012
PAHs	HIST3H2A	Helena Líbalová, Kateřina	Global gene expression changes in human	Particle and Fibre Toxicology	2012
PAHs	HIVEP1	Helena Líbalová, Kateřina	Global gene expression changes in human	Particle and Fibre Toxicology	2012
PAHs	HMMR	Helena Líbalová, Kateřina	Global gene expression changes in human	Particle and Fibre Toxicology	2012
PAHs	HN1	Helena Líbalová, Kateřina	Global gene expression changes in human	Particle and Fibre Toxicology	2012
PAHs	HNRNPA0	Helena Líbalová, Kateřina	Global gene expression changes in human	Particle and Fibre Toxicology	2012
PAHs	HNRNPC	Helena Líbalová, Kateřina	Global gene expression changes in human	Particle and Fibre Toxicology	2012
PAHs	HNRPH1	Helena Líbalová, Kateřina	Global gene expression changes in human	Particle and Fibre Toxicology	2012
PAHs	HNRPH3	Helena Líbalová, Kateřina	Global gene expression changes in human	Particle and Fibre Toxicology	2012
PAHs	HOXA4	Helena Líbalová, Kateřina	Global gene expression changes in human	Particle and Fibre Toxicology	2012
PAHs	HOXB2	Helena Líbalová, Kateřina	Global gene expression changes in human	Particle and Fibre Toxicology	2012
PAHs	HOXB4	Helena Líbalová, Kateřina	Global gene expression changes in human	Particle and Fibre Toxicology	2012
PAHs	HOXB8	Helena Líbalová, Kateřina	Global gene expression changes in human	Particle and Fibre Toxicology	2012
PAHs	HOXC6	Helena Líbalová, Kateřina	Global gene expression changes in human	Particle and Fibre Toxicology	2012
PAHs	HPS3	Helena Líbalová, Kateřina	Global gene expression changes in human	Particle and Fibre Toxicology	2012
PAHs	HPS4	Helena Líbalová, Kateřina	Global gene expression changes in human	Particle and Fibre Toxicology	2012
PAHs	HRASLS3	Helena Líbalová, Kateřina	Global gene expression changes in human	Particle and Fibre Toxicology	2012
PAHs	HSD11B1L	Helena Líbalová, Kateřina	Global gene expression changes in human	Particle and Fibre Toxicology	2012
PAHs	HSD17B2	Helena Líbalová, Kateřina	Global gene expression changes in human	Particle and Fibre Toxicology	2012
PAHs	HSF2BP	Helena Líbalová, Kateřina	Global gene expression changes in human	Particle and Fibre Toxicology	2012
PAHs	HSPA1B	Helena Líbalová, Kateřina	Global gene expression changes in human	Particle and Fibre Toxicology	2012
PAHs	HSPA1L	Helena Líbalová, Kateřina	Global gene expression changes in human	Particle and Fibre Toxicology	2012
PAHs	HSPA2	Helena Líbalová, Kateřina	Global gene expression changes in human	Particle and Fibre Toxicology	2012
PAHs	HSPA4	Helena Líbalová, Kateřina	Global gene expression changes in human	Particle and Fibre Toxicology	2012
PAHs	HSPA9	Helena Líbalová, Kateřina	Global gene expression changes in human	Particle and Fibre Toxicology	2012
PAHs	HSPC268	Helena Líbalová, Kateřina	Global gene expression changes in human	Particle and Fibre Toxicology	2012
PAHs	IARS2	Helena Líbalová, Kateřina	Global gene expression changes in human	Particle and Fibre Toxicology	2012
PAHs	ICK	Helena Líbalová, Kateřina	Global gene expression changes in human	Particle and Fibre Toxicology	2012
PAHs	IDH1	Helena Líbalová, Kateřina	Global gene expression changes in human	Particle and Fibre Toxicology	2012
PAHs	IFIT1	Helena Líbalová, Kateřina	Global gene expression changes in human	Particle and Fibre Toxicology	2012
PAHs	IFRD1	Helena Líbalová, Kateřina	Global gene expression changes in human	Particle and Fibre Toxicology	2012
PAHs	IGFBP2	Helena Líbalová, Kateřina	Global gene expression changes in human	Particle and Fibre Toxicology	2012
PAHs	IGFBP3	Helena Líbalová, Kateřina	Global gene expression changes in human	Particle and Fibre Toxicology	2012
PAHs	IIP45	Helena Líbalová, Kateřina	Global gene expression changes in human	Particle and Fibre Toxicology	2012
PAHs	IKBKB	Helena Líbalová, Kateřina	Global gene expression changes in human	Particle and Fibre Toxicology	2012
PAHs	IL11	Helena Líbalová, Kateřina	Global gene expression changes in human	Particle and Fibre Toxicology	2012
PAHs	IL6	Helena Líbalová, Kateřina	Global gene expression changes in human	Particle and Fibre Toxicology	2012
PAHs	INF2	Helena Líbalová, Kateřina	Global gene expression changes in human	Particle and Fibre Toxicology	2012
PAHs	ING2	Helena Líbalová, Kateřina	Global gene expression changes in human	Particle and Fibre Toxicology	2012
PAHs	INPPL1	Helena Líbalová, Kateřina	Global gene expression changes in human	Particle and Fibre Toxicology	2012
PAHs	INSIG1	Helena Líbalová, Kateřina	Global gene expression changes in human	Particle and Fibre Toxicology	2012
PAHs	INVS	Helena Líbalová, Kateřina	Global gene expression changes in human	Particle and Fibre Toxicology	2012
PAHs	IQWD1	Helena Líbalová, Kateřina	Global gene expression changes in human	Particle and Fibre Toxicology	2012
PAHs	IRF2BP2	Helena Líbalová, Kateřina	Global gene expression changes in human	Particle and Fibre Toxicology	2012
PAHs	ITPA	Helena Líbalová, Kateřina	Global gene expression changes in human	Particle and Fibre Toxicology	2012
PAHs	ITSN1	Helena Líbalová, Kateřina	Global gene expression changes in human	Particle and Fibre Toxicology	2012
PAHs	JOSD1	Helena Líbalová, Kateřina	Global gene expression changes in human	Particle and Fibre Toxicology	2012
PAHs	KBTBD2	Helena Líbalová, Kateřina	Global gene expression changes in human	Particle and Fibre Toxicology	2012
PAHs	KCNH2	Helena Líbalová, Kateřina	Global gene expression changes in human	Particle and Fibre Toxicology	2012
PAHs	KCNK2	Helena Líbalová, Kateřina	Global gene expression changes in human	Particle and Fibre Toxicology	2012
PAHs	KCTD12	Helena Líbalová, Kateřina	Global gene expression changes in human	Particle and Fibre Toxicology	2012
PAHs	KIAA0241	Helena Líbalová, Kateřina	Global gene expression changes in human	Particle and Fibre Toxicology	2012
PAHs	KIAA0323	Helena Líbalová, Kateřina	Global gene expression changes in human	Particle and Fibre Toxicology	2012
PAHs	KIAA0415	Helena Líbalová, Kateřina	Global gene expression changes in human	Particle and Fibre Toxicology	2012
PAHs	KIAA0427	Helena Líbalová, Kateřina	Global gene expression changes in human	Particle and Fibre Toxicology	2012
PAHs	KIAA1026	Helena Líbalová, Kateřina	Global gene expression changes in human	Particle and Fibre Toxicology	2012
PAHs	KIAA1211	Helena Líbalová, Kateřina	Global gene expression changes in human	Particle and Fibre Toxicology	2012
PAHs	KIAA1217	Helena Líbalová, Kateřina	Global gene expression changes in human	Particle and Fibre Toxicology	2012
PAHs	KIAA1279	Helena Líbalová, Kateřina	Global gene expression changes in human	Particle and Fibre Toxicology	2012
PAHs	KIAA1530	Helena Líbalová, Kateřina	Global gene expression changes in human	Particle and Fibre Toxicology	2012
PAHs	KIAA1641	Helena Líbalová, Kateřina	Global gene expression changes in human	Particle and Fibre Toxicology	2012
PAHs	KIAA1754	Helena Líbalová, Kateřina	Global gene expression changes in human	Particle and Fibre Toxicology	2012
PAHs	KIAA2010	Helena Líbalová, Kateřina	Global gene expression changes in human	Particle and Fibre Toxicology	2012
PAHs	KITLG	Helena Líbalová, Kateřina	Global gene expression changes in human	Particle and Fibre Toxicology	2012
PAHs	KLHL24	Helena Líbalová, Kateřina	Global gene expression changes in human	Particle and Fibre Toxicology	2012
PAHs	KLHL35	Helena Líbalová, Kateřina	Global gene expression changes in human	Particle and Fibre Toxicology	2012
PAHs	KLHL9	Helena Líbalová, Kateřina	Global gene expression changes in human	Particle and Fibre Toxicology	2012
PAHs	KRIT1	Helena Líbalová, Kateřina	Global gene expression changes in human	Particle and Fibre Toxicology	2012
PAHs	KRTCAP2	Helena Líbalová, Kateřina	Global gene expression changes in human	Particle and Fibre Toxicology	2012
PAHs	LACTB	Helena Líbalová, Kateřina	Global gene expression changes in human	Particle and Fibre Toxicology	2012
PAHs	LAMP1	Helena Líbalová, Kateřina	Global gene expression changes in human	Particle and Fibre Toxicology	2012
PAHs	LANCL2	Helena Líbalová, Kateřina	Global gene expression changes in human	Particle and Fibre Toxicology	2012
PAHs	LARGE	Helena Líbalová, Kateřina	Global gene expression changes in human	Particle and Fibre Toxicology	2012
PAHs	LAYN	Helena Líbalová, Kateřina	Global gene expression changes in human	Particle and Fibre Toxicology	2012
PAHs	LETMD1	Helena Líbalová, Kateřina	Global gene expression changes in human	Particle and Fibre Toxicology	2012
PAHs	LHFP	Helena Líbalová, Kateřina	Global gene expression changes in human	Particle and Fibre Toxicology	2012
PAHs	LIPA	Helena Líbalová, Kateřina	Global gene expression changes in human	Particle and Fibre Toxicology	2012
PAHs	LIPT1	Helena Líbalová, Kateřina	Global gene expression changes in human	Particle and Fibre Toxicology	2012
PAHs	LITAF	Helena Líbalová, Kateřina	Global gene expression changes in human	Particle and Fibre Toxicology	2012
PAHs	LLGL1	Helena Líbalová, Kateřina	Global gene expression changes in human	Particle and Fibre Toxicology	2012

PAHs	LMNB2	Helena Líbalová, Kateřina	Global gene expression changes in human	Particle and Fibre Toxicology	2012
PAHs	LOC221442	Helena Líbalová, Kateřina	Global gene expression changes in human	Particle and Fibre Toxicology	2012
PAHs	LOC221710	Helena Líbalová, Kateřina	Global gene expression changes in human	Particle and Fibre Toxicology	2012
PAHs	LOC23117	Helena Líbalová, Kateřina	Global gene expression changes in human	Particle and Fibre Toxicology	2012
PAHs	LOC26010	Helena Líbalová, Kateřina	Global gene expression changes in human	Particle and Fibre Toxicology	2012
PAHs	LOC284023	Helena Líbalová, Kateřina	Global gene expression changes in human	Particle and Fibre Toxicology	2012
PAHs	LOC286016	Helena Líbalová, Kateřina	Global gene expression changes in human	Particle and Fibre Toxicology	2012
PAHs	LOC375295	Helena Líbalová, Kateřina	Global gene expression changes in human	Particle and Fibre Toxicology	2012
PAHs	LOC388397	Helena Líbalová, Kateřina	Global gene expression changes in human	Particle and Fibre Toxicology	2012
PAHs	LOC400506	Helena Líbalová, Kateřina	Global gene expression changes in human	Particle and Fibre Toxicology	2012
PAHs	LOC401093	Helena Líbalová, Kateřina	Global gene expression changes in human	Particle and Fibre Toxicology	2012
PAHs	LOC401397	Helena Líbalová, Kateřina	Global gene expression changes in human	Particle and Fibre Toxicology	2012
PAHs	LOC554206	Helena Líbalová, Kateřina	Global gene expression changes in human	Particle and Fibre Toxicology	2012
PAHs	LOC644743	Helena Líbalová, Kateřina	Global gene expression changes in human	Particle and Fibre Toxicology	2012
PAHs	LOC644762	Helena Líbalová, Kateřina	Global gene expression changes in human	Particle and Fibre Toxicology	2012
PAHs	LOC646345	Helena Líbalová, Kateřina	Global gene expression changes in human	Particle and Fibre Toxicology	2012
PAHs	LOC647037	Helena Líbalová, Kateřina	Global gene expression changes in human	Particle and Fibre Toxicology	2012
PAHs	LOC648189	Helena Líbalová, Kateřina	Global gene expression changes in human	Particle and Fibre Toxicology	2012
PAHs	LOC649095	Helena Líbalová, Kateřina	Global gene expression changes in human	Particle and Fibre Toxicology	2012
PAHs	LOC650339	Helena Líbalová, Kateřina	Global gene expression changes in human	Particle and Fibre Toxicology	2012
PAHs	LOC652545	Helena Líbalová, Kateřina	Global gene expression changes in human	Particle and Fibre Toxicology	2012
PAHs	LOC653158	Helena Líbalová, Kateřina	Global gene expression changes in human	Particle and Fibre Toxicology	2012
PAHs	LOC653344	Helena Líbalová, Kateřina	Global gene expression changes in human	Particle and Fibre Toxicology	2012
PAHs	LOC653450	Helena Líbalová, Kateřina	Global gene expression changes in human	Particle and Fibre Toxicology	2012
PAHs	LOC653972	Helena Líbalová, Kateřina	Global gene expression changes in human	Particle and Fibre Toxicology	2012
PAHs	LOC728635	Helena Líbalová, Kateřina	Global gene expression changes in human	Particle and Fibre Toxicology	2012
PAHs	LOC728758	Helena Líbalová, Kateřina	Global gene expression changes in human	Particle and Fibre Toxicology	2012
PAHs	LOC729843	Helena Líbalová, Kateřina	Global gene expression changes in human	Particle and Fibre Toxicology	2012
PAHs	LOC730051	Helena Líbalová, Kateřina	Global gene expression changes in human	Particle and Fibre Toxicology	2012
PAHs	LOC732007	Helena Líbalová, Kateřina	Global gene expression changes in human	Particle and Fibre Toxicology	2012
PAHs	LOC732165	Helena Líbalová, Kateřina	Global gene expression changes in human	Particle and Fibre Toxicology	2012
PAHs	LOC81691	Helena Líbalová, Kateřina	Global gene expression changes in human	Particle and Fibre Toxicology	2012
PAHs	LPHN1	Helena Líbalová, Kateřina	Global gene expression changes in human	Particle and Fibre Toxicology	2012
PAHs	LRFN4	Helena Líbalová, Kateřina	Global gene expression changes in human	Particle and Fibre Toxicology	2012
PAHs	LRP5L	Helena Líbalová, Kateřina	Global gene expression changes in human	Particle and Fibre Toxicology	2012
PAHs	LRRC42	Helena Líbalová, Kateřina	Global gene expression changes in human	Particle and Fibre Toxicology	2012
PAHs	LRRC8D	Helena Líbalová, Kateřina	Global gene expression changes in human	Particle and Fibre Toxicology	2012
PAHs	LSM2	Helena Líbalová, Kateřina	Global gene expression changes in human	Particle and Fibre Toxicology	2012
PAHs	LSS	Helena Líbalová, Kateřina	Global gene expression changes in human	Particle and Fibre Toxicology	2012
PAHs	LTV1	Helena Líbalová, Kateřina	Global gene expression changes in human	Particle and Fibre Toxicology	2012
PAHs	LYAR	Helena Líbalová, Kateřina	Global gene expression changes in human	Particle and Fibre Toxicology	2012
PAHs	LYRM5	Helena Líbalová, Kateřina	Global gene expression changes in human	Particle and Fibre Toxicology	2012
PAHs	MAB21L2	Helena Líbalová, Kateřina	Global gene expression changes in human	Particle and Fibre Toxicology	2012
PAHs	MACROD1	Helena Líbalová, Kateřina	Global gene expression changes in human	Particle and Fibre Toxicology	2012
PAHs	MADD	Helena Líbalová, Kateřina	Global gene expression changes in human	Particle and Fibre Toxicology	2012
PAHs	MAGED2	Helena Líbalová, Kateřina	Global gene expression changes in human	Particle and Fibre Toxicology	2012
PAHs	Magmas	Helena Líbalová, Kateřina	Global gene expression changes in human	Particle and Fibre Toxicology	2012
PAHs	MAN2A2	Helena Líbalová, Kateřina	Global gene expression changes in human	Particle and Fibre Toxicology	2012
PAHs	MAP1A	Helena Líbalová, Kateřina	Global gene expression changes in human	Particle and Fibre Toxicology	2012
PAHs	MAP1LC3A	Helena Líbalová, Kateřina	Global gene expression changes in human	Particle and Fibre Toxicology	2012
PAHs	MAP3K7	Helena Líbalová, Kateřina	Global gene expression changes in human	Particle and Fibre Toxicology	2012
PAHs	MAPK14	Helena Líbalová, Kateřina	Global gene expression changes in human	Particle and Fibre Toxicology	2012
PAHs	MAPK7	Helena Líbalová, Kateřina	Global gene expression changes in human	Particle and Fibre Toxicology	2012
PAHs	MAPRE2	Helena Líbalová, Kateřina	Global gene expression changes in human	Particle and Fibre Toxicology	2012
PAHs	MARCKS	Helena Líbalová, Kateřina	Global gene expression changes in human	Particle and Fibre Toxicology	2012
PAHs	MARK3	Helena Líbalová, Kateřina	Global gene expression changes in human	Particle and Fibre Toxicology	2012
PAHs	MBD5	Helena Líbalová, Kateřina	Global gene expression changes in human	Particle and Fibre Toxicology	2012
PAHs	MBIP	Helena Líbalová, Kateřina	Global gene expression changes in human	Particle and Fibre Toxicology	2012
PAHs	MBP	Helena Líbalová, Kateřina	Global gene expression changes in human	Particle and Fibre Toxicology	2012
PAHs	MCM5	Helena Líbalová, Kateřina	Global gene expression changes in human	Particle and Fibre Toxicology	2012
PAHs	ME1	Helena Líbalová, Kateřina	Global gene expression changes in human	Particle and Fibre Toxicology	2012
PAHs	MED22	Helena Líbalová, Kateřina	Global gene expression changes in human	Particle and Fibre Toxicology	2012
PAHs	MED4	Helena Líbalová, Kateřina	Global gene expression changes in human	Particle and Fibre Toxicology	2012
PAHs	MED6	Helena Líbalová, Kateřina	Global gene expression changes in human	Particle and Fibre Toxicology	2012
PAHs	MEIS1	Helena Líbalová, Kateřina	Global gene expression changes in human	Particle and Fibre Toxicology	2012
PAHs	MEIS2	Helena Líbalová, Kateřina	Global gene expression changes in human	Particle and Fibre Toxicology	2012
PAHs	MELK	Helena Líbalová, Kateřina	Global gene expression changes in human	Particle and Fibre Toxicology	2012
PAHs	METTL1	Helena Líbalová, Kateřina	Global gene expression changes in human	Particle and Fibre Toxicology	2012
PAHs	MEX3D	Helena Líbalová, Kateřina	Global gene expression changes in human	Particle and Fibre Toxicology	2012
PAHs	MFAP1	Helena Líbalová, Kateřina	Global gene expression changes in human	Particle and Fibre Toxicology	2012
PAHs	MGC16121	Helena Líbalová, Kateřina	Global gene expression changes in human	Particle and Fibre Toxicology	2012
PAHs	MGST2	Helena Líbalová, Kateřina	Global gene expression changes in human	Particle and Fibre Toxicology	2012
PAHs	MLL4	Helena Líbalová, Kateřina	Global gene expression changes in human	Particle and Fibre Toxicology	2012
PAHs	MLLT11	Helena Líbalová, Kateřina	Global gene expression changes in human	Particle and Fibre Toxicology	2012
PAHs	MLLT6	Helena Líbalová, Kateřina	Global gene expression changes in human	Particle and Fibre Toxicology	2012
PAHs	MMP23B	Helena Líbalová, Kateřina	Global gene expression changes in human	Particle and Fibre Toxicology	2012
PAHs	MPHOSPH9	Helena Líbalová, Kateřina	Global gene expression changes in human	Particle and Fibre Toxicology	2012
PAHs	MPND	Helena Líbalová, Kateřina	Global gene expression changes in human	Particle and Fibre Toxicology	2012
PAHs	MRPL42	Helena Líbalová, Kateřina	Global gene expression changes in human	Particle and Fibre Toxicology	2012
PAHs	MRPS18B	Helena Líbalová, Kateřina	Global gene expression changes in human	Particle and Fibre Toxicology	2012
PAHs	MRPS24	Helena Líbalová, Kateřina	Global gene expression changes in human	Particle and Fibre Toxicology	2012
PAHs	MRPS33	Helena Líbalová, Kateřina	Global gene expression changes in human	Particle and Fibre Toxicology	2012
PAHs	MRRF	Helena Líbalová, Kateřina	Global gene expression changes in human	Particle and Fibre Toxicology	2012
PAHs	MSL3L1	Helena Líbalová, Kateřina	Global gene expression changes in human	Particle and Fibre Toxicology	2012
PAHs	MSRA	Helena Líbalová, Kateřina	Global gene expression changes in human	Particle and Fibre Toxicology	2012
PAHs	MTERF	Helena Líbalová, Kateřina	Global gene expression changes in human	Particle and Fibre Toxicology	2012
PAHs	MTHFD1L	Helena Líbalová, Kateřina	Global gene expression changes in human	Particle and Fibre Toxicology	2012
PAHs	MTIF2	Helena Líbalová, Kateřina	Global gene expression changes in human	Particle and Fibre Toxicology	2012
PAHs	MTMR3	Helena Líbalová, Kateřina	Global gene expression changes in human	Particle and Fibre Toxicology	2012

PAHs	MTX3	Helena Líbalová, Kateřina	Global gene expression changes in human	Particle and Fibre Toxicology	2012
PAHs	MUM1	Helena Líbalová, Kateřina	Global gene expression changes in human	Particle and Fibre Toxicology	2012
PAHs	MUS81	Helena Líbalová, Kateřina	Global gene expression changes in human	Particle and Fibre Toxicology	2012
PAHs	MUTYH	Helena Líbalová, Kateřina	Global gene expression changes in human	Particle and Fibre Toxicology	2012
PAHs	MXI1	Helena Líbalová, Kateřina	Global gene expression changes in human	Particle and Fibre Toxicology	2012
PAHs	MYL6	Helena Líbalová, Kateřina	Global gene expression changes in human	Particle and Fibre Toxicology	2012
PAHs	NAE1	Helena Líbalová, Kateřina	Global gene expression changes in human	Particle and Fibre Toxicology	2012
PAHs	NASP	Helena Líbalová, Kateřina	Global gene expression changes in human	Particle and Fibre Toxicology	2012
PAHs	NBN	Helena Líbalová, Kateřina	Global gene expression changes in human	Particle and Fibre Toxicology	2012
PAHs	NCAPG	Helena Líbalová, Kateřina	Global gene expression changes in human	Particle and Fibre Toxicology	2012
PAHs	NDRG3	Helena Líbalová, Kateřina	Global gene expression changes in human	Particle and Fibre Toxicology	2012
PAHs	NECAB3	Helena Líbalová, Kateřina	Global gene expression changes in human	Particle and Fibre Toxicology	2012
PAHs	NEDD4L	Helena Líbalová, Kateřina	Global gene expression changes in human	Particle and Fibre Toxicology	2012
PAHs	NEK11	Helena Líbalová, Kateřina	Global gene expression changes in human	Particle and Fibre Toxicology	2012
PAHs	NEK6	Helena Líbalová, Kateřina	Global gene expression changes in human	Particle and Fibre Toxicology	2012
PAHs	NELF	Helena Líbalová, Kateřina	Global gene expression changes in human	Particle and Fibre Toxicology	2012
PAHs	NFIL3	Helena Líbalová, Kateřina	Global gene expression changes in human	Particle and Fibre Toxicology	2012
PAHs	NGF	Helena Líbalová, Kateřina	Global gene expression changes in human	Particle and Fibre Toxicology	2012
PAHs	NGRN	Helena Líbalová, Kateřina	Global gene expression changes in human	Particle and Fibre Toxicology	2012
PAHs	NICN1	Helena Líbalová, Kateřina	Global gene expression changes in human	Particle and Fibre Toxicology	2012
PAHs	NIPSNAP3A	Helena Líbalová, Kateřina	Global gene expression changes in human	Particle and Fibre Toxicology	2012
PAHs	NLRX1	Helena Líbalová, Kateřina	Global gene expression changes in human	Particle and Fibre Toxicology	2012
PAHs	NME7	Helena Líbalová, Kateřina	Global gene expression changes in human	Particle and Fibre Toxicology	2012
PAHs	NOD1	Helena Líbalová, Kateřina	Global gene expression changes in human	Particle and Fibre Toxicology	2012
PAHs	NPAL3	Helena Líbalová, Kateřina	Global gene expression changes in human	Particle and Fibre Toxicology	2012
PAHs	NPHP3	Helena Líbalová, Kateřina	Global gene expression changes in human	Particle and Fibre Toxicology	2012
PAHs	NPM3	Helena Líbalová, Kateřina	Global gene expression changes in human	Particle and Fibre Toxicology	2012
PAHs	NR2F2	Helena Líbalová, Kateřina	Global gene expression changes in human	Particle and Fibre Toxicology	2012
PAHs	NR3C1	Helena Líbalová, Kateřina	Global gene expression changes in human	Particle and Fibre Toxicology	2012
PAHs	NRP1	Helena Líbalová, Kateřina	Global gene expression changes in human	Particle and Fibre Toxicology	2012
PAHs	NRSN2	Helena Líbalová, Kateřina	Global gene expression changes in human	Particle and Fibre Toxicology	2012
PAHs	NSF	Helena Líbalová, Kateřina	Global gene expression changes in human	Particle and Fibre Toxicology	2012
PAHs	NSMCE2	Helena Líbalová, Kateřina	Global gene expression changes in human	Particle and Fibre Toxicology	2012
PAHs	NUDT21	Helena Líbalová, Kateřina	Global gene expression changes in human	Particle and Fibre Toxicology	2012
PAHs	NUPL2	Helena Líbalová, Kateřina	Global gene expression changes in human	Particle and Fibre Toxicology	2012
PAHs	ODC1	Helena Líbalová, Kateřina	Global gene expression changes in human	Particle and Fibre Toxicology	2012
PAHs	ODF2	Helena Líbalová, Kateřina	Global gene expression changes in human	Particle and Fibre Toxicology	2012
PAHs	OIP5	Helena Líbalová, Kateřina	Global gene expression changes in human	Particle and Fibre Toxicology	2012
PAHs	ORAI1	Helena Líbalová, Kateřina	Global gene expression changes in human	Particle and Fibre Toxicology	2012
PAHs	ORC6L	Helena Líbalová, Kateřina	Global gene expression changes in human	Particle and Fibre Toxicology	2012
PAHs	OSBPL8	Helena Líbalová, Kateřina	Global gene expression changes in human	Particle and Fibre Toxicology	2012
PAHs	OTUD1	Helena Líbalová, Kateřina	Global gene expression changes in human	Particle and Fibre Toxicology	2012
PAHs	OTUD5	Helena Líbalová, Kateřina	Global gene expression changes in human	Particle and Fibre Toxicology	2012
PAHs	OVGP1	Helena Líbalová, Kateřina	Global gene expression changes in human	Particle and Fibre Toxicology	2012
PAHs	OXR1	Helena Líbalová, Kateřina	Global gene expression changes in human	Particle and Fibre Toxicology	2012
PAHs	P2RY5	Helena Líbalová, Kateřina	Global gene expression changes in human	Particle and Fibre Toxicology	2012
PAHs	PALB2	Helena Líbalová, Kateřina	Global gene expression changes in human	Particle and Fibre Toxicology	2012
PAHs	PAN2	Helena Líbalová, Kateřina	Global gene expression changes in human	Particle and Fibre Toxicology	2012
PAHs	PAPD5	Helena Líbalová, Kateřina	Global gene expression changes in human	Particle and Fibre Toxicology	2012
PAHs	PAPSS1	Helena Líbalová, Kateřina	Global gene expression changes in human	Particle and Fibre Toxicology	2012
PAHs	PARP2	Helena Líbalová, Kateřina	Global gene expression changes in human	Particle and Fibre Toxicology	2012
PAHs	PBK	Helena Líbalová, Kateřina	Global gene expression changes in human	Particle and Fibre Toxicology	2012
PAHs	PCDH18	Helena Líbalová, Kateřina	Global gene expression changes in human	Particle and Fibre Toxicology	2012
PAHs	PCDHB17	Helena Líbalová, Kateřina	Global gene expression changes in human	Particle and Fibre Toxicology	2012
PAHs	PCNA	Helena Líbalová, Kateřina	Global gene expression changes in human	Particle and Fibre Toxicology	2012
PAHs	PDCL3	Helena Líbalová, Kateřina	Global gene expression changes in human	Particle and Fibre Toxicology	2012
PAHs	PDGFRB	Helena Líbalová, Kateřina	Global gene expression changes in human	Particle and Fibre Toxicology	2012
PAHs	PDLIM3	Helena Líbalová, Kateřina	Global gene expression changes in human	Particle and Fibre Toxicology	2012
PAHs	PDLIM4	Helena Líbalová, Kateřina	Global gene expression changes in human	Particle and Fibre Toxicology	2012
PAHs	PDLIM7	Helena Líbalová, Kateřina	Global gene expression changes in human	Particle and Fibre Toxicology	2012
PAHs	PEA15	Helena Líbalová, Kateřina	Global gene expression changes in human	Particle and Fibre Toxicology	2012
PAHs	PEX11B	Helena Líbalová, Kateřina	Global gene expression changes in human	Particle and Fibre Toxicology	2012
PAHs	PEX16	Helena Líbalová, Kateřina	Global gene expression changes in human	Particle and Fibre Toxicology	2012
PAHs	PFKFB4	Helena Líbalová, Kateřina	Global gene expression changes in human	Particle and Fibre Toxicology	2012
PAHs	PFKP	Helena Líbalová, Kateřina	Global gene expression changes in human	Particle and Fibre Toxicology	2012
PAHs	PGA3	Helena Líbalová, Kateřina	Global gene expression changes in human	Particle and Fibre Toxicology	2012
PAHs	PGAM1	Helena Líbalová, Kateřina	Global gene expression changes in human	Particle and Fibre Toxicology	2012
PAHs	PGP	Helena Líbalová, Kateřina	Global gene expression changes in human	Particle and Fibre Toxicology	2012
PAHs	PHC3	Helena Líbalová, Kateřina	Global gene expression changes in human	Particle and Fibre Toxicology	2012
PAHs	PHF17	Helena Líbalová, Kateřina	Global gene expression changes in human	Particle and Fibre Toxicology	2012
PAHs	PHF3	Helena Líbalová, Kateřina	Global gene expression changes in human	Particle and Fibre Toxicology	2012
PAHs	PIB5PA	Helena Líbalová, Kateřina	Global gene expression changes in human	Particle and Fibre Toxicology	2012
PAHs	PIBF1	Helena Líbalová, Kateřina	Global gene expression changes in human	Particle and Fibre Toxicology	2012
PAHs	PIGN	Helena Líbalová, Kateřina	Global gene expression changes in human	Particle and Fibre Toxicology	2012
PAHs	PIGQ	Helena Líbalová, Kateřina	Global gene expression changes in human	Particle and Fibre Toxicology	2012
PAHs	PIGS	Helena Líbalová, Kateřina	Global gene expression changes in human	Particle and Fibre Toxicology	2012
PAHs	PIK3C2B	Helena Líbalová, Kateřina	Global gene expression changes in human	Particle and Fibre Toxicology	2012
PAHs	PIK3IP1	Helena Líbalová, Kateřina	Global gene expression changes in human	Particle and Fibre Toxicology	2012
PAHs	PIP5K2A	Helena Líbalová, Kateřina	Global gene expression changes in human	Particle and Fibre Toxicology	2012
PAHs	PKIA	Helena Líbalová, Kateřina	Global gene expression changes in human	Particle and Fibre Toxicology	2012
PAHs	PKM2	Helena Líbalová, Kateřina	Global gene expression changes in human	Particle and Fibre Toxicology	2012
PAHs	PKMYT1	Helena Líbalová, Kateřina	Global gene expression changes in human	Particle and Fibre Toxicology	2012
PAHs	PLA2G4C	Helena Líbalová, Kateřina	Global gene expression changes in human	Particle and Fibre Toxicology	2012
PAHs	PLAC9	Helena Líbalová, Kateřina	Global gene expression changes in human	Particle and Fibre Toxicology	2012
PAHs	PLCD1	Helena Líbalová, Kateřina	Global gene expression changes in human	Particle and Fibre Toxicology	2012
PAHs	PLCXD1	Helena Líbalová, Kateřina	Global gene expression changes in human	Particle and Fibre Toxicology	2012
PAHs	PLEKHA4	Helena Líbalová, Kateřina	Global gene expression changes in human	Particle and Fibre Toxicology	2012
PAHs	PLEKHA9	Helena Líbalová, Kateřina	Global gene expression changes in human	Particle and Fibre Toxicology	2012
PAHs	PLEKHH3	Helena Líbalová, Kateřina	Global gene expression changes in human	Particle and Fibre Toxicology	2012

PAHs	PLEKHO2	Helena Líbalová, Kateřina	Global gene expression changes in human	Particle and Fibre Toxicology	2012
PAHs	PLRG1	Helena Líbalová, Kateřina	Global gene expression changes in human	Particle and Fibre Toxicology	2012
PAHs	PNPLA6	Helena Líbalová, Kateřina	Global gene expression changes in human	Particle and Fibre Toxicology	2012
PAHs	PODXL2	Helena Líbalová, Kateřina	Global gene expression changes in human	Particle and Fibre Toxicology	2012
PAHs	POLA2	Helena Líbalová, Kateřina	Global gene expression changes in human	Particle and Fibre Toxicology	2012
PAHs	POLQ	Helena Líbalová, Kateřina	Global gene expression changes in human	Particle and Fibre Toxicology	2012
PAHs	POLR2A	Helena Líbalová, Kateřina	Global gene expression changes in human	Particle and Fibre Toxicology	2012
PAHs	POLR2H	Helena Líbalová, Kateřina	Global gene expression changes in human	Particle and Fibre Toxicology	2012
PAHs	POLR3C	Helena Líbalová, Kateřina	Global gene expression changes in human	Particle and Fibre Toxicology	2012
PAHs	POLRMT	Helena Líbalová, Kateřina	Global gene expression changes in human	Particle and Fibre Toxicology	2012
PAHs	POR	Helena Líbalová, Kateřina	Global gene expression changes in human	Particle and Fibre Toxicology	2012
PAHs	PPAN	Helena Líbalová, Kateřina	Global gene expression changes in human	Particle and Fibre Toxicology	2012
PAHs	PPAP2A	Helena Líbalová, Kateřina	Global gene expression changes in human	Particle and Fibre Toxicology	2012
PAHs	PPARD	Helena Líbalová, Kateřina	Global gene expression changes in human	Particle and Fibre Toxicology	2012
PAHs	PPAT	Helena Líbalová, Kateřina	Global gene expression changes in human	Particle and Fibre Toxicology	2012
PAHs	PPFIBP2	Helena Líbalová, Kateřina	Global gene expression changes in human	Particle and Fibre Toxicology	2012
PAHs	PPP1R12C	Helena Líbalová, Kateřina	Global gene expression changes in human	Particle and Fibre Toxicology	2012
PAHs	PPP1R14A	Helena Líbalová, Kateřina	Global gene expression changes in human	Particle and Fibre Toxicology	2012
PAHs	PPP1R3C	Helena Líbalová, Kateřina	Global gene expression changes in human	Particle and Fibre Toxicology	2012
PAHs	PRDM10	Helena Líbalová, Kateřina	Global gene expression changes in human	Particle and Fibre Toxicology	2012
PAHs	PRDX6	Helena Líbalová, Kateřina	Global gene expression changes in human	Particle and Fibre Toxicology	2012
PAHs	PRICKLE1	Helena Líbalová, Kateřina	Global gene expression changes in human	Particle and Fibre Toxicology	2012
PAHs	PRICKLE2	Helena Líbalová, Kateřina	Global gene expression changes in human	Particle and Fibre Toxicology	2012
PAHs	PRKD1	Helena Líbalová, Kateřina	Global gene expression changes in human	Particle and Fibre Toxicology	2012
PAHs	PROS1	Helena Líbalová, Kateřina	Global gene expression changes in human	Particle and Fibre Toxicology	2012
PAHs	PRPF38A	Helena Líbalová, Kateřina	Global gene expression changes in human	Particle and Fibre Toxicology	2012
PAHs	PRR14	Helena Líbalová, Kateřina	Global gene expression changes in human	Particle and Fibre Toxicology	2012
PAHs	PRR16	Helena Líbalová, Kateřina	Global gene expression changes in human	Particle and Fibre Toxicology	2012
PAHs	PRR6	Helena Líbalová, Kateřina	Global gene expression changes in human	Particle and Fibre Toxicology	2012
PAHs	PRR7	Helena Líbalová, Kateřina	Global gene expression changes in human	Particle and Fibre Toxicology	2012
PAHs	PRRG1	Helena Líbalová, Kateřina	Global gene expression changes in human	Particle and Fibre Toxicology	2012
PAHs	PRSS35	Helena Líbalová, Kateřina	Global gene expression changes in human	Particle and Fibre Toxicology	2012
PAHs	PSCD2	Helena Líbalová, Kateřina	Global gene expression changes in human	Particle and Fibre Toxicology	2012
PAHs	PSMD10	Helena Líbalová, Kateřina	Global gene expression changes in human	Particle and Fibre Toxicology	2012
PAHs	PTDSS2	Helena Líbalová, Kateřina	Global gene expression changes in human	Particle and Fibre Toxicology	2012
PAHs	PTGER2	Helena Líbalová, Kateřina	Global gene expression changes in human	Particle and Fibre Toxicology	2012
PAHs	PTMS	Helena Líbalová, Kateřina	Global gene expression changes in human	Particle and Fibre Toxicology	2012
PAHs	PTPLA	Helena Líbalová, Kateřina	Global gene expression changes in human	Particle and Fibre Toxicology	2012
PAHs	PTPN12	Helena Líbalová, Kateřina	Global gene expression changes in human	Particle and Fibre Toxicology	2012
PAHs	PTTG3	Helena Líbalová, Kateřina	Global gene expression changes in human	Particle and Fibre Toxicology	2012
PAHs	PVRL3	Helena Líbalová, Kateřina	Global gene expression changes in human	Particle and Fibre Toxicology	2012
PAHs	PYCR1	Helena Líbalová, Kateřina	Global gene expression changes in human	Particle and Fibre Toxicology	2012
PAHs	QDPR	Helena Líbalová, Kateřina	Global gene expression changes in human	Particle and Fibre Toxicology	2012
PAHs	QSOX1	Helena Líbalová, Kateřina	Global gene expression changes in human	Particle and Fibre Toxicology	2012
PAHs	RAB11A	Helena Líbalová, Kateřina	Global gene expression changes in human	Particle and Fibre Toxicology	2012
PAHs	RAB3IP	Helena Líbalová, Kateřina	Global gene expression changes in human	Particle and Fibre Toxicology	2012
PAHs	RAB7B	Helena Líbalová, Kateřina	Global gene expression changes in human	Particle and Fibre Toxicology	2012
PAHs	RAB7L1	Helena Líbalová, Kateřina	Global gene expression changes in human	Particle and Fibre Toxicology	2012
PAHs	RABGGTA	Helena Líbalová, Kateřina	Global gene expression changes in human	Particle and Fibre Toxicology	2012
PAHs	RABL4	Helena Líbalová, Kateřina	Global gene expression changes in human	Particle and Fibre Toxicology	2012
PAHs	RACGAP1	Helena Líbalová, Kateřina	Global gene expression changes in human	Particle and Fibre Toxicology	2012
PAHs	RAD51C	Helena Líbalová, Kateřina	Global gene expression changes in human	Particle and Fibre Toxicology	2012
PAHs	RAD54B	Helena Líbalová, Kateřina	Global gene expression changes in human	Particle and Fibre Toxicology	2012
PAHs	RARS2	Helena Líbalová, Kateřina	Global gene expression changes in human	Particle and Fibre Toxicology	2012
PAHs	RASA2	Helena Líbalová, Kateřina	Global gene expression changes in human	Particle and Fibre Toxicology	2012
PAHs	RASL11A	Helena Líbalová, Kateřina	Global gene expression changes in human	Particle and Fibre Toxicology	2012
PAHs	RASSF1	Helena Líbalová, Kateřina	Global gene expression changes in human	Particle and Fibre Toxicology	2012
PAHs	RBM25	Helena Líbalová, Kateřina	Global gene expression changes in human	Particle and Fibre Toxicology	2012
PAHs	RBM28	Helena Líbalová, Kateřina	Global gene expression changes in human	Particle and Fibre Toxicology	2012
PAHs	RBM4B	Helena Líbalová, Kateřina	Global gene expression changes in human	Particle and Fibre Toxicology	2012
PAHs	RBM5	Helena Líbalová, Kateřina	Global gene expression changes in human	Particle and Fibre Toxicology	2012
PAHs	RCAN1	Helena Líbalová, Kateřina	Global gene expression changes in human	Particle and Fibre Toxicology	2012
PAHs	RCAN2	Helena Líbalová, Kateřina	Global gene expression changes in human	Particle and Fibre Toxicology	2012
PAHs	RCOR2	Helena Líbalová, Kateřina	Global gene expression changes in human	Particle and Fibre Toxicology	2012
PAHs	REXO1	Helena Líbalová, Kateřina	Global gene expression changes in human	Particle and Fibre Toxicology	2012
PAHs	RFXAP	Helena Líbalová, Kateřina	Global gene expression changes in human	Particle and Fibre Toxicology	2012
PAHs	RGS17	Helena Líbalová, Kateřina	Global gene expression changes in human	Particle and Fibre Toxicology	2012
PAHs	RHOBTB3	Helena Líbalová, Kateřina	Global gene expression changes in human	Particle and Fibre Toxicology	2012
PAHs	RMND1	Helena Líbalová, Kateřina	Global gene expression changes in human	Particle and Fibre Toxicology	2012
PAHs	RNF112	Helena Líbalová, Kateřina	Global gene expression changes in human	Particle and Fibre Toxicology	2012
PAHs	RNF19B	Helena Líbalová, Kateřina	Global gene expression changes in human	Particle and Fibre Toxicology	2012
PAHs	RNF44	Helena Líbalová, Kateřina	Global gene expression changes in human	Particle and Fibre Toxicology	2012
PAHs	ROR1	Helena Líbalová, Kateřina	Global gene expression changes in human	Particle and Fibre Toxicology	2012
PAHs	RP5-1022P6.2	Helena Líbalová, Kateřina	Global gene expression changes in human	Particle and Fibre Toxicology	2012
PAHs	RPL22L1	Helena Líbalová, Kateřina	Global gene expression changes in human	Particle and Fibre Toxicology	2012
PAHs	RPP21	Helena Líbalová, Kateřina	Global gene expression changes in human	Particle and Fibre Toxicology	2012
PAHs	RPS6KA5	Helena Líbalová, Kateřina	Global gene expression changes in human	Particle and Fibre Toxicology	2012
PAHs	RPUSD3	Helena Líbalová, Kateřina	Global gene expression changes in human	Particle and Fibre Toxicology	2012
PAHs	RRAD	Helena Líbalová, Kateřina	Global gene expression changes in human	Particle and Fibre Toxicology	2012
PAHs	RRAS	Helena Líbalová, Kateřina	Global gene expression changes in human	Particle and Fibre Toxicology	2012
PAHs	RRP9	Helena Líbalová, Kateřina	Global gene expression changes in human	Particle and Fibre Toxicology	2012
PAHs	RUFY3	Helena Líbalová, Kateřina	Global gene expression changes in human	Particle and Fibre Toxicology	2012
PAHs	RUNX1T1	Helena Líbalová, Kateřina	Global gene expression changes in human	Particle and Fibre Toxicology	2012
PAHs	RWDD2A	Helena Líbalová, Kateřina	Global gene expression changes in human	Particle and Fibre Toxicology	2012
PAHs	S100A4	Helena Líbalová, Kateřina	Global gene expression changes in human	Particle and Fibre Toxicology	2012
PAHs	SAAL1	Helena Líbalová, Kateřina	Global gene expression changes in human	Particle and Fibre Toxicology	2012
PAHs	SAC3D1	Helena Líbalová, Kateřina	Global gene expression changes in human	Particle and Fibre Toxicology	2012
PAHs	SAP30L	Helena Líbalová, Kateřina	Global gene expression changes in human	Particle and Fibre Toxicology	2012
PAHs	SAR1A	Helena Líbalová, Kateřina	Global gene expression changes in human	Particle and Fibre Toxicology	2012

PAHs	SCAP	Helena Líbalová, Kateřina	Global gene expression changes in human	Particle and Fibre Toxicology	2012
PAHs	SCD	Helena Líbalová, Kateřina	Global gene expression changes in human	Particle and Fibre Toxicology	2012
PAHs	SCG2	Helena Líbalová, Kateřina	Global gene expression changes in human	Particle and Fibre Toxicology	2012
PAHs	SCHIP1	Helena Líbalová, Kateřina	Global gene expression changes in human	Particle and Fibre Toxicology	2012
PAHs	SCN2A	Helena Líbalová, Kateřina	Global gene expression changes in human	Particle and Fibre Toxicology	2012
PAHs	SCPEP1	Helena Líbalová, Kateřina	Global gene expression changes in human	Particle and Fibre Toxicology	2012
PAHs	SDF2L1	Helena Líbalová, Kateřina	Global gene expression changes in human	Particle and Fibre Toxicology	2012
PAHs	SEC24D	Helena Líbalová, Kateřina	Global gene expression changes in human	Particle and Fibre Toxicology	2012
PAHs	SEC63	Helena Líbalová, Kateřina	Global gene expression changes in human	Particle and Fibre Toxicology	2012
PAHs	SEPHS1	Helena Líbalová, Kateřina	Global gene expression changes in human	Particle and Fibre Toxicology	2012
PAHs	SEPSECS	Helena Líbalová, Kateřina	Global gene expression changes in human	Particle and Fibre Toxicology	2012
PAHs	SERPINH1	Helena Líbalová, Kateřina	Global gene expression changes in human	Particle and Fibre Toxicology	2012
PAHs	SETDB1	Helena Líbalová, Kateřina	Global gene expression changes in human	Particle and Fibre Toxicology	2012
PAHs	SGOL2	Helena Líbalová, Kateřina	Global gene expression changes in human	Particle and Fibre Toxicology	2012
PAHs	SH2B3	Helena Líbalová, Kateřina	Global gene expression changes in human	Particle and Fibre Toxicology	2012
PAHs	SH3YL1	Helena Líbalová, Kateřina	Global gene expression changes in human	Particle and Fibre Toxicology	2012
PAHs	SHC2	Helena Líbalová, Kateřina	Global gene expression changes in human	Particle and Fibre Toxicology	2012
PAHs	SIRPA	Helena Líbalová, Kateřina	Global gene expression changes in human	Particle and Fibre Toxicology	2012
PAHs	SIRT5	Helena Líbalová, Kateřina	Global gene expression changes in human	Particle and Fibre Toxicology	2012
PAHs	SLBP	Helena Líbalová, Kateřina	Global gene expression changes in human	Particle and Fibre Toxicology	2012
PAHs	SLC15A4	Helena Líbalová, Kateřina	Global gene expression changes in human	Particle and Fibre Toxicology	2012
PAHs	SLC25A15	Helena Líbalová, Kateřina	Global gene expression changes in human	Particle and Fibre Toxicology	2012
PAHs	SLC25A23	Helena Líbalová, Kateřina	Global gene expression changes in human	Particle and Fibre Toxicology	2012
PAHs	SLC25A26	Helena Líbalová, Kateřina	Global gene expression changes in human	Particle and Fibre Toxicology	2012
PAHs	SLC25A43	Helena Líbalová, Kateřina	Global gene expression changes in human	Particle and Fibre Toxicology	2012
PAHs	SLC27A1	Helena Líbalová, Kateřina	Global gene expression changes in human	Particle and Fibre Toxicology	2012
PAHs	SLC27A3	Helena Líbalová, Kateřina	Global gene expression changes in human	Particle and Fibre Toxicology	2012
PAHs	SLC29A3	Helena Líbalová, Kateřina	Global gene expression changes in human	Particle and Fibre Toxicology	2012
PAHs	SLC2A12	Helena Líbalová, Kateřina	Global gene expression changes in human	Particle and Fibre Toxicology	2012
PAHs	SLC37A3	Helena Líbalová, Kateřina	Global gene expression changes in human	Particle and Fibre Toxicology	2012
PAHs	SLC38A7	Helena Líbalová, Kateřina	Global gene expression changes in human	Particle and Fibre Toxicology	2012
PAHs	SLC39A3	Helena Líbalová, Kateřina	Global gene expression changes in human	Particle and Fibre Toxicology	2012
PAHs	SLC43A3	Helena Líbalová, Kateřina	Global gene expression changes in human	Particle and Fibre Toxicology	2012
PAHs	SLC46A3	Helena Líbalová, Kateřina	Global gene expression changes in human	Particle and Fibre Toxicology	2012
PAHs	SLC7A7	Helena Líbalová, Kateřina	Global gene expression changes in human	Particle and Fibre Toxicology	2012
PAHs	SLFN12	Helena Líbalová, Kateřina	Global gene expression changes in human	Particle and Fibre Toxicology	2012
PAHs	SLTM	Helena Líbalová, Kateřina	Global gene expression changes in human	Particle and Fibre Toxicology	2012
PAHs	SMARCD2	Helena Líbalová, Kateřina	Global gene expression changes in human	Particle and Fibre Toxicology	2012
PAHs	SMC6	Helena Líbalová, Kateřina	Global gene expression changes in human	Particle and Fibre Toxicology	2012
PAHs	SNIP1	Helena Líbalová, Kateřina	Global gene expression changes in human	Particle and Fibre Toxicology	2012
PAHs	SNPH	Helena Líbalová, Kateřina	Global gene expression changes in human	Particle and Fibre Toxicology	2012
PAHs	SNRPD1	Helena Líbalová, Kateřina	Global gene expression changes in human	Particle and Fibre Toxicology	2012
PAHs	SNRPN	Helena Líbalová, Kateřina	Global gene expression changes in human	Particle and Fibre Toxicology	2012
PAHs	SNX1	Helena Líbalová, Kateřina	Global gene expression changes in human	Particle and Fibre Toxicology	2012
PAHs	SNX11	Helena Líbalová, Kateřina	Global gene expression changes in human	Particle and Fibre Toxicology	2012
PAHs	SNX12	Helena Líbalová, Kateřina	Global gene expression changes in human	Particle and Fibre Toxicology	2012
PAHs	SNX16	Helena Líbalová, Kateřina	Global gene expression changes in human	Particle and Fibre Toxicology	2012
PAHs	SNX25	Helena Líbalová, Kateřina	Global gene expression changes in human	Particle and Fibre Toxicology	2012
PAHs	SNX26	Helena Líbalová, Kateřina	Global gene expression changes in human	Particle and Fibre Toxicology	2012
PAHs	SNX29	Helena Líbalová, Kateřina	Global gene expression changes in human	Particle and Fibre Toxicology	2012
PAHs	SNX4	Helena Líbalová, Kateřina	Global gene expression changes in human	Particle and Fibre Toxicology	2012
PAHs	SNX7	Helena Líbalová, Kateřina	Global gene expression changes in human	Particle and Fibre Toxicology	2012
PAHs	SOCS1	Helena Líbalová, Kateřina	Global gene expression changes in human	Particle and Fibre Toxicology	2012
PAHs	SOX8	Helena Líbalová, Kateřina	Global gene expression changes in human	Particle and Fibre Toxicology	2012
PAHs	SPATA2	Helena Líbalová, Kateřina	Global gene expression changes in human	Particle and Fibre Toxicology	2012
PAHs	SPIN4	Helena Líbalová, Kateřina	Global gene expression changes in human	Particle and Fibre Toxicology	2012
PAHs	SPIRE2	Helena Líbalová, Kateřina	Global gene expression changes in human	Particle and Fibre Toxicology	2012
PAHs	SPSB3	Helena Líbalová, Kateřina	Global gene expression changes in human	Particle and Fibre Toxicology	2012
PAHs	SPTLC2	Helena Líbalová, Kateřina	Global gene expression changes in human	Particle and Fibre Toxicology	2012
PAHs	SRC	Helena Líbalová, Kateřina	Global gene expression changes in human	Particle and Fibre Toxicology	2012
PAHs	SREBF1	Helena Líbalová, Kateřina	Global gene expression changes in human	Particle and Fibre Toxicology	2012
PAHs	SS18	Helena Líbalová, Kateřina	Global gene expression changes in human	Particle and Fibre Toxicology	2012
PAHs	SSBP2	Helena Líbalová, Kateřina	Global gene expression changes in human	Particle and Fibre Toxicology	2012
PAHs	SSR2	Helena Líbalová, Kateřina	Global gene expression changes in human	Particle and Fibre Toxicology	2012
PAHs	ST3GAL4	Helena Líbalová, Kateřina	Global gene expression changes in human	Particle and Fibre Toxicology	2012
PAHs	ST5	Helena Líbalová, Kateřina	Global gene expression changes in human	Particle and Fibre Toxicology	2012
PAHs	ST7	Helena Líbalová, Kateřina	Global gene expression changes in human	Particle and Fibre Toxicology	2012
PAHs	STAMBPL1	Helena Líbalová, Kateřina	Global gene expression changes in human	Particle and Fibre Toxicology	2012
PAHs	STARD7	Helena Líbalová, Kateřina	Global gene expression changes in human	Particle and Fibre Toxicology	2012
PAHs	STK17B	Helena Líbalová, Kateřina	Global gene expression changes in human	Particle and Fibre Toxicology	2012
PAHs	STK38	Helena Líbalová, Kateřina	Global gene expression changes in human	Particle and Fibre Toxicology	2012
PAHs	STX12	Helena Líbalová, Kateřina	Global gene expression changes in human	Particle and Fibre Toxicology	2012
PAHs	STX7	Helena Líbalová, Kateřina	Global gene expression changes in human	Particle and Fibre Toxicology	2012
PAHs	STXBP5	Helena Líbalová, Kateřina	Global gene expression changes in human	Particle and Fibre Toxicology	2012
PAHs	SUGT1	Helena Líbalová, Kateřina	Global gene expression changes in human	Particle and Fibre Toxicology	2012
PAHs	SURF4	Helena Líbalová, Kateřina	Global gene expression changes in human	Particle and Fibre Toxicology	2012
PAHs	SUSD2	Helena Líbalová, Kateřina	Global gene expression changes in human	Particle and Fibre Toxicology	2012
PAHs	SVEP1	Helena Líbalová, Kateřina	Global gene expression changes in human	Particle and Fibre Toxicology	2012
PAHs	SYPL1	Helena Líbalová, Kateřina	Global gene expression changes in human	Particle and Fibre Toxicology	2012
PAHs	TANK	Helena Líbalová, Kateřina	Global gene expression changes in human	Particle and Fibre Toxicology	2012
PAHs	TAX1BP1	Helena Líbalová, Kateřina	Global gene expression changes in human	Particle and Fibre Toxicology	2012
PAHs	TBC1D2	Helena Líbalová, Kateřina	Global gene expression changes in human	Particle and Fibre Toxicology	2012
PAHs	TBC1D3B	Helena Líbalová, Kateřina	Global gene expression changes in human	Particle and Fibre Toxicology	2012
PAHs	TCN2	Helena Líbalová, Kateřina	Global gene expression changes in human	Particle and Fibre Toxicology	2012
PAHs	TEK	Helena Líbalová, Kateřina	Global gene expression changes in human	Particle and Fibre Toxicology	2012
PAHs	TENC1	Helena Líbalová, Kateřina	Global gene expression changes in human	Particle and Fibre Toxicology	2012
PAHs	TGFBR2	Helena Líbalová, Kateřina	Global gene expression changes in human	Particle and Fibre Toxicology	2012
PAHs	THAP11	Helena Líbalová, Kateřina	Global gene expression changes in human	Particle and Fibre Toxicology	2012
PAHs	THBS2	Helena Líbalová, Kateřina	Global gene expression changes in human	Particle and Fibre Toxicology	2012

PAHs	THOC6	Helena Líbalová, Kateřina	Global gene expression changes in human	Particle and Fibre Toxicology	2012
PAHs	THY1	Helena Líbalová, Kateřina	Global gene expression changes in human	Particle and Fibre Toxicology	2012
PAHs	TIMM22	Helena Líbalová, Kateřina	Global gene expression changes in human	Particle and Fibre Toxicology	2012
PAHs	TLCD1	Helena Líbalová, Kateřina	Global gene expression changes in human	Particle and Fibre Toxicology	2012
PAHs	TLE4	Helena Líbalová, Kateřina	Global gene expression changes in human	Particle and Fibre Toxicology	2012
PAHs	TMED1	Helena Líbalová, Kateřina	Global gene expression changes in human	Particle and Fibre Toxicology	2012
PAHs	TMEM16D	Helena Líbalová, Kateřina	Global gene expression changes in human	Particle and Fibre Toxicology	2012
PAHs	TMEM22	Helena Líbalová, Kateřina	Global gene expression changes in human	Particle and Fibre Toxicology	2012
PAHs	TMEM26	Helena Líbalová, Kateřina	Global gene expression changes in human	Particle and Fibre Toxicology	2012
PAHs	TMEM50A	Helena Líbalová, Kateřina	Global gene expression changes in human	Particle and Fibre Toxicology	2012
PAHs	TMEM51	Helena Líbalová, Kateřina	Global gene expression changes in human	Particle and Fibre Toxicology	2012
PAHs	TMTC1	Helena Líbalová, Kateřina	Global gene expression changes in human	Particle and Fibre Toxicology	2012
PAHs	TNFAIP1	Helena Líbalová, Kateřina	Global gene expression changes in human	Particle and Fibre Toxicology	2012
PAHs	TNFRSF10B	Helena Líbalová, Kateřina	Global gene expression changes in human	Particle and Fibre Toxicology	2012
PAHs	TNRC6A	Helena Líbalová, Kateřina	Global gene expression changes in human	Particle and Fibre Toxicology	2012
PAHs	TNS3	Helena Líbalová, Kateřina	Global gene expression changes in human	Particle and Fibre Toxicology	2012
PAHs	TOMM70A	Helena Líbalová, Kateřina	Global gene expression changes in human	Particle and Fibre Toxicology	2012
PAHs	TOX	Helena Líbalová, Kateřina	Global gene expression changes in human	Particle and Fibre Toxicology	2012
PAHs	TPM2	Helena Líbalová, Kateřina	Global gene expression changes in human	Particle and Fibre Toxicology	2012
PAHs	TPX2	Helena Líbalová, Kateřina	Global gene expression changes in human	Particle and Fibre Toxicology	2012
PAHs	TRA2A	Helena Líbalová, Kateřina	Global gene expression changes in human	Particle and Fibre Toxicology	2012
PAHs	TRAFD1	Helena Líbalová, Kateřina	Global gene expression changes in human	Particle and Fibre Toxicology	2012
PAHs	TRAM2	Helena Líbalová, Kateřina	Global gene expression changes in human	Particle and Fibre Toxicology	2012
PAHs	TRIM11	Helena Líbalová, Kateřina	Global gene expression changes in human	Particle and Fibre Toxicology	2012
PAHs	TRIM24	Helena Líbalová, Kateřina	Global gene expression changes in human	Particle and Fibre Toxicology	2012
PAHs	TRIM27	Helena Líbalová, Kateřina	Global gene expression changes in human	Particle and Fibre Toxicology	2012
PAHs	TRIM32	Helena Líbalová, Kateřina	Global gene expression changes in human	Particle and Fibre Toxicology	2012
PAHs	TRIM33	Helena Líbalová, Kateřina	Global gene expression changes in human	Particle and Fibre Toxicology	2012
PAHs	TRIM39	Helena Líbalová, Kateřina	Global gene expression changes in human	Particle and Fibre Toxicology	2012
PAHs	TRIM44	Helena Líbalová, Kateřina	Global gene expression changes in human	Particle and Fibre Toxicology	2012
PAHs	TRIM47	Helena Líbalová, Kateřina	Global gene expression changes in human	Particle and Fibre Toxicology	2012
PAHs	TRIM5	Helena Líbalová, Kateřina	Global gene expression changes in human	Particle and Fibre Toxicology	2012
PAHs	TRIP4	Helena Líbalová, Kateřina	Global gene expression changes in human	Particle and Fibre Toxicology	2012
PAHs	TRMT1	Helena Líbalová, Kateřina	Global gene expression changes in human	Particle and Fibre Toxicology	2012
PAHs	TRMT11	Helena Líbalová, Kateřina	Global gene expression changes in human	Particle and Fibre Toxicology	2012
PAHs	TROAP	Helena Líbalová, Kateřina	Global gene expression changes in human	Particle and Fibre Toxicology	2012
PAHs	TROVE2	Helena Líbalová, Kateřina	Global gene expression changes in human	Particle and Fibre Toxicology	2012
PAHs	TRPM4	Helena Líbalová, Kateřina	Global gene expression changes in human	Particle and Fibre Toxicology	2012
PAHs	TRUB2	Helena Líbalová, Kateřina	Global gene expression changes in human	Particle and Fibre Toxicology	2012
PAHs	TSC22D3	Helena Líbalová, Kateřina	Global gene expression changes in human	Particle and Fibre Toxicology	2012
PAHs	TSGA14	Helena Líbalová, Kateřina	Global gene expression changes in human	Particle and Fibre Toxicology	2012
PAHs	TSLP	Helena Líbalová, Kateřina	Global gene expression changes in human	Particle and Fibre Toxicology	2012
PAHs	TSPYL1	Helena Líbalová, Kateřina	Global gene expression changes in human	Particle and Fibre Toxicology	2012
PAHs	TTC17	Helena Líbalová, Kateřina	Global gene expression changes in human	Particle and Fibre Toxicology	2012
PAHs	UBE2A	Helena Líbalová, Kateřina	Global gene expression changes in human	Particle and Fibre Toxicology	2012
PAHs	UBE2D3	Helena Líbalová, Kateřina	Global gene expression changes in human	Particle and Fibre Toxicology	2012
PAHs	UBE2G1	Helena Líbalová, Kateřina	Global gene expression changes in human	Particle and Fibre Toxicology	2012
PAHs	UBE2Q1	Helena Líbalová, Kateřina	Global gene expression changes in human	Particle and Fibre Toxicology	2012
PAHs	UBN1	Helena Líbalová, Kateřina	Global gene expression changes in human	Particle and Fibre Toxicology	2012
PAHs	UBQLN4	Helena Líbalová, Kateřina	Global gene expression changes in human	Particle and Fibre Toxicology	2012
PAHs	UCHL3	Helena Líbalová, Kateřina	Global gene expression changes in human	Particle and Fibre Toxicology	2012
PAHs	UCHL5IP	Helena Líbalová, Kateřina	Global gene expression changes in human	Particle and Fibre Toxicology	2012
PAHs	UCK1	Helena Líbalová, Kateřina	Global gene expression changes in human	Particle and Fibre Toxicology	2012
PAHs	UHRF1BP1L	Helena Líbalová, Kateřina	Global gene expression changes in human	Particle and Fibre Toxicology	2012
PAHs	ULK1	Helena Líbalová, Kateřina	Global gene expression changes in human	Particle and Fibre Toxicology	2012
PAHs	ULK3	Helena Líbalová, Kateřina	Global gene expression changes in human	Particle and Fibre Toxicology	2012
PAHs	UQCR	Helena Líbalová, Kateřina	Global gene expression changes in human	Particle and Fibre Toxicology	2012
PAHs	UQCRC1	Helena Líbalová, Kateřina	Global gene expression changes in human	Particle and Fibre Toxicology	2012
PAHs	USE1	Helena Líbalová, Kateřina	Global gene expression changes in human	Particle and Fibre Toxicology	2012
PAHs	USF1	Helena Líbalová, Kateřina	Global gene expression changes in human	Particle and Fibre Toxicology	2012
PAHs	USP47	Helena Líbalová, Kateřina	Global gene expression changes in human	Particle and Fibre Toxicology	2012
PAHs	VAMP4	Helena Líbalová, Kateřina	Global gene expression changes in human	Particle and Fibre Toxicology	2012
PAHs	VAMP5	Helena Líbalová, Kateřina	Global gene expression changes in human	Particle and Fibre Toxicology	2012
PAHs	VBP1	Helena Líbalová, Kateřina	Global gene expression changes in human	Particle and Fibre Toxicology	2012
PAHs	VCAM1	Helena Líbalová, Kateřina	Global gene expression changes in human	Particle and Fibre Toxicology	2012
PAHs	VCL	Helena Líbalová, Kateřina	Global gene expression changes in human	Particle and Fibre Toxicology	2012
PAHs	VGLL3	Helena Líbalová, Kateřina	Global gene expression changes in human	Particle and Fibre Toxicology	2012
PAHs	VPS18	Helena Líbalová, Kateřina	Global gene expression changes in human	Particle and Fibre Toxicology	2012
PAHs	VPS26B	Helena Líbalová, Kateřina	Global gene expression changes in human	Particle and Fibre Toxicology	2012
PAHs	VPS37D	Helena Líbalová, Kateřina	Global gene expression changes in human	Particle and Fibre Toxicology	2012
PAHs	WARS2	Helena Líbalová, Kateřina	Global gene expression changes in human	Particle and Fibre Toxicology	2012
PAHs	WBP11	Helena Líbalová, Kateřina	Global gene expression changes in human	Particle and Fibre Toxicology	2012
PAHs	WBSCR18	Helena Líbalová, Kateřina	Global gene expression changes in human	Particle and Fibre Toxicology	2012
PAHs	WDR23	Helena Líbalová, Kateřina	Global gene expression changes in human	Particle and Fibre Toxicology	2012
PAHs	WDR27	Helena Líbalová, Kateřina	Global gene expression changes in human	Particle and Fibre Toxicology	2012
PAHs	WDR44	Helena Líbalová, Kateřina	Global gene expression changes in human	Particle and Fibre Toxicology	2012
PAHs	WDR68	Helena Líbalová, Kateřina	Global gene expression changes in human	Particle and Fibre Toxicology	2012
PAHs	WDR74	Helena Líbalová, Kateřina	Global gene expression changes in human	Particle and Fibre Toxicology	2012
PAHs	WDR90	Helena Líbalová, Kateřina	Global gene expression changes in human	Particle and Fibre Toxicology	2012
PAHs	WDR91	Helena Líbalová, Kateřina	Global gene expression changes in human	Particle and Fibre Toxicology	2012
PAHs	WNK4	Helena Líbalová, Kateřina	Global gene expression changes in human	Particle and Fibre Toxicology	2012
PAHs	WRB	Helena Líbalová, Kateřina	Global gene expression changes in human	Particle and Fibre Toxicology	2012
PAHs	WRN	Helena Líbalová, Kateřina	Global gene expression changes in human	Particle and Fibre Toxicology	2012
PAHs	YRDC	Helena Líbalová, Kateřina	Global gene expression changes in human	Particle and Fibre Toxicology	2012
PAHs	YWHAH	Helena Líbalová, Kateřina	Global gene expression changes in human	Particle and Fibre Toxicology	2012
PAHs	YY1	Helena Líbalová, Kateřina	Global gene expression changes in human	Particle and Fibre Toxicology	2012
PAHs	ZAK	Helena Líbalová, Kateřina	Global gene expression changes in human	Particle and Fibre Toxicology	2012
PAHs	ZBTB46	Helena Líbalová, Kateřina	Global gene expression changes in human	Particle and Fibre Toxicology	2012
PAHs	ZC3H5	Helena Líbalová, Kateřina	Global gene expression changes in human	Particle and Fibre Toxicology	2012

PAHs	ZC3HAV1	Helena Líbalová, Kateřina	Global gene expression changes in human	Particle and Fibre Toxicology	2012
PAHs	ZCCHC24	Helena Líbalová, Kateřina	Global gene expression changes in human	Particle and Fibre Toxicology	2012
PAHs	ZDHHC16	Helena Líbalová, Kateřina	Global gene expression changes in human	Particle and Fibre Toxicology	2012
PAHs	ZFAND2A	Helena Líbalová, Kateřina	Global gene expression changes in human	Particle and Fibre Toxicology	2012
PAHs	ZFPM1	Helena Líbalová, Kateřina	Global gene expression changes in human	Particle and Fibre Toxicology	2012
PAHs	ZFYVE19	Helena Líbalová, Kateřina	Global gene expression changes in human	Particle and Fibre Toxicology	2012
PAHs	ZKSCAN3	Helena Líbalová, Kateřina	Global gene expression changes in human	Particle and Fibre Toxicology	2012
PAHs	ZMYND19	Helena Líbalová, Kateřina	Global gene expression changes in human	Particle and Fibre Toxicology	2012
PAHs	ZNF167	Helena Líbalová, Kateřina	Global gene expression changes in human	Particle and Fibre Toxicology	2012
PAHs	ZNF219	Helena Líbalová, Kateřina	Global gene expression changes in human	Particle and Fibre Toxicology	2012
PAHs	ZNF239	Helena Líbalová, Kateřina	Global gene expression changes in human	Particle and Fibre Toxicology	2012
PAHs	ZNF259	Helena Líbalová, Kateřina	Global gene expression changes in human	Particle and Fibre Toxicology	2012
PAHs	ZNF271	Helena Líbalová, Kateřina	Global gene expression changes in human	Particle and Fibre Toxicology	2012
PAHs	ZNF30	Helena Líbalová, Kateřina	Global gene expression changes in human	Particle and Fibre Toxicology	2012
PAHs	ZNF333	Helena Líbalová, Kateřina	Global gene expression changes in human	Particle and Fibre Toxicology	2012
PAHs	ZNF358	Helena Líbalová, Kateřina	Global gene expression changes in human	Particle and Fibre Toxicology	2012
PAHs	ZNF408	Helena Líbalová, Kateřina	Global gene expression changes in human	Particle and Fibre Toxicology	2012
PAHs	ZNF433	Helena Líbalová, Kateřina	Global gene expression changes in human	Particle and Fibre Toxicology	2012
PAHs	ZNF436	Helena Líbalová, Kateřina	Global gene expression changes in human	Particle and Fibre Toxicology	2012
PAHs	ZNF439	Helena Líbalová, Kateřina	Global gene expression changes in human	Particle and Fibre Toxicology	2012
PAHs	ZNF519	Helena Líbalová, Kateřina	Global gene expression changes in human	Particle and Fibre Toxicology	2012
PAHs	ZNF544	Helena Líbalová, Kateřina	Global gene expression changes in human	Particle and Fibre Toxicology	2012
PAHs	ZNF586	Helena Líbalová, Kateřina	Global gene expression changes in human	Particle and Fibre Toxicology	2012
PAHs	ZNF594	Helena Líbalová, Kateřina	Global gene expression changes in human	Particle and Fibre Toxicology	2012
PAHs	ZNF653	Helena Líbalová, Kateřina	Global gene expression changes in human	Particle and Fibre Toxicology	2012
PAHs	ZNF671	Helena Líbalová, Kateřina	Global gene expression changes in human	Particle and Fibre Toxicology	2012
PAHs	ZNF702P	Helena Líbalová, Kateřina	Global gene expression changes in human	Particle and Fibre Toxicology	2012
PAHs	ZNF787	Helena Líbalová, Kateřina	Global gene expression changes in human	Particle and Fibre Toxicology	2012
PAHs	ZSCAN2	Helena Líbalová, Kateřina	Global gene expression changes in human	Particle and Fibre Toxicology	2012
PAHs	ZUFSP	Helena Líbalová, Kateřina	Global gene expression changes in human	Particle and Fibre Toxicology	2012
PAHs	CYP1A1	K. Skupinska et al	A comparison of the concentration–effect	Archives of Toxicology	2007
PAHs	CYP1A2	K. Skupinska et al	A comparison of the concentration–effect	Archives of Toxicology	2007
PAHs	CYP1A1	Song MK, Kim YJ, Song M,	Formation of a 3,4-diol-1,2-epoxide	Environmental Toxicology and	2012
PAHs	CYP1B1	Song MK, Kim YJ, Song M,	Formation of a 3,4-diol-1,2-epoxide	Environmental Toxicology and	2012
PAHs	AKR1C1	Song MK, Kim YJ, Song M,	Formation of a 3,4-diol-1,2-epoxide	Environmental Toxicology and	2012
PAHs	AKR1C3	Song MK, Kim YJ, Song M,	Formation of a 3,4-diol-1,2-epoxide	Environmental Toxicology and	2012
PAHs	AKR1B10	Song MK, Kim YJ, Song M,	Formation of a 3,4-diol-1,2-epoxide	Environmental Toxicology and	2012
PAHs	GADD45A	Song MK, Kim YJ, Song M,	Formation of a 3,4-diol-1,2-epoxide	Environmental Toxicology and	2012
PAHs	CDKN1A	Song MK, Kim YJ, Song M,	Formation of a 3,4-diol-1,2-epoxide	Environmental Toxicology and	2012
PAHs	BTG2	Song MK, Kim YJ, Song M,	Formation of a 3,4-diol-1,2-epoxide	Environmental Toxicology and	2012
PAHs	ERCC1	Song MK, Kim YJ, Song M,	Formation of a 3,4-diol-1,2-epoxide	Environmental Toxicology and	2012
PAHs	POLH	Song MK, Kim YJ, Song M,	Formation of a 3,4-diol-1,2-epoxide	Environmental Toxicology and	2012
PAHs	CYP1A1	K. Skupinska et al	A comparison of the concentration–effect	Archives of Toxicology	2007
PAHs	CYP1A2	K. Skupinska et al	A comparison of the concentration–effect	Archives of Toxicology	2007
PAHs	CYP1A2	Kentaro Misaki, Saburo	Metabolic Enzyme Induction by HepG2	Chemical Research in Toxicology	2007
PAHs	CYP1B1	Kentaro Misaki, Saburo	Metabolic Enzyme Induction by HepG2	Chemical Research in Toxicology	2007
PAHs	AKR1C1	Kentaro Misaki, Saburo	Metabolic Enzyme Induction by HepG2	Chemical Research in Toxicology	2007
PAHs	NQO1	Kentaro Misaki, Saburo	Metabolic Enzyme Induction by HepG2	Chemical Research in Toxicology	2007
PAHs	GSTM1	Kentaro Misaki, Saburo	Metabolic Enzyme Induction by HepG2	Chemical Research in Toxicology	2007
PAHs	CYP1A1	K. Skupinska et al	A comparison of the concentration–effect	Archives of Toxicology	2007
PAHs	CYP1A2	K. Skupinska et al	A comparison of the concentration–effect	Archives of Toxicology	2007
PAHs	CYP1A1	Kentaro Misaki, Saburo	Metabolic Enzyme Induction by HepG2	Chemical Research in Toxicology	2007
PAHs	CYP1A2	Kentaro Misaki, Saburo	Metabolic Enzyme Induction by HepG2	Chemical Research in Toxicology	2007
PAHs	CYP1B1	Kentaro Misaki, Saburo	Metabolic Enzyme Induction by HepG2	Chemical Research in Toxicology	2007
PAHs	AKR1C1	Kentaro Misaki, Saburo	Metabolic Enzyme Induction by HepG2	Chemical Research in Toxicology	2007
PAHs	NQO1	Kentaro Misaki, Saburo	Metabolic Enzyme Induction by HepG2	Chemical Research in Toxicology	2007
PAHs	GSTM1	Kentaro Misaki, Saburo	Metabolic Enzyme Induction by HepG2	Chemical Research in Toxicology	2007
PAHs	FUS	Diodovich C, Malerba I,	Naphthalene Exposure: Effects on Gene	Journal of Biochemical and Molecular	2003
PAHs	TLS	Diodovich C, Malerba I,	Naphthalene Exposure: Effects on Gene	Journal of Biochemical and Molecular	2003
PAHs	TCF-1	Diodovich C, Malerba I,	Naphthalene Exposure: Effects on Gene	Journal of Biochemical and Molecular	2003
PAHs	IL-8	Diodovich C, Malerba I,	Naphthalene Exposure: Effects on Gene	Journal of Biochemical and Molecular	2003
PAHs	BCL-2	Diodovich C, Malerba I,	Naphthalene Exposure: Effects on Gene	Journal of Biochemical and Molecular	2003
PAHs	BAX	Diodovich C, Malerba I,	Naphthalene Exposure: Effects on Gene	Journal of Biochemical and Molecular	2003
PAHs	RAF-1	Diodovich C, Malerba I,	Naphthalene Exposure: Effects on Gene	Journal of Biochemical and Molecular	2003
PAHs	c-JUN	Diodovich C, Malerba I,	Naphthalene Exposure: Effects on Gene	Journal of Biochemical and Molecular	2003
PAHs	c-FOS	Diodovich C, Malerba I,	Naphthalene Exposure: Effects on Gene	Journal of Biochemical and Molecular	2003
PAHs	CYP1A1	Juei-Chuan C. Kang-Sickel,	Exposure to naphthalene induces naphthyl-	Biomarkers	2010
PAHs	CYP2C19	Juei-Chuan C. Kang-Sickel,	Exposure to naphthalene induces naphthyl-	Biomarkers	2010
PAHs	CYP2C8	Juei-Chuan C. Kang-Sickel,	Exposure to naphthalene induces naphthyl-	Biomarkers	2010
PAHs	CYP2C9	Juei-Chuan C. Kang-Sickel,	Exposure to naphthalene induces naphthyl-	Biomarkers	2010
PAHs	CYP2D6	Juei-Chuan C. Kang-Sickel,	Exposure to naphthalene induces naphthyl-	Biomarkers	2010
PAHs	CYP2E1	Juei-Chuan C. Kang-Sickel,	Exposure to naphthalene induces naphthyl-	Biomarkers	2010
PAHs	CYP3A4	Juei-Chuan C. Kang-Sickel,	Exposure to naphthalene induces naphthyl-	Biomarkers	2010
PAHs	CYP3A5	Juei-Chuan C. Kang-Sickel,	Exposure to naphthalene induces naphthyl-	Biomarkers	2010
PAHs	EPHX1	Juei-Chuan C. Kang-Sickel,	Exposure to naphthalene induces naphthyl-	Biomarkers	2010
PAHs	EPHX2	Juei-Chuan C. Kang-Sickel,	Exposure to naphthalene induces naphthyl-	Biomarkers	2010
PAHs	GSTM1	Juei-Chuan C. Kang-Sickel,	Exposure to naphthalene induces naphthyl-	Biomarkers	2010
PAHs	CYP1A1	Fabiola Castorena-Torres,	Changes in gene expression induced by	Toxicology in Vitro	2008
PAHs	CYP1B1	Fabiola Castorena-Torres,	Changes in gene expression induced by	Toxicology in Vitro	2008
PAHs	ERCC5	Fabiola Castorena-Torres,	Changes in gene expression induced by	Toxicology in Vitro	2008
PAHs	FDFT1	Fabiola Castorena-Torres,	Changes in gene expression induced by	Toxicology in Vitro	2008
PAHs	FTH1	Fabiola Castorena-Torres,	Changes in gene expression induced by	Toxicology in Vitro	2008
PAHs	FXYD3	Fabiola Castorena-Torres,	Changes in gene expression induced by	Toxicology in Vitro	2008
PAHs	PRDX1	Fabiola Castorena-Torres,	Changes in gene expression induced by	Toxicology in Vitro	2008
PAHs	PSMD7	Fabiola Castorena-Torres,	Changes in gene expression induced by	Toxicology in Vitro	2008
PAHs	PTGER3	Fabiola Castorena-Torres,	Changes in gene expression induced by	Toxicology in Vitro	2008
PAHs	CCNA2	Yvonne C.M. Staal, et al.	Modulation of gene expression and DNA	Carcinogenesis	2006
PAHs	CYB5	Yvonne C.M. Staal, et al.	Modulation of gene expression and DNA	Carcinogenesis	2006
PAHs	CYP1A1	Yvonne C.M. Staal, et al.	Modulation of gene expression and DNA	Carcinogenesis	2006

PAHs	CYP1A2	Yvonne C.M. Staal, et al.	Modulation of gene expression and DNA	Carcinogenesis	2006
PAHs	CYP2D6	Yvonne C.M. Staal, et al.	Modulation of gene expression and DNA	Carcinogenesis	2006
PAHs	CYP2E1	Yvonne C.M. Staal, et al.	Modulation of gene expression and DNA	Carcinogenesis	2006
PAHs	DBI	Yvonne C.M. Staal, et al.	Modulation of gene expression and DNA	Carcinogenesis	2006
PAHs	HAMP	Yvonne C.M. Staal, et al.	Modulation of gene expression and DNA	Carcinogenesis	2006
PAHs	HDAC1	Yvonne C.M. Staal, et al.	Modulation of gene expression and DNA	Carcinogenesis	2006
PAHs	HIST1H2AL	Yvonne C.M. Staal, et al.	Modulation of gene expression and DNA	Carcinogenesis	2006
PAHs	HMGCR	Yvonne C.M. Staal, et al.	Modulation of gene expression and DNA	Carcinogenesis	2006
PAHs	HMGCS1	Yvonne C.M. Staal, et al.	Modulation of gene expression and DNA	Carcinogenesis	2006
PAHs	IGF1	Yvonne C.M. Staal, et al.	Modulation of gene expression and DNA	Carcinogenesis	2006
PAHs	IGFBP1	Yvonne C.M. Staal, et al.	Modulation of gene expression and DNA	Carcinogenesis	2006
PAHs	LGALS3	Yvonne C.M. Staal, et al.	Modulation of gene expression and DNA	Carcinogenesis	2006
PAHs	NFKBIA	Yvonne C.M. Staal, et al.	Modulation of gene expression and DNA	Carcinogenesis	2006
PAHs	VAT1	Yvonne C.M. Staal, et al.	Modulation of gene expression and DNA	Carcinogenesis	2006
PAHs	VMP1	Yvonne C.M. Staal, et al.	Modulation of gene expression and DNA	Carcinogenesis	2006
PAHs	C15orf16	van Leeuwen DM, et al.	Genome-wide differential gene expression in	Mutation Research	2006
PAHs	DUSP15	van Leeuwen DM, et al.	Genome-wide differential gene expression in	Mutation Research	2006
PAHs	GPR20	van Leeuwen DM, et al.	Genome-wide differential gene expression in	Mutation Research	2006
PAHs	FHKL18	van Leeuwen DM, et al.	Genome-wide differential gene expression in	Mutation Research	2006
PAHs	NEUROG3	van Leeuwen DM, et al.	Genome-wide differential gene expression in	Mutation Research	2006
PAHs	TTS	van Leeuwen DM, et al.	Genome-wide differential gene expression in	Mutation Research	2006
PAHs	PINK1	van Leeuwen DM, et al.	Genome-wide differential gene expression in	Mutation Research	2006
PAHs	COX6A2	van Leeuwen DM, et al.	Genome-wide differential gene expression in	Mutation Research	2006
PAHs	HOWB8	van Leeuwen DM, et al.	Genome-wide differential gene expression in	Mutation Research	2006
PAHs	FXN	van Leeuwen DM, et al.	Genome-wide differential gene expression in	Mutation Research	2006
PAHs	MPST	van Leeuwen DM, et al.	Genome-wide differential gene expression in	Mutation Research	2006
PAHs	PCSK1N	van Leeuwen DM, et al.	Genome-wide differential gene expression in	Mutation Research	2006
PAHs	EMCN	van Leeuwen DM, et al.	Genome-wide differential gene expression in	Mutation Research	2006
PAHs	GPSM3	van Leeuwen DM, et al.	Genome-wide differential gene expression in	Mutation Research	2006
PAHs	SRGAP2	van Leeuwen DM, et al.	Genome-wide differential gene expression in	Mutation Research	2006
PAHs	CCS	van Leeuwen DM, et al.	Genome-wide differential gene expression in	Mutation Research	2006
PAHs	PP2447	van Leeuwen DM, et al.	Genome-wide differential gene expression in	Mutation Research	2006
PAHs	BBC3	van Leeuwen DM, et al.	Genome-wide differential gene expression in	Mutation Research	2006
PAHs	CXCL1	van Leeuwen DM, et al.	Genome-wide differential gene expression in	Mutation Research	2006
PAHs	GPR153	van Leeuwen DM, et al.	Genome-wide differential gene expression in	Mutation Research	2006
PAHs	TIP39	van Leeuwen DM, et al.	Genome-wide differential gene expression in	Mutation Research	2006
PAHs	GPR78	van Leeuwen DM, et al.	Genome-wide differential gene expression in	Mutation Research	2006
PAHs	HISTH1C	van Leeuwen DM, et al.	Genome-wide differential gene expression in	Mutation Research	2006
PAHs	ITGA2	Song MK, et al	Polycyclic aromatic hydrocarbons induce	Cancer Science	2011
PAHs	SNAI1	Song MK, et al	Polycyclic aromatic hydrocarbons induce	Cancer Science	2011
PAHs	AP-1	Song MK, et al	Polycyclic aromatic hydrocarbons induce	Cancer Science	2011
PAHs	ETS-1	Song MK, et al	Polycyclic aromatic hydrocarbons induce	Cancer Science	2011
PAHs	MMP1	Song MK, et al	Polycyclic aromatic hydrocarbons induce	Cancer Science	2011
PAHs	MMP25	Song MK, et al	Polycyclic aromatic hydrocarbons induce	Cancer Science	2011
PAHs	AADACL1	An YR, et al.	Differential expression of cell cycle related	BioChip Journal	2010
PAHs	CD109	An YR, et al.	Differential expression of cell cycle related	BioChip Journal	2010
PAHs	CTSB	An YR, et al.	Differential expression of cell cycle related	BioChip Journal	2010
PAHs	CDCA5	An YR, et al.	Differential expression of cell cycle related	BioChip Journal	2010
PAHs	TOP2A	An YR, et al.	Differential expression of cell cycle related	BioChip Journal	2010
PAHs	AURKB	An YR, et al.	Differential expression of cell cycle related	BioChip Journal	2010
PAHs	AURKA	An YR, et al.	Differential expression of cell cycle related	BioChip Journal	2010
PAHs	HMGB2	An YR, et al.	Differential expression of cell cycle related	BioChip Journal	2010
PAHs	STMN1	An YR, et al.	Differential expression of cell cycle related	BioChip Journal	2010
PAHs	UHRF1	An YR, et al.	Differential expression of cell cycle related	BioChip Journal	2010
PAHs	LMNB1	An YR, et al.	Differential expression of cell cycle related	BioChip Journal	2010
PAHs	TTK T	An YR, et al.	Differential expression of cell cycle related	BioChip Journal	2010
PAHs	AKT2	An YR, et al.	Differential expression of cell cycle related	BioChip Journal	2010
PAHs	CCNA2	An YR, et al.	Differential expression of cell cycle related	BioChip Journal	2010
PAHs	CCNB2	An YR, et al.	Differential expression of cell cycle related	BioChip Journal	2010
PAHs	PHGDH	An YR, et al.	Differential expression of cell cycle related	BioChip Journal	2010
PAHs	MAD2L	An YR, et al.	Differential expression of cell cycle related	BioChip Journal	2010
PAHs	MCM7	An YR, et al.	Differential expression of cell cycle related	BioChip Journal	2010
PAHs	NDC80	An YR, et al.	Differential expression of cell cycle related	BioChip Journal	2010
PAHs	PCSK6	An YR, et al.	Differential expression of cell cycle related	BioChip Journal	2010
PAHs	PLK1	An YR, et al.	Differential expression of cell cycle related	BioChip Journal	2010
PAHs	NOTCH	An YR, et al.	Differential expression of cell cycle related	BioChip Journal	2010
PAHs	CENPA	An YR, et al.	Differential expression of cell cycle related	BioChip Journal	2010
PAHs	FEN1	An YR, et al.	Differential expression of cell cycle related	BioChip Journal	2010
PAHs	ASPM	An YR, et al.	Differential expression of cell cycle related	BioChip Journal	2010
PAHs	CENPF	An YR, et al.	Differential expression of cell cycle related	BioChip Journal	2010
PAHs	CKS1B	An YR, et al.	Differential expression of cell cycle related	BioChip Journal	2010
PAHs	AGPAT	An YR, et al.	Differential expression of cell cycle related	BioChip Journal	2010
PAHs	PBK	An YR, et al.	Differential expression of cell cycle related	BioChip Journal	2010
PAHs	CYP1A1	Fabiola Castorena-Torres, et	Changes in gene expression induced by	Toxicology in Vitro	2008
PAHs	CYP1B1	Fabiola Castorena-Torres, et	Changes in gene expression induced by	Toxicology in Vitro	2008
PAHs	FTH1	Fabiola Castorena-Torres, et	Changes in gene expression induced by	Toxicology in Vitro	2008
PAHs	PRDX1	Fabiola Castorena-Torres, et	Changes in gene expression induced by	Toxicology in Vitro	2008
PAHs	PSMD7	Fabiola Castorena-Torres, et	Changes in gene expression induced by	Toxicology in Vitro	2008
PAHs	FDFT1	Fabiola Castorena-Torres, et	Changes in gene expression induced by	Toxicology in Vitro	2008
PAHs	PTGER3	Fabiola Castorena-Torres, et	Changes in gene expression induced by	Toxicology in Vitro	2008
PAHs	ERCC5	Fabiola Castorena-Torres, et	Changes in gene expression induced by	Toxicology in Vitro	2008
PAHs	FXYD3	Fabiola Castorena-Torres, et	Changes in gene expression induced by	Toxicology in Vitro	2008
PAHs	FLT	Campo, L., Fustinoni, S. &	Quantification of carcinogenic 4- to 6-ring	Analytical and Bioanalytical Chemistry	2011
PAHs	PYR	Campo, L., Fustinoni, S. &	Quantification of carcinogenic 4- to 6-ring	Analytical and Bioanalytical Chemistry	2011
PAHs	BaA	Campo, L., Fustinoni, S. &	Quantification of carcinogenic 4- to 6-ring	Analytical and Bioanalytical Chemistry	2011
PAHs	CHR	Campo, L., Fustinoni, S. &	Quantification of carcinogenic 4- to 6-ring	Analytical and Bioanalytical Chemistry	2011
PAHs	BbF	Campo, L., Fustinoni, S. &	Quantification of carcinogenic 4- to 6-ring	Analytical and Bioanalytical Chemistry	2011
PAHs	BkF	Campo, L., Fustinoni, S. &	Quantification of carcinogenic 4- to 6-ring	Analytical and Bioanalytical Chemistry	2011
PAHs	BaP	Campo, L., Fustinoni, S. &	Quantification of carcinogenic 4- to 6-ring	Analytical and Bioanalytical Chemistry	2011

PAHs	DahA	Campo, L., Fustinoni, S. &	Quantification of carcinogenic 4- to 6-ring	Analytical and Bioanalytical Chemistry	2011
PAHs	Ip	Campo, L., Fustinoni, S. &	Quantification of carcinogenic 4- to 6-ring	Analytical and Bioanalytical Chemistry	2011
PAHs	BghiP	Campo, L., Fustinoni, S. &	Quantification of carcinogenic 4- to 6-ring	Analytical and Bioanalytical Chemistry	2011
PAHs	Bcl2	E Micheli, A Altieri et al	Perylene and coronene derivatives binding to	Biochimie	2016
PAHs	Myc2345	E Micheli, A Altieri et al	Perylene and coronene derivatives binding to	Biochimie	2016
Coal Ash	CXCL8	Deering-Rice CE, et al.	Transient receptor potential vanilloid-1	Mol Pharmaco	2012
Coal Ash	IL6	Deering-Rice CE, et al.	Transient receptor potential vanilloid-1	Mol Pharmaco	2012
Coal Ash	CXCL8	Newland N, et al.	Agents associated with lung inflammation	Toxicol In Vitro	2008
Coal Ash	IL6	Carter JD, et al.	Cytokine production by human airway	Toxicol Appl Pharmacol	1997
Coal Ash	CXCL8	Carter JD, et al.	Cytokine production by human airway	Toxicol Appl Pharmacol	1997
Coal Ash	AATK	Nadadur SS, et al.	Endothelial effects of emission source	Toxicol In Vitro	2009
Coal Ash	ABL1	Nadadur SS, et al.	Endothelial effects of emission source	Toxicol In Vitro	2009
Coal Ash	AOC3	Nadadur SS, et al.	Endothelial effects of emission source	Toxicol In Vitro	2009
Coal Ash	ATP12A	Nadadur SS, et al.	Endothelial effects of emission source	Toxicol In Vitro	2009
Coal Ash	ATP1A2	Nadadur SS, et al.	Endothelial effects of emission source	Toxicol In Vitro	2009
Coal Ash	ATP1B1	Nadadur SS, et al.	Endothelial effects of emission source	Toxicol In Vitro	2009
Coal Ash	ATP1B4	Nadadur SS, et al.	Endothelial effects of emission source	Toxicol In Vitro	2009
Coal Ash	ATP2B1	Nadadur SS, et al.	Endothelial effects of emission source	Toxicol In Vitro	2009
Coal Ash	ATP2B2	Nadadur SS, et al.	Endothelial effects of emission source	Toxicol In Vitro	2009
Coal Ash	ATP2B3	Nadadur SS, et al.	Endothelial effects of emission source	Toxicol In Vitro	2009
Coal Ash	ATP5F1	Nadadur SS, et al.	Endothelial effects of emission source	Toxicol In Vitro	2009
Coal Ash	ATP5G3	Nadadur SS, et al.	Endothelial effects of emission source	Toxicol In Vitro	2009
Coal Ash	ATP5H	Nadadur SS, et al.	Endothelial effects of emission source	Toxicol In Vitro	2009
Coal Ash	ATP5J	Nadadur SS, et al.	Endothelial effects of emission source	Toxicol In Vitro	2009
Coal Ash	ATP6AP1	Nadadur SS, et al.	Endothelial effects of emission source	Toxicol In Vitro	2009
Coal Ash	ATP6V0B	Nadadur SS, et al.	Endothelial effects of emission source	Toxicol In Vitro	2009
Coal Ash	ATP6V0C	Nadadur SS, et al.	Endothelial effects of emission source	Toxicol In Vitro	2009
Coal Ash	ATP7B	Nadadur SS, et al.	Endothelial effects of emission source	Toxicol In Vitro	2009
Coal Ash	BMX	Nadadur SS, et al.	Endothelial effects of emission source	Toxicol In Vitro	2009
Coal Ash	CACNA1F	Nadadur SS, et al.	Endothelial effects of emission source	Toxicol In Vitro	2009
Coal Ash	CACNA1S	Nadadur SS, et al.	Endothelial effects of emission source	Toxicol In Vitro	2009
Coal Ash	CACNA2D2	Nadadur SS, et al.	Endothelial effects of emission source	Toxicol In Vitro	2009
Coal Ash	CACNB1	Nadadur SS, et al.	Endothelial effects of emission source	Toxicol In Vitro	2009
Coal Ash	CACNB3	Nadadur SS, et al.	Endothelial effects of emission source	Toxicol In Vitro	2009
Coal Ash	CACNG2	Nadadur SS, et al.	Endothelial effects of emission source	Toxicol In Vitro	2009
Coal Ash	CAMKK2	Nadadur SS, et al.	Endothelial effects of emission source	Toxicol In Vitro	2009
Coal Ash	CD36	Nadadur SS, et al.	Endothelial effects of emission source	Toxicol In Vitro	2009
Coal Ash	CDK16	Nadadur SS, et al.	Endothelial effects of emission source	Toxicol In Vitro	2009
Coal Ash	CDK17	Nadadur SS, et al.	Endothelial effects of emission source	Toxicol In Vitro	2009
Coal Ash	CDK18	Nadadur SS, et al.	Endothelial effects of emission source	Toxicol In Vitro	2009
Coal Ash	CDK6	Nadadur SS, et al.	Endothelial effects of emission source	Toxicol In Vitro	2009
Coal Ash	CDK8	Nadadur SS, et al.	Endothelial effects of emission source	Toxicol In Vitro	2009
Coal Ash	CEACAM8	Nadadur SS, et al.	Endothelial effects of emission source	Toxicol In Vitro	2009
Coal Ash	COL19A1	Nadadur SS, et al.	Endothelial effects of emission source	Toxicol In Vitro	2009
Coal Ash	COL1A1	Nadadur SS, et al.	Endothelial effects of emission source	Toxicol In Vitro	2009
Coal Ash	COL4A2	Nadadur SS, et al.	Endothelial effects of emission source	Toxicol In Vitro	2009
Coal Ash	COL4A6	Nadadur SS, et al.	Endothelial effects of emission source	Toxicol In Vitro	2009
Coal Ash	COL5A1	Nadadur SS, et al.	Endothelial effects of emission source	Toxicol In Vitro	2009
Coal Ash	COL6A1	Nadadur SS, et al.	Endothelial effects of emission source	Toxicol In Vitro	2009
Coal Ash	CSK	Nadadur SS, et al.	Endothelial effects of emission source	Toxicol In Vitro	2009
Coal Ash	CSNK2A2	Nadadur SS, et al.	Endothelial effects of emission source	Toxicol In Vitro	2009
Coal Ash	CXCL8	Nadadur SS, et al.	Endothelial effects of emission source	Toxicol In Vitro	2009
Coal Ash	CYP1A1	Nadadur SS, et al.	Endothelial effects of emission source	Toxicol In Vitro	2009
Coal Ash	DDIT3	Nadadur SS, et al.	Endothelial effects of emission source	Toxicol In Vitro	2009
Coal Ash	DGKG	Nadadur SS, et al.	Endothelial effects of emission source	Toxicol In Vitro	2009
Coal Ash	DGKQ	Nadadur SS, et al.	Endothelial effects of emission source	Toxicol In Vitro	2009
Coal Ash	EMILIN1	Nadadur SS, et al.	Endothelial effects of emission source	Toxicol In Vitro	2009
Coal Ash	EPHB1	Nadadur SS, et al.	Endothelial effects of emission source	Toxicol In Vitro	2009
Coal Ash	EPHB2	Nadadur SS, et al.	Endothelial effects of emission source	Toxicol In Vitro	2009
Coal Ash	FGF17	Nadadur SS, et al.	Endothelial effects of emission source	Toxicol In Vitro	2009
Coal Ash	FRS2	Nadadur SS, et al.	Endothelial effects of emission source	Toxicol In Vitro	2009
Coal Ash	GADD45G	Nadadur SS, et al.	Endothelial effects of emission source	Toxicol In Vitro	2009
Coal Ash	HBEGF	Nadadur SS, et al.	Endothelial effects of emission source	Toxicol In Vitro	2009
Coal Ash	ICAM2	Nadadur SS, et al.	Endothelial effects of emission source	Toxicol In Vitro	2009
Coal Ash	ICAM3	Nadadur SS, et al.	Endothelial effects of emission source	Toxicol In Vitro	2009
Coal Ash	IGFALS	Nadadur SS, et al.	Endothelial effects of emission source	Toxicol In Vitro	2009
Coal Ash	IGFBP3	Nadadur SS, et al.	Endothelial effects of emission source	Toxicol In Vitro	2009
Coal Ash	IGFBP5	Nadadur SS, et al.	Endothelial effects of emission source	Toxicol In Vitro	2009
Coal Ash	IGFBP7	Nadadur SS, et al.	Endothelial effects of emission source	Toxicol In Vitro	2009
Coal Ash	IL6	Nadadur SS, et al.	Endothelial effects of emission source	Toxicol In Vitro	2009
Coal Ash	ILK	Nadadur SS, et al.	Endothelial effects of emission source	Toxicol In Vitro	2009
Coal Ash	JAK3	Nadadur SS, et al.	Endothelial effects of emission source	Toxicol In Vitro	2009
Coal Ash	KCNE3	Nadadur SS, et al.	Endothelial effects of emission source	Toxicol In Vitro	2009
Coal Ash	KCNE5	Nadadur SS, et al.	Endothelial effects of emission source	Toxicol In Vitro	2009
Coal Ash	KCNF1	Nadadur SS, et al.	Endothelial effects of emission source	Toxicol In Vitro	2009
Coal Ash	KCNG2	Nadadur SS, et al.	Endothelial effects of emission source	Toxicol In Vitro	2009
Coal Ash	KCNH4	Nadadur SS, et al.	Endothelial effects of emission source	Toxicol In Vitro	2009
Coal Ash	KCNQ4	Nadadur SS, et al.	Endothelial effects of emission source	Toxicol In Vitro	2009
Coal Ash	LTA	Nadadur SS, et al.	Endothelial effects of emission source	Toxicol In Vitro	2009
Coal Ash	MAP2K6	Nadadur SS, et al.	Endothelial effects of emission source	Toxicol In Vitro	2009
Coal Ash	MAP3K2	Nadadur SS, et al.	Endothelial effects of emission source	Toxicol In Vitro	2009
Coal Ash	MAP3K4	Nadadur SS, et al.	Endothelial effects of emission source	Toxicol In Vitro	2009
Coal Ash	MAP4K4	Nadadur SS, et al.	Endothelial effects of emission source	Toxicol In Vitro	2009
Coal Ash	MAP4K5	Nadadur SS, et al.	Endothelial effects of emission source	Toxicol In Vitro	2009
Coal Ash	MEGF8	Nadadur SS, et al.	Endothelial effects of emission source	Toxicol In Vitro	2009
Coal Ash	MFAP2	Nadadur SS, et al.	Endothelial effects of emission source	Toxicol In Vitro	2009
Coal Ash	MKNK1	Nadadur SS, et al.	Endothelial effects of emission source	Toxicol In Vitro	2009
Coal Ash	MMP1	Nadadur SS, et al.	Endothelial effects of emission source	Toxicol In Vitro	2009
Coal Ash	MMP1	Nadadur SS, et al.	Endothelial effects of emission source	Toxicol In Vitro	2009

Coal Ash	MMP13	Nadadur SS, et al.	Endothelial effects of emission source	Toxicol In Vitro	2009
Coal Ash	MMP14	Nadadur SS, et al.	Endothelial effects of emission source	Toxicol In Vitro	2009
Coal Ash	MST1R	Nadadur SS, et al.	Endothelial effects of emission source	Toxicol In Vitro	2009
Coal Ash	MUC5AC	Nadadur SS, et al.	Endothelial effects of emission source	Toxicol In Vitro	2009
Coal Ash	P4HB	Nadadur SS, et al.	Endothelial effects of emission source	Toxicol In Vitro	2009
Coal Ash	PAFAH1B3	Nadadur SS, et al.	Endothelial effects of emission source	Toxicol In Vitro	2009
Coal Ash	PDGFB	Nadadur SS, et al.	Endothelial effects of emission source	Toxicol In Vitro	2009
Coal Ash	PECAM1	Nadadur SS, et al.	Endothelial effects of emission source	Toxicol In Vitro	2009
Coal Ash	PF4V1	Nadadur SS, et al.	Endothelial effects of emission source	Toxicol In Vitro	2009
Coal Ash	PFKP	Nadadur SS, et al.	Endothelial effects of emission source	Toxicol In Vitro	2009
Coal Ash	PIK3R3	Nadadur SS, et al.	Endothelial effects of emission source	Toxicol In Vitro	2009
Coal Ash	PLOD3	Nadadur SS, et al.	Endothelial effects of emission source	Toxicol In Vitro	2009
Coal Ash	PRKACG	Nadadur SS, et al.	Endothelial effects of emission source	Toxicol In Vitro	2009
Coal Ash	PRKCB	Nadadur SS, et al.	Endothelial effects of emission source	Toxicol In Vitro	2009
Coal Ash	PRKCI	Nadadur SS, et al.	Endothelial effects of emission source	Toxicol In Vitro	2009
Coal Ash	PRKCQ	Nadadur SS, et al.	Endothelial effects of emission source	Toxicol In Vitro	2009
Coal Ash	PRKG2	Nadadur SS, et al.	Endothelial effects of emission source	Toxicol In Vitro	2009
Coal Ash	PTK2	Nadadur SS, et al.	Endothelial effects of emission source	Toxicol In Vitro	2009
Coal Ash	PTK2B	Nadadur SS, et al.	Endothelial effects of emission source	Toxicol In Vitro	2009
Coal Ash	PTK6	Nadadur SS, et al.	Endothelial effects of emission source	Toxicol In Vitro	2009
Coal Ash	SCAMP3	Nadadur SS, et al.	Endothelial effects of emission source	Toxicol In Vitro	2009
Coal Ash	SLC10A1	Nadadur SS, et al.	Endothelial effects of emission source	Toxicol In Vitro	2009
Coal Ash	SLC12A3	Nadadur SS, et al.	Endothelial effects of emission source	Toxicol In Vitro	2009
Coal Ash	SLC12A4	Nadadur SS, et al.	Endothelial effects of emission source	Toxicol In Vitro	2009
Coal Ash	SLC12A7	Nadadur SS, et al.	Endothelial effects of emission source	Toxicol In Vitro	2009
Coal Ash	SLC14A2	Nadadur SS, et al.	Endothelial effects of emission source	Toxicol In Vitro	2009
Coal Ash	SLC16A5	Nadadur SS, et al.	Endothelial effects of emission source	Toxicol In Vitro	2009
Coal Ash	SLC17A1	Nadadur SS, et al.	Endothelial effects of emission source	Toxicol In Vitro	2009
Coal Ash	SLC1A1	Nadadur SS, et al.	Endothelial effects of emission source	Toxicol In Vitro	2009
Coal Ash	SLC1A3	Nadadur SS, et al.	Endothelial effects of emission source	Toxicol In Vitro	2009
Coal Ash	SLC1A4	Nadadur SS, et al.	Endothelial effects of emission source	Toxicol In Vitro	2009
Coal Ash	SLC1A5	Nadadur SS, et al.	Endothelial effects of emission source	Toxicol In Vitro	2009
Coal Ash	SLC22A7	Nadadur SS, et al.	Endothelial effects of emission source	Toxicol In Vitro	2009
Coal Ash	SLC25A5	Nadadur SS, et al.	Endothelial effects of emission source	Toxicol In Vitro	2009
Coal Ash	SLC2A2	Nadadur SS, et al.	Endothelial effects of emission source	Toxicol In Vitro	2009
Coal Ash	SLC2A4	Nadadur SS, et al.	Endothelial effects of emission source	Toxicol In Vitro	2009
Coal Ash	SLC30A4	Nadadur SS, et al.	Endothelial effects of emission source	Toxicol In Vitro	2009
Coal Ash	SLC31A1	Nadadur SS, et al.	Endothelial effects of emission source	Toxicol In Vitro	2009
Coal Ash	SLC3A2	Nadadur SS, et al.	Endothelial effects of emission source	Toxicol In Vitro	2009
Coal Ash	SLC6A1	Nadadur SS, et al.	Endothelial effects of emission source	Toxicol In Vitro	2009
Coal Ash	SLC6A6	Nadadur SS, et al.	Endothelial effects of emission source	Toxicol In Vitro	2009
Coal Ash	SLC6A9	Nadadur SS, et al.	Endothelial effects of emission source	Toxicol In Vitro	2009
Coal Ash	SLC7A1	Nadadur SS, et al.	Endothelial effects of emission source	Toxicol In Vitro	2009
Coal Ash	SLC9A3R2	Nadadur SS, et al.	Endothelial effects of emission source	Toxicol In Vitro	2009
Coal Ash	SMARCD1	Nadadur SS, et al.	Endothelial effects of emission source	Toxicol In Vitro	2009
Coal Ash	STK17A	Nadadur SS, et al.	Endothelial effects of emission source	Toxicol In Vitro	2009
Coal Ash	STK24	Nadadur SS, et al.	Endothelial effects of emission source	Toxicol In Vitro	2009
Coal Ash	STK4	Nadadur SS, et al.	Endothelial effects of emission source	Toxicol In Vitro	2009
Coal Ash	TAP2	Nadadur SS, et al.	Endothelial effects of emission source	Toxicol In Vitro	2009
Coal Ash	TIMP1	Nadadur SS, et al.	Endothelial effects of emission source	Toxicol In Vitro	2009
Coal Ash	TMEFF1	Nadadur SS, et al.	Endothelial effects of emission source	Toxicol In Vitro	2009
Coal Ash	TNF	Nadadur SS, et al.	Endothelial effects of emission source	Toxicol In Vitro	2009
Coal Ash	TNNI3K	Nadadur SS, et al.	Endothelial effects of emission source	Toxicol In Vitro	2009
Coal Ash	TYRO3	Nadadur SS, et al.	Endothelial effects of emission source	Toxicol In Vitro	2009
Coal Ash	VDAC2	Nadadur SS, et al.	Endothelial effects of emission source	Toxicol In Vitro	2009
Coal Ash	VEGFC	Nadadur SS, et al.	Endothelial effects of emission source	Toxicol In Vitro	2009
