# Supplementary material for: Integrative analysis to explore the biological association between environmental skin diseases and ambient particulate matter
Source: Sci Rep. 2022 Jun 13;12:9750. doi: 10.1038/s41598-022-13001-x (PMC9192598; doi:10.1038/s41598-022-13001-x)
Supplement: Supplementary file 11 — Supplementary Information 9. [file 41598_2022_13001_MOESM11_ESM.pdf]

Keywords	Gene symbol	Title	Journal	Year
PM2.5	MT1F	Fine Ambient Particles Induce Oxidative Stress and Metal Binding Genes in Human Alveolar Macrophages	Am J Respir Cell Mol Biol	2009
PM2.5	NCF1	Fine Ambient Particles Induce Oxidative Stress and Metal Binding Genes in Human Alveolar Macrophages	Am J Respir Cell Mol Biol	2009
PM2.5	MT1H	Fine Ambient Particles Induce Oxidative Stress and Metal Binding Genes in Human Alveolar Macrophages	Am J Respir Cell Mol Biol	2009
PM2.5	MT1G	Fine Ambient Particles Induce Oxidative Stress and Metal Binding Genes in Human Alveolar Macrophages	Am J Respir Cell Mol Biol	2009
PM2.5	MT1B	Fine Ambient Particles Induce Oxidative Stress and Metal Binding Genes in Human Alveolar Macrophages	Am J Respir Cell Mol Biol	2009
PM2.5	NINJ1	Fine Ambient Particles Induce Oxidative Stress and Metal Binding Genes in Human Alveolar Macrophages	Am J Respir Cell Mol Biol	2009
PM2.5	ADM	Fine Ambient Particles Induce Oxidative Stress and Metal Binding Genes in Human Alveolar Macrophages	Am J Respir Cell Mol Biol	2009
PM2.5	IL18BP	Fine Ambient Particles Induce Oxidative Stress and Metal Binding Genes in Human Alveolar Macrophages	Am J Respir Cell Mol Biol	2009
PM2.5	RELB	Fine Ambient Particles Induce Oxidative Stress and Metal Binding Genes in Human Alveolar Macrophages	Am J Respir Cell Mol Biol	2009
PM2.5	CYP1B1	Fine Ambient Particles Induce Oxidative Stress and Metal Binding Genes in Human Alveolar Macrophages	Am J Respir Cell Mol Biol	2009
PM2.5	ENO1	Fine Ambient Particles Induce Oxidative Stress and Metal Binding Genes in Human Alveolar Macrophages	Am J Respir Cell Mol Biol	2009
PM2.5	BCL3	Fine Ambient Particles Induce Oxidative Stress and Metal Binding Genes in Human Alveolar Macrophages	Am J Respir Cell Mol Biol	2009
PM2.5	PFKP	Fine Ambient Particles Induce Oxidative Stress and Metal Binding Genes in Human Alveolar Macrophages	Am J Respir Cell Mol Biol	2009
PM2.5	NQO1	Fine Ambient Particles Induce Oxidative Stress and Metal Binding Genes in Human Alveolar Macrophages	Am J Respir Cell Mol Biol	2009
PM2.5	ENO1B	Fine Ambient Particles Induce Oxidative Stress and Metal Binding Genes in Human Alveolar Macrophages	Am J Respir Cell Mol Biol	2009
PM2.5	MMP9	Fine Ambient Particles Induce Oxidative Stress and Metal Binding Genes in Human Alveolar Macrophages	Am J Respir Cell Mol Biol	2009
PM2.5	PTPRE	Fine Ambient Particles Induce Oxidative Stress and Metal Binding Genes in Human Alveolar Macrophages	Am J Respir Cell Mol Biol	2009
PM2.5	GAPDH	Fine Ambient Particles Induce Oxidative Stress and Metal Binding Genes in Human Alveolar Macrophages	Am J Respir Cell Mol Biol	2009
PM2.5	KYNU	Fine Ambient Particles Induce Oxidative Stress and Metal Binding Genes in Human Alveolar Macrophages	Am J Respir Cell Mol Biol	2009
PM2.5	DFNA5	Fine Ambient Particles Induce Oxidative Stress and Metal Binding Genes in Human Alveolar Macrophages	Am J Respir Cell Mol Biol	2009
PM2.5	AIFM2	Fine Ambient Particles Induce Oxidative Stress and Metal Binding Genes in Human Alveolar Macrophages	Am J Respir Cell Mol Biol	2009
PM2.5	TUBB	Fine Ambient Particles Induce Oxidative Stress and Metal Binding Genes in Human Alveolar Macrophages	Am J Respir Cell Mol Biol	2009
PM2.5	MPZL1	Fine Ambient Particles Induce Oxidative Stress and Metal Binding Genes in Human Alveolar Macrophages	Am J Respir Cell Mol Biol	2009
PM2.5	TFPI	Fine Ambient Particles Induce Oxidative Stress and Metal Binding Genes in Human Alveolar Macrophages	Am J Respir Cell Mol Biol	2009
PM2.5	PDXK	Fine Ambient Particles Induce Oxidative Stress and Metal Binding Genes in Human Alveolar Macrophages	Am J Respir Cell Mol Biol	2009
PM2.5	PPP1R15A	Fine Ambient Particles Induce Oxidative Stress and Metal Binding Genes in Human Alveolar Macrophages	Am J Respir Cell Mol Biol	2009
PM2.5	ATOX1	Fine Ambient Particles Induce Oxidative Stress and Metal Binding Genes in Human Alveolar Macrophages	Am J Respir Cell Mol Biol	2009
PM2.5	NR1H3	Fine Ambient Particles Induce Oxidative Stress and Metal Binding Genes in Human Alveolar Macrophages	Am J Respir Cell Mol Biol	2009
PM2.5	FGR	Fine Ambient Particles Induce Oxidative Stress and Metal Binding Genes in Human Alveolar Macrophages	Am J Respir Cell Mol Biol	2009
PM2.5	MYOD1	Fine Ambient Particles Induce Oxidative Stress and Metal Binding Genes in Human Alveolar Macrophages	Am J Respir Cell Mol Biol	2009
PM2.5	CHST11	Fine Ambient Particles Induce Oxidative Stress and Metal Binding Genes in Human Alveolar Macrophages	Am J Respir Cell Mol Biol	2009
PM2.5	GPR3	Fine Ambient Particles Induce Oxidative Stress and Metal Binding Genes in Human Alveolar Macrophages	Am J Respir Cell Mol Biol	2009
PM2.5	NRBP	Fine Ambient Particles Induce Oxidative Stress and Metal Binding Genes in Human Alveolar Macrophages	Am J Respir Cell Mol Biol	2009

PM2.5	CCL4	Fine Ambient Particles Induce Oxidative Stress and Metal Binding Genes in Human Alveolar Macrophages	Am J Respir Cell Mol Biol	2009
PM2.5	S100A8	Fine Ambient Particles Induce Oxidative Stress and Metal Binding Genes in Human Alveolar Macrophages	Am J Respir Cell Mol Biol	2009
PM2.5	CAMP	Fine Ambient Particles Induce Oxidative Stress and Metal Binding Genes in Human Alveolar Macrophages	Am J Respir Cell Mol Biol	2009
PM2.5	LAT2	Fine Ambient Particles Induce Oxidative Stress and Metal Binding Genes in Human Alveolar Macrophages	Am J Respir Cell Mol Biol	2009
PM2.5	SLCO2B1	Fine Ambient Particles Induce Oxidative Stress and Metal Binding Genes in Human Alveolar Macrophages	Am J Respir Cell Mol Biol	2009
PM2.5	CPVL	Fine Ambient Particles Induce Oxidative Stress and Metal Binding Genes in Human Alveolar Macrophages	Am J Respir Cell Mol Biol	2009
PM2.5	MNDA	Fine Ambient Particles Induce Oxidative Stress and Metal Binding Genes in Human Alveolar Macrophages	Am J Respir Cell Mol Biol	2009
PM2.5	MRC1	Fine Ambient Particles Induce Oxidative Stress and Metal Binding Genes in Human Alveolar Macrophages	Am J Respir Cell Mol Biol	2009
PM2.5	EDG6	Fine Ambient Particles Induce Oxidative Stress and Metal Binding Genes in Human Alveolar Macrophages	Am J Respir Cell Mol Biol	2009
PM2.5	PROS1	Fine Ambient Particles Induce Oxidative Stress and Metal Binding Genes in Human Alveolar Macrophages	Am J Respir Cell Mol Biol	2009
PM2.5	IFIT1	Fine Ambient Particles Induce Oxidative Stress and Metal Binding Genes in Human Alveolar Macrophages	Am J Respir Cell Mol Biol	2009
PM2.5	GCA	Fine Ambient Particles Induce Oxidative Stress and Metal Binding Genes in Human Alveolar Macrophages	Am J Respir Cell Mol Biol	2009
PM2.5	ADRB2	Fine Ambient Particles Induce Oxidative Stress and Metal Binding Genes in Human Alveolar Macrophages	Am J Respir Cell Mol Biol	2009
PM2.5	H2AFZ	Fine Ambient Particles Induce Oxidative Stress and Metal Binding Genes in Human Alveolar Macrophages	Am J Respir Cell Mol Biol	2009
PM2.5	CPE	Fine Ambient Particles Induce Oxidative Stress and Metal Binding Genes in Human Alveolar Macrophages	Am J Respir Cell Mol Biol	2009
PM2.5	CLEC4A	Fine Ambient Particles Induce Oxidative Stress and Metal Binding Genes in Human Alveolar Macrophages	Am J Respir Cell Mol Biol	2009
PM2.5	FBXL5	Fine Ambient Particles Induce Oxidative Stress and Metal Binding Genes in Human Alveolar Macrophages	Am J Respir Cell Mol Biol	2009
PM2.5	CXCL2	Fine Ambient Particles Induce Oxidative Stress and Metal Binding Genes in Human Alveolar Macrophages	Am J Respir Cell Mol Biol	2009
PM2.5	ASGR1	Fine Ambient Particles Induce Oxidative Stress and Metal Binding Genes in Human Alveolar Macrophages	Am J Respir Cell Mol Biol	2009
PM2.5	HIST1H4A	Fine Ambient Particles Induce Oxidative Stress and Metal Binding Genes in Human Alveolar Macrophages	Am J Respir Cell Mol Biol	2009
PM2.5	BZRP	Fine Ambient Particles Induce Oxidative Stress and Metal Binding Genes in Human Alveolar Macrophages	Am J Respir Cell Mol Biol	2009
PM2.5	RNF13	Fine Ambient Particles Induce Oxidative Stress and Metal Binding Genes in Human Alveolar Macrophages	Am J Respir Cell Mol Biol	2009
PM2.5	NET1	Fine Ambient Particles Induce Oxidative Stress and Metal Binding Genes in Human Alveolar Macrophages	Am J Respir Cell Mol Biol	2009
PM2.5	CSTA	Fine Ambient Particles Induce Oxidative Stress and Metal Binding Genes in Human Alveolar Macrophages	Am J Respir Cell Mol Biol	2009
PM2.5	PYCARD	Fine Ambient Particles Induce Oxidative Stress and Metal Binding Genes in Human Alveolar Macrophages	Am J Respir Cell Mol Biol	2009
PM2.5	FGL2	Fine Ambient Particles Induce Oxidative Stress and Metal Binding Genes in Human Alveolar Macrophages	Am J Respir Cell Mol Biol	2009
PM2.5	CAPZA2	Fine Ambient Particles Induce Oxidative Stress and Metal Binding Genes in Human Alveolar Macrophages	Am J Respir Cell Mol Biol	2009
PM2.5	C20ORF46	Fine Ambient Particles Induce Oxidative Stress and Metal Binding Genes in Human Alveolar Macrophages	Am J Respir Cell Mol Biol	2009
PM2.5	ECHDC1	Fine Ambient Particles Induce Oxidative Stress and Metal Binding Genes in Human Alveolar Macrophages	Am J Respir Cell Mol Biol	2009
PM2.5	PIGY	Fine Ambient Particles Induce Oxidative Stress and Metal Binding Genes in Human Alveolar Macrophages	Am J Respir Cell Mol Biol	2009
PM2.5	SLA	Fine Ambient Particles Induce Oxidative Stress and Metal Binding Genes in Human Alveolar Macrophages	Am J Respir Cell Mol Biol	2009
PM2.5	STMN1	Fine Ambient Particles Induce Oxidative Stress and Metal Binding Genes in Human Alveolar Macrophages	Am J Respir Cell Mol Biol	2009
PM2.5	PECAM1	Fine Ambient Particles Induce Oxidative Stress and Metal Binding Genes in Human Alveolar Macrophages	Am J Respir Cell Mol Biol	2009
PM2.5	CRIP1	Fine Ambient Particles Induce Oxidative Stress and Metal Binding Genes in Human Alveolar Macrophages	Am J Respir Cell Mol Biol	2009

PM2.5	MS4A6A	Fine Ambient Particles Induce Oxidative Stress and Metal Binding Genes in Human Alveolar Macrophages	Am J Respir Cell Mol Biol	2009
PM2.5	HIST1H3H	Fine Ambient Particles Induce Oxidative Stress and Metal Binding Genes in Human Alveolar Macrophages	Am J Respir Cell Mol Biol	2009
PM2.5	MS4A4A	Fine Ambient Particles Induce Oxidative Stress and Metal Binding Genes in Human Alveolar Macrophages	Am J Respir Cell Mol Biol	2009
PM2.5	VSIG4	Fine Ambient Particles Induce Oxidative Stress and Metal Binding Genes in Human Alveolar Macrophages	Am J Respir Cell Mol Biol	2009
PM2.5	BIRC1	Fine Ambient Particles Induce Oxidative Stress and Metal Binding Genes in Human Alveolar Macrophages	Am J Respir Cell Mol Biol	2009
PM2.5	CALM2	Fine Ambient Particles Induce Oxidative Stress and Metal Binding Genes in Human Alveolar Macrophages	Am J Respir Cell Mol Biol	2009
PM2.5	LPL	Fine Ambient Particles Induce Oxidative Stress and Metal Binding Genes in Human Alveolar Macrophages	Am J Respir Cell Mol Biol	2009
PM2.5	SERPINB2	Integrative transcriptomic and protein analysis of human bronchial BEAS-2B exposed to seasonal urban particulate matter	Environmental Pollution	2016
PM2.5	CYP1A1	Integrative transcriptomic and protein analysis of human bronchial BEAS-2B exposed to seasonal urban particulate matter	Environmental Pollution	2016
PM2.5	IL24	Integrative transcriptomic and protein analysis of human bronchial BEAS-2B exposed to seasonal urban particulate matter	Environmental Pollution	2016
PM2.5	MMP1	Integrative transcriptomic and protein analysis of human bronchial BEAS-2B exposed to seasonal urban particulate matter	Environmental Pollution	2016
PM2.5	CYP1B1	Integrative transcriptomic and protein analysis of human bronchial BEAS-2B exposed to seasonal urban particulate matter	Environmental Pollution	2016
PM2.5	KRT6B	Integrative transcriptomic and protein analysis of human bronchial BEAS-2B exposed to seasonal urban particulate matter	Environmental Pollution	2016
PM2.5	IL8	Integrative transcriptomic and protein analysis of human bronchial BEAS-2B exposed to seasonal urban particulate matter	Environmental Pollution	2016
PM2.5	CCL20	Integrative transcriptomic and protein analysis of human bronchial BEAS-2B exposed to seasonal urban particulate matter	Environmental Pollution	2016
PM2.5	EHF	Integrative transcriptomic and protein analysis of human bronchial BEAS-2B exposed to seasonal urban particulate matter	Environmental Pollution	2016
PM2.5	LOC344887	Integrative transcriptomic and protein analysis of human bronchial BEAS-2B exposed to seasonal urban particulate matter	Environmental Pollution	2016
PM2.5	IL1B	Integrative transcriptomic and protein analysis of human bronchial BEAS-2B exposed to seasonal urban particulate matter	Environmental Pollution	2016
PM2.5	ZBED2	Integrative transcriptomic and protein analysis of human bronchial BEAS-2B exposed to seasonal urban particulate matter	Environmental Pollution	2016
PM2.5	S100P	Integrative transcriptomic and protein analysis of human bronchial BEAS-2B exposed to seasonal urban particulate matter	Environmental Pollution	2016
PM2.5	SERPINB3	Integrative transcriptomic and protein analysis of human bronchial BEAS-2B exposed to seasonal urban particulate matter	Environmental Pollution	2016
PM2.5	SHISA2	Integrative transcriptomic and protein analysis of human bronchial BEAS-2B exposed to seasonal urban particulate matter	Environmental Pollution	2016
PM2.5	GDA	Integrative transcriptomic and protein analysis of human bronchial BEAS-2B exposed to seasonal urban particulate matter	Environmental Pollution	2016
PM2.5	NDRG1	Integrative transcriptomic and protein analysis of human bronchial BEAS-2B exposed to seasonal urban particulate matter	Environmental Pollution	2016
PM2.5	AHRR	Integrative transcriptomic and protein analysis of human bronchial BEAS-2B exposed to seasonal urban particulate matter	Environmental Pollution	2016

PM2.5	NAV3	Integrative transcriptomic and protein analysis of human bronchial BEAS-2B exposed to seasonal urban particulate matter	Environmental Pollution	2016
PM2.5	CXCL1	Integrative transcriptomic and protein analysis of human bronchial BEAS-2B exposed to seasonal urban particulate matter	Environmental Pollution	2016
PM2.5	RUNX2	Integrative transcriptomic and protein analysis of human bronchial BEAS-2B exposed to seasonal urban particulate matter	Environmental Pollution	2016
PM2.5	KYNU	Integrative transcriptomic and protein analysis of human bronchial BEAS-2B exposed to seasonal urban particulate matter	Environmental Pollution	2016
PM2.5	KRT6A	Integrative transcriptomic and protein analysis of human bronchial BEAS-2B exposed to seasonal urban particulate matter	Environmental Pollution	2016
PM2.5	NPTX1	Integrative transcriptomic and protein analysis of human bronchial BEAS-2B exposed to seasonal urban particulate matter	Environmental Pollution	2016
PM2.5	BIRC3	Integrative transcriptomic and protein analysis of human bronchial BEAS-2B exposed to seasonal urban particulate matter	Environmental Pollution	2016
PM2.5	EID3	Integrative transcriptomic and protein analysis of human bronchial BEAS-2B exposed to seasonal urban particulate matter	Environmental Pollution	2016
PM2.5	NQO1	Integrative transcriptomic and protein analysis of human bronchial BEAS-2B exposed to seasonal urban particulate matter	Environmental Pollution	2016
PM2.5	GCLM	Integrative transcriptomic and protein analysis of human bronchial BEAS-2B exposed to seasonal urban particulate matter	Environmental Pollution	2016
PM2.5	KRT6A	Integrative transcriptomic and protein analysis of human bronchial BEAS-2B exposed to seasonal urban particulate matter	Environmental Pollution	2016
PM2.5	CXCL5	Integrative transcriptomic and protein analysis of human bronchial BEAS-2B exposed to seasonal urban particulate matter	Environmental Pollution	2016
PM2.5	INSIG1	Integrative transcriptomic and protein analysis of human bronchial BEAS-2B exposed to seasonal urban particulate matter	Environmental Pollution	2016
PM2.5	TRIM16	Integrative transcriptomic and protein analysis of human bronchial BEAS-2B exposed to seasonal urban particulate matter	Environmental Pollution	2016
PM2.5	CEP290	Integrative transcriptomic and protein analysis of human bronchial BEAS-2B exposed to seasonal urban particulate matter	Environmental Pollution	2016
PM2.5	AKAP12	Integrative transcriptomic and protein analysis of human bronchial BEAS-2B exposed to seasonal urban particulate matter	Environmental Pollution	2016
PM2.5	EMR2	Integrative transcriptomic and protein analysis of human bronchial BEAS-2B exposed to seasonal urban particulate matter	Environmental Pollution	2016
PM2.5	LRRFIP1	Integrative transcriptomic and protein analysis of human bronchial BEAS-2B exposed to seasonal urban particulate matter	Environmental Pollution	2016
PM2.5	TIPARP	Integrative transcriptomic and protein analysis of human bronchial BEAS-2B exposed to seasonal urban particulate matter	Environmental Pollution	2016
PM2.5	PTGS2	Integrative transcriptomic and protein analysis of human bronchial BEAS-2B exposed to seasonal urban particulate matter	Environmental Pollution	2016
PM2.5	PIR	Integrative transcriptomic and protein analysis of human bronchial BEAS-2B exposed to seasonal urban particulate matter	Environmental Pollution	2016
PM2.5	CXCL2	Integrative transcriptomic and protein analysis of human bronchial BEAS-2B exposed to seasonal urban particulate matter	Environmental Pollution	2016

PM2.5	IL6R	Integrative transcriptomic and protein analysis of human bronchial BEAS-2B exposed to seasonal urban particulate matter	Environmental Pollution	2016
PM2.5	NFKBIZ	Integrative transcriptomic and protein analysis of human bronchial BEAS-2B exposed to seasonal urban particulate matter	Environmental Pollution	2016
PM2.5	BHLHE40	Integrative transcriptomic and protein analysis of human bronchial BEAS-2B exposed to seasonal urban particulate matter	Environmental Pollution	2016
PM2.5	LOC145757	Integrative transcriptomic and protein analysis of human bronchial BEAS-2B exposed to seasonal urban particulate matter	Environmental Pollution	2016
PM2.5	TNFAIP6	Integrative transcriptomic and protein analysis of human bronchial BEAS-2B exposed to seasonal urban particulate matter	Environmental Pollution	2016
PM2.5	LIF	Integrative transcriptomic and protein analysis of human bronchial BEAS-2B exposed to seasonal urban particulate matter	Environmental Pollution	2016
PM2.5	DHX9	Integrative transcriptomic and protein analysis of human bronchial BEAS-2B exposed to seasonal urban particulate matter	Environmental Pollution	2016
PM2.5	HIST1H2BG	Integrative transcriptomic and protein analysis of human bronchial BEAS-2B exposed to seasonal urban particulate matter	Environmental Pollution	2016
PM2.5	BOD1L1	Integrative transcriptomic and protein analysis of human bronchial BEAS-2B exposed to seasonal urban particulate matter	Environmental Pollution	2016
PM2.5	PTGES	Integrative transcriptomic and protein analysis of human bronchial BEAS-2B exposed to seasonal urban particulate matter	Environmental Pollution	2016
PM2.5	TNFAIP3	Integrative transcriptomic and protein analysis of human bronchial BEAS-2B exposed to seasonal urban particulate matter	Environmental Pollution	2016
PM2.5	CCDC71L	Integrative transcriptomic and protein analysis of human bronchial BEAS-2B exposed to seasonal urban particulate matter	Environmental Pollution	2016
PM2.5	AKR1C3	Integrative transcriptomic and protein analysis of human bronchial BEAS-2B exposed to seasonal urban particulate matter	Environmental Pollution	2016
PM2.5	MYH10	Integrative transcriptomic and protein analysis of human bronchial BEAS-2B exposed to seasonal urban particulate matter	Environmental Pollution	2016
PM2.5	MAP4K5	Integrative transcriptomic and protein analysis of human bronchial BEAS-2B exposed to seasonal urban particulate matter	Environmental Pollution	2016
PM2.5	DNER	Integrative transcriptomic and protein analysis of human bronchial BEAS-2B exposed to seasonal urban particulate matter	Environmental Pollution	2016
PM2.5	KIAA0368	Integrative transcriptomic and protein analysis of human bronchial BEAS-2B exposed to seasonal urban particulate matter	Environmental Pollution	2016
PM2.5	LOC100134445	Integrative transcriptomic and protein analysis of human bronchial BEAS-2B exposed to seasonal urban particulate matter	Environmental Pollution	2016
PM2.5	MGA	Integrative transcriptomic and protein analysis of human bronchial BEAS-2B exposed to seasonal urban particulate matter	Environmental Pollution	2016
PM2.5	DAPP1	Integrative transcriptomic and protein analysis of human bronchial BEAS-2B exposed to seasonal urban particulate matter	Environmental Pollution	2016
PM2.5	HSP90AA1	Integrative transcriptomic and protein analysis of human bronchial BEAS-2B exposed to seasonal urban particulate matter	Environmental Pollution	2016
PM2.5	LIMA1	Integrative transcriptomic and protein analysis of human bronchial BEAS-2B exposed to seasonal urban particulate matter	Environmental Pollution	2016

PM2.5	IQGAP1	Integrative transcriptomic and protein analysis of human bronchial BEAS-2B exposed to seasonal urban particulate matter	Environmental Pollution	2016
PM2.5	TOP1	Integrative transcriptomic and protein analysis of human bronchial BEAS-2B exposed to seasonal urban particulate matter	Environmental Pollution	2016
PM2.5	CDC42BPA	Integrative transcriptomic and protein analysis of human bronchial BEAS-2B exposed to seasonal urban particulate matter	Environmental Pollution	2016
PM2.5	LUZP1	Integrative transcriptomic and protein analysis of human bronchial BEAS-2B exposed to seasonal urban particulate matter	Environmental Pollution	2016
PM2.5	ARTN	Integrative transcriptomic and protein analysis of human bronchial BEAS-2B exposed to seasonal urban particulate matter	Environmental Pollution	2016
PM2.5	EHBP1	Integrative transcriptomic and protein analysis of human bronchial BEAS-2B exposed to seasonal urban particulate matter	Environmental Pollution	2016
PM2.5	SGOL2	Integrative transcriptomic and protein analysis of human bronchial BEAS-2B exposed to seasonal urban particulate matter	Environmental Pollution	2016
PM2.5	LMLN	Integrative transcriptomic and protein analysis of human bronchial BEAS-2B exposed to seasonal urban particulate matter	Environmental Pollution	2016
PM2.5	RALBP1	Integrative transcriptomic and protein analysis of human bronchial BEAS-2B exposed to seasonal urban particulate matter	Environmental Pollution	2016
PM2.5	ANKRD11	Integrative transcriptomic and protein analysis of human bronchial BEAS-2B exposed to seasonal urban particulate matter	Environmental Pollution	2016
PM2.5	SLC16A7	Integrative transcriptomic and protein analysis of human bronchial BEAS-2B exposed to seasonal urban particulate matter	Environmental Pollution	2016
PM2.5	SLC6A6	Integrative transcriptomic and protein analysis of human bronchial BEAS-2B exposed to seasonal urban particulate matter	Environmental Pollution	2016
PM2.5	MBP	Integrative transcriptomic and protein analysis of human bronchial BEAS-2B exposed to seasonal urban particulate matter	Environmental Pollution	2016
PM2.5	GEN1	Integrative transcriptomic and protein analysis of human bronchial BEAS-2B exposed to seasonal urban particulate matter	Environmental Pollution	2016
PM2.5	RUSC1-AS1	Integrative transcriptomic and protein analysis of human bronchial BEAS-2B exposed to seasonal urban particulate matter	Environmental Pollution	2016
PM2.5	PCYOX1	Integrative transcriptomic and protein analysis of human bronchial BEAS-2B exposed to seasonal urban particulate matter	Environmental Pollution	2016
PM2.5	C10orf118	Integrative transcriptomic and protein analysis of human bronchial BEAS-2B exposed to seasonal urban particulate matter	Environmental Pollution	2016
PM2.5	EPRS	Integrative transcriptomic and protein analysis of human bronchial BEAS-2B exposed to seasonal urban particulate matter	Environmental Pollution	2016
PM2.5	FAM161A	Integrative transcriptomic and protein analysis of human bronchial BEAS-2B exposed to seasonal urban particulate matter	Environmental Pollution	2016
PM2.5	C7orf57	Integrative transcriptomic and protein analysis of human bronchial BEAS-2B exposed to seasonal urban particulate matter	Environmental Pollution	2016
PM2.5	ABCA1	Integrative transcriptomic and protein analysis of human bronchial BEAS-2B exposed to seasonal urban particulate matter	Environmental Pollution	2016
PM2.5	MBTD1	Integrative transcriptomic and protein analysis of human bronchial BEAS-2B exposed to seasonal urban particulate matter	Environmental Pollution	2016

PM2.5	CREBRF	Integrative transcriptomic and protein analysis of human bronchial BEAS-2B exposed to seasonal urban particulate matter	Environmental Pollution	2016
PM2.5	IL1A	Integrative transcriptomic and protein analysis of human bronchial BEAS-2B exposed to seasonal urban particulate matter	Environmental Pollution	2016
PM2.5	OSBPL8	Integrative transcriptomic and protein analysis of human bronchial BEAS-2B exposed to seasonal urban particulate matter	Environmental Pollution	2016
PM2.5	MYO10	Integrative transcriptomic and protein analysis of human bronchial BEAS-2B exposed to seasonal urban particulate matter	Environmental Pollution	2016
PM2.5	NRCAM	Integrative transcriptomic and protein analysis of human bronchial BEAS-2B exposed to seasonal urban particulate matter	Environmental Pollution	2016
PM2.5	NEAT1	Integrative transcriptomic and protein analysis of human bronchial BEAS-2B exposed to seasonal urban particulate matter	Environmental Pollution	2016
PM2.5	ZFR	Integrative transcriptomic and protein analysis of human bronchial BEAS-2B exposed to seasonal urban particulate matter	Environmental Pollution	2016
PM2.5	TPR	Integrative transcriptomic and protein analysis of human bronchial BEAS-2B exposed to seasonal urban particulate matter	Environmental Pollution	2016
PM2.5	TNFSF15	Integrative transcriptomic and protein analysis of human bronchial BEAS-2B exposed to seasonal urban particulate matter	Environmental Pollution	2016
PM2.5	ESCO1	Integrative transcriptomic and protein analysis of human bronchial BEAS-2B exposed to seasonal urban particulate matter	Environmental Pollution	2016
PM2.5	SSH1	Integrative transcriptomic and protein analysis of human bronchial BEAS-2B exposed to seasonal urban particulate matter	Environmental Pollution	2016
PM2.5	PHLDB2	Integrative transcriptomic and protein analysis of human bronchial BEAS-2B exposed to seasonal urban particulate matter	Environmental Pollution	2016
PM2.5	RBM25	Integrative transcriptomic and protein analysis of human bronchial BEAS-2B exposed to seasonal urban particulate matter	Environmental Pollution	2016
PM2.5	PNISR	Integrative transcriptomic and protein analysis of human bronchial BEAS-2B exposed to seasonal urban particulate matter	Environmental Pollution	2016
PM2.5	EIF4G1	Integrative transcriptomic and protein analysis of human bronchial BEAS-2B exposed to seasonal urban particulate matter	Environmental Pollution	2016
PM2.5	ATRX	Integrative transcriptomic and protein analysis of human bronchial BEAS-2B exposed to seasonal urban particulate matter	Environmental Pollution	2016
PM2.5	WASL	Integrative transcriptomic and protein analysis of human bronchial BEAS-2B exposed to seasonal urban particulate matter	Environmental Pollution	2016
PM2.5	CDC27	Integrative transcriptomic and protein analysis of human bronchial BEAS-2B exposed to seasonal urban particulate matter	Environmental Pollution	2016
PM2.5	THBD	Integrative transcriptomic and protein analysis of human bronchial BEAS-2B exposed to seasonal urban particulate matter	Environmental Pollution	2016
PM2.5	LUC7L3	Integrative transcriptomic and protein analysis of human bronchial BEAS-2B exposed to seasonal urban particulate matter	Environmental Pollution	2016
PM2.5	NRIP1	Integrative transcriptomic and protein analysis of human bronchial BEAS-2B exposed to seasonal urban particulate matter	Environmental Pollution	2016
PM2.5	KLHL24	Integrative transcriptomic and protein analysis of human bronchial BEAS-2B exposed to seasonal urban particulate matter	Environmental Pollution	2016

PM2.5	LTN1	Integrative transcriptomic and protein analysis of human bronchial BEAS-2B exposed to seasonal urban particulate matter	Environmental Pollution	2016
PM2.5	MORF4L2	Integrative transcriptomic and protein analysis of human bronchial BEAS-2B exposed to seasonal urban particulate matter	Environmental Pollution	2016
PM2.5	NASP	Integrative transcriptomic and protein analysis of human bronchial BEAS-2B exposed to seasonal urban particulate matter	Environmental Pollution	2016
PM2.5	YTHDC2	Integrative transcriptomic and protein analysis of human bronchial BEAS-2B exposed to seasonal urban particulate matter	Environmental Pollution	2016
PM2.5	SDR16C5	Integrative transcriptomic and protein analysis of human bronchial BEAS-2B exposed to seasonal urban particulate matter	Environmental Pollution	2016
PM2.5	PIK3R1	Integrative transcriptomic and protein analysis of human bronchial BEAS-2B exposed to seasonal urban particulate matter	Environmental Pollution	2016
PM2.5	DDX17	Integrative transcriptomic and protein analysis of human bronchial BEAS-2B exposed to seasonal urban particulate matter	Environmental Pollution	2016
PM2.5	MPHOSPH8	Integrative transcriptomic and protein analysis of human bronchial BEAS-2B exposed to seasonal urban particulate matter	Environmental Pollution	2016
PM2.5	TMEM106B	Integrative transcriptomic and protein analysis of human bronchial BEAS-2B exposed to seasonal urban particulate matter	Environmental Pollution	2016
PM2.5	CFB	Integrative transcriptomic and protein analysis of human bronchial BEAS-2B exposed to seasonal urban particulate matter	Environmental Pollution	2016
PM2.5	SERPINB3	Integrative transcriptomic and protein analysis of human bronchial BEAS-2B exposed to seasonal urban particulate matter	Environmental Pollution	2016
PM2.5	GART	Integrative transcriptomic and protein analysis of human bronchial BEAS-2B exposed to seasonal urban particulate matter	Environmental Pollution	2016
PM2.5	BTBD7	Integrative transcriptomic and protein analysis of human bronchial BEAS-2B exposed to seasonal urban particulate matter	Environmental Pollution	2016
PM2.5	C1orf116	Integrative transcriptomic and protein analysis of human bronchial BEAS-2B exposed to seasonal urban particulate matter	Environmental Pollution	2016
PM2.5	NCOA3	Integrative transcriptomic and protein analysis of human bronchial BEAS-2B exposed to seasonal urban particulate matter	Environmental Pollution	2016
PM2.5	MYO6	Integrative transcriptomic and protein analysis of human bronchial BEAS-2B exposed to seasonal urban particulate matter	Environmental Pollution	2016
PM2.5	AKR1C1	Integrative transcriptomic and protein analysis of human bronchial BEAS-2B exposed to seasonal urban particulate matter	Environmental Pollution	2016
PM2.5	SON	Integrative transcriptomic and protein analysis of human bronchial BEAS-2B exposed to seasonal urban particulate matter	Environmental Pollution	2016
PM2.5	TXNRD1	Integrative transcriptomic and protein analysis of human bronchial BEAS-2B exposed to seasonal urban particulate matter	Environmental Pollution	2016
PM2.5	ETV5	Integrative transcriptomic and protein analysis of human bronchial BEAS-2B exposed to seasonal urban particulate matter	Environmental Pollution	2016
PM2.5	THRAP3	Integrative transcriptomic and protein analysis of human bronchial BEAS-2B exposed to seasonal urban particulate matter	Environmental Pollution	2016
PM2.5	MOB1A	Integrative transcriptomic and protein analysis of human bronchial BEAS-2B exposed to seasonal urban particulate matter	Environmental Pollution	2016

PM2.5	SGPP2	Integrative transcriptomic and protein analysis of human bronchial BEAS-2B exposed to seasonal urban particulate matter	Environmental Pollution	2016
PM2.5	FERMT1	Integrative transcriptomic and protein analysis of human bronchial BEAS-2B exposed to seasonal urban particulate matter	Environmental Pollution	2016
PM2.5	SAA1	Integrative transcriptomic and protein analysis of human bronchial BEAS-2B exposed to seasonal urban particulate matter	Environmental Pollution	2016
PM2.5	CCDC88A	Integrative transcriptomic and protein analysis of human bronchial BEAS-2B exposed to seasonal urban particulate matter	Environmental Pollution	2016
PM2.5	LINC00886	Integrative transcriptomic and protein analysis of human bronchial BEAS-2B exposed to seasonal urban particulate matter	Environmental Pollution	2016
PM2.5	C20orf197	Integrative transcriptomic and protein analysis of human bronchial BEAS-2B exposed to seasonal urban particulate matter	Environmental Pollution	2016
PM2.5	USP10	Integrative transcriptomic and protein analysis of human bronchial BEAS-2B exposed to seasonal urban particulate matter	Environmental Pollution	2016
PM2.5	NAA15	Integrative transcriptomic and protein analysis of human bronchial BEAS-2B exposed to seasonal urban particulate matter	Environmental Pollution	2016
PM2.5	ANKRD12	Integrative transcriptomic and protein analysis of human bronchial BEAS-2B exposed to seasonal urban particulate matter	Environmental Pollution	2016
PM2.5	RAB7L1	Integrative transcriptomic and protein analysis of human bronchial BEAS-2B exposed to seasonal urban particulate matter	Environmental Pollution	2016
PM2.5	SLC22A4	Integrative transcriptomic and protein analysis of human bronchial BEAS-2B exposed to seasonal urban particulate matter	Environmental Pollution	2016
PM2.5	FLJ10038	Integrative transcriptomic and protein analysis of human bronchial BEAS-2B exposed to seasonal urban particulate matter	Environmental Pollution	2016
PM2.5	EP400	Integrative transcriptomic and protein analysis of human bronchial BEAS-2B exposed to seasonal urban particulate matter	Environmental Pollution	2016
PM2.5	GREM1	Integrative transcriptomic and protein analysis of human bronchial BEAS-2B exposed to seasonal urban particulate matter	Environmental Pollution	2016
PM2.5	SPAG9	Integrative transcriptomic and protein analysis of human bronchial BEAS-2B exposed to seasonal urban particulate matter	Environmental Pollution	2016
PM2.5	C9orf169	Integrative transcriptomic and protein analysis of human bronchial BEAS-2B exposed to seasonal urban particulate matter	Environmental Pollution	2016
PM2.5	BMP2	Integrative transcriptomic and protein analysis of human bronchial BEAS-2B exposed to seasonal urban particulate matter	Environmental Pollution	2016
PM2.5	GNG12	Integrative transcriptomic and protein analysis of human bronchial BEAS-2B exposed to seasonal urban particulate matter	Environmental Pollution	2016
PM2.5	PHF3	Integrative transcriptomic and protein analysis of human bronchial BEAS-2B exposed to seasonal urban particulate matter	Environmental Pollution	2016
PM2.5	DYRK2	Integrative transcriptomic and protein analysis of human bronchial BEAS-2B exposed to seasonal urban particulate matter	Environmental Pollution	2016
PM2.5	SERPINA3	Integrative transcriptomic and protein analysis of human bronchial BEAS-2B exposed to seasonal urban particulate matter	Environmental Pollution	2016
PM2.5	MALAT1	Integrative transcriptomic and protein analysis of human bronchial BEAS-2B exposed to seasonal urban particulate matter	Environmental Pollution	2016

PM2.5	RSF1	Integrative transcriptomic and protein analysis of human bronchial BEAS-2B exposed to seasonal urban particulate matter	Environmental Pollution	2016
PM2.5	AGPAT9	Integrative transcriptomic and protein analysis of human bronchial BEAS-2B exposed to seasonal urban particulate matter	Environmental Pollution	2016
PM2.5	UBN1	Integrative transcriptomic and protein analysis of human bronchial BEAS-2B exposed to seasonal urban particulate matter	Environmental Pollution	2016
PM2.5	RASA2	Integrative transcriptomic and protein analysis of human bronchial BEAS-2B exposed to seasonal urban particulate matter	Environmental Pollution	2016
PM2.5	SMARCC1	Integrative transcriptomic and protein analysis of human bronchial BEAS-2B exposed to seasonal urban particulate matter	Environmental Pollution	2016
PM2.5	ZBED6	Integrative transcriptomic and protein analysis of human bronchial BEAS-2B exposed to seasonal urban particulate matter	Environmental Pollution	2016
PM2.5	EREG	Integrative transcriptomic and protein analysis of human bronchial BEAS-2B exposed to seasonal urban particulate matter	Environmental Pollution	2016
PM2.5	WHSC1L1	Integrative transcriptomic and protein analysis of human bronchial BEAS-2B exposed to seasonal urban particulate matter	Environmental Pollution	2016
PM2.5	BBX	Integrative transcriptomic and protein analysis of human bronchial BEAS-2B exposed to seasonal urban particulate matter	Environmental Pollution	2016
PM2.5	USP1	Integrative transcriptomic and protein analysis of human bronchial BEAS-2B exposed to seasonal urban particulate matter	Environmental Pollution	2016
PM2.5	ZAK	Integrative transcriptomic and protein analysis of human bronchial BEAS-2B exposed to seasonal urban particulate matter	Environmental Pollution	2016
PM2.5	KIAA1033	Integrative transcriptomic and protein analysis of human bronchial BEAS-2B exposed to seasonal urban particulate matter	Environmental Pollution	2016
PM2.5	TMEM156	Integrative transcriptomic and protein analysis of human bronchial BEAS-2B exposed to seasonal urban particulate matter	Environmental Pollution	2016
PM2.5	RIF1	Integrative transcriptomic and protein analysis of human bronchial BEAS-2B exposed to seasonal urban particulate matter	Environmental Pollution	2016
PM2.5	TLDC1	Integrative transcriptomic and protein analysis of human bronchial BEAS-2B exposed to seasonal urban particulate matter	Environmental Pollution	2016
PM2.5	LRP8	Integrative transcriptomic and protein analysis of human bronchial BEAS-2B exposed to seasonal urban particulate matter	Environmental Pollution	2016
PM2.5	TTC3	Integrative transcriptomic and protein analysis of human bronchial BEAS-2B exposed to seasonal urban particulate matter	Environmental Pollution	2016
PM2.5	UQCRC2	Integrative transcriptomic and protein analysis of human bronchial BEAS-2B exposed to seasonal urban particulate matter	Environmental Pollution	2016
PM2.5	KIF21A	Integrative transcriptomic and protein analysis of human bronchial BEAS-2B exposed to seasonal urban particulate matter	Environmental Pollution	2016
PM2.5	LIFR	Integrative transcriptomic and protein analysis of human bronchial BEAS-2B exposed to seasonal urban particulate matter	Environmental Pollution	2016
PM2.5	SRRM2	Integrative transcriptomic and protein analysis of human bronchial BEAS-2B exposed to seasonal urban particulate matter	Environmental Pollution	2016
PM2.5	EWSR1	Integrative transcriptomic and protein analysis of human bronchial BEAS-2B exposed to seasonal urban particulate matter	Environmental Pollution	2016

PM2.5	ILF3	Integrative transcriptomic and protein analysis of human bronchial BEAS-2B exposed to seasonal urban particulate matter	Environmental Pollution	2016
PM2.5	ARHGAP29	Integrative transcriptomic and protein analysis of human bronchial BEAS-2B exposed to seasonal urban particulate matter	Environmental Pollution	2016
PM2.5	LYN	Integrative transcriptomic and protein analysis of human bronchial BEAS-2B exposed to seasonal urban particulate matter	Environmental Pollution	2016
PM2.5	UBXN4	Integrative transcriptomic and protein analysis of human bronchial BEAS-2B exposed to seasonal urban particulate matter	Environmental Pollution	2016
PM2.5	AKR1C2	Integrative transcriptomic and protein analysis of human bronchial BEAS-2B exposed to seasonal urban particulate matter	Environmental Pollution	2016
PM2.5	PLAU	Integrative transcriptomic and protein analysis of human bronchial BEAS-2B exposed to seasonal urban particulate matter	Environmental Pollution	2016
PM2.5	ATXN7L3B	Integrative transcriptomic and protein analysis of human bronchial BEAS-2B exposed to seasonal urban particulate matter	Environmental Pollution	2016
PM2.5	IRAK2	Integrative transcriptomic and protein analysis of human bronchial BEAS-2B exposed to seasonal urban particulate matter	Environmental Pollution	2016
PM2.5	IGF2BP3	Integrative transcriptomic and protein analysis of human bronchial BEAS-2B exposed to seasonal urban particulate matter	Environmental Pollution	2016
PM2.5	TMEM45B	Integrative transcriptomic and protein analysis of human bronchial BEAS-2B exposed to seasonal urban particulate matter	Environmental Pollution	2016
PM2.5	ALDH1A3	Integrative transcriptomic and protein analysis of human bronchial BEAS-2B exposed to seasonal urban particulate matter	Environmental Pollution	2016
PM2.5	CXCL3	Integrative transcriptomic and protein analysis of human bronchial BEAS-2B exposed to seasonal urban particulate matter	Environmental Pollution	2016
PM2.5	NEMF	Integrative transcriptomic and protein analysis of human bronchial BEAS-2B exposed to seasonal urban particulate matter	Environmental Pollution	2016
PM2.5	ZMAT3	Integrative transcriptomic and protein analysis of human bronchial BEAS-2B exposed to seasonal urban particulate matter	Environmental Pollution	2016
PM2.5	HSP90B1	Integrative transcriptomic and protein analysis of human bronchial BEAS-2B exposed to seasonal urban particulate matter	Environmental Pollution	2016
PM2.5	FBXO45	Integrative transcriptomic and protein analysis of human bronchial BEAS-2B exposed to seasonal urban particulate matter	Environmental Pollution	2016
PM2.5	HECTD1	Integrative transcriptomic and protein analysis of human bronchial BEAS-2B exposed to seasonal urban particulate matter	Environmental Pollution	2016
PM2.5	SRXN1	Integrative transcriptomic and protein analysis of human bronchial BEAS-2B exposed to seasonal urban particulate matter	Environmental Pollution	2016
PM2.5	NFKBIA	Integrative transcriptomic and protein analysis of human bronchial BEAS-2B exposed to seasonal urban particulate matter	Environmental Pollution	2016
PM2.5	DYNC1H1	Integrative transcriptomic and protein analysis of human bronchial BEAS-2B exposed to seasonal urban particulate matter	Environmental Pollution	2016
PM2.5	SCAF11	Integrative transcriptomic and protein analysis of human bronchial BEAS-2B exposed to seasonal urban particulate matter	Environmental Pollution	2016
PM2.5	EGR1	Integrative transcriptomic and protein analysis of human bronchial BEAS-2B exposed to seasonal urban particulate matter	Environmental Pollution	2016

PM2.5	PRPF40A	Integrative transcriptomic and protein analysis of human bronchial BEAS-2B exposed to seasonal urban particulate matter	Environmental Pollution	2016
PM2.5	NEK1	Integrative transcriptomic and protein analysis of human bronchial BEAS-2B exposed to seasonal urban particulate matter	Environmental Pollution	2016
PM2.5	ID1	Integrative transcriptomic and protein analysis of human bronchial BEAS-2B exposed to seasonal urban particulate matter	Environmental Pollution	2016
PM2.5	MCTP2	Integrative transcriptomic and protein analysis of human bronchial BEAS-2B exposed to seasonal urban particulate matter	Environmental Pollution	2016
PM2.5	RASEF	Integrative transcriptomic and protein analysis of human bronchial BEAS-2B exposed to seasonal urban particulate matter	Environmental Pollution	2016
PM2.5	LOC100506710	Integrative transcriptomic and protein analysis of human bronchial BEAS-2B exposed to seasonal urban particulate matter	Environmental Pollution	2016
PM2.5	ANKRD17	Integrative transcriptomic and protein analysis of human bronchial BEAS-2B exposed to seasonal urban particulate matter	Environmental Pollution	2016
PM2.5	EIF3C	Integrative transcriptomic and protein analysis of human bronchial BEAS-2B exposed to seasonal urban particulate matter	Environmental Pollution	2016
PM2.5	TMEM154	Integrative transcriptomic and protein analysis of human bronchial BEAS-2B exposed to seasonal urban particulate matter	Environmental Pollution	2016
PM2.5	AQP3	Integrative transcriptomic and protein analysis of human bronchial BEAS-2B exposed to seasonal urban particulate matter	Environmental Pollution	2016
PM2.5	SERPINB6	Integrative transcriptomic and protein analysis of human bronchial BEAS-2B exposed to seasonal urban particulate matter	Environmental Pollution	2016
PM2.5	SLC37A2	Integrative transcriptomic and protein analysis of human bronchial BEAS-2B exposed to seasonal urban particulate matter	Environmental Pollution	2016
PM2.5	KLF5	Integrative transcriptomic and protein analysis of human bronchial BEAS-2B exposed to seasonal urban particulate matter	Environmental Pollution	2016
PM2.5	GJB2	Integrative transcriptomic and protein analysis of human bronchial BEAS-2B exposed to seasonal urban particulate matter	Environmental Pollution	2016
PM2.5	MAP4	Integrative transcriptomic and protein analysis of human bronchial BEAS-2B exposed to seasonal urban particulate matter	Environmental Pollution	2016
PM2.5	MATR3	Integrative transcriptomic and protein analysis of human bronchial BEAS-2B exposed to seasonal urban particulate matter	Environmental Pollution	2016
PM2.5	ICAM1	Integrative transcriptomic and protein analysis of human bronchial BEAS-2B exposed to seasonal urban particulate matter	Environmental Pollution	2016
PM2.5	TBL1XR1	Integrative transcriptomic and protein analysis of human bronchial BEAS-2B exposed to seasonal urban particulate matter	Environmental Pollution	2016
PM2.5	TTC17	Integrative transcriptomic and protein analysis of human bronchial BEAS-2B exposed to seasonal urban particulate matter	Environmental Pollution	2016
PM2.5	IL6	Integrative transcriptomic and protein analysis of human bronchial BEAS-2B exposed to seasonal urban particulate matter	Environmental Pollution	2016
PM2.5	NKTR	Integrative transcriptomic and protein analysis of human bronchial BEAS-2B exposed to seasonal urban particulate matter	Environmental Pollution	2016
PM2.5	KRT14	Integrative transcriptomic and protein analysis of human bronchial BEAS-2B exposed to seasonal urban particulate matter	Environmental Pollution	2016

PM2.5	DUSP6	Integrative transcriptomic and protein analysis of human bronchial BEAS-2B exposed to seasonal urban particulate matter	Environmental Pollution	2016
PM2.5	DCAF13	Integrative transcriptomic and protein analysis of human bronchial BEAS-2B exposed to seasonal urban particulate matter	Environmental Pollution	2016
PM2.5	DDX39B	Integrative transcriptomic and protein analysis of human bronchial BEAS-2B exposed to seasonal urban particulate matter	Environmental Pollution	2016
PM2.5	DDX24	Integrative transcriptomic and protein analysis of human bronchial BEAS-2B exposed to seasonal urban particulate matter	Environmental Pollution	2016
PM2.5	ACBD3	Integrative transcriptomic and protein analysis of human bronchial BEAS-2B exposed to seasonal urban particulate matter	Environmental Pollution	2016
PM2.5	SOWAHC	Integrative transcriptomic and protein analysis of human bronchial BEAS-2B exposed to seasonal urban particulate matter	Environmental Pollution	2016
PM2.5	MLKL	Integrative transcriptomic and protein analysis of human bronchial BEAS-2B exposed to seasonal urban particulate matter	Environmental Pollution	2016
PM2.5	SMG1	Integrative transcriptomic and protein analysis of human bronchial BEAS-2B exposed to seasonal urban particulate matter	Environmental Pollution	2016
PM2.5	ABCG1	Integrative transcriptomic and protein analysis of human bronchial BEAS-2B exposed to seasonal urban particulate matter	Environmental Pollution	2016
PM2.5	SNAI2	Integrative transcriptomic and protein analysis of human bronchial BEAS-2B exposed to seasonal urban particulate matter	Environmental Pollution	2016
PM2.5	SH3KBP1	Integrative transcriptomic and protein analysis of human bronchial BEAS-2B exposed to seasonal urban particulate matter	Environmental Pollution	2016
PM2.5	SOD2	Integrative transcriptomic and protein analysis of human bronchial BEAS-2B exposed to seasonal urban particulate matter	Environmental Pollution	2016
PM2.5	SERPINB4	Integrative transcriptomic and protein analysis of human bronchial BEAS-2B exposed to seasonal urban particulate matter	Environmental Pollution	2016
PM2.5	CDC42EP2	Integrative transcriptomic and protein analysis of human bronchial BEAS-2B exposed to seasonal urban particulate matter	Environmental Pollution	2016
PM2.5	MIDN	Integrative transcriptomic and protein analysis of human bronchial BEAS-2B exposed to seasonal urban particulate matter	Environmental Pollution	2016
PM2.5	GPAM	Integrative transcriptomic and protein analysis of human bronchial BEAS-2B exposed to seasonal urban particulate matter	Environmental Pollution	2016
PM2.5	TFRC	Integrative transcriptomic and protein analysis of human bronchial BEAS-2B exposed to seasonal urban particulate matter	Environmental Pollution	2016
PM2.5	THUMPD3	Integrative transcriptomic and protein analysis of human bronchial BEAS-2B exposed to seasonal urban particulate matter	Environmental Pollution	2016
PM2.5	HCAR3	Integrative transcriptomic and protein analysis of human bronchial BEAS-2B exposed to seasonal urban particulate matter	Environmental Pollution	2016
PM2.5	SLFN5	Integrative transcriptomic and protein analysis of human bronchial BEAS-2B exposed to seasonal urban particulate matter	Environmental Pollution	2016
PM2.5	LNPEP	Integrative transcriptomic and protein analysis of human bronchial BEAS-2B exposed to seasonal urban particulate matter	Environmental Pollution	2016
PM2.5	SMC3	Integrative transcriptomic and protein analysis of human bronchial BEAS-2B exposed to seasonal urban particulate matter	Environmental Pollution	2016

PM2.5	SFXN1	Integrative transcriptomic and protein analysis of human bronchial BEAS-2B exposed to seasonal urban particulate matter	Environmental Pollution	2016
PM2.5	DHRS3	Integrative transcriptomic and protein analysis of human bronchial BEAS-2B exposed to seasonal urban particulate matter	Environmental Pollution	2016
PM2.5	SECISBP2	Integrative transcriptomic and protein analysis of human bronchial BEAS-2B exposed to seasonal urban particulate matter	Environmental Pollution	2016
PM2.5	ZBTB38	Integrative transcriptomic and protein analysis of human bronchial BEAS-2B exposed to seasonal urban particulate matter	Environmental Pollution	2016
PM2.5	SEMA5A	Integrative transcriptomic and protein analysis of human bronchial BEAS-2B exposed to seasonal urban particulate matter	Environmental Pollution	2016
PM2.5	ITPRIPL2	Integrative transcriptomic and protein analysis of human bronchial BEAS-2B exposed to seasonal urban particulate matter	Environmental Pollution	2016
PM2.5	STRAP	Integrative transcriptomic and protein analysis of human bronchial BEAS-2B exposed to seasonal urban particulate matter	Environmental Pollution	2016
PM2.5	KIAA1551	Integrative transcriptomic and protein analysis of human bronchial BEAS-2B exposed to seasonal urban particulate matter	Environmental Pollution	2016
PM2.5	ZKSCAN1	Integrative transcriptomic and protein analysis of human bronchial BEAS-2B exposed to seasonal urban particulate matter	Environmental Pollution	2016
PM2.5	IRF2BPL	Integrative transcriptomic and protein analysis of human bronchial BEAS-2B exposed to seasonal urban particulate matter	Environmental Pollution	2016
PM2.5	UGT1A10	Integrative transcriptomic and protein analysis of human bronchial BEAS-2B exposed to seasonal urban particulate matter	Environmental Pollution	2016
PM2.5	SREK1	Integrative transcriptomic and protein analysis of human bronchial BEAS-2B exposed to seasonal urban particulate matter	Environmental Pollution	2016
PM2.5	BAZ1A	Integrative transcriptomic and protein analysis of human bronchial BEAS-2B exposed to seasonal urban particulate matter	Environmental Pollution	2016
PM2.5	ESF1	Integrative transcriptomic and protein analysis of human bronchial BEAS-2B exposed to seasonal urban particulate matter	Environmental Pollution	2016
PM2.5	LCA5	Integrative transcriptomic and protein analysis of human bronchial BEAS-2B exposed to seasonal urban particulate matter	Environmental Pollution	2016
PM2.5	MAP9	Integrative transcriptomic and protein analysis of human bronchial BEAS-2B exposed to seasonal urban particulate matter	Environmental Pollution	2016
PM2.5	MCM3AP-AS1	Integrative transcriptomic and protein analysis of human bronchial BEAS-2B exposed to seasonal urban particulate matter	Environmental Pollution	2016
PM2.5	NIPBL	Integrative transcriptomic and protein analysis of human bronchial BEAS-2B exposed to seasonal urban particulate matter	Environmental Pollution	2016
PM2.5	IREB2	Integrative transcriptomic and protein analysis of human bronchial BEAS-2B exposed to seasonal urban particulate matter	Environmental Pollution	2016
PM2.5	CASC5	Integrative transcriptomic and protein analysis of human bronchial BEAS-2B exposed to seasonal urban particulate matter	Environmental Pollution	2016
PM2.5	SPIN1	Integrative transcriptomic and protein analysis of human bronchial BEAS-2B exposed to seasonal urban particulate matter	Environmental Pollution	2016
PM2.5	KIF5B	Integrative transcriptomic and protein analysis of human bronchial BEAS-2B exposed to seasonal urban particulate matter	Environmental Pollution	2016

PM2.5	CCDC18	Integrative transcriptomic and protein analysis of human bronchial BEAS-2B exposed to seasonal urban particulate matter	Environmental Pollution	2016
PM2.5	USO1	Integrative transcriptomic and protein analysis of human bronchial BEAS-2B exposed to seasonal urban particulate matter	Environmental Pollution	2016
PM2.5	SF3B1	Integrative transcriptomic and protein analysis of human bronchial BEAS-2B exposed to seasonal urban particulate matter	Environmental Pollution	2016
PM2.5	RHOBTB3	Integrative transcriptomic and protein analysis of human bronchial BEAS-2B exposed to seasonal urban particulate matter	Environmental Pollution	2016
PM2.5	ANKRD36B	Integrative transcriptomic and protein analysis of human bronchial BEAS-2B exposed to seasonal urban particulate matter	Environmental Pollution	2016
PM2.5	HIST1H2BC	Integrative transcriptomic and protein analysis of human bronchial BEAS-2B exposed to seasonal urban particulate matter	Environmental Pollution	2016
PM2.5	ERC1	Integrative transcriptomic and protein analysis of human bronchial BEAS-2B exposed to seasonal urban particulate matter	Environmental Pollution	2016
PM2.5	DENND1B	Integrative transcriptomic and protein analysis of human bronchial BEAS-2B exposed to seasonal urban particulate matter	Environmental Pollution	2016
PM2.5	ASPM	Integrative transcriptomic and protein analysis of human bronchial BEAS-2B exposed to seasonal urban particulate matter	Environmental Pollution	2016
PM2.5	MIS18BP1	Integrative transcriptomic and protein analysis of human bronchial BEAS-2B exposed to seasonal urban particulate matter	Environmental Pollution	2016
PM2.5	BRD2	Integrative transcriptomic and protein analysis of human bronchial BEAS-2B exposed to seasonal urban particulate matter	Environmental Pollution	2016
PM2.5	S100A9	Integrative transcriptomic and protein analysis of human bronchial BEAS-2B exposed to seasonal urban particulate matter	Environmental Pollution	2016
PM2.5	ZNF567	Integrative transcriptomic and protein analysis of human bronchial BEAS-2B exposed to seasonal urban particulate matter	Environmental Pollution	2016
PM2.5	ELF1	Integrative transcriptomic and protein analysis of human bronchial BEAS-2B exposed to seasonal urban particulate matter	Environmental Pollution	2016
PM2.5	SCML1	Integrative transcriptomic and protein analysis of human bronchial BEAS-2B exposed to seasonal urban particulate matter	Environmental Pollution	2016
PM2.5	LIPG	Integrative transcriptomic and protein analysis of human bronchial BEAS-2B exposed to seasonal urban particulate matter	Environmental Pollution	2016
PM2.5	DCAF7	Integrative transcriptomic and protein analysis of human bronchial BEAS-2B exposed to seasonal urban particulate matter	Environmental Pollution	2016
PM2.5	KRT23	Integrative transcriptomic and protein analysis of human bronchial BEAS-2B exposed to seasonal urban particulate matter	Environmental Pollution	2016
PM2.5	MTSS1	Integrative transcriptomic and protein analysis of human bronchial BEAS-2B exposed to seasonal urban particulate matter	Environmental Pollution	2016
PM2.5	FAM117B	Integrative transcriptomic and protein analysis of human bronchial BEAS-2B exposed to seasonal urban particulate matter	Environmental Pollution	2016
PM2.5	USP16	Integrative transcriptomic and protein analysis of human bronchial BEAS-2B exposed to seasonal urban particulate matter	Environmental Pollution	2016
PM2.5	AFAP1-AS1	Integrative transcriptomic and protein analysis of human bronchial BEAS-2B exposed to seasonal urban particulate matter	Environmental Pollution	2016

PM2.5	LOC100506965	Integrative transcriptomic and protein analysis of human bronchial BEAS-2B exposed to seasonal urban particulate matter	Environmental Pollution	2016
PM2.5	IGFBP3	Integrative transcriptomic and protein analysis of human bronchial BEAS-2B exposed to seasonal urban particulate matter	Environmental Pollution	2016
PM2.5	CYGB	Integrative transcriptomic and protein analysis of human bronchial BEAS-2B exposed to seasonal urban particulate matter	Environmental Pollution	2016
PM2.5	ABCC3	Integrative transcriptomic and protein analysis of human bronchial BEAS-2B exposed to seasonal urban particulate matter	Environmental Pollution	2016
PM2.5	SART3	Integrative transcriptomic and protein analysis of human bronchial BEAS-2B exposed to seasonal urban particulate matter	Environmental Pollution	2016
PM2.5	PKN2	Integrative transcriptomic and protein analysis of human bronchial BEAS-2B exposed to seasonal urban particulate matter	Environmental Pollution	2016
PM2.5	SECTM1	Integrative transcriptomic and protein analysis of human bronchial BEAS-2B exposed to seasonal urban particulate matter	Environmental Pollution	2016
PM2.5	FAM120A	Integrative transcriptomic and protein analysis of human bronchial BEAS-2B exposed to seasonal urban particulate matter	Environmental Pollution	2016
PM2.5	CLMP	Integrative transcriptomic and protein analysis of human bronchial BEAS-2B exposed to seasonal urban particulate matter	Environmental Pollution	2016
PM2.5	FOXC1	Integrative transcriptomic and protein analysis of human bronchial BEAS-2B exposed to seasonal urban particulate matter	Environmental Pollution	2016
PM2.5	AASDH	Integrative transcriptomic and protein analysis of human bronchial BEAS-2B exposed to seasonal urban particulate matter	Environmental Pollution	2016
PM2.5	WASF2	Integrative transcriptomic and protein analysis of human bronchial BEAS-2B exposed to seasonal urban particulate matter	Environmental Pollution	2016
PM2.5	FAM83A	Integrative transcriptomic and protein analysis of human bronchial BEAS-2B exposed to seasonal urban particulate matter	Environmental Pollution	2016
PM2.5	AKAP13	Integrative transcriptomic and protein analysis of human bronchial BEAS-2B exposed to seasonal urban particulate matter	Environmental Pollution	2016
PM2.5	BRD4	Integrative transcriptomic and protein analysis of human bronchial BEAS-2B exposed to seasonal urban particulate matter	Environmental Pollution	2016
PM2.5	COIL	Integrative transcriptomic and protein analysis of human bronchial BEAS-2B exposed to seasonal urban particulate matter	Environmental Pollution	2016
PM2.5	IER3	Integrative transcriptomic and protein analysis of human bronchial BEAS-2B exposed to seasonal urban particulate matter	Environmental Pollution	2016
PM2.5	TM4SF1	Integrative transcriptomic and protein analysis of human bronchial BEAS-2B exposed to seasonal urban particulate matter	Environmental Pollution	2016
PM2.5	IL1R1	Integrative transcriptomic and protein analysis of human bronchial BEAS-2B exposed to seasonal urban particulate matter	Environmental Pollution	2016
PM2.5	SERPINB13	Integrative transcriptomic and protein analysis of human bronchial BEAS-2B exposed to seasonal urban particulate matter	Environmental Pollution	2016
PM2.5	CYCS	Integrative transcriptomic and protein analysis of human bronchial BEAS-2B exposed to seasonal urban particulate matter	Environmental Pollution	2016
PM2.5	WSB1	Integrative transcriptomic and protein analysis of human bronchial BEAS-2B exposed to seasonal urban particulate matter	Environmental Pollution	2016

PM2.5	SLCO4A1	Integrative transcriptomic and protein analysis of human bronchial BEAS-2B exposed to seasonal urban particulate matter	Environmental Pollution	2016
PM2.5	SAV1	Integrative transcriptomic and protein analysis of human bronchial BEAS-2B exposed to seasonal urban particulate matter	Environmental Pollution	2016
PM2.5	CCND1	Integrative transcriptomic and protein analysis of human bronchial BEAS-2B exposed to seasonal urban particulate matter	Environmental Pollution	2016
PM2.5	WNK1	Integrative transcriptomic and protein analysis of human bronchial BEAS-2B exposed to seasonal urban particulate matter	Environmental Pollution	2016
PM2.5	SLC25A36	Integrative transcriptomic and protein analysis of human bronchial BEAS-2B exposed to seasonal urban particulate matter	Environmental Pollution	2016
PM2.5	LPP	Integrative transcriptomic and protein analysis of human bronchial BEAS-2B exposed to seasonal urban particulate matter	Environmental Pollution	2016
PM2.5	DCAF16	Integrative transcriptomic and protein analysis of human bronchial BEAS-2B exposed to seasonal urban particulate matter	Environmental Pollution	2016
PM2.5	S100A8	Integrative transcriptomic and protein analysis of human bronchial BEAS-2B exposed to seasonal urban particulate matter	Environmental Pollution	2016
PM2.5	PRCD	Integrative transcriptomic and protein analysis of human bronchial BEAS-2B exposed to seasonal urban particulate matter	Environmental Pollution	2016
PM2.5	JAK1	Integrative transcriptomic and protein analysis of human bronchial BEAS-2B exposed to seasonal urban particulate matter	Environmental Pollution	2016
PM2.5	PSME4	Integrative transcriptomic and protein analysis of human bronchial BEAS-2B exposed to seasonal urban particulate matter	Environmental Pollution	2016
PM2.5	PRRC2C	Integrative transcriptomic and protein analysis of human bronchial BEAS-2B exposed to seasonal urban particulate matter	Environmental Pollution	2016
PM2.5	MCOLN3	Integrative transcriptomic and protein analysis of human bronchial BEAS-2B exposed to seasonal urban particulate matter	Environmental Pollution	2016
PM2.5	THBS2	Integrative transcriptomic and protein analysis of human bronchial BEAS-2B exposed to seasonal urban particulate matter	Environmental Pollution	2016
PM2.5	USP42	Integrative transcriptomic and protein analysis of human bronchial BEAS-2B exposed to seasonal urban particulate matter	Environmental Pollution	2016
PM2.5	ZMYM2	Integrative transcriptomic and protein analysis of human bronchial BEAS-2B exposed to seasonal urban particulate matter	Environmental Pollution	2016
PM2.5	API5	Integrative transcriptomic and protein analysis of human bronchial BEAS-2B exposed to seasonal urban particulate matter	Environmental Pollution	2016
PM2.5	ANGPTL4	Integrative transcriptomic and protein analysis of human bronchial BEAS-2B exposed to seasonal urban particulate matter	Environmental Pollution	2016
PM2.5	AASS	Integrative transcriptomic and protein analysis of human bronchial BEAS-2B exposed to seasonal urban particulate matter	Environmental Pollution	2016
PM2.5	CHI3L1	Integrative transcriptomic and protein analysis of human bronchial BEAS-2B exposed to seasonal urban particulate matter	Environmental Pollution	2016
PM2.5	GALNT1	Integrative transcriptomic and protein analysis of human bronchial BEAS-2B exposed to seasonal urban particulate matter	Environmental Pollution	2016
PM2.5	GNAS	Integrative transcriptomic and protein analysis of human bronchial BEAS-2B exposed to seasonal urban particulate matter	Environmental Pollution	2016

PM2.5	SMARCA4	Integrative transcriptomic and protein analysis of human bronchial BEAS-2B exposed to seasonal urban particulate matter	Environmental Pollution	2016
PM2.5	TM7SF3	Integrative transcriptomic and protein analysis of human bronchial BEAS-2B exposed to seasonal urban particulate matter	Environmental Pollution	2016
PM2.5	SERPINE1	Integrative transcriptomic and protein analysis of human bronchial BEAS-2B exposed to seasonal urban particulate matter	Environmental Pollution	2016
PM2.5	RBAK	Integrative transcriptomic and protein analysis of human bronchial BEAS-2B exposed to seasonal urban particulate matter	Environmental Pollution	2016
PM2.5	CCSER2	Integrative transcriptomic and protein analysis of human bronchial BEAS-2B exposed to seasonal urban particulate matter	Environmental Pollution	2016
PM2.5	ARHGAP18	Integrative transcriptomic and protein analysis of human bronchial BEAS-2B exposed to seasonal urban particulate matter	Environmental Pollution	2016
PM2.5	TRMT13	Integrative transcriptomic and protein analysis of human bronchial BEAS-2B exposed to seasonal urban particulate matter	Environmental Pollution	2016
PM2.5	PROSC	Integrative transcriptomic and protein analysis of human bronchial BEAS-2B exposed to seasonal urban particulate matter	Environmental Pollution	2016
PM2.5	DENR	Integrative transcriptomic and protein analysis of human bronchial BEAS-2B exposed to seasonal urban particulate matter	Environmental Pollution	2016
PM2.5	LDLR	Integrative transcriptomic and protein analysis of human bronchial BEAS-2B exposed to seasonal urban particulate matter	Environmental Pollution	2016
PM2.5	DESI2	Integrative transcriptomic and protein analysis of human bronchial BEAS-2B exposed to seasonal urban particulate matter	Environmental Pollution	2016
PM2.5	ZNF148	Integrative transcriptomic and protein analysis of human bronchial BEAS-2B exposed to seasonal urban particulate matter	Environmental Pollution	2016
PM2.5	SQSTM1	Integrative transcriptomic and protein analysis of human bronchial BEAS-2B exposed to seasonal urban particulate matter	Environmental Pollution	2016
PM2.5	ATP2B4	Integrative transcriptomic and protein analysis of human bronchial BEAS-2B exposed to seasonal urban particulate matter	Environmental Pollution	2016
PM2.5	RNF213	Integrative transcriptomic and protein analysis of human bronchial BEAS-2B exposed to seasonal urban particulate matter	Environmental Pollution	2016
PM2.5	PTK6	Integrative transcriptomic and protein analysis of human bronchial BEAS-2B exposed to seasonal urban particulate matter	Environmental Pollution	2016
PM2.5	ANXA10	Integrative transcriptomic and protein analysis of human bronchial BEAS-2B exposed to seasonal urban particulate matter	Environmental Pollution	2016
PM2.5	GATAD1	Integrative transcriptomic and protein analysis of human bronchial BEAS-2B exposed to seasonal urban particulate matter	Environmental Pollution	2016
PM2.5	CCNG2	Integrative transcriptomic and protein analysis of human bronchial BEAS-2B exposed to seasonal urban particulate matter	Environmental Pollution	2016
PM2.5	G0S2	Integrative transcriptomic and protein analysis of human bronchial BEAS-2B exposed to seasonal urban particulate matter	Environmental Pollution	2016
PM2.5	ABLIM1	Integrative transcriptomic and protein analysis of human bronchial BEAS-2B exposed to seasonal urban particulate matter	Environmental Pollution	2016
PM2.5	FGFBP1	Integrative transcriptomic and protein analysis of human bronchial BEAS-2B exposed to seasonal urban particulate matter	Environmental Pollution	2016

PM2.5	TOP2A	Integrative transcriptomic and protein analysis of human bronchial BEAS-2B exposed to seasonal urban particulate matter	Environmental Pollution	2016
PM2.5	GCLC	Integrative transcriptomic and protein analysis of human bronchial BEAS-2B exposed to seasonal urban particulate matter	Environmental Pollution	2016
PM2.5	EFNB2	Integrative transcriptomic and protein analysis of human bronchial BEAS-2B exposed to seasonal urban particulate matter	Environmental Pollution	2016
PM2.5	GBP1	Integrative transcriptomic and protein analysis of human bronchial BEAS-2B exposed to seasonal urban particulate matter	Environmental Pollution	2016
PM2.5	FUCA1	Integrative transcriptomic and protein analysis of human bronchial BEAS-2B exposed to seasonal urban particulate matter	Environmental Pollution	2016
PM2.5	SPEN	Integrative transcriptomic and protein analysis of human bronchial BEAS-2B exposed to seasonal urban particulate matter	Environmental Pollution	2016
PM2.5	LSM14B	Integrative transcriptomic and protein analysis of human bronchial BEAS-2B exposed to seasonal urban particulate matter	Environmental Pollution	2016
PM2.5	CHD9	Integrative transcriptomic and protein analysis of human bronchial BEAS-2B exposed to seasonal urban particulate matter	Environmental Pollution	2016
PM2.5	EDEM3	Integrative transcriptomic and protein analysis of human bronchial BEAS-2B exposed to seasonal urban particulate matter	Environmental Pollution	2016
PM2.5	CCAR1	Integrative transcriptomic and protein analysis of human bronchial BEAS-2B exposed to seasonal urban particulate matter	Environmental Pollution	2016
PM2.5	SMC4	Integrative transcriptomic and protein analysis of human bronchial BEAS-2B exposed to seasonal urban particulate matter	Environmental Pollution	2016
PM2.5	HSPA4	Integrative transcriptomic and protein analysis of human bronchial BEAS-2B exposed to seasonal urban particulate matter	Environmental Pollution	2016
PM2.5	STK4	Integrative transcriptomic and protein analysis of human bronchial BEAS-2B exposed to seasonal urban particulate matter	Environmental Pollution	2016
PM2.5	CBL	Integrative transcriptomic and protein analysis of human bronchial BEAS-2B exposed to seasonal urban particulate matter	Environmental Pollution	2016
PM2.5	ITGA2	Integrative transcriptomic and protein analysis of human bronchial BEAS-2B exposed to seasonal urban particulate matter	Environmental Pollution	2016
PM2.5	WIPI2	Integrative transcriptomic and protein analysis of human bronchial BEAS-2B exposed to seasonal urban particulate matter	Environmental Pollution	2016
PM2.5	HIP1	Integrative transcriptomic and protein analysis of human bronchial BEAS-2B exposed to seasonal urban particulate matter	Environmental Pollution	2016
PM2.5	SLC38A1	Integrative transcriptomic and protein analysis of human bronchial BEAS-2B exposed to seasonal urban particulate matter	Environmental Pollution	2016
PM2.5	PHACTR2	Integrative transcriptomic and protein analysis of human bronchial BEAS-2B exposed to seasonal urban particulate matter	Environmental Pollution	2016
PM2.5	PTER	Integrative transcriptomic and protein analysis of human bronchial BEAS-2B exposed to seasonal urban particulate matter	Environmental Pollution	2016
PM2.5	HBEGF	Integrative transcriptomic and protein analysis of human bronchial BEAS-2B exposed to seasonal urban particulate matter	Environmental Pollution	2016
PM2.5	GLG1	Integrative transcriptomic and protein analysis of human bronchial BEAS-2B exposed to seasonal urban particulate matter	Environmental Pollution	2016

PM2.5	ZBTB43	Integrative transcriptomic and protein analysis of human bronchial BEAS-2B exposed to seasonal urban particulate matter	Environmental Pollution	2016
PM2.5	IL6ST	Integrative transcriptomic and protein analysis of human bronchial BEAS-2B exposed to seasonal urban particulate matter	Environmental Pollution	2016
PM2.5	PDP1	Integrative transcriptomic and protein analysis of human bronchial BEAS-2B exposed to seasonal urban particulate matter	Environmental Pollution	2016
PM2.5	INHBA	Integrative transcriptomic and protein analysis of human bronchial BEAS-2B exposed to seasonal urban particulate matter	Environmental Pollution	2016
PM2.5	FOXQ1	Integrative transcriptomic and protein analysis of human bronchial BEAS-2B exposed to seasonal urban particulate matter	Environmental Pollution	2016
PM2.5	HIST2H2BE	Integrative transcriptomic and protein analysis of human bronchial BEAS-2B exposed to seasonal urban particulate matter	Environmental Pollution	2016
PM2.5	KMT2A	Integrative transcriptomic and protein analysis of human bronchial BEAS-2B exposed to seasonal urban particulate matter	Environmental Pollution	2016
PM2.5	HS3ST1	Integrative transcriptomic and protein analysis of human bronchial BEAS-2B exposed to seasonal urban particulate matter	Environmental Pollution	2016
PM2.5	COX20	Integrative transcriptomic and protein analysis of human bronchial BEAS-2B exposed to seasonal urban particulate matter	Environmental Pollution	2016
PM2.5	CSPP1	Integrative transcriptomic and protein analysis of human bronchial BEAS-2B exposed to seasonal urban particulate matter	Environmental Pollution	2016
PM2.5	TFPI2	Integrative transcriptomic and protein analysis of human bronchial BEAS-2B exposed to seasonal urban particulate matter	Environmental Pollution	2016
PM2.5	CTSL1P8	Integrative transcriptomic and protein analysis of human bronchial BEAS-2B exposed to seasonal urban particulate matter	Environmental Pollution	2016
PM2.5	HADHA	Integrative transcriptomic and protein analysis of human bronchial BEAS-2B exposed to seasonal urban particulate matter	Environmental Pollution	2016
PM2.5	PDGFA	Integrative transcriptomic and protein analysis of human bronchial BEAS-2B exposed to seasonal urban particulate matter	Environmental Pollution	2016
PM2.5	FTH1	Integrative transcriptomic and protein analysis of human bronchial BEAS-2B exposed to seasonal urban particulate matter	Environmental Pollution	2016
PM2.5	MTDH	Integrative transcriptomic and protein analysis of human bronchial BEAS-2B exposed to seasonal urban particulate matter	Environmental Pollution	2016
PM2.5	CLIP1	Integrative transcriptomic and protein analysis of human bronchial BEAS-2B exposed to seasonal urban particulate matter	Environmental Pollution	2016
PM2.5	FAM60A	Integrative transcriptomic and protein analysis of human bronchial BEAS-2B exposed to seasonal urban particulate matter	Environmental Pollution	2016
PM2.5	TBL1X	Integrative transcriptomic and protein analysis of human bronchial BEAS-2B exposed to seasonal urban particulate matter	Environmental Pollution	2016
PM2.5	SLX4IP	Integrative transcriptomic and protein analysis of human bronchial BEAS-2B exposed to seasonal urban particulate matter	Environmental Pollution	2016
PM2.5	KLF9	Integrative transcriptomic and protein analysis of human bronchial BEAS-2B exposed to seasonal urban particulate matter	Environmental Pollution	2016
PM2.5	CCNC	Integrative transcriptomic and protein analysis of human bronchial BEAS-2B exposed to seasonal urban particulate matter	Environmental Pollution	2016

PM2.5	SEC63	Integrative transcriptomic and protein analysis of human bronchial BEAS-2B exposed to seasonal urban particulate matter	Environmental Pollution	2016
PM2.5	SPATS2	Integrative transcriptomic and protein analysis of human bronchial BEAS-2B exposed to seasonal urban particulate matter	Environmental Pollution	2016
PM2.5	PAPD5	Integrative transcriptomic and protein analysis of human bronchial BEAS-2B exposed to seasonal urban particulate matter	Environmental Pollution	2016
PM2.5	THOC2	Integrative transcriptomic and protein analysis of human bronchial BEAS-2B exposed to seasonal urban particulate matter	Environmental Pollution	2016
PM2.5	SETD2	Integrative transcriptomic and protein analysis of human bronchial BEAS-2B exposed to seasonal urban particulate matter	Environmental Pollution	2016
PM2.5	TCERG1	Integrative transcriptomic and protein analysis of human bronchial BEAS-2B exposed to seasonal urban particulate matter	Environmental Pollution	2016
PM2.5	KDM4A	Integrative transcriptomic and protein analysis of human bronchial BEAS-2B exposed to seasonal urban particulate matter	Environmental Pollution	2016
PM2.5	LOC100506325	Integrative transcriptomic and protein analysis of human bronchial BEAS-2B exposed to seasonal urban particulate matter	Environmental Pollution	2016
PM2.5	METTL15	Integrative transcriptomic and protein analysis of human bronchial BEAS-2B exposed to seasonal urban particulate matter	Environmental Pollution	2016
PM2.5	CHD4	Integrative transcriptomic and protein analysis of human bronchial BEAS-2B exposed to seasonal urban particulate matter	Environmental Pollution	2016
PM2.5	TAOK1	Integrative transcriptomic and protein analysis of human bronchial BEAS-2B exposed to seasonal urban particulate matter	Environmental Pollution	2016
PM2.5	RNF125	Integrative transcriptomic and protein analysis of human bronchial BEAS-2B exposed to seasonal urban particulate matter	Environmental Pollution	2016
PM2.5	XIAP	Integrative transcriptomic and protein analysis of human bronchial BEAS-2B exposed to seasonal urban particulate matter	Environmental Pollution	2016
PM2.5	PCLO	Integrative transcriptomic and protein analysis of human bronchial BEAS-2B exposed to seasonal urban particulate matter	Environmental Pollution	2016
PM2.5	SUPT16H	Integrative transcriptomic and protein analysis of human bronchial BEAS-2B exposed to seasonal urban particulate matter	Environmental Pollution	2016
PM2.5	G2E3	Integrative transcriptomic and protein analysis of human bronchial BEAS-2B exposed to seasonal urban particulate matter	Environmental Pollution	2016
PM2.5	BCLAF1	Integrative transcriptomic and protein analysis of human bronchial BEAS-2B exposed to seasonal urban particulate matter	Environmental Pollution	2016
PM2.5	TCF25	Integrative transcriptomic and protein analysis of human bronchial BEAS-2B exposed to seasonal urban particulate matter	Environmental Pollution	2016
PM2.5	PTPLB	Integrative transcriptomic and protein analysis of human bronchial BEAS-2B exposed to seasonal urban particulate matter	Environmental Pollution	2016
PM2.5	SRPK2	Integrative transcriptomic and protein analysis of human bronchial BEAS-2B exposed to seasonal urban particulate matter	Environmental Pollution	2016
PM2.5	IFI16	Integrative transcriptomic and protein analysis of human bronchial BEAS-2B exposed to seasonal urban particulate matter	Environmental Pollution	2016
PM2.5	THAP6	Integrative transcriptomic and protein analysis of human bronchial BEAS-2B exposed to seasonal urban particulate matter	Environmental Pollution	2016

PM2.5	PPFIBP1	Integrative transcriptomic and protein analysis of human bronchial BEAS-2B exposed to seasonal urban particulate matter	Environmental Pollution	2016
PM2.5	ZNF12	Integrative transcriptomic and protein analysis of human bronchial BEAS-2B exposed to seasonal urban particulate matter	Environmental Pollution	2016
PM2.5	PTP4A2	Integrative transcriptomic and protein analysis of human bronchial BEAS-2B exposed to seasonal urban particulate matter	Environmental Pollution	2016
PM2.5	PPP1R10	Integrative transcriptomic and protein analysis of human bronchial BEAS-2B exposed to seasonal urban particulate matter	Environmental Pollution	2016
PM2.5	TNFRSF6B	Integrative transcriptomic and protein analysis of human bronchial BEAS-2B exposed to seasonal urban particulate matter	Environmental Pollution	2016
PM2.5	AK4	Integrative transcriptomic and protein analysis of human bronchial BEAS-2B exposed to seasonal urban particulate matter	Environmental Pollution	2016
PM2.5	ETV6	Integrative transcriptomic and protein analysis of human bronchial BEAS-2B exposed to seasonal urban particulate matter	Environmental Pollution	2016
PM2.5	MTUS1	Integrative transcriptomic and protein analysis of human bronchial BEAS-2B exposed to seasonal urban particulate matter	Environmental Pollution	2016
PM2.5	CSNK1G1	Integrative transcriptomic and protein analysis of human bronchial BEAS-2B exposed to seasonal urban particulate matter	Environmental Pollution	2016
PM2.5	SPG7	Integrative transcriptomic and protein analysis of human bronchial BEAS-2B exposed to seasonal urban particulate matter	Environmental Pollution	2016
PM2.5	NFIC	Integrative transcriptomic and protein analysis of human bronchial BEAS-2B exposed to seasonal urban particulate matter	Environmental Pollution	2016
PM2.5	ST6GALNAC5	Integrative transcriptomic and protein analysis of human bronchial BEAS-2B exposed to seasonal urban particulate matter	Environmental Pollution	2016
PM2.5	DHRS2	Integrative transcriptomic and protein analysis of human bronchial BEAS-2B exposed to seasonal urban particulate matter	Environmental Pollution	2016
PM2.5	TSPAN1	Integrative transcriptomic and protein analysis of human bronchial BEAS-2B exposed to seasonal urban particulate matter	Environmental Pollution	2016
PM2.5	ARHGAP26	Integrative transcriptomic and protein analysis of human bronchial BEAS-2B exposed to seasonal urban particulate matter	Environmental Pollution	2016
PM2.5	DKK1	Integrative transcriptomic and protein analysis of human bronchial BEAS-2B exposed to seasonal urban particulate matter	Environmental Pollution	2016
PM2.5	ANKRD2	Integrative transcriptomic and protein analysis of human bronchial BEAS-2B exposed to seasonal urban particulate matter	Environmental Pollution	2016
PM2.5	ZFHX4-AS1	Integrative transcriptomic and protein analysis of human bronchial BEAS-2B exposed to seasonal urban particulate matter	Environmental Pollution	2016
PM2.5	SCEL	Integrative transcriptomic and protein analysis of human bronchial BEAS-2B exposed to seasonal urban particulate matter	Environmental Pollution	2016
PM2.5	ABI3BP	Integrative transcriptomic and protein analysis of human bronchial BEAS-2B exposed to seasonal urban particulate matter	Environmental Pollution	2016
PM2.5	SULT1E1	Integrative transcriptomic and protein analysis of human bronchial BEAS-2B exposed to seasonal urban particulate matter	Environmental Pollution	2016
PM2.5	PEG10	Integrative transcriptomic and protein analysis of human bronchial BEAS-2B exposed to seasonal urban particulate matter	Environmental Pollution	2016

PM2.5	FILIP1L	Integrative transcriptomic and protein analysis of human bronchial BEAS-2B exposed to seasonal urban particulate matter	Environmental Pollution	2016
PM2.5	NSAP11	Integrative transcriptomic and protein analysis of human bronchial BEAS-2B exposed to seasonal urban particulate matter	Environmental Pollution	2016
PM2.5	IFIT1	Integrative transcriptomic and protein analysis of human bronchial BEAS-2B exposed to seasonal urban particulate matter	Environmental Pollution	2016
PM2.5	IFI44	Integrative transcriptomic and protein analysis of human bronchial BEAS-2B exposed to seasonal urban particulate matter	Environmental Pollution	2016
PM2.5	JUP	The Use of Protein-Protein Interactions for the Analysis of the Associations between PM2.5 and Some Diseases	Biomed Res Int.	2016
PM2.5	PTGS2	The Use of Protein-Protein Interactions for the Analysis of the Associations between PM2.5 and Some Diseases	Biomed Res Int.	2016
PM2.5	CYP1A1	The Use of Protein-Protein Interactions for the Analysis of the Associations between PM2.5 and Some Diseases	Biomed Res Int.	2016
PM2.5	PPARGC1A	The Use of Protein-Protein Interactions for the Analysis of the Associations between PM2.5 and Some Diseases	Biomed Res Int.	2016
PM2.5	CCL2	The Use of Protein-Protein Interactions for the Analysis of the Associations between PM2.5 and Some Diseases	Biomed Res Int.	2016
PM2.5	ID1	The Use of Protein-Protein Interactions for the Analysis of the Associations between PM2.5 and Some Diseases	Biomed Res Int.	2016
PM2.5	SGK1	The Use of Protein-Protein Interactions for the Analysis of the Associations between PM2.5 and Some Diseases	Biomed Res Int.	2016
PM2.5	FBN2	The Use of Protein-Protein Interactions for the Analysis of the Associations between PM2.5 and Some Diseases	Biomed Res Int.	2016
PM2.5	ID2	The Use of Protein-Protein Interactions for the Analysis of the Associations between PM2.5 and Some Diseases	Biomed Res Int.	2016
PM2.5	PROC	The Use of Protein-Protein Interactions for the Analysis of the Associations between PM2.5 and Some Diseases	Biomed Res Int.	2016
PM2.5	FGG	The Use of Protein-Protein Interactions for the Analysis of the Associations between PM2.5 and Some Diseases	Biomed Res Int.	2016
PM2.5	BMP6	The Use of Protein-Protein Interactions for the Analysis of the Associations between PM2.5 and Some Diseases	Biomed Res Int.	2016
PM2.5	EGR1	The Use of Protein-Protein Interactions for the Analysis of the Associations between PM2.5 and Some Diseases	Biomed Res Int.	2016
PM2.5	IL6ST	The Use of Protein-Protein Interactions for the Analysis of the Associations between PM2.5 and Some Diseases	Biomed Res Int.	2016
PM2.5	STAT4	The Use of Protein-Protein Interactions for the Analysis of the Associations between PM2.5 and Some Diseases	Biomed Res Int.	2016
PM2.5	SOX9	The Use of Protein-Protein Interactions for the Analysis of the Associations between PM2.5 and Some Diseases	Biomed Res Int.	2016
PM2.5	TLR5	Health Risk Assessment for Air Pollutants: Alterations in Lung and Cardiac Gene Expression in Mice Exposed to Milano Winter Fine Particulate Matter (PM2.5)	PLOS One	2014
PM2.5	TLR3	Health Risk Assessment for Air Pollutants: Alterations in Lung and Cardiac Gene Expression in Mice Exposed to Milano Winter Fine Particulate Matter (PM2.5)	PLOS One	2014

PM2.5	TIRAP	Health Risk Assessment for Air Pollutants: Alterations in Lung and Cardiac Gene Expression in Mice Exposed to Milano Winter Fine Particulate Matter (PM2.5)	PLOS One	2014
PM2.5	JUN	Health Risk Assessment for Air Pollutants: Alterations in Lung and Cardiac Gene Expression in Mice Exposed to Milano Winter Fine Particulate Matter (PM2.5)	PLOS One	2014
PM2.5	CHUK/IKKA	Health Risk Assessment for Air Pollutants: Alterations in Lung and Cardiac Gene Expression in Mice Exposed to Milano Winter Fine Particulate Matter (PM2.5)	PLOS One	2014
PM2.5	CASP8	Health Risk Assessment for Air Pollutants: Alterations in Lung and Cardiac Gene Expression in Mice Exposed to Milano Winter Fine Particulate Matter (PM2.5)	PLOS One	2014
PM2.5	UBE2N	Health Risk Assessment for Air Pollutants: Alterations in Lung and Cardiac Gene Expression in Mice Exposed to Milano Winter Fine Particulate Matter (PM2.5)	PLOS One	2014
PM2.5	ET-1	Health Risk Assessment for Air Pollutants: Alterations in Lung and Cardiac Gene Expression in Mice Exposed to Milano Winter Fine Particulate Matter (PM2.5)	PLOS One	2014
PM2.5	HSP70	Health Risk Assessment for Air Pollutants: Alterations in Lung and Cardiac Gene Expression in Mice Exposed to Milano Winter Fine Particulate Matter (PM2.5)	PLOS One	2014
PM2.5	OGG1/2	Health Risk Assessment for Air Pollutants: Alterations in Lung and Cardiac Gene Expression in Mice Exposed to Milano Winter Fine Particulate Matter (PM2.5)	PLOS One	2014
PM2.5	HO-1	Health Risk Assessment for Air Pollutants: Alterations in Lung and Cardiac Gene Expression in Mice Exposed to Milano Winter Fine Particulate Matter (PM2.5)	PLOS One	2014
PM2.5	MPO	Health Risk Assessment for Air Pollutants: Alterations in Lung and Cardiac Gene Expression in Mice Exposed to Milano Winter Fine Particulate Matter (PM2.5)	PLOS One	2014
PM2.5	CASP8-P18	Health Risk Assessment for Air Pollutants: Alterations in Lung and Cardiac Gene Expression in Mice Exposed to Milano Winter Fine Particulate Matter (PM2.5)	PLOS One	2014
PM2.5	CASP3-P17	Health Risk Assessment for Air Pollutants: Alterations in Lung and Cardiac Gene Expression in Mice Exposed to Milano Winter Fine Particulate Matter (PM2.5)	PLOS One	2014
PM2.5	PH3/H3	Health Risk Assessment for Air Pollutants: Alterations in Lung and Cardiac Gene Expression in Mice Exposed to Milano Winter Fine Particulate Matter (PM2.5)	PLOS One	2014
PM2.5	PAK2	Global gene expression profiling of human bronchial epithelial cells exposed to airborne fine particulate matter collected from Wuhan, China	Toxicology letters	2014
PM2.5	ARPC2	Global gene expression profiling of human bronchial epithelial cells exposed to airborne fine particulate matter collected from Wuhan, China	Toxicology letters	2014
PM2.5	FGF2	Global gene expression profiling of human bronchial epithelial cells exposed to airborne fine particulate matter collected from Wuhan, China	Toxicology letters	2014
PM2.5	F2R	Global gene expression profiling of human bronchial epithelial cells exposed to airborne fine particulate matter collected from Wuhan, China	Toxicology letters	2014
PM2.5	ITGA1	Global gene expression profiling of human bronchial epithelial cells exposed to airborne fine particulate matter collected from Wuhan, China	Toxicology letters	2014

PM2.5	NRAS	Global gene expression profiling of human bronchial epithelial cells exposed to airborne fine particulate matter collected from Wuhan, China	Toxicology letters	2014
PM2.5	SSH2	Global gene expression profiling of human bronchial epithelial cells exposed to airborne fine particulate matter collected from Wuhan, China	Toxicology letters	2014
PM2.5	PAK4	Global gene expression profiling of human bronchial epithelial cells exposed to airborne fine particulate matter collected from Wuhan, China	Toxicology letters	2014
PM2.5	ARPC5L	Global gene expression profiling of human bronchial epithelial cells exposed to airborne fine particulate matter collected from Wuhan, China	Toxicology letters	2014
PM2.5	PAK6	Global gene expression profiling of human bronchial epithelial cells exposed to airborne fine particulate matter collected from Wuhan, China	Toxicology letters	2014
PM2.5	EGFR	Global gene expression profiling of human bronchial epithelial cells exposed to airborne fine particulate matter collected from Wuhan, China	Toxicology letters	2014
PM2.5	SCIN	Global gene expression profiling of human bronchial epithelial cells exposed to airborne fine particulate matter collected from Wuhan, China	Toxicology letters	2014
PM2.5	ORC5L	Global gene expression profiling of human bronchial epithelial cells exposed to airborne fine particulate matter collected from Wuhan, China	Toxicology letters	2014
PM2.5	CHEK2	Global gene expression profiling of human bronchial epithelial cells exposed to airborne fine particulate matter collected from Wuhan, China	Toxicology letters	2014
PM2.5	ANAPC5	Global gene expression profiling of human bronchial epithelial cells exposed to airborne fine particulate matter collected from Wuhan, China	Toxicology letters	2014
PM2.5	ANAPC1	Global gene expression profiling of human bronchial epithelial cells exposed to airborne fine particulate matter collected from Wuhan, China	Toxicology letters	2014
PM2.5	PTTG2	Global gene expression profiling of human bronchial epithelial cells exposed to airborne fine particulate matter collected from Wuhan, China	Toxicology letters	2014
PM2.5	CDKN2C	Global gene expression profiling of human bronchial epithelial cells exposed to airborne fine particulate matter collected from Wuhan, China	Toxicology letters	2014
PM2.5	SESN1	Global gene expression profiling of human bronchial epithelial cells exposed to airborne fine particulate matter collected from Wuhan, China	Toxicology letters	2014
PM2.5	PMAIP1	Global gene expression profiling of human bronchial epithelial cells exposed to airborne fine particulate matter collected from Wuhan, China	Toxicology letters	2014
PM2.5	CD82	Global gene expression profiling of human bronchial epithelial cells exposed to airborne fine particulate matter collected from Wuhan, China	Toxicology letters	2014
PM2.5	LRDD	Global gene expression profiling of human bronchial epithelial cells exposed to airborne fine particulate matter collected from Wuhan, China	Toxicology letters	2014
PM2.5	BCL2	Global gene expression profiling of human bronchial epithelial cells exposed to airborne fine particulate matter collected from Wuhan, China	Toxicology letters	2014
PM2.5	PARD6B	Global gene expression profiling of human bronchial epithelial cells exposed to airborne fine particulate matter collected from Wuhan, China	Toxicology letters	2014
PM2.5	MAGI3	Global gene expression profiling of human bronchial epithelial cells exposed to airborne fine particulate matter collected from Wuhan, China	Toxicology letters	2014
PM2.5	MPDZ	Global gene expression profiling of human bronchial epithelial cells exposed to airborne fine particulate matter collected from Wuhan, China	Toxicology letters	2014
PM2.5	CGN	Global gene expression profiling of human bronchial epithelial cells exposed to airborne fine particulate matter collected from Wuhan, China	Toxicology letters	2014

PM2.5	RAB13	Global gene expression profiling of human bronchial epithelial cells exposed to airborne fine particulate matter collected from Wuhan, China	Toxicology letters	2014
PM2.5	CASP10	Global gene expression profiling of human bronchial epithelial cells exposed to airborne fine particulate matter collected from Wuhan, China	Toxicology letters	2014
PM2.5	TRAF2	Global gene expression profiling of human bronchial epithelial cells exposed to airborne fine particulate matter collected from Wuhan, China	Toxicology letters	2014
PM2.5	CASP6	Global gene expression profiling of human bronchial epithelial cells exposed to airborne fine particulate matter collected from Wuhan, China	Toxicology letters	2014
PM2.5	ADCY6	Global gene expression profiling of human bronchial epithelial cells exposed to airborne fine particulate matter collected from Wuhan, China	Toxicology letters	2014
PM2.5	TUBB3	Global gene expression profiling of human bronchial epithelial cells exposed to airborne fine particulate matter collected from Wuhan, China	Toxicology letters	2014
PM2.5	RASGRP2	Global gene expression profiling of human bronchial epithelial cells exposed to airborne fine particulate matter collected from Wuhan, China	Toxicology letters	2014
PM2.5	MAP3K5	Global gene expression profiling of human bronchial epithelial cells exposed to airborne fine particulate matter collected from Wuhan, China	Toxicology letters	2014
PM2.5	BDNF	Global gene expression profiling of human bronchial epithelial cells exposed to airborne fine particulate matter collected from Wuhan, China	Toxicology letters	2014
PM2.5	HSPA8	Global gene expression profiling of human bronchial epithelial cells exposed to airborne fine particulate matter collected from Wuhan, China	Toxicology letters	2014
PM2.5	HSPB1	Global gene expression profiling of human bronchial epithelial cells exposed to airborne fine particulate matter collected from Wuhan, China	Toxicology letters	2014
PM2.5	DUSP16	Global gene expression profiling of human bronchial epithelial cells exposed to airborne fine particulate matter collected from Wuhan, China	Toxicology letters	2014
PM2.5	ABCB10	Global gene expression profiling of human bronchial epithelial cells exposed to airborne fine particulate matter collected from Wuhan, China	Toxicology letters	2014
PM2.5	ABCD3	Global gene expression profiling of human bronchial epithelial cells exposed to airborne fine particulate matter collected from Wuhan, China	Toxicology letters	2014
PM2.5	ABCC5	Global gene expression profiling of human bronchial epithelial cells exposed to airborne fine particulate matter collected from Wuhan, China	Toxicology letters	2014
PM2.5	TNFSF12	Global gene expression profiling of human bronchial epithelial cells exposed to airborne fine particulate matter collected from Wuhan, China	Toxicology letters	2014
PM2.5	INHBE	Global gene expression profiling of human bronchial epithelial cells exposed to airborne fine particulate matter collected from Wuhan, China	Toxicology letters	2014
PM2.5	XCR1	Global gene expression profiling of human bronchial epithelial cells exposed to airborne fine particulate matter collected from Wuhan, China	Toxicology letters	2014
PM2.5	IL10RB	Global gene expression profiling of human bronchial epithelial cells exposed to airborne fine particulate matter collected from Wuhan, China	Toxicology letters	2014
PM2.5	CXCL16	Global gene expression profiling of human bronchial epithelial cells exposed to airborne fine particulate matter collected from Wuhan, China	Toxicology letters	2014
PM2.5	RPS6	Global gene expression profiling of human bronchial epithelial cells exposed to airborne fine particulate matter collected from Wuhan, China	Toxicology letters	2014
PM2.5	LOC388720	Global gene expression profiling of human bronchial epithelial cells exposed to airborne fine particulate matter collected from Wuhan, China	Toxicology letters	2014

PM2.5	RPS27A	Global gene expression profiling of human bronchial epithelial cells exposed to airborne fine particulate matter collected from Wuhan, China	Toxicology letters	2014
PM2.5	RPL26	Global gene expression profiling of human bronchial epithelial cells exposed to airborne fine particulate matter collected from Wuhan, China	Toxicology letters	2014
PM2.5	RPL21	Global gene expression profiling of human bronchial epithelial cells exposed to airborne fine particulate matter collected from Wuhan, China	Toxicology letters	2014
PM2.5	RPL12	Global gene expression profiling of human bronchial epithelial cells exposed to airborne fine particulate matter collected from Wuhan, China	Toxicology letters	2014
PM2.5	RPL29	Global gene expression profiling of human bronchial epithelial cells exposed to airborne fine particulate matter collected from Wuhan, China	Toxicology letters	2014
PM2.5	LOC283412	Global gene expression profiling of human bronchial epithelial cells exposed to airborne fine particulate matter collected from Wuhan, China	Toxicology letters	2014
PM2.5	RPSA	Global gene expression profiling of human bronchial epithelial cells exposed to airborne fine particulate matter collected from Wuhan, China	Toxicology letters	2014
PM2.5	RPS10	Global gene expression profiling of human bronchial epithelial cells exposed to airborne fine particulate matter collected from Wuhan, China	Toxicology letters	2014
PM2.5	RPS23	Global gene expression profiling of human bronchial epithelial cells exposed to airborne fine particulate matter collected from Wuhan, China	Toxicology letters	2014
PM2.5	RPL35	Global gene expression profiling of human bronchial epithelial cells exposed to airborne fine particulate matter collected from Wuhan, China	Toxicology letters	2014
PM2.5	LOC440737	Global gene expression profiling of human bronchial epithelial cells exposed to airborne fine particulate matter collected from Wuhan, China	Toxicology letters	2014
PM2.5	RPS3	Global gene expression profiling of human bronchial epithelial cells exposed to airborne fine particulate matter collected from Wuhan, China	Toxicology letters	2014
PM2.5	RPS26	Global gene expression profiling of human bronchial epithelial cells exposed to airborne fine particulate matter collected from Wuhan, China	Toxicology letters	2014
PM2.5	LOC441377	Global gene expression profiling of human bronchial epithelial cells exposed to airborne fine particulate matter collected from Wuhan, China	Toxicology letters	2014
PM2.5	RPL13	Global gene expression profiling of human bronchial epithelial cells exposed to airborne fine particulate matter collected from Wuhan, China	Toxicology letters	2014
PM2.5	RPL13A	Global gene expression profiling of human bronchial epithelial cells exposed to airborne fine particulate matter collected from Wuhan, China	Toxicology letters	2014
PM2.5	RPL38	Global gene expression profiling of human bronchial epithelial cells exposed to airborne fine particulate matter collected from Wuhan, China	Toxicology letters	2014
PM2.5	RPS13	Global gene expression profiling of human bronchial epithelial cells exposed to airborne fine particulate matter collected from Wuhan, China	Toxicology letters	2014
PM2.5	RPL34	Global gene expression profiling of human bronchial epithelial cells exposed to airborne fine particulate matter collected from Wuhan, China	Toxicology letters	2014
PM2.5	RPS15A	Global gene expression profiling of human bronchial epithelial cells exposed to airborne fine particulate matter collected from Wuhan, China	Toxicology letters	2014
PM2.5	LOC391656	Global gene expression profiling of human bronchial epithelial cells exposed to airborne fine particulate matter collected from Wuhan, China	Toxicology letters	2014
PM2.5	RPS8	Global gene expression profiling of human bronchial epithelial cells exposed to airborne fine particulate matter collected from Wuhan, China	Toxicology letters	2014

PM2.5	RPL14	Global gene expression profiling of human bronchial epithelial cells exposed to airborne fine particulate matter collected from Wuhan, China	Toxicology letters	2014
PM2.5	RPLP1	Global gene expression profiling of human bronchial epithelial cells exposed to airborne fine particulate matter collected from Wuhan, China	Toxicology letters	2014
PM2.5	UBE2J2	Global gene expression profiling of human bronchial epithelial cells exposed to airborne fine particulate matter collected from Wuhan, China	Toxicology letters	2014
PM2.5	UBE3A	Global gene expression profiling of human bronchial epithelial cells exposed to airborne fine particulate matter collected from Wuhan, China	Toxicology letters	2014
PM2.5	HERC2	Global gene expression profiling of human bronchial epithelial cells exposed to airborne fine particulate matter collected from Wuhan, China	Toxicology letters	2014
PM2.5	DET1	Global gene expression profiling of human bronchial epithelial cells exposed to airborne fine particulate matter collected from Wuhan, China	Toxicology letters	2014
PM2.5	HERC4	Global gene expression profiling of human bronchial epithelial cells exposed to airborne fine particulate matter collected from Wuhan, China	Toxicology letters	2014
PM2.5	FANCL	Global gene expression profiling of human bronchial epithelial cells exposed to airborne fine particulate matter collected from Wuhan, China	Toxicology letters	2014
PM2.5	UBE3C	Global gene expression profiling of human bronchial epithelial cells exposed to airborne fine particulate matter collected from Wuhan, China	Toxicology letters	2014
PM2.5	FBXO2	Global gene expression profiling of human bronchial epithelial cells exposed to airborne fine particulate matter collected from Wuhan, China	Toxicology letters	2014
PM2.5	UBE2S	Global gene expression profiling of human bronchial epithelial cells exposed to airborne fine particulate matter collected from Wuhan, China	Toxicology letters	2014
PM2.5	UBE2L3	Global gene expression profiling of human bronchial epithelial cells exposed to airborne fine particulate matter collected from Wuhan, China	Toxicology letters	2014
PM2.5	MLH1	Global gene expression profiling of human bronchial epithelial cells exposed to airborne fine particulate matter collected from Wuhan, China	Toxicology letters	2014
PM2.5	RPA2	Global gene expression profiling of human bronchial epithelial cells exposed to airborne fine particulate matter collected from Wuhan, China	Toxicology letters	2014
PM2.5	POLD1	Global gene expression profiling of human bronchial epithelial cells exposed to airborne fine particulate matter collected from Wuhan, China	Toxicology letters	2014
PM2.5	RPA3	Global gene expression profiling of human bronchial epithelial cells exposed to airborne fine particulate matter collected from Wuhan, China	Toxicology letters	2014
PM2.5	PMS2	Global gene expression profiling of human bronchial epithelial cells exposed to airborne fine particulate matter collected from Wuhan, China	Toxicology letters	2014
PM2.5	RFC3	Global gene expression profiling of human bronchial epithelial cells exposed to airborne fine particulate matter collected from Wuhan, China	Toxicology letters	2014
PM2.5	TOP3A	Global gene expression profiling of human bronchial epithelial cells exposed to airborne fine particulate matter collected from Wuhan, China	Toxicology letters	2014
PM2.5	NBN	Global gene expression profiling of human bronchial epithelial cells exposed to airborne fine particulate matter collected from Wuhan, China	Toxicology letters	2014
PM2.5	RAD51L3	Global gene expression profiling of human bronchial epithelial cells exposed to airborne fine particulate matter collected from Wuhan, China	Toxicology letters	2014
PM2.5	GTF2H3	Global gene expression profiling of human bronchial epithelial cells exposed to airborne fine particulate matter collected from Wuhan, China	Toxicology letters	2014

PM2.5	ERCC5	Global gene expression profiling of human bronchial epithelial cells exposed to airborne fine particulate matter collected from Wuhan, China	Toxicology letters	2014
PM2.5	POMP	Global gene expression profiling of human bronchial epithelial cells exposed to airborne fine particulate matter collected from Wuhan, China	Toxicology letters	2014
PM2.5	PSMC4	Global gene expression profiling of human bronchial epithelial cells exposed to airborne fine particulate matter collected from Wuhan, China	Toxicology letters	2014
PM2.5	LOC652826	Global gene expression profiling of human bronchial epithelial cells exposed to airborne fine particulate matter collected from Wuhan, China	Toxicology letters	2014
PM2.5	PSMB3	Global gene expression profiling of human bronchial epithelial cells exposed to airborne fine particulate matter collected from Wuhan, China	Toxicology letters	2014
PM2.5	PSMD12	Global gene expression profiling of human bronchial epithelial cells exposed to airborne fine particulate matter collected from Wuhan, China	Toxicology letters	2014
PM2.5	PSMD3	Global gene expression profiling of human bronchial epithelial cells exposed to airborne fine particulate matter collected from Wuhan, China	Toxicology letters	2014
PM2.5	POLR1B	Global gene expression profiling of human bronchial epithelial cells exposed to airborne fine particulate matter collected from Wuhan, China	Toxicology letters	2014
PM2.5	POLR2L	Global gene expression profiling of human bronchial epithelial cells exposed to airborne fine particulate matter collected from Wuhan, China	Toxicology letters	2014
PM2.5	POLR2G	Global gene expression profiling of human bronchial epithelial cells exposed to airborne fine particulate matter collected from Wuhan, China	Toxicology letters	2014
PM2.5	GGPS1	Global gene expression profiling of human bronchial epithelial cells exposed to airborne fine particulate matter collected from Wuhan, China	Toxicology letters	2014
PM2.5	FDPS	Global gene expression profiling of human bronchial epithelial cells exposed to airborne fine particulate matter collected from Wuhan, China	Toxicology letters	2014
PM2.5	EBP	Global gene expression profiling of human bronchial epithelial cells exposed to airborne fine particulate matter collected from Wuhan, China	Toxicology letters	2014
PM2.5	TM7SF2	Global gene expression profiling of human bronchial epithelial cells exposed to airborne fine particulate matter collected from Wuhan, China	Toxicology letters	2014
PM2.5	CYP51A1	Global gene expression profiling of human bronchial epithelial cells exposed to airborne fine particulate matter collected from Wuhan, China	Toxicology letters	2014
PM2.5	NSDHL	Global gene expression profiling of human bronchial epithelial cells exposed to airborne fine particulate matter collected from Wuhan, China	Toxicology letters	2014
PM2.5	PDE9A	Global gene expression profiling of human bronchial epithelial cells exposed to airborne fine particulate matter collected from Wuhan, China	Toxicology letters	2014
PM2.5	PNPT1	Global gene expression profiling of human bronchial epithelial cells exposed to airborne fine particulate matter collected from Wuhan, China	Toxicology letters	2014
PM2.5	ATIC	Global gene expression profiling of human bronchial epithelial cells exposed to airborne fine particulate matter collected from Wuhan, China	Toxicology letters	2014
PM2.5	PFAS	Global gene expression profiling of human bronchial epithelial cells exposed to airborne fine particulate matter collected from Wuhan, China	Toxicology letters	2014
PM2.5	AK2	Global gene expression profiling of human bronchial epithelial cells exposed to airborne fine particulate matter collected from Wuhan, China	Toxicology letters	2014
PM2.5	ADSS	Global gene expression profiling of human bronchial epithelial cells exposed to airborne fine particulate matter collected from Wuhan, China	Toxicology letters	2014

PM2.5	NDUFA9	Global gene expression profiling of human bronchial epithelial cells exposed to airborne fine particulate matter collected from Wuhan, China	Toxicology letters	2014
PM2.5	COX15	Global gene expression profiling of human bronchial epithelial cells exposed to airborne fine particulate matter collected from Wuhan, China	Toxicology letters	2014
PM2.5	NDUFS2	Global gene expression profiling of human bronchial epithelial cells exposed to airborne fine particulate matter collected from Wuhan, China	Toxicology letters	2014
PM2.5	COX7C	Global gene expression profiling of human bronchial epithelial cells exposed to airborne fine particulate matter collected from Wuhan, China	Toxicology letters	2014
PM2.5	ATP5B	Global gene expression profiling of human bronchial epithelial cells exposed to airborne fine particulate matter collected from Wuhan, China	Toxicology letters	2014
PM2.5	hCG_25371	Global gene expression profiling of human bronchial epithelial cells exposed to airborne fine particulate matter collected from Wuhan, China	Toxicology letters	2014
PM2.5	UQCRH	Global gene expression profiling of human bronchial epithelial cells exposed to airborne fine particulate matter collected from Wuhan, China	Toxicology letters	2014
PM2.5	ATP5E	Global gene expression profiling of human bronchial epithelial cells exposed to airborne fine particulate matter collected from Wuhan, China	Toxicology letters	2014
PM2.5	COX5B	Global gene expression profiling of human bronchial epithelial cells exposed to airborne fine particulate matter collected from Wuhan, China	Toxicology letters	2014
PM2.5	NDUFV1	Global gene expression profiling of human bronchial epithelial cells exposed to airborne fine particulate matter collected from Wuhan, China	Toxicology letters	2014
PM2.5	LCLAT1	Global gene expression profiling of human bronchial epithelial cells exposed to airborne fine particulate matter collected from Wuhan, China	Toxicology letters	2014
PM2.5	MAOB	Global gene expression profiling of human bronchial epithelial cells exposed to airborne fine particulate matter collected from Wuhan, China	Toxicology letters	2014
PM2.5	MIF	Global gene expression profiling of human bronchial epithelial cells exposed to airborne fine particulate matter collected from Wuhan, China	Toxicology letters	2014
PM2.5	GOT1	Global gene expression profiling of human bronchial epithelial cells exposed to airborne fine particulate matter collected from Wuhan, China	Toxicology letters	2014
PM2.5	CMPK1	Global gene expression profiling of human bronchial epithelial cells exposed to airborne fine particulate matter collected from Wuhan, China	Toxicology letters	2014
PM2.5	UPRT	Global gene expression profiling of human bronchial epithelial cells exposed to airborne fine particulate matter collected from Wuhan, China	Toxicology letters	2014
PM2.5	TRMT11	Global gene expression profiling of human bronchial epithelial cells exposed to airborne fine particulate matter collected from Wuhan, China	Toxicology letters	2014
PM2.5	CMAS	Global gene expression profiling of human bronchial epithelial cells exposed to airborne fine particulate matter collected from Wuhan, China	Toxicology letters	2014
PM2.5	PHPT1	Global gene expression profiling of human bronchial epithelial cells exposed to airborne fine particulate matter collected from Wuhan, China	Toxicology letters	2014
PM2.5	NPL	Global gene expression profiling of human bronchial epithelial cells exposed to airborne fine particulate matter collected from Wuhan, China	Toxicology letters	2014
PM2.5	CYB5R3	Global gene expression profiling of human bronchial epithelial cells exposed to airborne fine particulate matter collected from Wuhan, China	Toxicology letters	2014
PM2.5	LDHAL6A	Global gene expression profiling of human bronchial epithelial cells exposed to airborne fine particulate matter collected from Wuhan, China	Toxicology letters	2014

PM2.5	PGAM2	Global gene expression profiling of human bronchial epithelial cells exposed to airborne fine particulate matter collected from Wuhan, China	Toxicology letters	2014
PM2.5	ENO3	Global gene expression profiling of human bronchial epithelial cells exposed to airborne fine particulate matter collected from Wuhan, China	Toxicology letters	2014
PM2.5	ALAD	Global gene expression profiling of human bronchial epithelial cells exposed to airborne fine particulate matter collected from Wuhan, China	Toxicology letters	2014
PM2.5	FECH	Global gene expression profiling of human bronchial epithelial cells exposed to airborne fine particulate matter collected from Wuhan, China	Toxicology letters	2014
PM2.5	LYPLA1	Global gene expression profiling of human bronchial epithelial cells exposed to airborne fine particulate matter collected from Wuhan, China	Toxicology letters	2014
PM2.5	CHKB	Global gene expression profiling of human bronchial epithelial cells exposed to airborne fine particulate matter collected from Wuhan, China	Toxicology letters	2014
PM2.5	ETNK1	Global gene expression profiling of human bronchial epithelial cells exposed to airborne fine particulate matter collected from Wuhan, China	Toxicology letters	2014
PM2.5	AKR1B10	Global gene expression profiling of human bronchial epithelial cells exposed to airborne fine particulate matter collected from Wuhan, China	Toxicology letters	2014
PM2.5	GLA	Global gene expression profiling of human bronchial epithelial cells exposed to airborne fine particulate matter collected from Wuhan, China	Toxicology letters	2014
PM2.5	MGLL	Global gene expression profiling of human bronchial epithelial cells exposed to airborne fine particulate matter collected from Wuhan, China	Toxicology letters	2014
PM2.5	B4GALT3	Global gene expression profiling of human bronchial epithelial cells exposed to airborne fine particulate matter collected from Wuhan, China	Toxicology letters	2014
PM2.5	FUT6	Global gene expression profiling of human bronchial epithelial cells exposed to airborne fine particulate matter collected from Wuhan, China	Toxicology letters	2014
PM2.5	ST8SIA1	Global gene expression profiling of human bronchial epithelial cells exposed to airborne fine particulate matter collected from Wuhan, China	Toxicology letters	2014
PM2.5	AARS	Global gene expression profiling of human bronchial epithelial cells exposed to airborne fine particulate matter collected from Wuhan, China	Toxicology letters	2014
PM2.5	GALNT11	Global gene expression profiling of human bronchial epithelial cells exposed to airborne fine particulate matter collected from Wuhan, China	Toxicology letters	2014
PM2.5	OGT	Global gene expression profiling of human bronchial epithelial cells exposed to airborne fine particulate matter collected from Wuhan, China	Toxicology letters	2014
PM2.5	HNMT	Global gene expression profiling of human bronchial epithelial cells exposed to airborne fine particulate matter collected from Wuhan, China	Toxicology letters	2014
PM2.5	PPID	Global gene expression profiling of human bronchial epithelial cells exposed to airborne fine particulate matter collected from Wuhan, China	Toxicology letters	2014
PM2.5	UBB	Global gene expression profiling of human bronchial epithelial cells exposed to airborne fine particulate matter collected from Wuhan, China	Toxicology letters	2014
PM2.5	EGLN3	Global gene expression profiling of human bronchial epithelial cells exposed to airborne fine particulate matter collected from Wuhan, China	Toxicology letters	2014
PM2.5	CREB1	Global gene expression profiling of human bronchial epithelial cells exposed to airborne fine particulate matter collected from Wuhan, China	Toxicology letters	2014
PM2.5	DAPK3	Global gene expression profiling of human bronchial epithelial cells exposed to airborne fine particulate matter collected from Wuhan, China	Toxicology letters	2014

PM2.5	FZD8	Global gene expression profiling of human bronchial epithelial cells exposed to airborne fine particulate matter collected from Wuhan, China	Toxicology letters	2014
PM2.5	CKS1B	Global gene expression profiling of human bronchial epithelial cells exposed to airborne fine particulate matter collected from Wuhan, China	Toxicology letters	2014
PM2.5	TPM3	Global gene expression profiling of human bronchial epithelial cells exposed to airborne fine particulate matter collected from Wuhan, China	Toxicology letters	2014
PM2.5	ALS2	Global gene expression profiling of human bronchial epithelial cells exposed to airborne fine particulate matter collected from Wuhan, China	Toxicology letters	2014
PM2.5	SOD1	Global gene expression profiling of human bronchial epithelial cells exposed to airborne fine particulate matter collected from Wuhan, China	Toxicology letters	2014
PM2.5	MC1R	Global gene expression profiling of human bronchial epithelial cells exposed to airborne fine particulate matter collected from Wuhan, China	Toxicology letters	2014
PM2.5	C2	Global gene expression profiling of human bronchial epithelial cells exposed to airborne fine particulate matter collected from Wuhan, China	Toxicology letters	2014
PM2.5	C1QB	Global gene expression profiling of human bronchial epithelial cells exposed to airborne fine particulate matter collected from Wuhan, China	Toxicology letters	2014
PM2.5	CD59	Global gene expression profiling of human bronchial epithelial cells exposed to airborne fine particulate matter collected from Wuhan, China	Toxicology letters	2014
PM2.5	CFD	Global gene expression profiling of human bronchial epithelial cells exposed to airborne fine particulate matter collected from Wuhan, China	Toxicology letters	2014
PM2.5	RFXAP	Global gene expression profiling of human bronchial epithelial cells exposed to airborne fine particulate matter collected from Wuhan, China	Toxicology letters	2014
PM2.5	HLA-C	Global gene expression profiling of human bronchial epithelial cells exposed to airborne fine particulate matter collected from Wuhan, China	Toxicology letters	2014
PM2.5	TNFRSF10B	Global gene expression profiling of human bronchial epithelial cells exposed to airborne fine particulate matter collected from Wuhan, China	Toxicology letters	2014
PM2.5	DDB2	Global gene expression profiling of human bronchial epithelial cells exposed to airborne fine particulate matter collected from Wuhan, China	Toxicology letters	2014
PM2.5	TRADD	Global gene expression profiling of human bronchial epithelial cells exposed to airborne fine particulate matter collected from Wuhan, China	Toxicology letters	2014
PM2.5	CAPN1	Global gene expression profiling of human bronchial epithelial cells exposed to airborne fine particulate matter collected from Wuhan, China	Toxicology letters	2014
PM2.5	IL3RA	Global gene expression profiling of human bronchial epithelial cells exposed to airborne fine particulate matter collected from Wuhan, China	Toxicology letters	2014
PM2.5	GSN	Global gene expression profiling of human bronchial epithelial cells exposed to airborne fine particulate matter collected from Wuhan, China	Toxicology letters	2014
PM2.5	DIAPH1	Global gene expression profiling of human bronchial epithelial cells exposed to airborne fine particulate matter collected from Wuhan, China	Toxicology letters	2014
PM2.5	GNA12	Global gene expression profiling of human bronchial epithelial cells exposed to airborne fine particulate matter collected from Wuhan, China	Toxicology letters	2014
PM2.5	BAIAP2	Global gene expression profiling of human bronchial epithelial cells exposed to airborne fine particulate matter collected from Wuhan, China	Toxicology letters	2014
PM2.5	MAP3K7IP2	Global gene expression profiling of human bronchial epithelial cells exposed to airborne fine particulate matter collected from Wuhan, China	Toxicology letters	2014

PM2.5	NFKB2	Global gene expression profiling of human bronchial epithelial cells exposed to airborne fine particulate matter collected from Wuhan, China	Toxicology letters	2014
PM2.5	PRKCA	Global gene expression profiling of human bronchial epithelial cells exposed to airborne fine particulate matter collected from Wuhan, China	Toxicology letters	2014
PM2.5	PPP5C	Global gene expression profiling of human bronchial epithelial cells exposed to airborne fine particulate matter collected from Wuhan, China	Toxicology letters	2014
PM2.5	CXCL10	Global gene expression profiling of human bronchial epithelial cells exposed to airborne fine particulate matter collected from Wuhan, China	Toxicology letters	2014
PM2.5	RPS12	Global gene expression profiling of human bronchial epithelial cells exposed to airborne fine particulate matter collected from Wuhan, China	Toxicology letters	2014
PM2.5	RPS21	Global gene expression profiling of human bronchial epithelial cells exposed to airborne fine particulate matter collected from Wuhan, China	Toxicology letters	2014
PM2.5	RPL7	Global gene expression profiling of human bronchial epithelial cells exposed to airborne fine particulate matter collected from Wuhan, China	Toxicology letters	2014
PM2.5	POLR3H	Global gene expression profiling of human bronchial epithelial cells exposed to airborne fine particulate matter collected from Wuhan, China	Toxicology letters	2014
PM2.5	POLR3D	Global gene expression profiling of human bronchial epithelial cells exposed to airborne fine particulate matter collected from Wuhan, China	Toxicology letters	2014
PM2.5	SYVN1	Global gene expression profiling of human bronchial epithelial cells exposed to airborne fine particulate matter collected from Wuhan, China	Toxicology letters	2014
PM2.5	MGRN1	Global gene expression profiling of human bronchial epithelial cells exposed to airborne fine particulate matter collected from Wuhan, China	Toxicology letters	2014
PM2.5	ERCC4	Global gene expression profiling of human bronchial epithelial cells exposed to airborne fine particulate matter collected from Wuhan, China	Toxicology letters	2014
PM2.5	GGCX	Global gene expression profiling of human bronchial epithelial cells exposed to airborne fine particulate matter collected from Wuhan, China	Toxicology letters	2014
PM2.5	NT5E	Global gene expression profiling of human bronchial epithelial cells exposed to airborne fine particulate matter collected from Wuhan, China	Toxicology letters	2014
PM2.5	DCK	Global gene expression profiling of human bronchial epithelial cells exposed to airborne fine particulate matter collected from Wuhan, China	Toxicology letters	2014
PM2.5	GSTO1	Global gene expression profiling of human bronchial epithelial cells exposed to airborne fine particulate matter collected from Wuhan, China	Toxicology letters	2014
PM2.5	CYP2B6	Global gene expression profiling of human bronchial epithelial cells exposed to airborne fine particulate matter collected from Wuhan, China	Toxicology letters	2014
PM2.5	AKR1C4	Global gene expression profiling of human bronchial epithelial cells exposed to airborne fine particulate matter collected from Wuhan, China	Toxicology letters	2014
PM10	CRAT	Health effects of ambient particulate matter--biological mechanisms and inflammatory responses to in vitro and in vivo particle exposures.	Inhal Toxicol.	2008
PM10	MYLPF	Health effects of ambient particulate matter--biological mechanisms and inflammatory responses to in vitro and in vivo particle exposures.	Inhal Toxicol.	2008
PM10	S100G	Health effects of ambient particulate matter--biological mechanisms and inflammatory responses to in vitro and in vivo particle exposures.	Inhal Toxicol.	2008
PM10	IER3	Health effects of ambient particulate matter--biological mechanisms and inflammatory responses to in vitro and in vivo particle exposures.	Inhal Toxicol.	2008

PM10	TIMP3	Health effects of ambient particulate matter--biological mechanisms and inflammatory responses to in vitro and in vivo particle exposures.	Inhal Toxicol.	2008
PM10	ITIH2	Health effects of ambient particulate matter--biological mechanisms and inflammatory responses to in vitro and in vivo particle exposures.	Inhal Toxicol.	2008
PM10	MB	Health effects of ambient particulate matter--biological mechanisms and inflammatory responses to in vitro and in vivo particle exposures.	Inhal Toxicol.	2008
PM10	TNNI3	Health effects of ambient particulate matter--biological mechanisms and inflammatory responses to in vitro and in vivo particle exposures.	Inhal Toxicol.	2008
PM10	PLOD1	Health effects of ambient particulate matter--biological mechanisms and inflammatory responses to in vitro and in vivo particle exposures.	Inhal Toxicol.	2008
PM10	TNNT2	Health effects of ambient particulate matter--biological mechanisms and inflammatory responses to in vitro and in vivo particle exposures.	Inhal Toxicol.	2008
PM10	DNASE1	Health effects of ambient particulate matter--biological mechanisms and inflammatory responses to in vitro and in vivo particle exposures.	Inhal Toxicol.	2008
PM10	SNAG1	Health effects of ambient particulate matter--biological mechanisms and inflammatory responses to in vitro and in vivo particle exposures.	Inhal Toxicol.	2008
PM10	K11RIK	Health effects of ambient particulate matter--biological mechanisms and inflammatory responses to in vitro and in vivo particle exposures.	Inhal Toxicol.	2008
PM10	ATP5H	Health effects of ambient particulate matter--biological mechanisms and inflammatory responses to in vitro and in vivo particle exposures.	Inhal Toxicol.	2008
PM10	SCN1B	Health effects of ambient particulate matter--biological mechanisms and inflammatory responses to in vitro and in vivo particle exposures.	Inhal Toxicol.	2008
PM10	CYP1B1	Comparison of gene expression profiles induced by coarse, fine, and ultrafine particulate matter.	J Toxicol Environ Health A	2011
PM10	CYP1A1	Comparison of gene expression profiles induced by coarse, fine, and ultrafine particulate matter.	J Toxicol Environ Health A	2011
PM10	IL13RA2	Comparison of gene expression profiles induced by coarse, fine, and ultrafine particulate matter.	J Toxicol Environ Health A	2011
PM10	HMOX1	Comparison of gene expression profiles induced by coarse, fine, and ultrafine particulate matter.	J Toxicol Environ Health A	2011
PM10	TXNRD1	Comparison of gene expression profiles induced by coarse, fine, and ultrafine particulate matter.	J Toxicol Environ Health A	2011
PM10	CYP4F11	Comparison of gene expression profiles induced by coarse, fine, and ultrafine particulate matter.	J Toxicol Environ Health A	2011
PM10	NQO1	Comparison of gene expression profiles induced by coarse, fine, and ultrafine particulate matter.	J Toxicol Environ Health A	2011
PM10	HSPA1A	Comparison of gene expression profiles induced by coarse, fine, and ultrafine particulate matter.	J Toxicol Environ Health A	2011
PM10	DHR59	Comparison of gene expression profiles induced by coarse, fine, and ultrafine particulate matter.	J Toxicol Environ Health A	2011
PM10	SQSTM1	Comparison of gene expression profiles induced by coarse, fine, and ultrafine particulate matter.	J Toxicol Environ Health A	2011
PM10	FOSL1	Comparison of gene expression profiles induced by coarse, fine, and ultrafine particulate matter.	J Toxicol Environ Health A	2011
PM10	GCLM	Comparison of gene expression profiles induced by coarse, fine, and ultrafine particulate matter.	J Toxicol Environ Health A	2011
PM10	SLC7A11	Comparison of gene expression profiles induced by coarse, fine, and ultrafine particulate matter.	J Toxicol Environ Health A	2011
PM10	LRP8	Comparison of gene expression profiles induced by coarse, fine, and ultrafine particulate matter.	J Toxicol Environ Health A	2011
PM10	LPIN1	Comparison of gene expression profiles induced by coarse, fine, and ultrafine particulate matter.	J Toxicol Environ Health A	2011
PM10	SLC6A6	Comparison of gene expression profiles induced by coarse, fine, and ultrafine particulate matter.	J Toxicol Environ Health A	2011
PM10	AKR1C1	Comparison of gene expression profiles induced by coarse, fine, and ultrafine particulate matter.	J Toxicol Environ Health A	2011
PM10	CEACAM1	Comparison of gene expression profiles induced by coarse, fine, and ultrafine particulate matter.	J Toxicol Environ Health A	2011

PM10	NAV3	Comparison of gene expression profiles induced by coarse, fine, and ultrafine particulate matter.	J Toxicol Environ Health A	2011
PM10	IL1RL1	Comparison of gene expression profiles induced by coarse, fine, and ultrafine particulate matter.	J Toxicol Environ Health A	2011
PM10	PSRC1	Comparison of gene expression profiles induced by coarse, fine, and ultrafine particulate matter.	J Toxicol Environ Health A	2011
PM10	PTTG1	Comparison of gene expression profiles induced by coarse, fine, and ultrafine particulate matter.	J Toxicol Environ Health A	2011
PM10	HSF2BP	Comparison of gene expression profiles induced by coarse, fine, and ultrafine particulate matter.	J Toxicol Environ Health A	2011
PM10	ID3	Comparison of gene expression profiles induced by coarse, fine, and ultrafine particulate matter.	J Toxicol Environ Health A	2011
PM10	BUB1	Comparison of gene expression profiles induced by coarse, fine, and ultrafine particulate matter.	J Toxicol Environ Health A	2011
PM10	CCNB2	Comparison of gene expression profiles induced by coarse, fine, and ultrafine particulate matter.	J Toxicol Environ Health A	2011
PM10	HMMR	Comparison of gene expression profiles induced by coarse, fine, and ultrafine particulate matter.	J Toxicol Environ Health A	2011
PM10	CALML3	Comparison of gene expression profiles induced by coarse, fine, and ultrafine particulate matter.	J Toxicol Environ Health A	2011
PM10	NDC80	Comparison of gene expression profiles induced by coarse, fine, and ultrafine particulate matter.	J Toxicol Environ Health A	2011
PM10	CBLB	Comparison of gene expression profiles induced by coarse, fine, and ultrafine particulate matter.	J Toxicol Environ Health A	2011
PM10	CXCL14	Comparison of gene expression profiles induced by coarse, fine, and ultrafine particulate matter.	J Toxicol Environ Health A	2011
PM10	VSNL1	Comparison of gene expression profiles induced by coarse, fine, and ultrafine particulate matter.	J Toxicol Environ Health A	2011
PM10	UBE2C	Comparison of gene expression profiles induced by coarse, fine, and ultrafine particulate matter.	J Toxicol Environ Health A	2011
PM10	CCNB1	Comparison of gene expression profiles induced by coarse, fine, and ultrafine particulate matter.	J Toxicol Environ Health A	2011
PM10	DLGAP5	Comparison of gene expression profiles induced by coarse, fine, and ultrafine particulate matter.	J Toxicol Environ Health A	2011
PM10	CDC20	Comparison of gene expression profiles induced by coarse, fine, and ultrafine particulate matter.	J Toxicol Environ Health A	2011
PM10	WNT4	Comparison of gene expression profiles induced by coarse, fine, and ultrafine particulate matter.	J Toxicol Environ Health A	2011
PM10	METTL7A	Comparison of gene expression profiles induced by coarse, fine, and ultrafine particulate matter.	J Toxicol Environ Health A	2011
PM10	KIF20A	Comparison of gene expression profiles induced by coarse, fine, and ultrafine particulate matter.	J Toxicol Environ Health A	2011
PM10	PALMD	Comparison of gene expression profiles induced by coarse, fine, and ultrafine particulate matter.	J Toxicol Environ Health A	2011
PM10	MMP1	Gene expression profiling and pathway analysis of human bronchial epithelial cells exposed to airborne particulate matter collected from Saudi Arabia	Toxicol Appl Pharmacol	2012
PM10	SERPINB2	Gene expression profiling and pathway analysis of human bronchial epithelial cells exposed to airborne particulate matter collected from Saudi Arabia	Toxicol Appl Pharmacol	2012
PM10	IL24	Gene expression profiling and pathway analysis of human bronchial epithelial cells exposed to airborne particulate matter collected from Saudi Arabia	Toxicol Appl Pharmacol	2012
PM10	HMOX1	Gene expression profiling and pathway analysis of human bronchial epithelial cells exposed to airborne particulate matter collected from Saudi Arabia	Toxicol Appl Pharmacol	2012
PM10	GPNMB	Gene expression profiling and pathway analysis of human bronchial epithelial cells exposed to airborne particulate matter collected from Saudi Arabia	Toxicol Appl Pharmacol	2012
PM10	RRAGD	Gene expression profiling and pathway analysis of human bronchial epithelial cells exposed to airborne particulate matter collected from Saudi Arabia	Toxicol Appl Pharmacol	2012
PM10	SLC7A11	Gene expression profiling and pathway analysis of human bronchial epithelial cells exposed to airborne particulate matter collected from Saudi Arabia	Toxicol Appl Pharmacol	2012
PM10	TRIM16L	Gene expression profiling and pathway analysis of human bronchial epithelial cells exposed to airborne particulate matter collected from Saudi Arabia	Toxicol Appl Pharmacol	2012

PM10	CCND1	Gene expression profiling and pathway analysis of human bronchial epithelial cells exposed to airborne particulate matter collected from Saudi Arabia	Toxicol Appl Pharmacol	2012
PM10	LOC344887	Gene expression profiling and pathway analysis of human bronchial epithelial cells exposed to airborne particulate matter collected from Saudi Arabia	Toxicol Appl Pharmacol	2012
PM10	STC2	Gene expression profiling and pathway analysis of human bronchial epithelial cells exposed to airborne particulate matter collected from Saudi Arabia	Toxicol Appl Pharmacol	2012
PM10	ETV5	Gene expression profiling and pathway analysis of human bronchial epithelial cells exposed to airborne particulate matter collected from Saudi Arabia	Toxicol Appl Pharmacol	2012
PM10	SRXN1	Gene expression profiling and pathway analysis of human bronchial epithelial cells exposed to airborne particulate matter collected from Saudi Arabia	Toxicol Appl Pharmacol	2012
PM10	TM4SF19	Gene expression profiling and pathway analysis of human bronchial epithelial cells exposed to airborne particulate matter collected from Saudi Arabia	Toxicol Appl Pharmacol	2012
PM10	DDIT3	Gene expression profiling and pathway analysis of human bronchial epithelial cells exposed to airborne particulate matter collected from Saudi Arabia	Toxicol Appl Pharmacol	2012
PM10	EPHA4	Gene expression profiling and pathway analysis of human bronchial epithelial cells exposed to airborne particulate matter collected from Saudi Arabia	Toxicol Appl Pharmacol	2012
PM10	TNFRSF11B	Gene expression profiling and pathway analysis of human bronchial epithelial cells exposed to airborne particulate matter collected from Saudi Arabia	Toxicol Appl Pharmacol	2012
PM10	ITGB8	Gene expression profiling and pathway analysis of human bronchial epithelial cells exposed to airborne particulate matter collected from Saudi Arabia	Toxicol Appl Pharmacol	2012
PM10	PDGFRB	Gene expression profiling and pathway analysis of human bronchial epithelial cells exposed to airborne particulate matter collected from Saudi Arabia	Toxicol Appl Pharmacol	2012
PM10	ADAMTS15	Gene expression profiling and pathway analysis of human bronchial epithelial cells exposed to airborne particulate matter collected from Saudi Arabia	Toxicol Appl Pharmacol	2012
PM10	ID2	Gene expression profiling and pathway analysis of human bronchial epithelial cells exposed to airborne particulate matter collected from Saudi Arabia	Toxicol Appl Pharmacol	2012
PM10	SMAD6	Gene expression profiling and pathway analysis of human bronchial epithelial cells exposed to airborne particulate matter collected from Saudi Arabia	Toxicol Appl Pharmacol	2012
PM10	TNFSF10	Gene expression profiling and pathway analysis of human bronchial epithelial cells exposed to airborne particulate matter collected from Saudi Arabia	Toxicol Appl Pharmacol	2012
PM10	BMP4	Gene expression profiling and pathway analysis of human bronchial epithelial cells exposed to airborne particulate matter collected from Saudi Arabia	Toxicol Appl Pharmacol	2012
PM10	BDKRB1	Gene expression profiling and pathway analysis of human bronchial epithelial cells exposed to airborne particulate matter collected from Saudi Arabia	Toxicol Appl Pharmacol	2012
PM10	PLAC8	Gene expression profiling and pathway analysis of human bronchial epithelial cells exposed to airborne particulate matter collected from Saudi Arabia	Toxicol Appl Pharmacol	2012
PM10	ATOH8	Gene expression profiling and pathway analysis of human bronchial epithelial cells exposed to airborne particulate matter collected from Saudi Arabia	Toxicol Appl Pharmacol	2012
PM10	IGFBP3	Gene expression profiling and pathway analysis of human bronchial epithelial cells exposed to airborne particulate matter collected from Saudi Arabia	Toxicol Appl Pharmacol	2012
PM10	SULF1	Gene expression profiling and pathway analysis of human bronchial epithelial cells exposed to airborne particulate matter collected from Saudi Arabia	Toxicol Appl Pharmacol	2012
PM10	DIO2	Gene expression profiling and pathway analysis of human bronchial epithelial cells exposed to airborne particulate matter collected from Saudi Arabia	Toxicol Appl Pharmacol	2012

PM10	IL24	Gene expression profiling and pathway analysis of human bronchial epithelial cells exposed to airborne particulate matter collected from Saudi Arabia	Toxicol Appl Pharmacol	2012
PM10	SERPINB2	Gene expression profiling and pathway analysis of human bronchial epithelial cells exposed to airborne particulate matter collected from Saudi Arabia	Toxicol Appl Pharmacol	2012
PM10	GPNMB	Gene expression profiling and pathway analysis of human bronchial epithelial cells exposed to airborne particulate matter collected from Saudi Arabia	Toxicol Appl Pharmacol	2012
PM10	AKR1C3	Gene expression profiling and pathway analysis of human bronchial epithelial cells exposed to airborne particulate matter collected from Saudi Arabia	Toxicol Appl Pharmacol	2012
PM10	SCD	Gene expression profiling and pathway analysis of human bronchial epithelial cells exposed to airborne particulate matter collected from Saudi Arabia	Toxicol Appl Pharmacol	2012
PM10	C9ORF150	Gene expression profiling and pathway analysis of human bronchial epithelial cells exposed to airborne particulate matter collected from Saudi Arabia	Toxicol Appl Pharmacol	2012
PM10	CYP1B1	Gene expression profiling and pathway analysis of human bronchial epithelial cells exposed to airborne particulate matter collected from Saudi Arabia	Toxicol Appl Pharmacol	2012
PM10	RRAGD	Gene expression profiling and pathway analysis of human bronchial epithelial cells exposed to airborne particulate matter collected from Saudi Arabia	Toxicol Appl Pharmacol	2012
PM10	TRIM16L	Gene expression profiling and pathway analysis of human bronchial epithelial cells exposed to airborne particulate matter collected from Saudi Arabia	Toxicol Appl Pharmacol	2012
PM10	MMP1	Gene expression profiling and pathway analysis of human bronchial epithelial cells exposed to airborne particulate matter collected from Saudi Arabia	Toxicol Appl Pharmacol	2012
PM10	INSIG1	Gene expression profiling and pathway analysis of human bronchial epithelial cells exposed to airborne particulate matter collected from Saudi Arabia	Toxicol Appl Pharmacol	2012
PM10	ENPP2	Gene expression profiling and pathway analysis of human bronchial epithelial cells exposed to airborne particulate matter collected from Saudi Arabia	Toxicol Appl Pharmacol	2012
PM10	PPARG	Gene expression profiling and pathway analysis of human bronchial epithelial cells exposed to airborne particulate matter collected from Saudi Arabia	Toxicol Appl Pharmacol	2012
PM10	RAB27B	Gene expression profiling and pathway analysis of human bronchial epithelial cells exposed to airborne particulate matter collected from Saudi Arabia	Toxicol Appl Pharmacol	2012
PM10	PIR	Gene expression profiling and pathway analysis of human bronchial epithelial cells exposed to airborne particulate matter collected from Saudi Arabia	Toxicol Appl Pharmacol	2012
PM10	CLSTN2	Gene expression profiling and pathway analysis of human bronchial epithelial cells exposed to airborne particulate matter collected from Saudi Arabia	Toxicol Appl Pharmacol	2012
PM10	RNF150	Gene expression profiling and pathway analysis of human bronchial epithelial cells exposed to airborne particulate matter collected from Saudi Arabia	Toxicol Appl Pharmacol	2012
PM10	TACSTD2	Gene expression profiling and pathway analysis of human bronchial epithelial cells exposed to airborne particulate matter collected from Saudi Arabia	Toxicol Appl Pharmacol	2012
PM10	SERTAD4	Gene expression profiling and pathway analysis of human bronchial epithelial cells exposed to airborne particulate matter collected from Saudi Arabia	Toxicol Appl Pharmacol	2012
PM10	KIT	Gene expression profiling and pathway analysis of human bronchial epithelial cells exposed to airborne particulate matter collected from Saudi Arabia	Toxicol Appl Pharmacol	2012
PM10	LYPD6B	Gene expression profiling and pathway analysis of human bronchial epithelial cells exposed to airborne particulate matter collected from Saudi Arabia	Toxicol Appl Pharmacol	2012
PM10	IGFBP3	Gene expression profiling and pathway analysis of human bronchial epithelial cells exposed to airborne particulate matter collected from Saudi Arabia	Toxicol Appl Pharmacol	2012

PM10	ADAMTS5	Gene expression profiling and pathway analysis of human bronchial epithelial cells exposed to airborne particulate matter collected from Saudi Arabia	Toxicol Appl Pharmacol	2012
PM10	FN1	Gene expression profiling and pathway analysis of human bronchial epithelial cells exposed to airborne particulate matter collected from Saudi Arabia	Toxicol Appl Pharmacol	2012
PM10	DIO2	Gene expression profiling and pathway analysis of human bronchial epithelial cells exposed to airborne particulate matter collected from Saudi Arabia	Toxicol Appl Pharmacol	2012
PM10	FRY	Gene expression profiling and pathway analysis of human bronchial epithelial cells exposed to airborne particulate matter collected from Saudi Arabia	Toxicol Appl Pharmacol	2012
PM10	DHRS3	Gene expression profiling and pathway analysis of human bronchial epithelial cells exposed to airborne particulate matter collected from Saudi Arabia	Toxicol Appl Pharmacol	2012
PM10	NPPB	Gene expression profiling and pathway analysis of human bronchial epithelial cells exposed to airborne particulate matter collected from Saudi Arabia	Toxicol Appl Pharmacol	2012
PM10	SULF1	Gene expression profiling and pathway analysis of human bronchial epithelial cells exposed to airborne particulate matter collected from Saudi Arabia	Toxicol Appl Pharmacol	2012
PM10	TNFRSF11B	Gene expression profiling and pathway analysis of human bronchial epithelial cells exposed to airborne particulate matter collected from Saudi Arabia	Toxicol Appl Pharmacol	2012
PM10	GDF15	Microarray analysis of gene expression alteration in human middle ear epithelial cells induced by micro particle	Int J Pediatr Otorhinolaryngol	2013
PM10	HMOX1	Microarray analysis of gene expression alteration in human middle ear epithelial cells induced by micro particle	Int J Pediatr Otorhinolaryngol	2013
PM10	THBD	Microarray analysis of gene expression alteration in human middle ear epithelial cells induced by micro particle	Int J Pediatr Otorhinolaryngol	2013
PM10	INPP5D	Microarray analysis of gene expression alteration in human middle ear epithelial cells induced by micro particle	Int J Pediatr Otorhinolaryngol	2013
PM10	ITGB2	Microarray analysis of gene expression alteration in human middle ear epithelial cells induced by micro particle	Int J Pediatr Otorhinolaryngol	2013
PM10	PTGS2	Microarray analysis of gene expression alteration in human middle ear epithelial cells induced by micro particle	Int J Pediatr Otorhinolaryngol	2013
PM10	PGF	Microarray analysis of gene expression alteration in human middle ear epithelial cells induced by micro particle	Int J Pediatr Otorhinolaryngol	2013
PM10	CSF2	Microarray analysis of gene expression alteration in human middle ear epithelial cells induced by micro particle	Int J Pediatr Otorhinolaryngol	2013
PM10	TGM2	Microarray analysis of gene expression alteration in human middle ear epithelial cells induced by micro particle	Int J Pediatr Otorhinolaryngol	2013
PM10	FASN	Microarray analysis of gene expression alteration in human middle ear epithelial cells induced by micro particle	Int J Pediatr Otorhinolaryngol	2013
PM10	DDIT3	Microarray analysis of gene expression alteration in human middle ear epithelial cells induced by micro particle	Int J Pediatr Otorhinolaryngol	2013
PM10	TNFSF14	Microarray analysis of gene expression alteration in human middle ear epithelial cells induced by micro particle	Int J Pediatr Otorhinolaryngol	2013
PM10	CSF3	Microarray analysis of gene expression alteration in human middle ear epithelial cells induced by micro particle	Int J Pediatr Otorhinolaryngol	2013
PM10	HBEGF	Microarray analysis of gene expression alteration in human middle ear epithelial cells induced by micro particle	Int J Pediatr Otorhinolaryngol	2013

PM10	IL1B	Microarray analysis of gene expression alteration in human middle ear epithelial cells induced by micro particle	Int J Pediatr Otorhinolaryngol	2013
PM10	VEGFA	Microarray analysis of gene expression alteration in human middle ear epithelial cells induced by micro particle	Int J Pediatr Otorhinolaryngol	2013
PM10	EPOR	Microarray analysis of gene expression alteration in human middle ear epithelial cells induced by micro particle	Int J Pediatr Otorhinolaryngol	2013
PM10	PPARG	Microarray analysis of gene expression alteration in human middle ear epithelial cells induced by micro particle	Int J Pediatr Otorhinolaryngol	2013
PM10	CASP1	Microarray analysis of gene expression alteration in human middle ear epithelial cells induced by micro particle	Int J Pediatr Otorhinolaryngol	2013
PM10	NOD2	Microarray analysis of gene expression alteration in human middle ear epithelial cells induced by micro particle	Int J Pediatr Otorhinolaryngol	2013
PM10	PTGS1	Microarray analysis of gene expression alteration in human middle ear epithelial cells induced by micro particle	Int J Pediatr Otorhinolaryngol	2013
PM10	KLF4	Microarray analysis of gene expression alteration in human middle ear epithelial cells induced by micro particle	Int J Pediatr Otorhinolaryngol	2013
PM10	MKI67	Microarray analysis of gene expression alteration in human middle ear epithelial cells induced by micro particle	Int J Pediatr Otorhinolaryngol	2013
PM10	IGF1R	Microarray analysis of gene expression alteration in human middle ear epithelial cells induced by micro particle	Int J Pediatr Otorhinolaryngol	2013
PM10	RUNX3	Microarray analysis of gene expression alteration in human middle ear epithelial cells induced by micro particle	Int J Pediatr Otorhinolaryngol	2013
PM10	CAV1	Microarray analysis of gene expression alteration in human middle ear epithelial cells induced by micro particle	Int J Pediatr Otorhinolaryngol	2013
PM10	IGFBP3	Microarray analysis of gene expression alteration in human middle ear epithelial cells induced by micro particle	Int J Pediatr Otorhinolaryngol	2013
PM10	FST	Microarray analysis of gene expression alteration in human middle ear epithelial cells induced by micro particle	Int J Pediatr Otorhinolaryngol	2013
PM10	RUNX1	Microarray analysis of gene expression alteration in human middle ear epithelial cells induced by micro particle	Int J Pediatr Otorhinolaryngol	2013
PM10	DKK1	Microarray analysis of gene expression alteration in human middle ear epithelial cells induced by micro particle	Int J Pediatr Otorhinolaryngol	2013
PM10	TIMP1	Microarray analysis of gene expression alteration in human middle ear epithelial cells induced by micro particle	Int J Pediatr Otorhinolaryngol	2013
PM10	CTGF	Microarray analysis of gene expression alteration in human middle ear epithelial cells induced by micro particle	Int J Pediatr Otorhinolaryngol	2013
PM10	SERPINF1	Microarray analysis of gene expression alteration in human middle ear epithelial cells induced by micro particle	Int J Pediatr Otorhinolaryngol	2013
PM10	IL7R	Microarray analysis of gene expression alteration in human middle ear epithelial cells induced by micro particle	Int J Pediatr Otorhinolaryngol	2013
PM10	ITGB2	Microarray analysis of gene expression alteration in human middle ear epithelial cells induced by micro particle	Int J Pediatr Otorhinolaryngol	2013
PM10	FN1	Microarray analysis of gene expression alteration in human middle ear epithelial cells induced by micro particle	Int J Pediatr Otorhinolaryngol	2013

PM10	THBS1	Microarray analysis of gene expression alteration in human middle ear epithelial cells induced by micro particle	Int J Pediatr Otorhinolaryngol	2013
PM10	VIM	Microarray analysis of gene expression alteration in human middle ear epithelial cells induced by micro particle	Int J Pediatr Otorhinolaryngol	2013
PM10	SERPINE1	Microarray analysis of gene expression alteration in human middle ear epithelial cells induced by micro particle	Int J Pediatr Otorhinolaryngol	2013
PM10	TNC	Microarray analysis of gene expression alteration in human middle ear epithelial cells induced by micro particle	Int J Pediatr Otorhinolaryngol	2013
PM10	CXCL1	Microarray analysis of gene expression alteration in human middle ear epithelial cells induced by micro particle	Int J Pediatr Otorhinolaryngol	2013
PM10	ACTG2	Microarray analysis of gene expression alteration in human middle ear epithelial cells induced by micro particle	Int J Pediatr Otorhinolaryngol	2013
PM10	MMP9	Microarray analysis of gene expression alteration in human middle ear epithelial cells induced by micro particle	Int J Pediatr Otorhinolaryngol	2013
PM10	IL6	Microarray analysis of gene expression alteration in human middle ear epithelial cells induced by micro particle	Int J Pediatr Otorhinolaryngol	2013
PM10	EDN1	Microarray analysis of gene expression alteration in human middle ear epithelial cells induced by micro particle	Int J Pediatr Otorhinolaryngol	2013
PM10	MYCN	Microarray analysis of gene expression alteration in human middle ear epithelial cells induced by micro particle	Int J Pediatr Otorhinolaryngol	2013
PM10	NOTCH2	Air pollution and diabetes association: Modification by type 2 diabetes genetic risk score.	Environ Int	2016
PM10	PROX1	Air pollution and diabetes association: Modification by type 2 diabetes genetic risk score.	Environ Int	2016
PM10	GCKR	Air pollution and diabetes association: Modification by type 2 diabetes genetic risk score.	Environ Int	2016
PM10	THADA	Air pollution and diabetes association: Modification by type 2 diabetes genetic risk score.	Environ Int	2016
PM10	BCL11A	Air pollution and diabetes association: Modification by type 2 diabetes genetic risk score.	Environ Int	2016
PM10	RBMS1	Air pollution and diabetes association: Modification by type 2 diabetes genetic risk score.	Environ Int	2016
PM10	GRB14	Air pollution and diabetes association: Modification by type 2 diabetes genetic risk score.	Environ Int	2016
PM10	IRS1	Air pollution and diabetes association: Modification by type 2 diabetes genetic risk score.	Environ Int	2016
PM10	PPARG	Air pollution and diabetes association: Modification by type 2 diabetes genetic risk score.	Environ Int	2016
PM10	UBE2E2	Air pollution and diabetes association: Modification by type 2 diabetes genetic risk score.	Environ Int	2016
PM10	PSMD6	Air pollution and diabetes association: Modification by type 2 diabetes genetic risk score.	Environ Int	2016
PM10	ADAMTS9	Air pollution and diabetes association: Modification by type 2 diabetes genetic risk score.	Environ Int	2016
PM10	ADCY5	Air pollution and diabetes association: Modification by type 2 diabetes genetic risk score.	Environ Int	2016
PM10	IGF2BP2	Air pollution and diabetes association: Modification by type 2 diabetes genetic risk score.	Environ Int	2016
PM10	ST64GAL1	Air pollution and diabetes association: Modification by type 2 diabetes genetic risk score.	Environ Int	2016
PM10	ANKRD55	Air pollution and diabetes association: Modification by type 2 diabetes genetic risk score.	Environ Int	2016
PM10	ZBED3	Air pollution and diabetes association: Modification by type 2 diabetes genetic risk score.	Environ Int	2016
PM10	CDKAL1	Air pollution and diabetes association: Modification by type 2 diabetes genetic risk score.	Environ Int	2016
PM10	ZFAND3	Air pollution and diabetes association: Modification by type 2 diabetes genetic risk score.	Environ Int	2016

PM10	KCNK16	Air pollution and diabetes association: Modification by type 2 diabetes genetic risk score.	Environ Int	2016
PM10	DGKB	Air pollution and diabetes association: Modification by type 2 diabetes genetic risk score.	Environ Int	2016
PM10	JAZF1	Air pollution and diabetes association: Modification by type 2 diabetes genetic risk score.	Environ Int	2016
PM10	GCK	Air pollution and diabetes association: Modification by type 2 diabetes genetic risk score.	Environ Int	2016
PM10	GCC1	Air pollution and diabetes association: Modification by type 2 diabetes genetic risk score.	Environ Int	2016
PM10	KLF14	Air pollution and diabetes association: Modification by type 2 diabetes genetic risk score.	Environ Int	2016
PM10	ANK1	Air pollution and diabetes association: Modification by type 2 diabetes genetic risk score.	Environ Int	2016
PM10	TP53INP1	Air pollution and diabetes association: Modification by type 2 diabetes genetic risk score.	Environ Int	2016
PM10	SLC30A8	Air pollution and diabetes association: Modification by type 2 diabetes genetic risk score.	Environ Int	2016
PM10	GLIS3	Air pollution and diabetes association: Modification by type 2 diabetes genetic risk score.	Environ Int	2016
PM10	PTPRD	Air pollution and diabetes association: Modification by type 2 diabetes genetic risk score.	Environ Int	2016
PM10	CDKN2A/B	Air pollution and diabetes association: Modification by type 2 diabetes genetic risk score.	Environ Int	2016
PM10	TLE4	Air pollution and diabetes association: Modification by type 2 diabetes genetic risk score.	Environ Int	2016
PM10	TLE1	Air pollution and diabetes association: Modification by type 2 diabetes genetic risk score.	Environ Int	2016
PM10	CDC123	Air pollution and diabetes association: Modification by type 2 diabetes genetic risk score.	Environ Int	2016
PM10	VPS26A	Air pollution and diabetes association: Modification by type 2 diabetes genetic risk score.	Environ Int	2016
PM10	ZMIZ1	Air pollution and diabetes association: Modification by type 2 diabetes genetic risk score.	Environ Int	2016
PM10	HHEX/IDE	Air pollution and diabetes association: Modification by type 2 diabetes genetic risk score.	Environ Int	2016
PM10	TCF7L2	Air pollution and diabetes association: Modification by type 2 diabetes genetic risk score.	Environ Int	2016
PM10	DUSP8	Air pollution and diabetes association: Modification by type 2 diabetes genetic risk score.	Environ Int	2016
PM10	KCNQ1	Air pollution and diabetes association: Modification by type 2 diabetes genetic risk score.	Environ Int	2016
PM10	KCNJ11	Air pollution and diabetes association: Modification by type 2 diabetes genetic risk score.	Environ Int	2016
PM10	ARAP1	Air pollution and diabetes association: Modification by type 2 diabetes genetic risk score.	Environ Int	2016
PM10	MTNR1B	Air pollution and diabetes association: Modification by type 2 diabetes genetic risk score.	Environ Int	2016
PM10	CCND2	Air pollution and diabetes association: Modification by type 2 diabetes genetic risk score.	Environ Int	2016
PM10	KLHDC5	Air pollution and diabetes association: Modification by type 2 diabetes genetic risk score.	Environ Int	2016
PM10	HMGA2	Air pollution and diabetes association: Modification by type 2 diabetes genetic risk score.	Environ Int	2016
PM10	TSPAN8	Air pollution and diabetes association: Modification by type 2 diabetes genetic risk score.	Environ Int	2016
PM10	HNF1A	Air pollution and diabetes association: Modification by type 2 diabetes genetic risk score.	Environ Int	2016
PM10	SPRY2	Air pollution and diabetes association: Modification by type 2 diabetes genetic risk score.	Environ Int	2016
PM10	C2CD4A	Air pollution and diabetes association: Modification by type 2 diabetes genetic risk score.	Environ Int	2016
PM10	HMG20A	Air pollution and diabetes association: Modification by type 2 diabetes genetic risk score.	Environ Int	2016
PM10	ZFAND6	Air pollution and diabetes association: Modification by type 2 diabetes genetic risk score.	Environ Int	2016
PM10	AP3S2	Air pollution and diabetes association: Modification by type 2 diabetes genetic risk score.	Environ Int	2016

PM10	PRC1	Air pollution and diabetes association: Modification by type 2 diabetes genetic risk score.	Environ Int	2016
PM10	FTO	Air pollution and diabetes association: Modification by type 2 diabetes genetic risk score.	Environ Int	2016
PM10	BCAR1	Air pollution and diabetes association: Modification by type 2 diabetes genetic risk score.	Environ Int	2016
PM10	SRR	Air pollution and diabetes association: Modification by type 2 diabetes genetic risk score.	Environ Int	2016
PM10	HNF1B	Air pollution and diabetes association: Modification by type 2 diabetes genetic risk score.	Environ Int	2016
PM10	MC4R	Air pollution and diabetes association: Modification by type 2 diabetes genetic risk score.	Environ Int	2016
PM10	CILP2	Air pollution and diabetes association: Modification by type 2 diabetes genetic risk score.	Environ Int	2016
PM10	PEPD	Air pollution and diabetes association: Modification by type 2 diabetes genetic risk score.	Environ Int	2016
PM10	GIPR	Air pollution and diabetes association: Modification by type 2 diabetes genetic risk score.	Environ Int	2016
PM10	HNF4A	Air pollution and diabetes association: Modification by type 2 diabetes genetic risk score.	Environ Int	2016
PM10	CAT	Different Genes Interact with Particulate Matter and Tobacco Smoke Exposure in Affecting Lung Function Decline in the General Population	PLoS One	2012
PM10	GLRX2	Different Genes Interact with Particulate Matter and Tobacco Smoke Exposure in Affecting Lung Function Decline in the General Population	PLoS One	2012
PM10	GPX4	Different Genes Interact with Particulate Matter and Tobacco Smoke Exposure in Affecting Lung Function Decline in the General Population	PLoS One	2012
PM10	GPX7	Different Genes Interact with Particulate Matter and Tobacco Smoke Exposure in Affecting Lung Function Decline in the General Population	PLoS One	2012
PM10	GSR	Different Genes Interact with Particulate Matter and Tobacco Smoke Exposure in Affecting Lung Function Decline in the General Population	PLoS One	2012
PM10	NDUFA12	Different Genes Interact with Particulate Matter and Tobacco Smoke Exposure in Affecting Lung Function Decline in the General Population	PLoS One	2012
PM10	NDUFA13	Different Genes Interact with Particulate Matter and Tobacco Smoke Exposure in Affecting Lung Function Decline in the General Population	PLoS One	2012
PM10	NDUFA6	Different Genes Interact with Particulate Matter and Tobacco Smoke Exposure in Affecting Lung Function Decline in the General Population	PLoS One	2012
PM10	NDUFS1	Different Genes Interact with Particulate Matter and Tobacco Smoke Exposure in Affecting Lung Function Decline in the General Population	PLoS One	2012
PM10	NDUFS2	Different Genes Interact with Particulate Matter and Tobacco Smoke Exposure in Affecting Lung Function Decline in the General Population	PLoS One	2012
PM10	NDUFS3	Different Genes Interact with Particulate Matter and Tobacco Smoke Exposure in Affecting Lung Function Decline in the General Population	PLoS One	2012
PM10	NDUFS4	Different Genes Interact with Particulate Matter and Tobacco Smoke Exposure in Affecting Lung Function Decline in the General Population	PLoS One	2012
PM10	NDUFS8	Different Genes Interact with Particulate Matter and Tobacco Smoke Exposure in Affecting Lung Function Decline in the General Population	PLoS One	2012
PM10	PARK7	Different Genes Interact with Particulate Matter and Tobacco Smoke Exposure in Affecting Lung Function Decline in the General Population	PLoS One	2012
PM10	PARK2	Different Genes Interact with Particulate Matter and Tobacco Smoke Exposure in Affecting Lung Function Decline in the General Population	PLoS One	2012
PM10	PRDX3	Different Genes Interact with Particulate Matter and Tobacco Smoke Exposure in Affecting Lung Function Decline in the General Population	PLoS One	2012

PM10	PRDX5	Different Genes Interact with Particulate Matter and Tobacco Smoke Exposure in Affecting Lung Function Decline in the General Population	PLoS One	2012
PM10	PSEN1	Different Genes Interact with Particulate Matter and Tobacco Smoke Exposure in Affecting Lung Function Decline in the General Population	PLoS One	2012
PM10	SNCA	Different Genes Interact with Particulate Matter and Tobacco Smoke Exposure in Affecting Lung Function Decline in the General Population	PLoS One	2012
PM10	SOD2	Different Genes Interact with Particulate Matter and Tobacco Smoke Exposure in Affecting Lung Function Decline in the General Population	PLoS One	2012
PM10	TXN2	Different Genes Interact with Particulate Matter and Tobacco Smoke Exposure in Affecting Lung Function Decline in the General Population	PLoS One	2012
PM10	TXNRD2	Different Genes Interact with Particulate Matter and Tobacco Smoke Exposure in Affecting Lung Function Decline in the General Population	PLoS One	2012
PM10	UCP2	Different Genes Interact with Particulate Matter and Tobacco Smoke Exposure in Affecting Lung Function Decline in the General Population	PLoS One	2012
PM10	BCL2	Different Genes Interact with Particulate Matter and Tobacco Smoke Exposure in Affecting Lung Function Decline in the General Population	PLoS One	2012
PM10	BCL2L1	Different Genes Interact with Particulate Matter and Tobacco Smoke Exposure in Affecting Lung Function Decline in the General Population	PLoS One	2012
PM10	CASP6	Different Genes Interact with Particulate Matter and Tobacco Smoke Exposure in Affecting Lung Function Decline in the General Population	PLoS One	2012
PM10	CDK1	Different Genes Interact with Particulate Matter and Tobacco Smoke Exposure in Affecting Lung Function Decline in the General Population	PLoS One	2012
PM10	CHUK	Different Genes Interact with Particulate Matter and Tobacco Smoke Exposure in Affecting Lung Function Decline in the General Population	PLoS One	2012
PM10	MAP2K1	Different Genes Interact with Particulate Matter and Tobacco Smoke Exposure in Affecting Lung Function Decline in the General Population	PLoS One	2012
PM10	NFKB1	Different Genes Interact with Particulate Matter and Tobacco Smoke Exposure in Affecting Lung Function Decline in the General Population	PLoS One	2012
PM10	PLCG1	Different Genes Interact with Particulate Matter and Tobacco Smoke Exposure in Affecting Lung Function Decline in the General Population	PLoS One	2012
PM10	PRKCA	Different Genes Interact with Particulate Matter and Tobacco Smoke Exposure in Affecting Lung Function Decline in the General Population	PLoS One	2012
PM10	RELA	Different Genes Interact with Particulate Matter and Tobacco Smoke Exposure in Affecting Lung Function Decline in the General Population	PLoS One	2012
PM10	TP53	Different Genes Interact with Particulate Matter and Tobacco Smoke Exposure in Affecting Lung Function Decline in the General Population	PLoS One	2012
